# OSA Is Associated With the Human Gut Microbiota Composition and Functional Potential in the Population-Based Swedish CardioPulmonary bioImage Study

**DOI:** 10.1016/j.chest.2023.03.010

**Published:** 2023-03-15

**Authors:** Gabriel Baldanzi, Sergi Sayols-Baixeras, Jenny Theorell-Haglöw, Koen F. Dekkers, Ulf Hammar, Diem Nguyen, Yi-Ting Lin, Shafqat Ahmad, Jacob Bak Holm, Henrik Bjørn Nielsen, Louise Brunkwall, Christian Benedict, Jonathan Cedernaes, Sanna Koskiniemi, Mia Phillipson, Lars Lind, Johan Sundström, Göran Bergström, Gunnar Engström, J. Gustav Smith, Marju Orho-Melander, Johan Ärnlöv, Beatrice Kennedy, Eva Lindberg, Tove Fall

**Affiliations:** a Department of Medical Sciences, Molecular Epidemiology and Science for Life Laboratory, Uppsala University, Uppsala, Sweden; b CIBER Cardiovascular Diseases (CIBERCV), Instituto de Salud Carlos III, Madrid, Spain; c Department of Medical Sciences, Respiratory, Allergy and Sleep Research, Uppsala University, Uppsala, Sweden; d Division of Family Medicine and Primary Care, Department of Neurobiology, Care Science and Society, Karolinska Institute, Huddinge, Sweden; e Department of Family Medicine, Kaohsiung Medical University Hospital, Kaohsiung Medical University, Taiwan; f Preventive Medicine Division, Harvard Medical School, Brigham and Women’s Hospital, Boston, MA; g Clinical Microbiomics A/S, Copenhagen, Denmark; h Department of Clinical Sciences in Malmö, Lund University Diabetes Center, Lund University, Malmö, Sweden; i Molecular Neuropharmacology (Sleep Science Lab), Department of Pharmaceutical Biosciences, Uppsala University, Uppsala, Sweden; j Department of Medical Sciences, Transplantation and Regenerative Medicine, Uppsala University, Uppsala, Sweden; k Department of Medical Cell Biology and Science for Life Laboratory, Uppsala University, Uppsala, Sweden; l Department of Cell and Molecular Biology, Uppsala University, Uppsala, Sweden; m Department of Medical Sciences, Clinical Epidemiology, Uppsala University, Uppsala, Sweden; n The George Institute for Global Health, University of New South Wales, Sydney, NSW, Australia; o Department of Molecular and Clinical Medicine, Institute of Medicine, Sahlgrenska Academy, University of Gothenburg, Gothenburg, Sweden; p Department of Clinical Physiology, Sahlgrenska University Hospital, Region Västra Götaland, Gothenburg, Sweden; q The Wallenberg Laboratory/Department of Molecular and Clinical Medicine, Institute of Medicine, Gothenburg University and the Department of Cardiology, Sahlgrenska University Hospital, Gothenburg, Sweden; r Department of Cardiology, Clinical Sciences, Lund University and Skåne University Hospital, Lund, Sweden; s Wallenberg Center for Molecular Medicine and Lund University Diabetes Center, Lund University, Lund, Sweden; t School of Health and Social Studies, Dalarna University, Falun, Sweden

**Keywords:** epidemiology, microbiota, OSA

## Abstract

**Background:**

OSA is a common sleep-breathing disorder linked to increased risk of cardiovascular disease. Intermittent upper airway obstruction and hypoxia, hallmarks of OSA, have been shown in animal models to induce substantial changes to the gut microbiota composition, and subsequent transplantation of fecal matter to other animals induced changes in BP and glucose metabolism.

**Research Question:**

Does OSA in adults associate with the composition and functional potential of the human gut microbiota?

**Study Design and Methods:**

We used respiratory polygraphy data from up to 3,570 individuals 50 to 64 years of age from the population-based Swedish Cardiopulmonary bioimage Study combined with deep shotgun metagenomics of fecal samples to identify cross-sectional associations between three OSA parameters covering apneas and hypopneas, cumulative sleep time in hypoxia, and number of oxygen desaturation events with gut microbiota composition. Data collection about potential confounders was based on questionnaires, onsite anthropometric measurements, plasma metabolomics, and linkage with the Swedish Prescribed Drug Register.

**Results:**

We found that all three OSA parameters were associated with lower diversity of species in the gut. Furthermore, in multivariable-adjusted analysis, the OSA-related hypoxia parameters were associated with the relative abundance of 128 gut bacterial species, including higher abundance of *Blautia obeum* and *Collinsella aerofaciens*. The latter species was also independently associated with increased systolic BP. Furthermore, the cumulative time in hypoxia during sleep was associated with the abundance of genes involved in nine gut microbiota metabolic pathways, including propionate production from lactate. Finally, we observed two heterogeneous sets of plasma metabolites with opposite association with species positively and negatively associated with hypoxia parameters, respectively.

**Interpretation:**

OSA-related hypoxia, but not the number of apneas/hypopneas, is associated with specific gut microbiota species and functions. Our findings lay the foundation for future research on the gut microbiota-mediated health effects of OSA.

FOR EDITORIAL COMMENT, SEE PAGE 290

**Take-home Points**

**Study Question:** Does OSA in adults associate with the composition and functional potential of the human gut microbiota?

**Results:** OSA-related hypoxia was associated with the relative abundance of 128 gut bacterial species and with the abundance of genes involved in nine gut microbiota metabolic pathways.

**Interpretation:** Our findings suggest a connection between OSA-related hypoxia and alterations of specific species and functional potential of the gut microbiota and lay the foundation for future research on the gut microbiota-mediated health effects of OSA.

OSA is characterized by upper airway collapse episodes during sleep resulting in complete cessation (apneas) or reduction (hypopneas) of air flow and consequent intermittent hypoxia.^1^ The prevalence of OSA has been increasing, partially attributed to the worldwide rising prevalence of obesity,^2^ a well-described cause of OSA.^3^ Although OSA has been prospectively associated with cardiovascular disease independent of BMI,^4^^,^^5^ the mechanisms are not yet fully elucidated.^6^

The most common clinical parameter of OSA severity is the apnea-hypopnea index (AHI), which quantifies the number of apneas and hypopneas during sleep. However, AHI does not differentiate short apnea events with mild oxygen desaturation from prolonged events with severe hypoxia.^7^ To quantify the time in hypoxia, the percentage of sleep time with oxygen saturation < 90% (T90) is used.^8^ Finally, the oxygen desaturation index (ODI) quantifies the number of oxygen desaturation events,^9^ and it is the most suitable parameter to measure intermittent hypoxia.^10^ In sum, the three parameters are complementary to each other because they capture different dimensions of OSA.

Studies in animal models of OSA have found that intermittent hypoxia and airway obstruction produce substantial changes in the gut microbiota composition.^11^, ^12^, ^13^, ^14^ In turn, alterations of the gut microbiota induced by OSA may partly mediate the effects of OSA on adverse health outcomes, including hypertension and impaired glucose metabolism.^14^, ^15^, ^16^ Smaller studies in humans have linked OSA to the microbiota composition in the upper airways (n = 92)^17^ and the gut.^18^, ^19^, ^20^ Bikov et al^19^ reported a lower abundance of the phylum Actinobacteria and a higher abundance of Proteobacteria in patients with OSA (n = 19) compared with control subjects (n = 20), which was not confirmed in other studies of comparable size.^18^^,^^20^ More recently, Li et al^18^ found that AHI was associated with higher abundance of *Fusobacterium* species and lower abundance of *Peptoclostridium* species in 48 individuals with symptoms of OSA. However, these studies did not adjust for important confounders (eg, diet, medications) and were limited in their taxonomic resolution of the microbiota.

To overcome these limitations, adequately powered studies combining extensive information on confounders with species-level microbiota data are needed. Here, we used a validated method for population-wide screening for OSA (ApneaLink Air; ResMed)^21^^,^^22^ to investigate in a cross-sectional study how the OSA parameters AHI, T90, and ODI are associated with the human gut microbiota analyzed with shotgun metagenomic sequencing in up to 3,570 participants from the large population-based Swedish Cardiopulmonary Bioimage Study (SCAPIS). Moreover, given that animal studies suggested that the OSA-induced alterations are connected to cardiometabolic disturbances, we investigated whether the OSA-associated gut microbiota features were also associated with cardiovascular risk factors independent of OSA severity.

## Study Design and Methods

### Study Population

**e-Figure 1:**
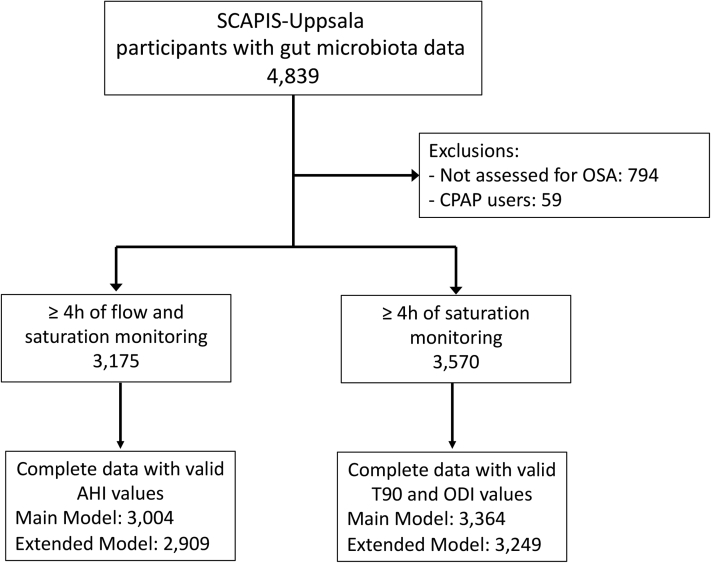
Study flowchart for the 4,839 SCAPIS-Uppsala participants with fecal metagenomics data.

From 2013 to 2018, a total of 30,154 people 50 to 64 years of age were randomly invited from the general population across six regions in Sweden to enroll in SCAPIS.^23^ For the present study, we included 4,045 participants from the Uppsala region with OSA data and gut microbiota data (e-Fig 1), of which 146 (3.6%) self-reported a doctor diagnosis of OSA. We excluded 59 participants who reported treatment with CPAP. The SCAPIS data collection and the present study were approved by the Swedish Ethical Review Authority (DNR 2018-315 B and amendment 2020-06597, and DNR 2010-228-31M, respectively). All participants provided written informed consent.

### OSA Assessment

Assessment for OSA was conducted using the ApneaLink Air device^22^ during one night at home. Apnea was defined as a reduction of breathing flow ≥ 80% for at least 10 s. Hypopnea was defined as a period of at least 10 s with a decrease in the baseline air flow of 30% to 80% combined with a decrease ≥ 4% in oxygen saturation. A desaturation event was defined as a decrease from baseline ≥ 4% in oxygen saturation. At least 4 h of air flow and oxygen saturation recordings were required to compute a valid AHI value, and at least 4 h of oxygen saturation recording was required to compute valid T90 and ODI values. The AHI was calculated as the mean number of apnea and hypopnea events, and ODI was calculated as the mean number of desaturation events per hour of total recording time. The T90 variable was computed by adding the time spent with an oxygen saturation < 90% and dividing by the total recording time.

Severity groups were defined as no OSA for AHI < 5, mild for AHI 5 to 14.9, moderate for AHI 15 to 29.9, and severe for AHI ≥ 30.^24^ For grouping based on T90, one group was composed of participants with a T90 = 0, whereas the remaining participants were grouped by tertiles (ie, groups T90 = 0, t1, t2, and t3). For the grouping based on ODI, the participants were divided by quartiles of ODI (ie, groups q1, q2, q3, and q4).

### Fecal Metagenomic Analysis

Detailed information on the fecal metagenomic analysis can be found in Dekkers et al.^25^ Briefly, participants were instructed at the first study site visit to collect fecal samples at home and store them in the freezer until the second study site visit. The average interval between visits was 15 days. At the study site, the samples were then kept at −20 °C until they were shipped 0 to 7 days later to the central biobank to be kept at −80 °C. Samples were sent to Clinical Microbiomics A/S (Copenhagen, Denmark) for DNA extraction, shotgun metagenomic sequencing with Illumina Novaseq 6000 system (Illumina), and taxonomic annotation. Each extraction round contained a negative and positive control (ZymoBIOMICS Microbial Community Standard, D6300; Zymo Research). All the negative control subjects showed no detectable DNA. All extractions of positive control subjects had a positive DNA signal. For the positive control samples, the coefficient of variation estimated by the Shannon diversity index was 3.05%. For 158 pairs of biologica replicates, the coefficient of variation was 1.49%.

Metagenomic species were defined as coabundant genes as described in Nielsen et al^26^ and reported as relative abundances. Species that were present in ≤ 1% of the participants were removed, resulting in 1,602 species for subsequent analyses. The taxonomic annotation of the metagenomic species was performed by mapping to National Center for Biotechnology Information's RefSeq^27^ database (downloaded on May 2, 2021). The putative metabolic profile of the species was defined in terms of gut metabolic modules (GMMs).^28^ The GMMs are 103 metabolic pathways, defined as a series of enzymatic steps represented by the Kyoto Encyclopedia of Genes and Genomes orthology identifiers.^28^ A species was considered to contain a GMM if it contained at least two-thirds of the Kyoto Encyclopedia of Genes and Genomes orthology of a module. For modules with three or fewer steps, all steps were required. For modules with alternative paths, only one path had to fulfil the criterion.

### Covariates

Participants answered an extensive questionnaire on demographic information, lifestyle, self-reported health, and diet.^23^ Smoking was categorized as no tobacco use, former tobacco use, or tobacco use. Education was categorized based on the highest level achieved: incomplete compulsory education, compulsory education, upper secondary education, or university education. Leisure time physical activity was self-reported as follows: mostly sedentary, moderate activity, regular and moderate activity, or regular exercise or training. According to birth country, participants were categorized into the following: born in Scandinavia (Sweden, Denmark, Norway or Finland), Europe, Asia, or other countries. Dietary information was assigned as missing for participants whose ln(total energy intake) was greater than the mean of ln(total energy intake) ± 3 SD in the study sample. From the food frequency questionnaires, variables were calculated to estimate alcohol intake (g/d),^29^ fiber intake (g/d),^30^ and total energy intake (kcal/d).^29^ The variable season consisted of 11 categories based on the month of the first study site visit.

Self-reported hypertension; lung disease (ie, COPD, chronic bronchitis, pulmonary emphysema); and use of medication for hypertension, hyperlipidemia, and/or diabetes were categorized as binary variables. Diabetes was defined as either a self-reported doctor diagnosis or as fasting plasma glucose ≥ 7.0 mM or glycated hemoglobin (HbA1c) ≥ 48 mol/mol. Impaired glucose tolerance was defined as no previous diabetes diagnosis and fasting glucose ≥ 6.1 and < 7.0 mM or HbA1c ≥ 42 and < 48 mol/mol. Individuals who used proton pump inhibitors and metformin users identified through the plasma metabolome. Previous antibiotic use (Anatomical Therapeutical Chemical code J01) was based on the Swedish Prescribed Drug Register.

### Plasma Metabolome Analysis

The fasting plasma samples were collected during the first site visit and stored at −80 °C in the central biobank until sent in random order to Metabolon Inc for metabolomics profiling, as previously described.^25^

### Statistical Analyses

**e-Figure 2:**
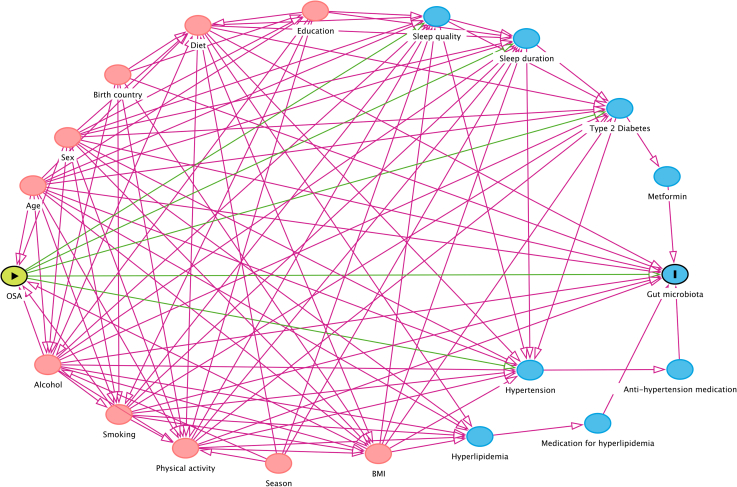
Directed acyclic graph depicting the causal assumptions on the effect of OSA on the gut microbiota composition. A directed edge (or “arrow”) from one node to another represents a direct effect between these two nodes. Green line: causal path; pink line: biasing path; pink nodes: ancestor of exposure and outcome; and blue nodes: ancestor of outcome.

We created a directed acyclic graph (DAG) (e-Fig 2) using the application DAGitty 3.0^31^ to identify the minimal set of confounders for adjustment. Therefore, the main model consisted of age, sex, smoking, alcohol intake, BMI, and DNA extraction plate to account for variation between batches. Given the complexity of the DAG and that potential confounders (eg, diet^32^) were not included in the minimal set, we constructed an extended model accounting for fiber intake, total energy intake, physical activity, education, birth country, and season. Sleep duration was not included because we regarded it as a mediator in the DAG. Unless otherwise stated, associations were investigated with partial Spearman correlation.

The Shannon index^33^ (alpha diversity) was used as a metric of gut microbiota richness and evenness, whereas the Bray-Curtis dissimilarity (beta diversity) was used as a metric of interindividual compositional difference. From the R package vegan,^34^ we used the function diversity to calculate the Shannon index and the function vegdist to calculate the Bray-Curtis dissimilarity based on the species relative abundance. To graphically compare beta diversity across groups of OSA severity, we conducted a principal coordinate analysis on the Bray-Curtis dissimilarity matrix, and differences were tested with permutational multivariate analysis of variance.

Given that BMI may influence or be influenced by gut microbiota species,^35^ BMI could be considered either a confounder or a source of reverse causation. Therefore, to investigate the association of OSA with individual species, we first applied the main model not including BMI as a screening step. The species identified in the screening were investigated adjusting for all main model covariates including BMI, and adjusting for the extended model. The *P* values were adjusted for multiple comparisons using the Benjamini-Hochberg method^36^ with a false discovery rate set at 5% and referred to as q values.

The species associated with at least one of the OSA parameters in the extended model were further examined in the following four sensitivity analyses: (1) included to the extended model the variables of medication use, more specifically metformin, proton pump inhibitors, and medications for hypertension and/or hyperlipidemia; (2) included waist-to-hip ratio in the extended model to further account for visceral adiposity; (3) excluded participants that had used any antibiotic in the 6 months before the first site visit; and (4) excluded participants with lung disease. The species identified in the extended model were investigated for co-occurrence using a probabilistic co-occurrence analysis implemented in the R package cooccur.^37^

We handled missing data using complete case analysis. In a secondary analysis, we imputed the AHI values for the participants who had valid T90 and ODI values but not valid AHI values. Multiple imputation was conducted using predicted mean matching with five nearest neighbors and 10 imputations including all covariates from the extended model, and Shannon index, T90, ODI, waist-to-hip ratio, and species relative abundance. A separate round of multiple imputation was performed for each species, and Rubin^38^ combination rules were applied to account for the uncertainty in the imputation. This analysis was conducted using the software Stata 15.1 (StataCorp). All other analyses were conducted using the R software version 4.1.1.

Effect modification by hemoglobin level was explored by categorizing participants into low or high hemoglobin groups based on the sex-specific median hemoglobin level. For every species, the pair of correlation coefficients obtained from the two groups was compared as described in Altman and Bland.^39^ Standard errors were estimated with bootsrapping for 1,000 iterations.

We conducted enrichment analyses for GMMs based on ranked *P* values from the extended model results, stratified by the direction of the correlation coefficients. From the GUTSY Atlas (https://gutsyatlas.serve.scilifelab.se/), we retrieved information on enrichment for metabolite groups in the species associations with plasma metabolites.^25^

**e-Figure 3:**
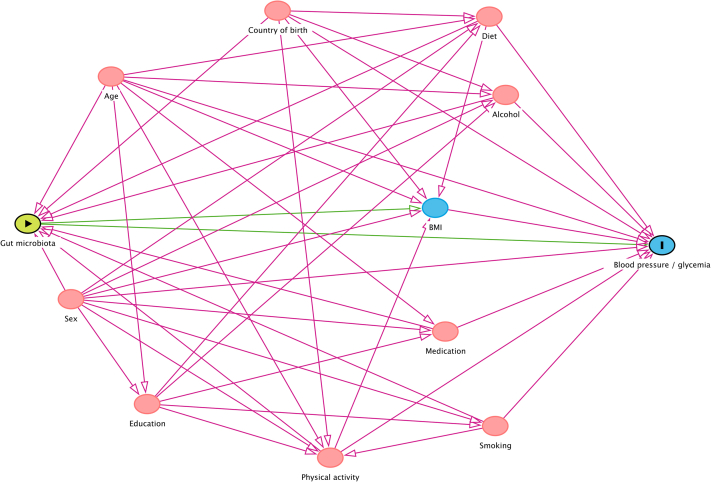
Directed acyclic graph depicting the causal assumptions on the effect of gut microbiota on BP and glycemia. A directed edge (or “arrow”) from one node to another represents a direct effect between these two nodes. Green line: causal path; pink line: biasing path; pink nodes: ancestor of exposure and outcome; and blue nodes: ancestor of outcome.

In a post hoc analysis, we assessed the association of our main gut microbiota findings with HbA1c, and systolic and diastolic BP with adjustment for age, sex, alcohol intake, smoking, fiber intake, total energy intake, physical activity, birth country, ODI, T90, AHI, and DNA extraction plates, as suggested by the DAG (e-Fig 3). We excluded users of medications for hypertension from the analyses with BP, and we excluded users of medications for diabetes from the association analysis with HbA1c. Finally, we added BMI to the model.

## Results

### Descriptive Statistics

The study sample consisted of 3,175 participants with valid AHI values (54% female) and 3,570 participants (52% female) with valid T90 and ODI values. The mean age was 57.7 years. Population characteristics are described in Table 1 and in e-Tables 1 and 2. The Spearman correlation coefficient between the OSA parameters was ρ = 0.56 for AHI and T90, ρ = 0.92 for AHI and ODI, and ρ = 0.63 for T90 and ODI.

**Table 1:** Participants’ Characteristics by OSA Severity Groups

Characteristic	All	No OSA (AHI < 5 events/h)	Mild (AHI 5-14.9 events/h)	Moderate (AHI 15-29.9 events/h)	Severe (AHI ≥ 30 events/h)
No. of participants	3,175 (100)	1,851 (58.3)	899 (28.3)	295 (9.3)	130 (4.1)
Age, y	57.7 (53.9-61.4)	56.8 (53.2-61.0)	58.8 (54.9-62.0)	59.4 (55.7-62.5)	59.5 (55.5-61.7)
Female	1,708 (53.8)	1,122 (60.6)	446 (49.6)	98 (33.2)	42 (32.3)
AHI, events/h	3.8 (1.5-8.6)	1.7 (0.9-3.1)	7.8 (6.2-10.4)	20.1 (17.2-24.1)	38.0 (33.8-49.4)
ODI, events/h	4.1 (1.7-9.2)	2.1 (1.0-3.6)	8.3 (6.0-11.0)	19.1 (15.3-23.7)	35.5 (29.2-42.6)
T90, %	2.0 (0.0-12.0)	1.0 (0.0-4.0)	5.0 (2.0-18.0)	12.0 (6.0-25.0)	20.0 (11.0-41.8)
BMI, kg/m^2^	26.3 (24.0-29.2)	25.2 (23.1-27.7)	27.5 (25.2-30.3)	28.9 (26.1-32.2)	30.0 (27.4-34.2)
WHR	0.9 (0.9-1.0)	0.9 (0.8-1.0)	0.9 (0.9-1.0)	1.0 (0.9-1.0)	1.0 (0.9-1.0)
Shannon index	4.2 (3.9-4.4)	4.2 (4.0-4.4)	4.1 (3.8-4.4)	4.1 (3.9-4.4)	4.0 (3.6-4.2)
SBP, mm Hg	124 (114-135)	121 (112-131)	127 (116-138)	129 (118-140)	132 (120-144)
DBP, mm Hg	76 (70-84)	75 (68-81)	78 (72-85)	80 (74-88)	82 (74-88)
HbA1c, mmol/mol	35 (33-38)	35 (33-37)	36 (34-38)	36 (34-39)	38 (35-41)
Hemoglobin, g/L	141 (133-150)	139 (132-147)	142 (134-151)	146 (138-154)	146 (139-153)
Tobacco use	249 (8.3)	137 (7.8)	79 (9.3)	20 (7.2)	13 (10.9)
Alcohol intake, g/d	5.5 (1.9-10.2)	5.2 (1.8-9.3)	6.1 (2.2-11.2)	7.2 (2.5-13.0)	6.3 (1.2-12.8)
Fiber intake, g/d	18.3 (13.1-25.1)	19.2 (13.7-26.1)	17.3 (12.4-23.5)	17.5 (12.3-23.4)	16.8 (12.8-23.8)
Total energy intake, kcal/d	1,611 (1,267-2,014)	1,624 (1,289-2,039)	1,594 (1,240-1,983)	1,593 (1,198-2,000)	1,672 (1,278-2,067)
Leisure time physical activity					
Mostly sedentary	306 (10.3)	135 (7.7)	106 (12.6)	41 (14.8)	24 (19.8)
Moderate activity	1,369 (45.9)	749 (42.8)	425 (50.7)	137 (49.5)	58 (47.9)
Regular and moderate activity	964 (32.3)	622 (35.6)	238 (28.4)	74 (26.7)	30 (24.8)
Regular exercise or training	346 (11.6)	243 (13.9)	69 (8.2)	25 (9.0)	9 (7.4)
Highest education^a^					
Compulsory	191 (6.3)	82 (4.6)	65 (7.5)	32 (11.3)	12 (9.9)
Upper secondary	247 (41.0)	681 (38.3)	368 (42.7)	136 (48.2)	62 (51.2)
University	1,593 (52.3)	1,012 (56.9)	421 (48.8)	113 (40.1)	47 (38.8)
Birth country					
Scandinavia	2,861 (90.4)	1,670 (90.5)	809 (90.3)	264 (89.5)	118 (90.8)
Europe	123 (3.9)	73 (4.0)	30 (3.3)	15 (5.1)	5 (3.8)
Asia	112 (3.5)	61 (3.3)	37 (4.1)	9 (3.1)	5 (3.8)
Other	70 (2.2)	41 (2.2)	20 (2.2)	7 (2.4)	2 (1.5)
Diabetes	253 (8.0)	106 (5.7)	90 (10.0)	28 (9.5)	29 (22.3)
Hypertension	674 (21.2)	292 (15.8)	235 (26.1)	104 (35.3)	43 (33.1)
Hyperlipidemia	368 (11.6)	169 (9.13)	124 (13.8)	52 (17.6)	23 (17.7)
Medications					
Metformin	91 (2.9)	30 (1.6)	38 (4.2)	9 (3.1)	14 (10.8)
Antihypertensive medication	585 (18.4)	244 (13.2)	207 (23.0)	92 (31.2)	42 (32.3)
Hyperlipidemia medication	236 (7.4)	92 (5.0)	90 (10.0)	37 (12.5)	17 (13.1)
PPI	94 (3.0)	42 (2.3)	25 (2.8)	19 (6.4)	8 (6.2)

Continuous variables are presented as median (interquartile range), and categorical variables are presented as absolute numbers (%). AHI = apnea-hypopnea index; DBP = diastolic BP; HbA1c = glycated hemoglobin; ODI = oxygen desaturation index; PPI = proton pump inhibitor; SBP = systolic BP; T90 = percentage of time with oxygen saturation < 90%; WHR = waist-to-hip ratio.

a Percentages do not add up to 100% because participants with incomplete compulsory education were not included in the table.

After removing participants with missing data on the main model covariates, there were 3,004 participants for the AHI analyses and 3,364 participants for the T90 and ODI analyses. In the analyses adjusted for the extended model covariates, there were 2,909 participants for the AHI analyses and 3,249 participants for the T90 and ODI analyses (e-Fig 1).

### Alpha and Beta Diversity

AHI, T90, and ODI were associated with lower alpha diversity after adjustment for the main and extended model covariates (AHI: ρ = −0.047, *P* = .013; T90: ρ = −0.038, *P* = .034, ODI: ρ = −0.055, *P* = .002). These results indicate a decreased gut microbiota richness and evenness in OSA (e-Table 3).

**Figure 1:**
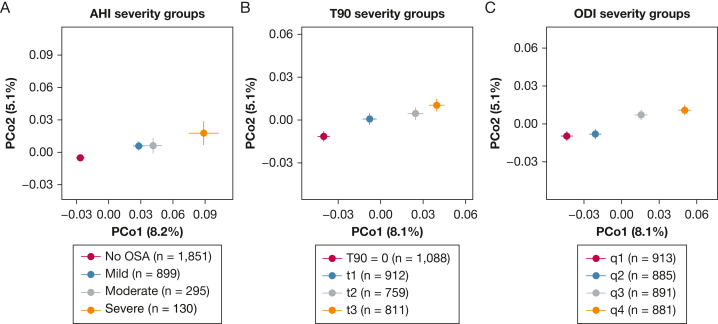
A-C, Principal coordinate analysis showing progressive differences in the overall gut microbiota composition, measured with Bray-Curtis dissimilarity, across groups of OSA severity. Closed circles represent the mean axis value per group, and bars represent the SEs. The percentages on the axes' labels represent the variance explained by each axis. A, AHI severity groups (No OSA: AHI < 5; Mild: AHI 5-14.9; Moderate: AHI 15-29.9; Severe: AHI ≥ 30). B, T90 severity groups were defined as one category including participants with T90 = 0, and the remaining participants divided by tertiles (t1: T90 = 1-3; t2: T90 = 4-14; t3: T90 ≥ 15). C, ODI severity groups were defined by quartiles of ODI (q1: ODI = 0-1.8; q2: ODI = 1.9-4.3; q3: ODI = 4.4-9.4; 4: ODI ≥ 9.5). AHI = apnea-hypopnea index; ODI = oxygen desaturation index; PCo = principal coordinate; T90 = percentage of time with oxygen saturation < 90%.

To determine whether OSA was associated with the overall gut microbiota composition, we analyzed the beta diversity across groups of AHI, T90, and ODI. We observed a separation of groups in order of severity along the first axis of the principal coordinate analysis (Fig 1). The separation was supported by the permutational multivariate analysis of variance adjusted for the main model (AHI groups: *R*^2^ = 0.5%, *P* = .0001; T90 groups: *R*^2^ = 0.2%, *P* = .013; ODI groups: *R*^2^ = 0.3%, *P* = .002) or the extended model (AHI: *R*^2^ = 0.5%, *P* = .0001; T90: *R*^2^ = 0.2%, *P* = .028; ODI: *R*^2^ = 0.2%, *P* = .007). Pairwise comparisons can be found in e-Table 4. Overall, the groups with lowest OSA severity differed from the respective two groups with highest severity (*P* < .05). These results indicate progressive differences in the gut microbiota composition with increased OSA severity even after accounting for confounders.

### OSA-Related Hypoxia Associated With Specific Gut Microbiota

**Figure 2:**
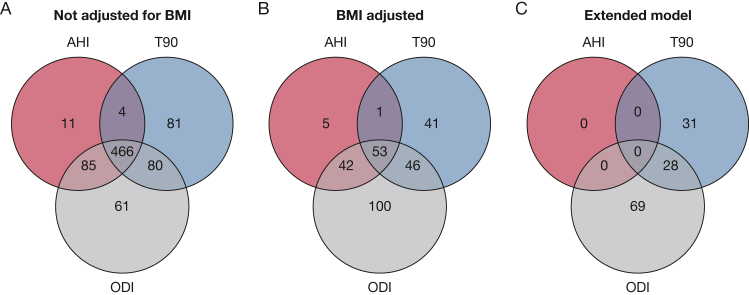
A-C, Number of microbiota species associated with AHI, T90, and/or ODI. Associations investigated using partial Spearman correlation. Adjustment for multiple comparisons using the Benjamini-Hochberg method with a 5% false discovery rate. A, Results from the main model (ie, adjustment for age, sex, smoking, alcohol intake, and DNA extraction plate) not including adjustment for BMI and (B) including adjustment for BMI. For AHI, n = 3,004. For T90 and ODI, n = 3,364. C, Results from the extended model (ie, further adjustment for fiber intake, total energy intake, leisure time physical activity, education, birth country, and season). For AHI, n = 2,909. For T90 and ODI, n = 3,249. AHI = apnea-hypopnea index; ODI = oxygen desaturation index; T90 = percentage of time with oxygen saturation < 90%.

In the analyses not adjusted for BMI, we found that AHI was associated with the abundance of 566 species, T90 was associated with the abundance of 631 species, and ODI was associated with the abundance of 692 species (e-Table 5, Fig 2A). After additional adjustment for BMI, AHI was associated with 101 species, T90 was associated with 141 species, and ODI was associated with 241 species (e-Table 6, Fig 2B).

After adjustment for the extended model, AHI was no longer associated with any species, T90 was associated with 59 species, and ODI was associated with 97 species (e-Table 7, Fig 2C). The parameters T90 and ODI were jointly associated with lower abundance of 22 unclassified species, 16 belonging to the order Eubacteriales, and with higher abundance of six species, including *Blautia obeum* (internal identifier: HG3A.0001), *Ruminococcus gnavus*, *Coprococcus comes*, and the recently isolated *Mediterraneibacter glycyrrhizinilyticus.*^39^

**e-Figure 4:**
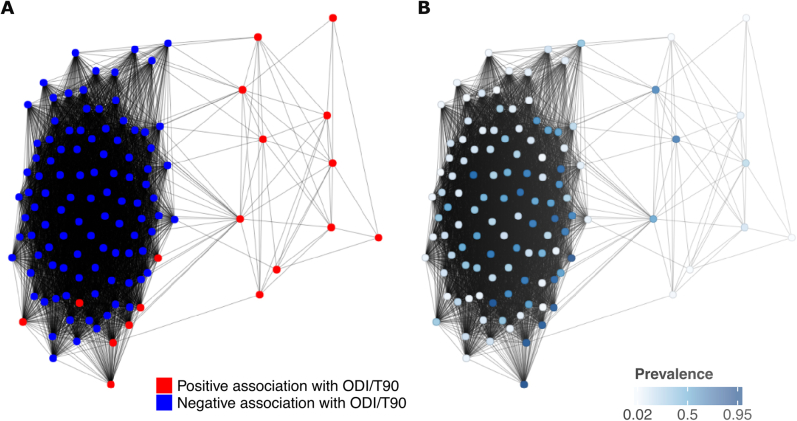
Co-occurrence network for the 117 species associated with T90 and/or ODI in the extended model (ie, age, sex, smoking, alcohol intake, BMI, fiber intake, total energy intake, physical activity, education, birth country, season, and DNA extraction plate) that had a prevalence <95%. (A) Species are labeled by the direction of the association with T90 and/or ODI. (B) Species are labeled by their prevalence.

Together, T90 and ODI were associated with 128 species. A co-occurrence network of these species is present in e-Figure 4. The results show a high co-occurrence of the species negatively associated with ODI/T90, even among rare species. The median relative abundance of the 28 positively associated species combined was 8.8% (interquartile range, 5.3%-14.6%), and the median relative abundance of the 100 negatively associated species combined was 9.3% (interquartile range, 4.0%-15.2%). The relative abundance of individuals species can be found in e-Table 8. In the sensitivity analysis adjusted for medication use (e-Table 9), all species remained associated with T90 and/or ODI (q < 0.05), as well as in the analysis further adjusted for waist-to-hip ratio. After exclusion of the 367 participants who had used antibiotics in the previous 6 months, all associations were retained except the association between ODI and *Eubacteriales* species (HG3A.0691). Finally, the results did not change after excluding participants with lung disease (n = 29). To ascertain that the null findings for AHI were not caused by lower power, we imputed the AHI values for the 340 participants who had information on the extended model covariates and valid T90 and ODI values, but not valid AHI values. Even after imputation, we did not observe any associations for AHI in the extended model (e-Table 10).

### T90 and ODI Associations With Species by Hemoglobin Level

Because hemoglobin level affects the oxygen delivery to tissues,^40^ we hypothesized that OSA-microbiota associations could be different between individuals with higher or lower hemoglobin levels. Therefore, we assessed the effect modification by hemoglobin levels on the 128 species associated with T90 and/or ODI (e-Table 11) by stratifying participants into high or low hemoglobin level groups. In the association between ODI and *Eubacteriales* species (HG3A.1026), we detected a difference between participants with high or low hemoglobin levels (ρ_low_ = 0.008, ρ_high_ = −0.12, heterogeneity q = 0.02). For all other species, heterogeneity q > 0.05. Therefore, we could not ascertain that hemoglobin level was an effect modifier on the associations between OSA-related hypoxia and gut microbiota species.

### Metabolic Pathways Enriched in T90-Associated and/or ODI-Associated Species

**Figure 3:**
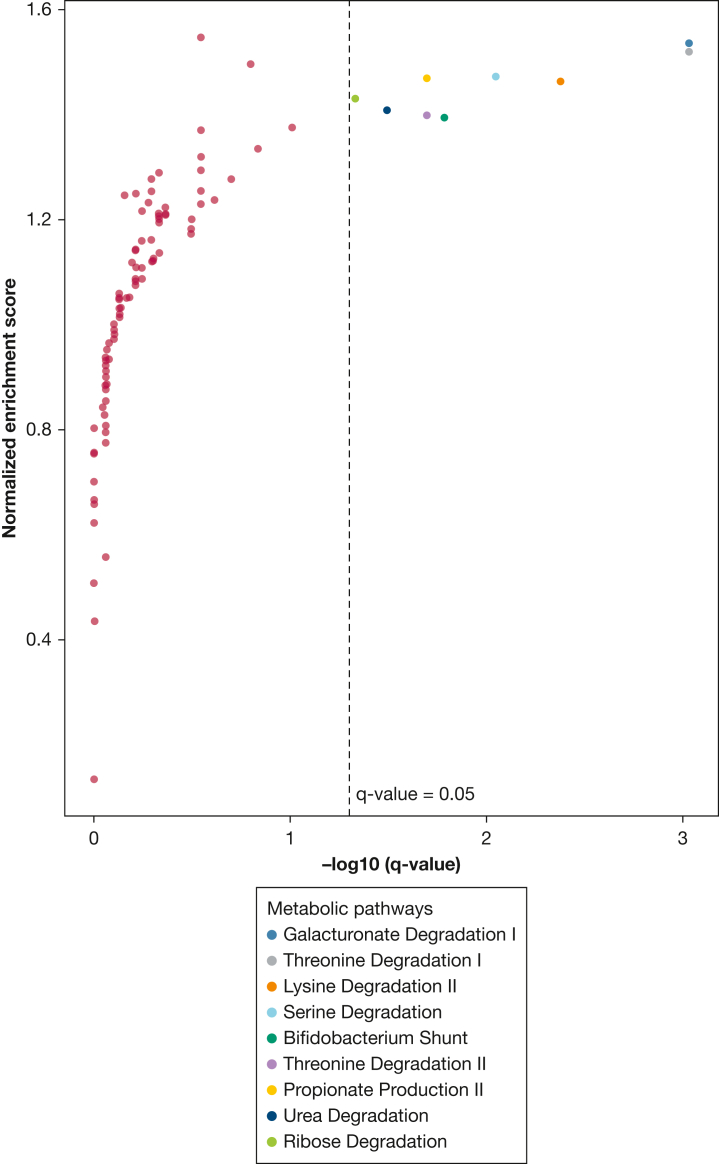
Enrichment for gut metabolic modules among positive associations between percentage of time with oxygen saturation < 90% and gut microbiota species. The pathway enrichment analysis was conducted on the ranked *P* values obtained from Spearman correlations adjusted for age, sex, alcohol intake, smoking, BMI, fiber intake, total energy intake, leisure time physical activity, education, birth country, season, and DNA extraction plate.

To characterize the putative metabolic profile of the species associated with OSA, we performed enrichment analyses for metabolic pathways, defined as GMMs.^27^ We found no GMM enriched in the AHI or ODI associations (e-Table 12). Out of 103 GMMs assessed, the positive T90 associations with gut microbiota species were enriched for nine metabolic pathways (Fig 3), including threonine degradation I (q = 9.3 × 10^−4^) and propionate production II (q = 0.02). These results suggest that hypoxia during sleep may favor species with specific metabolic repertoires.

### Metabolic Fingerprints of the Species Associated With T90 and/or ODI

**Figure 4:**
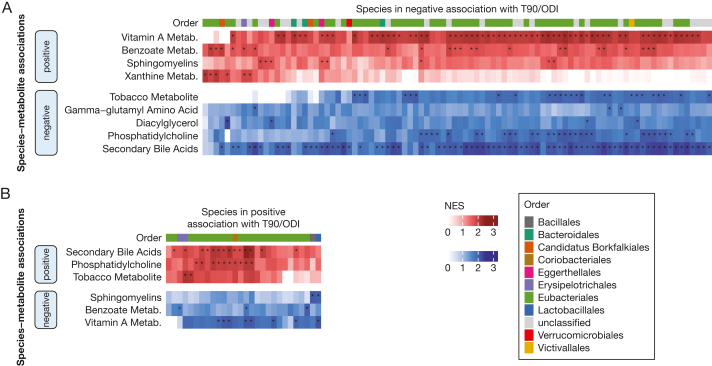
A-B, Relationship of the T90-associated and/or ODI-associated species with the plasma metabolome. T90 and ODI were (A) negatively associated with 100 species and (B) positively associated with 28 species. The heatmaps show the enriched metabolites groups in the associations between species and metabolites stratified by the direction of the associations. Enrichment results retrieved from the GUTSY Atlas (https://gutsyatlas.serve.scilifelab.se/).^24^ Multiple comparisons were adjusted for using the Benjamini-Hochberg method. Associations with an asterisk (∗) were identified considering a false discovery rate of 5%. Metab. = metabolism; NES = normalized enrichment score; ODI = oxygen desaturation index; T90 = percentage of time with oxygen saturation < 90%.

To characterize the metabolomic fingerprint of the 128 species associated with T90 and/or ODI in the extended model, we built heatmaps of enriched metabolite groups for every species based on the GUTSY Atlas.^24^ Several positively associated species were positively associated with secondary bile acids and phosphatidylcholine metabolites (Fig 4B), whereas the negatively associated species were negatively associated with these metabolites (Fig 4A). For 63 of the 100 species negatively associated with T90/ODI, we found enrichment for vitamin A metabolites, and for 22 species, we found enrichment for metabolites involved in benzoate metabolism. The species that were negatively associated with T90 or ODI were also negatively associated with tobacco metabolites.

Overall, these results indicated that the associations between the species and certain plasma metabolites had opposite directions depending on if the species abundance increased or decreased with hypoxia. The main differences were the pattern of association with secondary bile acids, benzoate metabolism, phosphatidylcholines, vitamin A, and tobacco metabolites.

### T90-Associated/ODI-Associated Species Also Associated With BP

**Figure 5:**
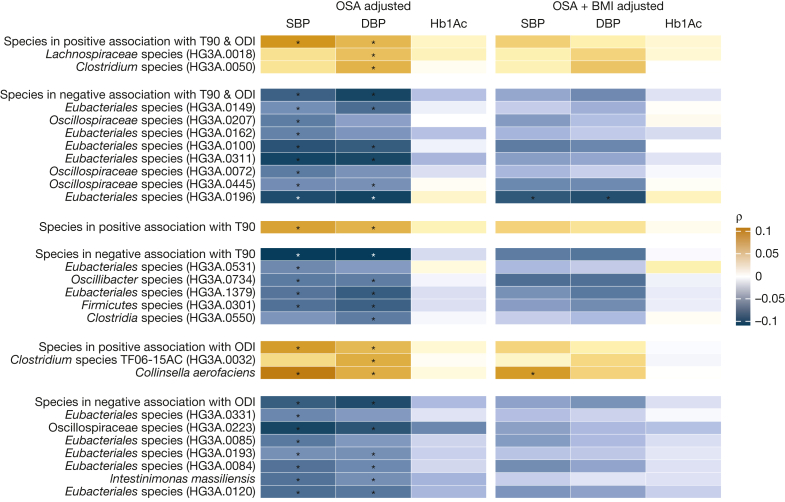
Association of T90-associated/ODI-associated species with BP and Hb1Ac. Heatmap showing the species associated with T90 and/or ODI that were also associated with SBP, DBP, or Hb1Ac. OSA adjusted: Spearman correlation adjusted for age, sex, alcohol intake, smoking, fiber intake, total energy intake, leisure time physical activity, birth country, apnea-hypopnea index, ODI, T90, and DNA extraction plate. OSA+BMI adjusted: additional adjustment for BMI. Multiple comparisons were adjusted for using the Benjamini-Hochberg method. Associations with an asterisk (∗) were identified considering a false discovery rate of 5%. DBP = diastolic BP; Hb1Ac = glycated hemoglobin; ODI = oxygen desaturation index; SBP = systolic BP; T90 = percentage of time with oxygen saturation < 90%.

**e-Figure 5:**
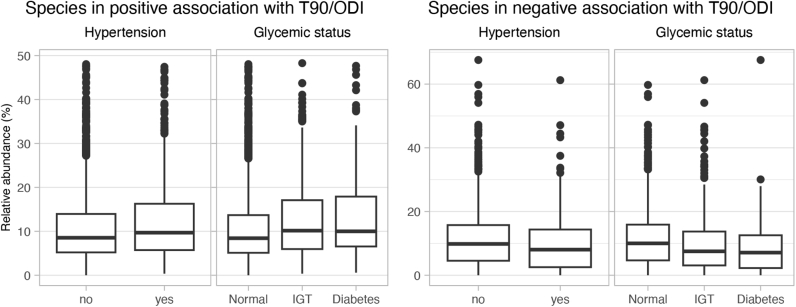
Box plot of the combined relative abundance of the 28 species in positive association and the 100 species in negative association with T90 and/or oxygen desaturation index (ODI) in the extended model. T90: percentage of time with oxygen saturation < 90%.

The combined relative abundance of the species associated with T90/ODI across participants with or without hypertension or impaired glucose tolerance and diabetes is shown in e-Figure 5. We found that the combined abundance of the species associated with T90/ODI was associated with systolic and diastolic BP (n = 2,335) but not with HbA1c (n = 2,786) (e-Table 13, Fig 5), independent of age, sex, alcohol intake, smoking, fiber intake, total energy intake, physical activity, birth country, ODI, T90, and AHI. Among the individual species, the abundance of *Collinsella aerofaciens* was associated with higher systolic and diastolic BP. Additionally, 13 species negatively associated with T90 or ODI were also associated with lower systolic and diastolic BP. After additional adjustment for BMI, *C aerofaciens* continued to associate with systolic BP (ρ = 0.073, q = 0.034), and *Eubacteriales* species (HG3A.0196) continued to negatively associate with systolic (ρ = −0.077, q = 0.031) and diastolic BP (ρ = −0.081, q = 0.015).

## Discussion

Here we presented the most comprehensive population-based study to date investigating the relationship of OSA with the human gut microbiota. We found evidence that OSA, especially OSA-related hypoxia, was associated with the composition and functional potential of the human gut microbiota. The OSA hypoxia parameters, namely T90 and ODI, were associated with the specific species after extensive adjustment for potential confounders (eg, treatment for diabetes, hypertension, hyperlipidemia, and gastritis/gastroesophageal reflux). We further noted enrichment for specific metabolic pathways shared by bacteria positively associated with T90.

Out of the 128 species associated with T90 and/or ODI, 28 were associated with both parameters. Among the six positive associations, four were annotated to the species level, namely *B obeum*, *R gnavus*, *C comes*, and *M glycyrrhizinilyticus*, all belonging to the family Lachnospiraceae. The other two belonged to the Lachnospiraceae family and Clostridiaceae family, respectively. An increased abundance of the Lachnospiraceae family has also been observed in mice subjected to intermittent hypoxia^11^ and in individuals with OSA and hypertension compared with control subjects with hypertension only.^40^ However, a reduced abundance of Lachnospiraceae has been reported in individuals with OSA and type 2 diabetes compared with control subjects.^41^ A higher abundance of *B obeum*, previously named *Ruminococcus obeum*, has been observed in individuals with insulin resistance.^42^ *R gnavus* has been associated with a higher incidence of type 2 diabetes in a large Finnish population-based cohort.^43^^,^ In the present study, we did not find any association of *B obeum* and *R gnavus* with HbA1c in models adjusted for age, sex, alcohol intake, smoking, fiber intake, total energy intake, physical activity, birth country, ODI, T90, and AHI. *C comes* is reported to decrease after bariatric surgery,^44^ a procedure known to improve OSA.^45^ There are previous findings that we were not able to replicate. A recent study in 48 individuals found that AHI was positively associated with the genus *Fusobacterium* after adjustment for age, sex, and BMI.^18^ In our study, all three OSA parameters had a positive association with a *Fusobacterium* species, *Fusobacterium nucleatum*, before BMI adjustment, but not after. In another study of 19 patients with OSA and 20 control subjects, patients with OSA had a higher abundance of the genera *Lactobacillus* and *Roseburia*.^19^ In our species-level analysis, ODI was associated with higher *Roseburia inulinivorans* and lower *Roseburia* species (HG3A.0391) abundance, underscoring heterogeneities in species-level associations within the same genus.

In our study, ODI was positively associated with *C aerofaciens*, which was in turn positively associated with systolic BP independent of BMI and OSA. *C aerofaciens* is an obligate anaerobe abundant in the human gut,^46^ with higher abundance in overweight and obese individuals.^47^ Higher abundance of *C aerofaciens* has also been observed in individuals with type 1 pulmonary hypertension,^48^ a condition associated with lower oxygen saturation.^49^ Longitudinal and experimental studies are necessary to understand whether the gut microbiota species identified in this study may have a role connecting OSA to hypertension and other cardiovascular risk factors. If those studies would confirm OSA-associated morbidities to be caused by gut microbiota alterations, the microbiota could act as a target for dietary, probiotic, or fecal microbiota transplantation interventions aiming to attenuate the cardiometabolic effects of OSA.

The OSA hypoxia parameters T90 and ODI were associated with the abundance of specific species after adjustment for the extended model covariates, while we could no longer detect associations for AHI in the extended model. ODI and AHI are highly correlated variables, and the discrepancy in results could be caused by the smaller sample size for AHI. The Spearman correlation for the AHI and ODI coefficients was 0.89. However, imputing AHI and using the larger dataset showed similar results to the complete case analysis. The AHI distribution might not have been fully reconstructed by the multiple imputation, resulting in less power. For the differences between T90 and AHI results, it is possible that the duration of the nocturnal hypoxia might be of greater importance when it comes to associations with specific gut microbiota species than the number of apnea and hypopnea events. Increasing attention has been given to OSA-related hypoxia because studies have shown that OSA-related hypoxia, unlike AHI, predicted increased cardiovascular mortality.^5^

Evidence in the literature suggests that intermittent host hypoxia directly affects the gut microbiota. Moreno-Indias et al^11^ showed that intermittent hypoxia applied during the mice’s rest phase resulted in enrichment for gut obligate anaerobes. Furthermore, intermittent hypoxia produced oscillations in the oxygen concentration inside the intestinal lumen close to the epithelium, providing a physiologic rationale for how the oxygenation level of the host could impact the gut microbiota environment.^11^

Both acute and chronic sleep fragmentation have been shown to affect the gut microbiota composition in rodents.^50^ Nevertheless, sleep fragmentation alone is unlikely to explain why the hypoxia parameters were associated with specific species in our study, whereas AHI was not. To better disentangle the associations because of sleep fragmentation from associations with nocturnal hypoxia, future studies would benefit from concomitant EEG monitoring, polysomnography assessment, and actigraphy.

One possible indirect mechanism through which OSA might affect the microbiota is through alterations in the host metabolism (eg, accumulation of lactate^51^), as described in patients with OSA.^51^^,^^52^ Experiments with labeled lactate showed that circulating lactate can cross the gut barrier into the intestinal lumen.^53^ Furthermore, a study in athletes found an increase in the gut of microbial genes involved in the conversion of lactate into propionate after exercise.^53^ It was hypothesized that the host lactate produced by exercise crossed into the intestinal lumen and favored bacteria that use lactate as a carbon source. This hypothesis corresponds with our findings; the pathway of propionate production from lactate was associated with T90. Supported by the existing literature, our study suggests an association between the hypoxia caused by OSA, plasma lactate, and the gut microbiota metabolic profile.

### Strengths and Limitations

The strengths of our study include the large sample size, temporal proximity between OSA assessment and fecal microbiota sampling, extensive adjustment for confounders, and objective assessment for OSA instead of self-reported diagnosis or a convenience sample of patients with OSA. Our population-based study provides a more generalizable picture of the association between OSA and the gut microbiota than studies of patients with a clinical diagnosis of OSA. The combination of a large sample size with shotgun metagenomic sequencing allowed us to conduct a comprehensive investigation at the species level and also of the microbial metabolic profile.

Nevertheless, there are limitations that need to be considered. Because of the cross-sectional design, we were not able to assess causality, and despite the extensive adjustment, we cannot rule out residual confounding. Moreover, even if the animal studies indicate a causal effect of OSA on the gut microbiota, an effect in the opposite direction is also plausible. For example, certain gut microbiota species may promote weight gain and thereby OSA.^54^ Additionally, our results may not extend to other populations because of the close connection between geographic location and the gut microbiota.^55^ Our results should be regarded as hypothesis-generating, and no direct clinical translation is implied. The ApneaLink Air two-channel device used to assess sleep apnea does not distinguish obstructive from central apneas. However, OSA is more common in the general population.^56^ Additionally, the T90 parameter is not able to differentiate hypoxia caused by OSA from nocturnal hypoxia of other etiologies. An assessment for OSA based on a single night may result in a certain degree of exposure misclassification,^57^ which could affect the precision, but would not bias our estimates. Finally, we excluded participants that used CPAP, a common treatment for OSA. This was done because there were only 59 CPAP users, and data on treatment compliance were not collected. Therefore, future studies should investigate whether the herein observed OSA-gut microbiota associations are affected by OSA therapy.

## Interpretation

In this largest study to date that has investigated the association between OSA and the human gut microbiota, we found that the objective parameters of OSA-related hypoxia, namely T90 and ODI, were independently associated with 59 and 97 gut microbiota species, respectively. In addition, we found that the gut microbiota associated with T90 was enriched for nine metabolic pathways. Our findings support a connection between the severity of OSA hypoxia and the gut microbiota composition. Future experimental studies are necessary to validate whether the identified microbial species may represent potential therapeutic targets to prevent or treat comorbidities of OSA.

## Funding/Support

Financial support was obtained in the form of grants from the 10.13039/501100000781European Research Council [Grants ERC-STG-2018-801965 to T. F., ERC-CoG-2014-649021 to M. O.-M., ERC-STG-2015-679242 to J. G. S.], the 10.13039/501100003793Swedish Heart-Lung Foundation [Grant Hjärt-Lungfonden, 2019-0505 to T. F., 2020-0485 to E. L., 2018-0343 to J. Ä., 2020-0711 to M. O.-M., 2020-0173 to G. E., 2019-0526 to J. G. S.], the 10.13039/501100004359Swedish Research Council [Grant VR, 2019-01471 to T. F., 2018-02784 to M. O.-M., 2019-01015 to J. Ä., 2020-00243 to J. Ä., 2019-01236 to G. E., 2021-02273 to J. G. S.], the Swedish Research Council for Sustainable Development [Grant FORMAS, 2020-00989 to S. A.], EASD/Novo Nordisk (to S. A.), Göran Gustafsson Foundation [2016 to T. F.], Axel and Signe Lagerman’s Foundation (to T. F.), and governmental funding of clinical research within the Swedish National Health Service (to J. G. S). Funding for SCAPIS was also provided by the Swedish Heart-Lung Foundation, the Knut and Alice Wallenberg Foundation, the Swedish Research Council and VINNOVA (Sweden’s Innovation Agency), the University of Gothenburg and Sahlgrenska University Hospital, Karolinska Institutet and Stockholm County Council, Linköping University and University Hospital, Lund University and Skåne University Hospital, Umeå University and University Hospital, and Uppsala University and University Hospital.

## Financial/Nonfinancial Disclosures

The authors have reported to *CHEST* the following: J. B. H. and H. B. N. are currently working at Clinical Microbiomics A/S. C. B. served as a scientific consultant for Repha GmBH, Langenhagen, Germany, between 2020 and 2021. J. S. has stock ownership in companies providing services to Amgen, AstraZeneca, Boehringer, Bayer, Eli Lilly, Itrim, Janssen, Novo Nordisk, Pfizer, and Takeda, outside the submitted work. J. Ä. has served on the advisory boards for AstraZeneca and Boehringer Ingelheim and has received lecturing fees from AstraZeneca and Novartis, all unrelated to the present work. None declared (G. Baldanzi, S. S.-B., J. T.-H., K. F. D., U. H., D. N., Y.-T. L., S. A., L. B., J. C., S. K., M. P., L. L., G. Bergström, G. E., J. G. S., M. O.-M., B. K., E. L., T. F.).

## Supplementary Data

e-Tables 1 to 4

e-Table 5

e-Table 6

e-Table 7

e-Table 8

e-Table 9

e-Table 10

e-Table 11

e-Table 12

e-Table 13

e-Online Data
