# Supplementary material for: OSA Is Associated With the Human Gut Microbiota Composition and Functional Potential in the Population-Based Swedish CardioPulmonary bioImage Study
Source: Chest. 2023 Mar 15;164(2):503–16. doi: 10.1016/j.chest.2023.03.010 (PMC10410248; doi:10.1016/j.chest.2023.03.010)
Supplement: e-Tables 1 to 4 [file mmc1.docx]

| **e-Tables for Baldanzi et al. Obstructive sleep apnea was associated with the human gut microbiota composition and functional potential in the population-based Swedish CardioPulmonary bioImage Study (SCAPIS)** | | | | | | |
| --- | --- | --- | --- | --- | --- | --- |
| e-Table 1. | Descriptive characteristics by groups of percentage of time with saturation below 90% (T90). | |  |  |  |  |
| e-Table 2. | Descriptive characteristics by groups of oxygen desaturation index (ODI). |  |  |  |  |  |
| e-Table 3. | Partial Spearman's correlations between AHI, T90, or ODI and Shannon diversity index. | |  |  |  |  |
| e-Table 4. | Pairwise comparisons of Bray-Curtis dissimilarity between OSA severity groups based on AHI, T90, or ODI using permutational analysis of variance (PERMANOVA). | | | | | |
| e-Table 5. | Partial Spearman’s correlations of AHI, T90, and ODI with microbiota species using the main model not including BMI | | | |  |  |
| e-Table 6. | Partial Spearman’s correlations of AHI, T90, and ODI with microbiota species using the complete main model. | | |  |  |  |
| e-Table 7. | Partial Spearman’s correlations of AHI, T90, and ODI with microbiota species using the extended main model. | | |  |  |  |
| e-Table 8. | Prevalence and relative abundance and taxonomy of the 128 species associated with T90/ODI | |  |  |  |  |
| e-Table 9. | Partial Spearman’s correlations of AHI with microbiota species using the extended model after imputing missing AHI values | | | |  |  |
| e-Table 10. | Sensitivity analysis for the species associated with T90/ODI in the extended model. Four sensitivity analyses: medication model, waist-hip ratio model, exclusion of antibiotic users, and exclusion of self-reported lung disease. | | | | | |
| e-Table 11. | Stratified analysis for hemoglobin level for the species associated with T90/ODI in the extended model. | | |  |  |  |
| e-Table 12. | Enrichment for gut metabolic modules (GMM) in the associations of AHI, T90, or ODI with microbiota species. | | |  |  |  |
| e-Table 13. | Partial Spearman’s correlations of microbiota features with systolic blood pressure (SBP), diastolic blood pressure (DBP), and glycated hemoglobin (Hb1Ac). | | | | | |

| **e-Table 1. Descriptive characteristics by groups of pergentage of time with saturation below 90% (T90).** | | | | | | |
| --- | --- | --- | --- | --- | --- | --- |
| Participants with T90 data were divided into one group with T90 = 0, and the remaining divided into three groups of similar size (t1, t2, and t3) in order of ascending T90 values. Continuous variables presented as median [interquartile range] and categorical variables presented as absolute numbers (%). AHI: apnea-hypopnea index; BMI: Body mass index; DBP: diastolic blood presssure; HbA1c: glycated hemoglobin; med.: medication; ODI: oxygen desaturation index; PPI: proton-pump inhibitors; SBP: systolic blood pressure. Percentages of highest education do not add to 100% because of participants who did not complete the compulsory education | | | | | |  |
|  | **All** | **T90=0** | **t1** | **t2** | **t3** |  |
| T90 range (%) |  | 0 | [1;3] | [4;14] | ≥15 |  |
| N | 3,570 | 1,088 | 912 | 759 | 811 |  |
| Age (years) | 57.7 [53.9;61.5] | 56.1 [52.7;60.3] | 57.3 [53.9;61.2] | 58.7 [54.8;61.8] | 59.6 [55.6;62.4] |  |
| Female | 1860 (52.1%) | 705 (64.8%) | 470 (51.5%) | 340 (44.8%) | 345 (42.5%) |  |
| AHI (events/h) | 3.8 [1.5;8.6] | 1.4 [0.7;2.7] | 4.2 [2.1;7.3] | 7.1 [3.2;14.6] | 8.1 [3.7;17.9] |  |
| ODI (events/h) | 4.3 [1.8;9.4] | 1.4 [0.8;2.6] | 4.5 [2.6;7.2] | 7.9 [4.0;14.1] | 9.8 [4.7;18.8] |  |
| T90 (%) | 2.0 [0.0;12.0] | 0.0 [0.0;0.0] | 1.0 [1.0;2.0] | 7.0 [5.0;10.0] | 30.0 [20.0;48.0] |  |
| BMI (kg/m^2^) | 26.3 [24.0;29.2] | 24.3 [22.5;26.4] | 25.9 [23.9;28.5] | 27.6 [25.3;30.3] | 28.5 [26.3;31.8] |  |
| WHR | 0.9 [0.9;1.0] | 0.9 [0.8;0.9] | 0.9 [0.9;1.0] | 0.9 [0.9;1.0] | 1.0 [0.9;1.0] |  |
| Shannon index | 4.2 [3.9;4.4] | 4.2 [4.0;4.4] | 4.2 [3.9;4.4] | 4.1 [3.9;4.4] | 4.1 [3.8;4.3] |  |
| SBP (mmHg) | 124 [114;134] | 120 [110;130] | 124 [114;134] | 125 [116;137] | 128 [118;138] |  |
| DBP (mmgHg) | 76 [70;83] | 74 [68;81] | 76 [70;82] | 78 [71;84] | 78 [73;86] |  |
| HbA1c (mmol/mol) | 35 [33;38] | 34 [33;36] | 35 [33;38] | 36 [34;38] | 37 [34;39] |  |
| Hemoglobin (g/L) | 141 [133;150] | 138 [130;146] | 140 [133;149] | 143 [135;152] | 145 [137;153] |  |
| Current smoker | 278 (8.24%) | 53 (5.10%) | 60 (7.00%) | 61 (8.41%) | 104 (13.8%) |  |
| Alcohol intake (g/day) | 5.5 [1.9;10.3] | 4.9 [1.7;8.4] | 5.7 [1.9;10.3] | 6.4 [2.1;11.3] | 6.1 [2.1;12.0] |  |
| Fiber intake (g/day) | 18.3 [13.1;25.2] | 19.8 [14.3;26.5] | 18.7 [13.5;25.8] | 17.7 [12.7;24.4] | 16.8 [11.6;23.2] |  |
| Total energy intake (kcal/day) | 1613 [1268;2036] | 1630 [1308;2029] | 1638 [1296;2054] | 1602 [1223;2046] | 1577 [1220;2003] |  |
| Leisure time physical activity |  |  |  |  |  |  |
| mostly sedentary | 345 (10.3%) | 56 (5.5%) | 76 (8.9%) | 92 (12.8%) | 121 (16.2%) |  |
| moderate activity | 1524 (45.6%) | 425 (41.4%) | 378 (44.4%) | 336 (46.9%) | 385 (51.6%) |  |
| regular and moderate activity | 1079 (32.3%) | 391 (38.1%) | 283 (33.3%) | 223 (31.1%) | 182 (24.4%) |  |
| regular exercise or training | 393 (11.8%) | 155 (15.1%) | 114 (13.4%) | 66 (9.2%) | 58 (7.8%) |  |
| Highest education |  |  |  |  |  |  |
| Compulsory | 219 (6.4%) | 50 (4.8%) | 46 (5.3%) | 50 (6.8%) | 73 (9.5%) |  |
| Upper secondary | 1430 (41.9%) | 381 (36.4%) | 369 (42.3%) | 330 (45.1%) | 350 (45.8%) |  |
| University | 1749 (51.2%) | 613 (58.6%) | 454 (52.0%) | 347 (47.5%) | 335 (43.8%) |  |
| Birth place |  |  |  |  |  |  |
| Scandinavia | 3212 (90.3%) | 986 (90.9%) | 820 (90.3%) | 688 (90.9%) | 718 (88.8%) |  |
| Europe | 144 (4.0%) | 37 (3.4%) | 30 (3.3%) | 30 (4.0%) | 47 (5.8%) |  |
| Asia | 129 (3.6%) | 40 (3.7%) | 41 (4.5%) | 19 (2.5%) | 29 (3.6%) |  |
| Other | 74 (2.1%) | 22 (2.0%) | 17 (1.9%) | 20 (2.6%) | 15 (1.9%) |  |
| Type 2 diabetes | 291 (8.2%) | 41 (3.8%) | 65 (7.1%) | 75 (9.9%) | 110 (13.6%) |  |
| Hypertension | 753 (21.1%) | 140 (12.9%) | 186 (20.4%) | 188 (24.8%) | 239 (29.5%) |  |
| Hyperlipidemia | 410 (11.5%) | 72 (6.62%) | 99 (10.9%) | 102 (13.4%) | 137 (16.9%) |  |
| Medications |  |  |  |  |  |  |
| metformin | 101 (2.83%) | 9 (0.83%) | 24 (2.63%) | 24 (3.16%) | 44 (5.43%) |  |
| antihypertensive med. | 656 (18.4%) | 112 (10.3%) | 158 (17.3%) | 165 (21.7%) | 221 (27.3%) |  |
| hyperlipidemia med. | 258 (7.2%) | 36 (3.3%) | 59 (6.5%) | 67 (8.8%) | 96 (11.8%) |  |
| PPI | 109 (3.1%) | 21 (1.9%) | 22 (2.4%) | 30 (4.0%) | 36 (4.4%) |  |
| ESS | 6 [3;9] | 5 [3;8] | 6 [3;9] | 6 [3;9] | 6 [3;9] |  |

| **e-Table 2. Descriptive characteristics by groups of oxygen desaturation index (ODI)** | | | | | |
| --- | --- | --- | --- | --- | --- |
| Participants with ODI data were divided into quartiles (q1, q2, q3, and q4). Continuous variables presented as median [interquartile range] and categorical variables presented as absolute numbers (%). AHI: apnea-hypopnea index; BMI: Body mass index; DBP: diastolic blood pressure; ESS: Epworth sleepiness scale; HbA1c: glycated hemoglobin; med.: medication; PPI: proton-pump inhibitors; SBP: systolic blood pressure; T90: percentage of time with oxygen saturation below 90%. Percentages of highest education do not add to 100% because of participants who did not complete the compulsory education. | | | | | |
|  | **All** | **q1** | **q2** | **q3** | **q4** |
| ODI range (events/h) |  | [0;1.8] | [1.9;4.3] | [4.4;9.4] | ≥9.5 |
| N | 3570 | 913 | 885 | 891 | 881 |
| Age (years) | 57.7 [53.9;61.5] | 56.2 [52.7;60.4] | 57.2 [53.6;61.1] | 58.5 [54.7;61.8] | 59.3 [55.6;62.2] |
| Female | 1860 (52.1%) | 610 (66.8%) | 490 (55.4%) | 434 (48.7%) | 326 (37.0%) |
| AHI (events/h) | 3.8 [1.5;8.6] | 0.8 [0.5;1.4] | 2.5 [1.7;3.5] | 5.4 [4.1;7.0] | 15.8 [10.4;24.6] |
| ODI (events/h) | 4.3 [1.8;9.4] | 0.9 [0.5;1.3] | 2.9 [2.3;3.5] | 6.2 [5.1;7.6] | 15.4 [11.6;22.9] |
| T90 (%) | 2.0 [0.0;12.0] | 0.0 [0.0;1.0] | 1.0 [0.0;6.0] | 3.0 [1.0;13.0] | 13.0 [6.0;29.0] |
| BMI (kg/m^2^) | 26.3 [24.0;29.2] | 24.2 [22.4;26.1] | 25.6 [23.6;27.9] | 27.2 [25.1;29.7] | 28.8 [26.3;32.3] |
| WHR | 0.9 [0.9;1.0] | 0.9 [0.8;0.9] | 0.9 [0.9;1.0] | 0.9 [0.9;1.0] | 1.0 [0.9;1.0] |
| Shannon index | 4.2 [3.9;4.4] | 4.2 [4.0;4.5] | 4.2 [3.9;4.4] | 4.1 [3.9;4.4] | 4.1 [3.8;4.3] |
| SBP (mmHg) | 124 [114;134] | 118 [110;128] | 122 [112;132] | 125 [116;136] | 128 [118;140] |
| DBP (mmgHg) | 76 [70;83] | 73 [68;80] | 76 [70;82] | 78 [71;84] | 79 [73;86] |
| HbA1c (mmol/mol) | 35 [33;38] | 34 [33;36] | 35 [33;37] | 36 [34;38] | 37 [34;39] |
| Hemoglobin (g/L) | 141 [133;150] | 138 [131;146] | 140 [132;149] | 142 [134;150] | 144 [137;153] |
| Current smoker | 278 (8.24%) | 59 (6.79%) | 68 (8.08%) | 78 (9.34%) | 73 (8.82%) |
| Alcohol intake (g/day) | 5.5 [1.9;10.3] | 4.7 [1.8;8.3] | 5.6 [1.8;10.2] | 5.9 [2.1;10.5] | 6.6 [2.1;12.5] |
| Fiber intake (g/day) | 18.3 [13.1;25.2] | 19.9 [14.6;26.6] | 19.1 [13.4;26.1] | 17.6 [12.8;25.0] | 16.8 [11.9;22.9] |
| Total energy intake (kcal/day) | 1613 [1268;2036] | 1615 [1287;2018] | 1636 [1296;2049] | 1637 [1281;2074] | 1571 [1205;1995] |
| Leisure time physical activity |  |  |  |  |  |
| mostly sedentary | 345 (10.3%) | 60 (6.9%) | 60 (7.2%) | 87 (10.5%) | 138 (16.8%) |
| moderate activity | 1524 (45.6%) | 339 (39.1%) | 360 (43.5%) | 409 (49.5%) | 416 (50.7%) |
| regular and moderate activity | 1079 (32.3%) | 333 (38.5%) | 300 (36.2%) | 241 (29.2%) | 205 (25.0%) |
| regular exercise or training | 393 (11.8%) | 134 (15.5%) | 108 (13.0%) | 89 (10.8%) | 62 (7.6%) |
| Highest education |  |  |  |  |  |
| Compulsory | 219 (6.4%) | 35 (4.0%) | 45 (5.3%) | 57 (6.7%) | 82 (9.8%) |
| Upper secondary | 1430 (41.9%) | 317 (36.2%) | 348 (40.8%) | 367 (43.3%) | 398 (47.4%) |
| University | 1749 (51.2%) | 522 (59.7%) | 456 (53.5%) | 415 (48.9%) | 356 (42.4%) |
| Birth place |  |  |  |  |  |
| Scandinavia | 3212 (90.3%) | 828 (91.0%) | 796 (90.2%) | 805 (90.9%) | 783 (88.9%) |
| Europe | 144 (4.0%) | 36 (4.0%) | 34 (3.9%) | 33 (3.7%) | 41 (4.7%) |
| Asia | 129 (3.6%) | 28 (3.1%) | 32 (3.6%) | 33 (3.7%) | 36 (4.1%) |
| Other | 74 (2.1%) | 18 (2.0%) | 20 (2.3%) | 15 (1.7%) | 21 (2.4%) |
| Type 2 diabetes | 291 (8.2%) | 41 (4.5%) | 51 (5.8%) | 84 (9.4%) | 115 (13.1%) |
| Hypertension | 753 (21.1%) | 99 (10.8%) | 157 (17.7%) | 220 (24.7%) | 277 (31.4%) |
| Hyperlipidemia | 410 (11.5%) | 63 (6.90%) | 87 (9.83%) | 116 (13.0%) | 144 (16.3%) |
| Medications |  |  |  |  |  |
| metformin | 101 (2.83%) | 4 (0.44%) | 13 (1.47%) | 31 (3.48%) | 53 (6.02%) |
| antihypertensive med. | 656 (18.4%) | 78 (8.5%) | 132 (14.9%) | 196 (22.0%) | 250 (28.4%) |
| hyperlipidemia med. | 258 (7.2%) | 30 (3.3%) | 44 (5.0%) | 75 (8.4%) | 109 (12.4%) |
| PPI | 109 (3.1%) | 14 (1.5%) | 23 (2.6%) | 29 (3.3%) | 43 (4.9%) |
| ESS | 6 [3;9] | 5 [3;8] | 6 [3;8] | 6 [3;9] | 6 [3;10] |

| **e-Table 3. Partial Spearman's correlations between AHI, T90, or ODI and Shannon diversity index.** | | | | | | | |  |  |
| --- | --- | --- | --- | --- | --- | --- | --- | --- | --- |
| Main model: adjustment for age, sex, smoking, alcohol intake, body mass index, and DNA extraction plate | | | | | | | | | |
| Extended model: additional adjustment for fiber intake, total energy intake, leisure physical activity, education, country of birth, and season. | | | | | | | | | |
| AHI: apnea-hypopnea index; ODI: oxygen desaturation index; T90: percentage of time with oxygen saturation below 90%. | | | | | | | | | |
|  |  |  |  |  |  |  |  |  |  |
| **exposure** | **Spearman's correlation** | **p-value** | **N** | **model** |  |  |  |  |  |
| AHI | -0.058 | 0.002 | 3004 | Main model |  |  |  |  |  |
| AHI | -0.047 | 0.013 | 2909 | Extended model |  |  |  |  |  |
| T90 | -0.043 | 0.013 | 3364 | Main model |  |  |  |  |  |
| T90 | -0.038 | 0.034 | 3249 | Extended model |  |  |  |  |  |
| ODI | -0.065 | 1.75E-04 | 3364 | Main model |  |  |  |  |  |
| ODI | -0.055 | 0.002 | 3249 | Extended model |  |  |  |  |  |

| **e-Table 4. Pairwise comparisons of Bray-Curtis dissimilarity between OSA severity groups based on AHI, T90, or ODI using permutational analysis of variance (PERMANOVA)** | | | | | | | | | | | | |
| --- | --- | --- | --- | --- | --- | --- | --- | --- | --- | --- | --- | --- |
| Main model: adjustment for age, sex, smoking, alcohol intake, body mass index, and DNA extraction plate | | | | | | | | |  |  |  |  |
| Extended model: additional adjustment for fiber intake, total energy intake, leisure physical activity, education, country of birth, and season. | | | | | | | | | | | |  |
| Groups based on AHI: No OSA: AHI<5; Mild: AHI 5–14.9; Moderate: AHI 15–29.9; Severe: AHI ≥30. | | | | | | | |  |  |  |  |  |
| Groups based on T90: one category including participants with T90 = 0, and the remaining participants divided into tertiles (t1: T90 = 1–3; t2: T90 = 4–14; and t3: T90 ≥15). | | | | | | | | | | | |  |
| Groups based on ODI: quartiles of ODI (q1: ODI = 0–1.8; q2: ODI = 1.9–4.3; q3: ODI = 4.4–9.4; and q4: ODI ≥ 9.5). | | | | | | | | | |  |  |  |
|  |  |  |  |  |  |  |  |  |  |  |  |  |
| **Main model** |  |  |  |  | **Extended model** | |  |  |  |  |  |  |
| **Groups based on AHI** | |  |  |  | **Groups based on AHI** | |  |  |  |  |  |  |
| **group.1** | **group.2** | **R2 (%)** | **p-value** |  | **group.1** | **group.2** | **R2 (%)** | **p-value** |  |  |  |  |
| No OSA | Mild | 0.2 | 0.002 |  | No OSA | Mild | 0.2 | 0.0022 |  |  |  |  |
| No OSA | Moderate | 0.23 | 0.004 |  | No OSA | Moderate | 0.23 | 0.0042 |  |  |  |  |
| No OSA | Severe | 0.3 | 0.001 |  | No OSA | Severe | 0.3 | 0.0013 |  |  |  |  |
| Mild | Moderate | 0.57 | 0.0004 |  | Mild | Moderate | 0.57 | 0.0004 |  |  |  |  |
| Mild | Severe | 0.12 | 0.261 |  | Mild | Severe | 0.12 | 0.2611 |  |  |  |  |
| Moderate | Severe | 0.83 | 0.014 |  | Moderate | Severe | 0.83 | 0.0142 |  |  |  |  |
|  |  |  |  |  |  |  |  |  |  |  |  |  |
| **Groups based on T90** | |  |  |  | **Groups based on T90** | |  |  |  |  |  |  |
| **group.1** | **group.2** | **R2 (%)** | **p-value** |  | **group.1** | **group.2** | **R2 (%)** | **p-value** |  |  |  |  |
| T90=0 | t1 | 0.07 | 0.216 |  | T90=0 | t1 | 0.07 | 0.2155 |  |  |  |  |
| T90=0 | t2 | 0.22 | 0.010 |  | T90=0 | t2 | 0.22 | 0.0101 |  |  |  |  |
| T90=0 | t3 | 0.24 | 0.006 |  | T90=0 | t3 | 0.24 | 0.0057 |  |  |  |  |
| t1 | t2 | 0.08 | 0.212 |  | t1 | t2 | 0.08 | 0.2123 |  |  |  |  |
| t1 | t3 | 0.09 | 0.185 |  | t1 | t3 | 0.09 | 0.1847 |  |  |  |  |
| t2 | t3 | 0.05 | 0.527 |  | t2 | t3 | 0.05 | 0.5272 |  |  |  |  |
|  |  |  |  |  |  |  |  |  |  |  |  |  |
| **Groups based on ODI** | |  |  |  | **Groups based on ODI** | |  |  |  |  |  |  |
| **group.1** | **group.2** | **R2 (%)** | **p-value** |  | **group.1** | **group.2** | **R2 (%)** | **p-value** |  |  |  |  |
| q1 | q2 | 0.05 | 0.469 |  | q1 | q2 | 0.05 | 0.4685 |  |  |  |  |
| q1 | q3 | 0.21 | 0.014 |  | q1 | q3 | 0.21 | 0.0136 |  |  |  |  |
| q1 | q4 | 0.35 | 0.001 |  | q1 | q4 | 0.35 | 9.00E-04 |  |  |  |  |
| q2 | q3 | 0.11 | 0.112 |  | q2 | q3 | 0.11 | 0.1116 |  |  |  |  |
| q2 | q4 | 0.27 | 0.006 |  | q2 | q4 | 0.27 | 0.0059 |  |  |  |  |
| q3 | q4 | 0.06 | 0.355 |  | q3 | q4 | 0.06 | 0.3547 |  |  |  |  |
