# Supplementary material for: OSA Is Associated With the Human Gut Microbiota Composition and Functional Potential in the Population-Based Swedish CardioPulmonary bioImage Study
Source: Chest. 2023 Mar 15;164(2):503–16. doi: 10.1016/j.chest.2023.03.010 (PMC10410248; doi:10.1016/j.chest.2023.03.010)
Supplement: e-Table 5 [file mmc2.docx]

e-Table 5. Partial Spearman’s correlations of AHI, T90, and ODI with microbiota species using the main model not including BMI

Associations adjusted for age, sex, smoking, alcohol intake, and DNA extraction plate. Adjustment for multiple testing using the Benjamini-Hochberg method and presented as q-values. Under the column "Metagenomics species", the information between parenthesis is the internal identifier for the respective species. AHI: apnea-hypopnea index; ODI: oxygen desaturation index; and T90: percentage of time with oxygen saturation below 90%.

**Metagenomic species exposure Spearman's correlation**

**p-value q-value N subspecies species genus family order class phylum**

Dorea formicigenerans (HG3A.0006)

| Blautia obeum (HG3A.0001) | T90 | 0.161 | 1.16E-20 | 9.26E-18 | 3364 | unclassified | Blautia obeum | Blautia | Lachnospiraceae | Eubacteriales | Clostridia | Firmicutes |
| --- | --- | --- | --- | --- | --- | --- | --- | --- | --- | --- | --- | --- |
| Eubacteriales sp. (HG3A.0100) | ODI | -0.16 | 2.60E-20 | 3.62E-17 | 3364 | unclassified | unclassified | unclassified | unclassified | Eubacteriales | Clostridia | Firmicutes |
| Eubacteriales sp. (HG3A.0311) | ODI | -0.159 | 4.52E-20 | 3.62E-17 | 3364 | unclassified | unclassified | unclassified | unclassified | Eubacteriales | Clostridia | Firmicutes |
| Blautia obeum (HG3A.0001) | ODI | 0.158 | 8.09E-20 | 4.32E-17 | 3364 | unclassified | Blautia obeum | Blautia | Lachnospiraceae | Eubacteriales | Clostridia | Firmicutes |
| Eubacteriales sp. (HG3A.0162) | T90 | -0.154 | 6.74E-19 | 3.60E-16 | 3364 | unclassified | unclassified | unclassified | unclassified | Eubacteriales | Clostridia | Firmicutes |
| Eubacteriales sp. (HG3A.0149) | ODI | -0.152 | 1.63E-18 | 6.55E-16 | 3364 | unclassified | unclassified | unclassified | unclassified | Eubacteriales | Clostridia | Firmicutes |
| Eubacteriales sp. (HG3A.0162) | ODI | -0.151 | 2.39E-18 | 7.67E-16 | 3364 | unclassified | unclassified | unclassified | unclassified | Eubacteriales | Clostridia | Firmicutes |
| Clostridia sp. (HG3A.0140) | ODI | -0.15 | 4.78E-18 | 1.28E-15 | 3364 | unclassified | unclassified | unclassified | unclassified | unclassified | Clostridia | Firmicutes |
| Oscillospiraceae sp. (HG3A.0072) | ODI | -0.149 | 8.96E-18 | 1.85E-15 | 3364 | unclassified | unclassified | unclassified | Oscillospiraceae | Eubacteriales | Clostridia | Firmicutes |
| Oscillospiraceae sp. (HG3A.0223) | ODI | -0.149 | 9.26E-18 | 1.85E-15 | 3364 | unclassified | unclassified | unclassified | Oscillospiraceae | Eubacteriales | Clostridia | Firmicutes |
| Blautia massiliensis (HG3A.0023) | ODI | 0.148 | 1.29E-17 | 2.07E-15 | 3364 | unclassified | Blautia massiliensis | Blautia | Lachnospiraceae | Eubacteriales | Clostridia | Firmicutes |
| Oscillospiraceae sp. (HG3A.0207) | ODI | -0.148 | 1.28E-17 | 2.07E-15 | 3364 | unclassified | unclassified | unclassified | Oscillospiraceae | Eubacteriales | Clostridia | Firmicutes |
| Eubacteriales sp. (HG3A.0242) | T90 | -0.147 | 2.40E-17 | 9.62E-15 | 3364 | unclassified | unclassified | unclassified | unclassified | Eubacteriales | Clostridia | Firmicutes |
| Dorea formicigenerans  (HG3A.0006) | ODI | 0.144 | 7.22E-17 | 1.05E-14 | 3364 | unclassified | Dorea  formicigenerans | Dorea | Lachnospiraceae | Eubacteriales | Clostridia | Firmicutes |
| Eubacteriales sp. (HG3A.0242) | ODI | -0.143 | 1.77E-16 | 2.36E-14 | 3364 | unclassified | unclassified | unclassified | unclassified | Eubacteriales | Clostridia | Firmicutes |
| Eubacteriales sp. (HG3A.0069) | ODI | -0.141 | 3.51E-16 | 4.33E-14 | 3364 | unclassified | unclassified | unclassified | unclassified | Eubacteriales | Clostridia | Firmicutes |
| Eubacteriales sp. (HG3A.0311) | T90 | -0.143 | 1.67E-16 | 5.37E-14 | 3364 | unclassified | unclassified | unclassified | unclassified | Eubacteriales | Clostridia | Firmicutes |

T90 0.166 7.86E-22 1.26E-18 3364 unclassified Dorea

formicigenerans

Dorea Lachnospiraceae Eubacteriales Clostridia Firmicutes

| Eubacteriales sp. (HG3A.0100) | T90 | -0.142 | 2.37E-16 | 6.32E-14 | 3364 | unclassified | unclassified | unclassified | unclassified | Eubacteriales | Clostridia | Firmicutes |
| --- | --- | --- | --- | --- | --- | --- | --- | --- | --- | --- | --- | --- |
| Eubacteriales sp. (HG3A.0118) | ODI | -0.14 | 7.04E-16 | 8.05E-14 | 3364 | unclassified | unclassified | unclassified | unclassified | Eubacteriales | Clostridia | Firmicutes |
| Clostridia sp. (HG3A.0140) | T90 | -0.14 | 5.16E-16 | 1.16E-13 | 3364 | unclassified | unclassified | unclassified | unclassified | unclassified | Clostridia | Firmicutes |
| Eubacteriales sp. (HG3A.0149) | T90 | -0.14 | 5.79E-16 | 1.16E-13 | 3364 | unclassified | unclassified | unclassified | unclassified | Eubacteriales | Clostridia | Firmicutes |
| Intestinimonas massiliensis  (HG3A.0198) | ODI | -0.137 | 2.47E-15 | 2.63E-13 | 3364 | unclassified | Intestinimonas  massiliensis | Intestinimonas | unclassified | Eubacteriales | Clostridia | Firmicutes |
| Coprococcus comes (HG3A.0016) | ODI | 0.136 | 3.56E-15 | 3.36E-13 | 3364 | unclassified | Coprococcus comes | Coprococcus | Lachnospiraceae | Eubacteriales | Clostridia | Firmicutes |
| Roseburia inulinivorans  (HG3A.0036) | ODI | 0.136 | 3.42E-15 | 3.36E-13 | 3364 | unclassified | Roseburia  inulinivorans | Roseburia | Lachnospiraceae | Eubacteriales | Clostridia | Firmicutes |
| Oscillospiraceae sp. (HG3A.0207) | T90 | -0.137 | 2.10E-15 | 3.74E-13 | 3364 | unclassified | unclassified | unclassified | Oscillospiraceae | Eubacteriales | Clostridia | Firmicutes |
| Eubacteriales sp. (HG3A.0331) | ODI | -0.136 | 4.60E-15 | 4.10E-13 | 3364 | unclassified | unclassified | unclassified | unclassified | Eubacteriales | Clostridia | Firmicutes |
| Mediterraneibacter glycyrrhizinilyticus (HG3A.0314) | T90 | 0.135 | 5.23E-15 | 8.38E-13 | 3364 | unclassified | Mediterraneibacter glycyrrhizinilyticus | Mediterraneibacter | Lachnospiraceae | Eubacteriales | Clostridia | Firmicutes |
| Eubacteriales sp. (HG3A.0506) | ODI | -0.132 | 2.21E-14 | 1.86E-12 | 3364 | unclassified | unclassified | unclassified | unclassified | Eubacteriales | Clostridia | Firmicutes |
| [Ruminococcus] gnavus (HG3A.0239) | ODI | 0.131 | 4.06E-14 | 3.25E-12 | 3364 | unclassified | [Ruminococcus] gnavus | Mediterraneibacter | Lachnospiraceae | Eubacteriales | Clostridia | Firmicutes |
| Oscillospiraceae sp. (HG3A.0072) | T90 | -0.131 | 3.47E-14 | 5.06E-12 | 3364 | unclassified | unclassified | unclassified | Oscillospiraceae | Eubacteriales | Clostridia | Firmicutes |
| Eubacteriales sp. (HG3A.0421) | ODI | -0.13 | 6.80E-14 | 5.19E-12 | 3364 | unclassified | unclassified | unclassified | unclassified | Eubacteriales | Clostridia | Firmicutes |
| Eubacteriales sp. (HG3A.0083) | ODI | -0.129 | 7.96E-14 | 5.79E-12 | 3364 | unclassified | unclassified | unclassified | unclassified | Eubacteriales | Clostridia | Firmicutes |
| Collinsella aerofaciens (HG3A.0019) | ODI | 0.129 | 9.87E-14 | 6.88E-12 | 3364 | unclassified | Collinsella aerofaciens | Collinsella | Coriobacteriaceae | Coriobacteriales | Coriobacteriia | Actinobacteria |
| Firmicutes sp. (HG3A.0397) | ODI | -0.129 | 1.04E-13 | 6.91E-12 | 3364 | unclassified | unclassified | unclassified | unclassified | unclassified | unclassified | Firmicutes |
| Eubacteriales sp. (HG3A.0118) | T90 | -0.13 | 5.74E-14 | 7.67E-12 | 3364 | unclassified | unclassified | unclassified | unclassified | Eubacteriales | Clostridia | Firmicutes |
| Eubacteriales sp. (HG3A.0383) | ODI | -0.128 | 1.51E-13 | 9.65E-12 | 3364 | unclassified | unclassified | unclassified | unclassified | Eubacteriales | Clostridia | Firmicutes |
| Eubacteriales sp. (HG3A.0373) | T90 | -0.129 | 9.56E-14 | 1.18E-11 | 3364 | unclassified | unclassified | unclassified | unclassified | Eubacteriales | Clostridia | Firmicutes |
| Eubacteriales sp. (HG3A.0311) | AHI | -0.142 | 9.34E-15 | 1.50E-11 | 3004 | unclassified | unclassified | unclassified | unclassified | Eubacteriales | Clostridia | Firmicutes |
| Eubacteriales sp. (HG3A.0162) | AHI | -0.14 | 2.12E-14 | 1.70E-11 | 3004 | unclassified | unclassified | unclassified | unclassified | Eubacteriales | Clostridia | Firmicutes |

| Eubacteriales sp. (HG3A.0211) | ODI | -0.126 | 3.28E-13 | 2.02E-11 | 3364 | unclassified | unclassified | unclassified | unclassified | Eubacteriales | Clostridia | Firmicutes |
| --- | --- | --- | --- | --- | --- | --- | --- | --- | --- | --- | --- | --- |
| Eubacteriales sp. (HG3A.0442) | ODI | -0.125 | 4.84E-13 | 2.87E-11 | 3364 | unclassified | unclassified | unclassified | unclassified | Eubacteriales | Clostridia | Firmicutes |
| Eubacteriales sp. (HG3A.0100) | AHI | -0.136 | 1.02E-13 | 5.46E-11 | 3004 | unclassified | unclassified | unclassified | unclassified | Eubacteriales | Clostridia | Firmicutes |
| Eubacteriales sp. (HG3A.0197) | ODI | -0.124 | 1.04E-12 | 5.75E-11 | 3364 | unclassified | unclassified | unclassified | unclassified | Eubacteriales | Clostridia | Firmicutes |
| Eubacteriales sp. (HG3A.0321) | ODI | -0.124 | 1.02E-12 | 5.75E-11 | 3364 | unclassified | unclassified | unclassified | unclassified | Eubacteriales | Clostridia | Firmicutes |
| Clostridia sp. (HG3A.0470) | ODI | -0.123 | 1.17E-12 | 6.18E-11 | 3364 | unclassified | unclassified | unclassified | unclassified | unclassified | Clostridia | Firmicutes |
| Clostridium sp. TF06-15AC  (HG3A.0032) | ODI | 0.123 | 1.20E-12 | 6.18E-11 | 3364 | unclassified | Clostridium sp. TF06-  15AC | Clostridium | Clostridiaceae | Eubacteriales | Clostridia | Firmicutes |
| Clostridia sp. (HG3A.0470) | T90 | -0.125 | 6.21E-13 | 6.63E-11 | 3364 | unclassified | unclassified | unclassified | unclassified | unclassified | Clostridia | Firmicutes |
| Eubacteriales sp. (HG3A.0383) | T90 | -0.125 | 5.87E-13 | 6.63E-11 | 3364 | unclassified | unclassified | unclassified | unclassified | Eubacteriales | Clostridia | Firmicutes |
| Firmicutes sp. (HG3A.0398) | ODI | -0.123 | 1.34E-12 | 6.72E-11 | 3364 | unclassified | unclassified | unclassified | unclassified | unclassified | unclassified | Firmicutes |
| Collinsella aerofaciens  (HG3A.0019) | T90 | 0.124 | 6.86E-13 | 6.87E-11 | 3364 | unclassified | Collinsella  aerofaciens | Collinsella | Coriobacteriaceae | Coriobacteriales | Coriobacteriia | Actinobacteria |
| Bacteria sp. (HG3A.0483) | ODI | -0.123 | 1.46E-12 | 7.10E-11 | 3364 | unclassified | unclassified | unclassified | unclassified | unclassified | unclassified | unclassified |
| Oscillospiraceae sp. (HG3A.0437) | ODI | -0.123 | 1.57E-12 | 7.38E-11 | 3364 | unclassified | unclassified | unclassified | Oscillospiraceae | Eubacteriales | Clostridia | Firmicutes |
| [Ruminococcus] gnavus T90 0.124 8.62E-13 8.13E-11 3364 unclassified [Ruminococcus] Mediterraneibacter Lachnospiraceae Eubacteriales Clostridia Firmicutes | | | | | | | | | | | | |
| (HG3A.0239) |  |  |  |  |  |  | gnavus |  |  |  |  |  |
| Eubacteriales sp. (HG3A.0125) | ODI | -0.122 | 1.99E-12 | 8.87E-11 | 3364 | unclassified | unclassified | unclassified | unclassified | Eubacteriales | Clostridia | Firmicutes |
| Eubacteriales sp. (HG3A.0316) | ODI | -0.122 | 1.95E-12 | 8.87E-11 | 3364 | unclassified | unclassified | unclassified | unclassified | Eubacteriales | Clostridia | Firmicutes |
| Blautia massiliensis (HG3A.0023) | T90 | 0.124 | 1.04E-12 | 9.27E-11 | 3364 | unclassified | Blautia massiliensis | Blautia | Lachnospiraceae | Eubacteriales | Clostridia | Firmicutes |
| Mediterraneibacter |  |  |  |  |  |  | Mediterraneibacter |  |  |  |  |  |
| glycyrrhizinilyticus (HG3A.0314) | ODI | 0.121 | 3.31E-12 | 1.43E-10 | 3364 | unclassified | glycyrrhizinilyticus Mediterraneibacter | | Lachnospiraceae | Eubacteriales | Clostridia | Firmicutes |
| Eubacteriales sp. (HG3A.0069) | AHI | -0.132 | 5.31E-13 | 1.70E-10 | 3004 | unclassified | unclassified | unclassified | unclassified | Eubacteriales | Clostridia | Firmicutes |
| Oscillospiraceae sp. (HG3A.0072) | AHI | -0.133 | 4.52E-13 | 1.70E-10 | 3004 | unclassified | unclassified | unclassified | Oscillospiraceae | Eubacteriales | Clostridia | Firmicutes |
| Oscillospiraceae sp. (HG3A.0207) | AHI | -0.132 | 6.42E-13 | 1.71E-10 | 3004 | unclassified | unclassified | unclassified | Oscillospiraceae | Eubacteriales | Clostridia | Firmicutes |
| Coprococcus comes (HG3A.0016) | T90 | 0.122 | 2.11E-12 | 1.78E-10 | 3364 | unclassified | Coprococcus comes | Coprococcus | Lachnospiraceae | Eubacteriales | Clostridia | Firmicutes |

| Eubacteriales sp. (HG3A.1379) | T90 | -0.122 | 2.29E-12 | 1.84E-10 | 3364 | unclassified | unclassified | unclassified | unclassified | Eubacteriales | Clostridia | Firmicutes |
| --- | --- | --- | --- | --- | --- | --- | --- | --- | --- | --- | --- | --- |
| Oscillospiraceae sp. (HG3A.0223) | T90 | -0.121 | 2.69E-12 | 2.05E-10 | 3364 | unclassified | unclassified | unclassified | Oscillospiraceae | Eubacteriales | Clostridia | Firmicutes |
| Firmicutes sp. (HG3A.0341) | ODI | -0.12 | 5.48E-12 | 2.31E-10 | 3364 | unclassified | unclassified | unclassified | unclassified | unclassified | unclassified | Firmicutes |
| Eubacteriales sp. (HG3A.0269) | ODI | -0.119 | 7.19E-12 | 2.95E-10 | 3364 | unclassified | unclassified | unclassified | unclassified | Eubacteriales | Clostridia | Firmicutes |
| Eubacteriales sp. (HG3A.0193) | ODI | -0.119 | 7.67E-12 | 3.01E-10 | 3364 | unclassified | unclassified | unclassified | unclassified | Eubacteriales | Clostridia | Firmicutes |
| Eubacteriales sp. (HG3A.0600) | ODI | -0.119 | 7.69E-12 | 3.01E-10 | 3364 | unclassified | unclassified | unclassified | unclassified | Eubacteriales | Clostridia | Firmicutes |
| Clostridia sp. (HG3A.0508) | ODI | -0.118 | 8.74E-12 | 3.33E-10 | 3364 | unclassified | unclassified | unclassified | unclassified | unclassified | Clostridia | Firmicutes |
| Clostridia sp. (HG3A.0385) | T90 | -0.12 | 4.82E-12 | 3.36E-10 | 3364 | unclassified | unclassified | unclassified | unclassified | unclassified | Clostridia | Firmicutes |
| Eubacteriales sp. (HG3A.0531) | T90 | -0.12 | 4.75E-12 | 3.36E-10 | 3364 | unclassified | unclassified | unclassified | unclassified | Eubacteriales | Clostridia | Firmicutes |
| Eubacteriales sp. (HG3A.0226) | ODI | -0.118 | 1.07E-11 | 3.99E-10 | 3364 | unclassified | unclassified | unclassified | unclassified | Eubacteriales | Clostridia | Firmicutes |
| Fusicatenibacter saccharivorans (HG3A.0004) | ODI | 0.118 | 1.17E-11 | 4.26E-10 | 3364 | unclassified | Fusicatenibacter saccharivorans | Fusicatenibacter | Lachnospiraceae | Eubacteriales | Clostridia | Firmicutes |
| Clostridiaceae sp. (HG3A.0431) | T90 | 0.119 | 7.24E-12 | 4.83E-10 | 3364 | unclassified | unclassified | unclassified | Clostridiaceae | Eubacteriales | Clostridia | Firmicutes |
| Eubacteriales sp. (HG3A.0120) | ODI | -0.117 | 1.41E-11 | 5.01E-10 | 3364 | unclassified | unclassified | unclassified | unclassified | Eubacteriales | Clostridia | Firmicutes |
| Blautia obeum (HG3A.0009) | T90 | 0.119 | 8.09E-12 | 5.04E-10 | 3364 | unclassified | Blautia obeum | Blautia | Lachnospiraceae | Eubacteriales | Clostridia | Firmicutes |
| Clostridia sp. (HG3A.0508) | T90 | -0.119 | 8.21E-12 | 5.04E-10 | 3364 | unclassified | unclassified | unclassified | unclassified | unclassified | Clostridia | Firmicutes |
| Eubacteriales sp. (HG3A.0600) | T90 | -0.118 | 8.50E-12 | 5.04E-10 | 3364 | unclassified | unclassified | unclassified | unclassified | Eubacteriales | Clostridia | Firmicutes |
| Clostridia sp. (HG3A.0385) | ODI | -0.116 | 1.87E-11 | 6.51E-10 | 3364 | unclassified | unclassified | unclassified | unclassified | unclassified | Clostridia | Firmicutes |
| Clostridia sp. (HG3A.0435) | ODI | -0.116 | 2.19E-11 | 7.45E-10 | 3364 | unclassified | unclassified | unclassified | unclassified | unclassified | Clostridia | Firmicutes |
| Eubacterium sp. AM49-13BH  (HG3A.0251) | ODI | -0.116 | 2.30E-11 | 7.67E-10 | 3364 | unclassified | Eubacterium sp.  AM49-13BH | Eubacterium | Eubacteriaceae | Eubacteriales | Clostridia | Firmicutes |
| Clostridia sp. (HG3A.0435) | T90 | -0.117 | 1.34E-11 | 7.68E-10 | 3364 | unclassified | unclassified | unclassified | unclassified | unclassified | Clostridia | Firmicutes |
| Blautia obeum (HG3A.0001) | AHI | 0.128 | 3.58E-12 | 8.17E-10 | 3004 | unclassified | Blautia obeum | Blautia | Lachnospiraceae | Eubacteriales | Clostridia | Firmicutes |

| Oscillospiraceae sp. (HG3A.0223) | AHI | -0.127 | 4.08E-12 | 8.17E-10 | 3004 | unclassified | unclassified | unclassified | Oscillospiraceae | Eubacteriales | Clostridia | Firmicutes |
| --- | --- | --- | --- | --- | --- | --- | --- | --- | --- | --- | --- | --- |
| Eubacteriales sp. (HG3A.0196) | ODI | -0.116 | 2.63E-11 | 8.60E-10 | 3364 | unclassified | unclassified | unclassified | unclassified | Eubacteriales | Clostridia | Firmicutes |
| Eubacteriales sp. (HG3A.0125) | T90 | -0.117 | 1.64E-11 | 9.06E-10 | 3364 | unclassified | unclassified | unclassified | unclassified | Eubacteriales | Clostridia | Firmicutes |
| Clostridia sp. (HG3A.0550) | T90 | -0.116 | 2.03E-11 | 1.05E-09 | 3364 | unclassified | unclassified | unclassified | unclassified | unclassified | Clostridia | Firmicutes |
| Eubacteriales sp. (HG3A.0321) | T90 | -0.116 | 2.00E-11 | 1.05E-09 | 3364 | unclassified | unclassified | unclassified | unclassified | Eubacteriales | Clostridia | Firmicutes |
| Eubacteriales sp. (HG3A.0506) | T90 | -0.116 | 2.16E-11 | 1.08E-09 | 3364 | unclassified | unclassified | unclassified | unclassified | Eubacteriales | Clostridia | Firmicutes |
| Firmicutes sp. (HG3A.0397) | AHI | -0.126 | 6.85E-12 | 1.22E-09 | 3004 | unclassified | unclassified | unclassified | unclassified | unclassified | unclassified | Firmicutes |
| Clostridium sp. AT4 (HG3A.0347) | T90 | 0.116 | 2.54E-11 | 1.23E-09 | 3364 | unclassified | Clostridium sp. AT4 | Clostridium | Clostridiaceae | Eubacteriales | Clostridia | Firmicutes |
| Blautia massiliensis (HG3A.0023) | AHI | 0.125 | 8.27E-12 | 1.33E-09 | 3004 | unclassified | Blautia massiliensis | Blautia | Lachnospiraceae | Eubacteriales | Clostridia | Firmicutes |
| Eubacteriales sp. (HG3A.0373) | ODI | -0.114 | 4.51E-11 | 1.44E-09 | 3364 | unclassified | unclassified | unclassified | unclassified | Eubacteriales | Clostridia | Firmicutes |
| Flavonifractor plautii (HG3A.0079) | T90 | 0.115 | 3.07E-11 | 1.44E-09 | 3364 | unclassified | Flavonifractor plautii | Flavonifractor | Oscillospiraceae | Eubacteriales | Clostridia | Firmicutes |
| Eubacteriales sp. (HG3A.0118) | AHI | -0.125 | 9.93E-12 | 1.45E-09 | 3004 | unclassified | unclassified | unclassified | unclassified | Eubacteriales | Clostridia | Firmicutes |
| Eubacteriales sp. (HG3A.0149) | AHI | -0.124 | 1.39E-11 | 1.86E-09 | 3004 | unclassified | unclassified | unclassified | unclassified | Eubacteriales | Clostridia | Firmicutes |
| Eubacteriales sp. (HG3A.0084) | ODI | -0.113 | 6.03E-11 | 1.90E-09 | 3364 | unclassified | unclassified | unclassified | unclassified | Eubacteriales | Clostridia | Firmicutes |
| Intestinimonas massiliensis (HG3A.0198) | AHI | -0.124 | 1.55E-11 | 1.91E-09 | 3004 | unclassified | Intestinimonas massiliensis | Intestinimonas | unclassified | Eubacteriales | Clostridia | Firmicutes |
| Dorea sp. AF36-15AT  (HG3A.0052) | T90 | 0.114 | 4.55E-11 | 2.08E-09 | 3364 | unclassified | Dorea sp. AF36-  15AT | Dorea | Lachnospiraceae | Eubacteriales | Clostridia | Firmicutes |
| Alistipes shahii (HG3A.0054) | ODI | -0.113 | 6.91E-11 | 2.13E-09 | 3364 | unclassified | Alistipes shahii | Alistipes | Rikenellaceae | Bacteroidales | Bacteroidia | Bacteroidetes |
| Eubacteriales sp. (HG3A.0421) | AHI | -0.123 | 2.17E-11 | 2.32E-09 | 3004 | unclassified | unclassified | unclassified | unclassified | Eubacteriales | Clostridia | Firmicutes |
| Firmicutes sp. (HG3A.0398) | AHI | -0.123 | 2.13E-11 | 2.32E-09 | 3004 | unclassified | unclassified | unclassified | unclassified | unclassified | unclassified | Firmicutes |
| Eubacteriales sp. (HG3A.0092) | ODI | -0.112 | 8.99E-11 | 2.72E-09 | 3364 | unclassified | unclassified | unclassified | unclassified | Eubacteriales | Clostridia | Firmicutes |
| Clostridia sp. (HG3A.0272) | ODI | -0.112 | 9.63E-11 | 2.86E-09 | 3364 | unclassified | unclassified | unclassified | unclassified | unclassified | Clostridia | Firmicutes |

Dorea formicigenerans (HG3A.0006)

AHI 0.122 2.86E-11 2.86E-09 3004 unclassified Dorea

formicigenerans

Dorea Lachnospiraceae Eubacteriales Clostridia Firmicutes

| Eubacteriales sp. (HG3A.0084) | AHI | -0.122 | 3.31E-11 | 3.12E-09 | 3004 | unclassified | unclassified | unclassified | unclassified | Eubacteriales | Clostridia | Firmicutes |
| --- | --- | --- | --- | --- | --- | --- | --- | --- | --- | --- | --- | --- |
| [Ruminococcus] torques  (HG3A.0034) | ODI | 0.112 | 1.09E-10 | 3.18E-09 | 3364 | unclassified | [Ruminococcus]  torques | Mediterraneibacter | Lachnospiraceae | Eubacteriales | Clostridia | Firmicutes |
| Eubacteriales sp. (HG3A.0085) | ODI | -0.112 | 1.12E-10 | 3.20E-09 | 3364 | unclassified | unclassified | unclassified | unclassified | Eubacteriales | Clostridia | Firmicutes |
| Intestinibacillus sp. Marseille- P4005 (HG3A.0168) | ODI | 0.112 | 1.16E-10 | 3.26E-09 | 3364 | unclassified | Intestinibacillus sp. Marseille-P4005 | Intestinibacillus | Eubacteriaceae | Eubacteriales | Clostridia | Firmicutes |
| Eubacteriales sp. (HG3A.0331) | T90 | -0.113 | 7.84E-11 | 3.49E-09 | 3364 | unclassified | unclassified | unclassified | unclassified | Eubacteriales | Clostridia | Firmicutes |
| Eubacteriales sp. (HG3A.0506) | AHI | -0.121 | 3.95E-11 | 3.51E-09 | 3004 | unclassified | unclassified | unclassified | unclassified | Eubacteriales | Clostridia | Firmicutes |
| Flavonifractor plautii (HG3A.0079) | ODI | 0.112 | 1.27E-10 | 3.51E-09 | 3364 | unclassified | Flavonifractor plautii | Flavonifractor | Oscillospiraceae | Eubacteriales | Clostridia | Firmicutes |
| Eubacteriales sp. (HG3A.0419) | T90 | -0.113 | 8.58E-11 | 3.71E-09 | 3364 | unclassified | unclassified | unclassified | unclassified | Eubacteriales | Clostridia | Firmicutes |
| [Ruminococcus] gnavus (HG3A.0239) | AHI | 0.121 | 4.84E-11 | 4.08E-09 | 3004 | unclassified | [Ruminococcus] gnavus | Mediterraneibacter | Lachnospiraceae | Eubacteriales | Clostridia | Firmicutes |
| Lachnospiraceae sp. (HG3A.0018) | ODI | 0.111 | 1.60E-10 | 4.33E-09 | 3364 | unclassified | unclassified | unclassified | Lachnospiraceae | Eubacteriales | Clostridia | Firmicutes |
| Eubacteriales sp. (HG3A.0156) | ODI | -0.111 | 1.68E-10 | 4.48E-09 | 3364 | unclassified | unclassified | unclassified | unclassified | Eubacteriales | Clostridia | Firmicutes |
| Clostridia sp. (HG3A.0094) | ODI | -0.11 | 1.98E-10 | 5.21E-09 | 3364 | unclassified | unclassified | unclassified | unclassified | unclassified | Clostridia | Firmicutes |
| Alistipes communis (HG3A.0064) | ODI | -0.11 | 2.03E-10 | 5.25E-09 | 3364 | unclassified | Alistipes communis | Alistipes | Rikenellaceae | Bacteroidales | Bacteroidia | Bacteroidetes |
| Victivallis vadensis (HG3A.0689) | ODI | -0.11 | 2.22E-10 | 5.65E-09 | 3364 | unclassified | Victivallis vadensis | Victivallis | Victivallaceae | Victivallales | Lentisphaeria | Lentisphaerae |
| Eubacteriales sp. (HG3A.0242) | AHI | -0.12 | 7.58E-11 | 6.07E-09 | 3004 | unclassified | unclassified | unclassified | unclassified | Eubacteriales | Clostridia | Firmicutes |
| Eubacteriales sp. (HG3A.0284) | ODI | -0.11 | 2.67E-10 | 6.68E-09 | 3364 | unclassified | unclassified | unclassified | unclassified | Eubacteriales | Clostridia | Firmicutes |
| Blautia obeum (HG3A.0009) | ODI | 0.109 | 2.80E-10 | 6.91E-09 | 3364 | unclassified | Blautia obeum | Blautia | Lachnospiraceae | Eubacteriales | Clostridia | Firmicutes |
| Eubacteriales sp. (HG3A.0179) | ODI | -0.109 | 2.93E-10 | 7.11E-09 | 3364 | unclassified | unclassified | unclassified | unclassified | Eubacteriales | Clostridia | Firmicutes |
| Clostridia sp. (HG3A.0140) | AHI | -0.119 | 1.03E-10 | 7.88E-09 | 3004 | unclassified | unclassified | unclassified | unclassified | unclassified | Clostridia | Firmicutes |
| Eubacteriales sp. (HG3A.0316) | T90 | -0.11 | 1.96E-10 | 8.26E-09 | 3364 | unclassified | unclassified | unclassified | unclassified | Eubacteriales | Clostridia | Firmicutes |
| Eubacteriales sp. (HG3A.0531) | ODI | -0.109 | 3.47E-10 | 8.29E-09 | 3364 | unclassified | unclassified | unclassified | unclassified | Eubacteriales | Clostridia | Firmicutes |

| Clostridia sp. (HG3A.0515) | ODI | -0.109 | 3.81E-10 | 8.78E-09 | 3364 | unclassified | unclassified | unclassified | unclassified | unclassified | Clostridia | Firmicutes |
| --- | --- | --- | --- | --- | --- | --- | --- | --- | --- | --- | --- | --- |
| Clostridium sp. AT4 (HG3A.0347) | ODI | 0.109 | 3.76E-10 | 8.78E-09 | 3364 | unclassified | Clostridium sp. AT4 | Clostridium | Clostridiaceae | Eubacteriales | Clostridia | Firmicutes |
| Oscillospiraceae sp. (HG3A.0060) | ODI | -0.109 | 3.84E-10 | 8.78E-09 | 3364 | unclassified | unclassified | unclassified | Oscillospiraceae | Eubacteriales | Clostridia | Firmicutes |
| Intestinimonas massiliensis (HG3A.0198) | T90 | -0.11 | 2.33E-10 | 9.58E-09 | 3364 | unclassified | Intestinimonas massiliensis | Intestinimonas | unclassified | Eubacteriales | Clostridia | Firmicutes |
| Collinsella aerofaciens  (HG3A.0019) | AHI | 0.118 | 1.40E-10 | 1.02E-08 | 3004 | unclassified | Collinsella  aerofaciens | Collinsella | Coriobacteriaceae | Coriobacteriales | Coriobacteriia | Actinobacteria |
| Firmicutes sp. (HG3A.0341) | AHI | -0.118 | 1.54E-10 | 1.07E-08 | 3004 | unclassified | unclassified | unclassified | unclassified | unclassified | unclassified | Firmicutes |
| Lachnospiraceae sp. (HG3A.0180) | ODI | -0.108 | 4.92E-10 | 1.11E-08 | 3364 | unclassified | unclassified | unclassified | Lachnospiraceae | Eubacteriales | Clostridia | Firmicutes |
| Bacteria sp. (HG3A.0634) | ODI | -0.108 | 5.22E-10 | 1.16E-08 | 3364 | unclassified | unclassified | unclassified | unclassified | unclassified | unclassified | unclassified |
| Eubacteriales sp. (HG3A.0083) | T90 | -0.109 | 2.98E-10 | 1.19E-08 | 3364 | unclassified | unclassified | unclassified | unclassified | Eubacteriales | Clostridia | Firmicutes |
| Clostridia sp. (HG3A.0815) | ODI | -0.108 | 5.53E-10 | 1.21E-08 | 3364 | unclassified | unclassified | unclassified | unclassified | unclassified | Clostridia | Firmicutes |
| Eubacteriales sp. (HG3A.0215) | ODI | -0.107 | 6.24E-10 | 1.35E-08 | 3364 | unclassified | unclassified | unclassified | unclassified | Eubacteriales | Clostridia | Firmicutes |
| Intestinibacillus sp. Marseille- P4005 (HG3A.0168) | T90 | 0.109 | 3.61E-10 | 1.41E-08 | 3364 | unclassified | Intestinibacillus sp. Marseille-P4005 | Intestinibacillus | Eubacteriaceae | Eubacteriales | Clostridia | Firmicutes |
| Eubacteriales sp. (HG3A.0085) | AHI | -0.117 | 2.28E-10 | 1.46E-08 | 3004 | unclassified | unclassified | unclassified | unclassified | Eubacteriales | Clostridia | Firmicutes |
| Eubacteriales sp. (HG3A.0125) | AHI | -0.117 | 2.19E-10 | 1.46E-08 | 3004 | unclassified | unclassified | unclassified | unclassified | Eubacteriales | Clostridia | Firmicutes |
| Clostridiaceae sp. (HG3A.0431) | ODI | 0.107 | 8.33E-10 | 1.78E-08 | 3364 | unclassified | unclassified | unclassified | Clostridiaceae | Eubacteriales | Clostridia | Firmicutes |
| Clostridia sp. (HG3A.0463) | ODI | -0.106 | 8.55E-10 | 1.80E-08 | 3364 | unclassified | unclassified | unclassified | unclassified | unclassified | Clostridia | Firmicutes |
| Eubacterium sp. AM49-13BH  (HG3A.0251) | AHI | -0.116 | 2.92E-10 | 1.80E-08 | 3004 | unclassified | Eubacterium sp.  AM49-13BH | Eubacterium | Eubacteriaceae | Eubacteriales | Clostridia | Firmicutes |
| Lachnospiraceae sp. (HG3A.0233) | ODI | -0.106 | 9.03E-10 | 1.88E-08 | 3364 | unclassified | unclassified | unclassified | Lachnospiraceae | Eubacteriales | Clostridia | Firmicutes |
| Eubacteriales sp. (HG3A.0083) | AHI | -0.115 | 3.51E-10 | 2.09E-08 | 3004 | unclassified | unclassified | unclassified | unclassified | Eubacteriales | Clostridia | Firmicutes |
| Clostridium sp. TF06-15AC (HG3A.0032) | T90 | 0.107 | 6.01E-10 | 2.29E-08 | 3364 | unclassified | Clostridium sp. TF06- 15AC | Clostridium | Clostridiaceae | Eubacteriales | Clostridia | Firmicutes |
| Oscillospiraceae sp. (HG3A.0437) | AHI | -0.115 | 4.05E-10 | 2.32E-08 | 3004 | unclassified | unclassified | unclassified | Oscillospiraceae | Eubacteriales | Clostridia | Firmicutes |

| Eubacteriales sp. (HG3A.0421) | T90 | -0.107 | 6.24E-10 | 2.33E-08 | 3364 | unclassified | unclassified | unclassified | unclassified | Eubacteriales | Clostridia | Firmicutes |
| --- | --- | --- | --- | --- | --- | --- | --- | --- | --- | --- | --- | --- |
| Eubacteriales sp. (HG3A.0197) | AHI | -0.115 | 4.42E-10 | 2.36E-08 | 3004 | unclassified | unclassified | unclassified | unclassified | Eubacteriales | Clostridia | Firmicutes |
| Eubacteriales sp. (HG3A.0284) | AHI | -0.115 | 4.37E-10 | 2.36E-08 | 3004 | unclassified | unclassified | unclassified | unclassified | Eubacteriales | Clostridia | Firmicutes |
| Eubacteriales sp. (HG3A.0211) | T90 | -0.107 | 6.52E-10 | 2.37E-08 | 3364 | unclassified | unclassified | unclassified | unclassified | Eubacteriales | Clostridia | Firmicutes |
| Oscillospiraceae sp. (HG3A.0429) | ODI | -0.106 | 1.17E-09 | 2.39E-08 | 3364 | unclassified | unclassified | unclassified | Oscillospiraceae | Eubacteriales | Clostridia | Firmicutes |
| Eubacteriales sp. (HG3A.0630) | T90 | -0.107 | 7.07E-10 | 2.52E-08 | 3364 | unclassified | unclassified | unclassified | unclassified | Eubacteriales | Clostridia | Firmicutes |
| Dorea sp. AF36-15AT (HG3A.0052) | ODI | 0.105 | 1.28E-09 | 2.56E-08 | 3364 | unclassified | Dorea sp. AF36- 15AT | Dorea | Lachnospiraceae | Eubacteriales | Clostridia | Firmicutes |
| Eubacteriales sp. (HG3A.1379) | ODI | -0.105 | 1.28E-09 | 2.56E-08 | 3364 | unclassified | unclassified | unclassified | unclassified | Eubacteriales | Clostridia | Firmicutes |
| Roseburia inulinivorans (HG3A.0036) | T90 | 0.107 | 7.56E-10 | 2.63E-08 | 3364 | unclassified | Roseburia inulinivorans | Roseburia | Lachnospiraceae | Eubacteriales | Clostridia | Firmicutes |
| Eubacteriales sp. (HG3A.0269) | T90 | -0.107 | 8.02E-10 | 2.73E-08 | 3364 | unclassified | unclassified | unclassified | unclassified | Eubacteriales | Clostridia | Firmicutes |
| Mediterraneibacter glycyrrhizinilyticus (HG3A.0314) | AHI | 0.114 | 5.49E-10 | 2.84E-08 | 3004 | unclassified | Mediterraneibacter glycyrrhizinilyticus | Mediterraneibacter | Lachnospiraceae | Eubacteriales | Clostridia | Firmicutes |
| Eubacteriales sp. (HG3A.0419) | ODI | -0.105 | 1.45E-09 | 2.87E-08 | 3364 | unclassified | unclassified | unclassified | unclassified | Eubacteriales | Clostridia | Firmicutes |
| Eubacteriales sp. (HG3A.0193) | AHI | -0.114 | 6.29E-10 | 3.15E-08 | 3004 | unclassified | unclassified | unclassified | unclassified | Eubacteriales | Clostridia | Firmicutes |
| Clostridia sp. (HG3A.0272) | T90 | -0.106 | 9.73E-10 | 3.18E-08 | 3364 | unclassified | unclassified | unclassified | unclassified | unclassified | Clostridia | Firmicutes |
| Firmicutes sp. (HG3A.0397) | T90 | -0.106 | 9.67E-10 | 3.18E-08 | 3364 | unclassified | unclassified | unclassified | unclassified | unclassified | unclassified | Firmicutes |
| [Ruminococcus] torques  (HG3A.0034) | T90 | 0.106 | 1.05E-09 | 3.37E-08 | 3364 | unclassified | [Ruminococcus]  torques | Mediterraneibacter | Lachnospiraceae | Eubacteriales | Clostridia | Firmicutes |
| Clostridia sp. (HG3A.0463) | T90 | -0.105 | 1.23E-09 | 3.85E-08 | 3364 | unclassified | unclassified | unclassified | unclassified | unclassified | Clostridia | Firmicutes |
| [Ruminococcus] torques  (HG3A.0088) | ODI | 0.104 | 2.01E-09 | 3.92E-08 | 3364 | unclassified | [Ruminococcus]  torques | Mediterraneibacter | Lachnospiraceae | Eubacteriales | Clostridia | Firmicutes |
| Anaerobutyricum hallii (HG3A.0012) | T90 | 0.105 | 1.29E-09 | 3.98E-08 | 3364 | unclassified | Anaerobutyricum hallii | Anaerobutyricum | Lachnospiraceae | Eubacteriales | Clostridia | Firmicutes |
| Clostridia sp. (HG3A.0682) | ODI | -0.104 | 2.15E-09 | 4.16E-08 | 3364 | unclassified | unclassified | unclassified | unclassified | unclassified | Clostridia | Firmicutes |
| Clostridia sp. (HG3A.0550) | ODI | -0.104 | 2.24E-09 | 4.22E-08 | 3364 | unclassified | unclassified | unclassified | unclassified | unclassified | Clostridia | Firmicutes |

| Oscillospiraceae sp. (HG3A.0693) | ODI | -0.104 | 2.24E-09 | 4.22E-08 | 3364 | unclassified | unclassified | unclassified | Oscillospiraceae | Eubacteriales | Clostridia | Firmicutes |
| --- | --- | --- | --- | --- | --- | --- | --- | --- | --- | --- | --- | --- |
| Eubacteriales sp. (HG3A.0154) | ODI | -0.104 | 2.35E-09 | 4.37E-08 | 3364 | unclassified | unclassified | unclassified | unclassified | Eubacteriales | Clostridia | Firmicutes |
| Eubacteriales sp. (HG3A.0477) | ODI | -0.104 | 2.38E-09 | 4.38E-08 | 3364 | unclassified | unclassified | unclassified | unclassified | Eubacteriales | Clostridia | Firmicutes |
| Firmicutes sp. (HG3A.0341) | T90 | -0.105 | 1.50E-09 | 4.54E-08 | 3364 | unclassified | unclassified | unclassified | unclassified | unclassified | unclassified | Firmicutes |
| Clostridium sp. (HG3A.0050) | ODI | 0.103 | 2.62E-09 | 4.77E-08 | 3364 | unclassified | unclassified | Clostridium | Clostridiaceae | Eubacteriales | Clostridia | Firmicutes |
| Eubacteriales sp. (HG3A.0342) | ODI | -0.103 | 2.92E-09 | 5.26E-08 | 3364 | unclassified | unclassified | unclassified | unclassified | Eubacteriales | Clostridia | Firmicutes |
| Lachnospiraceae sp. (HG3A.0399) | ODI | -0.103 | 2.97E-09 | 5.28E-08 | 3364 | unclassified | unclassified | unclassified | Lachnospiraceae | Eubacteriales | Clostridia | Firmicutes |
| Eubacteriales sp. (HG3A.0489) | T90 | -0.104 | 1.80E-09 | 5.33E-08 | 3364 | unclassified | unclassified | unclassified | unclassified | Eubacteriales | Clostridia | Firmicutes |
| Eubacteriales sp. (HG3A.0331) | AHI | -0.112 | 1.12E-09 | 5.41E-08 | 3004 | unclassified | unclassified | unclassified | unclassified | Eubacteriales | Clostridia | Firmicutes |
| Firmicutes sp. (HG3A.0398) | T90 | -0.104 | 1.86E-09 | 5.42E-08 | 3364 | unclassified | unclassified | unclassified | unclassified | unclassified | unclassified | Firmicutes |
| Oscillospiraceae sp. (HG3A.0210) | ODI | -0.103 | 3.09E-09 | 5.44E-08 | 3364 | unclassified | unclassified | unclassified | Oscillospiraceae | Eubacteriales | Clostridia | Firmicutes |
| Oscillospiraceae sp. (HG3A.0060) | AHI | -0.112 | 1.18E-09 | 5.54E-08 | 3004 | unclassified | unclassified | unclassified | Oscillospiraceae | Eubacteriales | Clostridia | Firmicutes |
| Eubacteriales sp. (HG3A.0363) | ODI | -0.102 | 3.52E-09 | 6.12E-08 | 3364 | unclassified | unclassified | unclassified | unclassified | Eubacteriales | Clostridia | Firmicutes |
| Eubacteriales sp. (HG3A.0250) | ODI | -0.102 | 4.00E-09 | 6.90E-08 | 3364 | unclassified | unclassified | unclassified | unclassified | Eubacteriales | Clostridia | Firmicutes |
| Eubacteriales sp. (HG3A.0196) | T90 | -0.103 | 2.53E-09 | 7.15E-08 | 3364 | unclassified | unclassified | unclassified | unclassified | Eubacteriales | Clostridia | Firmicutes |
| Oscillospiraceae sp. (HG3A.0437) | T90 | -0.103 | 2.55E-09 | 7.15E-08 | 3364 | unclassified | unclassified | unclassified | Oscillospiraceae | Eubacteriales | Clostridia | Firmicutes |
| Clostridia sp. (HG3A.0756) | ODI | -0.102 | 4.31E-09 | 7.34E-08 | 3364 | unclassified | unclassified | unclassified | unclassified | unclassified | Clostridia | Firmicutes |
| Eubacteriales sp. (HG3A.0156) | AHI | -0.111 | 1.65E-09 | 7.54E-08 | 3004 | unclassified | unclassified | unclassified | unclassified | Eubacteriales | Clostridia | Firmicutes |
| Dorea longicatena (HG3A.0039) | T90 | 0.103 | 2.77E-09 | 7.66E-08 | 3364 | unclassified | Dorea longicatena | Dorea | Lachnospiraceae | Eubacteriales | Clostridia | Firmicutes |
| Oscillospiraceae sp. (HG3A.0060) | T90 | -0.103 | 2.84E-09 | 7.71E-08 | 3364 | unclassified | unclassified | unclassified | Oscillospiraceae | Eubacteriales | Clostridia | Firmicutes |
| Eubacteriales sp. (HG3A.0161) | ODI | -0.102 | 4.69E-09 | 7.91E-08 | 3364 | unclassified | unclassified | unclassified | unclassified | Eubacteriales | Clostridia | Firmicutes |
| Lachnospiraceae sp. (HG3A.0233) | T90 | -0.102 | 3.95E-09 | 1.05E-07 | 3364 | unclassified | unclassified | unclassified | Lachnospiraceae | Eubacteriales | Clostridia | Firmicutes |

Odoribacter splanchnicus (HG3A.0041)

| Eubacteriales sp. (HG3A.0092) | T90 | -0.102 | 4.09E-09 | 1.07E-07 | 3364 | unclassified | unclassified | unclassified | unclassified | Eubacteriales | Clostridia | Firmicutes |
| --- | --- | --- | --- | --- | --- | --- | --- | --- | --- | --- | --- | --- |
| Eubacteriales sp. (HG3A.0229) | ODI | -0.101 | 6.61E-09 | 1.08E-07 | 3364 | unclassified | unclassified | unclassified | unclassified | Eubacteriales | Clostridia | Firmicutes |
| Eubacteriales sp. (HG3A.0381) | ODI | -0.101 | 6.52E-09 | 1.08E-07 | 3364 | unclassified | unclassified | unclassified | unclassified | Eubacteriales | Clostridia | Firmicutes |
| Clostridia sp. (HG3A.1053) | ODI | -0.101 | 6.91E-09 | 1.12E-07 | 3364 | unclassified | unclassified | unclassified | unclassified | unclassified | Clostridia | Firmicutes |
| Eggerthellales sp. (HG3A.0177) | ODI | -0.101 | 7.02E-09 | 1.12E-07 | 3364 | unclassified | unclassified | unclassified | unclassified | Eggerthellales | Coriobacteriia | Actinobacteria |
| Eubacteriales sp. (HG3A.0269) | AHI | -0.109 | 2.63E-09 | 1.14E-07 | 3004 | unclassified | unclassified | unclassified | unclassified | Eubacteriales | Clostridia | Firmicutes |
| Lachnospiraceae sp. (HG3A.0180) | AHI | -0.109 | 2.61E-09 | 1.14E-07 | 3004 | unclassified | unclassified | unclassified | Lachnospiraceae | Eubacteriales | Clostridia | Firmicutes |
| Eubacteriales sp. (HG3A.0144) | ODI | -0.1 | 7.98E-09 | 1.27E-07 | 3364 | unclassified | unclassified | unclassified | unclassified | Eubacteriales | Clostridia | Firmicutes |
| Eubacteriales sp. (HG3A.0856) | ODI | -0.1 | 9.01E-09 | 1.42E-07 | 3364 | unclassified | unclassified | unclassified | unclassified | Eubacteriales | Clostridia | Firmicutes |
| Eubacteriales sp. (HG3A.0363) | T90 | -0.101 | 5.62E-09 | 1.45E-07 | 3364 | unclassified | unclassified | unclassified | unclassified | Eubacteriales | Clostridia | Firmicutes |
| Eubacteriales sp. (HG3A.0703) | T90 | -0.101 | 6.06E-09 | 1.54E-07 | 3364 | unclassified | unclassified | unclassified | unclassified | Eubacteriales | Clostridia | Firmicutes |
| Blautia sp. (HG3A.0416) | ODI | 0.099 | 1.01E-08 | 1.56E-07 | 3364 | unclassified | unclassified | Blautia | Lachnospiraceae | Eubacteriales | Clostridia | Firmicutes |
| Eubacteriales sp. (HG3A.0338) | ODI | -0.099 | 1.04E-08 | 1.60E-07 | 3364 | unclassified | unclassified | unclassified | unclassified | Eubacteriales | Clostridia | Firmicutes |
| Lactobacillus gasseri (HG3A.0884) | ODI | 0.099 | 1.05E-08 | 1.60E-07 | 3364 | unclassified | Lactobacillus gasseri | Lactobacillus | Lactobacillaceae | Lactobacillales | Bacilli | Firmicutes |
| Eubacteriales sp. (HG3A.0069) | T90 | -0.101 | 6.53E-09 | 1.63E-07 | 3364 | unclassified | unclassified | unclassified | unclassified | Eubacteriales | Clostridia | Firmicutes |
| Dorea longicatena (HG3A.0039) | ODI | 0.099 | 1.09E-08 | 1.65E-07 | 3364 | unclassified | Dorea longicatena | Dorea | Lachnospiraceae | Eubacteriales | Clostridia | Firmicutes |
| Eubacteriales sp. (HG3A.0572) | ODI | -0.099 | 1.13E-08 | 1.69E-07 | 3364 | unclassified | unclassified | unclassified | unclassified | Eubacteriales | Clostridia | Firmicutes |
| Eubacterium sp. AF16-48  (HG3A.0219) | T90 | -0.101 | 6.98E-09 | 1.72E-07 | 3364 | unclassified | Eubacterium sp.  AF16-48 | Eubacterium | Eubacteriaceae | Eubacteriales | Clostridia | Firmicutes |
| Eubacteriales sp. (HG3A.0338) | T90 | -0.1 | 7.24E-09 | 1.76E-07 | 3364 | unclassified | unclassified | unclassified | unclassified | Eubacteriales | Clostridia | Firmicutes |
| Coprococcus comes (HG3A.0016) | AHI | 0.108 | 4.21E-09 | 1.77E-07 | 3004 | unclassified | Coprococcus comes | Coprococcus | Lachnospiraceae | Eubacteriales | Clostridia | Firmicutes |
| Roseburia inulinivorans (HG3A.0036) | AHI | 0.108 | 4.52E-09 | 1.86E-07 | 3004 | unclassified | Roseburia inulinivorans | Roseburia | Lachnospiraceae | Eubacteriales | Clostridia | Firmicutes |

ODI -0.101 6.37E-09 1.06E-07 3364 unclassified Odoribacter

splanchnicus

Odoribacter Odoribacteraceae Bacteroidales Bacteroidia Bacteroidetes

| Blautia sp. (HG3A.0416) | T90 | 0.1 | 8.01E-09 | 1.92E-07 | 3364 | unclassified | unclassified | Blautia | Lachnospiraceae | Eubacteriales | Clostridia | Firmicutes |
| --- | --- | --- | --- | --- | --- | --- | --- | --- | --- | --- | --- | --- |
| Eubacteriales sp. (HG3A.0312) | T90 | -0.1 | 8.26E-09 | 1.95E-07 | 3364 | unclassified | unclassified | unclassified | unclassified | Eubacteriales | Clostridia | Firmicutes |
| Alistipes senegalensis  (HG3A.0141) | ODI | -0.099 | 1.38E-08 | 2.05E-07 | 3364 | unclassified | Alistipes  senegalensis | Alistipes | Rikenellaceae | Bacteroidales | Bacteroidia | Bacteroidetes |
| Eubacteriales sp. (HG3A.0234) | T90 | -0.1 | 9.00E-09 | 2.09E-07 | 3364 | unclassified | unclassified | unclassified | unclassified | Eubacteriales | Clostridia | Firmicutes |
| Eubacteriales sp. (HG3A.0426) | T90 | -0.1 | 9.48E-09 | 2.14E-07 | 3364 | unclassified | unclassified | unclassified | unclassified | Eubacteriales | Clostridia | Firmicutes |
| Sellimonas intestinalis (HG3A.0417) | T90 | 0.1 | 9.42E-09 | 2.14E-07 | 3364 | unclassified | Sellimonas intestinalis | Sellimonas | Lachnospiraceae | Eubacteriales | Clostridia | Firmicutes |
| Eubacteriales sp. (HG3A.0443) | ODI | -0.098 | 1.47E-08 | 2.16E-07 | 3364 | unclassified | unclassified | unclassified | unclassified | Eubacteriales | Clostridia | Firmicutes |
| Alistipes shahii (HG3A.0054) | AHI | -0.107 | 5.74E-09 | 2.30E-07 | 3004 | unclassified | Alistipes shahii | Alistipes | Rikenellaceae | Bacteroidales | Bacteroidia | Bacteroidetes |
| Eubacteriales sp. (HG3A.0226) | AHI | -0.107 | 6.07E-09 | 2.37E-07 | 3004 | unclassified | unclassified | unclassified | unclassified | Eubacteriales | Clostridia | Firmicutes |
| Eubacteriales sp. (HG3A.0312) | ODI | -0.098 | 1.63E-08 | 2.37E-07 | 3364 | unclassified | unclassified | unclassified | unclassified | Eubacteriales | Clostridia | Firmicutes |
| Eubacteriales sp. (HG3A.0249) | ODI | -0.098 | 1.67E-08 | 2.42E-07 | 3364 | unclassified | unclassified | unclassified | unclassified | Eubacteriales | Clostridia | Firmicutes |
| Eubacteriales sp. (HG3A.0442) | T90 | -0.099 | 1.09E-08 | 2.43E-07 | 3364 | unclassified | unclassified | unclassified | unclassified | Eubacteriales | Clostridia | Firmicutes |
| Clostridia sp. (HG3A.0741) | ODI | -0.098 | 1.74E-08 | 2.49E-07 | 3364 | unclassified | unclassified | unclassified | unclassified | unclassified | Clostridia | Firmicutes |
| Eubacteriales sp. (HG3A.0120) | T90 | -0.099 | 1.16E-08 | 2.52E-07 | 3364 | unclassified | unclassified | unclassified | unclassified | Eubacteriales | Clostridia | Firmicutes |
| Eubacteriales sp. (HG3A.0144) | T90 | -0.099 | 1.16E-08 | 2.52E-07 | 3364 | unclassified | unclassified | unclassified | unclassified | Eubacteriales | Clostridia | Firmicutes |
| Eubacteriales sp. (HG3A.0474) | T90 | -0.099 | 1.19E-08 | 2.54E-07 | 3364 | unclassified | unclassified | unclassified | unclassified | Eubacteriales | Clostridia | Firmicutes |
| Eubacteriales sp. (HG3A.1086) | ODI | -0.098 | 1.81E-08 | 2.57E-07 | 3364 | unclassified | unclassified | unclassified | unclassified | Eubacteriales | Clostridia | Firmicutes |
| Oscillospiraceae sp. (HG3A.0384) | ODI | -0.098 | 1.83E-08 | 2.57E-07 | 3364 | unclassified | unclassified | unclassified | Oscillospiraceae | Eubacteriales | Clostridia | Firmicutes |
| Eubacteriales sp. (HG3A.0120) | AHI | -0.106 | 7.14E-09 | 2.72E-07 | 3004 | unclassified | unclassified | unclassified | unclassified | Eubacteriales | Clostridia | Firmicutes |
| Eubacteriales sp. (HG3A.0123) | T90 | 0.099 | 1.29E-08 | 2.72E-07 | 3364 | unclassified | unclassified | unclassified | unclassified | Eubacteriales | Clostridia | Firmicutes |
| Bacteria sp. (HG3A.0483) | T90 | -0.098 | 1.42E-08 | 2.96E-07 | 3364 | unclassified | unclassified | unclassified | unclassified | unclassified | unclassified | unclassified |
| Butyricicoccus sp. (HG3A.0008) | ODI | 0.097 | 2.13E-08 | 2.97E-07 | 3364 | unclassified | unclassified | Butyricicoccus | Clostridiaceae | Eubacteriales | Clostridia | Firmicutes |

| Clostridia sp. (HG3A.0094) | AHI | -0.106 | 7.98E-09 | 2.97E-07 | 3004 | unclassified | unclassified | unclassified | unclassified | unclassified | Clostridia | Firmicutes |
| --- | --- | --- | --- | --- | --- | --- | --- | --- | --- | --- | --- | --- |
| Clostridia sp. (HG3A.0728) | ODI | -0.097 | 2.32E-08 | 3.20E-07 | 3364 | unclassified | unclassified | unclassified | unclassified | unclassified | Clostridia | Firmicutes |
| Clostridia sp. (HG3A.0515) | AHI | -0.106 | 9.24E-09 | 3.29E-07 | 3004 | unclassified | unclassified | unclassified | unclassified | unclassified | Clostridia | Firmicutes |
| Fusicatenibacter saccharivorans (HG3A.0004) | AHI | 0.106 | 9.23E-09 | 3.29E-07 | 3004 | unclassified | Fusicatenibacter saccharivorans | Fusicatenibacter | Lachnospiraceae | Eubacteriales | Clostridia | Firmicutes |
| Firmicutes sp. (HG3A.0454) | T90 | -0.098 | 1.64E-08 | 3.37E-07 | 3364 | unclassified | unclassified | unclassified | unclassified | unclassified | unclassified | Firmicutes |
| Lachnospiraceae sp. (HG3A.0233) | AHI | -0.105 | 9.83E-09 | 3.42E-07 | 3004 | unclassified | unclassified | unclassified | Lachnospiraceae | Eubacteriales | Clostridia | Firmicutes |
| Clostridia sp. (HG3A.0515) | T90 | -0.098 | 1.73E-08 | 3.45E-07 | 3364 | unclassified | unclassified | unclassified | unclassified | unclassified | Clostridia | Firmicutes |
| Clostridia sp. (HG3A.0815) | T90 | -0.098 | 1.71E-08 | 3.45E-07 | 3364 | unclassified | unclassified | unclassified | unclassified | unclassified | Clostridia | Firmicutes |
| Clostridium sp. TF06-15AC  (HG3A.0032) | AHI | 0.105 | 1.09E-08 | 3.56E-07 | 3004 | unclassified | Clostridium sp. TF06-  15AC | Clostridium | Clostridiaceae | Eubacteriales | Clostridia | Firmicutes |
| Eubacteriales sp. (HG3A.0235) | AHI | -0.105 | 1.05E-08 | 3.56E-07 | 3004 | unclassified | unclassified | unclassified | unclassified | Eubacteriales | Clostridia | Firmicutes |
| Eubacteriales sp. (HG3A.0321) | AHI | -0.105 | 1.09E-08 | 3.56E-07 | 3004 | unclassified | unclassified | unclassified | unclassified | Eubacteriales | Clostridia | Firmicutes |
| Clostridia sp. (HG3A.0724) | ODI | -0.096 | 3.04E-08 | 4.13E-07 | 3364 | unclassified | unclassified | unclassified | unclassified | unclassified | Clostridia | Firmicutes |
| Eubacteriales sp. (HG3A.0153) | ODI | -0.096 | 3.04E-08 | 4.13E-07 | 3364 | unclassified | unclassified | unclassified | unclassified | Eubacteriales | Clostridia | Firmicutes |
| Eubacteriales sp. (HG3A.0196) | AHI | -0.105 | 1.29E-08 | 4.14E-07 | 3004 | unclassified | unclassified | unclassified | unclassified | Eubacteriales | Clostridia | Firmicutes |
| Eubacteriales sp. (HG3A.0442) | AHI | -0.104 | 1.32E-08 | 4.15E-07 | 3004 | unclassified | unclassified | unclassified | unclassified | Eubacteriales | Clostridia | Firmicutes |
| Clostridia sp. (HG3A.0599) | ODI | -0.096 | 3.22E-08 | 4.31E-07 | 3364 | unclassified | unclassified | unclassified | unclassified | unclassified | Clostridia | Firmicutes |
| Firmicutes sp. (HG3A.0454) | ODI | -0.096 | 3.23E-08 | 4.31E-07 | 3364 | unclassified | unclassified | unclassified | unclassified | unclassified | unclassified | Firmicutes |
| Clostridia sp. (HG3A.1020) | ODI | -0.096 | 3.26E-08 | 4.32E-07 | 3364 | unclassified | unclassified | unclassified | unclassified | unclassified | Clostridia | Firmicutes |
| Eubacteriales sp. (HG3A.0284) | T90 | -0.097 | 2.25E-08 | 4.45E-07 | 3364 | unclassified | unclassified | unclassified | unclassified | Eubacteriales | Clostridia | Firmicutes |
| Clostridia sp. (HG3A.0724) | AHI | -0.104 | 1.45E-08 | 4.47E-07 | 3004 | unclassified | unclassified | unclassified | unclassified | unclassified | Clostridia | Firmicutes |
| Clostridia sp. (HG3A.0470) | AHI | -0.104 | 1.48E-08 | 4.49E-07 | 3004 | unclassified | unclassified | unclassified | unclassified | unclassified | Clostridia | Firmicutes |

| Clostridia sp. (HG3A.0682) | AHI | -0.104 | 1.52E-08 | 4.50E-07 | 3004 | unclassified | unclassified | unclassified | unclassified | unclassified | Clostridia | Firmicutes |
| --- | --- | --- | --- | --- | --- | --- | --- | --- | --- | --- | --- | --- |
| Clostridia sp. (HG3A.1053) | AHI | -0.104 | 1.58E-08 | 4.61E-07 | 3004 | unclassified | unclassified | unclassified | unclassified | unclassified | Clostridia | Firmicutes |
| Eubacteriales sp. (HG3A.0316) | AHI | -0.104 | 1.66E-08 | 4.76E-07 | 3004 | unclassified | unclassified | unclassified | unclassified | Eubacteriales | Clostridia | Firmicutes |
| Eubacterium sp. AM49-13BH  (HG3A.0251) | T90 | -0.097 | 2.44E-08 | 4.76E-07 | 3364 | unclassified | Eubacterium sp.  AM49-13BH | Eubacterium | Eubacteriaceae | Eubacteriales | Clostridia | Firmicutes |
| Eubacteriales sp. (HG3A.0235) | ODI | -0.095 | 4.05E-08 | 5.32E-07 | 3364 | unclassified | unclassified | unclassified | unclassified | Eubacteriales | Clostridia | Firmicutes |
| Bacteria sp. (HG3A.0483) | AHI | -0.103 | 1.96E-08 | 5.40E-07 | 3004 | unclassified | unclassified | unclassified | unclassified | unclassified | unclassified | unclassified |
| Eubacteriales sp. (HG3A.0092) | AHI | -0.103 | 1.97E-08 | 5.40E-07 | 3004 | unclassified | unclassified | unclassified | unclassified | Eubacteriales | Clostridia | Firmicutes |
| Eubacteriales sp. (HG3A.0383) | AHI | -0.103 | 1.99E-08 | 5.40E-07 | 3004 | unclassified | unclassified | unclassified | unclassified | Eubacteriales | Clostridia | Firmicutes |
| Clostridium sp. (HG3A.0050) | T90 | 0.096 | 2.83E-08 | 5.47E-07 | 3364 | unclassified | unclassified | Clostridium | Clostridiaceae | Eubacteriales | Clostridia | Firmicutes |
| Eubacteriales sp. (HG3A.0405) | ODI | -0.095 | 4.22E-08 | 5.49E-07 | 3364 | unclassified | unclassified | unclassified | unclassified | Eubacteriales | Clostridia | Firmicutes |
| Butyricicoccus sp. (HG3A.0008) | T90 | 0.096 | 2.93E-08 | 5.59E-07 | 3364 | unclassified | unclassified | Butyricicoccus | Clostridiaceae | Eubacteriales | Clostridia | Firmicutes |
| Eubacteriales sp. (HG3A.0396) | T90 | -0.096 | 3.21E-08 | 6.04E-07 | 3364 | unclassified | unclassified | unclassified | unclassified | Eubacteriales | Clostridia | Firmicutes |
| Eubacteriales sp. (HG3A.0771) | T90 | -0.096 | 3.31E-08 | 6.17E-07 | 3364 | unclassified | unclassified | unclassified | unclassified | Eubacteriales | Clostridia | Firmicutes |
| Eubacteriales sp. (HG3A.0113) | ODI | -0.095 | 4.99E-08 | 6.45E-07 | 3364 | unclassified | unclassified | unclassified | unclassified | Eubacteriales | Clostridia | Firmicutes |
| Eubacteriales sp. (HG3A.0179) | AHI | -0.103 | 2.47E-08 | 6.46E-07 | 3004 | unclassified | unclassified | unclassified | unclassified | Eubacteriales | Clostridia | Firmicutes |
| Eubacteriales sp. (HG3A.0179) | T90 | -0.096 | 3.54E-08 | 6.46E-07 | 3364 | unclassified | unclassified | unclassified | unclassified | Eubacteriales | Clostridia | Firmicutes |
| Eubacteriales sp. (HG3A.0211) | AHI | -0.102 | 2.50E-08 | 6.46E-07 | 3004 | unclassified | unclassified | unclassified | unclassified | Eubacteriales | Clostridia | Firmicutes |
| Eubacteriales sp. (HG3A.1379) | AHI | -0.102 | 2.49E-08 | 6.46E-07 | 3004 | unclassified | unclassified | unclassified | unclassified | Eubacteriales | Clostridia | Firmicutes |
| Lachnospiraceae sp. (HG3A.0180) | T90 | -0.096 | 3.55E-08 | 6.46E-07 | 3364 | unclassified | unclassified | unclassified | Lachnospiraceae | Eubacteriales | Clostridia | Firmicutes |
| Eubacteriales sp. (HG3A.0653) | ODI | -0.095 | 5.13E-08 | 6.57E-07 | 3364 | unclassified | unclassified | unclassified | unclassified | Eubacteriales | Clostridia | Firmicutes |
| Clostridia sp. (HG3A.0508) | AHI | -0.102 | 2.59E-08 | 6.58E-07 | 3004 | unclassified | unclassified | unclassified | unclassified | unclassified | Clostridia | Firmicutes |
| Oscillospiraceae sp. (HG3A.0429) | AHI | -0.102 | 2.63E-08 | 6.58E-07 | 3004 | unclassified | unclassified | unclassified | Oscillospiraceae | Eubacteriales | Clostridia | Firmicutes |

| Eubacteriales sp. (HG3A.0578) | ODI | -0.094 | 5.40E-08 | 6.86E-07 | 3364 | unclassified | unclassified | unclassified | unclassified | Eubacteriales | Clostridia | Firmicutes |
| --- | --- | --- | --- | --- | --- | --- | --- | --- | --- | --- | --- | --- |
| Oscillospiraceae sp. (HG3A.0429) | T90 | -0.095 | 4.03E-08 | 7.26E-07 | 3364 | unclassified | unclassified | unclassified | Oscillospiraceae | Eubacteriales | Clostridia | Firmicutes |
| Clostridia sp. (HG3A.0368) | ODI | -0.094 | 6.46E-08 | 8.15E-07 | 3364 | unclassified | unclassified | unclassified | unclassified | unclassified | Clostridia | Firmicutes |
| Clostridia sp. (HG3A.0787) | ODI | -0.094 | 6.53E-08 | 8.17E-07 | 3364 | unclassified | unclassified | unclassified | unclassified | unclassified | Clostridia | Firmicutes |
| Eubacteriales sp. (HG3A.0381) | AHI | -0.102 | 3.38E-08 | 8.33E-07 | 3004 | unclassified | unclassified | unclassified | unclassified | Eubacteriales | Clostridia | Firmicutes |
| Clostridium sp. AT4 (HG3A.0347) | AHI | 0.101 | 3.72E-08 | 9.03E-07 | 3004 | unclassified | Clostridium sp. AT4 | Clostridium | Clostridiaceae | Eubacteriales | Clostridia | Firmicutes |
| Eubacteriales sp. (HG3A.0856) | T90 | -0.094 | 5.97E-08 | 1.05E-06 | 3364 | unclassified | unclassified | unclassified | unclassified | Eubacteriales | Clostridia | Firmicutes |
| Oscillospiraceae sp. (HG3A.0774) | T90 | -0.094 | 5.94E-08 | 1.05E-06 | 3364 | unclassified | unclassified | unclassified | Oscillospiraceae | Eubacteriales | Clostridia | Firmicutes |
| Bacteria sp. (HG3A.0634) | T90 | -0.094 | 6.14E-08 | 1.07E-06 | 3364 | unclassified | unclassified | unclassified | unclassified | unclassified | unclassified | unclassified |
| Alistipes shahii (HG3A.0054) | T90 | -0.094 | 6.60E-08 | 1.14E-06 | 3364 | unclassified | Alistipes shahii | Alistipes | Rikenellaceae | Bacteroidales | Bacteroidia | Bacteroidetes |
| Eubacteriales sp. (HG3A.0426) | ODI | -0.092 | 1.17E-07 | 1.45E-06 | 3364 | unclassified | unclassified | unclassified | unclassified | Eubacteriales | Clostridia | Firmicutes |
| Eubacteriales sp. (HG3A.0978) | T90 | -0.093 | 8.53E-08 | 1.45E-06 | 3364 | unclassified | unclassified | unclassified | unclassified | Eubacteriales | Clostridia | Firmicutes |
| Flavonifractor plautii (HG3A.0079) | AHI | 0.1 | 6.16E-08 | 1.47E-06 | 3004 | unclassified | Flavonifractor plautii | Flavonifractor | Oscillospiraceae | Eubacteriales | Clostridia | Firmicutes |
| Oscillospiraceae sp. (HG3A.0388) | ODI | -0.092 | 1.21E-07 | 1.49E-06 | 3364 | unclassified | unclassified | unclassified | Oscillospiraceae | Eubacteriales | Clostridia | Firmicutes |
| Eubacteriales sp. (HG3A.0312) | AHI | -0.099 | 6.39E-08 | 1.50E-06 | 3004 | unclassified | unclassified | unclassified | unclassified | Eubacteriales | Clostridia | Firmicutes |
| Clostridiaceae sp. (HG3A.0431) | AHI | 0.099 | 7.16E-08 | 1.66E-06 | 3004 | unclassified | unclassified | unclassified | Clostridiaceae | Eubacteriales | Clostridia | Firmicutes |
| Clostridium sp. OF03-18AA (HG3A.0119) | T90 | -0.093 | 9.96E-08 | 1.66E-06 | 3364 | unclassified | Clostridium sp. OF03 18AA | Clostridium | Clostridiaceae | Eubacteriales | Clostridia | Firmicutes |
| Eubacteriales sp. (HG3A.0443) | T90 | -0.092 | 1.01E-07 | 1.66E-06 | 3364 | unclassified | unclassified | unclassified | unclassified | Eubacteriales | Clostridia | Firmicutes |
| Lachnospiraceae sp. (HG3A.0018) | T90 | 0.092 | 1.01E-07 | 1.66E-06 | 3364 | unclassified | unclassified | unclassified | Lachnospiraceae | Eubacteriales | Clostridia | Firmicutes |
| Oscillospiraceae sp. (HG3A.0210) | T90 | -0.092 | 1.00E-07 | 1.66E-06 | 3364 | unclassified | unclassified | unclassified | Oscillospiraceae | Eubacteriales | Clostridia | Firmicutes |
| Eubacteriales sp. (HG3A.0373) | AHI | -0.099 | 7.31E-08 | 1.67E-06 | 3004 | unclassified | unclassified | unclassified | unclassified | Eubacteriales | Clostridia | Firmicutes |
| Eubacteriales sp. (HG3A.0489) | ODI | -0.091 | 1.37E-07 | 1.67E-06 | 3364 | unclassified | unclassified | unclassified | unclassified | Eubacteriales | Clostridia | Firmicutes |

| Oscillospiraceae sp. (HG3A.0343) | ODI | -0.091 | 1.37E-07 | 1.67E-06 | 3364 | unclassified | unclassified | unclassified | Oscillospiraceae | Eubacteriales | Clostridia | Firmicutes |
| --- | --- | --- | --- | --- | --- | --- | --- | --- | --- | --- | --- | --- |
| Eubacteriales sp. (HG3A.0249) | AHI | -0.099 | 7.43E-08 | 1.68E-06 | 3004 | unclassified | unclassified | unclassified | unclassified | Eubacteriales | Clostridia | Firmicutes |

Ruminococcus sp. AM42-11

(HG3A.0002)

T90

0.092

1.06E-07 1.71E-06 3364 unclassified

Ruminococcus sp.

AM42-11

Ruminococcus Oscillospiraceae Eubacteriales

Clostridia

Firmicutes

Roseburia intestinalis

(HG3A.0078)

ODI 0.091 1.43E-07 1.72E-06 3364 unclassified Roseburia intestinalis Roseburia Lachnospiraceae Eubacteriales Clostridia Firmicutes

Oscillospiraceae sp. (HG3A.0461) ODI -0.091 1.58E-07 1.89E-06 3364 unclassified unclassified unclassified Oscillospiraceae Eubacteriales Clostridia Firmicutes

Blautia obeum (HG3A.0009) AHI 0.098 8.83E-08 1.96E-06 3004 unclassified Blautia obeum Blautia Lachnospiraceae Eubacteriales Clostridia Firmicutes

Eubacteriales sp. (HG3A.0188) ODI -0.091 1.67E-07 1.98E-06 3364 unclassified unclassified unclassified unclassified Eubacteriales Clostridia Firmicutes

Clostridia sp. (HG3A.0741) AHI -0.098 9.27E-08 2.03E-06 3004 unclassified unclassified unclassified unclassified unclassified Clostridia Firmicutes

Akkermansia muciniphila

(HG3A.0110)

ODI

-0.091

1.84E-07 2.16E-06 3364 unclassified

Akkermansia

muciniphila

Akkermansia Akkermansiaceae Verrucomicrobiales Verrucomicrobi Verrucomicrobi

ae a

| Eubacteriales sp. (HG3A.0329) | ODI | -0.091 | 1.86E-07 | 2.17E-06 | 3364 | unclassified | unclassified | unclassified | unclassified | Eubacteriales | Clostridia | Firmicutes |
| --- | --- | --- | --- | --- | --- | --- | --- | --- | --- | --- | --- | --- |
| Eubacteriales sp. (HG3A.0771) | ODI | -0.09 | 1.95E-07 | 2.26E-06 | 3364 | unclassified | unclassified | unclassified | unclassified | Eubacteriales | Clostridia | Firmicutes |
| Firmicutes sp. (HG3A.0596) | ODI | -0.09 | 1.97E-07 | 2.27E-06 | 3364 | unclassified | unclassified | unclassified | unclassified | unclassified | unclassified | Firmicutes |

[Clostridium] innocuum

(HG3A.0365)

ODI

0.09

2.12E-07 2.41E-06 3364 unclassified

[Clostridium]

innocuum

Erysipelatoclostridiu Erysipelotrichace

m

ae

Erysipelotrichales Erysipelotrichia Firmicutes

Coprococcus eutactus

(HG3A.0155)

ODI -0.09 2.12E-07 2.41E-06 3364 unclassified Coprococcus

eutactus

Coprococcus Lachnospiraceae Eubacteriales Clostridia Firmicutes

Eubacteriales sp. (HG3A.0264)

ODI

-0.09

2.14E-07 2.41E-06 3364 unclassified

unclassified

unclassified

unclassified

Eubacteriales

Clostridia

Firmicutes

Clostridia sp. (HG3A.0879) ODI -0.09 2.17E-07 2.43E-06 3364 unclassified unclassified unclassified unclassified unclassified Clostridia Firmicutes

Oscillospiraceae sp. (HG3A.0384) T90

-0.091

1.52E-07 2.44E-06 3364 unclassified

unclassified

unclassified

Oscillospiraceae Eubacteriales

Clostridia

Firmicutes

Oscillospiraceae sp. (HG3A.0210) AHI -0.098 1.14E-07 2.46E-06 3004 unclassified unclassified unclassified Oscillospiraceae Eubacteriales Clostridia Firmicutes

Eubacteriales sp. (HG3A.0600)

AHI

-0.097

1.21E-07 2.59E-06 3004 unclassified

unclassified

unclassified

unclassified

Eubacteriales

Clostridia

Firmicutes

Clostridia sp. (HG3A.0893) ODI -0.09 2.36E-07 2.63E-06 3364 unclassified unclassified unclassified unclassified unclassified Clostridia Firmicutes

Blautia hydrogenotrophica

(HG3A.0430)

T90

0.091

1.74E-07 2.69E-06 3364 unclassified

Blautia

hydrogenotrophica

Blautia

Lachnospiraceae Eubacteriales

Clostridia

Firmicutes

Clostridia sp. (HG3A.0861) T90 -0.091 1.71E-07 2.69E-06 3364 unclassified unclassified unclassified unclassified unclassified Clostridia Firmicutes

| Eubacteriales sp. (HG3A.0084) | T90 | -0.091 | 1.73E-07 | 2.69E-06 | 3364 | unclassified | unclassified | unclassified | unclassified | Eubacteriales | Clostridia | Firmicutes |
| --- | --- | --- | --- | --- | --- | --- | --- | --- | --- | --- | --- | --- |
| Eubacteriales sp. (HG3A.0418) | T90 | -0.091 | 1.75E-07 | 2.69E-06 | 3364 | unclassified | unclassified | unclassified | unclassified | Eubacteriales | Clostridia | Firmicutes |
| Amedibacillus dolichus (HG3A.0798) | ODI | 0.09 | 2.51E-07 | 2.77E-06 | 3364 | unclassified | Amedibacillus dolichus | Amedibacillus | Erysipelotrichace ae | Erysipelotrichales | Erysipelotrichia | Firmicutes |
| Eubacteriales sp. (HG3A.0405) | T90 | -0.091 | 1.82E-07 | 2.78E-06 | 3364 | unclassified | unclassified | unclassified | unclassified | Eubacteriales | Clostridia | Firmicutes |
| Eubacteriales sp. (HG3A.0305) | ODI | -0.089 | 2.61E-07 | 2.86E-06 | 3364 | unclassified | unclassified | unclassified | unclassified | Eubacteriales | Clostridia | Firmicutes |
| Intestinibacillus sp. Marseille- P4005 (HG3A.0168) | AHI | 0.097 | 1.36E-07 | 2.87E-06 | 3004 | unclassified | Intestinibacillus sp. Marseille-P4005 | Intestinibacillus | Eubacteriaceae | Eubacteriales | Clostridia | Firmicutes |
| Alistipes communis (HG3A.0064) | AHI | -0.097 | 1.40E-07 | 2.88E-06 | 3004 | unclassified | Alistipes communis | Alistipes | Rikenellaceae | Bacteroidales | Bacteroidia | Bacteroidetes |
| [Ruminococcus] torques  (HG3A.0088) | AHI | 0.097 | 1.40E-07 | 2.88E-06 | 3004 | unclassified | [Ruminococcus]  torques | Mediterraneibacter | Lachnospiraceae | Eubacteriales | Clostridia | Firmicutes |
| Eubacteriales sp. (HG3A.0671) | ODI | -0.089 | 2.79E-07 | 3.02E-06 | 3364 | unclassified | unclassified | unclassified | unclassified | Eubacteriales | Clostridia | Firmicutes |
| Lactobacillus gasseri (HG3A.0884) | AHI | 0.097 | 1.49E-07 | 3.02E-06 | 3004 | unclassified | Lactobacillus gasseri | Lactobacillus | Lactobacillaceae | Lactobacillales | Bacilli | Firmicutes |
| Oscillospiraceae sp. (HG3A.1270) | ODI | -0.089 | 2.77E-07 | 3.02E-06 | 3364 | unclassified | unclassified | unclassified | Oscillospiraceae | Eubacteriales | Clostridia | Firmicutes |
| Eubacteriales sp. (HG3A.0234) | ODI | -0.089 | 2.84E-07 | 3.05E-06 | 3364 | unclassified | unclassified | unclassified | unclassified | Eubacteriales | Clostridia | Firmicutes |
| Eubacteriales sp. (HG3A.0630) | ODI | -0.089 | 2.86E-07 | 3.06E-06 | 3364 | unclassified | unclassified | unclassified | unclassified | Eubacteriales | Clostridia | Firmicutes |
| Eubacteriales sp. (HG3A.0352) | ODI | -0.089 | 2.94E-07 | 3.12E-06 | 3364 | unclassified | unclassified | unclassified | unclassified | Eubacteriales | Clostridia | Firmicutes |
| Oscillibacter sp. (HG3A.0245) | ODI | -0.089 | 2.97E-07 | 3.13E-06 | 3364 | unclassified | unclassified | Oscillibacter | Oscillospiraceae | Eubacteriales | Clostridia | Firmicutes |
| Eubacteriales sp. (HG3A.0309) | ODI | -0.089 | 3.08E-07 | 3.22E-06 | 3364 | unclassified | unclassified | unclassified | unclassified | Eubacteriales | Clostridia | Firmicutes |
| Firmicutes sp. (HG3A.0681) | ODI | -0.089 | 3.12E-07 | 3.24E-06 | 3364 | unclassified | unclassified | unclassified | unclassified | unclassified | unclassified | Firmicutes |
| Clostridia sp. (HG3A.0435) | AHI | -0.096 | 1.67E-07 | 3.34E-06 | 3004 | unclassified | unclassified | unclassified | unclassified | unclassified | Clostridia | Firmicutes |
| [Ruminococcus] torques (HG3A.0034) | AHI | 0.096 | 1.71E-07 | 3.39E-06 | 3004 | unclassified | [Ruminococcus] torques | Mediterraneibacter | Lachnospiraceae | Eubacteriales | Clostridia | Firmicutes |
| Clostridia sp. (HG3A.0756) | AHI | -0.096 | 1.78E-07 | 3.47E-06 | 3004 | unclassified | unclassified | unclassified | unclassified | unclassified | Clostridia | Firmicutes |
| Eubacteriales sp. (HG3A.0249) | T90 | -0.09 | 2.30E-07 | 3.47E-06 | 3364 | unclassified | unclassified | unclassified | unclassified | Eubacteriales | Clostridia | Firmicutes |

Clostridium sp. TM06-18 (HG3A.0048)

ODI 0.089 3.36E-07 3.48E-06 3364 unclassified Clostridium sp.

TM06-18

Clostridium Clostridiaceae Eubacteriales Clostridia Firmicutes

| [Ruminococcus] torques (HG3A.0088) | T90 | 0.09 | 2.41E-07 | 3.61E-06 | 3364 | unclassified | [Ruminococcus] torques | Mediterraneibacter | Lachnospiraceae | Eubacteriales | Clostridia | Firmicutes |
| --- | --- | --- | --- | --- | --- | --- | --- | --- | --- | --- | --- | --- |
| Clostridia sp. (HG3A.1057) | T90 | -0.09 | 2.46E-07 | 3.65E-06 | 3364 | unclassified | unclassified | unclassified | unclassified | unclassified | Clostridia | Firmicutes |
| Eubacteriales sp. (HG3A.0197) | T90 | -0.09 | 2.49E-07 | 3.66E-06 | 3364 | unclassified | unclassified | unclassified | unclassified | Eubacteriales | Clostridia | Firmicutes |
| Eubacteriales sp. (HG3A.0396) | ODI | -0.088 | 3.87E-07 | 3.95E-06 | 3364 | unclassified | unclassified | unclassified | unclassified | Eubacteriales | Clostridia | Firmicutes |
| Eubacteriales sp. (HG3A.0418) | ODI | -0.088 | 3.86E-07 | 3.95E-06 | 3364 | unclassified | unclassified | unclassified | unclassified | Eubacteriales | Clostridia | Firmicutes |
| Bacteria sp. (HG3A.0634) | AHI | -0.096 | 2.06E-07 | 3.97E-06 | 3004 | unclassified | unclassified | unclassified | unclassified | unclassified | unclassified | unclassified |
| Eubacteriales sp. (HG3A.0409) | ODI | -0.088 | 4.35E-07 | 4.41E-06 | 3364 | unclassified | unclassified | unclassified | unclassified | Eubacteriales | Clostridia | Firmicutes |
| Eubacteriales sp. (HG3A.0371) | ODI | -0.088 | 4.60E-07 | 4.64E-06 | 3364 | unclassified | unclassified | unclassified | unclassified | Eubacteriales | Clostridia | Firmicutes |
| Firmicutes sp. (HG3A.0301) | T90 | -0.089 | 3.21E-07 | 4.68E-06 | 3364 | unclassified | unclassified | unclassified | unclassified | unclassified | unclassified | Firmicutes |
| Anaerobutyricum hallii  (HG3A.0012) | ODI | 0.087 | 4.76E-07 | 4.74E-06 | 3364 | unclassified | Anaerobutyricum  hallii | Anaerobutyricum | Lachnospiraceae | Eubacteriales | Clostridia | Firmicutes |
| Firmicutes sp. (HG3A.0650) | ODI | -0.087 | 4.73E-07 | 4.74E-06 | 3364 | unclassified | unclassified | unclassified | unclassified | unclassified | unclassified | Firmicutes |
| Clostridia sp. (HG3A.0368) | T90 | -0.089 | 3.29E-07 | 4.75E-06 | 3364 | unclassified | unclassified | unclassified | unclassified | unclassified | Clostridia | Firmicutes |
| Lachnospiraceae sp. (HG3A.0236) | ODI | -0.087 | 4.82E-07 | 4.77E-06 | 3364 | unclassified | unclassified | unclassified | Lachnospiraceae | Eubacteriales | Clostridia | Firmicutes |
| Streptococcus parasanguinis (HG3A.0117) | ODI | 0.087 | 4.97E-07 | 4.88E-06 | 3364 | unclassified | Streptococcus parasanguinis | Streptococcus | Streptococcaceae | Lactobacillales | Bacilli | Firmicutes |
| Victivallis vadensis (HG3A.0689) | T90 | -0.089 | 3.42E-07 | 4.89E-06 | 3364 | unclassified | Victivallis vadensis | Victivallis | Victivallaceae | Victivallales | Lentisphaeria | Lentisphaerae |
| Eubacteriales sp. (HG3A.0474) | ODI | -0.087 | 5.09E-07 | 4.97E-06 | 3364 | unclassified | unclassified | unclassified | unclassified | Eubacteriales | Clostridia | Firmicutes |
| Eubacteriales sp. (HG3A.0790) | T90 | -0.088 | 3.54E-07 | 5.00E-06 | 3364 | unclassified | unclassified | unclassified | unclassified | Eubacteriales | Clostridia | Firmicutes |
| Oscillospiraceae sp. (HG3A.0256) | T90 | 0.088 | 3.56E-07 | 5.00E-06 | 3364 | unclassified | unclassified | unclassified | Oscillospiraceae | Eubacteriales | Clostridia | Firmicutes |
| Eubacteriales sp. (HG3A.0718) | ODI | -0.087 | 5.28E-07 | 5.10E-06 | 3364 | unclassified | unclassified | unclassified | unclassified | Eubacteriales | Clostridia | Firmicutes |

Eubacterium sp. AF16-48

(HG3A.0219)

ODI -0.087 5.29E-07 5.10E-06 3364 unclassified Eubacterium sp.

AF16-48

Eubacterium Eubacteriaceae Eubacteriales Clostridia Firmicutes

| Eubacteriales sp. (HG3A.0653) | AHI | -0.095 | 2.68E-07 | 5.11E-06 | 3004 | unclassified | unclassified | unclassified | unclassified | Eubacteriales | Clostridia | Firmicutes |
| --- | --- | --- | --- | --- | --- | --- | --- | --- | --- | --- | --- | --- |
| Eubacteriales sp. (HG3A.0352) | T90 | -0.088 | 3.99E-07 | 5.56E-06 | 3364 | unclassified | unclassified | unclassified | unclassified | Eubacteriales | Clostridia | Firmicutes |
| Eubacteriales sp. (HG3A.0453) | ODI | -0.087 | 5.98E-07 | 5.73E-06 | 3364 | unclassified | unclassified | unclassified | unclassified | Eubacteriales | Clostridia | Firmicutes |
| Anaerostipes caccae (HG3A.0747) | ODI | 0.087 | 6.06E-07 | 5.76E-06 | 3364 | unclassified | Anaerostipes caccae | Anaerostipes | Lachnospiraceae | Eubacteriales | Clostridia | Firmicutes |
| Clostridia sp. (HG3A.0724) | T90 | -0.088 | 4.17E-07 | 5.76E-06 | 3364 | unclassified | unclassified | unclassified | unclassified | unclassified | Clostridia | Firmicutes |
| Eubacteriales sp. (HG3A.0613) | ODI | -0.087 | 6.07E-07 | 5.76E-06 | 3364 | unclassified | unclassified | unclassified | unclassified | Eubacteriales | Clostridia | Firmicutes |
| Eubacteriales sp. (HG3A.0250) | AHI | -0.094 | 3.08E-07 | 5.81E-06 | 3004 | unclassified | unclassified | unclassified | unclassified | Eubacteriales | Clostridia | Firmicutes |
| Firmicutes sp. (HG3A.0681) | AHI | -0.094 | 3.18E-07 | 5.92E-06 | 3004 | unclassified | unclassified | unclassified | unclassified | unclassified | unclassified | Firmicutes |
| Odoribacter splanchnicus (HG3A.0041) | AHI | -0.094 | 3.26E-07 | 5.99E-06 | 3004 | unclassified | Odoribacter splanchnicus | Odoribacter | Odoribacteraceae | Bacteroidales | Bacteroidia | Bacteroidetes |
| Eubacteriales sp. (HG3A.0305) | T90 | -0.088 | 4.38E-07 | 6.00E-06 | 3364 | unclassified | unclassified | unclassified | unclassified | Eubacteriales | Clostridia | Firmicutes |
| Clostridia sp. (HG3A.0733) | ODI | -0.086 | 6.47E-07 | 6.09E-06 | 3364 | unclassified | unclassified | unclassified | unclassified | unclassified | Clostridia | Firmicutes |
| Eubacteriales sp. (HG3A.0857) | ODI | -0.086 | 6.50E-07 | 6.09E-06 | 3364 | unclassified | unclassified | unclassified | unclassified | Eubacteriales | Clostridia | Firmicutes |
| Eubacteriales sp. (HG3A.0593) | T90 | -0.088 | 4.55E-07 | 6.17E-06 | 3364 | unclassified | unclassified | unclassified | unclassified | Eubacteriales | Clostridia | Firmicutes |
| Clostridia sp. (HG3A.0521) | T90 | -0.088 | 4.67E-07 | 6.28E-06 | 3364 | unclassified | unclassified | unclassified | unclassified | unclassified | Clostridia | Firmicutes |
| Eubacteriales sp. (HG3A.0153) | AHI | -0.094 | 3.45E-07 | 6.29E-06 | 3004 | unclassified | unclassified | unclassified | unclassified | Eubacteriales | Clostridia | Firmicutes |
| Parvimonas micra (HG3A.1231) | ODI | 0.086 | 6.76E-07 | 6.29E-06 | 3364 | unclassified | Parvimonas micra | Parvimonas | Peptoniphilaceae | Tissierellales | Tissierellia | Firmicutes |
| Eubacteriales sp. (HG3A.0609) | ODI | -0.086 | 6.88E-07 | 6.37E-06 | 3364 | unclassified | unclassified | unclassified | unclassified | Eubacteriales | Clostridia | Firmicutes |
| Eubacteriales sp. (HG3A.0568) | ODI | -0.086 | 7.43E-07 | 6.84E-06 | 3364 | unclassified | unclassified | unclassified | unclassified | Eubacteriales | Clostridia | Firmicutes |
| Clostridia sp. (HG3A.0893) | T90 | -0.087 | 5.17E-07 | 6.90E-06 | 3364 | unclassified | unclassified | unclassified | unclassified | unclassified | Clostridia | Firmicutes |
| Oscillospiraceae sp. (HG3A.0461) | T90 | -0.087 | 5.22E-07 | 6.91E-06 | 3364 | unclassified | unclassified | unclassified | Oscillospiraceae | Eubacteriales | Clostridia | Firmicutes |
| Eubacteriales sp. (HG3A.0757) | ODI | -0.086 | 7.59E-07 | 6.95E-06 | 3364 | unclassified | unclassified | unclassified | unclassified | Eubacteriales | Clostridia | Firmicutes |
| Eubacteriales sp. (HG3A.0653) | T90 | -0.087 | 5.32E-07 | 6.99E-06 | 3364 | unclassified | unclassified | unclassified | unclassified | Eubacteriales | Clostridia | Firmicutes |

| Eubacteriales sp. (HG3A.0572) | AHI | -0.093 | 4.29E-07 | 7.72E-06 | 3004 | unclassified | unclassified | unclassified | unclassified | Eubacteriales | Clostridia | Firmicutes |
| --- | --- | --- | --- | --- | --- | --- | --- | --- | --- | --- | --- | --- |
| Eubacteriales sp. (HG3A.0363) | AHI | -0.093 | 4.36E-07 | 7.76E-06 | 3004 | unclassified | unclassified | unclassified | unclassified | Eubacteriales | Clostridia | Firmicutes |
| Clostridia sp. (HG3A.0752) | ODI | -0.085 | 8.69E-07 | 7.91E-06 | 3364 | unclassified | unclassified | unclassified | unclassified | unclassified | Clostridia | Firmicutes |
| Eubacteriales sp. (HG3A.0193) | T90 | -0.086 | 6.39E-07 | 8.32E-06 | 3364 | unclassified | unclassified | unclassified | unclassified | Eubacteriales | Clostridia | Firmicutes |
| Clostridia sp. (HG3A.0272) | AHI | -0.093 | 4.91E-07 | 8.54E-06 | 3004 | unclassified | unclassified | unclassified | unclassified | unclassified | Clostridia | Firmicutes |
| Clostridia sp. (HG3A.0385) | AHI | -0.093 | 4.89E-07 | 8.54E-06 | 3004 | unclassified | unclassified | unclassified | unclassified | unclassified | Clostridia | Firmicutes |
| Eubacteriales sp. (HG3A.0468) | T90 | -0.086 | 6.74E-07 | 8.71E-06 | 3364 | unclassified | unclassified | unclassified | unclassified | Eubacteriales | Clostridia | Firmicutes |
| Clostridia sp. (HG3A.0783) | T90 | -0.086 | 6.91E-07 | 8.85E-06 | 3364 | unclassified | unclassified | unclassified | unclassified | unclassified | Clostridia | Firmicutes |
| Eubacteriales sp. (HG3A.0568) | T90 | -0.086 | 6.99E-07 | 8.88E-06 | 3364 | unclassified | unclassified | unclassified | unclassified | Eubacteriales | Clostridia | Firmicutes |
| Eggerthellales sp. (HG3A.0177) | AHI | -0.092 | 5.22E-07 | 8.99E-06 | 3004 | unclassified | unclassified | unclassified | unclassified | Eggerthellales | Coriobacteriia | Actinobacteria |
| Lachnospiraceae sp. (HG3A.0018) | AHI | 0.092 | 5.64E-07 | 9.61E-06 | 3004 | unclassified | unclassified | unclassified | Lachnospiraceae | Eubacteriales | Clostridia | Firmicutes |
| Clostridia sp. (HG3A.0521) | ODI | -0.085 | 1.09E-06 | 9.84E-06 | 3364 | unclassified | unclassified | unclassified | unclassified | unclassified | Clostridia | Firmicutes |
| Sellimonas intestinalis (HG3A.0417) | ODI | 0.085 | 1.10E-06 | 9.87E-06 | 3364 | unclassified | Sellimonas intestinalis | Sellimonas | Lachnospiraceae | Eubacteriales | Clostridia | Firmicutes |
| Amedibacillus dolichus  (HG3A.0798) | AHI | 0.092 | 5.95E-07 | 1.00E-05 | 3004 | unclassified | Amedibacillus  dolichus | Amedibacillus | Erysipelotrichace  ae | Erysipelotrichales | Erysipelotrichia | Firmicutes |
| Eubacteriales sp. (HG3A.0153) | T90 | -0.086 | 7.94E-07 | 1.00E-05 | 3364 | unclassified | unclassified | unclassified | unclassified | Eubacteriales | Clostridia | Firmicutes |
| Streptococcus oralis subsp. oralis (HG3A.0705) | ODI | 0.084 | 1.17E-06 | 1.05E-05 | 3364 | Streptococcus oralis subsp.  oralis | Streptococcus oralis | Streptococcus | Streptococcaceae | Lactobacillales | Bacilli | Firmicutes |
| Eubacteriales sp. (HG3A.0154) | AHI | -0.092 | 6.54E-07 | 1.09E-05 | 3004 | unclassified | unclassified | unclassified | unclassified | Eubacteriales | Clostridia | Firmicutes |
| Clostridia sp. (HG3A.0728) | AHI | -0.091 | 6.71E-07 | 1.11E-05 | 3004 | unclassified | unclassified | unclassified | unclassified | unclassified | Clostridia | Firmicutes |
| Eubacteriales sp. (HG3A.0102) | ODI | -0.084 | 1.26E-06 | 1.13E-05 | 3364 | unclassified | unclassified | unclassified | unclassified | Eubacteriales | Clostridia | Firmicutes |
| Eubacteriales sp. (HG3A.0085) | T90 | -0.085 | 9.22E-07 | 1.15E-05 | 3364 | unclassified | unclassified | unclassified | unclassified | Eubacteriales | Clostridia | Firmicutes |
| Eubacteriales sp. (HG3A.0626) | ODI | -0.084 | 1.32E-06 | 1.16E-05 | 3364 | unclassified | unclassified | unclassified | unclassified | Eubacteriales | Clostridia | Firmicutes |

| Firmicutes sp. (HG3A.0541) | ODI | -0.084 | 1.33E-06 | 1.17E-05 | 3364 | unclassified | unclassified | unclassified | unclassified | unclassified | unclassified | Firmicutes |
| --- | --- | --- | --- | --- | --- | --- | --- | --- | --- | --- | --- | --- |
| Eubacteriales sp. (HG3A.0419) | AHI | -0.091 | 7.23E-07 | 1.18E-05 | 3004 | unclassified | unclassified | unclassified | unclassified | Eubacteriales | Clostridia | Firmicutes |
| Parvimonas micra (HG3A.1231) | T90 | 0.085 | 9.50E-07 | 1.18E-05 | 3364 | unclassified | Parvimonas micra | Parvimonas | Peptoniphilaceae | Tissierellales | Tissierellia | Firmicutes |
| Clostridia sp. (HG3A.1020) | T90 | -0.085 | 9.66E-07 | 1.19E-05 | 3364 | unclassified | unclassified | unclassified | unclassified | unclassified | Clostridia | Firmicutes |
| Eubacteriales sp. (HG3A.0477) | T90 | -0.085 | 9.89E-07 | 1.21E-05 | 3364 | unclassified | unclassified | unclassified | unclassified | Eubacteriales | Clostridia | Firmicutes |
| Roseburia intestinalis (HG3A.0078) | T90 | 0.085 | 9.95E-07 | 1.21E-05 | 3364 | unclassified | Roseburia intestinalis | Roseburia | Lachnospiraceae | Eubacteriales | Clostridia | Firmicutes |
| Fusicatenibacter saccharivorans (HG3A.0004) | T90 | 0.085 | 1.01E-06 | 1.22E-05 | 3364 | unclassified | Fusicatenibacter saccharivorans | Fusicatenibacter | Lachnospiraceae | Eubacteriales | Clostridia | Firmicutes |
| Collinsella sp. WCA1-178-WT-3 (M2) (HG3A.1245) | T90 | 0.085 | 1.03E-06 | 1.23E-05 | 3364 | unclassified | Collinsella sp. WCA1 178-WT-3 (M2) | Collinsella | Coriobacteriaceae | Coriobacteriales | Coriobacteriia | Actinobacteria |
| Eubacteriales sp. (HG3A.0829) | ODI | -0.084 | 1.40E-06 | 1.23E-05 | 3364 | unclassified | unclassified | unclassified | unclassified | Eubacteriales | Clostridia | Firmicutes |
| Eubacteriales sp. (HG3A.0342) | T90 | -0.085 | 1.10E-06 | 1.31E-05 | 3364 | unclassified | unclassified | unclassified | unclassified | Eubacteriales | Clostridia | Firmicutes |
| Clostridia sp. (HG3A.1217) | ODI | -0.083 | 1.55E-06 | 1.35E-05 | 3364 | unclassified | unclassified | unclassified | unclassified | unclassified | Clostridia | Firmicutes |
| Enterocloster citroniae (HG3A.0285) | T90 | 0.084 | 1.16E-06 | 1.35E-05 | 3364 | unclassified | Enterocloster citroniae | Enterocloster | Lachnospiraceae | Eubacteriales | Clostridia | Firmicutes |
| Eubacteriales sp. (HG3A.0113) | T90 | -0.084 | 1.16E-06 | 1.35E-05 | 3364 | unclassified | unclassified | unclassified | unclassified | Eubacteriales | Clostridia | Firmicutes |
| Rothia mucilaginosa (HG3A.0559) | ODI | 0.083 | 1.57E-06 | 1.36E-05 | 3364 | unclassified | Rothia mucilaginosa | Rothia | Micrococcaceae | Micrococcales | Actinomycetia | Actinobacteria |
| Streptococcus anginosus  (HG3A.0680) | AHI | 0.091 | 8.41E-07 | 1.36E-05 | 3004 | unclassified | Streptococcus  anginosus | Streptococcus | Streptococcaceae | Lactobacillales | Bacilli | Firmicutes |
| Eubacterium sp. AF16-48 (HG3A.0219) | AHI | -0.091 | 8.61E-07 | 1.38E-05 | 3004 | unclassified | Eubacterium sp.  AF16-48 | Eubacterium | Eubacteriaceae | Eubacteriales | Clostridia | Firmicutes |
| Eubacteriales sp. (HG3A.0132) | ODI | -0.083 | 1.64E-06 | 1.42E-05 | 3364 | unclassified | unclassified | unclassified | unclassified | Eubacteriales | Clostridia | Firmicutes |
| Eubacteriales sp. (HG3A.0516) | T90 | -0.084 | 1.24E-06 | 1.43E-05 | 3364 | unclassified | unclassified | unclassified | unclassified | Eubacteriales | Clostridia | Firmicutes |
| Oscillospiraceae sp. (HG3A.0739) | T90 | -0.084 | 1.26E-06 | 1.45E-05 | 3364 | unclassified | unclassified | unclassified | Oscillospiraceae | Eubacteriales | Clostridia | Firmicutes |
| Eubacteriales sp. (HG3A.0151) | ODI | -0.083 | 1.70E-06 | 1.46E-05 | 3364 | unclassified | unclassified | unclassified | unclassified | Eubacteriales | Clostridia | Firmicutes |

Alistipes senegalensis (HG3A.0141)

AHI -0.09 9.37E-07 1.47E-05 3004 unclassified Alistipes

senegalensis

Alistipes Rikenellaceae Bacteroidales Bacteroidia Bacteroidetes

| Eubacteriales sp. (HG3A.0229) | T90 | -0.084 | 1.29E-06 | 1.47E-05 | 3364 | unclassified | unclassified | unclassified | unclassified | Eubacteriales | Clostridia | Firmicutes |
| --- | --- | --- | --- | --- | --- | --- | --- | --- | --- | --- | --- | --- |
| Lachnospiraceae sp. (HG3A.0236) | AHI | -0.09 | 9.27E-07 | 1.47E-05 | 3004 | unclassified | unclassified | unclassified | Lachnospiraceae | Eubacteriales | Clostridia | Firmicutes |
| Blautia sp. (HG3A.0416) | AHI | 0.09 | 9.73E-07 | 1.50E-05 | 3004 | unclassified | unclassified | Blautia | Lachnospiraceae | Eubacteriales | Clostridia | Firmicutes |
| Eubacteriales sp. (HG3A.0309) | AHI | -0.09 | 9.62E-07 | 1.50E-05 | 3004 | unclassified | unclassified | unclassified | unclassified | Eubacteriales | Clostridia | Firmicutes |
| Firmicutes sp. (HG3A.0596) | AHI | -0.09 | 9.93E-07 | 1.51E-05 | 3004 | unclassified | unclassified | unclassified | unclassified | unclassified | unclassified | Firmicutes |
| Eubacteriales sp. (HG3A.0829) | AHI | -0.09 | 1.00E-06 | 1.52E-05 | 3004 | unclassified | unclassified | unclassified | unclassified | Eubacteriales | Clostridia | Firmicutes |
| Eubacteriales sp. (HG3A.0154) | T90 | -0.084 | 1.35E-06 | 1.54E-05 | 3364 | unclassified | unclassified | unclassified | unclassified | Eubacteriales | Clostridia | Firmicutes |
| Clostridia sp. (HG3A.0599) | AHI | -0.09 | 1.04E-06 | 1.55E-05 | 3004 | unclassified | unclassified | unclassified | unclassified | unclassified | Clostridia | Firmicutes |
| Clostridia sp. (HG3A.0682) | T90 | -0.084 | 1.37E-06 | 1.55E-05 | 3364 | unclassified | unclassified | unclassified | unclassified | unclassified | Clostridia | Firmicutes |
| Eubacteriales sp. (HG3A.0161) | AHI | -0.09 | 1.05E-06 | 1.56E-05 | 3004 | unclassified | unclassified | unclassified | unclassified | Eubacteriales | Clostridia | Firmicutes |
| Eubacteriales sp. (HG3A.0627) | ODI | -0.083 | 1.83E-06 | 1.56E-05 | 3364 | unclassified | unclassified | unclassified | unclassified | Eubacteriales | Clostridia | Firmicutes |
| Odoribacter splanchnicus  (HG3A.0041) | T90 | -0.084 | 1.40E-06 | 1.57E-05 | 3364 | unclassified | Odoribacter  splanchnicus | Odoribacter | Odoribacteraceae | Bacteroidales | Bacteroidia | Bacteroidetes |
| Eubacteriales sp. (HG3A.0409) | T90 | -0.084 | 1.43E-06 | 1.58E-05 | 3364 | unclassified | unclassified | unclassified | unclassified | Eubacteriales | Clostridia | Firmicutes |
| Eubacteriales sp. (HG3A.0613) | T90 | -0.084 | 1.43E-06 | 1.58E-05 | 3364 | unclassified | unclassified | unclassified | unclassified | Eubacteriales | Clostridia | Firmicutes |
| Enterocloster aldenensis (HG3A.0362) | T90 | 0.084 | 1.44E-06 | 1.59E-05 | 3364 | unclassified | Enterocloster aldenensis | Enterocloster | Lachnospiraceae | Eubacteriales | Clostridia | Firmicutes |
| Eubacteriales sp. (HG3A.0731) | ODI | -0.083 | 1.89E-06 | 1.60E-05 | 3364 | unclassified | unclassified | unclassified | unclassified | Eubacteriales | Clostridia | Firmicutes |
| Eubacteriales sp. (HG3A.0151) | AHI | -0.09 | 1.13E-06 | 1.66E-05 | 3004 | unclassified | unclassified | unclassified | unclassified | Eubacteriales | Clostridia | Firmicutes |
| Dorea sp. AF36-15AT  (HG3A.0052) | AHI | 0.089 | 1.17E-06 | 1.70E-05 | 3004 | unclassified | Dorea sp. AF36-  15AT | Dorea | Lachnospiraceae | Eubacteriales | Clostridia | Firmicutes |
| Eubacteriales sp. (HG3A.0790) | ODI | -0.082 | 2.14E-06 | 1.81E-05 | 3364 | unclassified | unclassified | unclassified | unclassified | Eubacteriales | Clostridia | Firmicutes |
| Eubacteriales sp. (HG3A.0711) | T90 | -0.083 | 1.68E-06 | 1.83E-05 | 3364 | unclassified | unclassified | unclassified | unclassified | Eubacteriales | Clostridia | Firmicutes |
| Oscillospiraceae sp. (HG3A.1270) | AHI | -0.089 | 1.28E-06 | 1.85E-05 | 3004 | unclassified | unclassified | unclassified | Oscillospiraceae | Eubacteriales | Clostridia | Firmicutes |
| Eubacteriales sp. (HG3A.0621) | T90 | -0.083 | 1.82E-06 | 1.97E-05 | 3364 | unclassified | unclassified | unclassified | unclassified | Eubacteriales | Clostridia | Firmicutes |

| Lachnospiraceae sp. (HG3A.0252) | T90 | -0.083 | 1.89E-06 | 2.03E-05 | 3364 | unclassified | unclassified | unclassified | Lachnospiraceae | Eubacteriales | Clostridia | Firmicutes |
| --- | --- | --- | --- | --- | --- | --- | --- | --- | --- | --- | --- | --- |
| Eubacterium ramulus  (HG3A.0068) | T90 | 0.083 | 1.94E-06 | 2.05E-05 | 3364 | unclassified | Eubacterium ramulus | Eubacterium | Eubacteriaceae | Eubacteriales | Clostridia | Firmicutes |
| Lachnospiraceae sp. (HG3A.0127) | T90 | -0.083 | 1.93E-06 | 2.05E-05 | 3364 | unclassified | unclassified | unclassified | Lachnospiraceae | Eubacteriales | Clostridia | Firmicutes |
| Firmicutes sp. (HG3A.0501) | ODI | -0.082 | 2.48E-06 | 2.08E-05 | 3364 | unclassified | unclassified | unclassified | unclassified | unclassified | unclassified | Firmicutes |
| Butyricimonas virosa (HG3A.0199) | ODI | -0.082 | 2.51E-06 | 2.09E-05 | 3364 | unclassified | Butyricimonas virosa | Butyricimonas | Odoribacteraceae | Bacteroidales | Bacteroidia | Bacteroidetes |
| Eubacteriales sp. (HG3A.0184) | ODI | -0.082 | 2.50E-06 | 2.09E-05 | 3364 | unclassified | unclassified | unclassified | unclassified | Eubacteriales | Clostridia | Firmicutes |
| Firmicutes sp. (HG3A.0650) | T90 | -0.083 | 2.01E-06 | 2.11E-05 | 3364 | unclassified | unclassified | unclassified | unclassified | unclassified | unclassified | Firmicutes |
| Clostridiaceae sp. (HG3A.0238) | T90 | -0.082 | 2.08E-06 | 2.17E-05 | 3364 | unclassified | unclassified | unclassified | Clostridiaceae | Eubacteriales | Clostridia | Firmicutes |
| Methanobrevibacter smithii (HG3A.0152) | ODI | -0.082 | 2.64E-06 | 2.18E-05 | 3364 | unclassified | Methanobrevibacter smithii | Methanobrevibacter | Methanobacteriac eae | Methanobacteriales | Methanobacteria | Euryarchaeota |
| Eubacteriales sp. (HG3A.0477) | AHI | -0.088 | 1.53E-06 | 2.19E-05 | 3004 | unclassified | unclassified | unclassified | unclassified | Eubacteriales | Clostridia | Firmicutes |
| Anaerostipes caccae (HG3A.0747) | T90 | 0.082 | 2.15E-06 | 2.23E-05 | 3364 | unclassified | Anaerostipes caccae | Anaerostipes | Lachnospiraceae | Eubacteriales | Clostridia | Firmicutes |
| Oscillospiraceae sp. (HG3A.0665) | T90 | -0.082 | 2.19E-06 | 2.26E-05 | 3364 | unclassified | unclassified | unclassified | Oscillospiraceae | Eubacteriales | Clostridia | Firmicutes |
| Eubacteriales sp. (HG3A.0291) | T90 | -0.082 | 2.46E-06 | 2.53E-05 | 3364 | unclassified | unclassified | unclassified | unclassified | Eubacteriales | Clostridia | Firmicutes |
| Eubacteriales sp. (HG3A.0426) | AHI | -0.088 | 1.86E-06 | 2.63E-05 | 3004 | unclassified | unclassified | unclassified | unclassified | Eubacteriales | Clostridia | Firmicutes |
| Eubacteriales sp. (HG3A.0215) | T90 | -0.082 | 2.60E-06 | 2.65E-05 | 3364 | unclassified | unclassified | unclassified | unclassified | Eubacteriales | Clostridia | Firmicutes |
| Traorella massiliensis  (HG3A.0669) | T90 | -0.082 | 2.65E-06 | 2.69E-05 | 3364 | unclassified | Traorella  massiliensis | Traorella | Erysipelotrichace  ae | Erysipelotrichales | Erysipelotrichia | Firmicutes |
| Eubacteriales sp. (HG3A.0229) | AHI | -0.088 | 1.93E-06 | 2.72E-05 | 3004 | unclassified | unclassified | unclassified | unclassified | Eubacteriales | Clostridia | Firmicutes |
| Eubacteriales sp. (HG3A.0250) | T90 | -0.081 | 2.75E-06 | 2.77E-05 | 3364 | unclassified | unclassified | unclassified | unclassified | Eubacteriales | Clostridia | Firmicutes |
| Clostridia sp. (HG3A.1217) | AHI | -0.087 | 2.00E-06 | 2.78E-05 | 3004 | unclassified | unclassified | unclassified | unclassified | unclassified | Clostridia | Firmicutes |
| Eubacteriales sp. (HG3A.0123) | ODI | 0.081 | 3.40E-06 | 2.78E-05 | 3364 | unclassified | unclassified | unclassified | unclassified | Eubacteriales | Clostridia | Firmicutes |
| Eubacterium ramulus (HG3A.0068) | ODI | 0.081 | 3.39E-06 | 2.78E-05 | 3364 | unclassified | Eubacterium ramulus | Eubacterium | Eubacteriaceae | Eubacteriales | Clostridia | Firmicutes |
| Eubacteriales sp. (HG3A.0288) | ODI | -0.081 | 3.45E-06 | 2.80E-05 | 3364 | unclassified | unclassified | unclassified | unclassified | Eubacteriales | Clostridia | Firmicutes |

| Eubacteriales sp. (HG3A.0291) | ODI | -0.081 | 3.46E-06 | 2.80E-05 | 3364 | unclassified | unclassified | unclassified | unclassified | Eubacteriales | Clostridia | Firmicutes |
| --- | --- | --- | --- | --- | --- | --- | --- | --- | --- | --- | --- | --- |
| Eubacteriales sp. (HG3A.0405) | AHI | -0.087 | 2.14E-06 | 2.93E-05 | 3004 | unclassified | unclassified | unclassified | unclassified | Eubacteriales | Clostridia | Firmicutes |
| Eubacteriales sp. (HG3A.0771) | AHI | -0.087 | 2.14E-06 | 2.93E-05 | 3004 | unclassified | unclassified | unclassified | unclassified | Eubacteriales | Clostridia | Firmicutes |
| Clostridia sp. (HG3A.0756) | T90 | -0.081 | 3.04E-06 | 3.05E-05 | 3364 | unclassified | unclassified | unclassified | unclassified | unclassified | Clostridia | Firmicutes |
| Oscillospiraceae sp. (HG3A.0693) | T90 | -0.081 | 3.06E-06 | 3.05E-05 | 3364 | unclassified | unclassified | unclassified | Oscillospiraceae | Eubacteriales | Clostridia | Firmicutes |
| Candidatus Borkfalkiales sp.  (HG3A.1329) | T90 | -0.081 | 3.14E-06 | 3.11E-05 | 3364 | unclassified | unclassified | unclassified | unclassified | Candidatus  Borkfalkiales | Clostridia | Firmicutes |
| [Clostridium] innocuum (HG3A.0365) | T90 | 0.081 | 3.19E-06 | 3.12E-05 | 3364 | unclassified | [Clostridium] innocuum | Erysipelatoclostridiu m | Erysipelotrichace ae | Erysipelotrichales | Erysipelotrichia | Firmicutes |
| Clostridium sp. TM06-18  (HG3A.0048) | T90 | 0.081 | 3.18E-06 | 3.12E-05 | 3364 | unclassified | Clostridium sp.  TM06-18 | Clostridium | Clostridiaceae | Eubacteriales | Clostridia | Firmicutes |
| Oscillospiraceae sp. (HG3A.0445) | T90 | -0.081 | 3.23E-06 | 3.13E-05 | 3364 | unclassified | unclassified | unclassified | Oscillospiraceae | Eubacteriales | Clostridia | Firmicutes |
| Firmicutes sp. (HG3A.0587) | ODI | -0.08 | 3.93E-06 | 3.16E-05 | 3364 | unclassified | unclassified | unclassified | unclassified | unclassified | unclassified | Firmicutes |
| Anaerotruncus colihominis (HG3A.0307) | ODI | 0.08 | 4.15E-06 | 3.33E-05 | 3364 | unclassified | Anaerotruncus colihominis | Anaerotruncus | Oscillospiraceae | Eubacteriales | Clostridia | Firmicutes |
| Eubacteriales sp. (HG3A.0718) | AHI | -0.087 | 2.53E-06 | 3.44E-05 | 3004 | unclassified | unclassified | unclassified | unclassified | Eubacteriales | Clostridia | Firmicutes |
| Clostridia sp. (HG3A.0276) | ODI | -0.08 | 4.34E-06 | 3.46E-05 | 3364 | unclassified | unclassified | unclassified | unclassified | unclassified | Clostridia | Firmicutes |
| Clostridia sp. (HG3A.0728) | T90 | -0.08 | 3.59E-06 | 3.46E-05 | 3364 | unclassified | unclassified | unclassified | unclassified | unclassified | Clostridia | Firmicutes |
| Eubacteriales sp. (HG3A.0730) | T90 | -0.08 | 3.61E-06 | 3.46E-05 | 3364 | unclassified | unclassified | unclassified | unclassified | Eubacteriales | Clostridia | Firmicutes |
| Eubacteriales sp. (HG3A.0376) | T90 | -0.08 | 3.65E-06 | 3.48E-05 | 3364 | unclassified | unclassified | unclassified | unclassified | Eubacteriales | Clostridia | Firmicutes |
| Eubacteriales sp. (HG3A.0864) | T90 | -0.08 | 3.72E-06 | 3.53E-05 | 3364 | unclassified | unclassified | unclassified | unclassified | Eubacteriales | Clostridia | Firmicutes |
| Eubacteriales sp. (HG3A.0188) | AHI | -0.086 | 2.69E-06 | 3.62E-05 | 3004 | unclassified | unclassified | unclassified | unclassified | Eubacteriales | Clostridia | Firmicutes |
| Eubacteriales sp. (HG3A.0264) | AHI | -0.086 | 2.74E-06 | 3.65E-05 | 3004 | unclassified | unclassified | unclassified | unclassified | Eubacteriales | Clostridia | Firmicutes |
| Eubacteriales sp. (HG3A.0329) | T90 | -0.08 | 3.87E-06 | 3.65E-05 | 3364 | unclassified | unclassified | unclassified | unclassified | Eubacteriales | Clostridia | Firmicutes |
| Anaerotruncus colihominis (HG3A.0307) | T90 | 0.08 | 4.00E-06 | 3.75E-05 | 3364 | unclassified | Anaerotruncus colihominis | Anaerotruncus | Oscillospiraceae | Eubacteriales | Clostridia | Firmicutes |
| Clostridia sp. (HG3A.0094) | T90 | -0.08 | 4.08E-06 | 3.80E-05 | 3364 | unclassified | unclassified | unclassified | unclassified | unclassified | Clostridia | Firmicutes |

| Eubacteriales sp. (HG3A.0254) | ODI | 0.079 | 4.86E-06 | 3.84E-05 | 3364 | unclassified | unclassified | unclassified | unclassified | Eubacteriales | Clostridia | Firmicutes |
| --- | --- | --- | --- | --- | --- | --- | --- | --- | --- | --- | --- | --- |
| Eubacteriales sp. (HG3A.0531) | AHI | -0.086 | 2.90E-06 | 3.84E-05 | 3004 | unclassified | unclassified | unclassified | unclassified | Eubacteriales | Clostridia | Firmicutes |
| Eubacteriales sp. (HG3A.0864) | ODI | -0.079 | 4.87E-06 | 3.84E-05 | 3364 | unclassified | unclassified | unclassified | unclassified | Eubacteriales | Clostridia | Firmicutes |
| Eubacteriales sp. (HG3A.0859) | ODI | -0.079 | 4.93E-06 | 3.87E-05 | 3364 | unclassified | unclassified | unclassified | unclassified | Eubacteriales | Clostridia | Firmicutes |
| Enterocloster citroniae (HG3A.0285) | ODI | 0.079 | 4.97E-06 | 3.88E-05 | 3364 | unclassified | Enterocloster citroniae | Enterocloster | Lachnospiraceae | Eubacteriales | Clostridia | Firmicutes |
| Oscillospiraceae sp. (HG3A.0445) | ODI | -0.079 | 4.98E-06 | 3.88E-05 | 3364 | unclassified | unclassified | unclassified | Oscillospiraceae | Eubacteriales | Clostridia | Firmicutes |
| Ruminococcus sp. AM42-11 (HG3A.0002) | ODI | 0.079 | 5.03E-06 | 3.89E-05 | 3364 | unclassified | Ruminococcus sp.  AM42-11 | Ruminococcus | Oscillospiraceae | Eubacteriales | Clostridia | Firmicutes |
| Eubacteriales sp. (HG3A.0621) | ODI | -0.079 | 5.12E-06 | 3.94E-05 | 3364 | unclassified | unclassified | unclassified | unclassified | Eubacteriales | Clostridia | Firmicutes |
| Oscillospiraceae sp. (HG3A.0774) | ODI | -0.079 | 5.14E-06 | 3.94E-05 | 3364 | unclassified | unclassified | unclassified | Oscillospiraceae | Eubacteriales | Clostridia | Firmicutes |
| Ruminococcus sp. AF46-10NS  (HG3A.0271) | T90 | 0.08 | 4.26E-06 | 3.94E-05 | 3364 | unclassified | Ruminococcus sp.  AF46-10NS | Ruminococcus | Oscillospiraceae | Eubacteriales | Clostridia | Firmicutes |
| Barnesiella intestinihominis (HG3A.0055) | ODI | -0.079 | 5.18E-06 | 3.95E-05 | 3364 | unclassified | Barnesiella intestinihominis | Barnesiella | Barnesiellaceae | Bacteroidales | Bacteroidia | Bacteroidetes |
| Haemophilus parainfluenzae (HG3A.0181) | T90 | -0.08 | 4.44E-06 | 4.08E-05 | 3364 | unclassified | Haemophilus parainfluenzae | Haemophilus | Pasteurellaceae | Pasteurellales | Gammaproteob acteria | Proteobacteria |
| Eggerthellaceae sp. (HG3A.0171) | ODI | -0.079 | 5.52E-06 | 4.19E-05 | 3364 | unclassified | unclassified | unclassified | Eggerthellaceae | Eggerthellales | Coriobacteriia | Actinobacteria |
| Clostridia sp. (HG3A.1020) | AHI | -0.086 | 3.22E-06 | 4.22E-05 | 3004 | unclassified | unclassified | unclassified | unclassified | unclassified | Clostridia | Firmicutes |
| Victivallis lenta (HG3A.0525) | ODI | -0.079 | 5.62E-06 | 4.25E-05 | 3364 | unclassified | Victivallis lenta | Victivallis | Victivallaceae | Victivallales | Lentisphaeria | Lentisphaerae |
| Eubacteriales sp. (HG3A.0163) | ODI | -0.079 | 5.71E-06 | 4.29E-05 | 3364 | unclassified | unclassified | unclassified | unclassified | Eubacteriales | Clostridia | Firmicutes |
| Eubacteriales sp. (HG3A.0215) | AHI | -0.086 | 3.29E-06 | 4.29E-05 | 3004 | unclassified | unclassified | unclassified | unclassified | Eubacteriales | Clostridia | Firmicutes |
| Eubacteriales sp. (HG3A.0254) | AHI | 0.086 | 3.36E-06 | 4.34E-05 | 3004 | unclassified | unclassified | unclassified | unclassified | Eubacteriales | Clostridia | Firmicutes |
| Eubacteriales sp. (HG3A.0516) | ODI | -0.079 | 5.89E-06 | 4.41E-05 | 3364 | unclassified | unclassified | unclassified | unclassified | Eubacteriales | Clostridia | Firmicutes |
| Eubacteriales sp. (HG3A.0137) | ODI | -0.079 | 6.18E-06 | 4.58E-05 | 3364 | unclassified | unclassified | unclassified | unclassified | Eubacteriales | Clostridia | Firmicutes |
| Firmicutes sp. (HG3A.1085) | ODI | -0.079 | 6.18E-06 | 4.58E-05 | 3364 | unclassified | unclassified | unclassified | unclassified | unclassified | unclassified | Firmicutes |

| Clostridia sp. (HG3A.0750) | ODI | -0.079 | 6.24E-06 | 4.60E-05 | 3364 | unclassified | unclassified | unclassified | unclassified | unclassified | Clostridia | Firmicutes |
| --- | --- | --- | --- | --- | --- | --- | --- | --- | --- | --- | --- | --- |
| Firmicutes sp. (HG3A.0436) | ODI | -0.078 | 6.34E-06 | 4.66E-05 | 3364 | unclassified | unclassified | unclassified | unclassified | unclassified | unclassified | Firmicutes |
| Roseburia intestinalis  (HG3A.0078) | AHI | 0.085 | 3.64E-06 | 4.67E-05 | 3004 | unclassified | Roseburia intestinalis | Roseburia | Lachnospiraceae | Eubacteriales | Clostridia | Firmicutes |
| Eubacteriales sp. (HG3A.0376) | ODI | -0.078 | 6.50E-06 | 4.73E-05 | 3364 | unclassified | unclassified | unclassified | unclassified | Eubacteriales | Clostridia | Firmicutes |
| Eubacteriales sp. (HG3A.0439) | ODI | -0.078 | 6.48E-06 | 4.73E-05 | 3364 | unclassified | unclassified | unclassified | unclassified | Eubacteriales | Clostridia | Firmicutes |
| Eubacteriales sp. (HG3A.0730) | ODI | -0.078 | 6.54E-06 | 4.74E-05 | 3364 | unclassified | unclassified | unclassified | unclassified | Eubacteriales | Clostridia | Firmicutes |
| Clostridia sp. (HG3A.0879) | T90 | -0.079 | 5.23E-06 | 4.79E-05 | 3364 | unclassified | unclassified | unclassified | unclassified | unclassified | Clostridia | Firmicutes |
| Oscillospiraceae sp. (HG3A.1173) | ODI | -0.078 | 6.64E-06 | 4.79E-05 | 3364 | unclassified | unclassified | unclassified | Oscillospiraceae | Eubacteriales | Clostridia | Firmicutes |
| Coprococcus sp. (HG3A.0404) | ODI | 0.078 | 6.71E-06 | 4.82E-05 | 3364 | unclassified | unclassified | Coprococcus | Lachnospiraceae | Eubacteriales | Clostridia | Firmicutes |
| Eubacteriales sp. (HG3A.0254) | T90 | 0.079 | 5.33E-06 | 4.85E-05 | 3364 | unclassified | unclassified | unclassified | unclassified | Eubacteriales | Clostridia | Firmicutes |
| Eubacteriales sp. (HG3A.0113) | AHI | -0.085 | 3.91E-06 | 4.97E-05 | 3004 | unclassified | unclassified | unclassified | unclassified | Eubacteriales | Clostridia | Firmicutes |
| Eubacteriales sp. (HG3A.0577) | ODI | -0.078 | 6.98E-06 | 4.99E-05 | 3364 | unclassified | unclassified | unclassified | unclassified | Eubacteriales | Clostridia | Firmicutes |
| Oscillospiraceae sp. (HG3A.0944) | T90 | -0.079 | 5.71E-06 | 5.17E-05 | 3364 | unclassified | unclassified | unclassified | Oscillospiraceae | Eubacteriales | Clostridia | Firmicutes |
| Eubacteriales sp. (HG3A.0572) | T90 | -0.079 | 5.91E-06 | 5.32E-05 | 3364 | unclassified | unclassified | unclassified | unclassified | Eubacteriales | Clostridia | Firmicutes |
| Acidaminococcus intestini  (HG3A.0407) | ODI | 0.078 | 7.56E-06 | 5.36E-05 | 3364 | unclassified | Acidaminococcus  intestini | Acidaminococcus | Acidaminococcac  eae | Acidaminococcales | Negativicutes | Firmicutes |
| Eubacteriales sp. (HG3A.0908) | ODI | -0.078 | 7.56E-06 | 5.36E-05 | 3364 | unclassified | unclassified | unclassified | unclassified | Eubacteriales | Clostridia | Firmicutes |
| Oscillibacter sp. (HG3A.0734) | T90 | -0.079 | 5.99E-06 | 5.36E-05 | 3364 | unclassified | unclassified | Oscillibacter | Oscillospiraceae | Eubacteriales | Clostridia | Firmicutes |
| Clostridia sp. (HG3A.1057) | ODI | -0.078 | 7.67E-06 | 5.42E-05 | 3364 | unclassified | unclassified | unclassified | unclassified | unclassified | Clostridia | Firmicutes |
| Streptococcus anginosus  (HG3A.0680) | T90 | 0.079 | 6.17E-06 | 5.49E-05 | 3364 | unclassified | Streptococcus  anginosus | Streptococcus | Streptococcaceae | Lactobacillales | Bacilli | Firmicutes |
| Clostridia sp. (HG3A.1008) | T90 | -0.078 | 6.36E-06 | 5.63E-05 | 3364 | unclassified | unclassified | unclassified | unclassified | unclassified | Clostridia | Firmicutes |
| Streptococcus salivarius  (HG3A.0071) | ODI | 0.078 | 8.02E-06 | 5.63E-05 | 3364 | unclassified | Streptococcus  salivarius | Streptococcus | Streptococcaceae | Lactobacillales | Bacilli | Firmicutes |
| Clostridia sp. (HG3A.0931) | T90 | -0.078 | 6.41E-06 | 5.64E-05 | 3364 | unclassified | unclassified | unclassified | unclassified | unclassified | Clostridia | Firmicutes |

| Eubacteriales sp. (HG3A.0184) | T90 | -0.078 | 6.48E-06 | 5.67E-05 | 3364 | unclassified | unclassified | unclassified | unclassified | Eubacteriales | Clostridia | Firmicutes |
| --- | --- | --- | --- | --- | --- | --- | --- | --- | --- | --- | --- | --- |
| Clostridia sp. (HG3A.0741) | T90 | -0.078 | 6.61E-06 | 5.75E-05 | 3364 | unclassified | unclassified | unclassified | unclassified | unclassified | Clostridia | Firmicutes |
| Veillonella rogosae (HG3A.0324) | AHI | -0.084 | 4.62E-06 | 5.83E-05 | 3004 | unclassified | Veillonella rogosae | Veillonella | Veillonellaceae | Veillonellales | Negativicutes | Firmicutes |
| Clostridia sp. (HG3A.0512) | ODI | -0.077 | 8.35E-06 | 5.84E-05 | 3364 | unclassified | unclassified | unclassified | unclassified | unclassified | Clostridia | Firmicutes |
| Gemella morbillorum  (HG3A.1782) | ODI | 0.077 | 8.46E-06 | 5.89E-05 | 3364 | unclassified | Gemella morbillorum | Gemella | unclassified | Bacillales | Bacilli | Firmicutes |
| Clostridia sp. (HG3A.0929) | T90 | -0.078 | 6.90E-06 | 5.97E-05 | 3364 | unclassified | unclassified | unclassified | unclassified | unclassified | Clostridia | Firmicutes |
| Eubacteriales sp. (HG3A.0978) | ODI | -0.077 | 8.67E-06 | 6.01E-05 | 3364 | unclassified | unclassified | unclassified | unclassified | Eubacteriales | Clostridia | Firmicutes |
| Ruminococcus sp. AF46-10NS (HG3A.0271) | ODI | 0.077 | 8.70E-06 | 6.01E-05 | 3364 | unclassified | Ruminococcus sp.  AF46-10NS | Ruminococcus | Oscillospiraceae | Eubacteriales | Clostridia | Firmicutes |
| Eubacteriales sp. (HG3A.0703) | ODI | -0.077 | 8.89E-06 | 6.11E-05 | 3364 | unclassified | unclassified | unclassified | unclassified | Eubacteriales | Clostridia | Firmicutes |
| Clostridia sp. (HG3A.1053) | T90 | -0.078 | 7.30E-06 | 6.29E-05 | 3364 | unclassified | unclassified | unclassified | unclassified | unclassified | Clostridia | Firmicutes |
| Eubacteriales sp. (HG3A.0856) | AHI | -0.084 | 5.02E-06 | 6.29E-05 | 3004 | unclassified | unclassified | unclassified | unclassified | Eubacteriales | Clostridia | Firmicutes |
| Eubacteriales sp. (HG3A.0472) | ODI | -0.077 | 9.32E-06 | 6.38E-05 | 3364 | unclassified | unclassified | unclassified | unclassified | Eubacteriales | Clostridia | Firmicutes |
| Eubacteriales sp. (HG3A.0489) | AHI | -0.084 | 5.25E-06 | 6.52E-05 | 3004 | unclassified | unclassified | unclassified | unclassified | Eubacteriales | Clostridia | Firmicutes |
| Clostridiaceae sp. (HG3A.0238) | ODI | -0.077 | 9.64E-06 | 6.57E-05 | 3364 | unclassified | unclassified | unclassified | Clostridiaceae | Eubacteriales | Clostridia | Firmicutes |
| Eubacteriales sp. (HG3A.0329) | AHI | -0.084 | 5.36E-06 | 6.61E-05 | 3004 | unclassified | unclassified | unclassified | unclassified | Eubacteriales | Clostridia | Firmicutes |
| Eubacteriales sp. (HG3A.0730) | AHI | -0.084 | 5.44E-06 | 6.62E-05 | 3004 | unclassified | unclassified | unclassified | unclassified | Eubacteriales | Clostridia | Firmicutes |
| Eubacteriales sp. (HG3A.0864) | AHI | -0.084 | 5.46E-06 | 6.62E-05 | 3004 | unclassified | unclassified | unclassified | unclassified | Eubacteriales | Clostridia | Firmicutes |
| Eubacteriales sp. (HG3A.0418) | AHI | -0.084 | 5.59E-06 | 6.72E-05 | 3004 | unclassified | unclassified | unclassified | unclassified | Eubacteriales | Clostridia | Firmicutes |
| Eubacteriales sp. (HG3A.0474) | AHI | -0.084 | 5.62E-06 | 6.72E-05 | 3004 | unclassified | unclassified | unclassified | unclassified | Eubacteriales | Clostridia | Firmicutes |
| Oscillospiraceae sp. (HG3A.0384) | AHI | -0.083 | 5.76E-06 | 6.84E-05 | 3004 | unclassified | unclassified | unclassified | Oscillospiraceae | Eubacteriales | Clostridia | Firmicutes |
| Oscillospiraceae sp. (HG3A.0388) | T90 | -0.077 | 8.24E-06 | 7.06E-05 | 3364 | unclassified | unclassified | unclassified | Oscillospiraceae | Eubacteriales | Clostridia | Firmicutes |
| Eubacteriales sp. (HG3A.1086) | AHI | -0.083 | 6.08E-06 | 7.17E-05 | 3004 | unclassified | unclassified | unclassified | unclassified | Eubacteriales | Clostridia | Firmicutes |

| Eubacteriales sp. (HG3A.0322) | ODI | -0.077 | 1.07E-05 | 7.25E-05 | 3364 | unclassified | unclassified | unclassified | unclassified | Eubacteriales | Clostridia | Firmicutes |
| --- | --- | --- | --- | --- | --- | --- | --- | --- | --- | --- | --- | --- |
| Eubacteriales sp. (HG3A.0505) | ODI | -0.076 | 1.11E-05 | 7.51E-05 | 3364 | unclassified | unclassified | unclassified | unclassified | Eubacteriales | Clostridia | Firmicutes |
| Lachnospiraceae sp. (HG3A.0399) | T90 | -0.077 | 8.81E-06 | 7.51E-05 | 3364 | unclassified | unclassified | unclassified | Lachnospiraceae | Eubacteriales | Clostridia | Firmicutes |
| Clostridia sp. (HG3A.1375) | ODI | -0.076 | 1.12E-05 | 7.55E-05 | 3364 | unclassified | unclassified | unclassified | unclassified | unclassified | Clostridia | Firmicutes |
| Firmicutes sp. (HG3A.0587) | AHI | -0.083 | 6.51E-06 | 7.61E-05 | 3004 | unclassified | unclassified | unclassified | unclassified | unclassified | unclassified | Firmicutes |
| Coprobacillus cateniformis (HG3A.0456) | T90 | 0.077 | 9.46E-06 | 8.01E-05 | 3364 | unclassified | Coprobacillus cateniformis | Coprobacillus | Coprobacillaceae | Erysipelotrichales | Erysipelotrichia | Firmicutes |
| Oscillibacter sp. (HG3A.0245) | T90 | -0.077 | 9.50E-06 | 8.01E-05 | 3364 | unclassified | unclassified | Oscillibacter | Oscillospiraceae | Eubacteriales | Clostridia | Firmicutes |
| Clostridia sp. (HG3A.1010) | ODI | -0.076 | 1.20E-05 | 8.05E-05 | 3364 | unclassified | unclassified | unclassified | unclassified | unclassified | Clostridia | Firmicutes |
| Eubacteriales sp. (HG3A.0132) | AHI | -0.083 | 7.03E-06 | 8.16E-05 | 3004 | unclassified | unclassified | unclassified | unclassified | Eubacteriales | Clostridia | Firmicutes |
| Eubacteriales sp. (HG3A.0637) | AHI | -0.083 | 7.11E-06 | 8.20E-05 | 3004 | unclassified | unclassified | unclassified | unclassified | Eubacteriales | Clostridia | Firmicutes |
| Clostridia sp. (HG3A.0815) | AHI | -0.083 | 7.22E-06 | 8.26E-05 | 3004 | unclassified | unclassified | unclassified | unclassified | unclassified | Clostridia | Firmicutes |
| Eubacteriales sp. (HG3A.0226) | T90 | -0.077 | 1.00E-05 | 8.43E-05 | 3364 | unclassified | unclassified | unclassified | unclassified | Eubacteriales | Clostridia | Firmicutes |
| Eubacteriales sp. (HG3A.0144) | AHI | -0.082 | 7.53E-06 | 8.55E-05 | 3004 | unclassified | unclassified | unclassified | unclassified | Eubacteriales | Clostridia | Firmicutes |
| Blautia hydrogenotrophica (HG3A.0430) | ODI | 0.076 | 1.30E-05 | 8.67E-05 | 3364 | unclassified | Blautia hydrogenotrophica | Blautia | Lachnospiraceae | Eubacteriales | Clostridia | Firmicutes |
| Coprococcus eutactus  (HG3A.0155) | T90 | -0.077 | 1.05E-05 | 8.79E-05 | 3364 | unclassified | Coprococcus  eutactus | Coprococcus | Lachnospiraceae | Eubacteriales | Clostridia | Firmicutes |
| Eubacteriales sp. (HG3A.0626) | T90 | -0.076 | 1.08E-05 | 8.94E-05 | 3364 | unclassified | unclassified | unclassified | unclassified | Eubacteriales | Clostridia | Firmicutes |
| Streptococcus anginosus  (HG3A.0680) | ODI | 0.076 | 1.35E-05 | 8.98E-05 | 3364 | unclassified | Streptococcus  anginosus | Streptococcus | Streptococcaceae | Lactobacillales | Bacilli | Firmicutes |
| Oscillospiraceae sp. (HG3A.0693) | AHI | -0.082 | 7.97E-06 | 8.99E-05 | 3004 | unclassified | unclassified | unclassified | Oscillospiraceae | Eubacteriales | Clostridia | Firmicutes |
| Staphylococcus aureus  (HG3A.1538) | T90 | 0.076 | 1.14E-05 | 9.44E-05 | 3364 | unclassified | Staphylococcus  aureus | Staphylococcus | Staphylococcacea  e | Bacillales | Bacilli | Firmicutes |
| Erysipelotrichales sp. (HG3A.1207) | T90 | 0.076 | 1.15E-05 | 9.46E-05 | 3364 | unclassified | unclassified | unclassified | unclassified | Erysipelotrichales | Erysipelotrichia | Firmicutes |
| Eubacteriales sp. (HG3A.0102) | AHI | -0.082 | 8.61E-06 | 9.64E-05 | 3004 | unclassified | unclassified | unclassified | unclassified | Eubacteriales | Clostridia | Firmicutes |
| Eubacteriales sp. (HG3A.0857) | T90 | -0.076 | 1.18E-05 | 9.64E-05 | 3364 | unclassified | unclassified | unclassified | unclassified | Eubacteriales | Clostridia | Firmicutes |

| Eubacteriales sp. (HG3A.0087) | AHI | -0.082 | 8.70E-06 | 9.68E-05 | 3004 | unclassified | unclassified | unclassified | unclassified | Eubacteriales | Clostridia | Firmicutes |
| --- | --- | --- | --- | --- | --- | --- | --- | --- | --- | --- | --- | --- |
| Clostridiaceae sp. (HG3A.0238) | AHI | -0.082 | 8.78E-06 | 9.70E-05 | 3004 | unclassified | unclassified | unclassified | Clostridiaceae | Eubacteriales | Clostridia | Firmicutes |
| Clostridia sp. (HG3A.0463) | AHI | -0.082 | 8.98E-06 | 9.86E-05 | 3004 | unclassified | unclassified | unclassified | unclassified | unclassified | Clostridia | Firmicutes |
| Clostridia sp. (HG3A.0550) | AHI | -0.082 | 9.05E-06 | 9.86E-05 | 3004 | unclassified | unclassified | unclassified | unclassified | unclassified | Clostridia | Firmicutes |
| Clostridia sp. (HG3A.0276) | T90 | -0.076 | 1.22E-05 | 9.92E-05 | 3364 | unclassified | unclassified | unclassified | unclassified | unclassified | Clostridia | Firmicutes |
| Candidatus Borkfalkiales sp. (HG3A.1397) | ODI | -0.075 | 1.52E-05 | 1.00E-04 | 3364 | unclassified | unclassified | unclassified | unclassified | Candidatus Borkfalkiales | Clostridia | Firmicutes |
| Eubacteriales sp. (HG3A.0441) | ODI | -0.075 | 1.51E-05 | 1.00E-04 | 3364 | unclassified | unclassified | unclassified | unclassified | Eubacteriales | Clostridia | Firmicutes |
| Lachnospiraceae sp. (HG3A.0748) | ODI | -0.075 | 1.54E-05 | 1.01E-04 | 3364 | unclassified | unclassified | unclassified | Lachnospiraceae | Eubacteriales | Clostridia | Firmicutes |
| Anaerotruncus massiliensis  (HG3A.0460) | ODI | -0.075 | 1.55E-05 | 1.02E-04 | 3364 | unclassified | Anaerotruncus  massiliensis | Anaerotruncus | Oscillospiraceae | Eubacteriales | Clostridia | Firmicutes |
| Firmicutes sp. (HG3A.0501) | AHI | -0.081 | 9.73E-06 | 1.05E-04 | 3004 | unclassified | unclassified | unclassified | unclassified | unclassified | unclassified | Firmicutes |
| Veillonella rogosae (HG3A.0324) | ODI | -0.075 | 1.62E-05 | 1.05E-04 | 3364 | unclassified | Veillonella rogosae | Veillonella | Veillonellaceae | Veillonellales | Negativicutes | Firmicutes |
| Victivallis lenta (HG3A.0525) | T90 | -0.076 | 1.33E-05 | 1.08E-04 | 3364 | unclassified | Victivallis lenta | Victivallis | Victivallaceae | Victivallales | Lentisphaeria | Lentisphaerae |
| Eubacteriales sp. (HG3A.0698) | T90 | 0.076 | 1.35E-05 | 1.09E-04 | 3364 | unclassified | unclassified | unclassified | unclassified | Eubacteriales | Clostridia | Firmicutes |
| Eubacteriales sp. (HG3A.0486) | ODI | -0.075 | 1.71E-05 | 1.11E-04 | 3364 | unclassified | unclassified | unclassified | unclassified | Eubacteriales | Clostridia | Firmicutes |
| Eubacteriales sp. (HG3A.0545) | T90 | -0.075 | 1.41E-05 | 1.13E-04 | 3364 | unclassified | unclassified | unclassified | unclassified | Eubacteriales | Clostridia | Firmicutes |
| Ruminococcus champanellensis (HG3A.0716) | ODI | -0.075 | 1.75E-05 | 1.13E-04 | 3364 | unclassified | Ruminococcus champanellensis | Ruminococcus | Oscillospiraceae | Eubacteriales | Clostridia | Firmicutes |
| Blastocystis sp. subtype 4  (HG3A.0446) | ODI | -0.075 | 1.77E-05 | 1.14E-04 | 3364 | unclassified | Blastocystis sp.  subtype 4 | Blastocystis | Blastocystidae | Opalinata | Bigyra | unclassified |
| Eubacteriales sp. (HG3A.0381) | T90 | -0.075 | 1.48E-05 | 1.18E-04 | 3364 | unclassified | unclassified | unclassified | unclassified | Eubacteriales | Clostridia | Firmicutes |
| Oscillospiraceae sp. (HG3A.0343) | AHI | -0.081 | 1.11E-05 | 1.19E-04 | 3004 | unclassified | unclassified | unclassified | Oscillospiraceae | Eubacteriales | Clostridia | Firmicutes |
| Clostridia sp. (HG3A.0645) | T90 | -0.075 | 1.53E-05 | 1.20E-04 | 3364 | unclassified | unclassified | unclassified | unclassified | unclassified | Clostridia | Firmicutes |
| Clostridia sp. (HG3A.0787) | T90 | -0.075 | 1.53E-05 | 1.20E-04 | 3364 | unclassified | unclassified | unclassified | unclassified | unclassified | Clostridia | Firmicutes |

| Eubacteriales sp. (HG3A.0188) | T90 | -0.075 | 1.53E-05 | 1.20E-04 | 3364 | unclassified | unclassified | unclassified | unclassified | Eubacteriales | Clostridia | Firmicutes |
| --- | --- | --- | --- | --- | --- | --- | --- | --- | --- | --- | --- | --- |
| Oscillospiraceae sp. (HG3A.0343) | T90 | -0.075 | 1.51E-05 | 1.20E-04 | 3364 | unclassified | unclassified | unclassified | Oscillospiraceae | Eubacteriales | Clostridia | Firmicutes |
| [Clostridium] symbiosum (HG3A.0370) | ODI | 0.074 | 1.88E-05 | 1.21E-04 | 3364 | unclassified | [Clostridium] symbiosum | Lachnoclostridium | Lachnospiraceae | Eubacteriales | Clostridia | Firmicutes |
| Oscillospiraceae sp. (HG3A.0475) | ODI | -0.074 | 1.90E-05 | 1.21E-04 | 3364 | unclassified | unclassified | unclassified | Oscillospiraceae | Eubacteriales | Clostridia | Firmicutes |
| Candidatus Borkfalkia ceftriaxoniphila (HG3A.0595) | T90 | -0.075 | 1.59E-05 | 1.24E-04 | 3364 | unclassified | Candidatus Borkfalkia ceftriaxoniphila | Candidatus Borkfalkia | Candidatus Borkfalkiaceae | Candidatus Borkfalkiales | Clostridia | Firmicutes |
| Eubacteriales sp. (HG3A.0342) | AHI | -0.081 | 1.16E-05 | 1.24E-04 | 3004 | unclassified | unclassified | unclassified | unclassified | Eubacteriales | Clostridia | Firmicutes |
| Oscillospiraceae sp. (HG3A.0849) | T90 | -0.075 | 1.62E-05 | 1.25E-04 | 3364 | unclassified | unclassified | unclassified | Oscillospiraceae | Eubacteriales | Clostridia | Firmicutes |
| Anaerostipes caccae (HG3A.0747) | AHI | 0.081 | 1.20E-05 | 1.28E-04 | 3004 | unclassified | Anaerostipes caccae | Anaerostipes | Lachnospiraceae | Eubacteriales | Clostridia | Firmicutes |
| Oscillospiraceae sp. (HG3A.0461) | AHI | -0.081 | 1.21E-05 | 1.28E-04 | 3004 | unclassified | unclassified | unclassified | Oscillospiraceae | Eubacteriales | Clostridia | Firmicutes |
| Eubacteriales sp. (HG3A.0711) | ODI | -0.074 | 2.06E-05 | 1.31E-04 | 3364 | unclassified | unclassified | unclassified | unclassified | Eubacteriales | Clostridia | Firmicutes |
| Butyricicoccus sp. (HG3A.0008) | AHI | 0.08 | 1.27E-05 | 1.33E-04 | 3004 | unclassified | unclassified | Butyricicoccus | Clostridiaceae | Eubacteriales | Clostridia | Firmicutes |
| Eubacteriales sp. (HG3A.0128) | T90 | 0.075 | 1.73E-05 | 1.33E-04 | 3364 | unclassified | unclassified | unclassified | unclassified | Eubacteriales | Clostridia | Firmicutes |
| Anaerobutyricum hallii (HG3A.0112) | ODI | 0.074 | 2.13E-05 | 1.35E-04 | 3364 | unclassified | Anaerobutyricum hallii | Anaerobutyricum | Lachnospiraceae | Eubacteriales | Clostridia | Firmicutes |
| Blautia sp. SG-772 (HG3A.0063) | ODI | 0.074 | 2.16E-05 | 1.36E-04 | 3364 | unclassified | Blautia sp. SG-772 | Blautia | Lachnospiraceae | Eubacteriales | Clostridia | Firmicutes |
| Clostridium sp. OF03-18AA (HG3A.0119) | ODI | -0.074 | 2.15E-05 | 1.36E-04 | 3364 | unclassified | Clostridium sp. OF03 18AA | Clostridium | Clostridiaceae | Eubacteriales | Clostridia | Firmicutes |
| Eubacteriales sp. (HG3A.0263) | ODI | -0.074 | 2.18E-05 | 1.36E-04 | 3364 | unclassified | unclassified | unclassified | unclassified | Eubacteriales | Clostridia | Firmicutes |
| Candidatus Borkfalkia ceftriaxoniphila (HG3A.0595) | ODI | -0.074 | 2.23E-05 | 1.37E-04 | 3364 | unclassified | Candidatus Borkfalkia ceftriaxoniphila | Candidatus Borkfalkia | Candidatus Borkfalkiaceae | Candidatus Borkfalkiales | Clostridia | Firmicutes |
| Eubacteriales sp. (HG3A.0280) | AHI | -0.08 | 1.31E-05 | 1.37E-04 | 3004 | unclassified | unclassified | unclassified | unclassified | Eubacteriales | Clostridia | Firmicutes |
| Eubacteriales sp. (HG3A.0537) | ODI | -0.074 | 2.21E-05 | 1.37E-04 | 3364 | unclassified | unclassified | unclassified | unclassified | Eubacteriales | Clostridia | Firmicutes |

Haemophilus parainfluenzae

(HG3A.0181) ODI -0.074 2.22E-05 1.37E-04 3364 unclassified

Haemophilus

parainfluenzae Haemophilus Pasteurellaceae Pasteurellales

Gammaproteob

acteria Proteobacteria

| Oscillospiraceae sp. (HG3A.0507) | ODI | -0.074 | 2.20E-05 | 1.37E-04 | 3364 | unclassified | unclassified | unclassified | Oscillospiraceae | Eubacteriales | Clostridia | Firmicutes |
| --- | --- | --- | --- | --- | --- | --- | --- | --- | --- | --- | --- | --- |
| Clostridia sp. (HG3A.0750) | AHI | -0.08 | 1.36E-05 | 1.39E-04 | 3004 | unclassified | unclassified | unclassified | unclassified | unclassified | Clostridia | Firmicutes |
| Eubacteriales sp. (HG3A.0184) | AHI | -0.08 | 1.35E-05 | 1.39E-04 | 3004 | unclassified | unclassified | unclassified | unclassified | Eubacteriales | Clostridia | Firmicutes |
| Firmicutes sp. (HG3A.0650) | AHI | -0.08 | 1.37E-05 | 1.40E-04 | 3004 | unclassified | unclassified | unclassified | unclassified | unclassified | unclassified | Firmicutes |
| Eubacteriales sp. (HG3A.0537) | T90 | -0.074 | 1.86E-05 | 1.42E-04 | 3364 | unclassified | unclassified | unclassified | unclassified | Eubacteriales | Clostridia | Firmicutes |
| Eubacteriales sp. (HG3A.0577) | AHI | -0.08 | 1.40E-05 | 1.42E-04 | 3004 | unclassified | unclassified | unclassified | unclassified | Eubacteriales | Clostridia | Firmicutes |
| Veillonella rogosae (HG3A.0324) | T90 | -0.074 | 1.86E-05 | 1.42E-04 | 3364 | unclassified | Veillonella rogosae | Veillonella | Veillonellaceae | Veillonellales | Negativicutes | Firmicutes |
| Eubacteriales sp. (HG3A.0377) | ODI | -0.074 | 2.33E-05 | 1.43E-04 | 3364 | unclassified | unclassified | unclassified | unclassified | Eubacteriales | Clostridia | Firmicutes |
| Eubacteriales sp. (HG3A.0230) | AHI | -0.08 | 1.47E-05 | 1.48E-04 | 3004 | unclassified | unclassified | unclassified | unclassified | Eubacteriales | Clostridia | Firmicutes |
| Eubacteriales sp. (HG3A.0637) | ODI | -0.073 | 2.43E-05 | 1.49E-04 | 3364 | unclassified | unclassified | unclassified | unclassified | Eubacteriales | Clostridia | Firmicutes |
| Eubacteriales sp. (HG3A.0280) | ODI | -0.073 | 2.51E-05 | 1.53E-04 | 3364 | unclassified | unclassified | unclassified | unclassified | Eubacteriales | Clostridia | Firmicutes |
| Firmicutes sp. (HG3A.0587) | T90 | -0.074 | 2.01E-05 | 1.53E-04 | 3364 | unclassified | unclassified | unclassified | unclassified | unclassified | unclassified | Firmicutes |
| Bacteroidales sp. (HG3A.1236) | ODI | -0.073 | 2.54E-05 | 1.54E-04 | 3364 | unclassified | unclassified | unclassified | unclassified | Bacteroidales | Bacteroidia | Bacteroidetes |
| Eubacteriales sp. (HG3A.0594) | ODI | -0.073 | 2.57E-05 | 1.55E-04 | 3364 | unclassified | unclassified | unclassified | unclassified | Eubacteriales | Clostridia | Firmicutes |
| Eubacteriales sp. (HG3A.0630) | AHI | -0.079 | 1.57E-05 | 1.57E-04 | 3004 | unclassified | unclassified | unclassified | unclassified | Eubacteriales | Clostridia | Firmicutes |
| Clostridia sp. (HG3A.0512) | T90 | -0.074 | 2.11E-05 | 1.60E-04 | 3364 | unclassified | unclassified | unclassified | unclassified | unclassified | Clostridia | Firmicutes |
| Eubacteriales sp. (HG3A.1086) | T90 | -0.074 | 2.13E-05 | 1.60E-04 | 3364 | unclassified | unclassified | unclassified | unclassified | Eubacteriales | Clostridia | Firmicutes |
| Firmicutes sp. (HG3A.0454) | AHI | -0.079 | 1.61E-05 | 1.60E-04 | 3004 | unclassified | unclassified | unclassified | unclassified | unclassified | unclassified | Firmicutes |
| Eubacteriales sp. (HG3A.0453) | T90 | -0.074 | 2.18E-05 | 1.63E-04 | 3364 | unclassified | unclassified | unclassified | unclassified | Eubacteriales | Clostridia | Firmicutes |
| Eubacteriales sp. (HG3A.0635) | ODI | -0.073 | 2.71E-05 | 1.63E-04 | 3364 | unclassified | unclassified | unclassified | unclassified | Eubacteriales | Clostridia | Firmicutes |
| Oscillospiraceae sp. (HG3A.0475) | T90 | -0.074 | 2.20E-05 | 1.64E-04 | 3364 | unclassified | unclassified | unclassified | Oscillospiraceae | Eubacteriales | Clostridia | Firmicutes |
| Clostridia sp. (HG3A.1504) | AHI | -0.079 | 1.69E-05 | 1.66E-04 | 3004 | unclassified | unclassified | unclassified | unclassified | unclassified | Clostridia | Firmicutes |

| Eubacteriales sp. (HG3A.0578) | AHI | -0.079 | 1.69E-05 | 1.66E-04 | 3004 | unclassified | unclassified | unclassified | unclassified | Eubacteriales | Clostridia | Firmicutes |
| --- | --- | --- | --- | --- | --- | --- | --- | --- | --- | --- | --- | --- |
| Lactobacillus gasseri (HG3A.0884) | T90 | 0.074 | 2.26E-05 | 1.67E-04 | 3364 | unclassified | Lactobacillus gasseri | Lactobacillus | Lactobacillaceae | Lactobacillales | Bacilli | Firmicutes |
| Eubacteriales sp. (HG3A.0338) | AHI | -0.079 | 1.80E-05 | 1.76E-04 | 3004 | unclassified | unclassified | unclassified | unclassified | Eubacteriales | Clostridia | Firmicutes |
| Firmicutes sp. (HG3A.0596) | T90 | -0.073 | 2.40E-05 | 1.77E-04 | 3364 | unclassified | unclassified | unclassified | unclassified | unclassified | unclassified | Firmicutes |
| Lachnospiraceae sp. (HG3A.0399) | AHI | -0.079 | 1.83E-05 | 1.77E-04 | 3004 | unclassified | unclassified | unclassified | Lachnospiraceae | Eubacteriales | Clostridia | Firmicutes |
| Eubacteriales sp. (HG3A.0396) | AHI | -0.079 | 1.84E-05 | 1.78E-04 | 3004 | unclassified | unclassified | unclassified | unclassified | Eubacteriales | Clostridia | Firmicutes |
| Victivallis vadensis (HG3A.0689) | AHI | -0.079 | 1.86E-05 | 1.79E-04 | 3004 | unclassified | Victivallis vadensis | Victivallis | Victivallaceae | Victivallales | Lentisphaeria | Lentisphaerae |
| Clostridia sp. (HG3A.0645) | ODI | -0.072 | 3.05E-05 | 1.83E-04 | 3364 | unclassified | unclassified | unclassified | unclassified | unclassified | Clostridia | Firmicutes |
| Oscillibacter sp. (HG3A.0245) | AHI | -0.079 | 1.92E-05 | 1.83E-04 | 3004 | unclassified | unclassified | Oscillibacter | Oscillospiraceae | Eubacteriales | Clostridia | Firmicutes |
| Parvimonas micra (HG3A.1231) | AHI | 0.079 | 1.95E-05 | 1.85E-04 | 3004 | unclassified | Parvimonas micra | Parvimonas | Peptoniphilaceae | Tissierellales | Tissierellia | Firmicutes |
| Streptococcus mutans (HG3A.0677) | T90 | 0.073 | 2.58E-05 | 1.90E-04 | 3364 | unclassified | Streptococcus mutans | Streptococcus | Streptococcaceae | Lactobacillales | Bacilli | Firmicutes |
| Eubacteriales sp. (HG3A.0518) | ODI | -0.072 | 3.23E-05 | 1.93E-04 | 3364 | unclassified | unclassified | unclassified | unclassified | Eubacteriales | Clostridia | Firmicutes |
| Firmicutes sp. (HG3A.0581) | T90 | -0.073 | 2.66E-05 | 1.95E-04 | 3364 | unclassified | unclassified | unclassified | unclassified | unclassified | unclassified | Firmicutes |
| Firmicutes sp. (HG3A.0526) | T90 | -0.073 | 2.77E-05 | 2.02E-04 | 3364 | unclassified | unclassified | unclassified | unclassified | unclassified | unclassified | Firmicutes |
| Clostridia sp. (HG3A.1504) | ODI | -0.072 | 3.41E-05 | 2.03E-04 | 3364 | unclassified | unclassified | unclassified | unclassified | unclassified | Clostridia | Firmicutes |
| Butyricimonas virosa  (HG3A.0199) | T90 | -0.073 | 2.83E-05 | 2.05E-04 | 3364 | unclassified | Butyricimonas virosa | Butyricimonas | Odoribacteraceae | Bacteroidales | Bacteroidia | Bacteroidetes |
| Clostridia sp. (HG3A.0733) | T90 | -0.073 | 2.88E-05 | 2.08E-04 | 3364 | unclassified | unclassified | unclassified | unclassified | unclassified | Clostridia | Firmicutes |
| Ruthenibacterium lactatiformans (HG3A.0020) | T90 | 0.073 | 2.92E-05 | 2.09E-04 | 3364 | unclassified | Ruthenibacterium lactatiformans | Ruthenibacterium | Oscillospiraceae | Eubacteriales | Clostridia | Firmicutes |
| Eubacteriales sp. (HG3A.0161) | T90 | -0.073 | 2.96E-05 | 2.11E-04 | 3364 | unclassified | unclassified | unclassified | unclassified | Eubacteriales | Clostridia | Firmicutes |
| Eubacteriales sp. (HG3A.0505) | T90 | -0.073 | 3.01E-05 | 2.14E-04 | 3364 | unclassified | unclassified | unclassified | unclassified | Eubacteriales | Clostridia | Firmicutes |
| Eubacteriales sp. (HG3A.0548) | ODI | -0.072 | 3.62E-05 | 2.14E-04 | 3364 | unclassified | unclassified | unclassified | unclassified | Eubacteriales | Clostridia | Firmicutes |

| Lachnospiraceae sp. (HG3A.0127) | ODI | -0.072 | 3.60E-05 | 2.14E-04 | 3364 | unclassified | unclassified | unclassified | Lachnospiraceae | Eubacteriales | Clostridia | Firmicutes |
| --- | --- | --- | --- | --- | --- | --- | --- | --- | --- | --- | --- | --- |
| Eubacteriales sp. (HG3A.0757) | T90 | -0.072 | 3.04E-05 | 2.16E-04 | 3364 | unclassified | unclassified | unclassified | unclassified | Eubacteriales | Clostridia | Firmicutes |
| Oscillibacter sp. (HG3A.0734) | ODI | -0.072 | 3.83E-05 | 2.26E-04 | 3364 | unclassified | unclassified | Oscillibacter | Oscillospiraceae | Eubacteriales | Clostridia | Firmicutes |
| Clostridium sp. (HG3A.0050) | AHI | 0.078 | 2.43E-05 | 2.28E-04 | 3004 | unclassified | unclassified | Clostridium | Clostridiaceae | Eubacteriales | Clostridia | Firmicutes |
| Eubacteriales sp. (HG3A.0409) | AHI | -0.078 | 2.44E-05 | 2.28E-04 | 3004 | unclassified | unclassified | unclassified | unclassified | Eubacteriales | Clostridia | Firmicutes |
| Eubacteriales sp. (HG3A.0443) | AHI | -0.078 | 2.46E-05 | 2.29E-04 | 3004 | unclassified | unclassified | unclassified | unclassified | Eubacteriales | Clostridia | Firmicutes |
| Alistipes indistinctus (HG3A.0121) | ODI | -0.071 | 3.92E-05 | 2.30E-04 | 3364 | unclassified | Alistipes indistinctus | Alistipes | Rikenellaceae | Bacteroidales | Bacteroidia | Bacteroidetes |
| Oscillospiraceae sp. (HG3A.0944) | ODI | -0.071 | 3.98E-05 | 2.33E-04 | 3364 | unclassified | unclassified | unclassified | Oscillospiraceae | Eubacteriales | Clostridia | Firmicutes |
| Eubacteriales sp. (HG3A.0637) | T90 | -0.072 | 3.31E-05 | 2.34E-04 | 3364 | unclassified | unclassified | unclassified | unclassified | Eubacteriales | Clostridia | Firmicutes |
| Firmicutes sp. (HG3A.0681) | T90 | -0.072 | 3.34E-05 | 2.35E-04 | 3364 | unclassified | unclassified | unclassified | unclassified | unclassified | unclassified | Firmicutes |
| Sellimonas intestinalis  (HG3A.0417) | AHI | 0.078 | 2.54E-05 | 2.35E-04 | 3004 | unclassified | Sellimonas  intestinalis | Sellimonas | Lachnospiraceae | Eubacteriales | Clostridia | Firmicutes |
| Dorea longicatena (HG3A.0039) | AHI | 0.077 | 2.59E-05 | 2.38E-04 | 3004 | unclassified | Dorea longicatena | Dorea | Lachnospiraceae | Eubacteriales | Clostridia | Firmicutes |
| Blastocystis sp. subtype 4  (HG3A.0446) | T90 | -0.072 | 3.45E-05 | 2.40E-04 | 3364 | unclassified | Blastocystis sp.  subtype 4 | Blastocystis | Blastocystidae | Opalinata | Bigyra | unclassified |
| Eubacteriales sp. (HG3A.0715) | ODI | -0.071 | 4.11E-05 | 2.40E-04 | 3364 | unclassified | unclassified | unclassified | unclassified | Eubacteriales | Clostridia | Firmicutes |
| Oscillospiraceae sp. (HG3A.0146) | T90 | -0.072 | 3.46E-05 | 2.40E-04 | 3364 | unclassified | unclassified | unclassified | Oscillospiraceae | Eubacteriales | Clostridia | Firmicutes |
| Pseudoflavonifractor sp. (HG3A.0844) | T90 | -0.072 | 3.44E-05 | 2.40E-04 | 3364 | unclassified | unclassified | Pseudoflavonifractor | Oscillospiraceae | Eubacteriales | Clostridia | Firmicutes |
| Clostridium sp. TM06-18  (HG3A.0048) | AHI | 0.077 | 2.68E-05 | 2.46E-04 | 3004 | unclassified | Clostridium sp.  TM06-18 | Clostridium | Clostridiaceae | Eubacteriales | Clostridia | Firmicutes |
| Oscillospiraceae sp. (HG3A.0256) | AHI | 0.077 | 2.71E-05 | 2.46E-04 | 3004 | unclassified | unclassified | unclassified | Oscillospiraceae | Eubacteriales | Clostridia | Firmicutes |
| Collinsella sp. WCA1-178-WT-3  (M2) (HG3A.1245) | ODI | 0.071 | 4.27E-05 | 2.48E-04 | 3364 | unclassified | Collinsella sp. WCA1  178-WT-3 (M2) | Collinsella | Coriobacteriaceae | Coriobacteriales | Coriobacteriia | Actinobacteria |
| Alloscardovia omnicolens (HG3A.1279) | ODI | 0.071 | 4.37E-05 | 2.53E-04 | 3364 | unclassified | Alloscardovia omnicolens | Alloscardovia | Bifidobacteriaceae | Bifidobacteriales | Actinomycetia | Actinobacteria |
| Eubacteriales sp. (HG3A.0235) | T90 | -0.072 | 3.67E-05 | 2.54E-04 | 3364 | unclassified | unclassified | unclassified | unclassified | Eubacteriales | Clostridia | Firmicutes |
| Streptococcus gordonii (HG3A.0713) | ODI | 0.071 | 4.43E-05 | 2.55E-04 | 3364 | unclassified | Streptococcus gordonii | Streptococcus | Streptococcaceae | Lactobacillales | Bacilli | Firmicutes |

| Lachnospiraceae sp. (HG3A.0748) | AHI | -0.077 | 2.83E-05 | 2.56E-04 | 3004 | unclassified | unclassified | unclassified | Lachnospiraceae | Eubacteriales | Clostridia | Firmicutes |
| --- | --- | --- | --- | --- | --- | --- | --- | --- | --- | --- | --- | --- |
| Eubacteriales sp. (HG3A.0652) | T90 | -0.072 | 3.74E-05 | 2.57E-04 | 3364 | unclassified | unclassified | unclassified | unclassified | Eubacteriales | Clostridia | Firmicutes |
| Victivallales sp. (HG3A.0824) | ODI | -0.071 | 4.64E-05 | 2.66E-04 | 3364 | unclassified | unclassified | unclassified | unclassified | Victivallales | Lentisphaeria | Lentisphaerae |
| Eubacteriales sp. (HG3A.0758) | T90 | -0.071 | 3.90E-05 | 2.67E-04 | 3364 | unclassified | unclassified | unclassified | unclassified | Eubacteriales | Clostridia | Firmicutes |
| Eubacteriales sp. (HG3A.0136) | ODI | -0.071 | 4.76E-05 | 2.72E-04 | 3364 | unclassified | unclassified | unclassified | unclassified | Eubacteriales | Clostridia | Firmicutes |
| Coprococcus eutactus (HG3A.0155) | AHI | -0.077 | 3.06E-05 | 2.75E-04 | 3004 | unclassified | Coprococcus eutactus | Coprococcus | Lachnospiraceae | Eubacteriales | Clostridia | Firmicutes |
| Firmicutes sp. (HG3A.0526) | ODI | -0.071 | 4.88E-05 | 2.78E-04 | 3364 | unclassified | unclassified | unclassified | unclassified | unclassified | unclassified | Firmicutes |
| Eubacteriales sp. (HG3A.0408) | T90 | -0.071 | 4.15E-05 | 2.83E-04 | 3364 | unclassified | unclassified | unclassified | unclassified | Eubacteriales | Clostridia | Firmicutes |
| Collinsella sp. WCA1-178-WT-3  (M2) (HG3A.1245) | AHI | 0.077 | 3.18E-05 | 2.84E-04 | 3004 | unclassified | Collinsella sp. WCA1  178-WT-3 (M2) | Collinsella | Coriobacteriaceae | Coriobacteriales | Coriobacteriia | Actinobacteria |
| Clostridia sp. (HG3A.0011) | ODI | 0.07 | 5.02E-05 | 2.85E-04 | 3364 | unclassified | unclassified | unclassified | unclassified | unclassified | Clostridia | Firmicutes |
| Eubacteriales sp. (HG3A.0305) | AHI | -0.077 | 3.24E-05 | 2.88E-04 | 3004 | unclassified | unclassified | unclassified | unclassified | Eubacteriales | Clostridia | Firmicutes |
| Eubacteriales sp. (HG3A.0386) | ODI | -0.07 | 5.09E-05 | 2.88E-04 | 3364 | unclassified | unclassified | unclassified | unclassified | Eubacteriales | Clostridia | Firmicutes |
| Eggerthella lenta (HG3A.0225) | ODI | 0.07 | 5.12E-05 | 2.89E-04 | 3364 | unclassified | Eggerthella lenta | Eggerthella | Eggerthellaceae | Eggerthellales | Coriobacteriia | Actinobacteria |
| [Clostridium] innocuum (HG3A.0365) | AHI | 0.076 | 3.28E-05 | 2.90E-04 | 3004 | unclassified | [Clostridium] innocuum | Erysipelatoclostridiu m | Erysipelotrichace ae | Erysipelotrichales | Erysipelotrichia | Firmicutes |
| Eubacteriales sp. (HG3A.0908) | T90 | -0.071 | 4.33E-05 | 2.94E-04 | 3364 | unclassified | unclassified | unclassified | unclassified | Eubacteriales | Clostridia | Firmicutes |
| Eubacteriales sp. (HG3A.0881) | ODI | -0.07 | 5.26E-05 | 2.96E-04 | 3364 | unclassified | unclassified | unclassified | unclassified | Eubacteriales | Clostridia | Firmicutes |
| Eubacteriales sp. (HG3A.1227) | ODI | -0.07 | 5.40E-05 | 3.02E-04 | 3364 | unclassified | unclassified | unclassified | unclassified | Eubacteriales | Clostridia | Firmicutes |
| Coprococcus sp. (HG3A.0404) | T90 | 0.071 | 4.52E-05 | 3.04E-04 | 3364 | unclassified | unclassified | Coprococcus | Lachnospiraceae | Eubacteriales | Clostridia | Firmicutes |
| Eubacteriales sp. (HG3A.0244) | T90 | -0.071 | 4.50E-05 | 3.04E-04 | 3364 | unclassified | unclassified | unclassified | unclassified | Eubacteriales | Clostridia | Firmicutes |
| Eubacteriales sp. (HG3A.0371) | T90 | -0.071 | 4.87E-05 | 3.24E-04 | 3364 | unclassified | unclassified | unclassified | unclassified | Eubacteriales | Clostridia | Firmicutes |
| Firmicutes sp. (HG3A.1054) | T90 | -0.071 | 4.85E-05 | 3.24E-04 | 3364 | unclassified | unclassified | unclassified | unclassified | unclassified | unclassified | Firmicutes |
| Pediococcus acidilactici (HG3A.1468) | T90 | 0.071 | 4.85E-05 | 3.24E-04 | 3364 | unclassified | Pediococcus acidilactici | Pediococcus | Lactobacillaceae | Lactobacillales | Bacilli | Firmicutes |

| Clostridia sp. (HG3A.0767) | T90 | -0.071 | 4.95E-05 | 3.26E-04 | 3364 | unclassified | unclassified | unclassified | unclassified | unclassified | Clostridia | Firmicutes |
| --- | --- | --- | --- | --- | --- | --- | --- | --- | --- | --- | --- | --- |
| Eubacteriales sp. (HG3A.0652) | ODI | -0.07 | 5.86E-05 | 3.26E-04 | 3364 | unclassified | unclassified | unclassified | unclassified | Eubacteriales | Clostridia | Firmicutes |
| Eubacteriales sp. (HG3A.0671) | T90 | -0.071 | 4.93E-05 | 3.26E-04 | 3364 | unclassified | unclassified | unclassified | unclassified | Eubacteriales | Clostridia | Firmicutes |
| Eubacteriales sp. (HG3A.0870) | ODI | -0.07 | 5.84E-05 | 3.26E-04 | 3364 | unclassified | unclassified | unclassified | unclassified | Eubacteriales | Clostridia | Firmicutes |
| Bacteria sp. (HG3A.0218) | ODI | -0.07 | 6.20E-05 | 3.42E-04 | 3364 | unclassified | unclassified | unclassified | unclassified | unclassified | unclassified | unclassified |
| Clostridia sp. (HG3A.1039) | ODI | -0.07 | 6.19E-05 | 3.42E-04 | 3364 | unclassified | unclassified | unclassified | unclassified | unclassified | Clostridia | Firmicutes |
| Acidaminococcus intestini  (HG3A.0407) | AHI | 0.076 | 3.90E-05 | 3.43E-04 | 3004 | unclassified | Acidaminococcus  intestini | Acidaminococcus | Acidaminococcac  eae | Acidaminococcales | Negativicutes | Firmicutes |
| Eubacteriales sp. (HG3A.0786) | T90 | 0.07 | 5.23E-05 | 3.44E-04 | 3364 | unclassified | unclassified | unclassified | unclassified | Eubacteriales | Clostridia | Firmicutes |
| Roseburia sp. AM59-24XD  (HG3A.0391) | ODI | -0.07 | 6.25E-05 | 3.44E-04 | 3364 | unclassified | Roseburia sp. AM59-  24XD | Roseburia | Lachnospiraceae | Eubacteriales | Clostridia | Firmicutes |
| Eubacteriales sp. (HG3A.0232) | ODI | -0.07 | 6.32E-05 | 3.47E-04 | 3364 | unclassified | unclassified | unclassified | unclassified | Eubacteriales | Clostridia | Firmicutes |
| Clostridia sp. (HG3A.0787) | AHI | -0.076 | 4.05E-05 | 3.55E-04 | 3004 | unclassified | unclassified | unclassified | unclassified | unclassified | Clostridia | Firmicutes |
| Eubacteriales sp. (HG3A.0289) | ODI | -0.069 | 6.52E-05 | 3.55E-04 | 3364 | unclassified | unclassified | unclassified | unclassified | Eubacteriales | Clostridia | Firmicutes |
| Eubacteriales sp. (HG3A.0593) | ODI | -0.069 | 6.50E-05 | 3.55E-04 | 3364 | unclassified | unclassified | unclassified | unclassified | Eubacteriales | Clostridia | Firmicutes |
| Eubacteriales sp. (HG3A.0956) | ODI | -0.069 | 6.54E-05 | 3.55E-04 | 3364 | unclassified | unclassified | unclassified | unclassified | Eubacteriales | Clostridia | Firmicutes |
| Ruminococcus sp. AF46-10NS  (HG3A.0271) | AHI | 0.075 | 4.11E-05 | 3.58E-04 | 3004 | unclassified | Ruminococcus sp.  AF46-10NS | Ruminococcus | Oscillospiraceae | Eubacteriales | Clostridia | Firmicutes |
| Victivallales sp. (HG3A.0824) | T90 | -0.07 | 5.47E-05 | 3.58E-04 | 3364 | unclassified | unclassified | unclassified | unclassified | Victivallales | Lentisphaeria | Lentisphaerae |
| Subdoligranulum sp. APC924/74  (HG3A.0015) | ODI | -0.069 | 6.79E-05 | 3.68E-04 | 3364 | unclassified | Subdoligranulum sp.  APC924/74 | Subdoligranulum | Oscillospiraceae | Eubacteriales | Clostridia | Firmicutes |
| Lachnospiraceae sp. (HG3A.0393) | T90 | 0.07 | 5.69E-05 | 3.69E-04 | 3364 | unclassified | unclassified | unclassified | Lachnospiraceae | Eubacteriales | Clostridia | Firmicutes |
| Oscillospiraceae sp. (HG3A.0616) | T90 | -0.07 | 5.70E-05 | 3.69E-04 | 3364 | unclassified | unclassified | unclassified | Oscillospiraceae | Eubacteriales | Clostridia | Firmicutes |
| Bacteroides cellulosilyticus (HG3A.0108) | ODI | -0.069 | 6.93E-05 | 3.73E-04 | 3364 | unclassified | Bacteroides cellulosilyticus | Bacteroides | Bacteroidaceae | Bacteroidales | Bacteroidia | Bacteroidetes |
| Eubacterium sp. (HG3A.0214) | ODI | 0.069 | 6.94E-05 | 3.73E-04 | 3364 | unclassified | unclassified | Eubacterium | Eubacteriaceae | Eubacteriales | Clostridia | Firmicutes |

| Firmicutes sp. (HG3A.0301) | ODI | -0.069 | 7.07E-05 | 3.79E-04 | 3364 | unclassified | unclassified | unclassified | unclassified | unclassified | unclassified | Firmicutes |
| --- | --- | --- | --- | --- | --- | --- | --- | --- | --- | --- | --- | --- |
| Eubacteriales sp. (HG3A.0349) | AHI | -0.075 | 4.39E-05 | 3.80E-04 | 3004 | unclassified | unclassified | unclassified | unclassified | Eubacteriales | Clostridia | Firmicutes |
| Eggerthellales sp. (HG3A.0177) | T90 | -0.07 | 5.90E-05 | 3.81E-04 | 3364 | unclassified | unclassified | unclassified | unclassified | Eggerthellales | Coriobacteriia | Actinobacteria |
| Bacteria sp. (HG3A.0361) | T90 | -0.07 | 6.13E-05 | 3.94E-04 | 3364 | unclassified | unclassified | unclassified | unclassified | unclassified | unclassified | unclassified |
| Firmicutes sp. (HG3A.0541) | AHI | -0.075 | 4.63E-05 | 3.99E-04 | 3004 | unclassified | unclassified | unclassified | unclassified | unclassified | unclassified | Firmicutes |
| Clostridium sp. OF03-18AA (HG3A.0119)  Erysipelatoclostridium sp. (HG3A.0313) | AHI  AHI | -0.075  -0.075 | 4.76E-05  4.88E-05 | 4.08E-04  4.16E-04 | 3004  3004 | unclassified  unclassified | Clostridium sp. OF03 18AA  unclassified E | Clostridium  rysipelatoclostridi m | Clostridiaceae  u Erysipelotrichace ae | Eubacteriales  Erysipelotrichales | Clostridia  Erysipelotrichia | Firmicutes  Firmicutes |
| Eubacteriales sp. (HG3A.0377) | AHI | -0.075 | 4.91E-05 | 4.16E-04 | 3004 | unclassified | unclassified | unclassified | unclassified | Eubacteriales | Clostridia | Firmicutes |
| Eubacteriales sp. (HG3A.0230) | ODI | -0.069 | 7.83E-05 | 4.18E-04 | 3364 | unclassified | unclassified | unclassified | unclassified | Eubacteriales | Clostridia | Firmicutes |
| Bacteria sp. (HG3A.0492) | AHI | -0.075 | 5.05E-05 | 4.26E-04 | 3004 | unclassified | unclassified | unclassified | unclassified | unclassified | unclassified | unclassified |
| Eubacteriales sp. (HG3A.0158) | ODI | -0.069 | 8.09E-05 | 4.31E-04 | 3364 | unclassified | unclassified | unclassified | unclassified | Eubacteriales | Clostridia | Firmicutes |
| Rothia mucilaginosa (HG3A.0559) | AHI | 0.075 | 5.17E-05 | 4.34E-04 | 3004 | unclassified | Rothia mucilaginosa | Rothia | Micrococcaceae | Micrococcales | Actinomycetia | Actinobacteria |
| Eubacteriales sp. (HG3A.0457) | ODI | -0.068 | 8.41E-05 | 4.46E-04 | 3364 | unclassified | unclassified | unclassified | unclassified | Eubacteriales | Clostridia | Firmicutes |
| Eubacteriales sp. (HG3A.0594) | T90 | -0.069 | 6.99E-05 | 4.48E-04 | 3364 | unclassified | unclassified | unclassified | unclassified | Eubacteriales | Clostridia | Firmicutes |
| Oscillospiraceae sp. (HG3A.0256) | ODI | 0.068 | 8.52E-05 | 4.51E-04 | 3364 | unclassified | unclassified | unclassified | Oscillospiraceae | Eubacteriales | Clostridia | Firmicutes |
| Eubacteriales sp. (HG3A.0592) | T90 | -0.069 | 7.08E-05 | 4.52E-04 | 3364 | unclassified | unclassified | unclassified | unclassified | Eubacteriales | Clostridia | Firmicutes |
| Anaerostipes sp. BG01 (HG3A.1509) | ODI | 0.068 | 8.72E-05 | 4.60E-04 | 3364 | unclassified | Anaerostipes sp.  BG01 | Anaerostipes | Lachnospiraceae | Eubacteriales | Clostridia | Firmicutes |
| Oscillospiraceae sp. (HG3A.0612) | T90 | -0.069 | 7.28E-05 | 4.63E-04 | 3364 | unclassified | unclassified | unclassified | Oscillospiraceae | Eubacteriales | Clostridia | Firmicutes |
| Eubacteriales sp. (HG3A.0087) | ODI | -0.068 | 8.89E-05 | 4.67E-04 | 3364 | unclassified | unclassified | unclassified | unclassified | Eubacteriales | Clostridia | Firmicutes |
| Eubacteriales sp. (HG3A.0128) | ODI | 0.068 | 9.23E-05 | 4.83E-04 | 3364 | unclassified | unclassified | unclassified | unclassified | Eubacteriales | Clostridia | Firmicutes |
| Firmicutes sp. (HG3A.1075) | T90 | -0.069 | 7.67E-05 | 4.86E-04 | 3364 | unclassified | unclassified | unclassified | unclassified | unclassified | unclassified | Firmicutes |
| Eubacteriales sp. (HG3A.0270) | ODI | -0.068 | 9.51E-05 | 4.96E-04 | 3364 | unclassified | unclassified | unclassified | unclassified | Eubacteriales | Clostridia | Firmicutes |

Rikenellaceae sp. (HG3A.1022) ODI -0.068 9.56E-05 4.97E-04 3364 unclassified unclassified unclassified Rikenellaceae Bacteroidales Bacteroidia Bacteroidetes

Methanobrevibacter smithii

(HG3A.0152)

T90 -0.069 8.05E-05 5.08E-04 3364 unclassified Methanobrevibacter

smithii

Methanobrevibacter Methanobacteriac

eae

Methanobacteriales Methanobacteria Euryarchaeota

Firmicutes sp. (HG3A.1082) ODI -0.068 9.81E-05 5.09E-04 3364 unclassified unclassified unclassified unclassified unclassified unclassified Firmicutes

Eubacteriales sp. (HG3A.0264) T90 -0.068 8.16E-05 5.13E-04 3364 unclassified unclassified unclassified unclassified Eubacteriales Clostridia Firmicutes

Eubacteriales sp. (HG3A.0320) ODI -0.068 9.92E-05 5.13E-04 3364 unclassified unclassified unclassified unclassified Eubacteriales Clostridia Firmicutes

Alloscardovia omnicolens

(HG3A.1279)

T90 0.068 8.29E-05 5.17E-04 3364 unclassified Alloscardovia

omnicolens

Alloscardovia Bifidobacteriaceae Bifidobacteriales Actinomycetia Actinobacteria

Firmicutes sp. (HG3A.0541)

T90

-0.068

8.32E-05 5.17E-04 3364 unclassified

unclassified

unclassified

unclassified

unclassified

unclassified Firmicutes

Streptococcus oralis subsp. oralis

(HG3A.0705) T90 0.068 8.32E-05 5.17E-04 3364

Streptococcus

oralis subsp. oralis

Streptococcus parasanguinis

(HG3A.0117)

T90

0.068

8.50E-05 5.26E-04 3364 unclassified

Streptococcus

parasanguinis

Streptococcus Streptococcaceae Lactobacillales

Bacilli

Firmicutes

Streptococcus oralis Streptococcus Streptococcaceae Lactobacillales Bacilli Firmicutes

Coprococcus sp. AF21-14LB (HG3A.1047)

Clostridia sp. (HG3A.0479)

T90

-0.068

8.69E-05 5.35E-04 3364 unclassified

unclassified

unclassified

unclassified

unclassified

Clostridia

Firmicutes

AHI 0.074 6.38E-05 5.33E-04 3004 unclassified Coprococcus sp.

AF21-14LB

Coprococcus Lachnospiraceae Eubacteriales Clostridia Firmicutes

Eubacteriales sp. (HG3A.0545) AHI -0.074 6.46E-05 5.35E-04 3004 unclassified unclassified unclassified unclassified Eubacteriales Clostridia Firmicutes

Eubacteriales sp. (HG3A.0627)

AHI

-0.074

6.48E-05 5.35E-04 3004 unclassified

unclassified

unclassified

unclassified

Eubacteriales

Clostridia

Firmicutes

Blastocystis sp. subtype 4

(HG3A.0446)

AHI -0.074 6.53E-05 5.36E-04 3004 unclassified Blastocystis sp.

subtype 4

Blastocystis Blastocystidae Opalinata Bigyra unclassified

Streptococcus mutans

(HG3A.0677)

ODI

0.067

1.05E-04 5.39E-04 3364 unclassified

Streptococcus

mutans

Streptococcus Streptococcaceae Lactobacillales

Bacilli

Firmicutes

Pseudoruminococcus massiliensis

T90 -0.068 8.80E-05 5.40E-04 3364 unclassified Pseudoruminococcus Pseudoruminococcu

Oscillospiraceae Eubacteriales Clostridia Firmicutes

(HG3A.0346) massiliensis s

Bacteroides nordii (HG3A.0290)

ODI

-0.067

1.05E-04 5.42E-04 3364 unclassified Bacteroides nordii

Bacteroides

Bacteroidaceae Bacteroidales

Bacteroidia Bacteroidetes

Firmicutes sp. (HG3A.0769) ODI -0.067 1.06E-04 5.44E-04 3364 unclassified unclassified unclassified unclassified unclassified unclassified Firmicutes

Firmicutes sp. (HG3A.1075)

ODI

-0.067

1.08E-04 5.49E-04 3364 unclassified

unclassified

unclassified

unclassified

unclassified

unclassified Firmicutes

Lachnospiraceae sp. (HG3A.0252) ODI -0.067 1.08E-04 5.50E-04 3364 unclassified unclassified unclassified Lachnospiraceae Eubacteriales Clostridia Firmicutes

Eubacteriales sp. (HG3A.0668)

ODI

-0.067

1.11E-04 5.61E-04 3364 unclassified

unclassified

unclassified

unclassified

Eubacteriales

Clostridia

Firmicutes

| Eubacteriales sp. (HG3A.0568) | AHI | -0.073 | 6.88E-05 | 5.62E-04 | 3004 | unclassified | unclassified | unclassified | unclassified | Eubacteriales | Clostridia | Firmicutes |
| --- | --- | --- | --- | --- | --- | --- | --- | --- | --- | --- | --- | --- |
| Eubacteriales sp. (HG3A.0718) | T90 | -0.068 | 9.33E-05 | 5.70E-04 | 3364 | unclassified | unclassified | unclassified | unclassified | Eubacteriales | Clostridia | Firmicutes |
| Eubacteriales sp. (HG3A.0158) | AHI | -0.073 | 7.08E-05 | 5.76E-04 | 3004 | unclassified | unclassified | unclassified | unclassified | Eubacteriales | Clostridia | Firmicutes |
| Anaerotruncus colihominis (HG3A.0307) | AHI | 0.073 | 7.22E-05 | 5.84E-04 | 3004 | unclassified | Anaerotruncus colihominis | Anaerotruncus | Oscillospiraceae | Eubacteriales | Clostridia | Firmicutes |
| Bacteria sp. (HG3A.0911) | T90 | -0.068 | 9.58E-05 | 5.84E-04 | 3364 | unclassified | unclassified | unclassified | unclassified | unclassified | unclassified | unclassified |
| Scardovia wiggsiae (HG3A.1737) | T90 | 0.068 | 9.72E-05 | 5.90E-04 | 3364 | unclassified | Scardovia wiggsiae | Scardovia | Bifidobacteriaceae | Bifidobacteriales | Actinomycetia | Actinobacteria |
| Clostridia sp. (HG3A.0521) | AHI | -0.073 | 7.41E-05 | 5.96E-04 | 3004 | unclassified | unclassified | unclassified | unclassified | unclassified | Clostridia | Firmicutes |
| Clostridia sp. (HG3A.1217) | T90 | -0.068 | 9.88E-05 | 5.97E-04 | 3364 | unclassified | unclassified | unclassified | unclassified | unclassified | Clostridia | Firmicutes |
| Tyzzerella nexilis (HG3A.0574) | T90 | 0.068 | 9.97E-05 | 6.00E-04 | 3364 | unclassified | Tyzzerella nexilis | Tyzzerella | Lachnospiraceae | Eubacteriales | Clostridia | Firmicutes |
| Clostridia sp. (HG3A.0752) | T90 | -0.068 | 1.00E-04 | 6.02E-04 | 3364 | unclassified | unclassified | unclassified | unclassified | unclassified | Clostridia | Firmicutes |
| Clostridia sp. (HG3A.0368) | AHI | -0.073 | 7.61E-05 | 6.10E-04 | 3004 | unclassified | unclassified | unclassified | unclassified | unclassified | Clostridia | Firmicutes |
| Eubacteriales sp. (HG3A.0132) | T90 | -0.067 | 1.03E-04 | 6.19E-04 | 3364 | unclassified | unclassified | unclassified | unclassified | Eubacteriales | Clostridia | Firmicutes |
| Eubacteriales sp. (HG3A.0260) | ODI | -0.067 | 1.23E-04 | 6.21E-04 | 3364 | unclassified | unclassified | unclassified | unclassified | Eubacteriales | Clostridia | Firmicutes |
| Gemella morbillorum (HG3A.1782) | T90 | 0.067 | 1.04E-04 | 6.22E-04 | 3364 | unclassified | Gemella morbillorum | Gemella | unclassified | Bacillales | Bacilli | Firmicutes |
| Eubacteriales sp. (HG3A.0696) | ODI | -0.067 | 1.24E-04 | 6.24E-04 | 3364 | unclassified | unclassified | unclassified | unclassified | Eubacteriales | Clostridia | Firmicutes |
| Alistipes communis (HG3A.0064) | T90 | -0.067 | 1.05E-04 | 6.25E-04 | 3364 | unclassified | Alistipes communis | Alistipes | Rikenellaceae | Bacteroidales | Bacteroidia | Bacteroidetes |
| Ruminococcus champanellensis (HG3A.0716) | T90 | -0.067 | 1.07E-04 | 6.33E-04 | 3364 | unclassified | Ruminococcus champanellensis | Ruminococcus | Oscillospiraceae | Eubacteriales | Clostridia | Firmicutes |
| Clostridia sp. (HG3A.0931) | ODI | -0.067 | 1.26E-04 | 6.34E-04 | 3364 | unclassified | unclassified | unclassified | unclassified | unclassified | Clostridia | Firmicutes |
| Eubacteriales sp. (HG3A.0509) | T90 | -0.067 | 1.09E-04 | 6.39E-04 | 3364 | unclassified | unclassified | unclassified | unclassified | Eubacteriales | Clostridia | Firmicutes |
| Clostridia sp. (HG3A.1058) | ODI | -0.066 | 1.31E-04 | 6.54E-04 | 3364 | unclassified | unclassified | unclassified | unclassified | unclassified | Clostridia | Firmicutes |
| Firmicutes sp. (HG3A.1054) | ODI | -0.066 | 1.31E-04 | 6.54E-04 | 3364 | unclassified | unclassified | unclassified | unclassified | unclassified | unclassified | Firmicutes |

| Eubacteriales sp. (HG3A.0635) | T90 | -0.067 | 1.12E-04 | 6.55E-04 | 3364 | unclassified | unclassified | unclassified | unclassified | Eubacteriales | Clostridia | Firmicutes |
| --- | --- | --- | --- | --- | --- | --- | --- | --- | --- | --- | --- | --- |
| Eubacteriales sp. (HG3A.0545) | ODI | -0.066 | 1.34E-04 | 6.63E-04 | 3364 | unclassified | unclassified | unclassified | unclassified | Eubacteriales | Clostridia | Firmicutes |
| Eubacteriales sp. (HG3A.0758) | ODI | -0.066 | 1.34E-04 | 6.63E-04 | 3364 | unclassified | unclassified | unclassified | unclassified | Eubacteriales | Clostridia | Firmicutes |
| Actinomycetaceae sp.  (HG3A.1068) | ODI | -0.066 | 1.35E-04 | 6.65E-04 | 3364 | unclassified | unclassified | unclassified | Actinomycetaceae | Actinomycetales | Actinomycetia | Actinobacteria |
| Clostridia sp. (HG3A.0479) | ODI | -0.066 | 1.35E-04 | 6.65E-04 | 3364 | unclassified | unclassified | unclassified | unclassified | unclassified | Clostridia | Firmicutes |
| Eggerthella lenta (HG3A.0225) | AHI | 0.072 | 8.73E-05 | 6.96E-04 | 3004 | unclassified | Eggerthella lenta | Eggerthella | Eggerthellaceae | Eggerthellales | Coriobacteriia | Actinobacteria |
| Clostridia sp. (HG3A.0599) | T90 | -0.067 | 1.20E-04 | 7.03E-04 | 3364 | unclassified | unclassified | unclassified | unclassified | unclassified | Clostridia | Firmicutes |
| Eubacteriales sp. (HG3A.0578) | T90 | -0.067 | 1.21E-04 | 7.03E-04 | 3364 | unclassified | unclassified | unclassified | unclassified | Eubacteriales | Clostridia | Firmicutes |
| Firmicutes sp. (HG3A.0581) | ODI | -0.066 | 1.44E-04 | 7.07E-04 | 3364 | unclassified | unclassified | unclassified | unclassified | unclassified | unclassified | Firmicutes |
| Subdoligranulum sp. APC924/74  (HG3A.0015) | AHI | -0.072 | 8.96E-05 | 7.10E-04 | 3004 | unclassified | Subdoligranulum sp.  APC924/74 | Subdoligranulum | Oscillospiraceae | Eubacteriales | Clostridia | Firmicutes |
| Eubacteriales sp. (HG3A.0617) | ODI | -0.066 | 1.46E-04 | 7.16E-04 | 3364 | unclassified | unclassified | unclassified | unclassified | Eubacteriales | Clostridia | Firmicutes |
| Eubacteriales sp. (HG3A.0138) | ODI | -0.066 | 1.47E-04 | 7.19E-04 | 3364 | unclassified | unclassified | unclassified | unclassified | Eubacteriales | Clostridia | Firmicutes |
| Eubacteriales sp. (HG3A.0232) | T90 | -0.067 | 1.24E-04 | 7.19E-04 | 3364 | unclassified | unclassified | unclassified | unclassified | Eubacteriales | Clostridia | Firmicutes |
| Firmicutes sp. (HG3A.0641) | ODI | -0.066 | 1.48E-04 | 7.21E-04 | 3364 | unclassified | unclassified | unclassified | unclassified | unclassified | unclassified | Firmicutes |
| Oscillospiraceae sp. (HG3A.0388) | AHI | -0.072 | 9.18E-05 | 7.25E-04 | 3004 | unclassified | unclassified | unclassified | Oscillospiraceae | Eubacteriales | Clostridia | Firmicutes |
| Clostridia sp. (HG3A.1504) | T90 | -0.067 | 1.25E-04 | 7.26E-04 | 3364 | unclassified | unclassified | unclassified | unclassified | unclassified | Clostridia | Firmicutes |
| Alistipes sp. AF17-16 (HG3A.0150) | ODI | -0.066 | 1.52E-04 | 7.35E-04 | 3364 | unclassified | Alistipes sp. AF17-16 | Alistipes | Rikenellaceae | Bacteroidales | Bacteroidia | Bacteroidetes |
| Eubacteriales sp. (HG3A.0178) | ODI | -0.066 | 1.52E-04 | 7.35E-04 | 3364 | unclassified | unclassified | unclassified | unclassified | Eubacteriales | Clostridia | Firmicutes |
| Clostridia sp. (HG3A.0783) | ODI | -0.066 | 1.53E-04 | 7.40E-04 | 3364 | unclassified | unclassified | unclassified | unclassified | unclassified | Clostridia | Firmicutes |
| Eubacteriales sp. (HG3A.0267) | ODI | -0.066 | 1.55E-04 | 7.42E-04 | 3364 | unclassified | unclassified | unclassified | unclassified | Eubacteriales | Clostridia | Firmicutes |
| Eubacteriales sp. (HG3A.0514) | ODI | -0.066 | 1.55E-04 | 7.42E-04 | 3364 | unclassified | unclassified | unclassified | unclassified | Eubacteriales | Clostridia | Firmicutes |
| Eubacteriales sp. (HG3A.0910) | ODI | -0.066 | 1.56E-04 | 7.45E-04 | 3364 | unclassified | unclassified | unclassified | unclassified | Eubacteriales | Clostridia | Firmicutes |

| Eubacteriales sp. (HG3A.0244) | ODI | -0.066 | 1.57E-04 | 7.51E-04 | 3364 | unclassified | unclassified | unclassified | unclassified Eubacteriales | Clostridia | Firmicutes |
| --- | --- | --- | --- | --- | --- | --- | --- | --- | --- | --- | --- |
| Akkermansia muciniphila  (HG3A.0110) | T90 | -0.066 | 1.31E-04 | 7.53E-04 | 3364 | unclassified | Akkermansia  muciniphila | Akkermansia | Akkermansiaceae Verrucomicrobiales | Verrucomicrobi  ae | Verrucomicrobi  a |
| Erysipelotrichales sp. (HG3A.0303) | ODI | -0.066 | 1.60E-04 | 7.59E-04 | 3364 | unclassified | unclassified | unclassified | unclassified Erysipelotrichales | Erysipelotrichia | Firmicutes |
| Eubacteriales sp. (HG3A.0349) | ODI | -0.066 | 1.61E-04 | 7.63E-04 | 3364 | unclassified | unclassified | unclassified | unclassified Eubacteriales | Clostridia | Firmicutes |
| Eubacteriales sp. (HG3A.0967) | ODI | -0.066 | 1.61E-04 | 7.63E-04 | 3364 | unclassified | unclassified | unclassified | unclassified Eubacteriales | Clostridia | Firmicutes |
| Lachnospiraceae sp. (HG3A.0127) | AHI | -0.072 | 9.74E-05 | 7.65E-04 | 3004 | unclassified | unclassified | unclassified | Lachnospiraceae Eubacteriales | Clostridia | Firmicutes |
| Alloscardovia omnicolens (HG3A.1279) | AHI | 0.072 | 9.84E-05 | 7.69E-04 | 3004 | unclassified | Alloscardovia omnicolens | Alloscardovia | Bifidobacteriaceae Bifidobacteriales | Actinomycetia | Actinobacteria |
| Eubacteriales sp. (HG3A.0308) | ODI | -0.066 | 1.64E-04 | 7.75E-04 | 3364 | unclassified | unclassified | unclassified | unclassified Eubacteriales | Clostridia | Firmicutes |
| Clostridia sp. (HG3A.0746) | ODI | -0.065 | 1.67E-04 | 7.84E-04 | 3364 | unclassified | unclassified | unclassified | unclassified unclassified | Clostridia | Firmicutes |
| Firmicutes sp. (HG3A.0501) | T90 | -0.066 | 1.37E-04 | 7.87E-04 | 3364 | unclassified | unclassified | unclassified | unclassified unclassified | unclassified | Firmicutes |
| Blautia sp. AF19-10LB (HG3A.0157) | AHI | -0.071 | 1.03E-04 | 8.03E-04 | 3004 | unclassified | Blautia sp. AF19- 10LB | Blautia | Lachnospiraceae Eubacteriales | Clostridia | Firmicutes |
| Clostridia sp. (HG3A.0706) | ODI | -0.065 | 1.74E-04 | 8.15E-04 | 3364 | unclassified | unclassified | unclassified | unclassified unclassified | Clostridia | Firmicutes |
| Clostridia sp. (HG3A.0893) | AHI | -0.071 | 1.06E-04 | 8.20E-04 | 3004 | unclassified | unclassified | unclassified | unclassified unclassified | Clostridia | Firmicutes |
| Streptococcus parasanguinis (HG3A.0117) | AHI | 0.071 | 1.08E-04 | 8.34E-04 | 3004 | unclassified | Streptococcus parasanguinis | Streptococcus | Streptococcaceae Lactobacillales | Bacilli | Firmicutes |
| Eubacteriales sp. (HG3A.1294) | ODI | -0.065 | 1.83E-04 | 8.54E-04 | 3364 | unclassified | unclassified | unclassified | unclassified Eubacteriales | Clostridia | Firmicutes |
| Clostridia sp. (HG3A.0479) | AHI | -0.071 | 1.13E-04 | 8.59E-04 | 3004 | unclassified | unclassified | unclassified | unclassified unclassified | Clostridia | Firmicutes |
| Oscillospiraceae sp. (HG3A.0445) | AHI | -0.071 | 1.12E-04 | 8.59E-04 | 3004 | unclassified | unclassified | unclassified | Oscillospiraceae Eubacteriales | Clostridia | Firmicutes |
| Latilactobacillus sakei subsp. sakei (HG3A.0836) | T90 | 0.066 | 1.51E-04 | 8.65E-04 | 3364 | Latilactobacillus sakei subsp.  sakei | Latilactobacillus sakei | Latilactobacillus | Lactobacillaceae Lactobacillales | Bacilli | Firmicutes |
| Firmicutes sp. (HG3A.0923) | ODI | -0.065 | 1.87E-04 | 8.71E-04 | 3364 | unclassified | unclassified | unclassified | unclassified unclassified | unclassified | Firmicutes |
| Eggerthella lenta (HG3A.0225) | T90 | 0.066 | 1.54E-04 | 8.81E-04 | 3364 | unclassified | Eggerthella lenta | Eggerthella | Eggerthellaceae Eggerthellales | Coriobacteriia | Actinobacteria |
| Erysipelotrichales sp. (HG3A.0283) | T90 | -0.066 | 1.58E-04 | 8.99E-04 | 3364 | unclassified | unclassified | unclassified | unclassified Erysipelotrichales | Erysipelotrichia | Firmicutes |

| Oscillospiraceae sp. (HG3A.0665) | ODI | -0.065 | 1.95E-04 | 9.07E-04 | 3364 | unclassified | unclassified | unclassified | Oscillospiraceae | Eubacteriales | Clostridia | Firmicutes |
| --- | --- | --- | --- | --- | --- | --- | --- | --- | --- | --- | --- | --- |
| Clostridia sp. (HG3A.0752) | AHI | -0.071 | 1.20E-04 | 9.11E-04 | 3004 | unclassified | unclassified | unclassified | unclassified | unclassified | Clostridia | Firmicutes |
| Eubacteriales sp. (HG3A.0328) | ODI | -0.065 | 2.00E-04 | 9.26E-04 | 3364 | unclassified | unclassified | unclassified | unclassified | Eubacteriales | Clostridia | Firmicutes |
| Eubacteriales sp. (HG3A.0335) | ODI | 0.065 | 2.01E-04 | 9.26E-04 | 3364 | unclassified | unclassified | unclassified | unclassified | Eubacteriales | Clostridia | Firmicutes |
| Parasutterella excrementihominis (HG3A.0159) | T90 | -0.066 | 1.64E-04 | 9.26E-04 | 3364 | unclassified | Parasutterella excrementihominis | Parasutterella | Sutterellaceae | Burkholderiales | Betaproteobacte ria | Proteobacteria |
| Eubacteriales sp. (HG3A.0757) | AHI | -0.071 | 1.24E-04 | 9.35E-04 | 3004 | unclassified | unclassified | unclassified | unclassified | Eubacteriales | Clostridia | Firmicutes |
| Eubacteriales sp. (HG3A.0859) | T90 | -0.065 | 1.66E-04 | 9.36E-04 | 3364 | unclassified | unclassified | unclassified | unclassified | Eubacteriales | Clostridia | Firmicutes |
| Firmicutes sp. (HG3A.1054) | AHI | -0.071 | 1.25E-04 | 9.42E-04 | 3004 | unclassified | unclassified | unclassified | unclassified | unclassified | unclassified | Firmicutes |
| Eubacteriales sp. (HG3A.0711) | AHI | -0.071 | 1.27E-04 | 9.48E-04 | 3004 | unclassified | unclassified | unclassified | unclassified | Eubacteriales | Clostridia | Firmicutes |
| Eubacteriales sp. (HG3A.0565) | ODI | -0.064 | 2.07E-04 | 9.55E-04 | 3364 | unclassified | unclassified | unclassified | unclassified | Eubacteriales | Clostridia | Firmicutes |
| Blautia producta (HG3A.0905) | ODI | 0.064 | 2.08E-04 | 9.57E-04 | 3364 | unclassified | Blautia producta | Blautia | Lachnospiraceae | Eubacteriales | Clostridia | Firmicutes |
| Eubacteriales sp. (HG3A.0858) | T90 | -0.065 | 1.72E-04 | 9.68E-04 | 3364 | unclassified | unclassified | unclassified | unclassified | Eubacteriales | Clostridia | Firmicutes |
| Eubacteriales sp. (HG3A.0151) | T90 | -0.065 | 1.73E-04 | 9.72E-04 | 3364 | unclassified | unclassified | unclassified | unclassified | Eubacteriales | Clostridia | Firmicutes |
| Candidatus Borkfalkiales sp. (HG3A.1284) | ODI | -0.064 | 2.14E-04 | 9.79E-04 | 3364 | unclassified | unclassified | unclassified | unclassified | Candidatus Borkfalkiales | Clostridia | Firmicutes |
| Eubacteriales sp. (HG3A.0697) | ODI | -0.064 | 2.15E-04 | 9.81E-04 | 3364 | unclassified | unclassified | unclassified | unclassified | Eubacteriales | Clostridia | Firmicutes |
| Clostridia sp. (HG3A.0841) | ODI | -0.064 | 2.16E-04 | 9.84E-04 | 3364 | unclassified | unclassified | unclassified | unclassified | unclassified | Clostridia | Firmicutes |
| Clostridia sp. (HG3A.0767) | ODI | -0.064 | 2.18E-04 | 9.91E-04 | 3364 | unclassified | unclassified | unclassified | unclassified | unclassified | Clostridia | Firmicutes |
| Eubacteriales sp. (HG3A.1377) | ODI | -0.064 | 2.21E-04 | 9.98E-04 | 3364 | unclassified | unclassified | unclassified | unclassified | Eubacteriales | Clostridia | Firmicutes |
| Amedibacillus dolichus  (HG3A.0798) | T90 | 0.064 | 2.23E-04 | 0.001 | 3364 | unclassified | Amedibacillus  dolichus | Amedibacillus | Erysipelotrichace  ae | Erysipelotrichales | Erysipelotrichia | Firmicutes |
| Anaerobutyricum hallii (HG3A.0112) | AHI | 0.069 | 1.77E-04 | 0.001 | 3004 | unclassified | Anaerobutyricum hallii | Anaerobutyricum | Lachnospiraceae | Eubacteriales | Clostridia | Firmicutes |

Anaerotruncus massiliensis (HG3A.0460)

AHI -0.07 1.51E-04 0.001 3004 unclassified Anaerotruncus

massiliensis

Anaerotruncus Oscillospiraceae Eubacteriales Clostridia Firmicutes

| Barnesiella intestinihominis (HG3A.0055) | AHI | -0.069 | 1.66E-04 | 0.001 | 3004 | unclassified | Barnesiella intestinihominis | Barnesiella | Barnesiellaceae | Bacteroidales | Bacteroidia | Bacteroidetes |
| --- | --- | --- | --- | --- | --- | --- | --- | --- | --- | --- | --- | --- |
| Blautia hydrogenotrophica  (HG3A.0430) | AHI | 0.068 | 2.22E-04 | 0.001 | 3004 | unclassified | Blautia  hydrogenotrophica | Blautia | Lachnospiraceae | Eubacteriales | Clostridia | Firmicutes |
| Blautia sp. SG-772 (HG3A.0063) | AHI | 0.069 | 1.63E-04 | 0.001 | 3004 | unclassified | Blautia sp. SG-772 | Blautia | Lachnospiraceae | Eubacteriales | Clostridia | Firmicutes |
| Blautia sp. SG-772 (HG3A.0063) | T90 | 0.065 | 2.02E-04 | 0.001 | 3364 | unclassified | Blautia sp. SG-772 | Blautia | Lachnospiraceae | Eubacteriales | Clostridia | Firmicutes |
| Candidatus Borkfalkiales sp. (HG3A.1329) | ODI | -0.063 | 2.79E-04 | 0.001 | 3364 | unclassified | unclassified | unclassified | unclassified | Candidatus Borkfalkiales | Clostridia | Firmicutes |
| Clostridia sp. (HG3A.0733) | AHI | -0.07 | 1.59E-04 | 0.001 | 3004 | unclassified | unclassified | unclassified | unclassified | unclassified | Clostridia | Firmicutes |
| Clostridia sp. (HG3A.0852) | AHI | -0.07 | 1.50E-04 | 0.001 | 3004 | unclassified | unclassified | unclassified | unclassified | unclassified | Clostridia | Firmicutes |
| Clostridia sp. (HG3A.0852) | ODI | -0.064 | 2.26E-04 | 0.001 | 3364 | unclassified | unclassified | unclassified | unclassified | unclassified | Clostridia | Firmicutes |
| Clostridia sp. (HG3A.0933) | ODI | -0.064 | 2.51E-04 | 0.001 | 3364 | unclassified | unclassified | unclassified | unclassified | unclassified | Clostridia | Firmicutes |
| Clostridia sp. (HG3A.1008) | ODI | -0.063 | 2.78E-04 | 0.001 | 3364 | unclassified | unclassified | unclassified | unclassified | unclassified | Clostridia | Firmicutes |
| Clostridia sp. (HG3A.1062) | ODI | -0.064 | 2.35E-04 | 0.001 | 3364 | unclassified | unclassified | unclassified | unclassified | unclassified | Clostridia | Firmicutes |
| Clostridia sp. (HG3A.1141) | ODI | -0.062 | 3.38E-04 | 0.001 | 3364 | unclassified | unclassified | unclassified | unclassified | unclassified | Clostridia | Firmicutes |
| Clostridia sp. (HG3A.1148) | AHI | -0.07 | 1.59E-04 | 0.001 | 3004 | unclassified | unclassified | unclassified | unclassified | unclassified | Clostridia | Firmicutes |
| Clostridia sp. (HG3A.1356) | T90 | -0.065 | 1.90E-04 | 0.001 | 3364 | unclassified | unclassified | unclassified | unclassified | unclassified | Clostridia | Firmicutes |
| Clostridium sp. AF37-5 (HG3A.0076) | ODI | -0.063 | 2.66E-04 | 0.001 | 3364 | unclassified | Clostridium sp. AF37- 5 | Clostridium | Clostridiaceae | Eubacteriales | Clostridia | Firmicutes |
| [Clostridium] symbiosum  (HG3A.0370) | AHI | 0.07 | 1.59E-04 | 0.001 | 3004 | unclassified | [Clostridium]  symbiosum | Lachnoclostridium | Lachnospiraceae | Eubacteriales | Clostridia | Firmicutes |
| Coprococcus sp. AM27-12LB (HG3A.0687) | ODI | 0.063 | 2.98E-04 | 0.001 | 3364 | unclassified | Coprococcus sp. AM27-12LB | Coprococcus | Lachnospiraceae | Eubacteriales | Clostridia | Firmicutes |
| Coprococcus sp. (HG3A.0404) | AHI | 0.068 | 2.22E-04 | 0.001 | 3004 | unclassified | unclassified | Coprococcus | Lachnospiraceae | Eubacteriales | Clostridia | Firmicutes |
| Eggerthellaceae sp. (HG3A.0171) | AHI | -0.068 | 2.05E-04 | 0.001 | 3004 | unclassified | unclassified | unclassified | Eggerthellaceae | Eggerthellales | Coriobacteriia | Actinobacteria |
| Enterocloster aldenensis  (HG3A.0362) | ODI | 0.064 | 2.37E-04 | 0.001 | 3364 | unclassified | Enterocloster  aldenensis | Enterocloster | Lachnospiraceae | Eubacteriales | Clostridia | Firmicutes |
| Erysipelatoclostridium sp. (HG3A.0313) | ODI | -0.063 | 2.83E-04 | 0.001 | 3364 | unclassified | unclassified | Erysipelatoclostridiu m | Erysipelotrichace ae | Erysipelotrichales | Erysipelotrichia | Firmicutes |

| Eubacteriales sp. (HG3A.0081) | AHI | -0.07 | 1.44E-04 | 0.001 | 3004 | unclassified | unclassified | unclassified | unclassified | Eubacteriales | Clostridia | Firmicutes |
| --- | --- | --- | --- | --- | --- | --- | --- | --- | --- | --- | --- | --- |
| Eubacteriales sp. (HG3A.0081) | ODI | -0.062 | 3.32E-04 | 0.001 | 3364 | unclassified | unclassified | unclassified | unclassified | Eubacteriales | Clostridia | Firmicutes |
| Eubacteriales sp. (HG3A.0102) | T90 | -0.064 | 2.18E-04 | 0.001 | 3364 | unclassified | unclassified | unclassified | unclassified | Eubacteriales | Clostridia | Firmicutes |
| Eubacteriales sp. (HG3A.0128) | AHI | 0.07 | 1.59E-04 | 0.001 | 3004 | unclassified | unclassified | unclassified | unclassified | Eubacteriales | Clostridia | Firmicutes |
| Eubacteriales sp. (HG3A.0137) | AHI | -0.069 | 1.89E-04 | 0.001 | 3004 | unclassified | unclassified | unclassified | unclassified | Eubacteriales | Clostridia | Firmicutes |
| Eubacteriales sp. (HG3A.0163) | T90 | -0.063 | 2.81E-04 | 0.001 | 3364 | unclassified | unclassified | unclassified | unclassified | Eubacteriales | Clostridia | Firmicutes |
| Eubacteriales sp. (HG3A.0189) | ODI | -0.064 | 2.57E-04 | 0.001 | 3364 | unclassified | unclassified | unclassified | unclassified | Eubacteriales | Clostridia | Firmicutes |
| Eubacteriales sp. (HG3A.0234) | AHI | -0.068 | 2.04E-04 | 0.001 | 3004 | unclassified | unclassified | unclassified | unclassified | Eubacteriales | Clostridia | Firmicutes |
| Eubacteriales sp. (HG3A.0282) | ODI | -0.063 | 2.76E-04 | 0.001 | 3364 | unclassified | unclassified | unclassified | unclassified | Eubacteriales | Clostridia | Firmicutes |
| Eubacteriales sp. (HG3A.0288) | AHI | -0.07 | 1.44E-04 | 0.001 | 3004 | unclassified | unclassified | unclassified | unclassified | Eubacteriales | Clostridia | Firmicutes |
| Eubacteriales sp. (HG3A.0441) | AHI | -0.069 | 1.94E-04 | 0.001 | 3004 | unclassified | unclassified | unclassified | unclassified | Eubacteriales | Clostridia | Firmicutes |
| Eubacteriales sp. (HG3A.0450) | ODI | -0.063 | 3.17E-04 | 0.001 | 3364 | unclassified | unclassified | unclassified | unclassified | Eubacteriales | Clostridia | Firmicutes |
| Eubacteriales sp. (HG3A.0468) | ODI | -0.063 | 2.72E-04 | 0.001 | 3364 | unclassified | unclassified | unclassified | unclassified | Eubacteriales | Clostridia | Firmicutes |
| Eubacteriales sp. (HG3A.0486) | AHI | -0.069 | 1.61E-04 | 0.001 | 3004 | unclassified | unclassified | unclassified | unclassified | Eubacteriales | Clostridia | Firmicutes |
| Eubacteriales sp. (HG3A.0486) | T90 | -0.065 | 1.89E-04 | 0.001 | 3364 | unclassified | unclassified | unclassified | unclassified | Eubacteriales | Clostridia | Firmicutes |
| Eubacteriales sp. (HG3A.0498) | ODI | 0.063 | 3.21E-04 | 0.001 | 3364 | unclassified | unclassified | unclassified | unclassified | Eubacteriales | Clostridia | Firmicutes |
| Eubacteriales sp. (HG3A.0518) | T90 | -0.064 | 2.23E-04 | 0.001 | 3364 | unclassified | unclassified | unclassified | unclassified | Eubacteriales | Clostridia | Firmicutes |
| Eubacteriales sp. (HG3A.0537) | AHI | -0.07 | 1.58E-04 | 0.001 | 3004 | unclassified | unclassified | unclassified | unclassified | Eubacteriales | Clostridia | Firmicutes |
| Eubacteriales sp. (HG3A.0540) | AHI | -0.07 | 1.43E-04 | 0.001 | 3004 | unclassified | unclassified | unclassified | unclassified | Eubacteriales | Clostridia | Firmicutes |
| Eubacteriales sp. (HG3A.0613) | AHI | -0.069 | 1.67E-04 | 0.001 | 3004 | unclassified | unclassified | unclassified | unclassified | Eubacteriales | Clostridia | Firmicutes |
| Eubacteriales sp. (HG3A.0626) | AHI | -0.07 | 1.43E-04 | 0.001 | 3004 | unclassified | unclassified | unclassified | unclassified | Eubacteriales | Clostridia | Firmicutes |
| Eubacteriales sp. (HG3A.0628) | ODI | -0.063 | 2.86E-04 | 0.001 | 3364 | unclassified | unclassified | unclassified | unclassified | Eubacteriales | Clostridia | Firmicutes |

| Eubacteriales sp. (HG3A.0656) | T90 | -0.065 | 1.95E-04 | 0.001 | 3364 | unclassified | unclassified | unclassified | unclassified | Eubacteriales | Clostridia | Firmicutes |
| --- | --- | --- | --- | --- | --- | --- | --- | --- | --- | --- | --- | --- |
| Eubacteriales sp. (HG3A.0697) | T90 | -0.065 | 1.91E-04 | 0.001 | 3364 | unclassified | unclassified | unclassified | unclassified | Eubacteriales | Clostridia | Firmicutes |
| Eubacteriales sp. (HG3A.0698) | ODI | 0.064 | 2.42E-04 | 0.001 | 3364 | unclassified | unclassified | unclassified | unclassified | Eubacteriales | Clostridia | Firmicutes |
| Eubacteriales sp. (HG3A.0760) | T90 | -0.064 | 2.09E-04 | 0.001 | 3364 | unclassified | unclassified | unclassified | unclassified | Eubacteriales | Clostridia | Firmicutes |
| Eubacteriales sp. (HG3A.0846) | T90 | -0.063 | 2.78E-04 | 0.001 | 3364 | unclassified | unclassified | unclassified | unclassified | Eubacteriales | Clostridia | Firmicutes |
| Eubacteriales sp. (HG3A.0976) | ODI | -0.064 | 2.29E-04 | 0.001 | 3364 | unclassified | unclassified | unclassified | unclassified | Eubacteriales | Clostridia | Firmicutes |
| Eubacteriales sp. (HG3A.0977) | T90 | -0.065 | 2.05E-04 | 0.001 | 3364 | unclassified | unclassified | unclassified | unclassified | Eubacteriales | Clostridia | Firmicutes |
| Eubacteriales sp. (HG3A.1167) | T90 | -0.065 | 1.79E-04 | 0.001 | 3364 | unclassified | unclassified | unclassified | unclassified | Eubacteriales | Clostridia | Firmicutes |
| Eubacteriales sp. (HG3A.1167) | ODI | -0.063 | 2.68E-04 | 0.001 | 3364 | unclassified | unclassified | unclassified | unclassified | Eubacteriales | Clostridia | Firmicutes |
| Firmicutes sp. (HG3A.1124) | ODI | -0.063 | 2.73E-04 | 0.001 | 3364 | unclassified | unclassified | unclassified | unclassified | unclassified | unclassified | Firmicutes |
| Flavonifractor sp. An10  (HG3A.0495) | ODI | -0.063 | 2.77E-04 | 0.001 | 3364 | unclassified | Flavonifractor sp.  An10 | Flavonifractor | Oscillospiraceae | Eubacteriales | Clostridia | Firmicutes |
| Lachnospiraceae sp. (HG3A.0236) | T90 | -0.065 | 2.04E-04 | 0.001 | 3364 | unclassified | unclassified | unclassified | Lachnospiraceae | Eubacteriales | Clostridia | Firmicutes |
| Limosilactobacillus fermentum  (HG3A.0990) | ODI | 0.064 | 2.23E-04 | 0.001 | 3364 | unclassified | Limosilactobacillus  fermentum | Limosilactobacillus | Lactobacillaceae | Lactobacillales | Bacilli | Firmicutes |
| Oscillibacter sp. PEA192 (HG3A.0021) | T90 | 0.064 | 2.37E-04 | 0.001 | 3364 | unclassified | Oscillibacter sp.  PEA192 | Oscillibacter | Oscillospiraceae | Eubacteriales | Clostridia | Firmicutes |
| Oscillibacter sp. (HG3A.0734) | AHI | -0.068 | 1.99E-04 | 0.001 | 3004 | unclassified | unclassified | Oscillibacter | Oscillospiraceae | Eubacteriales | Clostridia | Firmicutes |
| Oscillospiraceae sp. (HG3A.0612) | ODI | -0.064 | 2.50E-04 | 0.001 | 3364 | unclassified | unclassified | unclassified | Oscillospiraceae | Eubacteriales | Clostridia | Firmicutes |
| Parasutterella excrementihominis (HG3A.0159) | AHI | -0.07 | 1.49E-04 | 0.001 | 3004 | unclassified | Parasutterella excrementihominis | Parasutterella | Sutterellaceae | Burkholderiales | Betaproteobacte ria | Proteobacteria |
| Rikenellaceae sp. (HG3A.1022) | AHI | -0.07 | 1.48E-04 | 0.001 | 3004 | unclassified | unclassified | unclassified | Rikenellaceae | Bacteroidales | Bacteroidia | Bacteroidetes |
| Ruminococcus champanellensis (HG3A.0716) | AHI | -0.069 | 1.82E-04 | 0.001 | 3004 | unclassified | Ruminococcus champanellensis | Ruminococcus | Oscillospiraceae | Eubacteriales | Clostridia | Firmicutes |
| Alistipes dispar (HG3A.0281) | ODI | -0.061 | 4.39E-04 | 0.002 | 3364 | unclassified | Alistipes dispar | Alistipes | Rikenellaceae | Bacteroidales | Bacteroidia | Bacteroidetes |

Anaerobutyricum hallii (HG3A.0012)

AHI 0.066 3.49E-04 0.002 3004 unclassified Anaerobutyricum

hallii

Anaerobutyricum Lachnospiraceae Eubacteriales Clostridia Firmicutes

| Bacteria sp. (HG3A.0218) | AHI | -0.065 | 3.78E-04 | 0.002 | 3004 | unclassified | unclassified | unclassified | unclassified | unclassified | unclassified | unclassified |
| --- | --- | --- | --- | --- | --- | --- | --- | --- | --- | --- | --- | --- |
| Bacteria sp. (HG3A.0459) | ODI | -0.06 | 5.31E-04 | 0.002 | 3364 | unclassified | unclassified | unclassified | unclassified | unclassified | unclassified | unclassified |
| Barnesiella intestinihominis (HG3A.0055) | T90 | -0.061 | 4.49E-04 | 0.002 | 3364 | unclassified | Barnesiella intestinihominis | Barnesiella | Barnesiellaceae | Bacteroidales | Bacteroidia | Bacteroidetes |
| Blautia producta (HG3A.0619) | T90 | 0.06 | 5.21E-04 | 0.002 | 3364 | unclassified | Blautia producta | Blautia | Lachnospiraceae | Eubacteriales | Clostridia | Firmicutes |
| Blautia producta (HG3A.0619) | ODI | 0.062 | 3.60E-04 | 0.002 | 3364 | unclassified | Blautia producta | Blautia | Lachnospiraceae | Eubacteriales | Clostridia | Firmicutes |
| Blautia sp. AF19-10LB  (HG3A.0157) | ODI | -0.06 | 5.53E-04 | 0.002 | 3364 | unclassified | Blautia sp. AF19-  10LB | Blautia | Lachnospiraceae | Eubacteriales | Clostridia | Firmicutes |
| Butyricimonas virosa (HG3A.0199) | AHI | -0.067 | 2.94E-04 | 0.002 | 3004 | unclassified | Butyricimonas virosa | Butyricimonas | Odoribacteraceae | Bacteroidales | Bacteroidia | Bacteroidetes |
| Butyrivibrio crossotus  (HG3A.0413) | T90 | -0.063 | 2.92E-04 | 0.002 | 3364 | unclassified | Butyrivibrio  crossotus | Butyrivibrio | Lachnospiraceae | Eubacteriales | Clostridia | Firmicutes |
| Clostridia sp. (HG3A.0879) | AHI | -0.067 | 2.91E-04 | 0.002 | 3004 | unclassified | unclassified | unclassified | unclassified | unclassified | Clostridia | Firmicutes |
| Clostridia sp. (HG3A.0885) | ODI | -0.06 | 5.56E-04 | 0.002 | 3364 | unclassified | unclassified | unclassified | unclassified | unclassified | Clostridia | Firmicutes |
| Clostridia sp. (HG3A.1039) | AHI | -0.065 | 3.95E-04 | 0.002 | 3004 | unclassified | unclassified | unclassified | unclassified | unclassified | Clostridia | Firmicutes |
| Clostridia sp. (HG3A.1057) | AHI | -0.066 | 3.15E-04 | 0.002 | 3004 | unclassified | unclassified | unclassified | unclassified | unclassified | Clostridia | Firmicutes |
| Clostridia sp. (HG3A.1205) | T90 | -0.062 | 3.70E-04 | 0.002 | 3364 | unclassified | unclassified | unclassified | unclassified | unclassified | Clostridia | Firmicutes |
| Clostridiaceae sp. (HG3A.0330) | T90 | -0.062 | 3.43E-04 | 0.002 | 3364 | unclassified | unclassified | unclassified | Clostridiaceae | Eubacteriales | Clostridia | Firmicutes |
| Clostridium sp. AF15-31 (HG3A.0293) | T90 | -0.062 | 3.37E-04 | 0.002 | 3364 | unclassified | Clostridium sp. AF15- 31 | Clostridium | Clostridiaceae | Eubacteriales | Clostridia | Firmicutes |
| Coprobacillus sp. (HG3A.0022) | ODI | 0.061 | 4.15E-04 | 0.002 | 3364 | unclassified | unclassified | Coprobacillus | Coprobacillaceae | Erysipelotrichales | Erysipelotrichia | Firmicutes |
| Coprococcus sp. AF21-14LB (HG3A.1047) | T90 | 0.062 | 3.41E-04 | 0.002 | 3364 | unclassified | Coprococcus sp. AF21-14LB | Coprococcus | Lachnospiraceae | Eubacteriales | Clostridia | Firmicutes |
| Coprococcus sp. AF21-14LB  (HG3A.1047) | ODI | 0.061 | 4.44E-04 | 0.002 | 3364 | unclassified | Coprococcus sp.  AF21-14LB | Coprococcus | Lachnospiraceae | Eubacteriales | Clostridia | Firmicutes |
| Desulfovibrionales sp. (HG3A.0266) | ODI | -0.061 | 4.64E-04 | 0.002 | 3364 | unclassified | unclassified | unclassified | unclassified | Desulfovibrionales | Deltaproteobact eria | Proteobacteria |
| Dorea sp. AF24-7LB (HG3A.0086) | T90 | 0.061 | 4.14E-04 | 0.002 | 3364 | unclassified | Dorea sp. AF24-7LB | Dorea | Lachnospiraceae | Eubacteriales | Clostridia | Firmicutes |
| Eggerthellales sp. (HG3A.0848) | ODI | -0.061 | 4.85E-04 | 0.002 | 3364 | unclassified | unclassified | unclassified | unclassified | Eggerthellales | Coriobacteriia | Actinobacteria |
| Eubacteriales sp. (HG3A.0136) | AHI | -0.067 | 2.73E-04 | 0.002 | 3004 | unclassified | unclassified | unclassified | unclassified | Eubacteriales | Clostridia | Firmicutes |

| Eubacteriales sp. (HG3A.0158) | T90 | -0.062 | 3.34E-04 | 0.002 | 3364 | unclassified | unclassified | unclassified | unclassified | Eubacteriales | Clostridia | Firmicutes |
| --- | --- | --- | --- | --- | --- | --- | --- | --- | --- | --- | --- | --- |
| Eubacteriales sp. (HG3A.0213) | ODI | -0.062 | 3.71E-04 | 0.002 | 3364 | unclassified | unclassified | unclassified | unclassified | Eubacteriales | Clostridia | Firmicutes |
| Eubacteriales sp. (HG3A.0263) | T90 | -0.061 | 4.06E-04 | 0.002 | 3364 | unclassified | unclassified | unclassified | unclassified | Eubacteriales | Clostridia | Firmicutes |
| Eubacteriales sp. (HG3A.0322) | AHI | -0.067 | 2.60E-04 | 0.002 | 3004 | unclassified | unclassified | unclassified | unclassified | Eubacteriales | Clostridia | Firmicutes |
| Eubacteriales sp. (HG3A.0323) | T90 | -0.061 | 4.90E-04 | 0.002 | 3364 | unclassified | unclassified | unclassified | unclassified | Eubacteriales | Clostridia | Firmicutes |
| Eubacteriales sp. (HG3A.0323) | ODI | -0.06 | 5.97E-04 | 0.002 | 3364 | unclassified | unclassified | unclassified | unclassified | Eubacteriales | Clostridia | Firmicutes |
| Eubacteriales sp. (HG3A.0371) | AHI | -0.067 | 2.49E-04 | 0.002 | 3004 | unclassified | unclassified | unclassified | unclassified | Eubacteriales | Clostridia | Firmicutes |
| Eubacteriales sp. (HG3A.0377) | T90 | -0.063 | 2.86E-04 | 0.002 | 3364 | unclassified | unclassified | unclassified | unclassified | Eubacteriales | Clostridia | Firmicutes |
| Eubacteriales sp. (HG3A.0406) | T90 | -0.062 | 3.54E-04 | 0.002 | 3364 | unclassified | unclassified | unclassified | unclassified | Eubacteriales | Clostridia | Firmicutes |
| Eubacteriales sp. (HG3A.0406) | ODI | -0.061 | 4.90E-04 | 0.002 | 3364 | unclassified | unclassified | unclassified | unclassified | Eubacteriales | Clostridia | Firmicutes |
| Eubacteriales sp. (HG3A.0441) | T90 | -0.062 | 4.02E-04 | 0.002 | 3364 | unclassified | unclassified | unclassified | unclassified | Eubacteriales | Clostridia | Firmicutes |
| Eubacteriales sp. (HG3A.0493) | T90 | -0.063 | 3.07E-04 | 0.002 | 3364 | unclassified | unclassified | unclassified | unclassified | Eubacteriales | Clostridia | Firmicutes |
| Eubacteriales sp. (HG3A.0505) | AHI | -0.066 | 3.26E-04 | 0.002 | 3004 | unclassified | unclassified | unclassified | unclassified | Eubacteriales | Clostridia | Firmicutes |
| Eubacteriales sp. (HG3A.0509) | ODI | -0.06 | 6.19E-04 | 0.002 | 3364 | unclassified | unclassified | unclassified | unclassified | Eubacteriales | Clostridia | Firmicutes |
| Eubacteriales sp. (HG3A.0518) | AHI | -0.066 | 3.13E-04 | 0.002 | 3004 | unclassified | unclassified | unclassified | unclassified | Eubacteriales | Clostridia | Firmicutes |
| Eubacteriales sp. (HG3A.0528) | ODI | -0.062 | 3.62E-04 | 0.002 | 3364 | unclassified | unclassified | unclassified | unclassified | Eubacteriales | Clostridia | Firmicutes |
| Eubacteriales sp. (HG3A.0540) | ODI | -0.062 | 3.84E-04 | 0.002 | 3364 | unclassified | unclassified | unclassified | unclassified | Eubacteriales | Clostridia | Firmicutes |
| Eubacteriales sp. (HG3A.0548) | T90 | -0.063 | 3.05E-04 | 0.002 | 3364 | unclassified | unclassified | unclassified | unclassified | Eubacteriales | Clostridia | Firmicutes |
| Eubacteriales sp. (HG3A.0573) | ODI | -0.062 | 3.60E-04 | 0.002 | 3364 | unclassified | unclassified | unclassified | unclassified | Eubacteriales | Clostridia | Firmicutes |
| Eubacteriales sp. (HG3A.0594) | AHI | -0.066 | 3.21E-04 | 0.002 | 3004 | unclassified | unclassified | unclassified | unclassified | Eubacteriales | Clostridia | Firmicutes |
| Eubacteriales sp. (HG3A.0604) | T90 | -0.061 | 4.45E-04 | 0.002 | 3364 | unclassified | unclassified | unclassified | unclassified | Eubacteriales | Clostridia | Firmicutes |
| Eubacteriales sp. (HG3A.0656) | ODI | -0.061 | 4.83E-04 | 0.002 | 3364 | unclassified | unclassified | unclassified | unclassified | Eubacteriales | Clostridia | Firmicutes |

| Eubacteriales sp. (HG3A.0668) | T90 | -0.061 | 4.09E-04 | 0.002 | 3364 | unclassified | unclassified | unclassified | unclassified | Eubacteriales | Clostridia | Firmicutes |
| --- | --- | --- | --- | --- | --- | --- | --- | --- | --- | --- | --- | --- |
| Eubacteriales sp. (HG3A.0691) | ODI | -0.06 | 5.60E-04 | 0.002 | 3364 | unclassified | unclassified | unclassified | unclassified | Eubacteriales | Clostridia | Firmicutes |
| Eubacteriales sp. (HG3A.0692) | T90 | 0.062 | 3.70E-04 | 0.002 | 3364 | unclassified | unclassified | unclassified | unclassified | Eubacteriales | Clostridia | Firmicutes |
| Eubacteriales sp. (HG3A.0696) | AHI | -0.066 | 3.67E-04 | 0.002 | 3004 | unclassified | unclassified | unclassified | unclassified | Eubacteriales | Clostridia | Firmicutes |
| Eubacteriales sp. (HG3A.0701) | ODI | -0.062 | 3.91E-04 | 0.002 | 3364 | unclassified | unclassified | unclassified | unclassified | Eubacteriales | Clostridia | Firmicutes |
| Eubacteriales sp. (HG3A.0731) | AHI | -0.066 | 3.16E-04 | 0.002 | 3004 | unclassified | unclassified | unclassified | unclassified | Eubacteriales | Clostridia | Firmicutes |
| Eubacteriales sp. (HG3A.0731) | T90 | -0.063 | 3.02E-04 | 0.002 | 3364 | unclassified | unclassified | unclassified | unclassified | Eubacteriales | Clostridia | Firmicutes |
| Eubacteriales sp. (HG3A.0744) | T90 | -0.063 | 3.03E-04 | 0.002 | 3364 | unclassified | unclassified | unclassified | unclassified | Eubacteriales | Clostridia | Firmicutes |
| Eubacteriales sp. (HG3A.0744) | ODI | -0.059 | 6.40E-04 | 0.002 | 3364 | unclassified | unclassified | unclassified | unclassified | Eubacteriales | Clostridia | Firmicutes |
| Eubacteriales sp. (HG3A.0781) | ODI | -0.061 | 4.52E-04 | 0.002 | 3364 | unclassified | unclassified | unclassified | unclassified | Eubacteriales | Clostridia | Firmicutes |
| Eubacteriales sp. (HG3A.0794) | ODI | -0.062 | 3.79E-04 | 0.002 | 3364 | unclassified | unclassified | unclassified | unclassified | Eubacteriales | Clostridia | Firmicutes |
| Eubacteriales sp. (HG3A.0829) | T90 | -0.061 | 4.94E-04 | 0.002 | 3364 | unclassified | unclassified | unclassified | unclassified | Eubacteriales | Clostridia | Firmicutes |
| Eubacteriales sp. (HG3A.0857) | AHI | -0.066 | 3.70E-04 | 0.002 | 3004 | unclassified | unclassified | unclassified | unclassified | Eubacteriales | Clostridia | Firmicutes |
| Eubacteriales sp. (HG3A.0887) | ODI | -0.061 | 4.06E-04 | 0.002 | 3364 | unclassified | unclassified | unclassified | unclassified | Eubacteriales | Clostridia | Firmicutes |
| Eubacteriales sp. (HG3A.0910) | T90 | -0.061 | 4.11E-04 | 0.002 | 3364 | unclassified | unclassified | unclassified | unclassified | Eubacteriales | Clostridia | Firmicutes |
| Eubacteriales sp. (HG3A.0976) | T90 | -0.062 | 3.94E-04 | 0.002 | 3364 | unclassified | unclassified | unclassified | unclassified | Eubacteriales | Clostridia | Firmicutes |
| Eubacteriales sp. (HG3A.1026) | ODI | -0.061 | 4.25E-04 | 0.002 | 3364 | unclassified | unclassified | unclassified | unclassified | Eubacteriales | Clostridia | Firmicutes |
| Eubacteriales sp. (HG3A.1051) | T90 | -0.063 | 3.12E-04 | 0.002 | 3364 | unclassified | unclassified | unclassified | unclassified | Eubacteriales | Clostridia | Firmicutes |
| Eubacteriales sp. (HG3A.1377) | AHI | -0.068 | 2.28E-04 | 0.002 | 3004 | unclassified | unclassified | unclassified | unclassified | Eubacteriales | Clostridia | Firmicutes |
| Eubacteriales sp. (HG3A.1422) | T90 | -0.061 | 4.04E-04 | 0.002 | 3364 | unclassified | unclassified | unclassified | unclassified | Eubacteriales | Clostridia | Firmicutes |
| Eubacterium sp. (HG3A.0214) | AHI | 0.066 | 3.39E-04 | 0.002 | 3004 | unclassified | unclassified | Eubacterium | Eubacteriaceae | Eubacteriales | Clostridia | Firmicutes |
| Evtepia gabavorous (HG3A.0114) | T90 | 0.061 | 4.29E-04 | 0.002 | 3364 | unclassified | Evtepia gabavorous | Evtepia | unclassified | Eubacteriales | Clostridia | Firmicutes |

| Faecalibacterium prausnitzii  (HG3A.0025) | ODI | 0.06 | 6.03E-04 | 0.002 | 3364 | unclassified | Faecalibacterium  prausnitzii | Faecalibacterium | Oscillospiraceae | Eubacteriales | Clostridia | Firmicutes |
| --- | --- | --- | --- | --- | --- | --- | --- | --- | --- | --- | --- | --- |
| Firmicutes sp. (HG3A.0436) | AHI | -0.067 | 2.62E-04 | 0.002 | 3004 | unclassified | unclassified | unclassified | unclassified | unclassified | unclassified | Firmicutes |
| Firmicutes sp. (HG3A.0874) | T90 | -0.06 | 5.23E-04 | 0.002 | 3364 | unclassified | unclassified | unclassified | unclassified | unclassified | unclassified | Firmicutes |
| Firmicutes sp. (HG3A.0874) | ODI | -0.062 | 3.72E-04 | 0.002 | 3364 | unclassified | unclassified | unclassified | unclassified | unclassified | unclassified | Firmicutes |
| Firmicutes sp. (HG3A.1085) | T90 | -0.061 | 4.39E-04 | 0.002 | 3364 | unclassified | unclassified | unclassified | unclassified | unclassified | unclassified | Firmicutes |
| Firmicutes sp. (HG3A.1471) | T90 | -0.06 | 5.03E-04 | 0.002 | 3364 | unclassified | unclassified | unclassified | unclassified | unclassified | unclassified | Firmicutes |
| Fusobacterium nucleatum subsp. animalis (HG3A.1418) | T90 | 0.06 | 5.22E-04 | 0.002 | 3364 | Fusobacterium nucleatum subsp. animalis | Fusobacterium nucleatum | Fusobacterium | Fusobacteriaceae | Fusobacteriales | Fusobacteriia | Fusobacteria |
| Gemmiger formicilis (HG3A.0027) | ODI | 0.062 | 3.81E-04 | 0.002 | 3364 | unclassified | Gemmiger formicilis | Gemmiger | unclassified | Eubacteriales | Clostridia | Firmicutes |
| Haemophilus parainfluenzae (HG3A.0181) | AHI | -0.067 | 2.50E-04 | 0.002 | 3004 | unclassified | Haemophilus parainfluenzae | Haemophilus | Pasteurellaceae | Pasteurellales | Gammaproteob acteria | Proteobacteria |
| Holdemanella sp. (HG3A.0366) | ODI | 0.06 | 5.78E-04 | 0.002 | 3364 | unclassified | unclassified | Holdemanella | Erysipelotrichace  ae | Erysipelotrichales | Erysipelotrichia | Firmicutes |
| Lachnospiraceae sp. (HG3A.0748) | T90 | -0.063 | 3.22E-04 | 0.002 | 3364 | unclassified | unclassified | unclassified | Lachnospiraceae | Eubacteriales | Clostridia | Firmicutes |
| Lachnospiraceae sp. (HG3A.0855) | ODI | -0.061 | 4.13E-04 | 0.002 | 3364 | unclassified | unclassified | unclassified | Lachnospiraceae | Eubacteriales | Clostridia | Firmicutes |
| Latilactobacillus sakei subsp. sakei (HG3A.0836) | ODI | 0.061 | 4.22E-04 | 0.002 | 3364 | Latilactobacillus sakei subsp. sakei | Latilactobacillus sakei | Latilactobacillus | Lactobacillaceae | Lactobacillales | Bacilli | Firmicutes |
| Limosilactobacillus vaginalis  (HG3A.1341) | T90 | 0.063 | 2.91E-04 | 0.002 | 3364 | unclassified | Limosilactobacillus  vaginalis | Limosilactobacillus | Lactobacillaceae | Lactobacillales | Bacilli | Firmicutes |
| Oscillibacter sp. PEA192 (HG3A.0021) | ODI | 0.059 | 6.31E-04 | 0.002 | 3364 | unclassified | Oscillibacter sp.  PEA192 | Oscillibacter | Oscillospiraceae | Eubacteriales | Clostridia | Firmicutes |
| Oscillospiraceae sp. (HG3A.0134) | AHI | -0.066 | 3.55E-04 | 0.002 | 3004 | unclassified | unclassified | unclassified | Oscillospiraceae | Eubacteriales | Clostridia | Firmicutes |
| Oscillospiraceae sp. (HG3A.0475) | AHI | -0.066 | 3.35E-04 | 0.002 | 3004 | unclassified | unclassified | unclassified | Oscillospiraceae | Eubacteriales | Clostridia | Firmicutes |
| Oscillospiraceae sp. (HG3A.0507) | T90 | -0.06 | 5.19E-04 | 0.002 | 3364 | unclassified | unclassified | unclassified | Oscillospiraceae | Eubacteriales | Clostridia | Firmicutes |
| Oscillospiraceae sp. (HG3A.0576) | T90 | -0.061 | 4.67E-04 | 0.002 | 3364 | unclassified | unclassified | unclassified | Oscillospiraceae | Eubacteriales | Clostridia | Firmicutes |
| Oscillospiraceae sp. (HG3A.0849) | ODI | -0.061 | 4.62E-04 | 0.002 | 3364 | unclassified | unclassified | unclassified | Oscillospiraceae | Eubacteriales | Clostridia | Firmicutes |

| Oscillospiraceae sp. (HG3A.1173) | T90 | -0.062 | 3.66E-04 | 0.002 | 3364 | unclassified | unclassified | unclassified | Oscillospiraceae | Eubacteriales | Clostridia | Firmicutes |
| --- | --- | --- | --- | --- | --- | --- | --- | --- | --- | --- | --- | --- |
| Streptococcus oralis subsp. oralis (HG3A.0705) | AHI | 0.067 | 2.99E-04 | 0.002 | 3004 | Streptococcus oralis subsp.  oralis | Streptococcus oralis | Streptococcus | Streptococcaceae | Lactobacillales | Bacilli | Firmicutes |
| Traorella massiliensis (HG3A.0669) | ODI | -0.062 | 3.70E-04 | 0.002 | 3364 | unclassified | Traorella massiliensis | Traorella | Erysipelotrichace ae | Erysipelotrichales | Erysipelotrichia | Firmicutes |
| Victivallis lenta (HG3A.0525) | AHI | -0.067 | 3.01E-04 | 0.002 | 3004 | unclassified | Victivallis lenta | Victivallis | Victivallaceae | Victivallales | Lentisphaeria | Lentisphaerae |
| Alistipes provencensis (HG3A.0877) | ODI | -0.059 | 6.52E-04 | 0.003 | 3364 | unclassified | Alistipes provencensis | Alistipes | Rikenellaceae | Bacteroidales | Bacteroidia | Bacteroidetes |
| Bacteroidales sp. (HG3A.1236) | T90 | -0.059 | 7.63E-04 | 0.003 | 3364 | unclassified | unclassified | unclassified | unclassified | Bacteroidales | Bacteroidia | Bacteroidetes |
| Butyrivibrio crossotus (HG3A.0413) | ODI | -0.058 | 7.84E-04 | 0.003 | 3364 | unclassified | Butyrivibrio crossotus | Butyrivibrio | Lachnospiraceae | Eubacteriales | Clostridia | Firmicutes |
| Clostridia sp. (HG3A.0011) | T90 | 0.06 | 5.34E-04 | 0.003 | 3364 | unclassified | unclassified | unclassified | unclassified | unclassified | Clostridia | Firmicutes |
| Clostridia sp. (HG3A.0828) | T90 | -0.06 | 5.92E-04 | 0.003 | 3364 | unclassified | unclassified | unclassified | unclassified | unclassified | Clostridia | Firmicutes |
| Clostridia sp. (HG3A.0929) | ODI | -0.058 | 9.06E-04 | 0.003 | 3364 | unclassified | unclassified | unclassified | unclassified | unclassified | Clostridia | Firmicutes |
| Clostridia sp. (HG3A.0946) | ODI | -0.059 | 6.90E-04 | 0.003 | 3364 | unclassified | unclassified | unclassified | unclassified | unclassified | Clostridia | Firmicutes |
| Clostridia sp. (HG3A.1010) | T90 | -0.059 | 6.43E-04 | 0.003 | 3364 | unclassified | unclassified | unclassified | unclassified | unclassified | Clostridia | Firmicutes |
| Clostridia sp. (HG3A.1139) | ODI | -0.058 | 8.91E-04 | 0.003 | 3364 | unclassified | unclassified | unclassified | unclassified | unclassified | Clostridia | Firmicutes |
| Clostridium sp. AF34-13  (HG3A.0173) | T90 | -0.06 | 5.47E-04 | 0.003 | 3364 | unclassified | Clostridium sp. AF34-  13 | Clostridium | Clostridiaceae | Eubacteriales | Clostridia | Firmicutes |
| [Clostridium] symbiosum (HG3A.0370) | T90 | 0.06 | 5.31E-04 | 0.003 | 3364 | unclassified | [Clostridium] symbiosum | Lachnoclostridium | Lachnospiraceae | Eubacteriales | Clostridia | Firmicutes |
| Enterocloster clostridioformis (HG3A.0686) | T90 | 0.059 | 7.25E-04 | 0.003 | 3364 | unclassified | Enterocloster clostridioformis | Enterocloster | Lachnospiraceae | Eubacteriales | Clostridia | Firmicutes |
| Erysipelotrichales sp. (HG3A.0283) | AHI | -0.065 | 4.21E-04 | 0.003 | 3004 | unclassified | unclassified | unclassified | unclassified | Erysipelotrichales | Erysipelotrichia | Firmicutes |
| Erysipelotrichales sp.  (HG3A.0283) | ODI | -0.059 | 7.39E-04 | 0.003 | 3364 | unclassified | unclassified | unclassified | unclassified | Erysipelotrichales | Erysipelotrichia | Firmicutes |
| Erysipelotrichales sp. (HG3A.1207) | ODI | 0.058 | 8.52E-04 | 0.003 | 3364 | unclassified | unclassified | unclassified | unclassified | Erysipelotrichales | Erysipelotrichia | Firmicutes |
| Eubacteriales sp. (HG3A.0093) | T90 | -0.059 | 6.39E-04 | 0.003 | 3364 | unclassified | unclassified | unclassified | unclassified | Eubacteriales | Clostridia | Firmicutes |
| Eubacteriales sp. (HG3A.0192) | ODI | -0.059 | 6.78E-04 | 0.003 | 3364 | unclassified | unclassified | unclassified | unclassified | Eubacteriales | Clostridia | Firmicutes |

| Eubacteriales sp. (HG3A.0244) | AHI | -0.063 | 5.87E-04 | 0.003 | 3004 | unclassified | unclassified | unclassified | unclassified | Eubacteriales | Clostridia | Firmicutes |
| --- | --- | --- | --- | --- | --- | --- | --- | --- | --- | --- | --- | --- |
| Eubacteriales sp. (HG3A.0289) | T90 | -0.06 | 5.29E-04 | 0.003 | 3364 | unclassified | unclassified | unclassified | unclassified | Eubacteriales | Clostridia | Firmicutes |
| Eubacteriales sp. (HG3A.0334) | ODI | -0.059 | 7.03E-04 | 0.003 | 3364 | unclassified | unclassified | unclassified | unclassified | Eubacteriales | Clostridia | Firmicutes |
| Eubacteriales sp. (HG3A.0335) | T90 | 0.06 | 5.75E-04 | 0.003 | 3364 | unclassified | unclassified | unclassified | unclassified | Eubacteriales | Clostridia | Firmicutes |
| Eubacteriales sp. (HG3A.0350) | ODI | -0.059 | 6.59E-04 | 0.003 | 3364 | unclassified | unclassified | unclassified | unclassified | Eubacteriales | Clostridia | Firmicutes |
| Eubacteriales sp. (HG3A.0367) | T90 | -0.059 | 7.09E-04 | 0.003 | 3364 | unclassified | unclassified | unclassified | unclassified | Eubacteriales | Clostridia | Firmicutes |
| Eubacteriales sp. (HG3A.0376) | AHI | -0.065 | 4.15E-04 | 0.003 | 3004 | unclassified | unclassified | unclassified | unclassified | Eubacteriales | Clostridia | Firmicutes |
| Eubacteriales sp. (HG3A.0472) | T90 | -0.059 | 7.31E-04 | 0.003 | 3364 | unclassified | unclassified | unclassified | unclassified | Eubacteriales | Clostridia | Firmicutes |
| Eubacteriales sp. (HG3A.0493) | ODI | -0.059 | 6.68E-04 | 0.003 | 3364 | unclassified | unclassified | unclassified | unclassified | Eubacteriales | Clostridia | Firmicutes |
| Eubacteriales sp. (HG3A.0516) | AHI | -0.065 | 4.22E-04 | 0.003 | 3004 | unclassified | unclassified | unclassified | unclassified | Eubacteriales | Clostridia | Firmicutes |
| Eubacteriales sp. (HG3A.0580) | T90 | -0.059 | 6.81E-04 | 0.003 | 3364 | unclassified | unclassified | unclassified | unclassified | Eubacteriales | Clostridia | Firmicutes |
| Eubacteriales sp. (HG3A.0592) | ODI | -0.058 | 8.18E-04 | 0.003 | 3364 | unclassified | unclassified | unclassified | unclassified | Eubacteriales | Clostridia | Firmicutes |
| Eubacteriales sp. (HG3A.0609) | AHI | -0.064 | 4.72E-04 | 0.003 | 3004 | unclassified | unclassified | unclassified | unclassified | Eubacteriales | Clostridia | Firmicutes |
| Eubacteriales sp. (HG3A.0633) | T90 | -0.06 | 5.53E-04 | 0.003 | 3364 | unclassified | unclassified | unclassified | unclassified | Eubacteriales | Clostridia | Firmicutes |
| Eubacteriales sp. (HG3A.0633) | ODI | -0.058 | 9.27E-04 | 0.003 | 3364 | unclassified | unclassified | unclassified | unclassified | Eubacteriales | Clostridia | Firmicutes |
| Eubacteriales sp. (HG3A.0656) | AHI | -0.065 | 4.45E-04 | 0.003 | 3004 | unclassified | unclassified | unclassified | unclassified | Eubacteriales | Clostridia | Firmicutes |
| Eubacteriales sp. (HG3A.0664) | T90 | 0.059 | 6.63E-04 | 0.003 | 3364 | unclassified | unclassified | unclassified | unclassified | Eubacteriales | Clostridia | Firmicutes |
| Eubacteriales sp. (HG3A.0694) | ODI | -0.059 | 7.66E-04 | 0.003 | 3364 | unclassified | unclassified | unclassified | unclassified | Eubacteriales | Clostridia | Firmicutes |
| Eubacteriales sp. (HG3A.0759) | ODI | -0.059 | 7.35E-04 | 0.003 | 3364 | unclassified | unclassified | unclassified | unclassified | Eubacteriales | Clostridia | Firmicutes |
| Eubacteriales sp. (HG3A.0821) | ODI | -0.059 | 6.91E-04 | 0.003 | 3364 | unclassified | unclassified | unclassified | unclassified | Eubacteriales | Clostridia | Firmicutes |
| Eubacteriales sp. (HG3A.0859) | AHI | -0.064 | 4.89E-04 | 0.003 | 3004 | unclassified | unclassified | unclassified | unclassified | Eubacteriales | Clostridia | Firmicutes |
| Eubacteriales sp. (HG3A.0902) | ODI | -0.059 | 7.14E-04 | 0.003 | 3364 | unclassified | unclassified | unclassified | unclassified | Eubacteriales | Clostridia | Firmicutes |

| Eubacteriales sp. (HG3A.0908) | AHI | -0.063 | 5.73E-04 | 0.003 | 3004 | unclassified | unclassified | unclassified | unclassified | Eubacteriales | Clostridia | Firmicutes |
| --- | --- | --- | --- | --- | --- | --- | --- | --- | --- | --- | --- | --- |
| Eubacteriales sp. (HG3A.0914) | ODI | -0.058 | 7.73E-04 | 0.003 | 3364 | unclassified | unclassified | unclassified | unclassified | Eubacteriales | Clostridia | Firmicutes |
| Eubacteriales sp. (HG3A.0962) | T90 | -0.06 | 5.34E-04 | 0.003 | 3364 | unclassified | unclassified | unclassified | unclassified | Eubacteriales | Clostridia | Firmicutes |
| Eubacteriales sp. (HG3A.0970) | ODI | -0.058 | 8.91E-04 | 0.003 | 3364 | unclassified | unclassified | unclassified | unclassified | Eubacteriales | Clostridia | Firmicutes |
| Eubacteriales sp. (HG3A.1006) | ODI | -0.059 | 7.36E-04 | 0.003 | 3364 | unclassified | unclassified | unclassified | unclassified | Eubacteriales | Clostridia | Firmicutes |
| Eubacteriales sp. (HG3A.1063) | ODI | -0.058 | 9.00E-04 | 0.003 | 3364 | unclassified | unclassified | unclassified | unclassified | Eubacteriales | Clostridia | Firmicutes |
| Eubacteriales sp. (HG3A.1103) | ODI | -0.058 | 8.52E-04 | 0.003 | 3364 | unclassified | unclassified | unclassified | unclassified | Eubacteriales | Clostridia | Firmicutes |
| Firmicutes sp. (HG3A.0641) | T90 | -0.058 | 7.67E-04 | 0.003 | 3364 | unclassified | unclassified | unclassified | unclassified | unclassified | unclassified | Firmicutes |
| Firmicutes sp. (HG3A.0948) | ODI | -0.059 | 7.29E-04 | 0.003 | 3364 | unclassified | unclassified | unclassified | unclassified | unclassified | unclassified | Firmicutes |
| Lachnospiraceae sp. (HG3A.0252) | AHI | -0.065 | 4.31E-04 | 0.003 | 3004 | unclassified | unclassified | unclassified | Lachnospiraceae | Eubacteriales | Clostridia | Firmicutes |
| Lachnospiraceae sp. (HG3A.0393) | ODI | 0.059 | 7.23E-04 | 0.003 | 3364 | unclassified | unclassified | unclassified | Lachnospiraceae | Eubacteriales | Clostridia | Firmicutes |
| Methanobrevibacter smithii (HG3A.0152) | AHI | -0.063 | 5.83E-04 | 0.003 | 3004 | unclassified | Methanobrevibacter smithii | Methanobrevibacter | Methanobacteriac eae | Methanobacteriales | Methanobacteria | Euryarchaeota |
| Oscillospiraceae sp. (HG3A.0507) | AHI | -0.064 | 5.01E-04 | 0.003 | 3004 | unclassified | unclassified | unclassified | Oscillospiraceae | Eubacteriales | Clostridia | Firmicutes |
| Oscillospiraceae sp. (HG3A.0739) | ODI | -0.059 | 7.22E-04 | 0.003 | 3364 | unclassified | unclassified | unclassified | Oscillospiraceae | Eubacteriales | Clostridia | Firmicutes |
| Parasutterella excrementihominis (HG3A.0159) | ODI | -0.059 | 6.70E-04 | 0.003 | 3364 | unclassified | Parasutterella excrementihominis | Parasutterella | Sutterellaceae | Burkholderiales | Betaproteobacte ria | Proteobacteria |
| Proteobacteria sp. (HG3A.0360) | ODI | -0.058 | 9.13E-04 | 0.003 | 3364 | unclassified | unclassified | unclassified | unclassified | unclassified | unclassified | Proteobacteria |
| Pseudoflavonifractor sp. An184  (HG3A.0253) | ODI | -0.058 | 8.78E-04 | 0.003 | 3364 | unclassified | Pseudoflavonifractor  sp. An184 | Pseudoflavonifractor | Oscillospiraceae | Eubacteriales | Clostridia | Firmicutes |
| Roseburia sp. AM59-24XD (HG3A.0391) | AHI | -0.065 | 4.47E-04 | 0.003 | 3004 | unclassified | Roseburia sp. AM59- 24XD | Roseburia | Lachnospiraceae | Eubacteriales | Clostridia | Firmicutes |
| Rothia mucilaginosa (HG3A.0559) | T90 | 0.059 | 6.21E-04 | 0.003 | 3364 | unclassified | Rothia mucilaginosa | Rothia | Micrococcaceae | Micrococcales | Actinomycetia | Actinobacteria |
| Streptococcus salivarius (HG3A.0071) | AHI | 0.065 | 4.31E-04 | 0.003 | 3004 | unclassified | Streptococcus salivarius | Streptococcus | Streptococcaceae | Lactobacillales | Bacilli | Firmicutes |

Sutterella seckii (HG3A.0561) ODI 0.059 6.99E-04 0.003 3364 unclassified Sutterella seckii Sutterella Sutterellaceae Burkholderiales Betaproteobacte

ria

Proteobacteria

| Bacteria sp. (HG3A.1096) | T90 | -0.057 | 0.001 | 0.004 | 3364 | unclassified | unclassified | unclassified | unclassified | unclassified | unclassified | unclassified |
| --- | --- | --- | --- | --- | --- | --- | --- | --- | --- | --- | --- | --- |
| Butyricicoccus sp. OM04-18BH  (HG3A.0139) | AHI | -0.062 | 8.08E-04 | 0.004 | 3004 | unclassified | Butyricicoccus sp.  OM04-18BH | Butyricicoccus | Clostridiaceae | Eubacteriales | Clostridia | Firmicutes |
| Butyricicoccus sp. OM04-18BH (HG3A.0139) | ODI | -0.057 | 0.001 | 0.004 | 3364 | unclassified | Butyricicoccus sp.  OM04-18BH | Butyricicoccus | Clostridiaceae | Eubacteriales | Clostridia | Firmicutes |
| Candidatus Borkfalkiales sp.  (HG3A.1397) | T90 | -0.058 | 8.45E-04 | 0.004 | 3364 | unclassified | unclassified | unclassified | unclassified | Candidatus  Borkfalkiales | Clostridia | Firmicutes |
| Clostridia sp. (HG3A.0746) | AHI | -0.062 | 8.40E-04 | 0.004 | 3004 | unclassified | unclassified | unclassified | unclassified | unclassified | Clostridia | Firmicutes |
| Clostridia sp. (HG3A.0828) | ODI | -0.056 | 0.001 | 0.004 | 3364 | unclassified | unclassified | unclassified | unclassified | unclassified | Clostridia | Firmicutes |
| Clostridia sp. (HG3A.1062) | T90 | -0.058 | 8.07E-04 | 0.004 | 3364 | unclassified | unclassified | unclassified | unclassified | unclassified | Clostridia | Firmicutes |
| Clostridia sp. (HG3A.1111) | AHI | -0.062 | 7.81E-04 | 0.004 | 3004 | unclassified | unclassified | unclassified | unclassified | unclassified | Clostridia | Firmicutes |
| Clostridia sp. (HG3A.1157) | ODI | -0.056 | 0.001 | 0.004 | 3364 | unclassified | unclassified | unclassified | unclassified | unclassified | Clostridia | Firmicutes |
| Clostridia sp. (HG3A.1375) | T90 | -0.058 | 8.45E-04 | 0.004 | 3364 | unclassified | unclassified | unclassified | unclassified | unclassified | Clostridia | Firmicutes |
| Clostridiaceae sp. (HG3A.0330) | ODI | -0.057 | 0.001 | 0.004 | 3364 | unclassified | unclassified | unclassified | Clostridiaceae | Eubacteriales | Clostridia | Firmicutes |
| Enterocloster clostridioformis (HG3A.0686) | ODI | 0.057 | 0.001 | 0.004 | 3364 | unclassified | Enterocloster clostridioformis | Enterocloster | Lachnospiraceae | Eubacteriales | Clostridia | Firmicutes |
| Erysipelotrichales sp. (HG3A.0303) | AHI | -0.062 | 8.15E-04 | 0.004 | 3004 | unclassified | unclassified | unclassified | unclassified | Erysipelotrichales | Erysipelotrichia | Firmicutes |
| Erysipelotrichales sp.  (HG3A.1207) | AHI | 0.062 | 7.17E-04 | 0.004 | 3004 | unclassified | unclassified | unclassified | unclassified | Erysipelotrichales | Erysipelotrichia | Firmicutes |
| Eubacteriales sp. (HG3A.0116) | AHI | -0.062 | 7.69E-04 | 0.004 | 3004 | unclassified | unclassified | unclassified | unclassified | Eubacteriales | Clostridia | Firmicutes |
| Eubacteriales sp. (HG3A.0116) | ODI | -0.057 | 9.78E-04 | 0.004 | 3364 | unclassified | unclassified | unclassified | unclassified | Eubacteriales | Clostridia | Firmicutes |
| Eubacteriales sp. (HG3A.0156) | T90 | -0.057 | 9.88E-04 | 0.004 | 3364 | unclassified | unclassified | unclassified | unclassified | Eubacteriales | Clostridia | Firmicutes |
| Eubacteriales sp. (HG3A.0175) | AHI | -0.062 | 7.45E-04 | 0.004 | 3004 | unclassified | unclassified | unclassified | unclassified | Eubacteriales | Clostridia | Firmicutes |
| Eubacteriales sp. (HG3A.0260) | AHI | -0.063 | 6.77E-04 | 0.004 | 3004 | unclassified | unclassified | unclassified | unclassified | Eubacteriales | Clostridia | Firmicutes |
| Eubacteriales sp. (HG3A.0263) | AHI | -0.063 | 6.44E-04 | 0.004 | 3004 | unclassified | unclassified | unclassified | unclassified | Eubacteriales | Clostridia | Firmicutes |
| Eubacteriales sp. (HG3A.0267) | AHI | -0.063 | 6.21E-04 | 0.004 | 3004 | unclassified | unclassified | unclassified | unclassified | Eubacteriales | Clostridia | Firmicutes |

| Eubacteriales sp. (HG3A.0291) | AHI | -0.062 | 8.24E-04 | 0.004 | 3004 | unclassified | unclassified | unclassified | unclassified | Eubacteriales | Clostridia | Firmicutes |
| --- | --- | --- | --- | --- | --- | --- | --- | --- | --- | --- | --- | --- |
| Eubacteriales sp. (HG3A.0320) | T90 | -0.058 | 7.82E-04 | 0.004 | 3364 | unclassified | unclassified | unclassified | unclassified | Eubacteriales | Clostridia | Firmicutes |
| Eubacteriales sp. (HG3A.0427) | ODI | -0.057 | 0.001 | 0.004 | 3364 | unclassified | unclassified | unclassified | unclassified | Eubacteriales | Clostridia | Firmicutes |
| Eubacteriales sp. (HG3A.0428) | ODI | -0.056 | 0.001 | 0.004 | 3364 | unclassified | unclassified | unclassified | unclassified | Eubacteriales | Clostridia | Firmicutes |
| Eubacteriales sp. (HG3A.0457) | AHI | -0.062 | 7.88E-04 | 0.004 | 3004 | unclassified | unclassified | unclassified | unclassified | Eubacteriales | Clostridia | Firmicutes |
| Eubacteriales sp. (HG3A.0589) | T90 | -0.058 | 8.83E-04 | 0.004 | 3364 | unclassified | unclassified | unclassified | unclassified | Eubacteriales | Clostridia | Firmicutes |
| Eubacteriales sp. (HG3A.0627) | T90 | -0.058 | 8.68E-04 | 0.004 | 3364 | unclassified | unclassified | unclassified | unclassified | Eubacteriales | Clostridia | Firmicutes |
| Eubacteriales sp. (HG3A.0628) | T90 | -0.058 | 8.35E-04 | 0.004 | 3364 | unclassified | unclassified | unclassified | unclassified | Eubacteriales | Clostridia | Firmicutes |
| Eubacteriales sp. (HG3A.0654) | AHI | -0.062 | 7.30E-04 | 0.004 | 3004 | unclassified | unclassified | unclassified | unclassified | Eubacteriales | Clostridia | Firmicutes |
| Eubacteriales sp. (HG3A.0671) | AHI | -0.061 | 8.50E-04 | 0.004 | 3004 | unclassified | unclassified | unclassified | unclassified | Eubacteriales | Clostridia | Firmicutes |
| Eubacteriales sp. (HG3A.0703) | AHI | -0.063 | 6.70E-04 | 0.004 | 3004 | unclassified | unclassified | unclassified | unclassified | Eubacteriales | Clostridia | Firmicutes |
| Eubacteriales sp. (HG3A.0736) | T90 | -0.058 | 9.35E-04 | 0.004 | 3364 | unclassified | unclassified | unclassified | unclassified | Eubacteriales | Clostridia | Firmicutes |
| Eubacteriales sp. (HG3A.0758) | AHI | -0.063 | 6.61E-04 | 0.004 | 3004 | unclassified | unclassified | unclassified | unclassified | Eubacteriales | Clostridia | Firmicutes |
| Eubacteriales sp. (HG3A.0790) | AHI | -0.062 | 7.30E-04 | 0.004 | 3004 | unclassified | unclassified | unclassified | unclassified | Eubacteriales | Clostridia | Firmicutes |
| Eubacteriales sp. (HG3A.0807) | ODI | -0.057 | 9.87E-04 | 0.004 | 3364 | unclassified | unclassified | unclassified | unclassified | Eubacteriales | Clostridia | Firmicutes |
| Eubacteriales sp. (HG3A.0873) | ODI | -0.057 | 9.81E-04 | 0.004 | 3364 | unclassified | unclassified | unclassified | unclassified | Eubacteriales | Clostridia | Firmicutes |
| Eubacteriales sp. (HG3A.0881) | AHI | -0.063 | 6.44E-04 | 0.004 | 3004 | unclassified | unclassified | unclassified | unclassified | Eubacteriales | Clostridia | Firmicutes |
| Eubacteriales sp. (HG3A.0964) | AHI | -0.062 | 8.02E-04 | 0.004 | 3004 | unclassified | unclassified | unclassified | unclassified | Eubacteriales | Clostridia | Firmicutes |
| Eubacteriales sp. (HG3A.1067) | T90 | -0.057 | 9.99E-04 | 0.004 | 3364 | unclassified | unclassified | unclassified | unclassified | Eubacteriales | Clostridia | Firmicutes |
| Eubacteriales sp. (HG3A.1227) | AHI | -0.062 | 8.31E-04 | 0.004 | 3004 | unclassified | unclassified | unclassified | unclassified | Eubacteriales | Clostridia | Firmicutes |
| Eubacteriales sp. (HG3A.1294) | T90 | -0.058 | 8.83E-04 | 0.004 | 3364 | unclassified | unclassified | unclassified | unclassified | Eubacteriales | Clostridia | Firmicutes |
| Eubacteriales sp. (HG3A.1377) | T90 | -0.057 | 9.78E-04 | 0.004 | 3364 | unclassified | unclassified | unclassified | unclassified | Eubacteriales | Clostridia | Firmicutes |

| Eubacteriales sp. (HG3A.1439) | T90 | -0.057 | 9.74E-04 | 0.004 | 3364 | unclassified | unclassified | unclassified | unclassified | Eubacteriales | Clostridia | Firmicutes |
| --- | --- | --- | --- | --- | --- | --- | --- | --- | --- | --- | --- | --- |
| Firmicutes sp. (HG3A.0436) | T90 | -0.057 | 9.51E-04 | 0.004 | 3364 | unclassified | unclassified | unclassified | unclassified | unclassified | unclassified | Firmicutes |
| Firmicutes sp. (HG3A.0581) | AHI | -0.063 | 6.85E-04 | 0.004 | 3004 | unclassified | unclassified | unclassified | unclassified | unclassified | unclassified | Firmicutes |
| Firmicutes sp. (HG3A.0641) | AHI | -0.062 | 8.16E-04 | 0.004 | 3004 | unclassified | unclassified | unclassified | unclassified | unclassified | unclassified | Firmicutes |
| Gemmiger formicilis (HG3A.0027) | AHI | 0.063 | 6.55E-04 | 0.004 | 3004 | unclassified | Gemmiger formicilis | Gemmiger | unclassified | Eubacteriales | Clostridia | Firmicutes |
| Intestinimonas sp. (HG3A.1018) | T90 | -0.057 | 9.84E-04 | 0.004 | 3364 | unclassified | unclassified | Intestinimonas | unclassified | Eubacteriales | Clostridia | Firmicutes |
| Lachnoclostridium sp. (HG3A.0655)  Limosilactobacillus vaginalis (HG3A.1341) | ODI  AHI | 0.057  0.061 | 0.001  8.44E-04 | 0.004  0.004 | 3364  3004 | unclassified  unclassified | unclassified  Limosilactobacillus vaginalis | Lachnoclostridium  Limosilactobacillus | Lachnospiraceae  Lactobacillaceae | Eubacteriales  Lactobacillales | Clostridia  Bacilli | Firmicutes  Firmicutes |
| Oscillospiraceae sp. (HG3A.0380) | AHI | -0.062 | 7.63E-04 | 0.004 | 3004 | unclassified | unclassified | unclassified | Oscillospiraceae | Eubacteriales | Clostridia | Firmicutes |
| Oscillospiraceae sp. (HG3A.0576) | ODI | -0.056 | 0.001 | 0.004 | 3364 | unclassified | unclassified | unclassified | Oscillospiraceae | Eubacteriales | Clostridia | Firmicutes |
| Oscillospiraceae sp. (HG3A.0805) | ODI | -0.056 | 0.001 | 0.004 | 3364 | unclassified | unclassified | unclassified | Oscillospiraceae | Eubacteriales | Clostridia | Firmicutes |
| Oscillospiraceae sp. (HG3A.0966) | T90 | -0.058 | 8.67E-04 | 0.004 | 3364 | unclassified | unclassified | unclassified | Oscillospiraceae | Eubacteriales | Clostridia | Firmicutes |
| Oscillospiraceae sp. (HG3A.0966) | ODI | -0.057 | 0.001 | 0.004 | 3364 | unclassified | unclassified | unclassified | Oscillospiraceae | Eubacteriales | Clostridia | Firmicutes |
| Oscillospiraceae sp. (HG3A.1173) | AHI | -0.063 | 6.84E-04 | 0.004 | 3004 | unclassified | unclassified | unclassified | Oscillospiraceae | Eubacteriales | Clostridia | Firmicutes |
| Oscillospiraceae sp. (HG3A.1270) | T90 | -0.058 | 7.80E-04 | 0.004 | 3364 | unclassified | unclassified | unclassified | Oscillospiraceae | Eubacteriales | Clostridia | Firmicutes |
| Parolsenella catena (HG3A.0499) | ODI | 0.057 | 0.001 | 0.004 | 3364 | unclassified | Parolsenella catena | Parolsenella | Atopobiaceae | Coriobacteriales | Coriobacteriia | Actinobacteria |

Pseudoflavonifractor sp. (HG3A.0844)

Pseudoruminococcus massiliensis

(HG3A.0346)

ODI

-0.056

0.001

0.004 3364 unclassified

Pseudoruminococcus Pseudoruminococcu Oscillospiraceae

massiliensis

s

Eubacteriales

Clostridia

Firmicutes

ODI -0.056 0.001 0.004 3364 unclassified unclassified Pseudoflavonifractor Oscillospiraceae Eubacteriales Clostridia Firmicutes

Ruthenibacterium lactatiformans Ruthenibacterium

| (HG3A.0020) | AHI | 0.062 | 8.18E-04 | 0.004 | 3004 | unclassified | lactatiformans | Ruthenibacterium | Oscillospiraceae | Eubacteriales Clostridia Firmicutes |
| --- | --- | --- | --- | --- | --- | --- | --- | --- | --- | --- |
| Sutterella seckii (HG3A.0561) | AHI | 0.062 | 8.10E-04 | 0.004 | 3004 | unclassified | Sutterella seckii | Sutterella | Sutterellaceae | Burkholderiales Betaproteobacte Proteobacteria  ria |

Alistipes provencensis (HG3A.0877)

T90 -0.056 0.001 0.005 3364 unclassified Alistipes

provencensis

Alistipes Rikenellaceae Bacteroidales Bacteroidia Bacteroidetes

| Alistipes timonensis (HG3A.0586) | ODI | -0.055 | 0.002 | 0.005 | 3364 | unclassified | Alistipes timonensis | Alistipes | Rikenellaceae | Bacteroidales | Bacteroidia | Bacteroidetes |
| --- | --- | --- | --- | --- | --- | --- | --- | --- | --- | --- | --- | --- |
| Anaeroglobus geminatus  (HG3A.1818) | ODI | 0.055 | 0.002 | 0.005 | 3364 | unclassified | Anaeroglobus  geminatus | Anaeroglobus | Veillonellaceae | Veillonellales | Negativicutes | Firmicutes |
| Bacteria sp. (HG3A.0492) | ODI | -0.055 | 0.001 | 0.005 | 3364 | unclassified | unclassified | unclassified | unclassified | unclassified | unclassified | unclassified |
| Bacteria sp. (HG3A.1096) | ODI | -0.055 | 0.001 | 0.005 | 3364 | unclassified | unclassified | unclassified | unclassified | unclassified | unclassified | unclassified |
| Clostridia sp. (HG3A.0401) | ODI | -0.055 | 0.002 | 0.005 | 3364 | unclassified | unclassified | unclassified | unclassified | unclassified | Clostridia | Firmicutes |
| Clostridia sp. (HG3A.0918) | T90 | -0.056 | 0.001 | 0.005 | 3364 | unclassified | unclassified | unclassified | unclassified | unclassified | Clostridia | Firmicutes |
| Clostridia sp. (HG3A.1010) | AHI | -0.061 | 9.14E-04 | 0.005 | 3004 | unclassified | unclassified | unclassified | unclassified | unclassified | Clostridia | Firmicutes |
| Clostridia sp. (HG3A.1111) | ODI | -0.056 | 0.001 | 0.005 | 3364 | unclassified | unclassified | unclassified | unclassified | unclassified | Clostridia | Firmicutes |
| Clostridia sp. (HG3A.1403) | ODI | -0.056 | 0.001 | 0.005 | 3364 | unclassified | unclassified | unclassified | unclassified | unclassified | Clostridia | Firmicutes |
| Clostridia sp. (HG3A.1410) | ODI | -0.055 | 0.002 | 0.005 | 3364 | unclassified | unclassified | unclassified | unclassified | unclassified | Clostridia | Firmicutes |
| Clostridia sp. (HG3A.1493) | ODI | -0.056 | 0.001 | 0.005 | 3364 | unclassified | unclassified | unclassified | unclassified | unclassified | Clostridia | Firmicutes |
| Clostridium sp. AF37-5  (HG3A.0076) | T90 | -0.056 | 0.001 | 0.005 | 3364 | unclassified | Clostridium sp. AF37-  5 | Clostridium | Clostridiaceae | Eubacteriales | Clostridia | Firmicutes |
| Eubacteriales sp. (HG3A.0123) | AHI | 0.061 | 9.24E-04 | 0.005 | 3004 | unclassified | unclassified | unclassified | unclassified | Eubacteriales | Clostridia | Firmicutes |
| Eubacteriales sp. (HG3A.0282) | AHI | -0.06 | 0.001 | 0.005 | 3004 | unclassified | unclassified | unclassified | unclassified | Eubacteriales | Clostridia | Firmicutes |
| Eubacteriales sp. (HG3A.0322) | T90 | -0.057 | 0.001 | 0.005 | 3364 | unclassified | unclassified | unclassified | unclassified | Eubacteriales | Clostridia | Firmicutes |
| Eubacteriales sp. (HG3A.0350) | AHI | -0.06 | 0.001 | 0.005 | 3004 | unclassified | unclassified | unclassified | unclassified | Eubacteriales | Clostridia | Firmicutes |
| Eubacteriales sp. (HG3A.0428) | AHI | -0.061 | 8.58E-04 | 0.005 | 3004 | unclassified | unclassified | unclassified | unclassified | Eubacteriales | Clostridia | Firmicutes |
| Eubacteriales sp. (HG3A.0453) | AHI | -0.061 | 9.23E-04 | 0.005 | 3004 | unclassified | unclassified | unclassified | unclassified | Eubacteriales | Clostridia | Firmicutes |
| Eubacteriales sp. (HG3A.0502) | T90 | -0.056 | 0.001 | 0.005 | 3364 | unclassified | unclassified | unclassified | unclassified | Eubacteriales | Clostridia | Firmicutes |
| Eubacteriales sp. (HG3A.0546) | ODI | -0.056 | 0.001 | 0.005 | 3364 | unclassified | unclassified | unclassified | unclassified | Eubacteriales | Clostridia | Firmicutes |
| Eubacteriales sp. (HG3A.0577) | T90 | -0.056 | 0.001 | 0.005 | 3364 | unclassified | unclassified | unclassified | unclassified | Eubacteriales | Clostridia | Firmicutes |
| Eubacteriales sp. (HG3A.0624) | ODI | -0.055 | 0.001 | 0.005 | 3364 | unclassified | unclassified | unclassified | unclassified | Eubacteriales | Clostridia | Firmicutes |

| Eubacteriales sp. (HG3A.0635) | AHI | -0.061 | 9.98E-04 | 0.005 | 3004 | unclassified | unclassified | unclassified | unclassified | Eubacteriales | Clostridia | Firmicutes |
| --- | --- | --- | --- | --- | --- | --- | --- | --- | --- | --- | --- | --- |
| Eubacteriales sp. (HG3A.0701) | T90 | -0.056 | 0.001 | 0.005 | 3364 | unclassified | unclassified | unclassified | unclassified | Eubacteriales | Clostridia | Firmicutes |
| Eubacteriales sp. (HG3A.0715) | T90 | -0.056 | 0.001 | 0.005 | 3364 | unclassified | unclassified | unclassified | unclassified | Eubacteriales | Clostridia | Firmicutes |
| Eubacteriales sp. (HG3A.0759) | T90 | -0.056 | 0.001 | 0.005 | 3364 | unclassified | unclassified | unclassified | unclassified | Eubacteriales | Clostridia | Firmicutes |
| Eubacteriales sp. (HG3A.0760) | ODI | -0.056 | 0.001 | 0.005 | 3364 | unclassified | unclassified | unclassified | unclassified | Eubacteriales | Clostridia | Firmicutes |
| Eubacteriales sp. (HG3A.0870) | AHI | -0.061 | 9.38E-04 | 0.005 | 3004 | unclassified | unclassified | unclassified | unclassified | Eubacteriales | Clostridia | Firmicutes |
| Eubacteriales sp. (HG3A.0970) | T90 | -0.057 | 0.001 | 0.005 | 3364 | unclassified | unclassified | unclassified | unclassified | Eubacteriales | Clostridia | Firmicutes |
| Eubacteriales sp. (HG3A.1019) | AHI | -0.061 | 9.67E-04 | 0.005 | 3004 | unclassified | unclassified | unclassified | unclassified | Eubacteriales | Clostridia | Firmicutes |
| Eubacteriales sp. (HG3A.1126) | T90 | -0.057 | 0.001 | 0.005 | 3364 | unclassified | unclassified | unclassified | unclassified | Eubacteriales | Clostridia | Firmicutes |
| Eubacteriales sp. (HG3A.1445) | AHI | -0.061 | 8.98E-04 | 0.005 | 3004 | unclassified | unclassified | unclassified | unclassified | Eubacteriales | Clostridia | Firmicutes |
| Eubacterium ramulus (HG3A.0068) | AHI | 0.061 | 9.24E-04 | 0.005 | 3004 | unclassified | Eubacterium ramulus | Eubacterium | Eubacteriaceae | Eubacteriales | Clostridia | Firmicutes |
| Faecalibacterium prausnitzii  (HG3A.0025) | T90 | 0.056 | 0.001 | 0.005 | 3364 | unclassified | Faecalibacterium  prausnitzii | Faecalibacterium | Oscillospiraceae | Eubacteriales | Clostridia | Firmicutes |
| Faecalibacterium sp. (HG3A.0073) | AHI | -0.061 | 9.14E-04 | 0.005 | 3004 | unclassified | unclassified | Faecalibacterium | Oscillospiraceae | Eubacteriales | Clostridia | Firmicutes |
| Firmicutes sp. (HG3A.0526) | AHI | -0.061 | 0.001 | 0.005 | 3004 | unclassified | unclassified | unclassified | unclassified | unclassified | unclassified | Firmicutes |
| Flavonifractor sp. An10 (HG3A.0495) | T90 | -0.057 | 0.001 | 0.005 | 3364 | unclassified | Flavonifractor sp.  An10 | Flavonifractor | Oscillospiraceae | Eubacteriales | Clostridia | Firmicutes |
| Gemella morbillorum  (HG3A.1782) | AHI | 0.06 | 0.001 | 0.005 | 3004 | unclassified | Gemella morbillorum | Gemella | unclassified | Bacillales | Bacilli | Firmicutes |
| Latilactobacillus sakei subsp. sakei (HG3A.0836) | AHI | 0.061 | 9.90E-04 | 0.005 | 3004 | Latilactobacillus sakei subsp. sakei | Latilactobacillus sakei | Latilactobacillus | Lactobacillaceae | Lactobacillales | Bacilli | Firmicutes |
| Limosilactobacillus vaginalis  (HG3A.1341) | ODI | 0.055 | 0.001 | 0.005 | 3364 | unclassified | Limosilactobacillus  vaginalis | Limosilactobacillus | Lactobacillaceae | Lactobacillales | Bacilli | Firmicutes |
| Oscillospiraceae sp. (HG3A.0616) | ODI | -0.055 | 0.002 | 0.005 | 3364 | unclassified | unclassified | unclassified | Oscillospiraceae | Eubacteriales | Clostridia | Firmicutes |
| Parabacteroides goldsteinii  (HG3A.0279) | ODI | -0.055 | 0.002 | 0.005 | 3364 | unclassified | Parabacteroides  goldsteinii | Parabacteroides | Tannerellaceae | Bacteroidales | Bacteroidia | Bacteroidetes |
| Ruminococcus sp. (HG3A.0126) | ODI | -0.056 | 0.001 | 0.005 | 3364 | unclassified | unclassified | Ruminococcus | Oscillospiraceae | Eubacteriales | Clostridia | Firmicutes |

Staphylococcus aureus (HG3A.1538)

| Sutterella wadsworthensis (HG3A.0143) | T90 | -0.057 | 0.001 | 0.005 | 3364 | unclassified | Sutterella wadsworthensis | Sutterella Sutterellaceae | Burkholderiales | Betaproteobacte ria | Proteobacteria |
| --- | --- | --- | --- | --- | --- | --- | --- | --- | --- | --- | --- |
| Anaerostipes hadrus (HG3A.0003) | ODI | 0.054 | 0.002 | 0.006 | 3364 | unclassified | Anaerostipes hadrus | Anaerostipes Lachnospiraceae | Eubacteriales | Clostridia | Firmicutes |
| Clostridia sp. (HG3A.0706) | T90 | -0.056 | 0.001 | 0.006 | 3364 | unclassified | unclassified | unclassified unclassified | unclassified | Clostridia | Firmicutes |
| Clostridia sp. (HG3A.1205) | AHI | -0.06 | 0.001 | 0.006 | 3004 | unclassified | unclassified | unclassified unclassified | unclassified | Clostridia | Firmicutes |
| Clostridium sp. AF37-5 (HG3A.0076) | AHI | -0.06 | 0.001 | 0.006 | 3004 | unclassified | Clostridium sp. AF37- 5 | Clostridium Clostridiaceae | Eubacteriales | Clostridia | Firmicutes |
| Coprobacillus sp. (HG3A.0022) | T90 | 0.055 | 0.001 | 0.006 | 3364 | unclassified | unclassified | Coprobacillus Coprobacillaceae | Erysipelotrichales | Erysipelotrichia | Firmicutes |
| Coprococcus catus (HG3A.0037) | T90 | 0.056 | 0.001 | 0.006 | 3364 | unclassified | Coprococcus catus | Coprococcus Lachnospiraceae | Eubacteriales | Clostridia | Firmicutes |
| Enterocloster citroniae  (HG3A.0285) | AHI | 0.059 | 0.001 | 0.006 | 3004 | unclassified | Enterocloster  citroniae | Enterocloster Lachnospiraceae | Eubacteriales | Clostridia | Firmicutes |
| Erysipelatoclostridium sp. (HG3A.0313) | T90 | -0.055 | 0.001 | 0.006 | 3364 | unclassified | unclassified | Erysipelatoclostridiu Erysipelotrichace m ae | Erysipelotrichales | Erysipelotrichia | Firmicutes |
| Eubacteriales sp. (HG3A.0267) | T90 | -0.055 | 0.002 | 0.006 | 3364 | unclassified | unclassified | unclassified unclassified | Eubacteriales | Clostridia | Firmicutes |
| Eubacteriales sp. (HG3A.0280) | T90 | -0.055 | 0.002 | 0.006 | 3364 | unclassified | unclassified | unclassified unclassified | Eubacteriales | Clostridia | Firmicutes |
| Eubacteriales sp. (HG3A.0309) | T90 | -0.055 | 0.001 | 0.006 | 3364 | unclassified | unclassified | unclassified unclassified | Eubacteriales | Clostridia | Firmicutes |
| Eubacteriales sp. (HG3A.0350) | T90 | -0.055 | 0.002 | 0.006 | 3364 | unclassified | unclassified | unclassified unclassified | Eubacteriales | Clostridia | Firmicutes |
| Eubacteriales sp. (HG3A.0511) | ODI | -0.054 | 0.002 | 0.006 | 3364 | unclassified | unclassified | unclassified unclassified | Eubacteriales | Clostridia | Firmicutes |
| Eubacteriales sp. (HG3A.0548) | AHI | -0.059 | 0.001 | 0.006 | 3004 | unclassified | unclassified | unclassified unclassified | Eubacteriales | Clostridia | Firmicutes |
| Eubacteriales sp. (HG3A.0573) | AHI | -0.06 | 0.001 | 0.006 | 3004 | unclassified | unclassified | unclassified unclassified | Eubacteriales | Clostridia | Firmicutes |
| Eubacteriales sp. (HG3A.0580) | ODI | -0.055 | 0.002 | 0.006 | 3364 | unclassified | unclassified | unclassified unclassified | Eubacteriales | Clostridia | Firmicutes |
| Eubacteriales sp. (HG3A.0589) | ODI | -0.054 | 0.002 | 0.006 | 3364 | unclassified | unclassified | unclassified unclassified | Eubacteriales | Clostridia | Firmicutes |
| Eubacteriales sp. (HG3A.0617) | AHI | -0.059 | 0.001 | 0.006 | 3004 | unclassified | unclassified | unclassified unclassified | Eubacteriales | Clostridia | Firmicutes |
| Eubacteriales sp. (HG3A.0670) | AHI | -0.06 | 0.001 | 0.006 | 3004 | unclassified | unclassified | unclassified unclassified | Eubacteriales | Clostridia | Firmicutes |
| Eubacteriales sp. (HG3A.0670) | ODI | -0.054 | 0.002 | 0.006 | 3364 | unclassified | unclassified | unclassified unclassified | Eubacteriales | Clostridia | Firmicutes |

ODI 0.056 0.001 0.005 3364 unclassified Staphylococcus

aureus

Staphylococcus Staphylococcacea

e

Bacillales Bacilli Firmicutes

| Eubacteriales sp. (HG3A.0696) | T90 | -0.055 | 0.002 | 0.006 | 3364 | unclassified | unclassified | unclassified | unclassified | Eubacteriales | Clostridia | Firmicutes |
| --- | --- | --- | --- | --- | --- | --- | --- | --- | --- | --- | --- | --- |
| Eubacteriales sp. (HG3A.0736) | ODI | -0.054 | 0.002 | 0.006 | 3364 | unclassified | unclassified | unclassified | unclassified | Eubacteriales | Clostridia | Firmicutes |
| Eubacteriales sp. (HG3A.0794) | AHI | -0.06 | 0.001 | 0.006 | 3004 | unclassified | unclassified | unclassified | unclassified | Eubacteriales | Clostridia | Firmicutes |
| Eubacteriales sp. (HG3A.0914) | T90 | -0.055 | 0.001 | 0.006 | 3364 | unclassified | unclassified | unclassified | unclassified | Eubacteriales | Clostridia | Firmicutes |
| Eubacteriales sp. (HG3A.0924) | T90 | -0.055 | 0.002 | 0.006 | 3364 | unclassified | unclassified | unclassified | unclassified | Eubacteriales | Clostridia | Firmicutes |
| Eubacteriales sp. (HG3A.1006) | T90 | -0.055 | 0.002 | 0.006 | 3364 | unclassified | unclassified | unclassified | unclassified | Eubacteriales | Clostridia | Firmicutes |
| Eubacteriales sp. (HG3A.1019) | ODI | -0.055 | 0.002 | 0.006 | 3364 | unclassified | unclassified | unclassified | unclassified | Eubacteriales | Clostridia | Firmicutes |
| Eubacteriales sp. (HG3A.1078) | ODI | -0.054 | 0.002 | 0.006 | 3364 | unclassified | unclassified | unclassified | unclassified | Eubacteriales | Clostridia | Firmicutes |
| Eubacteriales sp. (HG3A.1243) | T90 | -0.055 | 0.002 | 0.006 | 3364 | unclassified | unclassified | unclassified | unclassified | Eubacteriales | Clostridia | Firmicutes |
| Faecalibacterium sp. (HG3A.0073) | T90 | -0.055 | 0.001 | 0.006 | 3364 | unclassified | unclassified | Faecalibacterium | Oscillospiraceae | Eubacteriales | Clostridia | Firmicutes |
| Firmicutes sp. (HG3A.0570) | T90 | -0.055 | 0.002 | 0.006 | 3364 | unclassified | unclassified | unclassified | unclassified | unclassified | unclassified | Firmicutes |
| Firmicutes sp. (HG3A.0817) | T90 | -0.056 | 0.001 | 0.006 | 3364 | unclassified | unclassified | unclassified | unclassified | unclassified | unclassified | Firmicutes |
| Firmicutes sp. (HG3A.0923) | T90 | -0.055 | 0.002 | 0.006 | 3364 | unclassified | unclassified | unclassified | unclassified | unclassified | unclassified | Firmicutes |
| Firmicutes sp. (HG3A.1014) | T90 | -0.055 | 0.002 | 0.006 | 3364 | unclassified | unclassified | unclassified | unclassified | unclassified | unclassified | Firmicutes |
| Firmicutes sp. (HG3A.1075) | AHI | -0.06 | 0.001 | 0.006 | 3004 | unclassified | unclassified | unclassified | unclassified | unclassified | unclassified | Firmicutes |
| Firmicutes sp. (HG3A.1085) | AHI | -0.059 | 0.001 | 0.006 | 3004 | unclassified | unclassified | unclassified | unclassified | unclassified | unclassified | Firmicutes |
| Fusobacterium nucleatum subsp. animalis (HG3A.1418) | ODI | 0.055 | 0.002 | 0.006 | 3364 | Fusobacterium nucleatum subsp.  animalis | Fusobacterium nucleatum | Fusobacterium | Fusobacteriaceae | Fusobacteriales | Fusobacteriia | Fusobacteria |
| Limosilactobacillus fermentum (HG3A.0990) | AHI | 0.06 | 0.001 | 0.006 | 3004 | unclassified | Limosilactobacillus fermentum | Limosilactobacillus | Lactobacillaceae | Lactobacillales | Bacilli | Firmicutes |
| Massilistercora timonensis  (HG3A.0458) | ODI | -0.054 | 0.002 | 0.006 | 3364 | unclassified | Massilistercora  timonensis | Massilistercora | unclassified | Eubacteriales | Clostridia | Firmicutes |
| Oscillospiraceae sp. (HG3A.0382) | ODI | -0.054 | 0.002 | 0.006 | 3364 | unclassified | unclassified | unclassified | Oscillospiraceae | Eubacteriales | Clostridia | Firmicutes |
| Oscillospiraceae sp. (HG3A.0774) | AHI | -0.06 | 0.001 | 0.006 | 3004 | unclassified | unclassified | unclassified | Oscillospiraceae | Eubacteriales | Clostridia | Firmicutes |

| Veillonella tobetsuensis  (HG3A.1344) | T90 | -0.055 | 0.002 | 0.006 | 3364 | unclassified | Veillonella  tobetsuensis | Veillonella | Veillonellaceae | Veillonellales | Negativicutes | Firmicutes |
| --- | --- | --- | --- | --- | --- | --- | --- | --- | --- | --- | --- | --- |
| Alistipes senegalensis  (HG3A.0141) | T90 | -0.054 | 0.002 | 0.007 | 3364 | unclassified | Alistipes  senegalensis | Alistipes | Rikenellaceae | Bacteroidales | Bacteroidia | Bacteroidetes |
| Bacteroidales sp. (HG3A.1002) | T90 | -0.054 | 0.002 | 0.007 | 3364 | unclassified | unclassified | unclassified | unclassified | Bacteroidales | Bacteroidia | Bacteroidetes |
| Clostridia sp. (HG3A.0661) | AHI | -0.059 | 0.001 | 0.007 | 3004 | unclassified | unclassified | unclassified | unclassified | unclassified | Clostridia | Firmicutes |
| Clostridia sp. (HG3A.0661) | ODI | -0.054 | 0.002 | 0.007 | 3364 | unclassified | unclassified | unclassified | unclassified | unclassified | Clostridia | Firmicutes |
| Clostridia sp. (HG3A.0861) | ODI | -0.053 | 0.002 | 0.007 | 3364 | unclassified | unclassified | unclassified | unclassified | unclassified | Clostridia | Firmicutes |
| Clostridia sp. (HG3A.1062) | AHI | -0.058 | 0.002 | 0.007 | 3004 | unclassified | unclassified | unclassified | unclassified | unclassified | Clostridia | Firmicutes |
| Clostridia sp. (HG3A.1111) | T90 | -0.054 | 0.002 | 0.007 | 3364 | unclassified | unclassified | unclassified | unclassified | unclassified | Clostridia | Firmicutes |
| Clostridia sp. (HG3A.1262) | ODI | -0.053 | 0.002 | 0.007 | 3364 | unclassified | unclassified | unclassified | unclassified | unclassified | Clostridia | Firmicutes |
| Clostridia sp. (HG3A.1375) | AHI | -0.059 | 0.001 | 0.007 | 3004 | unclassified | unclassified | unclassified | unclassified | unclassified | Clostridia | Firmicutes |
| Clostridium sp. M62/1 (HG3A.0354) | ODI | 0.054 | 0.002 | 0.007 | 3364 | unclassified | Clostridium sp.  M62/1 | Clostridium | Clostridiaceae | Eubacteriales | Clostridia | Firmicutes |
| Dorea phocaeensis (HG3A.0865) | AHI | 0.059 | 0.001 | 0.007 | 3004 | unclassified | Dorea phocaeensis | Dorea | Lachnospiraceae | Eubacteriales | Clostridia | Firmicutes |
| Eggerthellales sp. (HG3A.0174) | ODI | -0.054 | 0.002 | 0.007 | 3364 | unclassified | unclassified | unclassified | unclassified | Eggerthellales | Coriobacteriia | Actinobacteria |
| Eubacteriales sp. (HG3A.0308) | AHI | -0.059 | 0.001 | 0.007 | 3004 | unclassified | unclassified | unclassified | unclassified | Eubacteriales | Clostridia | Firmicutes |
| Eubacteriales sp. (HG3A.0335) | AHI | 0.058 | 0.002 | 0.007 | 3004 | unclassified | unclassified | unclassified | unclassified | Eubacteriales | Clostridia | Firmicutes |
| Eubacteriales sp. (HG3A.0390) | ODI | -0.053 | 0.002 | 0.007 | 3364 | unclassified | unclassified | unclassified | unclassified | Eubacteriales | Clostridia | Firmicutes |
| Eubacteriales sp. (HG3A.0450) | AHI | -0.058 | 0.002 | 0.007 | 3004 | unclassified | unclassified | unclassified | unclassified | Eubacteriales | Clostridia | Firmicutes |
| Eubacteriales sp. (HG3A.0457) | T90 | -0.054 | 0.002 | 0.007 | 3364 | unclassified | unclassified | unclassified | unclassified | Eubacteriales | Clostridia | Firmicutes |
| Eubacteriales sp. (HG3A.0511) | T90 | -0.055 | 0.002 | 0.007 | 3364 | unclassified | unclassified | unclassified | unclassified | Eubacteriales | Clostridia | Firmicutes |
| Eubacteriales sp. (HG3A.0540) | T90 | -0.054 | 0.002 | 0.007 | 3364 | unclassified | unclassified | unclassified | unclassified | Eubacteriales | Clostridia | Firmicutes |
| Eubacteriales sp. (HG3A.0546) | AHI | -0.059 | 0.001 | 0.007 | 3004 | unclassified | unclassified | unclassified | unclassified | Eubacteriales | Clostridia | Firmicutes |
| Eubacteriales sp. (HG3A.0557) | AHI | -0.058 | 0.002 | 0.007 | 3004 | unclassified | unclassified | unclassified | unclassified | Eubacteriales | Clostridia | Firmicutes |

| Eubacteriales sp. (HG3A.0621) | AHI | -0.059 | 0.001 | 0.007 | 3004 | unclassified | unclassified | unclassified | unclassified | Eubacteriales | Clostridia | Firmicutes |
| --- | --- | --- | --- | --- | --- | --- | --- | --- | --- | --- | --- | --- |
| Eubacteriales sp. (HG3A.0649) | ODI | -0.053 | 0.002 | 0.007 | 3364 | unclassified | unclassified | unclassified | unclassified | Eubacteriales | Clostridia | Firmicutes |
| Eubacteriales sp. (HG3A.0654) | ODI | -0.054 | 0.002 | 0.007 | 3364 | unclassified | unclassified | unclassified | unclassified | Eubacteriales | Clostridia | Firmicutes |
| Eubacteriales sp. (HG3A.0827) | T90 | -0.054 | 0.002 | 0.007 | 3364 | unclassified | unclassified | unclassified | unclassified | Eubacteriales | Clostridia | Firmicutes |
| Firmicutes sp. (HG3A.1471) | ODI | -0.054 | 0.002 | 0.007 | 3364 | unclassified | unclassified | unclassified | unclassified | unclassified | unclassified | Firmicutes |
| Oscillibacter sp. PEA192  (HG3A.0021) | AHI | 0.059 | 0.001 | 0.007 | 3004 | unclassified | Oscillibacter sp.  PEA192 | Oscillibacter | Oscillospiraceae | Eubacteriales | Clostridia | Firmicutes |
| Oscillospiraceae sp. (HG3A.0134) | ODI | -0.054 | 0.002 | 0.007 | 3364 | unclassified | unclassified | unclassified | Oscillospiraceae | Eubacteriales | Clostridia | Firmicutes |
| Oscillospiraceae sp. (HG3A.0146) | AHI | -0.059 | 0.001 | 0.007 | 3004 | unclassified | unclassified | unclassified | Oscillospiraceae | Eubacteriales | Clostridia | Firmicutes |
| Pediococcus acidilactici (HG3A.1468) | ODI | 0.053 | 0.002 | 0.007 | 3364 | unclassified | Pediococcus acidilactici | Pediococcus | Lactobacillaceae | Lactobacillales | Bacilli | Firmicutes |
| Pediococcus pentosaceus  (HG3A.1246) | ODI | 0.053 | 0.002 | 0.007 | 3364 | unclassified | Pediococcus  pentosaceus | Pediococcus | Lactobacillaceae | Lactobacillales | Bacilli | Firmicutes |
| Proteobacteria sp. (HG3A.0360) | T90 | -0.055 | 0.002 | 0.007 | 3364 | unclassified | unclassified | unclassified | unclassified | unclassified | unclassified | Proteobacteria |
| Ruthenibacterium lactatiformans (HG3A.0020) | ODI | 0.053 | 0.002 | 0.007 | 3364 | unclassified | Ruthenibacterium lactatiformans | Ruthenibacterium | Oscillospiraceae | Eubacteriales | Clostridia | Firmicutes |
| Streptococcus salivarius (HG3A.0071) | T90 | 0.055 | 0.002 | 0.007 | 3364 | unclassified | Streptococcus salivarius | Streptococcus | Streptococcaceae | Lactobacillales | Bacilli | Firmicutes |
| Allisonella histaminiformans (HG3A.0332) | T90 | 0.054 | 0.002 | 0.008 | 3364 | unclassified | Allisonella histaminiformans | Allisonella | Veillonellaceae | Veillonellales | Negativicutes | Firmicutes |
| Clostridia sp. (HG3A.0401) | AHI | -0.057 | 0.002 | 0.008 | 3004 | unclassified | unclassified | unclassified | unclassified | unclassified | Clostridia | Firmicutes |
| Clostridia sp. (HG3A.1108) | ODI | -0.053 | 0.002 | 0.008 | 3364 | unclassified | unclassified | unclassified | unclassified | unclassified | Clostridia | Firmicutes |
| Clostridia sp. (HG3A.1127) | ODI | -0.052 | 0.003 | 0.008 | 3364 | unclassified | unclassified | unclassified | unclassified | unclassified | Clostridia | Firmicutes |
| Clostridia sp. (HG3A.1493) | T90 | -0.053 | 0.002 | 0.008 | 3364 | unclassified | unclassified | unclassified | unclassified | unclassified | Clostridia | Firmicutes |
| Coprobacter fastidiosus (HG3A.0182) | ODI | -0.053 | 0.002 | 0.008 | 3364 | unclassified | Coprobacter fastidiosus | Coprobacter | Barnesiellaceae | Bacteroidales | Bacteroidia | Bacteroidetes |
| Coprococcus catus (HG3A.0037) | ODI | 0.052 | 0.003 | 0.008 | 3364 | unclassified | Coprococcus catus | Coprococcus | Lachnospiraceae | Eubacteriales | Clostridia | Firmicutes |
| Coprococcus sp. OM04-5BH (HG3A.1028) | AHI | -0.058 | 0.002 | 0.008 | 3004 | unclassified | Coprococcus sp. OM04-5BH | Coprococcus | Lachnospiraceae | Eubacteriales | Clostridia | Firmicutes |

Coprococcus sp. OM04-5BH (HG3A.1028)

| Desulfovibrionales sp. (HG3A.0727) | AHI | 0.058 | 0.002 | 0.008 | 3004 | unclassified | unclassified | unclassified | unclassified | Desulfovibrionales | Deltaproteobact eria | Proteobacteria |
| --- | --- | --- | --- | --- | --- | --- | --- | --- | --- | --- | --- | --- |
| Eubacteriales sp. (HG3A.0270) | AHI | -0.058 | 0.002 | 0.008 | 3004 | unclassified | unclassified | unclassified | unclassified | Eubacteriales | Clostridia | Firmicutes |
| Eubacteriales sp. (HG3A.0439) | T90 | -0.054 | 0.002 | 0.008 | 3364 | unclassified | unclassified | unclassified | unclassified | Eubacteriales | Clostridia | Firmicutes |
| Eubacteriales sp. (HG3A.0551) | ODI | -0.053 | 0.002 | 0.008 | 3364 | unclassified | unclassified | unclassified | unclassified | Eubacteriales | Clostridia | Firmicutes |
| Eubacteriales sp. (HG3A.0609) | T90 | -0.054 | 0.002 | 0.008 | 3364 | unclassified | unclassified | unclassified | unclassified | Eubacteriales | Clostridia | Firmicutes |
| Eubacteriales sp. (HG3A.0715) | AHI | -0.057 | 0.002 | 0.008 | 3004 | unclassified | unclassified | unclassified | unclassified | Eubacteriales | Clostridia | Firmicutes |
| Eubacteriales sp. (HG3A.1003) | AHI | -0.058 | 0.002 | 0.008 | 3004 | unclassified | unclassified | unclassified | unclassified | Eubacteriales | Clostridia | Firmicutes |
| Eubacteriales sp. (HG3A.1129) | ODI | -0.053 | 0.002 | 0.008 | 3364 | unclassified | unclassified | unclassified | unclassified | Eubacteriales | Clostridia | Firmicutes |
| Eubacteriales sp. (HG3A.1167) | AHI | -0.058 | 0.002 | 0.008 | 3004 | unclassified | unclassified | unclassified | unclassified | Eubacteriales | Clostridia | Firmicutes |
| Eubacterium sp. AF17-7  (HG3A.0165) | AHI | -0.058 | 0.002 | 0.008 | 3004 | unclassified | Eubacterium sp.  AF17-7 | Eubacterium | Eubacteriaceae | Eubacteriales | Clostridia | Firmicutes |
| Firmicutes sp. (HG3A.0860) | T90 | -0.053 | 0.002 | 0.008 | 3364 | unclassified | unclassified | unclassified | unclassified | unclassified | unclassified | Firmicutes |
| Lachnospiraceae sp. (HG3A.0257) | ODI | -0.053 | 0.002 | 0.008 | 3364 | unclassified | unclassified | unclassified | Lachnospiraceae | Eubacteriales | Clostridia | Firmicutes |
| Lachnospiraceae sp. (HG3A.0393) | AHI | 0.058 | 0.002 | 0.008 | 3004 | unclassified | unclassified | unclassified | Lachnospiraceae | Eubacteriales | Clostridia | Firmicutes |
| Limosilactobacillus oris  (HG3A.1462) | T90 | 0.054 | 0.002 | 0.008 | 3364 | unclassified | Limosilactobacillus  oris | Limosilactobacillus | Lactobacillaceae | Lactobacillales | Bacilli | Firmicutes |
| Olsenella sp. AF21-51 (HG3A.0690) | ODI | -0.053 | 0.002 | 0.008 | 3364 | unclassified | Olsenella sp. AF21- 51 | Olsenella | Atopobiaceae | Coriobacteriales | Coriobacteriia | Actinobacteria |
| Oscillospiraceae sp. (HG3A.0412) | ODI | -0.053 | 0.003 | 0.008 | 3364 | unclassified | unclassified | unclassified | Oscillospiraceae | Eubacteriales | Clostridia | Firmicutes |
| Parolsenella catena (HG3A.0499) | AHI | 0.058 | 0.002 | 0.008 | 3004 | unclassified | Parolsenella catena | Parolsenella | Atopobiaceae | Coriobacteriales | Coriobacteriia | Actinobacteria |
| Parolsenella catena (HG3A.0499) | T90 | 0.054 | 0.002 | 0.008 | 3364 | unclassified | Parolsenella catena | Parolsenella | Atopobiaceae | Coriobacteriales | Coriobacteriia | Actinobacteria |
| Anaerobutyricum hallii (HG3A.0112) | T90 | 0.053 | 0.002 | 0.009 | 3364 | unclassified | Anaerobutyricum hallii | Anaerobutyricum | Lachnospiraceae | Eubacteriales | Clostridia | Firmicutes |
| Anaerostipes sp. BG01  (HG3A.1509) | AHI | 0.057 | 0.002 | 0.009 | 3004 | unclassified | Anaerostipes sp.  BG01 | Anaerostipes | Lachnospiraceae | Eubacteriales | Clostridia | Firmicutes |
| Clostridia sp. (HG3A.0645) | AHI | -0.057 | 0.002 | 0.009 | 3004 | unclassified | unclassified | unclassified | unclassified | unclassified | Clostridia | Firmicutes |

ODI -0.053 0.002 0.008 3364 unclassified Coprococcus sp.

OM04-5BH

Coprococcus Lachnospiraceae Eubacteriales Clostridia Firmicutes

| Clostridia sp. (HG3A.0783) | AHI | -0.057 | 0.002 | 0.009 | 3004 | unclassified | unclassified | unclassified | unclassified | unclassified | Clostridia | Firmicutes |
| --- | --- | --- | --- | --- | --- | --- | --- | --- | --- | --- | --- | --- |
| Clostridia sp. (HG3A.1141) | AHI | -0.057 | 0.002 | 0.009 | 3004 | unclassified | unclassified | unclassified | unclassified | unclassified | Clostridia | Firmicutes |
| Clostridia sp. (HG3A.1403) | AHI | -0.057 | 0.002 | 0.009 | 3004 | unclassified | unclassified | unclassified | unclassified | unclassified | Clostridia | Firmicutes |
| Clostridiaceae sp. (HG3A.0491) | ODI | 0.052 | 0.003 | 0.009 | 3364 | unclassified | unclassified | unclassified | Clostridiaceae | Eubacteriales | Clostridia | Firmicutes |
| Erysipelotrichales sp.  (HG3A.0809) | AHI | 0.057 | 0.002 | 0.009 | 3004 | unclassified | unclassified | unclassified | unclassified | Erysipelotrichales | Erysipelotrichia | Firmicutes |
| Eubacteriales sp. (HG3A.0148) | AHI | -0.057 | 0.002 | 0.009 | 3004 | unclassified | unclassified | unclassified | unclassified | Eubacteriales | Clostridia | Firmicutes |
| Eubacteriales sp. (HG3A.0260) | T90 | -0.053 | 0.002 | 0.009 | 3364 | unclassified | unclassified | unclassified | unclassified | Eubacteriales | Clostridia | Firmicutes |
| Eubacteriales sp. (HG3A.0358) | ODI | -0.052 | 0.003 | 0.009 | 3364 | unclassified | unclassified | unclassified | unclassified | Eubacteriales | Clostridia | Firmicutes |
| Eubacteriales sp. (HG3A.0432) | ODI | -0.052 | 0.003 | 0.009 | 3364 | unclassified | unclassified | unclassified | unclassified | Eubacteriales | Clostridia | Firmicutes |
| Eubacteriales sp. (HG3A.0604) | AHI | -0.057 | 0.002 | 0.009 | 3004 | unclassified | unclassified | unclassified | unclassified | Eubacteriales | Clostridia | Firmicutes |
| Eubacteriales sp. (HG3A.0670) | T90 | -0.053 | 0.003 | 0.009 | 3364 | unclassified | unclassified | unclassified | unclassified | Eubacteriales | Clostridia | Firmicutes |
| Eubacteriales sp. (HG3A.0781) | T90 | -0.053 | 0.002 | 0.009 | 3364 | unclassified | unclassified | unclassified | unclassified | Eubacteriales | Clostridia | Firmicutes |
| Eubacteriales sp. (HG3A.0878) | T90 | -0.053 | 0.002 | 0.009 | 3364 | unclassified | unclassified | unclassified | unclassified | Eubacteriales | Clostridia | Firmicutes |
| Eubacteriales sp. (HG3A.0970) | AHI | -0.057 | 0.002 | 0.009 | 3004 | unclassified | unclassified | unclassified | unclassified | Eubacteriales | Clostridia | Firmicutes |
| Eubacteriales sp. (HG3A.1067) | ODI | -0.052 | 0.003 | 0.009 | 3364 | unclassified | unclassified | unclassified | unclassified | Eubacteriales | Clostridia | Firmicutes |
| Eubacteriales sp. (HG3A.1129) | T90 | -0.053 | 0.002 | 0.009 | 3364 | unclassified | unclassified | unclassified | unclassified | Eubacteriales | Clostridia | Firmicutes |
| Eubacteriales sp. (HG3A.1199) | T90 | 0.053 | 0.002 | 0.009 | 3364 | unclassified | unclassified | unclassified | unclassified | Eubacteriales | Clostridia | Firmicutes |
| Eubacteriales sp. (HG3A.1257) | T90 | -0.053 | 0.002 | 0.009 | 3364 | unclassified | unclassified | unclassified | unclassified | Eubacteriales | Clostridia | Firmicutes |
| Eubacteriales sp. (HG3A.1332) | ODI | -0.052 | 0.003 | 0.009 | 3364 | unclassified | unclassified | unclassified | unclassified | Eubacteriales | Clostridia | Firmicutes |
| Firmicutes sp. (HG3A.0948) | T90 | -0.053 | 0.002 | 0.009 | 3364 | unclassified | unclassified | unclassified | unclassified | unclassified | unclassified | Firmicutes |
| Firmicutes sp. (HG3A.1048) | T90 | -0.053 | 0.003 | 0.009 | 3364 | unclassified | unclassified | unclassified | unclassified | unclassified | unclassified | Firmicutes |
| Firmicutes sp. (HG3A.1162) | T90 | -0.053 | 0.002 | 0.009 | 3364 | unclassified | unclassified | unclassified | unclassified | unclassified | unclassified | Firmicutes |

Holdemanella sp. (HG3A.0366) AHI 0.057 0.002 0.009 3004 unclassified unclassified Holdemanella Erysipelotrichace

ae

Erysipelotrichales Erysipelotrichia Firmicutes

| Lachnotalea sp. AF33-28 (HG3A.0403) | ODI | -0.052 | 0.003 | 0.009 | 3364 | unclassified | Lachnotalea sp. AF33- 28 | Lachnotalea | Lachnospiraceae | Eubacteriales | Clostridia | Firmicutes |
| --- | --- | --- | --- | --- | --- | --- | --- | --- | --- | --- | --- | --- |
| Alistipes timonensis (HG3A.0586) | AHI | -0.056 | 0.002 | 0.01 | 3004 | unclassified | Alistipes timonensis | Alistipes | Rikenellaceae | Bacteroidales | Bacteroidia | Bacteroidetes |
| Alistipes timonensis (HG3A.0586) | T90 | -0.052 | 0.003 | 0.01 | 3364 | unclassified | Alistipes timonensis | Alistipes | Rikenellaceae | Bacteroidales | Bacteroidia | Bacteroidetes |
| Clostridia sp. (HG3A.0845) | T90 | -0.052 | 0.003 | 0.01 | 3364 | unclassified | unclassified | unclassified | unclassified | unclassified | Clostridia | Firmicutes |
| Clostridia sp. (HG3A.0918) | ODI | -0.051 | 0.003 | 0.01 | 3364 | unclassified | unclassified | unclassified | unclassified | unclassified | Clostridia | Firmicutes |
| Clostridia sp. (HG3A.1205) | ODI | -0.051 | 0.003 | 0.01 | 3364 | unclassified | unclassified | unclassified | unclassified | unclassified | Clostridia | Firmicutes |
| Clostridia sp. (HG3A.1262) | T90 | -0.052 | 0.003 | 0.01 | 3364 | unclassified | unclassified | unclassified | unclassified | unclassified | Clostridia | Firmicutes |
| Clostridium sp. OM07-9AC  (HG3A.0448) | T90 | -0.052 | 0.003 | 0.01 | 3364 | unclassified | Clostridium sp.  OM07-9AC | Clostridium | Clostridiaceae | Eubacteriales | Clostridia | Firmicutes |
| Enterocloster aldenensis (HG3A.0362) | AHI | 0.056 | 0.002 | 0.01 | 3004 | unclassified | Enterocloster aldenensis | Enterocloster | Lachnospiraceae | Eubacteriales | Clostridia | Firmicutes |
| Eubacteriales sp. (HG3A.0138) | AHI | -0.056 | 0.002 | 0.01 | 3004 | unclassified | unclassified | unclassified | unclassified | Eubacteriales | Clostridia | Firmicutes |
| Eubacteriales sp. (HG3A.0213) | AHI | -0.056 | 0.002 | 0.01 | 3004 | unclassified | unclassified | unclassified | unclassified | Eubacteriales | Clostridia | Firmicutes |
| Eubacteriales sp. (HG3A.0367) | ODI | -0.051 | 0.003 | 0.01 | 3364 | unclassified | unclassified | unclassified | unclassified | Eubacteriales | Clostridia | Firmicutes |
| Eubacteriales sp. (HG3A.0565) | AHI | -0.056 | 0.002 | 0.01 | 3004 | unclassified | unclassified | unclassified | unclassified | Eubacteriales | Clostridia | Firmicutes |
| Eubacteriales sp. (HG3A.0692) | ODI | 0.051 | 0.003 | 0.01 | 3364 | unclassified | unclassified | unclassified | unclassified | Eubacteriales | Clostridia | Firmicutes |
| Eubacteriales sp. (HG3A.0754) | ODI | -0.051 | 0.003 | 0.01 | 3364 | unclassified | unclassified | unclassified | unclassified | Eubacteriales | Clostridia | Firmicutes |
| Eubacteriales sp. (HG3A.0962) | ODI | -0.051 | 0.003 | 0.01 | 3364 | unclassified | unclassified | unclassified | unclassified | Eubacteriales | Clostridia | Firmicutes |
| Eubacteriales sp. (HG3A.0977) | ODI | -0.051 | 0.003 | 0.01 | 3364 | unclassified | unclassified | unclassified | unclassified | Eubacteriales | Clostridia | Firmicutes |
| Eubacteriales sp. (HG3A.1078) | T90 | -0.052 | 0.003 | 0.01 | 3364 | unclassified | unclassified | unclassified | unclassified | Eubacteriales | Clostridia | Firmicutes |
| Eubacteriales sp. (HG3A.1243) | AHI | -0.056 | 0.002 | 0.01 | 3004 | unclassified | unclassified | unclassified | unclassified | Eubacteriales | Clostridia | Firmicutes |
| Eubacteriales sp. (HG3A.1354) | T90 | -0.052 | 0.003 | 0.01 | 3364 | unclassified | unclassified | unclassified | unclassified | Eubacteriales | Clostridia | Firmicutes |
| Eubacteriales sp. (HG3A.1445) | ODI | -0.051 | 0.003 | 0.01 | 3364 | unclassified | unclassified | unclassified | unclassified | Eubacteriales | Clostridia | Firmicutes |

Eubacterium sp. AF22-8LB (HG3A.0838)

| Firmicutes sp. (HG3A.0570) | ODI | -0.051 | 0.003 | 0.01 | 3364 | unclassified | unclassified | unclassified | unclassified | unclassified | unclassified | Firmicutes |
| --- | --- | --- | --- | --- | --- | --- | --- | --- | --- | --- | --- | --- |
| Firmicutes sp. (HG3A.0874) | AHI | -0.056 | 0.002 | 0.01 | 3004 | unclassified | unclassified | unclassified | unclassified | unclassified | unclassified | Firmicutes |
| Senegalimassilia anaerobia (HG3A.0129) | T90 | 0.052 | 0.003 | 0.01 | 3364 | unclassified | Senegalimassilia anaerobia | Senegalimassilia | Coriobacteriaceae | Coriobacteriales | Coriobacteriia | Actinobacteria |
| Bacteroides cellulosilyticus (HG3A.0108) | AHI | -0.055 | 0.003 | 0.011 | 3004 | unclassified | Bacteroides cellulosilyticus | Bacteroides | Bacteroidaceae | Bacteroidales | Bacteroidia | Bacteroidetes |
| Blautia argi (HG3A.1450) | ODI | 0.051 | 0.003 | 0.011 | 3364 | unclassified | Blautia argi | Blautia | Lachnospiraceae | Eubacteriales | Clostridia | Firmicutes |
| Blautia sp. AF19-10LB  (HG3A.0157) | T90 | -0.052 | 0.003 | 0.011 | 3364 | unclassified | Blautia sp. AF19-  10LB | Blautia | Lachnospiraceae | Eubacteriales | Clostridia | Firmicutes |
| Catenibacterium mitsuokai (HG3A.0775) | AHI | 0.055 | 0.003 | 0.011 | 3004 | unclassified | Catenibacterium mitsuokai | Catenibacterium | Coprobacillaceae | Erysipelotrichales | Erysipelotrichia | Firmicutes |
| Clostridia sp. (HG3A.0512) | AHI | -0.055 | 0.003 | 0.011 | 3004 | unclassified | unclassified | unclassified | unclassified | unclassified | Clostridia | Firmicutes |
| Clostridia sp. (HG3A.0660) | AHI | -0.055 | 0.003 | 0.011 | 3004 | unclassified | unclassified | unclassified | unclassified | unclassified | Clostridia | Firmicutes |
| Clostridia sp. (HG3A.0996) | AHI | -0.055 | 0.003 | 0.011 | 3004 | unclassified | unclassified | unclassified | unclassified | unclassified | Clostridia | Firmicutes |
| Clostridium sp. SN20 (HG3A.0603) | AHI | 0.056 | 0.003 | 0.011 | 3004 | unclassified | Clostridium sp. SN20 | Clostridium | Clostridiaceae | Eubacteriales | Clostridia | Firmicutes |
| Coprobacillus sp. (HG3A.0022) | AHI | 0.056 | 0.003 | 0.011 | 3004 | unclassified | unclassified | Coprobacillus | Coprobacillaceae | Erysipelotrichales | Erysipelotrichia | Firmicutes |
| Eggerthellales sp. (HG3A.0174) | AHI | -0.055 | 0.003 | 0.011 | 3004 | unclassified | unclassified | unclassified | unclassified | Eggerthellales | Coriobacteriia | Actinobacteria |
| Erysipelotrichales sp.  (HG3A.0809) | ODI | 0.051 | 0.004 | 0.011 | 3364 | unclassified | unclassified | unclassified | unclassified | Erysipelotrichales | Erysipelotrichia | Firmicutes |
| Eubacteriales sp. (HG3A.0080) | T90 | 0.052 | 0.003 | 0.011 | 3364 | unclassified | unclassified | unclassified | unclassified | Eubacteriales | Clostridia | Firmicutes |
| Eubacteriales sp. (HG3A.0232) | AHI | -0.056 | 0.003 | 0.011 | 3004 | unclassified | unclassified | unclassified | unclassified | Eubacteriales | Clostridia | Firmicutes |
| Eubacteriales sp. (HG3A.0472) | AHI | -0.055 | 0.003 | 0.011 | 3004 | unclassified | unclassified | unclassified | unclassified | Eubacteriales | Clostridia | Firmicutes |
| Eubacteriales sp. (HG3A.0476) | AHI | -0.056 | 0.003 | 0.011 | 3004 | unclassified | unclassified | unclassified | unclassified | Eubacteriales | Clostridia | Firmicutes |
| Eubacteriales sp. (HG3A.0496) | ODI | -0.051 | 0.004 | 0.011 | 3364 | unclassified | unclassified | unclassified | unclassified | Eubacteriales | Clostridia | Firmicutes |
| Eubacteriales sp. (HG3A.0528) | AHI | -0.056 | 0.003 | 0.011 | 3004 | unclassified | unclassified | unclassified | unclassified | Eubacteriales | Clostridia | Firmicutes |

ODI 0.051 0.003 0.01 3364 unclassified Eubacterium sp.

AF22-8LB

Eubacterium Eubacteriaceae Eubacteriales Clostridia Firmicutes

| Eubacteriales sp. (HG3A.0668) | AHI | -0.056 | 0.002 | 0.011 | 3004 | unclassified | unclassified | unclassified | unclassified | Eubacteriales | Clostridia | Firmicutes |
| --- | --- | --- | --- | --- | --- | --- | --- | --- | --- | --- | --- | --- |
| Eubacteriales sp. (HG3A.0694) | AHI | -0.055 | 0.003 | 0.011 | 3004 | unclassified | unclassified | unclassified | unclassified | Eubacteriales | Clostridia | Firmicutes |
| Eubacteriales sp. (HG3A.0697) | AHI | -0.055 | 0.003 | 0.011 | 3004 | unclassified | unclassified | unclassified | unclassified | Eubacteriales | Clostridia | Firmicutes |
| Eubacteriales sp. (HG3A.0698) | AHI | 0.055 | 0.003 | 0.011 | 3004 | unclassified | unclassified | unclassified | unclassified | Eubacteriales | Clostridia | Firmicutes |
| Eubacteriales sp. (HG3A.0792) | ODI | -0.051 | 0.004 | 0.011 | 3364 | unclassified | unclassified | unclassified | unclassified | Eubacteriales | Clostridia | Firmicutes |
| Eubacteriales sp. (HG3A.1045) | ODI | -0.051 | 0.004 | 0.011 | 3364 | unclassified | unclassified | unclassified | unclassified | Eubacteriales | Clostridia | Firmicutes |
| Eubacteriales sp. (HG3A.1103) | AHI | -0.055 | 0.003 | 0.011 | 3004 | unclassified | unclassified | unclassified | unclassified | Eubacteriales | Clostridia | Firmicutes |
| Lachnospiraceae sp. (HG3A.1190) | AHI | -0.056 | 0.003 | 0.011 | 3004 | unclassified | unclassified | unclassified | Lachnospiraceae | Eubacteriales | Clostridia | Firmicutes |
| Oscillospiraceae sp. (HG3A.0382) | T90 | -0.052 | 0.003 | 0.011 | 3364 | unclassified | unclassified | unclassified | Oscillospiraceae | Eubacteriales | Clostridia | Firmicutes |
| Alistipes sp. An66 (HG3A.1535) | ODI | -0.05 | 0.004 | 0.012 | 3364 | unclassified | Alistipes sp. An66 | Alistipes | Rikenellaceae | Bacteroidales | Bacteroidia | Bacteroidetes |
| Anaerotruncus massiliensis (HG3A.0460) | T90 | -0.051 | 0.003 | 0.012 | 3364 | unclassified | Anaerotruncus massiliensis | Anaerotruncus | Oscillospiraceae | Eubacteriales | Clostridia | Firmicutes |
| Bacteria sp. (HG3A.0459) | AHI | -0.055 | 0.003 | 0.012 | 3004 | unclassified | unclassified | unclassified | unclassified | unclassified | unclassified | unclassified |
| Clostridia sp. (HG3A.0931) | AHI | -0.055 | 0.003 | 0.012 | 3004 | unclassified | unclassified | unclassified | unclassified | unclassified | Clostridia | Firmicutes |
| Clostridia sp. (HG3A.0933) | T90 | -0.051 | 0.003 | 0.012 | 3364 | unclassified | unclassified | unclassified | unclassified | unclassified | Clostridia | Firmicutes |
| Clostridia sp. (HG3A.1157) | T90 | -0.051 | 0.003 | 0.012 | 3364 | unclassified | unclassified | unclassified | unclassified | unclassified | Clostridia | Firmicutes |
| Eubacteriales sp. (HG3A.0175) | ODI | -0.05 | 0.004 | 0.012 | 3364 | unclassified | unclassified | unclassified | unclassified | Eubacteriales | Clostridia | Firmicutes |
| Eubacteriales sp. (HG3A.0221) | ODI | -0.05 | 0.004 | 0.012 | 3364 | unclassified | unclassified | unclassified | unclassified | Eubacteriales | Clostridia | Firmicutes |
| Eubacteriales sp. (HG3A.0352) | AHI | -0.055 | 0.003 | 0.012 | 3004 | unclassified | unclassified | unclassified | unclassified | Eubacteriales | Clostridia | Firmicutes |
| Eubacteriales sp. (HG3A.0406) | AHI | -0.055 | 0.003 | 0.012 | 3004 | unclassified | unclassified | unclassified | unclassified | Eubacteriales | Clostridia | Firmicutes |
| Eubacteriales sp. (HG3A.0427) | AHI | -0.055 | 0.003 | 0.012 | 3004 | unclassified | unclassified | unclassified | unclassified | Eubacteriales | Clostridia | Firmicutes |
| Eubacteriales sp. (HG3A.0439) | AHI | -0.055 | 0.003 | 0.012 | 3004 | unclassified | unclassified | unclassified | unclassified | Eubacteriales | Clostridia | Firmicutes |
| Eubacteriales sp. (HG3A.0649) | T90 | -0.051 | 0.003 | 0.012 | 3364 | unclassified | unclassified | unclassified | unclassified | Eubacteriales | Clostridia | Firmicutes |

| Eubacteriales sp. (HG3A.0652) | AHI | -0.055 | 0.003 | 0.012 | 3004 | unclassified | unclassified | unclassified | unclassified | Eubacteriales | Clostridia | Firmicutes |
| --- | --- | --- | --- | --- | --- | --- | --- | --- | --- | --- | --- | --- |
| Eubacteriales sp. (HG3A.0751) | ODI | -0.05 | 0.004 | 0.012 | 3364 | unclassified | unclassified | unclassified | unclassified | Eubacteriales | Clostridia | Firmicutes |
| Eubacteriales sp. (HG3A.0792) | T90 | -0.051 | 0.003 | 0.012 | 3364 | unclassified | unclassified | unclassified | unclassified | Eubacteriales | Clostridia | Firmicutes |
| Eubacteriales sp. (HG3A.0807) | T90 | -0.051 | 0.003 | 0.012 | 3364 | unclassified | unclassified | unclassified | unclassified | Eubacteriales | Clostridia | Firmicutes |
| Eubacteriales sp. (HG3A.1087) | ODI | -0.05 | 0.004 | 0.012 | 3364 | unclassified | unclassified | unclassified | unclassified | Eubacteriales | Clostridia | Firmicutes |
| Eubacteriales sp. (HG3A.1239) | ODI | -0.05 | 0.004 | 0.012 | 3364 | unclassified | unclassified | unclassified | unclassified | Eubacteriales | Clostridia | Firmicutes |
| Eubacteriales sp. (HG3A.1321) | ODI | -0.05 | 0.004 | 0.012 | 3364 | unclassified | unclassified | unclassified | unclassified | Eubacteriales | Clostridia | Firmicutes |
| Firmicutes sp. (HG3A.1082) | T90 | -0.051 | 0.003 | 0.012 | 3364 | unclassified | unclassified | unclassified | unclassified | unclassified | unclassified | Firmicutes |
| Hungatella hathewayi (HG3A.0455) | T90 | 0.051 | 0.003 | 0.012 | 3364 | unclassified | Hungatella hathewayi | Hungatella | Clostridiaceae | Eubacteriales | Clostridia | Firmicutes |
| Hydrogeniiclostidium mannosilyticum (HG3A.0294) | ODI | 0.05 | 0.004 | 0.012 | 3364 | unclassified | Hydrogeniiclostidium mannosilyticum | Hydrogeniiclostidiu m | Oscillospiraceae | Eubacteriales | Clostridia | Firmicutes |
| Streptococcus gordonii (HG3A.0713) | AHI | 0.055 | 0.003 | 0.012 | 3004 | unclassified | Streptococcus gordonii | Streptococcus | Streptococcaceae | Lactobacillales | Bacilli | Firmicutes |
| Alistipes indistinctus (HG3A.0121) | AHI | -0.054 | 0.003 | 0.013 | 3004 | unclassified | Alistipes indistinctus | Alistipes | Rikenellaceae | Bacteroidales | Bacteroidia | Bacteroidetes |
| Anaerostipes hadrus (HG3A.0003) | T90 | 0.05 | 0.004 | 0.013 | 3364 | unclassified | Anaerostipes hadrus | Anaerostipes | Lachnospiraceae | Eubacteriales | Clostridia | Firmicutes |
| Bacteria sp. (HG3A.1545) | T90 | -0.051 | 0.004 | 0.013 | 3364 | unclassified | unclassified | unclassified | unclassified | unclassified | unclassified | unclassified |
| Bacteroidales sp. (HG3A.1446) | T90 | -0.05 | 0.004 | 0.013 | 3364 | unclassified | unclassified | unclassified | unclassified | Bacteroidales | Bacteroidia | Bacteroidetes |
| Clostridia sp. (HG3A.0946) | AHI | -0.054 | 0.003 | 0.013 | 3004 | unclassified | unclassified | unclassified | unclassified | unclassified | Clostridia | Firmicutes |
| Clostridia sp. (HG3A.0996) | ODI | -0.049 | 0.004 | 0.013 | 3364 | unclassified | unclassified | unclassified | unclassified | unclassified | Clostridia | Firmicutes |
| Clostridia sp. (HG3A.1008) | AHI | -0.054 | 0.003 | 0.013 | 3004 | unclassified | unclassified | unclassified | unclassified | unclassified | Clostridia | Firmicutes |
| Clostridia sp. (HG3A.1038) | ODI | -0.05 | 0.004 | 0.013 | 3364 | unclassified | unclassified | unclassified | unclassified | unclassified | Clostridia | Firmicutes |
| Clostridia sp. (HG3A.1058) | T90 | -0.05 | 0.004 | 0.013 | 3364 | unclassified | unclassified | unclassified | unclassified | unclassified | Clostridia | Firmicutes |
| Clostridia sp. (HG3A.1486) | T90 | -0.05 | 0.004 | 0.013 | 3364 | unclassified | unclassified | unclassified | unclassified | unclassified | Clostridia | Firmicutes |

| Clostridiaceae sp. (HG3A.0471) | ODI | -0.05 | 0.004 | 0.013 | 3364 | unclassified | unclassified | unclassified | Clostridiaceae | Eubacteriales | Clostridia | Firmicutes |
| --- | --- | --- | --- | --- | --- | --- | --- | --- | --- | --- | --- | --- |
| Clostridium sp. SN20 (HG3A.0603) | ODI | 0.049 | 0.004 | 0.013 | 3364 | unclassified | Clostridium sp. SN20 | Clostridium | Clostridiaceae | Eubacteriales | Clostridia | Firmicutes |
| Dorea sp. AF24-7LB (HG3A.0086) | ODI | 0.049 | 0.004 | 0.013 | 3364 | unclassified | Dorea sp. AF24-7LB | Dorea | Lachnospiraceae | Eubacteriales | Clostridia | Firmicutes |
| Eggerthellales sp. (HG3A.0848) | AHI | -0.054 | 0.003 | 0.013 | 3004 | unclassified | unclassified | unclassified | unclassified | Eggerthellales | Coriobacteriia | Actinobacteria |
| Eubacteriales sp. (HG3A.0137) | T90 | -0.051 | 0.004 | 0.013 | 3364 | unclassified | unclassified | unclassified | unclassified | Eubacteriales | Clostridia | Firmicutes |
| Eubacteriales sp. (HG3A.0270) | T90 | -0.05 | 0.004 | 0.013 | 3364 | unclassified | unclassified | unclassified | unclassified | Eubacteriales | Clostridia | Firmicutes |
| Eubacteriales sp. (HG3A.0547) | T90 | -0.05 | 0.004 | 0.013 | 3364 | unclassified | unclassified | unclassified | unclassified | Eubacteriales | Clostridia | Firmicutes |
| Eubacteriales sp. (HG3A.0967) | T90 | -0.05 | 0.004 | 0.013 | 3364 | unclassified | unclassified | unclassified | unclassified | Eubacteriales | Clostridia | Firmicutes |
| Eubacteriales sp. (HG3A.0973) | ODI | -0.05 | 0.004 | 0.013 | 3364 | unclassified | unclassified | unclassified | unclassified | Eubacteriales | Clostridia | Firmicutes |
| Eubacteriales sp. (HG3A.0983) | T90 | -0.051 | 0.004 | 0.013 | 3364 | unclassified | unclassified | unclassified | unclassified | Eubacteriales | Clostridia | Firmicutes |
| Eubacteriales sp. (HG3A.1134) | ODI | -0.049 | 0.005 | 0.013 | 3364 | unclassified | unclassified | unclassified | unclassified | Eubacteriales | Clostridia | Firmicutes |
| Eubacteriales sp. (HG3A.1321) | AHI | -0.054 | 0.003 | 0.013 | 3004 | unclassified | unclassified | unclassified | unclassified | Eubacteriales | Clostridia | Firmicutes |
| Eubacteriales sp. (HG3A.1332) | T90 | -0.051 | 0.004 | 0.013 | 3364 | unclassified | unclassified | unclassified | unclassified | Eubacteriales | Clostridia | Firmicutes |
| Lachnoclostridium sp. (HG3A.0655) | AHI | 0.054 | 0.003 | 0.013 | 3004 | unclassified | unclassified | Lachnoclostridium | Lachnospiraceae | Eubacteriales | Clostridia | Firmicutes |
| Lachnospiraceae sp. (HG3A.0831) | ODI | -0.049 | 0.004 | 0.013 | 3364 | unclassified | unclassified | unclassified | Lachnospiraceae | Eubacteriales | Clostridia | Firmicutes |
| Lachnotalea sp. AF33-28 (HG3A.0403) | AHI | -0.054 | 0.003 | 0.013 | 3004 | unclassified | Lachnotalea sp. AF33- 28 | Lachnotalea | Lachnospiraceae | Eubacteriales | Clostridia | Firmicutes |
| Oscillibacter sp. (HG3A.0243) | ODI | -0.05 | 0.004 | 0.013 | 3364 | unclassified | unclassified | Oscillibacter | Oscillospiraceae | Eubacteriales | Clostridia | Firmicutes |
| Oscillospiraceae sp. (HG3A.0380) | ODI | -0.05 | 0.004 | 0.013 | 3364 | unclassified | unclassified | unclassified | Oscillospiraceae | Eubacteriales | Clostridia | Firmicutes |
| Oxalobacter formigenes  (HG3A.0552) | T90 | -0.05 | 0.004 | 0.013 | 3364 | unclassified | Oxalobacter  formigenes | Oxalobacter | Oxalobacteraceae | Burkholderiales | Betaproteobacte  ria | Proteobacteria |
| Ruminococcus sp. (HG3A.0126) | AHI | -0.054 | 0.003 | 0.013 | 3004 | unclassified | unclassified | Ruminococcus | Oscillospiraceae | Eubacteriales | Clostridia | Firmicutes |
| Streptococcus gordonii  (HG3A.0713) | T90 | 0.05 | 0.004 | 0.013 | 3364 | unclassified | Streptococcus  gordonii | Streptococcus | Streptococcaceae | Lactobacillales | Bacilli | Firmicutes |
| Alistipes dispar (HG3A.0281) | AHI | -0.054 | 0.003 | 0.014 | 3004 | unclassified | Alistipes dispar | Alistipes | Rikenellaceae | Bacteroidales | Bacteroidia | Bacteroidetes |

Alistipes sp. AF17-16 (HG3A.0150)

AHI -0.054 0.004 0.014 3004 unclassified Alistipes sp. AF17-16 Alistipes Rikenellaceae Bacteroidales Bacteroidia Bacteroidetes

| Bacteroidales sp. (HG3A.1236) | AHI | -0.054 | 0.004 | 0.014 | 3004 | unclassified | unclassified | unclassified | unclassified | Bacteroidales | Bacteroidia | Bacteroidetes |
| --- | --- | --- | --- | --- | --- | --- | --- | --- | --- | --- | --- | --- |
| Clostridia sp. (HG3A.0660) | ODI | -0.049 | 0.005 | 0.014 | 3364 | unclassified | unclassified | unclassified | unclassified | unclassified | Clostridia | Firmicutes |
| Clostridiaceae sp. (HG3A.0608) | AHI | -0.053 | 0.004 | 0.014 | 3004 | unclassified | unclassified | unclassified | Clostridiaceae | Eubacteriales | Clostridia | Firmicutes |
| Clostridium sp. M62/1  (HG3A.0354) | T90 | 0.05 | 0.004 | 0.014 | 3364 | unclassified | Clostridium sp.  M62/1 | Clostridium | Clostridiaceae | Eubacteriales | Clostridia | Firmicutes |
| Clostridium sp. SN20 (HG3A.0603) | T90 | 0.05 | 0.004 | 0.014 | 3364 | unclassified | Clostridium sp. SN20 | Clostridium | Clostridiaceae | Eubacteriales | Clostridia | Firmicutes |
| Collinsella intestinalis  (HG3A.0802) | T90 | 0.05 | 0.004 | 0.014 | 3364 | unclassified | Collinsella  intestinalis | Collinsella | Coriobacteriaceae | Coriobacteriales | Coriobacteriia | Actinobacteria |
| Desulfovibrionales sp. (HG3A.0266) | AHI | -0.054 | 0.003 | 0.014 | 3004 | unclassified | unclassified | unclassified | unclassified | Desulfovibrionales | Deltaproteobact eria | Proteobacteria |
| Eubacteriales sp. (HG3A.0334) | AHI | -0.053 | 0.004 | 0.014 | 3004 | unclassified | unclassified | unclassified | unclassified | Eubacteriales | Clostridia | Firmicutes |
| Eubacteriales sp. (HG3A.0498) | T90 | 0.05 | 0.004 | 0.014 | 3364 | unclassified | unclassified | unclassified | unclassified | Eubacteriales | Clostridia | Firmicutes |
| Eubacteriales sp. (HG3A.0557) | ODI | -0.049 | 0.005 | 0.014 | 3364 | unclassified | unclassified | unclassified | unclassified | Eubacteriales | Clostridia | Firmicutes |
| Eubacteriales sp. (HG3A.0781) | AHI | -0.054 | 0.004 | 0.014 | 3004 | unclassified | unclassified | unclassified | unclassified | Eubacteriales | Clostridia | Firmicutes |
| Eubacteriales sp. (HG3A.0873) | T90 | -0.05 | 0.004 | 0.014 | 3364 | unclassified | unclassified | unclassified | unclassified | Eubacteriales | Clostridia | Firmicutes |
| Eubacteriales sp. (HG3A.0910) | AHI | -0.054 | 0.003 | 0.014 | 3004 | unclassified | unclassified | unclassified | unclassified | Eubacteriales | Clostridia | Firmicutes |
| Eubacteriales sp. (HG3A.0964) | ODI | -0.049 | 0.005 | 0.014 | 3364 | unclassified | unclassified | unclassified | unclassified | Eubacteriales | Clostridia | Firmicutes |
| Eubacteriales sp. (HG3A.1154) | AHI | -0.054 | 0.004 | 0.014 | 3004 | unclassified | unclassified | unclassified | unclassified | Eubacteriales | Clostridia | Firmicutes |
| Firmicutes sp. (HG3A.1162) | ODI | -0.049 | 0.005 | 0.014 | 3364 | unclassified | unclassified | unclassified | unclassified | unclassified | unclassified | Firmicutes |
| Olsenella sp. AF21-51 (HG3A.0690) | AHI | -0.053 | 0.004 | 0.014 | 3004 | unclassified | Olsenella sp. AF21- 51 | Olsenella | Atopobiaceae | Coriobacteriales | Coriobacteriia | Actinobacteria |
| Oxalobacter sp. (HG3A.1218) | T90 | -0.05 | 0.004 | 0.014 | 3364 | unclassified | unclassified | Oxalobacter | Oxalobacteraceae | Burkholderiales | Betaproteobacte  ria | Proteobacteria |
| Candidatus Borkfalkia Candidatus Candidatus Candidatus Candidatus  ceftriaxoniphila (HG3A.0595) AHI -0.053 0.004 0.015 3004 unclassified Borkfalkia Borkfalkia Borkfalkiaceae Borkfalkiales Clostridia Firmicutes  ceftriaxoniphila | | | | | | | | | | | | |

Clostridia sp. (HG3A.0276) AHI -0.053 0.004 0.015 3004 unclassified unclassified unclassified unclassified unclassified Clostridia Firmicutes

| Clostridia sp. (HG3A.1486) | ODI | -0.049 | 0.005 | 0.015 | 3364 | unclassified | unclassified | unclassified | unclassified | unclassified | Clostridia | Firmicutes |
| --- | --- | --- | --- | --- | --- | --- | --- | --- | --- | --- | --- | --- |
| Clostridiaceae sp. (HG3A.0608) | ODI | -0.048 | 0.005 | 0.015 | 3364 | unclassified | unclassified | unclassified | Clostridiaceae | Eubacteriales | Clostridia | Firmicutes |
| Clostridium sp. AF34-13 (HG3A.0173) | AHI | -0.053 | 0.004 | 0.015 | 3004 | unclassified | Clostridium sp. AF34- 13 | Clostridium | Clostridiaceae | Eubacteriales | Clostridia | Firmicutes |
| Coprobacter fastidiosus  (HG3A.0182) | AHI | -0.053 | 0.004 | 0.015 | 3004 | unclassified | Coprobacter  fastidiosus | Coprobacter | Barnesiellaceae | Bacteroidales | Bacteroidia | Bacteroidetes |
| Coprococcus sp. AM27-12LB (HG3A.0687) | T90 | 0.049 | 0.004 | 0.015 | 3364 | unclassified | Coprococcus sp. AM27-12LB | Coprococcus | Lachnospiraceae | Eubacteriales | Clostridia | Firmicutes |
| Eubacteriales sp. (HG3A.0186) | ODI | -0.049 | 0.005 | 0.015 | 3364 | unclassified | unclassified | unclassified | unclassified | Eubacteriales | Clostridia | Firmicutes |
| Eubacteriales sp. (HG3A.0392) | ODI | -0.049 | 0.005 | 0.015 | 3364 | unclassified | unclassified | unclassified | unclassified | Eubacteriales | Clostridia | Firmicutes |
| Eubacteriales sp. (HG3A.0496) | T90 | -0.049 | 0.005 | 0.015 | 3364 | unclassified | unclassified | unclassified | unclassified | Eubacteriales | Clostridia | Firmicutes |
| Eubacteriales sp. (HG3A.0498) | AHI | 0.053 | 0.004 | 0.015 | 3004 | unclassified | unclassified | unclassified | unclassified | Eubacteriales | Clostridia | Firmicutes |
| Eubacteriales sp. (HG3A.0691) | AHI | -0.053 | 0.004 | 0.015 | 3004 | unclassified | unclassified | unclassified | unclassified | Eubacteriales | Clostridia | Firmicutes |
| Eubacteriales sp. (HG3A.0759) | AHI | -0.053 | 0.004 | 0.015 | 3004 | unclassified | unclassified | unclassified | unclassified | Eubacteriales | Clostridia | Firmicutes |
| Eubacteriales sp. (HG3A.0950) | ODI | -0.049 | 0.005 | 0.015 | 3364 | unclassified | unclassified | unclassified | unclassified | Eubacteriales | Clostridia | Firmicutes |
| Firmicutes sp. (HG3A.0464) | T90 | -0.05 | 0.004 | 0.015 | 3364 | unclassified | unclassified | unclassified | unclassified | unclassified | unclassified | Firmicutes |
| Lachnospiraceae sp. (HG3A.0855) | AHI | -0.053 | 0.004 | 0.015 | 3004 | unclassified | unclassified | unclassified | Lachnospiraceae | Eubacteriales | Clostridia | Firmicutes |
| Massilistercora timonensis (HG3A.0458) | AHI | -0.053 | 0.004 | 0.015 | 3004 | unclassified | Massilistercora timonensis | Massilistercora | unclassified | Eubacteriales | Clostridia | Firmicutes |
| Mogibacterium kristiansenii  (HG3A.0522) | AHI | 0.053 | 0.004 | 0.015 | 3004 | unclassified | Mogibacterium  kristiansenii | Mogibacterium | Clostridiales  Family XIII. | Eubacteriales | Clostridia | Firmicutes |
| Streptococcus sobrinus (HG3A.1366) | T90 | 0.049 | 0.005 | 0.015 | 3364 | unclassified | Streptococcus sobrinus | Streptococcus | Streptococcaceae | Lactobacillales | Bacilli | Firmicutes |
| Blautia producta (HG3A.0905) | T90 | 0.049 | 0.005 | 0.016 | 3364 | unclassified | Blautia producta | Blautia | Lachnospiraceae | Eubacteriales | Clostridia | Firmicutes |
| Candidatus Borkfalkiales sp. (HG3A.1397) | AHI | -0.052 | 0.004 | 0.016 | 3004 | unclassified | unclassified | unclassified | unclassified | Candidatus Borkfalkiales | Clostridia | Firmicutes |
| Clostridia sp. (HG3A.0767) | AHI | -0.053 | 0.004 | 0.016 | 3004 | unclassified | unclassified | unclassified | unclassified | unclassified | Clostridia | Firmicutes |
| Enterocloster clostridioformis (HG3A.0686) | AHI | 0.053 | 0.004 | 0.016 | 3004 | unclassified | Enterocloster clostridioformis | Enterocloster | Lachnospiraceae | Eubacteriales | Clostridia | Firmicutes |

| Eubacteriales sp. (HG3A.0432) | T90 | -0.049 | 0.005 | 0.016 | 3364 | unclassified | unclassified | unclassified | unclassified | Eubacteriales | Clostridia | Firmicutes |
| --- | --- | --- | --- | --- | --- | --- | --- | --- | --- | --- | --- | --- |
| Eubacteriales sp. (HG3A.0604) | ODI | -0.048 | 0.005 | 0.016 | 3364 | unclassified | unclassified | unclassified | unclassified | Eubacteriales | Clostridia | Firmicutes |
| Eubacteriales sp. (HG3A.0610) | T90 | 0.049 | 0.005 | 0.016 | 3364 | unclassified | unclassified | unclassified | unclassified | Eubacteriales | Clostridia | Firmicutes |
| Eubacteriales sp. (HG3A.0639) | T90 | -0.049 | 0.005 | 0.016 | 3364 | unclassified | unclassified | unclassified | unclassified | Eubacteriales | Clostridia | Firmicutes |
| Eubacteriales sp. (HG3A.0692) | AHI | 0.053 | 0.004 | 0.016 | 3004 | unclassified | unclassified | unclassified | unclassified | Eubacteriales | Clostridia | Firmicutes |
| Eubacteriales sp. (HG3A.0791) | AHI | -0.053 | 0.004 | 0.016 | 3004 | unclassified | unclassified | unclassified | unclassified | Eubacteriales | Clostridia | Firmicutes |
| Eubacteriales sp. (HG3A.0870) | T90 | -0.049 | 0.005 | 0.016 | 3364 | unclassified | unclassified | unclassified | unclassified | Eubacteriales | Clostridia | Firmicutes |
| Eubacteriales sp. (HG3A.0977) | AHI | -0.053 | 0.004 | 0.016 | 3004 | unclassified | unclassified | unclassified | unclassified | Eubacteriales | Clostridia | Firmicutes |
| Hydrogeniiclostidium mannosilyticum (HG3A.0294) | T90 | 0.049 | 0.005 | 0.016 | 3364 | unclassified | Hydrogeniiclostidium mannosilyticum | Hydrogeniiclostidiu m | Oscillospiraceae | Eubacteriales | Clostridia | Firmicutes |
| Mesosutterella multiformis (HG3A.0520) | AHI | -0.053 | 0.004 | 0.016 | 3004 | unclassified | Mesosutterella multiformis | Mesosutterella | Sutterellaceae | Burkholderiales | Betaproteobacte ria | Proteobacteria |
| Oscillospiraceae sp. (HG3A.1588) | T90 | -0.049 | 0.005 | 0.016 | 3364 | unclassified | unclassified | unclassified | Oscillospiraceae | Eubacteriales | Clostridia | Firmicutes |
| Scardovia wiggsiae (HG3A.1737) | ODI | 0.048 | 0.005 | 0.016 | 3364 | unclassified | Scardovia wiggsiae | Scardovia | Bifidobacteriaceae | Bifidobacteriales | Actinomycetia | Actinobacteria |
| Streptococcus mutans  (HG3A.0677) | AHI | 0.053 | 0.004 | 0.016 | 3004 | unclassified | Streptococcus  mutans | Streptococcus | Streptococcaceae | Lactobacillales | Bacilli | Firmicutes |
| Allisonella histaminiformans (HG3A.0332) | AHI | 0.052 | 0.005 | 0.017 | 3004 | unclassified | Allisonella histaminiformans | Allisonella | Veillonellaceae | Veillonellales | Negativicutes | Firmicutes |
| Bacteroidales sp. (HG3A.0789) | T90 | -0.049 | 0.005 | 0.017 | 3364 | unclassified | unclassified | unclassified | unclassified | Bacteroidales | Bacteroidia | Bacteroidetes |
| Candidatus Borkfalkiales sp. (HG3A.1329) | AHI | -0.052 | 0.005 | 0.017 | 3004 | unclassified | unclassified | unclassified | unclassified | Candidatus Borkfalkiales | Clostridia | Firmicutes |
| Coprococcus sp. AM27-12LB  (HG3A.0687) | AHI | 0.052 | 0.005 | 0.017 | 3004 | unclassified | Coprococcus sp.  AM27-12LB | Coprococcus | Lachnospiraceae | Eubacteriales | Clostridia | Firmicutes |
| Dorea phocaeensis (HG3A.0865) | ODI | 0.048 | 0.006 | 0.017 | 3364 | unclassified | Dorea phocaeensis | Dorea | Lachnospiraceae | Eubacteriales | Clostridia | Firmicutes |
| Eubacteriales sp. (HG3A.0221) | T90 | -0.049 | 0.005 | 0.017 | 3364 | unclassified | unclassified | unclassified | unclassified | Eubacteriales | Clostridia | Firmicutes |
| Eubacteriales sp. (HG3A.0349) | T90 | -0.049 | 0.005 | 0.017 | 3364 | unclassified | unclassified | unclassified | unclassified | Eubacteriales | Clostridia | Firmicutes |
| Eubacteriales sp. (HG3A.0386) | AHI | -0.052 | 0.005 | 0.017 | 3004 | unclassified | unclassified | unclassified | unclassified | Eubacteriales | Clostridia | Firmicutes |

Firmicutes sp. (HG3A.1124) AHI -0.052 0.005 0.017 3004 unclassified unclassified unclassified unclassified unclassified unclassified Firmicutes

Hydrogeniiclostidium

mannosilyticum (HG3A.0294) AHI 0.052 0.005 0.017 3004 unclassified

Hydrogeniiclostidium mannosilyticum

Hydrogeniiclostidiu

m Oscillospiraceae Eubacteriales Clostridia Firmicutes

Oscillospiraceae sp. (HG3A.0616) AHI

-0.052

0.005

0.017 3004 unclassified

unclassified

unclassified

Oscillospiraceae Eubacteriales

Clostridia

Firmicutes

Staphylococcus aureus

(HG3A.1538)

AHI 0.052 0.005 0.017 3004 unclassified Staphylococcus

aureus

Staphylococcus Staphylococcacea

e

Bacillales Bacilli Firmicutes

Streptococcus sobrinus

(HG3A.1366)

ODI

0.048

0.006

0.017 3364 unclassified

Streptococcus

sobrinus

Streptococcus Streptococcaceae Lactobacillales

Bacilli

Firmicutes

Atopobiaceae sp. (HG3A.0937) T90 0.048 0.005 0.018 3364 unclassified unclassified unclassified Atopobiaceae Coriobacteriales Coriobacteriia Actinobacteria

Bacteria sp. (HG3A.0218)

T90

-0.048

0.006

0.018 3364 unclassified

unclassified

unclassified

unclassified

unclassified

unclassified unclassified

Bacteroidales sp. (HG3A.0147) ODI -0.048 0.006 0.018 3364 unclassified unclassified unclassified unclassified Bacteroidales Bacteroidia Bacteroidetes

Eubacteriales sp. (HG3A.0148)

ODI

-0.047

0.006

0.018 3364 unclassified

unclassified

unclassified

unclassified

Eubacteriales

Clostridia

Firmicutes

Eubacteriales sp. (HG3A.0192) AHI -0.052 0.005 0.018 3004 unclassified unclassified unclassified unclassified Eubacteriales Clostridia Firmicutes

Eubacteriales sp. (HG3A.0192)

T90

-0.048

0.005

0.018 3364 unclassified

unclassified

unclassified

unclassified

Eubacteriales

Clostridia

Firmicutes

Eubacteriales sp. (HG3A.0482) T90 -0.048 0.005 0.018 3364 unclassified unclassified unclassified unclassified Eubacteriales Clostridia Firmicutes

Eubacteriales sp. (HG3A.0664)

AHI

0.052

0.005

0.018 3004 unclassified

unclassified

unclassified

unclassified

Eubacteriales

Clostridia

Firmicutes

Firmicutes sp. (HG3A.1345) ODI -0.047 0.006 0.018 3364 unclassified unclassified unclassified unclassified unclassified unclassified Firmicutes

Intestinimonas butyriciproducens

(HG3A.0187)

AHI

-0.052

0.005

0.018 3004 unclassified

Intestinimonas

butyriciproducens

Intestinimonas

unclassified

Eubacteriales

Clostridia

Firmicutes

Intestinimonas butyriciproducens

(HG3A.0187) ODI -0.047 0.006 0.018 3364 unclassified

Intestinimonas

butyriciproducens Intestinimonas unclassified Eubacteriales Clostridia Firmicutes

Phascolarctobacterium

succinatutens (HG3A.0315)

ODI

0.047

0.006

0.018 3364

unclassified

Phascolarctobacteriu Phascolarctobacteriu Acidaminococcac

m succinatutens m

eae

Acidaminococcales Negativicutes Firmicutes

Sutterella sp. KLE1602

(HG3A.0228)

ODI 0.047 0.006 0.018 3364 unclassified Sutterella sp.

KLE1602

Sutterella Sutterellaceae Burkholderiales Betaproteobacte

ria

Proteobacteria

Bacteroides intestinalis

(HG3A.0265)

ODI

-0.047

0.007

0.019 3364 unclassified

Bacteroides

intestinalis

Bacteroides

Bacteroidaceae Bacteroidales

Bacteroidia Bacteroidetes

Blautia producta (HG3A.0619) AHI 0.051 0.005 0.019 3004 unclassified Blautia producta Blautia Lachnospiraceae Eubacteriales Clostridia Firmicutes

| Clostridia sp. (HG3A.1139) | T90 | -0.048 | 0.006 | 0.019 | 3364 | unclassified | unclassified | unclassified | unclassified | unclassified | Clostridia | Firmicutes |
| --- | --- | --- | --- | --- | --- | --- | --- | --- | --- | --- | --- | --- |
| Clostridia sp. (HG3A.1148) | ODI | -0.047 | 0.007 | 0.019 | 3364 | unclassified | unclassified | unclassified | unclassified | unclassified | Clostridia | Firmicutes |
| Clostridia sp. (HG3A.1252) | ODI | -0.047 | 0.007 | 0.019 | 3364 | unclassified | unclassified | unclassified | unclassified | unclassified | Clostridia | Firmicutes |
| Eubacteriales sp. (HG3A.0369) | ODI | -0.047 | 0.007 | 0.019 | 3364 | unclassified | unclassified | unclassified | unclassified | Eubacteriales | Clostridia | Firmicutes |
| Eubacteriales sp. (HG3A.0490) | T90 | -0.048 | 0.006 | 0.019 | 3364 | unclassified | unclassified | unclassified | unclassified | Eubacteriales | Clostridia | Firmicutes |
| Eubacteriales sp. (HG3A.0956) | T90 | -0.048 | 0.006 | 0.019 | 3364 | unclassified | unclassified | unclassified | unclassified | Eubacteriales | Clostridia | Firmicutes |
| Eubacteriales sp. (HG3A.1026) | T90 | -0.048 | 0.006 | 0.019 | 3364 | unclassified | unclassified | unclassified | unclassified | Eubacteriales | Clostridia | Firmicutes |
| Eubacteriales sp. (HG3A.1094) | ODI | -0.047 | 0.007 | 0.019 | 3364 | unclassified | unclassified | unclassified | unclassified | Eubacteriales | Clostridia | Firmicutes |
| Eubacteriales sp. (HG3A.1187) | T90 | -0.048 | 0.006 | 0.019 | 3364 | unclassified | unclassified | unclassified | unclassified | Eubacteriales | Clostridia | Firmicutes |
| Faecalibacterium prausnitzii  (HG3A.0010) | T90 | 0.048 | 0.006 | 0.019 | 3364 | unclassified | Faecalibacterium  prausnitzii | Faecalibacterium | Oscillospiraceae | Eubacteriales | Clostridia | Firmicutes |
| Gemmiger formicilis (HG3A.0027) | T90 | 0.048 | 0.006 | 0.019 | 3364 | unclassified | Gemmiger formicilis | Gemmiger | unclassified | Eubacteriales | Clostridia | Firmicutes |
| Oscillospiraceae sp. (HG3A.0412) | T90 | -0.048 | 0.006 | 0.019 | 3364 | unclassified | unclassified | unclassified | Oscillospiraceae | Eubacteriales | Clostridia | Firmicutes |
| Prevotella colorans (HG3A.1470) | T90 | -0.048 | 0.006 | 0.019 | 3364 | unclassified | Prevotella colorans | Prevotella | Prevotellaceae | Bacteroidales | Bacteroidia | Bacteroidetes |
| Subdoligranulum sp. APC924/74  (HG3A.0015) | T90 | -0.048 | 0.006 | 0.019 | 3364 | unclassified | Subdoligranulum sp.  APC924/74 | Subdoligranulum | Oscillospiraceae | Eubacteriales | Clostridia | Firmicutes |
| Alistipes sp. An66 (HG3A.1535) | AHI | -0.051 | 0.005 | 0.02 | 3004 | unclassified | Alistipes sp. An66 | Alistipes | Rikenellaceae | Bacteroidales | Bacteroidia | Bacteroidetes |
| Clostridia sp. (HG3A.1058) | AHI | -0.051 | 0.006 | 0.02 | 3004 | unclassified | unclassified | unclassified | unclassified | unclassified | Clostridia | Firmicutes |
| Clostridia sp. (HG3A.1609) | T90 | -0.048 | 0.006 | 0.02 | 3364 | unclassified | unclassified | unclassified | unclassified | unclassified | Clostridia | Firmicutes |
| Desulfovibrio fairfieldensis  (HG3A.0529) | ODI | -0.047 | 0.007 | 0.02 | 3364 | unclassified | Desulfovibrio  fairfieldensis | Desulfovibrio | Desulfovibrionace  ae | Desulfovibrionales | Deltaproteobact  eria | Proteobacteria |
| Eubacteriales sp. (HG3A.0163) | AHI | -0.051 | 0.006 | 0.02 | 3004 | unclassified | unclassified | unclassified | unclassified | Eubacteriales | Clostridia | Firmicutes |
| Eubacteriales sp. (HG3A.0282) | T90 | -0.048 | 0.006 | 0.02 | 3364 | unclassified | unclassified | unclassified | unclassified | Eubacteriales | Clostridia | Firmicutes |
| Eubacteriales sp. (HG3A.0345) | ODI | -0.047 | 0.007 | 0.02 | 3364 | unclassified | unclassified | unclassified | unclassified | Eubacteriales | Clostridia | Firmicutes |
| Eubacteriales sp. (HG3A.0544) | T90 | -0.047 | 0.006 | 0.02 | 3364 | unclassified | unclassified | unclassified | unclassified | Eubacteriales | Clostridia | Firmicutes |

| Eubacteriales sp. (HG3A.0551) | AHI | -0.051 | 0.006 | 0.02 | 3004 | unclassified | unclassified | unclassified | unclassified | Eubacteriales | Clostridia | Firmicutes |
| --- | --- | --- | --- | --- | --- | --- | --- | --- | --- | --- | --- | --- |
| Eubacteriales sp. (HG3A.0644) | T90 | -0.048 | 0.006 | 0.02 | 3364 | unclassified | unclassified | unclassified | unclassified | Eubacteriales | Clostridia | Firmicutes |
| Eubacteriales sp. (HG3A.0663) | ODI | -0.047 | 0.007 | 0.02 | 3364 | unclassified | unclassified | unclassified | unclassified | Eubacteriales | Clostridia | Firmicutes |
| Eubacteriales sp. (HG3A.0664) | ODI | 0.047 | 0.007 | 0.02 | 3364 | unclassified | unclassified | unclassified | unclassified | Eubacteriales | Clostridia | Firmicutes |
| Eubacteriales sp. (HG3A.0754) | T90 | -0.048 | 0.006 | 0.02 | 3364 | unclassified | unclassified | unclassified | unclassified | Eubacteriales | Clostridia | Firmicutes |
| Eubacteriales sp. (HG3A.1103) | T90 | -0.048 | 0.006 | 0.02 | 3364 | unclassified | unclassified | unclassified | unclassified | Eubacteriales | Clostridia | Firmicutes |
| Eubacteriales sp. (HG3A.1239) | AHI | -0.051 | 0.006 | 0.02 | 3004 | unclassified | unclassified | unclassified | unclassified | Eubacteriales | Clostridia | Firmicutes |
| Faecalibacterium sp. (HG3A.0073) | ODI | -0.047 | 0.007 | 0.02 | 3364 | unclassified | unclassified | Faecalibacterium | Oscillospiraceae | Eubacteriales | Clostridia | Firmicutes |
| Lachnospiraceae sp. (HG3A.0172) | AHI | -0.051 | 0.006 | 0.02 | 3004 | unclassified | unclassified | unclassified | Lachnospiraceae | Eubacteriales | Clostridia | Firmicutes |
| Lachnospiraceae sp. (HG3A.0172) | ODI | -0.047 | 0.007 | 0.02 | 3364 | unclassified | unclassified | unclassified | Lachnospiraceae | Eubacteriales | Clostridia | Firmicutes |
| Lachnospiraceae sp. (HG3A.0257) | AHI | -0.051 | 0.006 | 0.02 | 3004 | unclassified | unclassified | unclassified | Lachnospiraceae | Eubacteriales | Clostridia | Firmicutes |
| Oscillospiraceae sp. (HG3A.0412) | AHI | -0.051 | 0.006 | 0.02 | 3004 | unclassified | unclassified | unclassified | Oscillospiraceae | Eubacteriales | Clostridia | Firmicutes |
| Pseudoflavonifractor sp. (HG3A.0844) | AHI | -0.051 | 0.005 | 0.02 | 3004 | unclassified | unclassified | Pseudoflavonifractor | Oscillospiraceae | Eubacteriales | Clostridia | Firmicutes |
| Alistipes sp. AF17-16  (HG3A.0150) | T90 | -0.047 | 0.007 | 0.021 | 3364 | unclassified | Alistipes sp. AF17-16 | Alistipes | Rikenellaceae | Bacteroidales | Bacteroidia | Bacteroidetes |
| Anaerotignum lactatifermentans (HG3A.0676) | T90 | 0.047 | 0.007 | 0.021 | 3364 | unclassified | Anaerotignum lactatifermentans | Anaerotignum | Lachnospiraceae | Eubacteriales | Clostridia | Firmicutes |
| Bacteria sp. (HG3A.0911) | ODI | -0.046 | 0.008 | 0.021 | 3364 | unclassified | unclassified | unclassified | unclassified | unclassified | unclassified | unclassified |
| Bacteroidales sp. (HG3A.1002) | AHI | -0.051 | 0.006 | 0.021 | 3004 | unclassified | unclassified | unclassified | unclassified | Bacteroidales | Bacteroidia | Bacteroidetes |
| Bacteroidales sp. (HG3A.1002) | ODI | -0.046 | 0.008 | 0.021 | 3364 | unclassified | unclassified | unclassified | unclassified | Bacteroidales | Bacteroidia | Bacteroidetes |
| Bacteroides caccae (HG3A.0066) | ODI | -0.047 | 0.007 | 0.021 | 3364 | unclassified | Bacteroides caccae | Bacteroides | Bacteroidaceae | Bacteroidales | Bacteroidia | Bacteroidetes |
| Clostridia sp. (HG3A.1076) | ODI | -0.046 | 0.008 | 0.021 | 3364 | unclassified | unclassified | unclassified | unclassified | unclassified | Clostridia | Firmicutes |
| Clostridia sp. (HG3A.1157) | AHI | -0.051 | 0.006 | 0.021 | 3004 | unclassified | unclassified | unclassified | unclassified | unclassified | Clostridia | Firmicutes |

Clostridium sp. AF34-13 (HG3A.0173)

| Clostridium sp. M62/1 (HG3A.0354) | AHI | 0.051 | 0.006 | 0.021 | 3004 | unclassified | Clostridium sp.  M62/1 | Clostridium | Clostridiaceae | Eubacteriales | Clostridia | Firmicutes |
| --- | --- | --- | --- | --- | --- | --- | --- | --- | --- | --- | --- | --- |
| Eggerthellaceae sp. (HG3A.0171) | T90 | -0.047 | 0.007 | 0.021 | 3364 | unclassified | unclassified | unclassified | Eggerthellaceae | Eggerthellales | Coriobacteriia | Actinobacteria |
| Eubacteriales sp. (HG3A.0062) | ODI | -0.047 | 0.007 | 0.021 | 3364 | unclassified | unclassified | unclassified | unclassified | Eubacteriales | Clostridia | Firmicutes |
| Eubacteriales sp. (HG3A.0136) | T90 | -0.047 | 0.007 | 0.021 | 3364 | unclassified | unclassified | unclassified | unclassified | Eubacteriales | Clostridia | Firmicutes |
| Eubacteriales sp. (HG3A.0289) | AHI | -0.051 | 0.006 | 0.021 | 3004 | unclassified | unclassified | unclassified | unclassified | Eubacteriales | Clostridia | Firmicutes |
| Eubacteriales sp. (HG3A.0320) | AHI | -0.051 | 0.006 | 0.021 | 3004 | unclassified | unclassified | unclassified | unclassified | Eubacteriales | Clostridia | Firmicutes |
| Eubacteriales sp. (HG3A.0476) | ODI | -0.046 | 0.008 | 0.021 | 3364 | unclassified | unclassified | unclassified | unclassified | Eubacteriales | Clostridia | Firmicutes |
| Eubacteriales sp. (HG3A.0482) | ODI | -0.047 | 0.007 | 0.021 | 3364 | unclassified | unclassified | unclassified | unclassified | Eubacteriales | Clostridia | Firmicutes |
| Eubacteriales sp. (HG3A.0535) | T90 | -0.047 | 0.007 | 0.021 | 3364 | unclassified | unclassified | unclassified | unclassified | Eubacteriales | Clostridia | Firmicutes |
| Eubacteriales sp. (HG3A.0618) | AHI | -0.051 | 0.006 | 0.021 | 3004 | unclassified | unclassified | unclassified | unclassified | Eubacteriales | Clostridia | Firmicutes |
| Eubacteriales sp. (HG3A.0643) | AHI | -0.051 | 0.006 | 0.021 | 3004 | unclassified | unclassified | unclassified | unclassified | Eubacteriales | Clostridia | Firmicutes |
| Eubacteriales sp. (HG3A.0820) | ODI | -0.047 | 0.007 | 0.021 | 3364 | unclassified | unclassified | unclassified | unclassified | Eubacteriales | Clostridia | Firmicutes |
| Eubacteriales sp. (HG3A.1136) | T90 | -0.047 | 0.007 | 0.021 | 3364 | unclassified | unclassified | unclassified | unclassified | Eubacteriales | Clostridia | Firmicutes |
| Eubacteriales sp. (HG3A.1154) | ODI | -0.047 | 0.007 | 0.021 | 3364 | unclassified | unclassified | unclassified | unclassified | Eubacteriales | Clostridia | Firmicutes |
| Eubacteriales sp. (HG3A.1227) | T90 | -0.047 | 0.006 | 0.021 | 3364 | unclassified | unclassified | unclassified | unclassified | Eubacteriales | Clostridia | Firmicutes |
| Firmicutes sp. (HG3A.0915) | T90 | -0.047 | 0.007 | 0.021 | 3364 | unclassified | unclassified | unclassified | unclassified | unclassified | unclassified | Firmicutes |
| Firmicutes sp. (HG3A.1048) | ODI | -0.046 | 0.008 | 0.021 | 3364 | unclassified | unclassified | unclassified | unclassified | unclassified | unclassified | Firmicutes |
| Lachnospiraceae sp. (HG3A.0855) | T90 | -0.047 | 0.006 | 0.021 | 3364 | unclassified | unclassified | unclassified | Lachnospiraceae | Eubacteriales | Clostridia | Firmicutes |
| Lachnospiraceae sp. (HG3A.1155) | ODI | -0.046 | 0.008 | 0.021 | 3364 | unclassified | unclassified | unclassified | Lachnospiraceae | Eubacteriales | Clostridia | Firmicutes |
| Lachnospiraceae sp. (HG3A.1525) | ODI | -0.046 | 0.008 | 0.021 | 3364 | unclassified | unclassified | unclassified | Lachnospiraceae | Eubacteriales | Clostridia | Firmicutes |
| Lactobacillus acidophilus (HG3A.0672) | T90 | -0.047 | 0.007 | 0.021 | 3364 | unclassified | Lactobacillus acidophilus | Lactobacillus | Lactobacillaceae | Lactobacillales | Bacilli | Firmicutes |

ODI -0.046 0.008 0.021 3364 unclassified Clostridium sp. AF34-

13

Clostridium Clostridiaceae Eubacteriales Clostridia Firmicutes

Levilactobacillus brevis (HG3A.1848)

| Prevotella sp. (HG3A.1009) | ODI | 0.047 | 0.007 | 0.021 | 3364 | unclassified | unclassified | Prevotella | Prevotellaceae | Bacteroidales | Bacteroidia | Bacteroidetes |
| --- | --- | --- | --- | --- | --- | --- | --- | --- | --- | --- | --- | --- |
| Barnesiellaceae sp. (HG3A.1180) | AHI | -0.05 | 0.006 | 0.022 | 3004 | unclassified | unclassified | unclassified | Barnesiellaceae | Bacteroidales | Bacteroidia | Bacteroidetes |
| Barnesiellaceae sp. (HG3A.1180) | ODI | -0.046 | 0.008 | 0.022 | 3364 | unclassified | unclassified | unclassified | Barnesiellaceae | Bacteroidales | Bacteroidia | Bacteroidetes |
| Catenibacterium mitsuokai  (HG3A.0775) | ODI | 0.046 | 0.008 | 0.022 | 3364 | unclassified | Catenibacterium  mitsuokai | Catenibacterium | Coprobacillaceae | Erysipelotrichales | Erysipelotrichia | Firmicutes |
| Clostridia sp. (HG3A.0564) | AHI | -0.05 | 0.006 | 0.022 | 3004 | unclassified | unclassified | unclassified | unclassified | unclassified | Clostridia | Firmicutes |
| Clostridia sp. (HG3A.0909) | ODI | -0.046 | 0.008 | 0.022 | 3364 | unclassified | unclassified | unclassified | unclassified | unclassified | Clostridia | Firmicutes |
| Clostridia sp. (HG3A.1137) | T90 | -0.047 | 0.007 | 0.022 | 3364 | unclassified | unclassified | unclassified | unclassified | unclassified | Clostridia | Firmicutes |
| Eisenbergiella tayi (HG3A.0355) | T90 | 0.047 | 0.007 | 0.022 | 3364 | unclassified | Eisenbergiella tayi | Eisenbergiella | Lachnospiraceae | Eubacteriales | Clostridia | Firmicutes |
| Eubacteriales sp. (HG3A.0138) | T90 | -0.047 | 0.007 | 0.022 | 3364 | unclassified | unclassified | unclassified | unclassified | Eubacteriales | Clostridia | Firmicutes |
| Eubacteriales sp. (HG3A.0328) | T90 | -0.047 | 0.007 | 0.022 | 3364 | unclassified | unclassified | unclassified | unclassified | Eubacteriales | Clostridia | Firmicutes |
| Eubacteriales sp. (HG3A.0821) | AHI | -0.05 | 0.006 | 0.022 | 3004 | unclassified | unclassified | unclassified | unclassified | Eubacteriales | Clostridia | Firmicutes |
| Eubacteriales sp. (HG3A.0972) | T90 | -0.047 | 0.007 | 0.022 | 3364 | unclassified | unclassified | unclassified | unclassified | Eubacteriales | Clostridia | Firmicutes |
| Lachnospiraceae sp. (HG3A.0899) | ODI | -0.046 | 0.008 | 0.022 | 3364 | unclassified | unclassified | unclassified | Lachnospiraceae | Eubacteriales | Clostridia | Firmicutes |
| Longicatena caecimuris  (HG3A.0571) | ODI | 0.046 | 0.008 | 0.022 | 3364 | unclassified | Longicatena  caecimuris | Longicatena | Erysipelotrichace  ae | Erysipelotrichales | Erysipelotrichia | Firmicutes |
| Clostridia sp. (HG3A.0661) | T90 | -0.046 | 0.008 | 0.023 | 3364 | unclassified | unclassified | unclassified | unclassified | unclassified | Clostridia | Firmicutes |
| Clostridia sp. (HG3A.0706) | AHI | -0.05 | 0.007 | 0.023 | 3004 | unclassified | unclassified | unclassified | unclassified | unclassified | Clostridia | Firmicutes |
| Clostridia sp. (HG3A.1039) | T90 | -0.047 | 0.007 | 0.023 | 3364 | unclassified | unclassified | unclassified | unclassified | unclassified | Clostridia | Firmicutes |
| Clostridia sp. (HG3A.1254) | ODI | -0.046 | 0.009 | 0.023 | 3364 | unclassified | unclassified | unclassified | unclassified | unclassified | Clostridia | Firmicutes |
| Clostridia sp. (HG3A.1427) | ODI | -0.046 | 0.009 | 0.023 | 3364 | unclassified | unclassified | unclassified | unclassified | unclassified | Clostridia | Firmicutes |
| Eubacteriales sp. (HG3A.0178) | AHI | -0.05 | 0.007 | 0.023 | 3004 | unclassified | unclassified | unclassified | unclassified | Eubacteriales | Clostridia | Firmicutes |
| Eubacteriales sp. (HG3A.0204) | ODI | -0.046 | 0.008 | 0.023 | 3364 | unclassified | unclassified | unclassified | unclassified | Eubacteriales | Clostridia | Firmicutes |

ODI 0.046 0.008 0.021 3364 unclassified Levilactobacillus

brevis

Levilactobacillus Lactobacillaceae Lactobacillales Bacilli Firmicutes

| Eubacteriales sp. (HG3A.0273) | ODI | -0.046 | 0.008 | 0.023 | 3364 | unclassified | unclassified | unclassified | unclassified | Eubacteriales | Clostridia | Firmicutes |
| --- | --- | --- | --- | --- | --- | --- | --- | --- | --- | --- | --- | --- |
| Eubacteriales sp. (HG3A.0473) | ODI | -0.046 | 0.008 | 0.023 | 3364 | unclassified | unclassified | unclassified | unclassified | Eubacteriales | Clostridia | Firmicutes |
| Eubacteriales sp. (HG3A.0514) | T90 | -0.047 | 0.007 | 0.023 | 3364 | unclassified | unclassified | unclassified | unclassified | Eubacteriales | Clostridia | Firmicutes |
| Eubacteriales sp. (HG3A.0593) | AHI | -0.05 | 0.007 | 0.023 | 3004 | unclassified | unclassified | unclassified | unclassified | Eubacteriales | Clostridia | Firmicutes |
| Eubacteriales sp. (HG3A.0762) | ODI | -0.046 | 0.009 | 0.023 | 3364 | unclassified | unclassified | unclassified | unclassified | Eubacteriales | Clostridia | Firmicutes |
| Eubacteriales sp. (HG3A.0832) | ODI | -0.046 | 0.009 | 0.023 | 3364 | unclassified | unclassified | unclassified | unclassified | Eubacteriales | Clostridia | Firmicutes |
| Eubacteriales sp. (HG3A.0978) | AHI | -0.05 | 0.007 | 0.023 | 3004 | unclassified | unclassified | unclassified | unclassified | Eubacteriales | Clostridia | Firmicutes |
| Eubacteriales sp. (HG3A.1219) | T90 | -0.046 | 0.008 | 0.023 | 3364 | unclassified | unclassified | unclassified | unclassified | Eubacteriales | Clostridia | Firmicutes |
| Eubacteriales sp. (HG3A.1250) | ODI | -0.046 | 0.009 | 0.023 | 3364 | unclassified | unclassified | unclassified | unclassified | Eubacteriales | Clostridia | Firmicutes |
| Eubacteriales sp. (HG3A.1269) | ODI | -0.046 | 0.009 | 0.023 | 3364 | unclassified | unclassified | unclassified | unclassified | Eubacteriales | Clostridia | Firmicutes |
| Firmicutes sp. (HG3A.1082) | AHI | -0.05 | 0.007 | 0.023 | 3004 | unclassified | unclassified | unclassified | unclassified | unclassified | unclassified | Firmicutes |
| Lachnospiraceae sp. (HG3A.0903) | ODI | -0.046 | 0.009 | 0.023 | 3364 | unclassified | unclassified | unclassified | Lachnospiraceae | Eubacteriales | Clostridia | Firmicutes |
| Lachnospiraceae sp. (HG3A.1190) | ODI | -0.046 | 0.009 | 0.023 | 3364 | unclassified | unclassified | unclassified | Lachnospiraceae | Eubacteriales | Clostridia | Firmicutes |

Lacticaseibacillus paracasei

subsp. paracasei (HG3A.0853)

T90

0.046

0.008

0.023

Lacticaseibacillus 3364 paracasei subsp.

paracasei

Lacticaseibacillus

paracasei

Lacticaseibacillus Lactobacillaceae

Lactobacillales

Bacilli

Firmicutes

Pseudoruminococcus massiliensis AHI -0.05 0.007 0.023 3004 unclassified Pseudoruminococcus Pseudoruminococcu

(HG3A.0346) massiliensis s

Oscillospiraceae Eubacteriales Clostridia Firmicutes

Tyzzerella nexilis (HG3A.0574)

ODI

0.046

0.008

0.023 3364 unclassified Tyzzerella nexilis

Tyzzerella

Lachnospiraceae Eubacteriales

Clostridia

Firmicutes

Actinomyces sp. ICM58

(HG3A.0410)

ODI 0.045 0.009 0.024 3364 unclassified Actinomyces sp.

ICM58

Actinomyces Actinomycetaceae Actinomycetales Actinomycetia Actinobacteria

Bacteroidales sp. (HG3A.0340)

T90

-0.046

0.008

0.024 3364 unclassified

unclassified

unclassified

unclassified

Bacteroidales

Bacteroidia Bacteroidetes

Clostridia sp. (HG3A.0519) ODI -0.045 0.009 0.024 3364 unclassified unclassified unclassified unclassified unclassified Clostridia Firmicutes

Clostridia sp. (HG3A.1192)

T90

-0.046

0.008

0.024 3364 unclassified

unclassified

unclassified

unclassified

unclassified

Clostridia

Firmicutes

Clostridiaceae sp. (HG3A.0491) T90 0.046 0.008 0.024 3364 unclassified unclassified unclassified Clostridiaceae Eubacteriales Clostridia Firmicutes

| Clostridium sp. AF15-31  (HG3A.0293) | AHI | -0.05 | 0.007 | 0.024 | 3004 | unclassified | Clostridium sp. AF15-  31 | Clostridium | Clostridiaceae | Eubacteriales | Clostridia | Firmicutes |
| --- | --- | --- | --- | --- | --- | --- | --- | --- | --- | --- | --- | --- |
| Eubacteriales sp. (HG3A.0325) | AHI | -0.05 | 0.007 | 0.024 | 3004 | unclassified | unclassified | unclassified | unclassified | Eubacteriales | Clostridia | Firmicutes |
| Eubacteriales sp. (HG3A.0543) | ODI | -0.045 | 0.009 | 0.024 | 3364 | unclassified | unclassified | unclassified | unclassified | Eubacteriales | Clostridia | Firmicutes |
| Eubacteriales sp. (HG3A.0547) | ODI | -0.045 | 0.009 | 0.024 | 3364 | unclassified | unclassified | unclassified | unclassified | Eubacteriales | Clostridia | Firmicutes |
| Eubacteriales sp. (HG3A.0614) | T90 | -0.046 | 0.008 | 0.024 | 3364 | unclassified | unclassified | unclassified | unclassified | Eubacteriales | Clostridia | Firmicutes |
| Eubacteriales sp. (HG3A.0791) | T90 | -0.046 | 0.008 | 0.024 | 3364 | unclassified | unclassified | unclassified | unclassified | Eubacteriales | Clostridia | Firmicutes |
| Eubacteriales sp. (HG3A.0887) | AHI | -0.05 | 0.007 | 0.024 | 3004 | unclassified | unclassified | unclassified | unclassified | Eubacteriales | Clostridia | Firmicutes |
| Eubacteriales sp. (HG3A.1439) | ODI | -0.045 | 0.009 | 0.024 | 3364 | unclassified | unclassified | unclassified | unclassified | Eubacteriales | Clostridia | Firmicutes |
| Eubacterium sp. (HG3A.0214) | T90 | 0.046 | 0.008 | 0.024 | 3364 | unclassified | unclassified | Eubacterium | Eubacteriaceae | Eubacteriales | Clostridia | Firmicutes |
| Firmicutes sp. (HG3A.1124) | T90 | -0.046 | 0.008 | 0.024 | 3364 | unclassified | unclassified | unclassified | unclassified | unclassified | unclassified | Firmicutes |
| Flavonifractor sp. An10 (HG3A.0495) | AHI | -0.05 | 0.007 | 0.024 | 3004 | unclassified | Flavonifractor sp.  An10 | Flavonifractor | Oscillospiraceae | Eubacteriales | Clostridia | Firmicutes |
| Oscillibacter sp. (HG3A.0243) | T90 | -0.046 | 0.008 | 0.024 | 3364 | unclassified | unclassified | Oscillibacter | Oscillospiraceae | Eubacteriales | Clostridia | Firmicutes |
| Oxalobacter formigenes (HG3A.1755) | T90 | -0.046 | 0.008 | 0.024 | 3364 | unclassified | Oxalobacter formigenes | Oxalobacter | Oxalobacteraceae | Burkholderiales | Betaproteobacte ria | Proteobacteria |
| Oxalobacter sp. (HG3A.1218) | ODI | -0.045 | 0.009 | 0.024 | 3364 | unclassified | unclassified | Oxalobacter | Oxalobacteraceae | Burkholderiales | Betaproteobacte  ria | Proteobacteria |
| Phocaeicola plebeius (HG3A.0423) | ODI | -0.045 | 0.009 | 0.024 | 3364 | unclassified | Phocaeicola plebeius | Phocaeicola | unclassified | Bacteroidales | Bacteroidia | Bacteroidetes |
| Ruminococcus sp. (HG3A.0126) | T90 | -0.046 | 0.008 | 0.024 | 3364 | unclassified | unclassified | Ruminococcus | Oscillospiraceae | Eubacteriales | Clostridia | Firmicutes |
| Sutterella wadsworthensis (HG3A.0143) | ODI | -0.045 | 0.009 | 0.024 | 3364 | unclassified | Sutterella wadsworthensis | Sutterella | Sutterellaceae | Burkholderiales | Betaproteobacte ria | Proteobacteria |
| Clostridia sp. (HG3A.1452) | AHI | -0.049 | 0.007 | 0.025 | 3004 | unclassified | unclassified | unclassified | unclassified | unclassified | Clostridia | Firmicutes |
| Eubacteriales sp. (HG3A.0511) | AHI | -0.049 | 0.007 | 0.025 | 3004 | unclassified | unclassified | unclassified | unclassified | Eubacteriales | Clostridia | Firmicutes |
| Eubacteriales sp. (HG3A.0701) | AHI | -0.049 | 0.007 | 0.025 | 3004 | unclassified | unclassified | unclassified | unclassified | Eubacteriales | Clostridia | Firmicutes |
| Eubacteriales sp. (HG3A.0858) | ODI | -0.045 | 0.01 | 0.025 | 3364 | unclassified | unclassified | unclassified | unclassified | Eubacteriales | Clostridia | Firmicutes |
| Eubacteriales sp. (HG3A.1199) | ODI | 0.045 | 0.01 | 0.025 | 3364 | unclassified | unclassified | unclassified | unclassified | Eubacteriales | Clostridia | Firmicutes |

(HG3A.0344)

| Eubacteriales sp. (HG3A.1256) | T90 | -0.046 | 0.008 | 0.025 | 3364 | unclassified | unclassified | unclassified | unclassified | Eubacteriales | Clostridia | Firmicutes |
| --- | --- | --- | --- | --- | --- | --- | --- | --- | --- | --- | --- | --- |
| Firmicutes sp. (HG3A.1195)  Roseburia sp. AM16-25 | ODI  AHI | -0.045  -0.049 | 0.01  0.007 | 0.025  0.025 | 3364  3004 | unclassified  unclassified | unclassified  Roseburia sp. AM16- | unclassified  Roseburia | unclassified  Lachnospiraceae | unclassified  Eubacteriales | unclassified  Clostridia | Firmicutes  Firmicutes |

Turicibacter sanguinis (HG3A.0274)

Akkermansia sp. BIOML-A59

(HG3A.0800)

AHI

-0.049

0.008

0.026 3004 unclassified

Akkermansia sp.

BIOML-A59

Akkermansia Akkermansiaceae Verrucomicrobiales Verrucomicrobi Verrucomicrobi

ae a

25

ODI -0.045 0.01 0.025 3364 unclassified Turicibacter

sanguinis

Turicibacter Turicibacteraceae Erysipelotrichales Erysipelotrichia Firmicutes

Anaeromassilibacillus sp. An250 (HG3A.0169)

Clostridia sp. (HG3A.0750)

T90

-0.046

0.009

0.026 3364 unclassified

unclassified

unclassified

unclassified

unclassified

Clostridia

Firmicutes

ODI -0.045 0.01 0.026 3364 unclassified Anaeromassilibacillu Anaeromassilibacillu Oscillospiraceae Eubacteriales Clostridia Firmicutes s sp. An250 s

Eubacteriales sp. (HG3A.0325) ODI -0.045 0.01 0.026 3364 unclassified unclassified unclassified unclassified Eubacteriales Clostridia Firmicutes

Eubacteriales sp. (HG3A.0751)

T90

-0.046

0.009

0.026 3364 unclassified

unclassified

unclassified

unclassified

Eubacteriales

Clostridia

Firmicutes

Eubacteriales sp. (HG3A.0902) T90 -0.045 0.009 0.026 3364 unclassified unclassified unclassified unclassified Eubacteriales Clostridia Firmicutes

Latilactobacillus curvatus

(HG3A.1505)

T90

0.046

0.009

0.026 3364 unclassified

Latilactobacillus

curvatus

Latilactobacillus Lactobacillaceae Lactobacillales

Bacilli

Firmicutes

Phascolarctobacterium Phascolarctobacteriu Phascolarctobacteriu Acidaminococcac

succinatutens (HG3A.0315) T90 0.046 0.009 0.026 3364 unclassified

m succinatutens m

eae Acidaminococcales Negativicutes Firmicutes

Ruminococcus sp. AM42-11

(HG3A.0002)

AHI

0.049

0.008

0.026 3004 unclassified

Ruminococcus sp.

AM42-11

Ruminococcus Oscillospiraceae Eubacteriales

Clostridia

Firmicutes

Scardovia wiggsiae (HG3A.1737) AHI 0.049 0.008 0.026 3004 unclassified Scardovia wiggsiae Scardovia Bifidobacteriaceae Bifidobacteriales Actinomycetia Actinobacteria

Senegalimassilia anaerobia (HG3A.0129)

Anaeroglobus geminatus (HG3A.1818)

Bacteroidales sp. (HG3A.0894)

T90

-0.045

0.009

0.027 3364 unclassified

unclassified

unclassified

unclassified

Bacteroidales

Bacteroidia Bacteroidetes

ODI 0.045 0.01 0.026 3364 unclassified Senegalimassilia

anaerobia

T90 0.045 0.009 0.027 3364 unclassified Anaeroglobus

geminatus

Senegalimassilia Coriobacteriaceae Coriobacteriales Coriobacteriia Actinobacteria Anaeroglobus Veillonellaceae Veillonellales Negativicutes Firmicutes

Candidatus Borkfalkiales sp. (HG3A.1284)

Eubacteriales sp. (HG3A.0189)

AHI

-0.049

0.008

0.027 3004 unclassified

unclassified

unclassified

unclassified

Eubacteriales

Clostridia

Firmicutes

AHI -0.049 0.008 0.027 3004 unclassified unclassified unclassified unclassified Candidatus

Borkfalkiales

Clostridia Firmicutes

Eubacteriales sp. (HG3A.0916) T90 -0.045 0.009 0.027 3364 unclassified unclassified unclassified unclassified Eubacteriales Clostridia Firmicutes

Faecalibacterium prausnitzii

(HG3A.0241)

T90

-0.045

0.009

0.027 3364 unclassified

Faecalibacterium

prausnitzii

Faecalibacterium Oscillospiraceae

Eubacteriales

Clostridia

Firmicutes

Parabacteroides goldsteinii (HG3A.0279)

| Bacteria sp. (HG3A.1543) | ODI | -0.044 | 0.011 | 0.028 | 3364 | unclassified | unclassified | unclassified | unclassified | unclassified | unclassified | unclassified |
| --- | --- | --- | --- | --- | --- | --- | --- | --- | --- | --- | --- | --- |
| Clostridia sp. (HG3A.0564) | ODI | -0.044 | 0.011 | 0.028 | 3364 | unclassified | unclassified | unclassified | unclassified | unclassified | Clostridia | Firmicutes |
| Clostridia sp. (HG3A.0946) | T90 | -0.045 | 0.01 | 0.028 | 3364 | unclassified | unclassified | unclassified | unclassified | unclassified | Clostridia | Firmicutes |
| Clostridia sp. (HG3A.1220) | T90 | -0.045 | 0.01 | 0.028 | 3364 | unclassified | unclassified | unclassified | unclassified | unclassified | Clostridia | Firmicutes |
| Clostridia sp. (HG3A.1247) | ODI | -0.044 | 0.011 | 0.028 | 3364 | unclassified | unclassified | unclassified | unclassified | unclassified | Clostridia | Firmicutes |
| Clostridium sp. AF15-31  (HG3A.0293) | ODI | -0.044 | 0.011 | 0.028 | 3364 | unclassified | Clostridium sp. AF15-  31 | Clostridium | Clostridiaceae | Eubacteriales | Clostridia | Firmicutes |
| Erysipelotrichales sp. (HG3A.0303) | T90 | -0.045 | 0.009 | 0.028 | 3364 | unclassified | unclassified | unclassified | unclassified | Erysipelotrichales | Erysipelotrichia | Firmicutes |
| Eubacteriales sp. (HG3A.0364) | ODI | -0.044 | 0.011 | 0.028 | 3364 | unclassified | unclassified | unclassified | unclassified | Eubacteriales | Clostridia | Firmicutes |
| Eubacteriales sp. (HG3A.0369) | T90 | -0.045 | 0.01 | 0.028 | 3364 | unclassified | unclassified | unclassified | unclassified | Eubacteriales | Clostridia | Firmicutes |
| Eubacteriales sp. (HG3A.0644) | ODI | -0.044 | 0.011 | 0.028 | 3364 | unclassified | unclassified | unclassified | unclassified | Eubacteriales | Clostridia | Firmicutes |
| Eubacteriales sp. (HG3A.0685) | ODI | -0.044 | 0.011 | 0.028 | 3364 | unclassified | unclassified | unclassified | unclassified | Eubacteriales | Clostridia | Firmicutes |
| Eubacteriales sp. (HG3A.1131) | T90 | -0.045 | 0.009 | 0.028 | 3364 | unclassified | unclassified | unclassified | unclassified | Eubacteriales | Clostridia | Firmicutes |
| Eubacteriales sp. (HG3A.1257) | ODI | -0.044 | 0.011 | 0.028 | 3364 | unclassified | unclassified | unclassified | unclassified | Eubacteriales | Clostridia | Firmicutes |
| Olsenella sp. AF21-51  (HG3A.0690) | T90 | -0.045 | 0.01 | 0.028 | 3364 | unclassified | Olsenella sp. AF21-  51 | Olsenella | Atopobiaceae | Coriobacteriales | Coriobacteriia | Actinobacteria |
| Bacteria sp. (HG3A.0708) | T90 | -0.045 | 0.01 | 0.029 | 3364 | unclassified | unclassified | unclassified | unclassified | unclassified | unclassified | unclassified |
| Bacteria sp. (HG3A.1545) | ODI | -0.044 | 0.011 | 0.029 | 3364 | unclassified | unclassified | unclassified | unclassified | unclassified | unclassified | unclassified |
| Clostridia sp. (HG3A.0011) | AHI | 0.048 | 0.009 | 0.029 | 3004 | unclassified | unclassified | unclassified | unclassified | unclassified | Clostridia | Firmicutes |
| Clostridia sp. (HG3A.0909) | T90 | -0.045 | 0.01 | 0.029 | 3364 | unclassified | unclassified | unclassified | unclassified | unclassified | Clostridia | Firmicutes |
| Eubacteriales sp. (HG3A.0588) | T90 | -0.045 | 0.01 | 0.029 | 3364 | unclassified | unclassified | unclassified | unclassified | Eubacteriales | Clostridia | Firmicutes |
| Eubacteriales sp. (HG3A.0832) | AHI | -0.048 | 0.009 | 0.029 | 3004 | unclassified | unclassified | unclassified | unclassified | Eubacteriales | Clostridia | Firmicutes |
| Eubacteriales sp. (HG3A.0973) | T90 | -0.045 | 0.01 | 0.029 | 3364 | unclassified | unclassified | unclassified | unclassified | Eubacteriales | Clostridia | Firmicutes |
| Eubacteriales sp. (HG3A.1067) | AHI | -0.048 | 0.009 | 0.029 | 3004 | unclassified | unclassified | unclassified | unclassified | Eubacteriales | Clostridia | Firmicutes |

AHI -0.049 0.008 0.027 3004 unclassified Parabacteroides

goldsteinii

Parabacteroides Tannerellaceae Bacteroidales Bacteroidia Bacteroidetes

| Mogibacterium kristiansenii  (HG3A.0522) | ODI | 0.044 | 0.012 | 0.029 | 3364 | unclassified | Mogibacterium  kristiansenii | Mogibacterium | Clostridiales  Family XIII. | Eubacteriales | Clostridia | Firmicutes |
| --- | --- | --- | --- | --- | --- | --- | --- | --- | --- | --- | --- | --- |
| Proteobacteria sp. (HG3A.0327) | ODI | -0.044 | 0.012 | 0.029 | 3364 | unclassified | unclassified | unclassified | unclassified | unclassified | unclassified | Proteobacteria |
| Bacteria sp. (HG3A.1349) | ODI | -0.044 | 0.012 | 0.03 | 3364 | unclassified | unclassified | unclassified | unclassified | unclassified | unclassified | unclassified |
| Dialister pneumosintes  (HG3A.1496) | AHI | 0.048 | 0.009 | 0.03 | 3004 | unclassified | Dialister  pneumosintes | Dialister | Veillonellaceae | Veillonellales | Negativicutes | Firmicutes |
| Erysipelotrichaceae sp. (HG3A.0867) | ODI | 0.044 | 0.012 | 0.03 | 3364 | unclassified | unclassified | unclassified | Erysipelotrichace ae | Erysipelotrichales | Erysipelotrichia | Firmicutes |
| Eubacteriales sp. (HG3A.0447) | ODI | -0.044 | 0.012 | 0.03 | 3364 | unclassified | unclassified | unclassified | unclassified | Eubacteriales | Clostridia | Firmicutes |
| Eubacterium sp. AF17-7 (HG3A.0165) | ODI | -0.044 | 0.012 | 0.03 | 3364 | unclassified | Eubacterium sp.  AF17-7 | Eubacterium | Eubacteriaceae | Eubacteriales | Clostridia | Firmicutes |
| Anaeroglobus geminatus  (HG3A.1818) | AHI | 0.048 | 0.009 | 0.031 | 3004 | unclassified | Anaeroglobus  geminatus | Anaeroglobus | Veillonellaceae | Veillonellales | Negativicutes | Firmicutes |
| Clostridia sp. (HG3A.1076) | AHI | -0.048 | 0.009 | 0.031 | 3004 | unclassified | unclassified | unclassified | unclassified | unclassified | Clostridia | Firmicutes |
| Clostridia sp. (HG3A.1410) | AHI | -0.048 | 0.009 | 0.031 | 3004 | unclassified | unclassified | unclassified | unclassified | unclassified | Clostridia | Firmicutes |
| Clostridium sp. OM07-9AC (HG3A.0448) | ODI | -0.044 | 0.012 | 0.031 | 3364 | unclassified | Clostridium sp. OM07-9AC | Clostridium | Clostridiaceae | Eubacteriales | Clostridia | Firmicutes |
| Eggerthellales sp. (HG3A.0848) | T90 | -0.044 | 0.011 | 0.031 | 3364 | unclassified | unclassified | unclassified | unclassified | Eggerthellales | Coriobacteriia | Actinobacteria |
| Enterococcus faecium (HG3A.0886) | T90 | 0.044 | 0.011 | 0.031 | 3364 | unclassified | Enterococcus faecium | Enterococcus | Enterococcaceae | Lactobacillales | Bacilli | Firmicutes |
| Eubacteriales sp. (HG3A.0273) | T90 | -0.044 | 0.011 | 0.031 | 3364 | unclassified | unclassified | unclassified | unclassified | Eubacteriales | Clostridia | Firmicutes |
| Eubacteriales sp. (HG3A.0820) | T90 | -0.044 | 0.011 | 0.031 | 3364 | unclassified | unclassified | unclassified | unclassified | Eubacteriales | Clostridia | Firmicutes |
| Eubacteriales sp. (HG3A.1102) | ODI | -0.044 | 0.012 | 0.031 | 3364 | unclassified | unclassified | unclassified | unclassified | Eubacteriales | Clostridia | Firmicutes |
| Evtepia gabavorous (HG3A.0114) | ODI | 0.043 | 0.012 | 0.031 | 3364 | unclassified | Evtepia gabavorous | Evtepia | unclassified | Eubacteriales | Clostridia | Firmicutes |
| Firmicutes sp. (HG3A.0769) | T90 | -0.044 | 0.011 | 0.031 | 3364 | unclassified | unclassified | unclassified | unclassified | unclassified | unclassified | Firmicutes |
| Longicatena caecimuris (HG3A.0571) | AHI | 0.048 | 0.009 | 0.031 | 3004 | unclassified | Longicatena caecimuris | Longicatena | Erysipelotrichace ae | Erysipelotrichales | Erysipelotrichia | Firmicutes |
| Allisonella histaminiformans (HG3A.0332) | ODI | 0.043 | 0.013 | 0.032 | 3364 | unclassified | Allisonella histaminiformans | Allisonella | Veillonellaceae | Veillonellales | Negativicutes | Firmicutes |

Bifidobacterium animalis subsp.

lactis (HG3A.0513)

T90

-0.044

0.011

0.032

Bifidobacterium 3364 animalis subsp.

lactis

Bifidobacterium

animalis

Bifidobacterium Bifidobacteriaceae Bifidobacteriales Actinomycetia Actinobacteria

| Blautia argi (HG3A.1450) | T90 | 0.044 | 0.011 | 0.032 | 3364 | unclassified | Blautia argi | Blautia | Lachnospiraceae | Eubacteriales | Clostridia | Firmicutes |
| --- | --- | --- | --- | --- | --- | --- | --- | --- | --- | --- | --- | --- |
| Butyricicoccus sp. OM04-18BH (HG3A.0139) | T90 | -0.044 | 0.011 | 0.032 | 3364 | unclassified | Butyricicoccus sp.  OM04-18BH | Butyricicoccus | Clostridiaceae | Eubacteriales | Clostridia | Firmicutes |
| Clostridium perfringens  (HG3A.0959) | T90 | 0.044 | 0.011 | 0.032 | 3364 | unclassified | Clostridium  perfringens | Clostridium | Clostridiaceae | Eubacteriales | Clostridia | Firmicutes |
| Erysipelatoclostridium ramosum (HG3A.0538) | T90 | 0.044 | 0.011 | 0.032 | 3364 | unclassified | Erysipelatoclostridiu m ramosum | Erysipelatoclostridiu m | Erysipelotrichace ae | Erysipelotrichales | Erysipelotrichia | Firmicutes |
| Erysipelotrichaceae sp.  (HG3A.0867) | T90 | 0.044 | 0.011 | 0.032 | 3364 | unclassified | unclassified | unclassified | Erysipelotrichace  ae | Erysipelotrichales | Erysipelotrichia | Firmicutes |
| Eubacteriales sp. (HG3A.0178) | T90 | -0.044 | 0.011 | 0.032 | 3364 | unclassified | unclassified | unclassified | unclassified | Eubacteriales | Clostridia | Firmicutes |
| Eubacteriales sp. (HG3A.0773) | T90 | -0.044 | 0.011 | 0.032 | 3364 | unclassified | unclassified | unclassified | unclassified | Eubacteriales | Clostridia | Firmicutes |
| Eubacteriales sp. (HG3A.0854) | T90 | 0.044 | 0.011 | 0.032 | 3364 | unclassified | unclassified | unclassified | unclassified | Eubacteriales | Clostridia | Firmicutes |
| Eubacteriales sp. (HG3A.0881) | T90 | -0.044 | 0.012 | 0.032 | 3364 | unclassified | unclassified | unclassified | unclassified | Eubacteriales | Clostridia | Firmicutes |
| Eubacteriales sp. (HG3A.1026) | AHI | -0.048 | 0.01 | 0.032 | 3004 | unclassified | unclassified | unclassified | unclassified | Eubacteriales | Clostridia | Firmicutes |
| Eubacteriales sp. (HG3A.1191) | ODI | -0.043 | 0.013 | 0.032 | 3364 | unclassified | unclassified | unclassified | unclassified | Eubacteriales | Clostridia | Firmicutes |
| Firmicutes sp. (HG3A.1195) | AHI | -0.048 | 0.01 | 0.032 | 3004 | unclassified | unclassified | unclassified | unclassified | unclassified | unclassified | Firmicutes |
| Roseburia faecis (HG3A.0058) | ODI | 0.043 | 0.013 | 0.032 | 3364 | unclassified | Roseburia faecis | Roseburia | Lachnospiraceae | Eubacteriales | Clostridia | Firmicutes |
| Sutterella sp. KLE1602 (HG3A.0228) | AHI | 0.047 | 0.01 | 0.032 | 3004 | unclassified | Sutterella sp. KLE1602 | Sutterella | Sutterellaceae | Burkholderiales | Betaproteobacte ria | Proteobacteria |
| Sutterellaceae sp. (HG3A.1122) | T90 | 0.044 | 0.011 | 0.032 | 3364 | unclassified | unclassified | unclassified | Sutterellaceae | Burkholderiales | Betaproteobacte  ria | Proteobacteria |
| Bacteroides nordii (HG3A.0290) | AHI | -0.047 | 0.01 | 0.033 | 3004 | unclassified | Bacteroides nordii | Bacteroides | Bacteroidaceae | Bacteroidales | Bacteroidia | Bacteroidetes |
| Clostridia sp. (HG3A.1192) | ODI | -0.043 | 0.013 | 0.033 | 3364 | unclassified | unclassified | unclassified | unclassified | unclassified | Clostridia | Firmicutes |
| Clostridia sp. (HG3A.1220) | ODI | -0.043 | 0.013 | 0.033 | 3364 | unclassified | unclassified | unclassified | unclassified | unclassified | Clostridia | Firmicutes |
| Dorea sp. AF24-7LB (HG3A.0086) | AHI | 0.047 | 0.01 | 0.033 | 3004 | unclassified | Dorea sp. AF24-7LB | Dorea | Lachnospiraceae | Eubacteriales | Clostridia | Firmicutes |
| Eubacteriales sp. (HG3A.0230) | T90 | -0.044 | 0.012 | 0.033 | 3364 | unclassified | unclassified | unclassified | unclassified | Eubacteriales | Clostridia | Firmicutes |
| Eubacteriales sp. (HG3A.0935) | ODI | -0.043 | 0.013 | 0.033 | 3364 | unclassified | unclassified | unclassified | unclassified | Eubacteriales | Clostridia | Firmicutes |
| Eubacteriales sp. (HG3A.1292) | ODI | -0.043 | 0.013 | 0.033 | 3364 | unclassified | unclassified | unclassified | unclassified | Eubacteriales | Clostridia | Firmicutes |

| Firmicutes sp. (HG3A.0769) | AHI | -0.047 | 0.01 | 0.033 | 3004 | unclassified | unclassified | unclassified | unclassified | unclassified | unclassified | Firmicutes |
| --- | --- | --- | --- | --- | --- | --- | --- | --- | --- | --- | --- | --- |
| Lachnospiraceae sp. (HG3A.0217) | T90 | -0.044 | 0.012 | 0.033 | 3364 | unclassified | unclassified | unclassified | Lachnospiraceae | Eubacteriales | Clostridia | Firmicutes |
| Roseburia sp. AM16-25  (HG3A.0344) | ODI | -0.043 | 0.014 | 0.033 | 3364 | unclassified | Roseburia sp. AM16-  25 | Roseburia | Lachnospiraceae | Eubacteriales | Clostridia | Firmicutes |
| Bacteria sp. (HG3A.0459) | T90 | -0.044 | 0.012 | 0.034 | 3364 | unclassified | unclassified | unclassified | unclassified | unclassified | unclassified | unclassified |
| Clostridia sp. (HG3A.0885) | T90 | -0.044 | 0.012 | 0.034 | 3364 | unclassified | unclassified | unclassified | unclassified | unclassified | Clostridia | Firmicutes |
| Hungatella hathewayi (HG3A.0455) | ODI | 0.043 | 0.014 | 0.034 | 3364 | unclassified | Hungatella hathewayi | Hungatella | Clostridiaceae | Eubacteriales | Clostridia | Firmicutes |
| Lachnospiraceae sp. (HG3A.0172) | T90 | -0.044 | 0.012 | 0.034 | 3364 | unclassified | unclassified | unclassified | Lachnospiraceae | Eubacteriales | Clostridia | Firmicutes |
| Bacteroides nordii (HG3A.0290) | T90 | -0.043 | 0.013 | 0.035 | 3364 | unclassified | Bacteroides nordii | Bacteroides | Bacteroidaceae | Bacteroidales | Bacteroidia | Bacteroidetes |
| Blautia producta (HG3A.0905) | AHI | 0.047 | 0.011 | 0.035 | 3004 | unclassified | Blautia producta | Blautia | Lachnospiraceae | Eubacteriales | Clostridia | Firmicutes |
| Clostridia sp. (HG3A.0996) | T90 | -0.043 | 0.012 | 0.035 | 3364 | unclassified | unclassified | unclassified | unclassified | unclassified | Clostridia | Firmicutes |
| Desulfovibrionales sp.  (HG3A.0727) | ODI | 0.043 | 0.014 | 0.035 | 3364 | unclassified | unclassified | unclassified | unclassified | Desulfovibrionales | Deltaproteobact  eria | Proteobacteria |
| Eubacteriales sp. (HG3A.0323) | AHI | -0.047 | 0.011 | 0.035 | 3004 | unclassified | unclassified | unclassified | unclassified | Eubacteriales | Clostridia | Firmicutes |
| Eubacteriales sp. (HG3A.0589) | AHI | -0.047 | 0.011 | 0.035 | 3004 | unclassified | unclassified | unclassified | unclassified | Eubacteriales | Clostridia | Firmicutes |
| Eubacteriales sp. (HG3A.1003) | ODI | -0.043 | 0.014 | 0.035 | 3364 | unclassified | unclassified | unclassified | unclassified | Eubacteriales | Clostridia | Firmicutes |
| Eubacteriales sp. (HG3A.1305) | AHI | -0.047 | 0.011 | 0.035 | 3004 | unclassified | unclassified | unclassified | unclassified | Eubacteriales | Clostridia | Firmicutes |
| Eubacterium sp. AF22-8LB (HG3A.0838) | AHI | 0.047 | 0.011 | 0.035 | 3004 | unclassified | Eubacterium sp.  AF22-8LB | Eubacterium | Eubacteriaceae | Eubacteriales | Clostridia | Firmicutes |
| Longicatena caecimuris  (HG3A.0571) | T90 | 0.043 | 0.013 | 0.035 | 3364 | unclassified | Longicatena  caecimuris | Longicatena | Erysipelotrichace  ae | Erysipelotrichales | Erysipelotrichia | Firmicutes |
| Oscillospiraceae sp. (HG3A.0805) | T90 | -0.043 | 0.013 | 0.035 | 3364 | unclassified | unclassified | unclassified | Oscillospiraceae | Eubacteriales | Clostridia | Firmicutes |
| Peptostreptococcaceae sp.  (HG3A.0200) | T90 | -0.043 | 0.013 | 0.035 | 3364 | unclassified | unclassified | unclassified | Peptostreptococca  ceae | Eubacteriales | Clostridia | Firmicutes |
| Alistipes ihumii (HG3A.0106) | ODI | -0.042 | 0.015 | 0.036 | 3364 | unclassified | Alistipes ihumii | Alistipes | Rikenellaceae | Bacteroidales | Bacteroidia | Bacteroidetes |
| Bacteroidales sp. (HG3A.0894) | ODI | -0.042 | 0.015 | 0.036 | 3364 | unclassified | unclassified | unclassified | unclassified | Bacteroidales | Bacteroidia | Bacteroidetes |
| Clostridia sp. (HG3A.0933) | AHI | -0.047 | 0.011 | 0.036 | 3004 | unclassified | unclassified | unclassified | unclassified | unclassified | Clostridia | Firmicutes |

| Clostridia sp. (HG3A.1298) | ODI | -0.042 | 0.015 | 0.036 | 3364 | unclassified | unclassified | unclassified | unclassified | unclassified | Clostridia | Firmicutes |
| --- | --- | --- | --- | --- | --- | --- | --- | --- | --- | --- | --- | --- |
| Coprococcus sp. OM04-5BH (HG3A.1028) | T90 | -0.043 | 0.013 | 0.036 | 3364 | unclassified | Coprococcus sp. OM04-5BH | Coprococcus | Lachnospiraceae | Eubacteriales | Clostridia | Firmicutes |
| Eubacteriales sp. (HG3A.0390) | T90 | -0.043 | 0.013 | 0.036 | 3364 | unclassified | unclassified | unclassified | unclassified | Eubacteriales | Clostridia | Firmicutes |
| Eubacteriales sp. (HG3A.0939) | AHI | -0.047 | 0.011 | 0.036 | 3004 | unclassified | unclassified | unclassified | unclassified | Eubacteriales | Clostridia | Firmicutes |
| Eubacteriales sp. (HG3A.1226) | ODI | -0.042 | 0.015 | 0.036 | 3364 | unclassified | unclassified | unclassified | unclassified | Eubacteriales | Clostridia | Firmicutes |
| Faecalibacterium prausnitzii (HG3A.0010) | ODI | 0.042 | 0.015 | 0.036 | 3364 | unclassified | Faecalibacterium prausnitzii | Faecalibacterium | Oscillospiraceae | Eubacteriales | Clostridia | Firmicutes |
| Firmicutes sp. (HG3A.1050) | ODI | -0.042 | 0.015 | 0.036 | 3364 | unclassified | unclassified | unclassified | unclassified | unclassified | unclassified | Firmicutes |
| Lachnospiraceae sp. (HG3A.0625) | T90 | 0.043 | 0.013 | 0.036 | 3364 | unclassified | unclassified | unclassified | Lachnospiraceae | Eubacteriales | Clostridia | Firmicutes |
| Oscillospiraceae sp. (HG3A.0146) | ODI | -0.042 | 0.015 | 0.036 | 3364 | unclassified | unclassified | unclassified | Oscillospiraceae | Eubacteriales | Clostridia | Firmicutes |
| Acidaminococcus intestini (HG3A.0407) | T90 | 0.043 | 0.014 | 0.037 | 3364 | unclassified | Acidaminococcus intestini | Acidaminococcus | Acidaminococcac eae | Acidaminococcales | Negativicutes | Firmicutes |
| Alistipes sp. (HG3A.1385) | ODI | -0.042 | 0.015 | 0.037 | 3364 | unclassified | unclassified | Alistipes | Rikenellaceae | Bacteroidales | Bacteroidia | Bacteroidetes |
| Bacteria sp. (HG3A.1553) | T90 | -0.043 | 0.014 | 0.037 | 3364 | unclassified | unclassified | unclassified | unclassified | unclassified | unclassified | unclassified |
| Clostridia sp. (HG3A.1108) | AHI | -0.046 | 0.012 | 0.037 | 3004 | unclassified | unclassified | unclassified | unclassified | unclassified | Clostridia | Firmicutes |
| Clostridia sp. (HG3A.1193) | T90 | -0.043 | 0.013 | 0.037 | 3364 | unclassified | unclassified | unclassified | unclassified | unclassified | Clostridia | Firmicutes |
| Clostridia sp. (HG3A.1625) | ODI | -0.042 | 0.015 | 0.037 | 3364 | unclassified | unclassified | unclassified | unclassified | unclassified | Clostridia | Firmicutes |
| Eubacteriales sp. (HG3A.0288) | T90 | -0.043 | 0.014 | 0.037 | 3364 | unclassified | unclassified | unclassified | unclassified | Eubacteriales | Clostridia | Firmicutes |
| Eubacteriales sp. (HG3A.0302) | ODI | -0.042 | 0.015 | 0.037 | 3364 | unclassified | unclassified | unclassified | unclassified | Eubacteriales | Clostridia | Firmicutes |
| Eubacteriales sp. (HG3A.0328) | AHI | -0.046 | 0.012 | 0.037 | 3004 | unclassified | unclassified | unclassified | unclassified | Eubacteriales | Clostridia | Firmicutes |
| Eubacteriales sp. (HG3A.0390) | AHI | -0.046 | 0.012 | 0.037 | 3004 | unclassified | unclassified | unclassified | unclassified | Eubacteriales | Clostridia | Firmicutes |
| Eubacteriales sp. (HG3A.1078) | AHI | -0.046 | 0.012 | 0.037 | 3004 | unclassified | unclassified | unclassified | unclassified | Eubacteriales | Clostridia | Firmicutes |
| Eubacteriales sp. (HG3A.1109) | T90 | -0.043 | 0.014 | 0.037 | 3364 | unclassified | unclassified | unclassified | unclassified | Eubacteriales | Clostridia | Firmicutes |
| Eubacteriales sp. (HG3A.1126) | ODI | -0.042 | 0.015 | 0.037 | 3364 | unclassified | unclassified | unclassified | unclassified | Eubacteriales | Clostridia | Firmicutes |

| Firmicutes sp. (HG3A.0948) | AHI | -0.046 | 0.012 | 0.037 | 3004 | unclassified | unclassified | unclassified | unclassified | unclassified | unclassified | Firmicutes |
| --- | --- | --- | --- | --- | --- | --- | --- | --- | --- | --- | --- | --- |
| Lachnospiraceae sp. (HG3A.1641) | AHI | 0.046 | 0.012 | 0.037 | 3004 | unclassified | unclassified | unclassified | Lachnospiraceae | Eubacteriales | Clostridia | Firmicutes |

Oxalobacter sp. (HG3A.1097) T90 -0.043 0.013 0.037 3364 unclassified unclassified Oxalobacter Oxalobacteraceae Burkholderiales Betaproteobacte

ria

Proteobacteria

Parabacteroides gordonii

(HG3A.0989)

T90

-0.043

0.014

0.037 3364 unclassified

Parabacteroides

gordonii

Parabacteroides Tannerellaceae Bacteroidales

Bacteroidia Bacteroidetes

Streptococcus gallolyticus subsp. gallolyticus (HG3A.1651)

ODI 0.042 0.015 0.037 3364

Streptococcus

gallolyticus subsp. gallolyticus

Streptococcus gallolyticus

Streptococcus Streptococcaceae Lactobacillales Bacilli Firmicutes

Bacteria sp. (HG3A.0500) AHI -0.046 0.012 0.038 3004 unclassified unclassified unclassified unclassified unclassified unclassified unclassified Clostridia sp. (HG3A.0845) ODI -0.042 0.016 0.038 3364 unclassified unclassified unclassified unclassified unclassified Clostridia Firmicutes

Clostridia sp. (HG3A.1262)

AHI

-0.046

0.012

0.038 3004 unclassified

unclassified

unclassified

unclassified

unclassified

Clostridia

Firmicutes

Erysipelatoclostridium ramosum

AHI 0.046 0.012 0.038 3004 unclassified Erysipelatoclostridiu

Erysipelatoclostridiu Erysipelotrichace

Erysipelotrichales Erysipelotrichia Firmicutes

(HG3A.0538) m ramosum m ae

Eubacteriaceae sp. (HG3A.0591)

T90

0.043

0.014

0.038 3364 unclassified

unclassified

unclassified

Eubacteriaceae Eubacteriales

Clostridia

Firmicutes

Eubacteriales sp. (HG3A.0473) T90 -0.043 0.014 0.038 3364 unclassified unclassified unclassified unclassified Eubacteriales Clostridia Firmicutes

Eubacteriales sp. (HG3A.0514)

AHI

-0.046

0.012

0.038 3004 unclassified

unclassified

unclassified

unclassified

Eubacteriales

Clostridia

Firmicutes

Eubacteriales sp. (HG3A.1177) T90 -0.043 0.014 0.038 3364 unclassified unclassified unclassified unclassified Eubacteriales Clostridia Firmicutes

Eubacteriales sp. (HG3A.1256)

ODI

-0.042

0.016

0.038 3364 unclassified

unclassified

unclassified

unclassified

Eubacteriales

Clostridia

Firmicutes

Lachnospiraceae sp. (HG3A.0903) AHI -0.046 0.012 0.038 3004 unclassified unclassified unclassified Lachnospiraceae Eubacteriales Clostridia Firmicutes

Oscillospiraceae sp. (HG3A.0665) AHI

-0.046

0.012

0.038 3004 unclassified

unclassified

unclassified

Oscillospiraceae Eubacteriales

Clostridia

Firmicutes

Peptostreptococcaceae sp.

(HG3A.0200)

ODI -0.042 0.016 0.038 3364 unclassified unclassified unclassified Peptostreptococca

ceae

Eubacteriales Clostridia Firmicutes

Clostridia sp. (HG3A.0841)

T90

-0.043

0.014

0.039 3364 unclassified

unclassified

unclassified

unclassified

unclassified

Clostridia

Firmicutes

Clostridia sp. (HG3A.1417) ODI -0.042 0.016 0.039 3364 unclassified unclassified unclassified unclassified unclassified Clostridia Firmicutes

Collinsella phocaeensis

(HG3A.1340)

T90

0.043

0.014

0.039 3364 unclassified

Collinsella

phocaeensis

Collinsella

Coriobacteriaceae Coriobacteriales Coriobacteriia Actinobacteria

Eubacteriales sp. (HG3A.0935) AHI -0.046 0.013 0.039 3004 unclassified unclassified unclassified unclassified Eubacteriales Clostridia Firmicutes

| Eubacteriales sp. (HG3A.1030) | ODI | -0.042 | 0.016 | 0.039 | 3364 | unclassified | unclassified | unclassified | unclassified | Eubacteriales | Clostridia | Firmicutes |
| --- | --- | --- | --- | --- | --- | --- | --- | --- | --- | --- | --- | --- |
| Eubacteriales sp. (HG3A.1094) | AHI | -0.046 | 0.013 | 0.039 | 3004 | unclassified | unclassified | unclassified | unclassified | Eubacteriales | Clostridia | Firmicutes |
| Eubacteriales sp. (HG3A.1243) | ODI | -0.042 | 0.016 | 0.039 | 3364 | unclassified | unclassified | unclassified | unclassified | Eubacteriales | Clostridia | Firmicutes |
| Oscillospiraceae sp. (HG3A.0576) | AHI | -0.046 | 0.013 | 0.039 | 3004 | unclassified | unclassified | unclassified | Oscillospiraceae | Eubacteriales | Clostridia | Firmicutes |
| Oscillospiraceae sp. (HG3A.0739) | AHI | -0.046 | 0.013 | 0.039 | 3004 | unclassified | unclassified | unclassified | Oscillospiraceae | Eubacteriales | Clostridia | Firmicutes |
| Oscillospiraceae sp. (HG3A.0966) | AHI | -0.046 | 0.013 | 0.039 | 3004 | unclassified | unclassified | unclassified | Oscillospiraceae | Eubacteriales | Clostridia | Firmicutes |
| Succinatimonas hippei (HG3A.1322) | ODI | 0.042 | 0.016 | 0.039 | 3364 | unclassified | Succinatimonas hippei | Succinatimonas | Succinivibrionace ae | Aeromonadales | Gammaproteob acteria | Proteobacteria |
| Bacteroides caccae (HG3A.0066) | AHI | -0.046 | 0.013 | 0.04 | 3004 | unclassified | Bacteroides caccae | Bacteroides | Bacteroidaceae | Bacteroidales | Bacteroidia | Bacteroidetes |
| Clostridia sp. (HG3A.0885) | AHI | -0.046 | 0.013 | 0.04 | 3004 | unclassified | unclassified | unclassified | unclassified | unclassified | Clostridia | Firmicutes |
| Clostridia sp. (HG3A.1035) | T90 | -0.042 | 0.015 | 0.04 | 3364 | unclassified | unclassified | unclassified | unclassified | unclassified | Clostridia | Firmicutes |
| Clostridia sp. (HG3A.1127) | AHI | -0.046 | 0.013 | 0.04 | 3004 | unclassified | unclassified | unclassified | unclassified | unclassified | Clostridia | Firmicutes |
| Coprococcus catus (HG3A.0037) | AHI | 0.046 | 0.013 | 0.04 | 3004 | unclassified | Coprococcus catus | Coprococcus | Lachnospiraceae | Eubacteriales | Clostridia | Firmicutes |
| Eubacteriales sp. (HG3A.0663) | T90 | -0.042 | 0.015 | 0.04 | 3364 | unclassified | unclassified | unclassified | unclassified | Eubacteriales | Clostridia | Firmicutes |
| Eubacteriales sp. (HG3A.0744) | AHI | -0.046 | 0.013 | 0.04 | 3004 | unclassified | unclassified | unclassified | unclassified | Eubacteriales | Clostridia | Firmicutes |
| Eubacteriales sp. (HG3A.0902) | AHI | -0.046 | 0.013 | 0.04 | 3004 | unclassified | unclassified | unclassified | unclassified | Eubacteriales | Clostridia | Firmicutes |
| Faecalibacterium prausnitzii  (HG3A.0241) | AHI | -0.046 | 0.013 | 0.04 | 3004 | unclassified | Faecalibacterium  prausnitzii | Faecalibacterium | Oscillospiraceae | Eubacteriales | Clostridia | Firmicutes |
| Firmicutes sp. (HG3A.1345) | T90 | -0.042 | 0.015 | 0.04 | 3364 | unclassified | unclassified | unclassified | unclassified | unclassified | unclassified | Firmicutes |
| Ruminococcus sp. AF17-22AC  (HG3A.0208) | T90 | 0.042 | 0.015 | 0.04 | 3364 | unclassified | Ruminococcus sp.  AF17-22AC | Ruminococcus | Oscillospiraceae | Eubacteriales | Clostridia | Firmicutes |
| Streptococcus agalactiae (HG3A.1733) | ODI | 0.042 | 0.017 | 0.04 | 3364 | unclassified | Streptococcus agalactiae | Streptococcus | Streptococcaceae | Lactobacillales | Bacilli | Firmicutes |
| Agathobaculum desmolans  (HG3A.1429) | T90 | 0.042 | 0.016 | 0.041 | 3364 | unclassified | Agathobaculum  desmolans | Agathobaculum | Oscillospiraceae | Eubacteriales | Clostridia | Firmicutes |
| Anaerotignum lactatifermentans (HG3A.0676) | ODI | 0.041 | 0.017 | 0.041 | 3364 | unclassified | Anaerotignum lactatifermentans | Anaerotignum | Lachnospiraceae | Eubacteriales | Clostridia | Firmicutes |

| Bacteria sp. (HG3A.1096) | AHI | -0.045 | 0.014 | 0.041 | 3004 | unclassified | unclassified | unclassified | unclassified | unclassified | unclassified | unclassified |
| --- | --- | --- | --- | --- | --- | --- | --- | --- | --- | --- | --- | --- |
| Clostridia sp. (HG3A.1452) | ODI | -0.041 | 0.017 | 0.041 | 3364 | unclassified | unclassified | unclassified | unclassified | unclassified | Clostridia | Firmicutes |
| Clostridia sp. (HG3A.1493) | AHI | -0.045 | 0.014 | 0.041 | 3004 | unclassified | unclassified | unclassified | unclassified | unclassified | Clostridia | Firmicutes |
| Eubacteriales sp. (HG3A.0186) | AHI | -0.045 | 0.014 | 0.041 | 3004 | unclassified | unclassified | unclassified | unclassified | Eubacteriales | Clostridia | Firmicutes |
| Eubacteriales sp. (HG3A.0685) | AHI | -0.045 | 0.014 | 0.041 | 3004 | unclassified | unclassified | unclassified | unclassified | Eubacteriales | Clostridia | Firmicutes |
| Eubacteriales sp. (HG3A.1285) | ODI | -0.041 | 0.017 | 0.041 | 3364 | unclassified | unclassified | unclassified | unclassified | Eubacteriales | Clostridia | Firmicutes |
| Eubacteriales sp. (HG3A.1473) | T90 | -0.042 | 0.016 | 0.041 | 3364 | unclassified | unclassified | unclassified | unclassified | Eubacteriales | Clostridia | Firmicutes |
| Holdemanella sp. (HG3A.0366) | T90 | 0.042 | 0.015 | 0.041 | 3364 | unclassified | unclassified | Holdemanella | Erysipelotrichace ae | Erysipelotrichales | Erysipelotrichia | Firmicutes |
| Oscillospiraceae sp. (HG3A.0944) | AHI | -0.045 | 0.014 | 0.041 | 3004 | unclassified | unclassified | unclassified | Oscillospiraceae | Eubacteriales | Clostridia | Firmicutes |
| Phocaeicola plebeius (HG3A.0423) | AHI | -0.046 | 0.013 | 0.041 | 3004 | unclassified | Phocaeicola plebeius | Phocaeicola | unclassified | Bacteroidales | Bacteroidia | Bacteroidetes |
| Clostridia sp. (HG3A.0746) | T90 | -0.042 | 0.016 | 0.042 | 3364 | unclassified | unclassified | unclassified | unclassified | unclassified | Clostridia | Firmicutes |
| Eubacteriales sp. (HG3A.0666) | AHI | -0.045 | 0.014 | 0.042 | 3004 | unclassified | unclassified | unclassified | unclassified | Eubacteriales | Clostridia | Firmicutes |
| Eubacteriales sp. (HG3A.0666) | T90 | -0.042 | 0.016 | 0.042 | 3364 | unclassified | unclassified | unclassified | unclassified | Eubacteriales | Clostridia | Firmicutes |
| Limosilactobacillus fermentum (HG3A.0990) | T90 | 0.042 | 0.016 | 0.042 | 3364 | unclassified | Limosilactobacillus fermentum | Limosilactobacillus | Lactobacillaceae | Lactobacillales | Bacilli | Firmicutes |
| Butyrivibrio crossotus  (HG3A.0413) | AHI | -0.045 | 0.014 | 0.043 | 3004 | unclassified | Butyrivibrio  crossotus | Butyrivibrio | Lachnospiraceae | Eubacteriales | Clostridia | Firmicutes |
| Clostridia sp. (HG3A.0401) | T90 | -0.042 | 0.016 | 0.043 | 3364 | unclassified | unclassified | unclassified | unclassified | unclassified | Clostridia | Firmicutes |
| Eubacteriales sp. (HG3A.0334) | T90 | -0.042 | 0.016 | 0.043 | 3364 | unclassified | unclassified | unclassified | unclassified | Eubacteriales | Clostridia | Firmicutes |
| Eubacteriales sp. (HG3A.0851) | T90 | -0.042 | 0.016 | 0.043 | 3364 | unclassified | unclassified | unclassified | unclassified | Eubacteriales | Clostridia | Firmicutes |
| Eubacteriales sp. (HG3A.1573) | AHI | -0.045 | 0.014 | 0.043 | 3004 | unclassified | unclassified | unclassified | unclassified | Eubacteriales | Clostridia | Firmicutes |
| Oscillospiraceae sp. (HG3A.0806) | T90 | -0.042 | 0.016 | 0.043 | 3364 | unclassified | unclassified | unclassified | Oscillospiraceae | Eubacteriales | Clostridia | Firmicutes |
| Oscillospiraceae sp. (HG3A.1421) | ODI | 0.041 | 0.018 | 0.043 | 3364 | unclassified | unclassified | unclassified | Oscillospiraceae | Eubacteriales | Clostridia | Firmicutes |

| Anaerotignum lactatifermentans (HG3A.0676) | AHI | 0.045 | 0.015 | 0.044 | 3004 | unclassified | Anaerotignum lactatifermentans | Anaerotignum | Lachnospiraceae | Eubacteriales | Clostridia | Firmicutes |
| --- | --- | --- | --- | --- | --- | --- | --- | --- | --- | --- | --- | --- |
| Bacteria sp. (HG3A.1543) | AHI | -0.045 | 0.015 | 0.044 | 3004 | unclassified | unclassified | unclassified | unclassified | unclassified | unclassified | unclassified |
| Clostridiaceae sp. (HG3A.0330) | AHI | -0.045 | 0.015 | 0.044 | 3004 | unclassified | unclassified | unclassified | Clostridiaceae | Eubacteriales | Clostridia | Firmicutes |
| [Clostridium] spiroforme  (HG3A.0259) | AHI | 0.045 | 0.015 | 0.044 | 3004 | unclassified | [Clostridium]  spiroforme | Erysipelatoclostridiu  m | Erysipelotrichace  ae | Erysipelotrichales | Erysipelotrichia | Firmicutes |
| Eubacteriales sp. (HG3A.0762) | T90 | -0.042 | 0.017 | 0.044 | 3364 | unclassified | unclassified | unclassified | unclassified | Eubacteriales | Clostridia | Firmicutes |
| Massilistercora timonensis  (HG3A.0458) | T90 | -0.042 | 0.017 | 0.044 | 3364 | unclassified | Massilistercora  timonensis | Massilistercora | unclassified | Eubacteriales | Clostridia | Firmicutes |
| Oxalobacter formigenes (HG3A.0552) | AHI | -0.045 | 0.015 | 0.044 | 3004 | unclassified | Oxalobacter formigenes | Oxalobacter | Oxalobacteraceae | Burkholderiales | Betaproteobacte ria | Proteobacteria |
| Veillonella tobetsuensis  (HG3A.1344) | ODI | -0.041 | 0.019 | 0.044 | 3364 | unclassified | Veillonella  tobetsuensis | Veillonella | Veillonellaceae | Veillonellales | Negativicutes | Firmicutes |
| Bacteroides cellulosilyticus (HG3A.0108) | T90 | -0.041 | 0.017 | 0.045 | 3364 | unclassified | Bacteroides cellulosilyticus | Bacteroides | Bacteroidaceae | Bacteroidales | Bacteroidia | Bacteroidetes |
| Blautia argi (HG3A.1450) | AHI | 0.045 | 0.015 | 0.045 | 3004 | unclassified | Blautia argi | Blautia | Lachnospiraceae | Eubacteriales | Clostridia | Firmicutes |
| Eubacteriales sp. (HG3A.0080) | ODI | 0.041 | 0.019 | 0.045 | 3364 | unclassified | unclassified | unclassified | unclassified | Eubacteriales | Clostridia | Firmicutes |
| Eubacteriales sp. (HG3A.0333) | ODI | -0.041 | 0.019 | 0.045 | 3364 | unclassified | unclassified | unclassified | unclassified | Eubacteriales | Clostridia | Firmicutes |
| Eubacteriales sp. (HG3A.0496) | AHI | -0.045 | 0.015 | 0.045 | 3004 | unclassified | unclassified | unclassified | unclassified | Eubacteriales | Clostridia | Firmicutes |
| Eubacteriales sp. (HG3A.0551) | T90 | -0.041 | 0.017 | 0.045 | 3364 | unclassified | unclassified | unclassified | unclassified | Eubacteriales | Clostridia | Firmicutes |
| Eubacteriales sp. (HG3A.0985) | AHI | 0.045 | 0.016 | 0.045 | 3004 | unclassified | unclassified | unclassified | unclassified | Eubacteriales | Clostridia | Firmicutes |
| Firmicutes sp. (HG3A.0301) | AHI | -0.045 | 0.015 | 0.045 | 3004 | unclassified | unclassified | unclassified | unclassified | unclassified | unclassified | Firmicutes |
| Firmicutes sp. (HG3A.1091) | T90 | -0.042 | 0.017 | 0.045 | 3364 | unclassified | unclassified | unclassified | unclassified | unclassified | unclassified | Firmicutes |
| Lachnospiraceae sp. (HG3A.0831) | AHI | -0.045 | 0.015 | 0.045 | 3004 | unclassified | unclassified | unclassified | Lachnospiraceae | Eubacteriales | Clostridia | Firmicutes |
| Oscillibacter sp. (HG3A.0046) | AHI | -0.045 | 0.015 | 0.045 | 3004 | unclassified | unclassified | Oscillibacter | Oscillospiraceae | Eubacteriales | Clostridia | Firmicutes |
| Oscillospiraceae sp. (HG3A.0849) | AHI | -0.045 | 0.015 | 0.045 | 3004 | unclassified | unclassified | unclassified | Oscillospiraceae | Eubacteriales | Clostridia | Firmicutes |
| Ruminococcus sp. (HG3A.0337) | ODI | -0.041 | 0.019 | 0.045 | 3364 | unclassified | unclassified | Ruminococcus | Oscillospiraceae | Eubacteriales | Clostridia | Firmicutes |
| Bacteria sp. (HG3A.0500) | ODI | -0.041 | 0.02 | 0.046 | 3364 | unclassified | unclassified | unclassified | unclassified | unclassified | unclassified | unclassified |

| Clostridia sp. (HG3A.0828) | AHI | -0.044 | 0.016 | 0.046 | 3004 | unclassified | unclassified | unclassified | unclassified | unclassified | Clostridia | Firmicutes |
| --- | --- | --- | --- | --- | --- | --- | --- | --- | --- | --- | --- | --- |
| Clostridia sp. (HG3A.1417) | T90 | -0.041 | 0.018 | 0.046 | 3364 | unclassified | unclassified | unclassified | unclassified | unclassified | Clostridia | Firmicutes |
| Eubacteriales sp. (HG3A.0628) | AHI | -0.044 | 0.016 | 0.046 | 3004 | unclassified | unclassified | unclassified | unclassified | Eubacteriales | Clostridia | Firmicutes |
| Eubacteriales sp. (HG3A.0654) | T90 | -0.041 | 0.018 | 0.046 | 3364 | unclassified | unclassified | unclassified | unclassified | Eubacteriales | Clostridia | Firmicutes |
| Eubacteriales sp. (HG3A.0956) | AHI | -0.044 | 0.016 | 0.046 | 3004 | unclassified | unclassified | unclassified | unclassified | Eubacteriales | Clostridia | Firmicutes |
| Eubacteriales sp. (HG3A.1294) | AHI | -0.045 | 0.016 | 0.046 | 3004 | unclassified | unclassified | unclassified | unclassified | Eubacteriales | Clostridia | Firmicutes |
| Faecalibacterium sp. OF04-11AC (HG3A.0070) | AHI | -0.044 | 0.016 | 0.046 | 3004 | unclassified | Faecalibacterium sp.  OF04-11AC | Faecalibacterium | Oscillospiraceae | Eubacteriales | Clostridia | Firmicutes |
| Firmicutes sp. (HG3A.1290) | T90 | -0.041 | 0.018 | 0.046 | 3364 | unclassified | unclassified | unclassified | unclassified | unclassified | unclassified | Firmicutes |
| Ligilactobacillus salivarius (HG3A.0919) | ODI | 0.041 | 0.02 | 0.046 | 3364 | unclassified | Ligilactobacillus salivarius | Ligilactobacillus | Lactobacillaceae | Lactobacillales | Bacilli | Firmicutes |
| Oscillospiraceae sp. (HG3A.0134) | T90 | -0.041 | 0.018 | 0.046 | 3364 | unclassified | unclassified | unclassified | Oscillospiraceae | Eubacteriales | Clostridia | Firmicutes |
| Oscillospiraceae sp. (HG3A.0765) | AHI | -0.044 | 0.016 | 0.046 | 3004 | unclassified | unclassified | unclassified | Oscillospiraceae | Eubacteriales | Clostridia | Firmicutes |
| Solobacterium moorei  (HG3A.1589) | T90 | 0.041 | 0.018 | 0.046 | 3364 | unclassified | Solobacterium  moorei | Solobacterium | Erysipelotrichace  ae | Erysipelotrichales | Erysipelotrichia | Firmicutes |
| Bacteroides intestinalis (HG3A.0265) | T90 | -0.041 | 0.018 | 0.047 | 3364 | unclassified | Bacteroides intestinalis | Bacteroides | Bacteroidaceae | Bacteroidales | Bacteroidia | Bacteroidetes |
| Clostridia sp. (HG3A.1139) | AHI | -0.044 | 0.016 | 0.047 | 3004 | unclassified | unclassified | unclassified | unclassified | unclassified | Clostridia | Firmicutes |
| Clostridia sp. (HG3A.1141) | T90 | -0.041 | 0.019 | 0.047 | 3364 | unclassified | unclassified | unclassified | unclassified | unclassified | Clostridia | Firmicutes |
| Clostridia sp. (HG3A.1410) | T90 | -0.041 | 0.018 | 0.047 | 3364 | unclassified | unclassified | unclassified | unclassified | unclassified | Clostridia | Firmicutes |
| Dialister pneumosintes (HG3A.1496) | ODI | 0.041 | 0.02 | 0.047 | 3364 | unclassified | Dialister pneumosintes | Dialister | Veillonellaceae | Veillonellales | Negativicutes | Firmicutes |
| Enterocloster sp. (HG3A.1529) | T90 | 0.041 | 0.018 | 0.047 | 3364 | unclassified | unclassified | Enterocloster | Lachnospiraceae | Eubacteriales | Clostridia | Firmicutes |
| Erysipelatoclostridium ramosum (HG3A.0538) | ODI | 0.04 | 0.02 | 0.047 | 3364 | unclassified | Erysipelatoclostridiu m ramosum | Erysipelatoclostridiu m | Erysipelotrichace ae | Erysipelotrichales | Erysipelotrichia | Firmicutes |
| Eubacteriales sp. (HG3A.0428) | T90 | -0.041 | 0.018 | 0.047 | 3364 | unclassified | unclassified | unclassified | unclassified | Eubacteriales | Clostridia | Firmicutes |
| Eubacteriales sp. (HG3A.0473) | AHI | -0.044 | 0.017 | 0.047 | 3004 | unclassified | unclassified | unclassified | unclassified | Eubacteriales | Clostridia | Firmicutes |
| Eubacteriales sp. (HG3A.0536) | T90 | -0.041 | 0.018 | 0.047 | 3364 | unclassified | unclassified | unclassified | unclassified | Eubacteriales | Clostridia | Firmicutes |

| Eubacteriales sp. (HG3A.0717) | ODI | -0.04 | 0.02 | 0.047 | 3364 | unclassified | unclassified | unclassified | unclassified | Eubacteriales | Clostridia | Firmicutes |
| --- | --- | --- | --- | --- | --- | --- | --- | --- | --- | --- | --- | --- |
| Eubacteriales sp. (HG3A.0868) | T90 | -0.041 | 0.018 | 0.047 | 3364 | unclassified | unclassified | unclassified | unclassified | Eubacteriales | Clostridia | Firmicutes |
| Oscillospiraceae sp. (HG3A.0382) | AHI | -0.044 | 0.016 | 0.047 | 3004 | unclassified | unclassified | unclassified | Oscillospiraceae | Eubacteriales | Clostridia | Firmicutes |
| Oscillospiraceae sp. (HG3A.0805) | AHI | -0.044 | 0.016 | 0.047 | 3004 | unclassified | unclassified | unclassified | Oscillospiraceae | Eubacteriales | Clostridia | Firmicutes |

Pediococcus pentosaceus

(HG3A.1246)

AHI

0.044

0.016

0.047 3004 unclassified

Pediococcus

pentosaceus

Pediococcus Lactobacillaceae Lactobacillales

Bacilli

Firmicutes

Akkermansia muciniphila

(HG3A.0110)

AHI -0.044 0.017 0.048 3004 unclassified Akkermansia

muciniphila

Akkermansia Akkermansiaceae Verrucomicrobiales Verrucomicrobi

ae

Verrucomicrobi

a

Alistipes provencensis

(HG3A.0877)

AHI

-0.044

0.017

0.048 3004 unclassified

Alistipes

provencensis

Alistipes

Rikenellaceae

Bacteroidales

Bacteroidia Bacteroidetes

Clostridia sp. (HG3A.1128) ODI -0.04 0.021 0.048 3364 unclassified unclassified unclassified unclassified unclassified Clostridia Firmicutes

Eubacteriales sp. (HG3A.0221)

AHI

-0.044

0.017

0.048 3004 unclassified

unclassified

unclassified

unclassified

Eubacteriales

Clostridia

Firmicutes

Eubacteriales sp. (HG3A.0618) ODI -0.04 0.021 0.048 3364 unclassified unclassified unclassified unclassified Eubacteriales Clostridia Firmicutes

Eubacteriales sp. (HG3A.0694)

T90

-0.041

0.019

0.048 3364 unclassified

unclassified

unclassified

unclassified

Eubacteriales

Clostridia

Firmicutes

Eubacteriales sp. (HG3A.0976) AHI -0.044 0.017 0.048 3004 unclassified unclassified unclassified unclassified Eubacteriales Clostridia Firmicutes

Eubacteriales sp. (HG3A.1393)

T90

-0.041

0.019

0.048 3364 unclassified

unclassified

unclassified

unclassified

Eubacteriales

Clostridia

Firmicutes

Prevotella sp. (HG3A.1040) ODI 0.04 0.021 0.048 3364 unclassified unclassified Prevotella Prevotellaceae Bacteroidales Bacteroidia Bacteroidetes

Bacteria sp. (HG3A.0839)

ODI

-0.04

0.021

0.049 3364 unclassified

unclassified

unclassified

unclassified

unclassified

unclassified unclassified

Eubacteriales sp. (HG3A.0204) AHI -0.044 0.017 0.049 3004 unclassified unclassified unclassified unclassified Eubacteriales Clostridia Firmicutes

Eubacteriales sp. (HG3A.0565)

T90

-0.041

0.019

0.049 3364 unclassified

unclassified

unclassified

unclassified

Eubacteriales

Clostridia

Firmicutes

Eubacteriales sp. (HG3A.0647) ODI -0.04 0.021 0.049 3364 unclassified unclassified unclassified unclassified Eubacteriales Clostridia Firmicutes

Eubacteriales sp. (HG3A.0985)

ODI

0.04

0.021

0.049 3364 unclassified

unclassified

unclassified

unclassified

Eubacteriales

Clostridia

Firmicutes

Eubacteriales sp. (HG3A.1546) T90 -0.041 0.019 0.049 3364 unclassified unclassified unclassified unclassified Eubacteriales Clostridia Firmicutes

Oscillospiraceae sp. (HG3A.1491) T90

-0.041

0.019

0.049 3364 unclassified

unclassified

unclassified

Oscillospiraceae Eubacteriales

Clostridia

Firmicutes

Oxalobacter sp. (HG3A.1218) AHI -0.044 0.017 0.049 3004 unclassified unclassified Oxalobacter Oxalobacteraceae Burkholderiales Betaproteobacte

ria

Proteobacteria

| Bacteria sp. (HG3A.0361) | ODI | -0.04 | 0.022 | 0.05 | 3364 | unclassified | unclassified | unclassified | unclassified | unclassified | unclassified | unclassified |
| --- | --- | --- | --- | --- | --- | --- | --- | --- | --- | --- | --- | --- |
| Clostridia sp. (HG3A.1356) | ODI | -0.04 | 0.021 | 0.05 | 3364 | unclassified | unclassified | unclassified | unclassified | unclassified | Clostridia | Firmicutes |
| Clostridiaceae sp. (HG3A.1567) | AHI | -0.044 | 0.018 | 0.05 | 3004 | unclassified | unclassified | unclassified | Clostridiaceae | Eubacteriales | Clostridia | Firmicutes |
| Eubacteriales sp. (HG3A.0971) | ODI | -0.04 | 0.022 | 0.05 | 3364 | unclassified | unclassified | unclassified | unclassified | Eubacteriales | Clostridia | Firmicutes |
| Lachnospiraceae sp. (HG3A.0899) | AHI | -0.044 | 0.018 | 0.05 | 3004 | unclassified | unclassified | unclassified | Lachnospiraceae | Eubacteriales | Clostridia | Firmicutes |
| Turicibacter sanguinis  (HG3A.0274) | AHI | -0.044 | 0.018 | 0.05 | 3004 | unclassified | Turicibacter  sanguinis | Turicibacter | Turicibacteraceae | Erysipelotrichales | Erysipelotrichia | Firmicutes |
| Tyzzerella nexilis (HG3A.0574) | AHI | 0.044 | 0.018 | 0.05 | 3004 | unclassified | Tyzzerella nexilis | Tyzzerella | Lachnospiraceae | Eubacteriales | Clostridia | Firmicutes |
| Anaerostipes sp. BG01  (HG3A.1509) | T90 | 0.04 | 0.02 | 0.051 | 3364 | unclassified | Anaerostipes sp.  BG01 | Anaerostipes | Lachnospiraceae | Eubacteriales | Clostridia | Firmicutes |
| Eubacteriales sp. (HG3A.0061) | ODI | 0.04 | 0.022 | 0.051 | 3364 | unclassified | unclassified | unclassified | unclassified | Eubacteriales | Clostridia | Firmicutes |
| Eubacteriales sp. (HG3A.0306) | AHI | -0.044 | 0.018 | 0.051 | 3004 | unclassified | unclassified | unclassified | unclassified | Eubacteriales | Clostridia | Firmicutes |
| Eubacteriales sp. (HG3A.0573) | T90 | -0.04 | 0.02 | 0.051 | 3364 | unclassified | unclassified | unclassified | unclassified | Eubacteriales | Clostridia | Firmicutes |
| Eubacteriales sp. (HG3A.1484) | T90 | -0.04 | 0.02 | 0.051 | 3364 | unclassified | unclassified | unclassified | unclassified | Eubacteriales | Clostridia | Firmicutes |
| Eubacteriales sp. (HG3A.1721) | T90 | -0.04 | 0.02 | 0.051 | 3364 | unclassified | unclassified | unclassified | unclassified | Eubacteriales | Clostridia | Firmicutes |
| Faecalibacterium prausnitzii  (HG3A.0029) | T90 | 0.04 | 0.02 | 0.051 | 3364 | unclassified | Faecalibacterium  prausnitzii | Faecalibacterium | Oscillospiraceae | Eubacteriales | Clostridia | Firmicutes |
| Lachnospiraceae sp. (HG3A.0217) | ODI | -0.04 | 0.022 | 0.051 | 3364 | unclassified | unclassified | unclassified | Lachnospiraceae | Eubacteriales | Clostridia | Firmicutes |
| Oscillibacter sp. (HG3A.0243) | AHI | -0.044 | 0.018 | 0.051 | 3004 | unclassified | unclassified | Oscillibacter | Oscillospiraceae | Eubacteriales | Clostridia | Firmicutes |
| Oscillospiraceae sp. (HG3A.1161) | ODI | 0.04 | 0.022 | 0.051 | 3364 | unclassified | unclassified | unclassified | Oscillospiraceae | Eubacteriales | Clostridia | Firmicutes |
| Bacteria sp. (HG3A.0839) | AHI | -0.043 | 0.019 | 0.052 | 3004 | unclassified | unclassified | unclassified | unclassified | unclassified | unclassified | unclassified |
| Bacteroidales sp. (HG3A.0894) | AHI | -0.043 | 0.019 | 0.052 | 3004 | unclassified | unclassified | unclassified | unclassified | Bacteroidales | Bacteroidia | Bacteroidetes |
| Eubacteriales sp. (HG3A.0493) | AHI | -0.043 | 0.019 | 0.052 | 3004 | unclassified | unclassified | unclassified | unclassified | Eubacteriales | Clostridia | Firmicutes |
| Eubacteriales sp. (HG3A.0760) | AHI | -0.043 | 0.019 | 0.052 | 3004 | unclassified | unclassified | unclassified | unclassified | Eubacteriales | Clostridia | Firmicutes |

Lachnoclostridium sp. (HG3A.0655)

T90 0.04 0.021 0.052 3364 unclassified unclassified Lachnoclostridium Lachnospiraceae Eubacteriales Clostridia Firmicutes

| Oscillospiraceae sp. (HG3A.0765) | ODI | -0.04 | 0.023 | 0.052 | 3364 | unclassified | unclassified | unclassified | Oscillospiraceae | Eubacteriales | Clostridia | Firmicutes |
| --- | --- | --- | --- | --- | --- | --- | --- | --- | --- | --- | --- | --- |
| Eubacteriaceae sp. (HG3A.1012) | T90 | -0.04 | 0.021 | 0.053 | 3364 | unclassified | unclassified | unclassified | Eubacteriaceae | Eubacteriales | Clostridia | Firmicutes |
| Eubacteriales sp. (HG3A.0624) | AHI | -0.043 | 0.019 | 0.053 | 3004 | unclassified | unclassified | unclassified | unclassified | Eubacteriales | Clostridia | Firmicutes |
| Eubacteriales sp. (HG3A.0935) | T90 | -0.04 | 0.021 | 0.053 | 3364 | unclassified | unclassified | unclassified | unclassified | Eubacteriales | Clostridia | Firmicutes |
| Eubacteriales sp. (HG3A.0950) | T90 | -0.04 | 0.021 | 0.053 | 3364 | unclassified | unclassified | unclassified | unclassified | Eubacteriales | Clostridia | Firmicutes |
| Eubacteriales sp. (HG3A.1063) | T90 | -0.04 | 0.021 | 0.053 | 3364 | unclassified | unclassified | unclassified | unclassified | Eubacteriales | Clostridia | Firmicutes |
| Firmicutes sp. (HG3A.0923) | AHI | -0.043 | 0.019 | 0.053 | 3004 | unclassified | unclassified | unclassified | unclassified | unclassified | unclassified | Firmicutes |
| Firmicutes sp. (HG3A.1195) | T90 | -0.04 | 0.021 | 0.053 | 3364 | unclassified | unclassified | unclassified | unclassified | unclassified | unclassified | Firmicutes |
| Oscillospiraceae sp. (HG3A.1161) | T90 | 0.04 | 0.021 | 0.053 | 3364 | unclassified | unclassified | unclassified | Oscillospiraceae | Eubacteriales | Clostridia | Firmicutes |
| Streptococcus sobrinus  (HG3A.1366) | AHI | 0.043 | 0.019 | 0.053 | 3004 | unclassified | Streptococcus  sobrinus | Streptococcus | Streptococcaceae | Lactobacillales | Bacilli | Firmicutes |
| Clostridia sp. (HG3A.0553) | AHI | -0.043 | 0.02 | 0.054 | 3004 | unclassified | unclassified | unclassified | unclassified | unclassified | Clostridia | Firmicutes |
| Clostridia sp. (HG3A.1252) | T90 | -0.04 | 0.022 | 0.054 | 3364 | unclassified | unclassified | unclassified | unclassified | unclassified | Clostridia | Firmicutes |
| Clostridia sp. (HG3A.1417) | AHI | -0.043 | 0.02 | 0.054 | 3004 | unclassified | unclassified | unclassified | unclassified | unclassified | Clostridia | Firmicutes |
| Eubacteriales sp. (HG3A.0482) | AHI | -0.043 | 0.02 | 0.054 | 3004 | unclassified | unclassified | unclassified | unclassified | Eubacteriales | Clostridia | Firmicutes |
| Eubacteriales sp. (HG3A.0642) | ODI | -0.039 | 0.023 | 0.054 | 3364 | unclassified | unclassified | unclassified | unclassified | Eubacteriales | Clostridia | Firmicutes |
| Eubacteriales sp. (HG3A.0873) | AHI | -0.043 | 0.02 | 0.054 | 3004 | unclassified | unclassified | unclassified | unclassified | Eubacteriales | Clostridia | Firmicutes |
| Eubacteriales sp. (HG3A.1045) | T90 | -0.04 | 0.022 | 0.054 | 3364 | unclassified | unclassified | unclassified | unclassified | Eubacteriales | Clostridia | Firmicutes |
| Eubacteriales sp. (HG3A.1369) | ODI | -0.039 | 0.024 | 0.054 | 3364 | unclassified | unclassified | unclassified | unclassified | Eubacteriales | Clostridia | Firmicutes |
| Hungatella hathewayi (HG3A.0287) | T90 | 0.04 | 0.022 | 0.054 | 3364 | unclassified | Hungatella hathewayi | Hungatella | Clostridiaceae | Eubacteriales | Clostridia | Firmicutes |
| Oscillospiraceae sp. (HG3A.1516) | ODI | -0.039 | 0.023 | 0.054 | 3364 | unclassified | unclassified | unclassified | Oscillospiraceae | Eubacteriales | Clostridia | Firmicutes |

Fusobacterium nucleatum subsp.

animalis (HG3A.1418)

AHI

0.043

0.02

0.055

Fusobacterium 3004 nucleatum subsp.

animalis

Fusobacterium

nucleatum

Fusobacterium Fusobacteriaceae Fusobacteriales Fusobacteriia Fusobacteria

Pediococcus acidilactici (HG3A.1468)

| Veillonella tobetsuensis (HG3A.1344) | AHI | -0.043 | 0.02 | 0.055 | 3004 | unclassified | Veillonella tobetsuensis | Veillonella | Veillonellaceae | Veillonellales | Negativicutes | Firmicutes |
| --- | --- | --- | --- | --- | --- | --- | --- | --- | --- | --- | --- | --- |
| Bifidobacterium longum subsp. longum (HG3A.0038) | T90 | -0.04 | 0.023 | 0.056 | 3364 | Bifidobacterium longum subsp.  longum | Bifidobacterium longum | Bifidobacterium | Bifidobacteriaceae | Bifidobacteriales | Actinomycetia | Actinobacteria |
| Clostridia sp. (HG3A.0519) | T90 | -0.04 | 0.023 | 0.056 | 3364 | unclassified | unclassified | unclassified | unclassified | unclassified | Clostridia | Firmicutes |
| Eubacteriales sp. (HG3A.0296) | AHI | -0.043 | 0.02 | 0.056 | 3004 | unclassified | unclassified | unclassified | unclassified | Eubacteriales | Clostridia | Firmicutes |
| Eubacteriales sp. (HG3A.0764) | ODI | -0.039 | 0.025 | 0.056 | 3364 | unclassified | unclassified | unclassified | unclassified | Eubacteriales | Clostridia | Firmicutes |
| Firmicutes sp. (HG3A.0570) | AHI | -0.043 | 0.021 | 0.056 | 3004 | unclassified | unclassified | unclassified | unclassified | unclassified | unclassified | Firmicutes |
| Mesosutterella multiformis (HG3A.0520) | ODI | -0.039 | 0.025 | 0.056 | 3364 | unclassified | Mesosutterella multiformis | Mesosutterella | Sutterellaceae | Burkholderiales | Betaproteobacte ria | Proteobacteria |
| Prevotella sp. (HG3A.1040) | AHI | 0.043 | 0.021 | 0.056 | 3004 | unclassified | unclassified | Prevotella | Prevotellaceae | Bacteroidales | Bacteroidia | Bacteroidetes |
| Bacteria sp. (HG3A.1373) | ODI | -0.039 | 0.025 | 0.057 | 3364 | unclassified | unclassified | unclassified | unclassified | unclassified | unclassified | unclassified |
| Eubacteriales sp. (HG3A.0702) | ODI | -0.039 | 0.025 | 0.057 | 3364 | unclassified | unclassified | unclassified | unclassified | Eubacteriales | Clostridia | Firmicutes |
| Anaerostipes hadrus (HG3A.0003) | AHI | 0.042 | 0.021 | 0.058 | 3004 | unclassified | Anaerostipes hadrus | Anaerostipes | Lachnospiraceae | Eubacteriales | Clostridia | Firmicutes |
| Eubacteriales sp. (HG3A.0359) | ODI | -0.039 | 0.026 | 0.058 | 3364 | unclassified | unclassified | unclassified | unclassified | Eubacteriales | Clostridia | Firmicutes |
| Eubacteriales sp. (HG3A.1134) | AHI | -0.042 | 0.021 | 0.058 | 3004 | unclassified | unclassified | unclassified | unclassified | Eubacteriales | Clostridia | Firmicutes |
| Eubacteriales sp. (HG3A.1281) | T90 | -0.039 | 0.023 | 0.058 | 3364 | unclassified | unclassified | unclassified | unclassified | Eubacteriales | Clostridia | Firmicutes |
| Firmicutes sp. (HG3A.1345) | AHI | -0.042 | 0.021 | 0.058 | 3004 | unclassified | unclassified | unclassified | unclassified | unclassified | unclassified | Firmicutes |
| Lachnospiraceae sp. (HG3A.1098) | T90 | -0.039 | 0.023 | 0.058 | 3364 | unclassified | unclassified | unclassified | Lachnospiraceae | Eubacteriales | Clostridia | Firmicutes |
| Sutterellaceae sp. (HG3A.1122) | ODI | 0.039 | 0.026 | 0.058 | 3364 | unclassified | unclassified | unclassified | Sutterellaceae | Burkholderiales | Betaproteobacte ria | Proteobacteria |
| Acidaminococcales sp.  (HG3A.0542) | ODI | -0.039 | 0.026 | 0.059 | 3364 | unclassified | unclassified | unclassified | unclassified | Acidaminococcales | Negativicutes | Firmicutes |
| Bacteria sp. (HG3A.0638) | ODI | -0.039 | 0.026 | 0.059 | 3364 | unclassified | unclassified | unclassified | unclassified | unclassified | unclassified | unclassified |
| Eisenbergiella massiliensis  (HG3A.0425) | T90 | 0.039 | 0.024 | 0.059 | 3364 | unclassified | Eisenbergiella  massiliensis | Eisenbergiella | Lachnospiraceae | Eubacteriales | Clostridia | Firmicutes |
| Eubacteriales sp. (HG3A.0202) | ODI | 0.039 | 0.026 | 0.059 | 3364 | unclassified | unclassified | unclassified | unclassified | Eubacteriales | Clostridia | Firmicutes |

AHI 0.043 0.02 0.055 3004 unclassified Pediococcus

acidilactici

Pediococcus Lactobacillaceae Lactobacillales Bacilli Firmicutes

| Eubacteriales sp. (HG3A.0480) | ODI | -0.039 | 0.026 | 0.059 | 3364 | unclassified | unclassified | unclassified | unclassified | Eubacteriales | Clostridia | Firmicutes |
| --- | --- | --- | --- | --- | --- | --- | --- | --- | --- | --- | --- | --- |
| Eubacteriales sp. (HG3A.0510) | ODI | -0.039 | 0.026 | 0.059 | 3364 | unclassified | unclassified | unclassified | unclassified | Eubacteriales | Clostridia | Firmicutes |
| Eubacteriales sp. (HG3A.0854) | ODI | 0.039 | 0.026 | 0.059 | 3364 | unclassified | unclassified | unclassified | unclassified | Eubacteriales | Clostridia | Firmicutes |
| Phocaeicola vulgatus (HG3A.0005) | T90 | -0.039 | 0.024 | 0.059 | 3364 | unclassified | Phocaeicola vulgatus | Phocaeicola | Bacteroidaceae | Bacteroidales | Bacteroidia | Bacteroidetes |
| Clostridia sp. (HG3A.0553) | ODI | -0.039 | 0.027 | 0.06 | 3364 | unclassified | unclassified | unclassified | unclassified | unclassified | Clostridia | Firmicutes |
| Eubacteriales sp. (HG3A.0333) | T90 | -0.039 | 0.024 | 0.06 | 3364 | unclassified | unclassified | unclassified | unclassified | Eubacteriales | Clostridia | Firmicutes |
| Eubacteriales sp. (HG3A.0592) | AHI | -0.042 | 0.022 | 0.06 | 3004 | unclassified | unclassified | unclassified | unclassified | Eubacteriales | Clostridia | Firmicutes |
| Eubacteriales sp. (HG3A.0791) | ODI | -0.038 | 0.027 | 0.06 | 3364 | unclassified | unclassified | unclassified | unclassified | Eubacteriales | Clostridia | Firmicutes |
| Eubacteriales sp. (HG3A.0924) | ODI | -0.038 | 0.027 | 0.06 | 3364 | unclassified | unclassified | unclassified | unclassified | Eubacteriales | Clostridia | Firmicutes |
| Eubacteriales sp. (HG3A.1369) | AHI | -0.042 | 0.022 | 0.06 | 3004 | unclassified | unclassified | unclassified | unclassified | Eubacteriales | Clostridia | Firmicutes |
| Ruminococcus bicirculans  (HG3A.0067) | T90 | -0.039 | 0.025 | 0.06 | 3364 | unclassified | Ruminococcus  bicirculans | Ruminococcus | Oscillospiraceae | Eubacteriales | Clostridia | Firmicutes |
| Clostridia sp. (HG3A.1247) | AHI | -0.042 | 0.023 | 0.061 | 3004 | unclassified | unclassified | unclassified | unclassified | unclassified | Clostridia | Firmicutes |
| Escherichia sp. 4726-5  (HG3A.1469) | AHI | 0.042 | 0.023 | 0.061 | 3004 | unclassified | Escherichia sp. 4726-  5 | Escherichia | Enterobacteriacea  e | Enterobacterales | Gammaproteob  acteria | Proteobacteria |
| Eubacteriales sp. (HG3A.0386) | T90 | -0.039 | 0.025 | 0.061 | 3364 | unclassified | unclassified | unclassified | unclassified | Eubacteriales | Clostridia | Firmicutes |
| Eubacteriales sp. (HG3A.0427) | T90 | -0.039 | 0.025 | 0.061 | 3364 | unclassified | unclassified | unclassified | unclassified | Eubacteriales | Clostridia | Firmicutes |
| Eubacteriales sp. (HG3A.0536) | ODI | -0.038 | 0.028 | 0.061 | 3364 | unclassified | unclassified | unclassified | unclassified | Eubacteriales | Clostridia | Firmicutes |
| Eubacteriales sp. (HG3A.0633) | AHI | -0.042 | 0.023 | 0.061 | 3004 | unclassified | unclassified | unclassified | unclassified | Eubacteriales | Clostridia | Firmicutes |
| Eubacteriales sp. (HG3A.0643) | ODI | -0.038 | 0.027 | 0.061 | 3364 | unclassified | unclassified | unclassified | unclassified | Eubacteriales | Clostridia | Firmicutes |
| Eubacteriales sp. (HG3A.0678) | AHI | -0.042 | 0.023 | 0.061 | 3004 | unclassified | unclassified | unclassified | unclassified | Eubacteriales | Clostridia | Firmicutes |
| Eubacteriales sp. (HG3A.0685) | T90 | -0.039 | 0.025 | 0.061 | 3364 | unclassified | unclassified | unclassified | unclassified | Eubacteriales | Clostridia | Firmicutes |
| Eubacteriales sp. (HG3A.1051) | ODI | -0.038 | 0.027 | 0.061 | 3364 | unclassified | unclassified | unclassified | unclassified | Eubacteriales | Clostridia | Firmicutes |
| Firmicutes sp. (HG3A.0915) | ODI | -0.038 | 0.027 | 0.061 | 3364 | unclassified | unclassified | unclassified | unclassified | unclassified | unclassified | Firmicutes |

| Lachnospiraceae sp. (HG3A.1240) | ODI | 0.038 | 0.027 | 0.061 | 3364 | unclassified | unclassified | unclassified | Lachnospiraceae | Eubacteriales | Clostridia | Firmicutes |
| --- | --- | --- | --- | --- | --- | --- | --- | --- | --- | --- | --- | --- |
| Lachnospiraceae sp. (HG3A.1525) | T90 | -0.039 | 0.025 | 0.061 | 3364 | unclassified | unclassified | unclassified | Lachnospiraceae | Eubacteriales | Clostridia | Firmicutes |
| Agathobaculum sp. (HG3A.0679) | T90 | 0.039 | 0.025 | 0.062 | 3364 | unclassified | unclassified | Agathobaculum | Oscillospiraceae | Eubacteriales | Clostridia | Firmicutes |
| Bacteroidales sp. (HG3A.0452) | ODI | -0.038 | 0.028 | 0.062 | 3364 | unclassified | unclassified | unclassified | unclassified | Bacteroidales | Bacteroidia | Bacteroidetes |
| Eubacteriales sp. (HG3A.1072) | ODI | -0.038 | 0.028 | 0.062 | 3364 | unclassified | unclassified | unclassified | unclassified | Eubacteriales | Clostridia | Firmicutes |
| Oscillospiraceae sp. (HG3A.0998) | T90 | -0.039 | 0.026 | 0.062 | 3364 | unclassified | unclassified | unclassified | Oscillospiraceae | Eubacteriales | Clostridia | Firmicutes |
| Akkermansia sp. BIOML-A59 T90 -0.039 0.026 0.063 3364 unclassified Akkermansia sp. Akkermansia Akkermansiaceae Verrucomicrobiales Verrucomicrobi Verrucomicrobi  (HG3A.0800) BIOML-A59 ae a | | | | | | | | | | | | |
| Bacteria sp. (HG3A.1117) | T90 | -0.039 | 0.026 | 0.063 | 3364 | unclassified | unclassified | unclassified | unclassified | unclassified | unclassified | unclassified |
| Clostridia sp. (HG3A.1143) | ODI | -0.038 | 0.029 | 0.063 | 3364 | unclassified | unclassified | unclassified | unclassified | unclassified | Clostridia | Firmicutes |
| Eubacteriales sp. (HG3A.0476) | T90 | -0.039 | 0.026 | 0.063 | 3364 | unclassified | unclassified | unclassified | unclassified | Eubacteriales | Clostridia | Firmicutes |
| Eubacteriales sp. (HG3A.0821) | T90 | -0.039 | 0.026 | 0.063 | 3364 | unclassified | unclassified | unclassified | unclassified | Eubacteriales | Clostridia | Firmicutes |
| Eubacteriales sp. (HG3A.1129) | AHI | -0.042 | 0.024 | 0.063 | 3004 | unclassified | unclassified | unclassified | unclassified | Eubacteriales | Clostridia | Firmicutes |
| Eubacteriales sp. (HG3A.1199) | AHI | 0.042 | 0.023 | 0.063 | 3004 | unclassified | unclassified | unclassified | unclassified | Eubacteriales | Clostridia | Firmicutes |
| Eubacteriales sp. (HG3A.1305) | ODI | -0.038 | 0.028 | 0.063 | 3364 | unclassified | unclassified | unclassified | unclassified | Eubacteriales | Clostridia | Firmicutes |
| Fenollaria massiliensis  (HG3A.1255) | ODI | -0.038 | 0.028 | 0.063 | 3364 | unclassified | Fenollaria  massiliensis | Fenollaria | unclassified | Eubacteriales | Clostridia | Firmicutes |
| Firmicutes sp. (HG3A.1461) | T90 | -0.039 | 0.026 | 0.063 | 3364 | unclassified | unclassified | unclassified | unclassified | unclassified | unclassified | Firmicutes |
| Porphyromonadaceae sp.  (HG3A.1216) | ODI | -0.038 | 0.028 | 0.063 | 3364 | unclassified | unclassified | unclassified | Porphyromonadac  eae | Bacteroidales | Bacteroidia | Bacteroidetes |
| Bacteria sp. (HG3A.1373) | T90 | -0.038 | 0.027 | 0.064 | 3364 | unclassified | unclassified | unclassified | unclassified | unclassified | unclassified | unclassified |
| Bacteroidales sp. (HG3A.1378) | T90 | -0.039 | 0.026 | 0.064 | 3364 | unclassified | unclassified | unclassified | unclassified | Bacteroidales | Bacteroidia | Bacteroidetes |
| Faecalibacterium sp. (HG3A.0042) | T90 | -0.039 | 0.026 | 0.064 | 3364 | unclassified | unclassified | Faecalibacterium | Oscillospiraceae | Eubacteriales | Clostridia | Firmicutes |
| Lachnospiraceae sp. (HG3A.0903) | T90 | -0.039 | 0.027 | 0.064 | 3364 | unclassified | unclassified | unclassified | Lachnospiraceae | Eubacteriales | Clostridia | Firmicutes |
| Eubacteriales sp. (HG3A.1025) | ODI | -0.038 | 0.029 | 0.065 | 3364 | unclassified | unclassified | unclassified | unclassified | Eubacteriales | Clostridia | Firmicutes |

| Firmicutes sp. (HG3A.1115) | AHI | -0.041 | 0.024 | 0.065 | 3004 | unclassified | unclassified | unclassified | unclassified | unclassified | unclassified | Firmicutes |
| --- | --- | --- | --- | --- | --- | --- | --- | --- | --- | --- | --- | --- |
| Mesosutterella multiformis (HG3A.0520) | T90 | -0.038 | 0.027 | 0.065 | 3364 | unclassified | Mesosutterella multiformis | Mesosutterella | Sutterellaceae | Burkholderiales | Betaproteobacte ria | Proteobacteria |
| Succinatimonas hippei  (HG3A.1322) | T90 | 0.038 | 0.027 | 0.065 | 3364 | unclassified | Succinatimonas  hippei | Succinatimonas | Succinivibrionace  ae | Aeromonadales | Gammaproteob  acteria | Proteobacteria |
| Bacteria sp. (HG3A.0708) | ODI | -0.038 | 0.03 | 0.066 | 3364 | unclassified | unclassified | unclassified | unclassified | unclassified | unclassified | unclassified |
| Clostridia sp. (HG3A.0898) | T90 | -0.038 | 0.028 | 0.066 | 3364 | unclassified | unclassified | unclassified | unclassified | unclassified | Clostridia | Firmicutes |
| Erysipelotrichales sp. (HG3A.0809) | T90 | 0.038 | 0.028 | 0.066 | 3364 | unclassified | unclassified | unclassified | unclassified | Erysipelotrichales | Erysipelotrichia | Firmicutes |
| Eubacteriales sp. (HG3A.0480) | T90 | -0.038 | 0.028 | 0.066 | 3364 | unclassified | unclassified | unclassified | unclassified | Eubacteriales | Clostridia | Firmicutes |
| Eubacteriales sp. (HG3A.0557) | T90 | -0.038 | 0.028 | 0.066 | 3364 | unclassified | unclassified | unclassified | unclassified | Eubacteriales | Clostridia | Firmicutes |
| Eubacteriales sp. (HG3A.0939) | ODI | -0.038 | 0.03 | 0.066 | 3364 | unclassified | unclassified | unclassified | unclassified | Eubacteriales | Clostridia | Firmicutes |
| Eubacteriales sp. (HG3A.0984) | T90 | -0.038 | 0.028 | 0.066 | 3364 | unclassified | unclassified | unclassified | unclassified | Eubacteriales | Clostridia | Firmicutes |
| Eubacteriales sp. (HG3A.1280) | T90 | -0.038 | 0.028 | 0.066 | 3364 | unclassified | unclassified | unclassified | unclassified | Eubacteriales | Clostridia | Firmicutes |
| Eubacteriales sp. (HG3A.1305) | T90 | -0.038 | 0.028 | 0.066 | 3364 | unclassified | unclassified | unclassified | unclassified | Eubacteriales | Clostridia | Firmicutes |
| Faecalibacterium sp. OF04-11AC  (HG3A.0070) | T90 | -0.038 | 0.028 | 0.066 | 3364 | unclassified | Faecalibacterium sp.  OF04-11AC | Faecalibacterium | Oscillospiraceae | Eubacteriales | Clostridia | Firmicutes |
| Actinomyces sp. ICM58 (HG3A.0410) | AHI | 0.041 | 0.025 | 0.067 | 3004 | unclassified | Actinomyces sp.  ICM58 | Actinomyces | Actinomycetaceae | Actinomycetales | Actinomycetia | Actinobacteria |
| Clostridia sp. (HG3A.1585) | ODI | -0.038 | 0.031 | 0.067 | 3364 | unclassified | unclassified | unclassified | unclassified | unclassified | Clostridia | Firmicutes |
| Eubacteriales sp. (HG3A.0678) | ODI | -0.038 | 0.031 | 0.067 | 3364 | unclassified | unclassified | unclassified | unclassified | Eubacteriales | Clostridia | Firmicutes |
| Eubacteriales sp. (HG3A.0755) | AHI | -0.041 | 0.025 | 0.067 | 3004 | unclassified | unclassified | unclassified | unclassified | Eubacteriales | Clostridia | Firmicutes |
| Eubacteriales sp. (HG3A.0967) | AHI | -0.041 | 0.025 | 0.067 | 3004 | unclassified | unclassified | unclassified | unclassified | Eubacteriales | Clostridia | Firmicutes |
| Eubacteriales sp. (HG3A.1484) | ODI | -0.038 | 0.03 | 0.067 | 3364 | unclassified | unclassified | unclassified | unclassified | Eubacteriales | Clostridia | Firmicutes |
| Lachnospiraceae sp. (HG3A.1098) | ODI | -0.038 | 0.031 | 0.067 | 3364 | unclassified | unclassified | unclassified | Lachnospiraceae | Eubacteriales | Clostridia | Firmicutes |
| Bacteroidales sp. (HG3A.0340) | AHI | -0.041 | 0.026 | 0.068 | 3004 | unclassified | unclassified | unclassified | unclassified | Bacteroidales | Bacteroidia | Bacteroidetes |

Bifidobacterium pullorum subsp.

gallinarum (HG3A.1171)

T90

0.038

0.029

0.068

Bifidobacterium 3364 pullorum subsp.

gallinarum

Bifidobacterium

pullorum

Bifidobacterium Bifidobacteriaceae Bifidobacteriales Actinomycetia Actinobacteria

| Clostridiaceae sp. (HG3A.0608) | T90 | -0.038 | 0.029 | 0.068 | 3364 | unclassified | unclassified | unclassified | Clostridiaceae | Eubacteriales | Clostridia | Firmicutes |
| --- | --- | --- | --- | --- | --- | --- | --- | --- | --- | --- | --- | --- |
| Eubacteriales sp. (HG3A.0502) | ODI | -0.037 | 0.031 | 0.068 | 3364 | unclassified | unclassified | unclassified | unclassified | Eubacteriales | Clostridia | Firmicutes |
| Eubacteriales sp. (HG3A.0764) | AHI | -0.041 | 0.026 | 0.068 | 3004 | unclassified | unclassified | unclassified | unclassified | Eubacteriales | Clostridia | Firmicutes |
| Eubacteriales sp. (HG3A.0897) | ODI | -0.037 | 0.031 | 0.068 | 3364 | unclassified | unclassified | unclassified | unclassified | Eubacteriales | Clostridia | Firmicutes |
| Eubacteriales sp. (HG3A.1191) | AHI | -0.041 | 0.026 | 0.068 | 3004 | unclassified | unclassified | unclassified | unclassified | Eubacteriales | Clostridia | Firmicutes |
| Eubacteriales sp. (HG3A.1320) | T90 | -0.038 | 0.029 | 0.068 | 3364 | unclassified | unclassified | unclassified | unclassified | Eubacteriales | Clostridia | Firmicutes |
| Intestinimonas sp. (HG3A.1149) | T90 | -0.038 | 0.029 | 0.068 | 3364 | unclassified | unclassified | Intestinimonas | unclassified | Eubacteriales | Clostridia | Firmicutes |
| Parabacteroides goldsteinii (HG3A.0279) | T90 | -0.038 | 0.029 | 0.068 | 3364 | unclassified | Parabacteroides goldsteinii | Parabacteroides | Tannerellaceae | Bacteroidales | Bacteroidia | Bacteroidetes |
| Proteobacteria sp. (HG3A.0327) | AHI | -0.041 | 0.026 | 0.068 | 3004 | unclassified | unclassified | unclassified | unclassified | unclassified | unclassified | Proteobacteria |
| Proteobacteria sp. (HG3A.0360) | AHI | -0.041 | 0.026 | 0.068 | 3004 | unclassified | unclassified | unclassified | unclassified | unclassified | unclassified | Proteobacteria |
| Clostridia sp. (HG3A.1038) | AHI | -0.041 | 0.027 | 0.069 | 3004 | unclassified | unclassified | unclassified | unclassified | unclassified | Clostridia | Firmicutes |
| Clostridia sp. (HG3A.1346) | AHI | -0.041 | 0.026 | 0.069 | 3004 | unclassified | unclassified | unclassified | unclassified | unclassified | Clostridia | Firmicutes |
| Clostridiaceae sp. (HG3A.0491) | AHI | 0.041 | 0.026 | 0.069 | 3004 | unclassified | unclassified | unclassified | Clostridiaceae | Eubacteriales | Clostridia | Firmicutes |
| Coprobacillus cateniformis (HG3A.0456) | AHI | 0.041 | 0.027 | 0.069 | 3004 | unclassified | Coprobacillus cateniformis | Coprobacillus | Coprobacillaceae | Erysipelotrichales | Erysipelotrichia | Firmicutes |
| Eubacteriales sp. (HG3A.0392) | AHI | -0.041 | 0.026 | 0.069 | 3004 | unclassified | unclassified | unclassified | unclassified | Eubacteriales | Clostridia | Firmicutes |
| Eubacteriales sp. (HG3A.0510) | AHI | -0.041 | 0.026 | 0.069 | 3004 | unclassified | unclassified | unclassified | unclassified | Eubacteriales | Clostridia | Firmicutes |
| Eubacteriales sp. (HG3A.0663) | AHI | -0.041 | 0.026 | 0.069 | 3004 | unclassified | unclassified | unclassified | unclassified | Eubacteriales | Clostridia | Firmicutes |
| Eubacteriales sp. (HG3A.0785) | T90 | -0.038 | 0.03 | 0.069 | 3364 | unclassified | unclassified | unclassified | unclassified | Eubacteriales | Clostridia | Firmicutes |
| Eubacteriales sp. (HG3A.0914) | AHI | -0.041 | 0.027 | 0.069 | 3004 | unclassified | unclassified | unclassified | unclassified | Eubacteriales | Clostridia | Firmicutes |
| Eubacteriales sp. (HG3A.0947) | T90 | -0.038 | 0.03 | 0.069 | 3364 | unclassified | unclassified | unclassified | unclassified | Eubacteriales | Clostridia | Firmicutes |

| Eubacteriales sp. (HG3A.1006) | AHI | -0.041 | 0.026 | 0.069 | 3004 | unclassified | unclassified | unclassified | unclassified | Eubacteriales | Clostridia | Firmicutes |
| --- | --- | --- | --- | --- | --- | --- | --- | --- | --- | --- | --- | --- |
| Eubacteriales sp. (HG3A.1250) | AHI | -0.041 | 0.026 | 0.069 | 3004 | unclassified | unclassified | unclassified | unclassified | Eubacteriales | Clostridia | Firmicutes |
| Firmicutes sp. (HG3A.0860) | AHI | -0.041 | 0.026 | 0.069 | 3004 | unclassified | unclassified | unclassified | unclassified | unclassified | unclassified | Firmicutes |
| Lachnospiraceae sp. (HG3A.0246) | T90 | -0.038 | 0.03 | 0.069 | 3364 | unclassified | unclassified | unclassified | Lachnospiraceae | Eubacteriales | Clostridia | Firmicutes |
| Oscillospiraceae sp. (HG3A.0780) | T90 | -0.038 | 0.03 | 0.069 | 3364 | unclassified | unclassified | unclassified | Oscillospiraceae | Eubacteriales | Clostridia | Firmicutes |
| Eubacteriales sp. (HG3A.0061) | T90 | 0.038 | 0.03 | 0.07 | 3364 | unclassified | unclassified | unclassified | unclassified | Eubacteriales | Clostridia | Firmicutes |
| Eubacteriales sp. (HG3A.0702) | T90 | -0.038 | 0.03 | 0.07 | 3364 | unclassified | unclassified | unclassified | unclassified | Eubacteriales | Clostridia | Firmicutes |
| Eubacteriales sp. (HG3A.0827) | ODI | -0.037 | 0.032 | 0.07 | 3364 | unclassified | unclassified | unclassified | unclassified | Eubacteriales | Clostridia | Firmicutes |
| Firmicutes sp. (HG3A.1115) | ODI | -0.037 | 0.032 | 0.07 | 3364 | unclassified | unclassified | unclassified | unclassified | unclassified | unclassified | Firmicutes |
| Bacteroides intestinalis (HG3A.0265) | AHI | -0.041 | 0.028 | 0.071 | 3004 | unclassified | Bacteroides intestinalis | Bacteroides | Bacteroidaceae | Bacteroidales | Bacteroidia | Bacteroidetes |
| Clostridia sp. (HG3A.1609) | ODI | -0.037 | 0.033 | 0.071 | 3364 | unclassified | unclassified | unclassified | unclassified | unclassified | Clostridia | Firmicutes |
| Eubacteriales sp. (HG3A.1042) | AHI | -0.041 | 0.028 | 0.071 | 3004 | unclassified | unclassified | unclassified | unclassified | Eubacteriales | Clostridia | Firmicutes |
| Faecalibacterium prausnitzii  (HG3A.0029) | ODI | 0.037 | 0.033 | 0.071 | 3364 | unclassified | Faecalibacterium  prausnitzii | Faecalibacterium | Oscillospiraceae | Eubacteriales | Clostridia | Firmicutes |
| Faecalicatena orotica (HG3A.1910) | T90 | 0.038 | 0.031 | 0.071 | 3364 | unclassified | Faecalicatena orotica | Faecalicatena | Lachnospiraceae | Eubacteriales | Clostridia | Firmicutes |
| Firmicutes sp. (HG3A.0810) | ODI | -0.037 | 0.033 | 0.071 | 3364 | unclassified | unclassified | unclassified | unclassified | unclassified | unclassified | Firmicutes |
| Latilactobacillus curvatus (HG3A.1505) | ODI | 0.037 | 0.033 | 0.071 | 3364 | unclassified | Latilactobacillus curvatus | Latilactobacillus | Lactobacillaceae | Lactobacillales | Bacilli | Firmicutes |
| Alistipes dispar (HG3A.0281) | T90 | -0.037 | 0.031 | 0.072 | 3364 | unclassified | Alistipes dispar | Alistipes | Rikenellaceae | Bacteroidales | Bacteroidia | Bacteroidetes |
| Bacteria sp. (HG3A.1349) | AHI | -0.04 | 0.028 | 0.072 | 3004 | unclassified | unclassified | unclassified | unclassified | unclassified | unclassified | unclassified |
| Clostridia sp. (HG3A.1530) | T90 | -0.037 | 0.031 | 0.072 | 3364 | unclassified | unclassified | unclassified | unclassified | unclassified | Clostridia | Firmicutes |
| Erysipelotrichales sp. (HG3A.0737) | ODI | 0.037 | 0.033 | 0.072 | 3364 | unclassified | unclassified | unclassified | unclassified | Erysipelotrichales | Erysipelotrichia | Firmicutes |
| Hungatella hathewayi  (HG3A.0451) | T90 | 0.037 | 0.031 | 0.072 | 3364 | unclassified | Hungatella hathewayi | Hungatella | Clostridiaceae | Eubacteriales | Clostridia | Firmicutes |
| Ruminococcus sp. (HG3A.0337) | T90 | -0.037 | 0.031 | 0.072 | 3364 | unclassified | unclassified | Ruminococcus | Oscillospiraceae | Eubacteriales | Clostridia | Firmicutes |

| Bacteroidales sp. (HG3A.0818) | T90 | -0.037 | 0.032 | 0.073 | 3364 | unclassified | unclassified | unclassified | unclassified | Bacteroidales | Bacteroidia | Bacteroidetes |
| --- | --- | --- | --- | --- | --- | --- | --- | --- | --- | --- | --- | --- |
| Eubacteriales sp. (HG3A.0345) | AHI | -0.04 | 0.029 | 0.073 | 3004 | unclassified | unclassified | unclassified | unclassified | Eubacteriales | Clostridia | Firmicutes |
| Eubacteriales sp. (HG3A.0438) | AHI | 0.04 | 0.029 | 0.073 | 3004 | unclassified | unclassified | unclassified | unclassified | Eubacteriales | Clostridia | Firmicutes |
| Eubacteriales sp. (HG3A.0502) | AHI | -0.04 | 0.029 | 0.073 | 3004 | unclassified | unclassified | unclassified | unclassified | Eubacteriales | Clostridia | Firmicutes |
| Eubacteriales sp. (HG3A.1459) | T90 | 0.037 | 0.032 | 0.073 | 3364 | unclassified | unclassified | unclassified | unclassified | Eubacteriales | Clostridia | Firmicutes |
| Oscillospiraceae sp. (HG3A.0648) | T90 | -0.037 | 0.032 | 0.073 | 3364 | unclassified | unclassified | unclassified | Oscillospiraceae | Eubacteriales | Clostridia | Firmicutes |
| Roseburia faecis (HG3A.0058) | AHI | 0.04 | 0.029 | 0.073 | 3004 | unclassified | Roseburia faecis | Roseburia | Lachnospiraceae | Eubacteriales | Clostridia | Firmicutes |
| Sutterellaceae sp. (HG3A.1122) | AHI | 0.04 | 0.029 | 0.073 | 3004 | unclassified | unclassified | unclassified | Sutterellaceae | Burkholderiales | Betaproteobacte ria | Proteobacteria |
| Bacteria sp. (HG3A.1251) | T90 | -0.037 | 0.032 | 0.074 | 3364 | unclassified | unclassified | unclassified | unclassified | unclassified | unclassified | unclassified |
| Bacteroides fragilis (HG3A.0745) | AHI | 0.04 | 0.029 | 0.074 | 3004 | unclassified | Bacteroides fragilis | Bacteroides | Bacteroidaceae | Bacteroidales | Bacteroidia | Bacteroidetes |
| Clostridia sp. (HG3A.1362) | ODI | -0.037 | 0.034 | 0.074 | 3364 | unclassified | unclassified | unclassified | unclassified | unclassified | Clostridia | Firmicutes |
| Eubacteriales sp. (HG3A.0740) | T90 | -0.037 | 0.032 | 0.074 | 3364 | unclassified | unclassified | unclassified | unclassified | Eubacteriales | Clostridia | Firmicutes |
| Eubacteriales sp. (HG3A.1351) | T90 | -0.037 | 0.032 | 0.074 | 3364 | unclassified | unclassified | unclassified | unclassified | Eubacteriales | Clostridia | Firmicutes |
| Eubacterium sp. AF17-7 (HG3A.0165) | T90 | -0.037 | 0.032 | 0.074 | 3364 | unclassified | Eubacterium sp.  AF17-7 | Eubacterium | Eubacteriaceae | Eubacteriales | Clostridia | Firmicutes |
| Bacteroides uniformis  (HG3A.0007) | ODI | -0.037 | 0.035 | 0.075 | 3364 | unclassified | Bacteroides  uniformis | Bacteroides | Bacteroidaceae | Bacteroidales | Bacteroidia | Bacteroidetes |
| Eubacteriales sp. (HG3A.0348) | ODI | -0.037 | 0.035 | 0.075 | 3364 | unclassified | unclassified | unclassified | unclassified | Eubacteriales | Clostridia | Firmicutes |
| Eubacteriales sp. (HG3A.0392) | T90 | -0.037 | 0.033 | 0.075 | 3364 | unclassified | unclassified | unclassified | unclassified | Eubacteriales | Clostridia | Firmicutes |
| Eubacteriales sp. (HG3A.0736) | AHI | -0.04 | 0.029 | 0.075 | 3004 | unclassified | unclassified | unclassified | unclassified | Eubacteriales | Clostridia | Firmicutes |
| Eubacteriales sp. (HG3A.0740) | AHI | -0.04 | 0.03 | 0.075 | 3004 | unclassified | unclassified | unclassified | unclassified | Eubacteriales | Clostridia | Firmicutes |
| Eubacteriales sp. (HG3A.0807) | AHI | -0.04 | 0.029 | 0.075 | 3004 | unclassified | unclassified | unclassified | unclassified | Eubacteriales | Clostridia | Firmicutes |
| Eubacteriales sp. (HG3A.0851) | ODI | -0.037 | 0.035 | 0.075 | 3364 | unclassified | unclassified | unclassified | unclassified | Eubacteriales | Clostridia | Firmicutes |
| Eubacteriales sp. (HG3A.1072) | AHI | -0.04 | 0.029 | 0.075 | 3004 | unclassified | unclassified | unclassified | unclassified | Eubacteriales | Clostridia | Firmicutes |

| Eubacteriales sp. (HG3A.1351) | ODI | -0.037 | 0.035 | 0.075 | 3364 | unclassified | unclassified | unclassified | unclassified | Eubacteriales | Clostridia | Firmicutes |
| --- | --- | --- | --- | --- | --- | --- | --- | --- | --- | --- | --- | --- |
| Eubacteriales sp. (HG3A.1419) | ODI | -0.037 | 0.035 | 0.075 | 3364 | unclassified | unclassified | unclassified | unclassified | Eubacteriales | Clostridia | Firmicutes |
| Klebsiella quasipneumoniae subsp. similipneumoniae (HG3A.1629) | T90 | 0.037 | 0.033 | 0.075 | 3364 | Klebsiella quasipneumoniae subsp.  similipneumoniae | Klebsiella quasipneumoniae | Klebsiella | Enterobacteriacea e | Enterobacterales | Gammaproteob acteria | Proteobacteria |
| Oscillospiraceae sp. (HG3A.1133) | ODI | 0.037 | 0.035 | 0.075 | 3364 | unclassified | unclassified | unclassified | Oscillospiraceae | Eubacteriales | Clostridia | Firmicutes |
| Oscillospiraceae sp. (HG3A.1588) | ODI | -0.037 | 0.035 | 0.075 | 3364 | unclassified | unclassified | unclassified | Oscillospiraceae | Eubacteriales | Clostridia | Firmicutes |
| Prevotella colorans (HG3A.1470) | ODI | -0.037 | 0.035 | 0.075 | 3364 | unclassified | Prevotella colorans | Prevotella | Prevotellaceae | Bacteroidales | Bacteroidia | Bacteroidetes |
| Alistipes putredinis (HG3A.0033) | AHI | -0.04 | 0.03 | 0.076 | 3004 | unclassified | Alistipes putredinis | Alistipes | Rikenellaceae | Bacteroidales | Bacteroidia | Bacteroidetes |
| Atopobiaceae sp. (HG3A.0704) | ODI | 0.037 | 0.036 | 0.076 | 3364 | unclassified | unclassified | unclassified | Atopobiaceae | Coriobacteriales | Coriobacteriia | Actinobacteria |
| Bacteria sp. (HG3A.1251) | AHI | -0.04 | 0.03 | 0.076 | 3004 | unclassified | unclassified | unclassified | unclassified | unclassified | unclassified | unclassified |
| Eubacteriales sp. (HG3A.1419) | AHI | -0.04 | 0.03 | 0.076 | 3004 | unclassified | unclassified | unclassified | unclassified | Eubacteriales | Clostridia | Firmicutes |
| Eubacterium sp. AF34-35BH  (HG3A.0231) | T90 | -0.037 | 0.033 | 0.076 | 3364 | unclassified | Eubacterium sp.  AF34-35BH | Eubacterium | Eubacteriaceae | Eubacteriales | Clostridia | Firmicutes |
| Alistipes onderdonkii subsp. vulgaris (HG3A.0047) | ODI | -0.036 | 0.036 | 0.077 | 3364 | Alistipes onderdonkii subsp. vulgaris | Alistipes onderdonkii | Alistipes | Rikenellaceae | Bacteroidales | Bacteroidia | Bacteroidetes |
| Eubacteriales sp. (HG3A.1313) | AHI | -0.04 | 0.031 | 0.077 | 3004 | unclassified | unclassified | unclassified | unclassified | Eubacteriales | Clostridia | Firmicutes |
| Turicimonas muris (HG3A.1338) | ODI | -0.036 | 0.036 | 0.077 | 3364 | unclassified | Turicimonas muris | Turicimonas | Sutterellaceae | Burkholderiales | Betaproteobacte ria | Proteobacteria |
| Clostridia sp. (HG3A.1346) | ODI | -0.036 | 0.037 | 0.078 | 3364 | unclassified | unclassified | unclassified | unclassified | unclassified | Clostridia | Firmicutes |
| Eubacteriales sp. (HG3A.0666) | ODI | -0.036 | 0.037 | 0.078 | 3364 | unclassified | unclassified | unclassified | unclassified | Eubacteriales | Clostridia | Firmicutes |
| Butyricimonas sp. Marseille- P3923 (HG3A.1206) | ODI | -0.036 | 0.038 | 0.079 | 3364 | unclassified | Butyricimonas sp. Marseille-P3923 | Butyricimonas | Odoribacteraceae | Bacteroidales | Bacteroidia | Bacteroidetes |
| Eubacteriales sp. (HG3A.0204) | T90 | -0.037 | 0.035 | 0.079 | 3364 | unclassified | unclassified | unclassified | unclassified | Eubacteriales | Clostridia | Firmicutes |
| Eubacteriales sp. (HG3A.1147) | ODI | -0.036 | 0.038 | 0.079 | 3364 | unclassified | unclassified | unclassified | unclassified | Eubacteriales | Clostridia | Firmicutes |
| Firmicutes sp. (HG3A.1222) | ODI | -0.036 | 0.037 | 0.079 | 3364 | unclassified | unclassified | unclassified | unclassified | unclassified | unclassified | Firmicutes |

| Leuconostoc lactis (HG3A.1458) | AHI | 0.04 | 0.031 | 0.079 | 3004 | unclassified | Leuconostoc lactis | Leuconostoc | Lactobacillaceae | Lactobacillales | Bacilli | Firmicutes |
| --- | --- | --- | --- | --- | --- | --- | --- | --- | --- | --- | --- | --- |
| Phocaeicola barnesiae (HG3A.1475) | AHI | -0.04 | 0.031 | 0.079 | 3004 | unclassified | Phocaeicola barnesiae | Phocaeicola | unclassified | Bacteroidales | Bacteroidia | Bacteroidetes |
| Candidatus Borkfalkiales sp.  (HG3A.1284) | T90 | -0.037 | 0.035 | 0.08 | 3364 | unclassified | unclassified | unclassified | unclassified | Candidatus  Borkfalkiales | Clostridia | Firmicutes |
| Eubacteriales sp. (HG3A.0468) | AHI | -0.04 | 0.032 | 0.08 | 3004 | unclassified | unclassified | unclassified | unclassified | Eubacteriales | Clostridia | Firmicutes |
| Eubacteriales sp. (HG3A.0786) | AHI | 0.04 | 0.032 | 0.08 | 3004 | unclassified | unclassified | unclassified | unclassified | Eubacteriales | Clostridia | Firmicutes |
| Eubacteriales sp. (HG3A.1226) | T90 | -0.037 | 0.035 | 0.08 | 3364 | unclassified | unclassified | unclassified | unclassified | Eubacteriales | Clostridia | Firmicutes |
| Lachnospiraceae sp. (HG3A.1435) | AHI | -0.04 | 0.032 | 0.08 | 3004 | unclassified | unclassified | unclassified | Lachnospiraceae | Eubacteriales | Clostridia | Firmicutes |
| Bacteria sp. (HG3A.1274) | ODI | -0.036 | 0.038 | 0.081 | 3364 | unclassified | unclassified | unclassified | unclassified | unclassified | unclassified | unclassified |
| Bacteroidales sp. (HG3A.0452) | AHI | -0.039 | 0.033 | 0.081 | 3004 | unclassified | unclassified | unclassified | unclassified | Bacteroidales | Bacteroidia | Bacteroidetes |
| Betaproteobacteria sp. (HG3A.1138) | T90 | 0.037 | 0.036 | 0.081 | 3364 | unclassified | unclassified | unclassified | unclassified | unclassified | Betaproteobacte ria | Proteobacteria |
| Clostridia sp. (HG3A.0845) | AHI | -0.039 | 0.033 | 0.081 | 3004 | unclassified | unclassified | unclassified | unclassified | unclassified | Clostridia | Firmicutes |
| Eubacteriales sp. (HG3A.0296) | ODI | -0.036 | 0.039 | 0.081 | 3364 | unclassified | unclassified | unclassified | unclassified | Eubacteriales | Clostridia | Firmicutes |
| Eubacteriales sp. (HG3A.0306) | ODI | -0.036 | 0.039 | 0.081 | 3364 | unclassified | unclassified | unclassified | unclassified | Eubacteriales | Clostridia | Firmicutes |
| Eubacteriales sp. (HG3A.1257) | AHI | -0.039 | 0.033 | 0.081 | 3004 | unclassified | unclassified | unclassified | unclassified | Eubacteriales | Clostridia | Firmicutes |
| Hungatella hathewayi  (HG3A.0455) | AHI | 0.039 | 0.033 | 0.081 | 3004 | unclassified | Hungatella hathewayi | Hungatella | Clostridiaceae | Eubacteriales | Clostridia | Firmicutes |
| Alistipes indistinctus (HG3A.0121) | T90 | -0.036 | 0.036 | 0.082 | 3364 | unclassified | Alistipes indistinctus | Alistipes | Rikenellaceae | Bacteroidales | Bacteroidia | Bacteroidetes |
| Clostridia sp. (HG3A.1286) | ODI | -0.036 | 0.039 | 0.082 | 3364 | unclassified | unclassified | unclassified | unclassified | unclassified | Clostridia | Firmicutes |
| Clostridium perfringens (HG3A.0959) | ODI | 0.036 | 0.039 | 0.082 | 3364 | unclassified | Clostridium perfringens | Clostridium | Clostridiaceae | Eubacteriales | Clostridia | Firmicutes |
| Eubacteriales sp. (HG3A.0148) | T90 | -0.036 | 0.036 | 0.082 | 3364 | unclassified | unclassified | unclassified | unclassified | Eubacteriales | Clostridia | Firmicutes |
| Eubacteriales sp. (HG3A.0480) | AHI | -0.039 | 0.033 | 0.082 | 3004 | unclassified | unclassified | unclassified | unclassified | Eubacteriales | Clostridia | Firmicutes |
| Eubacteriales sp. (HG3A.0606) | AHI | -0.039 | 0.033 | 0.082 | 3004 | unclassified | unclassified | unclassified | unclassified | Eubacteriales | Clostridia | Firmicutes |
| Eubacteriales sp. (HG3A.0717) | T90 | -0.036 | 0.036 | 0.082 | 3364 | unclassified | unclassified | unclassified | unclassified | Eubacteriales | Clostridia | Firmicutes |

Lachnospiraceae sp. (HG3A.1209) ODI -0.036 0.039 0.082 3364 unclassified unclassified unclassified Lachnospiraceae Eubacteriales Clostridia Firmicutes

Megamonas funiformis

(HG3A.0684)

T90

0.036

0.036

0.082 3364 unclassified

Megamonas

funiformis

Megamonas

Selenomonadacea Selenomonadales

e

Negativicutes Firmicutes

| Alistipes sp. An66 (HG3A.1535) | T90 | -0.036 | 0.037 | 0.083 | 3364 | unclassified | Alistipes sp. An66 | Alistipes | Rikenellaceae | Bacteroidales | Bacteroidia | Bacteroidetes |
| --- | --- | --- | --- | --- | --- | --- | --- | --- | --- | --- | --- | --- |
| Clostridia sp. (HG3A.1625) | T90 | -0.036 | 0.037 | 0.083 | 3364 | unclassified | unclassified | unclassified | unclassified | unclassified | Clostridia | Firmicutes |
| Eubacteriales sp. (HG3A.0369) | AHI | -0.039 | 0.034 | 0.083 | 3004 | unclassified | unclassified | unclassified | unclassified | Eubacteriales | Clostridia | Firmicutes |
| Firmicutes sp. (HG3A.0811) | ODI | -0.036 | 0.04 | 0.083 | 3364 | unclassified | unclassified | unclassified | unclassified | unclassified | unclassified | Firmicutes |
| Firmicutes sp. (HG3A.1162) | AHI | -0.039 | 0.034 | 0.083 | 3004 | unclassified | unclassified | unclassified | unclassified | unclassified | unclassified | Firmicutes |

Lactobacillus delbrueckii subsp.

bulgaricus (HG3A.0465)

ODI

-0.036

0.04

0.083

Lactobacillus 3364 delbrueckii subsp.

bulgaricus

Lactobacillus

delbrueckii

Lactobacillus Lactobacillaceae Lactobacillales

Bacilli

Firmicutes

Parasutterella sp. (HG3A.0485) T90 -0.036 0.037 0.083 3364 unclassified unclassified Parasutterella Sutterellaceae Burkholderiales Betaproteobacte

ria

Proteobacteria

Betaproteobacteria sp.

(HG3A.1138)

ODI

0.036

0.041

0.084 3364 unclassified

unclassified

unclassified

unclassified

unclassified

Betaproteobacte Proteobacteria

ria

Clostridia sp. (HG3A.1143) AHI -0.039 0.034 0.084 3004 unclassified unclassified unclassified unclassified unclassified Clostridia Firmicutes

Desulfovibrio fairfieldensis

(HG3A.0529)

AHI

-0.039

0.034

0.084 3004 unclassified

Desulfovibrio

fairfieldensis

Desulfovibrio

Desulfovibrionace Desulfovibrionales Deltaproteobact

ae

eria

Proteobacteria

Eubacteriales sp. (HG3A.0543) T90 -0.036 0.037 0.084 3364 unclassified unclassified unclassified unclassified Eubacteriales Clostridia Firmicutes

Eubacteriales sp. (HG3A.0851)

AHI

-0.039

0.034

0.084 3004 unclassified

unclassified

unclassified

unclassified

Eubacteriales

Clostridia

Firmicutes

Klebsiella quasipneumoniae subsp. similipneumoniae (HG3A.1629)

ODI 0.036 0.04 0.084 3364

Klebsiella

quasipneumoniae subsp. similipneumoniae

Klebsiella quasipneumoniae

Klebsiella Enterobacteriacea

e

Enterobacterales Gammaproteob

acteria

Proteobacteria

Bacteria sp. (HG3A.1780) AHI -0.039 0.035 0.085 3004 unclassified unclassified unclassified unclassified unclassified unclassified unclassified Clostridia sp. (HG3A.1585) T90 -0.036 0.038 0.085 3364 unclassified unclassified unclassified unclassified unclassified Clostridia Firmicutes

[Clostridium] spiroforme

(HG3A.0259)

ODI

0.036

0.041

0.085 3364 unclassified

[Clostridium]

spiroforme

Erysipelatoclostridiu Erysipelotrichace

m

ae

Erysipelotrichales Erysipelotrichia Firmicutes

Eubacteriales sp. (HG3A.0528) T90 -0.036 0.038 0.085 3364 unclassified unclassified unclassified unclassified Eubacteriales Clostridia Firmicutes

Megasphaera elsdenii

(HG3A.0921)

T90

0.036

0.038

0.085 3364 unclassified

Megasphaera

elsdenii

Megasphaera

Veillonellaceae

Veillonellales Negativicutes Firmicutes

Oscillospiraceae sp. (HG3A.0098) T90 0.036 0.038 0.085 3364 unclassified unclassified unclassified Oscillospiraceae Eubacteriales Clostridia Firmicutes

Ruminococcus sp. AM28-41

(HG3A.0631)

ODI

0.036

0.041

0.085 3364 unclassified

Ruminococcus sp.

AM28-41

Ruminococcus Oscillospiraceae Eubacteriales

Clostridia

Firmicutes

Streptococcus

| Streptococcus gallolyticus subsp.  gallolyticus (HG3A.1651) | AHI | 0.039 | 0.035 | 0.085 | 3004 | gallolyticus  subsp. | Streptococcus  gallolyticus | Streptococcus | Streptococcaceae | Lactobacillales | Bacilli | Firmicutes |
| --- | --- | --- | --- | --- | --- | --- | --- | --- | --- | --- | --- | --- |
|  |  |  |  |  |  | gallolyticus |  |  |  |  |  |  |
| Bacteria sp. (HG3A.1553) | ODI | -0.035 | 0.042 | 0.086 | 3364 | unclassified | unclassified | unclassified | unclassified | unclassified | unclassified | unclassified |
| Blautia hansenii (HG3A.0981) | ODI | 0.035 | 0.042 | 0.086 | 3364 | unclassified | Blautia hansenii | Blautia | Lachnospiraceae | Eubacteriales | Clostridia | Firmicutes |
| Clostridia sp. (HG3A.1427) | AHI | -0.039 | 0.035 | 0.086 | 3004 | unclassified | unclassified | unclassified | unclassified | unclassified | Clostridia | Firmicutes |
| Eubacteriales sp. (HG3A.0103) | ODI | -0.035 | 0.042 | 0.086 | 3364 | unclassified | unclassified | unclassified | unclassified | Eubacteriales | Clostridia | Firmicutes |
| Eubacteriales sp. (HG3A.0846) | ODI | -0.035 | 0.042 | 0.086 | 3364 | unclassified | unclassified | unclassified | unclassified | Eubacteriales | Clostridia | Firmicutes |
| Eubacteriales sp. (HG3A.1087) | AHI | -0.039 | 0.035 | 0.086 | 3004 | unclassified | unclassified | unclassified | unclassified | Eubacteriales | Clostridia | Firmicutes |
| Eubacteriales sp. (HG3A.1292) | T90 | -0.036 | 0.039 | 0.086 | 3364 | unclassified | unclassified | unclassified | unclassified | Eubacteriales | Clostridia | Firmicutes |
[truncated: 492,270 more chars]
