# Supplementary material for: OSA Is Associated With the Human Gut Microbiota Composition and Functional Potential in the Population-Based Swedish CardioPulmonary bioImage Study
Source: Chest. 2023 Mar 15;164(2):503–16. doi: 10.1016/j.chest.2023.03.010 (PMC10410248; doi:10.1016/j.chest.2023.03.010)
Supplement: e-Table 6 [file mmc3.docx]

e-Table 6. Partial Spearman’s correlations of AHI, T90, and ODI with microbiota species using the complete main model

Only the species identified in the model not adjusted for body mass index (BMI) were included in this analysis. Associations adjusted for age, sex, smoking, alcohol intake, BMI, and DNA extraction plate. Adjustment for multiple testing using the Benjamini-Hochberg method and presented as q- values. Under the column "Metagenomics species", the information between pararenthesis is the internal identifier for the respective species. AHI: apnea- hypopnea index; ODI: oxygen desaturation index; and T90: percentage of time with oxygen saturation below 90%.

**Metagenomic species exposure**

**Spearman's**

**correlation p-value q-value N subspecies species genus family order class phylum**

Oscillospiraceae sp. (HG3A.0207)

| Blautia obeum (HG3A.0001) | T90 | 0.092 | 1.32E-07 | 4.45E-05 | 3364 unclassified | Blautia obeum | Blautia | Lachnospiraceae | Eubacteriales | Clostridia | Firmicutes |
| --- | --- | --- | --- | --- | --- | --- | --- | --- | --- | --- | --- |
| Dorea formicigenerans (HG3A.0006) | T90 | 0.093 | 8.58E-08 | 4.45E-05 | 3364 unclassified | Dorea formicigenerans | Dorea | Lachnospiraceae | Eubacteriales | Clostridia | Firmicutes |
| Mediterraneibacter glycyrrhizinilyticus (HG3A.0314) | T90 | 0.091 | 1.70E-07 | 4.45E-05 | 3364 unclassified | Mediterraneibacter glycyrrhizinilyticus | Mediterraneibact er | Lachnospiraceae | Eubacteriales | Clostridia | Firmicutes |
| Eubacteriales sp.  (HG3A.0100) | ODI | -0.091 | 1.86E-07 | 7.32E-05 | 3364 unclassified | unclassified | unclassified | unclassified | Eubacteriales | Clostridia | Firmicutes |
| Oscillospiraceae sp. (HG3A.0207) | T90 | -0.086 | 6.58E-07 | 1.30E-04 | 3364 unclassified | unclassified | unclassified | Oscillospiraceae | Eubacteriales | Clostridia | Firmicutes |
| Blautia obeum  (HG3A.0001) | ODI | 0.084 | 1.45E-06 | 2.45E-04 | 3364 unclassified | Blautia obeum | Blautia | Lachnospiraceae | Eubacteriales | Clostridia | Firmicutes |
| Clostridia sp. (HG3A.0515) | ODI | -0.083 | 1.74E-06 | 2.45E-04 | 3364 unclassified | unclassified | unclassified | unclassified | unclassified | Clostridia | Firmicutes |
| Eubacteriales sp.  (HG3A.0069) | ODI | -0.083 | 1.87E-06 | 2.45E-04 | 3364 unclassified | unclassified | unclassified | unclassified | Eubacteriales | Clostridia | Firmicutes |
| Eubacteriales sp. (HG3A.0442) | ODI | -0.085 | 1.09E-06 | 2.45E-04 | 3364 unclassified | unclassified | unclassified | unclassified | Eubacteriales | Clostridia | Firmicutes |
| Eubacteriales sp.  (HG3A.0162) | T90 | -0.083 | 1.63E-06 | 2.57E-04 | 3364 unclassified | unclassified | unclassified | unclassified | Eubacteriales | Clostridia | Firmicutes |
| Eubacteriales sp. (HG3A.0242) | T90 | -0.082 | 2.16E-06 | 2.83E-04 | 3364 unclassified | unclassified | unclassified | unclassified | Eubacteriales | Clostridia | Firmicutes |
| Eubacteriales sp.  (HG3A.0149) | ODI | -0.082 | 2.70E-06 | 3.04E-04 | 3364 unclassified | unclassified | unclassified | unclassified | Eubacteriales | Clostridia | Firmicutes |
| Eubacteriales sp. (HG3A.0506) | ODI | -0.081 | 3.44E-06 | 3.39E-04 | 3364 unclassified | unclassified | unclassified | unclassified | Eubacteriales | Clostridia | Firmicutes |
| Eubacteriales sp.  (HG3A.0156) | ODI | -0.079 | 5.78E-06 | 4.81E-04 | 3364 unclassified | unclassified | unclassified | unclassified | Eubacteriales | Clostridia | Firmicutes |
| Eubacteriales sp. (HG3A.0197) | ODI | -0.078 | 6.91E-06 | 4.81E-04 | 3364 unclassified | unclassified | unclassified | unclassified | Eubacteriales | Clostridia | Firmicutes |

ODI -0.096 3.59E-08 2.83E-05 3364 unclassified unclassified unclassified Oscillospiraceae Eubacteriales Clostridia Firmicutes

Oscillospiraceae sp. (HG3A.0072)

| [Ruminococcus] gnavus (HG3A.0239) | ODI | 0.078 | 7.32E-06 | 4.81E-04 | 3364 unclassified | [Ruminococcus] gnavus | Mediterraneibact er | Lachnospiraceae | Eubacteriales | Clostridia | Firmicutes |
| --- | --- | --- | --- | --- | --- | --- | --- | --- | --- | --- | --- |
| Eubacteriales sp.  (HG3A.0226) | ODI | -0.077 | 9.09E-06 | 5.51E-04 | 3364 unclassified | unclassified | unclassified | unclassified | Eubacteriales | Clostridia | Firmicutes |
| Eubacteriales sp. (HG3A.0162) | ODI | -0.077 | 1.05E-05 | 5.92E-04 | 3364 unclassified | unclassified | unclassified | unclassified | Eubacteriales | Clostridia | Firmicutes |
| Clostridia sp.  (HG3A.0140) | ODI | -0.076 | 1.19E-05 | 6.27E-04 | 3364 unclassified | unclassified | unclassified | unclassified | unclassified | Clostridia | Firmicutes |
| Eubacteriales sp. (HG3A.0196) | ODI | -0.075 | 1.41E-05 | 6.97E-04 | 3364 unclassified | unclassified | unclassified | unclassified | Eubacteriales | Clostridia | Firmicutes |
| Bacteria sp.  (HG3A.0483) | ODI | -0.075 | 1.69E-05 | 7.36E-04 | 3364 unclassified | unclassified | unclassified | unclassified | unclassified | unclassified | unclassified |
| Firmicutes sp. (HG3A.0397) | ODI | -0.075 | 1.69E-05 | 7.36E-04 | 3364 unclassified | unclassified | unclassified | unclassified | unclassified | unclassified | Firmicutes |
| Firmicutes sp.  (HG3A.0398) | ODI | -0.075 | 1.78E-05 | 7.36E-04 | 3364 unclassified | unclassified | unclassified | unclassified | unclassified | unclassified | Firmicutes |
| Eubacteriales sp. (HG3A.0242) | ODI | -0.074 | 1.98E-05 | 7.54E-04 | 3364 unclassified | unclassified | unclassified | unclassified | Eubacteriales | Clostridia | Firmicutes |
| Intestinibacillus sp. Marseille-P4005 (HG3A.0168) | ODI | 0.074 | 2.01E-05 | 7.54E-04 | 3364 unclassified | Intestinibacillus sp. Marseille-P4005 | Intestinibacillus | Eubacteriaceae | Eubacteriales | Clostridia | Firmicutes |
| Bacteria sp. (HG3A.0634) | ODI | -0.073 | 2.92E-05 | 0.001 | 3364 unclassified | unclassified | unclassified | unclassified | unclassified | unclassified | unclassified |
| Blautia massiliensis  (HG3A.0023) | ODI | 0.071 | 4.33E-05 | 0.001 | 3364 unclassified | Blautia massiliensis | Blautia | Lachnospiraceae | Eubacteriales | Clostridia | Firmicutes |
| Clostridia sp. (HG3A.0728) | ODI | -0.072 | 3.78E-05 | 0.001 | 3364 unclassified | unclassified | unclassified | unclassified | unclassified | Clostridia | Firmicutes |
| Coprococcus comes (HG3A.0016) | ODI | 0.071 | 4.06E-05 | 0.001 | 3364 unclassified | Coprococcus comes | Coprococcus | Lachnospiraceae | Eubacteriales | Clostridia | Firmicutes |
| Eubacteriales sp. (HG3A.0084) | ODI | -0.072 | 3.51E-05 | 0.001 | 3364 unclassified | unclassified | unclassified | unclassified | Eubacteriales | Clostridia | Firmicutes |
| Eubacteriales sp.  (HG3A.0085) | ODI | -0.071 | 4.36E-05 | 0.001 | 3364 unclassified | unclassified | unclassified | unclassified | Eubacteriales | Clostridia | Firmicutes |
| Eubacteriales sp. (HG3A.0118) | ODI | -0.07 | 5.36E-05 | 0.001 | 3364 unclassified | unclassified | unclassified | unclassified | Eubacteriales | Clostridia | Firmicutes |
| Eubacteriales sp.  (HG3A.0123) | T90 | 0.076 | 1.12E-05 | 0.001 | 3364 unclassified | unclassified | unclassified | unclassified | Eubacteriales | Clostridia | Firmicutes |
| Eubacteriales sp. (HG3A.0269) | ODI | -0.07 | 5.47E-05 | 0.001 | 3364 unclassified | unclassified | unclassified | unclassified | Eubacteriales | Clostridia | Firmicutes |

ODI -0.078 6.95E-06 4.81E-04 3364 unclassified unclassified unclassified Oscillospiraceae Eubacteriales Clostridia Firmicutes

Eubacteriales sp. (HG3A.0311)

ODI -0.07 5.53E-05 0.001 3364 unclassified unclassified unclassified unclassified Eubacteriales Clostridia Firmicutes

| Eubacteriales sp. (HG3A.0331) | ODI | -0.07 | 5.40E-05 | 0.001 | 3364 unclassified | unclassified | unclassified | unclassified | Eubacteriales | Clostridia | Firmicutes |
| --- | --- | --- | --- | --- | --- | --- | --- | --- | --- | --- | --- |
| Eubacteriales sp.  (HG3A.0421) | ODI | -0.071 | 4.62E-05 | 0.001 | 3364 unclassified | unclassified | unclassified | unclassified | Eubacteriales | Clostridia | Firmicutes |
| Mediterraneibacter glycyrrhizinilyticus (HG3A.0314) | ODI | 0.072 | 3.51E-05 | 0.001 | 3364 unclassified | Mediterraneibacter glycyrrhizinilyticus | Mediterraneibact er | Lachnospiraceae | Eubacteriales | Clostridia | Firmicutes |
| Oscillospiraceae sp.  (HG3A.0437) | ODI | -0.071 | 4.77E-05 | 0.001 | 3364 unclassified | unclassified | unclassified | Oscillospiraceae | Eubacteriales | Clostridia | Firmicutes |
| Roseburia inulinivorans (HG3A.0036) | ODI | 0.072 | 3.05E-05 | 0.001 | 3364 unclassified | Roseburia inulinivorans | Roseburia | Lachnospiraceae | Eubacteriales | Clostridia | Firmicutes |
| Clostridia sp.  (HG3A.0470) | T90 | -0.072 | 3.78E-05 | 0.002 | 3364 unclassified | unclassified | unclassified | unclassified | unclassified | Clostridia | Firmicutes |
| Clostridia sp. (HG3A.0470) | ODI | -0.067 | 1.15E-04 | 0.002 | 3364 unclassified | unclassified | unclassified | unclassified | unclassified | Clostridia | Firmicutes |
| Clostridia sp.  (HG3A.0515) | T90 | -0.072 | 3.44E-05 | 0.002 | 3364 unclassified | unclassified | unclassified | unclassified | unclassified | Clostridia | Firmicutes |
| Clostridiaceae sp. (HG3A.0431) | T90 | 0.073 | 2.88E-05 | 0.002 | 3364 unclassified | unclassified | unclassified | Clostridiaceae | Eubacteriales | Clostridia | Firmicutes |
| Eubacteriales sp.  (HG3A.0100) | T90 | -0.074 | 1.98E-05 | 0.002 | 3364 unclassified | unclassified | unclassified | unclassified | Eubacteriales | Clostridia | Firmicutes |
| Eubacteriales sp. (HG3A.0149) | T90 | -0.072 | 3.49E-05 | 0.002 | 3364 unclassified | unclassified | unclassified | unclassified | Eubacteriales | Clostridia | Firmicutes |
| Eubacteriales sp.  (HG3A.0250) | ODI | -0.068 | 8.26E-05 | 0.002 | 3364 unclassified | unclassified | unclassified | unclassified | Eubacteriales | Clostridia | Firmicutes |
| Eubacteriales sp. (HG3A.0309) | ODI | -0.067 | 1.13E-04 | 0.002 | 3364 unclassified | unclassified | unclassified | unclassified | Eubacteriales | Clostridia | Firmicutes |
| Eubacteriales sp.  (HG3A.0321) | ODI | -0.068 | 1.01E-04 | 0.002 | 3364 unclassified | unclassified | unclassified | unclassified | Eubacteriales | Clostridia | Firmicutes |
| Eubacteriales sp. (HG3A.0703) | T90 | -0.075 | 1.65E-05 | 0.002 | 3364 unclassified | unclassified | unclassified | unclassified | Eubacteriales | Clostridia | Firmicutes |
| Firmicutes sp.  (HG3A.0341) | ODI | -0.067 | 1.14E-04 | 0.002 | 3364 unclassified | unclassified | unclassified | unclassified | unclassified | unclassified | Firmicutes |
| Flavonifractor plautii (HG3A.0079) | T90 | 0.074 | 2.13E-05 | 0.002 | 3364 unclassified | Flavonifractor plautii | Flavonifractor | Oscillospiraceae | Eubacteriales | Clostridia | Firmicutes |

Flavonifractor plautii (HG3A.0079)

ODI 0.068 1.00E-04 0.002 3364 unclassified Flavonifractor plautii Flavonifractor Oscillospiraceae Eubacteriales Clostridia Firmicutes

Fusicatenibacter saccharivorans

(HG3A.0004)

ODI

0.068 9.66E-05

0.002 3364 unclassified

Fusicatenibacter

saccharivorans

Fusicatenibacter Lachnospiraceae

Eubacteriales

Clostridia

Firmicutes

| Intestinibacillus sp. Marseille-P4005 (HG3A.0168) | T90 | 0.072 | 3.05E-05 | 0.002 | 3364 unclassified | Intestinibacillus sp. Marseille-P4005 | Intestinibacillus | Eubacteriaceae | Eubacteriales | Clostridia | Firmicutes |
| --- | --- | --- | --- | --- | --- | --- | --- | --- | --- | --- | --- |
| Lachnospiraceae sp. (HG3A.0018) | ODI | 0.066 | 1.34E-04 | 0.002 | 3364 unclassified | unclassified | unclassified | Lachnospiraceae | Eubacteriales | Clostridia | Firmicutes |
| [Ruminococcus] gnavus (HG3A.0239) | T90 | 0.073 | 2.90E-05 | 0.002 | 3364 unclassified | [Ruminococcus] gnavus | Mediterraneibact er | Lachnospiraceae | Eubacteriales | Clostridia | Firmicutes |
| Victivallis vadensis (HG3A.0689) | ODI | -0.069 | 7.94E-05 | 0.002 | 3364 unclassified | Victivallis vadensis | Victivallis | Victivallaceae | Victivallales | Lentisphaeria | Lentisphaerae |
| Alistipes shahii  (HG3A.0054) | ODI | -0.066 | 1.51E-04 | 0.003 | 3364 unclassified | Alistipes shahii | Alistipes | Rikenellaceae | Bacteroidales | Bacteroidia | Bacteroidetes |
| Clostridia sp. (HG3A.0140) | T90 | -0.069 | 6.61E-05 | 0.003 | 3364 unclassified | unclassified | unclassified | unclassified | unclassified | Clostridia | Firmicutes |
| Clostridia sp.  (HG3A.0272) | ODI | -0.065 | 1.68E-04 | 0.003 | 3364 unclassified | unclassified | unclassified | unclassified | unclassified | Clostridia | Firmicutes |
| Clostridia sp. (HG3A.0508) | T90 | -0.068 | 9.19E-05 | 0.003 | 3364 unclassified | unclassified | unclassified | unclassified | unclassified | Clostridia | Firmicutes |
| Clostridia sp.  (HG3A.0508) | ODI | -0.065 | 1.81E-04 | 0.003 | 3364 unclassified | unclassified | unclassified | unclassified | unclassified | Clostridia | Firmicutes |
| Collinsella aerofaciens (HG3A.0019) | ODI | 0.065 | 1.74E-04 | 0.003 | 3364 unclassified | Collinsella aerofaciens | Collinsella | Coriobacteriaceae | Coriobacteriales | Coriobacteriia | Actinobacteria |
| Dorea formicigenerans (HG3A.0006) | ODI | 0.064 | 2.21E-04 | 0.003 | 3364 unclassified | Dorea formicigenerans | Dorea | Lachnospiraceae | Eubacteriales | Clostridia | Firmicutes |
| Dorea sp. AF36-15AT (HG3A.0052) | T90 | 0.069 | 7.47E-05 | 0.003 | 3364 unclassified | Dorea sp. AF36-15AT | Dorea | Lachnospiraceae | Eubacteriales | Clostridia | Firmicutes |
| Eggerthellales sp.  (HG3A.0177) | ODI | -0.065 | 1.99E-04 | 0.003 | 3364 unclassified | unclassified | unclassified | unclassified | Eggerthellales | Coriobacteriia | Actinobacteria |
| Eubacteriales sp. (HG3A.0215) | ODI | -0.065 | 1.85E-04 | 0.003 | 3364 unclassified | unclassified | unclassified | unclassified | Eubacteriales | Clostridia | Firmicutes |
| Eubacteriales sp.  (HG3A.0468) | T90 | -0.069 | 8.02E-05 | 0.003 | 3364 unclassified | unclassified | unclassified | unclassified | Eubacteriales | Clostridia | Firmicutes |
| Eubacteriales sp. (HG3A.0572) | ODI | -0.066 | 1.44E-04 | 0.003 | 3364 unclassified | unclassified | unclassified | unclassified | Eubacteriales | Clostridia | Firmicutes |
| Eubacteriales sp.  (HG3A.1379) | T90 | -0.07 | 5.65E-05 | 0.003 | 3364 unclassified | unclassified | unclassified | unclassified | Eubacteriales | Clostridia | Firmicutes |
| Oscillospiraceae sp. (HG3A.0445) | ODI | -0.065 | 2.02E-04 | 0.003 | 3364 unclassified | unclassified | unclassified | Oscillospiraceae | Eubacteriales | Clostridia | Firmicutes |

[Ruminococcus] torques

(HG3A.0034) ODI 0.066 1.53E-04 0.003 3364 unclassified [Ruminococcus] torques

Mediterraneibact

er Lachnospiraceae Eubacteriales Clostridia Firmicutes

| Staphylococcus aureus (HG3A.1538) | T90 | 0.068 | 8.77E-05 | 0.003 | 3364 unclassified | Staphylococcus aureus | Staphylococcus | Staphylococcaceae | Bacillales | Bacilli | Firmicutes |
| --- | --- | --- | --- | --- | --- | --- | --- | --- | --- | --- | --- |
| Blautia obeum  (HG3A.0009) | T90 | 0.067 | 1.03E-04 | 0.004 | 3364 unclassified | Blautia obeum | Blautia | Lachnospiraceae | Eubacteriales | Clostridia | Firmicutes |
| Clostridia sp. (HG3A.0515) | AHI | -0.077 | 3.22E-05 | 0.004 | 3004 unclassified | unclassified | unclassified | unclassified | unclassified | Clostridia | Firmicutes |
| Clostridia sp.  (HG3A.0682) | ODI | -0.063 | 2.78E-04 | 0.004 | 3364 unclassified | unclassified | unclassified | unclassified | unclassified | Clostridia | Firmicutes |
| Coprococcus eutactus (HG3A.0155) | ODI | -0.063 | 2.87E-04 | 0.004 | 3364 unclassified | Coprococcus eutactus | Coprococcus | Lachnospiraceae | Eubacteriales | Clostridia | Firmicutes |
| Eubacteriales sp.  (HG3A.0069) | AHI | -0.076 | 3.42E-05 | 0.004 | 3004 unclassified | unclassified | unclassified | unclassified | Eubacteriales | Clostridia | Firmicutes |
| Eubacteriales sp. (HG3A.0083) | ODI | -0.063 | 2.99E-04 | 0.004 | 3364 unclassified | unclassified | unclassified | unclassified | Eubacteriales | Clostridia | Firmicutes |
| Eubacteriales sp.  (HG3A.0084) | AHI | -0.08 | 1.31E-05 | 0.004 | 3004 unclassified | unclassified | unclassified | unclassified | Eubacteriales | Clostridia | Firmicutes |
| Eubacteriales sp. (HG3A.0085) | AHI | -0.079 | 1.83E-05 | 0.004 | 3004 unclassified | unclassified | unclassified | unclassified | Eubacteriales | Clostridia | Firmicutes |
| Eubacteriales sp.  (HG3A.0120) | ODI | -0.063 | 3.16E-04 | 0.004 | 3364 unclassified | unclassified | unclassified | unclassified | Eubacteriales | Clostridia | Firmicutes |
| Eubacteriales sp. (HG3A.0179) | ODI | -0.064 | 2.54E-04 | 0.004 | 3364 unclassified | unclassified | unclassified | unclassified | Eubacteriales | Clostridia | Firmicutes |
| Eubacteriales sp.  (HG3A.0718) | ODI | -0.062 | 3.43E-04 | 0.004 | 3364 unclassified | unclassified | unclassified | unclassified | Eubacteriales | Clostridia | Firmicutes |
| Eubacteriales sp. (HG3A.0829) | ODI | -0.063 | 2.67E-04 | 0.004 | 3364 unclassified | unclassified | unclassified | unclassified | Eubacteriales | Clostridia | Firmicutes |
| Firmicutes sp.  (HG3A.0398) | AHI | -0.079 | 2.01E-05 | 0.004 | 3004 unclassified | unclassified | unclassified | unclassified | unclassified | unclassified | Firmicutes |
| Oscillospiraceae sp. (HG3A.0207) | AHI | -0.081 | 1.15E-05 | 0.004 | 3004 unclassified | unclassified | unclassified | Oscillospiraceae | Eubacteriales | Clostridia | Firmicutes |
| Oscillospiraceae sp.  (HG3A.0445) | T90 | -0.067 | 1.22E-04 | 0.004 | 3364 unclassified | unclassified | unclassified | Oscillospiraceae | Eubacteriales | Clostridia | Firmicutes |
| Alistipes communis (HG3A.0064) | ODI | -0.061 | 4.66E-04 | 0.005 | 3364 unclassified | Alistipes communis | Alistipes | Rikenellaceae | Bacteroidales | Bacteroidia | Bacteroidetes |
| Clostridia sp.  (HG3A.0435) | T90 | -0.066 | 1.58E-04 | 0.005 | 3364 unclassified | unclassified | unclassified | unclassified | unclassified | Clostridia | Firmicutes |
| Clostridia sp. (HG3A.0435) | ODI | -0.062 | 4.05E-04 | 0.005 | 3364 unclassified | unclassified | unclassified | unclassified | unclassified | Clostridia | Firmicutes |

Clostridia sp. (HG3A.0931)

T90 -0.065 1.68E-04 0.005 3364 unclassified unclassified unclassified unclassified unclassified Clostridia Firmicutes

| Clostridium sp. TF06- 15AC (HG3A.0032) | ODI | 0.062 | 3.97E-04 | 0.005 | 3364 unclassified | Clostridium sp. TF06- 15AC | Clostridium | Clostridiaceae | Eubacteriales | Clostridia | Firmicutes |
| --- | --- | --- | --- | --- | --- | --- | --- | --- | --- | --- | --- |
| Eubacteriales sp.  (HG3A.0125) | ODI | -0.061 | 4.43E-04 | 0.005 | 3364 unclassified | unclassified | unclassified | unclassified | Eubacteriales | Clostridia | Firmicutes |
| Eubacteriales sp. (HG3A.0234) | T90 | -0.066 | 1.63E-04 | 0.005 | 3364 unclassified | unclassified | unclassified | unclassified | Eubacteriales | Clostridia | Firmicutes |
| Eubacteriales sp.  (HG3A.0342) | ODI | -0.062 | 3.96E-04 | 0.005 | 3364 unclassified | unclassified | unclassified | unclassified | Eubacteriales | Clostridia | Firmicutes |
| Eubacteriales sp. (HG3A.0506) | T90 | -0.065 | 1.86E-04 | 0.005 | 3364 unclassified | unclassified | unclassified | unclassified | Eubacteriales | Clostridia | Firmicutes |
| Lachnospiraceae sp.  (HG3A.0180) | ODI | -0.061 | 4.47E-04 | 0.005 | 3364 unclassified | unclassified | unclassified | Lachnospiraceae | Eubacteriales | Clostridia | Firmicutes |
| Oscillibacter sp. (HG3A.0734) | T90 | -0.066 | 1.45E-04 | 0.005 | 3364 unclassified | unclassified | Oscillibacter | Oscillospiraceae | Eubacteriales | Clostridia | Firmicutes |
| Oscillospiraceae sp.  (HG3A.0223) | ODI | -0.061 | 4.54E-04 | 0.005 | 3364 unclassified | unclassified | unclassified | Oscillospiraceae | Eubacteriales | Clostridia | Firmicutes |
| Clostridia sp. (HG3A.0599) | ODI | -0.06 | 5.44E-04 | 0.006 | 3364 unclassified | unclassified | unclassified | unclassified | unclassified | Clostridia | Firmicutes |
| Clostridium sp.  (HG3A.0050) | ODI | 0.06 | 5.65E-04 | 0.006 | 3364 unclassified | unclassified | Clostridium | Clostridiaceae | Eubacteriales | Clostridia | Firmicutes |
| Collinsella aerofaciens (HG3A.0019) | T90 | 0.064 | 2.55E-04 | 0.006 | 3364 unclassified | Collinsella aerofaciens | Collinsella | Coriobacteriaceae | Coriobacteriales | Coriobacteriia | Actinobacteria |
| Eubacteriales sp.  (HG3A.0118) | T90 | -0.063 | 2.82E-04 | 0.006 | 3364 unclassified | unclassified | unclassified | unclassified | Eubacteriales | Clostridia | Firmicutes |
| Eubacteriales sp. (HG3A.0196) | T90 | -0.064 | 2.50E-04 | 0.006 | 3364 unclassified | unclassified | unclassified | unclassified | Eubacteriales | Clostridia | Firmicutes |
| Eubacteriales sp.  (HG3A.0284) | ODI | -0.06 | 5.20E-04 | 0.006 | 3364 unclassified | unclassified | unclassified | unclassified | Eubacteriales | Clostridia | Firmicutes |
| Eubacteriales sp. (HG3A.0291) | ODI | -0.06 | 5.90E-04 | 0.006 | 3364 unclassified | unclassified | unclassified | unclassified | Eubacteriales | Clostridia | Firmicutes |
| Eubacteriales sp.  (HG3A.0419) | T90 | -0.064 | 2.48E-04 | 0.006 | 3364 unclassified | unclassified | unclassified | unclassified | Eubacteriales | Clostridia | Firmicutes |
| Eubacteriales sp. (HG3A.0609) | ODI | -0.06 | 5.66E-04 | 0.006 | 3364 unclassified | unclassified | unclassified | unclassified | Eubacteriales | Clostridia | Firmicutes |
| Oscillospiraceae sp.  (HG3A.0256) | T90 | 0.064 | 2.15E-04 | 0.006 | 3364 unclassified | unclassified | unclassified | Oscillospiraceae | Eubacteriales | Clostridia | Firmicutes |
| Oscillospiraceae sp. (HG3A.1270) | ODI | -0.06 | 5.56E-04 | 0.006 | 3364 unclassified | unclassified | unclassified | Oscillospiraceae | Eubacteriales | Clostridia | Firmicutes |

Ruminococcus sp.

AM42-11 (HG3A.0002) T90 0.063 2.84E-04 0.006 3364 unclassified

Ruminococcus sp. AM42-

11 Ruminococcus Oscillospiraceae Eubacteriales Clostridia Firmicutes

| Sellimonas intestinalis (HG3A.0417) | T90 | 0.063 | 2.73E-04 | 0.006 | 3364 unclassified | Sellimonas intestinalis | Sellimonas | Lachnospiraceae | Eubacteriales Clostridia | Firmicutes |
| --- | --- | --- | --- | --- | --- | --- | --- | --- | --- | --- |
| Blautia hydrogenotrophica  (HG3A.0430) | T90 | 0.062 | 3.43E-04 | 0.007 | 3364 unclassified | Blautia hydrogenotrophica | Blautia | Lachnospiraceae | Eubacteriales Clostridia | Firmicutes |
| Clostridia sp. (HG3A.1020) | ODI | -0.059 | 6.51E-04 | 0.007 | 3364 unclassified | unclassified | unclassified | unclassified | unclassified Clostridia | Firmicutes |
| Clostridium sp. AT4  (HG3A.0347) | T90 | 0.062 | 3.52E-04 | 0.007 | 3364 unclassified | Clostridium sp. AT4 | Clostridium | Clostridiaceae | Eubacteriales Clostridia | Firmicutes |
| Eubacteriales sp. (HG3A.0189) | ODI | -0.059 | 6.32E-04 | 0.007 | 3364 unclassified | unclassified | unclassified | unclassified | Eubacteriales Clostridia | Firmicutes |
| Eubacteriales sp.  (HG3A.0211) | ODI | -0.059 | 6.55E-04 | 0.007 | 3364 unclassified | unclassified | unclassified | unclassified | Eubacteriales Clostridia | Firmicutes |
| Eubacteriales sp. (HG3A.0264) | ODI | -0.059 | 7.31E-04 | 0.007 | 3364 unclassified | unclassified | unclassified | unclassified | Eubacteriales Clostridia | Firmicutes |
| Eubacteriales sp.  (HG3A.0291) | T90 | -0.062 | 3.71E-04 | 0.007 | 3364 unclassified | unclassified | unclassified | unclassified | Eubacteriales Clostridia | Firmicutes |
| Eubacteriales sp. (HG3A.0321) | T90 | -0.062 | 3.39E-04 | 0.007 | 3364 unclassified | unclassified | unclassified | unclassified | Eubacteriales Clostridia | Firmicutes |
| Eubacteriales sp.  (HG3A.0383) | ODI | -0.059 | 6.43E-04 | 0.007 | 3364 unclassified | unclassified | unclassified | unclassified | Eubacteriales Clostridia | Firmicutes |
| Eubacteriales sp. (HG3A.0531) | T90 | -0.062 | 3.49E-04 | 0.007 | 3364 unclassified | unclassified | unclassified | unclassified | Eubacteriales Clostridia | Firmicutes |
| Eubacteriales sp.  (HG3A.0630) | T90 | -0.062 | 3.29E-04 | 0.007 | 3364 unclassified | unclassified | unclassified | unclassified | Eubacteriales Clostridia | Firmicutes |
| Oscillospiraceae sp. (HG3A.0072) | T90 | -0.062 | 3.38E-04 | 0.007 | 3364 unclassified | unclassified | unclassified | Oscillospiraceae | Eubacteriales Clostridia | Firmicutes |
| Akkermansia muciniphila  (HG3A.0110) | ODI | -0.058 | 8.40E-04 | 0.008 | 3364 unclassified | Akkermansia muciniphila | Akkermansia | Akkermansiaceae | Verrucomicrobia Verrucomicrobiales e | Verrucomicrob ia |
| Clostridia sp. (HG3A.0272) | T90 | -0.061 | 4.68E-04 | 0.008 | 3364 unclassified | unclassified | unclassified | unclassified | unclassified Clostridia | Firmicutes |
| Clostridia sp.  (HG3A.0682) | AHI | -0.071 | 1.24E-04 | 0.008 | 3004 unclassified | unclassified | unclassified | unclassified | unclassified Clostridia | Firmicutes |
| Clostridia sp. (HG3A.0756) | ODI | -0.058 | 7.89E-04 | 0.008 | 3364 unclassified | unclassified | unclassified | unclassified | unclassified Clostridia | Firmicutes |
| Eubacteriales sp.  (HG3A.0100) | AHI | -0.071 | 1.04E-04 | 0.008 | 3004 unclassified | unclassified | unclassified | unclassified | Eubacteriales Clostridia | Firmicutes |
| Eubacteriales sp. (HG3A.0102) | ODI | -0.058 | 8.05E-04 | 0.008 | 3364 unclassified | unclassified | unclassified | unclassified | Eubacteriales Clostridia | Firmicutes |

Eubacteriales sp. (HG3A.0156)

AHI -0.071 1.07E-04 0.008 3004 unclassified unclassified unclassified unclassified Eubacteriales Clostridia Firmicutes

| Eubacteriales sp.  (HG3A.0193) | ODI | -0.058 | 7.96E-04 | 0.008 | 3364 unclassified | unclassified | unclassified | unclassified | Eubacteriales | Clostridia | Firmicutes |
| --- | --- | --- | --- | --- | --- | --- | --- | --- | --- | --- | --- |
| Eubacteriales sp.  (HG3A.0229) | ODI | -0.058 | 8.16E-04 | 0.008 | 3364 unclassified | unclassified | unclassified | unclassified | Eubacteriales | Clostridia | Firmicutes |
| Eubacteriales sp. (HG3A.0235) | ODI | -0.058 | 9.29E-04 | 0.008 | 3364 unclassified | unclassified | unclassified | unclassified | Eubacteriales | Clostridia | Firmicutes |
| Eubacteriales sp.  (HG3A.0373) | T90 | -0.061 | 4.64E-04 | 0.008 | 3364 unclassified | unclassified | unclassified | unclassified | Eubacteriales | Clostridia | Firmicutes |
| Eubacteriales sp. (HG3A.0506) | AHI | -0.073 | 7.86E-05 | 0.008 | 3004 unclassified | unclassified | unclassified | unclassified | Eubacteriales | Clostridia | Firmicutes |
| Eubacteriales sp.  (HG3A.0698) | T90 | 0.061 | 4.51E-04 | 0.008 | 3364 unclassified | unclassified | unclassified | unclassified | Eubacteriales | Clostridia | Firmicutes |
| Eubacteriales sp. (HG3A.0829) | AHI | -0.071 | 1.20E-04 | 0.008 | 3004 unclassified | unclassified | unclassified | unclassified | Eubacteriales | Clostridia | Firmicutes |
| Firmicutes sp.  (HG3A.0397) | AHI | -0.072 | 1.02E-04 | 0.008 | 3004 unclassified | unclassified | unclassified | unclassified | unclassified | unclassified | Firmicutes |
| Oscillibacter sp. (HG3A.0245) | ODI | -0.058 | 8.50E-04 | 0.008 | 3364 unclassified | unclassified | Oscillibacter | Oscillospiraceae | Eubacteriales | Clostridia | Firmicutes |
| Oscillibacter sp.  (HG3A.0734) | ODI | -0.058 | 8.98E-04 | 0.008 | 3364 unclassified | unclassified | Oscillibacter | Oscillospiraceae | Eubacteriales | Clostridia | Firmicutes |
| Roseburia sp. AM59- 24XD (HG3A.0391) | ODI | -0.058 | 8.49E-04 | 0.008 | 3364 unclassified | Roseburia sp. AM59- 24XD | Roseburia | Lachnospiraceae | Eubacteriales | Clostridia | Firmicutes |
| [Ruminococcus] torques (HG3A.0034) | T90 | 0.061 | 4.24E-04 | 0.008 | 3364 unclassified | [Ruminococcus] torques | Mediterraneibact er | Lachnospiraceae | Eubacteriales | Clostridia | Firmicutes |
| Clostridia sp. (HG3A.0094) | ODI | -0.057 | 0.001 | 0.009 | 3364 unclassified | unclassified | unclassified | unclassified | unclassified | Clostridia | Firmicutes |
| Clostridia sp.  (HG3A.0861) | T90 | -0.06 | 5.57E-04 | 0.009 | 3364 unclassified | unclassified | unclassified | unclassified | unclassified | Clostridia | Firmicutes |
| Eubacteriales sp. (HG3A.0062) | ODI | -0.057 | 0.001 | 0.009 | 3364 unclassified | unclassified | unclassified | unclassified | Eubacteriales | Clostridia | Firmicutes |
| Eubacteriales sp.  (HG3A.0154) | ODI | -0.057 | 0.001 | 0.009 | 3364 unclassified | unclassified | unclassified | unclassified | Eubacteriales | Clostridia | Firmicutes |
| Eubacteriales sp. (HG3A.0162) | AHI | -0.069 | 1.69E-04 | 0.009 | 3004 unclassified | unclassified | unclassified | unclassified | Eubacteriales | Clostridia | Firmicutes |
| Eubacteriales sp.  (HG3A.0235) | AHI | -0.069 | 1.64E-04 | 0.009 | 3004 unclassified | unclassified | unclassified | unclassified | Eubacteriales | Clostridia | Firmicutes |
| Eubacteriales sp. (HG3A.0731) | ODI | -0.057 | 0.001 | 0.009 | 3364 unclassified | unclassified | unclassified | unclassified | Eubacteriales | Clostridia | Firmicutes |
| Eubacteriales sp.  (HG3A.0864) | ODI | -0.057 | 0.001 | 0.009 | 3364 unclassified | unclassified | unclassified | unclassified | Eubacteriales | Clostridia | Firmicutes |
| Eubacteriales sp. (HG3A.1026) | ODI | -0.057 | 9.66E-04 | 0.009 | 3364 unclassified | unclassified | unclassified | unclassified | Eubacteriales | Clostridia | Firmicutes |

Eubacterium sp. (HG3A.0214)

| Firmicutes sp. (HG3A.0341) | AHI | -0.07 | 1.58E-04 | 0.009 | 3004 unclassified | unclassified | unclassified | unclassified | unclassified | unclassified | Firmicutes |
| --- | --- | --- | --- | --- | --- | --- | --- | --- | --- | --- | --- |
| Oscillospiraceae sp.  (HG3A.0693) | ODI | -0.057 | 0.001 | 0.009 | 3364 unclassified | unclassified | unclassified | Oscillospiraceae | Eubacteriales | Clostridia | Firmicutes |
| Anaerostipes caccae (HG3A.0747) | ODI | 0.056 | 0.001 | 0.01 | 3364 unclassified | Anaerostipes caccae | Anaerostipes | Lachnospiraceae | Eubacteriales | Clostridia | Firmicutes |
| Clostridia sp.  (HG3A.0728) | AHI | -0.068 | 2.35E-04 | 0.01 | 3004 unclassified | unclassified | unclassified | unclassified | unclassified | Clostridia | Firmicutes |
| Clostridia sp. (HG3A.1053) | ODI | -0.056 | 0.001 | 0.01 | 3364 unclassified | unclassified | unclassified | unclassified | unclassified | Clostridia | Firmicutes |
| Clostridiaceae sp.  (HG3A.0431) | ODI | 0.056 | 0.001 | 0.01 | 3364 unclassified | unclassified | unclassified | Clostridiaceae | Eubacteriales | Clostridia | Firmicutes |
| Dorea sp. AF36-15AT (HG3A.0052) | ODI | 0.056 | 0.001 | 0.01 | 3364 unclassified | Dorea sp. AF36-15AT | Dorea | Lachnospiraceae | Eubacteriales | Clostridia | Firmicutes |
| Eubacteriales sp.  (HG3A.0153) | ODI | -0.056 | 0.001 | 0.01 | 3364 unclassified | unclassified | unclassified | unclassified | Eubacteriales | Clostridia | Firmicutes |
| Eubacteriales sp. (HG3A.0197) | AHI | -0.068 | 2.15E-04 | 0.01 | 3004 unclassified | unclassified | unclassified | unclassified | Eubacteriales | Clostridia | Firmicutes |
| Eubacteriales sp.  (HG3A.0249) | ODI | -0.056 | 0.001 | 0.01 | 3364 unclassified | unclassified | unclassified | unclassified | Eubacteriales | Clostridia | Firmicutes |
| Eubacteriales sp. (HG3A.0381) | ODI | -0.056 | 0.001 | 0.01 | 3364 unclassified | unclassified | unclassified | unclassified | Eubacteriales | Clostridia | Firmicutes |
| Eubacteriales sp.  (HG3A.0383) | T90 | -0.059 | 6.23E-04 | 0.01 | 3364 unclassified | unclassified | unclassified | unclassified | Eubacteriales | Clostridia | Firmicutes |
| Eubacteriales sp. (HG3A.0409) | ODI | -0.056 | 0.001 | 0.01 | 3364 unclassified | unclassified | unclassified | unclassified | Eubacteriales | Clostridia | Firmicutes |
| Lachnospiraceae sp.  (HG3A.0236) | ODI | -0.056 | 0.001 | 0.01 | 3364 unclassified | unclassified | unclassified | Lachnospiraceae | Eubacteriales | Clostridia | Firmicutes |
| Oscillospiraceae sp. (HG3A.0210) | ODI | -0.056 | 0.001 | 0.01 | 3364 unclassified | unclassified | unclassified | Oscillospiraceae | Eubacteriales | Clostridia | Firmicutes |
| [Ruminococcus] gnavus  (HG3A.0239) | AHI | 0.068 | 2.00E-04 | 0.01 | 3004 unclassified | [Ruminococcus] gnavus | Mediterraneibact  er | Lachnospiraceae | Eubacteriales | Clostridia | Firmicutes |
| Bacteria sp. (HG3A.0634) | T90 | -0.059 | 7.17E-04 | 0.011 | 3364 unclassified | unclassified | unclassified | unclassified | unclassified | unclassified | unclassified |
| Clostridia sp.  (HG3A.0733) | ODI | -0.055 | 0.002 | 0.011 | 3364 unclassified | unclassified | unclassified | unclassified | unclassified | Clostridia | Firmicutes |
| Coprococcus comes (HG3A.0016) | T90 | 0.058 | 7.79E-04 | 0.011 | 3364 unclassified | Coprococcus comes | Coprococcus | Lachnospiraceae | Eubacteriales | Clostridia | Firmicutes |
| Eubacteriales sp.  (HG3A.0123) | ODI | 0.055 | 0.002 | 0.011 | 3364 unclassified | unclassified | unclassified | unclassified | Eubacteriales | Clostridia | Firmicutes |
| Eubacteriales sp. (HG3A.0125) | T90 | -0.058 | 7.70E-04 | 0.011 | 3364 unclassified | unclassified | unclassified | unclassified | Eubacteriales | Clostridia | Firmicutes |

ODI 0.057 0.001 0.009 3364 unclassified unclassified Eubacterium Eubacteriaceae Eubacteriales Clostridia Firmicutes

Eubacteriales sp. (HG3A.0269)

T90 -0.059 7.15E-04 0.011 3364 unclassified unclassified unclassified unclassified Eubacteriales Clostridia Firmicutes

| Eubacteriales sp. (HG3A.0284) | AHI | -0.067 | 2.60E-04 | 0.011 | 3004 unclassified | unclassified | unclassified | unclassified | Eubacteriales | Clostridia | Firmicutes |
| --- | --- | --- | --- | --- | --- | --- | --- | --- | --- | --- | --- |
| Eubacteriales sp.  (HG3A.0288) | ODI | -0.055 | 0.001 | 0.011 | 3364 unclassified | unclassified | unclassified | unclassified | Eubacteriales | Clostridia | Firmicutes |
| Eubacteriales sp. (HG3A.0864) | T90 | -0.059 | 6.78E-04 | 0.011 | 3364 unclassified | unclassified | unclassified | unclassified | Eubacteriales | Clostridia | Firmicutes |
| Firmicutes sp.  (HG3A.1085) | ODI | -0.055 | 0.002 | 0.011 | 3364 unclassified | unclassified | unclassified | unclassified | unclassified | unclassified | Firmicutes |
| Lachnospiraceae sp. (HG3A.0233) | ODI | -0.055 | 0.001 | 0.011 | 3364 unclassified | unclassified | unclassified | Lachnospiraceae | Eubacteriales | Clostridia | Firmicutes |
| Pediococcus acidilactici (HG3A.1468) | T90 | 0.059 | 7.02E-04 | 0.011 | 3364 unclassified | Pediococcus acidilactici | Pediococcus | Lactobacillaceae | Lactobacillales | Bacilli | Firmicutes |
| Anaerostipes sp. BG01 (HG3A.1509) | ODI | 0.055 | 0.002 | 0.012 | 3364 unclassified | Anaerostipes sp. BG01 | Anaerostipes | Lachnospiraceae | Eubacteriales | Clostridia | Firmicutes |
| Blautia obeum  (HG3A.0009) | ODI | 0.054 | 0.002 | 0.012 | 3364 unclassified | Blautia obeum | Blautia | Lachnospiraceae | Eubacteriales | Clostridia | Firmicutes |
| Clostridia sp. (HG3A.0550) | T90 | -0.058 | 8.97E-04 | 0.012 | 3364 unclassified | unclassified | unclassified | unclassified | unclassified | Clostridia | Firmicutes |
| Clostridia sp.  (HG3A.0741) | ODI | -0.055 | 0.002 | 0.012 | 3364 unclassified | unclassified | unclassified | unclassified | unclassified | Clostridia | Firmicutes |
| Clostridia sp. (HG3A.0752) | ODI | -0.054 | 0.002 | 0.012 | 3364 unclassified | unclassified | unclassified | unclassified | unclassified | Clostridia | Firmicutes |
| Eubacteriales sp.  (HG3A.0489) | T90 | -0.058 | 8.55E-04 | 0.012 | 3364 unclassified | unclassified | unclassified | unclassified | Eubacteriales | Clostridia | Firmicutes |
| Eubacteriales sp. (HG3A.0617) | ODI | -0.054 | 0.002 | 0.012 | 3364 unclassified | unclassified | unclassified | unclassified | Eubacteriales | Clostridia | Firmicutes |
| Eubacteriales sp.  (HG3A.1086) | ODI | -0.054 | 0.002 | 0.012 | 3364 unclassified | unclassified | unclassified | unclassified | Eubacteriales | Clostridia | Firmicutes |
| Oscillospiraceae sp. (HG3A.1173) | ODI | -0.055 | 0.002 | 0.012 | 3364 unclassified | unclassified | unclassified | Oscillospiraceae | Eubacteriales | Clostridia | Firmicutes |
| [Ruminococcus] torques (HG3A.0088) | ODI | 0.054 | 0.002 | 0.012 | 3364 unclassified | [Ruminococcus] torques | Mediterraneibact er | Lachnospiraceae | Eubacteriales | Clostridia | Firmicutes |
| Eubacteriales sp. (HG3A.0329) | ODI | -0.054 | 0.002 | 0.013 | 3364 unclassified | unclassified | unclassified | unclassified | Eubacteriales | Clostridia | Firmicutes |
| Eubacteriales sp.  (HG3A.0442) | T90 | -0.057 | 9.84E-04 | 0.013 | 3364 unclassified | unclassified | unclassified | unclassified | Eubacteriales | Clostridia | Firmicutes |
| Eubacteriales sp. (HG3A.0956) | ODI | -0.053 | 0.002 | 0.013 | 3364 unclassified | unclassified | unclassified | unclassified | Eubacteriales | Clostridia | Firmicutes |

Firmicutes sp.

# (HG3A.0501)

ODI -0.054 0.002 0.013 3364 unclassified unclassified unclassified unclassified unclassified unclassified Firmicutes

| Intestinimonas Intestinimonas  massiliensis ODI -0.054 0.002 0.013 3364 unclassified massiliensis Intestinimonas unclassified Eubacteriales Clostridia Firmicutes  (HG3A.0198) | | | | | | | | | | | |
| --- | --- | --- | --- | --- | --- | --- | --- | --- | --- | --- | --- |
| Lachnospiraceae sp.  (HG3A.0855) | ODI | -0.054 | 0.002 | 0.013 | 3364 unclassified | unclassified | unclassified | Lachnospiraceae | Eubacteriales | Clostridia | Firmicutes |
| Blautia sp. (HG3A.0416) | ODI | 0.053 | 0.002 | 0.014 | 3364 unclassified | unclassified | Blautia | Lachnospiraceae | Eubacteriales | Clostridia | Firmicutes |
| Clostridia sp.  (HG3A.0385) | T90 | -0.057 | 0.001 | 0.014 | 3364 unclassified | unclassified | unclassified | unclassified | unclassified | Clostridia | Firmicutes |
| Eubacteriales sp. (HG3A.0311) | T90 | -0.057 | 0.001 | 0.014 | 3364 unclassified | unclassified | unclassified | unclassified | Eubacteriales | Clostridia | Firmicutes |
| Eubacteriales sp.  (HG3A.0443) | ODI | -0.053 | 0.002 | 0.014 | 3364 unclassified | unclassified | unclassified | unclassified | Eubacteriales | Clostridia | Firmicutes |
| Eubacteriales sp. (HG3A.0864) | AHI | -0.065 | 4.04E-04 | 0.014 | 3004 unclassified | unclassified | unclassified | unclassified | Eubacteriales | Clostridia | Firmicutes |
| Eubacteriales sp.  (HG3A.1019) | ODI | -0.053 | 0.002 | 0.014 | 3364 unclassified | unclassified | unclassified | unclassified | Eubacteriales | Clostridia | Firmicutes |
| Firmicutes sp. (HG3A.0436) | ODI | -0.053 | 0.002 | 0.014 | 3364 unclassified | unclassified | unclassified | unclassified | unclassified | unclassified | Firmicutes |
| Mediterraneibacter glycyrrhizinilyticus  (HG3A.0314) | AHI | 0.066 | 3.62E-04 | 0.014 | 3004 unclassified | Mediterraneibacter glycyrrhizinilyticus | Mediterraneibact er | Lachnospiraceae | Eubacteriales | Clostridia | Firmicutes |
| Oscillospiraceae sp. (HG3A.0072) | AHI | -0.065 | 3.84E-04 | 0.014 | 3004 unclassified | unclassified | unclassified | Oscillospiraceae | Eubacteriales | Clostridia | Firmicutes |
| Bacteria sp.  (HG3A.0634) | AHI | -0.065 | 4.49E-04 | 0.015 | 3004 unclassified | unclassified | unclassified | unclassified | unclassified | unclassified | unclassified |
| Blautia sp. (HG3A.0416) | T90 | 0.056 | 0.001 | 0.015 | 3364 unclassified | unclassified | Blautia | Lachnospiraceae | Eubacteriales | Clostridia | Firmicutes |
| Clostridia sp.  (HG3A.0879) | ODI | -0.053 | 0.002 | 0.015 | 3364 unclassified | unclassified | unclassified | unclassified | unclassified | Clostridia | Firmicutes |
| Collinsella aerofaciens (HG3A.0019) | AHI | 0.063 | 5.80E-04 | 0.015 | 3004 unclassified | Collinsella aerofaciens | Collinsella | Coriobacteriaceae | Coriobacteriales | Coriobacteriia | Actinobacteria |
| Eubacteriales sp.  (HG3A.0196) | AHI | -0.064 | 5.16E-04 | 0.015 | 3004 unclassified | unclassified | unclassified | unclassified | Eubacteriales | Clostridia | Firmicutes |
| Eubacteriales sp. (HG3A.0226) | AHI | -0.064 | 4.99E-04 | 0.015 | 3004 unclassified | unclassified | unclassified | unclassified | Eubacteriales | Clostridia | Firmicutes |
| Eubacteriales sp.  (HG3A.0421) | AHI | -0.064 | 4.66E-04 | 0.015 | 3004 unclassified | unclassified | unclassified | unclassified | Eubacteriales | Clostridia | Firmicutes |
| Eubacteriales sp. (HG3A.0442) | AHI | -0.063 | 5.67E-04 | 0.015 | 3004 unclassified | unclassified | unclassified | unclassified | Eubacteriales | Clostridia | Firmicutes |

Eubacteriales sp. (HG3A.0572)

AHI -0.064 5.50E-04 0.015 3004 unclassified unclassified unclassified unclassified Eubacteriales Clostridia Firmicutes

| Lachnospiraceae sp.  (HG3A.0180) | AHI | -0.064 | 5.38E-04 | 0.015 | 3004 unclassified | unclassified | unclassified | Lachnospiraceae | Eubacteriales | Clostridia | Firmicutes |
| --- | --- | --- | --- | --- | --- | --- | --- | --- | --- | --- | --- |
| Clostridia sp.  (HG3A.1053) | AHI | -0.063 | 6.86E-04 | 0.016 | 3004 unclassified | unclassified | unclassified | unclassified | unclassified | Clostridia | Firmicutes |
| Erysipelotrichales sp. (HG3A.1207) | T90 | 0.056 | 0.001 | 0.016 | 3364 unclassified | unclassified | unclassified | unclassified | Erysipelotrichales | Erysipelotrichia | Firmicutes |
| Eubacteriales sp.  (HG3A.0269) | AHI | -0.063 | 6.84E-04 | 0.016 | 3004 unclassified | unclassified | unclassified | unclassified | Eubacteriales | Clostridia | Firmicutes |
| Eubacteriales sp. (HG3A.0309) | AHI | -0.062 | 6.92E-04 | 0.016 | 3004 unclassified | unclassified | unclassified | unclassified | Eubacteriales | Clostridia | Firmicutes |
| Eubacteriales sp.  (HG3A.0419) | ODI | -0.052 | 0.003 | 0.016 | 3364 unclassified | unclassified | unclassified | unclassified | Eubacteriales | Clostridia | Firmicutes |
| Eubacteriales sp. (HG3A.0757) | ODI | -0.052 | 0.003 | 0.016 | 3364 unclassified | unclassified | unclassified | unclassified | Eubacteriales | Clostridia | Firmicutes |
| Firmicutes sp.  (HG3A.0398) | T90 | -0.056 | 0.001 | 0.016 | 3364 unclassified | unclassified | unclassified | unclassified | unclassified | unclassified | Firmicutes |
| Oscillospiraceae sp. (HG3A.1270) | AHI | -0.062 | 6.92E-04 | 0.016 | 3004 unclassified | unclassified | unclassified | Oscillospiraceae | Eubacteriales | Clostridia | Firmicutes |
| Clostridia sp.  (HG3A.0463) | ODI | -0.052 | 0.003 | 0.017 | 3364 unclassified | unclassified | unclassified | unclassified | unclassified | Clostridia | Firmicutes |
| Clostridia sp. (HG3A.0815) | ODI | -0.052 | 0.003 | 0.017 | 3364 unclassified | unclassified | unclassified | unclassified | unclassified | Clostridia | Firmicutes |
| Clostridia sp.  (HG3A.0931) | ODI | -0.052 | 0.003 | 0.017 | 3364 unclassified | unclassified | unclassified | unclassified | unclassified | Clostridia | Firmicutes |
| Clostridia sp. (HG3A.1010) | ODI | -0.052 | 0.003 | 0.017 | 3364 unclassified | unclassified | unclassified | unclassified | unclassified | Clostridia | Firmicutes |
| Eubacteriales sp.  (HG3A.0234) | ODI | -0.052 | 0.003 | 0.017 | 3364 unclassified | unclassified | unclassified | unclassified | Eubacteriales | Clostridia | Firmicutes |
| Eubacteriales sp. (HG3A.0316) | ODI | -0.052 | 0.003 | 0.017 | 3364 unclassified | unclassified | unclassified | unclassified | Eubacteriales | Clostridia | Firmicutes |
| Firmicutes sp.  (HG3A.0681) | ODI | -0.052 | 0.003 | 0.017 | 3364 unclassified | unclassified | unclassified | unclassified | unclassified | unclassified | Firmicutes |
| Oscillospiraceae sp. (HG3A.0437) | AHI | -0.062 | 7.70E-04 | 0.017 | 3004 unclassified | unclassified | unclassified | Oscillospiraceae | Eubacteriales | Clostridia | Firmicutes |
| Clostridium sp. AT4  (HG3A.0347) | ODI | 0.051 | 0.003 | 0.018 | 3364 unclassified | Clostridium sp. AT4 | Clostridium | Clostridiaceae | Eubacteriales | Clostridia | Firmicutes |
| [Clostridium] symbiosum (HG3A.0370) | ODI | 0.051 | 0.003 | 0.018 | 3364 unclassified | [Clostridium] symbiosum | Lachnoclostridiu m | Lachnospiraceae | Eubacteriales | Clostridia | Firmicutes |
| Eubacteriales sp.  (HG3A.0161) | ODI | -0.051 | 0.003 | 0.018 | 3364 unclassified | unclassified | unclassified | unclassified | Eubacteriales | Clostridia | Firmicutes |
| Eubacteriales sp. (HG3A.0610) | T90 | 0.055 | 0.001 | 0.018 | 3364 unclassified | unclassified | unclassified | unclassified | Eubacteriales | Clostridia | Firmicutes |

Lachnospiraceae sp. (HG3A.0257)

| Parvimonas micra (HG3A.1231) | ODI | 0.051 | 0.003 | 0.018 | 3364 unclassified | Parvimonas micra | Parvimonas | Peptoniphilaceae | Tissierellales | Tissierellia | Firmicutes |
| --- | --- | --- | --- | --- | --- | --- | --- | --- | --- | --- | --- |
| Alistipes provencensis (HG3A.0877) | ODI | -0.051 | 0.004 | 0.019 | 3364 unclassified | Alistipes provencensis | Alistipes | Rikenellaceae | Bacteroidales | Bacteroidia | Bacteroidetes |
| Eubacteriales sp. (HG3A.0486) | ODI | -0.051 | 0.003 | 0.019 | 3364 unclassified | unclassified | unclassified | unclassified | Eubacteriales | Clostridia | Firmicutes |
| Eubacteriales sp.  (HG3A.0578) | ODI | -0.051 | 0.003 | 0.019 | 3364 unclassified | unclassified | unclassified | unclassified | Eubacteriales | Clostridia | Firmicutes |
| Eubacteriales sp. (HG3A.0856) | ODI | -0.051 | 0.004 | 0.019 | 3364 unclassified | unclassified | unclassified | unclassified | Eubacteriales | Clostridia | Firmicutes |
| Eubacteriales sp.  (HG3A.1294) | ODI | -0.051 | 0.003 | 0.019 | 3364 unclassified | unclassified | unclassified | unclassified | Eubacteriales | Clostridia | Firmicutes |
| Anaerobutyricum hallii (HG3A.0012) | T90 | 0.054 | 0.002 | 0.02 | 3364 unclassified | Anaerobutyricum hallii | Anaerobutyricum | Lachnospiraceae | Eubacteriales | Clostridia | Firmicutes |
| Clostridia sp.  (HG3A.0728) | T90 | -0.054 | 0.002 | 0.02 | 3364 unclassified | unclassified | unclassified | unclassified | unclassified | Clostridia | Firmicutes |
| Clostridia sp. (HG3A.0750) | ODI | -0.05 | 0.004 | 0.02 | 3364 unclassified | unclassified | unclassified | unclassified | unclassified | Clostridia | Firmicutes |
| Clostridium sp.  (HG3A.0050) | T90 | 0.054 | 0.002 | 0.02 | 3364 unclassified | unclassified | Clostridium | Clostridiaceae | Eubacteriales | Clostridia | Firmicutes |
| Eubacteriales sp. (HG3A.0396) | T90 | -0.054 | 0.002 | 0.02 | 3364 unclassified | unclassified | unclassified | unclassified | Eubacteriales | Clostridia | Firmicutes |
| Eubacteriales sp.  (HG3A.0426) | T90 | -0.054 | 0.002 | 0.02 | 3364 unclassified | unclassified | unclassified | unclassified | Eubacteriales | Clostridia | Firmicutes |
| Eubacteriales sp. (HG3A.0978) | T90 | -0.055 | 0.002 | 0.02 | 3364 unclassified | unclassified | unclassified | unclassified | Eubacteriales | Clostridia | Firmicutes |
| Oscillospiraceae sp.  (HG3A.0616) | T90 | -0.054 | 0.002 | 0.02 | 3364 unclassified | unclassified | unclassified | Oscillospiraceae | Eubacteriales | Clostridia | Firmicutes |
| Alistipes shahii (HG3A.0054) | AHI | -0.061 | 9.59E-04 | 0.021 | 3004 unclassified | Alistipes shahii | Alistipes | Rikenellaceae | Bacteroidales | Bacteroidia | Bacteroidetes |
| Clostridia sp.  (HG3A.0929) | T90 | -0.054 | 0.002 | 0.021 | 3364 unclassified | unclassified | unclassified | unclassified | unclassified | Clostridia | Firmicutes |
| Enterocloster aldenensis (HG3A.0362) | T90 | 0.054 | 0.002 | 0.021 | 3364 unclassified | Enterocloster aldenensis | Enterocloster | Lachnospiraceae | Eubacteriales | Clostridia | Firmicutes |
| Eubacteriales sp.  (HG3A.0250) | AHI | -0.061 | 9.84E-04 | 0.021 | 3004 unclassified | unclassified | unclassified | unclassified | Eubacteriales | Clostridia | Firmicutes |
| Alistipes senegalensis (HG3A.0141) | ODI | -0.05 | 0.004 | 0.022 | 3364 unclassified | Alistipes senegalensis | Alistipes | Rikenellaceae | Bacteroidales | Bacteroidia | Bacteroidetes |

ODI -0.051 0.003 0.018 3364 unclassified unclassified unclassified Lachnospiraceae Eubacteriales Clostridia Firmicutes

Candidatus Borkfalkiales sp. (HG3A.1329)

| Clostridia sp. (HG3A.0385) | ODI | -0.05 | 0.004 | 0.022 | 3364 unclassified | unclassified | unclassified | unclassified | unclassified | Clostridia | Firmicutes |
| --- | --- | --- | --- | --- | --- | --- | --- | --- | --- | --- | --- |
| Clostridia sp.  (HG3A.0463) | T90 | -0.053 | 0.002 | 0.022 | 3364 unclassified | unclassified | unclassified | unclassified | unclassified | Clostridia | Firmicutes |
| Clostridia sp. (HG3A.0787) | ODI | -0.05 | 0.004 | 0.022 | 3364 unclassified | unclassified | unclassified | unclassified | unclassified | Clostridia | Firmicutes |
| Enterocloster citroniae (HG3A.0285) | T90 | 0.054 | 0.002 | 0.022 | 3364 unclassified | Enterocloster citroniae | Enterocloster | Lachnospiraceae | Eubacteriales | Clostridia | Firmicutes |
| Eubacteriales sp. (HG3A.0092) | ODI | -0.05 | 0.004 | 0.022 | 3364 unclassified | unclassified | unclassified | unclassified | Eubacteriales | Clostridia | Firmicutes |
| Eubacteriales sp.  (HG3A.0371) | ODI | -0.05 | 0.004 | 0.022 | 3364 unclassified | unclassified | unclassified | unclassified | Eubacteriales | Clostridia | Firmicutes |
| Firmicutes sp. (HG3A.0341) | T90 | -0.053 | 0.002 | 0.022 | 3364 unclassified | unclassified | unclassified | unclassified | unclassified | unclassified | Firmicutes |
| Firmicutes sp.  (HG3A.0454) | T90 | -0.053 | 0.002 | 0.022 | 3364 unclassified | unclassified | unclassified | unclassified | unclassified | unclassified | Firmicutes |
| Lachnospiraceae sp. (HG3A.0233) | T90 | -0.053 | 0.002 | 0.022 | 3364 unclassified | unclassified | unclassified | Lachnospiraceae | Eubacteriales | Clostridia | Firmicutes |
| Lachnospiraceae sp.  (HG3A.0399) | ODI | -0.05 | 0.004 | 0.022 | 3364 unclassified | unclassified | unclassified | Lachnospiraceae | Eubacteriales | Clostridia | Firmicutes |
| Anaerostipes caccae (HG3A.0747) | T90 | 0.053 | 0.002 | 0.023 | 3364 unclassified | Anaerostipes caccae | Anaerostipes | Lachnospiraceae | Eubacteriales | Clostridia | Firmicutes |
| Clostridia sp.  (HG3A.0094) | AHI | -0.059 | 0.001 | 0.023 | 3004 unclassified | unclassified | unclassified | unclassified | unclassified | Clostridia | Firmicutes |
| Eubacteriales sp. (HG3A.0193) | AHI | -0.059 | 0.001 | 0.023 | 3004 unclassified | unclassified | unclassified | unclassified | Eubacteriales | Clostridia | Firmicutes |
| Eubacteriales sp.  (HG3A.0249) | AHI | -0.06 | 0.001 | 0.023 | 3004 unclassified | unclassified | unclassified | unclassified | Eubacteriales | Clostridia | Firmicutes |
| Eubacteriales sp. (HG3A.0311) | AHI | -0.06 | 0.001 | 0.023 | 3004 unclassified | unclassified | unclassified | unclassified | Eubacteriales | Clostridia | Firmicutes |
| Eubacteriales sp.  (HG3A.0381) | AHI | -0.059 | 0.001 | 0.023 | 3004 unclassified | unclassified | unclassified | unclassified | Eubacteriales | Clostridia | Firmicutes |
| Eubacteriales sp. (HG3A.0409) | T90 | -0.053 | 0.002 | 0.023 | 3364 unclassified | unclassified | unclassified | unclassified | Eubacteriales | Clostridia | Firmicutes |
| Eubacteriales sp.  (HG3A.0509) | T90 | -0.053 | 0.002 | 0.023 | 3364 unclassified | unclassified | unclassified | unclassified | Eubacteriales | Clostridia | Firmicutes |
| Eubacteriales sp. (HG3A.0593) | T90 | -0.053 | 0.002 | 0.023 | 3364 unclassified | unclassified | unclassified | unclassified | Eubacteriales | Clostridia | Firmicutes |

T90 -0.054 0.002 0.022 3364 unclassified unclassified unclassified unclassified

Candidatus

Borkfalkiales Clostridia Firmicutes

Eubacteriales sp. (HG3A.0718)

| Eubacteriales sp. (HG3A.0977) | T90 | -0.053 | 0.002 | 0.023 | 3364 unclassified | unclassified | unclassified | unclassified | Eubacteriales | Clostridia | Firmicutes |
| --- | --- | --- | --- | --- | --- | --- | --- | --- | --- | --- | --- |
| Fusicatenibacter saccharivorans  (HG3A.0004) | AHI | 0.059 | 0.001 | 0.023 | 3004 unclassified | Fusicatenibacter saccharivorans | Fusicatenibacter | Lachnospiraceae | Eubacteriales | Clostridia | Firmicutes |
| Intestinibacillus sp. Marseille-P4005 (HG3A.0168) | AHI | 0.059 | 0.001 | 0.023 | 3004 unclassified | Intestinibacillus sp. Marseille-P4005 | Intestinibacillus | Eubacteriaceae | Eubacteriales | Clostridia | Firmicutes |
| Lachnoclostridium sp.  (HG3A.0655) | ODI | 0.049 | 0.004 | 0.023 | 3364 unclassified | unclassified | Lachnoclostridiu  m | Lachnospiraceae | Eubacteriales | Clostridia | Firmicutes |
| Lachnospiraceae sp. (HG3A.0236) | AHI | -0.06 | 0.001 | 0.023 | 3004 unclassified | unclassified | unclassified | Lachnospiraceae | Eubacteriales | Clostridia | Firmicutes |
| Anaerotruncus colihominis  (HG3A.0307) | ODI | 0.049 | 0.005 | 0.024 | 3364 unclassified | Anaerotruncus colihominis | Anaerotruncus | Oscillospiraceae | Eubacteriales | Clostridia | Firmicutes |
| Coprobacillus cateniformis (HG3A.0456) | T90 | 0.052 | 0.003 | 0.024 | 3364 unclassified | Coprobacillus cateniformis | Coprobacillus | Coprobacillaceae | Erysipelotrichales | Erysipelotrichia | Firmicutes |
| Coprobacillus sp.  (HG3A.0022) | ODI | 0.049 | 0.005 | 0.024 | 3364 unclassified | unclassified | Coprobacillus | Coprobacillaceae | Erysipelotrichales | Erysipelotrichia | Firmicutes |
| Eggerthellaceae sp. (HG3A.0171) | ODI | -0.049 | 0.005 | 0.024 | 3364 unclassified | unclassified | unclassified | Eggerthellaceae | Eggerthellales | Coriobacteriia | Actinobacteria |
| Eubacteriales sp.  (HG3A.0230) | AHI | -0.059 | 0.001 | 0.024 | 3004 unclassified | unclassified | unclassified | unclassified | Eubacteriales | Clostridia | Firmicutes |
| Eubacteriales sp. (HG3A.0668) | ODI | -0.049 | 0.005 | 0.024 | 3364 unclassified | unclassified | unclassified | unclassified | Eubacteriales | Clostridia | Firmicutes |
| Eubacteriales sp.  (HG3A.1191) | ODI | -0.049 | 0.005 | 0.024 | 3364 unclassified | unclassified | unclassified | unclassified | Eubacteriales | Clostridia | Firmicutes |
| Lachnospiraceae sp. (HG3A.0625) | T90 | 0.052 | 0.003 | 0.024 | 3364 unclassified | unclassified | unclassified | Lachnospiraceae | Eubacteriales | Clostridia | Firmicutes |
| Lactobacillus acidophilus  (HG3A.0672) | T90 | -0.052 | 0.003 | 0.024 | 3364 unclassified | Lactobacillus acidophilus | Lactobacillus | Lactobacillaceae | Lactobacillales | Bacilli | Firmicutes |
| Amedibacillus dolichus (HG3A.0798) | ODI | 0.049 | 0.005 | 0.025 | 3364 unclassified | Amedibacillus dolichus | Amedibacillus | Erysipelotrichaceae | Erysipelotrichales | Erysipelotrichia | Firmicutes |
| Clostridia sp.  (HG3A.1008) | T90 | -0.052 | 0.003 | 0.025 | 3364 unclassified | unclassified | unclassified | unclassified | unclassified | Clostridia | Firmicutes |
| Eubacteriales sp. (HG3A.0232) | ODI | -0.049 | 0.005 | 0.025 | 3364 unclassified | unclassified | unclassified | unclassified | Eubacteriales | Clostridia | Firmicutes |

AHI -0.059 0.001 0.023 3004 unclassified unclassified unclassified unclassified Eubacteriales Clostridia Firmicutes

Eubacteriales sp. (HG3A.1379)

| Firmicutes sp. (HG3A.0397) | T90 | -0.052 | 0.003 | 0.025 | 3364 unclassified | unclassified | unclassified unclassified | unclassified | unclassified | Firmicutes |
| --- | --- | --- | --- | --- | --- | --- | --- | --- | --- | --- |
| Firmicutes sp.  (HG3A.0454) | ODI | -0.048 | 0.005 | 0.025 | 3364 unclassified | unclassified | unclassified unclassified | unclassified | unclassified | Firmicutes |
| Holdemanella sp. (HG3A.0366) | ODI | 0.049 | 0.005 | 0.025 | 3364 unclassified | unclassified | Holdemanella Erysipelotrichaceae | Erysipelotrichales | Erysipelotrichia | Firmicutes |
| Oscillospiraceae sp.  (HG3A.0437) | T90 | -0.052 | 0.003 | 0.025 | 3364 unclassified | unclassified | unclassified Oscillospiraceae | Eubacteriales | Clostridia | Firmicutes |
| Roseburia intestinalis (HG3A.0078) | ODI | 0.049 | 0.005 | 0.025 | 3364 unclassified | Roseburia intestinalis | Roseburia Lachnospiraceae | Eubacteriales | Clostridia | Firmicutes |
| Bacteria sp.  (HG3A.0911) | T90 | -0.051 | 0.003 | 0.026 | 3364 unclassified | unclassified | unclassified unclassified | unclassified | unclassified | unclassified |
| Eubacteriales sp. (HG3A.0600) | T90 | -0.052 | 0.003 | 0.026 | 3364 unclassified | unclassified | unclassified unclassified | Eubacteriales | Clostridia | Firmicutes |
| Eubacteriales sp.  (HG3A.0771) | T90 | -0.051 | 0.003 | 0.026 | 3364 unclassified | unclassified | unclassified unclassified | Eubacteriales | Clostridia | Firmicutes |
| Parvimonas micra (HG3A.1231) | T90 | 0.052 | 0.003 | 0.026 | 3364 unclassified | Parvimonas micra | Parvimonas Peptoniphilaceae | Tissierellales | Tissierellia | Firmicutes |
| Acidaminococcus intestini (HG3A.0407) | ODI | 0.048 | 0.006 | 0.027 | 3364 unclassified | Acidaminococcus intestini | Acidaminococcus Acidaminococcaceae | Acidaminococcales | Negativicutes | Firmicutes |
| Bacteria sp. (HG3A.0483) | AHI | -0.058 | 0.002 | 0.027 | 3004 unclassified | unclassified | unclassified unclassified | unclassified | unclassified | unclassified |
| Blautia obeum  (HG3A.0001) | AHI | 0.058 | 0.002 | 0.027 | 3004 unclassified | Blautia obeum | Blautia Lachnospiraceae | Eubacteriales | Clostridia | Firmicutes |
| Butyricicoccus sp. OM04-18BH (HG3A.0139) | ODI | -0.048 | 0.006 | 0.027 | 3364 unclassified | Butyricicoccus sp. OM04- 18BH | Butyricicoccus Clostridiaceae | Eubacteriales | Clostridia | Firmicutes |
| Clostridia sp.  (HG3A.0750) | AHI | -0.058 | 0.002 | 0.027 | 3004 unclassified | unclassified | unclassified unclassified | unclassified | Clostridia | Firmicutes |
| Eubacteriales sp. (HG3A.0118) | AHI | -0.058 | 0.002 | 0.027 | 3004 unclassified | unclassified | unclassified unclassified | Eubacteriales | Clostridia | Firmicutes |
| Eubacteriales sp.  (HG3A.0600) | ODI | -0.048 | 0.006 | 0.027 | 3364 unclassified | unclassified | unclassified unclassified | Eubacteriales | Clostridia | Firmicutes |
| Firmicutes sp. (HG3A.0681) | AHI | -0.058 | 0.002 | 0.027 | 3004 unclassified | unclassified | unclassified unclassified | unclassified | unclassified | Firmicutes |
| Clostridia sp.  (HG3A.0724) | AHI | -0.057 | 0.002 | 0.028 | 3004 unclassified | unclassified | unclassified unclassified | unclassified | Clostridia | Firmicutes |
| Clostridia sp. (HG3A.0741) | AHI | -0.057 | 0.002 | 0.028 | 3004 unclassified | unclassified | unclassified unclassified | unclassified | Clostridia | Firmicutes |

ODI -0.049 0.005 0.025 3364 unclassified unclassified unclassified unclassified Eubacteriales Clostridia Firmicutes

Eubacteriales sp. (HG3A.0153)

| Eubacteriales sp. (HG3A.0179) | AHI | -0.057 | 0.002 | 0.028 | 3004 unclassified | unclassified | unclassified | unclassified | Eubacteriales | Clostridia | Firmicutes |
| --- | --- | --- | --- | --- | --- | --- | --- | --- | --- | --- | --- |
| Eubacteriales sp.  (HG3A.0427) | ODI | -0.048 | 0.006 | 0.028 | 3364 unclassified | unclassified | unclassified | unclassified | Eubacteriales | Clostridia | Firmicutes |
| Eubacterium sp. (HG3A.0214) | AHI | 0.057 | 0.002 | 0.028 | 3004 unclassified | unclassified | Eubacterium | Eubacteriaceae | Eubacteriales | Clostridia | Firmicutes |
| [Ruminococcus] torques (HG3A.0034) | AHI | 0.057 | 0.002 | 0.028 | 3004 unclassified | [Ruminococcus] torques | Mediterraneibact er | Lachnospiraceae | Eubacteriales | Clostridia | Firmicutes |
| Anaerotruncus colihominis (HG3A.0307) | T90 | 0.051 | 0.004 | 0.029 | 3364 unclassified | Anaerotruncus colihominis | Anaerotruncus | Oscillospiraceae | Eubacteriales | Clostridia | Firmicutes |
| Blautia massiliensis  (HG3A.0023) | AHI | 0.057 | 0.002 | 0.029 | 3004 unclassified | Blautia massiliensis | Blautia | Lachnospiraceae | Eubacteriales | Clostridia | Firmicutes |
| Clostridia sp. (HG3A.0645) | T90 | -0.051 | 0.004 | 0.029 | 3364 unclassified | unclassified | unclassified | unclassified | unclassified | Clostridia | Firmicutes |
| Coprococcus sp.  (HG3A.0404) | ODI | 0.048 | 0.006 | 0.029 | 3364 unclassified | unclassified | Coprococcus | Lachnospiraceae | Eubacteriales | Clostridia | Firmicutes |
| Eubacteriales sp. (HG3A.0179) | T90 | -0.051 | 0.004 | 0.029 | 3364 unclassified | unclassified | unclassified | unclassified | Eubacteriales | Clostridia | Firmicutes |
| Eubacteriales sp.  (HG3A.0474) | T90 | -0.051 | 0.004 | 0.029 | 3364 unclassified | unclassified | unclassified | unclassified | Eubacteriales | Clostridia | Firmicutes |
| Eubacteriales sp. (HG3A.0730) | T90 | -0.051 | 0.004 | 0.029 | 3364 unclassified | unclassified | unclassified | unclassified | Eubacteriales | Clostridia | Firmicutes |
| Eubacteriales sp.  (HG3A.1019) | AHI | -0.056 | 0.002 | 0.029 | 3004 unclassified | unclassified | unclassified | unclassified | Eubacteriales | Clostridia | Firmicutes |
| Firmicutes sp. (HG3A.0301) | T90 | -0.051 | 0.004 | 0.029 | 3364 unclassified | unclassified | unclassified | unclassified | unclassified | unclassified | Firmicutes |
| Gemella morbillorum (HG3A.1782) | ODI | 0.047 | 0.006 | 0.029 | 3364 unclassified | Gemella morbillorum | Gemella | unclassified | Bacillales | Bacilli | Firmicutes |
| Anaerobutyricum hallii (HG3A.0112) | ODI | 0.047 | 0.007 | 0.03 | 3364 unclassified | Anaerobutyricum hallii | Anaerobutyricum | Lachnospiraceae | Eubacteriales | Clostridia | Firmicutes |
| Eggerthellales sp.  (HG3A.0177) | AHI | -0.056 | 0.002 | 0.03 | 3004 unclassified | unclassified | unclassified | unclassified | Eggerthellales | Coriobacteriia | Actinobacteria |
| Eisenbergiella tayi (HG3A.0355) | T90 | 0.05 | 0.004 | 0.03 | 3364 unclassified | Eisenbergiella tayi | Eisenbergiella | Lachnospiraceae | Eubacteriales | Clostridia | Firmicutes |
| Eubacteriales sp.  (HG3A.0149) | AHI | -0.056 | 0.002 | 0.03 | 3004 unclassified | unclassified | unclassified | unclassified | Eubacteriales | Clostridia | Firmicutes |
| Eubacteriales sp. (HG3A.0698) | ODI | 0.047 | 0.007 | 0.03 | 3364 unclassified | unclassified | unclassified | unclassified | Eubacteriales | Clostridia | Firmicutes |

AHI -0.057 0.002 0.028 3004 unclassified unclassified unclassified unclassified Eubacteriales Clostridia Firmicutes

Eubacteriales sp. (HG3A.0964)

| Firmicutes sp. (HG3A.0501) | AHI | -0.056 | 0.002 | 0.03 | 3004 unclassified | unclassified | unclassified | unclassified | unclassified | unclassified | Firmicutes |
| --- | --- | --- | --- | --- | --- | --- | --- | --- | --- | --- | --- |
| Lachnospiraceae sp.  (HG3A.0233) | AHI | -0.056 | 0.002 | 0.03 | 3004 unclassified | unclassified | unclassified | Lachnospiraceae | Eubacteriales | Clostridia | Firmicutes |
| Eubacteriales sp. (HG3A.0102) | AHI | -0.055 | 0.003 | 0.031 | 3004 unclassified | unclassified | unclassified | unclassified | Eubacteriales | Clostridia | Firmicutes |
| Eubacteriales sp.  (HG3A.0125) | AHI | -0.055 | 0.003 | 0.031 | 3004 unclassified | unclassified | unclassified | unclassified | Eubacteriales | Clostridia | Firmicutes |
| Eubacteriales sp. (HG3A.0703) | ODI | -0.047 | 0.007 | 0.031 | 3364 unclassified | unclassified | unclassified | unclassified | Eubacteriales | Clostridia | Firmicutes |
| Flavonifractor plautii (HG3A.0079) | AHI | 0.055 | 0.003 | 0.031 | 3004 unclassified | Flavonifractor plautii | Flavonifractor | Oscillospiraceae | Eubacteriales | Clostridia | Firmicutes |
| Ruminococcus sp.  AM42-11 (HG3A.0002) | ODI | 0.047 | 0.007 | 0.031 | 3364 unclassified | Ruminococcus sp. AM42- 11 | Ruminococcus | Oscillospiraceae | Eubacteriales | Clostridia | Firmicutes |
| Bacteria sp.  (HG3A.0483) | T90 | -0.05 | 0.004 | 0.032 | 3364 unclassified | unclassified | unclassified | unclassified | unclassified | unclassified | unclassified |
| Eubacteriales sp. (HG3A.0624) | ODI | -0.047 | 0.007 | 0.032 | 3364 unclassified | unclassified | unclassified | unclassified | Eubacteriales | Clostridia | Firmicutes |
| Clostridia sp.  (HG3A.0645) | ODI | -0.047 | 0.008 | 0.033 | 3364 unclassified | unclassified | unclassified | unclassified | unclassified | Clostridia | Firmicutes |
| Clostridia sp. (HG3A.1217) | ODI | -0.046 | 0.008 | 0.033 | 3364 unclassified | unclassified | unclassified | unclassified | unclassified | Clostridia | Firmicutes |
| Enterocloster citroniae (HG3A.0285) | ODI | 0.046 | 0.008 | 0.033 | 3364 unclassified | Enterocloster citroniae | Enterocloster | Lachnospiraceae | Eubacteriales | Clostridia | Firmicutes |
| Eubacteriales sp. (HG3A.0128) | T90 | 0.05 | 0.004 | 0.033 | 3364 unclassified | unclassified | unclassified | unclassified | Eubacteriales | Clostridia | Firmicutes |
| Eubacteriales sp.  (HG3A.0531) | ODI | -0.046 | 0.008 | 0.033 | 3364 unclassified | unclassified | unclassified | unclassified | Eubacteriales | Clostridia | Firmicutes |
| Eubacteriales sp. (HG3A.0565) | ODI | -0.046 | 0.008 | 0.033 | 3364 unclassified | unclassified | unclassified | unclassified | Eubacteriales | Clostridia | Firmicutes |
| Eubacteriales sp.  (HG3A.0730) | ODI | -0.047 | 0.007 | 0.033 | 3364 unclassified | unclassified | unclassified | unclassified | Eubacteriales | Clostridia | Firmicutes |
| Eubacterium sp. AM49- 13BH (HG3A.0251) | ODI | -0.046 | 0.008 | 0.033 | 3364 unclassified | Eubacterium sp. AM49- 13BH | Eubacterium | Eubacteriaceae | Eubacteriales | Clostridia | Firmicutes |
| Firmicutes sp.  (HG3A.0641) | ODI | -0.046 | 0.008 | 0.033 | 3364 unclassified | unclassified | unclassified | unclassified | unclassified | unclassified | Firmicutes |
| Lachnospiraceae sp. (HG3A.0180) | T90 | -0.05 | 0.004 | 0.033 | 3364 unclassified | unclassified | unclassified | Lachnospiraceae | Eubacteriales | Clostridia | Firmicutes |

AHI -0.056 0.002 0.03 3004 unclassified unclassified unclassified unclassified Eubacteriales Clostridia Firmicutes

Traorella massiliensis

(HG3A.0669) T90 -0.05 0.004 0.033 3364 unclassified Traorella massiliensis Traorella Erysipelotrichaceae Erysipelotrichales Erysipelotrichia Firmicutes

fermentum (HG3A.0990)

| Candidatus Candidatus  Borkfalkiales sp. ODI -0.046 0.008 0.034 3364 unclassified unclassified unclassified unclassified Borkfalkiales Clostridia Firmicutes  (HG3A.1397) | | | | | | | | | | | |
| --- | --- | --- | --- | --- | --- | --- | --- | --- | --- | --- | --- |
| Clostridium sp. SN20  (HG3A.0603) | ODI | 0.046 | 0.008 | 0.034 | 3364 unclassified | Clostridium sp. SN20 | Clostridium | Clostridiaceae | Eubacteriales | Clostridia | Firmicutes |
| Eubacteriales sp. (HG3A.0453) | ODI | -0.046 | 0.008 | 0.034 | 3364 unclassified | unclassified | unclassified | unclassified | Eubacteriales | Clostridia | Firmicutes |
| Eubacteriales sp.  (HG3A.0671) | ODI | -0.046 | 0.008 | 0.034 | 3364 unclassified | unclassified | unclassified | unclassified | Eubacteriales | Clostridia | Firmicutes |
| Eubacteriales sp. (HG3A.0976) | ODI | -0.046 | 0.008 | 0.034 | 3364 unclassified | unclassified | unclassified | unclassified | Eubacteriales | Clostridia | Firmicutes |
| Eubacteriales sp.  (HG3A.1377) | ODI | -0.046 | 0.008 | 0.034 | 3364 unclassified | unclassified | unclassified | unclassified | Eubacteriales | Clostridia | Firmicutes |
| Firmicutes sp. (HG3A.0650) | ODI | -0.046 | 0.008 | 0.034 | 3364 unclassified | unclassified | unclassified | unclassified | unclassified | unclassified | Firmicutes |
| Oscillospiraceae sp.  (HG3A.0429) | ODI | -0.046 | 0.008 | 0.034 | 3364 unclassified | unclassified | unclassified | Oscillospiraceae | Eubacteriales | Clostridia | Firmicutes |
| Amedibacillus dolichus (HG3A.0798) | AHI | 0.055 | 0.003 | 0.035 | 3004 unclassified | Amedibacillus dolichus | Amedibacillus | Erysipelotrichaceae | Erysipelotrichales | Erysipelotrichia | Firmicutes |
| Clostridia sp.  (HG3A.1020) | T90 | -0.049 | 0.005 | 0.035 | 3364 unclassified | unclassified | unclassified | unclassified | unclassified | Clostridia | Firmicutes |
| Clostridia sp. (HG3A.1375) | ODI | -0.046 | 0.009 | 0.035 | 3364 unclassified | unclassified | unclassified | unclassified | unclassified | Clostridia | Firmicutes |
| Coprococcus eutactus (HG3A.0155) | T90 | -0.049 | 0.005 | 0.035 | 3364 unclassified | Coprococcus eutactus | Coprococcus | Lachnospiraceae | Eubacteriales | Clostridia | Firmicutes |
| Eubacteriales sp. (HG3A.0249) | T90 | -0.049 | 0.005 | 0.035 | 3364 unclassified | unclassified | unclassified | unclassified | Eubacteriales | Clostridia | Firmicutes |
| Eubacteriales sp.  (HG3A.0592) | T90 | -0.049 | 0.005 | 0.035 | 3364 unclassified | unclassified | unclassified | unclassified | Eubacteriales | Clostridia | Firmicutes |
| Eubacteriales sp. (HG3A.0653) | ODI | -0.046 | 0.009 | 0.035 | 3364 unclassified | unclassified | unclassified | unclassified | Eubacteriales | Clostridia | Firmicutes |
| Eubacteriales sp. (HG3A.0908)  Limosilactobacillus | ODI | -0.046 | 0.009 | 0.035 | 3364 unclassified | unclassified  Limosilactobacillus | unclassified  Limosilactobacill | unclassified | Eubacteriales | Clostridia | Firmicutes |

ODI 0.046 0.009 0.035 3364 unclassified

fermentum

us Lactobacillaceae Lactobacillales Bacilli Firmicutes

Oscillospiraceae sp. (HG3A.0146)

T90 -0.049 0.005 0.035 3364 unclassified unclassified unclassified Oscillospiraceae Eubacteriales Clostridia Firmicutes

| Clostridia sp.  (HG3A.1504) | ODI | -0.045 | 0.009 | 0.036 | 3364 unclassified | unclassified | unclassified | unclassified | unclassified | Clostridia | Firmicutes |
| --- | --- | --- | --- | --- | --- | --- | --- | --- | --- | --- | --- |
| Clostridium sp. M62/1 (HG3A.0354) | ODI | 0.045 | 0.009 | 0.036 | 3364 unclassified | Clostridium sp. M62/1 | Clostridium | Clostridiaceae | Eubacteriales | Clostridia | Firmicutes |
| Eubacteriales sp. (HG3A.0081) | ODI | -0.045 | 0.009 | 0.036 | 3364 unclassified | unclassified | unclassified | unclassified | Eubacteriales | Clostridia | Firmicutes |
| Eubacteriales sp.  (HG3A.0284) | T90 | -0.049 | 0.005 | 0.036 | 3364 unclassified | unclassified | unclassified | unclassified | Eubacteriales | Clostridia | Firmicutes |
| Eubacteriales sp. (HG3A.0443) | T90 | -0.049 | 0.005 | 0.036 | 3364 unclassified | unclassified | unclassified | unclassified | Eubacteriales | Clostridia | Firmicutes |
| Eubacteriales sp.  (HG3A.0084) | T90 | -0.048 | 0.005 | 0.037 | 3364 unclassified | unclassified | unclassified | unclassified | Eubacteriales | Clostridia | Firmicutes |
| Eubacteriales sp. (HG3A.0270) | ODI | -0.045 | 0.009 | 0.037 | 3364 unclassified | unclassified | unclassified | unclassified | Eubacteriales | Clostridia | Firmicutes |
| Eubacteriales sp.  (HG3A.0421) | T90 | -0.049 | 0.005 | 0.037 | 3364 unclassified | unclassified | unclassified | unclassified | Eubacteriales | Clostridia | Firmicutes |
| Eubacteriales sp. (HG3A.0786) | T90 | 0.048 | 0.005 | 0.037 | 3364 unclassified | unclassified | unclassified | unclassified | Eubacteriales | Clostridia | Firmicutes |
| Eubacteriales sp.  (HG3A.0964) | ODI | -0.045 | 0.01 | 0.037 | 3364 unclassified | unclassified | unclassified | unclassified | Eubacteriales | Clostridia | Firmicutes |
| Oscillospiraceae sp. (HG3A.0060) | ODI | -0.045 | 0.009 | 0.037 | 3364 unclassified | unclassified | unclassified | Oscillospiraceae | Eubacteriales | Clostridia | Firmicutes |
| Eubacteriales sp.  (HG3A.0691) | ODI | -0.045 | 0.01 | 0.038 | 3364 unclassified | unclassified | unclassified | unclassified | Eubacteriales | Clostridia | Firmicutes |
| Oscillospiraceae sp. (HG3A.0060) | AHI | -0.054 | 0.003 | 0.038 | 3004 unclassified | unclassified | unclassified | Oscillospiraceae | Eubacteriales | Clostridia | Firmicutes |
| Staphylococcus aureus (HG3A.1538) | ODI | 0.045 | 0.01 | 0.038 | 3364 unclassified | Staphylococcus aureus | Staphylococcus | Staphylococcaceae | Bacillales | Bacilli | Firmicutes |
| Clostridia sp. (HG3A.0599) | AHI | -0.054 | 0.004 | 0.039 | 3004 unclassified | unclassified | unclassified | unclassified | unclassified | Clostridia | Firmicutes |
| Clostridia sp.  (HG3A.1504) | AHI | -0.053 | 0.004 | 0.039 | 3004 unclassified | unclassified | unclassified | unclassified | unclassified | Clostridia | Firmicutes |
| Clostridium sp. TF06- 15AC (HG3A.0032) | AHI | 0.054 | 0.004 | 0.039 | 3004 unclassified | Clostridium sp. TF06- 15AC | Clostridium | Clostridiaceae | Eubacteriales | Clostridia | Firmicutes |
| Eubacteriales sp.  (HG3A.0120) | AHI | -0.054 | 0.004 | 0.039 | 3004 unclassified | unclassified | unclassified | unclassified | Eubacteriales | Clostridia | Firmicutes |
| Eubacteriales sp. (HG3A.0151) | AHI | -0.053 | 0.004 | 0.039 | 3004 unclassified | unclassified | unclassified | unclassified | Eubacteriales | Clostridia | Firmicutes |

Eubacteriales sp. (HG3A.0254)

AHI 0.053 0.004 0.039 3004 unclassified unclassified unclassified unclassified Eubacteriales Clostridia Firmicutes

| Eubacteriales sp.  (HG3A.0264) | AHI | -0.054 | 0.004 | 0.039 | 3004 unclassified | unclassified | unclassified | unclassified | Eubacteriales | Clostridia | Firmicutes |
| --- | --- | --- | --- | --- | --- | --- | --- | --- | --- | --- | --- |
| Eubacteriales sp.  (HG3A.0626) | ODI | -0.045 | 0.01 | 0.039 | 3364 unclassified | unclassified | unclassified | unclassified | Eubacteriales | Clostridia | Firmicutes |
| Eubacteriales sp. (HG3A.0730) | AHI | -0.053 | 0.004 | 0.039 | 3004 unclassified | unclassified | unclassified | unclassified | Eubacteriales | Clostridia | Firmicutes |
| Eubacteriales sp.  (HG3A.1379) | AHI | -0.053 | 0.004 | 0.039 | 3004 unclassified | unclassified | unclassified | unclassified | Eubacteriales | Clostridia | Firmicutes |
| Lachnospiraceae sp. (HG3A.0018) | AHI | 0.054 | 0.004 | 0.039 | 3004 unclassified | unclassified | unclassified | Lachnospiraceae | Eubacteriales | Clostridia | Firmicutes |
| Candidatus Borkfalkiales sp.  (HG3A.1284) | ODI | -0.044 | 0.011 | 0.04 | 3364 unclassified | unclassified | unclassified | unclassified | Candidatus Borkfalkiales | Clostridia | Firmicutes |
| Clostridia sp. (HG3A.0660) | ODI | -0.044 | 0.011 | 0.04 | 3364 unclassified | unclassified | unclassified | unclassified | unclassified | Clostridia | Firmicutes |
| Clostridia sp.  (HG3A.0746) | ODI | -0.044 | 0.011 | 0.04 | 3364 unclassified | unclassified | unclassified | unclassified | unclassified | Clostridia | Firmicutes |
| Eubacteriales sp. (HG3A.0715) | ODI | -0.044 | 0.011 | 0.04 | 3364 unclassified | unclassified | unclassified | unclassified | Eubacteriales | Clostridia | Firmicutes |
| Firmicutes sp.  (HG3A.1075) | ODI | -0.044 | 0.011 | 0.04 | 3364 unclassified | unclassified | unclassified | unclassified | unclassified | unclassified | Firmicutes |
| Lachnospiraceae sp. (HG3A.0018) | T90 | 0.048 | 0.006 | 0.04 | 3364 unclassified | unclassified | unclassified | Lachnospiraceae | Eubacteriales | Clostridia | Firmicutes |
| Ruminococcus champanellensis  (HG3A.0716) | ODI | -0.044 | 0.011 | 0.04 | 3364 unclassified | Ruminococcus champanellensis | Ruminococcus | Oscillospiraceae | Eubacteriales | Clostridia | Firmicutes |
| Sellimonas intestinalis (HG3A.0417) | ODI | 0.044 | 0.011 | 0.04 | 3364 unclassified | Sellimonas intestinalis | Sellimonas | Lachnospiraceae | Eubacteriales | Clostridia | Firmicutes |
| Alistipes communis  (HG3A.0064) | AHI | -0.053 | 0.004 | 0.041 | 3004 unclassified | Alistipes communis | Alistipes | Rikenellaceae | Bacteroidales | Bacteroidia | Bacteroidetes |
| Alistipes provencensis (HG3A.0877) | T90 | -0.048 | 0.006 | 0.041 | 3364 unclassified | Alistipes provencensis | Alistipes | Rikenellaceae | Bacteroidales | Bacteroidia | Bacteroidetes |
| Blautia massiliensis (HG3A.0023) | T90 | 0.048 | 0.006 | 0.041 | 3364 unclassified | Blautia massiliensis | Blautia | Lachnospiraceae | Eubacteriales | Clostridia | Firmicutes |
| Clostridia sp. (HG3A.1148) | AHI | -0.053 | 0.004 | 0.041 | 3004 unclassified | unclassified | unclassified | unclassified | unclassified | Clostridia | Firmicutes |
| Eubacteriales sp.  (HG3A.0331) | T90 | -0.048 | 0.006 | 0.041 | 3364 unclassified | unclassified | unclassified | unclassified | Eubacteriales | Clostridia | Firmicutes |
| Blautia |  |  |  |  |  |  |  |  |  |  |  |
| hydrogenotrophica ODI 0.044 0.011 0.042 3364 unclassified Blautia hydrogenotrophica Blautia Lachnospiraceae Eubacteriales Clostridia Firmicutes  (HG3A.0430) | | | | | | | | | | | |

Eubacteriales sp. (HG3A.0604)

| Eubacterium sp. AF16- 48 (HG3A.0219) | T90 | -0.047 | 0.006 | 0.042 | 3364 unclassified | Eubacterium sp. AF16-48 | Eubacterium | Eubacteriaceae | Eubacteriales | Clostridia | Firmicutes |
| --- | --- | --- | --- | --- | --- | --- | --- | --- | --- | --- | --- |
| Clostridium sp. TF06- 15AC (HG3A.0032) | T90 | 0.047 | 0.007 | 0.043 | 3364 unclassified | Clostridium sp. TF06- 15AC | Clostridium | Clostridiaceae | Eubacteriales | Clostridia | Firmicutes |
| Collinsella sp. WCA1- 178-WT-3 (M2) (HG3A.1245) | T90 | 0.047 | 0.007 | 0.043 | 3364 unclassified | Collinsella sp. WCA1-178- WT-3 (M2) | Collinsella | Coriobacteriaceae | Coriobacteriales | Coriobacteriia | Actinobacteria |
| Eubacteriales sp.  (HG3A.0151) | ODI | -0.044 | 0.012 | 0.043 | 3364 unclassified | unclassified | unclassified | unclassified | Eubacteriales | Clostridia | Firmicutes |
| Eubacteriales sp. (HG3A.0870) | ODI | -0.044 | 0.012 | 0.043 | 3364 unclassified | unclassified | unclassified | unclassified | Eubacteriales | Clostridia | Firmicutes |
| Alistipes shahii  (HG3A.0054) | T90 | -0.047 | 0.007 | 0.044 | 3364 unclassified | Alistipes shahii | Alistipes | Rikenellaceae | Bacteroidales | Bacteroidia | Bacteroidetes |
| Clostridia sp. (HG3A.1057) | T90 | -0.047 | 0.007 | 0.044 | 3364 unclassified | unclassified | unclassified | unclassified | unclassified | Clostridia | Firmicutes |
| Clostridia sp.  (HG3A.1205) | T90 | -0.046 | 0.008 | 0.044 | 3364 unclassified | unclassified | unclassified | unclassified | unclassified | Clostridia | Firmicutes |
| Clostridium sp. SN20 (HG3A.0603) | T90 | 0.046 | 0.008 | 0.044 | 3364 unclassified | Clostridium sp. SN20 | Clostridium | Clostridiaceae | Eubacteriales | Clostridia | Firmicutes |
| Eubacteriales sp.  (HG3A.0232) | T90 | -0.047 | 0.007 | 0.044 | 3364 unclassified | unclassified | unclassified | unclassified | Eubacteriales | Clostridia | Firmicutes |
| Eubacteriales sp. (HG3A.0250) | T90 | -0.047 | 0.007 | 0.044 | 3364 unclassified | unclassified | unclassified | unclassified | Eubacteriales | Clostridia | Firmicutes |
| Eubacteriales sp.  (HG3A.0321) | AHI | -0.052 | 0.005 | 0.044 | 3004 unclassified | unclassified | unclassified | unclassified | Eubacteriales | Clostridia | Firmicutes |
| Eubacteriales sp. (HG3A.0621) | T90 | -0.047 | 0.007 | 0.044 | 3364 unclassified | unclassified | unclassified | unclassified | Eubacteriales | Clostridia | Firmicutes |
| Eubacteriales sp.  (HG3A.0635) | ODI | -0.044 | 0.012 | 0.044 | 3364 unclassified | unclassified | unclassified | unclassified | Eubacteriales | Clostridia | Firmicutes |
| Eubacteriales sp. (HG3A.0856) | T90 | -0.047 | 0.007 | 0.044 | 3364 unclassified | unclassified | unclassified | unclassified | Eubacteriales | Clostridia | Firmicutes |
| Eubacterium sp. AF22- 8LB (HG3A.0838) | ODI | 0.044 | 0.012 | 0.044 | 3364 unclassified | Eubacterium sp. AF22- 8LB | Eubacterium | Eubacteriaceae | Eubacteriales | Clostridia | Firmicutes |
| Firmicutes sp. (HG3A.1075) | T90 | -0.047 | 0.007 | 0.044 | 3364 unclassified | unclassified | unclassified | unclassified | unclassified | unclassified | Firmicutes |
| Oscillospiraceae sp.  (HG3A.0210) | T90 | -0.047 | 0.007 | 0.044 | 3364 unclassified | unclassified | unclassified | Oscillospiraceae | Eubacteriales | Clostridia | Firmicutes |
| Oscillospiraceae sp. (HG3A.0739) | T90 | -0.047 | 0.007 | 0.044 | 3364 unclassified | unclassified | unclassified | Oscillospiraceae | Eubacteriales | Clostridia | Firmicutes |

T90 -0.047 0.006 0.042 3364 unclassified unclassified unclassified unclassified Eubacteriales Clostridia Firmicutes

Roseburia inulinivorans

(HG3A.0036) AHI 0.052 0.005 0.044 3004 unclassified Roseburia inulinivorans Roseburia Lachnospiraceae Eubacteriales Clostridia Firmicutes

| Victivallis vadensis (HG3A.0689) | T90 | -0.046 | 0.008 | 0.044 | 3364 unclassified | Victivallis vadensis | Victivallis | Victivallaceae | Victivallales | Lentisphaeria | Lentisphaerae |
| --- | --- | --- | --- | --- | --- | --- | --- | --- | --- | --- | --- |
| Clostridia sp.  (HG3A.0368) | ODI | -0.043 | 0.013 | 0.045 | 3364 unclassified | unclassified | unclassified | unclassified | unclassified | Clostridia | Firmicutes |
| Clostridia sp. (HG3A.0756) | AHI | -0.052 | 0.005 | 0.045 | 3004 unclassified | unclassified | unclassified | unclassified | unclassified | Clostridia | Firmicutes |
| Eubacteriales sp.  (HG3A.0081) | AHI | -0.052 | 0.005 | 0.045 | 3004 unclassified | unclassified | unclassified | unclassified | Eubacteriales | Clostridia | Firmicutes |
| Eubacteriales sp. (HG3A.0263) | ODI | -0.043 | 0.013 | 0.045 | 3364 unclassified | unclassified | unclassified | unclassified | Eubacteriales | Clostridia | Firmicutes |
| Eubacteriales sp.  (HG3A.0282) | ODI | -0.043 | 0.013 | 0.045 | 3364 unclassified | unclassified | unclassified | unclassified | Eubacteriales | Clostridia | Firmicutes |
| Eubacteriales sp. (HG3A.0320) | ODI | -0.043 | 0.013 | 0.045 | 3364 unclassified | unclassified | unclassified | unclassified | Eubacteriales | Clostridia | Firmicutes |
| Eubacteriales sp.  (HG3A.0396) | ODI | -0.043 | 0.013 | 0.045 | 3364 unclassified | unclassified | unclassified | unclassified | Eubacteriales | Clostridia | Firmicutes |
| Eubacteriales sp. (HG3A.0426) | ODI | -0.043 | 0.013 | 0.045 | 3364 unclassified | unclassified | unclassified | unclassified | Eubacteriales | Clostridia | Firmicutes |
| Eubacteriales sp.  (HG3A.0509) | ODI | -0.043 | 0.013 | 0.045 | 3364 unclassified | unclassified | unclassified | unclassified | Eubacteriales | Clostridia | Firmicutes |
| Eubacteriales sp. (HG3A.1103) | ODI | -0.043 | 0.013 | 0.045 | 3364 unclassified | unclassified | unclassified | unclassified | Eubacteriales | Clostridia | Firmicutes |
| Lactobacillus gasseri (HG3A.0884) | ODI | 0.043 | 0.013 | 0.045 | 3364 unclassified | Lactobacillus gasseri | Lactobacillus | Lactobacillaceae | Lactobacillales | Bacilli | Firmicutes |
| Longicatena caecimuris (HG3A.0571) | ODI | 0.043 | 0.013 | 0.045 | 3364 unclassified | Longicatena caecimuris | Longicatena | Erysipelotrichaceae | Erysipelotrichales | Erysipelotrichia | Firmicutes |
| Oscillospiraceae sp.  (HG3A.0382) | ODI | -0.043 | 0.013 | 0.045 | 3364 unclassified | unclassified | unclassified | Oscillospiraceae | Eubacteriales | Clostridia | Firmicutes |
| Oscillospiraceae sp. (HG3A.0445) | AHI | -0.052 | 0.005 | 0.045 | 3004 unclassified | unclassified | unclassified | Oscillospiraceae | Eubacteriales | Clostridia | Firmicutes |
| Roseburia sp. AM59- 24XD (HG3A.0391) | AHI | -0.052 | 0.005 | 0.045 | 3004 unclassified | Roseburia sp. AM59- 24XD | Roseburia | Lachnospiraceae | Eubacteriales | Clostridia | Firmicutes |
| Eubacteriales sp. (HG3A.0153) | T90 | -0.046 | 0.008 | 0.046 | 3364 unclassified | unclassified | unclassified | unclassified | Eubacteriales | Clostridia | Firmicutes |

Firmicutes sp. (HG3A.0596)

ODI -0.043 0.013 0.046 3364 unclassified unclassified unclassified unclassified unclassified unclassified Firmicutes

Haemophilus parainfluenzae

(HG3A.0181)

T90

-0.046

0.008

0.046 3364 unclassified

Haemophilus

parainfluenzae

Haemophilus Pasteurellaceae

Pasteurellales

Gammaproteoba

cteria Proteobacteria

| Oscillibacter sp. PEA192 (HG3A.0021) | T90 | 0.046 | 0.008 | 0.046 | 3364 unclassified | Oscillibacter sp. PEA192 | Oscillibacter | Oscillospiraceae | Eubacteriales | Clostridia | Firmicutes |
| --- | --- | --- | --- | --- | --- | --- | --- | --- | --- | --- | --- |
| Bacteria sp. (HG3A.0492) | AHI | -0.051 | 0.005 | 0.047 | 3004 unclassified | unclassified | unclassified | unclassified | unclassified | unclassified | unclassified |
| Clostridia sp.  (HG3A.0470) | AHI | -0.051 | 0.005 | 0.047 | 3004 unclassified | unclassified | unclassified | unclassified | unclassified | Clostridia | Firmicutes |
| Clostridia sp. (HG3A.0508) | AHI | -0.051 | 0.005 | 0.047 | 3004 unclassified | unclassified | unclassified | unclassified | unclassified | Clostridia | Firmicutes |
| Coprococcus comes (HG3A.0016) | AHI | 0.051 | 0.005 | 0.047 | 3004 unclassified | Coprococcus comes | Coprococcus | Lachnospiraceae | Eubacteriales | Clostridia | Firmicutes |
| Eubacteriales sp. (HG3A.0280) | AHI | -0.051 | 0.005 | 0.047 | 3004 unclassified | unclassified | unclassified | unclassified | Eubacteriales | Clostridia | Firmicutes |
| Eubacteriales sp.  (HG3A.0546) | ODI | -0.043 | 0.014 | 0.047 | 3364 unclassified | unclassified | unclassified | unclassified | Eubacteriales | Clostridia | Firmicutes |
| Oscillibacter sp. (HG3A.0245) | T90 | -0.046 | 0.008 | 0.047 | 3364 unclassified | unclassified | Oscillibacter | Oscillospiraceae | Eubacteriales | Clostridia | Firmicutes |
| Oscillospiraceae sp.  (HG3A.0256) | AHI | 0.051 | 0.006 | 0.047 | 3004 unclassified | unclassified | unclassified | Oscillospiraceae | Eubacteriales | Clostridia | Firmicutes |
| Butyricicoccus sp. (HG3A.0008) | ODI | 0.043 | 0.014 | 0.048 | 3364 unclassified | unclassified | Butyricicoccus | Clostridiaceae | Eubacteriales | Clostridia | Firmicutes |
| Butyricimonas virosa  (HG3A.0199) | ODI | -0.042 | 0.015 | 0.048 | 3364 unclassified | Butyricimonas virosa | Butyricimonas | Odoribacteraceae | Bacteroidales | Bacteroidia | Bacteroidetes |
| Clostridia sp. (HG3A.1020) | AHI | -0.051 | 0.006 | 0.048 | 3004 unclassified | unclassified | unclassified | unclassified | unclassified | Clostridia | Firmicutes |
| Clostridium sp. SN20  (HG3A.0603) | AHI | 0.051 | 0.006 | 0.048 | 3004 unclassified | Clostridium sp. SN20 | Clostridium | Clostridiaceae | Eubacteriales | Clostridia | Firmicutes |
| Coprococcus sp. AF21- 14LB (HG3A.1047) | AHI | 0.051 | 0.006 | 0.048 | 3004 unclassified | Coprococcus sp. AF21- 14LB | Coprococcus | Lachnospiraceae | Eubacteriales | Clostridia | Firmicutes |
| Eubacteriales sp.  (HG3A.0230) | ODI | -0.043 | 0.014 | 0.048 | 3364 unclassified | unclassified | unclassified | unclassified | Eubacteriales | Clostridia | Firmicutes |
| Eubacteriales sp. (HG3A.0260) | ODI | -0.043 | 0.014 | 0.048 | 3364 unclassified | unclassified | unclassified | unclassified | Eubacteriales | Clostridia | Firmicutes |
| Eubacteriales sp.  (HG3A.0280) | ODI | -0.042 | 0.015 | 0.048 | 3364 unclassified | unclassified | unclassified | unclassified | Eubacteriales | Clostridia | Firmicutes |
| Eubacteriales sp. (HG3A.0377) | ODI | -0.043 | 0.014 | 0.048 | 3364 unclassified | unclassified | unclassified | unclassified | Eubacteriales | Clostridia | Firmicutes |

Eubacteriales sp. (HG3A.0771)

ODI -0.043 0.014 0.048 3364 unclassified unclassified unclassified unclassified Eubacteriales Clostridia Firmicutes

| Eubacterium sp. AM49- 13BH (HG3A.0251) | AHI | -0.051 | 0.006 | 0.048 | 3004 unclassified | Eubacterium sp. AM49- 13BH | Eubacterium Eubacteriaceae | Eubacteriales | Clostridia | Firmicutes |
| --- | --- | --- | --- | --- | --- | --- | --- | --- | --- | --- |
| Faecalibacterium prausnitzii (HG3A.0025) | ODI | 0.043 | 0.014 | 0.048 | 3364 unclassified | Faecalibacterium prausnitzii | Faecalibacterium Oscillospiraceae | Eubacteriales | Clostridia | Firmicutes |
| Firmicutes sp. (HG3A.0923) | ODI | -0.043 | 0.014 | 0.048 | 3364 unclassified | unclassified | unclassified unclassified | unclassified | unclassified | Firmicutes |
| Eubacteriales sp.  (HG3A.0242) | AHI | -0.05 | 0.006 | 0.049 | 3004 unclassified | unclassified | unclassified unclassified | Eubacteriales | Clostridia | Firmicutes |
| Eubacteriales sp. (HG3A.0545) | T90 | -0.046 | 0.009 | 0.049 | 3364 unclassified | unclassified | unclassified unclassified | Eubacteriales | Clostridia | Firmicutes |
| Eubacteriales sp.  (HG3A.0758) | T90 | -0.046 | 0.009 | 0.049 | 3364 unclassified | unclassified | unclassified unclassified | Eubacteriales | Clostridia | Firmicutes |
| Oscillibacter sp. (HG3A.0734) | AHI | -0.05 | 0.006 | 0.049 | 3004 unclassified | unclassified | Oscillibacter Oscillospiraceae | Eubacteriales | Clostridia | Firmicutes |
| Oscillospiraceae sp.  (HG3A.0210) | AHI | -0.05 | 0.006 | 0.049 | 3004 unclassified | unclassified | unclassified Oscillospiraceae | Eubacteriales | Clostridia | Firmicutes |
| Alistipes sp. An66 (HG3A.1535) | ODI | -0.042 | 0.015 | 0.05 | 3364 unclassified | Alistipes sp. An66 | Alistipes Rikenellaceae | Bacteroidales | Bacteroidia | Bacteroidetes |
| Clostridia sp.  (HG3A.0783) | T90 | -0.045 | 0.009 | 0.05 | 3364 unclassified | unclassified | unclassified unclassified | unclassified | Clostridia | Firmicutes |
| Clostridiaceae sp. (HG3A.0431) | AHI | 0.05 | 0.006 | 0.05 | 3004 unclassified | unclassified | unclassified Clostridiaceae | Eubacteriales | Clostridia | Firmicutes |
| Eubacteriaceae sp.  (HG3A.0591) | T90 | 0.045 | 0.009 | 0.05 | 3364 unclassified | unclassified | unclassified Eubacteriaceae | Eubacteriales | Clostridia | Firmicutes |
| Eubacteriales sp. (HG3A.0887) | ODI | -0.042 | 0.015 | 0.05 | 3364 unclassified | unclassified | unclassified unclassified | Eubacteriales | Clostridia | Firmicutes |
| Oscillospiraceae sp.  (HG3A.0774) | T90 | -0.045 | 0.009 | 0.05 | 3364 unclassified | unclassified | unclassified Oscillospiraceae | Eubacteriales | Clostridia | Firmicutes |
| Eubacteriales sp. (HG3A.0113) | ODI | -0.042 | 0.016 | 0.051 | 3364 unclassified | unclassified | unclassified unclassified | Eubacteriales | Clostridia | Firmicutes |
| Eubacteriales sp.  (HG3A.0468) | ODI | -0.042 | 0.016 | 0.051 | 3364 unclassified | unclassified | unclassified unclassified | Eubacteriales | Clostridia | Firmicutes |
| Firmicutes sp. (HG3A.0769) | ODI | -0.042 | 0.016 | 0.051 | 3364 unclassified | unclassified | unclassified unclassified | unclassified | unclassified | Firmicutes |
| Olsenella sp. AF21-51  (HG3A.0690) | ODI | -0.042 | 0.016 | 0.051 | 3364 unclassified | Olsenella sp. AF21-51 | Olsenella Atopobiaceae | Coriobacteriales | Coriobacteriia | Actinobacteria |
| Ruthenibacterium Ruthenibacterium  lactatiformans T90 0.045 0.009 0.051 3364 unclassified lactatiformans Ruthenibacterium Oscillospiraceae Eubacteriales Clostridia Firmicutes  (HG3A.0020) | | | | | | | | | | |

Alistipes sp. AF17-16 (HG3A.0150)

ODI -0.042 0.017 0.052 3364 unclassified Alistipes sp. AF17-16 Alistipes Rikenellaceae Bacteroidales Bacteroidia Bacteroidetes

| Clostridia sp.  (HG3A.0885) | ODI | -0.042 | 0.017 | 0.052 | 3364 unclassified | unclassified | unclassified | unclassified | unclassified | Clostridia | Firmicutes |
| --- | --- | --- | --- | --- | --- | --- | --- | --- | --- | --- | --- |
| Clostridia sp.  (HG3A.1356) | T90 | -0.045 | 0.01 | 0.052 | 3364 unclassified | unclassified | unclassified | unclassified | unclassified | Clostridia | Firmicutes |
| Eubacteriales sp. (HG3A.0120) | T90 | -0.045 | 0.01 | 0.052 | 3364 unclassified | unclassified | unclassified | unclassified | Eubacteriales | Clostridia | Firmicutes |
| Eubacterium ramulus (HG3A.0068) | T90 | 0.045 | 0.01 | 0.052 | 3364 unclassified | Eubacterium ramulus | Eubacterium | Eubacteriaceae | Eubacteriales | Clostridia | Firmicutes |
| Firmicutes sp. (HG3A.0541) | ODI | -0.042 | 0.016 | 0.052 | 3364 unclassified | unclassified | unclassified | unclassified | unclassified | unclassified | Firmicutes |
| Lachnospiraceae sp.  (HG3A.0127) | T90 | -0.045 | 0.01 | 0.052 | 3364 unclassified | unclassified | unclassified | Lachnospiraceae | Eubacteriales | Clostridia | Firmicutes |
| Clostridium sp. AT4 (HG3A.0347) | AHI | 0.05 | 0.007 | 0.053 | 3004 unclassified | Clostridium sp. AT4 | Clostridium | Clostridiaceae | Eubacteriales | Clostridia | Firmicutes |
| Eubacteriales sp.  (HG3A.0329) | AHI | -0.05 | 0.007 | 0.053 | 3004 unclassified | unclassified | unclassified | unclassified | Eubacteriales | Clostridia | Firmicutes |
| Eubacteriales sp. (HG3A.0572) | T90 | -0.045 | 0.01 | 0.053 | 3364 unclassified | unclassified | unclassified | unclassified | Eubacteriales | Clostridia | Firmicutes |
| Oscillospiraceae sp.  (HG3A.0380) | AHI | -0.05 | 0.007 | 0.053 | 3004 unclassified | unclassified | unclassified | Oscillospiraceae | Eubacteriales | Clostridia | Firmicutes |
| [Ruminococcus] torques (HG3A.0088) | AHI | 0.05 | 0.007 | 0.053 | 3004 unclassified | [Ruminococcus] torques | Mediterraneibact er | Lachnospiraceae | Eubacteriales | Clostridia | Firmicutes |
| Eubacteriales sp.  (HG3A.0854) | T90 | 0.045 | 0.01 | 0.054 | 3364 unclassified | unclassified | unclassified | unclassified | Eubacteriales | Clostridia | Firmicutes |
| Firmicutes sp. (HG3A.0587) | ODI | -0.041 | 0.017 | 0.054 | 3364 unclassified | unclassified | unclassified | unclassified | unclassified | unclassified | Firmicutes |
| Butyricicoccus sp.  (HG3A.0008) | T90 | 0.044 | 0.011 | 0.055 | 3364 unclassified | unclassified | Butyricicoccus | Clostridiaceae | Eubacteriales | Clostridia | Firmicutes |
| Eubacteriales sp. (HG3A.0328) | ODI | -0.041 | 0.018 | 0.055 | 3364 unclassified | unclassified | unclassified | unclassified | Eubacteriales | Clostridia | Firmicutes |
| Eubacteriales sp.  (HG3A.0331) | AHI | -0.049 | 0.008 | 0.055 | 3004 unclassified | unclassified | unclassified | unclassified | Eubacteriales | Clostridia | Firmicutes |
| Eubacteriales sp. (HG3A.0390) | ODI | -0.041 | 0.018 | 0.055 | 3364 unclassified | unclassified | unclassified | unclassified | Eubacteriales | Clostridia | Firmicutes |
| Holdemanella sp.  (HG3A.0366) | AHI | 0.049 | 0.007 | 0.055 | 3004 unclassified | unclassified | Holdemanella | Erysipelotrichaceae | Erysipelotrichales | Erysipelotrichia | Firmicutes |
| Oscillospiraceae sp. (HG3A.0384) | ODI | -0.041 | 0.018 | 0.055 | 3364 unclassified | unclassified | unclassified | Oscillospiraceae | Eubacteriales | Clostridia | Firmicutes |
| Eubacteriales sp.  (HG3A.0093) | T90 | -0.044 | 0.011 | 0.056 | 3364 unclassified | unclassified | unclassified | unclassified | Eubacteriales | Clostridia | Firmicutes |
| Eubacteriales sp. (HG3A.0132) | ODI | -0.041 | 0.018 | 0.056 | 3364 unclassified | unclassified | unclassified | unclassified | Eubacteriales | Clostridia | Firmicutes |

Eubacteriales sp. (HG3A.0221)

| Eubacteriales sp. (HG3A.0349) | ODI | -0.041 | 0.018 | 0.056 | 3364 unclassified | unclassified | unclassified | unclassified | Eubacteriales | Clostridia | Firmicutes |
| --- | --- | --- | --- | --- | --- | --- | --- | --- | --- | --- | --- |
| Clostridia sp.  (HG3A.0140) | AHI | -0.049 | 0.008 | 0.057 | 3004 unclassified | unclassified | unclassified | unclassified | unclassified | Clostridia | Firmicutes |
| Clostridia sp. (HG3A.1127) | ODI | -0.041 | 0.019 | 0.057 | 3364 unclassified | unclassified | unclassified | unclassified | unclassified | Clostridia | Firmicutes |
| Eubacteriales sp.  (HG3A.0083) | AHI | -0.049 | 0.008 | 0.057 | 3004 unclassified | unclassified | unclassified | unclassified | Eubacteriales | Clostridia | Firmicutes |
| Eubacteriales sp. (HG3A.0128) | ODI | 0.041 | 0.019 | 0.057 | 3364 unclassified | unclassified | unclassified | unclassified | Eubacteriales | Clostridia | Firmicutes |
| Eubacteriales sp.  (HG3A.0439) | ODI | -0.041 | 0.019 | 0.057 | 3364 unclassified | unclassified | unclassified | unclassified | Eubacteriales | Clostridia | Firmicutes |
| Eubacteriales sp. (HG3A.0489) | ODI | -0.041 | 0.019 | 0.057 | 3364 unclassified | unclassified | unclassified | unclassified | Eubacteriales | Clostridia | Firmicutes |
| Eubacteriales sp.  (HG3A.0976) | T90 | -0.044 | 0.011 | 0.057 | 3364 unclassified | unclassified | unclassified | unclassified | Eubacteriales | Clostridia | Firmicutes |
| Bacteria sp. (HG3A.0361) | T90 | -0.044 | 0.012 | 0.058 | 3364 unclassified | unclassified | unclassified | unclassified | unclassified | unclassified | unclassified |
| Clostridia sp.  (HG3A.0815) | T90 | -0.044 | 0.012 | 0.058 | 3364 unclassified | unclassified | unclassified | unclassified | unclassified | Clostridia | Firmicutes |
| Eubacteriales sp. (HG3A.0083) | T90 | -0.044 | 0.012 | 0.058 | 3364 unclassified | unclassified | unclassified | unclassified | Eubacteriales | Clostridia | Firmicutes |
| Eubacteriales sp.  (HG3A.0136) | ODI | -0.041 | 0.02 | 0.058 | 3364 unclassified | unclassified | unclassified | unclassified | Eubacteriales | Clostridia | Firmicutes |
| Eubacteriales sp. (HG3A.0329) | T90 | -0.044 | 0.012 | 0.058 | 3364 unclassified | unclassified | unclassified | unclassified | Eubacteriales | Clostridia | Firmicutes |
| Eubacteriales sp.  (HG3A.0760) | T90 | -0.044 | 0.011 | 0.058 | 3364 unclassified | unclassified | unclassified | unclassified | Eubacteriales | Clostridia | Firmicutes |
| Eubacterium ramulus (HG3A.0068) | ODI | 0.041 | 0.019 | 0.058 | 3364 unclassified | Eubacterium ramulus | Eubacterium | Eubacteriaceae | Eubacteriales | Clostridia | Firmicutes |
| Firmicutes sp.  (HG3A.1054) | T90 | -0.044 | 0.012 | 0.058 | 3364 unclassified | unclassified | unclassified | unclassified | unclassified | unclassified | Firmicutes |
| Intestinimonas massiliensis (HG3A.0198) | AHI | -0.049 | 0.008 | 0.058 | 3004 unclassified | Intestinimonas massiliensis | Intestinimonas | unclassified | Eubacteriales | Clostridia | Firmicutes |
| Lachnospiraceae sp.  (HG3A.0393) | T90 | 0.044 | 0.012 | 0.058 | 3364 unclassified | unclassified | unclassified | Lachnospiraceae | Eubacteriales | Clostridia | Firmicutes |
| Oscillospiraceae sp. (HG3A.0380) | ODI | -0.041 | 0.019 | 0.058 | 3364 unclassified | unclassified | unclassified | Oscillospiraceae | Eubacteriales | Clostridia | Firmicutes |

ODI -0.041 0.018 0.056 3364 unclassified unclassified unclassified unclassified Eubacteriales Clostridia Firmicutes

Oxalobacter formigenes

(HG3A.0552) T90 -0.044 0.011 0.058 3364 unclassified Oxalobacter formigenes Oxalobacter Oxalobacteraceae Burkholderiales

Betaproteobacteri

a Proteobacteria

| Roseburia intestinalis (HG3A.0078) | T90 | 0.044 | 0.012 | 0.058 | 3364 unclassified | Roseburia intestinalis | Roseburia | Lachnospiraceae | Eubacteriales | Clostridia | Firmicutes |
| --- | --- | --- | --- | --- | --- | --- | --- | --- | --- | --- | --- |
| Ruminococcus sp. AF46 10NS (HG3A.0271) | T90 | 0.044 | 0.012 | 0.058 | 3364 unclassified | Ruminococcus sp. AF46- 10NS | Ruminococcus | Oscillospiraceae | Eubacteriales | Clostridia | Firmicutes |
| Clostridia sp. (HG3A.0512) | ODI | -0.04 | 0.02 | 0.059 | 3364 unclassified | unclassified | unclassified | unclassified | unclassified | Clostridia | Firmicutes |
| Clostridia sp.  (HG3A.0660) | AHI | -0.049 | 0.008 | 0.059 | 3004 unclassified | unclassified | unclassified | unclassified | unclassified | Clostridia | Firmicutes |
| Clostridium sp. TM06- 18 (HG3A.0048) | ODI | 0.041 | 0.02 | 0.059 | 3364 unclassified | Clostridium sp. TM06-18 | Clostridium | Clostridiaceae | Eubacteriales | Clostridia | Firmicutes |
| Eubacteriales sp.  (HG3A.0254) | ODI | 0.041 | 0.02 | 0.059 | 3364 unclassified | unclassified | unclassified | unclassified | Eubacteriales | Clostridia | Firmicutes |
| Eubacteriales sp. (HG3A.0621) | ODI | -0.04 | 0.02 | 0.059 | 3364 unclassified | unclassified | unclassified | unclassified | Eubacteriales | Clostridia | Firmicutes |
| Oscillibacter sp. PEA192 (HG3A.0021) | ODI | 0.04 | 0.02 | 0.059 | 3364 unclassified | Oscillibacter sp. PEA192 | Oscillibacter | Oscillospiraceae | Eubacteriales | Clostridia | Firmicutes |
| Oscillospiraceae sp. (HG3A.0256) | ODI | 0.041 | 0.02 | 0.059 | 3364 unclassified | unclassified | unclassified | Oscillospiraceae | Eubacteriales | Clostridia | Firmicutes |
| Clostridia sp.  (HG3A.0550) | ODI | -0.04 | 0.021 | 0.06 | 3364 unclassified | unclassified | unclassified | unclassified | unclassified | Clostridia | Firmicutes |
| Eubacteriales sp. (HG3A.0548) | ODI | -0.04 | 0.021 | 0.06 | 3364 unclassified | unclassified | unclassified | unclassified | Eubacteriales | Clostridia | Firmicutes |
| Eubacteriales sp.  (HG3A.0666) | T90 | -0.043 | 0.013 | 0.06 | 3364 unclassified | unclassified | unclassified | unclassified | Eubacteriales | Clostridia | Firmicutes |
| Firmicutes sp. (HG3A.0526) | T90 | -0.043 | 0.013 | 0.06 | 3364 unclassified | unclassified | unclassified | unclassified | unclassified | unclassified | Firmicutes |
| Clostridia sp.  (HG3A.0011) | ODI | 0.04 | 0.021 | 0.061 | 3364 unclassified | unclassified | unclassified | unclassified | unclassified | Clostridia | Firmicutes |
| Eubacteriales sp. (HG3A.0085) | T90 | -0.043 | 0.013 | 0.061 | 3364 unclassified | unclassified | unclassified | unclassified | Eubacteriales | Clostridia | Firmicutes |
| Eubacteriales sp.  (HG3A.0373) | ODI | -0.04 | 0.022 | 0.061 | 3364 unclassified | unclassified | unclassified | unclassified | Eubacteriales | Clostridia | Firmicutes |
| Eubacteriales sp. (HG3A.0568) | ODI | -0.04 | 0.021 | 0.061 | 3364 unclassified | unclassified | unclassified | unclassified | Eubacteriales | Clostridia | Firmicutes |

Eubacteriales sp. (HG3A.0668)

T90 -0.043 0.013 0.061 3364 unclassified unclassified unclassified unclassified Eubacteriales Clostridia Firmicutes

| Eubacteriales sp.  (HG3A.0859) | ODI | -0.04 | 0.021 | 0.061 | 3364 unclassified | unclassified | unclassified | unclassified | Eubacteriales | Clostridia | Firmicutes |
| --- | --- | --- | --- | --- | --- | --- | --- | --- | --- | --- | --- |
| Eubacteriales sp.  (HG3A.1294) | T90 | -0.043 | 0.013 | 0.061 | 3364 unclassified | unclassified | unclassified | unclassified | Eubacteriales | Clostridia | Firmicutes |
| Anaerostipes caccae (HG3A.0747) | AHI | 0.048 | 0.009 | 0.062 | 3004 unclassified | Anaerostipes caccae | Anaerostipes | Lachnospiraceae | Eubacteriales | Clostridia | Firmicutes |
| Blautia obeum  (HG3A.0009) | AHI | 0.048 | 0.009 | 0.062 | 3004 unclassified | Blautia obeum | Blautia | Lachnospiraceae | Eubacteriales | Clostridia | Firmicutes |
| Blautia sp. (HG3A.0416) | AHI | 0.048 | 0.009 | 0.062 | 3004 unclassified | unclassified | Blautia | Lachnospiraceae | Eubacteriales | Clostridia | Firmicutes |
| Clostridia sp.  (HG3A.1217) | AHI | -0.048 | 0.009 | 0.062 | 3004 unclassified | unclassified | unclassified | unclassified | unclassified | Clostridia | Firmicutes |
| Eubacteriales sp. (HG3A.0342) | T90 | -0.043 | 0.013 | 0.062 | 3364 unclassified | unclassified | unclassified | unclassified | Eubacteriales | Clostridia | Firmicutes |
| Eubacteriales sp.  (HG3A.0630) | ODI | -0.04 | 0.022 | 0.062 | 3364 unclassified | unclassified | unclassified | unclassified | Eubacteriales | Clostridia | Firmicutes |
| Eubacteriales sp. (HG3A.0653) | AHI | -0.048 | 0.009 | 0.062 | 3004 unclassified | unclassified | unclassified | unclassified | Eubacteriales | Clostridia | Firmicutes |
| Firmicutes sp.  (HG3A.0948) | ODI | -0.04 | 0.022 | 0.062 | 3364 unclassified | unclassified | unclassified | unclassified | unclassified | unclassified | Firmicutes |
| Roseburia intestinalis (HG3A.0078) | AHI | 0.048 | 0.009 | 0.062 | 3004 unclassified | Roseburia intestinalis | Roseburia | Lachnospiraceae | Eubacteriales | Clostridia | Firmicutes |
| Blautia argi  (HG3A.1450) | ODI | 0.04 | 0.022 | 0.063 | 3364 unclassified | Blautia argi | Blautia | Lachnospiraceae | Eubacteriales | Clostridia | Firmicutes |
| Clostridia sp. (HG3A.0272) | AHI | -0.048 | 0.01 | 0.063 | 3004 unclassified | unclassified | unclassified | unclassified | unclassified | Clostridia | Firmicutes |
| Clostridia sp.  (HG3A.0682) | T90 | -0.043 | 0.014 | 0.063 | 3364 unclassified | unclassified | unclassified | unclassified | unclassified | Clostridia | Firmicutes |
| Eubacteriales sp. (HG3A.0080) | T90 | 0.043 | 0.014 | 0.063 | 3364 unclassified | unclassified | unclassified | unclassified | Eubacteriales | Clostridia | Firmicutes |
| Eubacteriales sp.  (HG3A.0349) | AHI | -0.048 | 0.01 | 0.063 | 3004 unclassified | unclassified | unclassified | unclassified | Eubacteriales | Clostridia | Firmicutes |
| Lactobacillus gasseri (HG3A.0884) | AHI | 0.048 | 0.01 | 0.063 | 3004 unclassified | Lactobacillus gasseri | Lactobacillus | Lactobacillaceae | Lactobacillales | Bacilli | Firmicutes |
| Butyricicoccus sp. OM04-18BH  (HG3A.0139) | AHI | -0.048 | 0.01 | 0.064 | 3004 unclassified | Butyricicoccus sp. OM04- 18BH | Butyricicoccus | Clostridiaceae | Eubacteriales | Clostridia | Firmicutes |
| [Clostridium] innocuum (HG3A.0365) | ODI | 0.04 | 0.023 | 0.064 | 3364 unclassified | [Clostridium] innocuum | Erysipelatoclostri dium | Erysipelotrichaceae | Erysipelotrichales | Erysipelotrichia | Firmicutes |

Coprobacillus sp. (HG3A.0022)

T90 0.043 0.014 0.064 3364 unclassified unclassified Coprobacillus Coprobacillaceae Erysipelotrichales Erysipelotrichia Firmicutes

| Eubacteriales sp.  (HG3A.0316) | T90 | -0.043 | 0.014 | 0.064 | 3364 unclassified | unclassified | unclassified unclassified | Eubacteriales | Clostridia | Firmicutes |
| --- | --- | --- | --- | --- | --- | --- | --- | --- | --- | --- |
| Eubacteriales sp.  (HG3A.0568) | T90 | -0.043 | 0.014 | 0.064 | 3364 unclassified | unclassified | unclassified unclassified | Eubacteriales | Clostridia | Firmicutes |
| Eubacteriales sp. (HG3A.0910) | ODI | -0.04 | 0.023 | 0.064 | 3364 unclassified | unclassified | unclassified unclassified | Eubacteriales | Clostridia | Firmicutes |
| Clostridia sp.  (HG3A.0879) | T90 | -0.043 | 0.015 | 0.065 | 3364 unclassified | unclassified | unclassified unclassified | unclassified | Clostridia | Firmicutes |
| Eubacteriales sp. (HG3A.0514) | ODI | -0.039 | 0.023 | 0.065 | 3364 unclassified | unclassified | unclassified unclassified | Eubacteriales | Clostridia | Firmicutes |
| Firmicutes sp.  (HG3A.0650) | T90 | -0.043 | 0.015 | 0.065 | 3364 unclassified | unclassified | unclassified unclassified | unclassified | unclassified | Firmicutes |
| Lachnospiraceae sp. (HG3A.1190) | AHI | -0.047 | 0.01 | 0.065 | 3004 unclassified | unclassified | unclassified Lachnospiraceae | Eubacteriales | Clostridia | Firmicutes |
| Oscillospiraceae sp.  (HG3A.0060) | T90 | -0.042 | 0.015 | 0.065 | 3364 unclassified | unclassified | unclassified Oscillospiraceae | Eubacteriales | Clostridia | Firmicutes |
| Roseburia inulinivorans (HG3A.0036) | T90 | 0.042 | 0.015 | 0.065 | 3364 unclassified | Roseburia inulinivorans | Roseburia Lachnospiraceae | Eubacteriales | Clostridia | Firmicutes |
| Veillonella rogosae  (HG3A.0324) | AHI | -0.047 | 0.01 | 0.065 | 3004 unclassified | Veillonella rogosae | Veillonella Veillonellaceae | Veillonellales | Negativicutes | Firmicutes |
| Acidaminococcus intestini (HG3A.0407) | AHI | 0.047 | 0.011 | 0.066 | 3004 unclassified | Acidaminococcus intestini | Acidaminococcus Acidaminococcaceae | Acidaminococcales | Negativicutes | Firmicutes |
| Coprococcus sp. OM04- 5BH (HG3A.1028) | AHI | -0.047 | 0.011 | 0.066 | 3004 unclassified | Coprococcus sp. OM04- 5BH | Coprococcus Lachnospiraceae | Eubacteriales | Clostridia | Firmicutes |
| Eubacteriales sp. (HG3A.0197) | T90 | -0.042 | 0.015 | 0.066 | 3364 unclassified | unclassified | unclassified unclassified | Eubacteriales | Clostridia | Firmicutes |
| Eubacteriales sp.  (HG3A.0696) | ODI | -0.039 | 0.024 | 0.066 | 3364 unclassified | unclassified | unclassified unclassified | Eubacteriales | Clostridia | Firmicutes |
| Eubacteriales sp. (HG3A.1026) | T90 | -0.042 | 0.015 | 0.066 | 3364 unclassified | unclassified | unclassified unclassified | Eubacteriales | Clostridia | Firmicutes |
| Eubacteriales sp.  (HG3A.1129) | ODI | -0.039 | 0.024 | 0.066 | 3364 unclassified | unclassified | unclassified unclassified | Eubacteriales | Clostridia | Firmicutes |
| Firmicutes sp. (HG3A.0526) | ODI | -0.039 | 0.024 | 0.066 | 3364 unclassified | unclassified | unclassified unclassified | unclassified | unclassified | Firmicutes |
| Oscillospiraceae sp.  (HG3A.0223) | AHI | -0.047 | 0.011 | 0.066 | 3004 unclassified | unclassified | unclassified Oscillospiraceae | Eubacteriales | Clostridia | Firmicutes |
| Parvimonas micra (HG3A.1231) | AHI | 0.047 | 0.01 | 0.066 | 3004 unclassified | Parvimonas micra | Parvimonas Peptoniphilaceae | Tissierellales | Tissierellia | Firmicutes |

Desulfovibrionales sp. (HG3A.0727)

AHI 0.047 0.011 0.067 3004 unclassified unclassified unclassified unclassified Desulfovibrionales Deltaproteobacte Proteobacteria

ria

er

| Eubacteriales sp.  (HG3A.0254) | T90 | 0.042 | 0.015 | 0.067 | 3364 unclassified | unclassified | unclassified unclassified | Eubacteriales | Clostridia | Firmicutes |
| --- | --- | --- | --- | --- | --- | --- | --- | --- | --- | --- |
| Eubacteriales sp.  (HG3A.0486) | AHI | -0.047 | 0.011 | 0.067 | 3004 unclassified | unclassified | unclassified unclassified | Eubacteriales | Clostridia | Firmicutes |
| Eubacteriales sp. (HG3A.1191) | AHI | -0.047 | 0.011 | 0.067 | 3004 unclassified | unclassified | unclassified unclassified | Eubacteriales | Clostridia | Firmicutes |
| Oscillospiraceae sp.  (HG3A.0429) | AHI | -0.047 | 0.011 | 0.068 | 3004 unclassified | unclassified | unclassified Oscillospiraceae | Eubacteriales | Clostridia | Firmicutes |
| Clostridia sp. (HG3A.0724) | ODI | -0.039 | 0.025 | 0.069 | 3364 unclassified | unclassified | unclassified unclassified | unclassified | Clostridia | Firmicutes |
| Coprococcus eutactus (HG3A.0155) | AHI | -0.046 | 0.012 | 0.069 | 3004 unclassified | Coprococcus eutactus | Coprococcus Lachnospiraceae | Eubacteriales | Clostridia | Firmicutes |
| Dorea formicigenerans (HG3A.0006) | AHI | 0.046 | 0.012 | 0.069 | 3004 unclassified | Dorea formicigenerans | Dorea Lachnospiraceae | Eubacteriales | Clostridia | Firmicutes |
| Eubacteriales sp.  (HG3A.0546) | AHI | -0.046 | 0.012 | 0.069 | 3004 unclassified | unclassified | unclassified unclassified | Eubacteriales | Clostridia | Firmicutes |
| Eubacteriales sp. (HG3A.0718) | T90 | -0.042 | 0.016 | 0.069 | 3364 unclassified | unclassified | unclassified unclassified | Eubacteriales | Clostridia | Firmicutes |
| Eubacteriales sp.  (HG3A.0854) | ODI | 0.039 | 0.025 | 0.069 | 3364 unclassified | unclassified | unclassified unclassified | Eubacteriales | Clostridia | Firmicutes |
| Firmicutes sp. (HG3A.0587) | AHI | -0.046 | 0.012 | 0.069 | 3004 unclassified | unclassified | unclassified unclassified | unclassified | unclassified | Firmicutes |
| Lachnospiraceae sp.  (HG3A.0257) | AHI | -0.046 | 0.012 | 0.069 | 3004 unclassified | unclassified | unclassified Lachnospiraceae | Eubacteriales | Clostridia | Firmicutes |
| Mesosutterella multiformis (HG3A.0520) | AHI | -0.046 | 0.012 | 0.069 | 3004 unclassified | Mesosutterella multiformis | Mesosutterella Sutterellaceae | Burkholderiales | Betaproteobacteri a | Proteobacteria |
| Methanobrevibacter smithii (HG3A.0152) | ODI | -0.039 | 0.025 | 0.069 | 3364 unclassified | Methanobrevibacter smithii | Methanobrevibact Methanobacteriaceae | Methanobacteriales | Methanobacteria | Euryarchaeota |
| Oscillospiraceae sp. (HG3A.0388) | ODI | -0.039 | 0.025 | 0.069 | 3364 unclassified | unclassified | unclassified Oscillospiraceae | Eubacteriales | Clostridia | Firmicutes |
| Pediococcus acidilactici (HG3A.1468) | ODI | 0.039 | 0.025 | 0.069 | 3364 unclassified | Pediococcus acidilactici | Pediococcus Lactobacillaceae | Lactobacillales | Bacilli | Firmicutes |
| Ruminococcus sp. AF46 10NS (HG3A.0271) | ODI | 0.039 | 0.025 | 0.069 | 3364 unclassified | Ruminococcus sp. AF46- 10NS | Ruminococcus Oscillospiraceae | Eubacteriales | Clostridia | Firmicutes |

Streptococcus

anginosus (HG3A.0680) AHI 0.046 0.012 0.069 3004 unclassified Streptococcus anginosus Streptococcus Streptococcaceae Lactobacillales Bacilli Firmicutes

| Eubacteriales sp.  (HG3A.0229) | T90 | -0.042 | 0.016 | 0.07 | 3364 unclassified | unclassified | unclassified | unclassified | Eubacteriales | Clostridia | Firmicutes |
| --- | --- | --- | --- | --- | --- | --- | --- | --- | --- | --- | --- |
| Eubacteriales sp.  (HG3A.1239) | ODI | -0.039 | 0.026 | 0.07 | 3364 unclassified | unclassified | unclassified | unclassified | Eubacteriales | Clostridia | Firmicutes |
| Clostridia sp. (HG3A.1141) | ODI | -0.039 | 0.027 | 0.071 | 3364 unclassified | unclassified | unclassified | unclassified | unclassified | Clostridia | Firmicutes |
| Eubacteriales sp.  (HG3A.0758) | ODI | -0.039 | 0.027 | 0.071 | 3364 unclassified | unclassified | unclassified | unclassified | Eubacteriales | Clostridia | Firmicutes |
| Eubacteriales sp. (HG3A.0902) | ODI | -0.039 | 0.026 | 0.071 | 3364 unclassified | unclassified | unclassified | unclassified | Eubacteriales | Clostridia | Firmicutes |
| Clostridia sp.  (HG3A.0479) | T90 | -0.042 | 0.017 | 0.072 | 3364 unclassified | unclassified | unclassified | unclassified | unclassified | Clostridia | Firmicutes |
| Eubacteriales sp. (HG3A.1377) | AHI | -0.046 | 0.013 | 0.072 | 3004 unclassified | unclassified | unclassified | unclassified | Eubacteriales | Clostridia | Firmicutes |
| Clostridia sp.  (HG3A.0733) | T90 | -0.041 | 0.018 | 0.073 | 3364 unclassified | unclassified | unclassified | unclassified | unclassified | Clostridia | Firmicutes |
| Clostridium sp. M62/1 (HG3A.0354) | T90 | 0.041 | 0.017 | 0.073 | 3364 unclassified | Clostridium sp. M62/1 | Clostridium | Clostridiaceae | Eubacteriales | Clostridia | Firmicutes |
| Eubacteriales sp.  (HG3A.0092) | T90 | -0.041 | 0.017 | 0.073 | 3364 unclassified | unclassified | unclassified | unclassified | Eubacteriales | Clostridia | Firmicutes |
| Eubacteriales sp. (HG3A.0211) | T90 | -0.041 | 0.017 | 0.073 | 3364 unclassified | unclassified | unclassified | unclassified | Eubacteriales | Clostridia | Firmicutes |
| Eubacteriales sp.  (HG3A.0790) | T90 | -0.041 | 0.018 | 0.073 | 3364 unclassified | unclassified | unclassified | unclassified | Eubacteriales | Clostridia | Firmicutes |
| Eubacteriales sp. (HG3A.0807) | ODI | -0.038 | 0.028 | 0.073 | 3364 unclassified | unclassified | unclassified | unclassified | Eubacteriales | Clostridia | Firmicutes |
| Faecalibacterium prausnitzii (HG3A.0010) | T90 | 0.041 | 0.018 | 0.073 | 3364 unclassified | Faecalibacterium prausnitzii | Faecalibacterium | Oscillospiraceae | Eubacteriales | Clostridia | Firmicutes |
| Firmicutes sp. (HG3A.0581) | T90 | -0.041 | 0.018 | 0.073 | 3364 unclassified | unclassified | unclassified | unclassified | unclassified | unclassified | Firmicutes |
| Lachnoclostridium sp.  (HG3A.0655) | AHI | 0.046 | 0.013 | 0.073 | 3004 unclassified | unclassified | Lachnoclostridiu  m | Lachnospiraceae | Eubacteriales | Clostridia | Firmicutes |
| Oscillospiraceae sp. (HG3A.1421) | ODI | 0.038 | 0.027 | 0.073 | 3364 unclassified | unclassified | unclassified | Oscillospiraceae | Eubacteriales | Clostridia | Firmicutes |
| Actinomycetaceae sp.  (HG3A.1068) | ODI | -0.038 | 0.028 | 0.074 | 3364 unclassified | unclassified | unclassified | Actinomycetaceae | Actinomycetales | Actinomycetia | Actinobacteria |
| [Clostridium] symbiosum (HG3A.0370) | AHI | 0.046 | 0.014 | 0.074 | 3004 unclassified | [Clostridium] symbiosum | Lachnoclostridiu m | Lachnospiraceae | Eubacteriales | Clostridia | Firmicutes |
| Eubacteriales sp.  (HG3A.0540) | AHI | -0.046 | 0.013 | 0.074 | 3004 unclassified | unclassified | unclassified | unclassified | Eubacteriales | Clostridia | Firmicutes |
| Firmicutes sp. (HG3A.0596) | AHI | -0.045 | 0.014 | 0.074 | 3004 unclassified | unclassified | unclassified | unclassified | unclassified | unclassified | Firmicutes |

Clostridia sp. (HG3A.0479)

ODI -0.038 0.029 0.075 3364 unclassified unclassified unclassified unclassified unclassified Clostridia Firmicutes

| Eggerthella lenta (HG3A.0225) | ODI | 0.038 | 0.028 | 0.075 | 3364 unclassified | Eggerthella lenta | Eggerthella | Eggerthellaceae | Eggerthellales | Coriobacteriia | Actinobacteria |
| --- | --- | --- | --- | --- | --- | --- | --- | --- | --- | --- | --- |
| Eubacteriales sp.  (HG3A.0486) | T90 | -0.041 | 0.019 | 0.076 | 3364 unclassified | unclassified | unclassified | unclassified | Eubacteriales | Clostridia | Firmicutes |
| Blautia producta (HG3A.0905) | ODI | 0.038 | 0.03 | 0.077 | 3364 unclassified | Blautia producta | Blautia | Lachnospiraceae | Eubacteriales | Clostridia | Firmicutes |
| Blautia sp. SG-772  (HG3A.0063) | ODI | 0.038 | 0.03 | 0.077 | 3364 unclassified | Blautia sp. SG-772 | Blautia | Lachnospiraceae | Eubacteriales | Clostridia | Firmicutes |
| Clostridia sp. (HG3A.1403) | ODI | -0.038 | 0.03 | 0.077 | 3364 unclassified | unclassified | unclassified | unclassified | unclassified | Clostridia | Firmicutes |
| Coprococcus sp.  (HG3A.0404) | T90 | 0.041 | 0.019 | 0.077 | 3364 unclassified | unclassified | Coprococcus | Lachnospiraceae | Eubacteriales | Clostridia | Firmicutes |
| Eubacteriales sp. (HG3A.0977) | ODI | -0.038 | 0.03 | 0.077 | 3364 unclassified | unclassified | unclassified | unclassified | Eubacteriales | Clostridia | Firmicutes |
| Alistipes indistinctus (HG3A.0121) | ODI | -0.038 | 0.03 | 0.078 | 3364 unclassified | Alistipes indistinctus | Alistipes | Rikenellaceae | Bacteroidales | Bacteroidia | Bacteroidetes |
| Blautia producta (HG3A.0619) | ODI | 0.038 | 0.031 | 0.078 | 3364 unclassified | Blautia producta | Blautia | Lachnospiraceae | Eubacteriales | Clostridia | Firmicutes |
| Clostridia sp.  (HG3A.0435) | AHI | -0.045 | 0.014 | 0.078 | 3004 unclassified | unclassified | unclassified | unclassified | unclassified | Clostridia | Firmicutes |
| Clostridia sp. (HG3A.1504) | T90 | -0.041 | 0.019 | 0.078 | 3364 unclassified | unclassified | unclassified | unclassified | unclassified | Clostridia | Firmicutes |
| Clostridiaceae sp.  (HG3A.0238) | T90 | -0.041 | 0.02 | 0.078 | 3364 unclassified | unclassified | unclassified | Clostridiaceae | Eubacteriales | Clostridia | Firmicutes |
| Eubacteriales sp. (HG3A.0128) | AHI | 0.045 | 0.015 | 0.078 | 3004 unclassified | unclassified | unclassified | unclassified | Eubacteriales | Clostridia | Firmicutes |
| Eubacteriales sp.  (HG3A.0154) | AHI | -0.045 | 0.015 | 0.078 | 3004 unclassified | unclassified | unclassified | unclassified | Eubacteriales | Clostridia | Firmicutes |
| Eubacteriales sp. (HG3A.0377) | AHI | -0.045 | 0.015 | 0.078 | 3004 unclassified | unclassified | unclassified | unclassified | Eubacteriales | Clostridia | Firmicutes |
| Eubacteriales sp.  (HG3A.0613) | ODI | -0.038 | 0.03 | 0.078 | 3364 unclassified | unclassified | unclassified | unclassified | Eubacteriales | Clostridia | Firmicutes |
| Eubacteriales sp. (HG3A.0666) | AHI | -0.045 | 0.015 | 0.078 | 3004 unclassified | unclassified | unclassified | unclassified | Eubacteriales | Clostridia | Firmicutes |
| Eubacteriales sp.  (HG3A.1030) | ODI | -0.038 | 0.031 | 0.078 | 3364 unclassified | unclassified | unclassified | unclassified | Eubacteriales | Clostridia | Firmicutes |
| Eubacteriales sp. (HG3A.1445) | AHI | -0.045 | 0.015 | 0.078 | 3004 unclassified | unclassified | unclassified | unclassified | Eubacteriales | Clostridia | Firmicutes |

Firmicutes sp.

# (HG3A.1054)

ODI -0.038 0.031 0.078 3364 unclassified unclassified unclassified unclassified unclassified unclassified Firmicutes

# (HG3A.0855) (HG3A.0382)

[Ruminococcus] torques

(HG3A.0088)

T90

0.041

0.02

0.078 3364 unclassified [Ruminococcus] torques

Mediterraneibact

er

Lachnospiraceae

Eubacteriales

Clostridia

Firmicutes

| Lachnospiraceae sp. AHI | -0.045 | 0.015 | 0.078 | 3004 unclassified | unclassified | unclassified | Lachnospiraceae | Eubacteriales | Clostridia | Firmicutes |
| --- | --- | --- | --- | --- | --- | --- | --- | --- | --- | --- |
| Oscillospiraceae sp. T90 | -0.041 | 0.02 | 0.078 | 3364 unclassified | unclassified | unclassified | Oscillospiraceae | Eubacteriales | Clostridia | Firmicutes |

Sutterella seckii (HG3A.0561)

ODI 0.038 0.031 0.078 3364 unclassified Sutterella seckii Sutterella Sutterellaceae Burkholderiales Betaproteobacteri Proteobacteria

a

# (HG3A.0574) (HG3A.0450)

Haemophilus parainfluenzae

(HG3A.0181)

ODI

-0.038

0.031

0.079 3364 unclassified

Haemophilus

parainfluenzae

Haemophilus Pasteurellaceae

Pasteurellales

Gammaproteoba

cteria Proteobacteria

| Tyzzerella nexilis T90 | 0.041 | 0.02 | 0.078 | 3364 unclassified | Tyzzerella nexilis | Tyzzerella | Lachnospiraceae | Eubacteriales | Clostridia | Firmicutes |
| --- | --- | --- | --- | --- | --- | --- | --- | --- | --- | --- |
| Eubacteriales sp. ODI | -0.038 | 0.031 | 0.079 | 3364 unclassified | unclassified | unclassified | unclassified | Eubacteriales | Clostridia | Firmicutes |

Odoribacter splanchnicus (HG3A.0041)

Bacteroidales sp.

(HG3A.1446)

T90

-0.04

0.02

0.08 3364 unclassified unclassified

unclassified

unclassified

Bacteroidales

Bacteroidia

Bacteroidetes

ODI -0.037 0.031 0.079 3364 unclassified Odoribacter splanchnicus Odoribacter Odoribacteraceae Bacteroidales Bacteroidia Bacteroidetes

Clostridia sp. (HG3A.0368)

Eubacteriales sp.

(HG3A.0069)

T90

-0.04

0.021

0.08 3364 unclassified unclassified

unclassified

unclassified

Eubacteriales

Clostridia

Firmicutes

T90 -0.04 0.021 0.08 3364 unclassified unclassified unclassified unclassified unclassified Clostridia Firmicutes

Eubacteriales sp. (HG3A.0418)

Eubacteriales sp.

(HG3A.0666)

ODI

-0.037

0.032

0.08 3364 unclassified unclassified

unclassified

unclassified

Eubacteriales

Clostridia

Firmicutes

T90 -0.04 0.02 0.08 3364 unclassified unclassified unclassified unclassified Eubacteriales Clostridia Firmicutes

Eubacteriales sp. (HG3A.0983)

Longicatena caecimuris

(HG3A.0571)

T90

0.04

0.02

0.08 3364 unclassified Longicatena caecimuris Longicatena

Erysipelotrichaceae Erysipelotrichales Erysipelotrichia Firmicutes

T90 -0.04 0.021 0.08 3364 unclassified unclassified unclassified unclassified Eubacteriales Clostridia Firmicutes

Oscillospiraceae sp. (HG3A.0475)

Clostridia sp.

(HG3A.0479)

AHI

-0.044

0.016

0.082 3004 unclassified unclassified

unclassified

unclassified

unclassified

Clostridia

Firmicutes

ODI -0.037 0.032 0.081 3364 unclassified unclassified unclassified Oscillospiraceae Eubacteriales Clostridia Firmicutes

Eubacteriales sp. (HG3A.1129)

Bacteroides nordii

(HG3A.0290)

ODI

-0.037

0.033

0.083 3364 unclassified Bacteroides nordii

Bacteroides

Bacteroidaceae

Bacteroidales

Bacteroidia

Bacteroidetes

T90 -0.04 0.022 0.082 3364 unclassified unclassified unclassified unclassified Eubacteriales Clostridia Firmicutes

Oscillospiraceae sp. (HG3A.0616)

ODI -0.037 0.033 0.083 3364 unclassified unclassified unclassified Oscillospiraceae Eubacteriales Clostridia Firmicutes

| Bacteroidales sp.  (HG3A.0789) | T90 | -0.04 | 0.022 | 0.084 | 3364 unclassified | unclassified | unclassified | unclassified | Bacteroidales | Bacteroidia Bacteroidetes |
| --- | --- | --- | --- | --- | --- | --- | --- | --- | --- | --- |
| Eubacteriales sp.  (HG3A.0221) | T90 | -0.04 | 0.022 | 0.084 | 3364 unclassified | unclassified | unclassified | unclassified | Eubacteriales | Clostridia Firmicutes |
| Eubacteriales sp. (HG3A.0493) | T90 | -0.04 | 0.022 | 0.084 | 3364 unclassified | unclassified | unclassified | unclassified | Eubacteriales | Clostridia Firmicutes |
| Eubacteriales sp.  (HG3A.0653) | T90 | -0.04 | 0.022 | 0.084 | 3364 unclassified | unclassified | unclassified | unclassified | Eubacteriales | Clostridia Firmicutes |
| Firmicutes sp. (HG3A.1082) | ODI | -0.037 | 0.034 | 0.084 | 3364 unclassified | unclassified | unclassified | unclassified | unclassified | unclassified Firmicutes |
| Rothia mucilaginosa  (HG3A.0559) | ODI | 0.037 | 0.034 | 0.084 | 3364 unclassified | Rothia mucilaginosa | Rothia | Micrococcaceae | Micrococcales | Actinomycetia Actinobacteria |
| Eubacteriales sp. (HG3A.0472) | ODI | -0.037 | 0.034 | 0.085 | 3364 unclassified | unclassified | unclassified | unclassified | Eubacteriales | Clostridia Firmicutes |
| Eubacteriales sp.  (HG3A.0908) | T90 | -0.04 | 0.023 | 0.085 | 3364 unclassified | unclassified | unclassified | unclassified | Eubacteriales | Clostridia Firmicutes |
| Bacteroidales sp. (HG3A.0894) | T90 | -0.04 | 0.023 | 0.086 | 3364 unclassified | unclassified | unclassified | unclassified | Bacteroidales | Bacteroidia Bacteroidetes |
| Coprobacillus sp.  (HG3A.0022) | AHI | 0.044 | 0.017 | 0.086 | 3004 unclassified | unclassified | Coprobacillus | Coprobacillaceae | Erysipelotrichales | Erysipelotrichia Firmicutes |
| Coprococcus sp. AM27- 12LB (HG3A.0687) | ODI | 0.037 | 0.035 | 0.086 | 3364 unclassified | Coprococcus sp. AM27- 12LB | Coprococcus | Lachnospiraceae | Eubacteriales | Clostridia Firmicutes |
| Eubacteriales sp.  (HG3A.0316) | AHI | -0.044 | 0.017 | 0.086 | 3004 unclassified | unclassified | unclassified | unclassified | Eubacteriales | Clostridia Firmicutes |
| Eubacteriales sp. (HG3A.0426) | AHI | -0.044 | 0.017 | 0.086 | 3004 unclassified | unclassified | unclassified | unclassified | Eubacteriales | Clostridia Firmicutes |
| Eubacteriales sp.  (HG3A.0545) | AHI | -0.044 | 0.017 | 0.086 | 3004 unclassified | unclassified | unclassified | unclassified | Eubacteriales | Clostridia Firmicutes |
| Eubacteriales sp. (HG3A.0794) | ODI | -0.037 | 0.035 | 0.086 | 3364 unclassified | unclassified | unclassified | unclassified | Eubacteriales | Clostridia Firmicutes |
| Streptococcus oralis subsp. oralis  (HG3A.0705) | ODI | 0.037 | 0.035 | 0.086 | Streptococcus 3364 oralis subsp.  oralis | Streptococcus oralis | Streptococcus | Streptococcaceae | Lactobacillales | Bacilli Firmicutes |
| Sutterella seckii (HG3A.0561) | AHI | 0.044 | 0.017 | 0.086 | 3004 unclassified | Sutterella seckii | Sutterella | Sutterellaceae | Burkholderiales | Betaproteobacteri Proteobacteria a |
| Alistipes senegalensis (HG3A.0141) | AHI | -0.044 | 0.018 | 0.087 | 3004 unclassified | Alistipes senegalensis | Alistipes | Rikenellaceae | Bacteroidales | Bacteroidia Bacteroidetes |
| Eubacteriales sp. (HG3A.0161) | AHI | -0.044 | 0.018 | 0.087 | 3004 unclassified | unclassified | unclassified | unclassified | Eubacteriales | Clostridia Firmicutes |

Eubacteriales sp. (HG3A.0288)

AHI -0.044 0.018 0.087 3004 unclassified unclassified unclassified unclassified Eubacteriales Clostridia Firmicutes

| Eubacteriales sp.  (HG3A.0409) | AHI | -0.044 | 0.017 | 0.087 | 3004 unclassified | unclassified | unclassified | unclassified | Eubacteriales | Clostridia | Firmicutes |
| --- | --- | --- | --- | --- | --- | --- | --- | --- | --- | --- | --- |
| Oscillibacter sp.  (HG3A.0245) | AHI | -0.044 | 0.018 | 0.087 | 3004 unclassified | unclassified | Oscillibacter | Oscillospiraceae | Eubacteriales | Clostridia | Firmicutes |
| Oscillospiraceae sp. (HG3A.0665) | T90 | -0.039 | 0.024 | 0.087 | 3364 unclassified | unclassified | unclassified | Oscillospiraceae | Eubacteriales | Clostridia | Firmicutes |
| Alistipes ihumii  (HG3A.0106) | ODI | -0.036 | 0.036 | 0.088 | 3364 unclassified | Alistipes ihumii | Alistipes | Rikenellaceae | Bacteroidales | Bacteroidia | Bacteroidetes |
| Eubacteriales sp. (HG3A.0312) | T90 | -0.039 | 0.024 | 0.088 | 3364 unclassified | unclassified | unclassified | unclassified | Eubacteriales | Clostridia | Firmicutes |
| Eubacteriales sp.  (HG3A.0573) | ODI | -0.036 | 0.036 | 0.088 | 3364 unclassified | unclassified | unclassified | unclassified | Eubacteriales | Clostridia | Firmicutes |
| Eubacteriales sp. (HG3A.0589) | T90 | -0.039 | 0.024 | 0.088 | 3364 unclassified | unclassified | unclassified | unclassified | Eubacteriales | Clostridia | Firmicutes |
| Faecalibacterium prausnitzii (HG3A.0025) | T90 | 0.039 | 0.024 | 0.088 | 3364 unclassified | Faecalibacterium prausnitzii | Faecalibacterium | Oscillospiraceae | Eubacteriales | Clostridia | Firmicutes |
| Catenibacterium mitsuokai (HG3A.0775) | AHI | 0.043 | 0.019 | 0.089 | 3004 unclassified | Catenibacterium mitsuokai | Catenibacterium | Coprobacillaceae | Erysipelotrichales | Erysipelotrichia | Firmicutes |
| Eubacteriales sp.  (HG3A.0132) | AHI | -0.043 | 0.018 | 0.089 | 3004 unclassified | unclassified | unclassified | unclassified | Eubacteriales | Clostridia | Firmicutes |
| Eubacteriales sp. (HG3A.0617) | AHI | -0.043 | 0.019 | 0.089 | 3004 unclassified | unclassified | unclassified | unclassified | Eubacteriales | Clostridia | Firmicutes |
| Eubacteriales sp.  (HG3A.0972) | T90 | -0.039 | 0.024 | 0.089 | 3364 unclassified | unclassified | unclassified | unclassified | Eubacteriales | Clostridia | Firmicutes |
| Firmicutes sp. (HG3A.0641) | AHI | -0.043 | 0.019 | 0.089 | 3004 unclassified | unclassified | unclassified | unclassified | unclassified | unclassified | Firmicutes |
| Eubacteriales sp.  (HG3A.0062) | AHI | -0.043 | 0.019 | 0.09 | 3004 unclassified | unclassified | unclassified | unclassified | Eubacteriales | Clostridia | Firmicutes |
| Eubacteriales sp. (HG3A.0229) | AHI | -0.043 | 0.019 | 0.09 | 3004 unclassified | unclassified | unclassified | unclassified | Eubacteriales | Clostridia | Firmicutes |
| Eubacteriales sp.  (HG3A.0557) | AHI | -0.043 | 0.02 | 0.09 | 3004 unclassified | unclassified | unclassified | unclassified | Eubacteriales | Clostridia | Firmicutes |
| Firmicutes sp. (HG3A.1054) | AHI | -0.043 | 0.019 | 0.09 | 3004 unclassified | unclassified | unclassified | unclassified | unclassified | unclassified | Firmicutes |
| Bacteroidales sp.  (HG3A.0894) | ODI | -0.036 | 0.038 | 0.091 | 3364 unclassified | unclassified | unclassified | unclassified | Bacteroidales | Bacteroidia | Bacteroidetes |
| Eubacteriales sp. (HG3A.0757) | T90 | -0.039 | 0.025 | 0.091 | 3364 unclassified | unclassified | unclassified | unclassified | Eubacteriales | Clostridia | Firmicutes |
| Eubacteriales sp.  (HG3A.1377) | T90 | -0.039 | 0.025 | 0.091 | 3364 unclassified | unclassified | unclassified | unclassified | Eubacteriales | Clostridia | Firmicutes |
| Lachnospiraceae sp. (HG3A.1190) | ODI | -0.036 | 0.038 | 0.091 | 3364 unclassified | unclassified | unclassified | Lachnospiraceae | Eubacteriales | Clostridia | Firmicutes |

Anaerobutyricum hallii (HG3A.0112)

| Blastocystis sp. subtype 4 (HG3A.0446) | ODI | -0.036 | 0.039 | 0.092 | 3364 unclassified | Blastocystis sp. subtype 4 | Blastocystis | Blastocystidae | Opalinata | Bigyra | unclassified |
| --- | --- | --- | --- | --- | --- | --- | --- | --- | --- | --- | --- |
| Clostridia sp.  (HG3A.0933) | ODI | -0.036 | 0.039 | 0.092 | 3364 unclassified | unclassified | unclassified | unclassified | unclassified | Clostridia | Firmicutes |
| Collinsella sp. WCA1- 178-WT-3 (M2) (HG3A.1245) | AHI | 0.043 | 0.02 | 0.092 | 3004 unclassified | Collinsella sp. WCA1-178- WT-3 (M2) | Collinsella | Coriobacteriaceae | Coriobacteriales | Coriobacteriia | Actinobacteria |
| Eubacteriales sp.  (HG3A.0087) | AHI | -0.043 | 0.02 | 0.092 | 3004 unclassified | unclassified | unclassified | unclassified | Eubacteriales | Clostridia | Firmicutes |
| Eubacteriales sp. (HG3A.0282) | AHI | -0.043 | 0.021 | 0.092 | 3004 unclassified | unclassified | unclassified | unclassified | Eubacteriales | Clostridia | Firmicutes |
| Eubacteriales sp.  (HG3A.0338) | T90 | -0.039 | 0.026 | 0.092 | 3364 unclassified | unclassified | unclassified | unclassified | Eubacteriales | Clostridia | Firmicutes |
| Eubacteriales sp. (HG3A.0427) | AHI | -0.043 | 0.02 | 0.092 | 3004 unclassified | unclassified | unclassified | unclassified | Eubacteriales | Clostridia | Firmicutes |
| Eubacteriales sp.  (HG3A.0498) | ODI | 0.036 | 0.038 | 0.092 | 3364 unclassified | unclassified | unclassified | unclassified | Eubacteriales | Clostridia | Firmicutes |
| Eubacteriales sp. (HG3A.0592) | ODI | -0.036 | 0.039 | 0.092 | 3364 unclassified | unclassified | unclassified | unclassified | Eubacteriales | Clostridia | Firmicutes |
| Eubacteriales sp.  (HG3A.0771) | AHI | -0.043 | 0.021 | 0.092 | 3004 unclassified | unclassified | unclassified | unclassified | Eubacteriales | Clostridia | Firmicutes |
| Eubacteriales sp. (HG3A.1332) | ODI | -0.036 | 0.038 | 0.092 | 3364 unclassified | unclassified | unclassified | unclassified | Eubacteriales | Clostridia | Firmicutes |
| Evtepia gabavorous  (HG3A.0114) | T90 | 0.039 | 0.026 | 0.092 | 3364 unclassified | Evtepia gabavorous | Evtepia | unclassified | Eubacteriales | Clostridia | Firmicutes |
| Firmicutes sp. (HG3A.0641) | T90 | -0.039 | 0.026 | 0.092 | 3364 unclassified | unclassified | unclassified | unclassified | unclassified | unclassified | Firmicutes |
| Firmicutes sp.  (HG3A.0874) | ODI | -0.036 | 0.039 | 0.092 | 3364 unclassified | unclassified | unclassified | unclassified | unclassified | unclassified | Firmicutes |
| Bacteroidales sp. (HG3A.1236) | ODI | -0.036 | 0.04 | 0.094 | 3364 unclassified | unclassified | unclassified | unclassified | Bacteroidales | Bacteroidia | Bacteroidetes |
| Dorea sp. AF36-15AT  (HG3A.0052) | AHI | 0.043 | 0.021 | 0.094 | 3004 unclassified | Dorea sp. AF36-15AT | Dorea | Lachnospiraceae | Eubacteriales | Clostridia | Firmicutes |
| Eubacteriales sp. (HG3A.0635) | T90 | -0.039 | 0.027 | 0.094 | 3364 unclassified | unclassified | unclassified | unclassified | Eubacteriales | Clostridia | Firmicutes |
| Oscillospiraceae sp.  (HG3A.0475) | T90 | -0.039 | 0.027 | 0.094 | 3364 unclassified | unclassified | unclassified | Oscillospiraceae | Eubacteriales | Clostridia | Firmicutes |
| Alistipes sp. An66 (HG3A.1535) | AHI | -0.042 | 0.022 | 0.095 | 3004 unclassified | Alistipes sp. An66 | Alistipes | Rikenellaceae | Bacteroidales | Bacteroidia | Bacteroidetes |

AHI 0.043 0.02 0.092 3004 unclassified Anaerobutyricum hallii Anaerobutyricum Lachnospiraceae Eubacteriales Clostridia Firmicutes

Anaerotruncus colihominis (HG3A.0307)

| Clostridia sp. (HG3A.0512) | T90 | -0.038 | 0.027 | 0.095 | 3364 unclassified | unclassified | unclassified | unclassified | unclassified | Clostridia | Firmicutes |
| --- | --- | --- | --- | --- | --- | --- | --- | --- | --- | --- | --- |
| Clostridia sp.  (HG3A.1205) | AHI | -0.042 | 0.022 | 0.095 | 3004 unclassified | unclassified | unclassified | unclassified | unclassified | Clostridia | Firmicutes |
| Eubacteriales sp. (HG3A.0215) | T90 | -0.038 | 0.027 | 0.095 | 3364 unclassified | unclassified | unclassified | unclassified | Eubacteriales | Clostridia | Firmicutes |
| Eubacteriales sp.  (HG3A.0656) | AHI | -0.042 | 0.022 | 0.095 | 3004 unclassified | unclassified | unclassified | unclassified | Eubacteriales | Clostridia | Firmicutes |
| Lachnospiraceae sp. (HG3A.0855) | T90 | -0.038 | 0.027 | 0.095 | 3364 unclassified | unclassified | unclassified | Lachnospiraceae | Eubacteriales | Clostridia | Firmicutes |
| Longicatena caecimuris (HG3A.0571) | AHI | 0.042 | 0.022 | 0.095 | 3004 unclassified | Longicatena caecimuris | Longicatena | Erysipelotrichaceae | Erysipelotrichales | Erysipelotrichia | Firmicutes |
| Oscillospiraceae sp. (HG3A.0507) | ODI | -0.036 | 0.04 | 0.095 | 3364 unclassified | unclassified | unclassified | Oscillospiraceae | Eubacteriales | Clostridia | Firmicutes |
| Clostridia sp.  (HG3A.1039) | ODI | -0.036 | 0.041 | 0.096 | 3364 unclassified | unclassified | unclassified | unclassified | unclassified | Clostridia | Firmicutes |
| Eubacteriales sp. (HG3A.0518) | ODI | -0.036 | 0.041 | 0.096 | 3364 unclassified | unclassified | unclassified | unclassified | Eubacteriales | Clostridia | Firmicutes |
| Eubacteriales sp.  (HG3A.0626) | T90 | -0.038 | 0.028 | 0.096 | 3364 unclassified | unclassified | unclassified | unclassified | Eubacteriales | Clostridia | Firmicutes |
| Eubacteriales sp. (HG3A.0637) | AHI | -0.042 | 0.022 | 0.096 | 3004 unclassified | unclassified | unclassified | unclassified | Eubacteriales | Clostridia | Firmicutes |
| Eubacteriales sp.  (HG3A.0643) | ODI | -0.036 | 0.041 | 0.096 | 3364 unclassified | unclassified | unclassified | unclassified | Eubacteriales | Clostridia | Firmicutes |
| Eubacteriales sp. (HG3A.0829) | T90 | -0.038 | 0.028 | 0.096 | 3364 unclassified | unclassified | unclassified | unclassified | Eubacteriales | Clostridia | Firmicutes |
| Eubacterium sp. AF22- 8LB (HG3A.0838) | AHI | 0.042 | 0.022 | 0.096 | 3004 unclassified | Eubacterium sp. AF22- 8LB | Eubacterium | Eubacteriaceae | Eubacteriales | Clostridia | Firmicutes |
| Eubacteriales sp. (HG3A.0188) | ODI | -0.035 | 0.042 | 0.097 | 3364 unclassified | unclassified | unclassified | unclassified | Eubacteriales | Clostridia | Firmicutes |
| Eubacteriales sp.  (HG3A.0363) | ODI | -0.035 | 0.042 | 0.097 | 3364 unclassified | unclassified | unclassified | unclassified | Eubacteriales | Clostridia | Firmicutes |
| Eubacteriales sp. (HG3A.0643) | AHI | -0.042 | 0.023 | 0.097 | 3004 unclassified | unclassified | unclassified | unclassified | Eubacteriales | Clostridia | Firmicutes |
| Eubacteriales sp.  (HG3A.0731) | AHI | -0.042 | 0.023 | 0.097 | 3004 unclassified | unclassified | unclassified | unclassified | Eubacteriales | Clostridia | Firmicutes |
| Parolsenella catena (HG3A.0499) | ODI | 0.035 | 0.042 | 0.097 | 3364 unclassified | Parolsenella catena | Parolsenella | Atopobiaceae | Coriobacteriales | Coriobacteriia | Actinobacteria |

AHI 0.042 0.021 0.095 3004 unclassified

Anaerotruncus

colihominis Anaerotruncus Oscillospiraceae Eubacteriales Clostridia Firmicutes

Ruminococcus sp. AF46

10NS (HG3A.0271) AHI 0.042 0.023 0.097 3004 unclassified

Ruminococcus sp. AF46-

10NS Ruminococcus Oscillospiraceae Eubacteriales Clostridia Firmicutes

| Senegalimassilia  anaerobia (HG3A.0129) | T90 | 0.038 | 0.028 | 0.097 | 3364 unclassified | Senegalimassilia anaerobia | Senegalimassilia | Coriobacteriaceae | Coriobacteriales | Coriobacteriia | Actinobacteria |
| --- | --- | --- | --- | --- | --- | --- | --- | --- | --- | --- | --- |
| Clostridium sp. AF34-13 (HG3A.0173) | T90 | -0.038 | 0.029 | 0.098 | 3364 unclassified | Clostridium sp. AF34-13 | Clostridium | Clostridiaceae | Eubacteriales | Clostridia | Firmicutes |
| Coprococcus sp. AF21- 14LB (HG3A.1047) | ODI | 0.035 | 0.043 | 0.098 | 3364 unclassified | Coprococcus sp. AF21- 14LB | Coprococcus | Lachnospiraceae | Eubacteriales | Clostridia | Firmicutes |
| Coprococcus sp. OM04- 5BH (HG3A.1028) | ODI | -0.035 | 0.043 | 0.098 | 3364 unclassified | Coprococcus sp. OM04- 5BH | Coprococcus | Lachnospiraceae | Eubacteriales | Clostridia | Firmicutes |
| Eubacteriales sp. (HG3A.0821) | ODI | -0.035 | 0.043 | 0.098 | 3364 unclassified | unclassified | unclassified | unclassified | Eubacteriales | Clostridia | Firmicutes |
| Firmicutes sp.  (HG3A.1124) | ODI | -0.035 | 0.043 | 0.098 | 3364 unclassified | unclassified | unclassified | unclassified | unclassified | unclassified | Firmicutes |
| Barnesiella intestinihominis (HG3A.0055) | ODI | -0.035 | 0.044 | 0.099 | 3364 unclassified | Barnesiella intestinihominis | Barnesiella | Barnesiellaceae | Bacteroidales | Bacteroidia | Bacteroidetes |
| Eubacteriales sp.  (HG3A.0773) | T90 | -0.038 | 0.029 | 0.099 | 3364 unclassified | unclassified | unclassified | unclassified | Eubacteriales | Clostridia | Firmicutes |
| Coprococcus sp. AF21- 14LB (HG3A.1047) | T90 | 0.038 | 0.03 | 0.1 | 3364 unclassified | Coprococcus sp. AF21- 14LB | Coprococcus | Lachnospiraceae | Eubacteriales | Clostridia | Firmicutes |
| Eubacteriales sp.  (HG3A.0604) | AHI | -0.042 | 0.024 | 0.1 | 3004 unclassified | unclassified | unclassified | unclassified | Eubacteriales | Clostridia | Firmicutes |
| Gemella morbillorum (HG3A.1782) | T90 | 0.038 | 0.03 | 0.101 | 3364 unclassified | Gemella morbillorum | Gemella | unclassified | Bacillales | Bacilli | Firmicutes |
| Eubacteriales sp.  (HG3A.0363) | T90 | -0.038 | 0.03 | 0.102 | 3364 unclassified | unclassified | unclassified | unclassified | Eubacteriales | Clostridia | Firmicutes |
| Eubacteriales sp. (HG3A.0441) | ODI | -0.035 | 0.045 | 0.102 | 3364 unclassified | unclassified | unclassified | unclassified | Eubacteriales | Clostridia | Firmicutes |
| Eubacteriales sp.  (HG3A.0627) | ODI | -0.035 | 0.045 | 0.102 | 3364 unclassified | unclassified | unclassified | unclassified | Eubacteriales | Clostridia | Firmicutes |
| Eubacteriales sp. (HG3A.0978) | ODI | -0.035 | 0.045 | 0.102 | 3364 unclassified | unclassified | unclassified | unclassified | Eubacteriales | Clostridia | Firmicutes |

Faecalibacterium

prausnitzii (HG3A.0010) ODI 0.035 0.045 0.102 3364 unclassified

Faecalibacterium

prausnitzii Faecalibacterium Oscillospiraceae Eubacteriales Clostridia Firmicutes

| Oscillospiraceae sp.  (HG3A.0429) | T90 | -0.038 | 0.031 | 0.102 | 3364 unclassified | unclassified | unclassified | Oscillospiraceae | Eubacteriales | Clostridia | Firmicutes |
| --- | --- | --- | --- | --- | --- | --- | --- | --- | --- | --- | --- |
| Parolsenella catena  (HG3A.0499) | AHI | 0.041 | 0.024 | 0.102 | 3004 unclassified | Parolsenella catena | Parolsenella | Atopobiaceae | Coriobacteriales | Coriobacteriia | Actinobacteria |
| Ruminococcus champanellensis (HG3A.0716) | T90 | -0.038 | 0.031 | 0.102 | 3364 unclassified | Ruminococcus champanellensis | Ruminococcus | Oscillospiraceae | Eubacteriales | Clostridia | Firmicutes |
| Firmicutes sp.  (HG3A.0501) | T90 | -0.037 | 0.031 | 0.103 | 3364 unclassified | unclassified | unclassified | unclassified | unclassified | unclassified | Firmicutes |
| Olsenella sp. AF21-51 (HG3A.0690) | AHI | -0.041 | 0.025 | 0.103 | 3004 unclassified | Olsenella sp. AF21-51 | Olsenella | Atopobiaceae | Coriobacteriales | Coriobacteriia | Actinobacteria |
| Atopobiaceae sp.  (HG3A.0937) | T90 | 0.037 | 0.032 | 0.104 | 3364 unclassified | unclassified | unclassified | Atopobiaceae | Coriobacteriales | Coriobacteriia | Actinobacteria |
| Clostridia sp. (HG3A.0756) | T90 | -0.037 | 0.032 | 0.104 | 3364 unclassified | unclassified | unclassified | unclassified | unclassified | Clostridia | Firmicutes |
| Enterococcus faecium (HG3A.0886) | T90 | 0.037 | 0.032 | 0.104 | 3364 unclassified | Enterococcus faecium | Enterococcus | Enterococcaceae | Lactobacillales | Bacilli | Firmicutes |
| Erysipelotrichales sp. (HG3A.1207) | ODI | 0.035 | 0.047 | 0.104 | 3364 unclassified | unclassified | unclassified | unclassified | Erysipelotrichales | Erysipelotrichia | Firmicutes |
| Eubacteriales sp.  (HG3A.0102) | T90 | -0.037 | 0.032 | 0.104 | 3364 unclassified | unclassified | unclassified | unclassified | Eubacteriales | Clostridia | Firmicutes |
| Eubacteriales sp. (HG3A.0408) | T90 | -0.037 | 0.032 | 0.104 | 3364 unclassified | unclassified | unclassified | unclassified | Eubacteriales | Clostridia | Firmicutes |
| Eubacteriales sp.  (HG3A.0474) | ODI | -0.035 | 0.047 | 0.104 | 3364 unclassified | unclassified | unclassified | unclassified | Eubacteriales | Clostridia | Firmicutes |
| Eubacteriales sp. (HG3A.0613) | T90 | -0.037 | 0.033 | 0.104 | 3364 unclassified | unclassified | unclassified | unclassified | Eubacteriales | Clostridia | Firmicutes |
| Eubacteriales sp.  (HG3A.0656) | T90 | -0.037 | 0.032 | 0.104 | 3364 unclassified | unclassified | unclassified | unclassified | Eubacteriales | Clostridia | Firmicutes |
| Eubacteriales sp. (HG3A.0697) | ODI | -0.035 | 0.047 | 0.104 | 3364 unclassified | unclassified | unclassified | unclassified | Eubacteriales | Clostridia | Firmicutes |
| Eubacteriales sp.  (HG3A.0977) | AHI | -0.041 | 0.025 | 0.104 | 3004 unclassified | unclassified | unclassified | unclassified | Eubacteriales | Clostridia | Firmicutes |
| Eubacteriales sp. (HG3A.1051) | T90 | -0.037 | 0.032 | 0.104 | 3364 unclassified | unclassified | unclassified | unclassified | Eubacteriales | Clostridia | Firmicutes |
| Firmicutes sp.  (HG3A.1014) | T90 | -0.037 | 0.032 | 0.104 | 3364 unclassified | unclassified | unclassified | unclassified | unclassified | unclassified | Firmicutes |
| Oscillospiraceae sp. (HG3A.1173) | T90 | -0.037 | 0.032 | 0.104 | 3364 unclassified | unclassified | unclassified | Oscillospiraceae | Eubacteriales | Clostridia | Firmicutes |
| Blautia producta  (HG3A.0619) | T90 | 0.037 | 0.034 | 0.105 | 3364 unclassified | Blautia producta | Blautia | Lachnospiraceae | Eubacteriales | Clostridia | Firmicutes |
| Clostridia sp. (HG3A.0521) | T90 | -0.037 | 0.034 | 0.105 | 3364 unclassified | unclassified | unclassified | unclassified | unclassified | Clostridia | Firmicutes |

Clostridium sp. OF03-

18AA (HG3A.0119) T90 -0.037 0.034 0.105 3364 unclassified

Clostridium sp. OF03-

18AA Clostridium Clostridiaceae Eubacteriales Clostridia Firmicutes

| Eubacteriales sp. (HG3A.0154) | T90 | -0.037 | 0.034 | 0.105 | 3364 unclassified | unclassified | unclassified | unclassified | Eubacteriales | Clostridia Firmicutes |
| --- | --- | --- | --- | --- | --- | --- | --- | --- | --- | --- |
| Eubacteriales sp.  (HG3A.0418) | ODI | -0.035 | 0.047 | 0.105 | 3364 unclassified | unclassified | unclassified | unclassified | Eubacteriales | Clostridia Firmicutes |
| Eubacteriales sp. (HG3A.0493) | ODI | -0.035 | 0.047 | 0.105 | 3364 unclassified | unclassified | unclassified | unclassified | Eubacteriales | Clostridia Firmicutes |
| Eubacteriales sp.  (HG3A.0516) | T90 | -0.037 | 0.034 | 0.105 | 3364 unclassified | unclassified | unclassified | unclassified | Eubacteriales | Clostridia Firmicutes |
| Lachnospiraceae sp. (HG3A.0748) | ODI | -0.034 | 0.048 | 0.105 | 3364 unclassified | unclassified | unclassified | Lachnospiraceae | Eubacteriales | Clostridia Firmicutes |
| Oscillospiraceae sp.  (HG3A.0384) | T90 | -0.037 | 0.034 | 0.105 | 3364 unclassified | unclassified | unclassified | Oscillospiraceae | Eubacteriales | Clostridia Firmicutes |
| Sutterellaceae sp. (HG3A.1122) | T90 | 0.037 | 0.033 | 0.105 | 3364 unclassified | unclassified | unclassified | Sutterellaceae | Burkholderiales | Betaproteobacteri Proteobacteria a |
| Eubacteriales sp.  (HG3A.0736) | T90 | -0.037 | 0.035 | 0.106 | 3364 unclassified | unclassified | unclassified | unclassified | Eubacteriales | Clostridia Firmicutes |
| Anaerostipes sp. BG01 (HG3A.1509) | AHI | 0.041 | 0.027 | 0.107 | 3004 unclassified | Anaerostipes sp. BG01 | Anaerostipes | Lachnospiraceae | Eubacteriales | Clostridia Firmicutes |
| Blastocystis sp. subtype 4 (HG3A.0446) | AHI | -0.041 | 0.027 | 0.107 | 3004 unclassified | Blastocystis sp. subtype 4 | Blastocystis | Blastocystidae | Opalinata | Bigyra unclassified |
| Clostridiaceae sp. (HG3A.0238) | AHI | -0.041 | 0.026 | 0.107 | 3004 unclassified | unclassified | unclassified | Clostridiaceae | Eubacteriales | Clostridia Firmicutes |
| Erysipelotrichales sp.  (HG3A.1207) | AHI | 0.041 | 0.027 | 0.107 | 3004 unclassified | unclassified | unclassified | unclassified | Erysipelotrichales | Erysipelotrichia Firmicutes |
| Eubacteriales sp. (HG3A.0211) | AHI | -0.041 | 0.026 | 0.107 | 3004 unclassified | unclassified | unclassified | unclassified | Eubacteriales | Clostridia Firmicutes |
| Eubacteriales sp.  (HG3A.0215) | AHI | -0.041 | 0.026 | 0.107 | 3004 unclassified | unclassified | unclassified | unclassified | Eubacteriales | Clostridia Firmicutes |
| Eubacteriales sp. (HG3A.0312) | AHI | -0.041 | 0.027 | 0.107 | 3004 unclassified | unclassified | unclassified | unclassified | Eubacteriales | Clostridia Firmicutes |
| Eubacteriales sp.  (HG3A.0697) | T90 | -0.037 | 0.035 | 0.107 | 3364 unclassified | unclassified | unclassified | unclassified | Eubacteriales | Clostridia Firmicutes |
| Eubacteriales sp. (HG3A.0711) | T90 | -0.037 | 0.035 | 0.107 | 3364 unclassified | unclassified | unclassified | unclassified | Eubacteriales | Clostridia Firmicutes |
| Firmicutes sp.  (HG3A.0650) | AHI | -0.041 | 0.027 | 0.107 | 3004 unclassified | unclassified | unclassified | unclassified | unclassified | unclassified Firmicutes |
| Firmicutes sp. (HG3A.0587) | T90 | -0.037 | 0.035 | 0.108 | 3364 unclassified | unclassified | unclassified | unclassified | unclassified | unclassified Firmicutes |

Eubacteriales sp. (HG3A.0334)

ODI -0.034 0.049 0.109 3364 unclassified unclassified unclassified unclassified Eubacteriales Clostridia Firmicutes

| Eubacteriales sp.  (HG3A.1026) | AHI | -0.041 | 0.028 | 0.109 | 3004 unclassified | unclassified | unclassified | unclassified | Eubacteriales | Clostridia | Firmicutes |
| --- | --- | --- | --- | --- | --- | --- | --- | --- | --- | --- | --- |
| Clostridia sp.  (HG3A.1076) | ODI | -0.034 | 0.05 | 0.11 | 3364 unclassified | unclassified | unclassified | unclassified | unclassified | Clostridia | Firmicutes |
| [Clostridium] symbiosum (HG3A.0370) | T90 | 0.036 | 0.036 | 0.11 | 3364 unclassified | [Clostridium] symbiosum | Lachnoclostridiu m | Lachnospiraceae | Eubacteriales | Clostridia | Firmicutes |
| Eubacteriales sp.  (HG3A.0163) | ODI | -0.034 | 0.05 | 0.11 | 3364 unclassified | unclassified | unclassified | unclassified | Eubacteriales | Clostridia | Firmicutes |
| Eubacteriales sp. (HG3A.0338) | ODI | -0.034 | 0.051 | 0.11 | 3364 unclassified | unclassified | unclassified | unclassified | Eubacteriales | Clostridia | Firmicutes |
| Eubacteriales sp.  (HG3A.0364) | ODI | -0.034 | 0.05 | 0.11 | 3364 unclassified | unclassified | unclassified | unclassified | Eubacteriales | Clostridia | Firmicutes |
| Eubacteriales sp. (HG3A.0540) | ODI | -0.034 | 0.05 | 0.11 | 3364 unclassified | unclassified | unclassified | unclassified | Eubacteriales | Clostridia | Firmicutes |
| Eubacteriales sp.  (HG3A.0589) | ODI | -0.034 | 0.05 | 0.11 | 3364 unclassified | unclassified | unclassified | unclassified | Eubacteriales | Clostridia | Firmicutes |
| Firmicutes sp. (HG3A.1085) | T90 | -0.036 | 0.036 | 0.11 | 3364 unclassified | unclassified | unclassified | unclassified | unclassified | unclassified | Firmicutes |
| Eubacteriales sp.  (HG3A.0092) | AHI | -0.04 | 0.029 | 0.112 | 3004 unclassified | unclassified | unclassified | unclassified | Eubacteriales | Clostridia | Firmicutes |
| Eubacteriales sp. (HG3A.0578) | AHI | -0.04 | 0.029 | 0.112 | 3004 unclassified | unclassified | unclassified | unclassified | Eubacteriales | Clostridia | Firmicutes |
| Clostridia sp.  (HG3A.0767) | T90 | -0.036 | 0.038 | 0.113 | 3364 unclassified | unclassified | unclassified | unclassified | unclassified | Clostridia | Firmicutes |
| Eubacteriales sp. (HG3A.0545) | ODI | -0.034 | 0.052 | 0.113 | 3364 unclassified | unclassified | unclassified | unclassified | Eubacteriales | Clostridia | Firmicutes |
| Eubacteriales sp.  (HG3A.0950) | ODI | -0.034 | 0.053 | 0.113 | 3364 unclassified | unclassified | unclassified | unclassified | Eubacteriales | Clostridia | Firmicutes |
| Clostridia sp. (HG3A.0841) | ODI | -0.034 | 0.053 | 0.114 | 3364 unclassified | unclassified | unclassified | unclassified | unclassified | Clostridia | Firmicutes |
| Clostridia sp.  (HG3A.1205) | ODI | -0.034 | 0.053 | 0.114 | 3364 unclassified | unclassified | unclassified | unclassified | unclassified | Clostridia | Firmicutes |
| Eubacteriales sp. (HG3A.0264) | T90 | -0.036 | 0.038 | 0.114 | 3364 unclassified | unclassified | unclassified | unclassified | Eubacteriales | Clostridia | Firmicutes |
| Eubacteriales sp.  (HG3A.0694) | ODI | -0.034 | 0.053 | 0.114 | 3364 unclassified | unclassified | unclassified | unclassified | Eubacteriales | Clostridia | Firmicutes |
| Eubacteriales sp. (HG3A.0731) | T90 | -0.036 | 0.038 | 0.114 | 3364 unclassified | unclassified | unclassified | unclassified | Eubacteriales | Clostridia | Firmicutes |
| Eubacteriales sp.  (HG3A.0910) | T90 | -0.036 | 0.038 | 0.114 | 3364 unclassified | unclassified | unclassified | unclassified | Eubacteriales | Clostridia | Firmicutes |
| Eubacterium sp. AF16- 48 (HG3A.0219) | AHI | -0.04 | 0.029 | 0.114 | 3004 unclassified | Eubacterium sp. AF16-48 | Eubacterium | Eubacteriaceae | Eubacteriales | Clostridia | Firmicutes |

Clostridia sp. (HG3A.0746)

AHI -0.04 0.03 0.115 3004 unclassified unclassified unclassified unclassified unclassified Clostridia Firmicutes

| Eubacteriales sp. (HG3A.0654) | AHI | -0.04 | 0.03 | 0.115 | 3004 unclassified | unclassified | unclassified | unclassified | Eubacteriales | Clostridia | Firmicutes |
| --- | --- | --- | --- | --- | --- | --- | --- | --- | --- | --- | --- |
| Eubacteriales sp.  (HG3A.0691) | AHI | -0.04 | 0.03 | 0.115 | 3004 unclassified | unclassified | unclassified | unclassified | Eubacteriales | Clostridia | Firmicutes |
| Eubacteriales sp. (HG3A.0857) | ODI | -0.034 | 0.054 | 0.115 | 3364 unclassified | unclassified | unclassified | unclassified | Eubacteriales | Clostridia | Firmicutes |
| Oscillospiraceae sp.  (HG3A.1173) | AHI | -0.04 | 0.03 | 0.115 | 3004 unclassified | unclassified | unclassified | Oscillospiraceae | Eubacteriales | Clostridia | Firmicutes |
| Clostridia sp. (HG3A.1008) | ODI | -0.033 | 0.054 | 0.116 | 3364 unclassified | unclassified | unclassified | unclassified | unclassified | Clostridia | Firmicutes |
| Eubacteriales sp.  (HG3A.0557) | ODI | -0.033 | 0.055 | 0.116 | 3364 unclassified | unclassified | unclassified | unclassified | Eubacteriales | Clostridia | Firmicutes |
| Eubacteriales sp. (HG3A.0614) | T90 | -0.036 | 0.039 | 0.116 | 3364 unclassified | unclassified | unclassified | unclassified | Eubacteriales | Clostridia | Firmicutes |
| Eubacteriales sp.  (HG3A.0618) | AHI | -0.04 | 0.031 | 0.116 | 3004 unclassified | unclassified | unclassified | unclassified | Eubacteriales | Clostridia | Firmicutes |
| Anaeroglobus geminatus (HG3A.1818) | ODI | 0.033 | 0.055 | 0.117 | 3364 unclassified | Anaeroglobus geminatus | Anaeroglobus | Veillonellaceae | Veillonellales | Negativicutes | Firmicutes |
| Eubacteriales sp.  (HG3A.0312) | ODI | -0.033 | 0.055 | 0.117 | 3364 unclassified | unclassified | unclassified | unclassified | Eubacteriales | Clostridia | Firmicutes |
| Barnesiellaceae sp. (HG3A.1180) | ODI | -0.033 | 0.056 | 0.118 | 3364 unclassified | unclassified | unclassified | Barnesiellaceae | Bacteroidales | Bacteroidia | Bacteroidetes |
| Clostridia sp.  (HG3A.0752) | T90 | -0.036 | 0.04 | 0.119 | 3364 unclassified | unclassified | unclassified | unclassified | unclassified | Clostridia | Firmicutes |
| Dorea longicatena (HG3A.0039) | T90 | 0.036 | 0.041 | 0.119 | 3364 unclassified | Dorea longicatena | Dorea | Lachnospiraceae | Eubacteriales | Clostridia | Firmicutes |
| Eubacteriales sp.  (HG3A.0760) | ODI | -0.033 | 0.057 | 0.119 | 3364 unclassified | unclassified | unclassified | unclassified | Eubacteriales | Clostridia | Firmicutes |
| Eubacteriales sp. (HG3A.1332) | T90 | -0.036 | 0.041 | 0.119 | 3364 unclassified | unclassified | unclassified | unclassified | Eubacteriales | Clostridia | Firmicutes |
| Eubacteriales sp.  (HG3A.1103) | AHI | -0.04 | 0.032 | 0.12 | 3004 unclassified | unclassified | unclassified | unclassified | Eubacteriales | Clostridia | Firmicutes |
| Firmicutes sp. (HG3A.0436) | AHI | -0.04 | 0.032 | 0.12 | 3004 unclassified | unclassified | unclassified | unclassified | unclassified | unclassified | Firmicutes |
| Firmicutes sp.  (HG3A.0874) | T90 | -0.036 | 0.041 | 0.12 | 3364 unclassified | unclassified | unclassified | unclassified | unclassified | unclassified | Firmicutes |
| Eubacteriales sp. (HG3A.0308) | ODI | -0.033 | 0.058 | 0.121 | 3364 unclassified | unclassified | unclassified | unclassified | Eubacteriales | Clostridia | Firmicutes |

Eubacteriales sp.

# (HG3A.0698)

AHI 0.039 0.032 0.121 3004 unclassified unclassified unclassified unclassified Eubacteriales Clostridia Firmicutes

| Veillonella rogosae  (HG3A.0324) | ODI | -0.033 | 0.058 | 0.121 | 3364 unclassified | Veillonella rogosae | Veillonella | Veillonellaceae | Veillonellales | Negativicutes | Firmicutes |
| --- | --- | --- | --- | --- | --- | --- | --- | --- | --- | --- | --- |
| Eubacteriales sp.  (HG3A.0577) | ODI | -0.033 | 0.059 | 0.122 | 3364 unclassified | unclassified | unclassified | unclassified | Eubacteriales | Clostridia | Firmicutes |
| Eubacteriales sp. (HG3A.0856) | AHI | -0.039 | 0.033 | 0.122 | 3004 unclassified | unclassified | unclassified | unclassified | Eubacteriales | Clostridia | Firmicutes |
| Clostridia sp.  (HG3A.0946) | ODI | -0.033 | 0.059 | 0.123 | 3364 unclassified | unclassified | unclassified | unclassified | unclassified | Clostridia | Firmicutes |
| Clostridia sp. (HG3A.1108) | ODI | -0.033 | 0.059 | 0.123 | 3364 unclassified | unclassified | unclassified | unclassified | unclassified | Clostridia | Firmicutes |
| Eggerthellaceae sp.  (HG3A.0171) | AHI | -0.039 | 0.033 | 0.123 | 3004 unclassified | unclassified | unclassified | Eggerthellaceae | Eggerthellales | Coriobacteriia | Actinobacteria |
| Eubacteriales sp. (HG3A.0342) | AHI | -0.039 | 0.033 | 0.123 | 3004 unclassified | unclassified | unclassified | unclassified | Eubacteriales | Clostridia | Firmicutes |
| Firmicutes sp.  (HG3A.1345) | ODI | -0.033 | 0.059 | 0.123 | 3364 unclassified | unclassified | unclassified | unclassified | unclassified | unclassified | Firmicutes |
| Clostridium sp. (HG3A.0050) | AHI | 0.039 | 0.034 | 0.124 | 3004 unclassified | unclassified | Clostridium | Clostridiaceae | Eubacteriales | Clostridia | Firmicutes |
| Eubacteriales sp.  (HG3A.0757) | AHI | -0.039 | 0.034 | 0.124 | 3004 unclassified | unclassified | unclassified | unclassified | Eubacteriales | Clostridia | Firmicutes |
| Clostridia sp. (HG3A.0733) | AHI | -0.039 | 0.035 | 0.125 | 3004 unclassified | unclassified | unclassified | unclassified | unclassified | Clostridia | Firmicutes |
| Clostridia sp.  (HG3A.0752) | AHI | -0.039 | 0.035 | 0.125 | 3004 unclassified | unclassified | unclassified | unclassified | unclassified | Clostridia | Firmicutes |
| Clostridia sp. (HG3A.1076) | AHI | -0.039 | 0.035 | 0.125 | 3004 unclassified | unclassified | unclassified | unclassified | unclassified | Clostridia | Firmicutes |
| Clostridia sp.  (HG3A.1141) | AHI | -0.039 | 0.035 | 0.125 | 3004 unclassified | unclassified | unclassified | unclassified | unclassified | Clostridia | Firmicutes |
| Eubacteriales sp. (HG3A.0870) | AHI | -0.039 | 0.035 | 0.125 | 3004 unclassified | unclassified | unclassified | unclassified | Eubacteriales | Clostridia | Firmicutes |
| Eubacteriales sp.  (HG3A.1086) | AHI | -0.039 | 0.035 | 0.125 | 3004 unclassified | unclassified | unclassified | unclassified | Eubacteriales | Clostridia | Firmicutes |
| Subdoligranulum sp. APC924/74 (HG3A.0015) | ODI | -0.033 | 0.06 | 0.125 | 3364 unclassified | Subdoligranulum sp. APC924/74 | Subdoligranulum | Oscillospiraceae | Eubacteriales | Clostridia | Firmicutes |
| Blastocystis sp. subtype 4 (HG3A.0446) | T90 | -0.035 | 0.044 | 0.126 | 3364 unclassified | Blastocystis sp. subtype 4 | Blastocystis | Blastocystidae | Opalinata | Bigyra | unclassified |
| Eubacteriales sp. (HG3A.0482) | T90 | -0.035 | 0.043 | 0.126 | 3364 unclassified | unclassified | unclassified | unclassified | Eubacteriales | Clostridia | Firmicutes |
| Eubacteriales sp.  (HG3A.0482) | ODI | -0.033 | 0.061 | 0.126 | 3364 unclassified | unclassified | unclassified | unclassified | Eubacteriales | Clostridia | Firmicutes |
| Eubacteriales sp. (HG3A.0604) | ODI | -0.033 | 0.061 | 0.126 | 3364 unclassified | unclassified | unclassified | unclassified | Eubacteriales | Clostridia | Firmicutes |

Firmicutes sp. (HG3A.0681)

T90 -0.035 0.044 0.126 3364 unclassified unclassified unclassified unclassified unclassified unclassified Firmicutes

| Limosilactobacillus Limosilactobacillus Limosilactobacill  fermentum AHI 0.039 0.036 0.126 3004 unclassified fermentum us Lactobacillaceae Lactobacillales Bacilli Firmicutes  (HG3A.0990) | | | | | | | | | | | |
| --- | --- | --- | --- | --- | --- | --- | --- | --- | --- | --- | --- |
| Clostridia sp.  (HG3A.0741) | T90 | -0.035 | 0.044 | 0.127 | 3364 unclassified | unclassified | unclassified | unclassified | unclassified | Clostridia | Firmicutes |
| Eubacteriales sp. (HG3A.0189) | AHI | -0.039 | 0.036 | 0.128 | 3004 unclassified | unclassified | unclassified | unclassified | Eubacteriales | Clostridia | Firmicutes |
| Subdoligranulum sp.  APC924/74 (HG3A.0015) | AHI | -0.039 | 0.037 | 0.128 | 3004 unclassified | Subdoligranulum sp. APC924/74 | Subdoligranulum | Oscillospiraceae | Eubacteriales | Clostridia | Firmicutes |
| Eubacteriales sp. (HG3A.0144) | T90 | -0.035 | 0.045 | 0.129 | 3364 unclassified | unclassified | unclassified | unclassified | Eubacteriales | Clostridia | Firmicutes |
| Eubacteriales sp.  (HG3A.0144) | ODI | -0.032 | 0.063 | 0.129 | 3364 unclassified | unclassified | unclassified | unclassified | Eubacteriales | Clostridia | Firmicutes |
| Eubacteriales sp. (HG3A.0758) | AHI | -0.038 | 0.037 | 0.129 | 3004 unclassified | unclassified | unclassified | unclassified | Eubacteriales | Clostridia | Firmicutes |
| Eubacteriales sp.  (HG3A.1087) | ODI | -0.032 | 0.063 | 0.129 | 3364 unclassified | unclassified | unclassified | unclassified | Eubacteriales | Clostridia | Firmicutes |
| Firmicutes sp. (HG3A.0915) | T90 | -0.035 | 0.045 | 0.129 | 3364 unclassified | unclassified | unclassified | unclassified | unclassified | unclassified | Firmicutes |
| Oscillospiraceae sp.  (HG3A.0223) | T90 | -0.035 | 0.045 | 0.129 | 3364 unclassified | unclassified | unclassified | Oscillospiraceae | Eubacteriales | Clostridia | Firmicutes |
| Clostridium sp. AF15-31 (HG3A.0293) | T90 | -0.035 | 0.046 | 0.13 | 3364 unclassified | Clostridium sp. AF15-31 | Clostridium | Clostridiaceae | Eubacteriales | Clostridia | Firmicutes |
| Eubacteriales sp.  (HG3A.1445) | ODI | -0.032 | 0.064 | 0.13 | 3364 unclassified | unclassified | unclassified | unclassified | Eubacteriales | Clostridia | Firmicutes |
| Lachnospiraceae sp. (HG3A.0748) | AHI | -0.038 | 0.037 | 0.13 | 3004 unclassified | unclassified | unclassified | Lachnospiraceae | Eubacteriales | Clostridia | Firmicutes |
| Candidatus  Borkfalkiales sp. (HG3A.1329) | ODI | -0.032 | 0.065 | 0.131 | 3364 unclassified | unclassified | unclassified | unclassified | Candidatus Borkfalkiales | Clostridia | Firmicutes |
| Eggerthella lenta (HG3A.0225) | T90 | 0.035 | 0.047 | 0.131 | 3364 unclassified | Eggerthella lenta | Eggerthella | Eggerthellaceae | Eggerthellales | Coriobacteriia | Actinobacteria |
| Gemmiger formicilis  (HG3A.0027) | ODI | 0.032 | 0.065 | 0.131 | 3364 unclassified | Gemmiger formicilis | Gemmiger | unclassified | Eubacteriales | Clostridia | Firmicutes |
| Ruminococcus sp. (HG3A.0337) | ODI | -0.032 | 0.065 | 0.131 | 3364 unclassified | unclassified | Ruminococcus | Oscillospiraceae | Eubacteriales | Clostridia | Firmicutes |

Veillonella rogosae

# (HG3A.0324)

T90 -0.035 0.046 0.131 3364 unclassified Veillonella rogosae Veillonella Veillonellaceae Veillonellales Negativicutes Firmicutes

Clostridium sp. TM06-

18 (HG3A.0048)

T90

0.035

0.047

0.132 3364 unclassified Clostridium sp. TM06-18 Clostridium

Clostridiaceae

Eubacteriales

Clostridia

Firmicutes

Eubacteriales sp.

# (HG3A.0736)

ODI -0.032 0.065 0.132 3364 unclassified unclassified unclassified unclassified Eubacteriales Clostridia Firmicutes

Eubacteriales sp.

(HG3A.1321)

AHI

-0.038

0.038

0.132 3004 unclassified unclassified

unclassified

unclassified

Eubacteriales

Clostridia

Firmicutes

Butyricimonas virosa

# (HG3A.0199)

T90 -0.034 0.048 0.133 3364 unclassified Butyricimonas virosa Butyricimonas Odoribacteraceae Bacteroidales Bacteroidia Bacteroidetes

Clostridiaceae sp.

(HG3A.0238)

ODI

-0.032

0.066

0.133 3364 unclassified unclassified

unclassified

Clostridiaceae

Eubacteriales

Clostridia

Firmicutes

Eubacteriales sp.

# (HG3A.0447)

ODI -0.032 0.067 0.133 3364 unclassified unclassified unclassified unclassified Eubacteriales Clostridia Firmicutes

Succinatimonas hippei

(HG3A.1322)

ODI

0.032

0.066

0.133 3364 unclassified Succinatimonas hippei

Succinatimonas Succinivibrionaceae Aeromonadales Gammaproteoba Proteobacteria

cteria

Clostridia sp.

# (HG3A.1062)

ODI -0.032 0.068 0.136 3364 unclassified unclassified unclassified unclassified unclassified Clostridia Firmicutes

Firmicutes sp.

(HG3A.0581)

ODI

-0.032

0.069

0.136 3364 unclassified unclassified

unclassified

unclassified

unclassified

unclassified

Firmicutes

Firmicutes sp.

# (HG3A.0860)

T90 -0.034 0.049 0.136 3364 unclassified unclassified unclassified unclassified unclassified unclassified Firmicutes

Firmicutes sp.

(HG3A.1195)

ODI

-0.032

0.068

0.136 3364 unclassified unclassified

unclassified

unclassified

unclassified

unclassified

Firmicutes

Eubacteriales sp.

# (HG3A.0267)

ODI -0.032 0.07 0.137 3364 unclassified unclassified unclassified unclassified Eubacteriales Clostridia Firmicutes

Eubacteriales sp.

(HG3A.0325)

ODI

-0.032

0.07

0.137 3364 unclassified unclassified

unclassified

unclassified

Eubacteriales

Clostridia

Firmicutes

Eubacteriales sp.

# (HG3A.0573)

AHI -0.038 0.04 0.137 3004 unclassified unclassified unclassified unclassified Eubacteriales Clostridia Firmicutes

Eubacteriales sp.

(HG3A.0790)

ODI

-0.032

0.069

0.137 3364 unclassified unclassified

unclassified

unclassified

Eubacteriales

Clostridia

Firmicutes

Eubacteriales sp.

# (HG3A.1227)

ODI -0.032 0.07 0.137 3364 unclassified unclassified unclassified unclassified Eubacteriales Clostridia Firmicutes

Firmicutes sp.

(HG3A.0948)

T90

-0.034

0.049

0.137 3364 unclassified unclassified

unclassified

unclassified

unclassified

unclassified

Firmicutes

Oscillibacter sp.

# (HG3A.0046)

AHI -0.038 0.04 0.137 3004 unclassified unclassified Oscillibacter Oscillospiraceae Eubacteriales Clostridia Firmicutes

Prevotella sp.

(HG3A.1009)

ODI

0.032

0.069

0.137 3364 unclassified unclassified

Prevotella

Prevotellaceae

Bacteroidales

Bacteroidia

Bacteroidetes

Barnesiellaceae sp.

# (HG3A.1180)

AHI -0.038 0.041 0.138 3004 unclassified unclassified unclassified Barnesiellaceae Bacteroidales Bacteroidia Bacteroidetes

Butyricicoccus sp. OM04-18BH

(HG3A.0139)

T90

-0.034

0.05

0.138 3364 unclassified

Butyricicoccus sp. OM04-

18BH

Butyricicoccus Clostridiaceae

Eubacteriales

Clostridia

Firmicutes

Clostridia sp. (HG3A.1010)

| Eubacteriales sp. (HG3A.0577) | AHI | -0.038 | 0.041 | 0.138 | 3004 unclassified | unclassified | unclassified | unclassified | Eubacteriales | Clostridia | Firmicutes |
| --- | --- | --- | --- | --- | --- | --- | --- | --- | --- | --- | --- |
| Anaerobutyricum hallii  (HG3A.0012) | ODI | 0.031 | 0.072 | 0.139 | 3364 unclassified | Anaerobutyricum hallii | Anaerobutyricum | Lachnospiraceae | Eubacteriales | Clostridia | Firmicutes |
| Clostridia sp. (HG3A.0706) | ODI | -0.031 | 0.071 | 0.139 | 3364 unclassified | unclassified | unclassified | unclassified | unclassified | Clostridia | Firmicutes |
| Clostridia sp.  (HG3A.1010) | AHI | -0.037 | 0.042 | 0.139 | 3004 unclassified | unclassified | unclassified | unclassified | unclassified | Clostridia | Firmicutes |
| Clostridia sp. (HG3A.1139) | ODI | -0.031 | 0.071 | 0.139 | 3364 unclassified | unclassified | unclassified | unclassified | unclassified | Clostridia | Firmicutes |
| Eggerthella lenta  (HG3A.0225) | AHI | 0.037 | 0.042 | 0.139 | 3004 unclassified | Eggerthella lenta | Eggerthella | Eggerthellaceae | Eggerthellales | Coriobacteriia | Actinobacteria |
| Eubacteriales sp. (HG3A.0419) | AHI | -0.037 | 0.043 | 0.139 | 3004 unclassified | unclassified | unclassified | unclassified | Eubacteriales | Clostridia | Firmicutes |
| Eubacteriales sp.  (HG3A.0609) | AHI | -0.037 | 0.042 | 0.139 | 3004 unclassified | unclassified | unclassified | unclassified | Eubacteriales | Clostridia | Firmicutes |
| Eubacteriales sp. (HG3A.0630) | AHI | -0.037 | 0.042 | 0.139 | 3004 unclassified | unclassified | unclassified | unclassified | Eubacteriales | Clostridia | Firmicutes |
| Eubacteriales sp.  (HG3A.0656) | ODI | -0.031 | 0.072 | 0.139 | 3364 unclassified | unclassified | unclassified | unclassified | Eubacteriales | Clostridia | Firmicutes |
| Eubacteriales sp. (HG3A.0696) | AHI | -0.037 | 0.043 | 0.139 | 3004 unclassified | unclassified | unclassified | unclassified | Eubacteriales | Clostridia | Firmicutes |
| Eubacteriales sp.  (HG3A.0967) | ODI | -0.031 | 0.072 | 0.139 | 3364 unclassified | unclassified | unclassified | unclassified | Eubacteriales | Clostridia | Firmicutes |
| Eubacteriales sp. (HG3A.0985) | AHI | 0.037 | 0.042 | 0.139 | 3004 unclassified | unclassified | unclassified | unclassified | Eubacteriales | Clostridia | Firmicutes |
| Mogibacterium kristiansenii  (HG3A.0522) | AHI | 0.037 | 0.042 | 0.139 | 3004 unclassified | Mogibacterium kristiansenii | Mogibacterium | Clostridiales Family  XIII. Incertae Sedis | Eubacteriales | Clostridia | Firmicutes |
| Oscillibacter sp. PEA192 (HG3A.0021) | AHI | 0.037 | 0.042 | 0.139 | 3004 unclassified | Oscillibacter sp. PEA192 | Oscillibacter | Oscillospiraceae | Eubacteriales | Clostridia | Firmicutes |
| Phascolarctobacterium succinatutens  (HG3A.0315) | ODI | 0.031 | 0.071 | 0.139 | 3364 unclassified | Phascolarctobacterium succinatutens | Phascolarctobact erium | Acidaminococcaceae | Acidaminococcales | Negativicutes | Firmicutes |
| Victivallis vadensis (HG3A.0689) | AHI | -0.037 | 0.043 | 0.139 | 3004 unclassified | Victivallis vadensis | Victivallis | Victivallaceae | Victivallales | Lentisphaeria | Lentisphaerae |
| Clostridia sp.  (HG3A.1057) | ODI | -0.031 | 0.073 | 0.14 | 3364 unclassified | unclassified | unclassified | unclassified | unclassified | Clostridia | Firmicutes |
| Eubacteriales sp. (HG3A.0320) | T90 | -0.034 | 0.051 | 0.14 | 3364 unclassified | unclassified | unclassified | unclassified | Eubacteriales | Clostridia | Firmicutes |

T90 -0.034 0.05 0.138 3364 unclassified unclassified unclassified unclassified unclassified Clostridia Firmicutes

Coprococcus sp. (HG3A.0404)

AHI 0.037 0.044 0.142 3004 unclassified unclassified Coprococcus Lachnospiraceae Eubacteriales Clostridia Firmicutes

| Eubacteriales sp. (HG3A.0305) | ODI | -0.031 | 0.074 | 0.142 | 3364 unclassified | unclassified | unclassified | unclassified | Eubacteriales | Clostridia | Firmicutes |
| --- | --- | --- | --- | --- | --- | --- | --- | --- | --- | --- | --- |
| Eubacteriales sp.  (HG3A.0453) | T90 | -0.034 | 0.053 | 0.143 | 3364 unclassified | unclassified | unclassified | unclassified | Eubacteriales | Clostridia | Firmicutes |
| Eubacteriales sp. (HG3A.0846) | T90 | -0.034 | 0.052 | 0.143 | 3364 unclassified | unclassified | unclassified | unclassified | Eubacteriales | Clostridia | Firmicutes |
| Olsenella sp. AF21-51  (HG3A.0690) | T90 | -0.034 | 0.052 | 0.143 | 3364 unclassified | Olsenella sp. AF21-51 | Olsenella | Atopobiaceae | Coriobacteriales | Coriobacteriia | Actinobacteria |
| Clostridia sp. (HG3A.1298) | ODI | -0.031 | 0.075 | 0.144 | 3364 unclassified | unclassified | unclassified | unclassified | unclassified | Clostridia | Firmicutes |
| Sellimonas intestinalis (HG3A.0417) | AHI | 0.037 | 0.045 | 0.144 | 3004 unclassified | Sellimonas intestinalis | Sellimonas | Lachnospiraceae | Eubacteriales | Clostridia | Firmicutes |
| Eubacteriales sp. (HG3A.0123) | AHI | 0.037 | 0.046 | 0.145 | 3004 unclassified | unclassified | unclassified | unclassified | Eubacteriales | Clostridia | Firmicutes |
| Eubacteriales sp.  (HG3A.0668) | AHI | -0.037 | 0.046 | 0.145 | 3004 unclassified | unclassified | unclassified | unclassified | Eubacteriales | Clostridia | Firmicutes |
| Fusicatenibacter saccharivorans (HG3A.0004) | T90 | 0.034 | 0.053 | 0.145 | 3364 unclassified | Fusicatenibacter saccharivorans | Fusicatenibacter | Lachnospiraceae | Eubacteriales | Clostridia | Firmicutes |
| Odoribacter  splanchnicus (HG3A.0041) | AHI | -0.037 | 0.045 | 0.145 | 3004 unclassified | Odoribacter splanchnicus | Odoribacter | Odoribacteraceae | Bacteroidales | Bacteroidia | Bacteroidetes |
| Oscillospiraceae sp. (HG3A.0693) | T90 | -0.034 | 0.054 | 0.145 | 3364 unclassified | unclassified | unclassified | Oscillospiraceae | Eubacteriales | Clostridia | Firmicutes |
| Oxalobacter formigenes (HG3A.0552) | AHI | -0.037 | 0.045 | 0.145 | 3004 unclassified | Oxalobacter formigenes | Oxalobacter | Oxalobacteraceae | Burkholderiales | Betaproteobacteri a | Proteobacteria |
| Clostridia sp. (HG3A.0661) | AHI | -0.037 | 0.047 | 0.146 | 3004 unclassified | unclassified | unclassified | unclassified | unclassified | Clostridia | Firmicutes |
| Clostridia sp.  (HG3A.0852) | AHI | -0.037 | 0.046 | 0.146 | 3004 unclassified | unclassified | unclassified | unclassified | unclassified | Clostridia | Firmicutes |
| Clostridia sp. (HG3A.0931) | AHI | -0.037 | 0.046 | 0.146 | 3004 unclassified | unclassified | unclassified | unclassified | unclassified | Clostridia | Firmicutes |
| Eubacteriales sp.  (HG3A.0985) | ODI | 0.031 | 0.076 | 0.146 | 3364 unclassified | unclassified | unclassified | unclassified | Eubacteriales | Clostridia | Firmicutes |
| Rikenellaceae sp. (HG3A.1022) | AHI | -0.037 | 0.047 | 0.146 | 3004 unclassified | unclassified | unclassified | Rikenellaceae | Bacteroidales | Bacteroidia | Bacteroidetes |

Bacteroides caccae

# (HG3A.0066)

ODI -0.031 0.078 0.147 3364 unclassified Bacteroides caccae Bacteroides Bacteroidaceae Bacteroidales Bacteroidia Bacteroidetes

r

| Blautia  hydrogenotrophica | AHI | 0.037 | 0.047 | 0.147 | 3004 unclassified | Blautia hydrogenotrophica | Blautia | Lachnospiraceae | Eubacteriales | Clostridia | Firmicutes |
| --- | --- | --- | --- | --- | --- | --- | --- | --- | --- | --- | --- |
| (HG3A.0430) |  |  |  |  |  |  |  |  |  |  |  |
| Clostridia sp.  (HG3A.0521) | ODI | -0.031 | 0.077 | 0.147 | 3364 unclassified | unclassified | unclassified | unclassified | unclassified | Clostridia | Firmicutes |
| Eubacteriales sp. (HG3A.0618) | ODI | -0.031 | 0.077 | 0.147 | 3364 unclassified | unclassified | unclassified | unclassified | Eubacteriales | Clostridia | Firmicutes |
| Eubacteriales sp.  (HG3A.1239) | AHI | -0.037 | 0.047 | 0.147 | 3004 unclassified | unclassified | unclassified | unclassified | Eubacteriales | Clostridia | Firmicutes |
| Sutterellaceae sp. (HG3A.1122) | ODI | 0.031 | 0.077 | 0.147 | 3364 unclassified | unclassified | unclassified | Sutterellaceae | Burkholderiales | Betaproteobacte a | i Proteobacteria |
| Clostridium sp. TM06- 18 (HG3A.0048) | AHI | 0.036 | 0.048 | 0.148 | 3004 unclassified | Clostridium sp. TM06-18 | Clostridium | Clostridiaceae | Eubacteriales | Clostridia | Firmicutes |
| Eubacteriales sp. (HG3A.0113) | T90 | -0.033 | 0.055 | 0.148 | 3364 unclassified | unclassified | unclassified | unclassified | Eubacteriales | Clostridia | Firmicutes |
| Eubacteriales sp.  (HG3A.0291) | AHI | -0.036 | 0.048 | 0.148 | 3004 unclassified | unclassified | unclassified | unclassified | Eubacteriales | Clostridia | Firmicutes |
| Eubacteriales sp. (HG3A.0383) | AHI | -0.036 | 0.048 | 0.148 | 3004 unclassified | unclassified | unclassified | unclassified | Eubacteriales | Clostridia | Firmicutes |
| Eubacteriales sp.  (HG3A.0593) | ODI | -0.031 | 0.078 | 0.148 | 3364 unclassified | unclassified | unclassified | unclassified | Eubacteriales | Clostridia | Firmicutes |
| Eubacteriales sp. (HG3A.0664) | T90 | 0.033 | 0.055 | 0.148 | 3364 unclassified | unclassified | unclassified | unclassified | Eubacteriales | Clostridia | Firmicutes |
| Eubacteriales sp.  (HG3A.0744) | T90 | -0.033 | 0.055 | 0.148 | 3364 unclassified | unclassified | unclassified | unclassified | Eubacteriales | Clostridia | Firmicutes |
| Eubacteriales sp. (HG3A.1187) | T90 | -0.033 | 0.056 | 0.148 | 3364 unclassified | unclassified | unclassified | unclassified | Eubacteriales | Clostridia | Firmicutes |
| Eubacteriales sp.  (HG3A.1219) | T90 | -0.033 | 0.056 | 0.148 | 3364 unclassified | unclassified | unclassified | unclassified | Eubacteriales | Clostridia | Firmicutes |
| Lactobacillus acidophilus (HG3A.0672) | ODI | -0.031 | 0.079 | 0.148 | 3364 unclassified | Lactobacillus acidophilus | Lactobacillus | Lactobacillaceae | Lactobacillales | Bacilli | Firmicutes |
| Parolsenella catena  (HG3A.0499) | T90 | 0.033 | 0.056 | 0.148 | 3364 unclassified | Parolsenella catena | Parolsenella | Atopobiaceae | Coriobacteriales | Coriobacteriia | Actinobacteria |
| Pseudoflavonifractor sp. An184 (HG3A.0253) | ODI | -0.031 | 0.079 | 0.148 | 3364 unclassified | Pseudoflavonifractor sp. An184 | Pseudoflavonifrac tor | Oscillospiraceae | Eubacteriales | Clostridia | Firmicutes |
| Eubacteriales sp.  (HG3A.0080) | ODI | 0.03 | 0.08 | 0.149 | 3364 unclassified | unclassified | unclassified | unclassified | Eubacteriales | Clostridia | Firmicutes |
| Lachnospiraceae sp. (HG3A.0127) | ODI | -0.03 | 0.08 | 0.149 | 3364 unclassified | unclassified | unclassified | Lachnospiraceae | Eubacteriales | Clostridia | Firmicutes |

Clostridia sp. (HG3A.1053)

T90 -0.033 0.057 0.15 3364 unclassified unclassified unclassified unclassified unclassified Clostridia Firmicutes

Clostridia sp.

(HG3A.1403)

AHI

-0.036

0.049

0.151 3004 unclassified unclassified

unclassified

unclassified

unclassified

Clostridia

Firmicutes

Eubacteriales sp.

# (HG3A.0213)

ODI -0.03 0.081 0.151 3364 unclassified unclassified unclassified unclassified Eubacteriales Clostridia Firmicutes

Eubacteriales sp.

(HG3A.0226)

T90

-0.033

0.058

0.151 3364 unclassified unclassified

unclassified

unclassified

Eubacteriales

Clostridia

Firmicutes

Eubacteriales sp.

# (HG3A.0235)

T90 -0.033 0.057 0.151 3364 unclassified unclassified unclassified unclassified Eubacteriales Clostridia Firmicutes

Eubacteriales sp.

(HG3A.0386)

ODI

-0.03

0.081

0.151 3364 unclassified unclassified

unclassified

unclassified

Eubacteriales

Clostridia

Firmicutes

Eubacteriales sp.

# (HG3A.1243)

T90 -0.033 0.058 0.151 3364 unclassified unclassified unclassified unclassified Eubacteriales Clostridia Firmicutes

| Latilactobacillus sakei |  |  |  |  | Latilactobacill |  | | | | | |
| --- | --- | --- | --- | --- | --- | --- | --- | --- | --- | --- | --- |
| subsp. sakei | T90 | 0.033 | 0.058 | 0.152 | 3364 us sakei | Latilactobacillus sakei | Latilactobacillus | Lactobacillaceae | Lactobacillales | Bacilli | Firmicutes |
| (HG3A.0836) subsp. sakei | | | | | | | | | | | |

Streptococcus salivarius

(HG3A.0071) ODI 0.03 0.082 0.152 3364 unclassified Streptococcus salivarius Streptococcus Streptococcaceae Lactobacillales Bacilli Firmicutes

Eubacteriales sp.

(HG3A.0204)

ODI

-0.03

0.083

0.153 3364 unclassified unclassified

unclassified

unclassified

Eubacteriales

Clostridia

Firmicutes

Eubacteriales sp.

# (HG3A.0396)

AHI -0.036 0.051 0.153 3004 unclassified unclassified unclassified unclassified Eubacteriales Clostridia Firmicutes

Eubacteriales sp.

(HG3A.0474)

AHI

-0.036

0.051

0.153 3004 unclassified unclassified

unclassified

unclassified

Eubacteriales

Clostridia

Firmicutes

Firmicutes sp.

# (HG3A.1075)

AHI -0.036 0.05 0.153 3004 unclassified unclassified unclassified unclassified unclassified unclassified Firmicutes

Gemmiger formicilis

(HG3A.0027)

AHI

0.036

0.05

0.153 3004 unclassified Gemmiger formicilis

Gemmiger

unclassified

Eubacteriales

Clostridia

Firmicutes

Oscillospiraceae sp.

# (HG3A.1421)

AHI 0.036 0.051 0.153 3004 unclassified unclassified unclassified Oscillospiraceae Eubacteriales Clostridia Firmicutes

Clostridia sp.

(HG3A.1148)

ODI

-0.03

0.083

0.154 3364 unclassified unclassified

unclassified

unclassified

unclassified

Clostridia

Firmicutes

Eubacteriales sp.

# (HG3A.0371)

T90 -0.033 0.059 0.154 3364 unclassified unclassified unclassified unclassified Eubacteriales Clostridia Firmicutes

Firmicutes sp.

(HG3A.0541)

AHI

-0.036

0.051

0.154 3004 unclassified unclassified

unclassified

unclassified

unclassified

unclassified

Firmicutes

Haemophilus

parainfluenzae (HG3A.0181)

Akkermansia

AHI -0.036 0.052 0.154 3004 unclassified

Haemophilus

parainfluenzae Haemophilus Pasteurellaceae Pasteurellales

Gammaproteoba

cteria Proteobacteria

Verrucomicrobia Verrucomicrob

| muciniphila T90  (HG3A.0110) | -0.033 | 0.06 | 0.155 | 3364 unclassified | Akkermansia mucin | iphila Akkermansia | Akkermansiaceae | Verrucomicrobia | les e ia |
| --- | --- | --- | --- | --- | --- | --- | --- | --- | --- |
| Clostridia sp. T90 | -0.033 | 0.06 | 0.155 | 3364 unclassified | unclassified | unclassified | unclassified | unclassified | Clostridia Firmicutes |

# (HG3A.0724)

| Clostridia sp.  (HG3A.1157) | ODI | -0.03 | 0.084 | 0.155 | 3364 unclassified | unclassified | unclassified | unclassified | unclassified | Clostridia | Firmicutes |
| --- | --- | --- | --- | --- | --- | --- | --- | --- | --- | --- | --- |
| Enterocloster aldenensis (HG3A.0362) | ODI | 0.03 | 0.084 | 0.155 | 3364 unclassified | Enterocloster aldenensis | Enterocloster | Lachnospiraceae | Eubacteriales | Clostridia | Firmicutes |
| Eubacteriales sp. (HG3A.1094) | ODI | -0.03 | 0.085 | 0.155 | 3364 unclassified | unclassified | unclassified | unclassified | Eubacteriales | Clostridia | Firmicutes |
| Oscillospiraceae sp.  (HG3A.0806) | T90 | -0.033 | 0.06 | 0.155 | 3364 unclassified | unclassified | unclassified | Oscillospiraceae | Eubacteriales | Clostridia | Firmicutes |
| Bacteroidales sp. (HG3A.0894) | AHI | -0.036 | 0.053 | 0.156 | 3004 unclassified | unclassified | unclassified | unclassified | Bacteroidales | Bacteroidia | Bacteroidetes |
| Eubacteriales sp.  (HG3A.0371) | AHI | -0.036 | 0.053 | 0.156 | 3004 unclassified | unclassified | unclassified | unclassified | Eubacteriales | Clostridia | Firmicutes |
| Eubacteriales sp. (HG3A.0703) | AHI | -0.036 | 0.053 | 0.156 | 3004 unclassified | unclassified | unclassified | unclassified | Eubacteriales | Clostridia | Firmicutes |
| Eubacteriales sp.  (HG3A.1167) | T90 | -0.033 | 0.061 | 0.156 | 3364 unclassified | unclassified | unclassified | unclassified | Eubacteriales | Clostridia | Firmicutes |
| Eubacteriales sp. (HG3A.1546) | T90 | -0.033 | 0.061 | 0.156 | 3364 unclassified | unclassified | unclassified | unclassified | Eubacteriales | Clostridia | Firmicutes |
| Oscillospiraceae sp.  (HG3A.0461) | ODI | -0.03 | 0.085 | 0.156 | 3364 unclassified | unclassified | unclassified | Oscillospiraceae | Eubacteriales | Clostridia | Firmicutes |
| Blautia argi (HG3A.1450) | T90 | 0.032 | 0.062 | 0.157 | 3364 unclassified | Blautia argi | Blautia | Lachnospiraceae | Eubacteriales | Clostridia | Firmicutes |
| Eubacteriales sp.  (HG3A.0305) | T90 | -0.032 | 0.062 | 0.157 | 3364 unclassified | unclassified | unclassified | unclassified | Eubacteriales | Clostridia | Firmicutes |
| Eubacteriales sp. (HG3A.0377) | T90 | -0.032 | 0.062 | 0.157 | 3364 unclassified | unclassified | unclassified | unclassified | Eubacteriales | Clostridia | Firmicutes |
| Eubacteriales sp.  (HG3A.0588) | T90 | -0.032 | 0.063 | 0.157 | 3364 unclassified | unclassified | unclassified | unclassified | Eubacteriales | Clostridia | Firmicutes |
| Eubacteriales sp. (HG3A.1103) | T90 | -0.032 | 0.063 | 0.157 | 3364 unclassified | unclassified | unclassified | unclassified | Eubacteriales | Clostridia | Firmicutes |
| Eubacteriales sp.  (HG3A.1321) | ODI | -0.03 | 0.086 | 0.157 | 3364 unclassified | unclassified | unclassified | unclassified | Eubacteriales | Clostridia | Firmicutes |
| Firmicutes sp. (HG3A.0923) | T90 | -0.032 | 0.062 | 0.157 | 3364 unclassified | unclassified | unclassified | unclassified | unclassified | unclassified | Firmicutes |
| Hungatella hathewayi (HG3A.0455) | T90 | 0.032 | 0.062 | 0.157 | 3364 unclassified | Hungatella hathewayi | Hungatella | Clostridiaceae | Eubacteriales | Clostridia | Firmicutes |
| Lachnospiraceae sp. (HG3A.1525) | ODI | -0.03 | 0.086 | 0.157 | 3364 unclassified | unclassified | unclassified | Lachnospiraceae | Eubacteriales | Clostridia | Firmicutes |
| Clostridia sp.  (HG3A.1111) | AHI | -0.036 | 0.054 | 0.158 | 3004 unclassified | unclassified | unclassified | unclassified | unclassified | Clostridia | Firmicutes |
| Eubacteriales sp. (HG3A.0390) | AHI | -0.035 | 0.054 | 0.158 | 3004 unclassified | unclassified | unclassified | unclassified | Eubacteriales | Clostridia | Firmicutes |

Eubacteriales sp. (HG3A.1243)

| Lachnospiraceae sp. (HG3A.0236) | T90 | -0.032 | 0.064 | 0.158 | 3364 unclassified | unclassified | unclassified | Lachnospiraceae | Eubacteriales | Clostridia | Firmicutes |
| --- | --- | --- | --- | --- | --- | --- | --- | --- | --- | --- | --- |
| Ruminococcus champanellensis  (HG3A.0716) | AHI | -0.035 | 0.055 | 0.158 | 3004 unclassified | Ruminococcus champanellensis | Ruminococcus | Oscillospiraceae | Eubacteriales | Clostridia | Firmicutes |
| Eubacteriales sp. (HG3A.0352) | ODI | -0.03 | 0.088 | 0.159 | 3364 unclassified | unclassified | unclassified | unclassified | Eubacteriales | Clostridia | Firmicutes |
| Eubacteriales sp.  (HG3A.0580) | T90 | -0.032 | 0.065 | 0.159 | 3364 unclassified | unclassified | unclassified | unclassified | Eubacteriales | Clostridia | Firmicutes |
| Eubacteriales sp. (HG3A.0807) | T90 | -0.032 | 0.065 | 0.159 | 3364 unclassified | unclassified | unclassified | unclassified | Eubacteriales | Clostridia | Firmicutes |
| Oscillospiraceae sp.  (HG3A.0849) | T90 | -0.032 | 0.065 | 0.159 | 3364 unclassified | unclassified | unclassified | Oscillospiraceae | Eubacteriales | Clostridia | Firmicutes |
| Blautia sp. SG-772 (HG3A.0063) | AHI | 0.035 | 0.056 | 0.16 | 3004 unclassified | Blautia sp. SG-772 | Blautia | Lachnospiraceae | Eubacteriales | Clostridia | Firmicutes |
| Clostridia sp.  (HG3A.1609) | T90 | -0.032 | 0.065 | 0.16 | 3364 unclassified | unclassified | unclassified | unclassified | unclassified | Clostridia | Firmicutes |
| [Clostridium] innocuum (HG3A.0365) | T90 | 0.032 | 0.066 | 0.16 | 3364 unclassified | [Clostridium] innocuum | Erysipelatoclostri dium | Erysipelotrichaceae | Erysipelotrichales | Erysipelotrichia | Firmicutes |
| Eggerthellales sp.  (HG3A.0177) | T90 | -0.032 | 0.065 | 0.16 | 3364 unclassified | unclassified | unclassified | unclassified | Eggerthellales | Coriobacteriia | Actinobacteria |
| Lachnospiraceae sp. (HG3A.0393) | ODI | 0.03 | 0.088 | 0.16 | 3364 unclassified | unclassified | unclassified | Lachnospiraceae | Eubacteriales | Clostridia | Firmicutes |
| Lachnospiraceae sp.  (HG3A.0899) | ODI | -0.03 | 0.088 | 0.16 | 3364 unclassified | unclassified | unclassified | Lachnospiraceae | Eubacteriales | Clostridia | Firmicutes |
| Staphylococcus aureus (HG3A.1538) | AHI | 0.035 | 0.056 | 0.16 | 3004 unclassified | Staphylococcus aureus | Staphylococcus | Staphylococcaceae | Bacillales | Bacilli | Firmicutes |
| Clostridia sp.  (HG3A.0929) | ODI | -0.03 | 0.089 | 0.161 | 3364 unclassified | unclassified | unclassified | unclassified | unclassified | Clostridia | Firmicutes |
| Collinsella sp. WCA1- 178-WT-3 (M2) (HG3A.1245) | ODI | 0.03 | 0.09 | 0.161 | 3364 unclassified | Collinsella sp. WCA1-178- WT-3 (M2) | Collinsella | Coriobacteriaceae | Coriobacteriales | Coriobacteriia | Actinobacteria |
| Eubacteriales sp.  (HG3A.0273) | ODI | -0.03 | 0.089 | 0.161 | 3364 unclassified | unclassified | unclassified | unclassified | Eubacteriales | Clostridia | Firmicutes |
| Eubacteriales sp. (HG3A.1045) | ODI | -0.03 | 0.09 | 0.161 | 3364 unclassified | unclassified | unclassified | unclassified | Eubacteriales | Clostridia | Firmicutes |
| Eubacteriales sp.  (HG3A.0352) | T90 | -0.032 | 0.067 | 0.162 | 3364 unclassified | unclassified | unclassified | unclassified | Eubacteriales | Clostridia | Firmicutes |
| Eubacteriales sp. (HG3A.0363) | AHI | -0.035 | 0.057 | 0.162 | 3004 unclassified | unclassified | unclassified | unclassified | Eubacteriales | Clostridia | Firmicutes |

AHI -0.035 0.055 0.158 3004 unclassified unclassified unclassified unclassified Eubacteriales Clostridia Firmicutes

Eubacteriales sp. (HG3A.0548)

T90 -0.032 0.067 0.162 3364 unclassified unclassified unclassified unclassified Eubacteriales Clostridia Firmicutes

# (HG3A.0423)

| Eubacteriales sp. (HG3A.0832) | ODI | -0.029 | 0.09 | 0.162 | 3364 unclassified | unclassified | unclassified | unclassified | Eubacteriales | Clostridia | Firmicutes |
| --- | --- | --- | --- | --- | --- | --- | --- | --- | --- | --- | --- |
| Eubacterium sp.  (HG3A.0214) | T90 | 0.032 | 0.067 | 0.162 | 3364 unclassified | unclassified | Eubacterium | Eubacteriaceae | Eubacteriales | Clostridia | Firmicutes |
| Parasutterella excrementihominis (HG3A.0159) | AHI | -0.035 | 0.056 | 0.162 | 3004 unclassified | Parasutterella excrementihominis | Parasutterella | Sutterellaceae | Burkholderiales | Betaproteobacteri a | Proteobacteria |
| Parasutterella excrementihominis  (HG3A.0159) | T90 | -0.032 | 0.067 | 0.162 | 3364 unclassified | Parasutterella excrementihominis | Parasutterella | Sutterellaceae | Burkholderiales | Betaproteobacteri a | Proteobacteria |
| Bacteria sp. (HG3A.1543) | ODI | -0.029 | 0.091 | 0.163 | 3364 unclassified | unclassified | unclassified | unclassified | unclassified | unclassified | unclassified |
| Clostridium sp. M62/1  (HG3A.0354) | AHI | 0.035 | 0.057 | 0.163 | 3004 unclassified | Clostridium sp. M62/1 | Clostridium | Clostridiaceae | Eubacteriales | Clostridia | Firmicutes |
| Eubacteriales sp. (HG3A.0062) | T90 | -0.032 | 0.068 | 0.163 | 3364 unclassified | unclassified | unclassified | unclassified | Eubacteriales | Clostridia | Firmicutes |
| Eubacteriales sp.  (HG3A.0358) | ODI | -0.029 | 0.092 | 0.163 | 3364 unclassified | unclassified | unclassified | unclassified | Eubacteriales | Clostridia | Firmicutes |
| Lachnospiraceae sp. (HG3A.0252) | T90 | -0.032 | 0.068 | 0.163 | 3364 unclassified | unclassified | unclassified | Lachnospiraceae | Eubacteriales | Clostridia | Firmicutes |
| Senegalimassilia  anaerobia (HG3A.0129) | ODI | 0.029 | 0.092 | 0.163 | 3364 unclassified | Senegalimassilia anaerobia | Senegalimassilia | Coriobacteriaceae | Coriobacteriales | Coriobacteriia | Actinobacteria |
| Erysipelatoclostridium sp. (HG3A.0313) | AHI | -0.035 | 0.058 | 0.164 | 3004 unclassified | unclassified | Erysipelatoclostri dium | Erysipelotrichaceae | Erysipelotrichales | Erysipelotrichia | Firmicutes |
| Eubacteriales sp.  (HG3A.0136) | AHI | -0.035 | 0.058 | 0.164 | 3004 unclassified | unclassified | unclassified | unclassified | Eubacteriales | Clostridia | Firmicutes |
| Blautia sp. AF19-10LB (HG3A.0157) | AHI | -0.035 | 0.059 | 0.165 | 3004 unclassified | Blautia sp. AF19-10LB | Blautia | Lachnospiraceae | Eubacteriales | Clostridia | Firmicutes |
| Eubacteriales sp.  (HG3A.0489) | AHI | -0.035 | 0.059 | 0.165 | 3004 unclassified | unclassified | unclassified | unclassified | Eubacteriales | Clostridia | Firmicutes |
| Eubacterium sp. AF16- 48 (HG3A.0219) | ODI | -0.029 | 0.094 | 0.165 | 3364 unclassified | Eubacterium sp. AF16-48 | Eubacterium | Eubacteriaceae | Eubacteriales | Clostridia | Firmicutes |
| Lachnospiraceae sp. (HG3A.1155)  Lactobacillus | ODI | -0.029 | 0.093 | 0.165 | 3364 unclassified | unclassified | unclassified | Lachnospiraceae | Eubacteriales | Clostridia | Firmicutes |
| acidophilus AHI  (HG3A.0672) | | -0.035 | 0.059 | 0.165 | 3004 unclassified | Lactobacillus acidophilus | Lactobacillus | Lactobacillaceae | Lactobacillales | Bacilli | Firmicutes |
| Phocaeicola plebeius ODI | | -0.029 | 0.093 | 0.165 | 3364 unclassified | Phocaeicola plebeius | Phocaeicola | unclassified | Bacteroidales | Bacteroidia | Bacteroidetes |

| Clostridia sp.  (HG3A.1039) | AHI | -0.035 | 0.06 | 0.166 | 3004 unclassified | unclassified | unclassified | unclassified | unclassified | Clostridia | Firmicutes |
| --- | --- | --- | --- | --- | --- | --- | --- | --- | --- | --- | --- |
| Eubacteriales sp.  (HG3A.0635) | AHI | -0.035 | 0.06 | 0.166 | 3004 unclassified | unclassified | unclassified | unclassified | Eubacteriales | Clostridia | Firmicutes |
| Oscillospiraceae sp. (HG3A.0693) | AHI | -0.035 | 0.06 | 0.166 | 3004 unclassified | unclassified | unclassified | Oscillospiraceae | Eubacteriales | Clostridia | Firmicutes |
| Clostridia sp.  (HG3A.0852) | ODI | -0.029 | 0.095 | 0.167 | 3364 unclassified | unclassified | unclassified | unclassified | unclassified | Clostridia | Firmicutes |
| Eubacteriales sp. (HG3A.1250) | ODI | -0.029 | 0.095 | 0.167 | 3364 unclassified | unclassified | unclassified | unclassified | Eubacteriales | Clostridia | Firmicutes |
| Victivallales sp.  (HG3A.0824) | ODI | -0.029 | 0.095 | 0.167 | 3364 unclassified | unclassified | unclassified | unclassified | Victivallales | Lentisphaeria | Lentisphaerae |
| Bacteria sp. (HG3A.0459) | ODI | -0.029 | 0.096 | 0.168 | 3364 unclassified | unclassified | unclassified | unclassified | unclassified | unclassified | unclassified |
| Dorea sp. AF24-7LB  (HG3A.0086) | T90 | 0.031 | 0.071 | 0.168 | 3364 unclassified | Dorea sp. AF24-7LB | Dorea | Lachnospiraceae | Eubacteriales | Clostridia | Firmicutes |
| Eubacteriales sp. (HG3A.0178) | ODI | -0.029 | 0.097 | 0.168 | 3364 unclassified | unclassified | unclassified | unclassified | Eubacteriales | Clostridia | Firmicutes |
| Eubacteriales sp.  (HG3A.0186) | ODI | -0.029 | 0.096 | 0.168 | 3364 unclassified | unclassified | unclassified | unclassified | Eubacteriales | Clostridia | Firmicutes |
| Faecalibacterium sp. (HG3A.0073) | AHI | -0.035 | 0.061 | 0.168 | 3004 unclassified | unclassified | Faecalibacterium | Oscillospiraceae | Eubacteriales | Clostridia | Firmicutes |
| Lachnoclostridium sp.  (HG3A.0655) | T90 | 0.031 | 0.071 | 0.168 | 3364 unclassified | unclassified | Lachnoclostridiu  m | Lachnospiraceae | Eubacteriales | Clostridia | Firmicutes |
| Eubacteriales sp. (HG3A.0476) | AHI | -0.034 | 0.062 | 0.169 | 3004 unclassified | unclassified | unclassified | unclassified | Eubacteriales | Clostridia | Firmicutes |
| Alistipes provencensis (HG3A.0877) | AHI | -0.034 | 0.062 | 0.17 | 3004 unclassified | Alistipes provencensis | Alistipes | Rikenellaceae | Bacteroidales | Bacteroidia | Bacteroidetes |
| Eubacteriales sp. (HG3A.1167) | ODI | -0.029 | 0.098 | 0.17 | 3364 unclassified | unclassified | unclassified | unclassified | Eubacteriales | Clostridia | Firmicutes |
| Eubacteriales sp.  (HG3A.0188) | AHI | -0.034 | 0.063 | 0.171 | 3004 unclassified | unclassified | unclassified | unclassified | Eubacteriales | Clostridia | Firmicutes |
| Eubacteriales sp. (HG3A.0443) | AHI | -0.034 | 0.064 | 0.171 | 3004 unclassified | unclassified | unclassified | unclassified | Eubacteriales | Clostridia | Firmicutes |
| Eubacteriales sp.  (HG3A.0565) | AHI | -0.034 | 0.063 | 0.171 | 3004 unclassified | unclassified | unclassified | unclassified | Eubacteriales | Clostridia | Firmicutes |
| Eubacteriales sp. (HG3A.0832) | AHI | -0.034 | 0.064 | 0.171 | 3004 unclassified | unclassified | unclassified | unclassified | Eubacteriales | Clostridia | Firmicutes |
| Eubacteriales sp.  (HG3A.1003) | AHI | -0.034 | 0.063 | 0.171 | 3004 unclassified | unclassified | unclassified | unclassified | Eubacteriales | Clostridia | Firmicutes |
| Firmicutes sp. (HG3A.1195) | AHI | -0.034 | 0.063 | 0.171 | 3004 unclassified | unclassified | unclassified | unclassified | unclassified | unclassified | Firmicutes |

Eubacteriales sp. (HG3A.0637)

ODI -0.029 0.099 0.172 3364 unclassified unclassified unclassified unclassified Eubacteriales Clostridia Firmicutes

| Sutterellaceae sp. (HG3A.1122) | AHI | 0.034 | 0.064 | 0.172 | 3004 unclassified | unclassified | unclassified | Sutterellaceae | Burkholderiales | Betaproteobacteri Proteobacteria a |
| --- | --- | --- | --- | --- | --- | --- | --- | --- | --- | --- |
| Clostridia sp.  (HG3A.0918) | T90 | -0.031 | 0.073 | 0.173 | 3364 unclassified | unclassified | unclassified | unclassified | unclassified | Clostridia Firmicutes |
| Eubacteriales sp. (HG3A.0418) | AHI | -0.034 | 0.065 | 0.173 | 3004 unclassified | unclassified | unclassified | unclassified | Eubacteriales | Clostridia Firmicutes |
| Eubacteriales sp.  (HG3A.0654) | ODI | -0.029 | 0.1 | 0.173 | 3364 unclassified | unclassified | unclassified | unclassified | Eubacteriales | Clostridia Firmicutes |
| Intestinimonas sp. (HG3A.1018) | T90 | -0.031 | 0.073 | 0.173 | 3364 unclassified | unclassified | Intestinimonas | unclassified | Eubacteriales | Clostridia Firmicutes |
| Clostridia sp.  (HG3A.0787) | T90 | -0.031 | 0.074 | 0.174 | 3364 unclassified | unclassified | unclassified | unclassified | unclassified | Clostridia Firmicutes |
| Eubacteriales sp. (HG3A.0263) | T90 | -0.031 | 0.074 | 0.174 | 3364 unclassified | unclassified | unclassified | unclassified | Eubacteriales | Clostridia Firmicutes |
| Eubacteriales sp.  (HG3A.0490) | T90 | -0.031 | 0.074 | 0.174 | 3364 unclassified | unclassified | unclassified | unclassified | Eubacteriales | Clostridia Firmicutes |
| Eubacteriales sp. (HG3A.1422) | T90 | -0.031 | 0.074 | 0.174 | 3364 unclassified | unclassified | unclassified | unclassified | Eubacteriales | Clostridia Firmicutes |
| Eubacteriales sp.  (HG3A.0535) | T90 | -0.031 | 0.075 | 0.175 | 3364 unclassified | unclassified | unclassified | unclassified | Eubacteriales | Clostridia Firmicutes |
| Agathobaculum desmolans (HG3A.1429) | T90 | 0.031 | 0.076 | 0.176 | 3364 unclassified | Agathobaculum desmolans | Agathobaculum | Oscillospiraceae | Eubacteriales | Clostridia Firmicutes |
| Anaerotignum  lactatifermentans (HG3A.0676) | T90 | 0.031 | 0.076 | 0.176 | 3364 unclassified | Anaerotignum lactatifermentans | Anaerotignum | Lachnospiraceae | Eubacteriales | Clostridia Firmicutes |
| Eubacteriales sp. (HG3A.0633) | T90 | -0.031 | 0.076 | 0.176 | 3364 unclassified | unclassified | unclassified | unclassified | Eubacteriales | Clostridia Firmicutes |
| Clostridia sp.  (HG3A.1035) | T90 | -0.031 | 0.078 | 0.177 | 3364 unclassified | unclassified | unclassified | unclassified | unclassified | Clostridia Firmicutes |
| Clostridia sp. (HG3A.1217) | T90 | -0.031 | 0.078 | 0.177 | 3364 unclassified | unclassified | unclassified | unclassified | unclassified | Clostridia Firmicutes |
| Eubacteriales sp.  (HG3A.0537) | T90 | -0.031 | 0.077 | 0.177 | 3364 unclassified | unclassified | unclassified | unclassified | Eubacteriales | Clostridia Firmicutes |
| Firmicutes sp. (HG3A.0436) | T90 | -0.031 | 0.077 | 0.177 | 3364 unclassified | unclassified | unclassified | unclassified | unclassified | unclassified Firmicutes |
| Oxalobacter sp.  (HG3A.1218) | T90 | -0.031 | 0.078 | 0.177 | 3364 unclassified | unclassified | Oxalobacter | Oxalobacteraceae | Burkholderiales | Betaproteobacteri Proteobacteria  a |
| Eubacteriales sp. (HG3A.0221) | AHI | -0.034 | 0.067 | 0.178 | 3004 unclassified | unclassified | unclassified | unclassified | Eubacteriales | Clostridia Firmicutes |

Eubacteriales sp.

# (HG3A.0289)

ODI -0.028 0.103 0.178 3364 unclassified unclassified unclassified unclassified Eubacteriales Clostridia Firmicutes

| Firmicutes sp.  (HG3A.0454) | AHI | -0.034 | 0.067 | 0.178 | 3004 unclassified | unclassified | unclassified | unclassified | unclassified | unclassified | Firmicutes |
| --- | --- | --- | --- | --- | --- | --- | --- | --- | --- | --- | --- |
| Firmicutes sp.  (HG3A.1085) | AHI | -0.034 | 0.068 | 0.178 | 3004 unclassified | unclassified | unclassified | unclassified | unclassified | unclassified | Firmicutes |
| Eubacteriales sp. (HG3A.0309) | T90 | -0.031 | 0.079 | 0.179 | 3364 unclassified | unclassified | unclassified | unclassified | Eubacteriales | Clostridia | Firmicutes |
| Eubacteriales sp.  (HG3A.0325) | AHI | -0.034 | 0.068 | 0.179 | 3004 unclassified | unclassified | unclassified | unclassified | Eubacteriales | Clostridia | Firmicutes |
| Eubacteriales sp. (HG3A.0639) | T90 | -0.031 | 0.079 | 0.179 | 3364 unclassified | unclassified | unclassified | unclassified | Eubacteriales | Clostridia | Firmicutes |
| Victivallales sp.  (HG3A.0824) | T90 | -0.031 | 0.079 | 0.179 | 3364 unclassified | unclassified | unclassified | unclassified | Victivallales | Lentisphaeria | Lentisphaerae |
| Eubacteriales sp. (HG3A.0477) | ODI | -0.028 | 0.105 | 0.18 | 3364 unclassified | unclassified | unclassified | unclassified | Eubacteriales | Clostridia | Firmicutes |
| Massilistercora timonensis  (HG3A.0458) | ODI | -0.028 | 0.105 | 0.18 | 3364 unclassified | Massilistercora timonensis | Massilistercora | unclassified | Eubacteriales | Clostridia | Firmicutes |
| Eubacteriales sp. (HG3A.0390) | T90 | -0.03 | 0.081 | 0.181 | 3364 unclassified | unclassified | unclassified | unclassified | Eubacteriales | Clostridia | Firmicutes |
| Bacteria sp.  (HG3A.1349) | ODI | -0.028 | 0.107 | 0.182 | 3364 unclassified | unclassified | unclassified | unclassified | unclassified | unclassified | unclassified |
| Clostridia sp. (HG3A.1058) | ODI | -0.028 | 0.106 | 0.182 | 3364 unclassified | unclassified | unclassified | unclassified | unclassified | Clostridia | Firmicutes |
| Clostridia sp.  (HG3A.1410) | ODI | -0.028 | 0.106 | 0.182 | 3364 unclassified | unclassified | unclassified | unclassified | unclassified | Clostridia | Firmicutes |
| Eggerthellales sp. (HG3A.0848) | ODI | -0.028 | 0.107 | 0.182 | 3364 unclassified | unclassified | unclassified | unclassified | Eggerthellales | Coriobacteriia | Actinobacteria |
| Eubacteriales sp.  (HG3A.0754) | ODI | -0.028 | 0.107 | 0.182 | 3364 unclassified | unclassified | unclassified | unclassified | Eubacteriales | Clostridia | Firmicutes |
| Streptococcus parasanguinis (HG3A.0117) | ODI | 0.028 | 0.107 | 0.182 | 3364 unclassified | Streptococcus parasanguinis | Streptococcus | Streptococcaceae | Lactobacillales | Bacilli | Firmicutes |
| Clostridia sp.  (HG3A.0893) | ODI | -0.028 | 0.109 | 0.183 | 3364 unclassified | unclassified | unclassified | unclassified | unclassified | Clostridia | Firmicutes |
| Erysipelotrichales sp. (HG3A.0303) | ODI | -0.028 | 0.108 | 0.183 | 3364 unclassified | unclassified | unclassified | unclassified | Erysipelotrichales | Erysipelotrichia | Firmicutes |
| Eubacteriales sp.  (HG3A.0516) | ODI | -0.028 | 0.108 | 0.183 | 3364 unclassified | unclassified | unclassified | unclassified | Eubacteriales | Clostridia | Firmicutes |
| Proteobacteria sp. (HG3A.0327) | ODI | -0.028 | 0.109 | 0.183 | 3364 unclassified | unclassified | unclassified | unclassified | unclassified | unclassified | Proteobacteria |

Pseudoruminococcus massiliensis (HG3A.0346)

T90 -0.03 0.081 0.183 3364 unclassified

Pseudoruminococcus massiliensis

Pseudoruminoco

ccus Oscillospiraceae Eubacteriales Clostridia Firmicutes

| Clostridia sp.  (HG3A.1128) | ODI | -0.028 | 0.11 | 0.184 | 3364 unclassified | unclassified | unclassified | unclassified | unclassified | Clostridia | Firmicutes |
| --- | --- | --- | --- | --- | --- | --- | --- | --- | --- | --- | --- |
| Eubacteriales sp.  (HG3A.0744) | ODI | -0.028 | 0.109 | 0.184 | 3364 unclassified | unclassified | unclassified | unclassified | Eubacteriales | Clostridia | Firmicutes |
| Rikenellaceae sp. (HG3A.1022) | ODI | -0.028 | 0.11 | 0.184 | 3364 unclassified | unclassified | unclassified | Rikenellaceae | Bacteroidales | Bacteroidia | Bacteroidetes |
| Streptococcus agalactiae (HG3A.1733) | ODI | 0.028 | 0.11 | 0.184 | 3364 unclassified | Streptococcus agalactiae | Streptococcus | Streptococcaceae | Lactobacillales | Bacilli | Firmicutes |
| Bacteroidales sp. (HG3A.0340) | T90 | -0.03 | 0.083 | 0.185 | 3364 unclassified | unclassified | unclassified | unclassified | Bacteroidales | Bacteroidia | Bacteroidetes |
| Candidatus Borkfalkia ceftriaxoniphila (HG3A.0595) | T90 | -0.03 | 0.083 | 0.185 | 3364 unclassified | Candidatus Borkfalkia ceftriaxoniphila | Candidatus Borkfalkia | Candidatus Borkfalkiaceae | Candidatus Borkfalkiales | Clostridia | Firmicutes |
| Clostridia sp. (HG3A.0011) | T90 | 0.03 | 0.083 | 0.185 | 3364 unclassified | unclassified | unclassified | unclassified | unclassified | Clostridia | Firmicutes |
| Eubacteriales sp.  (HG3A.1354) | T90 | -0.03 | 0.083 | 0.185 | 3364 unclassified | unclassified | unclassified | unclassified | Eubacteriales | Clostridia | Firmicutes |
| Clostridia sp. (HG3A.0828) | T90 | -0.03 | 0.085 | 0.186 | 3364 unclassified | unclassified | unclassified | unclassified | unclassified | Clostridia | Firmicutes |
| Eubacteriales sp.  (HG3A.0858) | T90 | -0.03 | 0.085 | 0.186 | 3364 unclassified | unclassified | unclassified | unclassified | Eubacteriales | Clostridia | Firmicutes |
| Eubacteriales sp. (HG3A.1199) | T90 | 0.03 | 0.085 | 0.186 | 3364 unclassified | unclassified | unclassified | unclassified | Eubacteriales | Clostridia | Firmicutes |
| Phascolarctobacterium succinatutens  (HG3A.0315) | T90 | 0.03 | 0.084 | 0.186 | 3364 unclassified | Phascolarctobacterium succinatutens | Phascolarctobact erium | Acidaminococcaceae | Acidaminococcales | Negativicutes | Firmicutes |
| Victivallis lenta (HG3A.0525) | ODI | -0.028 | 0.111 | 0.186 | 3364 unclassified | Victivallis lenta | Victivallis | Victivallaceae | Victivallales | Lentisphaeria | Lentisphaerae |
| Eubacteriales sp.  (HG3A.0260) | AHI | -0.033 | 0.072 | 0.187 | 3004 unclassified | unclassified | unclassified | unclassified | Eubacteriales | Clostridia | Firmicutes |
| Eubacteriales sp. (HG3A.0956) | T90 | -0.03 | 0.085 | 0.187 | 3364 unclassified | unclassified | unclassified | unclassified | Eubacteriales | Clostridia | Firmicutes |
| Eubacteriales sp.  (HG3A.1019) | T90 | -0.03 | 0.086 | 0.187 | 3364 unclassified | unclassified | unclassified | unclassified | Eubacteriales | Clostridia | Firmicutes |
| Eubacteriales sp. (HG3A.1126) | AHI | 0.033 | 0.072 | 0.187 | 3004 unclassified | unclassified | unclassified | unclassified | Eubacteriales | Clostridia | Firmicutes |
| Alistipes sp. AF17-16  (HG3A.0150) | AHI | -0.033 | 0.072 | 0.188 | 3004 unclassified | Alistipes sp. AF17-16 | Alistipes | Rikenellaceae | Bacteroidales | Bacteroidia | Bacteroidetes |
| Eubacteriales sp. (HG3A.0568) | AHI | -0.033 | 0.072 | 0.188 | 3004 unclassified | unclassified | unclassified | unclassified | Eubacteriales | Clostridia | Firmicutes |

Eubacteriales sp. (HG3A.0652)

T90 -0.03 0.086 0.188 3364 unclassified unclassified unclassified unclassified Eubacteriales Clostridia Firmicutes

Clostridia sp.

(HG3A.1038)

ODI

-0.028

0.114

0.189 3364 unclassified unclassified

unclassified

unclassified

unclassified

Clostridia

Firmicutes

Eubacteriales sp.

# (HG3A.0528)

ODI -0.028 0.114 0.189 3364 unclassified unclassified unclassified unclassified Eubacteriales Clostridia Firmicutes

Eubacteriales sp.

(HG3A.0537)

ODI

-0.027

0.114

0.189 3364 unclassified unclassified

unclassified

unclassified

Eubacteriales

Clostridia

Firmicutes

Eubacteriales sp.

# (HG3A.0637)

T90 -0.03 0.087 0.189 3364 unclassified unclassified unclassified unclassified Eubacteriales Clostridia Firmicutes

Dorea phocaeensis

(HG3A.0865)

AHI

0.033

0.074

0.19 3004 unclassified Dorea phocaeensis

Dorea

Lachnospiraceae

Eubacteriales

Clostridia

Firmicutes

Eubacteriales sp.

# (HG3A.0113)

AHI -0.033 0.075 0.19 3004 unclassified unclassified unclassified unclassified Eubacteriales Clostridia Firmicutes

Eubacteriales sp.

(HG3A.0270)

AHI

-0.033

0.074

0.19 3004 unclassified unclassified

unclassified

unclassified

Eubacteriales

Clostridia

Firmicutes

Eubacteriales sp.

# (HG3A.0450)

AHI -0.033 0.074 0.19 3004 unclassified unclassified unclassified unclassified Eubacteriales Clostridia Firmicutes

Eubacteriales sp.

(HG3A.1086)

T90

-0.03

0.088

0.19 3364 unclassified unclassified

unclassified

unclassified

Eubacteriales

Clostridia

Firmicutes

Lacticaseibacillus paracasei subsp. paracasei (HG3A.0853)

Lacticaseibaci

T90 0.03 0.088 0.19 3364 llus paracasei subsp.

paracasei

Lacticaseibacillus paracasei

Lacticaseibacillus Lactobacillaceae Lactobacillales Bacilli Firmicutes

Oscillospiraceae sp.

(HG3A.0616)

AHI

-0.033

0.074

0.19 3004 unclassified unclassified

unclassified

Oscillospiraceae

Eubacteriales

Clostridia

Firmicutes

Mesosutterella

| multiformis ODI  (HG3A.0520) | -0.027 | 0.116 | 0.191 | 3364 unclassified | multiformis | Mesosutterella | Sutterellaceae | Burkholderiales | a | Proteobacteria |
| --- | --- | --- | --- | --- | --- | --- | --- | --- | --- | --- |
| Oscillospiraceae sp. T90 | -0.03 | 0.089 | 0.192 | 3364 unclassified | unclassified | unclassified | Oscillospiraceae | Eubacteriales | Clostridia | Firmicutes |

Mesosutterella

Betaproteobacteri

# (HG3A.0966)

Erysipelatoclostridium

ramosum (HG3A.0538) T90 0.029 0.09 0.193 3364 unclassified

Erysipelatoclostridium ramosum

Erysipelatoclostri

dium Erysipelotrichaceae Erysipelotrichales Erysipelotrichia Firmicutes

Eubacteriales sp.

(HG3A.0381)

T90

-0.029

0.091

0.193 3364 unclassified unclassified

unclassified

unclassified

Eubacteriales

Clostridia

Firmicutes

Eubacteriales sp.

# (HG3A.1136)

T90 -0.029 0.091 0.193 3364 unclassified unclassified unclassified unclassified Eubacteriales Clostridia Firmicutes

Blautia sp. SG-772

(HG3A.0063)

T90

0.029

0.091

0.194 3364 unclassified Blautia sp. SG-772

Blautia

Lachnospiraceae

Eubacteriales

Clostridia

Firmicutes

Clostridia sp.

# (HG3A.0767)

ODI -0.027 0.118 0.195 3364 unclassified unclassified unclassified unclassified unclassified Clostridia Firmicutes

Clostridia sp.

(HG3A.0599)

T90

-0.029

0.092

0.196 3364 unclassified unclassified

unclassified

unclassified

unclassified

Clostridia

Firmicutes

Dorea longicatena

# (HG3A.0039)

ODI 0.027 0.12 0.196 3364 unclassified Dorea longicatena Dorea Lachnospiraceae Eubacteriales Clostridia Firmicutes

| Eubacteriales sp.  (HG3A.0626) | AHI | -0.033 | 0.077 | 0.196 | 3004 unclassified | unclassified | unclassified | unclassified | Eubacteriales | Clostridia | Firmicutes |
| --- | --- | --- | --- | --- | --- | --- | --- | --- | --- | --- | --- |
| Eubacteriales sp.  (HG3A.0628) | ODI | -0.027 | 0.119 | 0.196 | 3364 unclassified | unclassified | unclassified | unclassified | Eubacteriales | Clostridia | Firmicutes |
| Holdemanella sp. (HG3A.0366) | T90 | 0.029 | 0.093 | 0.196 | 3364 unclassified | unclassified | Holdemanella | Erysipelotrichaceae | Erysipelotrichales | Erysipelotrichia | Firmicutes |
| Oscillospiraceae sp.  (HG3A.0146) | AHI | -0.033 | 0.077 | 0.196 | 3004 unclassified | unclassified | unclassified | Oscillospiraceae | Eubacteriales | Clostridia | Firmicutes |
| Oscillospiraceae sp. (HG3A.0966) | ODI | -0.027 | 0.12 | 0.196 | 3364 unclassified | unclassified | unclassified | Oscillospiraceae | Eubacteriales | Clostridia | Firmicutes |
| Prevotella sp.  (HG3A.1040) | ODI | 0.027 | 0.12 | 0.196 | 3364 unclassified | unclassified | Prevotella | Prevotellaceae | Bacteroidales | Bacteroidia | Bacteroidetes |
| Roseburia faecis (HG3A.0058) | ODI | 0.027 | 0.12 | 0.196 | 3364 unclassified | Roseburia faecis | Roseburia | Lachnospiraceae | Eubacteriales | Clostridia | Firmicutes |
| Streptococcus anginosus (HG3A.0680) | T90 | 0.029 | 0.093 | 0.196 | 3364 unclassified | Streptococcus anginosus | Streptococcus | Streptococcaceae | Lactobacillales | Bacilli | Firmicutes |
| Blautia argi (HG3A.1450) | AHI | 0.032 | 0.078 | 0.197 | 3004 unclassified | Blautia argi | Blautia | Lachnospiraceae | Eubacteriales | Clostridia | Firmicutes |
| Eubacteriales sp.  (HG3A.0267) | AHI | -0.033 | 0.078 | 0.197 | 3004 unclassified | unclassified | unclassified | unclassified | Eubacteriales | Clostridia | Firmicutes |
| Eubacteriales sp. (HG3A.0627) | AHI | -0.032 | 0.079 | 0.197 | 3004 unclassified | unclassified | unclassified | unclassified | Eubacteriales | Clostridia | Firmicutes |
| Lachnospiraceae sp.  (HG3A.0127) | AHI | -0.032 | 0.079 | 0.197 | 3004 unclassified | unclassified | unclassified | Lachnospiraceae | Eubacteriales | Clostridia | Firmicutes |
| Clostridia sp. (HG3A.1262) | ODI | -0.027 | 0.122 | 0.198 | 3364 unclassified | unclassified | unclassified | unclassified | unclassified | Clostridia | Firmicutes |
| Dialister pneumosintes (HG3A.1496) | AHI | 0.032 | 0.079 | 0.198 | 3004 unclassified | Dialister pneumosintes | Dialister | Veillonellaceae | Veillonellales | Negativicutes | Firmicutes |
| Eubacteriales sp. (HG3A.0087) | ODI | -0.027 | 0.122 | 0.198 | 3364 unclassified | unclassified | unclassified | unclassified | Eubacteriales | Clostridia | Firmicutes |
| Eubacteriales sp.  (HG3A.0232) | AHI | -0.032 | 0.08 | 0.198 | 3004 unclassified | unclassified | unclassified | unclassified | Eubacteriales | Clostridia | Firmicutes |
| Eubacteriales sp. (HG3A.1078) | ODI | -0.027 | 0.123 | 0.199 | 3364 unclassified | unclassified | unclassified | unclassified | Eubacteriales | Clostridia | Firmicutes |
| Clostridia sp.  (HG3A.0787) | AHI | -0.032 | 0.082 | 0.2 | 3004 unclassified | unclassified | unclassified | unclassified | unclassified | Clostridia | Firmicutes |
| Eubacteriales sp. (HG3A.0138) | ODI | -0.027 | 0.123 | 0.2 | 3364 unclassified | unclassified | unclassified | unclassified | Eubacteriales | Clostridia | Firmicutes |
| Eubacteriales sp.  (HG3A.0148) | AHI | -0.032 | 0.082 | 0.2 | 3004 unclassified | unclassified | unclassified | unclassified | Eubacteriales | Clostridia | Firmicutes |
| Eubacteriales sp. (HG3A.0373) | AHI | -0.032 | 0.081 | 0.2 | 3004 unclassified | unclassified | unclassified | unclassified | Eubacteriales | Clostridia | Firmicutes |

Eubacteriales sp. (HG3A.0694)

| Firmicutes sp. (HG3A.0581) | AHI | -0.032 | 0.081 | 0.2 | 3004 unclassified | unclassified | unclassified | unclassified | unclassified | unclassified | Firmicutes |
| --- | --- | --- | --- | --- | --- | --- | --- | --- | --- | --- | --- |
| Roseburia sp. AM16-25  (HG3A.0344) | AHI | -0.032 | 0.081 | 0.2 | 3004 unclassified | Roseburia sp. AM16-25 | Roseburia | Lachnospiraceae | Eubacteriales | Clostridia | Firmicutes |
| Clostridia sp. (HG3A.1111) | ODI | -0.027 | 0.125 | 0.201 | 3364 unclassified | unclassified | unclassified | unclassified | unclassified | Clostridia | Firmicutes |
| Eubacteriales sp.  (HG3A.0715) | T90 | -0.029 | 0.096 | 0.201 | 3364 unclassified | unclassified | unclassified | unclassified | Eubacteriales | Clostridia | Firmicutes |
| Oscillospiraceae sp. (HG3A.0461) | T90 | -0.029 | 0.095 | 0.201 | 3364 unclassified | unclassified | unclassified | Oscillospiraceae | Eubacteriales | Clostridia | Firmicutes |
| Streptococcus gordonii (HG3A.0713) | ODI | 0.027 | 0.125 | 0.201 | 3364 unclassified | Streptococcus gordonii | Streptococcus | Streptococcaceae | Lactobacillales | Bacilli | Firmicutes |
| Bacteria sp. (HG3A.0911) | ODI | -0.027 | 0.126 | 0.202 | 3364 unclassified | unclassified | unclassified | unclassified | unclassified | unclassified | unclassified |
| Bacteria sp.  (HG3A.1543) | AHI | -0.032 | 0.083 | 0.202 | 3004 unclassified | unclassified | unclassified | unclassified | unclassified | unclassified | unclassified |
| Eubacteriales sp. (HG3A.0175) | AHI | -0.032 | 0.083 | 0.202 | 3004 unclassified | unclassified | unclassified | unclassified | Eubacteriales | Clostridia | Firmicutes |
| Mogibacterium kristiansenii  (HG3A.0522) | T90 | -0.029 | 0.096 | 0.202 | 3364 unclassified | Mogibacterium kristiansenii | Mogibacterium | Clostridiales Family  XIII. Incertae Sedis | Eubacteriales | Clostridia | Firmicutes |
| Pediococcus pentosaceus (HG3A.1246) | ODI | 0.027 | 0.126 | 0.202 | 3364 unclassified | Pediococcus pentosaceus | Pediococcus | Lactobacillaceae | Lactobacillales | Bacilli | Firmicutes |
| Clostridia sp.  (HG3A.0645) | AHI | -0.032 | 0.084 | 0.203 | 3004 unclassified | unclassified | unclassified | unclassified | unclassified | Clostridia | Firmicutes |
| Clostridia sp. (HG3A.1127) | AHI | -0.032 | 0.084 | 0.203 | 3004 unclassified | unclassified | unclassified | unclassified | unclassified | Clostridia | Firmicutes |
| Clostridia sp.  (HG3A.0276) | ODI | -0.026 | 0.128 | 0.204 | 3364 unclassified | unclassified | unclassified | unclassified | unclassified | Clostridia | Firmicutes |
| Clostridia sp. (HG3A.1427) | ODI | -0.027 | 0.127 | 0.204 | 3364 unclassified | unclassified | unclassified | unclassified | unclassified | Clostridia | Firmicutes |
| Eubacteriales sp.  (HG3A.0633) | ODI | -0.026 | 0.128 | 0.204 | 3364 unclassified | unclassified | unclassified | unclassified | Eubacteriales | Clostridia | Firmicutes |
| Eubacteriales sp. (HG3A.0759) | ODI | -0.026 | 0.128 | 0.204 | 3364 unclassified | unclassified | unclassified | unclassified | Eubacteriales | Clostridia | Firmicutes |
| Oscillibacter sp.  (HG3A.0046) | ODI | -0.026 | 0.128 | 0.204 | 3364 unclassified | unclassified | Oscillibacter | Oscillospiraceae | Eubacteriales | Clostridia | Firmicutes |
| Firmicutes sp. (HG3A.0301) | ODI | -0.026 | 0.129 | 0.205 | 3364 unclassified | unclassified | unclassified | unclassified | unclassified | unclassified | Firmicutes |

AHI -0.032 0.081 0.2 3004 unclassified unclassified unclassified unclassified Eubacteriales Clostridia Firmicutes

Oscillospiraceae sp. (HG3A.0384)

AHI -0.032 0.086 0.205 3004 unclassified unclassified unclassified Oscillospiraceae Eubacteriales Clostridia Firmicutes

| Ruminococcus sp. (HG3A.0337) | T90 | -0.029 | 0.098 | 0.205 | 3364 unclassified | unclassified | Ruminococcus | Oscillospiraceae | Eubacteriales | Clostridia | Firmicutes |
| --- | --- | --- | --- | --- | --- | --- | --- | --- | --- | --- | --- |
| Enterocloster  clostridioformis (HG3A.0686) | T90 | 0.029 | 0.099 | 0.206 | 3364 unclassified | Enterocloster clostridioformis | Enterocloster | Lachnospiraceae | Eubacteriales | Clostridia | Firmicutes |
| Eubacteriales sp. (HG3A.0273) | T90 | -0.029 | 0.1 | 0.206 | 3364 unclassified | unclassified | unclassified | unclassified | Eubacteriales | Clostridia | Firmicutes |
| Eubacteriales sp.  (HG3A.0473) | ODI | -0.026 | 0.13 | 0.206 | 3364 unclassified | unclassified | unclassified | unclassified | Eubacteriales | Clostridia | Firmicutes |
| Eubacteriales sp. (HG3A.0916) | T90 | -0.029 | 0.099 | 0.206 | 3364 unclassified | unclassified | unclassified | unclassified | Eubacteriales | Clostridia | Firmicutes |
| Eubacterium sp. AM49- 13BH (HG3A.0251) | T90 | -0.029 | 0.1 | 0.206 | 3364 unclassified | Eubacterium sp. AM49- 13BH | Eubacterium | Eubacteriaceae | Eubacteriales | Clostridia | Firmicutes |
| Levilactobacillus brevis (HG3A.1848) | ODI | 0.026 | 0.13 | 0.206 | 3364 unclassified | Levilactobacillus brevis | Levilactobacillus | Lactobacillaceae | Lactobacillales | Bacilli | Firmicutes |
| Succinatimonas hippei  (HG3A.1322) | T90 | 0.029 | 0.099 | 0.206 | 3364 unclassified | Succinatimonas hippei | Succinatimonas | Succinivibrionaceae | Aeromonadales | Gammaproteoba  cteria | Proteobacteria |
| Bacteria sp. (HG3A.1096) | T90 | -0.029 | 0.101 | 0.207 | 3364 unclassified | unclassified | unclassified | unclassified | unclassified | unclassified | unclassified |
| Eubacterium ramulus (HG3A.0068) | AHI | 0.032 | 0.087 | 0.207 | 3004 unclassified | Eubacterium ramulus | Eubacterium | Eubacteriaceae | Eubacteriales | Clostridia | Firmicutes |
| Clostridia sp. (HG3A.0893) | T90 | -0.028 | 0.101 | 0.208 | 3364 unclassified | unclassified | unclassified | unclassified | unclassified | Clostridia | Firmicutes |
| Eubacteriales sp.  (HG3A.0518) | T90 | -0.028 | 0.102 | 0.208 | 3364 unclassified | unclassified | unclassified | unclassified | Eubacteriales | Clostridia | Firmicutes |
| Eubacteriales sp. (HG3A.0335) | ODI | 0.026 | 0.132 | 0.209 | 3364 unclassified | unclassified | unclassified | unclassified | Eubacteriales | Clostridia | Firmicutes |
| Prevotella colorans  (HG3A.1470) | T90 | -0.028 | 0.103 | 0.209 | 3364 unclassified | Prevotella colorans | Prevotella | Prevotellaceae | Bacteroidales | Bacteroidia | Bacteroidetes |
| Candidatus Borkfalkia ceftriaxoniphila (HG3A.0595) | ODI | -0.026 | 0.134 | 0.21 | 3364 unclassified | Candidatus Borkfalkia ceftriaxoniphila | Candidatus Borkfalkia | Candidatus Borkfalkiaceae | Candidatus Borkfalkiales | Clostridia | Firmicutes |
| Candidatus  Borkfalkiales sp. (HG3A.1397) | T90 | -0.028 | 0.103 | 0.21 | 3364 unclassified | unclassified | unclassified | unclassified | Candidatus Borkfalkiales | Clostridia | Firmicutes |
| Eubacteriales sp. (HG3A.0260) | T90 | -0.028 | 0.104 | 0.21 | 3364 unclassified | unclassified | unclassified | unclassified | Eubacteriales | Clostridia | Firmicutes |

Eubacteriales sp.

# (HG3A.0441)

AHI -0.031 0.088 0.21 3004 unclassified unclassified unclassified unclassified Eubacteriales Clostridia Firmicutes

| Eubacteriales sp.  (HG3A.0685) | ODI | -0.026 | 0.134 | 0.21 | 3364 unclassified | unclassified | unclassified | unclassified | Eubacteriales | Clostridia | Firmicutes |
| --- | --- | --- | --- | --- | --- | --- | --- | --- | --- | --- | --- |
| Eubacteriales sp.  (HG3A.0773) | ODI | -0.026 | 0.133 | 0.21 | 3364 unclassified | unclassified | unclassified | unclassified | Eubacteriales | Clostridia | Firmicutes |
| Faecalibacterium sp. (HG3A.0073) | T90 | -0.028 | 0.104 | 0.21 | 3364 unclassified | unclassified | Faecalibacterium | Oscillospiraceae | Eubacteriales | Clostridia | Firmicutes |
| Enterocloster sp.  (HG3A.1529) | T90 | 0.028 | 0.104 | 0.211 | 3364 unclassified | unclassified | Enterocloster | Lachnospiraceae | Eubacteriales | Clostridia | Firmicutes |
| Eubacteriales sp. (HG3A.0794) | AHI | -0.031 | 0.089 | 0.211 | 3004 unclassified | unclassified | unclassified | unclassified | Eubacteriales | Clostridia | Firmicutes |
| Lachnospiraceae sp.  (HG3A.1641) | AHI | 0.031 | 0.089 | 0.211 | 3004 unclassified | unclassified | unclassified | Lachnospiraceae | Eubacteriales | Clostridia | Firmicutes |
| Firmicutes sp. (HG3A.1471) | T90 | -0.028 | 0.105 | 0.212 | 3364 unclassified | unclassified | unclassified | unclassified | unclassified | unclassified | Firmicutes |
| Alloscardovia omnicolens  (HG3A.1279) | ODI | 0.026 | 0.136 | 0.213 | 3364 unclassified | Alloscardovia omnicolens | Alloscardovia | Bifidobacteriaceae | Bifidobacteriales | Actinomycetia | Actinobacteria |
| Latilactobacillus sakei subsp. sakei (HG3A.0836) | ODI | 0.026 | 0.136 | 0.213 | Latilactobacill 3364 us sakei  subsp. sakei | Latilactobacillus sakei | Latilactobacillus | Lactobacillaceae | Lactobacillales | Bacilli | Firmicutes |
| Catenibacterium mitsuokai (HG3A.0775) | ODI | 0.026 | 0.137 | 0.214 | 3364 unclassified | Catenibacterium mitsuokai | Catenibacterium | Coprobacillaceae | Erysipelotrichales | Erysipelotrichia | Firmicutes |
| Erysipelotrichaceae sp. (HG3A.0867) | ODI | 0.026 | 0.138 | 0.214 | 3364 unclassified | unclassified | unclassified | Erysipelotrichaceae | Erysipelotrichales | Erysipelotrichia | Firmicutes |
| Eubacteriales sp.  (HG3A.0600) | AHI | -0.031 | 0.091 | 0.214 | 3004 unclassified | unclassified | unclassified | unclassified | Eubacteriales | Clostridia | Firmicutes |
| Eubacteriales sp. (HG3A.0624) | AHI | -0.031 | 0.091 | 0.214 | 3004 unclassified | unclassified | unclassified | unclassified | Eubacteriales | Clostridia | Firmicutes |
| Lachnospiraceae sp.  (HG3A.0399) | AHI | -0.031 | 0.091 | 0.214 | 3004 unclassified | unclassified | unclassified | Lachnospiraceae | Eubacteriales | Clostridia | Firmicutes |
| Eubacteriales sp. (HG3A.0263) | AHI | -0.031 | 0.092 | 0.215 | 3004 unclassified | unclassified | unclassified | unclassified | Eubacteriales | Clostridia | Firmicutes |
| Eubacteriales sp.  (HG3A.0518) | AHI | -0.031 | 0.092 | 0.215 | 3004 unclassified | unclassified | unclassified | unclassified | Eubacteriales | Clostridia | Firmicutes |
| Eubacteriales sp. (HG3A.0580) | ODI | -0.026 | 0.139 | 0.215 | 3364 unclassified | unclassified | unclassified | unclassified | Eubacteriales | Clostridia | Firmicutes |
| Eubacteriales sp.  (HG3A.0887) | AHI | -0.031 | 0.092 | 0.215 | 3004 unclassified | unclassified | unclassified | unclassified | Eubacteriales | Clostridia | Firmicutes |
| Clostridia sp. (HG3A.1254) | ODI | -0.026 | 0.14 | 0.216 | 3364 unclassified | unclassified | unclassified | unclassified | unclassified | Clostridia | Firmicutes |

Eggerthellales sp. (HG3A.0174)

ODI -0.026 0.14 0.216 3364 unclassified unclassified unclassified unclassified Eggerthellales Coriobacteriia Actinobacteria

| Eubacteriales sp.  (HG3A.1177) | T90 | -0.028 | 0.108 | 0.216 | 3364 unclassified | unclassified | unclassified | unclassified | Eubacteriales | Clostridia | Firmicutes |
| --- | --- | --- | --- | --- | --- | --- | --- | --- | --- | --- | --- |
| Firmicutes sp.  (HG3A.1162) | T90 | -0.028 | 0.108 | 0.216 | 3364 unclassified | unclassified | unclassified | unclassified | unclassified | unclassified | Firmicutes |
| Oscillospiraceae sp. (HG3A.0774) | ODI | -0.026 | 0.14 | 0.216 | 3364 unclassified | unclassified | unclassified | Oscillospiraceae | Eubacteriales | Clostridia | Firmicutes |
| Peptostreptococcaceae  sp. (HG3A.0200) | ODI | -0.026 | 0.141 | 0.216 | 3364 unclassified | unclassified | unclassified | Peptostreptococcacea  e | Eubacteriales | Clostridia | Firmicutes |
| Traorella massiliensis (HG3A.0669) | ODI | -0.026 | 0.14 | 0.216 | 3364 unclassified | Traorella massiliensis | Traorella | Erysipelotrichaceae | Erysipelotrichales | Erysipelotrichia | Firmicutes |
| Allisonella histaminiformans  (HG3A.0332) | T90 | 0.028 | 0.109 | 0.217 | 3364 unclassified | Allisonella histaminiformans | Allisonella | Veillonellaceae | Veillonellales | Negativicutes | Firmicutes |
| Eubacteriales sp. (HG3A.0427) | T90 | -0.028 | 0.109 | 0.217 | 3364 unclassified | unclassified | unclassified | unclassified | Eubacteriales | Clostridia | Firmicutes |
| Eubacteriales sp.  (HG3A.0873) | ODI | -0.026 | 0.142 | 0.217 | 3364 unclassified | unclassified | unclassified | unclassified | Eubacteriales | Clostridia | Firmicutes |
| Peptostreptococcaceae sp. (HG3A.0200) | T90 | -0.028 | 0.108 | 0.217 | 3364 unclassified | unclassified | unclassified | Peptostreptococcacea e | Eubacteriales | Clostridia | Firmicutes |
| Eubacteriales sp.  (HG3A.0696) | T90 | -0.028 | 0.11 | 0.219 | 3364 unclassified | unclassified | unclassified | unclassified | Eubacteriales | Clostridia | Firmicutes |
| Firmicutes sp. (HG3A.1345) | T90 | -0.028 | 0.11 | 0.219 | 3364 unclassified | unclassified | unclassified | unclassified | unclassified | unclassified | Firmicutes |
| Bacteroidales sp.  (HG3A.1002) | T90 | -0.028 | 0.112 | 0.22 | 3364 unclassified | unclassified | unclassified | unclassified | Bacteroidales | Bacteroidia | Bacteroidetes |
| Butyrivibrio crossotus (HG3A.0413) | T90 | -0.028 | 0.112 | 0.22 | 3364 unclassified | Butyrivibrio crossotus | Butyrivibrio | Lachnospiraceae | Eubacteriales | Clostridia | Firmicutes |
| Clostridia sp.  (HG3A.0564) | AHI | -0.031 | 0.095 | 0.22 | 3004 unclassified | unclassified | unclassified | unclassified | unclassified | Clostridia | Firmicutes |
| Clostridia sp. (HG3A.0879) | AHI | -0.031 | 0.095 | 0.22 | 3004 unclassified | unclassified | unclassified | unclassified | unclassified | Clostridia | Firmicutes |
| Eubacteriales sp.  (HG3A.0406) | T90 | -0.028 | 0.111 | 0.22 | 3364 unclassified | unclassified | unclassified | unclassified | Eubacteriales | Clostridia | Firmicutes |
| Eubacteriales sp. (HG3A.0543) | ODI | -0.025 | 0.144 | 0.22 | 3364 unclassified | unclassified | unclassified | unclassified | Eubacteriales | Clostridia | Firmicutes |
| Intestinimonas massiliensis  (HG3A.0198) | T90 | -0.028 | 0.112 | 0.22 | 3364 unclassified | Intestinimonas massiliensis | Intestinimonas | unclassified | Eubacteriales | Clostridia | Firmicutes |
| Eubacteriales sp. (HG3A.0652) | ODI | -0.025 | 0.145 | 0.221 | 3364 unclassified | unclassified | unclassified | unclassified | Eubacteriales | Clostridia | Firmicutes |

Butyricicoccus sp. (HG3A.0008)

AHI 0.031 0.097 0.222 3004 unclassified unclassified Butyricicoccus Clostridiaceae Eubacteriales Clostridia Firmicutes

| Eubacteriales sp.  (HG3A.1154) | AHI | -0.031 | 0.097 | 0.222 | 3004 unclassified | unclassified | unclassified | unclassified | Eubacteriales | Clostridia | Firmicutes |
| --- | --- | --- | --- | --- | --- | --- | --- | --- | --- | --- | --- |
| Firmicutes sp.  (HG3A.0874) | AHI | -0.031 | 0.096 | 0.222 | 3004 unclassified | unclassified | unclassified | unclassified | unclassified | unclassified | Firmicutes |
| Limosilactobacillus vaginalis (HG3A.1341) | T90 | 0.028 | 0.113 | 0.222 | 3364 unclassified | Limosilactobacillus vaginalis | Limosilactobacill us | Lactobacillaceae | Lactobacillales | Bacilli | Firmicutes |
| Prevotella sp.  (HG3A.1040) | AHI | 0.031 | 0.097 | 0.222 | 3004 unclassified | unclassified | Prevotella | Prevotellaceae | Bacteroidales | Bacteroidia | Bacteroidetes |
| Sutterella sp. KLE1602 (HG3A.0228) | AHI | 0.031 | 0.097 | 0.222 | 3004 unclassified | Sutterella sp. KLE1602 | Sutterella | Sutterellaceae | Burkholderiales | Betaproteobacteri a | Proteobacteria |
| Barnesiella intestinihominis  (HG3A.0055) | AHI | -0.03 | 0.098 | 0.223 | 3004 unclassified | Barnesiella intestinihominis | Barnesiella | Barnesiellaceae | Bacteroidales | Bacteroidia | Bacteroidetes |
| [Clostridium] spiroforme (HG3A.0259) | AHI | 0.03 | 0.099 | 0.223 | 3004 unclassified | [Clostridium] spiroforme | Erysipelatoclostri dium | Erysipelotrichaceae | Erysipelotrichales | Erysipelotrichia | Firmicutes |
| Desulfovibrionales sp.  (HG3A.0727) | ODI | 0.025 | 0.146 | 0.223 | 3364 unclassified | unclassified | unclassified | unclassified | Desulfovibrionales | Deltaproteobacte  ria | Proteobacteria |
| Eggerthellales sp. (HG3A.0174) | AHI | -0.03 | 0.098 | 0.223 | 3004 unclassified | unclassified | unclassified | unclassified | Eggerthellales | Coriobacteriia | Actinobacteria |
| Clostridiaceae sp.  (HG3A.0330) | T90 | -0.027 | 0.115 | 0.224 | 3364 unclassified | unclassified | unclassified | Clostridiaceae | Eubacteriales | Clostridia | Firmicutes |
| Eubacteriales sp. (HG3A.0701) | ODI | -0.025 | 0.147 | 0.224 | 3364 unclassified | unclassified | unclassified | unclassified | Eubacteriales | Clostridia | Firmicutes |
| Gemella morbillorum (HG3A.1782) | AHI | 0.03 | 0.099 | 0.224 | 3004 unclassified | Gemella morbillorum | Gemella | unclassified | Bacillales | Bacilli | Firmicutes |
| Bacteria sp. (HG3A.1096) | ODI | -0.025 | 0.149 | 0.225 | 3364 unclassified | unclassified | unclassified | unclassified | unclassified | unclassified | unclassified |
| Eubacteriales sp.  (HG3A.0184) | ODI | -0.025 | 0.148 | 0.225 | 3364 unclassified | unclassified | unclassified | unclassified | Eubacteriales | Clostridia | Firmicutes |
| Eubacteriales sp. (HG3A.0671) | T90 | -0.027 | 0.116 | 0.225 | 3364 unclassified | unclassified | unclassified | unclassified | Eubacteriales | Clostridia | Firmicutes |
| Eubacteriales sp.  (HG3A.0405) | ODI | -0.025 | 0.15 | 0.226 | 3364 unclassified | unclassified | unclassified | unclassified | Eubacteriales | Clostridia | Firmicutes |
| Eubacteriales sp. (HG3A.0540) | T90 | -0.027 | 0.116 | 0.226 | 3364 unclassified | unclassified | unclassified | unclassified | Eubacteriales | Clostridia | Firmicutes |
| Eubacteriales sp.  (HG3A.0781) | ODI | -0.025 | 0.15 | 0.226 | 3364 unclassified | unclassified | unclassified | unclassified | Eubacteriales | Clostridia | Firmicutes |
| Eubacteriales sp. (HG3A.1154) | ODI | -0.025 | 0.149 | 0.226 | 3364 unclassified | unclassified | unclassified | unclassified | Eubacteriales | Clostridia | Firmicutes |

| Anaerotignum  lactatifermentans | AHI | 0.03 | 0.102 | 0.227 | Anaerotignum  3004 unclassified lactatifermentans Anaerotignum Lachnospiraceae Eubacteriales Clostridia Firmicutes | | | | | |
| --- | --- | --- | --- | --- | --- | --- | --- | --- | --- | --- |
| (HG3A.0676) |  |  |  |  |  | | | | | |
| Candidatus Borkfalkiales sp. | AHI | -0.03 | 0.103 | 0.227 | Candidatus  3004 unclassified unclassified unclassified unclassified Borkfalkiales Clostridia Firmicutes | | | | | |
| (HG3A.1284) |  |  |  |  |  | | | | | |
| Clostridia sp.  (HG3A.0815) | AHI | -0.03 | 0.102 | 0.227 | 3004 unclassified | unclassified | unclassified | unclassified | unclassified | Clostridia Firmicutes |
| Clostridia sp. (HG3A.1375) | AHI | -0.03 | 0.103 | 0.227 | 3004 unclassified | unclassified | unclassified | unclassified | unclassified | Clostridia Firmicutes |
| Clostridia sp.  (HG3A.1452) | AHI | -0.03 | 0.103 | 0.227 | 3004 unclassified | unclassified | unclassified | unclassified | unclassified | Clostridia Firmicutes |
| Eubacteriales sp. (HG3A.0482) | AHI | -0.03 | 0.102 | 0.227 | 3004 unclassified | unclassified | unclassified | unclassified | Eubacteriales | Clostridia Firmicutes |
| Eubacteriales sp.  (HG3A.0670) | AHI | -0.03 | 0.102 | 0.227 | 3004 unclassified | unclassified | unclassified | unclassified | Eubacteriales | Clostridia Firmicutes |
| Firmicutes sp. (HG3A.0526) | AHI | -0.03 | 0.104 | 0.227 | 3004 unclassified | unclassified | unclassified | unclassified | unclassified | unclassified Firmicutes |
| Oscillospiraceae sp.  (HG3A.0475) | AHI | -0.03 | 0.102 | 0.227 | 3004 unclassified | unclassified | unclassified | Oscillospiraceae | Eubacteriales | Clostridia Firmicutes |
| Oxalobacter sp. (HG3A.1218) | AHI | -0.03 | 0.103 | 0.227 | 3004 unclassified | unclassified | Oxalobacter | Oxalobacteraceae | Burkholderiales | Betaproteobacteri Proteobacteria a |
| Clostridia sp.  (HG3A.0463) | AHI | -0.03 | 0.105 | 0.228 | 3004 unclassified | unclassified | unclassified | unclassified | unclassified | Clostridia Firmicutes |
| Clostridia sp. (HG3A.0885) | AHI | -0.03 | 0.105 | 0.228 | 3004 unclassified | unclassified | unclassified | unclassified | unclassified | Clostridia Firmicutes |
| Eubacteriales sp.  (HG3A.0234) | AHI | -0.03 | 0.104 | 0.228 | 3004 unclassified | unclassified | unclassified | unclassified | Eubacteriales | Clostridia Firmicutes |
| Eubacteriales sp. (HG3A.0939) | AHI | -0.03 | 0.104 | 0.228 | 3004 unclassified | unclassified | unclassified | unclassified | Eubacteriales | Clostridia Firmicutes |
| Rothia mucilaginosa  (HG3A.0559) | AHI | 0.03 | 0.105 | 0.228 | 3004 unclassified | Rothia mucilaginosa | Rothia | Micrococcaceae | Micrococcales | Actinomycetia Actinobacteria |
| Clostridia sp. (HG3A.1062) | T90 | -0.027 | 0.119 | 0.229 | 3364 unclassified | unclassified | unclassified | unclassified | unclassified | Clostridia Firmicutes |
| Clostridia sp.  (HG3A.1262) | T90 | -0.027 | 0.119 | 0.229 | 3364 unclassified | unclassified | unclassified | unclassified | unclassified | Clostridia Firmicutes |
| Erysipelotrichaceae sp. (HG3A.0867) | T90 | 0.027 | 0.12 | 0.229 | 3364 unclassified | unclassified | unclassified | Erysipelotrichaceae | Erysipelotrichales | Erysipelotrichia Firmicutes |
| Eubacteriales sp.  (HG3A.0924) | T90 | -0.027 | 0.12 | 0.229 | 3364 unclassified | unclassified | unclassified | unclassified | Eubacteriales | Clostridia Firmicutes |
| Eubacteriales sp. (HG3A.1063) | ODI | -0.025 | 0.152 | 0.229 | 3364 unclassified | unclassified | unclassified | unclassified | Eubacteriales | Clostridia Firmicutes |

Mesosutterella multiformis (HG3A.0520)

T90 -0.027 0.119 0.229 3364 unclassified

Mesosutterella

multiformis Mesosutterella Sutterellaceae Burkholderiales

Betaproteobacteri

a Proteobacteria

Bacteroides caccae (HG3A.0066)

Enterocloster clostridioformis (HG3A.0686)

AHI -0.03 0.106 0.23 3004 unclassified Bacteroides caccae Bacteroides Bacteroidaceae Bacteroidales Bacteroidia Bacteroidetes

Enterocloster

ODI 0.025 0.153 0.23 3364 unclassified clostridioformis Enterocloster Lachnospiraceae Eubacteriales Clostridia Firmicutes

Eubacteriales sp. (HG3A.0791)

Clostridia sp. (HG3A.0564)

Clostridia sp. (HG3A.1252)

Desulfovibrionales sp. (HG3A.0266)

Erysipelatoclostridium

Erysipelatoclostridium Erysipelatoclostri

i

AHI -0.03 0.107 0.231 3004 unclassified unclassified unclassified unclassified Eubacteriales Clostridia Firmicutes

ODI -0.025 0.156 0.232 3364 unclassified unclassified unclassified unclassified unclassified Clostridia Firmicutes

ODI -0.025 0.156 0.232 3364 unclassified unclassified unclassified unclassified unclassified Clostridia Firmicutes

ODI -0.025 0.156 0.232 3364 unclassified unclassified unclassified unclassified Desulfovibrionales Deltaproteobacte Proteobacteria

ria

# (HG3A.0308)

| ramosum (HG3A.0538) ODI  Eubacteriales sp. AHI | 0.025  -0.03 | 0.155  0.108 | 0.232  0.232 | 3364 unclassified  3004 unclassified | ramosum  unclassified | dium  unclassified | Erysipelotrichaceae  unclassified | Erysipelotrichale  Eubacteriales | s Erysipelotrich  Clostridia | a Firmicutes  Firmicutes |
| --- | --- | --- | --- | --- | --- | --- | --- | --- | --- | --- |
| Oscillospiraceae sp. T90 | -0.027 | 0.121 | 0.232 | 3364 unclassified | unclassified | unclassified | Oscillospiraceae | Eubacteriales | Clostridia | Firmicutes |
| Oxalobacter formigenes Betaproteobacteri | | | | | | | | | | |

(HG3A.1270)

(HG3A.0552) ODI -0.025 0.156 0.232 3364 unclassified Oxalobacter formigenes Oxalobacter Oxalobacteraceae Burkholderiales

a Proteobacteria

Oxalobacter formigenes

(HG3A.1755)

T90

-0.027

0.122

0.232 3364 unclassified Oxalobacter formigenes Oxalobacter

Oxalobacteraceae Burkholderiales

Betaproteobacteri

a

Proteobacteria

Oxalobacter sp.

# (HG3A.1218)

ODI -0.025 0.155 0.232 3364 unclassified unclassified Oxalobacter Oxalobacteraceae Burkholderiales Betaproteobacteri Proteobacteria

a

# (HG3A.0525) (HG3A.1535) (HG3A.0828)

| Victivallis lenta T90 | -0.027 | 0.122 | 0.232 | 3364 unclassified | Victivallis lenta | Victivallis | Victivallaceae | Victivallales | Lentisphaeria | Lentisphaerae |
| --- | --- | --- | --- | --- | --- | --- | --- | --- | --- | --- |
| Alistipes sp. An66 T90 | -0.027 | 0.123 | 0.233 | 3364 unclassified | Alistipes sp. An66 | Alistipes | Rikenellaceae | Bacteroidales | Bacteroidia | Bacteroidetes |
| Clostridia sp. ODI | -0.025 | 0.158 | 0.233 | 3364 unclassified | unclassified | unclassified | unclassified | unclassified | Clostridia | Firmicutes |
| Anaeromassilibacillus Anaeromassilibacillus sp. Anaeromassiliba | | | | | | | | | | |
| sp. An250 (HG3A.0169) ODI | -0.025 | 0.158 | 0.234 | 3364 unclassified | An250 | cillus | Oscillospiraceae | Eubacteriales | Clostridia | Firmicutes |
| Firmicutes sp. ODI | -0.025 | 0.159 | 0.234 | 3364 unclassified | unclassified | unclassified | unclassified | unclassified | unclassified | Firmicutes |
| Firmicutes sp. T90 | -0.027 | 0.123 | 0.234 | 3364 unclassified | unclassified | unclassified | unclassified | unclassified | unclassified | Firmicutes |

(HG3A.0915) (HG3A.1195)

er

| Clostridia sp.  (HG3A.1375) | T90 | -0.027 | 0.124 | 0.235 | 3364 unclassified | unclassified | unclassified unclassified | unclassified | Clostridia | Firmicutes |
| --- | --- | --- | --- | --- | --- | --- | --- | --- | --- | --- |
| Eubacteriales sp.  (HG3A.0270) | T90 | -0.027 | 0.125 | 0.235 | 3364 unclassified | unclassified | unclassified unclassified | Eubacteriales | Clostridia | Firmicutes |
| Eubacteriales sp. (HG3A.0282) | T90 | -0.027 | 0.124 | 0.235 | 3364 unclassified | unclassified | unclassified unclassified | Eubacteriales | Clostridia | Firmicutes |
| Lachnospiraceae sp.  (HG3A.0257) | T90 | -0.027 | 0.125 | 0.235 | 3364 unclassified | unclassified | unclassified Lachnospiraceae | Eubacteriales | Clostridia | Firmicutes |
| Lachnospiraceae sp. (HG3A.0899) | AHI | -0.029 | 0.11 | 0.235 | 3004 unclassified | unclassified | unclassified Lachnospiraceae | Eubacteriales | Clostridia | Firmicutes |
| Alloscardovia omnicolens  (HG3A.1279) | AHI | 0.029 | 0.111 | 0.236 | 3004 unclassified | Alloscardovia omnicolens | Alloscardovia Bifidobacteriaceae | Bifidobacteriales | Actinomycetia | Actinobacteria |
| Clostridia sp. (HG3A.0661) | ODI | -0.024 | 0.16 | 0.236 | 3364 unclassified | unclassified | unclassified unclassified | unclassified | Clostridia | Firmicutes |
| Clostridia sp.  (HG3A.1111) | T90 | -0.027 | 0.127 | 0.236 | 3364 unclassified | unclassified | unclassified unclassified | unclassified | Clostridia | Firmicutes |
| Eubacteriales sp. (HG3A.0715) | AHI | -0.029 | 0.111 | 0.236 | 3004 unclassified | unclassified | unclassified unclassified | Eubacteriales | Clostridia | Firmicutes |
| Eubacteriales sp.  (HG3A.0859) | T90 | -0.027 | 0.128 | 0.236 | 3364 unclassified | unclassified | unclassified unclassified | Eubacteriales | Clostridia | Firmicutes |
| Firmicutes sp. (HG3A.0541) | T90 | -0.027 | 0.128 | 0.236 | 3364 unclassified | unclassified | unclassified unclassified | unclassified | unclassified | Firmicutes |
| Firmicutes sp.  (HG3A.0596) | T90 | -0.027 | 0.127 | 0.236 | 3364 unclassified | unclassified | unclassified unclassified | unclassified | unclassified | Firmicutes |
| Methanobrevibacter smithii (HG3A.0152) | T90 | -0.027 | 0.126 | 0.236 | 3364 unclassified | Methanobrevibacter smithii | Methanobrevibact Methanobacteriaceae | Methanobacteriales | Methanobacteria | Euryarchaeota |
| Bifidobacterium animalis subsp. lactis  (HG3A.0513) | T90 | -0.026 | 0.128 | 0.237 | Bifidobacteriu 3364 m animalis  subsp. lactis | Bifidobacterium animalis | Bifidobacterium Bifidobacteriaceae | Bifidobacteriales | Actinomycetia | Actinobacteria |
| Eubacteriales sp. (HG3A.0392) | ODI | -0.024 | 0.162 | 0.238 | 3364 unclassified | unclassified | unclassified unclassified | Eubacteriales | Clostridia | Firmicutes |
| Eubacteriales sp.  (HG3A.0184) | AHI | -0.029 | 0.113 | 0.239 | 3004 unclassified | unclassified | unclassified unclassified | Eubacteriales | Clostridia | Firmicutes |
| Eubacteriales sp. (HG3A.0334) | AHI | -0.029 | 0.113 | 0.239 | 3004 unclassified | unclassified | unclassified unclassified | Eubacteriales | Clostridia | Firmicutes |
| Eubacteriales sp.  (HG3A.0935) | AHI | -0.029 | 0.113 | 0.239 | 3004 unclassified | unclassified | unclassified unclassified | Eubacteriales | Clostridia | Firmicutes |
| Eubacteriales sp. (HG3A.1067) | T90 | -0.026 | 0.13 | 0.239 | 3364 unclassified | unclassified | unclassified unclassified | Eubacteriales | Clostridia | Firmicutes |

Eubacteriales sp. (HG3A.1167)

AHI -0.029 0.113 0.239 3004 unclassified unclassified unclassified unclassified Eubacteriales Clostridia Firmicutes

| Erysipelatoclostridium ramosum (HG3A.0538) | AHI | 0.029 | 0.115 | 0.242 | 3004 unclassified | Erysipelatoclostridium ramosum | Erysipelatoclostri dium | Erysipelotrichaceae | Erysipelotrichales | Erysipelotrichia | Firmicutes |
| --- | --- | --- | --- | --- | --- | --- | --- | --- | --- | --- | --- |
| Eubacteriales sp.  (HG3A.0908) | AHI | -0.029 | 0.115 | 0.242 | 3004 unclassified | unclassified | unclassified | unclassified | Eubacteriales | Clostridia | Firmicutes |
| Eubacteriales sp. (HG3A.0935) | ODI | -0.024 | 0.165 | 0.243 | 3364 unclassified | unclassified | unclassified | unclassified | Eubacteriales | Clostridia | Firmicutes |
| Eubacteriales sp.  (HG3A.0137) | ODI | -0.024 | 0.167 | 0.244 | 3364 unclassified | unclassified | unclassified | unclassified | Eubacteriales | Clostridia | Firmicutes |
| Eubacteriales sp. (HG3A.0406) | ODI | -0.024 | 0.167 | 0.244 | 3364 unclassified | unclassified | unclassified | unclassified | Eubacteriales | Clostridia | Firmicutes |
| Clostridia sp.  (HG3A.1062) | AHI | -0.029 | 0.118 | 0.246 | 3004 unclassified | unclassified | unclassified | unclassified | unclassified | Clostridia | Firmicutes |
| Oscillospiraceae sp. (HG3A.0507) | AHI | -0.029 | 0.118 | 0.246 | 3004 unclassified | unclassified | unclassified | Oscillospiraceae | Eubacteriales | Clostridia | Firmicutes |
| Parabacteroides goldsteinii (HG3A.0279) | ODI | -0.024 | 0.169 | 0.246 | 3364 unclassified | Parabacteroides goldsteinii | Parabacteroides | Tannerellaceae | Bacteroidales | Bacteroidia | Bacteroidetes |
| Anaerostipes hadrus (HG3A.0003) | ODI | 0.024 | 0.17 | 0.247 | 3364 unclassified | Anaerostipes hadrus | Anaerostipes | Lachnospiraceae | Eubacteriales | Clostridia | Firmicutes |
| Eubacteriales sp.  (HG3A.0751) | ODI | -0.024 | 0.17 | 0.247 | 3364 unclassified | unclassified | unclassified | unclassified | Eubacteriales | Clostridia | Firmicutes |
| Hydrogeniiclostidium mannosilyticum (HG3A.0294) | ODI | 0.024 | 0.17 | 0.247 | 3364 unclassified | Hydrogeniiclostidium mannosilyticum | Hydrogeniiclostid ium | Oscillospiraceae | Eubacteriales | Clostridia | Firmicutes |
| Lachnotalea sp. AF33-28 (HG3A.0403) | AHI | -0.029 | 0.119 | 0.247 | 3004 unclassified | Lachnotalea sp. AF33-28 | Lachnotalea | Lachnospiraceae | Eubacteriales | Clostridia | Firmicutes |
| Clostridia sp. (HG3A.0845) | T90 | -0.026 | 0.135 | 0.248 | 3364 unclassified | unclassified | unclassified | unclassified | unclassified | Clostridia | Firmicutes |
| Clostridia sp.  (HG3A.0918) | ODI | -0.024 | 0.172 | 0.248 | 3364 unclassified | unclassified | unclassified | unclassified | unclassified | Clostridia | Firmicutes |
| Clostridium sp. AF34-13 (HG3A.0173) | AHI | -0.029 | 0.121 | 0.248 | 3004 unclassified | Clostridium sp. AF34-13 | Clostridium | Clostridiaceae | Eubacteriales | Clostridia | Firmicutes |
| Eubacteriales sp.  (HG3A.0116) | AHI | -0.029 | 0.121 | 0.248 | 3004 unclassified | unclassified | unclassified | unclassified | Eubacteriales | Clostridia | Firmicutes |
| Eubacteriales sp. (HG3A.0476) | ODI | -0.024 | 0.171 | 0.248 | 3364 unclassified | unclassified | unclassified | unclassified | Eubacteriales | Clostridia | Firmicutes |
| Eubacteriales sp.  (HG3A.0956) | AHI | -0.029 | 0.121 | 0.248 | 3004 unclassified | unclassified | unclassified | unclassified | Eubacteriales | Clostridia | Firmicutes |
| Eubacteriales sp. (HG3A.1292) | ODI | -0.024 | 0.172 | 0.248 | 3364 unclassified | unclassified | unclassified | unclassified | Eubacteriales | Clostridia | Firmicutes |

(HG3A.1473) (HG3A.1124)

| Eubacteriales sp. T90 | | -0.026 | 0.135 | 0.248 | 3364 unclassified | unclassified | unclassified | unclassified | Eubacteriales | Clostridia | Firmicutes |
| --- | --- | --- | --- | --- | --- | --- | --- | --- | --- | --- | --- |
| Firmicutes sp. AHI | | -0.029 | 0.121 | 0.248 | 3004 unclassified | unclassified | unclassified | unclassified | unclassified | unclassified | Firmicutes |
| Latilactobacillus sakei  subsp. sakei | AHI | 0.029 | 0.121 | 0.248 | Latilactobacill  3004 us sakei | Latilactobacillus sakei | Latilactobacillus | Lactobacillaceae | Lactobacillales | Bacilli | Firmicutes |
| (HG3A.0836) | |  |  |  | subsp. sakei |  |  |  |  | | |
| Succinatimonas hippei AHI (HG3A.1322) | | 0.029 | 0.12 | 0.248 | 3004 unclassified | Succinatimonas hippei | Succinatimonas | Succinivibrionaceae | Aeromonadales Gammaproteoba Proteobacteria  cteria | | |
| Eubacteriales sp. AHI | | -0.028 | 0.123 | 0.25 | 3004 unclassified | unclassified | unclassified | unclassified | Eubacteriales Clostridia Firmicutes | | |
| Flavonifractor sp. An10 ODI | | -0.024 | 0.174 | 0.25 | 3364 unclassified | Flavonifractor sp. An10 | Flavonifractor | Oscillospiraceae | Eubacteriales Clostridia Firmicutes | | |

(HG3A.0428)

(HG3A.0495)

Akkermansia sp. BIOML

Akkermansia sp. BIOML-

Verrucomicrobia Verrucomicrob

A59 (HG3A.0800) AHI -0.028 0.124 0.251 3004 unclassified A59 Akkermansia Akkermansiaceae Verrucomicrobiales e ia

Anaerotignum lactatifermentans

(HG3A.0676)

ODI

0.024

0.175

0.251 3364 unclassified

Anaerotignum

lactatifermentans

Anaerotignum Lachnospiraceae

Eubacteriales

Clostridia

Firmicutes

Clostridia sp.

# (HG3A.0996)

AHI -0.028 0.124 0.251 3004 unclassified unclassified unclassified unclassified unclassified Clostridia Firmicutes

Eubacteriales sp.

(HG3A.0186)

AHI

-0.028

0.124

0.251 3004 unclassified unclassified

unclassified

unclassified

Eubacteriales

Clostridia

Firmicutes

Eubacteriales sp.

# (HG3A.0496)

ODI -0.024 0.176 0.251 3364 unclassified unclassified unclassified unclassified Eubacteriales Clostridia Firmicutes

Eubacteriales sp.

(HG3A.0548)

AHI

-0.028

0.124

0.251 3004 unclassified unclassified

unclassified

unclassified

Eubacteriales

Clostridia

Firmicutes

Eubacteriales sp.

# (HG3A.0711)

ODI -0.024 0.175 0.251 3364 unclassified unclassified unclassified unclassified Eubacteriales Clostridia Firmicutes

Bacteria sp.

(HG3A.0839)

ODI

-0.023

0.178

0.254 3364 unclassified unclassified

unclassified

unclassified

unclassified

unclassified

unclassified

Clostridia sp.

# (HG3A.1128)

AHI -0.028 0.126 0.254 3004 unclassified unclassified unclassified unclassified unclassified Clostridia Firmicutes

Eubacteriales sp.

(HG3A.0151)

T90

-0.026

0.138

0.254 3364 unclassified unclassified

unclassified

unclassified

Eubacteriales

Clostridia

Firmicutes

Eubacteriales sp.

# (HG3A.0158)

AHI -0.028 0.126 0.254 3004 unclassified unclassified unclassified unclassified Eubacteriales Clostridia Firmicutes

Bacteroides intestinalis

(HG3A.0265)

ODI

-0.023

0.179

0.255 3364 unclassified Bacteroides intestinalis Bacteroides

Bacteroidaceae

Bacteroidales

Bacteroidia

Bacteroidetes

Clostridia sp.

# (HG3A.1157)

T90 -0.026 0.139 0.255 3364 unclassified unclassified unclassified unclassified unclassified Clostridia Firmicutes

Eubacteriales sp.

(HG3A.0345)

ODI

-0.023

0.178

0.255 3364 unclassified unclassified

unclassified

unclassified

Eubacteriales

Clostridia

Firmicutes

Clostridia sp. (HG3A.1157)

Eubacteriales sp.

(HG3A.0132)

T90

-0.026

0.14

0.256 3364 unclassified unclassified

unclassified

unclassified

Eubacteriales

Clostridia

Firmicutes

AHI -0.028 0.127 0.256 3004 unclassified unclassified unclassified unclassified unclassified Clostridia Firmicutes

Eubacteriales sp. (HG3A.0759)

Oscillospiraceae sp.

(HG3A.0388)

T90

-0.026

0.141

0.256 3364 unclassified unclassified

unclassified

Oscillospiraceae

Eubacteriales

Clostridia

Firmicutes

T90 -0.026 0.141 0.256 3364 unclassified unclassified unclassified unclassified Eubacteriales Clostridia Firmicutes

Eubacteriales sp. (HG3A.0670)

Alloscardovia omnicolens

(HG3A.1279)

T90

0.025

0.143

0.258 3364 unclassified Alloscardovia omnicolens Alloscardovia Bifidobacteriaceae Bifidobacteriales Actinomycetia Actinobacteria

ODI -0.023 0.181 0.257 3364 unclassified unclassified unclassified unclassified Eubacteriales Clostridia Firmicutes

Clostridia sp. (HG3A.0094)

Clostridia sp.

(HG3A.1108)

AHI

-0.028

0.129

0.258 3004 unclassified unclassified

unclassified

unclassified

unclassified

Clostridia

Firmicutes

T90 -0.026 0.142 0.258 3364 unclassified unclassified unclassified unclassified unclassified Clostridia Firmicutes

Eubacteriales sp. (HG3A.1078)

Firmicutes sp. (HG3A.1345)

Sutterella wadsworthensis (HG3A.0143)

Ruthenibacterium lactatiformans

(HG3A.0020)

AHI

0.028

0.13

0.259 3004 unclassified

Ruthenibacterium

lactatiformans

Ruthenibacterium Oscillospiraceae

Eubacteriales

Clostridia

Firmicutes

T90 -0.025 0.143 0.258 3364 unclassified unclassified unclassified unclassified Eubacteriales Clostridia Firmicutes

AHI -0.028 0.129 0.258 3004 unclassified unclassified unclassified unclassified unclassified unclassified Firmicutes

Betaproteobacteri

T90 -0.026 0.143 0.258 3364 unclassified Sutterella wadsworthensis Sutterella Sutterellaceae Burkholderiales a Proteobacteria

Clostridia sp. (HG3A.0385)

Coprococcus sp. OM04-

5BH (HG3A.1028)

T90

-0.025

0.147

0.261 3364 unclassified

Coprococcus sp. OM04-

5BH

Coprococcus Lachnospiraceae

Eubacteriales

Clostridia

Firmicutes

AHI -0.028 0.132 0.261 3004 unclassified unclassified unclassified unclassified unclassified Clostridia Firmicutes

Eubacteriales sp. (HG3A.0193)

Eubacteriales sp.

(HG3A.0376)

T90

-0.025

0.146

0.261 3364 unclassified unclassified

unclassified

unclassified

Eubacteriales

Clostridia

Firmicutes

T90 -0.025 0.146 0.261 3364 unclassified unclassified unclassified unclassified Eubacteriales Clostridia Firmicutes

Eubacteriales sp. (HG3A.0531)

Eubacteriales sp.

(HG3A.1393)

T90

-0.025

0.146

0.261 3364 unclassified unclassified

unclassified

unclassified

Eubacteriales

Clostridia

Firmicutes

AHI -0.028 0.132 0.261 3004 unclassified unclassified unclassified unclassified Eubacteriales Clostridia Firmicutes

Limosilactobacillus oris (HG3A.1462)

Eubacteriales sp.

(HG3A.0551)

ODI

-0.023

0.184

0.262 3364 unclassified unclassified

unclassified

unclassified

Eubacteriales

Clostridia

Firmicutes

T90 0.025 0.146 0.261 3364 unclassified Limosilactobacillus oris Limosilactobacill

us

Lactobacillaceae Lactobacillales Bacilli Firmicutes

Clostridia sp. (HG3A.0276)

| Eubacteriales sp. (HG3A.0754) | T90 | -0.025 | 0.148 | 0.263 | 3364 unclassified | unclassified | unclassified | unclassified | Eubacteriales | Clostridia | Firmicutes |
| --- | --- | --- | --- | --- | --- | --- | --- | --- | --- | --- | --- |
| Streptococcus anginosus (HG3A.0680) | ODI | 0.023 | 0.186 | 0.263 | 3364 unclassified | Streptococcus anginosus | Streptococcus | Streptococcaceae | Lactobacillales | Bacilli | Firmicutes |
| Eggerthellales sp. (HG3A.0848) | AHI | -0.028 | 0.134 | 0.264 | 3004 unclassified | unclassified | unclassified | unclassified | Eggerthellales | Coriobacteriia | Actinobacteria |
| Lachnospiraceae sp.  (HG3A.0393) | AHI | 0.028 | 0.134 | 0.264 | 3004 unclassified | unclassified | unclassified | Lachnospiraceae | Eubacteriales | Clostridia | Firmicutes |
| Anaerobutyricum hallii (HG3A.0112) | T90 | 0.025 | 0.15 | 0.265 | 3364 unclassified | Anaerobutyricum hallii | Anaerobutyricum | Lachnospiraceae | Eubacteriales | Clostridia | Firmicutes |
| Eubacteriales sp.  (HG3A.0950) | T90 | -0.025 | 0.15 | 0.265 | 3364 unclassified | unclassified | unclassified | unclassified | Eubacteriales | Clostridia | Firmicutes |
| Eubacteriales sp. (HG3A.0910) | AHI | -0.027 | 0.136 | 0.266 | 3004 unclassified | unclassified | unclassified | unclassified | Eubacteriales | Clostridia | Firmicutes |
| Eubacteriales sp.  (HG3A.1294) | AHI | -0.027 | 0.136 | 0.266 | 3004 unclassified | unclassified | unclassified | unclassified | Eubacteriales | Clostridia | Firmicutes |
| Eubacteriales sp. (HG3A.1305) | AHI | -0.028 | 0.135 | 0.266 | 3004 unclassified | unclassified | unclassified | unclassified | Eubacteriales | Clostridia | Firmicutes |
| Oscillospiraceae sp.  (HG3A.0382) | AHI | -0.027 | 0.136 | 0.266 | 3004 unclassified | unclassified | unclassified | Oscillospiraceae | Eubacteriales | Clostridia | Firmicutes |
| Bacteria sp. (HG3A.0459) | AHI | -0.027 | 0.138 | 0.267 | 3004 unclassified | unclassified | unclassified | unclassified | unclassified | unclassified | unclassified |
| Clostridiaceae sp.  (HG3A.0608) | AHI | -0.027 | 0.138 | 0.267 | 3004 unclassified | unclassified | unclassified | Clostridiaceae | Eubacteriales | Clostridia | Firmicutes |
| [Clostridium] innocuum (HG3A.0365) | AHI | 0.027 | 0.139 | 0.269 | 3004 unclassified | [Clostridium] innocuum | Erysipelatoclostri dium | Erysipelotrichaceae | Erysipelotrichales | Erysipelotrichia | Firmicutes |
| Erysipelotrichales sp.  (HG3A.0303) | AHI | -0.027 | 0.139 | 0.269 | 3004 unclassified | unclassified | unclassified | unclassified | Erysipelotrichales | Erysipelotrichia | Firmicutes |
| Eubacteriales sp. (HG3A.0350) | ODI | -0.023 | 0.191 | 0.269 | 3364 unclassified | unclassified | unclassified | unclassified | Eubacteriales | Clostridia | Firmicutes |
| Eubacteriales sp.  (HG3A.0857) | T90 | -0.025 | 0.153 | 0.269 | 3364 unclassified | unclassified | unclassified | unclassified | Eubacteriales | Clostridia | Firmicutes |
| Sutterella sp. KLE1602 (HG3A.0228) | ODI | 0.023 | 0.19 | 0.269 | 3364 unclassified | Sutterella sp. KLE1602 | Sutterella | Sutterellaceae | Burkholderiales | Betaproteobacteri a | Proteobacteria |
| Parasutterella excrementihominis  (HG3A.0159) | ODI | -0.023 | 0.192 | 0.27 | 3364 unclassified | Parasutterella excrementihominis | Parasutterella | Sutterellaceae | Burkholderiales | Betaproteobacteri a | Proteobacteria |
| Clostridia sp. (HG3A.0946) | AHI | -0.027 | 0.142 | 0.272 | 3004 unclassified | unclassified | unclassified | unclassified | unclassified | Clostridia | Firmicutes |

T90 -0.025 0.149 0.263 3364 unclassified unclassified unclassified unclassified unclassified Clostridia Firmicutes

Eubacteriales sp. (HG3A.0609)

| Eubacteriales sp. (HG3A.0976) | AHI | -0.027 | 0.141 | 0.272 | 3004 unclassified | unclassified | unclassified | unclassified | Eubacteriales | Clostridia | Firmicutes |
| --- | --- | --- | --- | --- | --- | --- | --- | --- | --- | --- | --- |
| Eubacteriales sp.  (HG3A.0902) | T90 | -0.025 | 0.156 | 0.273 | 3364 unclassified | unclassified | unclassified | unclassified | Eubacteriales | Clostridia | Firmicutes |
| Eubacteriales sp. (HG3A.1030) | T90 | -0.025 | 0.157 | 0.274 | 3364 unclassified | unclassified | unclassified | unclassified | Eubacteriales | Clostridia | Firmicutes |
| Mogibacterium kristiansenii  (HG3A.0522) | ODI | 0.023 | 0.195 | 0.274 | 3364 unclassified | Mogibacterium kristiansenii | Mogibacterium | Clostridiales Family  XIII. Incertae Sedis | Eubacteriales | Clostridia | Firmicutes |
| Proteobacteria sp. (HG3A.0360) | ODI | -0.023 | 0.196 | 0.275 | 3364 unclassified | unclassified | unclassified | unclassified | unclassified | unclassified | Proteobacteria |
| Clostridia sp.  (HG3A.1057) | AHI | -0.027 | 0.145 | 0.277 | 3004 unclassified | unclassified | unclassified | unclassified | unclassified | Clostridia | Firmicutes |
| Eubacteriales sp. (HG3A.0405) | AHI | -0.027 | 0.145 | 0.277 | 3004 unclassified | unclassified | unclassified | unclassified | Eubacteriales | Clostridia | Firmicutes |
| Firmicutes sp.  (HG3A.0948) | AHI | -0.027 | 0.145 | 0.277 | 3004 unclassified | unclassified | unclassified | unclassified | unclassified | unclassified | Firmicutes |
| Eubacteriales sp. (HG3A.0184) | T90 | -0.024 | 0.159 | 0.278 | 3364 unclassified | unclassified | unclassified | unclassified | Eubacteriales | Clostridia | Firmicutes |
| Eubacteriales sp.  (HG3A.0369) | ODI | -0.022 | 0.198 | 0.278 | 3364 unclassified | unclassified | unclassified | unclassified | Eubacteriales | Clostridia | Firmicutes |
| Eubacteriales sp. (HG3A.0498) | AHI | 0.027 | 0.146 | 0.278 | 3004 unclassified | unclassified | unclassified | unclassified | Eubacteriales | Clostridia | Firmicutes |
| Anaerotruncus massiliensis  (HG3A.0460) | ODI | -0.022 | 0.2 | 0.279 | 3364 unclassified | Anaerotruncus massiliensis | Anaerotruncus | Oscillospiraceae | Eubacteriales | Clostridia | Firmicutes |
| Eubacteriales sp. (HG3A.0881) | ODI | -0.022 | 0.2 | 0.279 | 3364 unclassified | unclassified | unclassified | unclassified | Eubacteriales | Clostridia | Firmicutes |
| Lachnospiraceae sp.  (HG3A.0831) | ODI | -0.022 | 0.2 | 0.279 | 3364 unclassified | unclassified | unclassified | Lachnospiraceae | Eubacteriales | Clostridia | Firmicutes |
| Eubacteriales sp. (HG3A.0985) | T90 | 0.024 | 0.161 | 0.28 | 3364 unclassified | unclassified | unclassified | unclassified | Eubacteriales | Clostridia | Firmicutes |
| Collinsella intestinalis (HG3A.0802) | T90 | 0.024 | 0.162 | 0.281 | 3364 unclassified | Collinsella intestinalis | Collinsella | Coriobacteriaceae | Coriobacteriales | Coriobacteriia | Actinobacteria |
| Firmicutes sp. (HG3A.1091) | T90 | -0.024 | 0.162 | 0.281 | 3364 unclassified | unclassified | unclassified | unclassified | unclassified | unclassified | Firmicutes |
| Limosilactobacillus vaginalis (HG3A.1341) | AHI | 0.027 | 0.148 | 0.281 | 3004 unclassified | Limosilactobacillus vaginalis | Limosilactobacill us | Lactobacillaceae | Lactobacillales | Bacilli | Firmicutes |
| Clostridia sp. (HG3A.0885) | T90 | -0.024 | 0.163 | 0.282 | 3364 unclassified | unclassified | unclassified | unclassified | unclassified | Clostridia | Firmicutes |

T90 -0.025 0.155 0.272 3364 unclassified unclassified unclassified unclassified Eubacteriales Clostridia Firmicutes

Hungatella hathewayi

(HG3A.0455) ODI 0.022 0.203 0.282 3364 unclassified Hungatella hathewayi Hungatella Clostridiaceae Eubacteriales Clostridia Firmicutes

Ruminococcus sp.

(HG3A.0126)

ODI

-0.022

0.203

0.282 3364 unclassified unclassified

Ruminococcus Oscillospiraceae

Eubacteriales

Clostridia

Firmicutes

Bacteroidales sp.

# (HG3A.1002)

AHI -0.027 0.15 0.284 3004 unclassified unclassified unclassified unclassified Bacteroidales Bacteroidia Bacteroidetes

Eubacteriales sp.

(HG3A.0618)

T90

-0.024

0.165

0.284 3364 unclassified unclassified

unclassified

unclassified

Eubacteriales

Clostridia

Firmicutes

Firmicutes sp.

# (HG3A.1162)

ODI -0.022 0.205 0.284 3364 unclassified unclassified unclassified unclassified unclassified unclassified Firmicutes

Ruthenibacterium lactatiformans

(HG3A.0020)

ODI

0.022

0.205

0.284 3364 unclassified

Ruthenibacterium

lactatiformans

Ruthenibacterium Oscillospiraceae

Eubacteriales

Clostridia

Firmicutes

Hydrogeniiclostidium

mannosilyticum (HG3A.0294)

Anaerostipes sp. BG01

(HG3A.1509)

T90

0.024

0.167

0.286 3364 unclassified Anaerostipes sp. BG01 Anaerostipes Lachnospiraceae

Eubacteriales

Clostridia

Firmicutes

T90 0.024 0.165 0.285 3364 unclassified

Hydrogeniiclostidium mannosilyticum

Hydrogeniiclostid

ium Oscillospiraceae Eubacteriales Clostridia Firmicutes

Clostridia sp. (HG3A.1137)

Bacteria sp.

(HG3A.0492)

ODI

-0.022

0.207

0.287 3364 unclassified unclassified

unclassified

unclassified

unclassified

unclassified

unclassified

T90 -0.024 0.166 0.286 3364 unclassified unclassified unclassified unclassified unclassified Clostridia Firmicutes

Lachnotalea sp. AF33-28

(HG3A.0403) ODI -0.022 0.208 0.287 3364 unclassified Lachnotalea sp. AF33-28 Lachnotalea Lachnospiraceae Eubacteriales Clostridia Firmicutes

Alistipes sp.

(HG3A.1385)

ODI

-0.022

0.209

0.288 3364 unclassified unclassified

Alistipes

Rikenellaceae

Bacteroidales

Bacteroidia

Bacteroidetes

Clostridium sp. OM07-

9AC (HG3A.0448) T90 -0.024 0.169 0.288 3364 unclassified

Clostridium sp. OM07-

9AC Clostridium Clostridiaceae Eubacteriales Clostridia Firmicutes

Eubacteriales sp.

(HG3A.0213)

AHI

-0.026

0.153

0.288 3004 unclassified unclassified

unclassified

unclassified

Eubacteriales

Clostridia

Firmicutes

Eubacteriales sp.

# (HG3A.0323)

T90 -0.024 0.17 0.288 3364 unclassified unclassified unclassified unclassified Eubacteriales Clostridia Firmicutes

Eubacteriales sp.

(HG3A.0405)

T90

-0.024

0.169

0.288 3364 unclassified unclassified

unclassified

unclassified

Eubacteriales

Clostridia

Firmicutes

Eubacteriales sp.

# (HG3A.0473)

T90 -0.024 0.17 0.288 3364 unclassified unclassified unclassified unclassified Eubacteriales Clostridia Firmicutes

Eubacteriales sp.

(HG3A.0643)

T90

-0.024

0.168

0.288 3364 unclassified unclassified

unclassified

unclassified

Eubacteriales

Clostridia

Firmicutes

Oscillospiraceae sp.

# (HG3A.0461)

AHI -0.026 0.153 0.288 3004 unclassified unclassified unclassified Oscillospiraceae Eubacteriales Clostridia Firmicutes

| Clostridia sp.  (HG3A.1192) | T90 | -0.024 | 0.171 | 0.289 | 3364 unclassified | unclassified | unclassified | unclassified | unclassified | Clostridia | Firmicutes |
| --- | --- | --- | --- | --- | --- | --- | --- | --- | --- | --- | --- |
| Lachnospiraceae sp.  (HG3A.0399) | T90 | -0.024 | 0.171 | 0.289 | 3364 unclassified | unclassified | unclassified | Lachnospiraceae | Eubacteriales | Clostridia | Firmicutes |
| Oscillospiraceae sp. (HG3A.1421) | T90 | 0.024 | 0.172 | 0.29 | 3364 unclassified | unclassified | unclassified | Oscillospiraceae | Eubacteriales | Clostridia | Firmicutes |
| Anaeroglobus geminatus (HG3A.1818) | T90 | 0.024 | 0.174 | 0.291 | 3364 unclassified | Anaeroglobus geminatus | Anaeroglobus | Veillonellaceae | Veillonellales | Negativicutes | Firmicutes |
| Clostridia sp. (HG3A.1486) | T90 | -0.024 | 0.174 | 0.291 | 3364 unclassified | unclassified | unclassified | unclassified | unclassified | Clostridia | Firmicutes |
| Eubacteriales sp.  (HG3A.0364) | T90 | -0.024 | 0.174 | 0.291 | 3364 unclassified | unclassified | unclassified | unclassified | Eubacteriales | Clostridia | Firmicutes |
| Eubacteriales sp. (HG3A.1109) | T90 | -0.024 | 0.174 | 0.291 | 3364 unclassified | unclassified | unclassified | unclassified | Eubacteriales | Clostridia | Firmicutes |
| Eubacteriales sp.  (HG3A.0457) | ODI | -0.022 | 0.212 | 0.292 | 3364 unclassified | unclassified | unclassified | unclassified | Eubacteriales | Clostridia | Firmicutes |
| Bacteria sp. (HG3A.0500) | AHI | -0.026 | 0.156 | 0.293 | 3004 unclassified | unclassified | unclassified | unclassified | unclassified | unclassified | unclassified |
| Eubacteriales sp.  (HG3A.0189) | T90 | -0.024 | 0.176 | 0.293 | 3364 unclassified | unclassified | unclassified | unclassified | Eubacteriales | Clostridia | Firmicutes |
| Eubacteriales sp. (HG3A.1094) | AHI | -0.026 | 0.156 | 0.293 | 3004 unclassified | unclassified | unclassified | unclassified | Eubacteriales | Clostridia | Firmicutes |
| Bacteria sp.  (HG3A.1545) | T90 | -0.023 | 0.177 | 0.294 | 3364 unclassified | unclassified | unclassified | unclassified | unclassified | unclassified | unclassified |
| Eubacteriales sp. (HG3A.0496) | T90 | -0.024 | 0.177 | 0.294 | 3364 unclassified | unclassified | unclassified | unclassified | Eubacteriales | Clostridia | Firmicutes |
| Eubacteriales sp.  (HG3A.0628) | T90 | -0.023 | 0.177 | 0.294 | 3364 unclassified | unclassified | unclassified | unclassified | Eubacteriales | Clostridia | Firmicutes |
| Eubacteriales sp. (HG3A.0670) | T90 | -0.023 | 0.178 | 0.294 | 3364 unclassified | unclassified | unclassified | unclassified | Eubacteriales | Clostridia | Firmicutes |
| Eubacteriales sp.  (HG3A.0204) | AHI | -0.026 | 0.158 | 0.295 | 3004 unclassified | unclassified | unclassified | unclassified | Eubacteriales | Clostridia | Firmicutes |
| Eubacteriales sp. (HG3A.0280) | T90 | -0.023 | 0.179 | 0.295 | 3364 unclassified | unclassified | unclassified | unclassified | Eubacteriales | Clostridia | Firmicutes |
| Eubacteriales sp.  (HG3A.0350) | AHI | -0.026 | 0.158 | 0.295 | 3004 unclassified | unclassified | unclassified | unclassified | Eubacteriales | Clostridia | Firmicutes |
| Lachnospiraceae sp. (HG3A.0903) | AHI | -0.026 | 0.158 | 0.295 | 3004 unclassified | unclassified | unclassified | Lachnospiraceae | Eubacteriales | Clostridia | Firmicutes |
| Bacteria sp.  (HG3A.0839) | AHI | -0.026 | 0.161 | 0.296 | 3004 unclassified | unclassified | unclassified | unclassified | unclassified | unclassified | unclassified |
| Erysipelotrichales sp. (HG3A.0809) | AHI | 0.026 | 0.161 | 0.296 | 3004 unclassified | unclassified | unclassified | unclassified | Erysipelotrichales | Erysipelotrichia | Firmicutes |

# (HG3A.0589) (HG3A.0711) (HG3A.0807)

Massilistercora timonensis

(HG3A.0458)

AHI

-0.026

0.16

0.296 3004 unclassified

Massilistercora

timonensis

Massilistercora unclassified

Eubacteriales

Clostridia

Firmicutes

| Eubacteriales sp. AHI | -0.026 | 0.161 | 0.296 | 3004 unclassified | unclassified | unclassified | unclassified | Eubacteriales | Clostridia | Firmicutes |
| --- | --- | --- | --- | --- | --- | --- | --- | --- | --- | --- |
| Eubacteriales sp. AHI | -0.026 | 0.16 | 0.296 | 3004 unclassified | unclassified | unclassified | unclassified | Eubacteriales | Clostridia | Firmicutes |
| Eubacteriales sp. AHI | -0.026 | 0.16 | 0.296 | 3004 unclassified | unclassified | unclassified | unclassified | Eubacteriales | Clostridia | Firmicutes |

Eubacteriales sp. (HG3A.0349)

Eubacteriales sp.

(HG3A.0428)

ODI

-0.022

0.216

0.297 3364 unclassified unclassified

unclassified

unclassified

Eubacteriales

Clostridia

Firmicutes

T90 -0.023 0.181 0.297 3364 unclassified unclassified unclassified unclassified Eubacteriales Clostridia Firmicutes

Eubacteriales sp. (HG3A.0498)

Eubacteriales sp.

(HG3A.1250)

AHI

-0.026

0.163

0.298 3004 unclassified unclassified

unclassified

unclassified

Eubacteriales

Clostridia

Firmicutes

T90 0.023 0.181 0.297 3364 unclassified unclassified unclassified unclassified Eubacteriales Clostridia Firmicutes

Clostridia sp. (HG3A.0933)

Eubacteriales sp.

(HG3A.0614)

ODI

-0.021

0.218

0.299 3364 unclassified unclassified

unclassified

unclassified

Eubacteriales

Clostridia

Firmicutes

T90 -0.023 0.183 0.299 3364 unclassified unclassified unclassified unclassified unclassified Clostridia Firmicutes

Eubacteriales sp. (HG3A.1129)

Eubacteriales sp.

(HG3A.0685)

AHI

-0.026

0.165

0.301 3004 unclassified unclassified

unclassified

unclassified

Eubacteriales

Clostridia

Firmicutes

AHI -0.026 0.164 0.299 3004 unclassified unclassified unclassified unclassified Eubacteriales Clostridia Firmicutes

Clostridium sp. AF34-13

(HG3A.0173) ODI -0.021 0.221 0.302 3364 unclassified Clostridium sp. AF34-13 Clostridium Clostridiaceae Eubacteriales Clostridia Firmicutes

Streptococcus gallolyticus subsp. gallolyticus

(HG3A.1651)

Streptococcus

ODI

0.021

0.22

0.302

3364 gallolyticus

subsp.

gallolyticus

Streptococcus gallolyticus Streptococcus Streptococcaceae Lactobacillales

Bacilli

Firmicutes

# (HG3A.0199)

| Butyricimonas virosa AHI | -0.025 | 0.168 | 0.304 | 3004 unclassified | Butyricimonas virosa | Butyricimonas | Odoribacteraceae | Bacteroidales | Bacteroidia | Bacteroidetes |
| --- | --- | --- | --- | --- | --- | --- | --- | --- | --- | --- |
| Clostridia sp. AHI | -0.025 | 0.168 | 0.304 | 3004 unclassified | unclassified | unclassified | unclassified | unclassified | Clostridia | Firmicutes |

(HG3A.1008)

Faecalibacterium

prausnitzii (HG3A.0241) T90 -0.023 0.186 0.304 3364 unclassified

Faecalibacterium

prausnitzii Faecalibacterium Oscillospiraceae Eubacteriales Clostridia Firmicutes

Coprococcus sp. AM27-

12LB (HG3A.0687)

T90

0.023

0.187

0.305 3364 unclassified

Coprococcus sp. AM27-

12LB

Coprococcus Lachnospiraceae

Eubacteriales

Clostridia

Firmicutes

Eubacteriales sp.

# (HG3A.0335)

T90 0.023 0.188 0.305 3364 unclassified unclassified unclassified unclassified Eubacteriales Clostridia Firmicutes

| Pediococcus acidilactici (HG3A.1468) | AHI | 0.025 | 0.168 | 0.305 | 3004 unclassified | Pediococcus acidilactici | Pediococcus | Lactobacillaceae | Lactobacillales | Bacilli | Firmicutes |
| --- | --- | --- | --- | --- | --- | --- | --- | --- | --- | --- | --- |
| Eubacterium sp. AF22- 8LB (HG3A.0838) | T90 | 0.023 | 0.189 | 0.306 | 3364 unclassified | Eubacterium sp. AF22- 8LB | Eubacterium | Eubacteriaceae | Eubacteriales | Clostridia | Firmicutes |
| Alistipes timonensis (HG3A.0586) | ODI | -0.021 | 0.225 | 0.307 | 3364 unclassified | Alistipes timonensis | Alistipes | Rikenellaceae | Bacteroidales | Bacteroidia | Bacteroidetes |
| Clostridia sp.  (HG3A.1452) | ODI | -0.021 | 0.225 | 0.307 | 3364 unclassified | unclassified | unclassified | unclassified | unclassified | Clostridia | Firmicutes |
| Clostridium sp. AF37-5 (HG3A.0076) | ODI | -0.021 | 0.226 | 0.307 | 3364 unclassified | Clostridium sp. AF37-5 | Clostridium | Clostridiaceae | Eubacteriales | Clostridia | Firmicutes |
| Dialister pneumosintes (HG3A.1496) | ODI | 0.021 | 0.226 | 0.307 | 3364 unclassified | Dialister pneumosintes | Dialister | Veillonellaceae | Veillonellales | Negativicutes | Firmicutes |
| Enterocloster citroniae (HG3A.0285) | AHI | 0.025 | 0.17 | 0.307 | 3004 unclassified | Enterocloster citroniae | Enterocloster | Lachnospiraceae | Eubacteriales | Clostridia | Firmicutes |
| Oscillospiraceae sp.  (HG3A.0380) | T90 | -0.023 | 0.19 | 0.308 | 3364 unclassified | unclassified | unclassified | Oscillospiraceae | Eubacteriales | Clostridia | Firmicutes |
| Allisonella histaminiformans (HG3A.0332) | AHI | 0.025 | 0.172 | 0.309 | 3004 unclassified | Allisonella histaminiformans | Allisonella | Veillonellaceae | Veillonellales | Negativicutes | Firmicutes |
| Eubacteriales sp.  (HG3A.0192) | ODI | -0.021 | 0.228 | 0.309 | 3364 unclassified | unclassified | unclassified | unclassified | Eubacteriales | Clostridia | Firmicutes |
| Eubacteriales sp. (HG3A.0320) | AHI | -0.025 | 0.173 | 0.309 | 3004 unclassified | unclassified | unclassified | unclassified | Eubacteriales | Clostridia | Firmicutes |
| Eubacteriales sp.  (HG3A.0759) | AHI | -0.025 | 0.172 | 0.309 | 3004 unclassified | unclassified | unclassified | unclassified | Eubacteriales | Clostridia | Firmicutes |
| Eubacteriales sp. (HG3A.0773) | AHI | -0.025 | 0.172 | 0.309 | 3004 unclassified | unclassified | unclassified | unclassified | Eubacteriales | Clostridia | Firmicutes |
| Eubacteriales sp.  (HG3A.1087) | AHI | -0.025 | 0.173 | 0.309 | 3004 unclassified | unclassified | unclassified | unclassified | Eubacteriales | Clostridia | Firmicutes |
| Alistipes dispar (HG3A.0281) | ODI | -0.021 | 0.229 | 0.31 | 3364 unclassified | Alistipes dispar | Alistipes | Rikenellaceae | Bacteroidales | Bacteroidia | Bacteroidetes |
| Firmicutes sp.  (HG3A.0817) | T90 | -0.023 | 0.193 | 0.311 | 3364 unclassified | unclassified | unclassified | unclassified | unclassified | unclassified | Firmicutes |
| Oscillospiraceae sp. (HG3A.0507) | T90 | -0.023 | 0.192 | 0.311 | 3364 unclassified | unclassified | unclassified | Oscillospiraceae | Eubacteriales | Clostridia | Firmicutes |
| Eubacteriales sp.  (HG3A.0432) | ODI | -0.021 | 0.232 | 0.313 | 3364 unclassified | unclassified | unclassified | unclassified | Eubacteriales | Clostridia | Firmicutes |
| Clostridia sp. (HG3A.0706) | T90 | -0.023 | 0.196 | 0.314 | 3364 unclassified | unclassified | unclassified | unclassified | unclassified | Clostridia | Firmicutes |

Eubacteriales sp. (HG3A.0328)

| Lachnospiraceae sp. (HG3A.0748) | T90 | -0.023 | 0.195 | 0.314 | 3364 unclassified | unclassified | unclassified | Lachnospiraceae | Eubacteriales | Clostridia Firmicutes |
| --- | --- | --- | --- | --- | --- | --- | --- | --- | --- | --- |
| Oscillospiraceae sp.  (HG3A.0576) | T90 | -0.023 | 0.195 | 0.314 | 3364 unclassified | unclassified | unclassified | Oscillospiraceae | Eubacteriales | Clostridia Firmicutes |
| Oscillospiraceae sp. (HG3A.0944) | T90 | -0.022 | 0.197 | 0.314 | 3364 unclassified | unclassified | unclassified | Oscillospiraceae | Eubacteriales | Clostridia Firmicutes |
| Blautia sp. AF19-10LB  (HG3A.0157) | ODI | -0.021 | 0.234 | 0.315 | 3364 unclassified | Blautia sp. AF19-10LB | Blautia | Lachnospiraceae | Eubacteriales | Clostridia Firmicutes |
| Eubacteriales sp. (HG3A.0323) | ODI | -0.021 | 0.235 | 0.315 | 3364 unclassified | unclassified | unclassified | unclassified | Eubacteriales | Clostridia Firmicutes |
| Lachnospiraceae sp.  (HG3A.1525) | T90 | -0.022 | 0.198 | 0.316 | 3364 unclassified | unclassified | unclassified | Lachnospiraceae | Eubacteriales | Clostridia Firmicutes |
| Lachnospiraceae sp. (HG3A.0903) | ODI | -0.021 | 0.236 | 0.317 | 3364 unclassified | unclassified | unclassified | Lachnospiraceae | Eubacteriales | Clostridia Firmicutes |
| Coprococcus catus  (HG3A.0037) | T90 | 0.022 | 0.2 | 0.318 | 3364 unclassified | Coprococcus catus | Coprococcus | Lachnospiraceae | Eubacteriales | Clostridia Firmicutes |
| Eubacteriales sp. (HG3A.0537) | AHI | -0.025 | 0.178 | 0.318 | 3004 unclassified | unclassified | unclassified | unclassified | Eubacteriales | Clostridia Firmicutes |
| Eubacteriales sp.  (HG3A.0760) | AHI | -0.025 | 0.179 | 0.318 | 3004 unclassified | unclassified | unclassified | unclassified | Eubacteriales | Clostridia Firmicutes |
| Eubacteriales sp. (HG3A.1102) | ODI | -0.021 | 0.238 | 0.318 | 3364 unclassified | unclassified | unclassified | unclassified | Eubacteriales | Clostridia Firmicutes |
| Amedibacillus dolichus (HG3A.0798) | T90 | 0.022 | 0.201 | 0.319 | 3364 unclassified | Amedibacillus dolichus | Amedibacillus | Erysipelotrichaceae | Erysipelotrichales | Erysipelotrichia Firmicutes |
| Blautia producta (HG3A.0905) | T90 | 0.022 | 0.202 | 0.319 | 3364 unclassified | Blautia producta | Blautia | Lachnospiraceae | Eubacteriales | Clostridia Firmicutes |
| Eubacteriales sp.  (HG3A.0578) | T90 | -0.022 | 0.201 | 0.319 | 3364 unclassified | unclassified | unclassified | unclassified | Eubacteriales | Clostridia Firmicutes |
| Oxalobacter sp. (HG3A.1097) | T90 | -0.022 | 0.202 | 0.319 | 3364 unclassified | unclassified | Oxalobacter | Oxalobacteraceae | Burkholderiales | Betaproteobacteri Proteobacteria a |
| Faecalibacterium prausnitzii (HG3A.0025) | AHI | 0.025 | 0.18 | 0.32 | 3004 unclassified | Faecalibacterium prausnitzii | Faecalibacterium | Oscillospiraceae | Eubacteriales | Clostridia Firmicutes |
| Firmicutes sp. (HG3A.0570) | T90 | -0.022 | 0.203 | 0.32 | 3364 unclassified | unclassified | unclassified | unclassified | unclassified | unclassified Firmicutes |
| Alistipes timonensis  (HG3A.0586) | AHI | -0.025 | 0.182 | 0.321 | 3004 unclassified | Alistipes timonensis | Alistipes | Rikenellaceae | Bacteroidales | Bacteroidia Bacteroidetes |
| Butyrivibrio crossotus (HG3A.0413) | ODI | -0.02 | 0.24 | 0.321 | 3364 unclassified | Butyrivibrio crossotus | Butyrivibrio | Lachnospiraceae | Eubacteriales | Clostridia Firmicutes |

T90 -0.022 0.196 0.314 3364 unclassified unclassified unclassified unclassified Eubacteriales Clostridia Firmicutes

Candidatus Borkfalkiales sp. (HG3A.1397)

| Eubacteriales sp. (HG3A.0854) | AHI | 0.025 | 0.183 | 0.321 | 3004 unclassified | unclassified | unclassified | unclassified | Eubacteriales | Clostridia | Firmicutes |
| --- | --- | --- | --- | --- | --- | --- | --- | --- | --- | --- | --- |
| Eubacteriales sp.  (HG3A.0902) | AHI | -0.025 | 0.182 | 0.321 | 3004 unclassified | unclassified | unclassified | unclassified | Eubacteriales | Clostridia | Firmicutes |
| Eubacteriales sp. (HG3A.1227) | AHI | -0.025 | 0.183 | 0.321 | 3004 unclassified | unclassified | unclassified | unclassified | Eubacteriales | Clostridia | Firmicutes |
| Hydrogeniiclostidium mannosilyticum  (HG3A.0294) | AHI | 0.024 | 0.184 | 0.321 | 3004 unclassified | Hydrogeniiclostidium mannosilyticum | Hydrogeniiclostid ium | Oscillospiraceae | Eubacteriales | Clostridia | Firmicutes |
| Lachnospiraceae sp. (HG3A.0172) | AHI | -0.024 | 0.184 | 0.321 | 3004 unclassified | unclassified | unclassified | Lachnospiraceae | Eubacteriales | Clostridia | Firmicutes |
| Oscillospiraceae sp.  (HG3A.0134) | AHI | -0.025 | 0.183 | 0.321 | 3004 unclassified | unclassified | unclassified | Oscillospiraceae | Eubacteriales | Clostridia | Firmicutes |
| Clostridia sp. (HG3A.0550) | AHI | -0.024 | 0.185 | 0.322 | 3004 unclassified | unclassified | unclassified | unclassified | unclassified | Clostridia | Firmicutes |
| Clostridia sp.  (HG3A.1486) | ODI | -0.02 | 0.242 | 0.323 | 3364 unclassified | unclassified | unclassified | unclassified | unclassified | Clostridia | Firmicutes |
| Eubacteriaceae sp. (HG3A.0591) | ODI | 0.02 | 0.243 | 0.323 | 3364 unclassified | unclassified | unclassified | Eubacteriaceae | Eubacteriales | Clostridia | Firmicutes |
| Roseburia sp. AM16-25  (HG3A.0344) | ODI | -0.02 | 0.242 | 0.323 | 3364 unclassified | Roseburia sp. AM16-25 | Roseburia | Lachnospiraceae | Eubacteriales | Clostridia | Firmicutes |
| Eubacteriales sp. (HG3A.0557) | T90 | -0.022 | 0.206 | 0.324 | 3364 unclassified | unclassified | unclassified | unclassified | Eubacteriales | Clostridia | Firmicutes |
| Odoribacter splanchnicus  (HG3A.0041) | T90 | -0.022 | 0.208 | 0.326 | 3364 unclassified | Odoribacter splanchnicus | Odoribacter | Odoribacteraceae | Bacteroidales | Bacteroidia | Bacteroidetes |
| Scardovia wiggsiae (HG3A.1737) | T90 | 0.022 | 0.208 | 0.326 | 3364 unclassified | Scardovia wiggsiae | Scardovia | Bifidobacteriaceae | Bifidobacteriales | Actinomycetia | Actinobacteria |
| Alistipes sp. AF17-16  (HG3A.0150) | T90 | -0.022 | 0.21 | 0.327 | 3364 unclassified | Alistipes sp. AF17-16 | Alistipes | Rikenellaceae | Bacteroidales | Bacteroidia | Bacteroidetes |
| Clostridia sp. (HG3A.0519) | ODI | -0.02 | 0.247 | 0.327 | 3364 unclassified | unclassified | unclassified | unclassified | unclassified | Clostridia | Firmicutes |
| Clostridia sp.  (HG3A.1139) | T90 | -0.022 | 0.21 | 0.327 | 3364 unclassified | unclassified | unclassified | unclassified | unclassified | Clostridia | Firmicutes |
| Eubacteriales sp. (HG3A.0441) | T90 | -0.022 | 0.211 | 0.327 | 3364 unclassified | unclassified | unclassified | unclassified | Eubacteriales | Clostridia | Firmicutes |
| Eubacteriales sp.  (HG3A.0791) | T90 | -0.022 | 0.211 | 0.327 | 3364 unclassified | unclassified | unclassified | unclassified | Eubacteriales | Clostridia | Firmicutes |
| Eubacteriales sp. (HG3A.0870) | T90 | -0.022 | 0.21 | 0.327 | 3364 unclassified | unclassified | unclassified | unclassified | Eubacteriales | Clostridia | Firmicutes |

AHI -0.025 0.181 0.321 3004 unclassified unclassified unclassified unclassified

Candidatus

Borkfalkiales Clostridia Firmicutes

Eubacteriales sp. (HG3A.0935)

T90 -0.022 0.211 0.327 3364 unclassified unclassified unclassified unclassified Eubacteriales Clostridia Firmicutes

| Eubacteriales sp. (HG3A.1199) | ODI | 0.02 | 0.246 | 0.327 | 3364 unclassified | unclassified | unclassified | unclassified | Eubacteriales | Clostridia | Firmicutes |
| --- | --- | --- | --- | --- | --- | --- | --- | --- | --- | --- | --- |
| Oscillospiraceae sp.  (HG3A.1588) | T90 | -0.022 | 0.211 | 0.327 | 3364 unclassified | unclassified | unclassified | Oscillospiraceae | Eubacteriales | Clostridia | Firmicutes |
| Bacteria sp. (HG3A.0218) | ODI | -0.02 | 0.25 | 0.328 | 3364 unclassified | unclassified | unclassified | unclassified | unclassified | unclassified | unclassified |
| Blautia producta  (HG3A.0619) | AHI | 0.024 | 0.189 | 0.328 | 3004 unclassified | Blautia producta | Blautia | Lachnospiraceae | Eubacteriales | Clostridia | Firmicutes |
| Clostridia sp. (HG3A.0783) | ODI | -0.02 | 0.25 | 0.328 | 3364 unclassified | unclassified | unclassified | unclassified | unclassified | Clostridia | Firmicutes |
| Clostridiaceae sp.  (HG3A.0491) | ODI | 0.02 | 0.247 | 0.328 | 3364 unclassified | unclassified | unclassified | Clostridiaceae | Eubacteriales | Clostridia | Firmicutes |
| [Clostridium] spiroforme (HG3A.0259) | ODI | 0.02 | 0.25 | 0.328 | 3364 unclassified | [Clostridium] spiroforme | Erysipelatoclostri dium | Erysipelotrichaceae | Erysipelotrichales | Erysipelotrichia | Firmicutes |
| Collinsella phocaeensis (HG3A.1340) | T90 | 0.022 | 0.213 | 0.328 | 3364 unclassified | Collinsella phocaeensis | Collinsella | Coriobacteriaceae | Coriobacteriales | Coriobacteriia | Actinobacteria |
| Desulfovibrio fairfieldensis (HG3A.0529) | ODI | -0.02 | 0.248 | 0.328 | 3364 unclassified | Desulfovibrio fairfieldensis | Desulfovibrio | Desulfovibrionaceae | Desulfovibrionales | Deltaproteobacte ria | Proteobacteria |
| Eubacteriales sp.  (HG3A.0697) | AHI | -0.024 | 0.19 | 0.329 | 3004 unclassified | unclassified | unclassified | unclassified | Eubacteriales | Clostridia | Firmicutes |
| Clostridium perfringens (HG3A.0959) | T90 | 0.022 | 0.216 | 0.33 | 3364 unclassified | Clostridium perfringens | Clostridium | Clostridiaceae | Eubacteriales | Clostridia | Firmicutes |
| Eubacteriales sp.  (HG3A.0244) | T90 | -0.022 | 0.215 | 0.33 | 3364 unclassified | unclassified | unclassified | unclassified | Eubacteriales | Clostridia | Firmicutes |
| Eubacteriales sp. (HG3A.0369) | T90 | -0.022 | 0.216 | 0.33 | 3364 unclassified | unclassified | unclassified | unclassified | Eubacteriales | Clostridia | Firmicutes |
| Eubacteriales sp.  (HG3A.0473) | AHI | -0.024 | 0.191 | 0.33 | 3004 unclassified | unclassified | unclassified | unclassified | Eubacteriales | Clostridia | Firmicutes |
| Eubacteriales sp. (HG3A.0544) | T90 | -0.022 | 0.216 | 0.33 | 3364 unclassified | unclassified | unclassified | unclassified | Eubacteriales | Clostridia | Firmicutes |
| Limosilactobacillus  fermentum (HG3A.0990) | T90 | 0.022 | 0.215 | 0.33 | 3364 unclassified | Limosilactobacillus fermentum | Limosilactobacill us | Lactobacillaceae | Lactobacillales | Bacilli | Firmicutes |
| Proteobacteria sp. (HG3A.0327) | AHI | -0.024 | 0.191 | 0.33 | 3004 unclassified | unclassified | unclassified | unclassified | unclassified | unclassified | Proteobacteria |

Eubacteriales sp.

# (HG3A.0644)

T90 -0.021 0.217 0.331 3364 unclassified unclassified unclassified unclassified Eubacteriales Clostridia Firmicutes

subsp.

| Clostridia sp.  (HG3A.1247) | ODI | -0.02 | 0.253 | 0.332 | 3364 unclassified | unclassified | unclassified | unclassified | unclassified | Clostridia | Firmicutes |
| --- | --- | --- | --- | --- | --- | --- | --- | --- | --- | --- | --- |
| Bacteroidales sp.  (HG3A.1236) | T90 | -0.021 | 0.219 | 0.334 | 3364 unclassified | unclassified | unclassified | unclassified | Bacteroidales | Bacteroidia | Bacteroidetes |
| Clostridia sp. (HG3A.0368) | AHI | -0.024 | 0.194 | 0.334 | 3004 unclassified | unclassified | unclassified | unclassified | unclassified | Clostridia | Firmicutes |
| Eubacteriales sp.  (HG3A.0161) | T90 | -0.021 | 0.22 | 0.334 | 3364 unclassified | unclassified | unclassified | unclassified | Eubacteriales | Clostridia | Firmicutes |
| Eubacteriales sp. (HG3A.0868) | T90 | -0.021 | 0.22 | 0.334 | 3364 unclassified | unclassified | unclassified | unclassified | Eubacteriales | Clostridia | Firmicutes |
| Oscillospiraceae sp.  (HG3A.0805) | ODI | -0.02 | 0.255 | 0.334 | 3364 unclassified | unclassified | unclassified | Oscillospiraceae | Eubacteriales | Clostridia | Firmicutes |
| Anaerostipes hadrus (HG3A.0003) | T90 | 0.021 | 0.222 | 0.335 | 3364 unclassified | Anaerostipes hadrus | Anaerostipes | Lachnospiraceae | Eubacteriales | Clostridia | Firmicutes |
| Fusobacterium nucleatum subsp. animalis (HG3A.1418) | T90 | 0.021 | 0.222 | 0.335 | Fusobacteriu 3364 m nucleatum  animalis | Fusobacterium nucleatum | Fusobacterium | Fusobacteriaceae | Fusobacteriales | Fusobacteriia | Fusobacteria |
| Ruminococcus sp. (HG3A.0126) | AHI | -0.024 | 0.195 | 0.335 | 3004 unclassified | unclassified | Ruminococcus | Oscillospiraceae | Eubacteriales | Clostridia | Firmicutes |
| Streptococcus oralis subsp. oralis  (HG3A.0705) | T90 | 0.021 | 0.222 | 0.335 | Streptococcus 3364 oralis subsp.  oralis | Streptococcus oralis | Streptococcus | Streptococcaceae | Lactobacillales | Bacilli | Firmicutes |
| Eubacteriales sp. (HG3A.0305) | AHI | -0.024 | 0.196 | 0.336 | 3004 unclassified | unclassified | unclassified | unclassified | Eubacteriales | Clostridia | Firmicutes |
| Turicibacter sanguinis (HG3A.0274) | ODI | -0.02 | 0.258 | 0.337 | 3364 unclassified | Turicibacter sanguinis | Turicibacter | Turicibacteraceae | Erysipelotrichales | Erysipelotrichia | Firmicutes |
| Clostridia sp. (HG3A.1193) | AHI | 0.024 | 0.199 | 0.339 | 3004 unclassified | unclassified | unclassified | unclassified | unclassified | Clostridia | Firmicutes |
| Eubacteriales sp.  (HG3A.0267) | T90 | -0.021 | 0.225 | 0.339 | 3364 unclassified | unclassified | unclassified | unclassified | Eubacteriales | Clostridia | Firmicutes |
| Eubacteriales sp. (HG3A.0664) | AHI | 0.024 | 0.199 | 0.339 | 3004 unclassified | unclassified | unclassified | unclassified | Eubacteriales | Clostridia | Firmicutes |
| Eubacteriales sp.  (HG3A.0685) | T90 | -0.021 | 0.227 | 0.339 | 3364 unclassified | unclassified | unclassified | unclassified | Eubacteriales | Clostridia | Firmicutes |
| Proteobacteria sp. (HG3A.0360) | T90 | -0.021 | 0.226 | 0.339 | 3364 unclassified | unclassified | unclassified | unclassified | unclassified | unclassified | Proteobacteria |
| Candidatus Borkfalkiales sp.  (HG3A.1329) | AHI | -0.024 | 0.2 | 0.34 | 3004 unclassified | unclassified | unclassified | unclassified | Candidatus Borkfalkiales | Clostridia | Firmicutes |
| Eubacteriales sp. (HG3A.0376) | ODI | -0.02 | 0.26 | 0.34 | 3364 unclassified | unclassified | unclassified | unclassified | Eubacteriales | Clostridia | Firmicutes |

Eubacteriales sp. (HG3A.0972)

ODI -0.02 0.261 0.34 3364 unclassified unclassified unclassified unclassified Eubacteriales Clostridia Firmicutes

| Roseburia faecis (HG3A.0058) | AHI | 0.024 | 0.2 | 0.34 | 3004 unclassified | Roseburia faecis | Roseburia | Lachnospiraceae | Eubacteriales | Clostridia | Firmicutes |
| --- | --- | --- | --- | --- | --- | --- | --- | --- | --- | --- | --- |
| Streptococcus sobrinus (HG3A.1366) | T90 | 0.021 | 0.227 | 0.34 | 3364 unclassified | Streptococcus sobrinus | Streptococcus | Streptococcaceae | Lactobacillales | Bacilli | Firmicutes |
| Clostridia sp. (HG3A.0660) | T90 | -0.021 | 0.23 | 0.342 | 3364 unclassified | unclassified | unclassified | unclassified | unclassified | Clostridia | Firmicutes |
| Clostridia sp.  (HG3A.1127) | T90 | -0.021 | 0.23 | 0.342 | 3364 unclassified | unclassified | unclassified | unclassified | unclassified | Clostridia | Firmicutes |
| Eubacteriales sp. (HG3A.0188) | T90 | -0.021 | 0.233 | 0.342 | 3364 unclassified | unclassified | unclassified | unclassified | Eubacteriales | Clostridia | Firmicutes |
| Eubacteriales sp.  (HG3A.0204) | T90 | -0.021 | 0.232 | 0.342 | 3364 unclassified | unclassified | unclassified | unclassified | Eubacteriales | Clostridia | Firmicutes |
| Eubacteriales sp. (HG3A.0367) | T90 | -0.021 | 0.23 | 0.342 | 3364 unclassified | unclassified | unclassified | unclassified | Eubacteriales | Clostridia | Firmicutes |
| Eubacteriales sp.  (HG3A.0565) | T90 | -0.021 | 0.232 | 0.342 | 3364 unclassified | unclassified | unclassified | unclassified | Eubacteriales | Clostridia | Firmicutes |
| Eubacteriales sp. (HG3A.0624) | T90 | -0.021 | 0.231 | 0.342 | 3364 unclassified | unclassified | unclassified | unclassified | Eubacteriales | Clostridia | Firmicutes |
| Eubacteriales sp.  (HG3A.0701) | T90 | -0.021 | 0.233 | 0.342 | 3364 unclassified | unclassified | unclassified | unclassified | Eubacteriales | Clostridia | Firmicutes |
| Eubacteriales sp. (HG3A.1239) | T90 | -0.021 | 0.23 | 0.342 | 3364 unclassified | unclassified | unclassified | unclassified | Eubacteriales | Clostridia | Firmicutes |
| Eubacteriales sp.  (HG3A.1256) | T90 | -0.021 | 0.232 | 0.342 | 3364 unclassified | unclassified | unclassified | unclassified | Eubacteriales | Clostridia | Firmicutes |
| Firmicutes sp. (HG3A.0769) | AHI | -0.023 | 0.203 | 0.344 | 3004 unclassified | unclassified | unclassified | unclassified | unclassified | unclassified | Firmicutes |
| Phocaeicola plebeius  (HG3A.0423) | AHI | -0.023 | 0.203 | 0.344 | 3004 unclassified | Phocaeicola plebeius | Phocaeicola | unclassified | Bacteroidales | Bacteroidia | Bacteroidetes |
| Eubacteriales sp. (HG3A.0649) | ODI | -0.019 | 0.265 | 0.345 | 3364 unclassified | unclassified | unclassified | unclassified | Eubacteriales | Clostridia | Firmicutes |
| Clostridia sp.  (HG3A.0996) | ODI | -0.019 | 0.266 | 0.346 | 3364 unclassified | unclassified | unclassified | unclassified | unclassified | Clostridia | Firmicutes |
| Eubacteriales sp. (HG3A.0509) | AHI | -0.023 | 0.205 | 0.346 | 3004 unclassified | unclassified | unclassified | unclassified | Eubacteriales | Clostridia | Firmicutes |
| Clostridia sp.  (HG3A.1609) | ODI | -0.019 | 0.268 | 0.347 | 3364 unclassified | unclassified | unclassified | unclassified | unclassified | Clostridia | Firmicutes |
| Coprococcus sp. AM27- 12LB (HG3A.0687) | AHI | 0.023 | 0.207 | 0.347 | 3004 unclassified | Coprococcus sp. AM27- 12LB | Coprococcus | Lachnospiraceae | Eubacteriales | Clostridia | Firmicutes |

Eubacteriales sp.

# (HG3A.0138)

AHI -0.023 0.207 0.347 3004 unclassified unclassified unclassified unclassified Eubacteriales Clostridia Firmicutes

| Eubacteriales sp.  (HG3A.0859) | AHI | -0.023 | 0.207 | 0.347 | 3004 unclassified | unclassified | unclassified | unclassified | Eubacteriales | Clostridia | Firmicutes |
| --- | --- | --- | --- | --- | --- | --- | --- | --- | --- | --- | --- |
| Lachnospiraceae sp.  (HG3A.0172) | ODI | -0.019 | 0.268 | 0.347 | 3364 unclassified | unclassified | unclassified | Lachnospiraceae | Eubacteriales | Clostridia | Firmicutes |
| Clostridia sp. (HG3A.0521) | AHI | -0.023 | 0.208 | 0.348 | 3004 unclassified | unclassified | unclassified | unclassified | unclassified | Clostridia | Firmicutes |
| Clostridiaceae sp.  (HG3A.0330) | ODI | -0.019 | 0.27 | 0.348 | 3364 unclassified | unclassified | unclassified | Clostridiaceae | Eubacteriales | Clostridia | Firmicutes |
| Eubacteriales sp. (HG3A.0148) | ODI | -0.019 | 0.27 | 0.348 | 3364 unclassified | unclassified | unclassified | unclassified | Eubacteriales | Clostridia | Firmicutes |
| Eubacteriales sp.  (HG3A.0970) | ODI | -0.019 | 0.27 | 0.348 | 3364 unclassified | unclassified | unclassified | unclassified | Eubacteriales | Clostridia | Firmicutes |
| Eubacteriales sp. (HG3A.0505) | ODI | -0.019 | 0.271 | 0.349 | 3364 unclassified | unclassified | unclassified | unclassified | Eubacteriales | Clostridia | Firmicutes |
| Bacteria sp.  (HG3A.1349) | AHI | -0.023 | 0.21 | 0.35 | 3004 unclassified | unclassified | unclassified | unclassified | unclassified | unclassified | unclassified |
| Eubacteriales sp. (HG3A.0333) | ODI | -0.019 | 0.272 | 0.35 | 3364 unclassified | unclassified | unclassified | unclassified | Eubacteriales | Clostridia | Firmicutes |
| Veillonella tobetsuensis (HG3A.1344) | T90 | -0.02 | 0.239 | 0.35 | 3364 unclassified | Veillonella tobetsuensis | Veillonella | Veillonellaceae | Veillonellales | Negativicutes | Firmicutes |
| Bacteroidales sp. (HG3A.1446) | ODI | -0.019 | 0.274 | 0.351 | 3364 unclassified | unclassified | unclassified | unclassified | Bacteroidales | Bacteroidia | Bacteroidetes |
| Clostridia sp.  (HG3A.1192) | ODI | -0.019 | 0.273 | 0.351 | 3364 unclassified | unclassified | unclassified | unclassified | unclassified | Clostridia | Firmicutes |
| Eubacteriales sp. (HG3A.0751) | T90 | -0.02 | 0.24 | 0.351 | 3364 unclassified | unclassified | unclassified | unclassified | Eubacteriales | Clostridia | Firmicutes |
| Bacteroidales sp.  (HG3A.0340) | AHI | -0.023 | 0.213 | 0.352 | 3004 unclassified | unclassified | unclassified | unclassified | Bacteroidales | Bacteroidia | Bacteroidetes |
| Eubacteriales sp. (HG3A.0613) | AHI | -0.023 | 0.212 | 0.352 | 3004 unclassified | unclassified | unclassified | unclassified | Eubacteriales | Clostridia | Firmicutes |
| Eubacteriales sp.  (HG3A.0621) | AHI | -0.023 | 0.211 | 0.352 | 3004 unclassified | unclassified | unclassified | unclassified | Eubacteriales | Clostridia | Firmicutes |
| Eubacteriales sp. (HG3A.0821) | AHI | -0.023 | 0.212 | 0.352 | 3004 unclassified | unclassified | unclassified | unclassified | Eubacteriales | Clostridia | Firmicutes |
| Oscillospiraceae sp.  (HG3A.0388) | AHI | -0.023 | 0.213 | 0.352 | 3004 unclassified | unclassified | unclassified | Oscillospiraceae | Eubacteriales | Clostridia | Firmicutes |
| Eubacteriales sp. (HG3A.0156) | T90 | -0.02 | 0.243 | 0.353 | 3364 unclassified | unclassified | unclassified | unclassified | Eubacteriales | Clostridia | Firmicutes |
| Eubacteriales sp.  (HG3A.0289) | T90 | -0.02 | 0.242 | 0.353 | 3364 unclassified | unclassified | unclassified | unclassified | Eubacteriales | Clostridia | Firmicutes |
| Streptococcus agalactiae (HG3A.1733) | AHI | 0.023 | 0.214 | 0.353 | 3004 unclassified | Streptococcus agalactiae | Streptococcus | Streptococcaceae | Lactobacillales | Bacilli | Firmicutes |

Firmicutes sp. (HG3A.0860)

| Firmicutes sp. (HG3A.0923) | AHI | -0.023 | 0.217 | 0.355 | 3004 unclassified | unclassified | unclassified | unclassified | unclassified | unclassified | Firmicutes |
| --- | --- | --- | --- | --- | --- | --- | --- | --- | --- | --- | --- |
| Oscillibacter sp.  (HG3A.0046) | T90 | -0.02 | 0.244 | 0.355 | 3364 unclassified | unclassified | Oscillibacter | Oscillospiraceae | Eubacteriales | Clostridia | Firmicutes |
| Peptostreptococcaceae sp. (HG3A.0200) | AHI | -0.023 | 0.217 | 0.355 | 3004 unclassified | unclassified | unclassified | Peptostreptococcacea e | Eubacteriales | Clostridia | Firmicutes |
| Eubacteriales sp.  (HG3A.0970) | T90 | -0.02 | 0.246 | 0.356 | 3364 unclassified | unclassified | unclassified | unclassified | Eubacteriales | Clostridia | Firmicutes |
| Clostridium sp. AF15-31 (HG3A.0293) | AHI | -0.023 | 0.218 | 0.357 | 3004 unclassified | Clostridium sp. AF15-31 | Clostridium | Clostridiaceae | Eubacteriales | Clostridia | Firmicutes |
| Alistipes timonensis  (HG3A.0586) | T90 | -0.02 | 0.248 | 0.359 | 3364 unclassified | Alistipes timonensis | Alistipes | Rikenellaceae | Bacteroidales | Bacteroidia | Bacteroidetes |
| Eubacteriales sp. (HG3A.1191) | T90 | -0.02 | 0.248 | 0.359 | 3364 unclassified | unclassified | unclassified | unclassified | Eubacteriales | Clostridia | Firmicutes |
| Oscillospiraceae sp.  (HG3A.1491) | T90 | -0.02 | 0.249 | 0.359 | 3364 unclassified | unclassified | unclassified | Oscillospiraceae | Eubacteriales | Clostridia | Firmicutes |
| Coprobacter fastidiosus (HG3A.0182) | ODI | -0.019 | 0.284 | 0.361 | 3364 unclassified | Coprobacter fastidiosus | Coprobacter | Barnesiellaceae | Bacteroidales | Bacteroidia | Bacteroidetes |
| Dorea phocaeensis  (HG3A.0865) | ODI | 0.019 | 0.283 | 0.361 | 3364 unclassified | Dorea phocaeensis | Dorea | Lachnospiraceae | Eubacteriales | Clostridia | Firmicutes |
| Enterococcus faecium (HG3A.0886) | ODI | 0.019 | 0.283 | 0.361 | 3364 unclassified | Enterococcus faecium | Enterococcus | Enterococcaceae | Lactobacillales | Bacilli | Firmicutes |
| Firmicutes sp.  (HG3A.1082) | T90 | -0.02 | 0.251 | 0.361 | 3364 unclassified | unclassified | unclassified | unclassified | unclassified | unclassified | Firmicutes |
| Oscillospiraceae sp. (HG3A.0343) | ODI | -0.019 | 0.282 | 0.361 | 3364 unclassified | unclassified | unclassified | Oscillospiraceae | Eubacteriales | Clostridia | Firmicutes |
| Eubacteriales sp.  (HG3A.0116) | ODI | -0.019 | 0.285 | 0.362 | 3364 unclassified | unclassified | unclassified | unclassified | Eubacteriales | Clostridia | Firmicutes |
| Eubacteriales sp. (HG3A.1067) | ODI | -0.019 | 0.286 | 0.362 | 3364 unclassified | unclassified | unclassified | unclassified | Eubacteriales | Clostridia | Firmicutes |
| Firmicutes sp.  (HG3A.1471) | ODI | -0.019 | 0.286 | 0.362 | 3364 unclassified | unclassified | unclassified | unclassified | unclassified | unclassified | Firmicutes |
| Clostridia sp. (HG3A.1298) | AHI | -0.022 | 0.223 | 0.363 | 3004 unclassified | unclassified | unclassified | unclassified | unclassified | Clostridia | Firmicutes |
| Clostridium sp. OF03- 18AA (HG3A.0119) | AHI | -0.022 | 0.223 | 0.363 | 3004 unclassified | Clostridium sp. OF03- 18AA | Clostridium | Clostridiaceae | Eubacteriales | Clostridia | Firmicutes |
| Eubacteriales sp. (HG3A.1285) | ODI | -0.019 | 0.287 | 0.363 | 3364 unclassified | unclassified | unclassified | unclassified | Eubacteriales | Clostridia | Firmicutes |

AHI -0.023 0.217 0.355 3004 unclassified unclassified unclassified unclassified unclassified unclassified Firmicutes

Firmicutes sp. (HG3A.0915)

| Eubacteriales sp. (HG3A.0325) | T90 | -0.02 | 0.255 | 0.364 | 3364 unclassified | unclassified | unclassified | unclassified | Eubacteriales | Clostridia | Firmicutes |
| --- | --- | --- | --- | --- | --- | --- | --- | --- | --- | --- | --- |
| Eubacteriales sp.  (HG3A.0350) | T90 | -0.02 | 0.254 | 0.364 | 3364 unclassified | unclassified | unclassified | unclassified | Eubacteriales | Clostridia | Firmicutes |
| Eubacteriales sp. (HG3A.0878) | T90 | -0.02 | 0.254 | 0.364 | 3364 unclassified | unclassified | unclassified | unclassified | Eubacteriales | Clostridia | Firmicutes |
| Parabacteroides  gordonii (HG3A.0989) | T90 | -0.02 | 0.253 | 0.364 | 3364 unclassified | Parabacteroides gordonii | Parabacteroides | Tannerellaceae | Bacteroidales | Bacteroidia | Bacteroidetes |
| Solobacterium moorei (HG3A.1589) | T90 | 0.02 | 0.255 | 0.364 | 3364 unclassified | Solobacterium moorei | Solobacterium | Erysipelotrichaceae | Erysipelotrichales | Erysipelotrichia | Firmicutes |
| Eubacteriales sp.  (HG3A.1305) | ODI | -0.018 | 0.291 | 0.368 | 3364 unclassified | unclassified | unclassified | unclassified | Eubacteriales | Clostridia | Firmicutes |
| Eubacteriales sp. (HG3A.0432) | T90 | -0.02 | 0.26 | 0.37 | 3364 unclassified | unclassified | unclassified | unclassified | Eubacteriales | Clostridia | Firmicutes |
| Eubacteriales sp.  (HG3A.0692) | T90 | 0.02 | 0.261 | 0.371 | 3364 unclassified | unclassified | unclassified | unclassified | Eubacteriales | Clostridia | Firmicutes |
| Eubacteriales sp. (HG3A.1305) | T90 | -0.02 | 0.261 | 0.371 | 3364 unclassified | unclassified | unclassified | unclassified | Eubacteriales | Clostridia | Firmicutes |
| Coprobacter fastidiosus (HG3A.0182) | AHI | -0.022 | 0.23 | 0.372 | 3004 unclassified | Coprobacter fastidiosus | Coprobacter | Barnesiellaceae | Bacteroidales | Bacteroidia | Bacteroidetes |
| Bacteroides cellulosilyticus (HG3A.0108) | ODI | -0.018 | 0.296 | 0.373 | 3364 unclassified | Bacteroides cellulosilyticus | Bacteroides | Bacteroidaceae | Bacteroidales | Bacteroidia | Bacteroidetes |
| Eubacteriales sp.  (HG3A.0528) | AHI | -0.022 | 0.231 | 0.375 | 3004 unclassified | unclassified | unclassified | unclassified | Eubacteriales | Clostridia | Firmicutes |
| Eubacteriales sp. (HG3A.0914) | ODI | -0.018 | 0.298 | 0.375 | 3364 unclassified | unclassified | unclassified | unclassified | Eubacteriales | Clostridia | Firmicutes |
| Eubacteriales sp.  (HG3A.0983) | ODI | -0.018 | 0.299 | 0.376 | 3364 unclassified | unclassified | unclassified | unclassified | Eubacteriales | Clostridia | Firmicutes |
| Alistipes ihumii (HG3A.0106) | AHI | -0.022 | 0.234 | 0.377 | 3004 unclassified | Alistipes ihumii | Alistipes | Rikenellaceae | Bacteroidales | Bacteroidia | Bacteroidetes |
| Bacteria sp.  (HG3A.0911) | AHI | -0.022 | 0.235 | 0.377 | 3004 unclassified | unclassified | unclassified | unclassified | unclassified | unclassified | unclassified |
| Clostridium sp. AF37-5 (HG3A.0076) | AHI | -0.022 | 0.234 | 0.377 | 3004 unclassified | Clostridium sp. AF37-5 | Clostridium | Clostridiaceae | Eubacteriales | Clostridia | Firmicutes |
| Eubacteriales sp.  (HG3A.0335) | AHI | 0.022 | 0.235 | 0.377 | 3004 unclassified | unclassified | unclassified | unclassified | Eubacteriales | Clostridia | Firmicutes |
| Eubacteriales sp. (HG3A.1131) | T90 | -0.019 | 0.267 | 0.378 | 3364 unclassified | unclassified | unclassified | unclassified | Eubacteriales | Clostridia | Firmicutes |

AHI -0.022 0.224 0.363 3004 unclassified unclassified unclassified unclassified unclassified unclassified Firmicutes

Erysipelotrichales sp. (HG3A.0809)

ODI 0.018 0.302 0.379 3364 unclassified unclassified unclassified unclassified Erysipelotrichales Erysipelotrichia Firmicutes

| Clostridia sp. (HG3A.0746) | T90 | -0.019 | 0.27 | 0.38 | 3364 unclassified | unclassified | unclassified | unclassified | unclassified | Clostridia | Firmicutes |
| --- | --- | --- | --- | --- | --- | --- | --- | --- | --- | --- | --- |
| Eubacteriales sp.  (HG3A.0514) | T90 | -0.019 | 0.269 | 0.38 | 3364 unclassified | unclassified | unclassified | unclassified | Eubacteriales | Clostridia | Firmicutes |
| Faecalibacterium sp. OF04-11AC (HG3A.0070) | T90 | -0.019 | 0.268 | 0.38 | 3364 unclassified | Faecalibacterium sp. OF04-11AC | Faecalibacterium | Oscillospiraceae | Eubacteriales | Clostridia | Firmicutes |
| Eubacteriales sp.  (HG3A.0664) | ODI | 0.018 | 0.304 | 0.381 | 3364 unclassified | unclassified | unclassified | unclassified | Eubacteriales | Clostridia | Firmicutes |
| Eubacteriales sp. (HG3A.0827) | T90 | -0.019 | 0.271 | 0.381 | 3364 unclassified | unclassified | unclassified | unclassified | Eubacteriales | Clostridia | Firmicutes |
| Enterocloster  clostridioformis (HG3A.0686) | AHI | 0.022 | 0.239 | 0.382 | 3004 unclassified | Enterocloster clostridioformis | Enterocloster | Lachnospiraceae | Eubacteriales | Clostridia | Firmicutes |
| Bacteroides nordii (HG3A.0290) | AHI | -0.021 | 0.244 | 0.383 | 3004 unclassified | Bacteroides nordii | Bacteroides | Bacteroidaceae | Bacteroidales | Bacteroidia | Bacteroidetes |
| Enterocloster aldenensis (HG3A.0362) | AHI | 0.022 | 0.241 | 0.383 | 3004 unclassified | Enterocloster aldenensis | Enterocloster | Lachnospiraceae | Eubacteriales | Clostridia | Firmicutes |
| Erysipelotrichales sp. (HG3A.0283) | AHI | -0.022 | 0.243 | 0.383 | 3004 unclassified | unclassified | unclassified | unclassified | Erysipelotrichales | Erysipelotrichia | Firmicutes |
| Eubacteriales sp.  (HG3A.0080) | AHI | 0.022 | 0.242 | 0.383 | 3004 unclassified | unclassified | unclassified | unclassified | Eubacteriales | Clostridia | Firmicutes |
| Eubacteriales sp. (HG3A.0364) | AHI | -0.022 | 0.241 | 0.383 | 3004 unclassified | unclassified | unclassified | unclassified | Eubacteriales | Clostridia | Firmicutes |
| Eubacteriales sp.  (HG3A.1573) | AHI | -0.022 | 0.242 | 0.383 | 3004 unclassified | unclassified | unclassified | unclassified | Eubacteriales | Clostridia | Firmicutes |
| Faecalibacterium prausnitzii (HG3A.0010) | AHI | 0.022 | 0.241 | 0.383 | 3004 unclassified | Faecalibacterium prausnitzii | Faecalibacterium | Oscillospiraceae | Eubacteriales | Clostridia | Firmicutes |
| Ruminococcus sp.  (HG3A.0337) | AHI | -0.022 | 0.24 | 0.383 | 3004 unclassified | unclassified | Ruminococcus | Oscillospiraceae | Eubacteriales | Clostridia | Firmicutes |
| Turicibacter sanguinis (HG3A.0274) | AHI | -0.022 | 0.243 | 0.383 | 3004 unclassified | Turicibacter sanguinis | Turicibacter | Turicibacteraceae | Erysipelotrichales | Erysipelotrichia | Firmicutes |
| Eubacteriales sp.  (HG3A.0511) | T90 | -0.019 | 0.275 | 0.385 | 3364 unclassified | unclassified | unclassified | unclassified | Eubacteriales | Clostridia | Firmicutes |
| Firmicutes sp. (HG3A.0464) | T90 | -0.019 | 0.274 | 0.385 | 3364 unclassified | unclassified | unclassified | unclassified | unclassified | unclassified | Firmicutes |

Eubacteriales sp.

# (HG3A.0163)

T90 -0.019 0.276 0.386 3364 unclassified unclassified unclassified unclassified Eubacteriales Clostridia Firmicutes

| Eubacteriales sp.  (HG3A.0649) | T90 | -0.019 | 0.277 | 0.386 | 3364 unclassified | unclassified | unclassified | unclassified | Eubacteriales | Clostridia | Firmicutes |
| --- | --- | --- | --- | --- | --- | --- | --- | --- | --- | --- | --- |
| Eubacteriales sp.  (HG3A.0873) | T90 | -0.019 | 0.277 | 0.386 | 3364 unclassified | unclassified | unclassified | unclassified | Eubacteriales | Clostridia | Firmicutes |
| Eubacteriales sp. (HG3A.0964) | T90 | -0.019 | 0.276 | 0.386 | 3364 unclassified | unclassified | unclassified | unclassified | Eubacteriales | Clostridia | Firmicutes |
| Flavonifractor sp. An10  (HG3A.0495) | T90 | -0.019 | 0.278 | 0.387 | 3364 unclassified | Flavonifractor sp. An10 | Flavonifractor | Oscillospiraceae | Eubacteriales | Clostridia | Firmicutes |
| Streptococcus sobrinus (HG3A.1366) | ODI | 0.018 | 0.311 | 0.388 | 3364 unclassified | Streptococcus sobrinus | Streptococcus | Streptococcaceae | Lactobacillales | Bacilli | Firmicutes |
| Eubacteriales sp.  (HG3A.0551) | AHI | -0.021 | 0.248 | 0.389 | 3004 unclassified | unclassified | unclassified | unclassified | Eubacteriales | Clostridia | Firmicutes |
| Eubacteriales sp. (HG3A.0663) | ODI | -0.018 | 0.311 | 0.389 | 3364 unclassified | unclassified | unclassified | unclassified | Eubacteriales | Clostridia | Firmicutes |
| Evtepia gabavorous  (HG3A.0114) | ODI | 0.018 | 0.312 | 0.389 | 3364 unclassified | Evtepia gabavorous | Evtepia | unclassified | Eubacteriales | Clostridia | Firmicutes |
| Barnesiellaceae sp. (HG3A.1180) | T90 | -0.019 | 0.281 | 0.39 | 3364 unclassified | unclassified | unclassified | Barnesiellaceae | Bacteroidales | Bacteroidia | Bacteroidetes |
| Eubacteriales sp.  (HG3A.0781) | AHI | -0.021 | 0.249 | 0.39 | 3004 unclassified | unclassified | unclassified | unclassified | Eubacteriales | Clostridia | Firmicutes |
| Streptococcus mutans (HG3A.0677) | T90 | 0.019 | 0.281 | 0.39 | 3364 unclassified | Streptococcus mutans | Streptococcus | Streptococcaceae | Lactobacillales | Bacilli | Firmicutes |
| Victivallis lenta  (HG3A.0525) | AHI | -0.021 | 0.25 | 0.391 | 3004 unclassified | Victivallis lenta | Victivallis | Victivallaceae | Victivallales | Lentisphaeria | Lentisphaerae |
| Eubacteriales sp. (HG3A.1045) | T90 | -0.019 | 0.284 | 0.392 | 3364 unclassified | unclassified | unclassified | unclassified | Eubacteriales | Clostridia | Firmicutes |
| Oscillospiraceae sp.  (HG3A.0765) | AHI | -0.021 | 0.252 | 0.393 | 3004 unclassified | unclassified | unclassified | Oscillospiraceae | Eubacteriales | Clostridia | Firmicutes |
| Alistipes indistinctus (HG3A.0121) | AHI | -0.021 | 0.253 | 0.394 | 3004 unclassified | Alistipes indistinctus | Alistipes | Rikenellaceae | Bacteroidales | Bacteroidia | Bacteroidetes |
| Clostridia sp.  (HG3A.0946) | T90 | -0.019 | 0.286 | 0.395 | 3364 unclassified | unclassified | unclassified | unclassified | unclassified | Clostridia | Firmicutes |
| Actinomycetaceae sp. (HG3A.1068) | T90 | 0.019 | 0.287 | 0.396 | 3364 unclassified | unclassified | unclassified | Actinomycetaceae | Actinomycetales | Actinomycetia | Actinobacteria |
| Erysipelotrichales sp.  (HG3A.0283) | T90 | -0.018 | 0.288 | 0.396 | 3364 unclassified | unclassified | unclassified | unclassified | Erysipelotrichales | Erysipelotrichia | Firmicutes |
| Eubacteriales sp. (HG3A.0472) | T90 | -0.018 | 0.288 | 0.396 | 3364 unclassified | unclassified | unclassified | unclassified | Eubacteriales | Clostridia | Firmicutes |
| Clostridia sp.  (HG3A.1038) | AHI | -0.021 | 0.259 | 0.399 | 3004 unclassified | unclassified | unclassified | unclassified | unclassified | Clostridia | Firmicutes |
| Clostridia sp. (HG3A.1427) | AHI | -0.021 | 0.258 | 0.399 | 3004 unclassified | unclassified | unclassified | unclassified | unclassified | Clostridia | Firmicutes |

Eubacteriales sp. (HG3A.0333)

| Faecalibacterium sp. Faecalibacterium sp.  OF04-11AC AHI -0.021 0.258 0.399 3004 unclassified OF04-11AC Faecalibacterium Oscillospiraceae Eubacteriales Clostridia Firmicutes  (HG3A.0070) | | | | | | | | | | | |
| --- | --- | --- | --- | --- | --- | --- | --- | --- | --- | --- | --- |
| Firmicutes sp.  (HG3A.1048) | T90 | -0.018 | 0.291 | 0.399 | 3364 unclassified | unclassified | unclassified | unclassified | unclassified | unclassified | Firmicutes |
| Parabacteroides  gordonii (HG3A.0989) | AHI | 0.021 | 0.257 | 0.399 | 3004 unclassified | Parabacteroides gordonii | Parabacteroides | Tannerellaceae | Bacteroidales | Bacteroidia | Bacteroidetes |
| Anaeromassilibacillus sp. An250 (HG3A.0169) | AHI | -0.021 | 0.26 | 0.402 | 3004 unclassified | Anaeromassilibacillus sp. An250 | Anaeromassiliba cillus | Oscillospiraceae | Eubacteriales | Clostridia | Firmicutes |
| Eubacteriales sp. (HG3A.1187) | ODI | 0.017 | 0.323 | 0.402 | 3364 unclassified | unclassified | unclassified | unclassified | Eubacteriales | Clostridia | Firmicutes |
| Bacteroidales sp.  (HG3A.1002) | ODI | -0.017 | 0.325 | 0.403 | 3364 unclassified | unclassified | unclassified | unclassified | Bacteroidales | Bacteroidia | Bacteroidetes |
| Eubacteriales sp. (HG3A.1243) | ODI | -0.017 | 0.324 | 0.403 | 3364 unclassified | unclassified | unclassified | unclassified | Eubacteriales | Clostridia | Firmicutes |
| Oscillospiraceae sp.  (HG3A.0665) | ODI | -0.017 | 0.326 | 0.403 | 3364 unclassified | unclassified | unclassified | Oscillospiraceae | Eubacteriales | Clostridia | Firmicutes |
| Bacteria sp. (HG3A.0708) | T90 | -0.018 | 0.298 | 0.406 | 3364 unclassified | unclassified | unclassified | unclassified | unclassified | unclassified | unclassified |
| Clostridia sp.  (HG3A.0767) | AHI | -0.021 | 0.264 | 0.406 | 3004 unclassified | unclassified | unclassified | unclassified | unclassified | Clostridia | Firmicutes |
| Eubacteriales sp. (HG3A.0505) | T90 | -0.018 | 0.299 | 0.406 | 3364 unclassified | unclassified | unclassified | unclassified | Eubacteriales | Clostridia | Firmicutes |
| Faecalibacterium sp.  (HG3A.0073) | ODI | -0.017 | 0.328 | 0.406 | 3364 unclassified | unclassified | Faecalibacterium | Oscillospiraceae | Eubacteriales | Clostridia | Firmicutes |
| Gemmiger formicilis (HG3A.0027) | T90 | 0.018 | 0.298 | 0.406 | 3364 unclassified | Gemmiger formicilis | Gemmiger | unclassified | Eubacteriales | Clostridia | Firmicutes |
| Lachnospiraceae sp.  (HG3A.1155) | T90 | -0.018 | 0.298 | 0.406 | 3364 unclassified | unclassified | unclassified | Lachnospiraceae | Eubacteriales | Clostridia | Firmicutes |
| Bacteria sp. (HG3A.1553) | T90 | -0.018 | 0.301 | 0.407 | 3364 unclassified | unclassified | unclassified | unclassified | unclassified | unclassified | unclassified |
| Clostridia sp.  (HG3A.0661) | T90 | -0.018 | 0.301 | 0.407 | 3364 unclassified | unclassified | unclassified | unclassified | unclassified | Clostridia | Firmicutes |
| Eubacteriales sp. (HG3A.0781) | T90 | -0.018 | 0.3 | 0.407 | 3364 unclassified | unclassified | unclassified | unclassified | Eubacteriales | Clostridia | Firmicutes |
| Eubacteriales sp.  (HG3A.0939) | ODI | -0.017 | 0.334 | 0.412 | 3364 unclassified | unclassified | unclassified | unclassified | Eubacteriales | Clostridia | Firmicutes |
| Limosilactobacillus vaginalis (HG3A.1341) | ODI | 0.017 | 0.334 | 0.412 | 3364 unclassified | Limosilactobacillus vaginalis | Limosilactobacill us | Lactobacillaceae | Lactobacillales | Bacilli | Firmicutes |

T90 -0.018 0.292 0.399 3364 unclassified unclassified unclassified unclassified Eubacteriales Clostridia Firmicutes

Coprococcus catus (HG3A.0037)

| Eubacteriales sp. (HG3A.1269) | ODI | -0.017 | 0.335 | 0.413 | 3364 unclassified | unclassified | unclassified | unclassified | Eubacteriales | Clostridia | Firmicutes |
| --- | --- | --- | --- | --- | --- | --- | --- | --- | --- | --- | --- |
| Streptococcus oralis subsp. oralis  (HG3A.0705) | AHI | 0.02 | 0.269 | 0.413 | Streptococcus 3004 oralis subsp.  oralis | Streptococcus oralis | Streptococcus | Streptococcaceae | Lactobacillales | Bacilli | Firmicutes |
| Bacteroides intestinalis (HG3A.0265) | T90 | -0.018 | 0.307 | 0.414 | 3364 unclassified | Bacteroides intestinalis | Bacteroides | Bacteroidaceae | Bacteroidales | Bacteroidia | Bacteroidetes |
| Eubacteriales sp.  (HG3A.0588) | ODI | -0.017 | 0.337 | 0.414 | 3364 unclassified | unclassified | unclassified | unclassified | Eubacteriales | Clostridia | Firmicutes |
| Eubacteriales sp. (HG3A.0592) | AHI | -0.02 | 0.27 | 0.414 | 3004 unclassified | unclassified | unclassified | unclassified | Eubacteriales | Clostridia | Firmicutes |
| Lachnospiraceae sp.  (HG3A.0217) | T90 | -0.018 | 0.307 | 0.414 | 3364 unclassified | unclassified | unclassified | Lachnospiraceae | Eubacteriales | Clostridia | Firmicutes |
| Clostridia sp. (HG3A.1252) | T90 | -0.018 | 0.31 | 0.416 | 3364 unclassified | unclassified | unclassified | unclassified | unclassified | Clostridia | Firmicutes |
| Eubacteriales sp.  (HG3A.1030) | AHI | -0.02 | 0.272 | 0.416 | 3004 unclassified | unclassified | unclassified | unclassified | Eubacteriales | Clostridia | Firmicutes |
| Faecalibacterium prausnitzii (HG3A.0241) | AHI | -0.02 | 0.272 | 0.416 | 3004 unclassified | Faecalibacterium prausnitzii | Faecalibacterium | Oscillospiraceae | Eubacteriales | Clostridia | Firmicutes |
| Firmicutes sp.  (HG3A.1124) | T90 | -0.018 | 0.31 | 0.416 | 3364 unclassified | unclassified | unclassified | unclassified | unclassified | unclassified | Firmicutes |
| Firmicutes sp. (HG3A.1290) | T90 | -0.018 | 0.311 | 0.416 | 3364 unclassified | unclassified | unclassified | unclassified | unclassified | unclassified | Firmicutes |
| Eubacteriales sp.  (HG3A.0881) | AHI | -0.02 | 0.274 | 0.417 | 3004 unclassified | unclassified | unclassified | unclassified | Eubacteriales | Clostridia | Firmicutes |
| Clostridiaceae sp. (HG3A.0608) | ODI | -0.017 | 0.342 | 0.418 | 3364 unclassified | unclassified | unclassified | Clostridiaceae | Eubacteriales | Clostridia | Firmicutes |
| Eubacteriales sp.  (HG3A.0175) | ODI | -0.017 | 0.341 | 0.418 | 3364 unclassified | unclassified | unclassified | unclassified | Eubacteriales | Clostridia | Firmicutes |
| Eubacteriales sp. (HG3A.0302) | ODI | -0.017 | 0.342 | 0.418 | 3364 unclassified | unclassified | unclassified | unclassified | Eubacteriales | Clostridia | Firmicutes |
| Eubacteriales sp.  (HG3A.0511) | ODI | -0.017 | 0.342 | 0.418 | 3364 unclassified | unclassified | unclassified | unclassified | Eubacteriales | Clostridia | Firmicutes |
| Anaerotruncus massiliensis (HG3A.0460) | AHI | -0.02 | 0.277 | 0.42 | 3004 unclassified | Anaerotruncus massiliensis | Anaerotruncus | Oscillospiraceae | Eubacteriales | Clostridia | Firmicutes |
| Eubacteriales sp.  (HG3A.0457) | AHI | -0.02 | 0.276 | 0.42 | 3004 unclassified | unclassified | unclassified | unclassified | Eubacteriales | Clostridia | Firmicutes |
| Eubacteriales sp. (HG3A.0588) | AHI | -0.02 | 0.277 | 0.42 | 3004 unclassified | unclassified | unclassified | unclassified | Eubacteriales | Clostridia | Firmicutes |

ODI 0.017 0.336 0.413 3364 unclassified Coprococcus catus Coprococcus Lachnospiraceae Eubacteriales Clostridia Firmicutes

Firmicutes sp. (HG3A.0769)

| Desulfovibrionales sp. (HG3A.0266) | AHI | -0.02 | 0.279 | 0.422 | 3004 unclassified | unclassified | unclassified | unclassified | Desulfovibrionales | Deltaproteobacte ria | Proteobacteria |
| --- | --- | --- | --- | --- | --- | --- | --- | --- | --- | --- | --- |
| Eubacteriales sp.  (HG3A.0144) | AHI | -0.02 | 0.279 | 0.422 | 3004 unclassified | unclassified | unclassified | unclassified | Eubacteriales | Clostridia | Firmicutes |
| Bacteroides intestinalis (HG3A.0265) | AHI | -0.02 | 0.282 | 0.423 | 3004 unclassified | Bacteroides intestinalis | Bacteroides | Bacteroidaceae | Bacteroidales | Bacteroidia | Bacteroidetes |
| Dorea sp. AF24-7LB  (HG3A.0086) | AHI | 0.02 | 0.282 | 0.423 | 3004 unclassified | Dorea sp. AF24-7LB | Dorea | Lachnospiraceae | Eubacteriales | Clostridia | Firmicutes |
| Eubacteriales sp. (HG3A.1292) | AHI | -0.02 | 0.281 | 0.423 | 3004 unclassified | unclassified | unclassified | unclassified | Eubacteriales | Clostridia | Firmicutes |
| Eubacterium sp. AF17-7 (HG3A.0165) | AHI | -0.02 | 0.281 | 0.423 | 3004 unclassified | Eubacterium sp. AF17-7 | Eubacterium | Eubacteriaceae | Eubacteriales | Clostridia | Firmicutes |
| Dorea sp. AF24-7LB (HG3A.0086) | ODI | 0.016 | 0.349 | 0.424 | 3364 unclassified | Dorea sp. AF24-7LB | Dorea | Lachnospiraceae | Eubacteriales | Clostridia | Firmicutes |
| Eubacteriales sp.  (HG3A.0970) | AHI | -0.02 | 0.283 | 0.424 | 3004 unclassified | unclassified | unclassified | unclassified | Eubacteriales | Clostridia | Firmicutes |
| Eubacteriales sp. (HG3A.0644) | ODI | -0.016 | 0.351 | 0.426 | 3364 unclassified | unclassified | unclassified | unclassified | Eubacteriales | Clostridia | Firmicutes |
| Eubacteriales sp.  (HG3A.0717) | ODI | -0.016 | 0.351 | 0.426 | 3364 unclassified | unclassified | unclassified | unclassified | Eubacteriales | Clostridia | Firmicutes |
| Clostridia sp. (HG3A.0512) | AHI | -0.02 | 0.286 | 0.427 | 3004 unclassified | unclassified | unclassified | unclassified | unclassified | Clostridia | Firmicutes |
| Eubacteriales sp.  (HG3A.0547) | T90 | -0.017 | 0.32 | 0.427 | 3364 unclassified | unclassified | unclassified | unclassified | Eubacteriales | Clostridia | Firmicutes |
| Lactobacillus gasseri (HG3A.0884) | T90 | 0.017 | 0.32 | 0.427 | 3364 unclassified | Lactobacillus gasseri | Lactobacillus | Lactobacillaceae | Lactobacillales | Bacilli | Firmicutes |
| Eubacteriales sp.  (HG3A.0406) | AHI | -0.02 | 0.287 | 0.428 | 3004 unclassified | unclassified | unclassified | unclassified | Eubacteriales | Clostridia | Firmicutes |
| Clostridia sp. (HG3A.0861) | ODI | -0.016 | 0.355 | 0.429 | 3364 unclassified | unclassified | unclassified | unclassified | unclassified | Clostridia | Firmicutes |
| Clostridia sp.  (HG3A.1417) | ODI | -0.016 | 0.355 | 0.429 | 3364 unclassified | unclassified | unclassified | unclassified | unclassified | Clostridia | Firmicutes |
| Firmicutes sp. (HG3A.0570) | ODI | -0.016 | 0.354 | 0.429 | 3364 unclassified | unclassified | unclassified | unclassified | unclassified | unclassified | Firmicutes |
| Lachnospiraceae sp.  (HG3A.0172) | T90 | -0.017 | 0.322 | 0.429 | 3364 unclassified | unclassified | unclassified | Lachnospiraceae | Eubacteriales | Clostridia | Firmicutes |
| Barnesiella Barnesiella  intestinihominis T90 -0.017 0.324 0.431 3364 unclassified intestinihominis Barnesiella Barnesiellaceae Bacteroidales Bacteroidia Bacteroidetes  (HG3A.0055) | | | | | | | | | | | |

T90 -0.017 0.315 0.421 3364 unclassified unclassified unclassified unclassified unclassified unclassified Firmicutes

Eubacteriales sp. (HG3A.0516)

Clostridia sp.

(HG3A.1356)

ODI

-0.016

0.358

0.432 3364 unclassified unclassified

unclassified

unclassified

unclassified

Clostridia

Firmicutes

AHI -0.02 0.29 0.431 3004 unclassified unclassified unclassified unclassified Eubacteriales Clostridia Firmicutes

Clostridia sp. (HG3A.1410)

Clostridia sp.

(HG3A.1139)

AHI

-0.019

0.295

0.437 3004 unclassified unclassified

unclassified

unclassified

unclassified

Clostridia

Firmicutes

AHI -0.019 0.293 0.434 3004 unclassified unclassified unclassified unclassified unclassified Clostridia Firmicutes

Eubacteriales sp. (HG3A.0914)

Oscillospiraceae sp.

(HG3A.0739)

ODI

-0.016

0.363

0.437 3364 unclassified unclassified

unclassified

Oscillospiraceae

Eubacteriales

Clostridia

Firmicutes

T90 -0.017 0.33 0.437 3364 unclassified unclassified unclassified unclassified Eubacteriales Clostridia Firmicutes

Anaeroglobus geminatus

(HG3A.1818) AHI 0.019 0.297 0.44 3004 unclassified Anaeroglobus geminatus Anaeroglobus Veillonellaceae Veillonellales Negativicutes Firmicutes

Eubacteriales sp.

(HG3A.1292)

T90

-0.017

0.332

0.44 3364 unclassified unclassified

unclassified

unclassified

Eubacteriales

Clostridia

Firmicutes

Clostridia sp.

# (HG3A.1417)

T90 -0.017 0.334 0.442 3364 unclassified unclassified unclassified unclassified unclassified Clostridia Firmicutes

Blautia producta

(HG3A.0905)

AHI

0.019

0.301

0.443 3004 unclassified Blautia producta

Blautia

Lachnospiraceae

Eubacteriales

Clostridia

Firmicutes

Eubacteriales sp.

# (HG3A.0158)

ODI -0.016 0.368 0.443 3364 unclassified unclassified unclassified unclassified Eubacteriales Clostridia Firmicutes

Eubacteriales sp.

(HG3A.0962)

AHI

0.019

0.302

0.443 3004 unclassified unclassified

unclassified

unclassified

Eubacteriales

Clostridia

Firmicutes

Streptococcus salivarius

(HG3A.0071) AHI 0.019 0.301 0.443 3004 unclassified Streptococcus salivarius Streptococcus Streptococcaceae Lactobacillales Bacilli Firmicutes

Oxalobacter sp.

(HG3A.1097)

ODI

0.016

0.37

0.444 3364 unclassified unclassified

Oxalobacter

Oxalobacteraceae Burkholderiales

Betaproteobacteri Proteobacteria

a

Bacteria sp.

# (HG3A.0218)

AHI -0.019 0.308 0.449 3004 unclassified unclassified unclassified unclassified unclassified unclassified unclassified

Bacteroidales sp.

(HG3A.1236)

AHI

-0.019

0.306

0.449 3004 unclassified unclassified

unclassified

unclassified

Bacteroidales

Bacteroidia

Bacteroidetes

Clostridia sp.

# (HG3A.1262)

AHI -0.019 0.308 0.449 3004 unclassified unclassified unclassified unclassified unclassified Clostridia Firmicutes

Eubacteriales sp.

(HG3A.1332)

AHI

-0.019

0.307

0.449 3004 unclassified unclassified

unclassified

unclassified

Eubacteriales

Clostridia

Firmicutes

Streptococcus

gallolyticus subsp. gallolyticus (HG3A.1651)

Eubacteriales sp.

(HG3A.1078)

AHI

-0.019

0.311

0.45 3004 unclassified unclassified

unclassified

unclassified

Eubacteriales

Clostridia

Firmicutes

Streptococcus

AHI 0.019 0.308 0.449 3004 gallolyticus subsp.

gallolyticus

Streptococcus gallolyticus Streptococcus Streptococcaceae Lactobacillales Bacilli Firmicutes

Lachnospiraceae sp. (HG3A.0831)

Methanobrevibacter

smithii (HG3A.0152)

AHI

-0.019

0.31

0.45 3004 unclassified

Methanobrevibacter

smithii

Methanobrevibact Methanobacteriaceae Methanobacteriales Methanobacteria Euryarchaeota

er

AHI -0.019 0.31 0.45 3004 unclassified unclassified unclassified Lachnospiraceae Eubacteriales Clostridia Firmicutes

Oxalobacter sp. (HG3A.1097)

Intestinimonas butyriciproducens

(HG3A.0187)

AHI

-0.019

0.315

0.454 3004 unclassified

Intestinimonas

butyriciproducens

Intestinimonas unclassified

Eubacteriales

Clostridia

Firmicutes

AHI 0.019 0.311 0.45 3004 unclassified unclassified Oxalobacter Oxalobacteraceae Burkholderiales Betaproteobacteri Proteobacteria

a

Oscillospiraceae sp. (HG3A.0765)

Eubacteriales sp.

(HG3A.0453)

AHI

-0.018

0.316

0.455 3004 unclassified unclassified

unclassified

unclassified

Eubacteriales

Clostridia

Firmicutes

ODI -0.015 0.379 0.454 3364 unclassified unclassified unclassified Oscillospiraceae Eubacteriales Clostridia Firmicutes

Eubacteriales sp. (HG3A.0476)

Oscillospiraceae sp.

(HG3A.0576)

ODI

-0.015

0.38

0.455 3364 unclassified unclassified

unclassified

Oscillospiraceae

Eubacteriales

Clostridia

Firmicutes

T90 -0.016 0.344 0.455 3364 unclassified unclassified unclassified unclassified Eubacteriales Clostridia Firmicutes

Oscillospiraceae sp. (HG3A.0612)

Oscillospiraceae sp.

(HG3A.0966)

AHI

-0.018

0.317

0.455 3004 unclassified unclassified

unclassified

Oscillospiraceae

Eubacteriales

Clostridia

Firmicutes

AHI 0.018 0.317 0.455 3004 unclassified unclassified unclassified Oscillospiraceae Eubacteriales Clostridia Firmicutes

Clostridia sp. (HG3A.1247)

Clostridia sp.

(HG3A.1493)

ODI

-0.015

0.382

0.456 3364 unclassified unclassified

unclassified

unclassified

unclassified

Clostridia

Firmicutes

AHI -0.018 0.318 0.456 3004 unclassified unclassified unclassified unclassified unclassified Clostridia Firmicutes

Erysipelatoclostridium sp. (HG3A.0313)

Oscillibacter sp.

(HG3A.0243)

ODI

-0.015

0.384

0.458 3364 unclassified unclassified

Oscillibacter

Oscillospiraceae

Eubacteriales

Clostridia

Firmicutes

ODI -0.015 0.382 0.456 3364 unclassified unclassified Erysipelatoclostri Erysipelotrichaceae Erysipelotrichales Erysipelotrichia Firmicutes

dium

Clostridia sp. (HG3A.1220)

Eubacteriales sp.

(HG3A.0334)

T90

-0.016

0.348

0.459 3364 unclassified unclassified

unclassified

unclassified

Eubacteriales

Clostridia

Firmicutes

T90 -0.016 0.349 0.459 3364 unclassified unclassified unclassified unclassified unclassified Clostridia Firmicutes

Bacteroidales sp. (HG3A.0340)

Eubacteriales sp.

(HG3A.0962)

T90

-0.016

0.35

0.46 3364 unclassified unclassified

unclassified

unclassified

Eubacteriales

Clostridia

Firmicutes

ODI -0.015 0.386 0.46 3364 unclassified unclassified unclassified unclassified Bacteroidales Bacteroidia Bacteroidetes

Parabacteroides

goldsteinii (HG3A.0279) AHI -0.018 0.322 0.46 3004 unclassified

Parabacteroides

goldsteinii Parabacteroides Tannerellaceae Bacteroidales Bacteroidia Bacteroidetes

Clostridia sp. (HG3A.0011)

Eubacteriales sp. (HG3A.1199)

AHI 0.018 0.324 0.461 3004 unclassified unclassified unclassified unclassified unclassified Clostridia Firmicutes

AHI 0.018 0.323 0.461 3004 unclassified unclassified unclassified unclassified Eubacteriales Clostridia Firmicutes

| Eubacteriales sp.  (HG3A.1256) | ODI | -0.015 | 0.389 | 0.462 | 3364 unclassified | unclassified | unclassified | unclassified | Eubacteriales | Clostridia | Firmicutes |
| --- | --- | --- | --- | --- | --- | --- | --- | --- | --- | --- | --- |
| Pseudoruminococcus massiliensis  (HG3A.0346) | ODI | -0.015 | 0.39 | 0.463 | 3364 unclassified | Pseudoruminococcus massiliensis | Pseudoruminoco ccus | Oscillospiraceae | Eubacteriales | Clostridia | Firmicutes |
| Clostridia sp. (HG3A.1417) | AHI | -0.018 | 0.326 | 0.464 | 3004 unclassified | unclassified | unclassified | unclassified | unclassified | Clostridia | Firmicutes |
| Eubacteriales sp.  (HG3A.0543) | T90 | -0.016 | 0.354 | 0.464 | 3364 unclassified | unclassified | unclassified | unclassified | Eubacteriales | Clostridia | Firmicutes |
| Eubacteriales sp. (HG3A.1134) | ODI | -0.015 | 0.392 | 0.464 | 3364 unclassified | unclassified | unclassified | unclassified | Eubacteriales | Clostridia | Firmicutes |
| Eubacteriales sp.  (HG3A.0230) | T90 | -0.016 | 0.355 | 0.465 | 3364 unclassified | unclassified | unclassified | unclassified | Eubacteriales | Clostridia | Firmicutes |
| Eubacteriales sp. (HG3A.0496) | AHI | -0.018 | 0.327 | 0.465 | 3004 unclassified | unclassified | unclassified | unclassified | Eubacteriales | Clostridia | Firmicutes |
| Clostridia sp.  (HG3A.1193) | T90 | -0.016 | 0.357 | 0.466 | 3364 unclassified | unclassified | unclassified | unclassified | unclassified | Clostridia | Firmicutes |
| Eubacteriales sp. (HG3A.0136) | T90 | -0.016 | 0.359 | 0.467 | 3364 unclassified | unclassified | unclassified | unclassified | Eubacteriales | Clostridia | Firmicutes |
| Eubacteriales sp.  (HG3A.0322) | ODI | -0.015 | 0.396 | 0.467 | 3364 unclassified | unclassified | unclassified | unclassified | Eubacteriales | Clostridia | Firmicutes |
| Eubacteriales sp. (HG3A.0594) | ODI | -0.015 | 0.395 | 0.467 | 3364 unclassified | unclassified | unclassified | unclassified | Eubacteriales | Clostridia | Firmicutes |
| Eubacteriales sp.  (HG3A.0878) | ODI | 0.015 | 0.395 | 0.467 | 3364 unclassified | unclassified | unclassified | unclassified | Eubacteriales | Clostridia | Firmicutes |
| Eubacteriales sp. (HG3A.0967) | T90 | -0.016 | 0.358 | 0.467 | 3364 unclassified | unclassified | unclassified | unclassified | Eubacteriales | Clostridia | Firmicutes |
| Eubacteriales sp.  (HG3A.0338) | AHI | -0.018 | 0.33 | 0.468 | 3004 unclassified | unclassified | unclassified | unclassified | Eubacteriales | Clostridia | Firmicutes |
| Eubacteriales sp. (HG3A.0654) | T90 | -0.016 | 0.36 | 0.468 | 3364 unclassified | unclassified | unclassified | unclassified | Eubacteriales | Clostridia | Firmicutes |
| Clostridia sp.  (HG3A.1141) | T90 | -0.016 | 0.361 | 0.469 | 3364 unclassified | unclassified | unclassified | unclassified | unclassified | Clostridia | Firmicutes |
| Eubacteriales sp. (HG3A.0137) | AHI | -0.018 | 0.332 | 0.469 | 3004 unclassified | unclassified | unclassified | unclassified | Eubacteriales | Clostridia | Firmicutes |
| Allisonella histaminiformans  (HG3A.0332) | ODI | 0.015 | 0.399 | 0.47 | 3364 unclassified | Allisonella histaminiformans | Allisonella | Veillonellaceae | Veillonellales | Negativicutes | Firmicutes |
| Eubacteriales sp. (HG3A.1250) | T90 | -0.016 | 0.363 | 0.47 | 3364 unclassified | unclassified | unclassified | unclassified | Eubacteriales | Clostridia | Firmicutes |
| Oscillospiraceae sp.  (HG3A.0146) | ODI | -0.015 | 0.401 | 0.471 | 3364 unclassified | unclassified | unclassified | Oscillospiraceae | Eubacteriales | Clostridia | Firmicutes |
| Prevotella colorans (HG3A.1470) | ODI | -0.015 | 0.401 | 0.471 | 3364 unclassified | Prevotella colorans | Prevotella | Prevotellaceae | Bacteroidales | Bacteroidia | Bacteroidetes |

Lacticaseibacillus paracasei subsp. paracasei (HG3A.0853)

Lacticaseibaci

ODI 0.015 0.403 0.473 3364 llus paracasei subsp.

paracasei

Lacticaseibacillus paracasei

Lacticaseibacillus Lactobacillaceae Lactobacillales Bacilli Firmicutes

Tyzzerella nexilis

(HG3A.0574)

ODI

0.015

0.404

0.473 3364 unclassified Tyzzerella nexilis

Tyzzerella

Lachnospiraceae

Eubacteriales

Clostridia

Firmicutes

Bacteria sp.

# (HG3A.1545)

ODI -0.014 0.406 0.475 3364 unclassified unclassified unclassified unclassified unclassified unclassified unclassified

Clostridia sp.

(HG3A.0893)

AHI

-0.018

0.337

0.475 3004 unclassified unclassified

unclassified

unclassified

unclassified

Clostridia

Firmicutes

Clostridiaceae sp.

# (HG3A.0491)

T90 0.016 0.367 0.475 3364 unclassified unclassified unclassified Clostridiaceae Eubacteriales Clostridia Firmicutes

Eubacteriales sp.

(HG3A.1257)

T90

-0.016

0.368

0.475 3364 unclassified unclassified

unclassified

unclassified

Eubacteriales

Clostridia

Firmicutes

Phascolarctobacterium

| succinatutens AHI  (HG3A.0315) | 0.018 | 0.337 | 0.475 | 3004 unclassified | succinatutens | erium | Acidaminococcaceae | Acidaminococcales | Negativicutes | Firmicutes |
| --- | --- | --- | --- | --- | --- | --- | --- | --- | --- | --- |
| Bacteria sp. ODI | -0.014 | 0.409 | 0.477 | 3364 unclassified | unclassified | unclassified | unclassified | unclassified | unclassified | unclassified |
| Eubacteriales sp. ODI | -0.014 | 0.409 | 0.477 | 3364 unclassified | unclassified | unclassified | unclassified | Eubacteriales | Clostridia | Firmicutes |
| Prevotella sp. AHI | 0.018 | 0.339 | 0.478 | 3004 unclassified | unclassified | Prevotella | Prevotellaceae | Bacteroidales | Bacteroidia | Bacteroidetes |

Phascolarctobacterium

Phascolarctobact

# (HG3A.0500) (HG3A.1003)

(HG3A.1009)

Pseudoflavonifractor sp. T90 -0.016 0.371 0.478 3364 unclassified unclassified Pseudoflavonifrac Oscillospiraceae Eubacteriales Clostridia Firmicutes

(HG3A.0844) tor

Roseburia faecis

(HG3A.0058)

T90

0.016

0.372

0.478 3364 unclassified Roseburia faecis

Roseburia

Lachnospiraceae

Eubacteriales

Clostridia

Firmicutes

Agathobaculum

desmolans (HG3A.1429)

Eubacteriales sp.

(HG3A.0093)

ODI

-0.014

0.414

0.479 3364 unclassified unclassified

unclassified

unclassified

Eubacteriales

Clostridia

Firmicutes

ODI 0.014 0.411 0.479 3364 unclassified

Agathobaculum

desmolans Agathobaculum Oscillospiraceae Eubacteriales Clostridia Firmicutes

Eubacteriales sp. (HG3A.1136)

Eubacteriales sp.

(HG3A.1226)

ODI

-0.014

0.414

0.479 3364 unclassified unclassified

unclassified

unclassified

Eubacteriales

Clostridia

Firmicutes

ODI -0.014 0.413 0.479 3364 unclassified unclassified unclassified unclassified Eubacteriales Clostridia Firmicutes

Hungatella hathewayi

(HG3A.0455) AHI 0.018 0.341 0.479 3004 unclassified Hungatella hathewayi Hungatella Clostridiaceae Eubacteriales Clostridia Firmicutes

Eubacteriales sp.

(HG3A.0093)

AHI

-0.017

0.343

0.481 3004 unclassified unclassified

unclassified

unclassified

Eubacteriales

Clostridia

Firmicutes

Eubacteriales sp.

# (HG3A.0472)

AHI -0.017 0.344 0.481 3004 unclassified unclassified unclassified unclassified Eubacteriales Clostridia Firmicutes

| Atopobiaceae sp.  (HG3A.0937) | AHI | 0.017 | 0.348 | 0.485 | 3004 unclassified | unclassified | unclassified | Atopobiaceae | Coriobacteriales | Coriobacteriia | Actinobacteria |
| --- | --- | --- | --- | --- | --- | --- | --- | --- | --- | --- | --- |
| Eubacteriales sp.  (HG3A.0477) | AHI | -0.017 | 0.348 | 0.485 | 3004 unclassified | unclassified | unclassified | unclassified | Eubacteriales | Clostridia | Firmicutes |
| Eubacteriales sp. (HG3A.0736) | AHI | -0.017 | 0.348 | 0.485 | 3004 unclassified | unclassified | unclassified | unclassified | Eubacteriales | Clostridia | Firmicutes |
| Ruminococcus sp. AF17 22AC (HG3A.0208) | T90 | 0.015 | 0.377 | 0.485 | 3364 unclassified | Ruminococcus sp. AF17- 22AC | Ruminococcus | Oscillospiraceae | Eubacteriales | Clostridia | Firmicutes |
| Alistipes communis (HG3A.0064) | T90 | -0.015 | 0.38 | 0.487 | 3364 unclassified | Alistipes communis | Alistipes | Rikenellaceae | Bacteroidales | Bacteroidia | Bacteroidetes |
| Clostridia sp.  (HG3A.1128) | T90 | 0.015 | 0.38 | 0.487 | 3364 unclassified | unclassified | unclassified | unclassified | unclassified | Clostridia | Firmicutes |
| Eubacteriales sp. (HG3A.0694) | T90 | -0.015 | 0.381 | 0.487 | 3364 unclassified | unclassified | unclassified | unclassified | Eubacteriales | Clostridia | Firmicutes |
| Eubacteriales sp.  (HG3A.0851) | AHI | -0.017 | 0.35 | 0.487 | 3004 unclassified | unclassified | unclassified | unclassified | Eubacteriales | Clostridia | Firmicutes |
| Massilistercora timonensis (HG3A.0458) | T90 | -0.015 | 0.38 | 0.487 | 3364 unclassified | Massilistercora timonensis | Massilistercora | unclassified | Eubacteriales | Clostridia | Firmicutes |
| Clostridia sp.  (HG3A.0933) | AHI | -0.017 | 0.353 | 0.489 | 3004 unclassified | unclassified | unclassified | unclassified | unclassified | Clostridia | Firmicutes |
| Eubacteriales sp. (HG3A.0328) | AHI | -0.017 | 0.352 | 0.489 | 3004 unclassified | unclassified | unclassified | unclassified | Eubacteriales | Clostridia | Firmicutes |
| Alistipes dispar  (HG3A.0281) | AHI | -0.017 | 0.357 | 0.491 | 3004 unclassified | Alistipes dispar | Alistipes | Rikenellaceae | Bacteroidales | Bacteroidia | Bacteroidetes |
| Clostridia sp. (HG3A.0401) | AHI | -0.017 | 0.356 | 0.491 | 3004 unclassified | unclassified | unclassified | unclassified | unclassified | Clostridia | Firmicutes |
| Clostridia sp.  (HG3A.0519) | T90 | -0.015 | 0.386 | 0.491 | 3364 unclassified | unclassified | unclassified | unclassified | unclassified | Clostridia | Firmicutes |
| Clostridia sp. (HG3A.0750) | T90 | -0.015 | 0.386 | 0.491 | 3364 unclassified | unclassified | unclassified | unclassified | unclassified | Clostridia | Firmicutes |
| Eubacteriales sp.  (HG3A.0439) | T90 | -0.015 | 0.385 | 0.491 | 3364 unclassified | unclassified | unclassified | unclassified | Eubacteriales | Clostridia | Firmicutes |
| Eubacteriales sp. (HG3A.1354) | AHI | 0.017 | 0.356 | 0.491 | 3004 unclassified | unclassified | unclassified | unclassified | Eubacteriales | Clostridia | Firmicutes |
| Eubacteriales sp.  (HG3A.0392) | AHI | -0.017 | 0.358 | 0.492 | 3004 unclassified | unclassified | unclassified | unclassified | Eubacteriales | Clostridia | Firmicutes |
| Eubacteriales sp. (HG3A.0671) | AHI | -0.017 | 0.359 | 0.492 | 3004 unclassified | unclassified | unclassified | unclassified | Eubacteriales | Clostridia | Firmicutes |
| Clostridia sp.  (HG3A.1252) | AHI | -0.017 | 0.36 | 0.493 | 3004 unclassified | unclassified | unclassified | unclassified | unclassified | Clostridia | Firmicutes |
| Eubacteriales sp. (HG3A.0878) | AHI | 0.017 | 0.36 | 0.493 | 3004 unclassified | unclassified | unclassified | unclassified | Eubacteriales | Clostridia | Firmicutes |

Clostridium sp. AF37-5

(HG3A.0076) T90 -0.015 0.39 0.494 3364 unclassified Clostridium sp. AF37-5 Clostridium Clostridiaceae Eubacteriales Clostridia Firmicutes

| Eggerthellaceae sp. (HG3A.0171) | T90 | -0.015 | 0.39 | 0.494 | 3364 unclassified | unclassified | unclassified | Eggerthellaceae | Eggerthellales | Coriobacteriia | Actinobacteria |
| --- | --- | --- | --- | --- | --- | --- | --- | --- | --- | --- | --- |
| Prevotella sp.  (HG3A.1009) | T90 | 0.015 | 0.39 | 0.494 | 3364 unclassified | unclassified | Prevotella | Prevotellaceae | Bacteroidales | Bacteroidia | Bacteroidetes |
| Eubacteriales sp. (HG3A.0439) | AHI | -0.017 | 0.362 | 0.495 | 3004 unclassified | unclassified | unclassified | unclassified | Eubacteriales | Clostridia | Firmicutes |
| Clostridia sp.  (HG3A.1493) | T90 | -0.015 | 0.393 | 0.497 | 3364 unclassified | unclassified | unclassified | unclassified | unclassified | Clostridia | Firmicutes |
| Oscillospiraceae sp. (HG3A.0849) | ODI | -0.014 | 0.43 | 0.497 | 3364 unclassified | unclassified | unclassified | Oscillospiraceae | Eubacteriales | Clostridia | Firmicutes |
| Eubacteriales sp.  (HG3A.0505) | AHI | -0.017 | 0.366 | 0.499 | 3004 unclassified | unclassified | unclassified | unclassified | Eubacteriales | Clostridia | Firmicutes |
| Eubacteriales sp. (HG3A.0950) | AHI | -0.017 | 0.366 | 0.499 | 3004 unclassified | unclassified | unclassified | unclassified | Eubacteriales | Clostridia | Firmicutes |
| Eubacteriaceae sp.  (HG3A.0591) | AHI | 0.017 | 0.37 | 0.503 | 3004 unclassified | unclassified | unclassified | Eubacteriaceae | Eubacteriales | Clostridia | Firmicutes |
| Eubacteriales sp. (HG3A.0617) | T90 | -0.015 | 0.4 | 0.504 | 3364 unclassified | unclassified | unclassified | unclassified | Eubacteriales | Clostridia | Firmicutes |
| Clostridia sp.  (HG3A.0706) | AHI | -0.016 | 0.373 | 0.505 | 3004 unclassified | unclassified | unclassified | unclassified | unclassified | Clostridia | Firmicutes |
| Eubacteriales sp. (HG3A.0514) | AHI | -0.016 | 0.373 | 0.505 | 3004 unclassified | unclassified | unclassified | unclassified | Eubacteriales | Clostridia | Firmicutes |
| Eubacteriales sp.  (HG3A.0593) | AHI | -0.016 | 0.372 | 0.505 | 3004 unclassified | unclassified | unclassified | unclassified | Eubacteriales | Clostridia | Firmicutes |
| Clostridia sp. (HG3A.0845) | ODI | -0.013 | 0.438 | 0.507 | 3364 unclassified | unclassified | unclassified | unclassified | unclassified | Clostridia | Firmicutes |
| Eubacteriales sp.  (HG3A.0851) | T90 | -0.015 | 0.404 | 0.509 | 3364 unclassified | unclassified | unclassified | unclassified | Eubacteriales | Clostridia | Firmicutes |
| Bacteria sp. (HG3A.1096) | AHI | -0.016 | 0.379 | 0.51 | 3004 unclassified | unclassified | unclassified | unclassified | unclassified | unclassified | unclassified |
| Clostridia sp.  (HG3A.1035) | ODI | -0.013 | 0.442 | 0.51 | 3364 unclassified | unclassified | unclassified | unclassified | unclassified | Clostridia | Firmicutes |
| Eubacteriales sp. (HG3A.0744) | AHI | -0.016 | 0.378 | 0.51 | 3004 unclassified | unclassified | unclassified | unclassified | Eubacteriales | Clostridia | Firmicutes |
| Senegalimassilia  anaerobia (HG3A.0129) | AHI | 0.016 | 0.378 | 0.51 | 3004 unclassified | Senegalimassilia anaerobia | Senegalimassilia | Coriobacteriaceae | Coriobacteriales | Coriobacteriia | Actinobacteria |
| Bacteroides caccae (HG3A.0066) | T90 | -0.014 | 0.41 | 0.511 | 3364 unclassified | Bacteroides caccae | Bacteroides | Bacteroidaceae | Bacteroidales | Bacteroidia | Bacteroidetes |

Candidatus Borkfalkiales sp. (HG3A.1284)

| Clostridia sp. (HG3A.0996) | T90 | -0.014 | 0.409 | 0.511 | 3364 unclassified | unclassified | unclassified | unclassified | unclassified | Clostridia | Firmicutes |
| --- | --- | --- | --- | --- | --- | --- | --- | --- | --- | --- | --- |
| Eubacteriales sp.  (HG3A.0288) | T90 | -0.014 | 0.409 | 0.511 | 3364 unclassified | unclassified | unclassified | unclassified | Eubacteriales | Clostridia | Firmicutes |
| Eubacteriales sp. (HG3A.0691) | T90 | -0.014 | 0.407 | 0.511 | 3364 unclassified | unclassified | unclassified | unclassified | Eubacteriales | Clostridia | Firmicutes |
| Eubacteriales sp.  (HG3A.0792) | T90 | -0.014 | 0.408 | 0.511 | 3364 unclassified | unclassified | unclassified | unclassified | Eubacteriales | Clostridia | Firmicutes |
| Eubacteriales sp. (HG3A.0701) | AHI | -0.016 | 0.382 | 0.513 | 3004 unclassified | unclassified | unclassified | unclassified | Eubacteriales | Clostridia | Firmicutes |
| Eubacteriales sp.  (HG3A.0547) | AHI | 0.016 | 0.383 | 0.514 | 3004 unclassified | unclassified | unclassified | unclassified | Eubacteriales | Clostridia | Firmicutes |
| Agathobaculum desmolans (HG3A.1429) | AHI | 0.016 | 0.385 | 0.515 | 3004 unclassified | Agathobaculum desmolans | Agathobaculum | Oscillospiraceae | Eubacteriales | Clostridia | Firmicutes |
| Eubacteriales sp.  (HG3A.0447) | T90 | -0.014 | 0.414 | 0.515 | 3364 unclassified | unclassified | unclassified | unclassified | Eubacteriales | Clostridia | Firmicutes |
| Eubacteriales sp. (HG3A.0939) | T90 | -0.014 | 0.414 | 0.515 | 3364 unclassified | unclassified | unclassified | unclassified | Eubacteriales | Clostridia | Firmicutes |
| Eubacteriales sp.  (HG3A.1067) | AHI | -0.016 | 0.387 | 0.516 | 3004 unclassified | unclassified | unclassified | unclassified | Eubacteriales | Clostridia | Firmicutes |
| Eubacteriales sp. (HG3A.0647) | ODI | -0.013 | 0.448 | 0.517 | 3364 unclassified | unclassified | unclassified | unclassified | Eubacteriales | Clostridia | Firmicutes |
| Eubacteriales sp.  (HG3A.1177) | ODI | -0.013 | 0.45 | 0.517 | 3364 unclassified | unclassified | unclassified | unclassified | Eubacteriales | Clostridia | Firmicutes |
| Firmicutes sp. (HG3A.1082) | AHI | -0.016 | 0.388 | 0.518 | 3004 unclassified | unclassified | unclassified | unclassified | unclassified | unclassified | Firmicutes |
| Eubacteriales sp.  (HG3A.0663) | T90 | -0.014 | 0.418 | 0.519 | 3364 unclassified | unclassified | unclassified | unclassified | Eubacteriales | Clostridia | Firmicutes |
| Lachnospiraceae sp. (HG3A.0903) | T90 | -0.014 | 0.42 | 0.52 | 3364 unclassified | unclassified | unclassified | Lachnospiraceae | Eubacteriales | Clostridia | Firmicutes |
| Ruminococcus sp.  AM42-11 (HG3A.0002) | AHI | 0.016 | 0.393 | 0.522 | 3004 unclassified | Ruminococcus sp. AM42- 11 | Ruminococcus | Oscillospiraceae | Eubacteriales | Clostridia | Firmicutes |
| Tyzzerella nexilis (HG3A.0574) | AHI | 0.016 | 0.393 | 0.522 | 3004 unclassified | Tyzzerella nexilis | Tyzzerella | Lachnospiraceae | Eubacteriales | Clostridia | Firmicutes |
| Actinomyces sp. ICM58  (HG3A.0410) | T90 | -0.014 | 0.423 | 0.523 | 3364 unclassified | Actinomyces sp. ICM58 | Actinomyces | Actinomycetaceae | Actinomycetales | Actinomycetia | Actinobacteria |
| Clostridia sp. (HG3A.1403) | T90 | -0.014 | 0.424 | 0.523 | 3364 unclassified | unclassified | unclassified | unclassified | unclassified | Clostridia | Firmicutes |

T90 -0.014 0.409 0.511 3364 unclassified unclassified unclassified unclassified

Candidatus

Borkfalkiales Clostridia Firmicutes

Eubacteriales sp. (HG3A.0490)

Eubacteriales sp.

(HG3A.1439)

AHI

0.016

0.395

0.524 3004 unclassified unclassified

unclassified

unclassified

Eubacteriales

Clostridia

Firmicutes

ODI -0.013 0.455 0.523 3364 unclassified unclassified unclassified unclassified Eubacteriales Clostridia Firmicutes

Eubacteriales sp. (HG3A.1187)

Clostridia sp.

(HG3A.1410)

T90

-0.014

0.427

0.526 3364 unclassified unclassified

unclassified

unclassified

unclassified

Clostridia

Firmicutes

AHI 0.016 0.396 0.525 3004 unclassified unclassified unclassified unclassified Eubacteriales Clostridia Firmicutes

Eubacteriales sp. (HG3A.0821)

Clostridia sp.

(HG3A.1058)

AHI

-0.016

0.4

0.527 3004 unclassified unclassified

unclassified

unclassified

unclassified

Clostridia

Firmicutes

T90 -0.014 0.427 0.526 3364 unclassified unclassified unclassified unclassified Eubacteriales Clostridia Firmicutes

Eubacteriales sp. (HG3A.0192)

Eubacteriales sp.

(HG3A.0543)

AHI

-0.016

0.398

0.527 3004 unclassified unclassified

unclassified

unclassified

Eubacteriales

Clostridia

Firmicutes

AHI -0.016 0.4 0.527 3004 unclassified unclassified unclassified unclassified Eubacteriales Clostridia Firmicutes

Ligilactobacillus

salivarius (HG3A.0919) ODI 0.013 0.46 0.528 3364 unclassified Ligilactobacillus salivarius Ligilactobacillus Lactobacillaceae Lactobacillales Bacilli Firmicutes

Clostridium sp. AF15-31

(HG3A.0293)

ODI

-0.013

0.463

0.53 3364 unclassified Clostridium sp. AF15-31 Clostridium

Clostridiaceae

Eubacteriales

Clostridia

Firmicutes

Eubacteriales sp.

# (HG3A.0502)

T90 -0.014 0.431 0.53 3364 unclassified unclassified unclassified unclassified Eubacteriales Clostridia Firmicutes

Eubacteriales sp.

(HG3A.0762)

ODI

-0.013

0.463

0.53 3364 unclassified unclassified

unclassified

unclassified

Eubacteriales

Clostridia

Firmicutes

Eubacteriales sp.

# (HG3A.0820)

ODI -0.013 0.464 0.53 3364 unclassified unclassified unclassified unclassified Eubacteriales Clostridia Firmicutes

Eubacteriales sp.

(HG3A.0244)

ODI

-0.013

0.465

0.531 3364 unclassified unclassified

unclassified

unclassified

Eubacteriales

Clostridia

Firmicutes

Eubacteriales sp.

# (HG3A.0573)

T90 -0.014 0.433 0.531 3364 unclassified unclassified unclassified unclassified Eubacteriales Clostridia Firmicutes

Eubacteriales sp.

(HG3A.0594)

T90

-0.014

0.434

0.531 3364 unclassified unclassified

unclassified

unclassified

Eubacteriales

Clostridia

Firmicutes

Eubacteriales sp.

# (HG3A.1126)

T90 -0.014 0.433 0.531 3364 unclassified unclassified unclassified unclassified Eubacteriales Clostridia Firmicutes

Eubacteriales sp.

(HG3A.0087)

T90

0.014

0.436

0.532 3364 unclassified unclassified

unclassified

unclassified

Eubacteriales

Clostridia

Firmicutes

Pediococcus

pentosaceus (HG3A.1246)

AHI 0.015 0.404 0.532 3004 unclassified Pediococcus pentosaceus Pediococcus Lactobacillaceae Lactobacillales Bacilli Firmicutes

| Fusobacterium Fusobacteriu  nucleatum subsp. ODI 0.013 0.47 0.535 3364 m nucleatum Fusobacterium nucleatum Fusobacterium Fusobacteriaceae Fusobacteriales Fusobacteriia Fusobacteria animalis (HG3A.1418) subsp.  animalis | | | | | | | | | | | |
| --- | --- | --- | --- | --- | --- | --- | --- | --- | --- | --- | --- |
| Blautia sp. AF19-10LB  (HG3A.0157) | T90 | -0.013 | 0.44 | 0.536 | 3364 unclassified | Blautia sp. AF19-10LB | Blautia | Lachnospiraceae | Eubacteriales | Clostridia | Firmicutes |
| Eubacteriales sp. (HG3A.0614) | AHI | -0.015 | 0.408 | 0.536 | 3004 unclassified | unclassified | unclassified | unclassified | Eubacteriales | Clostridia | Firmicutes |
| Ruminococcus sp.  (HG3A.0126) | T90 | -0.013 | 0.441 | 0.537 | 3364 unclassified | unclassified | Ruminococcus | Oscillospiraceae | Eubacteriales | Clostridia | Firmicutes |
| Oscillospiraceae sp. (HG3A.0343) | AHI | -0.015 | 0.41 | 0.538 | 3004 unclassified | unclassified | unclassified | Oscillospiraceae | Eubacteriales | Clostridia | Firmicutes |
| Clostridia sp.  (HG3A.1220) | ODI | -0.012 | 0.476 | 0.54 | 3364 unclassified | unclassified | unclassified | unclassified | unclassified | Clostridia | Firmicutes |
| Clostridium sp. OF03- 18AA (HG3A.0119) | ODI | -0.012 | 0.475 | 0.54 | 3364 unclassified | Clostridium sp. OF03- 18AA | Clostridium | Clostridiaceae | Eubacteriales | Clostridia | Firmicutes |
| Clostridium sp. OM07- 9AC (HG3A.0448) | ODI | -0.012 | 0.477 | 0.54 | 3364 unclassified | Clostridium sp. OM07- 9AC | Clostridium | Clostridiaceae | Eubacteriales | Clostridia | Firmicutes |
| Eubacteriales sp. (HG3A.0468) | AHI | -0.015 | 0.413 | 0.54 | 3004 unclassified | unclassified | unclassified | unclassified | Eubacteriales | Clostridia | Firmicutes |
| Firmicutes sp.  (HG3A.1014) | AHI | 0.015 | 0.413 | 0.54 | 3004 unclassified | unclassified | unclassified | unclassified | unclassified | unclassified | Firmicutes |
| Anaerobutyricum hallii (HG3A.0012) | AHI | 0.015 | 0.417 | 0.543 | 3004 unclassified | Anaerobutyricum hallii | Anaerobutyricum | Lachnospiraceae | Eubacteriales | Clostridia | Firmicutes |
| Eubacteriales sp.  (HG3A.0493) | AHI | -0.015 | 0.418 | 0.543 | 3004 unclassified | unclassified | unclassified | unclassified | Eubacteriales | Clostridia | Firmicutes |
| Eubacteriales sp. (HG3A.0754) | AHI | -0.015 | 0.417 | 0.543 | 3004 unclassified | unclassified | unclassified | unclassified | Eubacteriales | Clostridia | Firmicutes |
| Streptococcus parasanguinis  (HG3A.0117) | AHI | 0.015 | 0.418 | 0.543 | 3004 unclassified | Streptococcus parasanguinis | Streptococcus | Streptococcaceae | Lactobacillales | Bacilli | Firmicutes |
| Oscillibacter sp. (HG3A.0243) | T90 | -0.013 | 0.448 | 0.545 | 3364 unclassified | unclassified | Oscillibacter | Oscillospiraceae | Eubacteriales | Clostridia | Firmicutes |
| Oscillospiraceae sp.  (HG3A.0612) | T90 | -0.013 | 0.448 | 0.545 | 3364 unclassified | unclassified | unclassified | Oscillospiraceae | Eubacteriales | Clostridia | Firmicutes |
| Bacteroidales sp. (HG3A.0147) | ODI | -0.012 | 0.483 | 0.547 | 3364 unclassified | unclassified | unclassified | unclassified | Bacteroidales | Bacteroidia | Bacteroidetes |
| Clostridia sp.  (HG3A.0564) | T90 | -0.013 | 0.453 | 0.548 | 3364 unclassified | unclassified | unclassified | unclassified | unclassified | Clostridia | Firmicutes |
| Eubacteriales sp. (HG3A.0717) | T90 | -0.013 | 0.452 | 0.548 | 3364 unclassified | unclassified | unclassified | unclassified | Eubacteriales | Clostridia | Firmicutes |

Clostridia sp. (HG3A.0783)

AHI -0.015 0.424 0.549 3004 unclassified unclassified unclassified unclassified unclassified Clostridia Firmicutes

| Clostridia sp. (HG3A.1356) | AHI | -0.015 | 0.424 | 0.549 | 3004 unclassified | unclassified | unclassified | unclassified | unclassified | Clostridia | Firmicutes |
| --- | --- | --- | --- | --- | --- | --- | --- | --- | --- | --- | --- |
| Eubacteriales sp.  (HG3A.1393) | ODI | -0.012 | 0.485 | 0.549 | 3364 unclassified | unclassified | unclassified | unclassified | Eubacteriales | Clostridia | Firmicutes |
| Eubacteriales sp. (HG3A.0535) | ODI | -0.012 | 0.488 | 0.551 | 3364 unclassified | unclassified | unclassified | unclassified | Eubacteriales | Clostridia | Firmicutes |
| Eubacteriales sp.  (HG3A.0792) | ODI | -0.012 | 0.489 | 0.551 | 3364 unclassified | unclassified | unclassified | unclassified | Eubacteriales | Clostridia | Firmicutes |
| Actinomycetaceae sp. (HG3A.1068) | AHI | -0.015 | 0.427 | 0.552 | 3004 unclassified | unclassified | unclassified | Actinomycetaceae | Actinomycetales | Actinomycetia | Actinobacteria |
| Clostridiaceae sp.  (HG3A.0471) | AHI | 0.014 | 0.432 | 0.555 | 3004 unclassified | unclassified | unclassified | Clostridiaceae | Eubacteriales | Clostridia | Firmicutes |
| Eubacteriales sp. (HG3A.0273) | AHI | -0.015 | 0.432 | 0.555 | 3004 unclassified | unclassified | unclassified | unclassified | Eubacteriales | Clostridia | Firmicutes |
| Eubacteriales sp.  (HG3A.0610) | AHI | 0.015 | 0.431 | 0.555 | 3004 unclassified | unclassified | unclassified | unclassified | Eubacteriales | Clostridia | Firmicutes |
| Clostridiaceae sp. (HG3A.0471) | ODI | -0.012 | 0.495 | 0.556 | 3364 unclassified | unclassified | unclassified | Clostridiaceae | Eubacteriales | Clostridia | Firmicutes |
| Clostridium perfringens (HG3A.0959) | AHI | 0.014 | 0.434 | 0.556 | 3004 unclassified | Clostridium perfringens | Clostridium | Clostridiaceae | Eubacteriales | Clostridia | Firmicutes |
| Enterocloster sp. (HG3A.1529) | ODI | 0.012 | 0.494 | 0.556 | 3364 unclassified | unclassified | Enterocloster | Lachnospiraceae | Eubacteriales | Clostridia | Firmicutes |
| Eubacteriales sp.  (HG3A.0358) | AHI | -0.014 | 0.433 | 0.556 | 3004 unclassified | unclassified | unclassified | unclassified | Eubacteriales | Clostridia | Firmicutes |
| Eubacteriales sp. (HG3A.1285) | T90 | -0.013 | 0.46 | 0.556 | 3364 unclassified | unclassified | unclassified | unclassified | Eubacteriales | Clostridia | Firmicutes |
| Eubacteriales sp.  (HG3A.0610) | ODI | 0.012 | 0.498 | 0.559 | 3364 unclassified | unclassified | unclassified | unclassified | Eubacteriales | Clostridia | Firmicutes |
| Eubacteriales sp. (HG3A.0392) | T90 | -0.013 | 0.467 | 0.56 | 3364 unclassified | unclassified | unclassified | unclassified | Eubacteriales | Clostridia | Firmicutes |
| Eubacteriales sp.  (HG3A.0790) | AHI | -0.014 | 0.438 | 0.56 | 3004 unclassified | unclassified | unclassified | unclassified | Eubacteriales | Clostridia | Firmicutes |
| Eubacteriales sp. (HG3A.1094) | T90 | -0.013 | 0.466 | 0.56 | 3364 unclassified | unclassified | unclassified | unclassified | Eubacteriales | Clostridia | Firmicutes |
| Lachnospiraceae sp.  (HG3A.0899) | T90 | -0.013 | 0.466 | 0.56 | 3364 unclassified | unclassified | unclassified | Lachnospiraceae | Eubacteriales | Clostridia | Firmicutes |
| Lachnospiraceae sp. (HG3A.1641) | T90 | 0.013 | 0.468 | 0.56 | 3364 unclassified | unclassified | unclassified | Lachnospiraceae | Eubacteriales | Clostridia | Firmicutes |

Rothia mucilaginosa

# (HG3A.0559)

T90 0.013 0.468 0.56 3364 unclassified Rothia mucilaginosa Rothia Micrococcaceae Micrococcales Actinomycetia Actinobacteria

# (HG3A.0217)

| Sutterella seckii T90 (HG3A.0561) | | 0.013 | 0.464 | 0.56 | 3364 unclassified | Sutterella seckii | Sutterella | Sutterellaceae | Burkholderiales Betaproteobacteri Proteobacteria  a | | |
| --- | --- | --- | --- | --- | --- | --- | --- | --- | --- | --- | --- |
| Lachnospiraceae sp. AHI | | -0.014 | 0.44 | 0.561 | 3004 unclassified | unclassified | unclassified | Lachnospiraceae | Eubacteriales Clostridia Firmicutes | | |
| Bifidobacterium animalis subsp. lactis | AHI | -0.014 | 0.442 | 0.562 | Bifidobacteriu 3004 m animalis | Bifidobacterium animalis | Bifidobacterium | Bifidobacteriaceae | Bifidobacteriales | Actinomycetia | Actinobacteria |
| (HG3A.0513) subsp. lactis | | | | | | | | | | | |

Desulfovibrio fairfieldensis (HG3A.0529)

Oscillibacter sp.

(HG3A.0243)

AHI

-0.014

0.442

0.562 3004 unclassified unclassified

Oscillibacter

Oscillospiraceae

Eubacteriales

Clostridia

Firmicutes

AHI -0.014 0.442 0.562 3004 unclassified

Desulfovibrio

fairfieldensis Desulfovibrio Desulfovibrionaceae Desulfovibrionales

Deltaproteobacte

ria Proteobacteria

Lachnospiraceae sp. (HG3A.0217)

Latilactobacillus

curvatus (HG3A.1505)

T90

0.013

0.471

0.563 3364 unclassified Latilactobacillus curvatus Latilactobacillus Lactobacillaceae

Lactobacillales

Bacilli

Firmicutes

ODI -0.012 0.502 0.563 3364 unclassified unclassified unclassified Lachnospiraceae Eubacteriales Clostridia Firmicutes

Clostridia sp. (HG3A.0401)

Eubacteriales sp.

(HG3A.0791)

ODI

-0.012

0.508

0.568 3364 unclassified unclassified

unclassified

unclassified

Eubacteriales

Clostridia

Firmicutes

ODI -0.012 0.508 0.568 3364 unclassified unclassified unclassified unclassified unclassified Clostridia Firmicutes

Eubacteriales sp. (HG3A.0973)

Akkermansia muciniphila

(HG3A.0110)

AHI

-0.014

0.45

0.571 3004 unclassified Akkermansia muciniphila Akkermansia Akkermansiaceae

Verrucomicrobiales e

Verrucomicrobia Verrucomicrob

ia

ODI -0.011 0.51 0.569 3364 unclassified unclassified unclassified unclassified Eubacteriales Clostridia Firmicutes

Akkermansia sp. BIOML

Akkermansia sp. BIOML-

Verrucomicrobia Verrucomicrob

A59 (HG3A.0800) T90 -0.012 0.48 0.571 3364 unclassified A59 Akkermansia Akkermansiaceae Verrucomicrobiales e ia

Clostridia sp.

(HG3A.1038)

T90

-0.012

0.481

0.571 3364 unclassified unclassified

unclassified

unclassified

unclassified

Clostridia

Firmicutes

Clostridia sp.

# (HG3A.1058)

T90 -0.012 0.48 0.571 3364 unclassified unclassified unclassified unclassified unclassified Clostridia Firmicutes

Eubacteriales sp.

(HG3A.0081)

T90

-0.012

0.479

0.571 3364 unclassified unclassified

unclassified

unclassified

Eubacteriales

Clostridia

Firmicutes

Eubacteriales sp.

# (HG3A.0551)

T90 -0.012 0.48 0.571 3364 unclassified unclassified unclassified unclassified Eubacteriales Clostridia Firmicutes

Pseudoflavonifractor sp.

Pseudoflavonifractor sp. Pseudoflavonifrac

# (HG3A.1439) (HG3A.1546)

| An184 (HG3A.0253) AHI | -0.014 | 0.45 | 0.571 | 3004 unclassified | An184 | tor | Oscillospiraceae | Eubacteriales | Clostridia | Firmicutes |
| --- | --- | --- | --- | --- | --- | --- | --- | --- | --- | --- |
| Eubacteriales sp. T90 | -0.012 | 0.483 | 0.572 | 3364 unclassified | unclassified | unclassified | unclassified | Eubacteriales | Clostridia | Firmicutes |
| Eubacteriales sp. ODI | -0.011 | 0.513 | 0.572 | 3364 unclassified | unclassified | unclassified | unclassified | Eubacteriales | Clostridia | Firmicutes |

Flavonifractor sp. An10 (HG3A.0495)

AHI -0.014 0.452 0.572 3004 unclassified Flavonifractor sp. An10 Flavonifractor Oscillospiraceae Eubacteriales Clostridia Firmicutes

| Eubacteriales sp. (HG3A.0820) | T90 | -0.012 | 0.485 | 0.574 | 3364 unclassified | unclassified | unclassified | unclassified | Eubacteriales | Clostridia | Firmicutes |
| --- | --- | --- | --- | --- | --- | --- | --- | --- | --- | --- | --- |
| Eubacteriales sp.  (HG3A.0857) | AHI | -0.014 | 0.457 | 0.577 | 3004 unclassified | unclassified | unclassified | unclassified | Eubacteriales | Clostridia | Firmicutes |
| Eubacteriales sp. (HG3A.0178) | AHI | -0.014 | 0.458 | 0.578 | 3004 unclassified | unclassified | unclassified | unclassified | Eubacteriales | Clostridia | Firmicutes |
| Eubacteriales sp.  (HG3A.0511) | AHI | -0.014 | 0.459 | 0.578 | 3004 unclassified | unclassified | unclassified | unclassified | Eubacteriales | Clostridia | Firmicutes |
| Eubacteriales sp. (HG3A.1573) | T90 | -0.012 | 0.489 | 0.578 | 3364 unclassified | unclassified | unclassified | unclassified | Eubacteriales | Clostridia | Firmicutes |
| Eubacteriales sp.  (HG3A.0244) | AHI | -0.014 | 0.462 | 0.579 | 3004 unclassified | unclassified | unclassified | unclassified | Eubacteriales | Clostridia | Firmicutes |
| Intestinimonas sp. (HG3A.1018) | AHI | 0.014 | 0.46 | 0.579 | 3004 unclassified | unclassified | Intestinimonas | unclassified | Eubacteriales | Clostridia | Firmicutes |
| Lachnospiraceae sp.  (HG3A.1155) | AHI | -0.014 | 0.462 | 0.579 | 3004 unclassified | unclassified | unclassified | Lachnospiraceae | Eubacteriales | Clostridia | Firmicutes |
| Bacteria sp. (HG3A.0459) | T90 | -0.012 | 0.491 | 0.58 | 3364 unclassified | unclassified | unclassified | unclassified | unclassified | unclassified | unclassified |
| Lachnospiraceae sp.  (HG3A.0252) | ODI | -0.011 | 0.521 | 0.58 | 3364 unclassified | unclassified | unclassified | Lachnospiraceae | Eubacteriales | Clostridia | Firmicutes |
| Levilactobacillus brevis (HG3A.1848) | T90 | 0.012 | 0.492 | 0.58 | 3364 unclassified | Levilactobacillus brevis | Levilactobacillus | Lactobacillaceae | Lactobacillales | Bacilli | Firmicutes |
| Eubacteriales sp.  (HG3A.0345) | AHI | -0.013 | 0.465 | 0.581 | 3004 unclassified | unclassified | unclassified | unclassified | Eubacteriales | Clostridia | Firmicutes |
| Streptococcus gordonii (HG3A.0713) | AHI | 0.013 | 0.465 | 0.581 | 3004 unclassified | Streptococcus gordonii | Streptococcus | Streptococcaceae | Lactobacillales | Bacilli | Firmicutes |
| Eubacteriales sp.  (HG3A.0786) | AHI | 0.013 | 0.466 | 0.582 | 3004 unclassified | unclassified | unclassified | unclassified | Eubacteriales | Clostridia | Firmicutes |
| Eubacteriales sp. (HG3A.0692) | AHI | 0.013 | 0.47 | 0.585 | 3004 unclassified | unclassified | unclassified | unclassified | Eubacteriales | Clostridia | Firmicutes |
| Eubacteriales sp.  (HG3A.0158) | T90 | -0.012 | 0.498 | 0.586 | 3364 unclassified | unclassified | unclassified | unclassified | Eubacteriales | Clostridia | Firmicutes |
| Bacteroides nordii (HG3A.0290) | T90 | -0.012 | 0.5 | 0.587 | 3364 unclassified | Bacteroides nordii | Bacteroides | Bacteroidaceae | Bacteroidales | Bacteroidia | Bacteroidetes |
| Lachnospiraceae sp.  (HG3A.1641) | ODI | 0.011 | 0.528 | 0.587 | 3364 unclassified | unclassified | unclassified | Lachnospiraceae | Eubacteriales | Clostridia | Firmicutes |
| Dorea longicatena (HG3A.0039) | AHI | 0.013 | 0.473 | 0.588 | 3004 unclassified | Dorea longicatena | Dorea | Lachnospiraceae | Eubacteriales | Clostridia | Firmicutes |

Clostridium perfringens

(HG3A.0959) ODI 0.011 0.532 0.59 3364 unclassified Clostridium perfringens Clostridium Clostridiaceae Eubacteriales Clostridia Firmicutes

Intestinimonas sp.

(HG3A.1018)

ODI

0.011

0.532

0.59 3364 unclassified unclassified

Intestinimonas unclassified

Eubacteriales

Clostridia

Firmicutes

Clostridia sp.

# (HG3A.1039)

T90 -0.012 0.504 0.591 3364 unclassified unclassified unclassified unclassified unclassified Clostridia Firmicutes

Eggerthellales sp.

(HG3A.0848)

T90

-0.012

0.507

0.593 3364 unclassified unclassified

unclassified

unclassified

Eggerthellales

Coriobacteriia Actinobacteria

Eubacteriales sp.

# (HG3A.0477)

T90 -0.012 0.507 0.593 3364 unclassified unclassified unclassified unclassified Eubacteriales Clostridia Firmicutes

Eubacteriales sp.

(HG3A.0544)

AHI

0.013

0.479

0.594 3004 unclassified unclassified

unclassified

unclassified

Eubacteriales

Clostridia

Firmicutes

Clostridia sp.

# (HG3A.0845)

AHI -0.013 0.482 0.596 3004 unclassified unclassified unclassified unclassified unclassified Clostridia Firmicutes

Eubacteriales sp.

(HG3A.0376)

AHI

-0.013

0.482

0.596 3004 unclassified unclassified

unclassified

unclassified

Eubacteriales

Clostridia

Firmicutes

Oscillospiraceae sp.

# (HG3A.0944)

ODI -0.011 0.541 0.598 3364 unclassified unclassified unclassified Oscillospiraceae Eubacteriales Clostridia Firmicutes

Sutterella wadsworthensis

(HG3A.0143)

ODI

-0.011

0.542

0.598 3364 unclassified Sutterella wadsworthensis Sutterella

Sutterellaceae

Burkholderiales

Betaproteobacteri

a

Proteobacteria

Oscillospiraceae sp.

# (HG3A.0805)

AHI -0.013 0.485 0.599 3004 unclassified unclassified unclassified Oscillospiraceae Eubacteriales Clostridia Firmicutes

Eubacteriales sp.

(HG3A.0369)

AHI

-0.013

0.487

0.6 3004 unclassified unclassified

unclassified

unclassified

Eubacteriales

Clostridia

Firmicutes

Eubacteriales sp.

# (HG3A.1445)

T90 -0.011 0.514 0.6 3364 unclassified unclassified unclassified unclassified Eubacteriales Clostridia Firmicutes

Faecalibacterium

prausnitzii (HG3A.0241) ODI

-0.011

0.544

0.6 3364 unclassified

Faecalibacterium

prausnitzii

Faecalibacterium Oscillospiraceae

Eubacteriales

Clostridia

Firmicutes

Candidatus Borkfalkia ceftriaxoniphila (HG3A.0595)

AHI -0.013 0.492 0.601 3004 unclassified Candidatus Borkfalkia

ceftriaxoniphila

Candidatus Borkfalkia

Candidatus Borkfalkiaceae

Candidatus Borkfalkiales

Clostridia Firmicutes

Eubacteriales sp.

(HG3A.0289)

AHI

-0.013

0.49

0.601 3004 unclassified unclassified

unclassified

unclassified

Eubacteriales

Clostridia

Firmicutes

Eubacteriales sp.

# (HG3A.0447)

AHI -0.013 0.489 0.601 3004 unclassified unclassified unclassified unclassified Eubacteriales Clostridia Firmicutes

Eubacteriales sp.

(HG3A.0652)

AHI

-0.013

0.491

0.601 3004 unclassified unclassified

unclassified

unclassified

Eubacteriales

Clostridia

Firmicutes

Eubacteriales sp.

# (HG3A.1546)

AHI -0.013 0.491 0.601 3004 unclassified unclassified unclassified unclassified Eubacteriales Clostridia Firmicutes

Firmicutes sp.

(HG3A.1162)

AHI

-0.013

0.489

0.601 3004 unclassified unclassified

unclassified

unclassified

unclassified

unclassified

Firmicutes

Alistipes sp.

# (HG3A.1385)

AHI -0.013 0.495 0.604 3004 unclassified unclassified Alistipes Rikenellaceae Bacteroidales Bacteroidia Bacteroidetes

| Eubacteriales sp.  (HG3A.0192) | T90 | -0.011 | 0.518 | 0.604 | 3364 unclassified | unclassified | unclassified | unclassified | Eubacteriales | Clostridia | Firmicutes |
| --- | --- | --- | --- | --- | --- | --- | --- | --- | --- | --- | --- |
| Lachnospiraceae sp.  (HG3A.0252) | AHI | -0.012 | 0.499 | 0.607 | 3004 unclassified | unclassified | unclassified | Lachnospiraceae | Eubacteriales | Clostridia | Firmicutes |
| Clostridia sp. (HG3A.1148) | T90 | -0.011 | 0.524 | 0.609 | 3364 unclassified | unclassified | unclassified | unclassified | unclassified | Clostridia | Firmicutes |
| Desulfovibrionales sp.  (HG3A.0266) | T90 | 0.011 | 0.524 | 0.609 | 3364 unclassified | unclassified | unclassified | unclassified | Desulfovibrionales | Deltaproteobacte  ria | Proteobacteria |
| Clostridia sp. (HG3A.0841) | T90 | -0.011 | 0.53 | 0.614 | 3364 unclassified | unclassified | unclassified | unclassified | unclassified | Clostridia | Firmicutes |
| Rikenellaceae sp.  (HG3A.1022) | T90 | 0.011 | 0.53 | 0.614 | 3364 unclassified | unclassified | unclassified | Rikenellaceae | Bacteroidales | Bacteroidia | Bacteroidetes |
| Enterocloster sp. (HG3A.1529) | AHI | 0.012 | 0.508 | 0.617 | 3004 unclassified | unclassified | Enterocloster | Lachnospiraceae | Eubacteriales | Clostridia | Firmicutes |
| Eubacteriales sp.  (HG3A.0663) | AHI | -0.012 | 0.507 | 0.617 | 3004 unclassified | unclassified | unclassified | unclassified | Eubacteriales | Clostridia | Firmicutes |
| Eubacteriales sp. (HG3A.0628) | AHI | -0.012 | 0.509 | 0.618 | 3004 unclassified | unclassified | unclassified | unclassified | Eubacteriales | Clostridia | Firmicutes |
| Erysipelotrichaceae sp.  (HG3A.0867) | AHI | 0.012 | 0.512 | 0.619 | 3004 unclassified | unclassified | unclassified | Erysipelotrichaceae | Erysipelotrichales | Erysipelotrichia | Firmicutes |
| Streptococcus sobrinus (HG3A.1366) | AHI | 0.012 | 0.512 | 0.619 | 3004 unclassified | Streptococcus sobrinus | Streptococcus | Streptococcaceae | Lactobacillales | Bacilli | Firmicutes |
| Subdoligranulum sp. APC924/74  (HG3A.0015) | T90 | -0.011 | 0.536 | 0.62 | 3364 unclassified | Subdoligranulum sp. APC924/74 | Subdoligranulum | Oscillospiraceae | Eubacteriales | Clostridia | Firmicutes |
| Eubacteriales sp. (HG3A.0502) | ODI | 0.01 | 0.565 | 0.623 | 3364 unclassified | unclassified | unclassified | unclassified | Eubacteriales | Clostridia | Firmicutes |
| Firmicutes sp.  (HG3A.1290) | AHI | 0.012 | 0.516 | 0.623 | 3004 unclassified | unclassified | unclassified | unclassified | unclassified | unclassified | Firmicutes |
| Coprococcus catus (HG3A.0037) | AHI | 0.012 | 0.518 | 0.624 | 3004 unclassified | Coprococcus catus | Coprococcus | Lachnospiraceae | Eubacteriales | Clostridia | Firmicutes |
| Eubacteriales sp.  (HG3A.0116) | T90 | 0.011 | 0.54 | 0.624 | 3364 unclassified | unclassified | unclassified | unclassified | Eubacteriales | Clostridia | Firmicutes |
| Eubacteriales sp. (HG3A.0868) | AHI | -0.012 | 0.519 | 0.624 | 3004 unclassified | unclassified | unclassified | unclassified | Eubacteriales | Clostridia | Firmicutes |
| Eubacteriales sp.  (HG3A.1269) | AHI | -0.012 | 0.519 | 0.624 | 3004 unclassified | unclassified | unclassified | unclassified | Eubacteriales | Clostridia | Firmicutes |
| Roseburia sp. AM59- 24XD (HG3A.0391) | T90 | -0.011 | 0.541 | 0.624 | 3364 unclassified | Roseburia sp. AM59- 24XD | Roseburia | Lachnospiraceae | Eubacteriales | Clostridia | Firmicutes |
| Eubacteriales sp.  (HG3A.1006) | ODI | -0.01 | 0.567 | 0.625 | 3364 unclassified | unclassified | unclassified | unclassified | Eubacteriales | Clostridia | Firmicutes |
| Eubacteriales sp. (HG3A.0577) | T90 | -0.011 | 0.544 | 0.627 | 3364 unclassified | unclassified | unclassified | unclassified | Eubacteriales | Clostridia | Firmicutes |

# (HG3A.0345) (HG3A.0386) (HG3A.1087)

Intestinimonas butyriciproducens

(HG3A.0187)

ODI

-0.01

0.573

0.629 3364 unclassified

Intestinimonas

butyriciproducens

Intestinimonas unclassified

Eubacteriales

Clostridia

Firmicutes

| Eubacteriales sp. T90 | -0.01 | 0.547 | 0.629 | 3364 unclassified | unclassified | unclassified | unclassified | Eubacteriales | Clostridia | Firmicutes |
| --- | --- | --- | --- | --- | --- | --- | --- | --- | --- | --- |
| Eubacteriales sp. AHI | -0.012 | 0.524 | 0.629 | 3004 unclassified | unclassified | unclassified | unclassified | Eubacteriales | Clostridia | Firmicutes |
| Eubacteriales sp. T90 | -0.01 | 0.548 | 0.629 | 3364 unclassified | unclassified | unclassified | unclassified | Eubacteriales | Clostridia | Firmicutes |

Acidaminococcus

intestini (HG3A.0407) T90 0.01 0.55 0.631 3364 unclassified Acidaminococcus intestini Acidaminococcus Acidaminococcaceae Acidaminococcales Negativicutes Firmicutes

Butyrivibrio crossotus

(HG3A.0413)

AHI

-0.012

0.527

0.631 3004 unclassified Butyrivibrio crossotus Butyrivibrio

Lachnospiraceae

Eubacteriales

Clostridia

Firmicutes

Firmicutes sp.

# (HG3A.0860)

ODI -0.01 0.575 0.631 3364 unclassified unclassified unclassified unclassified unclassified unclassified Firmicutes

Clostridia sp.

(HG3A.0828)

AHI

-0.012

0.531

0.635 3004 unclassified unclassified

unclassified

unclassified

unclassified

Clostridia

Firmicutes

Eubacteriales sp.

# (HG3A.0547)

ODI -0.01 0.579 0.635 3364 unclassified unclassified unclassified unclassified Eubacteriales Clostridia Firmicutes

Eubacteriales sp.

(HG3A.0367)

ODI

-0.01

0.581

0.636 3364 unclassified unclassified

unclassified

unclassified

Eubacteriales

Clostridia

Firmicutes

Eubacteriales sp.

# (HG3A.0868)

ODI -0.01 0.582 0.636 3364 unclassified unclassified unclassified unclassified Eubacteriales Clostridia Firmicutes

Eubacteriales sp.

(HG3A.0762)

T90

-0.01

0.559

0.64 3364 unclassified unclassified

unclassified

unclassified

Eubacteriales

Clostridia

Firmicutes

Pseudoruminococcus

massiliensis (HG3A.0346)

Bacteria sp.

(HG3A.1545)

AHI

-0.011

0.538

0.642 3004 unclassified unclassified

unclassified

unclassified

unclassified

unclassified

unclassified

AHI -0.011 0.536 0.641 3004 unclassified

Pseudoruminococcus massiliensis

Pseudoruminoco

ccus Oscillospiraceae Eubacteriales Clostridia Firmicutes

Firmicutes sp. (HG3A.1048)

Eubacteriales sp.

(HG3A.0186)

T90

0.01

0.563

0.644 3364 unclassified unclassified

unclassified

unclassified

Eubacteriales

Clostridia

Firmicutes

ODI -0.009 0.588 0.642 3364 unclassified unclassified unclassified unclassified unclassified unclassified Firmicutes

Clostridiaceae sp. (HG3A.0491)

Eubacteriales sp.

(HG3A.1102)

T90

-0.01

0.566

0.645 3364 unclassified unclassified

unclassified

unclassified

Eubacteriales

Clostridia

Firmicutes

AHI 0.011 0.541 0.645 3004 unclassified unclassified unclassified Clostridiaceae Eubacteriales Clostridia Firmicutes

Streptococcus parasanguinis (HG3A.0117)

T90 0.01 0.565 0.645 3364 unclassified

Streptococcus

parasanguinis Streptococcus Streptococcaceae Lactobacillales Bacilli Firmicutes

Prevotella sp.

(HG3A.1040)

T90

-0.01

0.568

0.646 3364 unclassified unclassified

Prevotella

Prevotellaceae

Bacteroidales

Bacteroidia

Bacteroidetes

Bacteroides

cellulosilyticus (HG3A.0108)

Proteobacteria sp.

(HG3A.0327)

T90

0.01

0.578

0.656 3364 unclassified unclassified

unclassified

unclassified

unclassified

unclassified

Proteobacteria

T90 0.01 0.57 0.648 3364 unclassified

Bacteroides

cellulosilyticus Bacteroides Bacteroidaceae Bacteroidales Bacteroidia Bacteroidetes

Streptococcus gallolyticus subsp. gallolyticus (HG3A.1651)

Levilactobacillus brevis AHI

(HG3A.1848)

0.011

0.552

0.657 3004 unclassified Levilactobacillus brevis Levilactobacillus Lactobacillaceae

Lactobacillales

Bacilli

Firmicutes

Streptococcus

T90 -0.01 0.578 0.656 3364 gallolyticus subsp.

gallolyticus

Streptococcus gallolyticus Streptococcus Streptococcaceae Lactobacillales Bacilli Firmicutes

Anaerostipes hadrus (HG3A.0003)

Bacteria sp.

(HG3A.0361)

ODI

-0.009

0.604

0.658 3364 unclassified unclassified

unclassified

unclassified

unclassified

unclassified

unclassified

AHI 0.011 0.555 0.658 3004 unclassified Anaerostipes hadrus Anaerostipes Lachnospiraceae Eubacteriales Clostridia Firmicutes

Oscillospiraceae sp. (HG3A.0806)

Streptococcus mutans

(HG3A.0677)

ODI

0.009

0.605

0.659 3364 unclassified Streptococcus mutans Streptococcus Streptococcaceae Lactobacillales

Bacilli

Firmicutes

AHI -0.011 0.555 0.658 3004 unclassified unclassified unclassified Oscillospiraceae Eubacteriales Clostridia Firmicutes

Eubacteriales sp. (HG3A.0627)

Lachnospiraceae sp.

(HG3A.1525)

AHI

-0.011

0.561

0.664 3004 unclassified unclassified

unclassified

Lachnospiraceae

Eubacteriales

Clostridia

Firmicutes

T90 -0.009 0.587 0.664 3364 unclassified unclassified unclassified unclassified Eubacteriales Clostridia Firmicutes

Sutterella sp. KLE1602

(HG3A.0228) T90 0.009 0.586 0.664 3364 unclassified Sutterella sp. KLE1602 Sutterella Sutterellaceae Burkholderiales

Betaproteobacteri

a Proteobacteria

Bacteria sp.

(HG3A.0839)

T90

-0.009

0.591

0.665 3364 unclassified unclassified

unclassified

unclassified

unclassified

unclassified

unclassified

Eubacteriales sp.

# (HG3A.0639)

AHI -0.011 0.563 0.665 3004 unclassified unclassified unclassified unclassified Eubacteriales Clostridia Firmicutes

Eubacteriales sp.

(HG3A.1154)

T90

0.009

0.589

0.665 3364 unclassified unclassified

unclassified

unclassified

Eubacteriales

Clostridia

Firmicutes

Lachnotalea sp. AF33-28

(HG3A.0403) T90 0.009 0.59 0.665 3364 unclassified Lachnotalea sp. AF33-28 Lachnotalea Lachnospiraceae Eubacteriales Clostridia Firmicutes

Erysipelatoclostridium

sp. (HG3A.0313)

T90

-0.009

0.593

0.667 3364 unclassified unclassified

Erysipelatoclostri Erysipelotrichaceae Erysipelotrichales Erysipelotrichia Firmicutes

dium

Eubacteriales sp.

# (HG3A.1226)

Eubacteriales sp. (HG3A.1131)

T90 -0.009 0.594 0.667 3364 unclassified unclassified unclassified unclassified Eubacteriales Clostridia Firmicutes

AHI -0.011 0.568 0.67 3004 unclassified unclassified unclassified unclassified Eubacteriales Clostridia Firmicutes

| Bifidobacterium |  |  |  |  | Bifidobacteriu |  | | | | | |
| --- | --- | --- | --- | --- | --- | --- | --- | --- | --- | --- | --- |
| animalis subsp. lactis | ODI | -0.009 | 0.617 | 0.671 | 3364 m animalis | Bifidobacterium animalis | Bifidobacterium | Bifidobacteriaceae | Bifidobacteriales | Actinomycetia | Actinobacteria |
| (HG3A.0513) |  |  |  |  | subsp. lactis |  |  |  |  |  |  |
| Eubacteriales sp. (HG3A.0163) | AHI | -0.01 | 0.569 | 0.671 | 3004 unclassified | unclassified | unclassified | unclassified | Eubacteriales | Clostridia | Firmicutes |
| Bacteria sp.  (HG3A.0708) | ODI | -0.009 | 0.619 | 0.672 | 3364 unclassified | unclassified | unclassified | unclassified | unclassified | unclassified | unclassified |
| Eubacteriales sp. (HG3A.1256) | AHI | -0.01 | 0.572 | 0.673 | 3004 unclassified | unclassified | unclassified | unclassified | Eubacteriales | Clostridia | Firmicutes |
| Bacteroidales sp.  (HG3A.0789) | ODI | -0.009 | 0.622 | 0.674 | 3364 unclassified | unclassified | unclassified | unclassified | Bacteroidales | Bacteroidia | Bacteroidetes |
| Firmicutes sp. (HG3A.1091) | AHI | 0.01 | 0.574 | 0.674 | 3004 unclassified | unclassified | unclassified | unclassified | unclassified | unclassified | Firmicutes |
| Lachnospiraceae sp.  (HG3A.1190) | T90 | -0.009 | 0.602 | 0.674 | 3364 unclassified | unclassified | unclassified | Lachnospiraceae | Eubacteriales | Clostridia | Firmicutes |
| Oscillospiraceae sp. (HG3A.0412) | AHI | -0.01 | 0.575 | 0.674 | 3004 unclassified | unclassified | unclassified | Oscillospiraceae | Eubacteriales | Clostridia | Firmicutes |
| Veillonella tobetsuensis (HG3A.1344) | AHI | -0.01 | 0.575 | 0.674 | 3004 unclassified | Veillonella tobetsuensis | Veillonella | Veillonellaceae | Veillonellales | Negativicutes | Firmicutes |
| Dialister pneumosintes (HG3A.1496) | T90 | 0.009 | 0.604 | 0.676 | 3364 unclassified | Dialister pneumosintes | Dialister | Veillonellaceae | Veillonellales | Negativicutes | Firmicutes |
| Eubacteriales sp.  (HG3A.0408) | ODI | 0.009 | 0.625 | 0.677 | 3364 unclassified | unclassified | unclassified | unclassified | Eubacteriales | Clostridia | Firmicutes |
| Eubacteriales sp. (HG3A.1051) | ODI | -0.008 | 0.629 | 0.68 | 3364 unclassified | unclassified | unclassified | unclassified | Eubacteriales | Clostridia | Firmicutes |
| Eubacteriales sp.  (HG3A.1109) | AHI | 0.01 | 0.584 | 0.683 | 3004 unclassified | unclassified | unclassified | unclassified | Eubacteriales | Clostridia | Firmicutes |
| Eubacteriales sp. (HG3A.1219) | AHI | 0.01 | 0.587 | 0.685 | 3004 unclassified | unclassified | unclassified | unclassified | Eubacteriales | Clostridia | Firmicutes |
| Clostridia sp.  (HG3A.0929) | AHI | -0.01 | 0.588 | 0.686 | 3004 unclassified | unclassified | unclassified | unclassified | unclassified | Clostridia | Firmicutes |
| Eubacteriales sp. (HG3A.0967) | AHI | -0.01 | 0.593 | 0.689 | 3004 unclassified | unclassified | unclassified | unclassified | Eubacteriales | Clostridia | Firmicutes |
| Eubacteriales sp.  (HG3A.0978) | AHI | -0.01 | 0.592 | 0.689 | 3004 unclassified | unclassified | unclassified | unclassified | Eubacteriales | Clostridia | Firmicutes |
| Bacteria sp. (HG3A.1553) | ODI | -0.008 | 0.639 | 0.69 | 3364 unclassified | unclassified | unclassified | unclassified | unclassified | unclassified | unclassified |
| Eubacteriales sp.  (HG3A.1393) | AHI | -0.01 | 0.595 | 0.69 | 3004 unclassified | unclassified | unclassified | unclassified | Eubacteriales | Clostridia | Firmicutes |
| Eubacteriales sp. (HG3A.0536) | T90 | -0.009 | 0.62 | 0.693 | 3364 unclassified | unclassified | unclassified | unclassified | Eubacteriales | Clostridia | Firmicutes |

Clostridia sp. (HG3A.1192)

Eubacteriales sp.

(HG3A.1006)

T90

-0.008

0.626

0.699 3364 unclassified unclassified

unclassified

unclassified

Eubacteriales

Clostridia

Firmicutes

AHI -0.01 0.6 0.695 3004 unclassified unclassified unclassified unclassified unclassified Clostridia Firmicutes

Eubacteriales sp. (HG3A.0148)

Eubacteriales sp.

(HG3A.0322)

AHI

-0.01

0.606

0.701 3004 unclassified unclassified

unclassified

unclassified

Eubacteriales

Clostridia

Firmicutes

T90 -0.008 0.629 0.701 3364 unclassified unclassified unclassified unclassified Eubacteriales Clostridia Firmicutes

Eubacteriales sp. (HG3A.0457)

Eubacteriales sp.

(HG3A.1045)

AHI

-0.009

0.607

0.701 3004 unclassified unclassified

unclassified

unclassified

Eubacteriales

Clostridia

Firmicutes

T90 -0.008 0.63 0.701 3364 unclassified unclassified unclassified unclassified Eubacteriales Clostridia Firmicutes

Eubacteriales sp. (HG3A.0973)

Solobacterium moorei

(HG3A.1589)

AHI

0.009

0.612

0.706 3004 unclassified Solobacterium moorei Solobacterium Erysipelotrichaceae Erysipelotrichales Erysipelotrichia Firmicutes

T90 -0.008 0.632 0.702 3364 unclassified unclassified unclassified unclassified Eubacteriales Clostridia Firmicutes

Eubacteriales sp. (HG3A.0873)

Clostridia sp.

(HG3A.1254)

T90

-0.008

0.64

0.709 3364 unclassified unclassified

unclassified

unclassified

unclassified

Clostridia

Firmicutes

AHI -0.009 0.614 0.707 3004 unclassified unclassified unclassified unclassified Eubacteriales Clostridia Firmicutes

Eubacteriales sp. (HG3A.1227)

Ligilactobacillus

salivarius (HG3A.0919) AHI

0.009

0.617

0.709 3004 unclassified Ligilactobacillus salivarius Ligilactobacillus Lactobacillaceae

Lactobacillales

Bacilli

Firmicutes

T90 -0.008 0.641 0.709 3364 unclassified unclassified unclassified unclassified Eubacteriales Clostridia Firmicutes

Ligilactobacillus

salivarius (HG3A.0919) T90 0.008 0.64 0.709 3364 unclassified Ligilactobacillus salivarius Ligilactobacillus Lactobacillaceae Lactobacillales Bacilli Firmicutes

Erysipelotrichales sp.

(HG3A.0283)

ODI

-0.008

0.658

0.71 3364 unclassified unclassified

unclassified

unclassified

Erysipelotrichales Erysipelotrichia Firmicutes

Eubacteriales sp.

# (HG3A.0333)

AHI -0.009 0.619 0.711 3004 unclassified unclassified unclassified unclassified Eubacteriales Clostridia Firmicutes

Eubacteriales sp.

(HG3A.0594)

AHI

-0.009

0.62

0.711 3004 unclassified unclassified

unclassified

unclassified

Eubacteriales

Clostridia

Firmicutes

Eubacteriales sp.

# (HG3A.1102)

AHI -0.009 0.621 0.711 3004 unclassified unclassified unclassified unclassified Eubacteriales Clostridia Firmicutes

Eubacteriales sp.

(HG3A.0633)

AHI

-0.009

0.623

0.712 3004 unclassified unclassified

unclassified

unclassified

Eubacteriales

Clostridia

Firmicutes

Eggerthellales sp.

# (HG3A.0174)

T90 0.008 0.647 0.715 3364 unclassified unclassified unclassified unclassified Eggerthellales Coriobacteriia Actinobacteria

Oscillospiraceae sp.

(HG3A.0765)

T90

-0.008

0.648

0.715 3364 unclassified unclassified

unclassified

Oscillospiraceae

Eubacteriales

Clostridia

Firmicutes

Eubacteriales sp.

# (HG3A.0924)

AHI -0.009 0.631 0.72 3004 unclassified unclassified unclassified unclassified Eubacteriales Clostridia Firmicutes

| Eisenbergiella tayi  (HG3A.0355) | AHI | 0.009 | 0.635 | 0.725 | 3004 unclassified | Eisenbergiella tayi | Eisenbergiella | Lachnospiraceae | Eubacteriales | Clostridia | Firmicutes |
| --- | --- | --- | --- | --- | --- | --- | --- | --- | --- | --- | --- |
| Desulfovibrio fairfieldensis  (HG3A.0529) | T90 | 0.008 | 0.66 | 0.727 | 3364 unclassified | Desulfovibrio fairfieldensis | Desulfovibrio | Desulfovibrionaceae | Desulfovibrionales | Deltaproteobacte ria | Proteobacteria |
| Clostridia sp. (HG3A.1625) | ODI | -0.007 | 0.676 | 0.728 | 3364 unclassified | unclassified | unclassified | unclassified | unclassified | Clostridia | Firmicutes |
| Oscillospiraceae sp.  (HG3A.0944) | AHI | 0.009 | 0.639 | 0.728 | 3004 unclassified | unclassified | unclassified | Oscillospiraceae | Eubacteriales | Clostridia | Firmicutes |
| Lachnospiraceae sp. (HG3A.0625) | ODI | 0.007 | 0.678 | 0.729 | 3364 unclassified | unclassified | unclassified | Lachnospiraceae | Eubacteriales | Clostridia | Firmicutes |
| Firmicutes sp.  (HG3A.1050) | ODI | -0.007 | 0.68 | 0.73 | 3364 unclassified | unclassified | unclassified | unclassified | unclassified | unclassified | Firmicutes |
| Oscillospiraceae sp. (HG3A.0805) | T90 | -0.008 | 0.663 | 0.73 | 3364 unclassified | unclassified | unclassified | Oscillospiraceae | Eubacteriales | Clostridia | Firmicutes |
| Eubacteriales sp.  (HG3A.0851) | ODI | -0.007 | 0.684 | 0.733 | 3364 unclassified | unclassified | unclassified | unclassified | Eubacteriales | Clostridia | Firmicutes |
| Parabacteroides  gordonii (HG3A.0989) | ODI | 0.007 | 0.685 | 0.733 | 3364 unclassified | Parabacteroides gordonii | Parabacteroides | Tannerellaceae | Bacteroidales | Bacteroidia | Bacteroidetes |
| Eubacteriales sp.  (HG3A.0138) | T90 | -0.007 | 0.669 | 0.735 | 3364 unclassified | unclassified | unclassified | unclassified | Eubacteriales | Clostridia | Firmicutes |
| Bacteria sp. (HG3A.1349) | T90 | -0.007 | 0.672 | 0.738 | 3364 unclassified | unclassified | unclassified | unclassified | unclassified | unclassified | unclassified |
| Clostridia sp.  (HG3A.0852) | T90 | 0.007 | 0.673 | 0.738 | 3364 unclassified | unclassified | unclassified | unclassified | unclassified | Clostridia | Firmicutes |
| Bacteroidales sp. (HG3A.1446) | AHI | -0.008 | 0.656 | 0.744 | 3004 unclassified | unclassified | unclassified | unclassified | Bacteroidales | Bacteroidia | Bacteroidetes |
| Coprobacillus cateniformis  (HG3A.0456) | AHI | 0.008 | 0.654 | 0.744 | 3004 unclassified | Coprobacillus cateniformis | Coprobacillus | Coprobacillaceae | Erysipelotrichales | Erysipelotrichia | Firmicutes |
| Eubacteriales sp. (HG3A.1285) | AHI | -0.008 | 0.655 | 0.744 | 3004 unclassified | unclassified | unclassified | unclassified | Eubacteriales | Clostridia | Firmicutes |
| Clostridia sp.  (HG3A.0909) | T90 | -0.007 | 0.683 | 0.745 | 3364 unclassified | unclassified | unclassified | unclassified | unclassified | Clostridia | Firmicutes |
| Erysipelotrichales sp. (HG3A.0303) | T90 | -0.007 | 0.681 | 0.745 | 3364 unclassified | unclassified | unclassified | unclassified | Erysipelotrichales | Erysipelotrichia | Firmicutes |
| Eubacteriales sp.  (HG3A.0450) | T90 | -0.007 | 0.681 | 0.745 | 3364 unclassified | unclassified | unclassified | unclassified | Eubacteriales | Clostridia | Firmicutes |
| Firmicutes sp. (HG3A.0464) | ODI | 0.007 | 0.697 | 0.745 | 3364 unclassified | unclassified | unclassified | unclassified | unclassified | unclassified | Firmicutes |

Streptococcus salivarius

(HG3A.0071) T90 0.007 0.684 0.745 3364 unclassified Streptococcus salivarius Streptococcus Streptococcaceae Lactobacillales Bacilli Firmicutes

| Bacteroides Bacteroides  cellulosilyticus AHI -0.008 0.66 0.747 3004 unclassified cellulosilyticus Bacteroides Bacteroidaceae Bacteroidales Bacteroidia Bacteroidetes  (HG3A.0108) | | | | | | | | | | | |
| --- | --- | --- | --- | --- | --- | --- | --- | --- | --- | --- | --- |
| Clostridia sp.  (HG3A.0918) | AHI | -0.008 | 0.661 | 0.747 | 3004 unclassified | unclassified | unclassified | unclassified | unclassified | Clostridia | Firmicutes |
| Eubacteriales sp. (HG3A.0924) | ODI | -0.007 | 0.701 | 0.747 | 3364 unclassified | unclassified | unclassified | unclassified | Eubacteriales | Clostridia | Firmicutes |
| Firmicutes sp.  (HG3A.1014) | ODI | -0.007 | 0.701 | 0.747 | 3364 unclassified | unclassified | unclassified | unclassified | unclassified | unclassified | Firmicutes |
| Oscillospiraceae sp. (HG3A.0576) | AHI | -0.008 | 0.661 | 0.747 | 3004 unclassified | unclassified | unclassified | Oscillospiraceae | Eubacteriales | Clostridia | Firmicutes |
| Oscillospiraceae sp.  (HG3A.0806) | ODI | -0.007 | 0.701 | 0.747 | 3364 unclassified | unclassified | unclassified | Oscillospiraceae | Eubacteriales | Clostridia | Firmicutes |
| Eubacteriales sp. (HG3A.1063) | T90 | -0.007 | 0.688 | 0.749 | 3364 unclassified | unclassified | unclassified | unclassified | Eubacteriales | Clostridia | Firmicutes |
| Clostridia sp.  (HG3A.1493) | AHI | -0.008 | 0.665 | 0.75 | 3004 unclassified | unclassified | unclassified | unclassified | unclassified | Clostridia | Firmicutes |
| Eubacteriales sp. (HG3A.1063) | AHI | -0.008 | 0.672 | 0.757 | 3004 unclassified | unclassified | unclassified | unclassified | Eubacteriales | Clostridia | Firmicutes |
| Clostridiaceae sp.  (HG3A.0608) | T90 | -0.007 | 0.703 | 0.759 | 3364 unclassified | unclassified | unclassified | Clostridiaceae | Eubacteriales | Clostridia | Firmicutes |
| Dorea phocaeensis (HG3A.0865) | T90 | 0.007 | 0.699 | 0.759 | 3364 unclassified | Dorea phocaeensis | Dorea | Lachnospiraceae | Eubacteriales | Clostridia | Firmicutes |
| Eubacteriales sp.  (HG3A.0178) | T90 | -0.007 | 0.703 | 0.759 | 3364 unclassified | unclassified | unclassified | unclassified | Eubacteriales | Clostridia | Firmicutes |
| Eubacteriales sp. (HG3A.0308) | T90 | 0.007 | 0.702 | 0.759 | 3364 unclassified | unclassified | unclassified | unclassified | Eubacteriales | Clostridia | Firmicutes |
| Eubacteriales sp.  (HG3A.1131) | ODI | -0.006 | 0.714 | 0.759 | 3364 unclassified | unclassified | unclassified | unclassified | Eubacteriales | Clostridia | Firmicutes |
| Lachnospiraceae sp. (HG3A.0831) | T90 | -0.007 | 0.704 | 0.759 | 3364 unclassified | unclassified | unclassified | Lachnospiraceae | Eubacteriales | Clostridia | Firmicutes |
| Parabacteroides goldsteinii (HG3A.0279) | T90 | -0.007 | 0.701 | 0.759 | 3364 unclassified | Parabacteroides goldsteinii | Parabacteroides | Tannerellaceae | Bacteroidales | Bacteroidia | Bacteroidetes |
| Lachnospiraceae sp. (HG3A.0625) | AHI | 0.008 | 0.677 | 0.76 | 3004 unclassified | unclassified | unclassified | Lachnospiraceae | Eubacteriales | Clostridia | Firmicutes |
| Clostridia sp.  (HG3A.0841) | AHI | -0.008 | 0.68 | 0.761 | 3004 unclassified | unclassified | unclassified | unclassified | unclassified | Clostridia | Firmicutes |
| Clostridia sp. (HG3A.0861) | AHI | 0.008 | 0.678 | 0.761 | 3004 unclassified | unclassified | unclassified | unclassified | unclassified | Clostridia | Firmicutes |

Clostridia sp. (HG3A.1035)

AHI -0.008 0.68 0.761 3004 unclassified unclassified unclassified unclassified unclassified Clostridia Firmicutes

| Traorella massiliensis (HG3A.0669) | AHI | 0.008 | 0.681 | 0.761 | 3004 unclassified | Traorella massiliensis | Traorella | Erysipelotrichaceae | Erysipelotrichales | Erysipelotrichia | Firmicutes |
| --- | --- | --- | --- | --- | --- | --- | --- | --- | --- | --- | --- |
| Bacteria sp.  (HG3A.1543) | T90 | 0.007 | 0.708 | 0.762 | 3364 unclassified | unclassified | unclassified | unclassified | unclassified | unclassified | unclassified |
| Eubacteriales sp. (HG3A.0428) | T90 | -0.006 | 0.71 | 0.762 | 3364 unclassified | unclassified | unclassified | unclassified | Eubacteriales | Clostridia | Firmicutes |
| Eubacteriales sp.  (HG3A.1321) | T90 | -0.006 | 0.711 | 0.762 | 3364 unclassified | unclassified | unclassified | unclassified | Eubacteriales | Clostridia | Firmicutes |
| Firmicutes sp. (HG3A.1050) | T90 | 0.006 | 0.71 | 0.762 | 3364 unclassified | unclassified | unclassified | unclassified | unclassified | unclassified | Firmicutes |
| Pseudoflavonifractor sp. An184 (HG3A.0253) | T90 | -0.006 | 0.711 | 0.762 | 3364 unclassified | Pseudoflavonifractor sp. An184 | Pseudoflavonifrac tor | Oscillospiraceae | Eubacteriales | Clostridia | Firmicutes |
| Oscillospiraceae sp. (HG3A.0134) | T90 | 0.006 | 0.713 | 0.763 | 3364 unclassified | unclassified | unclassified | Oscillospiraceae | Eubacteriales | Clostridia | Firmicutes |
| Eubacteriales sp.  (HG3A.0887) | T90 | 0.006 | 0.718 | 0.768 | 3364 unclassified | unclassified | unclassified | unclassified | Eubacteriales | Clostridia | Firmicutes |
| Alistipes sp. (HG3A.1385) | T90 | -0.006 | 0.721 | 0.769 | 3364 unclassified | unclassified | Alistipes | Rikenellaceae | Bacteroidales | Bacteroidia | Bacteroidetes |
| Clostridiaceae sp.  (HG3A.0330) | AHI | -0.007 | 0.693 | 0.772 | 3004 unclassified | unclassified | unclassified | Clostridiaceae | Eubacteriales | Clostridia | Firmicutes |
| Eubacteriales sp. (HG3A.1257) | AHI | -0.007 | 0.694 | 0.772 | 3004 unclassified | unclassified | unclassified | unclassified | Eubacteriales | Clostridia | Firmicutes |
| Oscillospiraceae sp.  (HG3A.0739) | AHI | -0.007 | 0.694 | 0.772 | 3004 unclassified | unclassified | unclassified | Oscillospiraceae | Eubacteriales | Clostridia | Firmicutes |
| Clostridia sp. (HG3A.1452) | T90 | 0.006 | 0.725 | 0.773 | 3364 unclassified | unclassified | unclassified | unclassified | unclassified | Clostridia | Firmicutes |
| Clostridia sp.  (HG3A.0909) | ODI | -0.006 | 0.729 | 0.774 | 3364 unclassified | unclassified | unclassified | unclassified | unclassified | Clostridia | Firmicutes |
| Eubacteriales sp. (HG3A.0580) | AHI | -0.007 | 0.696 | 0.774 | 3004 unclassified | unclassified | unclassified | unclassified | Eubacteriales | Clostridia | Firmicutes |
| Eubacteriales sp.  (HG3A.1134) | AHI | -0.007 | 0.698 | 0.774 | 3004 unclassified | unclassified | unclassified | unclassified | Eubacteriales | Clostridia | Firmicutes |
| Clostridia sp. (HG3A.1247) | T90 | 0.006 | 0.73 | 0.775 | 3364 unclassified | unclassified | unclassified | unclassified | unclassified | Clostridia | Firmicutes |
| Eubacteriales sp.  (HG3A.0832) | T90 | -0.006 | 0.73 | 0.775 | 3364 unclassified | unclassified | unclassified | unclassified | Eubacteriales | Clostridia | Firmicutes |
| Streptococcus gordonii (HG3A.0713) | T90 | 0.006 | 0.729 | 0.775 | 3364 unclassified | Streptococcus gordonii | Streptococcus | Streptococcaceae | Lactobacillales | Bacilli | Firmicutes |

[Clostridium] spiroforme

(HG3A.0259) T90 -0.006 0.731 0.776 3364 unclassified [Clostridium] spiroforme

Erysipelatoclostri

dium Erysipelotrichaceae Erysipelotrichales Erysipelotrichia Firmicutes

tor

| Oscillospiraceae sp.  (HG3A.1588) | ODI | -0.006 | 0.732 | 0.777 | 3364 unclassified | unclassified | unclassified Oscillospiraceae | Eubacteriales | Clostridia | Firmicutes |
| --- | --- | --- | --- | --- | --- | --- | --- | --- | --- | --- |
| Eubacteriales sp.  (HG3A.0323) | AHI | -0.007 | 0.704 | 0.778 | 3004 unclassified | unclassified | unclassified unclassified | Eubacteriales | Clostridia | Firmicutes |
| Eubacteriales sp. (HG3A.0972) | AHI | -0.007 | 0.705 | 0.778 | 3004 unclassified | unclassified | unclassified unclassified | Eubacteriales | Clostridia | Firmicutes |
| Eubacteriales sp.  (HG3A.1473) | AHI | 0.007 | 0.703 | 0.778 | 3004 unclassified | unclassified | unclassified unclassified | Eubacteriales | Clostridia | Firmicutes |
| Firmicutes sp. (HG3A.0570) | AHI | -0.007 | 0.705 | 0.778 | 3004 unclassified | unclassified | unclassified unclassified | unclassified | unclassified | Firmicutes |
| Erysipelotrichales sp.  (HG3A.0809) | T90 | 0.006 | 0.735 | 0.779 | 3364 unclassified | unclassified | unclassified unclassified | Erysipelotrichales | Erysipelotrichia | Firmicutes |
| Desulfovibrionales sp. (HG3A.0727) | T90 | 0.006 | 0.737 | 0.78 | 3364 unclassified | unclassified | unclassified unclassified | Desulfovibrionales | Deltaproteobacte ria | Proteobacteria |
| Pseudoflavonifractor sp.  (HG3A.0844) | ODI | 0.006 | 0.736 | 0.78 | 3364 unclassified | unclassified | Pseudoflavonifrac Oscillospiraceae | Eubacteriales | Clostridia | Firmicutes |
| Eubacteriales sp. (HG3A.1473) | ODI | 0.006 | 0.738 | 0.781 | 3364 unclassified | unclassified | unclassified unclassified | Eubacteriales | Clostridia | Firmicutes |
| Eubacteriales sp.  (HG3A.0916) | ODI | -0.006 | 0.742 | 0.784 | 3364 unclassified | unclassified | unclassified unclassified | Eubacteriales | Clostridia | Firmicutes |
| Eubacteriales sp. (HG3A.0846) | AHI | 0.007 | 0.713 | 0.785 | 3004 unclassified | unclassified | unclassified unclassified | Eubacteriales | Clostridia | Firmicutes |
| Firmicutes sp.  (HG3A.1091) | ODI | 0.006 | 0.744 | 0.785 | 3364 unclassified | unclassified | unclassified unclassified | unclassified | unclassified | Firmicutes |
| Oscillospiraceae sp. (HG3A.0412) | ODI | -0.006 | 0.745 | 0.785 | 3364 unclassified | unclassified | unclassified Oscillospiraceae | Eubacteriales | Clostridia | Firmicutes |
| Eubacteriales sp.  (HG3A.1573) | ODI | -0.006 | 0.747 | 0.786 | 3364 unclassified | unclassified | unclassified unclassified | Eubacteriales | Clostridia | Firmicutes |
| Clostridia sp. (HG3A.1108) | T90 | -0.006 | 0.746 | 0.788 | 3364 unclassified | unclassified | unclassified unclassified | unclassified | Clostridia | Firmicutes |
| Eubacteriales sp.  (HG3A.0717) | AHI | -0.007 | 0.717 | 0.789 | 3004 unclassified | unclassified | unclassified unclassified | Eubacteriales | Clostridia | Firmicutes |
| Eubacteriales sp. (HG3A.0786) | ODI | 0.006 | 0.751 | 0.789 | 3364 unclassified | unclassified | unclassified unclassified | Eubacteriales | Clostridia | Firmicutes |
| Faecalibacterium sp. OF04-11AC  (HG3A.0070) | ODI | -0.005 | 0.753 | 0.791 | 3364 unclassified | Faecalibacterium sp. OF04-11AC | Faecalibacterium Oscillospiraceae | Eubacteriales | Clostridia | Firmicutes |
| Atopobiaceae sp. (HG3A.0937) | ODI | 0.005 | 0.759 | 0.795 | 3364 unclassified | unclassified | unclassified Atopobiaceae | Coriobacteriales | Coriobacteriia | Actinobacteria |
| Clostridia sp.  (HG3A.1625) | AHI | 0.006 | 0.725 | 0.795 | 3004 unclassified | unclassified | unclassified unclassified | unclassified | Clostridia | Firmicutes |
| Eubacteriales sp. (HG3A.1177) | AHI | -0.007 | 0.724 | 0.795 | 3004 unclassified | unclassified | unclassified unclassified | Eubacteriales | Clostridia | Firmicutes |

Alistipes ihumii (HG3A.0106)

| Eubacteriales sp. (HG3A.1126) | ODI | 0.005 | 0.764 | 0.8 | 3364 unclassified | unclassified | unclassified | unclassified | Eubacteriales Clostridia | Firmicutes |
| --- | --- | --- | --- | --- | --- | --- | --- | --- | --- | --- |
| Clostridiaceae sp.  (HG3A.0471) | T90 | 0.005 | 0.761 | 0.801 | 3364 unclassified | unclassified | unclassified | Clostridiaceae | Eubacteriales Clostridia | Firmicutes |
| Eubacteriales sp. (HG3A.0408) | AHI | 0.006 | 0.732 | 0.802 | 3004 unclassified | unclassified | unclassified | unclassified | Eubacteriales Clostridia | Firmicutes |
| Eubacteriales sp.  (HG3A.1003) | T90 | -0.005 | 0.762 | 0.802 | 3364 unclassified | unclassified | unclassified | unclassified | Eubacteriales Clostridia | Firmicutes |
| Proteobacteria sp. (HG3A.0360) | AHI | -0.006 | 0.734 | 0.804 | 3004 unclassified | unclassified | unclassified | unclassified | unclassified unclassified | Proteobacteria |
| Eubacteriales sp.  (HG3A.0858) | ODI | -0.005 | 0.771 | 0.805 | 3364 unclassified | unclassified | unclassified | unclassified | Eubacteriales Clostridia | Firmicutes |
| Firmicutes sp. (HG3A.1048) | AHI | 0.006 | 0.74 | 0.809 | 3004 unclassified | unclassified | unclassified | unclassified | unclassified unclassified | Firmicutes |
| Actinomyces sp. ICM58  (HG3A.0410) | AHI | 0.006 | 0.746 | 0.813 | 3004 unclassified | Actinomyces sp. ICM58 | Actinomyces | Actinomycetaceae | Actinomycetales Actinomycetia | Actinobacteria |
| Eubacteriales sp. (HG3A.0302) | T90 | -0.005 | 0.774 | 0.813 | 3364 unclassified | unclassified | unclassified | unclassified | Eubacteriales Clostridia | Firmicutes |
| Firmicutes sp.  (HG3A.0817) | AHI | 0.006 | 0.746 | 0.813 | 3004 unclassified | unclassified | unclassified | unclassified | unclassified unclassified | Firmicutes |
| Firmicutes sp. (HG3A.0817) | ODI | 0.005 | 0.779 | 0.813 | 3364 unclassified | unclassified | unclassified | unclassified | unclassified unclassified | Firmicutes |
| Clostridia sp.  (HG3A.1076) | T90 | -0.005 | 0.776 | 0.814 | 3364 unclassified | unclassified | unclassified | unclassified | unclassified Clostridia | Firmicutes |
| Eubacteriales sp. (HG3A.0692) | ODI | 0.005 | 0.782 | 0.815 | 3364 unclassified | unclassified | unclassified | unclassified | Eubacteriales Clostridia | Firmicutes |
| Firmicutes sp.  (HG3A.1471) | AHI | -0.006 | 0.75 | 0.816 | 3004 unclassified | unclassified | unclassified | unclassified | unclassified unclassified | Firmicutes |
| Oscillospiraceae sp. (HG3A.0774) | AHI | -0.006 | 0.752 | 0.817 | 3004 unclassified | unclassified | unclassified | Oscillospiraceae | Eubacteriales Clostridia | Firmicutes |
| Akkermansia sp. BIOML A59 (HG3A.0800) | ODI | -0.005 | 0.79 | 0.821 | 3364 unclassified | Akkermansia sp. BIOML- A59 | Akkermansia | Akkermansiaceae | Verrucomicrobia Verrucomicrobiales e | Verrucomicrob ia |
| Collinsella intestinalis (HG3A.0802) | ODI | 0.005 | 0.788 | 0.821 | 3364 unclassified | Collinsella intestinalis | Collinsella | Coriobacteriaceae | Coriobacteriales Coriobacteriia | Actinobacteria |
| Streptococcus agalactiae (HG3A.1733) | T90 | 0.005 | 0.784 | 0.821 | 3364 unclassified | Streptococcus agalactiae | Streptococcus | Streptococcaceae | Lactobacillales Bacilli | Firmicutes |
| Eisenbergiella tayi (HG3A.0355) | ODI | -0.005 | 0.795 | 0.825 | 3364 unclassified | Eisenbergiella tayi | Eisenbergiella | Lachnospiraceae | Eubacteriales Clostridia | Firmicutes |
[truncated: 22,967 more chars]
