# Supplementary material for: OSA Is Associated With the Human Gut Microbiota Composition and Functional Potential in the Population-Based Swedish CardioPulmonary bioImage Study
Source: Chest. 2023 Mar 15;164(2):503–16. doi: 10.1016/j.chest.2023.03.010 (PMC10410248; doi:10.1016/j.chest.2023.03.010)
Supplement: e-Table 7 [file mmc4.docx]

e-Table 7. Partial Spearman’s correlations of AHI, T90, and ODI with microbiota species using the extended main model

Only the species identified in the model not adjusted for body mass index (BMI) were included in this analysis. Associations adjusted for age, sex, smoking, alcohol intake, BMI, fiber intake, total energy intake, physical activity, education, birth country, season, and DNA extraction plate. Adjustment for multiple testing for those species identified in the model not adjust for BMI using the Benjamini-Hochberg method and presented as q-values. Under the column "Metagenomics species", the information between pararenthesis is the internal identifier for the respective species. AHI: apnea-hypopnea index; ODI: oxygen

| **Metagenomic species** | **exposure** | **Spearman's**  **correlation** | **p-value** | **q-value N** | **subspecies** | **species** | **genus** | **family** | **order** | **class** | **phylum** |
| --- | --- | --- | --- | --- | --- | --- | --- | --- | --- | --- | --- |
| Dorea formicigenerans (HG3A.0006) | T90 | 0.086 | 1.34E-06 | 0.001 | 3249 unclassified | Dorea  formicigenerans | Dorea | Lachnospiraceae | Eubacteriales | Clostridia | Firmicutes |
| Blautia obeum (HG3A.0001) | T90 | 0.079 | 7.97E-06 | 0.003 | 3249 unclassified | Blautia obeum | Blautia | Lachnospiraceae | Eubacteriales | Clostridia | Firmicutes |
| Eubacteriales sp. (HG3A.0069) | ODI | -0.074 | 2.85E-05 | 0.006 | 3249 unclassified | unclassified | unclassified | unclassified | Eubacteriales | Clostridia | Firmicutes |
| Eubacteriales sp. (HG3A.0100) | ODI | -0.073 | 3.58E-05 | 0.006 | 3249 unclassified | unclassified | unclassified | unclassified | Eubacteriales | Clostridia | Firmicutes |
| Eubacteriales sp. (HG3A.0149) | ODI | -0.074 | 2.86E-05 | 0.006 | 3249 unclassified | unclassified | unclassified | unclassified | Eubacteriales | Clostridia | Firmicutes |
| Eubacteriales sp. (HG3A.0442) | ODI | -0.075 | 2.17E-05 | 0.006 | 3249 unclassified | unclassified | unclassified | unclassified | Eubacteriales | Clostridia | Firmicutes |
| Oscillospiraceae sp. (HG3A.0207) | ODI | -0.077 | 1.62E-05 | 0.006 | 3249 unclassified | unclassified | unclassified | Oscillospiraceae | Eubacteriales | Clostridia | Firmicutes |
| Clostridia sp. (HG3A.0140) | ODI | -0.071 | 6.29E-05 | 0.007 | 3249 unclassified | unclassified | unclassified | unclassified | unclassified | Clostridia | Firmicutes |
| Clostridia sp. (HG3A.0515) | ODI | -0.071 | 7.06E-05 | 0.007 | 3249 unclassified | unclassified | unclassified | unclassified | unclassified | Clostridia | Firmicutes |
| Eubacteriales sp. (HG3A.0197) | ODI | -0.071 | 6.17E-05 | 0.007 | 3249 unclassified | unclassified | unclassified | unclassified | Eubacteriales | Clostridia | Firmicutes |
| Alistipes shahii (HG3A.0054) | ODI | -0.067 | 1.80E-04 | 0.008 | 3249 unclassified | Alistipes shahii | Alistipes | Rikenellaceae | Bacteroidales | Bacteroidia | Bacteroidetes |
| Bacteria sp. (HG3A.0634) | ODI | -0.067 | 1.66E-04 | 0.008 | 3249 unclassified | unclassified | unclassified | unclassified | unclassified | unclassified | unclassified |
| Eubacteriales sp. (HG3A.0156) | ODI | -0.067 | 1.48E-04 | 0.008 | 3249 unclassified | unclassified | unclassified | unclassified | Eubacteriales | Clostridia | Firmicutes |
| Eubacteriales sp. (HG3A.0269) | ODI | -0.067 | 1.69E-04 | 0.008 | 3249 unclassified | unclassified | unclassified | unclassified | Eubacteriales | Clostridia | Firmicutes |
| Eubacteriales sp. (HG3A.0572) | ODI | -0.067 | 1.70E-04 | 0.008 | 3249 unclassified | unclassified | unclassified | unclassified | Eubacteriales | Clostridia | Firmicutes |
| Firmicutes sp. (HG3A.0397) | ODI | -0.068 | 1.24E-04 | 0.008 | 3249 unclassified | unclassified | unclassified | unclassified | unclassified | unclassified | Firmicutes |
| Fusicatenibacter saccharivorans (HG3A.0004) | ODI | 0.069 | 9.56E-05 | 0.008 | 3249 unclassified | Fusicatenibacter saccharivorans | Fusicatenibacter | Lachnospiraceae | Eubacteriales | Clostridia | Firmicutes |
| Lachnospiraceae sp. (HG3A.0018) | ODI | 0.069 | 1.13E-04 | 0.008 | 3249 unclassified | unclassified | unclassified | Lachnospiraceae | Eubacteriales | Clostridia | Firmicutes |
| Oscillospiraceae sp. (HG3A.0072) | ODI | -0.069 | 1.14E-04 | 0.008 | 3249 unclassified | unclassified | unclassified | Oscillospiraceae | Eubacteriales | Clostridia | Firmicutes |
| Bacteria sp. (HG3A.0483) | ODI | -0.066 | 2.12E-04 | 0.009 | 3249 unclassified | unclassified | unclassified | unclassified | unclassified | unclassified | unclassified |
| Eubacteriales sp. (HG3A.0703) | T90 | -0.073 | 3.52E-05 | 0.009 | 3249 unclassified | unclassified | unclassified | unclassified | Eubacteriales | Clostridia | Firmicutes |

| Blautia obeum (HG3A.0001) | ODI | 0.064 | 2.94E-04 | 0.010 | 3249 unclassified | Blautia obeum | Blautia | Lachnospiraceae | Eubacteriales | Clostridia | Firmicutes |
| --- | --- | --- | --- | --- | --- | --- | --- | --- | --- | --- | --- |
| Clostridia sp. (HG3A.0140) | T90 | -0.071 | 5.84E-05 | 0.010 | 3249 unclassified | unclassified | unclassified | unclassified | unclassified | Clostridia | Firmicutes |
| Clostridia sp. (HG3A.0272) | ODI | -0.063 | 3.91E-04 | 0.010 | 3249 unclassified | unclassified | unclassified | unclassified | unclassified | Clostridia | Firmicutes |
| Clostridia sp. (HG3A.0728) | ODI | -0.063 | 3.53E-04 | 0.010 | 3249 unclassified | unclassified | unclassified | unclassified | unclassified | Clostridia | Firmicutes |
| Clostridium sp. TF06-15AC  (HG3A.0032) | ODI | 0.063 | 3.95E-04 | 0.010 | 3249 unclassified | Clostridium sp.  TF06-15AC | Clostridium | Clostridiaceae | Eubacteriales | Clostridia | Firmicutes |
| Coprococcus comes (HG3A.0016) | ODI | 0.065 | 2.32E-04 | 0.010 | 3249 unclassified | Coprococcus comes | Coprococcus | Lachnospiraceae | Eubacteriales | Clostridia | Firmicutes |
| Eggerthellales sp. (HG3A.0177) | ODI | -0.063 | 3.66E-04 | 0.010 | 3249 unclassified | unclassified | unclassified | unclassified | Eggerthellales | Coriobacteriia | Actinobacteria |
| Eubacteriales sp. (HG3A.0149) | T90 | -0.071 | 6.50E-05 | 0.010 | 3249 unclassified | unclassified | unclassified | unclassified | Eubacteriales | Clostridia | Firmicutes |
| Eubacteriales sp. (HG3A.0211) | ODI | -0.064 | 3.39E-04 | 0.010 | 3249 unclassified | unclassified | unclassified | unclassified | Eubacteriales | Clostridia | Firmicutes |
| Eubacteriales sp. (HG3A.0226) | ODI | -0.064 | 2.83E-04 | 0.010 | 3249 unclassified | unclassified | unclassified | unclassified | Eubacteriales | Clostridia | Firmicutes |
| Eubacteriales sp. (HG3A.0311) | ODI | -0.065 | 2.66E-04 | 0.010 | 3249 unclassified | unclassified | unclassified | unclassified | Eubacteriales | Clostridia | Firmicutes |
| Eubacteriales sp. (HG3A.0331) | ODI | -0.063 | 3.80E-04 | 0.010 | 3249 unclassified | unclassified | unclassified | unclassified | Eubacteriales | Clostridia | Firmicutes |
| Eubacteriales sp. (HG3A.0506) | ODI | -0.065 | 2.68E-04 | 0.010 | 3249 unclassified | unclassified | unclassified | unclassified | Eubacteriales | Clostridia | Firmicutes |
| Eubacteriales sp. (HG3A.0829) | ODI | -0.064 | 3.47E-04 | 0.010 | 3249 unclassified | unclassified | unclassified | unclassified | Eubacteriales | Clostridia | Firmicutes |
| [Ruminococcus] gnavus  (HG3A.0239) | ODI | 0.062 | 4.39E-04 | 0.011 | 3249 unclassified | [Ruminococcus]  gnavus | Mediterraneibacte  r | Lachnospiraceae | Eubacteriales | Clostridia | Firmicutes |
| Firmicutes sp. (HG3A.0398) | ODI | -0.063 | 4.24E-04 | 0.011 | 3249 unclassified | unclassified | unclassified | unclassified | unclassified | unclassified | Firmicutes |
| Eubacteriales sp. (HG3A.0242) | ODI | -0.062 | 4.95E-04 | 0.012 | 3249 unclassified | unclassified | unclassified | unclassified | Eubacteriales | Clostridia | Firmicutes |
| Mediterraneibacter glycyrrhizinilyticus (HG3A.0314) | T90 | 0.069 | 9.30E-05 | 0.012 | 3249 unclassified | Mediterraneibacter glycyrrhizinilyticus | Mediterraneibacte r | Lachnospiraceae | Eubacteriales | Clostridia | Firmicutes |
| Victivallis vadensis (HG3A.0689) | ODI | -0.062 | 5.34E-04 | 0.012 | 3249 unclassified | Victivallis vadensis | Victivallis | Victivallaceae | Victivallales | Lentisphaeria | Lentisphaerae |
| Clostridia sp. (HG3A.0682) | ODI | -0.061 | 6.35E-04 | 0.013 | 3249 unclassified | unclassified | unclassified | unclassified | unclassified | Clostridia | Firmicutes |
| Eubacteriales sp. (HG3A.0118) | ODI | -0.061 | 5.98E-04 | 0.013 | 3249 unclassified | unclassified | unclassified | unclassified | Eubacteriales | Clostridia | Firmicutes |
| Eubacteriales sp. (HG3A.0421) | ODI | -0.061 | 6.36E-04 | 0.013 | 3249 unclassified | unclassified | unclassified | unclassified | Eubacteriales | Clostridia | Firmicutes |
| Eubacteriales sp. (HG3A.0609) | ODI | -0.061 | 6.57E-04 | 0.013 | 3249 unclassified | unclassified | unclassified | unclassified | Eubacteriales | Clostridia | Firmicutes |
| Roseburia inulinivorans (HG3A.0036) | ODI | 0.061 | 6.03E-04 | 0.013 | 3249 unclassified | Roseburia inulinivorans | Roseburia | Lachnospiraceae | Eubacteriales | Clostridia | Firmicutes |
| Eubacteriales sp. (HG3A.0419) | T90 | -0.067 | 1.44E-04 | 0.014 | 3249 unclassified | unclassified | unclassified | unclassified | Eubacteriales | Clostridia | Firmicutes |
| Oscillospiraceae sp. (HG3A.0207) | T90 | -0.068 | 1.24E-04 | 0.014 | 3249 unclassified | unclassified | unclassified | Oscillospiraceae | Eubacteriales | Clostridia | Firmicutes |

| Alistipes communis (HG3A.0064) | ODI | -0.06 | 7.74E-04 | 0.015 | 3249 unclassified | Alistipes  communis | Alistipes | Rikenellaceae | Bacteroidales | Bacteroidia | Bacteroidetes |
| --- | --- | --- | --- | --- | --- | --- | --- | --- | --- | --- | --- |
| Clostridia sp. (HG3A.0470) | T90 | -0.065 | 2.33E-04 | 0.015 | 3249 unclassified | unclassified | unclassified | unclassified | unclassified | Clostridia | Firmicutes |
| Eubacteriales sp. (HG3A.0162) | T90 | -0.067 | 1.77E-04 | 0.015 | 3249 unclassified | unclassified | unclassified | unclassified | Eubacteriales | Clostridia | Firmicutes |
| Eubacteriales sp. (HG3A.0162) | ODI | -0.06 | 7.57E-04 | 0.015 | 3249 unclassified | unclassified | unclassified | unclassified | Eubacteriales | Clostridia | Firmicutes |
| Eubacteriales sp. (HG3A.0242) | T90 | -0.066 | 1.91E-04 | 0.015 | 3249 unclassified | unclassified | unclassified | unclassified | Eubacteriales | Clostridia | Firmicutes |
| Eubacteriales sp. (HG3A.0468) | T90 | -0.066 | 2.21E-04 | 0.015 | 3249 unclassified | unclassified | unclassified | unclassified | Eubacteriales | Clostridia | Firmicutes |
| Blautia massiliensis (HG3A.0023) | ODI | 0.058 | 0.001 | 0.019 | 3249 unclassified | Blautia  massiliensis | Blautia | Lachnospiraceae | Eubacteriales | Clostridia | Firmicutes |
| Clostridia sp. (HG3A.0435) | T90 | -0.064 | 3.28E-04 | 0.020 | 3249 unclassified | unclassified | unclassified | unclassified | unclassified | Clostridia | Firmicutes |
| Eubacteriales sp. (HG3A.0100) | T90 | -0.063 | 4.31E-04 | 0.021 | 3249 unclassified | unclassified | unclassified | unclassified | Eubacteriales | Clostridia | Firmicutes |
| Eubacteriales sp. (HG3A.0118) | T90 | -0.063 | 4.33E-04 | 0.021 | 3249 unclassified | unclassified | unclassified | unclassified | Eubacteriales | Clostridia | Firmicutes |
| Pediococcus acidilactici  (HG3A.1468) | T90 | 0.062 | 4.45E-04 | 0.021 | 3249 unclassified | Pediococcus  acidilactici | Pediococcus | Lactobacillaceae | Lactobacillales | Bacilli | Firmicutes |
| Staphylococcus aureus (HG3A.1538) | T90 | 0.062 | 4.35E-04 | 0.021 | 3249 unclassified | Staphylococcus aureus | Staphylococcus | Staphylococcaceae | Bacillales | Bacilli | Firmicutes |
| Oscillospiraceae sp. (HG3A.0223) | ODI | -0.057 | 0.001 | 0.023 | 3249 unclassified | unclassified | unclassified | Oscillospiraceae | Eubacteriales | Clostridia | Firmicutes |
| [Ruminococcus] gnavus (HG3A.0239) | T90 | 0.061 | 5.62E-04 | 0.024 | 3249 unclassified | [Ruminococcus] gnavus | Mediterraneibacte r | Lachnospiraceae | Eubacteriales | Clostridia | Firmicutes |
| Eubacteriales sp. (HG3A.0123) | T90 | 0.061 | 5.73E-04 | 0.024 | 3249 unclassified | unclassified | unclassified | unclassified | Eubacteriales | Clostridia | Firmicutes |
| Eubacteriales sp. (HG3A.0196) | ODI | -0.057 | 0.001 | 0.024 | 3249 unclassified | unclassified | unclassified | unclassified | Eubacteriales | Clostridia | Firmicutes |
| Eubacteriales sp. (HG3A.0321) | ODI | -0.057 | 0.001 | 0.024 | 3249 unclassified | unclassified | unclassified | unclassified | Eubacteriales | Clostridia | Firmicutes |
| Akkermansia muciniphila (HG3A.0110) | ODI | -0.056 | 0.001 | 0.025 | 3249 unclassified | Akkermansia muciniphila | Akkermansia | Akkermansiaceae | Verrucomicrobiale s | Verrucomicrobi ae | Verrucomicrobia |
| Clostridia sp. (HG3A.0508) | T90 | -0.061 | 6.32E-04 | 0.025 | 3249 unclassified | unclassified | unclassified | unclassified | unclassified | Clostridia | Firmicutes |
| Clostridia sp. (HG3A.0599) | ODI | -0.056 | 0.002 | 0.025 | 3249 unclassified | unclassified | unclassified | unclassified | unclassified | Clostridia | Firmicutes |
| Roseburia sp. AM59-24XD  (HG3A.0391) | ODI | -0.056 | 0.002 | 0.025 | 3249 unclassified | Roseburia sp.  AM59-24XD | Roseburia | Lachnospiraceae | Eubacteriales | Clostridia | Firmicutes |
| Bacteria sp. (HG3A.0634) | T90 | -0.06 | 7.85E-04 | 0.027 | 3249 unclassified | unclassified | unclassified | unclassified | unclassified | unclassified | unclassified |
| Clostridia sp. (HG3A.0435) | ODI | -0.056 | 0.002 | 0.027 | 3249 unclassified | unclassified | unclassified | unclassified | unclassified | Clostridia | Firmicutes |
| Clostridia sp. (HG3A.0470) | ODI | -0.055 | 0.002 | 0.027 | 3249 unclassified | unclassified | unclassified | unclassified | unclassified | Clostridia | Firmicutes |
| Coprococcus comes (HG3A.0016) | T90 | 0.06 | 7.27E-04 | 0.027 | 3249 unclassified | Coprococcus  comes | Coprococcus | Lachnospiraceae | Eubacteriales | Clostridia | Firmicutes |
| Eubacteriales sp. (HG3A.0085) | ODI | -0.055 | 0.002 | 0.027 | 3249 unclassified | unclassified | unclassified | unclassified | Eubacteriales | Clostridia | Firmicutes |
| Eubacteriales sp. (HG3A.0215) | ODI | -0.055 | 0.002 | 0.027 | 3249 unclassified | unclassified | unclassified | unclassified | Eubacteriales | Clostridia | Firmicutes |
| Eubacteriales sp. (HG3A.0234) | T90 | -0.06 | 7.69E-04 | 0.027 | 3249 unclassified | unclassified | unclassified | unclassified | Eubacteriales | Clostridia | Firmicutes |

| Eubacteriales sp. (HG3A.0250) | ODI | -0.055 | 0.002 | 0.027 | 3249 unclassified | unclassified | unclassified | unclassified | Eubacteriales | Clostridia | Firmicutes |
| --- | --- | --- | --- | --- | --- | --- | --- | --- | --- | --- | --- |
| Eubacteriales sp. (HG3A.1026) | ODI | -0.056 | 0.002 | 0.027 | 3249 unclassified | unclassified | unclassified | unclassified | Eubacteriales | Clostridia | Firmicutes |
| Firmicutes sp. (HG3A.0341) | ODI | -0.056 | 0.002 | 0.027 | 3249 unclassified | unclassified | unclassified | unclassified | unclassified | unclassified | Firmicutes |
| Oscillospiraceae sp. (HG3A.0445) | ODI | -0.055 | 0.002 | 0.027 | 3249 unclassified | unclassified | unclassified | Oscillospiraceae | Eubacteriales | Clostridia | Firmicutes |
| [Ruminococcus] torques  (HG3A.0034) | ODI | 0.054 | 0.002 | 0.028 | 3249 unclassified | [Ruminococcus]  torques | Mediterraneibacte  r | Lachnospiraceae | Eubacteriales | Clostridia | Firmicutes |
| Clostridia sp. (HG3A.0094) | ODI | -0.054 | 0.002 | 0.028 | 3249 unclassified | unclassified | unclassified | unclassified | unclassified | Clostridia | Firmicutes |
| Clostridia sp. (HG3A.0508) | ODI | -0.054 | 0.002 | 0.028 | 3249 unclassified | unclassified | unclassified | unclassified | unclassified | Clostridia | Firmicutes |
| Eubacteriales sp. (HG3A.0193) | ODI | -0.055 | 0.002 | 0.028 | 3249 unclassified | unclassified | unclassified | unclassified | Eubacteriales | Clostridia | Firmicutes |
| Eubacteriales sp. (HG3A.0291) | ODI | -0.054 | 0.002 | 0.028 | 3249 unclassified | unclassified | unclassified | unclassified | Eubacteriales | Clostridia | Firmicutes |
| Eubacteriales sp. (HG3A.0309) | ODI | -0.055 | 0.002 | 0.028 | 3249 unclassified | unclassified | unclassified | unclassified | Eubacteriales | Clostridia | Firmicutes |
| Eubacteriales sp. (HG3A.0311) | T90 | -0.059 | 8.88E-04 | 0.028 | 3249 unclassified | unclassified | unclassified | unclassified | Eubacteriales | Clostridia | Firmicutes |
| Eubacteriales sp. (HG3A.0635) | T90 | -0.059 | 8.70E-04 | 0.028 | 3249 unclassified | unclassified | unclassified | unclassified | Eubacteriales | Clostridia | Firmicutes |
| Eubacteriales sp. (HG3A.0635) | ODI | -0.054 | 0.002 | 0.028 | 3249 unclassified | unclassified | unclassified | unclassified | Eubacteriales | Clostridia | Firmicutes |
| Intestinibacillus sp. Marseille-P4005 (HG3A.0168) | ODI | 0.054 | 0.002 | 0.028 | 3249 unclassified | Intestinibacillus sp. Marseille-P4005 | Intestinibacillus | Eubacteriaceae | Eubacteriales | Clostridia | Firmicutes |
| Oscillospiraceae sp. (HG3A.0437) | ODI | -0.055 | 0.002 | 0.028 | 3249 unclassified | unclassified | unclassified | Oscillospiraceae | Eubacteriales | Clostridia | Firmicutes |
| Anaerobutyricum hallii (HG3A.0012) | T90 | 0.056 | 0.001 | 0.029 | 3249 unclassified | Anaerobutyricum hallii | Anaerobutyricum | Lachnospiraceae | Eubacteriales | Clostridia | Firmicutes |
| Bacteria sp. (HG3A.0911) | T90 | -0.056 | 0.002 | 0.029 | 3249 unclassified | unclassified | unclassified | unclassified | unclassified | unclassified | unclassified |
| Blautia obeum (HG3A.0009) | T90 | 0.058 | 0.001 | 0.029 | 3249 unclassified | Blautia obeum | Blautia | Lachnospiraceae | Eubacteriales | Clostridia | Firmicutes |
| Candidatus Borkfalkiales sp.  (HG3A.1329) | T90 | -0.058 | 0.001 | 0.029 | 3249 unclassified | unclassified | unclassified | unclassified | Candidatus  Borkfalkiales | Clostridia | Firmicutes |
| Clostridia sp. (HG3A.0861) | T90 | -0.058 | 0.001 | 0.029 | 3249 unclassified | unclassified | unclassified | unclassified | unclassified | Clostridia | Firmicutes |
| Clostridia sp. (HG3A.0931) | T90 | -0.058 | 0.001 | 0.029 | 3249 unclassified | unclassified | unclassified | unclassified | unclassified | Clostridia | Firmicutes |
| Clostridia sp. (HG3A.1008) | T90 | -0.057 | 0.001 | 0.029 | 3249 unclassified | unclassified | unclassified | unclassified | unclassified | Clostridia | Firmicutes |
| Clostridiaceae sp. (HG3A.0431) | T90 | 0.058 | 0.001 | 0.029 | 3249 unclassified | unclassified | unclassified | Clostridiaceae | Eubacteriales | Clostridia | Firmicutes |
| Dorea sp. AF36-15AT (HG3A.0052) | T90 | 0.059 | 9.82E-04 | 0.029 | 3249 unclassified | Dorea sp. AF36- 15AT | Dorea | Lachnospiraceae | Eubacteriales | Clostridia | Firmicutes |
| Erysipelotrichales sp. (HG3A.1207) | T90 | 0.057 | 0.001 | 0.029 | 3249 unclassified | unclassified | unclassified | unclassified | Erysipelotrichales | Erysipelotrichia | Firmicutes |
| Eubacteriales sp. (HG3A.0211) | T90 | -0.057 | 0.001 | 0.029 | 3249 unclassified | unclassified | unclassified | unclassified | Eubacteriales | Clostridia | Firmicutes |
| Eubacteriales sp. (HG3A.0291) | T90 | -0.057 | 0.001 | 0.029 | 3249 unclassified | unclassified | unclassified | unclassified | Eubacteriales | Clostridia | Firmicutes |

| Eubacteriales sp. (HG3A.0531) | T90 | -0.058 | 0.001 | 0.029 | 3249 unclassified | unclassified | unclassified | unclassified | Eubacteriales | Clostridia | Firmicutes |
| --- | --- | --- | --- | --- | --- | --- | --- | --- | --- | --- | --- |
| Lachnospiraceae sp. (HG3A.0018) | T90 | 0.056 | 0.002 | 0.029 | 3249 unclassified | unclassified | unclassified | Lachnospiraceae | Eubacteriales | Clostridia | Firmicutes |
| Oscillibacter sp. (HG3A.0734) | T90 | -0.056 | 0.002 | 0.029 | 3249 unclassified | unclassified | Oscillibacter | Oscillospiraceae | Eubacteriales | Clostridia | Firmicutes |
| Oscillospiraceae sp. (HG3A.0072) | T90 | -0.057 | 0.001 | 0.029 | 3249 unclassified | unclassified | unclassified | Oscillospiraceae | Eubacteriales | Clostridia | Firmicutes |
| Traorella massiliensis (HG3A.0669) | T90 | -0.059 | 9.41E-04 | 0.029 | 3249 unclassified | Traorella massiliensis | Traorella | Erysipelotrichacea e | Erysipelotrichales | Erysipelotrichia | Firmicutes |
| Clostridia sp. (HG3A.0550) | T90 | -0.055 | 0.002 | 0.031 | 3249 unclassified | unclassified | unclassified | unclassified | unclassified | Clostridia | Firmicutes |
| Clostridia sp. (HG3A.0645) | T90 | -0.055 | 0.002 | 0.031 | 3249 unclassified | unclassified | unclassified | unclassified | unclassified | Clostridia | Firmicutes |
| Eubacteriales sp. (HG3A.0084) | ODI | -0.053 | 0.003 | 0.031 | 3249 unclassified | unclassified | unclassified | unclassified | Eubacteriales | Clostridia | Firmicutes |
| Eubacteriales sp. (HG3A.0321) | T90 | -0.055 | 0.002 | 0.031 | 3249 unclassified | unclassified | unclassified | unclassified | Eubacteriales | Clostridia | Firmicutes |
| Eubacteriales sp. (HG3A.0506) | T90 | -0.055 | 0.002 | 0.031 | 3249 unclassified | unclassified | unclassified | unclassified | Eubacteriales | Clostridia | Firmicutes |
| Eubacteriales sp. (HG3A.1379) | T90 | -0.056 | 0.002 | 0.031 | 3249 unclassified | unclassified | unclassified | unclassified | Eubacteriales | Clostridia | Firmicutes |
| Firmicutes sp. (HG3A.0301) | T90 | -0.056 | 0.002 | 0.031 | 3249 unclassified | unclassified | unclassified | unclassified | unclassified | unclassified | Firmicutes |
| Intestinimonas massiliensis (HG3A.0198) | ODI | -0.053 | 0.003 | 0.031 | 3249 unclassified | Intestinimonas massiliensis | Intestinimonas | unclassified | Eubacteriales | Clostridia | Firmicutes |
| Oscillospiraceae sp. (HG3A.0445) | T90 | -0.055 | 0.002 | 0.031 | 3249 unclassified | unclassified | unclassified | Oscillospiraceae | Eubacteriales | Clostridia | Firmicutes |
| Clostridium sp. (HG3A.0050) | ODI | 0.053 | 0.003 | 0.033 | 3249 unclassified | unclassified | Clostridium | Clostridiaceae | Eubacteriales | Clostridia | Firmicutes |
| Eubacterium sp. (HG3A.0214) | ODI | 0.053 | 0.003 | 0.033 | 3249 unclassified | unclassified | Eubacterium | Eubacteriaceae | Eubacteriales | Clostridia | Firmicutes |
| Oscillospiraceae sp. (HG3A.1270) | ODI | -0.053 | 0.003 | 0.033 | 3249 unclassified | unclassified | unclassified | Oscillospiraceae | Eubacteriales | Clostridia | Firmicutes |
| Eubacteriales sp. (HG3A.0630) | T90 | -0.055 | 0.002 | 0.034 | 3249 unclassified | unclassified | unclassified | unclassified | Eubacteriales | Clostridia | Firmicutes |
| Clostridia sp. (HG3A.0515) | T90 | -0.054 | 0.002 | 0.036 | 3249 unclassified | unclassified | unclassified | unclassified | unclassified | Clostridia | Firmicutes |
| Coprococcus eutactus (HG3A.0155) | ODI | -0.052 | 0.003 | 0.036 | 3249 unclassified | Coprococcus  eutactus | Coprococcus | Lachnospiraceae | Eubacteriales | Clostridia | Firmicutes |
| Clostridia sp. (HG3A.0815) | ODI | -0.052 | 0.003 | 0.037 | 3249 unclassified | unclassified | unclassified | unclassified | unclassified | Clostridia | Firmicutes |
| Collinsella aerofaciens (HG3A.0019) | ODI | 0.052 | 0.003 | 0.037 | 3249 unclassified | Collinsella  aerofaciens | Collinsella | Coriobacteriaceae | Coriobacteriales | Coriobacteriia | Actinobacteria |
| Coprobacillus sp. (HG3A.0022) | ODI | 0.052 | 0.003 | 0.037 | 3249 unclassified | unclassified | Coprobacillus | Coprobacillaceae | Erysipelotrichales | Erysipelotrichia | Firmicutes |
| Clostridia sp. (HG3A.1057) | T90 | -0.053 | 0.003 | 0.041 | 3249 unclassified | unclassified | unclassified | unclassified | unclassified | Clostridia | Firmicutes |
| Clostridium sp. (HG3A.0050) | T90 | 0.053 | 0.003 | 0.041 | 3249 unclassified | unclassified | Clostridium | Clostridiaceae | Eubacteriales | Clostridia | Firmicutes |
| Eubacteriales sp. (HG3A.0383) | T90 | -0.053 | 0.003 | 0.041 | 3249 unclassified | unclassified | unclassified | unclassified | Eubacteriales | Clostridia | Firmicutes |
| Ruminococcus sp. AM42-11 (HG3A.0002) | T90 | 0.053 | 0.003 | 0.041 | 3249 unclassified | Ruminococcus sp. AM42-11 | Ruminococcus | Oscillospiraceae | Eubacteriales | Clostridia | Firmicutes |
| Eggerthellaceae sp. (HG3A.0171) | ODI | -0.051 | 0.004 | 0.042 | 3249 unclassified | unclassified | unclassified | Eggerthellaceae | Eggerthellales | Coriobacteriia | Actinobacteria |

| Eubacteriales sp. (HG3A.0439) | ODI | -0.051 | 0.004 | 0.042 | 3249 unclassified | unclassified | unclassified | unclassified | Eubacteriales | Clostridia | Firmicutes |
| --- | --- | --- | --- | --- | --- | --- | --- | --- | --- | --- | --- |
| Eubacteriales sp. (HG3A.0548) | ODI | -0.051 | 0.004 | 0.042 | 3249 unclassified | unclassified | unclassified | unclassified | Eubacteriales | Clostridia | Firmicutes |
| Eubacteriales sp. (HG3A.0691) | ODI | -0.051 | 0.004 | 0.042 | 3249 unclassified | unclassified | unclassified | unclassified | Eubacteriales | Clostridia | Firmicutes |
| Eubacteriales sp. (HG3A.0718) | ODI | -0.051 | 0.004 | 0.042 | 3249 unclassified | unclassified | unclassified | unclassified | Eubacteriales | Clostridia | Firmicutes |
| Eubacteriales sp. (HG3A.1019) | ODI | -0.051 | 0.004 | 0.042 | 3249 unclassified | unclassified | unclassified | unclassified | Eubacteriales | Clostridia | Firmicutes |
| Firmicutes sp. (HG3A.1085) | ODI | -0.051 | 0.004 | 0.042 | 3249 unclassified | unclassified | unclassified | unclassified | unclassified | unclassified | Firmicutes |
| Eubacteriales sp. (HG3A.0978) | T90 | -0.053 | 0.003 | 0.043 | 3249 unclassified | unclassified | unclassified | unclassified | Eubacteriales | Clostridia | Firmicutes |
| Anaerostipes sp. BG01 (HG3A.1509) | ODI | 0.05 | 0.005 | 0.044 | 3249 unclassified | Anaerostipes sp.  BG01 | Anaerostipes | Lachnospiraceae | Eubacteriales | Clostridia | Firmicutes |
| Candidatus Borkfalkiales sp. (HG3A.1397) | ODI | -0.05 | 0.005 | 0.044 | 3249 unclassified | unclassified | unclassified | unclassified | Candidatus Borkfalkiales | Clostridia | Firmicutes |
| Eubacteriales sp. (HG3A.0120) | ODI | -0.05 | 0.005 | 0.044 | 3249 unclassified | unclassified | unclassified | unclassified | Eubacteriales | Clostridia | Firmicutes |
| Eubacteriales sp. (HG3A.0153) | ODI | -0.05 | 0.005 | 0.044 | 3249 unclassified | unclassified | unclassified | unclassified | Eubacteriales | Clostridia | Firmicutes |
| Eubacteriales sp. (HG3A.0196) | T90 | -0.052 | 0.003 | 0.044 | 3249 unclassified | unclassified | unclassified | unclassified | Eubacteriales | Clostridia | Firmicutes |
| Eubacteriales sp. (HG3A.0956) | ODI | -0.05 | 0.005 | 0.044 | 3249 unclassified | unclassified | unclassified | unclassified | Eubacteriales | Clostridia | Firmicutes |
| Mediterraneibacter glycyrrhizinilyticus (HG3A.0314) | ODI | 0.05 | 0.005 | 0.044 | 3249 unclassified | Mediterraneibacter glycyrrhizinilyticus | Mediterraneibacte r | Lachnospiraceae | Eubacteriales | Clostridia | Firmicutes |
| Clostridia sp. (HG3A.0879) | ODI | -0.05 | 0.005 | 0.046 | 3249 unclassified | unclassified | unclassified | unclassified | unclassified | Clostridia | Firmicutes |
| Clostridia sp. (HG3A.1020) | ODI | -0.049 | 0.005 | 0.047 | 3249 unclassified | unclassified | unclassified | unclassified | unclassified | Clostridia | Firmicutes |
| Eubacteriales sp. (HG3A.0083) | ODI | -0.049 | 0.005 | 0.047 | 3249 unclassified | unclassified | unclassified | unclassified | Eubacteriales | Clostridia | Firmicutes |
| Eubacteriales sp. (HG3A.0489) | T90 | -0.052 | 0.004 | 0.047 | 3249 unclassified | unclassified | unclassified | unclassified | Eubacteriales | Clostridia | Firmicutes |
| Eubacteriales sp. (HG3A.0786) | T90 | 0.052 | 0.004 | 0.047 | 3249 unclassified | unclassified | unclassified | unclassified | Eubacteriales | Clostridia | Firmicutes |
| Flavonifractor plautii (HG3A.0079) | ODI | 0.05 | 0.005 | 0.047 | 3249 unclassified | Flavonifractor  plautii | Flavonifractor | Oscillospiraceae | Eubacteriales | Clostridia | Firmicutes |
| Alistipes provencensis (HG3A.0877) | ODI | -0.049 | 0.006 | 0.048 | 3249 unclassified | Alistipes provencensis | Alistipes | Rikenellaceae | Bacteroidales | Bacteroidia | Bacteroidetes |
| Eubacteriales sp. (HG3A.0229) | ODI | -0.049 | 0.006 | 0.048 | 3249 unclassified | unclassified | unclassified | unclassified | Eubacteriales | Clostridia | Firmicutes |
| Eubacteriales sp. (HG3A.0329) | ODI | -0.049 | 0.006 | 0.048 | 3249 unclassified | unclassified | unclassified | unclassified | Eubacteriales | Clostridia | Firmicutes |
| Eubacteriales sp. (HG3A.0419) | ODI | -0.049 | 0.006 | 0.048 | 3249 unclassified | unclassified | unclassified | unclassified | Eubacteriales | Clostridia | Firmicutes |
| Eubacteriales sp. (HG3A.1294) | ODI | -0.049 | 0.006 | 0.048 | 3249 unclassified | unclassified | unclassified | unclassified | Eubacteriales | Clostridia | Firmicutes |
| Lachnospiraceae sp. (HG3A.0855) | ODI | -0.049 | 0.006 | 0.048 | 3249 unclassified | unclassified | unclassified | Lachnospiraceae | Eubacteriales | Clostridia | Firmicutes |
| Eubacteriales sp. (HG3A.0269) | T90 | -0.051 | 0.004 | 0.050 | 3249 unclassified | unclassified | unclassified | unclassified | Eubacteriales | Clostridia | Firmicutes |
| Eubacteriales sp. (HG3A.0284) | ODI | -0.049 | 0.006 | 0.050 | 3249 unclassified | unclassified | unclassified | unclassified | Eubacteriales | Clostridia | Firmicutes |
| Eubacteriales sp. (HG3A.0731) | ODI | -0.049 | 0.006 | 0.050 | 3249 unclassified | unclassified | unclassified | unclassified | Eubacteriales | Clostridia | Firmicutes |
| Eubacteriales sp. (HG3A.0760) | T90 | -0.051 | 0.004 | 0.050 | 3249 unclassified | unclassified | unclassified | unclassified | Eubacteriales | Clostridia | Firmicutes |

| Candidatus Borkfalkiales sp.  (HG3A.1284) | ODI | -0.048 | 0.007 | 0.052 | 3249 unclassified | unclassified | unclassified | unclassified | Candidatus  Borkfalkiales | Clostridia | Firmicutes |
| --- | --- | --- | --- | --- | --- | --- | --- | --- | --- | --- | --- |
| Dorea formicigenerans (HG3A.0006) | ODI | 0.048 | 0.007 | 0.052 | 3249 unclassified | Dorea  formicigenerans | Dorea | Lachnospiraceae | Eubacteriales | Clostridia | Firmicutes |
| Eubacteriales sp. (HG3A.0189) | ODI | -0.048 | 0.007 | 0.052 | 3249 unclassified | unclassified | unclassified | unclassified | Eubacteriales | Clostridia | Firmicutes |
| [Ruminococcus] torques  (HG3A.0034) | T90 | 0.05 | 0.005 | 0.053 | 3249 unclassified | [Ruminococcus]  torques | Mediterraneibacte  r | Lachnospiraceae | Eubacteriales | Clostridia | Firmicutes |
| Clostridia sp. (HG3A.0272) | T90 | -0.051 | 0.004 | 0.053 | 3249 unclassified | unclassified | unclassified | unclassified | unclassified | Clostridia | Firmicutes |
| Clostridia sp. (HG3A.0783) | T90 | -0.051 | 0.004 | 0.053 | 3249 unclassified | unclassified | unclassified | unclassified | unclassified | Clostridia | Firmicutes |
| Eubacteriales sp. (HG3A.0125) | T90 | -0.05 | 0.005 | 0.053 | 3249 unclassified | unclassified | unclassified | unclassified | Eubacteriales | Clostridia | Firmicutes |
| Eubacteriales sp. (HG3A.0509) | T90 | -0.051 | 0.004 | 0.053 | 3249 unclassified | unclassified | unclassified | unclassified | Eubacteriales | Clostridia | Firmicutes |
| Eubacteriales sp. (HG3A.0977) | T90 | -0.051 | 0.004 | 0.053 | 3249 unclassified | unclassified | unclassified | unclassified | Eubacteriales | Clostridia | Firmicutes |
| Eubacteriales sp. (HG3A.1086) | ODI | -0.048 | 0.007 | 0.053 | 3249 unclassified | unclassified | unclassified | unclassified | Eubacteriales | Clostridia | Firmicutes |
| Lactobacillus acidophilus (HG3A.0672) | T90 | -0.05 | 0.005 | 0.053 | 3249 unclassified | Lactobacillus acidophilus | Lactobacillus | Lactobacillaceae | Lactobacillales | Bacilli | Firmicutes |
| Clostridia sp. (HG3A.1053) | ODI | -0.048 | 0.007 | 0.054 | 3249 unclassified | unclassified | unclassified | unclassified | unclassified | Clostridia | Firmicutes |
| Eubacteriales sp. (HG3A.0270) | ODI | -0.048 | 0.007 | 0.054 | 3249 unclassified | unclassified | unclassified | unclassified | Eubacteriales | Clostridia | Firmicutes |
| Eubacteriales sp. (HG3A.0288) | ODI | -0.048 | 0.007 | 0.054 | 3249 unclassified | unclassified | unclassified | unclassified | Eubacteriales | Clostridia | Firmicutes |
| Eubacteriales sp. (HG3A.0572) | T90 | -0.05 | 0.005 | 0.054 | 3249 unclassified | unclassified | unclassified | unclassified | Eubacteriales | Clostridia | Firmicutes |
| Flavonifractor plautii (HG3A.0079) | T90 | 0.05 | 0.005 | 0.054 | 3249 unclassified | Flavonifractor  plautii | Flavonifractor | Oscillospiraceae | Eubacteriales | Clostridia | Firmicutes |
| Clostridia sp. (HG3A.0660) | ODI | -0.047 | 0.008 | 0.056 | 3249 unclassified | unclassified | unclassified | unclassified | unclassified | Clostridia | Firmicutes |
| Eubacteriales sp. (HG3A.0316) | ODI | -0.047 | 0.008 | 0.056 | 3249 unclassified | unclassified | unclassified | unclassified | Eubacteriales | Clostridia | Firmicutes |
| Oscillibacter sp. (HG3A.0245) | ODI | -0.047 | 0.008 | 0.056 | 3249 unclassified | unclassified | Oscillibacter | Oscillospiraceae | Eubacteriales | Clostridia | Firmicutes |
| Oscillospiraceae sp. (HG3A.1173) | ODI | -0.047 | 0.008 | 0.056 | 3249 unclassified | unclassified | unclassified | Oscillospiraceae | Eubacteriales | Clostridia | Firmicutes |
| Clostridia sp. (HG3A.0385) | T90 | -0.05 | 0.005 | 0.057 | 3249 unclassified | unclassified | unclassified | unclassified | unclassified | Clostridia | Firmicutes |
| Eubacteriales sp. (HG3A.0125) | ODI | -0.047 | 0.008 | 0.057 | 3249 unclassified | unclassified | unclassified | unclassified | Eubacteriales | Clostridia | Firmicutes |
| Eubacteriales sp. (HG3A.0154) | ODI | -0.047 | 0.008 | 0.059 | 3249 unclassified | unclassified | unclassified | unclassified | Eubacteriales | Clostridia | Firmicutes |
| Eubacteriales sp. (HG3A.0282) | ODI | -0.047 | 0.008 | 0.059 | 3249 unclassified | unclassified | unclassified | unclassified | Eubacteriales | Clostridia | Firmicutes |
| Firmicutes sp. (HG3A.0681) | ODI | -0.047 | 0.009 | 0.059 | 3249 unclassified | unclassified | unclassified | unclassified | unclassified | unclassified | Firmicutes |
| Clostridia sp. (HG3A.0931) | ODI | -0.046 | 0.009 | 0.060 | 3249 unclassified | unclassified | unclassified | unclassified | unclassified | Clostridia | Firmicutes |
| Eubacteriales sp. (HG3A.0580) | T90 | -0.049 | 0.006 | 0.060 | 3249 unclassified | unclassified | unclassified | unclassified | Eubacteriales | Clostridia | Firmicutes |

| Eubacteriales sp. (HG3A.0600) | T90 | -0.049 | 0.006 | 0.060 | 3249 unclassified | unclassified | unclassified | unclassified | Eubacteriales | Clostridia | Firmicutes |
| --- | --- | --- | --- | --- | --- | --- | --- | --- | --- | --- | --- |
| Eubacteriales sp. (HG3A.0864) | ODI | -0.047 | 0.009 | 0.060 | 3249 unclassified | unclassified | unclassified | unclassified | Eubacteriales | Clostridia | Firmicutes |
| Oscillibacter sp. (HG3A.0734) | ODI | -0.046 | 0.009 | 0.060 | 3249 unclassified | unclassified | Oscillibacter | Oscillospiraceae | Eubacteriales | Clostridia | Firmicutes |
| Alistipes shahii (HG3A.0054) | T90 | -0.049 | 0.006 | 0.061 | 3249 unclassified | Alistipes shahii | Alistipes | Rikenellaceae | Bacteroidales | Bacteroidia | Bacteroidetes |
| Clostridia sp. (HG3A.0463) | T90 | -0.048 | 0.007 | 0.061 | 3249 unclassified | unclassified | unclassified | unclassified | unclassified | Clostridia | Firmicutes |
| Clostridia sp. (HG3A.0815) | T90 | -0.049 | 0.006 | 0.061 | 3249 unclassified | unclassified | unclassified | unclassified | unclassified | Clostridia | Firmicutes |
| Clostridium sp. AT4 (HG3A.0347) | T90 | 0.049 | 0.006 | 0.061 | 3249 unclassified | Clostridium sp.  AT4 | Clostridium | Clostridiaceae | Eubacteriales | Clostridia | Firmicutes |
| Eubacteriales sp. (HG3A.0329) | T90 | -0.048 | 0.007 | 0.061 | 3249 unclassified | unclassified | unclassified | unclassified | Eubacteriales | Clostridia | Firmicutes |
| Eubacteriales sp. (HG3A.0373) | T90 | -0.049 | 0.006 | 0.061 | 3249 unclassified | unclassified | unclassified | unclassified | Eubacteriales | Clostridia | Firmicutes |
| Eubacteriales sp. (HG3A.0442) | T90 | -0.049 | 0.006 | 0.061 | 3249 unclassified | unclassified | unclassified | unclassified | Eubacteriales | Clostridia | Firmicutes |
| Eubacteriales sp. (HG3A.0698) | T90 | 0.049 | 0.006 | 0.061 | 3249 unclassified | unclassified | unclassified | unclassified | Eubacteriales | Clostridia | Firmicutes |
| Oscillospiraceae sp. (HG3A.0256) | T90 | 0.048 | 0.007 | 0.061 | 3249 unclassified | unclassified | unclassified | Oscillospiraceae | Eubacteriales | Clostridia | Firmicutes |
| Oscillospiraceae sp. (HG3A.0384) | T90 | -0.049 | 0.006 | 0.061 | 3249 unclassified | unclassified | unclassified | Oscillospiraceae | Eubacteriales | Clostridia | Firmicutes |
| Oscillospiraceae sp. (HG3A.0739) | T90 | -0.048 | 0.006 | 0.061 | 3249 unclassified | unclassified | unclassified | Oscillospiraceae | Eubacteriales | Clostridia | Firmicutes |
| Blautia sp. SG-772 (HG3A.0063) | ODI | 0.046 | 0.01 | 0.062 | 3249 unclassified | Blautia sp. SG-772 | Blautia | Lachnospiraceae | Eubacteriales | Clostridia | Firmicutes |
| Clostridia sp. (HG3A.0645) | ODI | -0.046 | 0.01 | 0.062 | 3249 unclassified | unclassified | unclassified | unclassified | unclassified | Clostridia | Firmicutes |
| Clostridia sp. (HG3A.0756) | ODI | -0.046 | 0.009 | 0.062 | 3249 unclassified | unclassified | unclassified | unclassified | unclassified | Clostridia | Firmicutes |
| Eubacteriales sp. (HG3A.0102) | ODI | -0.046 | 0.01 | 0.062 | 3249 unclassified | unclassified | unclassified | unclassified | Eubacteriales | Clostridia | Firmicutes |
| Eubacteriales sp. (HG3A.0179) | ODI | -0.046 | 0.01 | 0.062 | 3249 unclassified | unclassified | unclassified | unclassified | Eubacteriales | Clostridia | Firmicutes |
| Eubacteriales sp. (HG3A.0342) | ODI | -0.046 | 0.01 | 0.062 | 3249 unclassified | unclassified | unclassified | unclassified | Eubacteriales | Clostridia | Firmicutes |
| Eubacteriales sp. (HG3A.0624) | ODI | -0.046 | 0.009 | 0.062 | 3249 unclassified | unclassified | unclassified | unclassified | Eubacteriales | Clostridia | Firmicutes |
| Firmicutes sp. (HG3A.0501) | ODI | -0.046 | 0.01 | 0.062 | 3249 unclassified | unclassified | unclassified | unclassified | unclassified | unclassified | Firmicutes |
| Holdemanella sp. (HG3A.0366) | ODI | 0.046 | 0.01 | 0.062 | 3249 unclassified | unclassified | Holdemanella | Erysipelotrichacea  e | Erysipelotrichales | Erysipelotrichia | Firmicutes |
| Lachnospiraceae sp. (HG3A.0180) | ODI | -0.046 | 0.01 | 0.062 | 3249 unclassified | unclassified | unclassified | Lachnospiraceae | Eubacteriales | Clostridia | Firmicutes |
| Oscillospiraceae sp. (HG3A.0429) | ODI | -0.046 | 0.01 | 0.062 | 3249 unclassified | unclassified | unclassified | Oscillospiraceae | Eubacteriales | Clostridia | Firmicutes |
| Clostridia sp. (HG3A.0746) | ODI | -0.046 | 0.01 | 0.063 | 3249 unclassified | unclassified | unclassified | unclassified | unclassified | Clostridia | Firmicutes |

| Clostridia sp. (HG3A.0741) | ODI | -0.045 | 0.01 | 0.064 | 3249 unclassified | unclassified | unclassified | unclassified | unclassified | Clostridia | Firmicutes |
| --- | --- | --- | --- | --- | --- | --- | --- | --- | --- | --- | --- |
| Clostridia sp. (HG3A.0929) | T90 | -0.048 | 0.007 | 0.064 | 3249 unclassified | unclassified | unclassified | unclassified | unclassified | Clostridia | Firmicutes |
| Eubacteriales sp. (HG3A.0144) | T90 | -0.048 | 0.007 | 0.064 | 3249 unclassified | unclassified | unclassified | unclassified | Eubacteriales | Clostridia | Firmicutes |
| Eubacteriales sp. (HG3A.0516) | T90 | -0.048 | 0.007 | 0.064 | 3249 unclassified | unclassified | unclassified | unclassified | Eubacteriales | Clostridia | Firmicutes |
| Intestinibacillus sp. Marseille-P4005  (HG3A.0168) | T90 | 0.048 | 0.007 | 0.064 | 3249 unclassified | Intestinibacillus sp.  Marseille-P4005 | Intestinibacillus | Eubacteriaceae | Eubacteriales | Clostridia | Firmicutes |
| Clostridia sp. (HG3A.0733) | ODI | -0.045 | 0.011 | 0.065 | 3249 unclassified | unclassified | unclassified | unclassified | unclassified | Clostridia | Firmicutes |
| Eubacteriales sp. (HG3A.0383) | ODI | -0.045 | 0.011 | 0.065 | 3249 unclassified | unclassified | unclassified | unclassified | Eubacteriales | Clostridia | Firmicutes |
| Eubacteriales sp. (HG3A.0964) | ODI | -0.045 | 0.011 | 0.065 | 3249 unclassified | unclassified | unclassified | unclassified | Eubacteriales | Clostridia | Firmicutes |
| Eubacteriales sp. (HG3A.0443) | ODI | -0.045 | 0.011 | 0.066 | 3249 unclassified | unclassified | unclassified | unclassified | Eubacteriales | Clostridia | Firmicutes |
| Eubacteriales sp. (HG3A.0573) | ODI | -0.045 | 0.011 | 0.066 | 3249 unclassified | unclassified | unclassified | unclassified | Eubacteriales | Clostridia | Firmicutes |
| Eubacteriales sp. (HG3A.0610) | T90 | 0.048 | 0.007 | 0.066 | 3249 unclassified | unclassified | unclassified | unclassified | Eubacteriales | Clostridia | Firmicutes |
| Eubacteriales sp. (HG3A.1103) | ODI | -0.045 | 0.011 | 0.066 | 3249 unclassified | unclassified | unclassified | unclassified | Eubacteriales | Clostridia | Firmicutes |
| Firmicutes sp. (HG3A.0436) | ODI | -0.045 | 0.011 | 0.066 | 3249 unclassified | unclassified | unclassified | unclassified | unclassified | unclassified | Firmicutes |
| Bacteria sp. (HG3A.0483) | T90 | -0.047 | 0.008 | 0.069 | 3249 unclassified | unclassified | unclassified | unclassified | unclassified | unclassified | unclassified |
| Eubacteriales sp. (HG3A.0482) | T90 | -0.047 | 0.008 | 0.069 | 3249 unclassified | unclassified | unclassified | unclassified | Eubacteriales | Clostridia | Firmicutes |
| Eubacteriales sp. (HG3A.0593) | T90 | -0.047 | 0.008 | 0.069 | 3249 unclassified | unclassified | unclassified | unclassified | Eubacteriales | Clostridia | Firmicutes |
| Eubacteriales sp. (HG3A.0771) | T90 | -0.047 | 0.008 | 0.069 | 3249 unclassified | unclassified | unclassified | unclassified | Eubacteriales | Clostridia | Firmicutes |
| Alistipes provencensis (HG3A.0877) | T90 | -0.047 | 0.009 | 0.071 | 3249 unclassified | Alistipes provencensis | Alistipes | Rikenellaceae | Bacteroidales | Bacteroidia | Bacteroidetes |
| Eubacteriales sp. (HG3A.0426) | T90 | -0.047 | 0.009 | 0.071 | 3249 unclassified | unclassified | unclassified | unclassified | Eubacteriales | Clostridia | Firmicutes |
| Firmicutes sp. (HG3A.0398) | T90 | -0.047 | 0.009 | 0.071 | 3249 unclassified | unclassified | unclassified | unclassified | unclassified | unclassified | Firmicutes |
| Oscillospiraceae sp. (HG3A.0388) | ODI | -0.044 | 0.012 | 0.071 | 3249 unclassified | unclassified | unclassified | Oscillospiraceae | Eubacteriales | Clostridia | Firmicutes |
| Eubacteriales sp. (HG3A.0235) | ODI | -0.044 | 0.013 | 0.072 | 3249 unclassified | unclassified | unclassified | unclassified | Eubacteriales | Clostridia | Firmicutes |
| Eubacteriales sp. (HG3A.1377) | ODI | -0.044 | 0.013 | 0.072 | 3249 unclassified | unclassified | unclassified | unclassified | Eubacteriales | Clostridia | Firmicutes |
| Eubacteriales sp. (HG3A.0396) | T90 | -0.046 | 0.009 | 0.073 | 3249 unclassified | unclassified | unclassified | unclassified | Eubacteriales | Clostridia | Firmicutes |
| Sutterella seckii (HG3A.0561) | ODI | 0.044 | 0.013 | 0.074 | 3249 unclassified | Sutterella seckii | Sutterella | Sutterellaceae | Burkholderiales | Betaproteobacte  ria | Proteobacteria |
| Bacteroidales sp. (HG3A.0789) | T90 | -0.046 | 0.009 | 0.075 | 3249 unclassified | unclassified | unclassified | unclassified | Bacteroidales | Bacteroidia | Bacteroidetes |
| Blautia hydrogenotrophica  (HG3A.0430) | T90 | 0.046 | 0.009 | 0.075 | 3249 unclassified | Blautia  hydrogenotrophica | Blautia | Lachnospiraceae | Eubacteriales | Clostridia | Firmicutes |
| Eubacteriales sp. (HG3A.0062) | ODI | -0.044 | 0.014 | 0.075 | 3249 unclassified | unclassified | unclassified | unclassified | Eubacteriales | Clostridia | Firmicutes |
| Eubacteriales sp. (HG3A.0249) | ODI | -0.044 | 0.014 | 0.075 | 3249 unclassified | unclassified | unclassified | unclassified | Eubacteriales | Clostridia | Firmicutes |

| Eubacteriales sp. (HG3A.0409) | ODI | -0.044 | 0.014 | 0.075 | 3249 unclassified | unclassified | unclassified | unclassified | Eubacteriales | Clostridia | Firmicutes |
| --- | --- | --- | --- | --- | --- | --- | --- | --- | --- | --- | --- |
| Eubacteriales sp. (HG3A.0578) | ODI | -0.044 | 0.014 | 0.075 | 3249 unclassified | unclassified | unclassified | unclassified | Eubacteriales | Clostridia | Firmicutes |
| Eubacteriales sp. (HG3A.0617) | ODI | -0.044 | 0.014 | 0.075 | 3249 unclassified | unclassified | unclassified | unclassified | Eubacteriales | Clostridia | Firmicutes |
| Eubacteriales sp. (HG3A.0698) | ODI | 0.044 | 0.014 | 0.075 | 3249 unclassified | unclassified | unclassified | unclassified | Eubacteriales | Clostridia | Firmicutes |
| Eubacteriales sp. (HG3A.0864) | T90 | -0.046 | 0.009 | 0.075 | 3249 unclassified | unclassified | unclassified | unclassified | Eubacteriales | Clostridia | Firmicutes |
| Eubacteriales sp. (HG3A.1191) | ODI | -0.044 | 0.014 | 0.075 | 3249 unclassified | unclassified | unclassified | unclassified | Eubacteriales | Clostridia | Firmicutes |
| Olsenella sp. AF21-51 (HG3A.0690) | ODI | -0.044 | 0.014 | 0.075 | 3249 unclassified | Olsenella sp. AF21- 51 | Olsenella | Atopobiaceae | Coriobacteriales | Coriobacteriia | Actinobacteria |
| Dorea sp. AF36-15AT (HG3A.0052) | ODI | 0.043 | 0.015 | 0.076 | 3249 unclassified | Dorea sp. AF36-  15AT | Dorea | Lachnospiraceae | Eubacteriales | Clostridia | Firmicutes |
| Eubacteriales sp. (HG3A.0230) | ODI | -0.043 | 0.014 | 0.076 | 3249 unclassified | unclassified | unclassified | unclassified | Eubacteriales | Clostridia | Firmicutes |
| Firmicutes sp. (HG3A.0341) | T90 | -0.046 | 0.01 | 0.076 | 3249 unclassified | unclassified | unclassified | unclassified | unclassified | unclassified | Firmicutes |
| Alistipes shahii (HG3A.0054) | AHI | -0.059 | 0.002 | 0.077 | 2909 unclassified | Alistipes shahii | Alistipes | Rikenellaceae | Bacteroidales | Bacteroidia | Bacteroidetes |
| Bacteria sp. (HG3A.0634) | AHI | -0.059 | 0.002 | 0.077 | 2909 unclassified | unclassified | unclassified | unclassified | unclassified | unclassified | unclassified |
| Clostridia sp. (HG3A.0515) | AHI | -0.063 | 7.53E-04 | 0.077 | 2909 unclassified | unclassified | unclassified | unclassified | unclassified | Clostridia | Firmicutes |
| Clostridia sp. (HG3A.0682) | AHI | -0.066 | 4.42E-04 | 0.077 | 2909 unclassified | unclassified | unclassified | unclassified | unclassified | Clostridia | Firmicutes |
| Clostridia sp. (HG3A.0728) | AHI | -0.06 | 0.001 | 0.077 | 2909 unclassified | unclassified | unclassified | unclassified | unclassified | Clostridia | Firmicutes |
| Eubacteriales sp. (HG3A.0069) | AHI | -0.065 | 5.59E-04 | 0.077 | 2909 unclassified | unclassified | unclassified | unclassified | Eubacteriales | Clostridia | Firmicutes |
| Eubacteriales sp. (HG3A.0084) | AHI | -0.061 | 0.001 | 0.077 | 2909 unclassified | unclassified | unclassified | unclassified | Eubacteriales | Clostridia | Firmicutes |
| Eubacteriales sp. (HG3A.0085) | AHI | -0.062 | 8.89E-04 | 0.077 | 2909 unclassified | unclassified | unclassified | unclassified | Eubacteriales | Clostridia | Firmicutes |
| Eubacteriales sp. (HG3A.0156) | AHI | -0.059 | 0.002 | 0.077 | 2909 unclassified | unclassified | unclassified | unclassified | Eubacteriales | Clostridia | Firmicutes |
| Eubacteriales sp. (HG3A.0197) | AHI | -0.06 | 0.001 | 0.077 | 2909 unclassified | unclassified | unclassified | unclassified | Eubacteriales | Clostridia | Firmicutes |
| Eubacteriales sp. (HG3A.0230) | AHI | -0.061 | 0.001 | 0.077 | 2909 unclassified | unclassified | unclassified | unclassified | Eubacteriales | Clostridia | Firmicutes |
| Eubacteriales sp. (HG3A.0269) | AHI | -0.059 | 0.002 | 0.077 | 2909 unclassified | unclassified | unclassified | unclassified | Eubacteriales | Clostridia | Firmicutes |
| Eubacteriales sp. (HG3A.0572) | AHI | -0.063 | 7.82E-04 | 0.077 | 2909 unclassified | unclassified | unclassified | unclassified | Eubacteriales | Clostridia | Firmicutes |
| Eubacteriales sp. (HG3A.0757) | ODI | -0.043 | 0.015 | 0.077 | 3249 unclassified | unclassified | unclassified | unclassified | Eubacteriales | Clostridia | Firmicutes |
| Eubacteriales sp. (HG3A.0829) | AHI | -0.07 | 2.08E-04 | 0.077 | 2909 unclassified | unclassified | unclassified | unclassified | Eubacteriales | Clostridia | Firmicutes |
| Firmicutes sp. (HG3A.0341) | AHI | -0.059 | 0.002 | 0.077 | 2909 unclassified | unclassified | unclassified | unclassified | unclassified | unclassified | Firmicutes |
| Firmicutes sp. (HG3A.0397) | AHI | -0.066 | 4.86E-04 | 0.077 | 2909 unclassified | unclassified | unclassified | unclassified | unclassified | unclassified | Firmicutes |
| Firmicutes sp. (HG3A.0398) | AHI | -0.065 | 5.36E-04 | 0.077 | 2909 unclassified | unclassified | unclassified | unclassified | unclassified | unclassified | Firmicutes |
| Fusicatenibacter saccharivorans (HG3A.0004) | AHI | 0.059 | 0.002 | 0.077 | 2909 unclassified | Fusicatenibacter saccharivorans | Fusicatenibacter | Lachnospiraceae | Eubacteriales | Clostridia | Firmicutes |

| Lachnospiraceae sp. (HG3A.0236) | ODI | -0.043 | 0.015 | 0.077 | 3249 unclassified | unclassified | unclassified | Lachnospiraceae | Eubacteriales | Clostridia | Firmicutes |
| --- | --- | --- | --- | --- | --- | --- | --- | --- | --- | --- | --- |
| Oscillospiraceae sp. (HG3A.0207) | AHI | -0.064 | 7.18E-04 | 0.077 | 2909 unclassified | unclassified | unclassified | Oscillospiraceae | Eubacteriales | Clostridia | Firmicutes |
| Bacteria sp. (HG3A.0361) | T90 | -0.046 | 0.01 | 0.078 | 3249 unclassified | unclassified | unclassified | unclassified | unclassified | unclassified | unclassified |
| Collinsella aerofaciens (HG3A.0019) | T90 | 0.046 | 0.01 | 0.078 | 3249 unclassified | Collinsella aerofaciens | Collinsella | Coriobacteriaceae | Coriobacteriales | Coriobacteriia | Actinobacteria |
| Coprococcus sp. (HG3A.0404) | T90 | 0.045 | 0.011 | 0.078 | 3249 unclassified | unclassified | Coprococcus | Lachnospiraceae | Eubacteriales | Clostridia | Firmicutes |
| Eubacteriales sp. (HG3A.0548) | T90 | -0.045 | 0.011 | 0.078 | 3249 unclassified | unclassified | unclassified | unclassified | Eubacteriales | Clostridia | Firmicutes |
| Faecalibacterium prausnitzii  (HG3A.0010) | T90 | 0.045 | 0.011 | 0.078 | 3249 unclassified | Faecalibacterium  prausnitzii | Faecalibacterium | Oscillospiraceae | Eubacteriales | Clostridia | Firmicutes |
| Lachnospiraceae sp. (HG3A.0233) | T90 | -0.046 | 0.01 | 0.078 | 3249 unclassified | unclassified | unclassified | Lachnospiraceae | Eubacteriales | Clostridia | Firmicutes |
| Lachnospiraceae sp. (HG3A.0625) | T90 | 0.045 | 0.011 | 0.078 | 3249 unclassified | unclassified | unclassified | Lachnospiraceae | Eubacteriales | Clostridia | Firmicutes |
| Oscillospiraceae sp. (HG3A.0774) | T90 | -0.045 | 0.011 | 0.078 | 3249 unclassified | unclassified | unclassified | Oscillospiraceae | Eubacteriales | Clostridia | Firmicutes |
| Oxalobacter formigenes  (HG3A.0552) | T90 | -0.045 | 0.011 | 0.079 | 3249 unclassified | Oxalobacter  formigenes | Oxalobacter | Oxalobacteraceae | Burkholderiales | Betaproteobacte  ria | Proteobacteria |
| Ruminococcus sp. AF46-10NS (HG3A.0271) | T90 | 0.045 | 0.011 | 0.079 | 3249 unclassified | Ruminococcus sp. AF46-10NS | Ruminococcus | Oscillospiraceae | Eubacteriales | Clostridia | Firmicutes |
| Eubacteriales sp. (HG3A.0081) | ODI | -0.043 | 0.016 | 0.082 | 3249 unclassified | unclassified | unclassified | unclassified | Eubacteriales | Clostridia | Firmicutes |
| Ruminococcus champanellensis (HG3A.0716) | ODI | -0.043 | 0.016 | 0.082 | 3249 unclassified | Ruminococcus champanellensis | Ruminococcus | Oscillospiraceae | Eubacteriales | Clostridia | Firmicutes |
| Eubacteriales sp. (HG3A.0092) | ODI | -0.043 | 0.016 | 0.083 | 3249 unclassified | unclassified | unclassified | unclassified | Eubacteriales | Clostridia | Firmicutes |
| Oscillospiraceae sp. (HG3A.0384) | ODI | -0.043 | 0.016 | 0.083 | 3249 unclassified | unclassified | unclassified | Oscillospiraceae | Eubacteriales | Clostridia | Firmicutes |
| Alistipes sp. AF17-16 (HG3A.0150) | ODI | -0.042 | 0.018 | 0.084 | 3249 unclassified | Alistipes sp. AF17-  16 | Alistipes | Rikenellaceae | Bacteroidales | Bacteroidia | Bacteroidetes |
| Clostridia sp. (HG3A.0787) | ODI | -0.042 | 0.017 | 0.084 | 3249 unclassified | unclassified | unclassified | unclassified | unclassified | Clostridia | Firmicutes |
| Clostridia sp. (HG3A.1010) | ODI | -0.042 | 0.018 | 0.084 | 3249 unclassified | unclassified | unclassified | unclassified | unclassified | Clostridia | Firmicutes |
| Clostridia sp. (HG3A.1375) | ODI | -0.042 | 0.017 | 0.084 | 3249 unclassified | unclassified | unclassified | unclassified | unclassified | Clostridia | Firmicutes |
| Eubacteriales sp. (HG3A.0123) | ODI | 0.042 | 0.017 | 0.084 | 3249 unclassified | unclassified | unclassified | unclassified | Eubacteriales | Clostridia | Firmicutes |
| Eubacteriales sp. (HG3A.0234) | ODI | -0.042 | 0.018 | 0.084 | 3249 unclassified | unclassified | unclassified | unclassified | Eubacteriales | Clostridia | Firmicutes |
| Eubacteriales sp. (HG3A.0371) | ODI | -0.042 | 0.018 | 0.084 | 3249 unclassified | unclassified | unclassified | unclassified | Eubacteriales | Clostridia | Firmicutes |
| Eubacteriales sp. (HG3A.0565) | ODI | -0.042 | 0.017 | 0.084 | 3249 unclassified | unclassified | unclassified | unclassified | Eubacteriales | Clostridia | Firmicutes |

| Eubacteriales sp. (HG3A.0703) | ODI | -0.042 | 0.017 | 0.084 | 3249 unclassified | unclassified | unclassified | unclassified | Eubacteriales | Clostridia | Firmicutes |
| --- | --- | --- | --- | --- | --- | --- | --- | --- | --- | --- | --- |
| Eubacteriales sp. (HG3A.0870) | ODI | -0.042 | 0.018 | 0.084 | 3249 unclassified | unclassified | unclassified | unclassified | Eubacteriales | Clostridia | Firmicutes |
| Roseburia intestinalis (HG3A.0078) | ODI | 0.042 | 0.018 | 0.084 | 3249 unclassified | Roseburia  intestinalis | Roseburia | Lachnospiraceae | Eubacteriales | Clostridia | Firmicutes |
| Bacteria sp. (HG3A.0492) | AHI | -0.055 | 0.003 | 0.085 | 2909 unclassified | unclassified | unclassified | unclassified | unclassified | unclassified | unclassified |
| Clostridia sp. (HG3A.0094) | AHI | -0.057 | 0.003 | 0.085 | 2909 unclassified | unclassified | unclassified | unclassified | unclassified | Clostridia | Firmicutes |
| Clostridia sp. (HG3A.0728) | T90 | -0.045 | 0.012 | 0.085 | 3249 unclassified | unclassified | unclassified | unclassified | unclassified | Clostridia | Firmicutes |
| Clostridia sp. (HG3A.0750) | ODI | -0.042 | 0.018 | 0.085 | 3249 unclassified | unclassified | unclassified | unclassified | unclassified | Clostridia | Firmicutes |
| Clostridia sp. (HG3A.1053) | AHI | -0.056 | 0.003 | 0.085 | 2909 unclassified | unclassified | unclassified | unclassified | unclassified | Clostridia | Firmicutes |
| Eggerthellales sp. (HG3A.0177) | AHI | -0.055 | 0.004 | 0.085 | 2909 unclassified | unclassified | unclassified | unclassified | Eggerthellales | Coriobacteriia | Actinobacteria |
| Eubacteriales sp. (HG3A.0153) | AHI | -0.055 | 0.004 | 0.085 | 2909 unclassified | unclassified | unclassified | unclassified | Eubacteriales | Clostridia | Firmicutes |
| Eubacteriales sp. (HG3A.0162) | AHI | -0.056 | 0.003 | 0.085 | 2909 unclassified | unclassified | unclassified | unclassified | Eubacteriales | Clostridia | Firmicutes |
| Eubacteriales sp. (HG3A.0235) | AHI | -0.056 | 0.003 | 0.085 | 2909 unclassified | unclassified | unclassified | unclassified | Eubacteriales | Clostridia | Firmicutes |
| Eubacteriales sp. (HG3A.0284) | AHI | -0.056 | 0.003 | 0.085 | 2909 unclassified | unclassified | unclassified | unclassified | Eubacteriales | Clostridia | Firmicutes |
| Eubacteriales sp. (HG3A.0390) | ODI | -0.042 | 0.018 | 0.085 | 3249 unclassified | unclassified | unclassified | unclassified | Eubacteriales | Clostridia | Firmicutes |
| Eubacteriales sp. (HG3A.0442) | AHI | -0.055 | 0.003 | 0.085 | 2909 unclassified | unclassified | unclassified | unclassified | Eubacteriales | Clostridia | Firmicutes |
| Eubacteriales sp. (HG3A.0506) | AHI | -0.057 | 0.002 | 0.085 | 2909 unclassified | unclassified | unclassified | unclassified | Eubacteriales | Clostridia | Firmicutes |
| Eubacteriales sp. (HG3A.0964) | AHI | -0.056 | 0.003 | 0.085 | 2909 unclassified | unclassified | unclassified | unclassified | Eubacteriales | Clostridia | Firmicutes |
| Eubacteriales sp. (HG3A.1019) | AHI | -0.056 | 0.003 | 0.085 | 2909 unclassified | unclassified | unclassified | unclassified | Eubacteriales | Clostridia | Firmicutes |
| Firmicutes sp. (HG3A.0681) | AHI | -0.055 | 0.004 | 0.085 | 2909 unclassified | unclassified | unclassified | unclassified | unclassified | unclassified | Firmicutes |
| Lachnospiraceae sp. (HG3A.0233) | ODI | -0.042 | 0.018 | 0.085 | 3249 unclassified | unclassified | unclassified | Lachnospiraceae | Eubacteriales | Clostridia | Firmicutes |
| Oscillospiraceae sp. (HG3A.0072) | AHI | -0.055 | 0.003 | 0.085 | 2909 unclassified | unclassified | unclassified | Oscillospiraceae | Eubacteriales | Clostridia | Firmicutes |
| Oscillospiraceae sp. (HG3A.1270) | AHI | -0.056 | 0.003 | 0.085 | 2909 unclassified | unclassified | unclassified | Oscillospiraceae | Eubacteriales | Clostridia | Firmicutes |
| Butyricicoccus sp. OM04-18BH  (HG3A.0139) | ODI | -0.042 | 0.019 | 0.087 | 3249 unclassified | Butyricicoccus sp.  OM04-18BH | Butyricicoccus | Clostridiaceae | Eubacteriales | Clostridia | Firmicutes |
| Blautia obeum (HG3A.0009) | ODI | 0.042 | 0.019 | 0.088 | 3249 unclassified | Blautia obeum | Blautia | Lachnospiraceae | Eubacteriales | Clostridia | Firmicutes |
| Eubacteriales sp. (HG3A.0486) | ODI | -0.042 | 0.019 | 0.088 | 3249 unclassified | unclassified | unclassified | unclassified | Eubacteriales | Clostridia | Firmicutes |
| Alistipes ihumii (HG3A.0106) | ODI | -0.041 | 0.02 | 0.089 | 3249 unclassified | Alistipes ihumii | Alistipes | Rikenellaceae | Bacteroidales | Bacteroidia | Bacteroidetes |

| Clostridia sp. (HG3A.0752) | ODI | -0.041 | 0.02 | 0.089 | 3249 unclassified | unclassified | unclassified | unclassified | unclassified | Clostridia | Firmicutes |
| --- | --- | --- | --- | --- | --- | --- | --- | --- | --- | --- | --- |
| Eubacteriales sp. (HG3A.0976) | ODI | -0.041 | 0.02 | 0.089 | 3249 unclassified | unclassified | unclassified | unclassified | Eubacteriales | Clostridia | Firmicutes |
| Lachnospiraceae sp. (HG3A.0018) | AHI | 0.054 | 0.004 | 0.089 | 2909 unclassified | unclassified | unclassified | Lachnospiraceae | Eubacteriales | Clostridia | Firmicutes |
| Lachnospiraceae sp. (HG3A.0399) | ODI | -0.041 | 0.02 | 0.089 | 3249 unclassified | unclassified | unclassified | Lachnospiraceae | Eubacteriales | Clostridia | Firmicutes |
| Oscillospiraceae sp. (HG3A.0693) | ODI | -0.041 | 0.02 | 0.089 | 3249 unclassified | unclassified | unclassified | Oscillospiraceae | Eubacteriales | Clostridia | Firmicutes |
| Parolsenella catena (HG3A.0499) | ODI | 0.041 | 0.02 | 0.089 | 3249 unclassified | Parolsenella catena | Parolsenella | Atopobiaceae | Coriobacteriales | Coriobacteriia | Actinobacteria |
| Alistipes senegalensis (HG3A.0141) | ODI | -0.041 | 0.021 | 0.090 | 3249 unclassified | Alistipes  senegalensis | Alistipes | Rikenellaceae | Bacteroidales | Bacteroidia | Bacteroidetes |
| Clostridia sp. (HG3A.0011) | ODI | 0.041 | 0.021 | 0.090 | 3249 unclassified | unclassified | unclassified | unclassified | unclassified | Clostridia | Firmicutes |
| Clostridia sp. (HG3A.0463) | ODI | -0.041 | 0.021 | 0.090 | 3249 unclassified | unclassified | unclassified | unclassified | unclassified | Clostridia | Firmicutes |
| Clostridium sp. TF06-15AC (HG3A.0032) | T90 | 0.044 | 0.013 | 0.090 | 3249 unclassified | Clostridium sp. TF06-15AC | Clostridium | Clostridiaceae | Eubacteriales | Clostridia | Firmicutes |
| Coprococcus sp. (HG3A.0404) | ODI | 0.041 | 0.021 | 0.090 | 3249 unclassified | unclassified | Coprococcus | Lachnospiraceae | Eubacteriales | Clostridia | Firmicutes |
| Eubacteriales sp. (HG3A.0443) | T90 | -0.044 | 0.013 | 0.090 | 3249 unclassified | unclassified | unclassified | unclassified | Eubacteriales | Clostridia | Firmicutes |
| Eubacteriales sp. (HG3A.0474) | T90 | -0.044 | 0.013 | 0.090 | 3249 unclassified | unclassified | unclassified | unclassified | Eubacteriales | Clostridia | Firmicutes |
| Eubacteriales sp. (HG3A.0509) | ODI | -0.041 | 0.021 | 0.090 | 3249 unclassified | unclassified | unclassified | unclassified | Eubacteriales | Clostridia | Firmicutes |
| Eubacteriales sp. (HG3A.0531) | ODI | -0.041 | 0.021 | 0.090 | 3249 unclassified | unclassified | unclassified | unclassified | Eubacteriales | Clostridia | Firmicutes |
| Eubacteriales sp. (HG3A.0730) | T90 | -0.044 | 0.013 | 0.090 | 3249 unclassified | unclassified | unclassified | unclassified | Eubacteriales | Clostridia | Firmicutes |
| Eubacteriales sp. (HG3A.0790) | T90 | -0.044 | 0.013 | 0.090 | 3249 unclassified | unclassified | unclassified | unclassified | Eubacteriales | Clostridia | Firmicutes |
| Faecalibacterium prausnitzii (HG3A.0025) | ODI | 0.041 | 0.021 | 0.090 | 3249 unclassified | Faecalibacterium prausnitzii | Faecalibacterium | Oscillospiraceae | Eubacteriales | Clostridia | Firmicutes |
| Lachnospiraceae sp. (HG3A.0257) | ODI | -0.041 | 0.021 | 0.090 | 3249 unclassified | unclassified | unclassified | Lachnospiraceae | Eubacteriales | Clostridia | Firmicutes |
| Victivallis vadensis (HG3A.0689) | T90 | -0.044 | 0.013 | 0.090 | 3249 unclassified | Victivallis vadensis | Victivallis | Victivallaceae | Victivallales | Lentisphaeria | Lentisphaerae |
| Eubacteriales sp. (HG3A.0311) | AHI | -0.054 | 0.004 | 0.091 | 2909 unclassified | unclassified | unclassified | unclassified | Eubacteriales | Clostridia | Firmicutes |
| Butyricicoccus sp. (HG3A.0008) | T90 | 0.044 | 0.014 | 0.092 | 3249 unclassified | unclassified | Butyricicoccus | Clostridiaceae | Eubacteriales | Clostridia | Firmicutes |
| [Ruminococcus] gnavus  (HG3A.0239) | AHI | 0.053 | 0.004 | 0.093 | 2909 unclassified | [Ruminococcus]  gnavus | Mediterraneibacte  r | Lachnospiraceae | Eubacteriales | Clostridia | Firmicutes |
| Eubacteriales sp. (HG3A.0421) | AHI | -0.053 | 0.004 | 0.093 | 2909 unclassified | unclassified | unclassified | unclassified | Eubacteriales | Clostridia | Firmicutes |
| Pseudoflavonifractor sp. An184  (HG3A.0253) | ODI | -0.041 | 0.022 | 0.093 | 3249 unclassified | Pseudoflavonifracto  r sp. An184 | Pseudoflavonifrac  tor | Oscillospiraceae | Eubacteriales | Clostridia | Firmicutes |

| Alistipes sp. An66 (HG3A.1535) | ODI | -0.04 | 0.023 | 0.094 | 3249 unclassified | Alistipes sp. An66 | Alistipes | Rikenellaceae | Bacteroidales | Bacteroidia | Bacteroidetes |
| --- | --- | --- | --- | --- | --- | --- | --- | --- | --- | --- | --- |
| Clostridia sp. (HG3A.0368) | ODI | -0.04 | 0.023 | 0.094 | 3249 unclassified | unclassified | unclassified | unclassified | unclassified | Clostridia | Firmicutes |
| Clostridium sp. SN20 (HG3A.0603) | ODI | 0.04 | 0.023 | 0.094 | 3249 unclassified | Clostridium sp. SN20 | Clostridium | Clostridiaceae | Eubacteriales | Clostridia | Firmicutes |
| Eubacteriales sp. (HG3A.0161) | ODI | -0.04 | 0.023 | 0.094 | 3249 unclassified | unclassified | unclassified | unclassified | Eubacteriales | Clostridia | Firmicutes |
| Eubacteriales sp. (HG3A.0264) | ODI | -0.04 | 0.023 | 0.094 | 3249 unclassified | unclassified | unclassified | unclassified | Eubacteriales | Clostridia | Firmicutes |
| Eubacteriales sp. (HG3A.0482) | ODI | -0.04 | 0.023 | 0.094 | 3249 unclassified | unclassified | unclassified | unclassified | Eubacteriales | Clostridia | Firmicutes |
| Actinomycetaceae sp. (HG3A.1068) | ODI | -0.04 | 0.024 | 0.097 | 3249 unclassified | unclassified | unclassified | Actinomycetaceae | Actinomycetales | Actinomycetia | Actinobacteria |
| Eubacteriales sp. (HG3A.0626) | ODI | -0.04 | 0.024 | 0.097 | 3249 unclassified | unclassified | unclassified | unclassified | Eubacteriales | Clostridia | Firmicutes |
| Coprobacillus sp. (HG3A.0022) | T90 | 0.043 | 0.015 | 0.098 | 3249 unclassified | unclassified | Coprobacillus | Coprobacillaceae | Erysipelotrichales | Erysipelotrichia | Firmicutes |
| Eubacteriales sp. (HG3A.0093) | T90 | -0.043 | 0.015 | 0.098 | 3249 unclassified | unclassified | unclassified | unclassified | Eubacteriales | Clostridia | Firmicutes |
| Eubacteriales sp. (HG3A.0983) | T90 | -0.043 | 0.015 | 0.098 | 3249 unclassified | unclassified | unclassified | unclassified | Eubacteriales | Clostridia | Firmicutes |
| Oscillospiraceae sp. (HG3A.0429) | T90 | -0.043 | 0.015 | 0.098 | 3249 unclassified | unclassified | unclassified | Oscillospiraceae | Eubacteriales | Clostridia | Firmicutes |
| Clostridia sp. (HG3A.1217) | ODI | -0.04 | 0.025 | 0.099 | 3249 unclassified | unclassified | unclassified | unclassified | unclassified | Clostridia | Firmicutes |
| Collinsella sp. WCA1-178-WT-3 |  |  |  |  |  | Collinsella sp.  WCA1-178-WT-3 |  |  |  |  |  |
| (M2) (HG3A.1245) | T90 | 0.043 | 0.016 | 0.099 | 3249 unclassified | (M2) | Collinsella | Coriobacteriaceae | Coriobacteriales | Coriobacteriia | Actinobacteria |
| Eubacteriales sp. (HG3A.0864) | AHI | -0.053 | 0.005 | 0.099 | 2909 unclassified | unclassified | unclassified | unclassified | Eubacteriales | Clostridia | Firmicutes |
| Eubacterium sp. AF22-8LB  (HG3A.0838) | ODI | 0.04 | 0.025 | 0.099 | 3249 unclassified | Eubacterium sp.  AF22-8LB | Eubacterium | Eubacteriaceae | Eubacteriales | Clostridia | Firmicutes |
| Eubacteriales sp. (HG3A.0100) | AHI | -0.053 | 0.005 | 0.100 | 2909 unclassified | unclassified | unclassified | unclassified | Eubacteriales | Clostridia | Firmicutes |
| Eubacteriales sp. (HG3A.0546) | ODI | -0.04 | 0.026 | 0.101 | 3249 unclassified | unclassified | unclassified | unclassified | Eubacteriales | Clostridia | Firmicutes |
| Eubacteriales sp. (HG3A.0592) | T90 | -0.043 | 0.016 | 0.101 | 3249 unclassified | unclassified | unclassified | unclassified | Eubacteriales | Clostridia | Firmicutes |
| Bacteria sp. (HG3A.0459) | ODI | -0.04 | 0.026 | 0.102 | 3249 unclassified | unclassified | unclassified | unclassified | unclassified | unclassified | unclassified |
| Eubacteriales sp. (HG3A.0856) | ODI | -0.04 | 0.026 | 0.102 | 3249 unclassified | unclassified | unclassified | unclassified | Eubacteriales | Clostridia | Firmicutes |
| Eubacteriales sp. (HG3A.0408) | T90 | -0.043 | 0.016 | 0.103 | 3249 unclassified | unclassified | unclassified | unclassified | Eubacteriales | Clostridia | Firmicutes |
| Oscillospiraceae sp. (HG3A.0223) | T90 | -0.042 | 0.017 | 0.104 | 3249 unclassified | unclassified | unclassified | Oscillospiraceae | Eubacteriales | Clostridia | Firmicutes |
| Eubacteriales sp. (HG3A.0696) | ODI | -0.039 | 0.027 | 0.105 | 3249 unclassified | unclassified | unclassified | unclassified | Eubacteriales | Clostridia | Firmicutes |
| Eubacteriales sp. (HG3A.0908) | ODI | -0.039 | 0.027 | 0.105 | 3249 unclassified | unclassified | unclassified | unclassified | Eubacteriales | Clostridia | Firmicutes |
| Eubacteriales sp. (HG3A.1026) | T90 | -0.042 | 0.017 | 0.105 | 3249 unclassified | unclassified | unclassified | unclassified | Eubacteriales | Clostridia | Firmicutes |
| Lactobacillus gasseri (HG3A.0884) | ODI | 0.039 | 0.027 | 0.105 | 3249 unclassified | Lactobacillus gasseri | Lactobacillus | Lactobacillaceae | Lactobacillales | Bacilli | Firmicutes |

| Bacteria sp. (HG3A.0483) | AHI | -0.051 | 0.006 | 0.106 | 2909 unclassified | unclassified | unclassified | unclassified | unclassified | unclassified | unclassified |
| --- | --- | --- | --- | --- | --- | --- | --- | --- | --- | --- | --- |
| Clostridia sp. (HG3A.0599) | AHI | -0.051 | 0.006 | 0.106 | 2909 unclassified | unclassified | unclassified | unclassified | unclassified | Clostridia | Firmicutes |
| Clostridia sp. (HG3A.0660) | AHI | -0.052 | 0.006 | 0.106 | 2909 unclassified | unclassified | unclassified | unclassified | unclassified | Clostridia | Firmicutes |
| Clostridia sp. (HG3A.0750) | AHI | -0.052 | 0.006 | 0.106 | 2909 unclassified | unclassified | unclassified | unclassified | unclassified | Clostridia | Firmicutes |
| Clostridium sp. TF06-15AC  (HG3A.0032) | AHI | 0.051 | 0.006 | 0.106 | 2909 unclassified | Clostridium sp.  TF06-15AC | Clostridium | Clostridiaceae | Eubacteriales | Clostridia | Firmicutes |
| Collinsella aerofaciens (HG3A.0019) | AHI | 0.051 | 0.006 | 0.106 | 2909 unclassified | Collinsella aerofaciens | Collinsella | Coriobacteriaceae | Coriobacteriales | Coriobacteriia | Actinobacteria |
| Eubacteriales sp. (HG3A.0744) | T90 | -0.042 | 0.018 | 0.106 | 3249 unclassified | unclassified | unclassified | unclassified | Eubacteriales | Clostridia | Firmicutes |
| Eubacteriales sp. (HG3A.0854) | T90 | 0.042 | 0.018 | 0.106 | 3249 unclassified | unclassified | unclassified | unclassified | Eubacteriales | Clostridia | Firmicutes |
| Eubacterium sp. (HG3A.0214) | AHI | 0.052 | 0.006 | 0.106 | 2909 unclassified | unclassified | Eubacterium | Eubacteriaceae | Eubacteriales | Clostridia | Firmicutes |
| Firmicutes sp. (HG3A.0397) | T90 | -0.042 | 0.018 | 0.106 | 3249 unclassified | unclassified | unclassified | unclassified | unclassified | unclassified | Firmicutes |
| Parolsenella catena (HG3A.0499) | T90 | 0.042 | 0.018 | 0.106 | 3249 unclassified | Parolsenella catena | Parolsenella | Atopobiaceae | Coriobacteriales | Coriobacteriia | Actinobacteria |
| Parvimonas micra (HG3A.1231) | T90 | 0.042 | 0.017 | 0.106 | 3249 unclassified | Parvimonas micra | Parvimonas | Peptoniphilaceae | Tissierellales | Tissierellia | Firmicutes |
| Clostridiaceae sp. (HG3A.0431) | ODI | 0.039 | 0.028 | 0.107 | 3249 unclassified | unclassified | unclassified | Clostridiaceae | Eubacteriales | Clostridia | Firmicutes |
| Methanobrevibacter smithii |  |  |  |  |  | Methanobrevibacte | Methanobrevibact | Methanobacteriace |  |  |  |
| (HG3A.0152) ODI -0.039 0.028 0.107 3249 unclassified r smithii er ae Methanobacteriales Methanobacteria Euryarchaeota | | | | | | | | | | | |
| Parvimonas micra (HG3A.1231) | ODI | 0.039 | 0.028 | 0.107 | 3249 unclassified | Parvimonas micra | Parvimonas | Peptoniphilaceae | Tissierellales | Tissierellia | Firmicutes |
| Eubacteriales sp. (HG3A.0381) | ODI | -0.039 | 0.029 | 0.108 | 3249 unclassified | unclassified | unclassified | unclassified | Eubacteriales | Clostridia | Firmicutes |
| Eubacteriales sp. (HG3A.1239) | ODI | -0.039 | 0.029 | 0.108 | 3249 unclassified | unclassified | unclassified | unclassified | Eubacteriales | Clostridia | Firmicutes |
| Sellimonas intestinalis (HG3A.0417) | T90 | 0.042 | 0.018 | 0.108 | 3249 unclassified | Sellimonas intestinalis | Sellimonas | Lachnospiraceae | Eubacteriales | Clostridia | Firmicutes |
| Amedibacillus dolichus (HG3A.0798) | ODI | 0.039 | 0.029 | 0.109 | 3249 unclassified | Amedibacillus  dolichus | Amedibacillus | Erysipelotrichacea  e | Erysipelotrichales | Erysipelotrichia | Firmicutes |
| Clostridia sp. (HG3A.1148) | AHI | -0.051 | 0.007 | 0.109 | 2909 unclassified | unclassified | unclassified | unclassified | unclassified | Clostridia | Firmicutes |
| Anaerobutyricum hallii (HG3A.0112) | ODI | 0.039 | 0.03 | 0.110 | 3249 unclassified | Anaerobutyricum  hallii | Anaerobutyricum | Lachnospiraceae | Eubacteriales | Clostridia | Firmicutes |
| Firmicutes sp. (HG3A.0650) | ODI | -0.039 | 0.03 | 0.111 | 3249 unclassified | unclassified | unclassified | unclassified | unclassified | unclassified | Firmicutes |
| Eubacteriales sp. (HG3A.0083) | T90 | -0.041 | 0.02 | 0.112 | 3249 unclassified | unclassified | unclassified | unclassified | Eubacteriales | Clostridia | Firmicutes |
| Eubacteriales sp. (HG3A.0316) | T90 | -0.042 | 0.019 | 0.112 | 3249 unclassified | unclassified | unclassified | unclassified | Eubacteriales | Clostridia | Firmicutes |
| Eubacteriales sp. (HG3A.0409) | T90 | -0.042 | 0.019 | 0.112 | 3249 unclassified | unclassified | unclassified | unclassified | Eubacteriales | Clostridia | Firmicutes |
| Eubacteriales sp. (HG3A.0545) | T90 | -0.042 | 0.019 | 0.112 | 3249 unclassified | unclassified | unclassified | unclassified | Eubacteriales | Clostridia | Firmicutes |

| Acidaminococcus intestini  (HG3A.0407) | ODI | 0.038 | 0.031 | 0.113 | 3249 unclassified | Acidaminococcus  intestini | Acidaminococcus | Acidaminococcace  ae | Acidaminococcales | Negativicutes | Firmicutes |
| --- | --- | --- | --- | --- | --- | --- | --- | --- | --- | --- | --- |
| Eubacteriales sp. (HG3A.0600) | ODI | -0.038 | 0.031 | 0.113 | 3249 unclassified | unclassified | unclassified | unclassified | Eubacteriales | Clostridia | Firmicutes |
| Eubacteriales sp. (HG3A.0730) | ODI | -0.038 | 0.031 | 0.113 | 3249 unclassified | unclassified | unclassified | unclassified | Eubacteriales | Clostridia | Firmicutes |
| Eubacteriales sp. (HG3A.1129) | ODI | -0.038 | 0.031 | 0.113 | 3249 unclassified | unclassified | unclassified | unclassified | Eubacteriales | Clostridia | Firmicutes |
| Firmicutes sp. (HG3A.0454) | ODI | -0.038 | 0.031 | 0.113 | 3249 unclassified | unclassified | unclassified | unclassified | unclassified | unclassified | Firmicutes |
| Butyricicoccus sp. (HG3A.0008) | ODI | 0.038 | 0.032 | 0.114 | 3249 unclassified | unclassified | Butyricicoccus | Clostridiaceae | Eubacteriales | Clostridia | Firmicutes |
| Coprococcus comes (HG3A.0016) | AHI | 0.05 | 0.008 | 0.114 | 2909 unclassified | Coprococcus  comes | Coprococcus | Lachnospiraceae | Eubacteriales | Clostridia | Firmicutes |
| Eubacteriales sp. (HG3A.0153) | T90 | -0.041 | 0.02 | 0.114 | 3249 unclassified | unclassified | unclassified | unclassified | Eubacteriales | Clostridia | Firmicutes |
| Eubacteriales sp. (HG3A.0178) | ODI | -0.038 | 0.032 | 0.114 | 3249 unclassified | unclassified | unclassified | unclassified | Eubacteriales | Clostridia | Firmicutes |
| Eubacteriales sp. (HG3A.0193) | AHI | -0.05 | 0.008 | 0.114 | 2909 unclassified | unclassified | unclassified | unclassified | Eubacteriales | Clostridia | Firmicutes |
| Eubacteriales sp. (HG3A.0226) | AHI | -0.05 | 0.007 | 0.114 | 2909 unclassified | unclassified | unclassified | unclassified | Eubacteriales | Clostridia | Firmicutes |
| Eubacteriales sp. (HG3A.0249) | AHI | -0.05 | 0.008 | 0.114 | 2909 unclassified | unclassified | unclassified | unclassified | Eubacteriales | Clostridia | Firmicutes |
| Eubacteriales sp. (HG3A.0309) | AHI | -0.05 | 0.007 | 0.114 | 2909 unclassified | unclassified | unclassified | unclassified | Eubacteriales | Clostridia | Firmicutes |
| Eubacteriales sp. (HG3A.0352) | T90 | -0.041 | 0.02 | 0.114 | 3249 unclassified | unclassified | unclassified | unclassified | Eubacteriales | Clostridia | Firmicutes |
| Eubacteriales sp. (HG3A.0390) | T90 | -0.041 | 0.02 | 0.114 | 3249 unclassified | unclassified | unclassified | unclassified | Eubacteriales | Clostridia | Firmicutes |
| Eubacteriales sp. (HG3A.0468) | ODI | -0.038 | 0.032 | 0.114 | 3249 unclassified | unclassified | unclassified | unclassified | Eubacteriales | Clostridia | Firmicutes |
| Eubacteriales sp. (HG3A.0857) | ODI | -0.038 | 0.032 | 0.114 | 3249 unclassified | unclassified | unclassified | unclassified | Eubacteriales | Clostridia | Firmicutes |
| Firmicutes sp. (HG3A.0501) | AHI | -0.05 | 0.008 | 0.114 | 2909 unclassified | unclassified | unclassified | unclassified | unclassified | unclassified | Firmicutes |
| Oscillospiraceae sp. (HG3A.0382) | ODI | -0.038 | 0.032 | 0.114 | 3249 unclassified | unclassified | unclassified | Oscillospiraceae | Eubacteriales | Clostridia | Firmicutes |
| Clostridia sp. (HG3A.1504) | ODI | -0.038 | 0.033 | 0.115 | 3249 unclassified | unclassified | unclassified | unclassified | unclassified | Clostridia | Firmicutes |
| Eubacteriales sp. (HG3A.0668) | ODI | -0.038 | 0.033 | 0.115 | 3249 unclassified | unclassified | unclassified | unclassified | Eubacteriales | Clostridia | Firmicutes |
| Gemmiger formicilis (HG3A.0027) | ODI | 0.038 | 0.033 | 0.115 | 3249 unclassified | Gemmiger formicilis | Gemmiger | unclassified | Eubacteriales | Clostridia | Firmicutes |
| Lachnospiraceae sp. (HG3A.1190) | ODI | -0.038 | 0.033 | 0.115 | 3249 unclassified | unclassified | unclassified | Lachnospiraceae | Eubacteriales | Clostridia | Firmicutes |
| [Ruminococcus] torques (HG3A.0034) | AHI | 0.05 | 0.008 | 0.116 | 2909 unclassified | [Ruminococcus] torques | Mediterraneibacte r | Lachnospiraceae | Eubacteriales | Clostridia | Firmicutes |
| Alistipes communis (HG3A.0064) | AHI | -0.05 | 0.008 | 0.116 | 2909 unclassified | Alistipes  communis | Alistipes | Rikenellaceae | Bacteroidales | Bacteroidia | Bacteroidetes |
| Amedibacillus dolichus (HG3A.0798) | AHI | 0.05 | 0.008 | 0.116 | 2909 unclassified | Amedibacillus dolichus | Amedibacillus | Erysipelotrichacea e | Erysipelotrichales | Erysipelotrichia | Firmicutes |
| Clostridium sp. AT4 (HG3A.0347) | ODI | 0.038 | 0.034 | 0.117 | 3249 unclassified | Clostridium sp.  AT4 | Clostridium | Clostridiaceae | Eubacteriales | Clostridia | Firmicutes |
| Eubacteriales sp. (HG3A.0652) | T90 | -0.041 | 0.021 | 0.117 | 3249 unclassified | unclassified | unclassified | unclassified | Eubacteriales | Clostridia | Firmicutes |

| Clostridium sp. SN20 (HG3A.0603) | T90 | 0.041 | 0.022 | 0.118 | 3249 unclassified | Clostridium sp.  SN20 | Clostridium | Clostridiaceae | Eubacteriales | Clostridia | Firmicutes |
| --- | --- | --- | --- | --- | --- | --- | --- | --- | --- | --- | --- |
| Eubacterium ramulus (HG3A.0068) | T90 | 0.041 | 0.022 | 0.118 | 3249 unclassified | Eubacterium ramulus | Eubacterium | Eubacteriaceae | Eubacteriales | Clostridia | Firmicutes |
| Clostridia sp. (HG3A.0724) | AHI | -0.049 | 0.009 | 0.119 | 2909 unclassified | unclassified | unclassified | unclassified | unclassified | Clostridia | Firmicutes |
| Eubacteriales sp. (HG3A.0149) | AHI | -0.049 | 0.009 | 0.119 | 2909 unclassified | unclassified | unclassified | unclassified | Eubacteriales | Clostridia | Firmicutes |
| Clostridia sp. (HG3A.1504) | AHI | -0.049 | 0.009 | 0.120 | 2909 unclassified | unclassified | unclassified | unclassified | unclassified | Clostridia | Firmicutes |
| Eubacteriales sp. (HG3A.0331) | T90 | -0.041 | 0.022 | 0.120 | 3249 unclassified | unclassified | unclassified | unclassified | Eubacteriales | Clostridia | Firmicutes |
| Mesosutterella multiformis  (HG3A.0520) | AHI | -0.049 | 0.009 | 0.120 | 2909 unclassified | Mesosutterella  multiformis | Mesosutterella | Sutterellaceae | Burkholderiales | Betaproteobacte  ria | Proteobacteria |
| Bacteroidales sp. (HG3A.1446) | T90 | -0.041 | 0.023 | 0.121 | 3249 unclassified | unclassified | unclassified | unclassified | Bacteroidales | Bacteroidia | Bacteroidetes |
| Clostridia sp. (HG3A.0385) | ODI | -0.037 | 0.035 | 0.121 | 3249 unclassified | unclassified | unclassified | unclassified | unclassified | Clostridia | Firmicutes |
| Oscillospiraceae sp. (HG3A.0616) | T90 | -0.041 | 0.023 | 0.121 | 3249 unclassified | unclassified | unclassified | Oscillospiraceae | Eubacteriales | Clostridia | Firmicutes |
| Firmicutes sp. (HG3A.0454) | T90 | -0.04 | 0.023 | 0.122 | 3249 unclassified | unclassified | unclassified | unclassified | unclassified | unclassified | Firmicutes |
| Clostridia sp. (HG3A.0479) | ODI | -0.037 | 0.036 | 0.123 | 3249 unclassified | unclassified | unclassified | unclassified | unclassified | Clostridia | Firmicutes |
| Clostridia sp. (HG3A.0741) | AHI | -0.049 | 0.01 | 0.123 | 2909 unclassified | unclassified | unclassified | unclassified | unclassified | Clostridia | Firmicutes |
| Eubacteriales sp. (HG3A.0666) | T90 | -0.04 | 0.023 | 0.123 | 3249 unclassified | unclassified | unclassified | unclassified | Eubacteriales | Clostridia | Firmicutes |
| Eubacteriales sp. (HG3A.0711) | T90 | -0.04 | 0.023 | 0.123 | 3249 unclassified | unclassified | unclassified | unclassified | Eubacteriales | Clostridia | Firmicutes |
| Eubacteriales sp. (HG3A.0580) | ODI | -0.037 | 0.037 | 0.125 | 3249 unclassified | unclassified | unclassified | unclassified | Eubacteriales | Clostridia | Firmicutes |
| Coprococcus eutactus (HG3A.0155) | T90 | -0.04 | 0.024 | 0.126 | 3249 unclassified | Coprococcus  eutactus | Coprococcus | Lachnospiraceae | Eubacteriales | Clostridia | Firmicutes |
| Eubacteriales sp. (HG3A.1377) | T90 | -0.04 | 0.024 | 0.126 | 3249 unclassified | unclassified | unclassified | unclassified | Eubacteriales | Clostridia | Firmicutes |
| Coprobacillus sp. (HG3A.0022) | AHI | 0.048 | 0.01 | 0.128 | 2909 unclassified | unclassified | Coprobacillus | Coprobacillaceae | Erysipelotrichales | Erysipelotrichia | Firmicutes |
| Eubacteriales sp. (HG3A.0081) | AHI | -0.048 | 0.011 | 0.128 | 2909 unclassified | unclassified | unclassified | unclassified | Eubacteriales | Clostridia | Firmicutes |
| Eubacteriales sp. (HG3A.0118) | AHI | -0.048 | 0.011 | 0.128 | 2909 unclassified | unclassified | unclassified | unclassified | Eubacteriales | Clostridia | Firmicutes |
| Eubacteriales sp. (HG3A.0250) | AHI | -0.048 | 0.01 | 0.128 | 2909 unclassified | unclassified | unclassified | unclassified | Eubacteriales | Clostridia | Firmicutes |
| Eubacteriales sp. (HG3A.0289) | ODI | -0.037 | 0.038 | 0.128 | 3249 unclassified | unclassified | unclassified | unclassified | Eubacteriales | Clostridia | Firmicutes |
| Eubacteriales sp. (HG3A.0718) | AHI | -0.048 | 0.011 | 0.128 | 2909 unclassified | unclassified | unclassified | unclassified | Eubacteriales | Clostridia | Firmicutes |
| Firmicutes sp. (HG3A.0923) | ODI | -0.037 | 0.038 | 0.128 | 3249 unclassified | unclassified | unclassified | unclassified | unclassified | unclassified | Firmicutes |
| Roseburia sp. AM59-24XD (HG3A.0391) | AHI | -0.048 | 0.011 | 0.128 | 2909 unclassified | Roseburia sp. AM59-24XD | Roseburia | Lachnospiraceae | Eubacteriales | Clostridia | Firmicutes |
| Bacteroides nordii (HG3A.0290) | ODI | -0.037 | 0.038 | 0.129 | 3249 unclassified | Bacteroides nordii | Bacteroides | Bacteroidaceae | Bacteroidales | Bacteroidia | Bacteroidetes |

| Clostridia sp. (HG3A.0767) | T90 | -0.04 | 0.025 | 0.129 | 3249 unclassified | unclassified | unclassified | unclassified | unclassified | Clostridia | Firmicutes |
| --- | --- | --- | --- | --- | --- | --- | --- | --- | --- | --- | --- |
| Eubacteriales sp. (HG3A.0621) | T90 | -0.04 | 0.025 | 0.129 | 3249 unclassified | unclassified | unclassified | unclassified | Eubacteriales | Clostridia | Firmicutes |
| Eubacteriales sp. (HG3A.0887) | ODI | -0.037 | 0.038 | 0.129 | 3249 unclassified | unclassified | unclassified | unclassified | Eubacteriales | Clostridia | Firmicutes |
| Firmicutes sp. (HG3A.1075) | T90 | -0.04 | 0.026 | 0.129 | 3249 unclassified | unclassified | unclassified | unclassified | unclassified | unclassified | Firmicutes |
| Parolsenella catena (HG3A.0499) | AHI | 0.048 | 0.011 | 0.129 | 2909 unclassified | Parolsenella catena | Parolsenella | Atopobiaceae | Coriobacteriales | Coriobacteriia | Actinobacteria |
| Roseburia intestinalis (HG3A.0078) | T90 | 0.04 | 0.025 | 0.129 | 3249 unclassified | Roseburia  intestinalis | Roseburia | Lachnospiraceae | Eubacteriales | Clostridia | Firmicutes |
| Eubacteriales sp. (HG3A.0426) | ODI | -0.037 | 0.039 | 0.130 | 3249 unclassified | unclassified | unclassified | unclassified | Eubacteriales | Clostridia | Firmicutes |
| Eubacteriales sp. (HG3A.0715) | ODI | -0.037 | 0.039 | 0.130 | 3249 unclassified | unclassified | unclassified | unclassified | Eubacteriales | Clostridia | Firmicutes |
| Eubacteriales sp. (HG3A.0758) | T90 | -0.04 | 0.026 | 0.131 | 3249 unclassified | unclassified | unclassified | unclassified | Eubacteriales | Clostridia | Firmicutes |
| Eubacteriales sp. (HG3A.1051) | T90 | -0.039 | 0.026 | 0.131 | 3249 unclassified | unclassified | unclassified | unclassified | Eubacteriales | Clostridia | Firmicutes |
| Eubacteriales sp. (HG3A.1219) | T90 | -0.039 | 0.027 | 0.131 | 3249 unclassified | unclassified | unclassified | unclassified | Eubacteriales | Clostridia | Firmicutes |
| Clostridia sp. (HG3A.0879) | T90 | -0.039 | 0.027 | 0.132 | 3249 unclassified | unclassified | unclassified | unclassified | unclassified | Clostridia | Firmicutes |
| Clostridia sp. (HG3A.1076) | ODI | -0.037 | 0.04 | 0.132 | 3249 unclassified | unclassified | unclassified | unclassified | unclassified | Clostridia | Firmicutes |
| Eubacteriales sp. (HG3A.0418) | T90 | -0.039 | 0.027 | 0.132 | 3249 unclassified | unclassified | unclassified | unclassified | Eubacteriales | Clostridia | Firmicutes |
| Eubacteriales sp. (HG3A.0829) | T90 | -0.039 | 0.027 | 0.132 | 3249 unclassified | unclassified | unclassified | unclassified | Eubacteriales | Clostridia | Firmicutes |
| Lachnospiraceae sp. (HG3A.1190) | AHI | -0.047 | 0.012 | 0.133 | 2909 unclassified | unclassified | unclassified | Lachnospiraceae | Eubacteriales | Clostridia | Firmicutes |
| Anaerostipes caccae (HG3A.0747) | ODI | 0.036 | 0.041 | 0.134 | 3249 unclassified | Anaerostipes caccae | Anaerostipes | Lachnospiraceae | Eubacteriales | Clostridia | Firmicutes |
| Eubacteriales sp. (HG3A.0328) | ODI | -0.036 | 0.041 | 0.134 | 3249 unclassified | unclassified | unclassified | unclassified | Eubacteriales | Clostridia | Firmicutes |
| Firmicutes sp. (HG3A.0769) | ODI | -0.036 | 0.041 | 0.134 | 3249 unclassified | unclassified | unclassified | unclassified | unclassified | unclassified | Firmicutes |
| Atopobiaceae sp. (HG3A.0937) | T90 | 0.039 | 0.029 | 0.135 | 3249 unclassified | unclassified | unclassified | Atopobiaceae | Coriobacteriales | Coriobacteriia | Actinobacteria |
| Clostridia sp. (HG3A.0368) | T90 | -0.039 | 0.029 | 0.135 | 3249 unclassified | unclassified | unclassified | unclassified | unclassified | Clostridia | Firmicutes |
| Eubacteriales sp. (HG3A.0120) | T90 | -0.039 | 0.028 | 0.135 | 3249 unclassified | unclassified | unclassified | unclassified | Eubacteriales | Clostridia | Firmicutes |
| Eubacteriales sp. (HG3A.0573) | AHI | -0.047 | 0.012 | 0.135 | 2909 unclassified | unclassified | unclassified | unclassified | Eubacteriales | Clostridia | Firmicutes |
| Eubacteriales sp. (HG3A.0626) | T90 | -0.039 | 0.029 | 0.135 | 3249 unclassified | unclassified | unclassified | unclassified | Eubacteriales | Clostridia | Firmicutes |
| Intestinimonas massiliensis (HG3A.0198) | T90 | -0.039 | 0.028 | 0.135 | 3249 unclassified | Intestinimonas massiliensis | Intestinimonas | unclassified | Eubacteriales | Clostridia | Firmicutes |
| Eubacteriales sp. (HG3A.1129) | T90 | -0.039 | 0.029 | 0.136 | 3249 unclassified | unclassified | unclassified | unclassified | Eubacteriales | Clostridia | Firmicutes |
| Eubacterium sp. AF16-48 (HG3A.0219) | T90 | -0.039 | 0.029 | 0.136 | 3249 unclassified | Eubacterium sp. AF16-48 | Eubacterium | Eubacteriaceae | Eubacteriales | Clostridia | Firmicutes |
| Eubacteriales sp. (HG3A.0794) | ODI | -0.036 | 0.042 | 0.137 | 3249 unclassified | unclassified | unclassified | unclassified | Eubacteriales | Clostridia | Firmicutes |
| Eubacteriales sp. (HG3A.0854) | ODI | 0.036 | 0.043 | 0.138 | 3249 unclassified | unclassified | unclassified | unclassified | Eubacteriales | Clostridia | Firmicutes |
| Firmicutes sp. (HG3A.0596) | ODI | -0.036 | 0.043 | 0.138 | 3249 unclassified | unclassified | unclassified | unclassified | unclassified | unclassified | Firmicutes |

| Firmicutes sp. (HG3A.1054) | T90 | -0.039 | 0.03 | 0.138 | 3249 unclassified | unclassified | unclassified | unclassified | unclassified | unclassified | Firmicutes |
| --- | --- | --- | --- | --- | --- | --- | --- | --- | --- | --- | --- |
| Pediococcus acidilactici  (HG3A.1468) | ODI | 0.036 | 0.043 | 0.138 | 3249 unclassified | Pediococcus  acidilactici | Pediococcus | Lactobacillaceae | Lactobacillales | Bacilli | Firmicutes |
| Eubacteriales sp. (HG3A.0092) | T90 | -0.038 | 0.031 | 0.139 | 3249 unclassified | unclassified | unclassified | unclassified | Eubacteriales | Clostridia | Firmicutes |
| Eubacteriales sp. (HG3A.0249) | T90 | -0.038 | 0.031 | 0.139 | 3249 unclassified | unclassified | unclassified | unclassified | Eubacteriales | Clostridia | Firmicutes |
| Eubacteriales sp. (HG3A.0511) | T90 | -0.038 | 0.031 | 0.139 | 3249 unclassified | unclassified | unclassified | unclassified | Eubacteriales | Clostridia | Firmicutes |
| Eubacteriales sp. (HG3A.1294) | T90 | -0.038 | 0.031 | 0.139 | 3249 unclassified | unclassified | unclassified | unclassified | Eubacteriales | Clostridia | Firmicutes |
| Oscillibacter sp. (HG3A.0245) | T90 | -0.038 | 0.031 | 0.139 | 3249 unclassified | unclassified | Oscillibacter | Oscillospiraceae | Eubacteriales | Clostridia | Firmicutes |
| Gemella morbillorum (HG3A.1782) | ODI | 0.036 | 0.044 | 0.141 | 3249 unclassified | Gemella  morbillorum | Gemella | unclassified | Bacillales | Bacilli | Firmicutes |
| Bacteroidales sp. (HG3A.0894) | ODI | -0.036 | 0.044 | 0.142 | 3249 unclassified | unclassified | unclassified | unclassified | Bacteroidales | Bacteroidia | Bacteroidetes |
| Mediterraneibacter glycyrrhizinilyticus (HG3A.0314) | AHI | 0.047 | 0.013 | 0.143 | 2909 unclassified | Mediterraneibacter glycyrrhizinilyticus | Mediterraneibacte r | Lachnospiraceae | Eubacteriales | Clostridia | Firmicutes |
| Oscillospiraceae sp. (HG3A.0210) | ODI | -0.036 | 0.045 | 0.143 | 3249 unclassified | unclassified | unclassified | Oscillospiraceae | Eubacteriales | Clostridia | Firmicutes |
| Eubacteriales sp. (HG3A.0367) | T90 | -0.038 | 0.032 | 0.144 | 3249 unclassified | unclassified | unclassified | unclassified | Eubacteriales | Clostridia | Firmicutes |
| Eubacteriales sp. (HG3A.1377) | AHI | -0.046 | 0.014 | 0.144 | 2909 unclassified | unclassified | unclassified | unclassified | Eubacteriales | Clostridia | Firmicutes |
| Intestinimonas massiliensis  (HG3A.0198) | AHI | -0.046 | 0.014 | 0.144 | 2909 unclassified | Intestinimonas  massiliensis | Intestinimonas | unclassified | Eubacteriales | Clostridia | Firmicutes |
| Lactobacillus gasseri (HG3A.0884) | AHI | 0.046 | 0.014 | 0.144 | 2909 unclassified | Lactobacillus gasseri | Lactobacillus | Lactobacillaceae | Lactobacillales | Bacilli | Firmicutes |
| Oscillospiraceae sp. (HG3A.0437) | AHI | -0.046 | 0.014 | 0.144 | 2909 unclassified | unclassified | unclassified | Oscillospiraceae | Eubacteriales | Clostridia | Firmicutes |
| Roseburia inulinivorans (HG3A.0036) | AHI | 0.046 | 0.014 | 0.144 | 2909 unclassified | Roseburia inulinivorans | Roseburia | Lachnospiraceae | Eubacteriales | Clostridia | Firmicutes |
| Clostridia sp. (HG3A.0479) | T90 | -0.038 | 0.033 | 0.145 | 3249 unclassified | unclassified | unclassified | unclassified | unclassified | Clostridia | Firmicutes |
| Eubacteriales sp. (HG3A.0427) | ODI | -0.035 | 0.046 | 0.145 | 3249 unclassified | unclassified | unclassified | unclassified | Eubacteriales | Clostridia | Firmicutes |
| Eubacteriales sp. (HG3A.0516) | ODI | -0.036 | 0.046 | 0.145 | 3249 unclassified | unclassified | unclassified | unclassified | Eubacteriales | Clostridia | Firmicutes |
| Lachnospiraceae sp. (HG3A.0180) | AHI | -0.046 | 0.014 | 0.145 | 2909 unclassified | unclassified | unclassified | Lachnospiraceae | Eubacteriales | Clostridia | Firmicutes |
| Eubacteriales sp. (HG3A.0232) | ODI | -0.035 | 0.047 | 0.146 | 3249 unclassified | unclassified | unclassified | unclassified | Eubacteriales | Clostridia | Firmicutes |
| Eubacteriales sp. (HG3A.0386) | ODI | -0.035 | 0.047 | 0.146 | 3249 unclassified | unclassified | unclassified | unclassified | Eubacteriales | Clostridia | Firmicutes |
| Clostridia sp. (HG3A.1020) | T90 | -0.038 | 0.033 | 0.147 | 3249 unclassified | unclassified | unclassified | unclassified | unclassified | Clostridia | Firmicutes |
| Eubacterium ramulus (HG3A.0068) | ODI | 0.035 | 0.048 | 0.147 | 3249 unclassified | Eubacterium ramulus | Eubacterium | Eubacteriaceae | Eubacteriales | Clostridia | Firmicutes |
| Firmicutes sp. (HG3A.0641) | ODI | -0.035 | 0.048 | 0.147 | 3249 unclassified | unclassified | unclassified | unclassified | unclassified | unclassified | Firmicutes |
| Firmicutes sp. (HG3A.1082) | ODI | -0.035 | 0.048 | 0.147 | 3249 unclassified | unclassified | unclassified | unclassified | unclassified | unclassified | Firmicutes |

| Lachnoclostridium sp. (HG3A.0655) | ODI | 0.035 | 0.047 | 0.147 | 3249 unclassified | unclassified | Lachnoclostridiu  m | Lachnospiraceae | Eubacteriales | Clostridia | Firmicutes |
| --- | --- | --- | --- | --- | --- | --- | --- | --- | --- | --- | --- |
| [Ruminococcus] torques (HG3A.0088) | ODI | 0.035 | 0.049 | 0.148 | 3249 unclassified | [Ruminococcus] torques | Mediterraneibacte r | Lachnospiraceae | Eubacteriales | Clostridia | Firmicutes |
| Blautia massiliensis (HG3A.0023) | AHI | 0.045 | 0.016 | 0.148 | 2909 unclassified | Blautia  massiliensis | Blautia | Lachnospiraceae | Eubacteriales | Clostridia | Firmicutes |
| Blautia sp. SG-772 (HG3A.0063) | T90 | 0.038 | 0.034 | 0.148 | 3249 unclassified | Blautia sp. SG-772 | Blautia | Lachnospiraceae | Eubacteriales | Clostridia | Firmicutes |
| Clostridia sp. (HG3A.0682) | T90 | -0.038 | 0.034 | 0.148 | 3249 unclassified | unclassified | unclassified | unclassified | unclassified | Clostridia | Firmicutes |
| Clostridia sp. (HG3A.1403) | ODI | -0.035 | 0.048 | 0.148 | 3249 unclassified | unclassified | unclassified | unclassified | unclassified | Clostridia | Firmicutes |
| Coprobacillus cateniformis  (HG3A.0456) | T90 | 0.038 | 0.034 | 0.148 | 3249 unclassified | Coprobacillus  cateniformis | Coprobacillus | Coprobacillaceae | Erysipelotrichales | Erysipelotrichia | Firmicutes |
| Coprococcus sp. AF21-14LB (HG3A.1047) | AHI | 0.045 | 0.017 | 0.148 | 2909 unclassified | Coprococcus sp. AF21-14LB | Coprococcus | Lachnospiraceae | Eubacteriales | Clostridia | Firmicutes |
| Desulfovibrionales sp. (HG3A.0727) | AHI | 0.045 | 0.016 | 0.148 | 2909 unclassified | unclassified | unclassified | unclassified | Desulfovibrionales | Deltaproteobact  eria | Proteobacteria |
| Eubacteriales sp. (HG3A.0196) | AHI | -0.045 | 0.016 | 0.148 | 2909 unclassified | unclassified | unclassified | unclassified | Eubacteriales | Clostridia | Firmicutes |
| Eubacteriales sp. (HG3A.0270) | T90 | -0.038 | 0.034 | 0.148 | 3249 unclassified | unclassified | unclassified | unclassified | Eubacteriales | Clostridia | Firmicutes |
| Eubacteriales sp. (HG3A.0329) | AHI | -0.046 | 0.015 | 0.148 | 2909 unclassified | unclassified | unclassified | unclassified | Eubacteriales | Clostridia | Firmicutes |
| Eubacteriales sp. (HG3A.0546) | AHI | -0.046 | 0.015 | 0.148 | 2909 unclassified | unclassified | unclassified | unclassified | Eubacteriales | Clostridia | Firmicutes |
| Eubacteriales sp. (HG3A.0635) | AHI | -0.046 | 0.015 | 0.148 | 2909 unclassified | unclassified | unclassified | unclassified | Eubacteriales | Clostridia | Firmicutes |
| Eubacteriales sp. (HG3A.0730) | AHI | -0.045 | 0.017 | 0.148 | 2909 unclassified | unclassified | unclassified | unclassified | Eubacteriales | Clostridia | Firmicutes |
| Eubacteriales sp. (HG3A.0856) | T90 | -0.038 | 0.034 | 0.148 | 3249 unclassified | unclassified | unclassified | unclassified | Eubacteriales | Clostridia | Firmicutes |
| Faecalibacterium prausnitzii  (HG3A.0025) | T90 | 0.038 | 0.034 | 0.148 | 3249 unclassified | Faecalibacterium  prausnitzii | Faecalibacterium | Oscillospiraceae | Eubacteriales | Clostridia | Firmicutes |
| Firmicutes sp. (HG3A.1075) | ODI | -0.035 | 0.048 | 0.148 | 3249 unclassified | unclassified | unclassified | unclassified | unclassified | unclassified | Firmicutes |
| Holdemanella sp. (HG3A.0366) | AHI | 0.045 | 0.016 | 0.148 | 2909 unclassified | unclassified | Holdemanella | Erysipelotrichacea  e | Erysipelotrichales | Erysipelotrichia | Firmicutes |
| Lachnospiraceae sp. (HG3A.0236) | AHI | -0.045 | 0.017 | 0.148 | 2909 unclassified | unclassified | unclassified | Lachnospiraceae | Eubacteriales | Clostridia | Firmicutes |
| Oscillospiraceae sp. (HG3A.0429) | AHI | -0.045 | 0.016 | 0.148 | 2909 unclassified | unclassified | unclassified | Oscillospiraceae | Eubacteriales | Clostridia | Firmicutes |
| Oscillospiraceae sp. (HG3A.0437) | T90 | -0.038 | 0.035 | 0.148 | 3249 unclassified | unclassified | unclassified | Oscillospiraceae | Eubacteriales | Clostridia | Firmicutes |
| Sutterella seckii (HG3A.0561) | AHI | 0.045 | 0.016 | 0.148 | 2909 unclassified | Sutterella seckii | Sutterella | Sutterellaceae | Burkholderiales | Betaproteobacte  ria | Proteobacteria |
| Enterocloster citroniae (HG3A.0285) | T90 | 0.037 | 0.036 | 0.152 | 3249 unclassified | Enterocloster citroniae | Enterocloster | Lachnospiraceae | Eubacteriales | Clostridia | Firmicutes |
| Eubacteriales sp. (HG3A.0773) | T90 | -0.037 | 0.036 | 0.152 | 3249 unclassified | unclassified | unclassified | unclassified | Eubacteriales | Clostridia | Firmicutes |

| Clostridia sp. (HG3A.0550) | ODI | -0.035 | 0.05 | 0.153 | 3249 unclassified | unclassified | unclassified | unclassified | unclassified | Clostridia | Firmicutes |
| --- | --- | --- | --- | --- | --- | --- | --- | --- | --- | --- | --- |
| Roseburia inulinivorans (HG3A.0036) | T90 | 0.037 | 0.036 | 0.153 | 3249 unclassified | Roseburia  inulinivorans | Roseburia | Lachnospiraceae | Eubacteriales | Clostridia | Firmicutes |
| Clostridia sp. (HG3A.0272) | AHI | -0.045 | 0.018 | 0.154 | 2909 unclassified | unclassified | unclassified | unclassified | unclassified | Clostridia | Firmicutes |
| Eubacteriales sp. (HG3A.0211) | AHI | -0.045 | 0.018 | 0.154 | 2909 unclassified | unclassified | unclassified | unclassified | Eubacteriales | Clostridia | Firmicutes |
| Clostridia sp. (HG3A.1205) | T90 | -0.037 | 0.037 | 0.155 | 3249 unclassified | unclassified | unclassified | unclassified | unclassified | Clostridia | Firmicutes |
| Eubacteriales sp. (HG3A.0453) | ODI | -0.035 | 0.051 | 0.155 | 3249 unclassified | unclassified | unclassified | unclassified | Eubacteriales | Clostridia | Firmicutes |
| Alistipes indistinctus (HG3A.0121) | ODI | -0.034 | 0.052 | 0.156 | 3249 unclassified | Alistipes indistinctus | Alistipes | Rikenellaceae | Bacteroidales | Bacteroidia | Bacteroidetes |
| Butyricimonas virosa (HG3A.0199) | ODI | -0.034 | 0.053 | 0.156 | 3249 unclassified | Butyricimonas  virosa | Butyricimonas | Odoribacteraceae | Bacteroidales | Bacteroidia | Bacteroidetes |
| Clostridia sp. (HG3A.1008) | ODI | -0.034 | 0.052 | 0.156 | 3249 unclassified | unclassified | unclassified | unclassified | unclassified | Clostridia | Firmicutes |
| Eubacteriales sp. (HG3A.0221) | ODI | -0.034 | 0.053 | 0.156 | 3249 unclassified | unclassified | unclassified | unclassified | Eubacteriales | Clostridia | Firmicutes |
| Eubacteriales sp. (HG3A.0282) | AHI | -0.044 | 0.018 | 0.156 | 2909 unclassified | unclassified | unclassified | unclassified | Eubacteriales | Clostridia | Firmicutes |
| Eubacteriales sp. (HG3A.0289) | T90 | -0.037 | 0.038 | 0.156 | 3249 unclassified | unclassified | unclassified | unclassified | Eubacteriales | Clostridia | Firmicutes |
| Eubacteriales sp. (HG3A.0338) | T90 | -0.037 | 0.038 | 0.156 | 3249 unclassified | unclassified | unclassified | unclassified | Eubacteriales | Clostridia | Firmicutes |
| Eubacteriales sp. (HG3A.0514) | ODI | -0.035 | 0.052 | 0.156 | 3249 unclassified | unclassified | unclassified | unclassified | Eubacteriales | Clostridia | Firmicutes |
| Faecalibacterium prausnitzii (HG3A.0010) | ODI | 0.035 | 0.052 | 0.156 | 3249 unclassified | Faecalibacterium prausnitzii | Faecalibacterium | Oscillospiraceae | Eubacteriales | Clostridia | Firmicutes |
| Oscillospiraceae sp. (HG3A.0461) | T90 | -0.037 | 0.038 | 0.156 | 3249 unclassified | unclassified | unclassified | Oscillospiraceae | Eubacteriales | Clostridia | Firmicutes |
| Clostridia sp. (HG3A.0724) | ODI | -0.034 | 0.053 | 0.157 | 3249 unclassified | unclassified | unclassified | unclassified | unclassified | Clostridia | Firmicutes |
| Erysipelotrichales sp. (HG3A.1207) | ODI | 0.034 | 0.054 | 0.157 | 3249 unclassified | unclassified | unclassified | unclassified | Erysipelotrichales | Erysipelotrichia | Firmicutes |
| Eubacteriales sp. (HG3A.0771) | ODI | -0.034 | 0.054 | 0.157 | 3249 unclassified | unclassified | unclassified | unclassified | Eubacteriales | Clostridia | Firmicutes |
| Eubacteriales sp. (HG3A.0807) | ODI | -0.034 | 0.054 | 0.157 | 3249 unclassified | unclassified | unclassified | unclassified | Eubacteriales | Clostridia | Firmicutes |
| Eubacteriales sp. (HG3A.1191) | AHI | -0.044 | 0.019 | 0.157 | 2909 unclassified | unclassified | unclassified | unclassified | Eubacteriales | Clostridia | Firmicutes |
| Clostridia sp. (HG3A.0140) | AHI | -0.044 | 0.02 | 0.158 | 2909 unclassified | unclassified | unclassified | unclassified | unclassified | Clostridia | Firmicutes |
| Clostridia sp. (HG3A.1205) | ODI | -0.034 | 0.055 | 0.158 | 3249 unclassified | unclassified | unclassified | unclassified | unclassified | Clostridia | Firmicutes |
| Clostridium sp. TM06-18  (HG3A.0048) | ODI | 0.034 | 0.056 | 0.158 | 3249 unclassified | Clostridium sp.  TM06-18 | Clostridium | Clostridiaceae | Eubacteriales | Clostridia | Firmicutes |
| Eubacteriales sp. (HG3A.0136) | ODI | -0.034 | 0.056 | 0.158 | 3249 unclassified | unclassified | unclassified | unclassified | Eubacteriales | Clostridia | Firmicutes |
| Eubacteriales sp. (HG3A.0163) | ODI | -0.034 | 0.056 | 0.158 | 3249 unclassified | unclassified | unclassified | unclassified | Eubacteriales | Clostridia | Firmicutes |
| Eubacteriales sp. (HG3A.0540) | AHI | -0.044 | 0.019 | 0.158 | 2909 unclassified | unclassified | unclassified | unclassified | Eubacteriales | Clostridia | Firmicutes |
| Eubacteriales sp. (HG3A.0545) | AHI | -0.044 | 0.02 | 0.158 | 2909 unclassified | unclassified | unclassified | unclassified | Eubacteriales | Clostridia | Firmicutes |

| Eubacteriales sp. (HG3A.0760) | ODI | -0.034 | 0.055 | 0.158 | 3249 unclassified | unclassified | unclassified | unclassified | Eubacteriales | Clostridia | Firmicutes |
| --- | --- | --- | --- | --- | --- | --- | --- | --- | --- | --- | --- |
| Eubacteriales sp. (HG3A.0859) | ODI | -0.034 | 0.056 | 0.158 | 3249 unclassified | unclassified | unclassified | unclassified | Eubacteriales | Clostridia | Firmicutes |
| Oscillospiraceae sp. (HG3A.0223) | AHI | -0.044 | 0.019 | 0.158 | 2909 unclassified | unclassified | unclassified | Oscillospiraceae | Eubacteriales | Clostridia | Firmicutes |
| Roseburia intestinalis (HG3A.0078) | AHI | 0.044 | 0.019 | 0.158 | 2909 unclassified | Roseburia  intestinalis | Roseburia | Lachnospiraceae | Eubacteriales | Clostridia | Firmicutes |
| Staphylococcus aureus (HG3A.1538) | ODI | 0.034 | 0.056 | 0.158 | 3249 unclassified | Staphylococcus aureus | Staphylococcus | Staphylococcaceae | Bacillales | Bacilli | Firmicutes |
| Eubacteriales sp. (HG3A.0744) | ODI | -0.034 | 0.057 | 0.160 | 3249 unclassified | unclassified | unclassified | unclassified | Eubacteriales | Clostridia | Firmicutes |
| Longicatena caecimuris (HG3A.0571) | ODI | 0.034 | 0.057 | 0.160 | 3249 unclassified | Longicatena caecimuris | Longicatena | Erysipelotrichacea e | Erysipelotrichales | Erysipelotrichia | Firmicutes |
| Ruminococcus sp. AF46-10NS  (HG3A.0271) | ODI | 0.034 | 0.057 | 0.160 | 3249 unclassified | Ruminococcus sp.  AF46-10NS | Ruminococcus | Oscillospiraceae | Eubacteriales | Clostridia | Firmicutes |
| Blautia argi (HG3A.1450) | ODI | 0.033 | 0.06 | 0.161 | 3249 unclassified | Blautia argi | Blautia | Lachnospiraceae | Eubacteriales | Clostridia | Firmicutes |
| Clostridia sp. (HG3A.0885) | ODI | -0.033 | 0.059 | 0.161 | 3249 unclassified | unclassified | unclassified | unclassified | unclassified | Clostridia | Firmicutes |
| Clostridia sp. (HG3A.1127) | ODI | -0.034 | 0.059 | 0.161 | 3249 unclassified | unclassified | unclassified | unclassified | unclassified | Clostridia | Firmicutes |
| Erysipelotrichales sp. (HG3A.0303) | ODI | -0.034 | 0.059 | 0.161 | 3249 unclassified | unclassified | unclassified | unclassified | Erysipelotrichales | Erysipelotrichia | Firmicutes |
| Eubacteriales sp. (HG3A.0132) | ODI | -0.034 | 0.059 | 0.161 | 3249 unclassified | unclassified | unclassified | unclassified | Eubacteriales | Clostridia | Firmicutes |
| Eubacteriales sp. (HG3A.0213) | ODI | -0.034 | 0.059 | 0.161 | 3249 unclassified | unclassified | unclassified | unclassified | Eubacteriales | Clostridia | Firmicutes |
| Eubacteriales sp. (HG3A.0540) | ODI | -0.034 | 0.059 | 0.161 | 3249 unclassified | unclassified | unclassified | unclassified | Eubacteriales | Clostridia | Firmicutes |
| Eubacteriales sp. (HG3A.0621) | ODI | -0.033 | 0.06 | 0.161 | 3249 unclassified | unclassified | unclassified | unclassified | Eubacteriales | Clostridia | Firmicutes |
| Eubacteriales sp. (HG3A.0633) | T90 | -0.037 | 0.039 | 0.161 | 3249 unclassified | unclassified | unclassified | unclassified | Eubacteriales | Clostridia | Firmicutes |
| Eubacteriales sp. (HG3A.0671) | ODI | -0.034 | 0.059 | 0.161 | 3249 unclassified | unclassified | unclassified | unclassified | Eubacteriales | Clostridia | Firmicutes |
| Eubacteriales sp. (HG3A.0691) | AHI | -0.044 | 0.02 | 0.161 | 2909 unclassified | unclassified | unclassified | unclassified | Eubacteriales | Clostridia | Firmicutes |
| Eubacteriales sp. (HG3A.0790) | ODI | -0.033 | 0.06 | 0.161 | 3249 unclassified | unclassified | unclassified | unclassified | Eubacteriales | Clostridia | Firmicutes |
| Eubacteriales sp. (HG3A.1087) | ODI | -0.033 | 0.06 | 0.161 | 3249 unclassified | unclassified | unclassified | unclassified | Eubacteriales | Clostridia | Firmicutes |
| Firmicutes sp. (HG3A.0541) | ODI | -0.033 | 0.06 | 0.161 | 3249 unclassified | unclassified | unclassified | unclassified | unclassified | unclassified | Firmicutes |
| Firmicutes sp. (HG3A.0587) | ODI | -0.034 | 0.058 | 0.161 | 3249 unclassified | unclassified | unclassified | unclassified | unclassified | unclassified | Firmicutes |
| Ruminococcus sp. AM42-11  (HG3A.0002) | ODI | 0.034 | 0.058 | 0.161 | 3249 unclassified | Ruminococcus sp.  AM42-11 | Ruminococcus | Oscillospiraceae | Eubacteriales | Clostridia | Firmicutes |
| Akkermansia muciniphila (HG3A.0110) | T90 | -0.036 | 0.04 | 0.162 | 3249 unclassified | Akkermansia muciniphila | Akkermansia | Akkermansiaceae | Verrucomicrobiale s | Verrucomicrobi ae | Verrucomicrobia |

| Anaerostipes caccae (HG3A.0747) | T90 | 0.036 | 0.04 | 0.162 | 3249 unclassified | Anaerostipes  caccae | Anaerostipes | Lachnospiraceae | Eubacteriales | Clostridia | Firmicutes |
| --- | --- | --- | --- | --- | --- | --- | --- | --- | --- | --- | --- |
| Eubacteriales sp. (HG3A.0102) | AHI | -0.044 | 0.021 | 0.162 | 2909 unclassified | unclassified | unclassified | unclassified | Eubacteriales | Clostridia | Firmicutes |
| Clostridia sp. (HG3A.0733) | T90 | -0.036 | 0.042 | 0.163 | 3249 unclassified | unclassified | unclassified | unclassified | unclassified | Clostridia | Firmicutes |
| Clostridia sp. (HG3A.1356) | T90 | -0.036 | 0.041 | 0.163 | 3249 unclassified | unclassified | unclassified | unclassified | unclassified | Clostridia | Firmicutes |
| Dorea longicatena (HG3A.0039) | T90 | 0.036 | 0.041 | 0.163 | 3249 unclassified | Dorea longicatena | Dorea | Lachnospiraceae | Eubacteriales | Clostridia | Firmicutes |
| Eggerthellales sp. (HG3A.0177) | T90 | -0.036 | 0.042 | 0.163 | 3249 unclassified | unclassified | unclassified | unclassified | Eggerthellales | Coriobacteriia | Actinobacteria |
| Eubacteriales sp. (HG3A.0320) | ODI | -0.033 | 0.061 | 0.163 | 3249 unclassified | unclassified | unclassified | unclassified | Eubacteriales | Clostridia | Firmicutes |
| Eubacteriales sp. (HG3A.0421) | T90 | -0.036 | 0.04 | 0.163 | 3249 unclassified | unclassified | unclassified | unclassified | Eubacteriales | Clostridia | Firmicutes |
| Eubacteriales sp. (HG3A.0857) | T90 | -0.036 | 0.042 | 0.163 | 3249 unclassified | unclassified | unclassified | unclassified | Eubacteriales | Clostridia | Firmicutes |
| Firmicutes sp. (HG3A.0650) | T90 | -0.036 | 0.041 | 0.163 | 3249 unclassified | unclassified | unclassified | unclassified | unclassified | unclassified | Firmicutes |
| Sutterellaceae sp. (HG3A.1122) | T90 | 0.036 | 0.042 | 0.163 | 3249 unclassified | unclassified | unclassified | Sutterellaceae | Burkholderiales | Betaproteobacte  ria | Proteobacteria |
| Barnesiellaceae sp. (HG3A.1180) | ODI | -0.033 | 0.062 | 0.164 | 3249 unclassified | unclassified | unclassified | Barnesiellaceae | Bacteroidales | Bacteroidia | Bacteroidetes |
| Clostridia sp. (HG3A.1504) | T90 | -0.036 | 0.042 | 0.164 | 3249 unclassified | unclassified | unclassified | unclassified | unclassified | Clostridia | Firmicutes |
| Enterococcus faecium (HG3A.0886) | T90 | 0.036 | 0.043 | 0.164 | 3249 unclassified | Enterococcus faecium | Enterococcus | Enterococcaceae | Lactobacillales | Bacilli | Firmicutes |
| Eubacteriales sp. (HG3A.0321) | AHI | -0.043 | 0.021 | 0.164 | 2909 unclassified | unclassified | unclassified | unclassified | Eubacteriales | Clostridia | Firmicutes |
| Oscillospiraceae sp. (HG3A.0665) | T90 | -0.036 | 0.043 | 0.164 | 3249 unclassified | unclassified | unclassified | Oscillospiraceae | Eubacteriales | Clostridia | Firmicutes |
| Oscillospiraceae sp. (HG3A.0966) | T90 | -0.036 | 0.043 | 0.164 | 3249 unclassified | unclassified | unclassified | Oscillospiraceae | Eubacteriales | Clostridia | Firmicutes |
| Senegalimassilia anaerobia (HG3A.0129) | T90 | 0.036 | 0.043 | 0.164 | 3249 unclassified | Senegalimassilia anaerobia | Senegalimassilia | Coriobacteriaceae | Coriobacteriales | Coriobacteriia | Actinobacteria |
| Senegalimassilia anaerobia  (HG3A.0129) | ODI | 0.033 | 0.062 | 0.164 | 3249 unclassified | Senegalimassilia  anaerobia | Senegalimassilia | Coriobacteriaceae | Coriobacteriales | Coriobacteriia | Actinobacteria |
| Clostridia sp. (HG3A.0521) | T90 | -0.036 | 0.044 | 0.165 | 3249 unclassified | unclassified | unclassified | unclassified | unclassified | Clostridia | Firmicutes |
| Blautia sp. SG-772 (HG3A.0063) | AHI | 0.043 | 0.022 | 0.166 | 2909 unclassified | Blautia sp. SG-772 | Blautia | Lachnospiraceae | Eubacteriales | Clostridia | Firmicutes |
| Clostridia sp. (HG3A.1020) | AHI | -0.043 | 0.022 | 0.166 | 2909 unclassified | unclassified | unclassified | unclassified | unclassified | Clostridia | Firmicutes |

| Clostridia sp. (HG3A.1205) | AHI | -0.043 | 0.022 | 0.166 | 2909 unclassified | unclassified | unclassified | unclassified | unclassified | Clostridia | Firmicutes |
| --- | --- | --- | --- | --- | --- | --- | --- | --- | --- | --- | --- |
| Eubacteriales sp. (HG3A.0280) | ODI | -0.033 | 0.064 | 0.167 | 3249 unclassified | unclassified | unclassified | unclassified | Eubacteriales | Clostridia | Firmicutes |
| Eubacteriales sp. (HG3A.0950) | ODI | -0.033 | 0.063 | 0.167 | 3249 unclassified | unclassified | unclassified | unclassified | Eubacteriales | Clostridia | Firmicutes |
| Clostridia sp. (HG3A.0508) | AHI | -0.043 | 0.023 | 0.168 | 2909 unclassified | unclassified | unclassified | unclassified | unclassified | Clostridia | Firmicutes |
| Eubacteriales sp. (HG3A.0493) | T90 | -0.036 | 0.045 | 0.168 | 3249 unclassified | unclassified | unclassified | unclassified | Eubacteriales | Clostridia | Firmicutes |
| Eubacteriales sp. (HG3A.0627) | ODI | -0.033 | 0.064 | 0.168 | 3249 unclassified | unclassified | unclassified | unclassified | Eubacteriales | Clostridia | Firmicutes |
| Firmicutes sp. (HG3A.1085) | T90 | -0.036 | 0.045 | 0.168 | 3249 unclassified | unclassified | unclassified | unclassified | unclassified | unclassified | Firmicutes |
| Eubacteriales sp. (HG3A.0197) | T90 | -0.036 | 0.046 | 0.169 | 3249 unclassified | unclassified | unclassified | unclassified | Eubacteriales | Clostridia | Firmicutes |
| Oscillospiraceae sp. (HG3A.0388) | T90 | -0.036 | 0.046 | 0.169 | 3249 unclassified | unclassified | unclassified | Oscillospiraceae | Eubacteriales | Clostridia | Firmicutes |
| Eubacteriales sp. (HG3A.0381) | AHI | -0.043 | 0.023 | 0.171 | 2909 unclassified | unclassified | unclassified | unclassified | Eubacteriales | Clostridia | Firmicutes |
| Eubacteriales sp. (HG3A.0758) | ODI | -0.033 | 0.065 | 0.171 | 3249 unclassified | unclassified | unclassified | unclassified | Eubacteriales | Clostridia | Firmicutes |
| Eubacteriales sp. (HG3A.0759) | ODI | -0.033 | 0.066 | 0.171 | 3249 unclassified | unclassified | unclassified | unclassified | Eubacteriales | Clostridia | Firmicutes |
| Clostridia sp. (HG3A.1057) | ODI | -0.033 | 0.066 | 0.172 | 3249 unclassified | unclassified | unclassified | unclassified | unclassified | Clostridia | Firmicutes |
| Eubacteriales sp. (HG3A.0653) | ODI | -0.033 | 0.067 | 0.172 | 3249 unclassified | unclassified | unclassified | unclassified | Eubacteriales | Clostridia | Firmicutes |
| Eubacteriales sp. (HG3A.0977) | ODI | -0.033 | 0.067 | 0.172 | 3249 unclassified | unclassified | unclassified | unclassified | Eubacteriales | Clostridia | Firmicutes |
| Firmicutes sp. (HG3A.1054) | ODI | -0.033 | 0.067 | 0.172 | 3249 unclassified | unclassified | unclassified | unclassified | unclassified | unclassified | Firmicutes |
| Firmicutes sp. (HG3A.1345) | ODI | -0.033 | 0.067 | 0.172 | 3249 unclassified | unclassified | unclassified | unclassified | unclassified | unclassified | Firmicutes |
| Oscillospiraceae sp. (HG3A.1421) | ODI | 0.033 | 0.067 | 0.172 | 3249 unclassified | unclassified | unclassified | Oscillospiraceae | Eubacteriales | Clostridia | Firmicutes |
| Eubacteriales sp. (HG3A.0666) | ODI | -0.032 | 0.069 | 0.174 | 3249 unclassified | unclassified | unclassified | unclassified | Eubacteriales | Clostridia | Firmicutes |
| Firmicutes sp. (HG3A.0948) | ODI | -0.032 | 0.069 | 0.174 | 3249 unclassified | unclassified | unclassified | unclassified | unclassified | unclassified | Firmicutes |
| Eubacteriales sp. (HG3A.0069) | T90 | -0.035 | 0.048 | 0.175 | 3249 unclassified | unclassified | unclassified | unclassified | Eubacteriales | Clostridia | Firmicutes |
| Eubacteriales sp. (HG3A.0128) | ODI | 0.032 | 0.069 | 0.175 | 3249 unclassified | unclassified | unclassified | unclassified | Eubacteriales | Clostridia | Firmicutes |
| Alistipes sp. An66 (HG3A.1535) | AHI | -0.042 | 0.026 | 0.176 | 2909 unclassified | Alistipes sp. An66 | Alistipes | Rikenellaceae | Bacteroidales | Bacteroidia | Bacteroidetes |
| Catenibacterium mitsuokai (HG3A.0775) | AHI | 0.042 | 0.026 | 0.176 | 2909 unclassified | Catenibacterium mitsuokai | Catenibacterium | Coprobacillaceae | Erysipelotrichales | Erysipelotrichia | Firmicutes |
| Clostridia sp. (HG3A.0470) | AHI | -0.042 | 0.025 | 0.176 | 2909 unclassified | unclassified | unclassified | unclassified | unclassified | Clostridia | Firmicutes |

| Clostridia sp. (HG3A.0479) | AHI | -0.042 | 0.026 | 0.176 | 2909 unclassified | unclassified | unclassified | unclassified | unclassified | Clostridia | Firmicutes |
| --- | --- | --- | --- | --- | --- | --- | --- | --- | --- | --- | --- |
| Clostridia sp. (HG3A.1076) | AHI | -0.042 | 0.026 | 0.176 | 2909 unclassified | unclassified | unclassified | unclassified | unclassified | Clostridia | Firmicutes |
| Clostridium sp. SN20 (HG3A.0603) | AHI | 0.042 | 0.024 | 0.176 | 2909 unclassified | Clostridium sp. SN20 | Clostridium | Clostridiaceae | Eubacteriales | Clostridia | Firmicutes |
| Eubacteriales sp. (HG3A.0331) | AHI | -0.042 | 0.025 | 0.176 | 2909 unclassified | unclassified | unclassified | unclassified | Eubacteriales | Clostridia | Firmicutes |
| Gemmiger formicilis (HG3A.0027) | AHI | 0.042 | 0.026 | 0.176 | 2909 unclassified | Gemmiger formicilis | Gemmiger | unclassified | Eubacteriales | Clostridia | Firmicutes |
| Lachnospiraceae sp. (HG3A.0233) | AHI | -0.042 | 0.026 | 0.176 | 2909 unclassified | unclassified | unclassified | Lachnospiraceae | Eubacteriales | Clostridia | Firmicutes |
| Lachnospiraceae sp. (HG3A.0855) | AHI | -0.042 | 0.026 | 0.176 | 2909 unclassified | unclassified | unclassified | Lachnospiraceae | Eubacteriales | Clostridia | Firmicutes |
| Olsenella sp. AF21-51 (HG3A.0690) | AHI | -0.042 | 0.026 | 0.176 | 2909 unclassified | Olsenella sp. AF21-  51 | Olsenella | Atopobiaceae | Coriobacteriales | Coriobacteriia | Actinobacteria |
| Eubacteriales sp. (HG3A.0080) | T90 | 0.035 | 0.048 | 0.177 | 3249 unclassified | unclassified | unclassified | unclassified | Eubacteriales | Clostridia | Firmicutes |
| Eubacteriales sp. (HG3A.0652) | ODI | -0.032 | 0.071 | 0.178 | 3249 unclassified | unclassified | unclassified | unclassified | Eubacteriales | Clostridia | Firmicutes |
| Clostridium sp. M62/1 (HG3A.0354) | ODI | 0.032 | 0.071 | 0.179 | 3249 unclassified | Clostridium sp. M62/1 | Clostridium | Clostridiaceae | Eubacteriales | Clostridia | Firmicutes |
| Eubacteriales sp. (HG3A.0273) | ODI | -0.032 | 0.072 | 0.179 | 3249 unclassified | unclassified | unclassified | unclassified | Eubacteriales | Clostridia | Firmicutes |
| Eubacteriales sp. (HG3A.0280) | AHI | -0.042 | 0.027 | 0.179 | 2909 unclassified | unclassified | unclassified | unclassified | Eubacteriales | Clostridia | Firmicutes |
| Eubacteriales sp. (HG3A.0666) | AHI | -0.041 | 0.027 | 0.179 | 2909 unclassified | unclassified | unclassified | unclassified | Eubacteriales | Clostridia | Firmicutes |
| Oscillospiraceae sp. (HG3A.0445) | AHI | -0.042 | 0.027 | 0.179 | 2909 unclassified | unclassified | unclassified | Oscillospiraceae | Eubacteriales | Clostridia | Firmicutes |
| Rothia mucilaginosa (HG3A.0559) | ODI | 0.032 | 0.072 | 0.179 | 3249 unclassified | Rothia  mucilaginosa | Rothia | Micrococcaceae | Micrococcales | Actinomycetia | Actinobacteria |
| Bacteroidales sp. (HG3A.0894) | T90 | -0.035 | 0.049 | 0.180 | 3249 unclassified | unclassified | unclassified | unclassified | Bacteroidales | Bacteroidia | Bacteroidetes |
| Eubacteriales sp. (HG3A.0284) | T90 | -0.035 | 0.05 | 0.180 | 3249 unclassified | unclassified | unclassified | unclassified | Eubacteriales | Clostridia | Firmicutes |
| Eubacteriales sp. (HG3A.0604) | T90 | -0.035 | 0.05 | 0.181 | 3249 unclassified | unclassified | unclassified | unclassified | Eubacteriales | Clostridia | Firmicutes |
| Firmicutes sp. (HG3A.1124) | ODI | -0.032 | 0.073 | 0.181 | 3249 unclassified | unclassified | unclassified | unclassified | unclassified | unclassified | Firmicutes |
| Clostridia sp. (HG3A.0756) | AHI | -0.041 | 0.028 | 0.182 | 2909 unclassified | unclassified | unclassified | unclassified | unclassified | Clostridia | Firmicutes |
| Clostridia sp. (HG3A.1217) | AHI | -0.041 | 0.029 | 0.182 | 2909 unclassified | unclassified | unclassified | unclassified | unclassified | Clostridia | Firmicutes |
| Eubacteriaceae sp. (HG3A.0591) | T90 | 0.035 | 0.051 | 0.182 | 3249 unclassified | unclassified | unclassified | Eubacteriaceae | Eubacteriales | Clostridia | Firmicutes |

| Eubacteriales sp. (HG3A.0084) | T90 | -0.035 | 0.051 | 0.182 | 3249 unclassified | unclassified | unclassified | unclassified | Eubacteriales | Clostridia | Firmicutes |
| --- | --- | --- | --- | --- | --- | --- | --- | --- | --- | --- | --- |
| Eubacteriales sp. (HG3A.0128) | AHI | 0.041 | 0.029 | 0.182 | 2909 unclassified | unclassified | unclassified | unclassified | Eubacteriales | Clostridia | Firmicutes |
| Eubacteriales sp. (HG3A.0179) | AHI | -0.041 | 0.029 | 0.182 | 2909 unclassified | unclassified | unclassified | unclassified | Eubacteriales | Clostridia | Firmicutes |
| Eubacteriales sp. (HG3A.1379) | AHI | -0.041 | 0.029 | 0.182 | 2909 unclassified | unclassified | unclassified | unclassified | Eubacteriales | Clostridia | Firmicutes |
| Intestinibacillus sp. Marseille-P4005  (HG3A.0168) | AHI | 0.041 | 0.029 | 0.182 | 2909 unclassified | Intestinibacillus sp.  Marseille-P4005 | Intestinibacillus | Eubacteriaceae | Eubacteriales | Clostridia | Firmicutes |
| Lachnospiraceae sp. (HG3A.0180) | T90 | -0.035 | 0.051 | 0.182 | 3249 unclassified | unclassified | unclassified | Lachnospiraceae | Eubacteriales | Clostridia | Firmicutes |
| Eubacteriales sp. (HG3A.0630) | ODI | -0.032 | 0.074 | 0.184 | 3249 unclassified | unclassified | unclassified | unclassified | Eubacteriales | Clostridia | Firmicutes |
| Eubacteriales sp. (HG3A.1332) | ODI | -0.032 | 0.076 | 0.186 | 3249 unclassified | unclassified | unclassified | unclassified | Eubacteriales | Clostridia | Firmicutes |
| Eubacteriales sp. (HG3A.1379) | ODI | -0.032 | 0.076 | 0.186 | 3249 unclassified | unclassified | unclassified | unclassified | Eubacteriales | Clostridia | Firmicutes |
| Massilistercora timonensis (HG3A.0458) | ODI | -0.032 | 0.076 | 0.186 | 3249 unclassified | Massilistercora timonensis | Massilistercora | unclassified | Eubacteriales | Clostridia | Firmicutes |
| Rikenellaceae sp. (HG3A.1022) | ODI | -0.032 | 0.076 | 0.186 | 3249 unclassified | unclassified | unclassified | Rikenellaceae | Bacteroidales | Bacteroidia | Bacteroidetes |
| Eubacteriales sp. (HG3A.0179) | T90 | -0.034 | 0.053 | 0.188 | 3249 unclassified | unclassified | unclassified | unclassified | Eubacteriales | Clostridia | Firmicutes |
| Eubacteriales sp. (HG3A.0376) | T90 | -0.034 | 0.054 | 0.188 | 3249 unclassified | unclassified | unclassified | unclassified | Eubacteriales | Clostridia | Firmicutes |
| Eubacteriales sp. (HG3A.0439) | T90 | -0.034 | 0.054 | 0.188 | 3249 unclassified | unclassified | unclassified | unclassified | Eubacteriales | Clostridia | Firmicutes |
| Methanobrevibacter smithii |  |  |  |  |  | Methanobrevibacte | Methanobrevibact | Methanobacteriace |  |  |  |
| (HG3A.0152) T90 -0.034 0.054 0.188 3249 unclassified r smithii er ae Methanobacteriales Methanobacteria Euryarchaeota | | | | | | | | | | | |
| Oscillospiraceae sp. (HG3A.0146) | T90 | -0.034 | 0.054 | 0.188 | 3249 unclassified | unclassified | unclassified | Oscillospiraceae | Eubacteriales | Clostridia | Firmicutes |
| Ruminococcus champanellensis  (HG3A.0716) | T90 | -0.034 | 0.054 | 0.189 | 3249 unclassified | Ruminococcus  champanellensis | Ruminococcus | Oscillospiraceae | Eubacteriales | Clostridia | Firmicutes |
| Clostridia sp. (HG3A.0435) | AHI | -0.041 | 0.031 | 0.190 | 2909 unclassified | unclassified | unclassified | unclassified | unclassified | Clostridia | Firmicutes |
| Firmicutes sp. (HG3A.1054) | AHI | -0.041 | 0.031 | 0.190 | 2909 unclassified | unclassified | unclassified | unclassified | unclassified | unclassified | Firmicutes |
| Flavonifractor plautii (HG3A.0079) | AHI | 0.041 | 0.031 | 0.190 | 2909 unclassified | Flavonifractor plautii | Flavonifractor | Oscillospiraceae | Eubacteriales | Clostridia | Firmicutes |
| Bacteroidales sp. (HG3A.1236) | ODI | -0.031 | 0.078 | 0.191 | 3249 unclassified | unclassified | unclassified | unclassified | Bacteroidales | Bacteroidia | Bacteroidetes |
| Eubacteriales sp. (HG3A.0151) | ODI | -0.031 | 0.08 | 0.191 | 3249 unclassified | unclassified | unclassified | unclassified | Eubacteriales | Clostridia | Firmicutes |
| Eubacteriales sp. (HG3A.0260) | ODI | -0.031 | 0.079 | 0.191 | 3249 unclassified | unclassified | unclassified | unclassified | Eubacteriales | Clostridia | Firmicutes |
| Eubacteriales sp. (HG3A.0493) | ODI | -0.031 | 0.08 | 0.191 | 3249 unclassified | unclassified | unclassified | unclassified | Eubacteriales | Clostridia | Firmicutes |
| Eubacteriales sp. (HG3A.0511) | ODI | -0.031 | 0.08 | 0.191 | 3249 unclassified | unclassified | unclassified | unclassified | Eubacteriales | Clostridia | Firmicutes |
| Eubacteriales sp. (HG3A.0978) | ODI | -0.031 | 0.08 | 0.191 | 3249 unclassified | unclassified | unclassified | unclassified | Eubacteriales | Clostridia | Firmicutes |
| Eubacteriales sp. (HG3A.1250) | ODI | -0.031 | 0.08 | 0.191 | 3249 unclassified | unclassified | unclassified | unclassified | Eubacteriales | Clostridia | Firmicutes |
| Eubacteriales sp. (HG3A.1321) | ODI | -0.031 | 0.078 | 0.191 | 3249 unclassified | unclassified | unclassified | unclassified | Eubacteriales | Clostridia | Firmicutes |

| Phascolarctobacterium succinatutens  (HG3A.0315) | ODI | 0.031 | 0.079 | 0.191 | 3249 unclassified | Phascolarctobacter  ium succinatutens | Phascolarctobact  erium | Acidaminococcace  ae | Acidaminococcales | Negativicutes | Firmicutes |
| --- | --- | --- | --- | --- | --- | --- | --- | --- | --- | --- | --- |
| Candidatus Borkfalkiales sp. (HG3A.1397) | T90 | -0.034 | 0.056 | 0.193 | 3249 unclassified | unclassified | unclassified | unclassified | Candidatus Borkfalkiales | Clostridia | Firmicutes |
| Eubacteriales sp. (HG3A.0125) | AHI | -0.04 | 0.033 | 0.193 | 2909 unclassified | unclassified | unclassified | unclassified | Eubacteriales | Clostridia | Firmicutes |
| Eubacteriales sp. (HG3A.0254) | AHI | 0.04 | 0.033 | 0.193 | 2909 unclassified | unclassified | unclassified | unclassified | Eubacteriales | Clostridia | Firmicutes |
| Eubacteriales sp. (HG3A.1103) | AHI | -0.04 | 0.033 | 0.193 | 2909 unclassified | unclassified | unclassified | unclassified | Eubacteriales | Clostridia | Firmicutes |
| Oscillibacter sp. (HG3A.0734) | AHI | -0.04 | 0.033 | 0.193 | 2909 unclassified | unclassified | Oscillibacter | Oscillospiraceae | Eubacteriales | Clostridia | Firmicutes |
| Rikenellaceae sp. (HG3A.1022) | AHI | -0.04 | 0.032 | 0.193 | 2909 unclassified | unclassified | unclassified | Rikenellaceae | Bacteroidales | Bacteroidia | Bacteroidetes |
| Clostridia sp. (HG3A.1141) | ODI | -0.031 | 0.082 | 0.194 | 3249 unclassified | unclassified | unclassified | unclassified | unclassified | Clostridia | Firmicutes |
| [Clostridium] symbiosum  (HG3A.0370) | ODI | 0.031 | 0.084 | 0.195 | 3249 unclassified | [Clostridium]  symbiosum | Lachnoclostridiu  m | Lachnospiraceae | Eubacteriales | Clostridia | Firmicutes |
| Candidatus Borkfalkiales sp. (HG3A.1329) | ODI | -0.031 | 0.083 | 0.195 | 3249 unclassified | unclassified | unclassified | unclassified | Candidatus Borkfalkiales | Clostridia | Firmicutes |
| Eggerthellales sp. (HG3A.0174) | ODI | -0.031 | 0.084 | 0.195 | 3249 unclassified | unclassified | unclassified | unclassified | Eggerthellales | Coriobacteriia | Actinobacteria |
| Eubacteriales sp. (HG3A.0186) | ODI | -0.031 | 0.083 | 0.195 | 3249 unclassified | unclassified | unclassified | unclassified | Eubacteriales | Clostridia | Firmicutes |
| Eubacteriales sp. (HG3A.0358) | ODI | -0.031 | 0.082 | 0.195 | 3249 unclassified | unclassified | unclassified | unclassified | Eubacteriales | Clostridia | Firmicutes |
| Eubacteriales sp. (HG3A.0545) | ODI | -0.031 | 0.083 | 0.195 | 3249 unclassified | unclassified | unclassified | unclassified | Eubacteriales | Clostridia | Firmicutes |
| Eubacteriales sp. (HG3A.0910) | ODI | -0.031 | 0.083 | 0.195 | 3249 unclassified | unclassified | unclassified | unclassified | Eubacteriales | Clostridia | Firmicutes |
| Eubacteriales sp. (HG3A.1045) | ODI | -0.031 | 0.084 | 0.195 | 3249 unclassified | unclassified | unclassified | unclassified | Eubacteriales | Clostridia | Firmicutes |
| Anaeroglobus geminatus  (HG3A.1818) | ODI | 0.031 | 0.085 | 0.196 | 3249 unclassified | Anaeroglobus  geminatus | Anaeroglobus | Veillonellaceae | Veillonellales | Negativicutes | Firmicutes |
| Lachnospiraceae sp. (HG3A.0748) | ODI | -0.031 | 0.085 | 0.196 | 3249 unclassified | unclassified | unclassified | Lachnospiraceae | Eubacteriales | Clostridia | Firmicutes |
| Blautia sp. (HG3A.0416) | ODI | 0.031 | 0.086 | 0.197 | 3249 unclassified | unclassified | Blautia | Lachnospiraceae | Eubacteriales | Clostridia | Firmicutes |
| Eubacteriales sp. (HG3A.0349) | ODI | -0.03 | 0.086 | 0.197 | 3249 unclassified | unclassified | unclassified | unclassified | Eubacteriales | Clostridia | Firmicutes |
| Eubacteriales sp. (HG3A.1030) | ODI | -0.031 | 0.086 | 0.197 | 3249 unclassified | unclassified | unclassified | unclassified | Eubacteriales | Clostridia | Firmicutes |
| Eubacteriales sp. (HG3A.1078) | ODI | -0.03 | 0.087 | 0.197 | 3249 unclassified | unclassified | unclassified | unclassified | Eubacteriales | Clostridia | Firmicutes |
| Oscillospiraceae sp. (HG3A.0966) | ODI | -0.03 | 0.087 | 0.197 | 3249 unclassified | unclassified | unclassified | Oscillospiraceae | Eubacteriales | Clostridia | Firmicutes |
| Clostridia sp. (HG3A.1039) | ODI | -0.03 | 0.088 | 0.198 | 3249 unclassified | unclassified | unclassified | unclassified | unclassified | Clostridia | Firmicutes |
| Eubacteriales sp. (HG3A.0144) | ODI | -0.03 | 0.087 | 0.198 | 3249 unclassified | unclassified | unclassified | unclassified | Eubacteriales | Clostridia | Firmicutes |
| Eubacteriales sp. (HG3A.0668) | T90 | -0.034 | 0.057 | 0.198 | 3249 unclassified | unclassified | unclassified | unclassified | Eubacteriales | Clostridia | Firmicutes |
| Butyricimonas virosa (HG3A.0199) | T90 | -0.034 | 0.058 | 0.199 | 3249 unclassified | Butyricimonas  virosa | Butyricimonas | Odoribacteraceae | Bacteroidales | Bacteroidia | Bacteroidetes |

| Clostridia sp. (HG3A.0746) | AHI | -0.04 | 0.035 | 0.199 | 2909 unclassified | unclassified | unclassified | unclassified | unclassified | Clostridia | Firmicutes |
| --- | --- | --- | --- | --- | --- | --- | --- | --- | --- | --- | --- |
| Clostridium sp. AF34-13  (HG3A.0173) | T90 | -0.034 | 0.058 | 0.199 | 3249 unclassified | Clostridium sp.  AF34-13 | Clostridium | Clostridiaceae | Eubacteriales | Clostridia | Firmicutes |
| Eubacteriales sp. (HG3A.0250) | T90 | -0.034 | 0.059 | 0.199 | 3249 unclassified | unclassified | unclassified | unclassified | Eubacteriales | Clostridia | Firmicutes |
| Eubacteriales sp. (HG3A.0349) | AHI | -0.04 | 0.035 | 0.199 | 2909 unclassified | unclassified | unclassified | unclassified | Eubacteriales | Clostridia | Firmicutes |
| Eubacteriales sp. (HG3A.0759) | T90 | -0.034 | 0.059 | 0.199 | 3249 unclassified | unclassified | unclassified | unclassified | Eubacteriales | Clostridia | Firmicutes |
| Eubacteriales sp. (HG3A.0870) | AHI | -0.04 | 0.035 | 0.199 | 2909 unclassified | unclassified | unclassified | unclassified | Eubacteriales | Clostridia | Firmicutes |
| Eubacteriales sp. (HG3A.1321) | AHI | -0.04 | 0.035 | 0.199 | 2909 unclassified | unclassified | unclassified | unclassified | Eubacteriales | Clostridia | Firmicutes |
| Bacteria sp. (HG3A.1543) | ODI | -0.03 | 0.089 | 0.200 | 3249 unclassified | unclassified | unclassified | unclassified | unclassified | unclassified | unclassified |
| Blautia obeum (HG3A.0001) | AHI | 0.039 | 0.037 | 0.200 | 2909 unclassified | Blautia obeum | Blautia | Lachnospiraceae | Eubacteriales | Clostridia | Firmicutes |
| Collinsella sp. WCA1-178-WT-3 |  |  |  |  |  | Collinsella sp.  WCA1-178-WT-3 |  |  |  |  |  |
| (M2) (HG3A.1245) | AHI | 0.039 | 0.037 | 0.200 | 2909 unclassified | (M2) | Collinsella | Coriobacteriaceae | Coriobacteriales | Coriobacteriia | Actinobacteria |
| Eubacteriales sp. (HG3A.0242) | AHI | -0.039 | 0.036 | 0.200 | 2909 unclassified | unclassified | unclassified | unclassified | Eubacteriales | Clostridia | Firmicutes |
| Eubacteriales sp. (HG3A.0426) | AHI | -0.039 | 0.036 | 0.200 | 2909 unclassified | unclassified | unclassified | unclassified | Eubacteriales | Clostridia | Firmicutes |
| Eubacteriales sp. (HG3A.0771) | AHI | -0.04 | 0.036 | 0.200 | 2909 unclassified | unclassified | unclassified | unclassified | Eubacteriales | Clostridia | Firmicutes |
| Eubacteriales sp. (HG3A.1445) | AHI | -0.039 | 0.037 | 0.200 | 2909 unclassified | unclassified | unclassified | unclassified | Eubacteriales | Clostridia | Firmicutes |
| Oscillospiraceae sp. (HG3A.0256) | AHI | 0.039 | 0.038 | 0.200 | 2909 unclassified | unclassified | unclassified | Oscillospiraceae | Eubacteriales | Clostridia | Firmicutes |
| Oxalobacter formigenes  (HG3A.0552) | AHI | -0.039 | 0.037 | 0.200 | 2909 unclassified | Oxalobacter  formigenes | Oxalobacter | Oxalobacteraceae | Burkholderiales | Betaproteobacte  ria | Proteobacteria |
| Parvimonas micra (HG3A.1231) | AHI | 0.039 | 0.037 | 0.200 | 2909 unclassified | Parvimonas micra | Parvimonas | Peptoniphilaceae | Tissierellales | Tissierellia | Firmicutes |
| Blautia obeum (HG3A.0009) | AHI | 0.039 | 0.038 | 0.201 | 2909 unclassified | Blautia obeum | Blautia | Lachnospiraceae | Eubacteriales | Clostridia | Firmicutes |
| Coprococcus sp. OM04-5BH (HG3A.1028) | AHI | -0.039 | 0.039 | 0.201 | 2909 unclassified | Coprococcus sp. OM04-5BH | Coprococcus | Lachnospiraceae | Eubacteriales | Clostridia | Firmicutes |
| Eggerthellaceae sp. (HG3A.0171) | AHI | -0.039 | 0.039 | 0.201 | 2909 unclassified | unclassified | unclassified | Eggerthellaceae | Eggerthellales | Coriobacteriia | Actinobacteria |
| Eubacteriales sp. (HG3A.0151) | AHI | -0.039 | 0.038 | 0.201 | 2909 unclassified | unclassified | unclassified | unclassified | Eubacteriales | Clostridia | Firmicutes |
| Eubacteriales sp. (HG3A.1354) | T90 | -0.033 | 0.06 | 0.201 | 3249 unclassified | unclassified | unclassified | unclassified | Eubacteriales | Clostridia | Firmicutes |
| Firmicutes sp. (HG3A.0860) | T90 | -0.033 | 0.06 | 0.201 | 3249 unclassified | unclassified | unclassified | unclassified | unclassified | unclassified | Firmicutes |
| Eubacteriales sp. (HG3A.0418) | ODI | -0.03 | 0.091 | 0.202 | 3249 unclassified | unclassified | unclassified | unclassified | Eubacteriales | Clostridia | Firmicutes |
| Eubacteriales sp. (HG3A.0489) | ODI | -0.03 | 0.09 | 0.202 | 3249 unclassified | unclassified | unclassified | unclassified | Eubacteriales | Clostridia | Firmicutes |
| Eubacteriales sp. (HG3A.0821) | ODI | -0.03 | 0.09 | 0.202 | 3249 unclassified | unclassified | unclassified | unclassified | Eubacteriales | Clostridia | Firmicutes |
| Firmicutes sp. (HG3A.1014) | T90 | -0.033 | 0.06 | 0.202 | 3249 unclassified | unclassified | unclassified | unclassified | unclassified | unclassified | Firmicutes |
| Blautia massiliensis (HG3A.0023) | T90 | 0.033 | 0.062 | 0.203 | 3249 unclassified | Blautia  massiliensis | Blautia | Lachnospiraceae | Eubacteriales | Clostridia | Firmicutes |

| Clostridiaceae sp. (HG3A.0238) | T90 | -0.033 | 0.061 | 0.203 | 3249 unclassified | unclassified | unclassified | Clostridiaceae | Eubacteriales | Clostridia | Firmicutes |
| --- | --- | --- | --- | --- | --- | --- | --- | --- | --- | --- | --- |
| Eubacteriales sp. (HG3A.0229) | T90 | -0.033 | 0.062 | 0.203 | 3249 unclassified | unclassified | unclassified | unclassified | Eubacteriales | Clostridia | Firmicutes |
| Eubacteriales sp. (HG3A.0718) | T90 | -0.033 | 0.062 | 0.203 | 3249 unclassified | unclassified | unclassified | unclassified | Eubacteriales | Clostridia | Firmicutes |
| Firmicutes sp. (HG3A.0681) | T90 | -0.033 | 0.061 | 0.203 | 3249 unclassified | unclassified | unclassified | unclassified | unclassified | unclassified | Firmicutes |
| Phascolarctobacterium succinatutens (HG3A.0315) | T90 | 0.033 | 0.062 | 0.203 | 3249 unclassified | Phascolarctobacter ium succinatutens | Phascolarctobact erium | Acidaminococcace ae | Acidaminococcales | Negativicutes | Firmicutes |
| Eubacteriales sp. (HG3A.0450) | ODI | -0.03 | 0.092 | 0.204 | 3249 unclassified | unclassified | unclassified | unclassified | Eubacteriales | Clostridia | Firmicutes |
| Eubacteriales sp. (HG3A.0557) | AHI | -0.039 | 0.04 | 0.204 | 2909 unclassified | unclassified | unclassified | unclassified | Eubacteriales | Clostridia | Firmicutes |
| Eubacteriales sp. (HG3A.0557) | ODI | -0.03 | 0.091 | 0.204 | 3249 unclassified | unclassified | unclassified | unclassified | Eubacteriales | Clostridia | Firmicutes |
| Lachnospiraceae sp. (HG3A.0899) | ODI | -0.03 | 0.092 | 0.204 | 3249 unclassified | unclassified | unclassified | Lachnospiraceae | Eubacteriales | Clostridia | Firmicutes |
| Clostridia sp. (HG3A.0011) | T90 | 0.033 | 0.064 | 0.206 | 3249 unclassified | unclassified | unclassified | unclassified | unclassified | Clostridia | Firmicutes |
| Clostridia sp. (HG3A.0599) | T90 | -0.033 | 0.064 | 0.206 | 3249 unclassified | unclassified | unclassified | unclassified | unclassified | Clostridia | Firmicutes |
| Olsenella sp. AF21-51 (HG3A.0690) | T90 | -0.033 | 0.064 | 0.207 | 3249 unclassified | Olsenella sp. AF21-  51 | Olsenella | Atopobiaceae | Coriobacteriales | Coriobacteriia | Actinobacteria |
| Eubacteriales sp. (HG3A.0120) | AHI | -0.038 | 0.041 | 0.208 | 2909 unclassified | unclassified | unclassified | unclassified | Eubacteriales | Clostridia | Firmicutes |
| Eubacteriales sp. (HG3A.0846) | T90 | -0.033 | 0.065 | 0.208 | 3249 unclassified | unclassified | unclassified | unclassified | Eubacteriales | Clostridia | Firmicutes |
| Eubacteriales sp. (HG3A.0377) | ODI | -0.03 | 0.095 | 0.209 | 3249 unclassified | unclassified | unclassified | unclassified | Eubacteriales | Clostridia | Firmicutes |
| Eubacteriales sp. (HG3A.0592) | ODI | -0.03 | 0.095 | 0.209 | 3249 unclassified | unclassified | unclassified | unclassified | Eubacteriales | Clostridia | Firmicutes |
| Eubacteriales sp. (HG3A.0316) | AHI | -0.038 | 0.041 | 0.210 | 2909 unclassified | unclassified | unclassified | unclassified | Eubacteriales | Clostridia | Firmicutes |
| Anaerotruncus colihominis  (HG3A.0307) | ODI | 0.029 | 0.098 | 0.212 | 3249 unclassified | Anaerotruncus  colihominis | Anaerotruncus | Oscillospiraceae | Eubacteriales | Clostridia | Firmicutes |
| Erysipelotrichales sp. (HG3A.1207) | AHI | 0.038 | 0.042 | 0.212 | 2909 unclassified | unclassified | unclassified | unclassified | Erysipelotrichales | Erysipelotrichia | Firmicutes |
| Eubacteriales sp. (HG3A.0396) | ODI | -0.03 | 0.097 | 0.212 | 3249 unclassified | unclassified | unclassified | unclassified | Eubacteriales | Clostridia | Firmicutes |
| Eubacteriales sp. (HG3A.0477) | ODI | -0.029 | 0.097 | 0.212 | 3249 unclassified | unclassified | unclassified | unclassified | Eubacteriales | Clostridia | Firmicutes |
| Eubacteriales sp. (HG3A.0486) | AHI | -0.038 | 0.042 | 0.212 | 2909 unclassified | unclassified | unclassified | unclassified | Eubacteriales | Clostridia | Firmicutes |
| Eubacteriales sp. (HG3A.0577) | ODI | -0.029 | 0.097 | 0.212 | 3249 unclassified | unclassified | unclassified | unclassified | Eubacteriales | Clostridia | Firmicutes |
| Eubacteriales sp. (HG3A.0617) | AHI | -0.038 | 0.042 | 0.212 | 2909 unclassified | unclassified | unclassified | unclassified | Eubacteriales | Clostridia | Firmicutes |
| Firmicutes sp. (HG3A.0587) | AHI | -0.038 | 0.043 | 0.212 | 2909 unclassified | unclassified | unclassified | unclassified | unclassified | unclassified | Firmicutes |
| Firmicutes sp. (HG3A.0596) | AHI | -0.038 | 0.043 | 0.212 | 2909 unclassified | unclassified | unclassified | unclassified | unclassified | unclassified | Firmicutes |
| Sutterellaceae sp. (HG3A.1122) | ODI | 0.03 | 0.097 | 0.212 | 3249 unclassified | unclassified | unclassified | Sutterellaceae | Burkholderiales | Betaproteobacte ria | Proteobacteria |
| Barnesiellaceae sp. (HG3A.1180) | AHI | -0.038 | 0.044 | 0.214 | 2909 unclassified | unclassified | unclassified | Barnesiellaceae | Bacteroidales | Bacteroidia | Bacteroidetes |

| Blastocystis sp. subtype 4  (HG3A.0446) | T90 | -0.033 | 0.067 | 0.214 | 3249 unclassified | Blastocystis sp.  subtype 4 | Blastocystis | Blastocystidae | Opalinata | Bigyra | unclassified |
| --- | --- | --- | --- | --- | --- | --- | --- | --- | --- | --- | --- |
| Clostridia sp. (HG3A.1148) | ODI | -0.029 | 0.099 | 0.215 | 3249 unclassified | unclassified | unclassified | unclassified | unclassified | Clostridia | Firmicutes |
| Eubacteriales sp. (HG3A.0113) | ODI | -0.029 | 0.1 | 0.215 | 3249 unclassified | unclassified | unclassified | unclassified | Eubacteriales | Clostridia | Firmicutes |
| Prevotella sp. (HG3A.1009) | ODI | 0.029 | 0.1 | 0.215 | 3249 unclassified | unclassified | Prevotella | Prevotellaceae | Bacteroidales | Bacteroidia | Bacteroidetes |
| Bacteria sp. (HG3A.0459) | AHI | -0.038 | 0.045 | 0.217 | 2909 unclassified | unclassified | unclassified | unclassified | unclassified | unclassified | unclassified |
| Eubacteriales sp. (HG3A.0578) | AHI | -0.038 | 0.045 | 0.217 | 2909 unclassified | unclassified | unclassified | unclassified | Eubacteriales | Clostridia | Firmicutes |
| Eubacteriales sp. (HG3A.1239) | AHI | -0.038 | 0.045 | 0.217 | 2909 unclassified | unclassified | unclassified | unclassified | Eubacteriales | Clostridia | Firmicutes |
| Eubacterium sp. AF22-8LB  (HG3A.0838) | AHI | 0.038 | 0.045 | 0.217 | 2909 unclassified | Eubacterium sp.  AF22-8LB | Eubacterium | Eubacteriaceae | Eubacteriales | Clostridia | Firmicutes |
| Intestinimonas sp. (HG3A.1018) | T90 | -0.032 | 0.068 | 0.217 | 3249 unclassified | unclassified | Intestinimonas | unclassified | Eubacteriales | Clostridia | Firmicutes |
| Pseudoruminococcus massiliensis  (HG3A.0346) | T90 | -0.032 | 0.069 | 0.217 | 3249 unclassified | Pseudoruminococc  us massiliensis | Pseudoruminococ  cus | Oscillospiraceae | Eubacteriales | Clostridia | Firmicutes |
| Anaerostipes sp. BG01 (HG3A.1509) | AHI | 0.037 | 0.047 | 0.218 | 2909 unclassified | Anaerostipes sp. BG01 | Anaerostipes | Lachnospiraceae | Eubacteriales | Clostridia | Firmicutes |
| Butyricicoccus sp. OM04-18BH  (HG3A.0139) | AHI | -0.037 | 0.048 | 0.218 | 2909 unclassified | Butyricicoccus sp.  OM04-18BH | Butyricicoccus | Clostridiaceae | Eubacteriales | Clostridia | Firmicutes |
| Clostridia sp. (HG3A.1403) | AHI | -0.038 | 0.046 | 0.218 | 2909 unclassified | unclassified | unclassified | unclassified | unclassified | Clostridia | Firmicutes |
| Clostridiaceae sp. (HG3A.0238) | AHI | -0.037 | 0.046 | 0.218 | 2909 unclassified | unclassified | unclassified | Clostridiaceae | Eubacteriales | Clostridia | Firmicutes |
| Clostridium sp. AT4 (HG3A.0347) | AHI | 0.037 | 0.048 | 0.218 | 2909 unclassified | Clostridium sp. AT4 | Clostridium | Clostridiaceae | Eubacteriales | Clostridia | Firmicutes |
| Eubacteriales sp. (HG3A.0264) | AHI | -0.037 | 0.047 | 0.218 | 2909 unclassified | unclassified | unclassified | unclassified | Eubacteriales | Clostridia | Firmicutes |
| Eubacteriales sp. (HG3A.0977) | AHI | -0.037 | 0.048 | 0.218 | 2909 unclassified | unclassified | unclassified | unclassified | Eubacteriales | Clostridia | Firmicutes |
| Oscillospiraceae sp. (HG3A.0380) | AHI | -0.037 | 0.048 | 0.218 | 2909 unclassified | unclassified | unclassified | Oscillospiraceae | Eubacteriales | Clostridia | Firmicutes |
| Oscillospiraceae sp. (HG3A.0576) | T90 | -0.032 | 0.069 | 0.218 | 3249 unclassified | unclassified | unclassified | Oscillospiraceae | Eubacteriales | Clostridia | Firmicutes |
| Clostridia sp. (HG3A.1108) | ODI | -0.029 | 0.102 | 0.219 | 3249 unclassified | unclassified | unclassified | unclassified | unclassified | Clostridia | Firmicutes |
| Eubacteriales sp. (HG3A.0643) | ODI | -0.029 | 0.103 | 0.219 | 3249 unclassified | unclassified | unclassified | unclassified | Eubacteriales | Clostridia | Firmicutes |
| Firmicutes sp. (HG3A.1195) | ODI | -0.029 | 0.102 | 0.219 | 3249 unclassified | unclassified | unclassified | unclassified | unclassified | unclassified | Firmicutes |
| Eubacteriales sp. (HG3A.0232) | T90 | -0.032 | 0.07 | 0.220 | 3249 unclassified | unclassified | unclassified | unclassified | Eubacteriales | Clostridia | Firmicutes |
| Lactobacillus acidophilus  (HG3A.0672) | ODI | -0.029 | 0.104 | 0.221 | 3249 unclassified | Lactobacillus  acidophilus | Lactobacillus | Lactobacillaceae | Lactobacillales | Bacilli | Firmicutes |
| Eubacteriales sp. (HG3A.1187) | T90 | -0.032 | 0.071 | 0.222 | 3249 unclassified | unclassified | unclassified | unclassified | Eubacteriales | Clostridia | Firmicutes |
| Firmicutes sp. (HG3A.0526) | T90 | -0.032 | 0.071 | 0.222 | 3249 unclassified | unclassified | unclassified | unclassified | unclassified | unclassified | Firmicutes |
| Clostridia sp. (HG3A.1137) | T90 | -0.032 | 0.072 | 0.223 | 3249 unclassified | unclassified | unclassified | unclassified | unclassified | Clostridia | Firmicutes |
| Clostridia sp. (HG3A.1609) | T90 | -0.032 | 0.072 | 0.223 | 3249 unclassified | unclassified | unclassified | unclassified | unclassified | Clostridia | Firmicutes |

| Eubacteriales sp. (HG3A.0613) | ODI | -0.029 | 0.105 | 0.223 | 3249 unclassified | unclassified | unclassified | unclassified | Eubacteriales | Clostridia | Firmicutes |
| --- | --- | --- | --- | --- | --- | --- | --- | --- | --- | --- | --- |
| Mesosutterella multiformis  (HG3A.0520) | ODI | -0.029 | 0.105 | 0.223 | 3249 unclassified | Mesosutterella  multiformis | Mesosutterella | Sutterellaceae | Burkholderiales | Betaproteobacte  ria | Proteobacteria |
| Coprococcus sp. AF21-14LB (HG3A.1047) | ODI | 0.029 | 0.107 | 0.224 | 3249 unclassified | Coprococcus sp. AF21-14LB | Coprococcus | Lachnospiraceae | Eubacteriales | Clostridia | Firmicutes |
| Eubacteriales sp. (HG3A.0334) | ODI | -0.029 | 0.106 | 0.224 | 3249 unclassified | unclassified | unclassified | unclassified | Eubacteriales | Clostridia | Firmicutes |
| Clostridia sp. (HG3A.0841) | ODI | -0.029 | 0.107 | 0.225 | 3249 unclassified | unclassified | unclassified | unclassified | unclassified | Clostridia | Firmicutes |
| Streptococcus anginosus  (HG3A.0680) | AHI | 0.037 | 0.05 | 0.226 | 2909 unclassified | Streptococcus  anginosus | Streptococcus | Streptococcaceae | Lactobacillales | Bacilli | Firmicutes |
| Eubacteriales sp. (HG3A.0873) | ODI | -0.029 | 0.108 | 0.227 | 3249 unclassified | unclassified | unclassified | unclassified | Eubacteriales | Clostridia | Firmicutes |
| Eubacteriales sp. (HG3A.0085) | T90 | -0.032 | 0.074 | 0.228 | 3249 unclassified | unclassified | unclassified | unclassified | Eubacteriales | Clostridia | Firmicutes |
| Fusicatenibacter saccharivorans (HG3A.0004) | T90 | 0.032 | 0.074 | 0.228 | 3249 unclassified | Fusicatenibacter saccharivorans | Fusicatenibacter | Lachnospiraceae | Eubacteriales | Clostridia | Firmicutes |
| Veillonella rogosae (HG3A.0324) | AHI | -0.037 | 0.051 | 0.228 | 2909 unclassified | Veillonella rogosae | Veillonella | Veillonellaceae | Veillonellales | Negativicutes | Firmicutes |
| Eubacteriales sp. (HG3A.1026) | AHI | -0.037 | 0.051 | 0.229 | 2909 unclassified | unclassified | unclassified | unclassified | Eubacteriales | Clostridia | Firmicutes |
| Eubacteriales sp. (HG3A.0736) | T90 | -0.032 | 0.076 | 0.231 | 3249 unclassified | unclassified | unclassified | unclassified | Eubacteriales | Clostridia | Firmicutes |
| Eubacteriales sp. (HG3A.0976) | T90 | -0.032 | 0.076 | 0.231 | 3249 unclassified | unclassified | unclassified | unclassified | Eubacteriales | Clostridia | Firmicutes |
| Blautia sp. (HG3A.0416) | T90 | 0.031 | 0.078 | 0.233 | 3249 unclassified | unclassified | Blautia | Lachnospiraceae | Eubacteriales | Clostridia | Firmicutes |
| Coprococcus sp. OM04-5BH (HG3A.1028) | ODI | -0.028 | 0.112 | 0.233 | 3249 unclassified | Coprococcus sp. OM04-5BH | Coprococcus | Lachnospiraceae | Eubacteriales | Clostridia | Firmicutes |
| Eubacteriales sp. (HG3A.0128) | T90 | 0.031 | 0.077 | 0.233 | 3249 unclassified | unclassified | unclassified | unclassified | Eubacteriales | Clostridia | Firmicutes |
| Eubacteriales sp. (HG3A.0325) | ODI | -0.028 | 0.112 | 0.233 | 3249 unclassified | unclassified | unclassified | unclassified | Eubacteriales | Clostridia | Firmicutes |
| Eubacterium sp. (HG3A.0214) | T90 | 0.031 | 0.078 | 0.233 | 3249 unclassified | unclassified | Eubacterium | Eubacteriaceae | Eubacteriales | Clostridia | Firmicutes |
| Lachnospiraceae sp. (HG3A.0855) | T90 | -0.031 | 0.078 | 0.233 | 3249 unclassified | unclassified | unclassified | Lachnospiraceae | Eubacteriales | Clostridia | Firmicutes |
| Lachnospiraceae sp. (HG3A.1525) | ODI | -0.028 | 0.113 | 0.233 | 3249 unclassified | unclassified | unclassified | Lachnospiraceae | Eubacteriales | Clostridia | Firmicutes |
| Limosilactobacillus fermentum (HG3A.0990) | ODI | 0.028 | 0.112 | 0.233 | 3249 unclassified | Limosilactobacillus fermentum | Limosilactobacillu s | Lactobacillaceae | Lactobacillales | Bacilli | Firmicutes |
| Oscillospiraceae sp. (HG3A.0380) | ODI | -0.028 | 0.112 | 0.233 | 3249 unclassified | unclassified | unclassified | Oscillospiraceae | Eubacteriales | Clostridia | Firmicutes |
| Bacteria sp. (HG3A.0492) | ODI | -0.028 | 0.113 | 0.234 | 3249 unclassified | unclassified | unclassified | unclassified | unclassified | unclassified | unclassified |
| Eubacteriales sp. (HG3A.0132) | AHI | -0.036 | 0.053 | 0.234 | 2909 unclassified | unclassified | unclassified | unclassified | Eubacteriales | Clostridia | Firmicutes |
| Eubacteriales sp. (HG3A.0472) | ODI | -0.028 | 0.114 | 0.234 | 3249 unclassified | unclassified | unclassified | unclassified | Eubacteriales | Clostridia | Firmicutes |
| Eubacteriales sp. (HG3A.0482) | AHI | -0.036 | 0.053 | 0.234 | 2909 unclassified | unclassified | unclassified | unclassified | Eubacteriales | Clostridia | Firmicutes |
| [Ruminococcus] torques (HG3A.0088) | AHI | 0.036 | 0.054 | 0.235 | 2909 unclassified | [Ruminococcus] torques | Mediterraneibacte r | Lachnospiraceae | Eubacteriales | Clostridia | Firmicutes |

| Eubacteriales sp. (HG3A.0548) | AHI | -0.036 | 0.054 | 0.235 | 2909 unclassified | unclassified | unclassified | unclassified | Eubacteriales | Clostridia | Firmicutes |
| --- | --- | --- | --- | --- | --- | --- | --- | --- | --- | --- | --- |
| Eubacteriales sp. (HG3A.0643) | AHI | -0.036 | 0.054 | 0.235 | 2909 unclassified | unclassified | unclassified | unclassified | Eubacteriales | Clostridia | Firmicutes |
| Eubacteriales sp. (HG3A.1227) | ODI | -0.028 | 0.114 | 0.235 | 3249 unclassified | unclassified | unclassified | unclassified | Eubacteriales | Clostridia | Firmicutes |
| Longicatena caecimuris (HG3A.0571) | T90 | 0.031 | 0.079 | 0.236 | 3249 unclassified | Longicatena caecimuris | Longicatena | Erysipelotrichacea e | Erysipelotrichales | Erysipelotrichia | Firmicutes |
| Anaerobutyricum hallii (HG3A.0012) | ODI | 0.028 | 0.118 | 0.237 | 3249 unclassified | Anaerobutyricum  hallii | Anaerobutyricum | Lachnospiraceae | Eubacteriales | Clostridia | Firmicutes |
| Clostridia sp. (HG3A.1427) | ODI | -0.028 | 0.118 | 0.237 | 3249 unclassified | unclassified | unclassified | unclassified | unclassified | Clostridia | Firmicutes |
| Clostridiaceae sp. (HG3A.0238) | ODI | -0.028 | 0.117 | 0.237 | 3249 unclassified | unclassified | unclassified | Clostridiaceae | Eubacteriales | Clostridia | Firmicutes |
| Eggerthella lenta (HG3A.0225) | ODI | 0.028 | 0.118 | 0.237 | 3249 unclassified | Eggerthella lenta | Eggerthella | Eggerthellaceae | Eggerthellales | Coriobacteriia | Actinobacteria |
| Eubacteriales sp. (HG3A.0254) | ODI | 0.028 | 0.116 | 0.237 | 3249 unclassified | unclassified | unclassified | unclassified | Eubacteriales | Clostridia | Firmicutes |
| Eubacteriales sp. (HG3A.0338) | ODI | -0.028 | 0.117 | 0.237 | 3249 unclassified | unclassified | unclassified | unclassified | Eubacteriales | Clostridia | Firmicutes |
| Eubacteriales sp. (HG3A.0633) | ODI | -0.028 | 0.117 | 0.237 | 3249 unclassified | unclassified | unclassified | unclassified | Eubacteriales | Clostridia | Firmicutes |
| Sellimonas intestinalis (HG3A.0417) | ODI | 0.028 | 0.118 | 0.237 | 3249 unclassified | Sellimonas intestinalis | Sellimonas | Lachnospiraceae | Eubacteriales | Clostridia | Firmicutes |
| Clostridia sp. (HG3A.0933) | ODI | -0.028 | 0.12 | 0.238 | 3249 unclassified | unclassified | unclassified | unclassified | unclassified | Clostridia | Firmicutes |
| Collinsella sp. WCA1-178-WT-3 |  |  |  |  |  | Collinsella sp.  WCA1-178-WT-3 |  |  |  |  |  |
| (M2) (HG3A.1245) | ODI | 0.028 | 0.119 | 0.238 | 3249 unclassified | (M2) | Collinsella | Coriobacteriaceae | Coriobacteriales | Coriobacteriia | Actinobacteria |
| Eubacteriales sp. (HG3A.0441) | ODI | -0.028 | 0.12 | 0.238 | 3249 unclassified | unclassified | unclassified | unclassified | Eubacteriales | Clostridia | Firmicutes |
| Eubacteriales sp. (HG3A.0568) | ODI | -0.028 | 0.119 | 0.238 | 3249 unclassified | unclassified | unclassified | unclassified | Eubacteriales | Clostridia | Firmicutes |
| Eubacteriales sp. (HG3A.0609) | AHI | -0.036 | 0.055 | 0.238 | 2909 unclassified | unclassified | unclassified | unclassified | Eubacteriales | Clostridia | Firmicutes |
| Flavonifractor sp. An10 (HG3A.0495) | ODI | -0.028 | 0.119 | 0.238 | 3249 unclassified | Flavonifractor sp. An10 | Flavonifractor | Oscillospiraceae | Eubacteriales | Clostridia | Firmicutes |
| Sutterella sp. KLE1602 (HG3A.0228) | ODI | 0.028 | 0.12 | 0.238 | 3249 unclassified | Sutterella sp.  KLE1602 | Sutterella | Sutterellaceae | Burkholderiales | Betaproteobacte  ria | Proteobacteria |
| Clostridia sp. (HG3A.0787) | T90 | -0.031 | 0.081 | 0.239 | 3249 unclassified | unclassified | unclassified | unclassified | unclassified | Clostridia | Firmicutes |
| Eubacteriales sp. (HG3A.0215) | T90 | -0.031 | 0.081 | 0.239 | 3249 unclassified | unclassified | unclassified | unclassified | Eubacteriales | Clostridia | Firmicutes |
| Eubacteriales sp. (HG3A.0757) | T90 | -0.031 | 0.082 | 0.239 | 3249 unclassified | unclassified | unclassified | unclassified | Eubacteriales | Clostridia | Firmicutes |
| Eubacteriales sp. (HG3A.0950) | T90 | -0.031 | 0.081 | 0.239 | 3249 unclassified | unclassified | unclassified | unclassified | Eubacteriales | Clostridia | Firmicutes |
| Victivallales sp. (HG3A.0824) | T90 | -0.031 | 0.081 | 0.239 | 3249 unclassified | unclassified | unclassified | unclassified | Victivallales | Lentisphaeria | Lentisphaerae |
| Anaerotruncus massiliensis  (HG3A.0460) | ODI | -0.027 | 0.122 | 0.240 | 3249 unclassified | Anaerotruncus  massiliensis | Anaerotruncus | Oscillospiraceae | Eubacteriales | Clostridia | Firmicutes |
| Eubacteriales sp. (HG3A.0263) | ODI | -0.027 | 0.122 | 0.240 | 3249 unclassified | unclassified | unclassified | unclassified | Eubacteriales | Clostridia | Firmicutes |
| Traorella massiliensis (HG3A.0669) | ODI | -0.027 | 0.122 | 0.240 | 3249 unclassified | Traorella  massiliensis | Traorella | Erysipelotrichacea  e | Erysipelotrichales | Erysipelotrichia | Firmicutes |
| Bacteria sp. (HG3A.0911) | ODI | -0.027 | 0.123 | 0.241 | 3249 unclassified | unclassified | unclassified | unclassified | unclassified | unclassified | unclassified |

| Oscillospiraceae sp. (HG3A.0849) | T90 | -0.031 | 0.083 | 0.241 | 3249 unclassified | unclassified | unclassified | Oscillospiraceae | Eubacteriales | Clostridia | Firmicutes |
| --- | --- | --- | --- | --- | --- | --- | --- | --- | --- | --- | --- |
| Coprococcus sp. (HG3A.0404) | AHI | 0.036 | 0.058 | 0.242 | 2909 unclassified | unclassified | Coprococcus | Lachnospiraceae | Eubacteriales | Clostridia | Firmicutes |
| Eubacteriales sp. (HG3A.0083) | AHI | -0.036 | 0.058 | 0.242 | 2909 unclassified | unclassified | unclassified | unclassified | Eubacteriales | Clostridia | Firmicutes |
| Eubacteriales sp. (HG3A.0175) | AHI | -0.036 | 0.056 | 0.242 | 2909 unclassified | unclassified | unclassified | unclassified | Eubacteriales | Clostridia | Firmicutes |
| Eubacteriales sp. (HG3A.0618) | AHI | -0.036 | 0.057 | 0.242 | 2909 unclassified | unclassified | unclassified | unclassified | Eubacteriales | Clostridia | Firmicutes |
| Firmicutes sp. (HG3A.0650) | AHI | -0.036 | 0.058 | 0.242 | 2909 unclassified | unclassified | unclassified | unclassified | unclassified | unclassified | Firmicutes |
| Ruminococcus champanellensis  (HG3A.0716) | AHI | -0.036 | 0.057 | 0.242 | 2909 unclassified | Ruminococcus  champanellensis | Ruminococcus | Oscillospiraceae | Eubacteriales | Clostridia | Firmicutes |
| Ruminococcus sp. AF46-10NS (HG3A.0271) | AHI | 0.036 | 0.057 | 0.242 | 2909 unclassified | Ruminococcus sp. AF46-10NS | Ruminococcus | Oscillospiraceae | Eubacteriales | Clostridia | Firmicutes |
| Eubacteriales sp. (HG3A.1154) | ODI | -0.027 | 0.125 | 0.243 | 3249 unclassified | unclassified | unclassified | unclassified | Eubacteriales | Clostridia | Firmicutes |
| Eubacterium sp. AM49-13BH (HG3A.0251) | ODI | -0.027 | 0.125 | 0.243 | 3249 unclassified | Eubacterium sp. AM49-13BH | Eubacterium | Eubacteriaceae | Eubacteriales | Clostridia | Firmicutes |
| Candidatus Borkfalkia ceftriaxoniphila |  |  |  |  |  | Candidatus  Borkfalkia | Candidatus | Candidatus | Candidatus |  |  |
| (HG3A.0595) | T90 | -0.031 | 0.084 | 0.244 | 3249 unclassified | ceftriaxoniphila | Borkfalkia | Borkfalkiaceae | Borkfalkiales | Clostridia | Firmicutes |
| Prevotella colorans (HG3A.1470) | T90 | -0.031 | 0.084 | 0.244 | 3249 unclassified | Prevotella colorans | Prevotella | Prevotellaceae | Bacteroidales | Bacteroidia | Bacteroidetes |
| Eubacteriales sp. (HG3A.0154) | T90 | -0.031 | 0.085 | 0.245 | 3249 unclassified | unclassified | unclassified | unclassified | Eubacteriales | Clostridia | Firmicutes |
| Blastocystis sp. subtype 4 (HG3A.0446) | ODI | -0.027 | 0.126 | 0.246 | 3249 unclassified | Blastocystis sp. subtype 4 | Blastocystis | Blastocystidae | Opalinata | Bigyra | unclassified |
| Enterocloster citroniae (HG3A.0285) | ODI | 0.027 | 0.127 | 0.246 | 3249 unclassified | Enterocloster  citroniae | Enterocloster | Lachnospiraceae | Eubacteriales | Clostridia | Firmicutes |
| Eubacteriales sp. (HG3A.0288) | AHI | -0.035 | 0.059 | 0.246 | 2909 unclassified | unclassified | unclassified | unclassified | Eubacteriales | Clostridia | Firmicutes |
| Eubacteriales sp. (HG3A.0568) | T90 | -0.03 | 0.086 | 0.248 | 3249 unclassified | unclassified | unclassified | unclassified | Eubacteriales | Clostridia | Firmicutes |
| Clostridium sp. (HG3A.0050) | AHI | 0.035 | 0.061 | 0.249 | 2909 unclassified | unclassified | Clostridium | Clostridiaceae | Eubacteriales | Clostridia | Firmicutes |
| Longicatena caecimuris (HG3A.0571) | AHI | 0.035 | 0.061 | 0.249 | 2909 unclassified | Longicatena  caecimuris | Longicatena | Erysipelotrichacea  e | Erysipelotrichales | Erysipelotrichia | Firmicutes |
| Enterocloster aldenensis (HG3A.0362) | T90 | 0.03 | 0.088 | 0.250 | 3249 unclassified | Enterocloster aldenensis | Enterocloster | Lachnospiraceae | Eubacteriales | Clostridia | Firmicutes |
| Eubacteriales sp. (HG3A.0305) | ODI | -0.027 | 0.13 | 0.250 | 3249 unclassified | unclassified | unclassified | unclassified | Eubacteriales | Clostridia | Firmicutes |
| Eubacteriales sp. (HG3A.0308) | ODI | -0.027 | 0.129 | 0.250 | 3249 unclassified | unclassified | unclassified | unclassified | Eubacteriales | Clostridia | Firmicutes |
| Eubacteriales sp. (HG3A.1332) | T90 | -0.03 | 0.088 | 0.250 | 3249 unclassified | unclassified | unclassified | unclassified | Eubacteriales | Clostridia | Firmicutes |
| Eubacteriales sp. (HG3A.1546) | T90 | -0.03 | 0.088 | 0.250 | 3249 unclassified | unclassified | unclassified | unclassified | Eubacteriales | Clostridia | Firmicutes |
| Clostridiaceae sp. (HG3A.0431) | AHI | 0.035 | 0.062 | 0.252 | 2909 unclassified | unclassified | unclassified | Clostridiaceae | Eubacteriales | Clostridia | Firmicutes |
| Eubacteriales sp. (HG3A.0696) | AHI | -0.035 | 0.062 | 0.252 | 2909 unclassified | unclassified | unclassified | unclassified | Eubacteriales | Clostridia | Firmicutes |
| Eubacteriales sp. (HG3A.0698) | AHI | 0.035 | 0.062 | 0.252 | 2909 unclassified | unclassified | unclassified | unclassified | Eubacteriales | Clostridia | Firmicutes |

| Eubacteriales sp. (HG3A.1445) | ODI | -0.027 | 0.131 | 0.252 | 3249 unclassified | unclassified | unclassified | unclassified | Eubacteriales | Clostridia | Firmicutes |
| --- | --- | --- | --- | --- | --- | --- | --- | --- | --- | --- | --- |
| Oxalobacter formigenes  (HG3A.0552) | ODI | -0.027 | 0.131 | 0.252 | 3249 unclassified | Oxalobacter  formigenes | Oxalobacter | Oxalobacteraceae | Burkholderiales | Betaproteobacte  ria | Proteobacteria |
| Oxalobacter sp. (HG3A.1218) | T90 | -0.03 | 0.089 | 0.252 | 3249 unclassified | unclassified | Oxalobacter | Oxalobacteraceae | Burkholderiales | Betaproteobacte ria | Proteobacteria |
| Eubacteriales sp. (HG3A.0902) | ODI | -0.027 | 0.132 | 0.253 | 3249 unclassified | unclassified | unclassified | unclassified | Eubacteriales | Clostridia | Firmicutes |
| Oscillibacter sp. (HG3A.0243) | ODI | -0.027 | 0.132 | 0.253 | 3249 unclassified | unclassified | Oscillibacter | Oscillospiraceae | Eubacteriales | Clostridia | Firmicutes |
| Barnesiella intestinihominis  (HG3A.0055) | ODI | -0.027 | 0.134 | 0.254 | 3249 unclassified | Barnesiella  intestinihominis | Barnesiella | Barnesiellaceae | Bacteroidales | Bacteroidia | Bacteroidetes |
| Eubacteriales sp. (HG3A.0697) | ODI | -0.027 | 0.133 | 0.254 | 3249 unclassified | unclassified | unclassified | unclassified | Eubacteriales | Clostridia | Firmicutes |
| Eubacteriales sp. (HG3A.1136) | T90 | -0.03 | 0.09 | 0.254 | 3249 unclassified | unclassified | unclassified | unclassified | Eubacteriales | Clostridia | Firmicutes |
| Firmicutes sp. (HG3A.0874) | ODI | -0.027 | 0.134 | 0.254 | 3249 unclassified | unclassified | unclassified | unclassified | unclassified | unclassified | Firmicutes |
| Odoribacter splanchnicus  (HG3A.0041) | ODI | -0.027 | 0.134 | 0.254 | 3249 unclassified | Odoribacter  splanchnicus | Odoribacter | Odoribacteraceae | Bacteroidales | Bacteroidia | Bacteroidetes |
| Oscillospiraceae sp. (HG3A.0060) | ODI | -0.027 | 0.135 | 0.254 | 3249 unclassified | unclassified | unclassified | Oscillospiraceae | Eubacteriales | Clostridia | Firmicutes |
| Acidaminococcus intestini  (HG3A.0407) | AHI | 0.035 | 0.064 | 0.257 | 2909 unclassified | Acidaminococcus  intestini | Acidaminococcus | Acidaminococcace  ae | Acidaminococcales | Negativicutes | Firmicutes |
| Subdoligranulum sp. APC924/74 (HG3A.0015) | ODI | -0.026 | 0.136 | 0.257 | 3249 unclassified | Subdoligranulum sp. APC924/74 | Subdoligranulum | Oscillospiraceae | Eubacteriales | Clostridia | Firmicutes |
| Clostridia sp. (HG3A.1062) | ODI | -0.026 | 0.138 | 0.258 | 3249 unclassified | unclassified | unclassified | unclassified | unclassified | Clostridia | Firmicutes |
| Eubacteriales sp. (HG3A.0736) | ODI | -0.026 | 0.137 | 0.258 | 3249 unclassified | unclassified | unclassified | unclassified | Eubacteriales | Clostridia | Firmicutes |
| Flavonifractor sp. An10 (HG3A.0495) | T90 | -0.03 | 0.092 | 0.259 | 3249 unclassified | Flavonifractor sp.  An10 | Flavonifractor | Oscillospiraceae | Eubacteriales | Clostridia | Firmicutes |
| Oscillospiraceae sp. (HG3A.0060) | T90 | -0.03 | 0.092 | 0.259 | 3249 unclassified | unclassified | unclassified | Oscillospiraceae | Eubacteriales | Clostridia | Firmicutes |
| Eubacteriales sp. (HG3A.0267) | ODI | -0.026 | 0.139 | 0.260 | 3249 unclassified | unclassified | unclassified | unclassified | Eubacteriales | Clostridia | Firmicutes |
| Eubacteriales sp. (HG3A.0345) | ODI | -0.026 | 0.14 | 0.261 | 3249 unclassified | unclassified | unclassified | unclassified | Eubacteriales | Clostridia | Firmicutes |
| Eubacteriales sp. (HG3A.1243) | T90 | -0.03 | 0.093 | 0.261 | 3249 unclassified | unclassified | unclassified | unclassified | Eubacteriales | Clostridia | Firmicutes |
| Anaerobutyricum hallii (HG3A.0112) | AHI | 0.035 | 0.066 | 0.262 | 2909 unclassified | Anaerobutyricum hallii | Anaerobutyricum | Lachnospiraceae | Eubacteriales | Clostridia | Firmicutes |
| Anaeromassilibacillus sp. An250  (HG3A.0169) | ODI | -0.026 | 0.142 | 0.262 | 3249 unclassified | Anaeromassilibacil  lus sp. An250 | Anaeromassilibac  illus | Oscillospiraceae | Eubacteriales | Clostridia | Firmicutes |
| Bacteria sp. (HG3A.0218) | ODI | -0.026 | 0.141 | 0.262 | 3249 unclassified | unclassified | unclassified | unclassified | unclassified | unclassified | unclassified |
| Blautia hydrogenotrophica  (HG3A.0430) | ODI | 0.026 | 0.141 | 0.262 | 3249 unclassified | Blautia  hydrogenotrophica | Blautia | Lachnospiraceae | Eubacteriales | Clostridia | Firmicutes |
| Candidatus Borkfalkiales sp. (HG3A.1284) | AHI | -0.035 | 0.066 | 0.262 | 2909 unclassified | unclassified | unclassified | unclassified | Candidatus Borkfalkiales | Clostridia | Firmicutes |
| Eubacteriales sp. (HG3A.0080) | ODI | 0.026 | 0.141 | 0.262 | 3249 unclassified | unclassified | unclassified | unclassified | Eubacteriales | Clostridia | Firmicutes |

| Eubacteriales sp. (HG3A.0282) | T90 | -0.03 | 0.095 | 0.262 | 3249 unclassified | unclassified | unclassified | unclassified | Eubacteriales | Clostridia | Firmicutes |
| --- | --- | --- | --- | --- | --- | --- | --- | --- | --- | --- | --- |
| Eubacteriales sp. (HG3A.0390) | AHI | -0.034 | 0.069 | 0.262 | 2909 unclassified | unclassified | unclassified | unclassified | Eubacteriales | Clostridia | Firmicutes |
| Eubacteriales sp. (HG3A.0476) | AHI | -0.034 | 0.068 | 0.262 | 2909 unclassified | unclassified | unclassified | unclassified | Eubacteriales | Clostridia | Firmicutes |
| Eubacteriales sp. (HG3A.0486) | T90 | -0.03 | 0.095 | 0.262 | 3249 unclassified | unclassified | unclassified | unclassified | Eubacteriales | Clostridia | Firmicutes |
| Eubacteriales sp. (HG3A.0498) | ODI | 0.026 | 0.142 | 0.262 | 3249 unclassified | unclassified | unclassified | unclassified | Eubacteriales | Clostridia | Firmicutes |
| Eubacteriales sp. (HG3A.0604) | AHI | -0.034 | 0.069 | 0.262 | 2909 unclassified | unclassified | unclassified | unclassified | Eubacteriales | Clostridia | Firmicutes |
| Eubacteriales sp. (HG3A.0618) | ODI | -0.026 | 0.143 | 0.262 | 3249 unclassified | unclassified | unclassified | unclassified | Eubacteriales | Clostridia | Firmicutes |
| Eubacteriales sp. (HG3A.0639) | T90 | -0.03 | 0.096 | 0.262 | 3249 unclassified | unclassified | unclassified | unclassified | Eubacteriales | Clostridia | Firmicutes |
| Eubacteriales sp. (HG3A.0653) | AHI | -0.034 | 0.067 | 0.262 | 2909 unclassified | unclassified | unclassified | unclassified | Eubacteriales | Clostridia | Firmicutes |
| Eubacteriales sp. (HG3A.0731) | AHI | -0.034 | 0.067 | 0.262 | 2909 unclassified | unclassified | unclassified | unclassified | Eubacteriales | Clostridia | Firmicutes |
| Eubacteriales sp. (HG3A.0758) | AHI | -0.034 | 0.068 | 0.262 | 2909 unclassified | unclassified | unclassified | unclassified | Eubacteriales | Clostridia | Firmicutes |
| Eubacteriales sp. (HG3A.0910) | T90 | -0.03 | 0.097 | 0.262 | 3249 unclassified | unclassified | unclassified | unclassified | Eubacteriales | Clostridia | Firmicutes |
| Eubacteriales sp. (HG3A.1126) | AHI | 0.034 | 0.068 | 0.262 | 2909 unclassified | unclassified | unclassified | unclassified | Eubacteriales | Clostridia | Firmicutes |
| Evtepia gabavorous (HG3A.0114) | T90 | 0.03 | 0.096 | 0.262 | 3249 unclassified | Evtepia gabavorous | Evtepia | unclassified | Eubacteriales | Clostridia | Firmicutes |
| Firmicutes sp. (HG3A.0501) | T90 | -0.03 | 0.094 | 0.262 | 3249 unclassified | unclassified | unclassified | unclassified | unclassified | unclassified | Firmicutes |
| Lactobacillus acidophilus  (HG3A.0672) | AHI | -0.035 | 0.067 | 0.262 | 2909 unclassified | Lactobacillus  acidophilus | Lactobacillus | Lactobacillaceae | Lactobacillales | Bacilli | Firmicutes |
| Mogibacterium kristiansenii |  |  |  |  |  | Mogibacterium |  | Clostridiales  Family XIII. |  |  |  |
| (HG3A.0522) | T90 | -0.03 | 0.095 | 0.262 | 3249 unclassified | kristiansenii | Mogibacterium | Incertae Sedis | Eubacteriales | Clostridia | Firmicutes |
| Oscillospiraceae sp. (HG3A.0060) | AHI | -0.034 | 0.069 | 0.262 | 2909 unclassified | unclassified | unclassified | Oscillospiraceae | Eubacteriales | Clostridia | Firmicutes |
| Oscillospiraceae sp. (HG3A.0475) | T90 | -0.03 | 0.094 | 0.262 | 3249 unclassified | unclassified | unclassified | Oscillospiraceae | Eubacteriales | Clostridia | Firmicutes |
| Oscillospiraceae sp. (HG3A.0616) | ODI | -0.026 | 0.142 | 0.262 | 3249 unclassified | unclassified | unclassified | Oscillospiraceae | Eubacteriales | Clostridia | Firmicutes |
| Succinatimonas hippei (HG3A.1322) | ODI | 0.026 | 0.143 | 0.262 | 3249 unclassified | Succinatimonas hippei | Succinatimonas | Succinivibrionacea e | Aeromonadales | Gammaproteob acteria | Proteobacteria |
| Sutterella sp. KLE1602 (HG3A.0228) | AHI | 0.035 | 0.066 | 0.262 | 2909 unclassified | Sutterella sp.  KLE1602 | Sutterella | Sutterellaceae | Burkholderiales | Betaproteobacte  ria | Proteobacteria |
| Eubacteriales sp. (HG3A.0161) | AHI | -0.034 | 0.07 | 0.263 | 2909 unclassified | unclassified | unclassified | unclassified | Eubacteriales | Clostridia | Firmicutes |
| Eubacteriales sp. (HG3A.0377) | AHI | -0.034 | 0.07 | 0.263 | 2909 unclassified | unclassified | unclassified | unclassified | Eubacteriales | Clostridia | Firmicutes |
| Alistipes provencensis (HG3A.0877) | AHI | -0.034 | 0.074 | 0.264 | 2909 unclassified | Alistipes provencensis | Alistipes | Rikenellaceae | Bacteroidales | Bacteroidia | Bacteroidetes |
| Bacteria sp. (HG3A.1543) | AHI | -0.034 | 0.072 | 0.264 | 2909 unclassified | unclassified | unclassified | unclassified | unclassified | unclassified | unclassified |
| Bacteroidales sp. (HG3A.0894) | AHI | -0.034 | 0.075 | 0.264 | 2909 unclassified | unclassified | unclassified | unclassified | Bacteroidales | Bacteroidia | Bacteroidetes |

| Eubacteriales sp. (HG3A.0154) | AHI | -0.033 | 0.075 | 0.264 | 2909 unclassified | unclassified | unclassified | unclassified | Eubacteriales | Clostridia | Firmicutes |
| --- | --- | --- | --- | --- | --- | --- | --- | --- | --- | --- | --- |
| Eubacteriales sp. (HG3A.0229) | AHI | -0.034 | 0.073 | 0.264 | 2909 unclassified | unclassified | unclassified | unclassified | Eubacteriales | Clostridia | Firmicutes |
| Eubacteriales sp. (HG3A.0254) | T90 | 0.029 | 0.098 | 0.264 | 3249 unclassified | unclassified | unclassified | unclassified | Eubacteriales | Clostridia | Firmicutes |
| Eubacteriales sp. (HG3A.0270) | AHI | -0.034 | 0.073 | 0.264 | 2909 unclassified | unclassified | unclassified | unclassified | Eubacteriales | Clostridia | Firmicutes |
| Eubacteriales sp. (HG3A.0419) | AHI | -0.034 | 0.074 | 0.264 | 2909 unclassified | unclassified | unclassified | unclassified | Eubacteriales | Clostridia | Firmicutes |
| Eubacteriales sp. (HG3A.0537) | T90 | -0.029 | 0.098 | 0.264 | 3249 unclassified | unclassified | unclassified | unclassified | Eubacteriales | Clostridia | Firmicutes |
| Eubacteriales sp. (HG3A.0540) | T90 | -0.029 | 0.099 | 0.264 | 3249 unclassified | unclassified | unclassified | unclassified | Eubacteriales | Clostridia | Firmicutes |
| Eubacteriales sp. (HG3A.0577) | AHI | -0.033 | 0.075 | 0.264 | 2909 unclassified | unclassified | unclassified | unclassified | Eubacteriales | Clostridia | Firmicutes |
| Eubacteriales sp. (HG3A.0613) | T90 | -0.029 | 0.099 | 0.264 | 3249 unclassified | unclassified | unclassified | unclassified | Eubacteriales | Clostridia | Firmicutes |
| Eubacteriales sp. (HG3A.0637) | AHI | -0.034 | 0.075 | 0.264 | 2909 unclassified | unclassified | unclassified | unclassified | Eubacteriales | Clostridia | Firmicutes |
| Eubacteriales sp. (HG3A.0757) | AHI | -0.033 | 0.076 | 0.264 | 2909 unclassified | unclassified | unclassified | unclassified | Eubacteriales | Clostridia | Firmicutes |
| Eubacteriales sp. (HG3A.0792) | T90 | -0.029 | 0.1 | 0.264 | 3249 unclassified | unclassified | unclassified | unclassified | Eubacteriales | Clostridia | Firmicutes |
| Eubacteriales sp. (HG3A.1078) | T90 | -0.029 | 0.099 | 0.264 | 3249 unclassified | unclassified | unclassified | unclassified | Eubacteriales | Clostridia | Firmicutes |
| Firmicutes sp. (HG3A.1195) | AHI | -0.034 | 0.073 | 0.264 | 2909 unclassified | unclassified | unclassified | unclassified | unclassified | unclassified | Firmicutes |
| Lachnospiraceae sp. (HG3A.0257) | AHI | -0.033 | 0.075 | 0.264 | 2909 unclassified | unclassified | unclassified | Lachnospiraceae | Eubacteriales | Clostridia | Firmicutes |
| Oscillibacter sp. (HG3A.0243) | T90 | -0.029 | 0.098 | 0.264 | 3249 unclassified | unclassified | Oscillibacter | Oscillospiraceae | Eubacteriales | Clostridia | Firmicutes |
| Oscillibacter sp. (HG3A.0245) | AHI | -0.034 | 0.071 | 0.264 | 2909 unclassified | unclassified | Oscillibacter | Oscillospiraceae | Eubacteriales | Clostridia | Firmicutes |
| Oscillospiraceae sp. (HG3A.0210) | AHI | -0.034 | 0.074 | 0.264 | 2909 unclassified | unclassified | unclassified | Oscillospiraceae | Eubacteriales | Clostridia | Firmicutes |
| Sutterellaceae sp. (HG3A.1122) | AHI | 0.034 | 0.075 | 0.264 | 2909 unclassified | unclassified | unclassified | Sutterellaceae | Burkholderiales | Betaproteobacte  ria | Proteobacteria |
| Victivallis vadensis (HG3A.0689) | AHI | -0.034 | 0.072 | 0.264 | 2909 unclassified | Victivallis vadensis | Victivallis | Victivallaceae | Victivallales | Lentisphaeria | Lentisphaerae |
| Eubacteriales sp. (HG3A.0696) | T90 | -0.029 | 0.1 | 0.265 | 3249 unclassified | unclassified | unclassified | unclassified | Eubacteriales | Clostridia | Firmicutes |
| Tyzzerella nexilis (HG3A.0574) | T90 | 0.029 | 0.101 | 0.265 | 3249 unclassified | Tyzzerella nexilis | Tyzzerella | Lachnospiraceae | Eubacteriales | Clostridia | Firmicutes |
| Clostridia sp. (HG3A.0512) | ODI | -0.026 | 0.145 | 0.266 | 3249 unclassified | unclassified | unclassified | unclassified | unclassified | Clostridia | Firmicutes |
| Gemella morbillorum (HG3A.1782) | T90 | 0.029 | 0.101 | 0.266 | 3249 unclassified | Gemella morbillorum | Gemella | unclassified | Bacillales | Bacilli | Firmicutes |
| Clostridia sp. (HG3A.1141) | AHI | -0.033 | 0.077 | 0.267 | 2909 unclassified | unclassified | unclassified | unclassified | unclassified | Clostridia | Firmicutes |
| Alistipes senegalensis (HG3A.0141) | AHI | -0.033 | 0.078 | 0.268 | 2909 unclassified | Alistipes senegalensis | Alistipes | Rikenellaceae | Bacteroidales | Bacteroidia | Bacteroidetes |
| Eubacterium sp. AF16-48  (HG3A.0219) | AHI | -0.033 | 0.079 | 0.268 | 2909 unclassified | Eubacterium sp.  AF16-48 | Eubacterium | Eubacteriaceae | Eubacteriales | Clostridia | Firmicutes |

| Mogibacterium kristiansenii (HG3A.0522) | AHI | 0.033 | 0.078 | 0.268 | 2909 unclassified | Mogibacterium kristiansenii | Mogibacterium | Clostridiales  Family XIII. Incertae Sedis | Eubacteriales | Clostridia | Firmicutes |
| --- | --- | --- | --- | --- | --- | --- | --- | --- | --- | --- | --- |
| Oscillospiraceae sp. (HG3A.0382) | T90 | -0.029 | 0.103 | 0.268 | 3249 unclassified | unclassified | unclassified | Oscillospiraceae | Eubacteriales | Clostridia | Firmicutes |
| Oscillospiraceae sp. (HG3A.1173) | AHI | -0.033 | 0.078 | 0.268 | 2909 unclassified | unclassified | unclassified | Oscillospiraceae | Eubacteriales | Clostridia | Firmicutes |
| Clostridia sp. (HG3A.1038) | ODI | -0.026 | 0.147 | 0.269 | 3249 unclassified | unclassified | unclassified | unclassified | unclassified | Clostridia | Firmicutes |
| Coprococcus eutactus (HG3A.0155) | AHI | -0.033 | 0.079 | 0.269 | 2909 unclassified | Coprococcus eutactus | Coprococcus | Lachnospiraceae | Eubacteriales | Clostridia | Firmicutes |
| Oscillospiraceae sp. (HG3A.0384) | AHI | -0.033 | 0.079 | 0.269 | 2909 unclassified | unclassified | unclassified | Oscillospiraceae | Eubacteriales | Clostridia | Firmicutes |
| Eubacteriales sp. (HG3A.1086) | AHI | -0.033 | 0.08 | 0.270 | 2909 unclassified | unclassified | unclassified | unclassified | Eubacteriales | Clostridia | Firmicutes |
| Eubacteriales sp. (HG3A.1154) | AHI | -0.033 | 0.081 | 0.270 | 2909 unclassified | unclassified | unclassified | unclassified | Eubacteriales | Clostridia | Firmicutes |
| Blastocystis sp. subtype 4 (HG3A.0446) | AHI | -0.033 | 0.082 | 0.273 | 2909 unclassified | Blastocystis sp. subtype 4 | Blastocystis | Blastocystidae | Opalinata | Bigyra | unclassified |
| Firmicutes sp. (HG3A.0641) | AHI | -0.033 | 0.082 | 0.273 | 2909 unclassified | unclassified | unclassified | unclassified | unclassified | unclassified | Firmicutes |
| Clostridia sp. (HG3A.0276) | T90 | -0.029 | 0.105 | 0.274 | 3249 unclassified | unclassified | unclassified | unclassified | unclassified | Clostridia | Firmicutes |
| Eubacteriales sp. (HG3A.0188) | ODI | -0.026 | 0.151 | 0.274 | 3249 unclassified | unclassified | unclassified | unclassified | Eubacteriales | Clostridia | Firmicutes |
| Eubacteriales sp. (HG3A.0697) | T90 | -0.029 | 0.106 | 0.274 | 3249 unclassified | unclassified | unclassified | unclassified | Eubacteriales | Clostridia | Firmicutes |
| Lachnospiraceae sp. (HG3A.0393) | T90 | 0.029 | 0.106 | 0.274 | 3249 unclassified | unclassified | unclassified | Lachnospiraceae | Eubacteriales | Clostridia | Firmicutes |
| Eubacteriales sp. (HG3A.0476) | ODI | -0.026 | 0.151 | 0.275 | 3249 unclassified | unclassified | unclassified | unclassified | Eubacteriales | Clostridia | Firmicutes |
| Eubacterium sp. AM49-13BH  (HG3A.0251) | AHI | -0.033 | 0.083 | 0.276 | 2909 unclassified | Eubacterium sp.  AM49-13BH | Eubacterium | Eubacteriaceae | Eubacteriales | Clostridia | Firmicutes |
| Firmicutes sp. (HG3A.0436) | AHI | -0.033 | 0.084 | 0.276 | 2909 unclassified | unclassified | unclassified | unclassified | unclassified | unclassified | Firmicutes |
| Oscillospiraceae sp. (HG3A.0507) | ODI | -0.025 | 0.152 | 0.276 | 3249 unclassified | unclassified | unclassified | Oscillospiraceae | Eubacteriales | Clostridia | Firmicutes |
| Firmicutes sp. (HG3A.0526) | ODI | -0.025 | 0.153 | 0.277 | 3249 unclassified | unclassified | unclassified | unclassified | unclassified | unclassified | Firmicutes |
| Eubacteriales sp. (HG3A.0373) | ODI | -0.025 | 0.154 | 0.278 | 3249 unclassified | unclassified | unclassified | unclassified | Eubacteriales | Clostridia | Firmicutes |
| Eubacteriales sp. (HG3A.0967) | ODI | -0.025 | 0.155 | 0.278 | 3249 unclassified | unclassified | unclassified | unclassified | Eubacteriales | Clostridia | Firmicutes |
| Oscillospiraceae sp. (HG3A.0461) | ODI | -0.025 | 0.155 | 0.278 | 3249 unclassified | unclassified | unclassified | Oscillospiraceae | Eubacteriales | Clostridia | Firmicutes |
| Oscillospiraceae sp. (HG3A.0805) | ODI | -0.025 | 0.155 | 0.278 | 3249 unclassified | unclassified | unclassified | Oscillospiraceae | Eubacteriales | Clostridia | Firmicutes |

| Ruminococcus sp. (HG3A.0337) | ODI | -0.025 | 0.156 | 0.279 | 3249 unclassified | unclassified | Ruminococcus | Oscillospiraceae | Eubacteriales | Clostridia | Firmicutes |
| --- | --- | --- | --- | --- | --- | --- | --- | --- | --- | --- | --- |
| Eubacteriales sp. (HG3A.0312) | T90 | -0.028 | 0.109 | 0.281 | 3249 unclassified | unclassified | unclassified | unclassified | Eubacteriales | Clostridia | Firmicutes |
| Eubacteriales sp. (HG3A.0363) | T90 | -0.028 | 0.109 | 0.281 | 3249 unclassified | unclassified | unclassified | unclassified | Eubacteriales | Clostridia | Firmicutes |
| Eubacteriales sp. (HG3A.0496) | T90 | -0.028 | 0.11 | 0.281 | 3249 unclassified | unclassified | unclassified | unclassified | Eubacteriales | Clostridia | Firmicutes |
| Eubacteriales sp. (HG3A.0807) | T90 | -0.028 | 0.111 | 0.281 | 3249 unclassified | unclassified | unclassified | unclassified | Eubacteriales | Clostridia | Firmicutes |
| Eubacteriales sp. (HG3A.0908) | T90 | -0.028 | 0.11 | 0.281 | 3249 unclassified | unclassified | unclassified | unclassified | Eubacteriales | Clostridia | Firmicutes |
| Eubacteriales sp. (HG3A.1103) | T90 | -0.028 | 0.11 | 0.281 | 3249 unclassified | unclassified | unclassified | unclassified | Eubacteriales | Clostridia | Firmicutes |
| Anaerotruncus colihominis (HG3A.0307) | T90 | 0.028 | 0.112 | 0.282 | 3249 unclassified | Anaerotruncus colihominis | Anaerotruncus | Oscillospiraceae | Eubacteriales | Clostridia | Firmicutes |
| Eubacteriales sp. (HG3A.0588) | T90 | -0.028 | 0.112 | 0.282 | 3249 unclassified | unclassified | unclassified | unclassified | Eubacteriales | Clostridia | Firmicutes |
| Latilactobacillus sakei subsp. sakei |  |  |  |  | Latilactobacillus  sakei subsp. | Latilactobacillus |  |  |  |  |  |
| (HG3A.0836) | T90 | 0.028 | 0.112 | 0.282 | 3249 sakei | sakei | Latilactobacillus | Lactobacillaceae | Lactobacillales | Bacilli | Firmicutes |
| Eubacteriales sp. (HG3A.0604) | ODI | -0.025 | 0.159 | 0.283 | 3249 unclassified | unclassified | unclassified | unclassified | Eubacteriales | Clostridia | Firmicutes |
| Clostridia sp. (HG3A.1058) | ODI | -0.025 | 0.16 | 0.284 | 3249 unclassified | unclassified | unclassified | unclassified | unclassified | Clostridia | Firmicutes |
| Eubacteriales sp. (HG3A.0192) | ODI | -0.025 | 0.16 | 0.284 | 3249 unclassified | unclassified | unclassified | unclassified | Eubacteriales | Clostridia | Firmicutes |
| Eubacteriales sp. (HG3A.0547) | T90 | -0.028 | 0.113 | 0.284 | 3249 unclassified | unclassified | unclassified | unclassified | Eubacteriales | Clostridia | Firmicutes |
| Clostridia sp. (HG3A.0276) | ODI | -0.025 | 0.161 | 0.285 | 3249 unclassified | unclassified | unclassified | unclassified | unclassified | Clostridia | Firmicutes |
| Eggerthellales sp. (HG3A.0848) | ODI | -0.025 | 0.161 | 0.285 | 3249 unclassified | unclassified | unclassified | unclassified | Eggerthellales | Coriobacteriia | Actinobacteria |
| Eubacteriales sp. (HG3A.1422) | T90 | -0.028 | 0.114 | 0.285 | 3249 unclassified | unclassified | unclassified | unclassified | Eubacteriales | Clostridia | Firmicutes |
| Eubacteriales sp. (HG3A.0363) | ODI | -0.025 | 0.162 | 0.286 | 3249 unclassified | unclassified | unclassified | unclassified | Eubacteriales | Clostridia | Firmicutes |
| Clostridia sp. (HG3A.0512) | T90 | -0.028 | 0.116 | 0.288 | 3249 unclassified | unclassified | unclassified | unclassified | unclassified | Clostridia | Firmicutes |
| Clostridia sp. (HG3A.0783) | ODI | -0.025 | 0.164 | 0.288 | 3249 unclassified | unclassified | unclassified | unclassified | unclassified | Clostridia | Firmicutes |
| Clostridia sp. (HG3A.1298) | ODI | -0.025 | 0.164 | 0.288 | 3249 unclassified | unclassified | unclassified | unclassified | unclassified | Clostridia | Firmicutes |
| Eubacteriales sp. (HG3A.0273) | T90 | -0.028 | 0.115 | 0.288 | 3249 unclassified | unclassified | unclassified | unclassified | Eubacteriales | Clostridia | Firmicutes |
| Oxalobacter sp. (HG3A.1218) | ODI | -0.025 | 0.164 | 0.288 | 3249 unclassified | unclassified | Oxalobacter | Oxalobacteraceae | Burkholderiales | Betaproteobacte  ria | Proteobacteria |
| Bacteria sp. (HG3A.0839) | ODI | -0.025 | 0.165 | 0.289 | 3249 unclassified | unclassified | unclassified | unclassified | unclassified | unclassified | unclassified |
| Bacteroides caccae (HG3A.0066) | ODI | -0.025 | 0.166 | 0.289 | 3249 unclassified | Bacteroides caccae | Bacteroides | Bacteroidaceae | Bacteroidales | Bacteroidia | Bacteroidetes |
| Eggerthella lenta (HG3A.0225) | T90 | 0.028 | 0.117 | 0.290 | 3249 unclassified | Eggerthella lenta | Eggerthella | Eggerthellaceae | Eggerthellales | Coriobacteriia | Actinobacteria |

| Oscillospiraceae sp. (HG3A.0256) | ODI | 0.025 | 0.168 | 0.290 | 3249 unclassified | unclassified | unclassified | Oscillospiraceae | Eubacteriales | Clostridia | Firmicutes |
| --- | --- | --- | --- | --- | --- | --- | --- | --- | --- | --- | --- |
| Proteobacteria sp. (HG3A.0327) | ODI | -0.025 | 0.167 | 0.290 | 3249 unclassified | unclassified | unclassified | unclassified | unclassified | unclassified | Proteobacteria |
| Roseburia faecis (HG3A.0058) | ODI | 0.025 | 0.168 | 0.290 | 3249 unclassified | Roseburia faecis | Roseburia | Lachnospiraceae | Eubacteriales | Clostridia | Firmicutes |
| Clostridia sp. (HG3A.1254) | ODI | -0.024 | 0.17 | 0.292 | 3249 unclassified | unclassified | unclassified | unclassified | unclassified | Clostridia | Firmicutes |
| Eubacteriales sp. (HG3A.1167) | ODI | -0.024 | 0.169 | 0.292 | 3249 unclassified | unclassified | unclassified | unclassified | Eubacteriales | Clostridia | Firmicutes |
| Oscillospiraceae sp. (HG3A.0475) | ODI | -0.024 | 0.169 | 0.292 | 3249 unclassified | unclassified | unclassified | Oscillospiraceae | Eubacteriales | Clostridia | Firmicutes |
| Lachnospiraceae sp. (HG3A.1155) | ODI | -0.024 | 0.171 | 0.293 | 3249 unclassified | unclassified | unclassified | Lachnospiraceae | Eubacteriales | Clostridia | Firmicutes |
| Clostridia sp. (HG3A.0752) | T90 | -0.028 | 0.119 | 0.294 | 3249 unclassified | unclassified | unclassified | unclassified | unclassified | Clostridia | Firmicutes |
| Akkermansia sp. BIOML-A59  (HG3A.0800) | AHI | -0.032 | 0.09 | 0.295 | 2909 unclassified | Akkermansia sp.  BIOML-A59 | Akkermansia | Akkermansiaceae | Verrucomicrobiale  s | Verrucomicrobi  ae | Verrucomicrobia |
| Bifidobacterium animalis subsp. |  |  |  |  | Bifidobacterium  animalis subsp. | Bifidobacterium |  |  |  |  |  |
| lactis (HG3A.0513) | T90 | -0.028 | 0.12 | 0.295 | 3249 lactis | animalis | Bifidobacterium | Bifidobacteriaceae | Bifidobacteriales | Actinomycetia | Actinobacteria |
| Clostridia sp. (HG3A.0706) | ODI | -0.024 | 0.173 | 0.295 | 3249 unclassified | unclassified | unclassified | unclassified | unclassified | Clostridia | Firmicutes |
| Coprococcus sp. AF21-14LB (HG3A.1047) | T90 | 0.028 | 0.12 | 0.295 | 3249 unclassified | Coprococcus sp. AF21-14LB | Coprococcus | Lachnospiraceae | Eubacteriales | Clostridia | Firmicutes |
| Coprococcus sp. AM27-12LB  (HG3A.0687) | ODI | 0.024 | 0.172 | 0.295 | 3249 unclassified | Coprococcus sp.  AM27-12LB | Coprococcus | Lachnospiraceae | Eubacteriales | Clostridia | Firmicutes |
| Desulfovibrionales sp. (HG3A.0266) | ODI | -0.024 | 0.176 | 0.295 | 3249 unclassified | unclassified | unclassified | unclassified | Desulfovibrionales | Deltaproteobact eria | Proteobacteria |
| Eggerthella lenta (HG3A.0225) | AHI | 0.032 | 0.091 | 0.295 | 2909 unclassified | Eggerthella lenta | Eggerthella | Eggerthellaceae | Eggerthellales | Coriobacteriia | Actinobacteria |
| Eggerthellales sp. (HG3A.0174) | AHI | -0.032 | 0.092 | 0.295 | 2909 unclassified | unclassified | unclassified | unclassified | Eggerthellales | Coriobacteriia | Actinobacteria |
| Eubacteriales sp. (HG3A.0116) | ODI | -0.024 | 0.174 | 0.295 | 3249 unclassified | unclassified | unclassified | unclassified | Eubacteriales | Clostridia | Firmicutes |
| Eubacteriales sp. (HG3A.0215) | AHI | -0.032 | 0.091 | 0.295 | 2909 unclassified | unclassified | unclassified | unclassified | Eubacteriales | Clostridia | Firmicutes |
| Eubacteriales sp. (HG3A.0406) | T90 | -0.028 | 0.121 | 0.295 | 3249 unclassified | unclassified | unclassified | unclassified | Eubacteriales | Clostridia | Firmicutes |
| Eubacteriales sp. (HG3A.0409) | AHI | -0.032 | 0.091 | 0.295 | 2909 unclassified | unclassified | unclassified | unclassified | Eubacteriales | Clostridia | Firmicutes |
| Eubacteriales sp. (HG3A.0474) | ODI | -0.024 | 0.176 | 0.295 | 3249 unclassified | unclassified | unclassified | unclassified | Eubacteriales | Clostridia | Firmicutes |
| Eubacteriales sp. (HG3A.0496) | ODI | -0.024 | 0.175 | 0.295 | 3249 unclassified | unclassified | unclassified | unclassified | Eubacteriales | Clostridia | Firmicutes |
| Eubacteriales sp. (HG3A.0544) | T90 | -0.028 | 0.12 | 0.295 | 3249 unclassified | unclassified | unclassified | unclassified | Eubacteriales | Clostridia | Firmicutes |
| Eubacteriales sp. (HG3A.0628) | ODI | -0.024 | 0.174 | 0.295 | 3249 unclassified | unclassified | unclassified | unclassified | Eubacteriales | Clostridia | Firmicutes |
| Eubacteriales sp. (HG3A.0656) | T90 | -0.028 | 0.122 | 0.295 | 3249 unclassified | unclassified | unclassified | unclassified | Eubacteriales | Clostridia | Firmicutes |
| Eubacteriales sp. (HG3A.0759) | AHI | -0.032 | 0.092 | 0.295 | 2909 unclassified | unclassified | unclassified | unclassified | Eubacteriales | Clostridia | Firmicutes |
| Eubacteriales sp. (HG3A.1109) | T90 | -0.027 | 0.122 | 0.295 | 3249 unclassified | unclassified | unclassified | unclassified | Eubacteriales | Clostridia | Firmicutes |
| Firmicutes sp. (HG3A.1345) | T90 | -0.028 | 0.121 | 0.295 | 3249 unclassified | unclassified | unclassified | unclassified | unclassified | unclassified | Firmicutes |

| Lachnospiraceae sp. (HG3A.0748) | AHI | -0.032 | 0.09 | 0.295 | 2909 unclassified | unclassified | unclassified | Lachnospiraceae | Eubacteriales | Clostridia | Firmicutes |
| --- | --- | --- | --- | --- | --- | --- | --- | --- | --- | --- | --- |
| Parabacteroides goldsteinii (HG3A.0279) | ODI | -0.024 | 0.175 | 0.295 | 3249 unclassified | Parabacteroides goldsteinii | Parabacteroides | Tannerellaceae | Bacteroidales | Bacteroidia | Bacteroidetes |
| Phocaeicola plebeius (HG3A.0423) | ODI | -0.024 | 0.174 | 0.295 | 3249 unclassified | Phocaeicola  plebeius | Phocaeicola | unclassified | Bacteroidales | Bacteroidia | Bacteroidetes |
| Streptococcus parasanguinis (HG3A.0117) | ODI | 0.024 | 0.176 | 0.295 | 3249 unclassified | Streptococcus parasanguinis | Streptococcus | Streptococcaceae | Lactobacillales | Bacilli | Firmicutes |
| Dorea formicigenerans (HG3A.0006) | AHI | 0.032 | 0.093 | 0.296 | 2909 unclassified | Dorea  formicigenerans | Dorea | Lachnospiraceae | Eubacteriales | Clostridia | Firmicutes |
| Eubacteriales sp. (HG3A.0589) | ODI | -0.024 | 0.176 | 0.296 | 3249 unclassified | unclassified | unclassified | unclassified | Eubacteriales | Clostridia | Firmicutes |
| Eubacteriales sp. (HG3A.0593) | ODI | -0.024 | 0.177 | 0.296 | 3249 unclassified | unclassified | unclassified | unclassified | Eubacteriales | Clostridia | Firmicutes |
| Prevotella sp. (HG3A.1040) | ODI | 0.024 | 0.177 | 0.296 | 3249 unclassified | unclassified | Prevotella | Prevotellaceae | Bacteroidales | Bacteroidia | Bacteroidetes |
| Clostridia sp. (HG3A.1010) | T90 | -0.027 | 0.124 | 0.297 | 3249 unclassified | unclassified | unclassified | unclassified | unclassified | Clostridia | Firmicutes |
| Clostridia sp. (HG3A.1039) | AHI | -0.032 | 0.094 | 0.297 | 2909 unclassified | unclassified | unclassified | unclassified | unclassified | Clostridia | Firmicutes |
| Eubacteriales sp. (HG3A.0116) | AHI | -0.032 | 0.094 | 0.297 | 2909 unclassified | unclassified | unclassified | unclassified | Eubacteriales | Clostridia | Firmicutes |
| Eubacteriales sp. (HG3A.0323) | T90 | -0.027 | 0.123 | 0.297 | 3249 unclassified | unclassified | unclassified | unclassified | Eubacteriales | Clostridia | Firmicutes |
| Eubacteriales sp. (HG3A.0371) | T90 | -0.027 | 0.124 | 0.297 | 3249 unclassified | unclassified | unclassified | unclassified | Eubacteriales | Clostridia | Firmicutes |
| Eubacteriales sp. (HG3A.0418) | AHI | -0.031 | 0.094 | 0.297 | 2909 unclassified | unclassified | unclassified | unclassified | Eubacteriales | Clostridia | Firmicutes |
| Eubacteriales sp. (HG3A.0589) | T90 | -0.027 | 0.124 | 0.297 | 3249 unclassified | unclassified | unclassified | unclassified | Eubacteriales | Clostridia | Firmicutes |
| Lachnospiraceae sp. (HG3A.0127) | T90 | -0.027 | 0.125 | 0.297 | 3249 unclassified | unclassified | unclassified | Lachnospiraceae | Eubacteriales | Clostridia | Firmicutes |
| Clostridia sp. (HG3A.1128) | ODI | -0.024 | 0.179 | 0.298 | 3249 unclassified | unclassified | unclassified | unclassified | unclassified | Clostridia | Firmicutes |
| Eubacteriales sp. (HG3A.0518) | ODI | -0.024 | 0.18 | 0.298 | 3249 unclassified | unclassified | unclassified | unclassified | Eubacteriales | Clostridia | Firmicutes |
| Eubacteriales sp. (HG3A.0694) | ODI | -0.024 | 0.18 | 0.298 | 3249 unclassified | unclassified | unclassified | unclassified | Eubacteriales | Clostridia | Firmicutes |
| Oxalobacter sp. (HG3A.1097) | ODI | 0.024 | 0.18 | 0.298 | 3249 unclassified | unclassified | Oxalobacter | Oxalobacteraceae | Burkholderiales | Betaproteobacte ria | Proteobacteria |
| Clostridia sp. (HG3A.1010) | AHI | -0.031 | 0.096 | 0.299 | 2909 unclassified | unclassified | unclassified | unclassified | unclassified | Clostridia | Firmicutes |
| Subdoligranulum sp. APC924/74 (HG3A.0015) | AHI | -0.031 | 0.096 | 0.299 | 2909 unclassified | Subdoligranulum sp. APC924/74 | Subdoligranulum | Oscillospiraceae | Eubacteriales | Clostridia | Firmicutes |
| Erysipelotrichales sp. (HG3A.0303) | AHI | -0.031 | 0.096 | 0.300 | 2909 unclassified | unclassified | unclassified | unclassified | Erysipelotrichales | Erysipelotrichia | Firmicutes |
| Eubacteriales sp. (HG3A.1473) | T90 | -0.027 | 0.127 | 0.301 | 3249 unclassified | unclassified | unclassified | unclassified | Eubacteriales | Clostridia | Firmicutes |

| Clostridia sp. (HG3A.0931) | AHI | -0.031 | 0.098 | 0.303 | 2909 unclassified | unclassified | unclassified | unclassified | unclassified | Clostridia | Firmicutes |
| --- | --- | --- | --- | --- | --- | --- | --- | --- | --- | --- | --- |
| Victivallales sp. (HG3A.0824) | ODI | -0.024 | 0.183 | 0.303 | 3249 unclassified | unclassified | unclassified | unclassified | Victivallales | Lentisphaeria | Lentisphaerae |
| Eubacteriales sp. (HG3A.0731) | T90 | -0.027 | 0.128 | 0.304 | 3249 unclassified | unclassified | unclassified | unclassified | Eubacteriales | Clostridia | Firmicutes |
| Eubacteriales sp. (HG3A.0832) | ODI | -0.024 | 0.186 | 0.306 | 3249 unclassified | unclassified | unclassified | unclassified | Eubacteriales | Clostridia | Firmicutes |
| Eubacteriales sp. (HG3A.1250) | AHI | -0.031 | 0.099 | 0.306 | 2909 unclassified | unclassified | unclassified | unclassified | Eubacteriales | Clostridia | Firmicutes |
| Clostridium sp. TM06-18 (HG3A.0048) | T90 | 0.027 | 0.13 | 0.307 | 3249 unclassified | Clostridium sp. TM06-18 | Clostridium | Clostridiaceae | Eubacteriales | Clostridia | Firmicutes |
| Eubacteriales sp. (HG3A.0163) | T90 | -0.027 | 0.131 | 0.307 | 3249 unclassified | unclassified | unclassified | unclassified | Eubacteriales | Clostridia | Firmicutes |
| Eubacteriales sp. (HG3A.0342) | T90 | -0.027 | 0.131 | 0.307 | 3249 unclassified | unclassified | unclassified | unclassified | Eubacteriales | Clostridia | Firmicutes |
| Clostridia sp. (HG3A.0918) | T90 | -0.027 | 0.133 | 0.308 | 3249 unclassified | unclassified | unclassified | unclassified | unclassified | Clostridia | Firmicutes |
| Eubacteriales sp. (HG3A.0653) | T90 | -0.027 | 0.133 | 0.308 | 3249 unclassified | unclassified | unclassified | unclassified | Eubacteriales | Clostridia | Firmicutes |
| Eubacteriales sp. (HG3A.0956) | T90 | -0.027 | 0.132 | 0.308 | 3249 unclassified | unclassified | unclassified | unclassified | Eubacteriales | Clostridia | Firmicutes |
| Firmicutes sp. (HG3A.0587) | T90 | -0.027 | 0.132 | 0.308 | 3249 unclassified | unclassified | unclassified | unclassified | unclassified | unclassified | Firmicutes |
| Clostridium sp. TM06-18  (HG3A.0048) | AHI | 0.031 | 0.101 | 0.309 | 2909 unclassified | Clostridium sp.  TM06-18 | Clostridium | Clostridiaceae | Eubacteriales | Clostridia | Firmicutes |
| Eubacteriales sp. (HG3A.0087) | AHI | -0.031 | 0.1 | 0.309 | 2909 unclassified | unclassified | unclassified | unclassified | Eubacteriales | Clostridia | Firmicutes |
| Eubacteriales sp. (HG3A.1063) | ODI | -0.023 | 0.188 | 0.310 | 3249 unclassified | unclassified | unclassified | unclassified | Eubacteriales | Clostridia | Firmicutes |
| Oscillospiraceae sp. (HG3A.0774) | ODI | -0.023 | 0.189 | 0.310 | 3249 unclassified | unclassified | unclassified | Oscillospiraceae | Eubacteriales | Clostridia | Firmicutes |
| Clostridia sp. (HG3A.1157) | ODI | -0.023 | 0.19 | 0.311 | 3249 unclassified | unclassified | unclassified | unclassified | unclassified | Clostridia | Firmicutes |
| Dorea sp. AF36-15AT (HG3A.0052) | AHI | 0.031 | 0.102 | 0.311 | 2909 unclassified | Dorea sp. AF36- 15AT | Dorea | Lachnospiraceae | Eubacteriales | Clostridia | Firmicutes |
| Prevotella sp. (HG3A.1040) | AHI | 0.031 | 0.102 | 0.311 | 2909 unclassified | unclassified | Prevotella | Prevotellaceae | Bacteroidales | Bacteroidia | Bacteroidetes |
| Clostridia sp. (HG3A.1410) | ODI | -0.023 | 0.191 | 0.312 | 3249 unclassified | unclassified | unclassified | unclassified | unclassified | Clostridia | Firmicutes |
| Clostridia sp. (HG3A.1247) | ODI | -0.023 | 0.192 | 0.314 | 3249 unclassified | unclassified | unclassified | unclassified | unclassified | Clostridia | Firmicutes |
| Eubacteriales sp. (HG3A.0087) | T90 | 0.026 | 0.137 | 0.314 | 3249 unclassified | unclassified | unclassified | unclassified | Eubacteriales | Clostridia | Firmicutes |
| Eubacteriales sp. (HG3A.0305) | T90 | -0.027 | 0.136 | 0.314 | 3249 unclassified | unclassified | unclassified | unclassified | Eubacteriales | Clostridia | Firmicutes |
| Eubacteriales sp. (HG3A.0447) | ODI | -0.023 | 0.193 | 0.314 | 3249 unclassified | unclassified | unclassified | unclassified | Eubacteriales | Clostridia | Firmicutes |
| Firmicutes sp. (HG3A.0923) | T90 | -0.026 | 0.137 | 0.314 | 3249 unclassified | unclassified | unclassified | unclassified | unclassified | unclassified | Firmicutes |
| Holdemanella sp. (HG3A.0366) | T90 | 0.026 | 0.137 | 0.314 | 3249 unclassified | unclassified | Holdemanella | Erysipelotrichacea e | Erysipelotrichales | Erysipelotrichia | Firmicutes |
| [Clostridium] symbiosum  (HG3A.0370) | AHI | 0.031 | 0.104 | 0.315 | 2909 unclassified | [Clostridium]  symbiosum | Lachnoclostridiu  m | Lachnospiraceae | Eubacteriales | Clostridia | Firmicutes |

| Clostridia sp. (HG3A.0661) | AHI | -0.031 | 0.105 | 0.315 | 2909 unclassified | unclassified | unclassified | unclassified | unclassified | Clostridia | Firmicutes |
| --- | --- | --- | --- | --- | --- | --- | --- | --- | --- | --- | --- |
| Clostridiaceae sp. (HG3A.0608) | AHI | -0.031 | 0.105 | 0.315 | 2909 unclassified | unclassified | unclassified | Clostridiaceae | Eubacteriales | Clostridia | Firmicutes |
| Eubacteriales sp. (HG3A.0123) | AHI | 0.03 | 0.105 | 0.316 | 2909 unclassified | unclassified | unclassified | unclassified | Eubacteriales | Clostridia | Firmicutes |
| Clostridia sp. (HG3A.0645) | AHI | -0.03 | 0.107 | 0.319 | 2909 unclassified | unclassified | unclassified | unclassified | unclassified | Clostridia | Firmicutes |
| Clostridia sp. (HG3A.1139) | ODI | -0.023 | 0.196 | 0.319 | 3249 unclassified | unclassified | unclassified | unclassified | unclassified | Clostridia | Firmicutes |
| Eubacteriales sp. (HG3A.0092) | AHI | -0.03 | 0.108 | 0.319 | 2909 unclassified | unclassified | unclassified | unclassified | Eubacteriales | Clostridia | Firmicutes |
| Eubacteriales sp. (HG3A.0624) | AHI | -0.03 | 0.109 | 0.319 | 2909 unclassified | unclassified | unclassified | unclassified | Eubacteriales | Clostridia | Firmicutes |
| Eubacteriales sp. (HG3A.0856) | AHI | -0.03 | 0.108 | 0.319 | 2909 unclassified | unclassified | unclassified | unclassified | Eubacteriales | Clostridia | Firmicutes |
| Firmicutes sp. (HG3A.1085) | AHI | -0.03 | 0.108 | 0.319 | 2909 unclassified | unclassified | unclassified | unclassified | unclassified | unclassified | Firmicutes |
| Eisenbergiella tayi (HG3A.0355) | ODI | -0.023 | 0.198 | 0.321 | 3249 unclassified | Eisenbergiella tayi | Eisenbergiella | Lachnospiraceae | Eubacteriales | Clostridia | Firmicutes |
| Eubacteriales sp. (HG3A.0102) | T90 | -0.026 | 0.141 | 0.321 | 3249 unclassified | unclassified | unclassified | unclassified | Eubacteriales | Clostridia | Firmicutes |
| Eubacteriales sp. (HG3A.0178) | T90 | -0.026 | 0.142 | 0.321 | 3249 unclassified | unclassified | unclassified | unclassified | Eubacteriales | Clostridia | Firmicutes |
| Eubacteriales sp. (HG3A.0221) | T90 | -0.026 | 0.141 | 0.321 | 3249 unclassified | unclassified | unclassified | unclassified | Eubacteriales | Clostridia | Firmicutes |
| Eubacteriales sp. (HG3A.0773) | ODI | -0.023 | 0.199 | 0.321 | 3249 unclassified | unclassified | unclassified | unclassified | Eubacteriales | Clostridia | Firmicutes |
| Firmicutes sp. (HG3A.1471) | T90 | -0.026 | 0.141 | 0.321 | 3249 unclassified | unclassified | unclassified | unclassified | unclassified | unclassified | Firmicutes |
| Levilactobacillus brevis (HG3A.1848) | ODI | 0.023 | 0.198 | 0.321 | 3249 unclassified | Levilactobacillus  brevis | Levilactobacillus | Lactobacillaceae | Lactobacillales | Bacilli | Firmicutes |
| Streptococcus agalactiae (HG3A.1733) | ODI | 0.023 | 0.199 | 0.321 | 3249 unclassified | Streptococcus agalactiae | Streptococcus | Streptococcaceae | Lactobacillales | Bacilli | Firmicutes |
| Eubacteriales sp. (HG3A.0703) | AHI | -0.03 | 0.11 | 0.322 | 2909 unclassified | unclassified | unclassified | unclassified | Eubacteriales | Clostridia | Firmicutes |
| Oscillospiraceae sp. (HG3A.1421) | AHI | 0.03 | 0.11 | 0.322 | 2909 unclassified | unclassified | unclassified | Oscillospiraceae | Eubacteriales | Clostridia | Firmicutes |
| Eubacteriales sp. (HG3A.0158) | AHI | -0.03 | 0.111 | 0.323 | 2909 unclassified | unclassified | unclassified | unclassified | Eubacteriales | Clostridia | Firmicutes |
| Eubacteriales sp. (HG3A.0565) | AHI | -0.03 | 0.112 | 0.323 | 2909 unclassified | unclassified | unclassified | unclassified | Eubacteriales | Clostridia | Firmicutes |
| Eubacteriales sp. (HG3A.0626) | AHI | -0.03 | 0.112 | 0.323 | 2909 unclassified | unclassified | unclassified | unclassified | Eubacteriales | Clostridia | Firmicutes |
| Clostridia sp. (HG3A.0879) | AHI | -0.03 | 0.116 | 0.324 | 2909 unclassified | unclassified | unclassified | unclassified | unclassified | Clostridia | Firmicutes |
| Eubacteriales sp. (HG3A.0291) | AHI | -0.03 | 0.115 | 0.324 | 2909 unclassified | unclassified | unclassified | unclassified | Eubacteriales | Clostridia | Firmicutes |
| Eubacteriales sp. (HG3A.0325) | AHI | -0.03 | 0.113 | 0.324 | 2909 unclassified | unclassified | unclassified | unclassified | Eubacteriales | Clostridia | Firmicutes |
| Eubacteriales sp. (HG3A.0371) | AHI | -0.03 | 0.115 | 0.324 | 2909 unclassified | unclassified | unclassified | unclassified | Eubacteriales | Clostridia | Firmicutes |
| Eubacteriales sp. (HG3A.0427) | AHI | -0.03 | 0.114 | 0.324 | 2909 unclassified | unclassified | unclassified | unclassified | Eubacteriales | Clostridia | Firmicutes |
| Eubacteriales sp. (HG3A.0794) | AHI | -0.03 | 0.114 | 0.324 | 2909 unclassified | unclassified | unclassified | unclassified | Eubacteriales | Clostridia | Firmicutes |
| Eubacteriales sp. (HG3A.0832) | AHI | -0.03 | 0.115 | 0.324 | 2909 unclassified | unclassified | unclassified | unclassified | Eubacteriales | Clostridia | Firmicutes |
| Eubacteriales sp. (HG3A.1003) | AHI | -0.03 | 0.113 | 0.324 | 2909 unclassified | unclassified | unclassified | unclassified | Eubacteriales | Clostridia | Firmicutes |
| Lachnoclostridium sp. (HG3A.0655) | AHI | 0.03 | 0.116 | 0.324 | 2909 unclassified | unclassified | Lachnoclostridiu m | Lachnospiraceae | Eubacteriales | Clostridia | Firmicutes |

| Alistipes sp. AF17-16 (HG3A.0150) | AHI | -0.029 | 0.118 | 0.325 | 2909 unclassified | Alistipes sp. AF17-  16 | Alistipes | Rikenellaceae | Bacteroidales | Bacteroidia | Bacteroidetes |
| --- | --- | --- | --- | --- | --- | --- | --- | --- | --- | --- | --- |
| Clostridia sp. (HG3A.0767) | ODI | -0.023 | 0.203 | 0.325 | 3249 unclassified | unclassified | unclassified | unclassified | unclassified | Clostridia | Firmicutes |
| Eubacteriales sp. (HG3A.0656) | AHI | -0.03 | 0.117 | 0.325 | 2909 unclassified | unclassified | unclassified | unclassified | Eubacteriales | Clostridia | Firmicutes |
| Eubacteriales sp. (HG3A.0685) | ODI | -0.023 | 0.202 | 0.325 | 3249 unclassified | unclassified | unclassified | unclassified | Eubacteriales | Clostridia | Firmicutes |
| Massilistercora timonensis  (HG3A.0458) | AHI | -0.029 | 0.118 | 0.325 | 2909 unclassified | Massilistercora  timonensis | Massilistercora | unclassified | Eubacteriales | Clostridia | Firmicutes |
| Oxalobacter sp. (HG3A.1218) | AHI | -0.029 | 0.117 | 0.325 | 2909 unclassified | unclassified | Oxalobacter | Oxalobacteraceae | Burkholderiales | Betaproteobacte ria | Proteobacteria |
| Eubacteriales sp. (HG3A.0406) | ODI | -0.023 | 0.204 | 0.326 | 3249 unclassified | unclassified | unclassified | unclassified | Eubacteriales | Clostridia | Firmicutes |
| Eubacteriales sp. (HG3A.0630) | AHI | -0.029 | 0.119 | 0.326 | 2909 unclassified | unclassified | unclassified | unclassified | Eubacteriales | Clostridia | Firmicutes |
| Eubacteriales sp. (HG3A.0939) | AHI | -0.029 | 0.119 | 0.326 | 2909 unclassified | unclassified | unclassified | unclassified | Eubacteriales | Clostridia | Firmicutes |
| Oscillospiraceae sp. (HG3A.1173) | T90 | -0.026 | 0.145 | 0.326 | 3249 unclassified | unclassified | unclassified | Oscillospiraceae | Eubacteriales | Clostridia | Firmicutes |
| Eubacteriales sp. (HG3A.0213) | AHI | -0.029 | 0.121 | 0.327 | 2909 unclassified | unclassified | unclassified | unclassified | Eubacteriales | Clostridia | Firmicutes |
| Eubacteriales sp. (HG3A.0887) | AHI | -0.029 | 0.121 | 0.327 | 2909 unclassified | unclassified | unclassified | unclassified | Eubacteriales | Clostridia | Firmicutes |
| Firmicutes sp. (HG3A.1345) | AHI | -0.029 | 0.121 | 0.327 | 2909 unclassified | unclassified | unclassified | unclassified | unclassified | unclassified | Firmicutes |
| Clostridia sp. (HG3A.0815) | AHI | -0.029 | 0.122 | 0.328 | 2909 unclassified | unclassified | unclassified | unclassified | unclassified | Clostridia | Firmicutes |
| Clostridia sp. (HG3A.0852) | AHI | -0.029 | 0.124 | 0.328 | 2909 unclassified | unclassified | unclassified | unclassified | unclassified | Clostridia | Firmicutes |
| Clostridia sp. (HG3A.1452) | AHI | -0.029 | 0.122 | 0.328 | 2909 unclassified | unclassified | unclassified | unclassified | unclassified | Clostridia | Firmicutes |
| Eubacteriales sp. (HG3A.0186) | AHI | -0.029 | 0.123 | 0.328 | 2909 unclassified | unclassified | unclassified | unclassified | Eubacteriales | Clostridia | Firmicutes |
| Eubacteriales sp. (HG3A.0312) | AHI | -0.029 | 0.124 | 0.328 | 2909 unclassified | unclassified | unclassified | unclassified | Eubacteriales | Clostridia | Firmicutes |
| Eubacterium ramulus (HG3A.0068) | AHI | 0.029 | 0.123 | 0.328 | 2909 unclassified | Eubacterium  ramulus | Eubacterium | Eubacteriaceae | Eubacteriales | Clostridia | Firmicutes |
| Firmicutes sp. (HG3A.0541) | AHI | -0.029 | 0.122 | 0.328 | 2909 unclassified | unclassified | unclassified | unclassified | unclassified | unclassified | Firmicutes |
| Butyricicoccus sp. OM04-18BH  (HG3A.0139) | T90 | -0.026 | 0.147 | 0.329 | 3249 unclassified | Butyricicoccus sp.  OM04-18BH | Butyricicoccus | Clostridiaceae | Eubacteriales | Clostridia | Firmicutes |
| Firmicutes sp. (HG3A.0915) | T90 | -0.026 | 0.147 | 0.329 | 3249 unclassified | unclassified | unclassified | unclassified | unclassified | unclassified | Firmicutes |
| Firmicutes sp. (HG3A.1091) | T90 | -0.026 | 0.147 | 0.329 | 3249 unclassified | unclassified | unclassified | unclassified | unclassified | unclassified | Firmicutes |
| Firmicutes sp. (HG3A.1162) | T90 | -0.026 | 0.147 | 0.329 | 3249 unclassified | unclassified | unclassified | unclassified | unclassified | unclassified | Firmicutes |
| Eubacteriales sp. (HG3A.0754) | ODI | -0.022 | 0.208 | 0.333 | 3249 unclassified | unclassified | unclassified | unclassified | Eubacteriales | Clostridia | Firmicutes |
| Clostridia sp. (HG3A.0828) | T90 | -0.026 | 0.151 | 0.335 | 3249 unclassified | unclassified | unclassified | unclassified | unclassified | Clostridia | Firmicutes |
| Clostridia sp. (HG3A.1157) | T90 | -0.025 | 0.152 | 0.337 | 3249 unclassified | unclassified | unclassified | unclassified | unclassified | Clostridia | Firmicutes |

| Clostridia sp. (HG3A.1217) | T90 | -0.025 | 0.152 | 0.337 | 3249 unclassified | unclassified | unclassified | unclassified | unclassified | Clostridia | Firmicutes |
| --- | --- | --- | --- | --- | --- | --- | --- | --- | --- | --- | --- |
| Eubacteriales sp. (HG3A.0312) | ODI | -0.022 | 0.212 | 0.337 | 3249 unclassified | unclassified | unclassified | unclassified | Eubacteriales | Clostridia | Firmicutes |
| Alistipes sp. An66 (HG3A.1535) | T90 | -0.025 | 0.154 | 0.338 | 3249 unclassified | Alistipes sp. An66 | Alistipes | Rikenellaceae | Bacteroidales | Bacteroidia | Bacteroidetes |
| Clostridia sp. (HG3A.0733) | AHI | -0.029 | 0.128 | 0.338 | 2909 unclassified | unclassified | unclassified | unclassified | unclassified | Clostridia | Firmicutes |
| Massilistercora timonensis (HG3A.0458) | T90 | -0.025 | 0.154 | 0.338 | 3249 unclassified | Massilistercora timonensis | Massilistercora | unclassified | Eubacteriales | Clostridia | Firmicutes |
| Parasutterella excrementihominis (HG3A.0159) | T90 | -0.025 | 0.153 | 0.338 | 3249 unclassified | Parasutterella excrementihominis | Parasutterella | Sutterellaceae | Burkholderiales | Betaproteobacte ria | Proteobacteria |
| Blautia producta (HG3A.0905) | ODI | 0.022 | 0.213 | 0.339 | 3249 unclassified | Blautia producta | Blautia | Lachnospiraceae | Eubacteriales | Clostridia | Firmicutes |
| Candidatus Borkfalkia ceftriaxoniphila |  |  |  |  |  | Candidatus  Borkfalkia | Candidatus | Candidatus | Candidatus |  |  |
| (HG3A.0595) | ODI | -0.022 | 0.216 | 0.339 | 3249 unclassified | ceftriaxoniphila | Borkfalkia | Borkfalkiaceae | Borkfalkiales | Clostridia | Firmicutes |
| Catenibacterium mitsuokai (HG3A.0775) | ODI | 0.022 | 0.213 | 0.339 | 3249 unclassified | Catenibacterium mitsuokai | Catenibacterium | Coprobacillaceae | Erysipelotrichales | Erysipelotrichia | Firmicutes |
| Clostridia sp. (HG3A.0828) | ODI | -0.022 | 0.214 | 0.339 | 3249 unclassified | unclassified | unclassified | unclassified | unclassified | Clostridia | Firmicutes |
| Eubacteriales sp. (HG3A.0364) | ODI | -0.022 | 0.216 | 0.339 | 3249 unclassified | unclassified | unclassified | unclassified | Eubacteriales | Clostridia | Firmicutes |
| Eubacteriales sp. (HG3A.0935) | ODI | -0.022 | 0.215 | 0.339 | 3249 unclassified | unclassified | unclassified | unclassified | Eubacteriales | Clostridia | Firmicutes |
| Firmicutes sp. (HG3A.0581) | ODI | -0.022 | 0.214 | 0.339 | 3249 unclassified | unclassified | unclassified | unclassified | unclassified | unclassified | Firmicutes |
| Lachnotalea sp. AF33-28  (HG3A.0403) | ODI | -0.022 | 0.216 | 0.339 | 3249 unclassified | Lachnotalea sp.  AF33-28 | Lachnotalea | Lachnospiraceae | Eubacteriales | Clostridia | Firmicutes |
| Clostridia sp. (HG3A.1375) | T90 | -0.025 | 0.156 | 0.340 | 3249 unclassified | unclassified | unclassified | unclassified | unclassified | Clostridia | Firmicutes |
| Eubacteriales sp. (HG3A.0858) | T90 | -0.025 | 0.156 | 0.340 | 3249 unclassified | unclassified | unclassified | unclassified | Eubacteriales | Clostridia | Firmicutes |
| Firmicutes sp. (HG3A.0581) | T90 | -0.025 | 0.156 | 0.340 | 3249 unclassified | unclassified | unclassified | unclassified | unclassified | unclassified | Firmicutes |
| Parasutterella excrementihominis (HG3A.0159) | AHI | -0.029 | 0.129 | 0.340 | 2909 unclassified | Parasutterella excrementihominis | Parasutterella | Sutterellaceae | Burkholderiales | Betaproteobacte ria | Proteobacteria |
| Clostridia sp. (HG3A.0724) | T90 | -0.025 | 0.159 | 0.344 | 3249 unclassified | unclassified | unclassified | unclassified | unclassified | Clostridia | Firmicutes |
| Eubacteriales sp. (HG3A.1243) | AHI | -0.028 | 0.132 | 0.345 | 2909 unclassified | unclassified | unclassified | unclassified | Eubacteriales | Clostridia | Firmicutes |
| Alistipes sp. AF17-16 (HG3A.0150) | T90 | -0.025 | 0.166 | 0.346 | 3249 unclassified | Alistipes sp. AF17- 16 | Alistipes | Rikenellaceae | Bacteroidales | Bacteroidia | Bacteroidetes |
| Bacteria sp. (HG3A.1096) | T90 | -0.025 | 0.167 | 0.346 | 3249 unclassified | unclassified | unclassified | unclassified | unclassified | unclassified | unclassified |
| Bacteria sp. (HG3A.1545) | T90 | -0.025 | 0.162 | 0.346 | 3249 unclassified | unclassified | unclassified | unclassified | unclassified | unclassified | unclassified |

| Bacteroidales sp. (HG3A.0340) | T90 | -0.025 | 0.161 | 0.346 | 3249 unclassified | unclassified | unclassified | unclassified | Bacteroidales | Bacteroidia | Bacteroidetes |
| --- | --- | --- | --- | --- | --- | --- | --- | --- | --- | --- | --- |
| Clostridia sp. (HG3A.0746) | T90 | -0.025 | 0.166 | 0.346 | 3249 unclassified | unclassified | unclassified | unclassified | unclassified | Clostridia | Firmicutes |
| Clostridia sp. (HG3A.1053) | T90 | -0.025 | 0.161 | 0.346 | 3249 unclassified | unclassified | unclassified | unclassified | unclassified | Clostridia | Firmicutes |
| Eggerthellaceae sp. (HG3A.0171) | T90 | -0.025 | 0.166 | 0.346 | 3249 unclassified | unclassified | unclassified | Eggerthellaceae | Eggerthellales | Coriobacteriia | Actinobacteria |
| Eubacteriales sp. (HG3A.0490) | T90 | -0.025 | 0.163 | 0.346 | 3249 unclassified | unclassified | unclassified | unclassified | Eubacteriales | Clostridia | Firmicutes |
| Eubacteriales sp. (HG3A.0972) | T90 | -0.025 | 0.163 | 0.346 | 3249 unclassified | unclassified | unclassified | unclassified | Eubacteriales | Clostridia | Firmicutes |
| Eubacteriales sp. (HG3A.1167) | T90 | -0.025 | 0.164 | 0.346 | 3249 unclassified | unclassified | unclassified | unclassified | Eubacteriales | Clostridia | Firmicutes |
| Eubacteriales sp. (HG3A.1177) | T90 | -0.025 | 0.167 | 0.346 | 3249 unclassified | unclassified | unclassified | unclassified | Eubacteriales | Clostridia | Firmicutes |
| Eubacteriales sp. (HG3A.1256) | T90 | -0.025 | 0.161 | 0.346 | 3249 unclassified | unclassified | unclassified | unclassified | Eubacteriales | Clostridia | Firmicutes |
| Firmicutes sp. (HG3A.0874) | T90 | -0.025 | 0.165 | 0.346 | 3249 unclassified | unclassified | unclassified | unclassified | unclassified | unclassified | Firmicutes |
| Limosilactobacillus vaginalis  (HG3A.1341) | T90 | 0.025 | 0.164 | 0.346 | 3249 unclassified | Limosilactobacillus  vaginalis | Limosilactobacillu  s | Lactobacillaceae | Lactobacillales | Bacilli | Firmicutes |
| Mesosutterella multiformis (HG3A.0520) | T90 | -0.025 | 0.164 | 0.346 | 3249 unclassified | Mesosutterella multiformis | Mesosutterella | Sutterellaceae | Burkholderiales | Betaproteobacte ria | Proteobacteria |
| Oscillospiraceae sp. (HG3A.1588) | T90 | -0.025 | 0.166 | 0.346 | 3249 unclassified | unclassified | unclassified | Oscillospiraceae | Eubacteriales | Clostridia | Firmicutes |
| Victivallis lenta (HG3A.0525) | T90 | -0.025 | 0.165 | 0.346 | 3249 unclassified | Victivallis lenta | Victivallis | Victivallaceae | Victivallales | Lentisphaeria | Lentisphaerae |
| Allisonella histaminiformans  (HG3A.0332) | T90 | 0.024 | 0.169 | 0.348 | 3249 unclassified | Allisonella  histaminiformans | Allisonella | Veillonellaceae | Veillonellales | Negativicutes | Firmicutes |
| Blautia argi (HG3A.1450) | T90 | 0.024 | 0.169 | 0.348 | 3249 unclassified | Blautia argi | Blautia | Lachnospiraceae | Eubacteriales | Clostridia | Firmicutes |
| Limosilactobacillus Limosilactobacillu | | | | | | | | | | | |
| Limosilactobacillus oris (HG3A.1462) | T90 | 0.024 | 0.17 | 0.348 | 3249 unclassified | oris | s | Lactobacillaceae | Lactobacillales | Bacilli | Firmicutes |
| Pseudoflavonifractor sp. An184 (HG3A.0253) | T90 | -0.024 | 0.169 | 0.348 | 3249 unclassified | Pseudoflavonifracto r sp. An184 | Pseudoflavonifrac tor | Oscillospiraceae | Eubacteriales | Clostridia | Firmicutes |
| Clostridia sp. (HG3A.0946) | ODI | -0.022 | 0.223 | 0.349 | 3249 unclassified | unclassified | unclassified | unclassified | unclassified | Clostridia | Firmicutes |
| Clostridia sp. (HG3A.1375) | AHI | -0.028 | 0.134 | 0.349 | 2909 unclassified | unclassified | unclassified | unclassified | unclassified | Clostridia | Firmicutes |
| Anaerostipes caccae (HG3A.0747) | AHI | 0.028 | 0.136 | 0.350 | 2909 unclassified | Anaerostipes  caccae | Anaerostipes | Lachnospiraceae | Eubacteriales | Clostridia | Firmicutes |
| Bacteria sp. (HG3A.0500) | AHI | -0.028 | 0.136 | 0.350 | 2909 unclassified | unclassified | unclassified | unclassified | unclassified | unclassified | unclassified |
| Candidatus Borkfalkiales sp.  (HG3A.1397) | AHI | -0.028 | 0.136 | 0.350 | 2909 unclassified | unclassified | unclassified | unclassified | Candidatus  Borkfalkiales | Clostridia | Firmicutes |
| Eubacteriales sp. (HG3A.0537) | ODI | -0.022 | 0.224 | 0.350 | 3249 unclassified | unclassified | unclassified | unclassified | Eubacteriales | Clostridia | Firmicutes |
| Eubacteriales sp. (HG3A.0654) | AHI | -0.028 | 0.136 | 0.350 | 2909 unclassified | unclassified | unclassified | unclassified | Eubacteriales | Clostridia | Firmicutes |

| Firmicutes sp. (HG3A.1124) | AHI | -0.028 | 0.135 | 0.350 | 2909 unclassified | unclassified | unclassified | unclassified | unclassified | unclassified | Firmicutes |
| --- | --- | --- | --- | --- | --- | --- | --- | --- | --- | --- | --- |
| Clostridia sp. (HG3A.1486) | T90 | -0.024 | 0.173 | 0.351 | 3249 unclassified | unclassified | unclassified | unclassified | unclassified | Clostridia | Firmicutes |
| Clostridium perfringens (HG3A.0959) | T90 | 0.024 | 0.173 | 0.351 | 3249 unclassified | Clostridium perfringens | Clostridium | Clostridiaceae | Eubacteriales | Clostridia | Firmicutes |
| Eubacteriales sp. (HG3A.0345) | T90 | -0.024 | 0.172 | 0.351 | 3249 unclassified | unclassified | unclassified | unclassified | Eubacteriales | Clostridia | Firmicutes |
| Lachnospiraceae sp. (HG3A.0899) | AHI | -0.028 | 0.137 | 0.351 | 2909 unclassified | unclassified | unclassified | Lachnospiraceae | Eubacteriales | Clostridia | Firmicutes |
| Oscillospiraceae sp. (HG3A.0944) | T90 | -0.024 | 0.173 | 0.351 | 3249 unclassified | unclassified | unclassified | Oscillospiraceae | Eubacteriales | Clostridia | Firmicutes |
| Bacteroides intestinalis (HG3A.0265) | ODI | -0.022 | 0.226 | 0.352 | 3249 unclassified | Bacteroides intestinalis | Bacteroides | Bacteroidaceae | Bacteroidales | Bacteroidia | Bacteroidetes |
| Eubacteriales sp. (HG3A.0093) | ODI | -0.022 | 0.226 | 0.352 | 3249 unclassified | unclassified | unclassified | unclassified | Eubacteriales | Clostridia | Firmicutes |
| Eubacteriales sp. (HG3A.0230) | T90 | -0.024 | 0.174 | 0.352 | 3249 unclassified | unclassified | unclassified | unclassified | Eubacteriales | Clostridia | Firmicutes |
| Eubacteriales sp. (HG3A.0628) | T90 | -0.024 | 0.174 | 0.352 | 3249 unclassified | unclassified | unclassified | unclassified | Eubacteriales | Clostridia | Firmicutes |
| Eubacteriales sp. (HG3A.0627) | AHI | -0.028 | 0.139 | 0.354 | 2909 unclassified | unclassified | unclassified | unclassified | Eubacteriales | Clostridia | Firmicutes |
| Eubacteriales sp. (HG3A.0873) | T90 | -0.024 | 0.176 | 0.355 | 3249 unclassified | unclassified | unclassified | unclassified | Eubacteriales | Clostridia | Firmicutes |
| Firmicutes sp. (HG3A.0641) | T90 | -0.024 | 0.177 | 0.355 | 3249 unclassified | unclassified | unclassified | unclassified | unclassified | unclassified | Firmicutes |
| Firmicutes sp. (HG3A.1075) | AHI | -0.028 | 0.14 | 0.355 | 2909 unclassified | unclassified | unclassified | unclassified | unclassified | unclassified | Firmicutes |
| Prevotella sp. (HG3A.1009) | T90 | 0.024 | 0.177 | 0.355 | 3249 unclassified | unclassified | Prevotella | Prevotellaceae | Bacteroidales | Bacteroidia | Bacteroidetes |
| Blautia argi (HG3A.1450) | AHI | 0.028 | 0.141 | 0.356 | 2909 unclassified | Blautia argi | Blautia | Lachnospiraceae | Eubacteriales | Clostridia | Firmicutes |
| Dialister pneumosintes (HG3A.1496) | AHI | 0.028 | 0.142 | 0.356 | 2909 unclassified | Dialister pneumosintes | Dialister | Veillonellaceae | Veillonellales | Negativicutes | Firmicutes |
| Eubacteriales sp. (HG3A.0383) | AHI | -0.028 | 0.141 | 0.356 | 2909 unclassified | unclassified | unclassified | unclassified | Eubacteriales | Clostridia | Firmicutes |
| Eubacteriales sp. (HG3A.0668) | AHI | -0.028 | 0.141 | 0.356 | 2909 unclassified | unclassified | unclassified | unclassified | Eubacteriales | Clostridia | Firmicutes |
| Clostridia sp. (HG3A.0756) | T90 | -0.024 | 0.179 | 0.357 | 3249 unclassified | unclassified | unclassified | unclassified | unclassified | Clostridia | Firmicutes |
| Eubacteriales sp. (HG3A.0473) | ODI | -0.021 | 0.23 | 0.357 | 3249 unclassified | unclassified | unclassified | unclassified | Eubacteriales | Clostridia | Firmicutes |
| Eubacteriales sp. (HG3A.0868) | T90 | -0.024 | 0.179 | 0.357 | 3249 unclassified | unclassified | unclassified | unclassified | Eubacteriales | Clostridia | Firmicutes |
| Eubacteriales sp. (HG3A.1094) | ODI | -0.021 | 0.23 | 0.357 | 3249 unclassified | unclassified | unclassified | unclassified | Eubacteriales | Clostridia | Firmicutes |
| Lacticaseibacillus paracasei subsp. |  |  |  |  | Lacticaseibacillus  paracasei subsp. | Lacticaseibacillus |  |  |  |  |  |
| paracasei (HG3A.0853) | T90 | 0.024 | 0.179 | 0.357 | 3249 paracasei | paracasei | Lacticaseibacillus | Lactobacillaceae | Lactobacillales | Bacilli | Firmicutes |
| Clostridiaceae sp. (HG3A.0608) | ODI | -0.021 | 0.231 | 0.358 | 3249 unclassified | unclassified | unclassified | Clostridiaceae | Eubacteriales | Clostridia | Firmicutes |

| Clostridium sp. M62/1 (HG3A.0354) | T90 | 0.024 | 0.18 | 0.358 | 3249 unclassified | Clostridium sp.  M62/1 | Clostridium | Clostridiaceae | Eubacteriales | Clostridia | Firmicutes |
| --- | --- | --- | --- | --- | --- | --- | --- | --- | --- | --- | --- |
| Eubacteriales sp. (HG3A.0670) | AHI | -0.028 | 0.143 | 0.358 | 2909 unclassified | unclassified | unclassified | unclassified | Eubacteriales | Clostridia | Firmicutes |
| Clostridia sp. (HG3A.1192) | T90 | -0.024 | 0.182 | 0.359 | 3249 unclassified | unclassified | unclassified | unclassified | unclassified | Clostridia | Firmicutes |
| Clostridia sp. (HG3A.1486) | ODI | -0.021 | 0.232 | 0.359 | 3249 unclassified | unclassified | unclassified | unclassified | unclassified | Clostridia | Firmicutes |
| Eubacteriales sp. (HG3A.0916) | T90 | -0.024 | 0.182 | 0.359 | 3249 unclassified | unclassified | unclassified | unclassified | Eubacteriales | Clostridia | Firmicutes |
| Clostridia sp. (HG3A.0752) | AHI | -0.027 | 0.145 | 0.360 | 2909 unclassified | unclassified | unclassified | unclassified | unclassified | Clostridia | Firmicutes |
| Clostridia sp. (HG3A.1008) | AHI | -0.027 | 0.144 | 0.360 | 2909 unclassified | unclassified | unclassified | unclassified | unclassified | Clostridia | Firmicutes |
| Odoribacter splanchnicus (HG3A.0041) | AHI | -0.027 | 0.145 | 0.360 | 2909 unclassified | Odoribacter splanchnicus | Odoribacter | Odoribacteraceae | Bacteroidales | Bacteroidia | Bacteroidetes |
| Clostridia sp. (HG3A.0564) | AHI | -0.027 | 0.147 | 0.361 | 2909 unclassified | unclassified | unclassified | unclassified | unclassified | Clostridia | Firmicutes |
| Clostridia sp. (HG3A.1128) | AHI | -0.027 | 0.147 | 0.361 | 2909 unclassified | unclassified | unclassified | unclassified | unclassified | Clostridia | Firmicutes |
| Firmicutes sp. (HG3A.0301) | ODI | -0.021 | 0.234 | 0.361 | 3249 unclassified | unclassified | unclassified | unclassified | unclassified | unclassified | Firmicutes |
| Eubacteriales sp. (HG3A.0244) | T90 | -0.024 | 0.184 | 0.362 | 3249 unclassified | unclassified | unclassified | unclassified | Eubacteriales | Clostridia | Firmicutes |
| Eubacteriales sp. (HG3A.1086) | T90 | -0.024 | 0.185 | 0.362 | 3249 unclassified | unclassified | unclassified | unclassified | Eubacteriales | Clostridia | Firmicutes |
| Firmicutes sp. (HG3A.0948) | T90 | -0.024 | 0.185 | 0.362 | 3249 unclassified | unclassified | unclassified | unclassified | unclassified | unclassified | Firmicutes |
| Bacteria sp. (HG3A.1349) | ODI | -0.021 | 0.237 | 0.363 | 3249 unclassified | unclassified | unclassified | unclassified | unclassified | unclassified | unclassified |
| Bacteroidales sp. (HG3A.1002) | T90 | -0.023 | 0.186 | 0.363 | 3249 unclassified | unclassified | unclassified | unclassified | Bacteroidales | Bacteroidia | Bacteroidetes |
| Clostridia sp. (HG3A.1252) | ODI | -0.021 | 0.237 | 0.363 | 3249 unclassified | unclassified | unclassified | unclassified | unclassified | Clostridia | Firmicutes |
| Dorea longicatena (HG3A.0039) | ODI | 0.021 | 0.237 | 0.363 | 3249 unclassified | Dorea longicatena | Dorea | Lachnospiraceae | Eubacteriales | Clostridia | Firmicutes |
| Eubacteriales sp. (HG3A.0136) | AHI | -0.027 | 0.148 | 0.363 | 2909 unclassified | unclassified | unclassified | unclassified | Eubacteriales | Clostridia | Firmicutes |
| Eubacteriales sp. (HG3A.0193) | T90 | -0.023 | 0.186 | 0.363 | 3249 unclassified | unclassified | unclassified | unclassified | Eubacteriales | Clostridia | Firmicutes |
| Eubacteriales sp. (HG3A.0352) | ODI | -0.021 | 0.238 | 0.363 | 3249 unclassified | unclassified | unclassified | unclassified | Eubacteriales | Clostridia | Firmicutes |
| Eubacteriales sp. (HG3A.0717) | ODI | -0.021 | 0.238 | 0.363 | 3249 unclassified | unclassified | unclassified | unclassified | Eubacteriales | Clostridia | Firmicutes |
| Latilactobacillus sakei subsp. sakei |  |  |  |  | Latilactobacillus  sakei subsp. | Latilactobacillus |  |  |  |  |  |
| (HG3A.0836) | ODI | 0.021 | 0.237 | 0.363 | 3249 sakei | sakei | Latilactobacillus | Lactobacillaceae | Lactobacillales | Bacilli | Firmicutes |

| Streptococcus oralis subsp. oralis (HG3A.0705) | ODI | 0.021 | 0.239 | 0.364 | Streptococcus  oralis subsp.  3249 oralis | Streptococcus oralis | Streptococcus | Streptococcaceae | Lactobacillales | Bacilli | Firmicutes |
| --- | --- | --- | --- | --- | --- | --- | --- | --- | --- | --- | --- |
| Clostridia sp. (HG3A.1111) | AHI | -0.027 | 0.15 | 0.367 | 2909 unclassified | unclassified | unclassified | unclassified | unclassified | Clostridia | Firmicutes |
| Streptococcus salivarius (HG3A.0071) | ODI | 0.021 | 0.242 | 0.367 | 3249 unclassified | Streptococcus salivarius | Streptococcus | Streptococcaceae | Lactobacillales | Bacilli | Firmicutes |
| Desulfovibrionales sp. (HG3A.0727) | ODI | 0.021 | 0.243 | 0.368 | 3249 unclassified | unclassified | unclassified | unclassified | Desulfovibrionales | Deltaproteobact  eria | Proteobacteria |
| Eubacteriales sp. (HG3A.0221) | AHI | -0.027 | 0.151 | 0.368 | 2909 unclassified | unclassified | unclassified | unclassified | Eubacteriales | Clostridia | Firmicutes |
| Eubacteriales sp. (HG3A.0474) | AHI | -0.027 | 0.151 | 0.368 | 2909 unclassified | unclassified | unclassified | unclassified | Eubacteriales | Clostridia | Firmicutes |
| Alistipes communis (HG3A.0064) | T90 | -0.023 | 0.191 | 0.370 | 3249 unclassified | Alistipes communis | Alistipes | Rikenellaceae | Bacteroidales | Bacteroidia | Bacteroidetes |
| Eubacteriales sp. (HG3A.0476) | T90 | -0.023 | 0.191 | 0.370 | 3249 unclassified | unclassified | unclassified | unclassified | Eubacteriales | Clostridia | Firmicutes |
| Eubacteriales sp. (HG3A.0511) | AHI | -0.027 | 0.153 | 0.370 | 2909 unclassified | unclassified | unclassified | unclassified | Eubacteriales | Clostridia | Firmicutes |
| Pediococcus pentosaceus  (HG3A.1246) | ODI | 0.021 | 0.245 | 0.370 | 3249 unclassified | Pediococcus  pentosaceus | Pediococcus | Lactobacillaceae | Lactobacillales | Bacilli | Firmicutes |
| Succinatimonas hippei (HG3A.1322) | T90 | 0.023 | 0.191 | 0.370 | 3249 unclassified | Succinatimonas hippei | Succinatimonas | Succinivibrionacea e | Aeromonadales | Gammaproteob acteria | Proteobacteria |
| Butyricicoccus sp. (HG3A.0008) | AHI | 0.027 | 0.154 | 0.372 | 2909 unclassified | unclassified | Butyricicoccus | Clostridiaceae | Eubacteriales | Clostridia | Firmicutes |
| Butyrivibrio crossotus (HG3A.0413) | T90 | -0.023 | 0.193 | 0.373 | 3249 unclassified | Butyrivibrio crossotus | Butyrivibrio | Lachnospiraceae | Eubacteriales | Clostridia | Firmicutes |
| Dorea phocaeensis (HG3A.0865) | AHI | 0.027 | 0.156 | 0.374 | 2909 unclassified | Dorea phocaeensis | Dorea | Lachnospiraceae | Eubacteriales | Clostridia | Firmicutes |
| Eubacteriales sp. (HG3A.0334) | AHI | -0.027 | 0.156 | 0.374 | 2909 unclassified | unclassified | unclassified | unclassified | Eubacteriales | Clostridia | Firmicutes |
| Eubacteriales sp. (HG3A.0363) | AHI | -0.027 | 0.155 | 0.374 | 2909 unclassified | unclassified | unclassified | unclassified | Eubacteriales | Clostridia | Firmicutes |
| Oscillibacter sp. PEA192 (HG3A.0021) | ODI | 0.021 | 0.248 | 0.374 | 3249 unclassified | Oscillibacter sp. PEA192 | Oscillibacter | Oscillospiraceae | Eubacteriales | Clostridia | Firmicutes |
| Eubacteriales sp. (HG3A.0175) | ODI | -0.02 | 0.249 | 0.375 | 3249 unclassified | unclassified | unclassified | unclassified | Eubacteriales | Clostridia | Firmicutes |
| Eubacteriales sp. (HG3A.0754) | T90 | -0.023 | 0.195 | 0.375 | 3249 unclassified | unclassified | unclassified | unclassified | Eubacteriales | Clostridia | Firmicutes |
| Lachnotalea sp. AF33-28  (HG3A.0403) | AHI | -0.027 | 0.157 | 0.376 | 2909 unclassified | Lachnotalea sp.  AF33-28 | Lachnotalea | Lachnospiraceae | Eubacteriales | Clostridia | Firmicutes |
| Clostridia sp. (HG3A.0094) | T90 | -0.023 | 0.196 | 0.377 | 3249 unclassified | unclassified | unclassified | unclassified | unclassified | Clostridia | Firmicutes |
| Clostridia sp. (HG3A.1262) | ODI | -0.02 | 0.251 | 0.377 | 3249 unclassified | unclassified | unclassified | unclassified | unclassified | Clostridia | Firmicutes |
| Haemophilus parainfluenzae (HG3A.0181) | T90 | -0.023 | 0.197 | 0.377 | 3249 unclassified | Haemophilus parainfluenzae | Haemophilus | Pasteurellaceae | Pasteurellales | Gammaproteob acteria | Proteobacteria |

| Clostridia sp. (HG3A.0996) | AHI | -0.026 | 0.159 | 0.378 | 2909 unclassified | unclassified | unclassified | unclassified | unclassified | Clostridia | Firmicutes |
| --- | --- | --- | --- | --- | --- | --- | --- | --- | --- | --- | --- |
| Eubacteriales sp. (HG3A.0342) | AHI | -0.026 | 0.16 | 0.378 | 2909 unclassified | unclassified | unclassified | unclassified | Eubacteriales | Clostridia | Firmicutes |
| Eubacteriales sp. (HG3A.0935) | AHI | -0.026 | 0.16 | 0.378 | 2909 unclassified | unclassified | unclassified | unclassified | Eubacteriales | Clostridia | Firmicutes |
| Anaerotruncus colihominis (HG3A.0307) | AHI | 0.026 | 0.161 | 0.379 | 2909 unclassified | Anaerotruncus colihominis | Anaerotruncus | Oscillospiraceae | Eubacteriales | Clostridia | Firmicutes |
| Clostridia sp. (HG3A.1057) | AHI | -0.026 | 0.162 | 0.379 | 2909 unclassified | unclassified | unclassified | unclassified | unclassified | Clostridia | Firmicutes |
| Eubacteriales sp. (HG3A.0267) | AHI | -0.026 | 0.162 | 0.379 | 2909 unclassified | unclassified | unclassified | unclassified | Eubacteriales | Clostridia | Firmicutes |
| Eubacteriales sp. (HG3A.0609) | T90 | -0.023 | 0.199 | 0.379 | 3249 unclassified | unclassified | unclassified | unclassified | Eubacteriales | Clostridia | Firmicutes |
| Oscillospiraceae sp. (HG3A.1270) | T90 | -0.023 | 0.199 | 0.379 | 3249 unclassified | unclassified | unclassified | Oscillospiraceae | Eubacteriales | Clostridia | Firmicutes |
| Ruthenibacterium lactatiformans  (HG3A.0020) | T90 | 0.023 | 0.199 | 0.379 | 3249 unclassified | Ruthenibacterium  lactatiformans | Ruthenibacterium | Oscillospiraceae | Eubacteriales | Clostridia | Firmicutes |
| Bacteria sp. (HG3A.0839) | AHI | -0.026 | 0.163 | 0.380 | 2909 unclassified | unclassified | unclassified | unclassified | unclassified | unclassified | unclassified |
| Eubacteriales sp. (HG3A.1087) | AHI | -0.026 | 0.163 | 0.380 | 2909 unclassified | unclassified | unclassified | unclassified | Eubacteriales | Clostridia | Firmicutes |
| Firmicutes sp. (HG3A.0817) | T90 | -0.023 | 0.201 | 0.380 | 3249 unclassified | unclassified | unclassified | unclassified | unclassified | unclassified | Firmicutes |
| Parabacteroides gordonii  (HG3A.0989) | T90 | -0.023 | 0.2 | 0.380 | 3249 unclassified | Parabacteroides  gordonii | Parabacteroides | Tannerellaceae | Bacteroidales | Bacteroidia | Bacteroidetes |
| Clostridia sp. (HG3A.1062) | T90 | -0.023 | 0.203 | 0.382 | 3249 unclassified | unclassified | unclassified | unclassified | unclassified | Clostridia | Firmicutes |
| Eubacteriales sp. (HG3A.0189) | AHI | -0.026 | 0.164 | 0.382 | 2909 unclassified | unclassified | unclassified | unclassified | Eubacteriales | Clostridia | Firmicutes |
| Eubacteriales sp. (HG3A.0827) | T90 | -0.023 | 0.202 | 0.382 | 3249 unclassified | unclassified | unclassified | unclassified | Eubacteriales | Clostridia | Firmicutes |
| Eubacteriales sp. (HG3A.0851) | T90 | -0.023 | 0.204 | 0.382 | 3249 unclassified | unclassified | unclassified | unclassified | Eubacteriales | Clostridia | Firmicutes |
| Eubacteriales sp. (HG3A.1019) | T90 | -0.023 | 0.203 | 0.382 | 3249 unclassified | unclassified | unclassified | unclassified | Eubacteriales | Clostridia | Firmicutes |
| Oxalobacter sp. (HG3A.1097) | T90 | -0.023 | 0.204 | 0.382 | 3249 unclassified | unclassified | Oxalobacter | Oxalobacteraceae | Burkholderiales | Betaproteobacte  ria | Proteobacteria |
| Bacteria sp. (HG3A.0708) | T90 | -0.022 | 0.207 | 0.384 | 3249 unclassified | unclassified | unclassified | unclassified | unclassified | unclassified | unclassified |
| Clostridia sp. (HG3A.1262) | T90 | -0.022 | 0.207 | 0.384 | 3249 unclassified | unclassified | unclassified | unclassified | unclassified | Clostridia | Firmicutes |
| Eubacteriales sp. (HG3A.0192) | T90 | -0.022 | 0.206 | 0.384 | 3249 unclassified | unclassified | unclassified | unclassified | Eubacteriales | Clostridia | Firmicutes |
| Oscillibacter sp. (HG3A.0046) | AHI | -0.026 | 0.166 | 0.384 | 2909 unclassified | unclassified | Oscillibacter | Oscillospiraceae | Eubacteriales | Clostridia | Firmicutes |
| Oxalobacter formigenes (HG3A.1755) | T90 | -0.022 | 0.207 | 0.384 | 3249 unclassified | Oxalobacter formigenes | Oxalobacter | Oxalobacteraceae | Burkholderiales | Betaproteobacte ria | Proteobacteria |
| Collinsella phocaeensis (HG3A.1340) | T90 | 0.022 | 0.209 | 0.385 | 3249 unclassified | Collinsella  phocaeensis | Collinsella | Coriobacteriaceae | Coriobacteriales | Coriobacteriia | Actinobacteria |
| Oscillospiraceae sp. (HG3A.0210) | T90 | -0.022 | 0.209 | 0.385 | 3249 unclassified | unclassified | unclassified | Oscillospiraceae | Eubacteriales | Clostridia | Firmicutes |

| Clostridium sp. OF03-18AA  (HG3A.0119) | T90 | -0.022 | 0.21 | 0.386 | 3249 unclassified | Clostridium sp.  OF03-18AA | Clostridium | Clostridiaceae | Eubacteriales | Clostridia | Firmicutes |
| --- | --- | --- | --- | --- | --- | --- | --- | --- | --- | --- | --- |
| Dorea sp. AF24-7LB (HG3A.0086) | T90 | 0.022 | 0.21 | 0.386 | 3249 unclassified | Dorea sp. AF24- 7LB | Dorea | Lachnospiraceae | Eubacteriales | Clostridia | Firmicutes |
| Eubacteriales sp. (HG3A.0443) | AHI | -0.026 | 0.167 | 0.386 | 2909 unclassified | unclassified | unclassified | unclassified | Eubacteriales | Clostridia | Firmicutes |
| Blautia sp. (HG3A.0416) | AHI | 0.026 | 0.168 | 0.387 | 2909 unclassified | unclassified | Blautia | Lachnospiraceae | Eubacteriales | Clostridia | Firmicutes |
| Coprococcus catus (HG3A.0037) | ODI | 0.02 | 0.259 | 0.387 | 3249 unclassified | Coprococcus catus | Coprococcus | Lachnospiraceae | Eubacteriales | Clostridia | Firmicutes |
| Eubacteriales sp. (HG3A.0113) | T90 | -0.022 | 0.211 | 0.387 | 3249 unclassified | unclassified | unclassified | unclassified | Eubacteriales | Clostridia | Firmicutes |
| Eubacteriales sp. (HG3A.0715) | T90 | -0.022 | 0.212 | 0.387 | 3249 unclassified | unclassified | unclassified | unclassified | Eubacteriales | Clostridia | Firmicutes |
| Eubacteriales sp. (HG3A.0717) | T90 | -0.022 | 0.212 | 0.387 | 3249 unclassified | unclassified | unclassified | unclassified | Eubacteriales | Clostridia | Firmicutes |
| Proteobacteria sp. (HG3A.0360) | ODI | -0.02 | 0.258 | 0.387 | 3249 unclassified | unclassified | unclassified | unclassified | unclassified | unclassified | Proteobacteria |
| Clostridia sp. (HG3A.1035) | T90 | -0.022 | 0.213 | 0.388 | 3249 unclassified | unclassified | unclassified | unclassified | unclassified | Clostridia | Firmicutes |
| Clostridia sp. (HG3A.1127) | AHI | -0.026 | 0.169 | 0.388 | 2909 unclassified | unclassified | unclassified | unclassified | unclassified | Clostridia | Firmicutes |
| Eubacteriales sp. (HG3A.0791) | AHI | -0.026 | 0.17 | 0.388 | 2909 unclassified | unclassified | unclassified | unclassified | Eubacteriales | Clostridia | Firmicutes |
| Succinatimonas hippei (HG3A.1322) | AHI | 0.026 | 0.169 | 0.388 | 2909 unclassified | Succinatimonas  hippei | Succinatimonas | Succinivibrionacea  e | Aeromonadales | Gammaproteob  acteria | Proteobacteria |
| Clostridia sp. (HG3A.0852) | ODI | -0.02 | 0.261 | 0.389 | 3249 unclassified | unclassified | unclassified | unclassified | unclassified | Clostridia | Firmicutes |
| Clostridia sp. (HG3A.0929) | ODI | -0.02 | 0.261 | 0.389 | 3249 unclassified | unclassified | unclassified | unclassified | unclassified | Clostridia | Firmicutes |
| Enterococcus faecium (HG3A.0886) | ODI | 0.02 | 0.262 | 0.389 | 3249 unclassified | Enterococcus faecium | Enterococcus | Enterococcaceae | Lactobacillales | Bacilli | Firmicutes |
| Eubacteriales sp. (HG3A.0516) | AHI | -0.026 | 0.172 | 0.389 | 2909 unclassified | unclassified | unclassified | unclassified | Eubacteriales | Clostridia | Firmicutes |
| Eubacteriales sp. (HG3A.0531) | AHI | -0.026 | 0.171 | 0.389 | 2909 unclassified | unclassified | unclassified | unclassified | Eubacteriales | Clostridia | Firmicutes |
| Eubacteriales sp. (HG3A.0670) | ODI | -0.02 | 0.26 | 0.389 | 3249 unclassified | unclassified | unclassified | unclassified | Eubacteriales | Clostridia | Firmicutes |
| Eubacteriales sp. (HG3A.0878) | ODI | 0.02 | 0.262 | 0.389 | 3249 unclassified | unclassified | unclassified | unclassified | Eubacteriales | Clostridia | Firmicutes |
| Faecalibacterium sp. (HG3A.0073) | AHI | -0.026 | 0.172 | 0.389 | 2909 unclassified | unclassified | Faecalibacterium | Oscillospiraceae | Eubacteriales | Clostridia | Firmicutes |
| Bacteroidales sp. (HG3A.1236) | T90 | -0.022 | 0.215 | 0.390 | 3249 unclassified | unclassified | unclassified | unclassified | Bacteroidales | Bacteroidia | Bacteroidetes |
| Oscillospiraceae sp. (HG3A.0388) | AHI | -0.026 | 0.173 | 0.390 | 2909 unclassified | unclassified | unclassified | Oscillospiraceae | Eubacteriales | Clostridia | Firmicutes |
| Coprococcus catus (HG3A.0037) | T90 | 0.022 | 0.216 | 0.391 | 3249 unclassified | Coprococcus catus | Coprococcus | Lachnospiraceae | Eubacteriales | Clostridia | Firmicutes |
| Eubacteriales sp. (HG3A.0062) | AHI | -0.026 | 0.174 | 0.392 | 2909 unclassified | unclassified | unclassified | unclassified | Eubacteriales | Clostridia | Firmicutes |

| Eubacteriales sp. (HG3A.0188) | AHI | -0.026 | 0.175 | 0.392 | 2909 unclassified | unclassified | unclassified | unclassified | Eubacteriales | Clostridia | Firmicutes |
| --- | --- | --- | --- | --- | --- | --- | --- | --- | --- | --- | --- |
| Anaerostipes hadrus (HG3A.0003) | ODI | 0.02 | 0.265 | 0.393 | 3249 unclassified | Anaerostipes  hadrus | Anaerostipes | Lachnospiraceae | Eubacteriales | Clostridia | Firmicutes |
| Clostridia sp. (HG3A.1452) | ODI | -0.02 | 0.266 | 0.394 | 3249 unclassified | unclassified | unclassified | unclassified | unclassified | Clostridia | Firmicutes |
| Eubacteriales sp. (HG3A.1187) | ODI | 0.02 | 0.267 | 0.394 | 3249 unclassified | unclassified | unclassified | unclassified | Eubacteriales | Clostridia | Firmicutes |
| [Clostridium] innocuum (HG3A.0365) | ODI | 0.02 | 0.27 | 0.396 | 3249 unclassified | [Clostridium] innocuum | Erysipelatoclostri dium | Erysipelotrichacea e | Erysipelotrichales | Erysipelotrichia | Firmicutes |
| Clostridia sp. (HG3A.0918) | ODI | -0.02 | 0.269 | 0.396 | 3249 unclassified | unclassified | unclassified | unclassified | unclassified | Clostridia | Firmicutes |
| Eubacteriales sp. (HG3A.0781) | ODI | -0.02 | 0.27 | 0.396 | 3249 unclassified | unclassified | unclassified | unclassified | Eubacteriales | Clostridia | Firmicutes |
| Eubacteriales sp. (HG3A.0450) | AHI | -0.025 | 0.178 | 0.397 | 2909 unclassified | unclassified | unclassified | unclassified | Eubacteriales | Clostridia | Firmicutes |
| Eubacteriales sp. (HG3A.1167) | AHI | -0.025 | 0.178 | 0.397 | 2909 unclassified | unclassified | unclassified | unclassified | Eubacteriales | Clostridia | Firmicutes |
| Latilactobacillus sakei subsp. sakei |  |  |  |  | Latilactobacillus  sakei subsp. | Latilactobacillus |  |  |  |  |  |
| (HG3A.0836) | AHI | 0.025 | 0.179 | 0.398 | 2909 sakei | sakei | Latilactobacillus | Lactobacillaceae | Lactobacillales | Bacilli | Firmicutes |
| Oscillospiraceae sp. (HG3A.0134) | AHI | -0.025 | 0.179 | 0.398 | 2909 unclassified | unclassified | unclassified | Oscillospiraceae | Eubacteriales | Clostridia | Firmicutes |
| Eubacteriales sp. (HG3A.0701) | ODI | -0.02 | 0.272 | 0.399 | 3249 unclassified | unclassified | unclassified | unclassified | Eubacteriales | Clostridia | Firmicutes |
| Clostridia sp. (HG3A.1108) | AHI | -0.025 | 0.181 | 0.400 | 2909 unclassified | unclassified | unclassified | unclassified | unclassified | Clostridia | Firmicutes |
| Eubacteriales sp. (HG3A.0367) | ODI | -0.019 | 0.274 | 0.400 | 3249 unclassified | unclassified | unclassified | unclassified | Eubacteriales | Clostridia | Firmicutes |
| Eubacteriales sp. (HG3A.0489) | AHI | -0.025 | 0.181 | 0.400 | 2909 unclassified | unclassified | unclassified | unclassified | Eubacteriales | Clostridia | Firmicutes |
| Veillonella rogosae (HG3A.0324) | ODI | -0.019 | 0.273 | 0.400 | 3249 unclassified | Veillonella rogosae | Veillonella | Veillonellaceae | Veillonellales | Negativicutes | Firmicutes |
| Lachnospiraceae sp. (HG3A.1525) | T90 | -0.022 | 0.222 | 0.401 | 3249 unclassified | unclassified | unclassified | Lachnospiraceae | Eubacteriales | Clostridia | Firmicutes |
| Alistipes ihumii (HG3A.0106) | AHI | -0.025 | 0.183 | 0.402 | 2909 unclassified | Alistipes ihumii | Alistipes | Rikenellaceae | Bacteroidales | Bacteroidia | Bacteroidetes |
| Alistipes sp. (HG3A.1385) | ODI | -0.019 | 0.276 | 0.402 | 3249 unclassified | unclassified | Alistipes | Rikenellaceae | Bacteroidales | Bacteroidia | Bacteroidetes |
| Oscillospiraceae sp. (HG3A.0576) | ODI | -0.019 | 0.277 | 0.402 | 3249 unclassified | unclassified | unclassified | Oscillospiraceae | Eubacteriales | Clostridia | Firmicutes |
| Eubacteriales sp. (HG3A.0439) | AHI | -0.025 | 0.184 | 0.403 | 2909 unclassified | unclassified | unclassified | unclassified | Eubacteriales | Clostridia | Firmicutes |
| Eisenbergiella tayi (HG3A.0355) | T90 | 0.022 | 0.225 | 0.406 | 3249 unclassified | Eisenbergiella tayi | Eisenbergiella | Lachnospiraceae | Eubacteriales | Clostridia | Firmicutes |
| Eubacteriales sp. (HG3A.0956) | AHI | -0.025 | 0.185 | 0.406 | 2909 unclassified | unclassified | unclassified | unclassified | Eubacteriales | Clostridia | Firmicutes |
| Eggerthellales sp. (HG3A.0848) | AHI | -0.025 | 0.187 | 0.407 | 2909 unclassified | unclassified | unclassified | unclassified | Eggerthellales | Coriobacteriia | Actinobacteria |
| Eubacteriales sp. (HG3A.1250) | T90 | -0.021 | 0.227 | 0.407 | 3249 unclassified | unclassified | unclassified | unclassified | Eubacteriales | Clostridia | Firmicutes |
| Eubacteriales sp. (HG3A.1294) | AHI | -0.025 | 0.186 | 0.407 | 2909 unclassified | unclassified | unclassified | unclassified | Eubacteriales | Clostridia | Firmicutes |
| Firmicutes sp. (HG3A.1195) | T90 | -0.021 | 0.227 | 0.407 | 3249 unclassified | unclassified | unclassified | unclassified | unclassified | unclassified | Firmicutes |
| Gemmiger formicilis (HG3A.0027) | T90 | 0.021 | 0.227 | 0.407 | 3249 unclassified | Gemmiger  formicilis | Gemmiger | unclassified | Eubacteriales | Clostridia | Firmicutes |

| Clostridiaceae sp. (HG3A.0471) | AHI | 0.025 | 0.188 | 0.408 | 2909 unclassified | unclassified | unclassified | Clostridiaceae | Eubacteriales | Clostridia | Firmicutes |
| --- | --- | --- | --- | --- | --- | --- | --- | --- | --- | --- | --- |
| Alistipes dispar (HG3A.0281) | ODI | -0.019 | 0.283 | 0.409 | 3249 unclassified | Alistipes dispar | Alistipes | Rikenellaceae | Bacteroidales | Bacteroidia | Bacteroidetes |
| Alistipes timonensis (HG3A.0586) | ODI | -0.019 | 0.282 | 0.409 | 3249 unclassified | Alistipes timonensis | Alistipes | Rikenellaceae | Bacteroidales | Bacteroidia | Bacteroidetes |
| Anaerotignum lactatifermentans  (HG3A.0676) | T90 | 0.021 | 0.229 | 0.409 | 3249 unclassified | Anaerotignum  lactatifermentans | Anaerotignum | Lachnospiraceae | Eubacteriales | Clostridia | Firmicutes |
| Clostridium sp. AF34-13 (HG3A.0173) | AHI | -0.025 | 0.189 | 0.409 | 2909 unclassified | Clostridium sp. AF34-13 | Clostridium | Clostridiaceae | Eubacteriales | Clostridia | Firmicutes |
| Eubacteriales sp. (HG3A.0158) | ODI | -0.019 | 0.283 | 0.409 | 3249 unclassified | unclassified | unclassified | unclassified | Eubacteriales | Clostridia | Firmicutes |
| Eubacteriales sp. (HG3A.0204) | ODI | -0.019 | 0.284 | 0.409 | 3249 unclassified | unclassified | unclassified | unclassified | Eubacteriales | Clostridia | Firmicutes |
| Eubacteriales sp. (HG3A.0502) | ODI | 0.019 | 0.285 | 0.409 | 3249 unclassified | unclassified | unclassified | unclassified | Eubacteriales | Clostridia | Firmicutes |
| Eubacteriales sp. (HG3A.0685) | T90 | -0.021 | 0.229 | 0.409 | 3249 unclassified | unclassified | unclassified | unclassified | Eubacteriales | Clostridia | Firmicutes |
| Eubacteriales sp. (HG3A.0983) | ODI | -0.019 | 0.285 | 0.409 | 3249 unclassified | unclassified | unclassified | unclassified | Eubacteriales | Clostridia | Firmicutes |
| Streptococcus gallolyticus subsp. |  |  |  |  | Streptococcus gallolyticus  subsp. | Streptococcus |  |  |  |  |  |
| gallolyticus (HG3A.1651) | ODI | 0.019 | 0.285 | 0.409 | 3249 gallolyticus | gallolyticus | Streptococcus | Streptococcaceae | Lactobacillales | Bacilli | Firmicutes |
| Turicibacter sanguinis (HG3A.0274) | ODI | -0.019 | 0.285 | 0.409 | 3249 unclassified | Turicibacter  sanguinis | Turicibacter | Turicibacteraceae | Erysipelotrichales | Erysipelotrichia | Firmicutes |
| Oscillibacter sp. (HG3A.0243) | AHI | -0.025 | 0.19 | 0.410 | 2909 unclassified | unclassified | Oscillibacter | Oscillospiraceae | Eubacteriales | Clostridia | Firmicutes |
| Eubacteriales sp. (HG3A.0441) | AHI | -0.025 | 0.192 | 0.411 | 2909 unclassified | unclassified | unclassified | unclassified | Eubacteriales | Clostridia | Firmicutes |
| Firmicutes sp. (HG3A.0581) | AHI | -0.025 | 0.192 | 0.411 | 2909 unclassified | unclassified | unclassified | unclassified | unclassified | unclassified | Firmicutes |
| Gemella morbillorum (HG3A.1782) | AHI | 0.025 | 0.192 | 0.411 | 2909 unclassified | Gemella  morbillorum | Gemella | unclassified | Bacillales | Bacilli | Firmicutes |
| Rothia mucilaginosa (HG3A.0559) | AHI | 0.025 | 0.193 | 0.411 | 2909 unclassified | Rothia mucilaginosa | Rothia | Micrococcaceae | Micrococcales | Actinomycetia | Actinobacteria |
| Clostridia sp. (HG3A.0660) | T90 | -0.021 | 0.232 | 0.412 | 3249 unclassified | unclassified | unclassified | unclassified | unclassified | Clostridia | Firmicutes |
| Eubacteriales sp. (HG3A.0985) | AHI | 0.024 | 0.193 | 0.412 | 2909 unclassified | unclassified | unclassified | unclassified | Eubacteriales | Clostridia | Firmicutes |
| Sellimonas intestinalis (HG3A.0417) | AHI | 0.024 | 0.195 | 0.413 | 2909 unclassified | Sellimonas  intestinalis | Sellimonas | Lachnospiraceae | Eubacteriales | Clostridia | Firmicutes |
| Bacteroides caccae (HG3A.0066) | AHI | -0.024 | 0.196 | 0.414 | 2909 unclassified | Bacteroides caccae | Bacteroides | Bacteroidaceae | Bacteroidales | Bacteroidia | Bacteroidetes |
| Butyrivibrio crossotus (HG3A.0413) | ODI | -0.019 | 0.289 | 0.414 | 3249 unclassified | Butyrivibrio  crossotus | Butyrivibrio | Lachnospiraceae | Eubacteriales | Clostridia | Firmicutes |
| Eubacteriales sp. (HG3A.0376) | ODI | -0.019 | 0.29 | 0.414 | 3249 unclassified | unclassified | unclassified | unclassified | Eubacteriales | Clostridia | Firmicutes |
| Eubacteriales sp. (HG3A.0760) | AHI | -0.024 | 0.196 | 0.415 | 2909 unclassified | unclassified | unclassified | unclassified | Eubacteriales | Clostridia | Firmicutes |
| Bacteria sp. (HG3A.0459) | T90 | -0.021 | 0.238 | 0.416 | 3249 unclassified | unclassified | unclassified | unclassified | unclassified | unclassified | unclassified |
| Eubacteriales sp. (HG3A.0184) | T90 | -0.021 | 0.236 | 0.416 | 3249 unclassified | unclassified | unclassified | unclassified | Eubacteriales | Clostridia | Firmicutes |

| Eubacteriales sp. (HG3A.0235) | T90 | -0.021 | 0.237 | 0.416 | 3249 unclassified | unclassified | unclassified | unclassified | Eubacteriales | Clostridia | Firmicutes |
| --- | --- | --- | --- | --- | --- | --- | --- | --- | --- | --- | --- |
| Eubacteriales sp. (HG3A.0322) | T90 | 0.021 | 0.238 | 0.416 | 3249 unclassified | unclassified | unclassified | unclassified | Eubacteriales | Clostridia | Firmicutes |
| Eubacteriales sp. (HG3A.1239) | T90 | -0.021 | 0.239 | 0.416 | 3249 unclassified | unclassified | unclassified | unclassified | Eubacteriales | Clostridia | Firmicutes |
| Lachnospiraceae sp. (HG3A.0252) | T90 | -0.021 | 0.238 | 0.416 | 3249 unclassified | unclassified | unclassified | Lachnospiraceae | Eubacteriales | Clostridia | Firmicutes |
| Oscillospiraceae sp. (HG3A.0693) | T90 | -0.021 | 0.239 | 0.416 | 3249 unclassified | unclassified | unclassified | Oscillospiraceae | Eubacteriales | Clostridia | Firmicutes |
| Proteobacteria sp. (HG3A.0360) | T90 | -0.021 | 0.235 | 0.416 | 3249 unclassified | unclassified | unclassified | unclassified | unclassified | unclassified | Proteobacteria |
| Pseudoflavonifractor sp. (HG3A.0844) | T90 | -0.021 | 0.238 | 0.416 | 3249 unclassified | unclassified | Pseudoflavonifrac tor | Oscillospiraceae | Eubacteriales | Clostridia | Firmicutes |
| Alistipes timonensis (HG3A.0586) | T90 | -0.021 | 0.243 | 0.417 | 3249 unclassified | Alistipes  timonensis | Alistipes | Rikenellaceae | Bacteroidales | Bacteroidia | Bacteroidetes |
| Barnesiellaceae sp. (HG3A.1180) | T90 | -0.021 | 0.242 | 0.417 | 3249 unclassified | unclassified | unclassified | Barnesiellaceae | Bacteroidales | Bacteroidia | Bacteroidetes |
| Clostridia sp. (HG3A.1127) | T90 | -0.021 | 0.242 | 0.417 | 3249 unclassified | unclassified | unclassified | unclassified | unclassified | Clostridia | Firmicutes |
| Clostridia sp. (HG3A.1193) | AHI | 0.024 | 0.199 | 0.417 | 2909 unclassified | unclassified | unclassified | unclassified | unclassified | Clostridia | Firmicutes |
| Clostridia sp. (HG3A.1220) | T90 | -0.021 | 0.242 | 0.417 | 3249 unclassified | unclassified | unclassified | unclassified | unclassified | Clostridia | Firmicutes |
| Eubacteriales sp. (HG3A.0396) | AHI | -0.024 | 0.198 | 0.417 | 2909 unclassified | unclassified | unclassified | unclassified | Eubacteriales | Clostridia | Firmicutes |
| Eubacteriales sp. (HG3A.0870) | T90 | -0.021 | 0.241 | 0.417 | 3249 unclassified | unclassified | unclassified | unclassified | Eubacteriales | Clostridia | Firmicutes |
| Eubacteriales sp. (HG3A.1045) | T90 | -0.021 | 0.242 | 0.417 | 3249 unclassified | unclassified | unclassified | unclassified | Eubacteriales | Clostridia | Firmicutes |
| Eubacteriales sp. (HG3A.1199) | T90 | 0.021 | 0.241 | 0.417 | 3249 unclassified | unclassified | unclassified | unclassified | Eubacteriales | Clostridia | Firmicutes |
| Desulfovibrio fairfieldensis (HG3A.0529) | ODI | -0.019 | 0.295 | 0.420 | 3249 unclassified | Desulfovibrio fairfieldensis | Desulfovibrio | Desulfovibrionacea e | Desulfovibrionales | Deltaproteobact eria | Proteobacteria |
| Streptococcus gordonii (HG3A.0713) | ODI | 0.019 | 0.295 | 0.420 | 3249 unclassified | Streptococcus  gordonii | Streptococcus | Streptococcaceae | Lactobacillales | Bacilli | Firmicutes |
| Clostridia sp. (HG3A.0741) | T90 | -0.021 | 0.246 | 0.421 | 3249 unclassified | unclassified | unclassified | unclassified | unclassified | Clostridia | Firmicutes |
| Erysipelotrichales sp. (HG3A.0303) | T90 | -0.02 | 0.25 | 0.421 | 3249 unclassified | unclassified | unclassified | unclassified | Erysipelotrichales | Erysipelotrichia | Firmicutes |
| Eubacteriales sp. (HG3A.0132) | T90 | -0.021 | 0.246 | 0.421 | 3249 unclassified | unclassified | unclassified | unclassified | Eubacteriales | Clostridia | Firmicutes |
| Eubacteriales sp. (HG3A.0320) | T90 | -0.02 | 0.249 | 0.421 | 3249 unclassified | unclassified | unclassified | unclassified | Eubacteriales | Clostridia | Firmicutes |
| Eubacteriales sp. (HG3A.0637) | T90 | -0.02 | 0.25 | 0.421 | 3249 unclassified | unclassified | unclassified | unclassified | Eubacteriales | Clostridia | Firmicutes |
| Eubacteriales sp. (HG3A.0691) | T90 | -0.021 | 0.248 | 0.421 | 3249 unclassified | unclassified | unclassified | unclassified | Eubacteriales | Clostridia | Firmicutes |
| Firmicutes sp. (HG3A.0596) | T90 | -0.021 | 0.249 | 0.421 | 3249 unclassified | unclassified | unclassified | unclassified | unclassified | unclassified | Firmicutes |

| Limosilactobacillus fermentum  (HG3A.0990) | AHI | 0.024 | 0.201 | 0.421 | 2909 unclassified | Limosilactobacillus  fermentum | Limosilactobacillu  s | Lactobacillaceae | Lactobacillales | Bacilli | Firmicutes |
| --- | --- | --- | --- | --- | --- | --- | --- | --- | --- | --- | --- |
| Peptostreptococcaceae sp. (HG3A.0200) | T90 | -0.021 | 0.248 | 0.421 | 3249 unclassified | unclassified | unclassified | Peptostreptococcac eae | Eubacteriales | Clostridia | Firmicutes |
| Staphylococcus aureus (HG3A.1538) | AHI | 0.024 | 0.201 | 0.421 | 2909 unclassified | Staphylococcus  aureus | Staphylococcus | Staphylococcaceae | Bacillales | Bacilli | Firmicutes |
| Clostridia sp. (HG3A.1062) | AHI | -0.024 | 0.203 | 0.422 | 2909 unclassified | unclassified | unclassified | unclassified | unclassified | Clostridia | Firmicutes |
| Lachnospiraceae sp. (HG3A.1641) | AHI | 0.024 | 0.203 | 0.422 | 2909 unclassified | unclassified | unclassified | Lachnospiraceae | Eubacteriales | Clostridia | Firmicutes |
| Clostridia sp. (HG3A.0661) | ODI | -0.018 | 0.298 | 0.423 | 3249 unclassified | unclassified | unclassified | unclassified | unclassified | Clostridia | Firmicutes |
| Clostridia sp. (HG3A.0787) | AHI | -0.024 | 0.205 | 0.424 | 2909 unclassified | unclassified | unclassified | unclassified | unclassified | Clostridia | Firmicutes |
| Clostridia sp. (HG3A.1247) | AHI | -0.024 | 0.205 | 0.424 | 2909 unclassified | unclassified | unclassified | unclassified | unclassified | Clostridia | Firmicutes |
| Firmicutes sp. (HG3A.1162) | ODI | -0.018 | 0.3 | 0.424 | 3249 unclassified | unclassified | unclassified | unclassified | unclassified | unclassified | Firmicutes |
| Pseudoruminococcus massiliensis (HG3A.0346) | ODI | -0.018 | 0.299 | 0.424 | 3249 unclassified | Pseudoruminococc us massiliensis | Pseudoruminococ cus | Oscillospiraceae | Eubacteriales | Clostridia | Firmicutes |
| Clostridia sp. (HG3A.0564) | ODI | -0.018 | 0.302 | 0.425 | 3249 unclassified | unclassified | unclassified | unclassified | unclassified | Clostridia | Firmicutes |
| Clostridia sp. (HG3A.1609) | ODI | -0.018 | 0.303 | 0.425 | 3249 unclassified | unclassified | unclassified | unclassified | unclassified | Clostridia | Firmicutes |
| Eubacteriales sp. (HG3A.0656) | ODI | -0.018 | 0.302 | 0.425 | 3249 unclassified | unclassified | unclassified | unclassified | Eubacteriales | Clostridia | Firmicutes |
| Eubacteriales sp. (HG3A.0792) | ODI | -0.018 | 0.301 | 0.425 | 3249 unclassified | unclassified | unclassified | unclassified | Eubacteriales | Clostridia | Firmicutes |
| Eubacteriales sp. (HG3A.1102) | ODI | -0.018 | 0.302 | 0.425 | 3249 unclassified | unclassified | unclassified | unclassified | Eubacteriales | Clostridia | Firmicutes |
| Eubacteriales sp. (HG3A.0226) | T90 | -0.02 | 0.254 | 0.426 | 3249 unclassified | unclassified | unclassified | unclassified | Eubacteriales | Clostridia | Firmicutes |
| Eubacteriales sp. (HG3A.0377) | T90 | -0.02 | 0.253 | 0.426 | 3249 unclassified | unclassified | unclassified | unclassified | Eubacteriales | Clostridia | Firmicutes |
| Oscillospiraceae sp. (HG3A.0616) | AHI | -0.024 | 0.207 | 0.426 | 2909 unclassified | unclassified | unclassified | Oscillospiraceae | Eubacteriales | Clostridia | Firmicutes |
| Oxalobacter sp. (HG3A.1097) | AHI | 0.024 | 0.207 | 0.426 | 2909 unclassified | unclassified | Oxalobacter | Oxalobacteraceae | Burkholderiales | Betaproteobacte  ria | Proteobacteria |
| Sutterella seckii (HG3A.0561) | T90 | 0.02 | 0.255 | 0.427 | 3249 unclassified | Sutterella seckii | Sutterella | Sutterellaceae | Burkholderiales | Betaproteobacte ria | Proteobacteria |
| Eubacteriales sp. (HG3A.1285) | ODI | -0.018 | 0.306 | 0.428 | 3249 unclassified | unclassified | unclassified | unclassified | Eubacteriales | Clostridia | Firmicutes |
| Clostridia sp. (HG3A.0893) | T90 | -0.02 | 0.257 | 0.429 | 3249 unclassified | unclassified | unclassified | unclassified | unclassified | Clostridia | Firmicutes |

| Eubacteriales sp. (HG3A.0962) | T90 | -0.02 | 0.257 | 0.429 | 3249 unclassified | unclassified | unclassified | unclassified | Eubacteriales | Clostridia | Firmicutes |
| --- | --- | --- | --- | --- | --- | --- | --- | --- | --- | --- | --- |
| Eubacteriales sp. (HG3A.0093) | AHI | -0.024 | 0.21 | 0.431 | 2909 unclassified | unclassified | unclassified | unclassified | Eubacteriales | Clostridia | Firmicutes |
| Eubacteriales sp. (HG3A.1126) | T90 | -0.02 | 0.259 | 0.431 | 3249 unclassified | unclassified | unclassified | unclassified | Eubacteriales | Clostridia | Firmicutes |
| Bacteroidales sp. (HG3A.1446) | ODI | -0.018 | 0.31 | 0.433 | 3249 unclassified | unclassified | unclassified | unclassified | Bacteroidales | Bacteroidia | Bacteroidetes |
| Clostridia sp. (HG3A.0996) | ODI | -0.018 | 0.311 | 0.434 | 3249 unclassified | unclassified | unclassified | unclassified | unclassified | Clostridia | Firmicutes |
| Victivallis lenta (HG3A.0525) | ODI | -0.018 | 0.312 | 0.434 | 3249 unclassified | Victivallis lenta | Victivallis | Victivallaceae | Victivallales | Lentisphaeria | Lentisphaerae |
| Lachnospiraceae sp. (HG3A.0399) | AHI | -0.023 | 0.213 | 0.435 | 2909 unclassified | unclassified | unclassified | Lachnospiraceae | Eubacteriales | Clostridia | Firmicutes |
| Eubacteriales sp. (HG3A.0614) | T90 | -0.02 | 0.262 | 0.436 | 3249 unclassified | unclassified | unclassified | unclassified | Eubacteriales | Clostridia | Firmicutes |
| Faecalibacterium sp. (HG3A.0073) | T90 | -0.02 | 0.263 | 0.436 | 3249 unclassified | unclassified | Faecalibacterium | Oscillospiraceae | Eubacteriales | Clostridia | Firmicutes |
| Oscillospiraceae sp. (HG3A.0612) | AHI | 0.023 | 0.214 | 0.436 | 2909 unclassified | unclassified | unclassified | Oscillospiraceae | Eubacteriales | Clostridia | Firmicutes |
| Eubacteriales sp. (HG3A.0649) | ODI | -0.018 | 0.314 | 0.437 | 3249 unclassified | unclassified | unclassified | unclassified | Eubacteriales | Clostridia | Firmicutes |
| Anaeroglobus geminatus (HG3A.1818) | T90 | 0.02 | 0.266 | 0.438 | 3249 unclassified | Anaeroglobus geminatus | Anaeroglobus | Veillonellaceae | Veillonellales | Negativicutes | Firmicutes |
| Clostridia sp. (HG3A.0933) | T90 | -0.02 | 0.268 | 0.438 | 3249 unclassified | unclassified | unclassified | unclassified | unclassified | Clostridia | Firmicutes |
| Clostridia sp. (HG3A.1193) | T90 | -0.02 | 0.266 | 0.438 | 3249 unclassified | unclassified | unclassified | unclassified | unclassified | Clostridia | Firmicutes |
| Eubacteriales sp. (HG3A.0453) | T90 | -0.02 | 0.267 | 0.438 | 3249 unclassified | unclassified | unclassified | unclassified | Eubacteriales | Clostridia | Firmicutes |
| Eubacteriales sp. (HG3A.0573) | T90 | -0.02 | 0.265 | 0.438 | 3249 unclassified | unclassified | unclassified | unclassified | Eubacteriales | Clostridia | Firmicutes |
| Eubacteriales sp. (HG3A.0781) | T90 | -0.02 | 0.264 | 0.438 | 3249 unclassified | unclassified | unclassified | unclassified | Eubacteriales | Clostridia | Firmicutes |
| Odoribacter splanchnicus (HG3A.0041) | T90 | -0.02 | 0.268 | 0.438 | 3249 unclassified | Odoribacter splanchnicus | Odoribacter | Odoribacteraceae | Bacteroidales | Bacteroidia | Bacteroidetes |
| Eubacteriales sp. (HG3A.0528) | ODI | -0.018 | 0.316 | 0.439 | 3249 unclassified | unclassified | unclassified | unclassified | Eubacteriales | Clostridia | Firmicutes |
| Bacteria sp. (HG3A.0911) | AHI | -0.023 | 0.221 | 0.440 | 2909 unclassified | unclassified | unclassified | unclassified | unclassified | unclassified | unclassified |
| Candidatus Borkfalkiales sp.  (HG3A.1329) | AHI | -0.023 | 0.222 | 0.440 | 2909 unclassified | unclassified | unclassified | unclassified | Candidatus  Borkfalkiales | Clostridia | Firmicutes |
| Clostridia sp. (HG3A.1157) | AHI | -0.023 | 0.221 | 0.440 | 2909 unclassified | unclassified | unclassified | unclassified | unclassified | Clostridia | Firmicutes |
| Clostridium sp. M62/1 (HG3A.0354) | AHI | 0.023 | 0.221 | 0.440 | 2909 unclassified | Clostridium sp.  M62/1 | Clostridium | Clostridiaceae | Eubacteriales | Clostridia | Firmicutes |
| Erysipelatoclostridium sp. (HG3A.0313) | AHI | -0.023 | 0.216 | 0.440 | 2909 unclassified | unclassified | Erysipelatoclostri dium | Erysipelotrichacea e | Erysipelotrichales | Erysipelotrichia | Firmicutes |

| Eubacteriales sp. (HG3A.0568) | AHI | -0.023 | 0.221 | 0.440 | 2909 unclassified | unclassified | unclassified | unclassified | Eubacteriales | Clostridia | Firmicutes |
| --- | --- | --- | --- | --- | --- | --- | --- | --- | --- | --- | --- |
| Eubacteriales sp. (HG3A.0600) | AHI | -0.023 | 0.218 | 0.440 | 2909 unclassified | unclassified | unclassified | unclassified | Eubacteriales | Clostridia | Firmicutes |
| Eubacteriales sp. (HG3A.0694) | AHI | -0.023 | 0.222 | 0.440 | 2909 unclassified | unclassified | unclassified | unclassified | Eubacteriales | Clostridia | Firmicutes |
| Eubacteriales sp. (HG3A.0715) | AHI | -0.023 | 0.22 | 0.440 | 2909 unclassified | unclassified | unclassified | unclassified | Eubacteriales | Clostridia | Firmicutes |
| Eubacteriales sp. (HG3A.0773) | AHI | -0.023 | 0.217 | 0.440 | 2909 unclassified | unclassified | unclassified | unclassified | Eubacteriales | Clostridia | Firmicutes |
| Eubacteriales sp. (HG3A.0908) | AHI | -0.023 | 0.219 | 0.440 | 2909 unclassified | unclassified | unclassified | unclassified | Eubacteriales | Clostridia | Firmicutes |
| Oscillospiraceae sp. (HG3A.0693) | AHI | -0.023 | 0.217 | 0.440 | 2909 unclassified | unclassified | unclassified | Oscillospiraceae | Eubacteriales | Clostridia | Firmicutes |
| Anaeromassilibacillus sp. An250 (HG3A.0169) | AHI | -0.023 | 0.224 | 0.443 | 2909 unclassified | Anaeromassilibacil lus sp. An250 | Anaeromassilibac illus | Oscillospiraceae | Eubacteriales | Clostridia | Firmicutes |
| Eubacteriales sp. (HG3A.0807) | AHI | -0.023 | 0.226 | 0.444 | 2909 unclassified | unclassified | unclassified | unclassified | Eubacteriales | Clostridia | Firmicutes |
| Eubacteriales sp. (HG3A.0914) | ODI | -0.018 | 0.321 | 0.444 | 3249 unclassified | unclassified | unclassified | unclassified | Eubacteriales | Clostridia | Firmicutes |
| Eubacteriales sp. (HG3A.0976) | AHI | -0.023 | 0.227 | 0.444 | 2909 unclassified | unclassified | unclassified | unclassified | Eubacteriales | Clostridia | Firmicutes |
| Haemophilus parainfluenzae (HG3A.0181) | ODI | -0.018 | 0.321 | 0.444 | 3249 unclassified | Haemophilus parainfluenzae | Haemophilus | Pasteurellaceae | Pasteurellales | Gammaproteob acteria | Proteobacteria |
| Oscillibacter sp. PEA192  (HG3A.0021) | AHI | 0.023 | 0.226 | 0.444 | 2909 unclassified | Oscillibacter sp.  PEA192 | Oscillibacter | Oscillospiraceae | Eubacteriales | Clostridia | Firmicutes |
| Blautia producta (HG3A.0619) | T90 | 0.02 | 0.273 | 0.445 | 3249 unclassified | Blautia producta | Blautia | Lachnospiraceae | Eubacteriales | Clostridia | Firmicutes |
| Blautia producta (HG3A.0619) | ODI | 0.018 | 0.324 | 0.446 | 3249 unclassified | Blautia producta | Blautia | Lachnospiraceae | Eubacteriales | Clostridia | Firmicutes |
| Dialister pneumosintes (HG3A.1496) | ODI | 0.018 | 0.324 | 0.446 | 3249 unclassified | Dialister pneumosintes | Dialister | Veillonellaceae | Veillonellales | Negativicutes | Firmicutes |
| Clostridia sp. (HG3A.1192) | ODI | -0.018 | 0.325 | 0.447 | 3249 unclassified | unclassified | unclassified | unclassified | unclassified | Clostridia | Firmicutes |
| Eubacteriales sp. (HG3A.0161) | T90 | -0.019 | 0.275 | 0.447 | 3249 unclassified | unclassified | unclassified | unclassified | Eubacteriales | Clostridia | Firmicutes |
| Faecalibacterium prausnitzii  (HG3A.0025) | AHI | 0.023 | 0.229 | 0.448 | 2909 unclassified | Faecalibacterium  prausnitzii | Faecalibacterium | Oscillospiraceae | Eubacteriales | Clostridia | Firmicutes |
| Eubacteriales sp. (HG3A.1305) | AHI | -0.023 | 0.231 | 0.449 | 2909 unclassified | unclassified | unclassified | unclassified | Eubacteriales | Clostridia | Firmicutes |
| Lachnospiraceae sp. (HG3A.0903) | AHI | -0.023 | 0.231 | 0.449 | 2909 unclassified | unclassified | unclassified | Lachnospiraceae | Eubacteriales | Clostridia | Firmicutes |
| Clostridia sp. (HG3A.1220) | ODI | -0.017 | 0.328 | 0.450 | 3249 unclassified | unclassified | unclassified | unclassified | unclassified | Clostridia | Firmicutes |
| Erysipelatoclostridium ramosum  (HG3A.0538) | ODI | 0.017 | 0.329 | 0.450 | 3249 unclassified | Erysipelatoclostridi  um ramosum | Erysipelatoclostri  dium | Erysipelotrichacea  e | Erysipelotrichales | Erysipelotrichia | Firmicutes |
| [Clostridium] spiroforme (HG3A.0259) | T90 | -0.019 | 0.278 | 0.451 | 3249 unclassified | [Clostridium] spiroforme | Erysipelatoclostri dium | Erysipelotrichacea e | Erysipelotrichales | Erysipelotrichia | Firmicutes |
| Clostridium sp. AF15-31  (HG3A.0293) | T90 | -0.019 | 0.278 | 0.451 | 3249 unclassified | Clostridium sp.  AF15-31 | Clostridium | Clostridiaceae | Eubacteriales | Clostridia | Firmicutes |
| Eubacteriales sp. (HG3A.1131) | T90 | -0.019 | 0.279 | 0.451 | 3249 unclassified | unclassified | unclassified | unclassified | Eubacteriales | Clostridia | Firmicutes |
| Eubacteriales sp. (HG3A.0308) | AHI | -0.022 | 0.234 | 0.452 | 2909 unclassified | unclassified | unclassified | unclassified | Eubacteriales | Clostridia | Firmicutes |

| Eubacterium sp. AF16-48  (HG3A.0219) | ODI | -0.017 | 0.33 | 0.452 | 3249 unclassified | Eubacterium sp.  AF16-48 | Eubacterium | Eubacteriaceae | Eubacteriales | Clostridia | Firmicutes |
| --- | --- | --- | --- | --- | --- | --- | --- | --- | --- | --- | --- |
| Oscillospiraceae sp. (HG3A.0461) | AHI | -0.022 | 0.233 | 0.452 | 2909 unclassified | unclassified | unclassified | Oscillospiraceae | Eubacteriales | Clostridia | Firmicutes |
| Enterocloster sp. (HG3A.1529) | T90 | 0.019 | 0.281 | 0.453 | 3249 unclassified | unclassified | Enterocloster | Lachnospiraceae | Eubacteriales | Clostridia | Firmicutes |
| Eubacteriales sp. (HG3A.0263) | T90 | -0.019 | 0.281 | 0.453 | 3249 unclassified | unclassified | unclassified | unclassified | Eubacteriales | Clostridia | Firmicutes |
| Haemophilus parainfluenzae (HG3A.0181) | AHI | -0.022 | 0.235 | 0.453 | 2909 unclassified | Haemophilus parainfluenzae | Haemophilus | Pasteurellaceae | Pasteurellales | Gammaproteob acteria | Proteobacteria |
| Mogibacterium kristiansenii |  |  |  |  |  | Mogibacterium |  | Clostridiales  Family XIII. |  |  |  |
| (HG3A.0522) | ODI | 0.017 | 0.332 | 0.453 | 3249 unclassified | kristiansenii | Mogibacterium | Incertae Sedis | Eubacteriales | Clostridia | Firmicutes |
| Pseudoflavonifractor sp. An184 (HG3A.0253) | AHI | -0.022 | 0.235 | 0.453 | 2909 unclassified | Pseudoflavonifracto r sp. An184 | Pseudoflavonifrac tor | Oscillospiraceae | Eubacteriales | Clostridia | Firmicutes |
| Eubacteriales sp. (HG3A.1067) | T90 | -0.019 | 0.283 | 0.454 | 3249 unclassified | unclassified | unclassified | unclassified | Eubacteriales | Clostridia | Firmicutes |
| Firmicutes sp. (HG3A.0541) | T90 | -0.019 | 0.282 | 0.454 | 3249 unclassified | unclassified | unclassified | unclassified | unclassified | unclassified | Firmicutes |
| Anaerotruncus massiliensis  (HG3A.0460) | AHI | -0.022 | 0.238 | 0.455 | 2909 unclassified | Anaerotruncus  massiliensis | Anaerotruncus | Oscillospiraceae | Eubacteriales | Clostridia | Firmicutes |
| Eubacteriales sp. (HG3A.1078) | AHI | -0.022 | 0.238 | 0.455 | 2909 unclassified | unclassified | unclassified | unclassified | Eubacteriales | Clostridia | Firmicutes |
| Firmicutes sp. (HG3A.0860) | AHI | -0.022 | 0.237 | 0.455 | 2909 unclassified | unclassified | unclassified | unclassified | unclassified | unclassified | Firmicutes |
| Allisonella histaminiformans (HG3A.0332) | AHI | 0.022 | 0.24 | 0.456 | 2909 unclassified | Allisonella histaminiformans | Allisonella | Veillonellaceae | Veillonellales | Negativicutes | Firmicutes |
| Intestinimonas butyriciproducens  (HG3A.0187) | AHI | -0.022 | 0.239 | 0.456 | 2909 unclassified | Intestinimonas  butyriciproducens | Intestinimonas | unclassified | Eubacteriales | Clostridia | Firmicutes |
| Sutterella wadsworthensis (HG3A.0143) | T90 | -0.019 | 0.285 | 0.456 | 3249 unclassified | Sutterella wadsworthensis | Sutterella | Sutterellaceae | Burkholderiales | Betaproteobacte ria | Proteobacteria |
| Veillonella rogosae (HG3A.0324) | T90 | -0.019 | 0.285 | 0.456 | 3249 unclassified | Veillonella rogosae | Veillonella | Veillonellaceae | Veillonellales | Negativicutes | Firmicutes |
| Clostridiaceae sp. (HG3A.0330) | T90 | -0.019 | 0.287 | 0.458 | 3249 unclassified | unclassified | unclassified | Clostridiaceae | Eubacteriales | Clostridia | Firmicutes |
| Erysipelatoclostridium ramosum  (HG3A.0538) | T90 | 0.019 | 0.289 | 0.459 | 3249 unclassified | Erysipelatoclostridi  um ramosum | Erysipelatoclostri  dium | Erysipelotrichacea  e | Erysipelotrichales | Erysipelotrichia | Firmicutes |
| Eubacteriales sp. (HG3A.0791) | T90 | -0.019 | 0.289 | 0.459 | 3249 unclassified | unclassified | unclassified | unclassified | Eubacteriales | Clostridia | Firmicutes |
| Eubacteriales sp. (HG3A.0062) | T90 | -0.019 | 0.292 | 0.462 | 3249 unclassified | unclassified | unclassified | unclassified | Eubacteriales | Clostridia | Firmicutes |
| Eubacteriales sp. (HG3A.1129) | AHI | -0.022 | 0.244 | 0.462 | 2909 unclassified | unclassified | unclassified | unclassified | Eubacteriales | Clostridia | Firmicutes |
| Firmicutes sp. (HG3A.0454) | AHI | -0.022 | 0.244 | 0.462 | 2909 unclassified | unclassified | unclassified | unclassified | unclassified | unclassified | Firmicutes |
| Clostridia sp. (HG3A.1427) | AHI | -0.022 | 0.245 | 0.463 | 2909 unclassified | unclassified | unclassified | unclassified | unclassified | Clostridia | Firmicutes |
| Eubacteriales sp. (HG3A.0518) | T90 | -0.019 | 0.292 | 0.463 | 3249 unclassified | unclassified | unclassified | unclassified | Eubacteriales | Clostridia | Firmicutes |
| Eubacteriales sp. (HG3A.0637) | ODI | -0.017 | 0.339 | 0.463 | 3249 unclassified | unclassified | unclassified | unclassified | Eubacteriales | Clostridia | Firmicutes |
| Intestinimonas butyriciproducens  (HG3A.0187) | ODI | -0.017 | 0.34 | 0.463 | 3249 unclassified | Intestinimonas  butyriciproducens | Intestinimonas | unclassified | Eubacteriales | Clostridia | Firmicutes |

| Clostridia sp. (HG3A.0893) | ODI | -0.017 | 0.343 | 0.464 | 3249 unclassified | unclassified | unclassified | unclassified | unclassified | Clostridia | Firmicutes |
| --- | --- | --- | --- | --- | --- | --- | --- | --- | --- | --- | --- |
| Eubacteriales sp. (HG3A.0087) | ODI | -0.017 | 0.344 | 0.464 | 3249 unclassified | unclassified | unclassified | unclassified | Eubacteriales | Clostridia | Firmicutes |
| Eubacteriales sp. (HG3A.0138) | ODI | -0.017 | 0.343 | 0.464 | 3249 unclassified | unclassified | unclassified | unclassified | Eubacteriales | Clostridia | Firmicutes |
| Eubacteriales sp. (HG3A.0543) | ODI | -0.017 | 0.343 | 0.464 | 3249 unclassified | unclassified | unclassified | unclassified | Eubacteriales | Clostridia | Firmicutes |
| Eubacteriales sp. (HG3A.0711) | ODI | -0.017 | 0.345 | 0.464 | 3249 unclassified | unclassified | unclassified | unclassified | Eubacteriales | Clostridia | Firmicutes |
| Firmicutes sp. (HG3A.0915) | ODI | -0.017 | 0.343 | 0.464 | 3249 unclassified | unclassified | unclassified | unclassified | unclassified | unclassified | Firmicutes |
| Blautia hydrogenotrophica (HG3A.0430) | AHI | 0.022 | 0.25 | 0.467 | 2909 unclassified | Blautia hydrogenotrophica | Blautia | Lachnospiraceae | Eubacteriales | Clostridia | Firmicutes |
| Clostridia sp. (HG3A.0463) | AHI | -0.022 | 0.249 | 0.467 | 2909 unclassified | unclassified | unclassified | unclassified | unclassified | Clostridia | Firmicutes |
| Eubacteriales sp. (HG3A.0178) | AHI | -0.022 | 0.249 | 0.467 | 2909 unclassified | unclassified | unclassified | unclassified | Eubacteriales | Clostridia | Firmicutes |
| Eubacteriales sp. (HG3A.0289) | AHI | -0.022 | 0.247 | 0.467 | 2909 unclassified | unclassified | unclassified | unclassified | Eubacteriales | Clostridia | Firmicutes |
| Pediococcus acidilactici (HG3A.1468) | AHI | 0.022 | 0.25 | 0.467 | 2909 unclassified | Pediococcus acidilactici | Pediococcus | Lactobacillaceae | Lactobacillales | Bacilli | Firmicutes |
| Clostridia sp. (HG3A.0550) | AHI | -0.022 | 0.251 | 0.468 | 2909 unclassified | unclassified | unclassified | unclassified | unclassified | Clostridia | Firmicutes |
| Eubacteriales sp. (HG3A.0323) | ODI | -0.017 | 0.349 | 0.468 | 3249 unclassified | unclassified | unclassified | unclassified | Eubacteriales | Clostridia | Firmicutes |
| Eubacteriales sp. (HG3A.0881) | ODI | -0.017 | 0.349 | 0.468 | 3249 unclassified | unclassified | unclassified | unclassified | Eubacteriales | Clostridia | Firmicutes |
| Lachnospiraceae sp. (HG3A.0903) | ODI | -0.017 | 0.348 | 0.468 | 3249 unclassified | unclassified | unclassified | Lachnospiraceae | Eubacteriales | Clostridia | Firmicutes |
| Oscillibacter sp. PEA192  (HG3A.0021) | T90 | 0.019 | 0.297 | 0.468 | 3249 unclassified | Oscillibacter sp.  PEA192 | Oscillibacter | Oscillospiraceae | Eubacteriales | Clostridia | Firmicutes |
| Oscillospiraceae sp. (HG3A.0805) | T90 | -0.019 | 0.297 | 0.468 | 3249 unclassified | unclassified | unclassified | Oscillospiraceae | Eubacteriales | Clostridia | Firmicutes |
| Roseburia sp. AM16-25 (HG3A.0344) | T90 | 0.019 | 0.298 | 0.468 | 3249 unclassified | Roseburia sp.  AM16-25 | Roseburia | Lachnospiraceae | Eubacteriales | Clostridia | Firmicutes |
| Eubacteriales sp. (HG3A.0654) | ODI | -0.017 | 0.35 | 0.469 | 3249 unclassified | unclassified | unclassified | unclassified | Eubacteriales | Clostridia | Firmicutes |
| Bacteria sp. (HG3A.0218) | AHI | -0.021 | 0.254 | 0.470 | 2909 unclassified | unclassified | unclassified | unclassified | unclassified | unclassified | unclassified |
| Eubacteriales sp. (HG3A.0369) | ODI | -0.017 | 0.353 | 0.470 | 3249 unclassified | unclassified | unclassified | unclassified | Eubacteriales | Clostridia | Firmicutes |
| Eubacteriales sp. (HG3A.0851) | AHI | -0.022 | 0.253 | 0.470 | 2909 unclassified | unclassified | unclassified | unclassified | Eubacteriales | Clostridia | Firmicutes |
| Eubacteriales sp. (HG3A.1393) | T90 | -0.018 | 0.299 | 0.470 | 3249 unclassified | unclassified | unclassified | unclassified | Eubacteriales | Clostridia | Firmicutes |
| Lachnospiraceae sp. (HG3A.0393) | ODI | 0.017 | 0.353 | 0.470 | 3249 unclassified | unclassified | unclassified | Lachnospiraceae | Eubacteriales | Clostridia | Firmicutes |
| Agathobaculum desmolans (HG3A.1429) | T90 | 0.018 | 0.301 | 0.471 | 3249 unclassified | Agathobaculum desmolans | Agathobaculum | Oscillospiraceae | Eubacteriales | Clostridia | Firmicutes |
| Clostridia sp. (HG3A.0885) | AHI | -0.021 | 0.255 | 0.471 | 2909 unclassified | unclassified | unclassified | unclassified | unclassified | Clostridia | Firmicutes |
| Desulfovibrionales sp. (HG3A.0266) | AHI | -0.021 | 0.257 | 0.473 | 2909 unclassified | unclassified | unclassified | unclassified | Desulfovibrionales | Deltaproteobact eria | Proteobacteria |

| Eubacteriales sp. (HG3A.0744) | AHI | -0.021 | 0.258 | 0.473 | 2909 unclassified | unclassified | unclassified | unclassified | Eubacteriales | Clostridia | Firmicutes |
| --- | --- | --- | --- | --- | --- | --- | --- | --- | --- | --- | --- |
| Oscillibacter sp. (HG3A.0046) | ODI | -0.016 | 0.355 | 0.473 | 3249 unclassified | unclassified | Oscillibacter | Oscillospiraceae | Eubacteriales | Clostridia | Firmicutes |
| Parabacteroides gordonii  (HG3A.0989) | AHI | 0.021 | 0.258 | 0.473 | 2909 unclassified | Parabacteroides  gordonii | Parabacteroides | Tannerellaceae | Bacteroidales | Bacteroidia | Bacteroidetes |
| Bacteria sp. (HG3A.0500) | ODI | -0.016 | 0.358 | 0.475 | 3249 unclassified | unclassified | unclassified | unclassified | unclassified | unclassified | unclassified |
| Eubacteriales sp. (HG3A.0878) | T90 | -0.018 | 0.304 | 0.475 | 3249 unclassified | unclassified | unclassified | unclassified | Eubacteriales | Clostridia | Firmicutes |
| Peptostreptococcaceae sp. (HG3A.0200) | ODI | -0.016 | 0.357 | 0.475 | 3249 unclassified | unclassified | unclassified | Peptostreptococcac eae | Eubacteriales | Clostridia | Firmicutes |
| Eubacteriales sp. (HG3A.1292) | ODI | -0.016 | 0.36 | 0.476 | 3249 unclassified | unclassified | unclassified | unclassified | Eubacteriales | Clostridia | Firmicutes |
| Eubacteriales sp. (HG3A.0405) | ODI | -0.016 | 0.361 | 0.478 | 3249 unclassified | unclassified | unclassified | unclassified | Eubacteriales | Clostridia | Firmicutes |
| Eubacteriales sp. (HG3A.0914) | T90 | -0.018 | 0.306 | 0.478 | 3249 unclassified | unclassified | unclassified | unclassified | Eubacteriales | Clostridia | Firmicutes |
| Clostridia sp. (HG3A.0368) | AHI | -0.021 | 0.261 | 0.479 | 2909 unclassified | unclassified | unclassified | unclassified | unclassified | Clostridia | Firmicutes |
| Bacteroides intestinalis (HG3A.0265) | T90 | -0.018 | 0.31 | 0.481 | 3249 unclassified | Bacteroides  intestinalis | Bacteroides | Bacteroidaceae | Bacteroidales | Bacteroidia | Bacteroidetes |
| Clostridia sp. (HG3A.0885) | T90 | -0.018 | 0.311 | 0.481 | 3249 unclassified | unclassified | unclassified | unclassified | unclassified | Clostridia | Firmicutes |
| Clostridiaceae sp. (HG3A.0608) | T90 | -0.018 | 0.312 | 0.481 | 3249 unclassified | unclassified | unclassified | Clostridiaceae | Eubacteriales | Clostridia | Firmicutes |
| Eubacteriales sp. (HG3A.0405) | T90 | -0.018 | 0.311 | 0.481 | 3249 unclassified | unclassified | unclassified | unclassified | Eubacteriales | Clostridia | Firmicutes |
| Eubacteriales sp. (HG3A.0685) | AHI | -0.021 | 0.263 | 0.481 | 2909 unclassified | unclassified | unclassified | unclassified | Eubacteriales | Clostridia | Firmicutes |
| Firmicutes sp. (HG3A.1082) | T90 | -0.018 | 0.311 | 0.481 | 3249 unclassified | unclassified | unclassified | unclassified | unclassified | unclassified | Firmicutes |
| Oscillospiraceae sp. (HG3A.0343) | ODI | -0.016 | 0.364 | 0.481 | 3249 unclassified | unclassified | unclassified | Oscillospiraceae | Eubacteriales | Clostridia | Firmicutes |
| Ruminococcus sp. AF17-22AC (HG3A.0208) | T90 | 0.018 | 0.31 | 0.481 | 3249 unclassified | Ruminococcus sp. AF17-22AC | Ruminococcus | Oscillospiraceae | Eubacteriales | Clostridia | Firmicutes |
| Clostridia sp. (HG3A.0521) | ODI | -0.016 | 0.366 | 0.482 | 3249 unclassified | unclassified | unclassified | unclassified | unclassified | Clostridia | Firmicutes |
| Limosilactobacillus vaginalis (HG3A.1341) | AHI | 0.021 | 0.264 | 0.482 | 2909 unclassified | Limosilactobacillus vaginalis | Limosilactobacillu s | Lactobacillaceae | Lactobacillales | Bacilli | Firmicutes |
| Turicibacter sanguinis (HG3A.0274) | AHI | -0.021 | 0.265 | 0.482 | 2909 unclassified | Turicibacter  sanguinis | Turicibacter | Turicibacteraceae | Erysipelotrichales | Erysipelotrichia | Firmicutes |
| Eubacteriales sp. (HG3A.0184) | AHI | -0.021 | 0.266 | 0.483 | 2909 unclassified | unclassified | unclassified | unclassified | Eubacteriales | Clostridia | Firmicutes |
| Firmicutes sp. (HG3A.0570) | T90 | -0.018 | 0.314 | 0.483 | 3249 unclassified | unclassified | unclassified | unclassified | unclassified | unclassified | Firmicutes |
| Bacteroides intestinalis (HG3A.0265) | AHI | -0.021 | 0.268 | 0.484 | 2909 unclassified | Bacteroides intestinalis | Bacteroides | Bacteroidaceae | Bacteroidales | Bacteroidia | Bacteroidetes |
| Eubacteriales sp. (HG3A.0711) | AHI | -0.021 | 0.268 | 0.484 | 2909 unclassified | unclassified | unclassified | unclassified | Eubacteriales | Clostridia | Firmicutes |
| Anaerostipes hadrus (HG3A.0003) | T90 | 0.018 | 0.318 | 0.485 | 3249 unclassified | Anaerostipes hadrus | Anaerostipes | Lachnospiraceae | Eubacteriales | Clostridia | Firmicutes |
| Anaerostipes sp. BG01 (HG3A.1509) | T90 | 0.018 | 0.316 | 0.485 | 3249 unclassified | Anaerostipes sp.  BG01 | Anaerostipes | Lachnospiraceae | Eubacteriales | Clostridia | Firmicutes |

| Clostridia sp. (HG3A.0783) | AHI | -0.021 | 0.269 | 0.485 | 2909 unclassified | unclassified | unclassified | unclassified | unclassified | Clostridia | Firmicutes |
| --- | --- | --- | --- | --- | --- | --- | --- | --- | --- | --- | --- |
| Eubacteriales sp. (HG3A.0264) | T90 | -0.018 | 0.318 | 0.485 | 3249 unclassified | unclassified | unclassified | unclassified | Eubacteriales | Clostridia | Firmicutes |
| Eubacteriales sp. (HG3A.0854) | AHI | 0.021 | 0.269 | 0.485 | 2909 unclassified | unclassified | unclassified | unclassified | Eubacteriales | Clostridia | Firmicutes |
| Eubacterium sp. AF22-8LB  (HG3A.0838) | T90 | 0.018 | 0.317 | 0.485 | 3249 unclassified | Eubacterium sp.  AF22-8LB | Eubacterium | Eubacteriaceae | Eubacteriales | Clostridia | Firmicutes |
| Fusobacterium  Fusobacterium nucleatum subsp. nucleatum subsp. Fusobacterium | | | | | | | | | | | |
| animalis (HG3A.1418) | T90 | 0.018 | 0.317 | 0.485 | 3249 animalis | nucleatum | Fusobacterium | Fusobacteriaceae | Fusobacteriales | Fusobacteriia | Fusobacteria |
| Eubacteriales sp. (HG3A.0535) | T90 | -0.018 | 0.319 | 0.486 | 3249 unclassified | unclassified | unclassified | unclassified | Eubacteriales | Clostridia | Firmicutes |
| Eubacteriales sp. (HG3A.0473) | T90 | -0.018 | 0.32 | 0.487 | 3249 unclassified | unclassified | unclassified | unclassified | Eubacteriales | Clostridia | Firmicutes |
| Eubacteriales sp. (HG3A.0578) | T90 | -0.018 | 0.322 | 0.488 | 3249 unclassified | unclassified | unclassified | unclassified | Eubacteriales | Clostridia | Firmicutes |
| Alistipes timonensis (HG3A.0586) | AHI | -0.021 | 0.273 | 0.489 | 2909 unclassified | Alistipes timonensis | Alistipes | Rikenellaceae | Bacteroidales | Bacteroidia | Bacteroidetes |
| Eubacteriales sp. (HG3A.0137) | T90 | 0.018 | 0.325 | 0.490 | 3249 unclassified | unclassified | unclassified | unclassified | Eubacteriales | Clostridia | Firmicutes |
| Eubacteriales sp. (HG3A.0428) | AHI | -0.021 | 0.274 | 0.490 | 2909 unclassified | unclassified | unclassified | unclassified | Eubacteriales | Clostridia | Firmicutes |
| Eubacteriales sp. (HG3A.0649) | T90 | -0.018 | 0.324 | 0.490 | 3249 unclassified | unclassified | unclassified | unclassified | Eubacteriales | Clostridia | Firmicutes |
| Eubacteriales sp. (HG3A.0964) | T90 | -0.017 | 0.325 | 0.490 | 3249 unclassified | unclassified | unclassified | unclassified | Eubacteriales | Clostridia | Firmicutes |
| Anaerotignum lactatifermentans  (HG3A.0676) | AHI | 0.02 | 0.278 | 0.493 | 2909 unclassified | Anaerotignum  lactatifermentans | Anaerotignum | Lachnospiraceae | Eubacteriales | Clostridia | Firmicutes |
| Barnesiella intestinihominis (HG3A.0055) | AHI | -0.02 | 0.276 | 0.493 | 2909 unclassified | Barnesiella intestinihominis | Barnesiella | Barnesiellaceae | Bacteroidales | Bacteroidia | Bacteroidetes |
| Eubacteriales sp. (HG3A.0148) | AHI | -0.02 | 0.278 | 0.493 | 2909 unclassified | unclassified | unclassified | unclassified | Eubacteriales | Clostridia | Firmicutes |
| Eubacteriales sp. (HG3A.0373) | AHI | -0.02 | 0.277 | 0.493 | 2909 unclassified | unclassified | unclassified | unclassified | Eubacteriales | Clostridia | Firmicutes |
| Parasutterella excrementihominis (HG3A.0159) | ODI | -0.016 | 0.375 | 0.493 | 3249 unclassified | Parasutterella excrementihominis | Parasutterella | Sutterellaceae | Burkholderiales | Betaproteobacte ria | Proteobacteria |
| Proteobacteria sp. (HG3A.0327) | AHI | -0.021 | 0.276 | 0.493 | 2909 unclassified | unclassified | unclassified | unclassified | unclassified | unclassified | Proteobacteria |
| Actinomyces sp. ICM58  (HG3A.0410) | T90 | -0.017 | 0.329 | 0.494 | 3249 unclassified | Actinomyces sp.  ICM58 | Actinomyces | Actinomycetaceae | Actinomycetales | Actinomycetia | Actinobacteria |
| Akkermansia sp. BIOML-A59 (HG3A.0800) | T90 | -0.017 | 0.331 | 0.494 | 3249 unclassified | Akkermansia sp. BIOML-A59 | Akkermansia | Akkermansiaceae | Verrucomicrobiale s | Verrucomicrobi ae | Verrucomicrobia |
| Eubacteriales sp. (HG3A.0267) | T90 | -0.017 | 0.331 | 0.494 | 3249 unclassified | unclassified | unclassified | unclassified | Eubacteriales | Clostridia | Firmicutes |
| Eubacteriales sp. (HG3A.0536) | T90 | -0.017 | 0.329 | 0.494 | 3249 unclassified | unclassified | unclassified | unclassified | Eubacteriales | Clostridia | Firmicutes |
| Oscillospiraceae sp. (HG3A.0806) | T90 | -0.017 | 0.33 | 0.494 | 3249 unclassified | unclassified | unclassified | Oscillospiraceae | Eubacteriales | Clostridia | Firmicutes |
| Eubacteriales sp. (HG3A.0985) | ODI | 0.016 | 0.377 | 0.495 | 3249 unclassified | unclassified | unclassified | unclassified | Eubacteriales | Clostridia | Firmicutes |
| Eubacteriales sp. (HG3A.0080) | AHI | 0.02 | 0.281 | 0.496 | 2909 unclassified | unclassified | unclassified | unclassified | Eubacteriales | Clostridia | Firmicutes |

| Barnesiella intestinihominis  (HG3A.0055) | T90 | -0.017 | 0.334 | 0.498 | 3249 unclassified | Barnesiella  intestinihominis | Barnesiella | Barnesiellaceae | Bacteroidales | Bacteroidia | Bacteroidetes |
| --- | --- | --- | --- | --- | --- | --- | --- | --- | --- | --- | --- |
| Clostridia sp. (HG3A.1111) | ODI | -0.016 | 0.38 | 0.498 | 3249 unclassified | unclassified | unclassified | unclassified | unclassified | Clostridia | Firmicutes |
| Eubacteriales sp. (HG3A.0924) | T90 | -0.017 | 0.335 | 0.498 | 3249 unclassified | unclassified | unclassified | unclassified | Eubacteriales | Clostridia | Firmicutes |
| Clostridia sp. (HG3A.0946) | AHI | -0.02 | 0.283 | 0.499 | 2909 unclassified | unclassified | unclassified | unclassified | unclassified | Clostridia | Firmicutes |
| Eubacteriales sp. (HG3A.0144) | AHI | -0.02 | 0.285 | 0.500 | 2909 unclassified | unclassified | unclassified | unclassified | Eubacteriales | Clostridia | Firmicutes |
| Eubacteriales sp. (HG3A.1227) | AHI | -0.02 | 0.284 | 0.500 | 2909 unclassified | unclassified | unclassified | unclassified | Eubacteriales | Clostridia | Firmicutes |
| Eubacteriales sp. (HG3A.0349) | T90 | -0.017 | 0.338 | 0.501 | 3249 unclassified | unclassified | unclassified | unclassified | Eubacteriales | Clostridia | Firmicutes |
| Oscillospiraceae sp. (HG3A.1491) | T90 | -0.017 | 0.339 | 0.501 | 3249 unclassified | unclassified | unclassified | Oscillospiraceae | Eubacteriales | Clostridia | Firmicutes |
| Alloscardovia omnicolens (HG3A.1279) | T90 | 0.017 | 0.341 | 0.503 | 3249 unclassified | Alloscardovia omnicolens | Alloscardovia | Bifidobacteriaceae | Bifidobacteriales | Actinomycetia | Actinobacteria |
| Clostridia sp. (HG3A.1417) | AHI | -0.02 | 0.289 | 0.503 | 2909 unclassified | unclassified | unclassified | unclassified | unclassified | Clostridia | Firmicutes |
| Eubacteriales sp. (HG3A.0496) | AHI | -0.02 | 0.287 | 0.503 | 2909 unclassified | unclassified | unclassified | unclassified | Eubacteriales | Clostridia | Firmicutes |
| Firmicutes sp. (HG3A.0874) | AHI | -0.02 | 0.289 | 0.503 | 2909 unclassified | unclassified | unclassified | unclassified | unclassified | unclassified | Firmicutes |
| Eubacteriales sp. (HG3A.0406) | AHI | -0.02 | 0.293 | 0.504 | 2909 unclassified | unclassified | unclassified | unclassified | Eubacteriales | Clostridia | Firmicutes |
| Eubacteriales sp. (HG3A.0518) | AHI | -0.02 | 0.289 | 0.504 | 2909 unclassified | unclassified | unclassified | unclassified | Eubacteriales | Clostridia | Firmicutes |
| Eubacteriales sp. (HG3A.0664) | T90 | 0.017 | 0.341 | 0.504 | 3249 unclassified | unclassified | unclassified | unclassified | Eubacteriales | Clostridia | Firmicutes |
| Eubacteriales sp. (HG3A.1256) | ODI | -0.015 | 0.385 | 0.504 | 3249 unclassified | unclassified | unclassified | unclassified | Eubacteriales | Clostridia | Firmicutes |
| Faecalibacterium prausnitzii (HG3A.0010) | AHI | 0.02 | 0.291 | 0.504 | 2909 unclassified | Faecalibacterium prausnitzii | Faecalibacterium | Oscillospiraceae | Eubacteriales | Clostridia | Firmicutes |
| Hydrogeniiclostidium mannosilyticum (HG3A.0294) | ODI | 0.015 | 0.385 | 0.504 | 3249 unclassified | Hydrogeniiclostidiu m mannosilyticum | Hydrogeniiclostidi um | Oscillospiraceae | Eubacteriales | Clostridia | Firmicutes |
| Methanobrevibacter smithii (HG3A.0152) | AHI | -0.02 | 0.29 | 0.504 | 2909 unclassified | Methanobrevibacte r smithii | Methanobrevibact er | Methanobacteriace ae | Methanobacteriales | Methanobacteria | Euryarchaeota |
| Phascolarctobacterium succinatutens  (HG3A.0315) | AHI | 0.02 | 0.292 | 0.504 | 2909 unclassified | Phascolarctobacter  ium succinatutens | Phascolarctobact  erium | Acidaminococcace  ae | Acidaminococcales | Negativicutes | Firmicutes |
| Roseburia faecis (HG3A.0058) | AHI | 0.02 | 0.291 | 0.504 | 2909 unclassified | Roseburia faecis | Roseburia | Lachnospiraceae | Eubacteriales | Clostridia | Firmicutes |
| Eubacteriales sp. (HG3A.0547) | ODI | -0.015 | 0.387 | 0.505 | 3249 unclassified | unclassified | unclassified | unclassified | Eubacteriales | Clostridia | Firmicutes |
| Eubacteriales sp. (HG3A.0309) | T90 | -0.017 | 0.343 | 0.506 | 3249 unclassified | unclassified | unclassified | unclassified | Eubacteriales | Clostridia | Firmicutes |
| Eubacteriales sp. (HG3A.0113) | AHI | -0.02 | 0.299 | 0.508 | 2909 unclassified | unclassified | unclassified | unclassified | Eubacteriales | Clostridia | Firmicutes |
| Eubacteriales sp. (HG3A.0234) | AHI | -0.02 | 0.297 | 0.508 | 2909 unclassified | unclassified | unclassified | unclassified | Eubacteriales | Clostridia | Firmicutes |
| Eubacteriales sp. (HG3A.0781) | AHI | -0.02 | 0.298 | 0.508 | 2909 unclassified | unclassified | unclassified | unclassified | Eubacteriales | Clostridia | Firmicutes |
| Eubacteriales sp. (HG3A.1573) | AHI | -0.02 | 0.296 | 0.508 | 2909 unclassified | unclassified | unclassified | unclassified | Eubacteriales | Clostridia | Firmicutes |
| Eubacterium sp. AF17-7  (HG3A.0165) | AHI | -0.02 | 0.296 | 0.508 | 2909 unclassified | Eubacterium sp.  AF17-7 | Eubacterium | Eubacteriaceae | Eubacteriales | Clostridia | Firmicutes |

| Eubacteriales sp. (HG3A.1094) | AHI | -0.02 | 0.3 | 0.509 | 2909 unclassified | unclassified | unclassified | unclassified | Eubacteriales | Clostridia | Firmicutes |
| --- | --- | --- | --- | --- | --- | --- | --- | --- | --- | --- | --- |
| Firmicutes sp. (HG3A.0436) | T90 | -0.017 | 0.347 | 0.509 | 3249 unclassified | unclassified | unclassified | unclassified | unclassified | unclassified | Firmicutes |
| Oscillospiraceae sp. (HG3A.0382) | AHI | -0.019 | 0.301 | 0.510 | 2909 unclassified | unclassified | unclassified | Oscillospiraceae | Eubacteriales | Clostridia | Firmicutes |
| Eubacteriales sp. (HG3A.0910) | AHI | -0.019 | 0.302 | 0.511 | 2909 unclassified | unclassified | unclassified | unclassified | Eubacteriales | Clostridia | Firmicutes |
| Oscillospiraceae sp. (HG3A.0739) | ODI | -0.015 | 0.393 | 0.512 | 3249 unclassified | unclassified | unclassified | Oscillospiraceae | Eubacteriales | Clostridia | Firmicutes |
| Eubacteriales sp. (HG3A.0260) | AHI | -0.019 | 0.304 | 0.513 | 2909 unclassified | unclassified | unclassified | unclassified | Eubacteriales | Clostridia | Firmicutes |
| Anaerotignum lactatifermentans (HG3A.0676) | ODI | 0.015 | 0.395 | 0.514 | 3249 unclassified | Anaerotignum lactatifermentans | Anaerotignum | Lachnospiraceae | Eubacteriales | Clostridia | Firmicutes |
| Clostridia sp. (HG3A.1417) | ODI | -0.015 | 0.397 | 0.514 | 3249 unclassified | unclassified | unclassified | unclassified | unclassified | Clostridia | Firmicutes |
| Eubacteriales sp. (HG3A.0498) | AHI | 0.019 | 0.305 | 0.514 | 2909 unclassified | unclassified | unclassified | unclassified | Eubacteriales | Clostridia | Firmicutes |
| Eubacteriales sp. (HG3A.0670) | T90 | -0.017 | 0.351 | 0.514 | 3249 unclassified | unclassified | unclassified | unclassified | Eubacteriales | Clostridia | Firmicutes |
| Eubacteriales sp. (HG3A.0939) | ODI | -0.015 | 0.396 | 0.514 | 3249 unclassified | unclassified | unclassified | unclassified | Eubacteriales | Clostridia | Firmicutes |
| Eubacteriales sp. (HG3A.1226) | ODI | -0.015 | 0.398 | 0.514 | 3249 unclassified | unclassified | unclassified | unclassified | Eubacteriales | Clostridia | Firmicutes |
| Eubacteriales sp. (HG3A.0158) | T90 | -0.017 | 0.352 | 0.516 | 3249 unclassified | unclassified | unclassified | unclassified | Eubacteriales | Clostridia | Firmicutes |
| Eubacteriales sp. (HG3A.0457) | ODI | -0.015 | 0.399 | 0.516 | 3249 unclassified | unclassified | unclassified | unclassified | Eubacteriales | Clostridia | Firmicutes |
| Eubacteriales sp. (HG3A.0350) | AHI | -0.019 | 0.308 | 0.517 | 2909 unclassified | unclassified | unclassified | unclassified | Eubacteriales | Clostridia | Firmicutes |
| Clostridium sp. AF34-13  (HG3A.0173) | ODI | -0.015 | 0.401 | 0.518 | 3249 unclassified | Clostridium sp.  AF34-13 | Clostridium | Clostridiaceae | Eubacteriales | Clostridia | Firmicutes |
| Erysipelatoclostridium ramosum (HG3A.0538) | AHI | 0.019 | 0.31 | 0.519 | 2909 unclassified | Erysipelatoclostridi um ramosum | Erysipelatoclostri dium | Erysipelotrichacea e | Erysipelotrichales | Erysipelotrichia | Firmicutes |
| Eubacteriales sp. (HG3A.0305) | AHI | -0.019 | 0.31 | 0.519 | 2909 unclassified | unclassified | unclassified | unclassified | Eubacteriales | Clostridia | Firmicutes |
| Eubacteriales sp. (HG3A.1285) | T90 | -0.016 | 0.356 | 0.520 | 3249 unclassified | unclassified | unclassified | unclassified | Eubacteriales | Clostridia | Firmicutes |
| Oscillospiraceae sp. (HG3A.1421) | T90 | 0.016 | 0.355 | 0.520 | 3249 unclassified | unclassified | unclassified | Oscillospiraceae | Eubacteriales | Clostridia | Firmicutes |
| Clostridia sp. (HG3A.1298) | AHI | -0.019 | 0.315 | 0.521 | 2909 unclassified | unclassified | unclassified | unclassified | unclassified | Clostridia | Firmicutes |
| Eubacteriales sp. (HG3A.0263) | AHI | -0.019 | 0.314 | 0.521 | 2909 unclassified | unclassified | unclassified | unclassified | Eubacteriales | Clostridia | Firmicutes |
| Eubacteriales sp. (HG3A.0821) | AHI | -0.019 | 0.314 | 0.521 | 2909 unclassified | unclassified | unclassified | unclassified | Eubacteriales | Clostridia | Firmicutes |
| Firmicutes sp. (HG3A.1014) | AHI | 0.019 | 0.314 | 0.521 | 2909 unclassified | unclassified | unclassified | unclassified | unclassified | unclassified | Firmicutes |
| Senegalimassilia anaerobia (HG3A.0129) | AHI | 0.019 | 0.312 | 0.521 | 2909 unclassified | Senegalimassilia anaerobia | Senegalimassilia | Coriobacteriaceae | Coriobacteriales | Coriobacteriia | Actinobacteria |
| [Clostridium] spiroforme  (HG3A.0259) | AHI | 0.019 | 0.317 | 0.522 | 2909 unclassified | [Clostridium]  spiroforme | Erysipelatoclostri  dium | Erysipelotrichacea  e | Erysipelotrichales | Erysipelotrichia | Firmicutes |

| Oscillospiraceae sp. (HG3A.0805) | AHI | -0.019 | 0.317 | 0.522 | 2909 unclassified | unclassified | unclassified | Oscillospiraceae | Eubacteriales | Clostridia | Firmicutes |
| --- | --- | --- | --- | --- | --- | --- | --- | --- | --- | --- | --- |
| Bacteria sp. (HG3A.1349) | AHI | -0.019 | 0.321 | 0.523 | 2909 unclassified | unclassified | unclassified | unclassified | unclassified | unclassified | unclassified |
| Eubacteriales sp. (HG3A.0473) | AHI | -0.019 | 0.321 | 0.523 | 2909 unclassified | unclassified | unclassified | unclassified | Eubacteriales | Clostridia | Firmicutes |
| Eubacteriales sp. (HG3A.0509) | AHI | -0.019 | 0.324 | 0.523 | 2909 unclassified | unclassified | unclassified | unclassified | Eubacteriales | Clostridia | Firmicutes |
| Eubacteriales sp. (HG3A.0537) | AHI | -0.019 | 0.32 | 0.523 | 2909 unclassified | unclassified | unclassified | unclassified | Eubacteriales | Clostridia | Firmicutes |
| Eubacteriales sp. (HG3A.0701) | T90 | -0.016 | 0.36 | 0.523 | 3249 unclassified | unclassified | unclassified | unclassified | Eubacteriales | Clostridia | Firmicutes |
| Eubacteriales sp. (HG3A.0857) | AHI | -0.019 | 0.322 | 0.523 | 2909 unclassified | unclassified | unclassified | unclassified | Eubacteriales | Clostridia | Firmicutes |
| Eubacteriales sp. (HG3A.0962) | AHI | 0.019 | 0.318 | 0.523 | 2909 unclassified | unclassified | unclassified | unclassified | Eubacteriales | Clostridia | Firmicutes |
| Firmicutes sp. (HG3A.0769) | AHI | -0.019 | 0.323 | 0.523 | 2909 unclassified | unclassified | unclassified | unclassified | unclassified | unclassified | Firmicutes |
| Lachnospiraceae sp. (HG3A.0748) | T90 | -0.016 | 0.359 | 0.523 | 3249 unclassified | unclassified | unclassified | Lachnospiraceae | Eubacteriales | Clostridia | Firmicutes |
| Oscillospiraceae sp. (HG3A.0507) | AHI | -0.019 | 0.322 | 0.523 | 2909 unclassified | unclassified | unclassified | Oscillospiraceae | Eubacteriales | Clostridia | Firmicutes |
| Oscillospiraceae sp. (HG3A.0966) | AHI | -0.019 | 0.323 | 0.523 | 2909 unclassified | unclassified | unclassified | Oscillospiraceae | Eubacteriales | Clostridia | Firmicutes |
| Ruthenibacterium lactatiformans (HG3A.0020) | AHI | 0.019 | 0.324 | 0.523 | 2909 unclassified | Ruthenibacterium lactatiformans | Ruthenibacterium | Oscillospiraceae | Eubacteriales | Clostridia | Firmicutes |
| Eubacteriales sp. (HG3A.0184) | ODI | -0.015 | 0.407 | 0.524 | 3249 unclassified | unclassified | unclassified | unclassified | Eubacteriales | Clostridia | Firmicutes |
| Hydrogeniiclostidium mannosilyticum (HG3A.0294) | AHI | 0.019 | 0.325 | 0.524 | 2909 unclassified | Hydrogeniiclostidiu m mannosilyticum | Hydrogeniiclostidi um | Oscillospiraceae | Eubacteriales | Clostridia | Firmicutes |
| Eubacteriales sp. (HG3A.0308) | T90 | 0.016 | 0.363 | 0.527 | 3249 unclassified | unclassified | unclassified | unclassified | Eubacteriales | Clostridia | Firmicutes |
| Eubacteriales sp. (HG3A.0902) | T90 | -0.016 | 0.365 | 0.528 | 3249 unclassified | unclassified | unclassified | unclassified | Eubacteriales | Clostridia | Firmicutes |
| Roseburia sp. AM16-25 (HG3A.0344) | AHI | -0.018 | 0.328 | 0.528 | 2909 unclassified | Roseburia sp.  AM16-25 | Roseburia | Lachnospiraceae | Eubacteriales | Clostridia | Firmicutes |
| Veillonella tobetsuensis (HG3A.1344) | T90 | -0.016 | 0.366 | 0.528 | 3249 unclassified | Veillonella tobetsuensis | Veillonella | Veillonellaceae | Veillonellales | Negativicutes | Firmicutes |
| Clostridia sp. (HG3A.0861) | ODI | -0.015 | 0.411 | 0.529 | 3249 unclassified | unclassified | unclassified | unclassified | unclassified | Clostridia | Firmicutes |
| Bacteroides nordii (HG3A.0290) | AHI | -0.018 | 0.33 | 0.530 | 2909 unclassified | Bacteroides nordii | Bacteroides | Bacteroidaceae | Bacteroidales | Bacteroidia | Bacteroidetes |
| Firmicutes sp. (HG3A.0948) | AHI | -0.018 | 0.333 | 0.533 | 2909 unclassified | unclassified | unclassified | unclassified | unclassified | unclassified | Firmicutes |
| Oscillospiraceae sp. (HG3A.0475) | AHI | -0.018 | 0.334 | 0.533 | 2909 unclassified | unclassified | unclassified | Oscillospiraceae | Eubacteriales | Clostridia | Firmicutes |
| Pseudoflavonifractor sp.  (HG3A.0844) | ODI | 0.014 | 0.415 | 0.533 | 3249 unclassified | unclassified | Pseudoflavonifrac  tor | Oscillospiraceae | Eubacteriales | Clostridia | Firmicutes |
| Butyricimonas virosa (HG3A.0199) | AHI | -0.018 | 0.336 | 0.535 | 2909 unclassified | Butyricimonas virosa | Butyricimonas | Odoribacteraceae | Bacteroidales | Bacteroidia | Bacteroidetes |

| Eubacteriales sp. (HG3A.0232) | AHI | -0.018 | 0.336 | 0.535 | 2909 unclassified | unclassified | unclassified | unclassified | Eubacteriales | Clostridia | Firmicutes |
| --- | --- | --- | --- | --- | --- | --- | --- | --- | --- | --- | --- |
| Eubacteriales sp. (HG3A.0859) | AHI | -0.018 | 0.337 | 0.535 | 2909 unclassified | unclassified | unclassified | unclassified | Eubacteriales | Clostridia | Firmicutes |
| Bacteroides caccae (HG3A.0066) | T90 | -0.016 | 0.371 | 0.536 | 3249 unclassified | Bacteroides caccae | Bacteroides | Bacteroidaceae | Bacteroidales | Bacteroidia | Bacteroidetes |
| Clostridia sp. (HG3A.1038) | AHI | -0.018 | 0.338 | 0.536 | 2909 unclassified | unclassified | unclassified | unclassified | unclassified | Clostridia | Firmicutes |
| Clostridium perfringens (HG3A.0959) | ODI | 0.014 | 0.418 | 0.536 | 3249 unclassified | Clostridium  perfringens | Clostridium | Clostridiaceae | Eubacteriales | Clostridia | Firmicutes |
| Eubacteriales sp. (HG3A.0392) | ODI | -0.014 | 0.42 | 0.537 | 3249 unclassified | unclassified | unclassified | unclassified | Eubacteriales | Clostridia | Firmicutes |
| Eubacteriales sp. (HG3A.1136) | ODI | -0.014 | 0.424 | 0.541 | 3249 unclassified | unclassified | unclassified | unclassified | Eubacteriales | Clostridia | Firmicutes |
| Lachnospiraceae sp. (HG3A.0831) | ODI | -0.014 | 0.424 | 0.541 | 3249 unclassified | unclassified | unclassified | Lachnospiraceae | Eubacteriales | Clostridia | Firmicutes |
| Bacteria sp. (HG3A.1096) | ODI | -0.014 | 0.426 | 0.542 | 3249 unclassified | unclassified | unclassified | unclassified | unclassified | unclassified | unclassified |
| Clostridia sp. (HG3A.0011) | AHI | 0.018 | 0.343 | 0.542 | 2909 unclassified | unclassified | unclassified | unclassified | unclassified | Clostridia | Firmicutes |
| Eubacteriales sp. (HG3A.0186) | T90 | 0.016 | 0.376 | 0.542 | 3249 unclassified | unclassified | unclassified | unclassified | Eubacteriales | Clostridia | Firmicutes |
| Firmicutes sp. (HG3A.0526) | AHI | -0.018 | 0.343 | 0.542 | 2909 unclassified | unclassified | unclassified | unclassified | unclassified | unclassified | Firmicutes |
| Lachnospiraceae sp. (HG3A.0127) | AHI | -0.018 | 0.344 | 0.542 | 2909 unclassified | unclassified | unclassified | Lachnospiraceae | Eubacteriales | Clostridia | Firmicutes |
| Anaeroglobus geminatus (HG3A.1818) | AHI | 0.018 | 0.345 | 0.543 | 2909 unclassified | Anaeroglobus geminatus | Anaeroglobus | Veillonellaceae | Veillonellales | Negativicutes | Firmicutes |
| Candidatus Borkfalkiales sp.  (HG3A.1284) | T90 | -0.016 | 0.378 | 0.543 | 3249 unclassified | unclassified | unclassified | unclassified | Candidatus  Borkfalkiales | Clostridia | Firmicutes |
| Bifidobacterium animalis subsp. |  |  |  |  | Bifidobacterium  animalis subsp. | Bifidobacterium |  |  |  |  |  |
| lactis (HG3A.0513) | AHI | -0.018 | 0.347 | 0.544 | 2909 lactis | animalis | Bifidobacterium | Bifidobacteriaceae | Bifidobacteriales | Actinomycetia | Actinobacteria |
| Blautia sp. AF19-10LB (HG3A.0157) | AHI | -0.018 | 0.348 | 0.545 | 2909 unclassified | Blautia sp. AF19-  10LB | Blautia | Lachnospiraceae | Eubacteriales | Clostridia | Firmicutes |
| Flavonifractor sp. An10 (HG3A.0495) | AHI | -0.018 | 0.348 | 0.545 | 2909 unclassified | Flavonifractor sp. An10 | Flavonifractor | Oscillospiraceae | Eubacteriales | Clostridia | Firmicutes |
| Lachnospiraceae sp. (HG3A.0127) | ODI | -0.014 | 0.429 | 0.545 | 3249 unclassified | unclassified | unclassified | Lachnospiraceae | Eubacteriales | Clostridia | Firmicutes |
| Prevotella colorans (HG3A.1470) | ODI | -0.014 | 0.43 | 0.545 | 3249 unclassified | Prevotella colorans | Prevotella | Prevotellaceae | Bacteroidales | Bacteroidia | Bacteroidetes |
| Eubacteriales sp. (HG3A.0588) | ODI | -0.014 | 0.431 | 0.546 | 3249 unclassified | unclassified | unclassified | unclassified | Eubacteriales | Clostridia | Firmicutes |
| Firmicutes sp. (HG3A.0464) | ODI | 0.014 | 0.432 | 0.547 | 3249 unclassified | unclassified | unclassified | unclassified | unclassified | unclassified | Firmicutes |

| Erysipelotrichales sp. (HG3A.0809) | AHI | 0.018 | 0.352 | 0.549 | 2909 unclassified | unclassified | unclassified | unclassified | Erysipelotrichales | Erysipelotrichia | Firmicutes |
| --- | --- | --- | --- | --- | --- | --- | --- | --- | --- | --- | --- |
| Bacteroidales sp. (HG3A.0147) | ODI | -0.014 | 0.437 | 0.552 | 3249 unclassified | unclassified | unclassified | unclassified | Bacteroidales | Bacteroidia | Bacteroidetes |
| Eubacteriales sp. (HG3A.1439) | T90 | -0.015 | 0.384 | 0.552 | 3249 unclassified | unclassified | unclassified | unclassified | Eubacteriales | Clostridia | Firmicutes |
| Streptococcus gallolyticus subsp. |  |  |  |  | Streptococcus gallolyticus  subsp. | Streptococcus |  |  |  |  |  |
| gallolyticus (HG3A.1651) | AHI | 0.017 | 0.354 | 0.552 | 2909 gallolyticus | gallolyticus | Streptococcus | Streptococcaceae | Lactobacillales | Bacilli | Firmicutes |
| Limosilactobacillus fermentum  (HG3A.0990) | T90 | 0.015 | 0.386 | 0.553 | 3249 unclassified | Limosilactobacillus  fermentum | Limosilactobacillu  s | Lactobacillaceae | Lactobacillales | Bacilli | Firmicutes |
| Bacteroides cellulosilyticus (HG3A.0108) | ODI | -0.014 | 0.439 | 0.554 | 3249 unclassified | Bacteroides cellulosilyticus | Bacteroides | Bacteroidaceae | Bacteroidales | Bacteroidia | Bacteroidetes |
| Atopobiaceae sp. (HG3A.0937) | AHI | 0.017 | 0.357 | 0.555 | 2909 unclassified | unclassified | unclassified | Atopobiaceae | Coriobacteriales | Coriobacteriia | Actinobacteria |
| Phocaeicola plebeius (HG3A.0423) | AHI | -0.017 | 0.359 | 0.557 | 2909 unclassified | Phocaeicola plebeius | Phocaeicola | unclassified | Bacteroidales | Bacteroidia | Bacteroidetes |
| Eubacteriales sp. (HG3A.0381) | T90 | -0.015 | 0.393 | 0.558 | 3249 unclassified | unclassified | unclassified | unclassified | Eubacteriales | Clostridia | Firmicutes |
| Eubacteriales sp. (HG3A.0985) | T90 | 0.015 | 0.391 | 0.558 | 3249 unclassified | unclassified | unclassified | unclassified | Eubacteriales | Clostridia | Firmicutes |
| Firmicutes sp. (HG3A.1290) | T90 | -0.015 | 0.39 | 0.558 | 3249 unclassified | unclassified | unclassified | unclassified | unclassified | unclassified | Firmicutes |
| Lachnospiraceae sp. (HG3A.0236) | T90 | -0.015 | 0.392 | 0.558 | 3249 unclassified | unclassified | unclassified | Lachnospiraceae | Eubacteriales | Clostridia | Firmicutes |
| Oscillospiraceae sp. (HG3A.0612) | T90 | -0.015 | 0.392 | 0.558 | 3249 unclassified | unclassified | unclassified | Oscillospiraceae | Eubacteriales | Clostridia | Firmicutes |
| Alistipes dispar (HG3A.0281) | AHI | -0.017 | 0.362 | 0.559 | 2909 unclassified | Alistipes dispar | Alistipes | Rikenellaceae | Bacteroidales | Bacteroidia | Bacteroidetes |
| Eubacteriales sp. (HG3A.0881) | T90 | 0.015 | 0.395 | 0.559 | 3249 unclassified | unclassified | unclassified | unclassified | Eubacteriales | Clostridia | Firmicutes |
| Eubacteriales sp. (HG3A.1030) | T90 | -0.015 | 0.395 | 0.559 | 3249 unclassified | unclassified | unclassified | unclassified | Eubacteriales | Clostridia | Firmicutes |
| Firmicutes sp. (HG3A.0923) | AHI | -0.017 | 0.362 | 0.559 | 2909 unclassified | unclassified | unclassified | unclassified | unclassified | unclassified | Firmicutes |
| Eubacteriales sp. (HG3A.0320) | AHI | -0.017 | 0.364 | 0.560 | 2909 unclassified | unclassified | unclassified | unclassified | Eubacteriales | Clostridia | Firmicutes |
| Eubacteriales sp. (HG3A.0652) | AHI | -0.017 | 0.365 | 0.560 | 2909 unclassified | unclassified | unclassified | unclassified | Eubacteriales | Clostridia | Firmicutes |
| Eubacteriales sp. (HG3A.0697) | AHI | -0.017 | 0.364 | 0.560 | 2909 unclassified | unclassified | unclassified | unclassified | Eubacteriales | Clostridia | Firmicutes |
| Eubacteriales sp. (HG3A.1354) | AHI | 0.017 | 0.366 | 0.560 | 2909 unclassified | unclassified | unclassified | unclassified | Eubacteriales | Clostridia | Firmicutes |
| Streptococcus mutans (HG3A.0677) | AHI | -0.017 | 0.365 | 0.560 | 2909 unclassified | Streptococcus mutans | Streptococcus | Streptococcaceae | Lactobacillales | Bacilli | Firmicutes |
| [Clostridium] symbiosum  (HG3A.0370) | T90 | 0.015 | 0.398 | 0.561 | 3249 unclassified | [Clostridium]  symbiosum | Lachnoclostridiu  m | Lachnospiraceae | Eubacteriales | Clostridia | Firmicutes |
| Clostridia sp. (HG3A.0519) | T90 | -0.015 | 0.398 | 0.561 | 3249 unclassified | unclassified | unclassified | unclassified | unclassified | Clostridia | Firmicutes |

| Eubacteriales sp. (HG3A.0192) | AHI | -0.017 | 0.368 | 0.561 | 2909 unclassified | unclassified | unclassified | unclassified | Eubacteriales | Clostridia | Firmicutes |
| --- | --- | --- | --- | --- | --- | --- | --- | --- | --- | --- | --- |
| Eubacteriales sp. (HG3A.0386) | AHI | -0.017 | 0.369 | 0.561 | 2909 unclassified | unclassified | unclassified | unclassified | Eubacteriales | Clostridia | Firmicutes |
| Eubacteriales sp. (HG3A.0588) | AHI | -0.017 | 0.369 | 0.561 | 2909 unclassified | unclassified | unclassified | unclassified | Eubacteriales | Clostridia | Firmicutes |
| Eubacteriales sp. (HG3A.1187) | AHI | 0.017 | 0.367 | 0.561 | 2909 unclassified | unclassified | unclassified | unclassified | Eubacteriales | Clostridia | Firmicutes |
| Clostridium sp. AF37-5 (HG3A.0076) | ODI | -0.013 | 0.449 | 0.565 | 3249 unclassified | Clostridium sp.  AF37-5 | Clostridium | Clostridiaceae | Eubacteriales | Clostridia | Firmicutes |
| Eubacteriales sp. (HG3A.0477) | T90 | -0.015 | 0.402 | 0.565 | 3249 unclassified | unclassified | unclassified | unclassified | Eubacteriales | Clostridia | Firmicutes |
| Eubacteriales sp. (HG3A.0081) | T90 | -0.015 | 0.403 | 0.566 | 3249 unclassified | unclassified | unclassified | unclassified | Eubacteriales | Clostridia | Firmicutes |
| Eubacteriales sp. (HG3A.0859) | T90 | -0.015 | 0.403 | 0.566 | 3249 unclassified | unclassified | unclassified | unclassified | Eubacteriales | Clostridia | Firmicutes |
| Eubacteriales sp. (HG3A.0878) | AHI | 0.017 | 0.373 | 0.566 | 2909 unclassified | unclassified | unclassified | unclassified | Eubacteriales | Clostridia | Firmicutes |
| Oscillospiraceae sp. (HG3A.0146) | AHI | -0.017 | 0.374 | 0.566 | 2909 unclassified | unclassified | unclassified | Oscillospiraceae | Eubacteriales | Clostridia | Firmicutes |
| Eubacteriales sp. (HG3A.0405) | AHI | -0.017 | 0.376 | 0.567 | 2909 unclassified | unclassified | unclassified | unclassified | Eubacteriales | Clostridia | Firmicutes |
| Eubacteriales sp. (HG3A.0621) | AHI | -0.017 | 0.376 | 0.567 | 2909 unclassified | unclassified | unclassified | unclassified | Eubacteriales | Clostridia | Firmicutes |
| Eubacteriales sp. (HG3A.0244) | ODI | -0.013 | 0.453 | 0.568 | 3249 unclassified | unclassified | unclassified | unclassified | Eubacteriales | Clostridia | Firmicutes |
| Eubacteriales sp. (HG3A.0614) | ODI | -0.013 | 0.452 | 0.568 | 3249 unclassified | unclassified | unclassified | unclassified | Eubacteriales | Clostridia | Firmicutes |
| Anaerobutyricum hallii (HG3A.0112) | T90 | 0.015 | 0.409 | 0.569 | 3249 unclassified | Anaerobutyricum  hallii | Anaerobutyricum | Lachnospiraceae | Eubacteriales | Clostridia | Firmicutes |
| Bacteria sp. (HG3A.1553) | T90 | -0.015 | 0.408 | 0.569 | 3249 unclassified | unclassified | unclassified | unclassified | unclassified | unclassified | unclassified |
| Eubacteriales sp. (HG3A.1191) | T90 | -0.015 | 0.408 | 0.569 | 3249 unclassified | unclassified | unclassified | unclassified | Eubacteriales | Clostridia | Firmicutes |
| Eubacteriales sp. (HG3A.1305) | T90 | -0.015 | 0.409 | 0.569 | 3249 unclassified | unclassified | unclassified | unclassified | Eubacteriales | Clostridia | Firmicutes |
| Bacteria sp. (HG3A.1545) | ODI | -0.013 | 0.457 | 0.572 | 3249 unclassified | unclassified | unclassified | unclassified | unclassified | unclassified | unclassified |
| Lachnospiraceae sp. (HG3A.1155) | T90 | -0.015 | 0.412 | 0.572 | 3249 unclassified | unclassified | unclassified | Lachnospiraceae | Eubacteriales | Clostridia | Firmicutes |
| Eubacteriales sp. (HG3A.0428) | ODI | -0.013 | 0.458 | 0.573 | 3249 unclassified | unclassified | unclassified | unclassified | Eubacteriales | Clostridia | Firmicutes |
| Bacteroidales sp. (HG3A.0340) | AHI | -0.016 | 0.383 | 0.574 | 2909 unclassified | unclassified | unclassified | unclassified | Bacteroidales | Bacteroidia | Bacteroidetes |
| Eubacteriales sp. (HG3A.1439) | AHI | 0.016 | 0.382 | 0.574 | 2909 unclassified | unclassified | unclassified | unclassified | Eubacteriales | Clostridia | Firmicutes |
| Oscillospiraceae sp. (HG3A.0765) | AHI | -0.016 | 0.384 | 0.574 | 2909 unclassified | unclassified | unclassified | Oscillospiraceae | Eubacteriales | Clostridia | Firmicutes |
| Pseudoruminococcus massiliensis  (HG3A.0346) | AHI | -0.016 | 0.381 | 0.574 | 2909 unclassified | Pseudoruminococc  us massiliensis | Pseudoruminococ  cus | Oscillospiraceae | Eubacteriales | Clostridia | Firmicutes |
| Eubacteriales sp. (HG3A.0427) | T90 | -0.014 | 0.415 | 0.575 | 3249 unclassified | unclassified | unclassified | unclassified | Eubacteriales | Clostridia | Firmicutes |
| Eubacteriales sp. (HG3A.0618) | T90 | -0.014 | 0.416 | 0.575 | 3249 unclassified | unclassified | unclassified | unclassified | Eubacteriales | Clostridia | Firmicutes |

| Prevotella sp. (HG3A.1009) | AHI | 0.016 | 0.385 | 0.575 | 2909 unclassified | unclassified | Prevotella | Prevotellaceae | Bacteroidales | Bacteroidia | Bacteroidetes |
| --- | --- | --- | --- | --- | --- | --- | --- | --- | --- | --- | --- |
| Ruminococcus sp. (HG3A.0337) | T90 | -0.014 | 0.416 | 0.575 | 3249 unclassified | unclassified | Ruminococcus | Oscillospiraceae | Eubacteriales | Clostridia | Firmicutes |
| Clostridia sp. (HG3A.0385) | AHI | -0.016 | 0.387 | 0.576 | 2909 unclassified | unclassified | unclassified | unclassified | unclassified | Clostridia | Firmicutes |
| Clostridia sp. (HG3A.0706) | T90 | -0.014 | 0.419 | 0.577 | 3249 unclassified | unclassified | unclassified | unclassified | unclassified | Clostridia | Firmicutes |
| Eubacteriales sp. (HG3A.0498) | T90 | 0.014 | 0.421 | 0.577 | 3249 unclassified | unclassified | unclassified | unclassified | Eubacteriales | Clostridia | Firmicutes |
| Eubacteriales sp. (HG3A.0973) | T90 | -0.014 | 0.421 | 0.577 | 3249 unclassified | unclassified | unclassified | unclassified | Eubacteriales | Clostridia | Firmicutes |
| Lachnoclostridium sp. (HG3A.0655) | T90 | 0.014 | 0.421 | 0.577 | 3249 unclassified | unclassified | Lachnoclostridiu m | Lachnospiraceae | Eubacteriales | Clostridia | Firmicutes |
| Streptococcus gallolyticus subsp. |  |  |  |  | Streptococcus  gallolyticus subsp. | Streptococcus |  |  |  |  |  |
| gallolyticus (HG3A.1651) | T90 | -0.014 | 0.419 | 0.577 | 3249 gallolyticus | gallolyticus | Streptococcus | Streptococcaceae | Lactobacillales | Bacilli | Firmicutes |
| Eubacteriales sp. (HG3A.1030) | AHI | -0.016 | 0.389 | 0.579 | 2909 unclassified | unclassified | unclassified | unclassified | Eubacteriales | Clostridia | Firmicutes |
| Eubacteriales sp. (HG3A.0325) | T90 | -0.014 | 0.424 | 0.580 | 3249 unclassified | unclassified | unclassified | unclassified | Eubacteriales | Clostridia | Firmicutes |
| Firmicutes sp. (HG3A.0915) | AHI | -0.016 | 0.391 | 0.580 | 2909 unclassified | unclassified | unclassified | unclassified | unclassified | unclassified | Firmicutes |
| Eubacteriales sp. (HG3A.0156) | T90 | -0.014 | 0.426 | 0.582 | 3249 unclassified | unclassified | unclassified | unclassified | Eubacteriales | Clostridia | Firmicutes |
| Clostridia sp. (HG3A.0519) | ODI | -0.013 | 0.468 | 0.583 | 3249 unclassified | unclassified | unclassified | unclassified | unclassified | Clostridia | Firmicutes |
| Firmicutes sp. (HG3A.0570) | ODI | -0.013 | 0.467 | 0.583 | 3249 unclassified | unclassified | unclassified | unclassified | unclassified | unclassified | Firmicutes |
| Eubacteriaceae sp. (HG3A.0591) | ODI | 0.013 | 0.469 | 0.584 | 3249 unclassified | unclassified | unclassified | Eubacteriaceae | Eubacteriales | Clostridia | Firmicutes |
| Clostridia sp. (HG3A.1128) | T90 | 0.014 | 0.43 | 0.585 | 3249 unclassified | unclassified | unclassified | unclassified | unclassified | Clostridia | Firmicutes |
| Eubacteriales sp. (HG3A.0671) | T90 | -0.014 | 0.429 | 0.585 | 3249 unclassified | unclassified | unclassified | unclassified | Eubacteriales | Clostridia | Firmicutes |
| Eubacteriales sp. (HG3A.0213) | T90 | -0.014 | 0.432 | 0.586 | 3249 unclassified | unclassified | unclassified | unclassified | Eubacteriales | Clostridia | Firmicutes |
| Eubacteriales sp. (HG3A.0369) | T90 | -0.014 | 0.432 | 0.586 | 3249 unclassified | unclassified | unclassified | unclassified | Eubacteriales | Clostridia | Firmicutes |
| Eubacteriales sp. (HG3A.0970) | ODI | -0.013 | 0.471 | 0.586 | 3249 unclassified | unclassified | unclassified | unclassified | Eubacteriales | Clostridia | Firmicutes |
| Clostridia sp. (HG3A.0946) | T90 | -0.014 | 0.435 | 0.587 | 3249 unclassified | unclassified | unclassified | unclassified | unclassified | Clostridia | Firmicutes |
| Clostridia sp. (HG3A.1252) | T90 | -0.014 | 0.434 | 0.587 | 3249 unclassified | unclassified | unclassified | unclassified | unclassified | Clostridia | Firmicutes |
| Eubacteriales sp. (HG3A.0647) | T90 | 0.014 | 0.435 | 0.587 | 3249 unclassified | unclassified | unclassified | unclassified | Eubacteriales | Clostridia | Firmicutes |
| Coprococcus sp. OM04-5BH  (HG3A.1028) | T90 | -0.014 | 0.437 | 0.588 | 3249 unclassified | Coprococcus sp.  OM04-5BH | Coprococcus | Lachnospiraceae | Eubacteriales | Clostridia | Firmicutes |

| Eubacteriales sp. (HG3A.1305) | ODI | -0.013 | 0.474 | 0.588 | 3249 unclassified | unclassified | unclassified | unclassified | Eubacteriales | Clostridia | Firmicutes |
| --- | --- | --- | --- | --- | --- | --- | --- | --- | --- | --- | --- |
| Streptococcus agalactiae  (HG3A.1733) | AHI | 0.016 | 0.397 | 0.588 | 2909 unclassified | Streptococcus  agalactiae | Streptococcus | Streptococcaceae | Lactobacillales | Bacilli | Firmicutes |
| Eubacteriales sp. (HG3A.0328) | T90 | -0.014 | 0.439 | 0.589 | 3249 unclassified | unclassified | unclassified | unclassified | Eubacteriales | Clostridia | Firmicutes |
| Eubacteriales sp. (HG3A.0970) | T90 | -0.014 | 0.439 | 0.589 | 3249 unclassified | unclassified | unclassified | unclassified | Eubacteriales | Clostridia | Firmicutes |
| Anaeromassilibacillus sp. An250 (HG3A.0169) | T90 | -0.014 | 0.44 | 0.590 | 3249 unclassified | Anaeromassilibacil lus sp. An250 | Anaeromassilibac illus | Oscillospiraceae | Eubacteriales | Clostridia | Firmicutes |
| Coprobacillus cateniformis  (HG3A.0456) | ODI | -0.013 | 0.478 | 0.592 | 3249 unclassified | Coprobacillus  cateniformis | Coprobacillus | Coprobacillaceae | Erysipelotrichales | Erysipelotrichia | Firmicutes |
| Eubacteriales sp. (HG3A.1199) | ODI | 0.013 | 0.479 | 0.592 | 3249 unclassified | unclassified | unclassified | unclassified | Eubacteriales | Clostridia | Firmicutes |
| Clostridia sp. (HG3A.0852) | T90 | 0.014 | 0.444 | 0.593 | 3249 unclassified | unclassified | unclassified | unclassified | unclassified | Clostridia | Firmicutes |
| Clostridia sp. (HG3A.1493) | ODI | -0.013 | 0.481 | 0.594 | 3249 unclassified | unclassified | unclassified | unclassified | unclassified | Clostridia | Firmicutes |
| Intestinimonas sp. (HG3A.1018) | AHI | 0.016 | 0.402 | 0.594 | 2909 unclassified | unclassified | Intestinimonas | unclassified | Eubacteriales | Clostridia | Firmicutes |
| Coprococcus catus (HG3A.0037) | AHI | 0.016 | 0.404 | 0.595 | 2909 unclassified | Coprococcus catus | Coprococcus | Lachnospiraceae | Eubacteriales | Clostridia | Firmicutes |
| Eubacteriales sp. (HG3A.0477) | AHI | -0.016 | 0.403 | 0.595 | 2909 unclassified | unclassified | unclassified | unclassified | Eubacteriales | Clostridia | Firmicutes |
| Streptococcus oralis subsp. oralis |  |  |  |  | Streptococcus  oralis subsp. | Streptococcus |  |  |  |  |  |
| (HG3A.0705) | T90 | 0.014 | 0.447 | 0.597 | 3249 oralis | oralis | Streptococcus | Streptococcaceae | Lactobacillales | Bacilli | Firmicutes |
| Eubacteriales sp. (HG3A.0260) | T90 | -0.013 | 0.449 | 0.598 | 3249 unclassified | unclassified | unclassified | unclassified | Eubacteriales | Clostridia | Firmicutes |
| Bacteroidales sp. (HG3A.0789) | ODI | -0.012 | 0.486 | 0.599 | 3249 unclassified | unclassified | unclassified | unclassified | Bacteroidales | Bacteroidia | Bacteroidetes |
| Peptostreptococcaceae sp.  (HG3A.0200) | AHI | -0.016 | 0.409 | 0.601 | 2909 unclassified | unclassified | unclassified | Peptostreptococcac  eae | Eubacteriales | Clostridia | Firmicutes |
| Clostridiaceae sp. (HG3A.0491) | ODI | 0.012 | 0.491 | 0.602 | 3249 unclassified | unclassified | unclassified | Clostridiaceae | Eubacteriales | Clostridia | Firmicutes |
| Dorea phocaeensis (HG3A.0865) | ODI | 0.012 | 0.492 | 0.602 | 3249 unclassified | Dorea phocaeensis | Dorea | Lachnospiraceae | Eubacteriales | Clostridia | Firmicutes |
| Eubacteriales sp. (HG3A.0350) | ODI | -0.012 | 0.489 | 0.602 | 3249 unclassified | unclassified | unclassified | unclassified | Eubacteriales | Clostridia | Firmicutes |
| Eubacteriales sp. (HG3A.0432) | ODI | -0.012 | 0.491 | 0.602 | 3249 unclassified | unclassified | unclassified | unclassified | Eubacteriales | Clostridia | Firmicutes |
| Eubacteriales sp. (HG3A.0514) | T90 | -0.013 | 0.452 | 0.602 | 3249 unclassified | unclassified | unclassified | unclassified | Eubacteriales | Clostridia | Firmicutes |
| Eubacteriales sp. (HG3A.1067) | ODI | -0.012 | 0.49 | 0.602 | 3249 unclassified | unclassified | unclassified | unclassified | Eubacteriales | Clostridia | Firmicutes |
| Actinomycetaceae sp. (HG3A.1068) | AHI | -0.015 | 0.411 | 0.604 | 2909 unclassified | unclassified | unclassified | Actinomycetaceae | Actinomycetales | Actinomycetia | Actinobacteria |
| Eubacteriales sp. (HG3A.0624) | T90 | -0.013 | 0.455 | 0.605 | 3249 unclassified | unclassified | unclassified | unclassified | Eubacteriales | Clostridia | Firmicutes |
| Eubacteriales sp. (HG3A.1134) | ODI | -0.012 | 0.496 | 0.606 | 3249 unclassified | unclassified | unclassified | unclassified | Eubacteriales | Clostridia | Firmicutes |
| Bacteroidales sp. (HG3A.1002) | AHI | -0.015 | 0.416 | 0.607 | 2909 unclassified | unclassified | unclassified | unclassified | Bacteroidales | Bacteroidia | Bacteroidetes |
| Eubacteriales sp. (HG3A.0664) | AHI | 0.015 | 0.415 | 0.607 | 2909 unclassified | unclassified | unclassified | unclassified | Eubacteriales | Clostridia | Firmicutes |
| Eubacteriales sp. (HG3A.0786) | AHI | 0.015 | 0.415 | 0.607 | 2909 unclassified | unclassified | unclassified | unclassified | Eubacteriales | Clostridia | Firmicutes |

| Anaerotruncus massiliensis  (HG3A.0460) | T90 | -0.013 | 0.459 | 0.608 | 3249 unclassified | Anaerotruncus  massiliensis | Anaerotruncus | Oscillospiraceae | Eubacteriales | Clostridia | Firmicutes |
| --- | --- | --- | --- | --- | --- | --- | --- | --- | --- | --- | --- |
| Eubacteriales sp. (HG3A.0151) | T90 | -0.013 | 0.458 | 0.608 | 3249 unclassified | unclassified | unclassified | unclassified | Eubacteriales | Clostridia | Firmicutes |
| Oscillospiraceae sp. (HG3A.0412) | T90 | -0.013 | 0.46 | 0.608 | 3249 unclassified | unclassified | unclassified | Oscillospiraceae | Eubacteriales | Clostridia | Firmicutes |
| Eubacteriales sp. (HG3A.0441) | T90 | -0.013 | 0.462 | 0.609 | 3249 unclassified | unclassified | unclassified | unclassified | Eubacteriales | Clostridia | Firmicutes |
| Eubacteriales sp. (HG3A.0457) | AHI | -0.015 | 0.418 | 0.609 | 2909 unclassified | unclassified | unclassified | unclassified | Eubacteriales | Clostridia | Firmicutes |
| Intestinimonas butyriciproducens  (HG3A.0187) | T90 | -0.013 | 0.462 | 0.609 | 3249 unclassified | Intestinimonas  butyriciproducens | Intestinimonas | unclassified | Eubacteriales | Clostridia | Firmicutes |
| Clostridium sp. AF37-5 (HG3A.0076) | AHI | -0.015 | 0.42 | 0.610 | 2909 unclassified | Clostridium sp. AF37-5 | Clostridium | Clostridiaceae | Eubacteriales | Clostridia | Firmicutes |
| Eubacteriales sp. (HG3A.0881) | AHI | -0.015 | 0.42 | 0.610 | 2909 unclassified | unclassified | unclassified | unclassified | Eubacteriales | Clostridia | Firmicutes |
| Eubacteriales sp. (HG3A.1269) | ODI | -0.012 | 0.5 | 0.610 | 3249 unclassified | unclassified | unclassified | unclassified | Eubacteriales | Clostridia | Firmicutes |
| Eubacteriales sp. (HG3A.0580) | AHI | -0.015 | 0.425 | 0.615 | 2909 unclassified | unclassified | unclassified | unclassified | Eubacteriales | Clostridia | Firmicutes |
| Clostridia sp. (HG3A.1111) | T90 | -0.013 | 0.469 | 0.616 | 3249 unclassified | unclassified | unclassified | unclassified | unclassified | Clostridia | Firmicutes |
| Clostridia sp. (HG3A.1417) | T90 | -0.013 | 0.469 | 0.616 | 3249 unclassified | unclassified | unclassified | unclassified | unclassified | Clostridia | Firmicutes |
| Bacteroidales sp. (HG3A.1236) | AHI | -0.015 | 0.43 | 0.617 | 2909 unclassified | unclassified | unclassified | unclassified | Bacteroidales | Bacteroidia | Bacteroidetes |
| Clostridia sp. (HG3A.0767) | AHI | -0.015 | 0.428 | 0.617 | 2909 unclassified | unclassified | unclassified | unclassified | unclassified | Clostridia | Firmicutes |
| Clostridium perfringens (HG3A.0959) | AHI | 0.015 | 0.431 | 0.617 | 2909 unclassified | Clostridium perfringens | Clostridium | Clostridiaceae | Eubacteriales | Clostridia | Firmicutes |
| Desulfovibrio fairfieldensis  (HG3A.0529) | AHI | -0.015 | 0.43 | 0.617 | 2909 unclassified | Desulfovibrio  fairfieldensis | Desulfovibrio | Desulfovibrionacea  e | Desulfovibrionales | Deltaproteobact  eria | Proteobacteria |
| Eubacteriales sp. (HG3A.0273) | AHI | -0.015 | 0.431 | 0.617 | 2909 unclassified | unclassified | unclassified | unclassified | Eubacteriales | Clostridia | Firmicutes |
| Eubacteriales sp. (HG3A.0613) | AHI | -0.015 | 0.43 | 0.617 | 2909 unclassified | unclassified | unclassified | unclassified | Eubacteriales | Clostridia | Firmicutes |
| Firmicutes sp. (HG3A.1290) | AHI | 0.015 | 0.433 | 0.617 | 2909 unclassified | unclassified | unclassified | unclassified | unclassified | unclassified | Firmicutes |
| Parabacteroides goldsteinii  (HG3A.0279) | AHI | -0.015 | 0.428 | 0.617 | 2909 unclassified | Parabacteroides  goldsteinii | Parabacteroides | Tannerellaceae | Bacteroidales | Bacteroidia | Bacteroidetes |
| Amedibacillus dolichus (HG3A.0798) | T90 | 0.013 | 0.472 | 0.619 | 3249 unclassified | Amedibacillus dolichus | Amedibacillus | Erysipelotrichacea e | Erysipelotrichales | Erysipelotrichia | Firmicutes |
| Eubacteriales sp. (HG3A.0751) | ODI | -0.012 | 0.51 | 0.619 | 3249 unclassified | unclassified | unclassified | unclassified | Eubacteriales | Clostridia | Firmicutes |
| Eubacteriales sp. (HG3A.0790) | AHI | -0.015 | 0.434 | 0.619 | 2909 unclassified | unclassified | unclassified | unclassified | Eubacteriales | Clostridia | Firmicutes |
| Firmicutes sp. (HG3A.0860) | ODI | -0.012 | 0.51 | 0.619 | 3249 unclassified | unclassified | unclassified | unclassified | unclassified | unclassified | Firmicutes |
| Oscillospiraceae sp. (HG3A.0665) | ODI | -0.012 | 0.509 | 0.619 | 3249 unclassified | unclassified | unclassified | Oscillospiraceae | Eubacteriales | Clostridia | Firmicutes |
| Blautia producta (HG3A.0905) | T90 | 0.013 | 0.474 | 0.620 | 3249 unclassified | Blautia producta | Blautia | Lachnospiraceae | Eubacteriales | Clostridia | Firmicutes |
| Firmicutes sp. (HG3A.1471) | ODI | -0.012 | 0.511 | 0.620 | 3249 unclassified | unclassified | unclassified | unclassified | unclassified | unclassified | Firmicutes |
| Clostridia sp. (HG3A.1252) | AHI | -0.015 | 0.437 | 0.622 | 2909 unclassified | unclassified | unclassified | unclassified | unclassified | Clostridia | Firmicutes |

| Enterocloster aldenensis  (HG3A.0362) | ODI | 0.012 | 0.514 | 0.622 | 3249 unclassified | Enterocloster  aldenensis | Enterocloster | Lachnospiraceae | Eubacteriales | Clostridia | Firmicutes |
| --- | --- | --- | --- | --- | --- | --- | --- | --- | --- | --- | --- |
| Hungatella hathewayi (HG3A.0455) | T90 | 0.013 | 0.476 | 0.622 | 3249 unclassified | Hungatella  hathewayi | Hungatella | Clostridiaceae | Eubacteriales | Clostridia | Firmicutes |
| Akkermansia sp. BIOML-A59 (HG3A.0800) | ODI | -0.012 | 0.517 | 0.624 | 3249 unclassified | Akkermansia sp. BIOML-A59 | Akkermansia | Akkermansiaceae | Verrucomicrobiale s | Verrucomicrobi ae | Verrucomicrobia |
| Erysipelotrichaceae sp. (HG3A.0867) | ODI | 0.012 | 0.517 | 0.624 | 3249 unclassified | unclassified | unclassified | Erysipelotrichacea  e | Erysipelotrichales | Erysipelotrichia | Firmicutes |
| Eubacteriales sp. (HG3A.0939) | T90 | -0.013 | 0.479 | 0.625 | 3249 unclassified | unclassified | unclassified | unclassified | Eubacteriales | Clostridia | Firmicutes |
| Victivallis lenta (HG3A.0525) | AHI | -0.014 | 0.442 | 0.627 | 2909 unclassified | Victivallis lenta | Victivallis | Victivallaceae | Victivallales | Lentisphaeria | Lentisphaerae |
| Eubacteriales sp. (HG3A.0528) | T90 | 0.013 | 0.482 | 0.628 | 3249 unclassified | unclassified | unclassified | unclassified | Eubacteriales | Clostridia | Firmicutes |
| Collinsella intestinalis (HG3A.0802) | ODI | -0.011 | 0.523 | 0.629 | 3249 unclassified | Collinsella  intestinalis | Collinsella | Coriobacteriaceae | Coriobacteriales | Coriobacteriia | Actinobacteria |
| Eubacteriales sp. (HG3A.0663) | ODI | -0.011 | 0.524 | 0.629 | 3249 unclassified | unclassified | unclassified | unclassified | Eubacteriales | Clostridia | Firmicutes |
| Eubacteriales sp. (HG3A.0762) | ODI | -0.011 | 0.525 | 0.629 | 3249 unclassified | unclassified | unclassified | unclassified | Eubacteriales | Clostridia | Firmicutes |
| Firmicutes sp. (HG3A.0769) | T90 | -0.012 | 0.483 | 0.629 | 3249 unclassified | unclassified | unclassified | unclassified | unclassified | unclassified | Firmicutes |
| Firmicutes sp. (HG3A.1048) | T90 | -0.012 | 0.485 | 0.629 | 3249 unclassified | unclassified | unclassified | unclassified | unclassified | unclassified | Firmicutes |
| Intestinimonas sp. (HG3A.1018) | ODI | 0.011 | 0.523 | 0.629 | 3249 unclassified | unclassified | Intestinimonas | unclassified | Eubacteriales | Clostridia | Firmicutes |
| Clostridia sp. (HG3A.1038) | T90 | -0.012 | 0.487 | 0.630 | 3249 unclassified | unclassified | unclassified | unclassified | unclassified | Clostridia | Firmicutes |
| Clostridium sp. AF15-31 (HG3A.0293) | AHI | -0.014 | 0.444 | 0.630 | 2909 unclassified | Clostridium sp. AF15-31 | Clostridium | Clostridiaceae | Eubacteriales | Clostridia | Firmicutes |
| Eubacteriales sp. (HG3A.0551) | ODI | -0.011 | 0.526 | 0.630 | 3249 unclassified | unclassified | unclassified | unclassified | Eubacteriales | Clostridia | Firmicutes |
| Ligilactobacillus salivarius (HG3A.0919) | T90 | 0.012 | 0.487 | 0.630 | 3249 unclassified | Ligilactobacillus salivarius | Ligilactobacillus | Lactobacillaceae | Lactobacillales | Bacilli | Firmicutes |
| Eubacteriales sp. (HG3A.0137) | ODI | -0.011 | 0.528 | 0.631 | 3249 unclassified | unclassified | unclassified | unclassified | Eubacteriales | Clostridia | Firmicutes |
| Eubacteriales sp. (HG3A.1003) | ODI | -0.011 | 0.527 | 0.631 | 3249 unclassified | unclassified | unclassified | unclassified | Eubacteriales | Clostridia | Firmicutes |
| Alloscardovia omnicolens  (HG3A.1279) | AHI | 0.014 | 0.448 | 0.632 | 2909 unclassified | Alloscardovia  omnicolens | Alloscardovia | Bifidobacteriaceae | Bifidobacteriales | Actinomycetia | Actinobacteria |
| Dorea sp. AF24-7LB (HG3A.0086) | AHI | 0.014 | 0.447 | 0.632 | 2909 unclassified | Dorea sp. AF24- 7LB | Dorea | Lachnospiraceae | Eubacteriales | Clostridia | Firmicutes |
| Eubacteriales sp. (HG3A.0138) | AHI | -0.014 | 0.448 | 0.632 | 2909 unclassified | unclassified | unclassified | unclassified | Eubacteriales | Clostridia | Firmicutes |
| Alistipes indistinctus (HG3A.0121) | AHI | -0.014 | 0.451 | 0.633 | 2909 unclassified | Alistipes indistinctus | Alistipes | Rikenellaceae | Bacteroidales | Bacteroidia | Bacteroidetes |
| Eubacteriales sp. (HG3A.0345) | AHI | -0.014 | 0.451 | 0.633 | 2909 unclassified | unclassified | unclassified | unclassified | Eubacteriales | Clostridia | Firmicutes |
| Eubacteriales sp. (HG3A.0528) | AHI | -0.014 | 0.45 | 0.633 | 2909 unclassified | unclassified | unclassified | unclassified | Eubacteriales | Clostridia | Firmicutes |
| Firmicutes sp. (HG3A.1082) | AHI | -0.014 | 0.452 | 0.633 | 2909 unclassified | unclassified | unclassified | unclassified | unclassified | unclassified | Firmicutes |
| Actinomycetaceae sp. (HG3A.1068) | T90 | 0.012 | 0.491 | 0.634 | 3249 unclassified | unclassified | unclassified | Actinomycetaceae | Actinomycetales | Actinomycetia | Actinobacteria |

| Clostridia sp. (HG3A.1410) | AHI | -0.014 | 0.454 | 0.634 | 2909 unclassified | unclassified | unclassified | unclassified | unclassified | Clostridia | Firmicutes |
| --- | --- | --- | --- | --- | --- | --- | --- | --- | --- | --- | --- |
| Eubacteriales sp. (HG3A.0364) | T90 | -0.012 | 0.492 | 0.634 | 3249 unclassified | unclassified | unclassified | unclassified | Eubacteriales | Clostridia | Firmicutes |
| Eubacteriales sp. (HG3A.0967) | T90 | -0.012 | 0.492 | 0.634 | 3249 unclassified | unclassified | unclassified | unclassified | Eubacteriales | Clostridia | Firmicutes |
| Eubacteriales sp. (HG3A.0472) | T90 | -0.012 | 0.495 | 0.635 | 3249 unclassified | unclassified | unclassified | unclassified | Eubacteriales | Clostridia | Firmicutes |
| Eubacteriales sp. (HG3A.0935) | T90 | -0.012 | 0.494 | 0.635 | 3249 unclassified | unclassified | unclassified | unclassified | Eubacteriales | Clostridia | Firmicutes |
| Faecalibacterium sp. OF04-11AC (HG3A.0070) | ODI | 0.011 | 0.533 | 0.635 | 3249 unclassified | Faecalibacterium sp. OF04-11AC | Faecalibacterium | Oscillospiraceae | Eubacteriales | Clostridia | Firmicutes |
| Eubacteriales sp. (HG3A.0175) | T90 | -0.012 | 0.496 | 0.636 | 3249 unclassified | unclassified | unclassified | unclassified | Eubacteriales | Clostridia | Firmicutes |
| Clostridium sp. AF37-5 (HG3A.0076) | T90 | -0.012 | 0.5 | 0.637 | 3249 unclassified | Clostridium sp. AF37-5 | Clostridium | Clostridiaceae | Eubacteriales | Clostridia | Firmicutes |
| Eubacteriales sp. (HG3A.1257) | T90 | -0.012 | 0.5 | 0.637 | 3249 unclassified | unclassified | unclassified | unclassified | Eubacteriales | Clostridia | Firmicutes |
| Eubacteriales sp. (HG3A.1321) | T90 | -0.012 | 0.499 | 0.637 | 3249 unclassified | unclassified | unclassified | unclassified | Eubacteriales | Clostridia | Firmicutes |
| Firmicutes sp. (HG3A.1124) | T90 | -0.012 | 0.501 | 0.637 | 3249 unclassified | unclassified | unclassified | unclassified | unclassified | unclassified | Firmicutes |
| Ligilactobacillus salivarius (HG3A.0919) | ODI | 0.011 | 0.535 | 0.637 | 3249 unclassified | Ligilactobacillus salivarius | Ligilactobacillus | Lactobacillaceae | Lactobacillales | Bacilli | Firmicutes |
| Clostridiaceae sp. (HG3A.0330) | ODI | -0.011 | 0.537 | 0.638 | 3249 unclassified | unclassified | unclassified | Clostridiaceae | Eubacteriales | Clostridia | Firmicutes |
| Eubacteriales sp. (HG3A.1292) | AHI | -0.014 | 0.459 | 0.639 | 2909 unclassified | unclassified | unclassified | unclassified | Eubacteriales | Clostridia | Firmicutes |
| Lachnospiraceae sp. (HG3A.0393) | AHI | 0.014 | 0.459 | 0.639 | 2909 unclassified | unclassified | unclassified | Lachnospiraceae | Eubacteriales | Clostridia | Firmicutes |
| Eubacteriales sp. (HG3A.0644) | T90 | -0.012 | 0.503 | 0.640 | 3249 unclassified | unclassified | unclassified | unclassified | Eubacteriales | Clostridia | Firmicutes |
| Oscillospiraceae sp. (HG3A.0507) | T90 | -0.012 | 0.505 | 0.640 | 3249 unclassified | unclassified | unclassified | Oscillospiraceae | Eubacteriales | Clostridia | Firmicutes |
| Clostridia sp. (HG3A.1139) | AHI | -0.014 | 0.462 | 0.642 | 2909 unclassified | unclassified | unclassified | unclassified | unclassified | Clostridia | Firmicutes |
| Eubacteriales sp. (HG3A.0189) | T90 | -0.012 | 0.507 | 0.642 | 3249 unclassified | unclassified | unclassified | unclassified | Eubacteriales | Clostridia | Firmicutes |
| Parabacteroides goldsteinii (HG3A.0279) | T90 | -0.012 | 0.508 | 0.642 | 3249 unclassified | Parabacteroides goldsteinii | Parabacteroides | Tannerellaceae | Bacteroidales | Bacteroidia | Bacteroidetes |
| Clostridia sp. (HG3A.0661) | T90 | -0.012 | 0.509 | 0.643 | 3249 unclassified | unclassified | unclassified | unclassified | unclassified | Clostridia | Firmicutes |
| Eubacteriales sp. (HG3A.0244) | AHI | -0.014 | 0.466 | 0.646 | 2909 unclassified | unclassified | unclassified | unclassified | Eubacteriales | Clostridia | Firmicutes |
| Eubacteriales sp. (HG3A.0589) | AHI | -0.014 | 0.465 | 0.646 | 2909 unclassified | unclassified | unclassified | unclassified | Eubacteriales | Clostridia | Firmicutes |
| Firmicutes sp. (HG3A.1050) | T90 | 0.012 | 0.513 | 0.647 | 3249 unclassified | unclassified | unclassified | unclassified | unclassified | unclassified | Firmicutes |
| Eubacteriales sp. (HG3A.0333) | T90 | -0.012 | 0.516 | 0.649 | 3249 unclassified | unclassified | unclassified | unclassified | Eubacteriales | Clostridia | Firmicutes |
| Eubacteriales sp. (HG3A.1332) | AHI | -0.014 | 0.47 | 0.650 | 2909 unclassified | unclassified | unclassified | unclassified | Eubacteriales | Clostridia | Firmicutes |
| Oscillospiraceae sp. (HG3A.0849) | ODI | -0.011 | 0.549 | 0.651 | 3249 unclassified | unclassified | unclassified | Oscillospiraceae | Eubacteriales | Clostridia | Firmicutes |
| Clostridia sp. (HG3A.0845) | T90 | -0.011 | 0.519 | 0.652 | 3249 unclassified | unclassified | unclassified | unclassified | unclassified | Clostridia | Firmicutes |
| Clostridiaceae sp. (HG3A.0471) | T90 | 0.011 | 0.519 | 0.652 | 3249 unclassified | unclassified | unclassified | Clostridiaceae | Eubacteriales | Clostridia | Firmicutes |

| Collinsella intestinalis (HG3A.0802) | T90 | 0.011 | 0.523 | 0.653 | 3249 unclassified | Collinsella  intestinalis | Collinsella | Coriobacteriaceae | Coriobacteriales | Coriobacteriia | Actinobacteria |
| --- | --- | --- | --- | --- | --- | --- | --- | --- | --- | --- | --- |
| Dorea sp. AF24-7LB (HG3A.0086) | ODI | 0.011 | 0.551 | 0.653 | 3249 unclassified | Dorea sp. AF24-  7LB | Dorea | Lachnospiraceae | Eubacteriales | Clostridia | Firmicutes |
| Eubacteriales sp. (HG3A.0148) | ODI | -0.011 | 0.552 | 0.653 | 3249 unclassified | unclassified | unclassified | unclassified | Eubacteriales | Clostridia | Firmicutes |
| Eubacteriales sp. (HG3A.0334) | T90 | -0.011 | 0.523 | 0.653 | 3249 unclassified | unclassified | unclassified | unclassified | Eubacteriales | Clostridia | Firmicutes |
| Eubacteriales sp. (HG3A.0505) | T90 | -0.011 | 0.524 | 0.653 | 3249 unclassified | unclassified | unclassified | unclassified | Eubacteriales | Clostridia | Firmicutes |
| Firmicutes sp. (HG3A.0464) | T90 | -0.011 | 0.521 | 0.653 | 3249 unclassified | unclassified | unclassified | unclassified | unclassified | unclassified | Firmicutes |
| Clostridia sp. (HG3A.1493) | T90 | -0.011 | 0.528 | 0.655 | 3249 unclassified | unclassified | unclassified | unclassified | unclassified | Clostridia | Firmicutes |
| Eubacteriales sp. (HG3A.0751) | T90 | -0.011 | 0.528 | 0.655 | 3249 unclassified | unclassified | unclassified | unclassified | Eubacteriales | Clostridia | Firmicutes |
| Lachnospiraceae sp. (HG3A.0399) | T90 | -0.011 | 0.528 | 0.655 | 3249 unclassified | unclassified | unclassified | Lachnospiraceae | Eubacteriales | Clostridia | Firmicutes |
| Proteobacteria sp. (HG3A.0327) | T90 | 0.011 | 0.527 | 0.655 | 3249 unclassified | unclassified | unclassified | unclassified | unclassified | unclassified | Proteobacteria |
| Clostridia sp. (HG3A.1058) | T90 | -0.011 | 0.531 | 0.657 | 3249 unclassified | unclassified | unclassified | unclassified | unclassified | Clostridia | Firmicutes |
| Eubacterium sp. AM49-13BH  (HG3A.0251) | T90 | -0.011 | 0.533 | 0.658 | 3249 unclassified | Eubacterium sp.  AM49-13BH | Eubacterium | Eubacteriaceae | Eubacteriales | Clostridia | Firmicutes |
| Phocaeicola plebeius (HG3A.0423) | T90 | 0.011 | 0.533 | 0.658 | 3249 unclassified | Phocaeicola plebeius | Phocaeicola | unclassified | Bacteroidales | Bacteroidia | Bacteroidetes |
| Scardovia wiggsiae (HG3A.1737) | T90 | 0.011 | 0.534 | 0.658 | 3249 unclassified | Scardovia wiggsiae | Scardovia | Bifidobacteriaceae | Bifidobacteriales | Actinomycetia | Actinobacteria |
| Erysipelotrichaceae sp. (HG3A.0867) | T90 | 0.011 | 0.538 | 0.660 | 3249 unclassified | unclassified | unclassified | Erysipelotrichacea e | Erysipelotrichales | Erysipelotrichia | Firmicutes |
| Eubacteriales sp. (HG3A.1226) | T90 | -0.011 | 0.538 | 0.660 | 3249 unclassified | unclassified | unclassified | unclassified | Eubacteriales | Clostridia | Firmicutes |
| Oscillibacter sp. (HG3A.0046) | T90 | -0.011 | 0.539 | 0.660 | 3249 unclassified | unclassified | Oscillibacter | Oscillospiraceae | Eubacteriales | Clostridia | Firmicutes |
| Clostridia sp. (HG3A.0996) | T90 | -0.011 | 0.542 | 0.662 | 3249 unclassified | unclassified | unclassified | unclassified | unclassified | Clostridia | Firmicutes |
| Lactobacillus gasseri (HG3A.0884) | T90 | 0.011 | 0.543 | 0.662 | 3249 unclassified | Lactobacillus gasseri | Lactobacillus | Lactobacillaceae | Lactobacillales | Bacilli | Firmicutes |
| Streptococcus anginosus  (HG3A.0680) | T90 | 0.011 | 0.542 | 0.662 | 3249 unclassified | Streptococcus  anginosus | Streptococcus | Streptococcaceae | Lactobacillales | Bacilli | Firmicutes |
| Eubacteriales sp. (HG3A.0335) | T90 | 0.011 | 0.544 | 0.663 | 3249 unclassified | unclassified | unclassified | unclassified | Eubacteriales | Clostridia | Firmicutes |
| Eubacteriales sp. (HG3A.0505) | ODI | -0.01 | 0.562 | 0.663 | 3249 unclassified | unclassified | unclassified | unclassified | Eubacteriales | Clostridia | Firmicutes |
| Eubacteriales sp. (HG3A.0547) | AHI | 0.013 | 0.48 | 0.663 | 2909 unclassified | unclassified | unclassified | unclassified | Eubacteriales | Clostridia | Firmicutes |
| Scardovia wiggsiae (HG3A.1737) | ODI | -0.01 | 0.562 | 0.663 | 3249 unclassified | Scardovia wiggsiae | Scardovia | Bifidobacteriaceae | Bifidobacteriales | Actinomycetia | Actinobacteria |
| Eubacteriales sp. (HG3A.0280) | T90 | -0.011 | 0.546 | 0.664 | 3249 unclassified | unclassified | unclassified | unclassified | Eubacteriales | Clostridia | Firmicutes |
| Oscillospiraceae sp. (HG3A.0380) | T90 | -0.011 | 0.547 | 0.665 | 3249 unclassified | unclassified | unclassified | Oscillospiraceae | Eubacteriales | Clostridia | Firmicutes |
| Ruminococcus sp. (HG3A.0337) | AHI | -0.013 | 0.483 | 0.665 | 2909 unclassified | unclassified | Ruminococcus | Oscillospiraceae | Eubacteriales | Clostridia | Firmicutes |

| Bacteria sp. (HG3A.0839) | T90 | -0.011 | 0.554 | 0.668 | 3249 unclassified | unclassified | unclassified | unclassified | unclassified | unclassified | unclassified |
| --- | --- | --- | --- | --- | --- | --- | --- | --- | --- | --- | --- |
| Clostridia sp. (HG3A.0909) | T90 | -0.011 | 0.552 | 0.668 | 3249 unclassified | unclassified | unclassified | unclassified | unclassified | Clostridia | Firmicutes |
| Eubacteriales sp. (HG3A.0565) | T90 | -0.011 | 0.554 | 0.668 | 3249 unclassified | unclassified | unclassified | unclassified | Eubacteriales | Clostridia | Firmicutes |
| Subdoligranulum sp. APC924/74 (HG3A.0015) | T90 | -0.011 | 0.551 | 0.668 | 3249 unclassified | Subdoligranulum sp. APC924/74 | Subdoligranulum | Oscillospiraceae | Eubacteriales | Clostridia | Firmicutes |
| Allisonella histaminiformans  (HG3A.0332) | ODI | 0.01 | 0.57 | 0.669 | 3249 unclassified | Allisonella  histaminiformans | Allisonella | Veillonellaceae | Veillonellales | Negativicutes | Firmicutes |
| Coprobacter fastidiosus (HG3A.0182) | ODI | -0.01 | 0.568 | 0.669 | 3249 unclassified | Coprobacter fastidiosus | Coprobacter | Barnesiellaceae | Bacteroidales | Bacteroidia | Bacteroidetes |
| Eubacteriales sp. (HG3A.0643) | T90 | -0.01 | 0.555 | 0.669 | 3249 unclassified | unclassified | unclassified | unclassified | Eubacteriales | Clostridia | Firmicutes |
| Eubacteriales sp. (HG3A.0851) | ODI | -0.01 | 0.57 | 0.669 | 3249 unclassified | unclassified | unclassified | unclassified | Eubacteriales | Clostridia | Firmicutes |
| Eubacteriales sp. (HG3A.1006) | T90 | -0.01 | 0.556 | 0.669 | 3249 unclassified | unclassified | unclassified | unclassified | Eubacteriales | Clostridia | Firmicutes |
| Oscillospiraceae sp. (HG3A.0765) | ODI | -0.01 | 0.57 | 0.669 | 3249 unclassified | unclassified | unclassified | Oscillospiraceae | Eubacteriales | Clostridia | Firmicutes |
| [Ruminococcus] torques  (HG3A.0088) | T90 | 0.01 | 0.561 | 0.670 | 3249 unclassified | [Ruminococcus]  torques | Mediterraneibacte  r | Lachnospiraceae | Eubacteriales | Clostridia | Firmicutes |
| Clostridia sp. (HG3A.1403) | T90 | -0.01 | 0.559 | 0.670 | 3249 unclassified | unclassified | unclassified | unclassified | unclassified | Clostridia | Firmicutes |
| Eubacteriales sp. (HG3A.0333) | ODI | -0.01 | 0.573 | 0.670 | 3249 unclassified | unclassified | unclassified | unclassified | Eubacteriales | Clostridia | Firmicutes |
| Lachnospiraceae sp. (HG3A.0903) | T90 | -0.01 | 0.559 | 0.670 | 3249 unclassified | unclassified | unclassified | Lachnospiraceae | Eubacteriales | Clostridia | Firmicutes |
| Roseburia faecis (HG3A.0058) | T90 | 0.01 | 0.561 | 0.670 | 3249 unclassified | Roseburia faecis | Roseburia | Lachnospiraceae | Eubacteriales | Clostridia | Firmicutes |
| Streptococcus sobrinus (HG3A.1366) | T90 | 0.01 | 0.561 | 0.670 | 3249 unclassified | Streptococcus sobrinus | Streptococcus | Streptococcaceae | Lactobacillales | Bacilli | Firmicutes |
| Eubacteriales sp. (HG3A.0543) | T90 | -0.01 | 0.563 | 0.671 | 3249 unclassified | unclassified | unclassified | unclassified | Eubacteriales | Clostridia | Firmicutes |
| Clostridia sp. (HG3A.1262) | AHI | -0.013 | 0.489 | 0.672 | 2909 unclassified | unclassified | unclassified | unclassified | unclassified | Clostridia | Firmicutes |
| Bacteroides nordii (HG3A.0290) | T90 | -0.01 | 0.567 | 0.674 | 3249 unclassified | Bacteroides nordii | Bacteroides | Bacteroidaceae | Bacteroidales | Bacteroidia | Bacteroidetes |
| Clostridia sp. (HG3A.1141) | T90 | -0.01 | 0.568 | 0.674 | 3249 unclassified | unclassified | unclassified | unclassified | unclassified | Clostridia | Firmicutes |
| Eubacteriales sp. (HG3A.1292) | T90 | -0.01 | 0.566 | 0.674 | 3249 unclassified | unclassified | unclassified | unclassified | Eubacteriales | Clostridia | Firmicutes |
| Oscillospiraceae sp. (HG3A.0806) | ODI | 0.01 | 0.576 | 0.674 | 3249 unclassified | unclassified | unclassified | Oscillospiraceae | Eubacteriales | Clostridia | Firmicutes |
| Clostridia sp. (HG3A.1039) | T90 | -0.01 | 0.57 | 0.676 | 3249 unclassified | unclassified | unclassified | unclassified | unclassified | Clostridia | Firmicutes |
| Clostridia sp. (HG3A.1625) | AHI | 0.013 | 0.493 | 0.677 | 2909 unclassified | unclassified | unclassified | unclassified | unclassified | Clostridia | Firmicutes |
| Eubacteriales sp. (HG3A.0692) | T90 | 0.01 | 0.572 | 0.677 | 3249 unclassified | unclassified | unclassified | unclassified | Eubacteriales | Clostridia | Firmicutes |
| Clostridia sp. (HG3A.1058) | AHI | -0.013 | 0.495 | 0.678 | 2909 unclassified | unclassified | unclassified | unclassified | unclassified | Clostridia | Firmicutes |
| Levilactobacillus brevis (HG3A.1848) | T90 | 0.01 | 0.574 | 0.678 | 3249 unclassified | Levilactobacillus  brevis | Levilactobacillus | Lactobacillaceae | Lactobacillales | Bacilli | Firmicutes |
| Eubacteriales sp. (HG3A.1445) | T90 | -0.01 | 0.576 | 0.679 | 3249 unclassified | unclassified | unclassified | unclassified | Eubacteriales | Clostridia | Firmicutes |

| [Clostridium] innocuum (HG3A.0365) | T90 | 0.01 | 0.582 | 0.681 | 3249 unclassified | [Clostridium]  innocuum | Erysipelatoclostri  dium | Erysipelotrichacea  e | Erysipelotrichales | Erysipelotrichia | Firmicutes |
| --- | --- | --- | --- | --- | --- | --- | --- | --- | --- | --- | --- |
| Clostridia sp. (HG3A.0841) | T90 | -0.01 | 0.58 | 0.681 | 3249 unclassified | unclassified | unclassified | unclassified | unclassified | Clostridia | Firmicutes |
| Clostridia sp. (HG3A.1139) | T90 | -0.01 | 0.582 | 0.681 | 3249 unclassified | unclassified | unclassified | unclassified | unclassified | Clostridia | Firmicutes |
| Erysipelotrichales sp. (HG3A.0283) | T90 | -0.01 | 0.579 | 0.681 | 3249 unclassified | unclassified | unclassified | unclassified | Erysipelotrichales | Erysipelotrichia | Firmicutes |
| Lacticaseibacillus paracasei subsp. |  |  |  |  | Lacticaseibacillus  paracasei subsp. | Lacticaseibacillus |  |  |  |  |  |
| paracasei (HG3A.0853) | ODI | 0.01 | 0.583 | 0.681 | 3249 paracasei | paracasei | Lacticaseibacillus | Lactobacillaceae | Lactobacillales | Bacilli | Firmicutes |
| Rothia mucilaginosa (HG3A.0559) | T90 | 0.01 | 0.579 | 0.681 | 3249 unclassified | Rothia mucilaginosa | Rothia | Micrococcaceae | Micrococcales | Actinomycetia | Actinobacteria |
| Clostridia sp. (HG3A.1356) | ODI | -0.01 | 0.587 | 0.682 | 3249 unclassified | unclassified | unclassified | unclassified | unclassified | Clostridia | Firmicutes |
| Eubacteriales sp. (HG3A.0973) | ODI | -0.01 | 0.585 | 0.682 | 3249 unclassified | unclassified | unclassified | unclassified | Eubacteriales | Clostridia | Firmicutes |
| Eubacteriales sp. (HG3A.1393) | ODI | -0.01 | 0.587 | 0.682 | 3249 unclassified | unclassified | unclassified | unclassified | Eubacteriales | Clostridia | Firmicutes |
| Coprococcus sp. AM27-12LB (HG3A.0687) | T90 | 0.01 | 0.588 | 0.684 | 3249 unclassified | Coprococcus sp. AM27-12LB | Coprococcus | Lachnospiraceae | Eubacteriales | Clostridia | Firmicutes |
| Eubacteriales sp. (HG3A.0188) | T90 | -0.01 | 0.587 | 0.684 | 3249 unclassified | unclassified | unclassified | unclassified | Eubacteriales | Clostridia | Firmicutes |
| Eubacteriales sp. (HG3A.0557) | T90 | -0.01 | 0.586 | 0.684 | 3249 unclassified | unclassified | unclassified | unclassified | Eubacteriales | Clostridia | Firmicutes |
| Eubacteriales sp. (HG3A.0762) | T90 | -0.01 | 0.586 | 0.684 | 3249 unclassified | unclassified | unclassified | unclassified | Eubacteriales | Clostridia | Firmicutes |
| Enterocloster clostridioformis (HG3A.0686) | T90 | 0.01 | 0.592 | 0.687 | 3249 unclassified | Enterocloster clostridioformis | Enterocloster | Lachnospiraceae | Eubacteriales | Clostridia | Firmicutes |
| Eubacteriales sp. (HG3A.0302) | T90 | 0.01 | 0.593 | 0.687 | 3249 unclassified | unclassified | unclassified | unclassified | Eubacteriales | Clostridia | Firmicutes |
| Eubacteriales sp. (HG3A.1573) | T90 | -0.01 | 0.591 | 0.687 | 3249 unclassified | unclassified | unclassified | unclassified | Eubacteriales | Clostridia | Firmicutes |
| Eubacteriales sp. (HG3A.0328) | AHI | -0.013 | 0.506 | 0.691 | 2909 unclassified | unclassified | unclassified | unclassified | Eubacteriales | Clostridia | Firmicutes |
| Eubacteriales sp. (HG3A.0592) | AHI | -0.013 | 0.506 | 0.691 | 2909 unclassified | unclassified | unclassified | unclassified | Eubacteriales | Clostridia | Firmicutes |
| Eubacteriales sp. (HG3A.0502) | T90 | -0.009 | 0.598 | 0.692 | 3249 unclassified | unclassified | unclassified | unclassified | Eubacteriales | Clostridia | Firmicutes |
| Eubacteriales sp. (HG3A.0820) | ODI | -0.009 | 0.598 | 0.692 | 3249 unclassified | unclassified | unclassified | unclassified | Eubacteriales | Clostridia | Firmicutes |
| Eubacteriales sp. (HG3A.0868) | ODI | -0.009 | 0.596 | 0.692 | 3249 unclassified | unclassified | unclassified | unclassified | Eubacteriales | Clostridia | Firmicutes |
| Desulfovibrio fairfieldensis (HG3A.0529) | T90 | 0.009 | 0.6 | 0.693 | 3249 unclassified | Desulfovibrio fairfieldensis | Desulfovibrio | Desulfovibrionacea e | Desulfovibrionales | Deltaproteobact eria | Proteobacteria |
| Eggerthellales sp. (HG3A.0848) | T90 | -0.009 | 0.603 | 0.695 | 3249 unclassified | unclassified | unclassified | unclassified | Eggerthellales | Coriobacteriia | Actinobacteria |
| Eubacteriales sp. (HG3A.0717) | AHI | -0.012 | 0.51 | 0.695 | 2909 unclassified | unclassified | unclassified | unclassified | Eubacteriales | Clostridia | Firmicutes |
| Eubacteriales sp. (HG3A.0335) | ODI | 0.009 | 0.602 | 0.696 | 3249 unclassified | unclassified | unclassified | unclassified | Eubacteriales | Clostridia | Firmicutes |
| Eubacteriales sp. (HG3A.0972) | ODI | -0.009 | 0.602 | 0.696 | 3249 unclassified | unclassified | unclassified | unclassified | Eubacteriales | Clostridia | Firmicutes |
| Clostridia sp. (HG3A.1148) | T90 | -0.009 | 0.606 | 0.697 | 3249 unclassified | unclassified | unclassified | unclassified | unclassified | Clostridia | Firmicutes |
| Eubacteriales sp. (HG3A.0970) | AHI | -0.012 | 0.512 | 0.697 | 2909 unclassified | unclassified | unclassified | unclassified | Eubacteriales | Clostridia | Firmicutes |
| Eubacteriales sp. (HG3A.1154) | T90 | 0.009 | 0.606 | 0.697 | 3249 unclassified | unclassified | unclassified | unclassified | Eubacteriales | Clostridia | Firmicutes |

| Oscillospiraceae sp. (HG3A.0412) | AHI | -0.012 | 0.513 | 0.697 | 2909 unclassified | unclassified | unclassified | Oscillospiraceae | Eubacteriales | Clostridia | Firmicutes |
| --- | --- | --- | --- | --- | --- | --- | --- | --- | --- | --- | --- |
| Eubacteriales sp. (HG3A.0617) | T90 | -0.009 | 0.608 | 0.698 | 3249 unclassified | unclassified | unclassified | unclassified | Eubacteriales | Clostridia | Firmicutes |
| Eubacteriales sp. (HG3A.0644) | ODI | -0.009 | 0.606 | 0.699 | 3249 unclassified | unclassified | unclassified | unclassified | Eubacteriales | Clostridia | Firmicutes |
| Eubacteriales sp. (HG3A.1177) | ODI | -0.009 | 0.607 | 0.699 | 3249 unclassified | unclassified | unclassified | unclassified | Eubacteriales | Clostridia | Firmicutes |
| Eubacteriales sp. (HG3A.0610) | AHI | 0.012 | 0.517 | 0.700 | 2909 unclassified | unclassified | unclassified | unclassified | Eubacteriales | Clostridia | Firmicutes |
| Firmicutes sp. (HG3A.0464) | AHI | 0.012 | 0.517 | 0.700 | 2909 unclassified | unclassified | unclassified | unclassified | unclassified | unclassified | Firmicutes |
| Lachnospiraceae sp. (HG3A.1190) | T90 | -0.009 | 0.61 | 0.700 | 3249 unclassified | unclassified | unclassified | Lachnospiraceae | Eubacteriales | Clostridia | Firmicutes |
| Coprobacter fastidiosus (HG3A.0182) | AHI | -0.012 | 0.522 | 0.702 | 2909 unclassified | Coprobacter  fastidiosus | Coprobacter | Barnesiellaceae | Bacteroidales | Bacteroidia | Bacteroidetes |
| Eubacteriales sp. (HG3A.0302) | ODI | -0.009 | 0.61 | 0.702 | 3249 unclassified | unclassified | unclassified | unclassified | Eubacteriales | Clostridia | Firmicutes |
| Eubacteriales sp. (HG3A.0358) | AHI | -0.012 | 0.521 | 0.702 | 2909 unclassified | unclassified | unclassified | unclassified | Eubacteriales | Clostridia | Firmicutes |
| Eubacteriales sp. (HG3A.0846) | AHI | 0.012 | 0.522 | 0.702 | 2909 unclassified | unclassified | unclassified | unclassified | Eubacteriales | Clostridia | Firmicutes |
| Eubacteriales sp. (HG3A.0950) | AHI | -0.012 | 0.52 | 0.702 | 2909 unclassified | unclassified | unclassified | unclassified | Eubacteriales | Clostridia | Firmicutes |
| Limosilactobacillus vaginalis (HG3A.1341) | ODI | 0.009 | 0.612 | 0.703 | 3249 unclassified | Limosilactobacillus vaginalis | Limosilactobacillu s | Lactobacillaceae | Lactobacillales | Bacilli | Firmicutes |
| Eubacteriales sp. (HG3A.0902) | AHI | -0.012 | 0.525 | 0.705 | 2909 unclassified | unclassified | unclassified | unclassified | Eubacteriales | Clostridia | Firmicutes |
| Erysipelotrichales sp. (HG3A.0809) | ODI | 0.009 | 0.618 | 0.707 | 3249 unclassified | unclassified | unclassified | unclassified | Erysipelotrichales | Erysipelotrichia | Firmicutes |
| Eubacteriales sp. (HG3A.0536) | ODI | -0.009 | 0.619 | 0.707 | 3249 unclassified | unclassified | unclassified | unclassified | Eubacteriales | Clostridia | Firmicutes |
| Streptococcus anginosus (HG3A.0680) | ODI | 0.009 | 0.616 | 0.707 | 3249 unclassified | Streptococcus anginosus | Streptococcus | Streptococcaceae | Lactobacillales | Bacilli | Firmicutes |
| Oscillospiraceae sp. (HG3A.0412) | ODI | -0.009 | 0.62 | 0.708 | 3249 unclassified | unclassified | unclassified | Oscillospiraceae | Eubacteriales | Clostridia | Firmicutes |
| Lachnospiraceae sp. (HG3A.0257) | T90 | -0.009 | 0.619 | 0.709 | 3249 unclassified | unclassified | unclassified | Lachnospiraceae | Eubacteriales | Clostridia | Firmicutes |
| Eubacteriales sp. (HG3A.1243) | ODI | -0.009 | 0.623 | 0.710 | 3249 unclassified | unclassified | unclassified | unclassified | Eubacteriales | Clostridia | Firmicutes |
| Bacteria sp. (HG3A.0361) | ODI | -0.009 | 0.627 | 0.714 | 3249 unclassified | unclassified | unclassified | unclassified | unclassified | unclassified | unclassified |
| Clostridia sp. (HG3A.0401) | AHI | -0.012 | 0.538 | 0.718 | 2909 unclassified | unclassified | unclassified | unclassified | unclassified | Clostridia | Firmicutes |
| Clostridia sp. (HG3A.0564) | T90 | -0.009 | 0.628 | 0.718 | 3249 unclassified | unclassified | unclassified | unclassified | unclassified | Clostridia | Firmicutes |
| Eubacteriales sp. (HG3A.0376) | AHI | -0.012 | 0.539 | 0.718 | 2909 unclassified | unclassified | unclassified | unclassified | Eubacteriales | Clostridia | Firmicutes |
| Eubacteriales sp. (HG3A.0493) | AHI | -0.012 | 0.537 | 0.718 | 2909 unclassified | unclassified | unclassified | unclassified | Eubacteriales | Clostridia | Firmicutes |
| Eubacteriales sp. (HG3A.0663) | T90 | -0.009 | 0.628 | 0.718 | 3249 unclassified | unclassified | unclassified | unclassified | Eubacteriales | Clostridia | Firmicutes |
| Eubacteriales sp. (HG3A.0736) | AHI | -0.012 | 0.536 | 0.718 | 2909 unclassified | unclassified | unclassified | unclassified | Eubacteriales | Clostridia | Firmicutes |
| Oxalobacter formigenes  (HG3A.1755) | AHI | 0.012 | 0.539 | 0.718 | 2909 unclassified | Oxalobacter  formigenes | Oxalobacter | Oxalobacteraceae | Burkholderiales | Betaproteobacte  ria | Proteobacteria |

| Streptococcus parasanguinis  (HG3A.0117) | T90 | 0.009 | 0.63 | 0.718 | 3249 unclassified | Streptococcus  parasanguinis | Streptococcus | Streptococcaceae | Lactobacillales | Bacilli | Firmicutes |
| --- | --- | --- | --- | --- | --- | --- | --- | --- | --- | --- | --- |
| Eubacteriales sp. (HG3A.0786) | ODI | 0.009 | 0.632 | 0.719 | 3249 unclassified | unclassified | unclassified | unclassified | Eubacteriales | Clostridia | Firmicutes |
| Hydrogeniiclostidium mannosilyticum (HG3A.0294) | T90 | 0.009 | 0.632 | 0.719 | 3249 unclassified | Hydrogeniiclostidiu m mannosilyticum | Hydrogeniiclostidi um | Oscillospiraceae | Eubacteriales | Clostridia | Firmicutes |
| Eubacteriales sp. (HG3A.1199) | AHI | 0.011 | 0.542 | 0.720 | 2909 unclassified | unclassified | unclassified | unclassified | Eubacteriales | Clostridia | Firmicutes |
| Eubacteriales sp. (HG3A.1219) | ODI | -0.008 | 0.634 | 0.720 | 3249 unclassified | unclassified | unclassified | unclassified | Eubacteriales | Clostridia | Firmicutes |
| Clostridia sp. (HG3A.1298) | T90 | 0.008 | 0.634 | 0.721 | 3249 unclassified | unclassified | unclassified | unclassified | unclassified | Clostridia | Firmicutes |
| Eubacteriales sp. (HG3A.0610) | ODI | 0.008 | 0.636 | 0.721 | 3249 unclassified | unclassified | unclassified | unclassified | Eubacteriales | Clostridia | Firmicutes |
| Eubacteriales sp. (HG3A.0544) | AHI | 0.011 | 0.544 | 0.722 | 2909 unclassified | unclassified | unclassified | unclassified | Eubacteriales | Clostridia | Firmicutes |
| Oscillospiraceae sp. (HG3A.0343) | T90 | -0.008 | 0.636 | 0.722 | 3249 unclassified | unclassified | unclassified | Oscillospiraceae | Eubacteriales | Clostridia | Firmicutes |
| Eubacteriales sp. (HG3A.1087) | T90 | -0.008 | 0.639 | 0.723 | 3249 unclassified | unclassified | unclassified | unclassified | Eubacteriales | Clostridia | Firmicutes |
| Prevotella sp. (HG3A.1040) | T90 | 0.008 | 0.638 | 0.723 | 3249 unclassified | unclassified | Prevotella | Prevotellaceae | Bacteroidales | Bacteroidia | Bacteroidetes |
| Eubacteriales sp. (HG3A.0502) | AHI | 0.011 | 0.548 | 0.726 | 2909 unclassified | unclassified | unclassified | unclassified | Eubacteriales | Clostridia | Firmicutes |
| Eubacteriales sp. (HG3A.0204) | AHI | -0.011 | 0.551 | 0.727 | 2909 unclassified | unclassified | unclassified | unclassified | Eubacteriales | Clostridia | Firmicutes |
| Eubacteriales sp. (HG3A.0468) | AHI | -0.011 | 0.55 | 0.727 | 2909 unclassified | unclassified | unclassified | unclassified | Eubacteriales | Clostridia | Firmicutes |
| Clostridia sp. (HG3A.0521) | AHI | -0.011 | 0.557 | 0.729 | 2909 unclassified | unclassified | unclassified | unclassified | unclassified | Clostridia | Firmicutes |
| Clostridia sp. (HG3A.0933) | AHI | -0.011 | 0.557 | 0.729 | 2909 unclassified | unclassified | unclassified | unclassified | unclassified | Clostridia | Firmicutes |
| Enterocloster citroniae (HG3A.0285) | AHI | 0.011 | 0.557 | 0.729 | 2909 unclassified | Enterocloster citroniae | Enterocloster | Lachnospiraceae | Eubacteriales | Clostridia | Firmicutes |
| Eubacteriales sp. (HG3A.0577) | T90 | -0.008 | 0.645 | 0.729 | 3249 unclassified | unclassified | unclassified | unclassified | Eubacteriales | Clostridia | Firmicutes |
| Oscillospiraceae sp. (HG3A.0944) | AHI | 0.011 | 0.554 | 0.729 | 2909 unclassified | unclassified | unclassified | Oscillospiraceae | Eubacteriales | Clostridia | Firmicutes |
| Pseudoflavonifractor sp.  (HG3A.0844) | AHI | 0.011 | 0.553 | 0.729 | 2909 unclassified | unclassified | Pseudoflavonifrac  tor | Oscillospiraceae | Eubacteriales | Clostridia | Firmicutes |
| Clostridia sp. (HG3A.1035) | ODI | -0.008 | 0.645 | 0.730 | 3249 unclassified | unclassified | unclassified | unclassified | unclassified | Clostridia | Firmicutes |
| Eubacteriales sp. (HG3A.0633) | AHI | -0.011 | 0.56 | 0.731 | 2909 unclassified | unclassified | unclassified | unclassified | Eubacteriales | Clostridia | Firmicutes |
| Oscillospiraceae sp. (HG3A.1588) | ODI | -0.008 | 0.647 | 0.731 | 3249 unclassified | unclassified | unclassified | Oscillospiraceae | Eubacteriales | Clostridia | Firmicutes |
| Alloscardovia omnicolens  (HG3A.1279) | ODI | 0.008 | 0.649 | 0.732 | 3249 unclassified | Alloscardovia  omnicolens | Alloscardovia | Bifidobacteriaceae | Bifidobacteriales | Actinomycetia | Actinobacteria |
| Clostridia sp. (HG3A.0861) | AHI | 0.011 | 0.562 | 0.732 | 2909 unclassified | unclassified | unclassified | unclassified | unclassified | Clostridia | Firmicutes |
| Collinsella intestinalis (HG3A.0802) | AHI | -0.011 | 0.565 | 0.732 | 2909 unclassified | Collinsella  intestinalis | Collinsella | Coriobacteriaceae | Coriobacteriales | Coriobacteriia | Actinobacteria |
| Coprococcus sp. AM27-12LB (HG3A.0687) | AHI | 0.011 | 0.563 | 0.732 | 2909 unclassified | Coprococcus sp. AM27-12LB | Coprococcus | Lachnospiraceae | Eubacteriales | Clostridia | Firmicutes |

| Eubacteriales sp. (HG3A.0514) | AHI | -0.011 | 0.563 | 0.732 | 2909 unclassified | unclassified | unclassified | unclassified | Eubacteriales | Clostridia | Firmicutes |
| --- | --- | --- | --- | --- | --- | --- | --- | --- | --- | --- | --- |
| Eubacteriales sp. (HG3A.0873) | AHI | -0.011 | 0.565 | 0.732 | 2909 unclassified | unclassified | unclassified | unclassified | Eubacteriales | Clostridia | Firmicutes |
| Firmicutes sp. (HG3A.1162) | AHI | -0.011 | 0.566 | 0.732 | 2909 unclassified | unclassified | unclassified | unclassified | unclassified | unclassified | Firmicutes |
| Lachnospiraceae sp. (HG3A.1155) | AHI | -0.011 | 0.566 | 0.732 | 2909 unclassified | unclassified | unclassified | Lachnospiraceae | Eubacteriales | Clostridia | Firmicutes |
| Oscillospiraceae sp. (HG3A.0343) | AHI | -0.011 | 0.568 | 0.732 | 2909 unclassified | unclassified | unclassified | Oscillospiraceae | Eubacteriales | Clostridia | Firmicutes |
| Bacteroidales sp. (HG3A.0340) | ODI | -0.008 | 0.653 | 0.737 | 3249 unclassified | unclassified | unclassified | unclassified | Bacteroidales | Bacteroidia | Bacteroidetes |
| Clostridia sp. (HG3A.0706) | AHI | -0.011 | 0.574 | 0.738 | 2909 unclassified | unclassified | unclassified | unclassified | unclassified | Clostridia | Firmicutes |
| Faecalibacterium prausnitzii (HG3A.0241) | AHI | -0.011 | 0.574 | 0.738 | 2909 unclassified | Faecalibacterium prausnitzii | Faecalibacterium | Oscillospiraceae | Eubacteriales | Clostridia | Firmicutes |
| Eubacteriales sp. (HG3A.0594) | ODI | -0.008 | 0.66 | 0.739 | 3249 unclassified | unclassified | unclassified | unclassified | Eubacteriales | Clostridia | Firmicutes |
| Eubacteriales sp. (HG3A.0627) | T90 | -0.008 | 0.655 | 0.739 | 3249 unclassified | unclassified | unclassified | unclassified | Eubacteriales | Clostridia | Firmicutes |
| Eubacteriales sp. (HG3A.1422) | ODI | 0.008 | 0.658 | 0.739 | 3249 unclassified | unclassified | unclassified | unclassified | Eubacteriales | Clostridia | Firmicutes |
| Oxalobacter formigenes (HG3A.1755) | ODI | 0.008 | 0.661 | 0.739 | 3249 unclassified | Oxalobacter formigenes | Oxalobacter | Oxalobacteraceae | Burkholderiales | Betaproteobacte ria | Proteobacteria |
| Parabacteroides gordonii  (HG3A.0989) | ODI | 0.008 | 0.658 | 0.739 | 3249 unclassified | Parabacteroides  gordonii | Parabacteroides | Tannerellaceae | Bacteroidales | Bacteroidia | Bacteroidetes |
| Roseburia sp. AM16-25 (HG3A.0344) | ODI | -0.008 | 0.658 | 0.739 | 3249 unclassified | Roseburia sp. AM16-25 | Roseburia | Lachnospiraceae | Eubacteriales | Clostridia | Firmicutes |
| Eubacteriales sp. (HG3A.1256) | AHI | -0.01 | 0.578 | 0.742 | 2909 unclassified | unclassified | unclassified | unclassified | Eubacteriales | Clostridia | Firmicutes |
| Anaerobutyricum hallii (HG3A.0012) | AHI | 0.01 | 0.58 | 0.743 | 2909 unclassified | Anaerobutyricum hallii | Anaerobutyricum | Lachnospiraceae | Eubacteriales | Clostridia | Firmicutes |
| Sutterella sp. KLE1602 (HG3A.0228) | T90 | 0.008 | 0.659 | 0.743 | 3249 unclassified | Sutterella sp.  KLE1602 | Sutterella | Sutterellaceae | Burkholderiales | Betaproteobacte  ria | Proteobacteria |
| Eubacteriales sp. (HG3A.0821) | T90 | -0.008 | 0.662 | 0.746 | 3249 unclassified | unclassified | unclassified | unclassified | Eubacteriales | Clostridia | Firmicutes |
| Enterocloster clostridioformis  (HG3A.0686) | ODI | 0.008 | 0.67 | 0.749 | 3249 unclassified | Enterocloster  clostridioformis | Enterocloster | Lachnospiraceae | Eubacteriales | Clostridia | Firmicutes |
| Eubacteriales sp. (HG3A.0204) | T90 | -0.008 | 0.667 | 0.750 | 3249 unclassified | unclassified | unclassified | unclassified | Eubacteriales | Clostridia | Firmicutes |
| Eubacteriales sp. (HG3A.0694) | T90 | -0.008 | 0.668 | 0.750 | 3249 unclassified | unclassified | unclassified | unclassified | Eubacteriales | Clostridia | Firmicutes |
| [Clostridium] innocuum (HG3A.0365) | AHI | 0.01 | 0.59 | 0.751 | 2909 unclassified | [Clostridium] innocuum | Erysipelatoclostri dium | Erysipelotrichacea e | Erysipelotrichales | Erysipelotrichia | Firmicutes |
| Alistipes sp. (HG3A.1385) | AHI | -0.01 | 0.593 | 0.751 | 2909 unclassified | unclassified | Alistipes | Rikenellaceae | Bacteroidales | Bacteroidia | Bacteroidetes |
| Eubacteriales sp. (HG3A.0352) | AHI | 0.01 | 0.591 | 0.751 | 2909 unclassified | unclassified | unclassified | unclassified | Eubacteriales | Clostridia | Firmicutes |
| Eubacteriales sp. (HG3A.0472) | AHI | -0.01 | 0.591 | 0.751 | 2909 unclassified | unclassified | unclassified | unclassified | Eubacteriales | Clostridia | Firmicutes |
| Lachnospiraceae sp. (HG3A.0831) | AHI | -0.01 | 0.593 | 0.751 | 2909 unclassified | unclassified | unclassified | Lachnospiraceae | Eubacteriales | Clostridia | Firmicutes |
| Oscillospiraceae sp. (HG3A.0576) | AHI | -0.01 | 0.589 | 0.751 | 2909 unclassified | unclassified | unclassified | Oscillospiraceae | Eubacteriales | Clostridia | Firmicutes |

| Ruminococcus sp. (HG3A.0126) | AHI | -0.01 | 0.589 | 0.751 | 2909 unclassified | unclassified | Ruminococcus | Oscillospiraceae | Eubacteriales | Clostridia | Firmicutes |
| --- | --- | --- | --- | --- | --- | --- | --- | --- | --- | --- | --- |
| Lachnospiraceae sp. (HG3A.0899) | T90 | -0.008 | 0.671 | 0.752 | 3249 unclassified | unclassified | unclassified | Lachnospiraceae | Eubacteriales | Clostridia | Firmicutes |
| Bacteria sp. (HG3A.1545) | AHI | -0.01 | 0.596 | 0.753 | 2909 unclassified | unclassified | unclassified | unclassified | unclassified | unclassified | unclassified |
| Clostridium sp. OM07-9AC  (HG3A.0448) | T90 | -0.008 | 0.673 | 0.753 | 3249 unclassified | Clostridium sp.  OM07-9AC | Clostridium | Clostridiaceae | Eubacteriales | Clostridia | Firmicutes |
| Turicibacter sanguinis (HG3A.0274) | T90 | -0.007 | 0.674 | 0.753 | 3249 unclassified | Turicibacter sanguinis | Turicibacter | Turicibacteraceae | Erysipelotrichales | Erysipelotrichia | Firmicutes |
| Desulfovibrionales sp. (HG3A.0266) | T90 | 0.007 | 0.675 | 0.754 | 3249 unclassified | unclassified | unclassified | unclassified | Desulfovibrionales | Deltaproteobact  eria | Proteobacteria |
| Clostridia sp. (HG3A.0909) | ODI | -0.007 | 0.681 | 0.760 | 3249 unclassified | unclassified | unclassified | unclassified | unclassified | Clostridia | Firmicutes |
| Eubacteriales sp. (HG3A.0138) | T90 | -0.007 | 0.682 | 0.760 | 3249 unclassified | unclassified | unclassified | unclassified | Eubacteriales | Clostridia | Firmicutes |
| Eubacteriales sp. (HG3A.0593) | AHI | -0.01 | 0.602 | 0.760 | 2909 unclassified | unclassified | unclassified | unclassified | Eubacteriales | Clostridia | Firmicutes |
| Erysipelotrichales sp. (HG3A.0283) | AHI | -0.01 | 0.611 | 0.761 | 2909 unclassified | unclassified | unclassified | unclassified | Erysipelotrichales | Erysipelotrichia | Firmicutes |
| Eubacteriales sp. (HG3A.0364) | AHI | -0.01 | 0.61 | 0.761 | 2909 unclassified | unclassified | unclassified | unclassified | Eubacteriales | Clostridia | Firmicutes |
| Eubacteriales sp. (HG3A.0392) | AHI | -0.01 | 0.61 | 0.761 | 2909 unclassified | unclassified | unclassified | unclassified | Eubacteriales | Clostridia | Firmicutes |
| Eubacteriales sp. (HG3A.0505) | AHI | -0.01 | 0.61 | 0.761 | 2909 unclassified | unclassified | unclassified | unclassified | Eubacteriales | Clostridia | Firmicutes |
| Eubacteriales sp. (HG3A.0754) | AHI | -0.01 | 0.605 | 0.761 | 2909 unclassified | unclassified | unclassified | unclassified | Eubacteriales | Clostridia | Firmicutes |
| Eubacteriales sp. (HG3A.1045) | AHI | -0.01 | 0.604 | 0.761 | 2909 unclassified | unclassified | unclassified | unclassified | Eubacteriales | Clostridia | Firmicutes |
| Eubacteriales sp. (HG3A.1109) | AHI | 0.01 | 0.611 | 0.761 | 2909 unclassified | unclassified | unclassified | unclassified | Eubacteriales | Clostridia | Firmicutes |
| Lachnospiraceae sp. (HG3A.1525) | AHI | -0.01 | 0.605 | 0.761 | 2909 unclassified | unclassified | unclassified | Lachnospiraceae | Eubacteriales | Clostridia | Firmicutes |
| Streptococcus parasanguinis  (HG3A.0117) | AHI | 0.01 | 0.607 | 0.761 | 2909 unclassified | Streptococcus  parasanguinis | Streptococcus | Streptococcaceae | Lactobacillales | Bacilli | Firmicutes |
| Eubacteriales sp. (HG3A.0701) | AHI | -0.009 | 0.614 | 0.763 | 2909 unclassified | unclassified | unclassified | unclassified | Eubacteriales | Clostridia | Firmicutes |
| [Clostridium] spiroforme  (HG3A.0259) | ODI | 0.007 | 0.687 | 0.766 | 3249 unclassified | [Clostridium]  spiroforme | Erysipelatoclostri  dium | Erysipelotrichacea  e | Erysipelotrichales | Erysipelotrichia | Firmicutes |
| Evtepia gabavorous (HG3A.0114) | ODI | 0.007 | 0.689 | 0.767 | 3249 unclassified | Evtepia gabavorous | Evtepia | unclassified | Eubacteriales | Clostridia | Firmicutes |
| Bifidobacterium animalis subsp. |  |  |  |  | Bifidobacterium  animalis subsp. | Bifidobacterium |  |  |  |  |  |
| lactis (HG3A.0513) | ODI | -0.007 | 0.691 | 0.768 | 3249 lactis | animalis | Bifidobacterium | Bifidobacteriaceae | Bifidobacteriales | Actinomycetia | Actinobacteria |
| Firmicutes sp. (HG3A.1048) | ODI | -0.007 | 0.692 | 0.768 | 3249 unclassified | unclassified | unclassified | unclassified | unclassified | unclassified | Firmicutes |
| Clostridium sp. AF15-31  (HG3A.0293) | ODI | -0.007 | 0.694 | 0.769 | 3249 unclassified | Clostridium sp.  AF15-31 | Clostridium | Clostridiaceae | Eubacteriales | Clostridia | Firmicutes |
| Ruthenibacterium lactatiformans (HG3A.0020) | ODI | 0.007 | 0.694 | 0.769 | 3249 unclassified | Ruthenibacterium lactatiformans | Ruthenibacterium | Oscillospiraceae | Eubacteriales | Clostridia | Firmicutes |
| Clostridiaceae sp. (HG3A.0491) | T90 | 0.007 | 0.692 | 0.770 | 3249 unclassified | unclassified | unclassified | Clostridiaceae | Eubacteriales | Clostridia | Firmicutes |

| Akkermansia muciniphila  (HG3A.0110) | AHI | -0.009 | 0.624 | 0.771 | 2909 unclassified | Akkermansia  muciniphila | Akkermansia | Akkermansiaceae | Verrucomicrobiale  s | Verrucomicrobi  ae | Verrucomicrobia |
| --- | --- | --- | --- | --- | --- | --- | --- | --- | --- | --- | --- |
| Clostridia sp. (HG3A.1427) | T90 | -0.007 | 0.695 | 0.771 | 3249 unclassified | unclassified | unclassified | unclassified | unclassified | Clostridia | Firmicutes |
| Eubacteriales sp. (HG3A.0302) | AHI | 0.009 | 0.622 | 0.771 | 2909 unclassified | unclassified | unclassified | unclassified | Eubacteriales | Clostridia | Firmicutes |
| Eubacteriales sp. (HG3A.0338) | AHI | -0.009 | 0.622 | 0.771 | 2909 unclassified | unclassified | unclassified | unclassified | Eubacteriales | Clostridia | Firmicutes |
| Eubacteriales sp. (HG3A.0639) | AHI | -0.009 | 0.624 | 0.771 | 2909 unclassified | unclassified | unclassified | unclassified | Eubacteriales | Clostridia | Firmicutes |
| Eubacteriales sp. (HG3A.1006) | ODI | -0.007 | 0.698 | 0.771 | 3249 unclassified | unclassified | unclassified | unclassified | Eubacteriales | Clostridia | Firmicutes |
| Eubacteriales sp. (HG3A.1269) | AHI | -0.009 | 0.625 | 0.771 | 2909 unclassified | unclassified | unclassified | unclassified | Eubacteriales | Clostridia | Firmicutes |
| Streptococcus mutans (HG3A.0677) | T90 | 0.007 | 0.695 | 0.771 | 3249 unclassified | Streptococcus  mutans | Streptococcus | Streptococcaceae | Lactobacillales | Bacilli | Firmicutes |
| Eubacteriales sp. (HG3A.1546) | ODI | -0.007 | 0.699 | 0.772 | 3249 unclassified | unclassified | unclassified | unclassified | Eubacteriales | Clostridia | Firmicutes |
| Eubacteriaceae sp. (HG3A.0591) | AHI | 0.009 | 0.628 | 0.773 | 2909 unclassified | unclassified | unclassified | Eubacteriaceae | Eubacteriales | Clostridia | Firmicutes |
| Eubacteriales sp. (HG3A.0163) | AHI | -0.009 | 0.63 | 0.774 | 2909 unclassified | unclassified | unclassified | unclassified | Eubacteriales | Clostridia | Firmicutes |
| Eubacteriales sp. (HG3A.1051) | AHI | 0.009 | 0.632 | 0.774 | 2909 unclassified | unclassified | unclassified | unclassified | Eubacteriales | Clostridia | Firmicutes |
| Traorella massiliensis (HG3A.0669) | AHI | 0.009 | 0.632 | 0.774 | 2909 unclassified | Traorella massiliensis | Traorella | Erysipelotrichacea e | Erysipelotrichales | Erysipelotrichia | Firmicutes |
| Clostridia sp. (HG3A.0750) | T90 | -0.007 | 0.7 | 0.776 | 3249 unclassified | unclassified | unclassified | unclassified | unclassified | Clostridia | Firmicutes |
| Clostridia sp. (HG3A.1192) | AHI | -0.009 | 0.635 | 0.776 | 2909 unclassified | unclassified | unclassified | unclassified | unclassified | Clostridia | Firmicutes |
| Clostridia sp. (HG3A.1220) | AHI | -0.009 | 0.634 | 0.776 | 2909 unclassified | unclassified | unclassified | unclassified | unclassified | Clostridia | Firmicutes |
| Clostridia sp. (HG3A.0893) | AHI | -0.009 | 0.637 | 0.777 | 2909 unclassified | unclassified | unclassified | unclassified | unclassified | Clostridia | Firmicutes |
| Eubacteriales sp. (HG3A.0551) | AHI | -0.009 | 0.638 | 0.777 | 2909 unclassified | unclassified | unclassified | unclassified | Eubacteriales | Clostridia | Firmicutes |
| Bacteria sp. (HG3A.1096) | AHI | -0.009 | 0.64 | 0.778 | 2909 unclassified | unclassified | unclassified | unclassified | unclassified | unclassified | unclassified |
| Clostridium sp. OF03-18AA  (HG3A.0119) | AHI | -0.009 | 0.643 | 0.779 | 2909 unclassified | Clostridium sp.  OF03-18AA | Clostridium | Clostridiaceae | Eubacteriales | Clostridia | Firmicutes |
| Eubacteriales sp. (HG3A.0628) | AHI | -0.009 | 0.643 | 0.779 | 2909 unclassified | unclassified | unclassified | unclassified | Eubacteriales | Clostridia | Firmicutes |
| Clostridia sp. (HG3A.1254) | T90 | -0.007 | 0.707 | 0.783 | 3249 unclassified | unclassified | unclassified | unclassified | unclassified | Clostridia | Firmicutes |
| Eubacteriales sp. (HG3A.0432) | AHI | 0.009 | 0.649 | 0.784 | 2909 unclassified | unclassified | unclassified | unclassified | Eubacteriales | Clostridia | Firmicutes |
| Pediococcus pentosaceus  (HG3A.1246) | AHI | 0.009 | 0.648 | 0.784 | 2909 unclassified | Pediococcus  pentosaceus | Pediococcus | Lactobacillaceae | Lactobacillales | Bacilli | Firmicutes |
| Atopobiaceae sp. (HG3A.0937) | ODI | 0.007 | 0.713 | 0.785 | 3249 unclassified | unclassified | unclassified | Atopobiaceae | Coriobacteriales | Coriobacteriia | Actinobacteria |
| Bacteroidales sp. (HG3A.0147) | T90 | -0.007 | 0.712 | 0.787 | 3249 unclassified | unclassified | unclassified | unclassified | Bacteroidales | Bacteroidia | Bacteroidetes |
| Clostridia sp. (HG3A.1108) | T90 | -0.006 | 0.716 | 0.789 | 3249 unclassified | unclassified | unclassified | unclassified | unclassified | Clostridia | Firmicutes |
| Eubacteriales sp. (HG3A.0868) | AHI | -0.008 | 0.655 | 0.789 | 2909 unclassified | unclassified | unclassified | unclassified | Eubacteriales | Clostridia | Firmicutes |
| Rikenellaceae sp. (HG3A.1022) | T90 | 0.006 | 0.715 | 0.789 | 3249 unclassified | unclassified | unclassified | Rikenellaceae | Bacteroidales | Bacteroidia | Bacteroidetes |
| Streptococcus salivarius  (HG3A.0071) | AHI | 0.008 | 0.654 | 0.789 | 2909 unclassified | Streptococcus  salivarius | Streptococcus | Streptococcaceae | Lactobacillales | Bacilli | Firmicutes |

| Anaerostipes hadrus (HG3A.0003) | AHI | 0.008 | 0.657 | 0.790 | 2909 unclassified | Anaerostipes  hadrus | Anaerostipes | Lachnospiraceae | Eubacteriales | Clostridia | Firmicutes |
| --- | --- | --- | --- | --- | --- | --- | --- | --- | --- | --- | --- |
| Eubacteriales sp. (HG3A.0136) | T90 | -0.006 | 0.718 | 0.790 | 3249 unclassified | unclassified | unclassified | unclassified | Eubacteriales | Clostridia | Firmicutes |
| Oscillospiraceae sp. (HG3A.0944) | ODI | -0.006 | 0.718 | 0.790 | 3249 unclassified | unclassified | unclassified | Oscillospiraceae | Eubacteriales | Clostridia | Firmicutes |
| Eubacteriales sp. (HG3A.0350) | T90 | -0.006 | 0.72 | 0.791 | 3249 unclassified | unclassified | unclassified | unclassified | Eubacteriales | Clostridia | Firmicutes |
| Veillonella tobetsuensis (HG3A.1344) | AHI | -0.008 | 0.659 | 0.791 | 2909 unclassified | Veillonella tobetsuensis | Veillonella | Veillonellaceae | Veillonellales | Negativicutes | Firmicutes |
| Eubacteriales sp. (HG3A.0546) | T90 | 0.006 | 0.726 | 0.792 | 3249 unclassified | unclassified | unclassified | unclassified | Eubacteriales | Clostridia | Firmicutes |
| Eubacteriales sp. (HG3A.0820) | T90 | -0.006 | 0.727 | 0.792 | 3249 unclassified | unclassified | unclassified | unclassified | Eubacteriales | Clostridia | Firmicutes |
| Eubacteriales sp. (HG3A.0887) | T90 | 0.006 | 0.722 | 0.792 | 3249 unclassified | unclassified | unclassified | unclassified | Eubacteriales | Clostridia | Firmicutes |
| Eubacteriales sp. (HG3A.1063) | T90 | -0.006 | 0.724 | 0.792 | 3249 unclassified | unclassified | unclassified | unclassified | Eubacteriales | Clostridia | Firmicutes |
| Lachnospiraceae sp. (HG3A.0217) | T90 | -0.006 | 0.727 | 0.792 | 3249 unclassified | unclassified | unclassified | Lachnospiraceae | Eubacteriales | Clostridia | Firmicutes |
| Lachnospiraceae sp. (HG3A.0831) | T90 | 0.006 | 0.727 | 0.792 | 3249 unclassified | unclassified | unclassified | Lachnospiraceae | Eubacteriales | Clostridia | Firmicutes |
| Lachnotalea sp. AF33-28  (HG3A.0403) | T90 | -0.006 | 0.729 | 0.793 | 3249 unclassified | Lachnotalea sp.  AF33-28 | Lachnotalea | Lachnospiraceae | Eubacteriales | Clostridia | Firmicutes |
| Streptococcus sobrinus (HG3A.1366) | ODI | 0.006 | 0.722 | 0.793 | 3249 unclassified | Streptococcus sobrinus | Streptococcus | Streptococcaceae | Lactobacillales | Bacilli | Firmicutes |
| Clostridia sp. (HG3A.1452) | T90 | 0.006 | 0.732 | 0.795 | 3249 unclassified | unclassified | unclassified | unclassified | unclassified | Clostridia | Firmicutes |
| Erysipelotrichales sp. (HG3A.0283) | ODI | 0.006 | 0.726 | 0.795 | 3249 unclassified | unclassified | unclassified | unclassified | Erysipelotrichales | Erysipelotrichia | Firmicutes |
| Eubacteriales sp. (HG3A.0846) | ODI | 0.006 | 0.726 | 0.795 | 3249 unclassified | unclassified | unclassified | unclassified | Eubacteriales | Clostridia | Firmicutes |
| Lachnospiraceae sp. (HG3A.1641) | T90 | 0.006 | 0.733 | 0.795 | 3249 unclassified | unclassified | unclassified | Lachnospiraceae | Eubacteriales | Clostridia | Firmicutes |
| Butyrivibrio crossotus (HG3A.0413) | AHI | -0.008 | 0.666 | 0.796 | 2909 unclassified | Butyrivibrio  crossotus | Butyrivibrio | Lachnospiraceae | Eubacteriales | Clostridia | Firmicutes |
| Candidatus Borkfalkia ceftriaxoniphila |  |  |  |  |  | Candidatus  Borkfalkia | Candidatus | Candidatus | Candidatus |  |  |
| (HG3A.0595) | AHI | -0.008 | 0.664 | 0.796 | 2909 unclassified | ceftriaxoniphila | Borkfalkia | Borkfalkiaceae | Borkfalkiales | Clostridia | Firmicutes |
| Enterocloster clostridioformis  (HG3A.0686) | AHI | 0.008 | 0.666 | 0.796 | 2909 unclassified | Enterocloster  clostridioformis | Enterocloster | Lachnospiraceae | Eubacteriales | Clostridia | Firmicutes |
| Clostridia sp. (HG3A.1410) | T90 | -0.006 | 0.736 | 0.797 | 3249 unclassified | unclassified | unclassified | unclassified | unclassified | Clostridia | Firmicutes |
| Erysipelotrichales sp. (HG3A.0809) | T90 | -0.006 | 0.737 | 0.797 | 3249 unclassified | unclassified | unclassified | unclassified | Erysipelotrichales | Erysipelotrichia | Firmicutes |
| Oscillospiraceae sp. (HG3A.0765) | T90 | -0.006 | 0.737 | 0.797 | 3249 unclassified | unclassified | unclassified | Oscillospiraceae | Eubacteriales | Clostridia | Firmicutes |
| Bacteria sp. (HG3A.1543) | T90 | 0.006 | 0.739 | 0.798 | 3249 unclassified | unclassified | unclassified | unclassified | unclassified | unclassified | unclassified |

| Solobacterium moorei (HG3A.1589) | T90 | 0.006 | 0.74 | 0.798 | 3249 unclassified | Solobacterium  moorei | Solobacterium | Erysipelotrichacea  e | Erysipelotrichales | Erysipelotrichia | Firmicutes |
| --- | --- | --- | --- | --- | --- | --- | --- | --- | --- | --- | --- |
| Oscillospiraceae sp. (HG3A.1491) | ODI | 0.006 | 0.731 | 0.799 | 3249 unclassified | unclassified | unclassified | Oscillospiraceae | Eubacteriales | Clostridia | Firmicutes |
| Ruminococcus sp. (HG3A.0126) | ODI | -0.006 | 0.73 | 0.799 | 3249 unclassified | unclassified | Ruminococcus | Oscillospiraceae | Eubacteriales | Clostridia | Firmicutes |
| Eubacteriales sp. (HG3A.0432) | T90 | -0.006 | 0.743 | 0.800 | 3249 unclassified | unclassified | unclassified | unclassified | Eubacteriales | Clostridia | Firmicutes |
| Bacteroidales sp. (HG3A.1446) | AHI | -0.008 | 0.674 | 0.805 | 2909 unclassified | unclassified | unclassified | unclassified | Bacteroidales | Bacteroidia | Bacteroidetes |
| Enterocloster sp. (HG3A.1529) | AHI | 0.008 | 0.679 | 0.806 | 2909 unclassified | unclassified | Enterocloster | Lachnospiraceae | Eubacteriales | Clostridia | Firmicutes |
| Eubacteriales sp. (HG3A.0490) | ODI | -0.006 | 0.738 | 0.806 | 3249 unclassified | unclassified | unclassified | unclassified | Eubacteriales | Clostridia | Firmicutes |
| Eubacteriales sp. (HG3A.1067) | AHI | -0.008 | 0.677 | 0.806 | 2909 unclassified | unclassified | unclassified | unclassified | Eubacteriales | Clostridia | Firmicutes |
| Eubacteriales sp. (HG3A.1131) | AHI | -0.008 | 0.676 | 0.806 | 2909 unclassified | unclassified | unclassified | unclassified | Eubacteriales | Clostridia | Firmicutes |
| Latilactobacillus curvatus  (HG3A.1505) | T90 | 0.006 | 0.75 | 0.806 | 3249 unclassified | Latilactobacillus  curvatus | Latilactobacillus | Lactobacillaceae | Lactobacillales | Bacilli | Firmicutes |
| Victivallales sp. (HG3A.0824) | AHI | 0.008 | 0.679 | 0.806 | 2909 unclassified | unclassified | unclassified | unclassified | Victivallales | Lentisphaeria | Lentisphaerae |
| Clostridia sp. (HG3A.1356) | AHI | -0.008 | 0.682 | 0.807 | 2909 unclassified | unclassified | unclassified | unclassified | unclassified | Clostridia | Firmicutes |
| Levilactobacillus brevis (HG3A.1848) | AHI | 0.008 | 0.682 | 0.807 | 2909 unclassified | Levilactobacillus brevis | Levilactobacillus | Lactobacillaceae | Lactobacillales | Bacilli | Firmicutes |
| Blautia producta (HG3A.0905) | AHI | 0.008 | 0.686 | 0.809 | 2909 unclassified | Blautia producta | Blautia | Lachnospiraceae | Eubacteriales | Clostridia | Firmicutes |
| Eubacteriales sp. (HG3A.1285) | AHI | -0.008 | 0.685 | 0.809 | 2909 unclassified | unclassified | unclassified | unclassified | Eubacteriales | Clostridia | Firmicutes |
| Clostridia sp. (HG3A.0828) | AHI | -0.008 | 0.689 | 0.811 | 2909 unclassified | unclassified | unclassified | unclassified | unclassified | Clostridia | Firmicutes |
| Clostridia sp. (HG3A.0512) | AHI | -0.008 | 0.69 | 0.812 | 2909 unclassified | unclassified | unclassified | unclassified | unclassified | Clostridia | Firmicutes |
| Eubacteriales sp. (HG3A.0594) | T90 | -0.005 | 0.757 | 0.813 | 3249 unclassified | unclassified | unclassified | unclassified | Eubacteriales | Clostridia | Firmicutes |
| Eubacteriales sp. (HG3A.1102) | T90 | -0.005 | 0.758 | 0.813 | 3249 unclassified | unclassified | unclassified | unclassified | Eubacteriales | Clostridia | Firmicutes |
| Bacteria sp. (HG3A.1553) | ODI | -0.006 | 0.749 | 0.815 | 3249 unclassified | unclassified | unclassified | unclassified | unclassified | unclassified | unclassified |
| Faecalibacterium sp. (HG3A.0073) | ODI | -0.006 | 0.747 | 0.815 | 3249 unclassified | unclassified | Faecalibacterium | Oscillospiraceae | Eubacteriales | Clostridia | Firmicutes |
| Eubacteriales sp. (HG3A.0827) | AHI | -0.007 | 0.696 | 0.817 | 2909 unclassified | unclassified | unclassified | unclassified | Eubacteriales | Clostridia | Firmicutes |
| Eubacteriales sp. (HG3A.0408) | ODI | 0.006 | 0.756 | 0.818 | 3249 unclassified | unclassified | unclassified | unclassified | Eubacteriales | Clostridia | Firmicutes |
| Eubacteriales sp. (HG3A.0791) | ODI | -0.006 | 0.754 | 0.818 | 3249 unclassified | unclassified | unclassified | unclassified | Eubacteriales | Clostridia | Firmicutes |
| Oscillospiraceae sp. (HG3A.0134) | ODI | -0.006 | 0.755 | 0.818 | 3249 unclassified | unclassified | unclassified | Oscillospiraceae | Eubacteriales | Clostridia | Firmicutes |
| Sutterella wadsworthensis  (HG3A.0143) | ODI | -0.006 | 0.755 | 0.818 | 3249 unclassified | Sutterella  wadsworthensis | Sutterella | Sutterellaceae | Burkholderiales | Betaproteobacte  ria | Proteobacteria |
| Bacteroidales sp. (HG3A.1002) | ODI | -0.005 | 0.758 | 0.819 | 3249 unclassified | unclassified | unclassified | unclassified | Bacteroidales | Bacteroidia | Bacteroidetes |
| Desulfovibrionales sp. (HG3A.0727) | T90 | 0.005 | 0.766 | 0.820 | 3249 unclassified | unclassified | unclassified | unclassified | Desulfovibrionales | Deltaproteobact  eria | Proteobacteria |
| Eubacteriales sp. (HG3A.0116) | T90 | -0.005 | 0.767 | 0.820 | 3249 unclassified | unclassified | unclassified | unclassified | Eubacteriales | Clostridia | Firmicutes |

| Eubacteriales sp. (HG3A.0692) | ODI | -0.005 | 0.76 | 0.821 | 3249 unclassified | unclassified | unclassified | unclassified | Eubacteriales | Clostridia | Firmicutes |
| --- | --- | --- | --- | --- | --- | --- | --- | --- | --- | --- | --- |
| Eubacteriales sp. (HG3A.1063) | AHI | -0.007 | 0.702 | 0.823 | 2909 unclassified | unclassified | unclassified | unclassified | Eubacteriales | Clostridia | Firmicutes |
| Ligilactobacillus salivarius  (HG3A.0919) | AHI | 0.007 | 0.703 | 0.823 | 2909 unclassified | Ligilactobacillus  salivarius | Ligilactobacillus | Lactobacillaceae | Lactobacillales | Bacilli | Firmicutes |
| Blautia producta (HG3A.0619) | AHI | 0.007 | 0.707 | 0.824 | 2909 unclassified | Blautia producta | Blautia | Lachnospiraceae | Eubacteriales | Clostridia | Firmicutes |
| Eubacteriales sp. (HG3A.1006) | AHI | 0.007 | 0.706 | 0.824 | 2909 unclassified | unclassified | unclassified | unclassified | Eubacteriales | Clostridia | Firmicutes |
| Lachnospiraceae sp. (HG3A.1641) | ODI | 0.005 | 0.765 | 0.824 | 3249 unclassified | unclassified | unclassified | Lachnospiraceae | Eubacteriales | Clostridia | Firmicutes |
| Oscillospiraceae sp. (HG3A.0739) | AHI | -0.007 | 0.705 | 0.824 | 2909 unclassified | unclassified | unclassified | Oscillospiraceae | Eubacteriales | Clostridia | Firmicutes |
| Firmicutes sp. (HG3A.1048) | AHI | 0.007 | 0.708 | 0.825 | 2909 unclassified | unclassified | unclassified | unclassified | unclassified | unclassified | Firmicutes |
| Bacteria sp. (HG3A.0708) | ODI | -0.005 | 0.77 | 0.828 | 3249 unclassified | unclassified | unclassified | unclassified | unclassified | unclassified | unclassified |
| Fusobacterium  Fusobacterium nucleatum subsp. nucleatum subsp. Fusobacterium | | | | | | | | | | | |
| animalis (HG3A.1418) | ODI | 0.005 | 0.769 | 0.828 | 3249 animalis | nucleatum | Fusobacterium | Fusobacteriaceae | Fusobacteriales | Fusobacteriia | Fusobacteria |
| Eubacteriales sp. (HG3A.1126) | ODI | 0.005 | 0.774 | 0.829 | 3249 unclassified | unclassified | unclassified | unclassified | Eubacteriales | Clostridia | Firmicutes |
| Eubacteriales sp. (HG3A.1546) | AHI | -0.007 | 0.714 | 0.829 | 2909 unclassified | unclassified | unclassified | unclassified | Eubacteriales | Clostridia | Firmicutes |
| Evtepia gabavorous (HG3A.0114) | AHI | -0.007 | 0.713 | 0.829 | 2909 unclassified | Evtepia gabavorous | Evtepia | unclassified | Eubacteriales | Clostridia | Firmicutes |
| Firmicutes sp. (HG3A.0817) | ODI | 0.005 | 0.772 | 0.829 | 3249 unclassified | unclassified | unclassified | unclassified | unclassified | unclassified | Firmicutes |
| Clostridia sp. (HG3A.1493) | AHI | -0.007 | 0.72 | 0.830 | 2909 unclassified | unclassified | unclassified | unclassified | unclassified | Clostridia | Firmicutes |
| Dorea longicatena (HG3A.0039) | AHI | 0.007 | 0.719 | 0.830 | 2909 unclassified | Dorea longicatena | Dorea | Lachnospiraceae | Eubacteriales | Clostridia | Firmicutes |
| Eubacteriales sp. (HG3A.1102) | AHI | -0.007 | 0.716 | 0.830 | 2909 unclassified | unclassified | unclassified | unclassified | Eubacteriales | Clostridia | Firmicutes |
| Hungatella hathewayi (HG3A.0455) | ODI | 0.005 | 0.776 | 0.830 | 3249 unclassified | Hungatella hathewayi | Hungatella | Clostridiaceae | Eubacteriales | Clostridia | Firmicutes |
| Streptococcus gordonii (HG3A.0713) | AHI | 0.007 | 0.718 | 0.830 | 2909 unclassified | Streptococcus  gordonii | Streptococcus | Streptococcaceae | Lactobacillales | Bacilli | Firmicutes |
| Collinsella phocaeensis (HG3A.1340) | ODI | -0.005 | 0.78 | 0.831 | 3249 unclassified | Collinsella phocaeensis | Collinsella | Coriobacteriaceae | Coriobacteriales | Coriobacteriia | Actinobacteria |
| Eubacteriales sp. (HG3A.0664) | ODI | 0.005 | 0.778 | 0.831 | 3249 unclassified | unclassified | unclassified | unclassified | Eubacteriales | Clostridia | Firmicutes |
| Tyzzerella nexilis (HG3A.0574) | ODI | 0.005 | 0.779 | 0.831 | 3249 unclassified | Tyzzerella nexilis | Tyzzerella | Lachnospiraceae | Eubacteriales | Clostridia | Firmicutes |
| Agathobaculum desmolans  (HG3A.1429) | AHI | 0.007 | 0.726 | 0.834 | 2909 unclassified | Agathobaculum  desmolans | Agathobaculum | Oscillospiraceae | Eubacteriales | Clostridia | Firmicutes |
| Eubacteriales sp. (HG3A.0369) | AHI | -0.007 | 0.725 | 0.834 | 2909 unclassified | unclassified | unclassified | unclassified | Eubacteriales | Clostridia | Firmicutes |
| Oscillospiraceae sp. (HG3A.0849) | AHI | 0.007 | 0.724 | 0.834 | 2909 unclassified | unclassified | unclassified | Oscillospiraceae | Eubacteriales | Clostridia | Firmicutes |
| Eubacteriales sp. (HG3A.0536) | AHI | -0.007 | 0.728 | 0.835 | 2909 unclassified | unclassified | unclassified | unclassified | Eubacteriales | Clostridia | Firmicutes |

| Lachnospiraceae sp. (HG3A.0172) | AHI | -0.006 | 0.732 | 0.837 | 2909 unclassified | unclassified | unclassified | Lachnospiraceae | Eubacteriales | Clostridia | Firmicutes |
| --- | --- | --- | --- | --- | --- | --- | --- | --- | --- | --- | --- |
| Streptococcus oralis subsp. oralis |  |  |  |  | Streptococcus  oralis subsp. | Streptococcus |  |  |  |  |  |
| (HG3A.0705) | AHI | 0.006 | 0.731 | 0.837 | 2909 oralis | oralis | Streptococcus | Streptococcaceae | Lactobacillales | Bacilli | Firmicutes |
| Eubacteriales sp. (HG3A.0490) | AHI | 0.006 | 0.737 | 0.838 | 2909 unclassified | unclassified | unclassified | unclassified | Eubacteriales | Clostridia | Firmicutes |
| Eubacteriales sp. (HG3A.1226) | AHI | -0.006 | 0.737 | 0.838 | 2909 unclassified | unclassified | unclassified | unclassified | Eubacteriales | Clostridia | Firmicutes |
| Eubacteriales sp. (HG3A.1257) | AHI | -0.006 | 0.737 | 0.838 | 2909 unclassified | unclassified | unclassified | unclassified | Eubacteriales | Clostridia | Firmicutes |
| Tyzzerella nexilis (HG3A.0574) | AHI | 0.006 | 0.734 | 0.838 | 2909 unclassified | Tyzzerella nexilis | Tyzzerella | Lachnospiraceae | Eubacteriales | Clostridia | Firmicutes |
| Erysipelatoclostridium sp.  (HG3A.0313) | ODI | -0.005 | 0.789 | 0.840 | 3249 unclassified | unclassified | Erysipelatoclostri  dium | Erysipelotrichacea  e | Erysipelotrichales | Erysipelotrichia | Firmicutes |
| Eubacteriales sp. (HG3A.0614) | AHI | -0.006 | 0.741 | 0.841 | 2909 unclassified | unclassified | unclassified | unclassified | Eubacteriales | Clostridia | Firmicutes |
| Bacteria sp. (HG3A.0500) | T90 | -0.005 | 0.788 | 0.842 | 3249 unclassified | unclassified | unclassified | unclassified | unclassified | unclassified | unclassified |
| Clostridia sp. (HG3A.1247) | T90 | 0.005 | 0.79 | 0.842 | 3249 unclassified | unclassified | unclassified | unclassified | unclassified | Clostridia | Firmicutes |
| Lachnospiraceae sp. (HG3A.0172) | T90 | 0.005 | 0.789 | 0.842 | 3249 unclassified | unclassified | unclassified | Lachnospiraceae | Eubacteriales | Clostridia | Firmicutes |
| Catenibacterium mitsuokai (HG3A.0775) | T90 | 0.005 | 0.793 | 0.843 | 3249 unclassified | Catenibacterium mitsuokai | Catenibacterium | Coprobacillaceae | Erysipelotrichales | Erysipelotrichia | Firmicutes |
| Clostridia sp. (HG3A.1193) | ODI | -0.005 | 0.793 | 0.844 | 3249 unclassified | unclassified | unclassified | unclassified | unclassified | Clostridia | Firmicutes |
| Eubacteriales sp. (HG3A.0647) | ODI | -0.005 | 0.795 | 0.844 | 3249 unclassified | unclassified | unclassified | unclassified | Eubacteriales | Clostridia | Firmicutes |
| Eubacteriales sp. (HG3A.1439) | ODI | 0.005 | 0.796 | 0.844 | 3249 unclassified | unclassified | unclassified | unclassified | Eubacteriales | Clostridia | Firmicutes |
| Ruminococcus sp. AF17-22AC (HG3A.0208) | ODI | 0.005 | 0.799 | 0.846 | 3249 unclassified | Ruminococcus sp. AF17-22AC | Ruminococcus | Oscillospiraceae | Eubacteriales | Clostridia | Firmicutes |
| Coprobacter fastidiosus (HG3A.0182) | T90 | 0.004 | 0.801 | 0.851 | 3249 unclassified | Coprobacter  fastidiosus | Coprobacter | Barnesiellaceae | Bacteroidales | Bacteroidia | Bacteroidetes |
| Ruminococcus sp. AF17-22AC (HG3A.0208) | AHI | 0.006 | 0.75 | 0.851 | 2909 unclassified | Ruminococcus sp. AF17-22AC | Ruminococcus | Oscillospiraceae | Eubacteriales | Clostridia | Firmicutes |
| Eubacteriales sp. (HG3A.0751) | AHI | 0.006 | 0.756 | 0.856 | 2909 unclassified | unclassified | unclassified | unclassified | Eubacteriales | Clostridia | Firmicutes |
| Bacteria sp. (HG3A.0361) | AHI | 0.006 | 0.76 | 0.857 | 2909 unclassified | unclassified | unclassified | unclassified | unclassified | unclassified | unclassified |
| Enterocloster aldenensis  (HG3A.0362) | AHI | 0.006 | 0.762 | 0.857 | 2909 unclassified | Enterocloster  aldenensis | Enterocloster | Lachnospiraceae | Eubacteriales | Clostridia | Firmicutes |
| Eubacteriales sp. (HG3A.0453) | AHI | -0.006 | 0.762 | 0.857 | 2909 unclassified | unclassified | unclassified | unclassified | Eubacteriales | Clostridia | Firmicutes |
| Eubacteriales sp. (HG3A.0535) | AHI | 0.006 | 0.761 | 0.857 | 2909 unclassified | unclassified | unclassified | unclassified | Eubacteriales | Clostridia | Firmicutes |
| Oscillospiraceae sp. (HG3A.1491) | AHI | 0.006 | 0.758 | 0.857 | 2909 unclassified | unclassified | unclassified | Oscillospiraceae | Eubacteriales | Clostridia | Firmicutes |
| Faecalibacterium sp. OF04-11AC  (HG3A.0070) | AHI | -0.006 | 0.765 | 0.858 | 2909 unclassified | Faecalibacterium  sp. OF04-11AC | Faecalibacterium | Oscillospiraceae | Eubacteriales | Clostridia | Firmicutes |
| Actinomyces sp. ICM58 (HG3A.0410) | ODI | -0.004 | 0.813 | 0.860 | 3249 unclassified | Actinomyces sp. ICM58 | Actinomyces | Actinomycetaceae | Actinomycetales | Actinomycetia | Actinobacteria |

| Bacteroidales sp. (HG3A.0147) | AHI | -0.006 | 0.77 | 0.862 | 2909 unclassified | unclassified | unclassified | unclassified | Bacteroidales | Bacteroidia | Bacteroidetes |
| --- | --- | --- | --- | --- | --- | --- | --- | --- | --- | --- | --- |
| Eubacteriales sp. (HG3A.0322) | AHI | 0.005 | 0.771 | 0.862 | 2909 unclassified | unclassified | unclassified | unclassified | Eubacteriales | Clostridia | Firmicutes |
| Eubacteriales sp. (HG3A.0978) | AHI | -0.005 | 0.771 | 0.862 | 2909 unclassified | unclassified | unclassified | unclassified | Eubacteriales | Clostridia | Firmicutes |
| Lacticaseibacillus paracasei subsp. |  |  |  |  | Lacticaseibacillus  paracasei subsp. | Lacticaseibacillus |  |  |  |  |  |
| paracasei (HG3A.0853) | AHI | -0.005 | 0.775 | 0.865 | 2909 paracasei | paracasei | Lacticaseibacillus | Lactobacillaceae | Lactobacillales | Bacilli | Firmicutes |
| Oscillospiraceae sp. (HG3A.0134) | T90 | -0.004 | 0.815 | 0.865 | 3249 unclassified | unclassified | unclassified | Oscillospiraceae | Eubacteriales | Clostridia | Firmicutes |
| Clostridia sp. (HG3A.0918) | AHI | -0.005 | 0.777 | 0.866 | 2909 unclassified | unclassified | unclassified | unclassified | unclassified | Clostridia | Firmicutes |
[truncated: 466,603 more chars]
