# Supplementary material for: OSA Is Associated With the Human Gut Microbiota Composition and Functional Potential in the Population-Based Swedish CardioPulmonary bioImage Study
Source: Chest. 2023 Mar 15;164(2):503–16. doi: 10.1016/j.chest.2023.03.010 (PMC10410248; doi:10.1016/j.chest.2023.03.010)
Supplement: e-Table 8 [file mmc5.docx]

e-Table 8. Prevalence and relative abundance and taxonomy of the 128 species associated with T90/ODI

Table showing the prevalence, and median and percentiles of relative abundance of the 128 species associated with T90/ODI after adjustment for the extended model covariates

**Metagenomic species Prevalence (%) p5 (%) p25 (%) Median (%) p75 (%) p90 (%) genus family order class phylum**

| Akkermansia muciniphila (HG3A.0110) | 77.5 | 0.00 | 0.00 | 0.16 | 1.25 | 5.00 | Akkermansia | Akkermansiaceae | Verrucomicrobiales | Verrucomicrobiae | Verrucomicrobia |
| --- | --- | --- | --- | --- | --- | --- | --- | --- | --- | --- | --- |
| Alistipes communis (HG3A.0064) | 81.0 | 0.00 | 0.01 | 0.09 | 0.26 | 0.83 | Alistipes | Rikenellaceae | Bacteroidales | Bacteroidia | Bacteroidetes |
| Alistipes provencensis (HG3A.0877) | 4.6 | 0.00 | 0.00 | 0.00 | 0.00 | 0.00 | Alistipes | Rikenellaceae | Bacteroidales | Bacteroidia | Bacteroidetes |
| Alistipes shahii (HG3A.0054) | 88.0 | 0.00 | 0.04 | 0.18 | 0.45 | 1.24 | Alistipes | Rikenellaceae | Bacteroidales | Bacteroidia | Bacteroidetes |
| Anaerobutyricum hallii (HG3A.0012) | 98.0 | 0.03 | 0.21 | 0.42 | 0.78 | 1.79 | Anaerobutyricum | Lachnospiraceae | Eubacteriales | Clostridia | Firmicutes |
| Anaerostipes sp. BG01 (HG3A.1509) | 2.2 | 0.00 | 0.00 | 0.00 | 0.00 | 0.00 | Anaerostipes | Lachnospiraceae | Eubacteriales | Clostridia | Firmicutes |
| Bacteria sp. (HG3A.0483) | 23.5 | 0.00 | 0.00 | 0.00 | 0.00 | 0.05 | unclassified | unclassified | unclassified | unclassified | unclassified |
| Bacteria sp. (HG3A.0634) | 20.2 | 0.00 | 0.00 | 0.00 | 0.00 | 0.05 | unclassified | unclassified | unclassified | unclassified | unclassified |
| Bacteria sp. (HG3A.0911) | 8.1 | 0.00 | 0.00 | 0.00 | 0.00 | 0.01 | unclassified | unclassified | unclassified | unclassified | unclassified |
| Blautia massiliensis (HG3A.0023) | 97.5 | 0.02 | 0.09 | 0.24 | 0.67 | 2.40 | Blautia | Lachnospiraceae | Eubacteriales | Clostridia | Firmicutes |
| Blautia obeum (HG3A.0001) | 99.8 | 0.13 | 0.45 | 0.93 | 1.89 | 5.92 | Blautia | Lachnospiraceae | Eubacteriales | Clostridia | Firmicutes |
| Blautia obeum (HG3A.0009) | 96.9 | 0.01 | 0.06 | 0.14 | 0.32 | 1.00 | Blautia | Lachnospiraceae | Eubacteriales | Clostridia | Firmicutes |
| Candidatus Borkfalkiales sp. (HG3A.1329) | 4.0 | 0.00 | 0.00 | 0.00 | 0.00 | 0.00 | unclassified | unclassified | Candidatus  Borkfalkiales | Clostridia | Firmicutes |
| Candidatus Borkfalkiales sp. (HG3A.1397) | 2.7 | 0.00 | 0.00 | 0.00 | 0.00 | 0.00 | unclassified | unclassified | Candidatus Borkfalkiales | Clostridia | Firmicutes |
| Clostridia sp. (HG3A.0094) | 80.7 | 0.00 | 0.01 | 0.08 | 0.42 | 3.17 | unclassified | unclassified | unclassified | Clostridia | Firmicutes |
| Clostridia sp. (HG3A.0140) | 58.7 | 0.00 | 0.00 | 0.02 | 0.23 | 2.27 | unclassified | unclassified | unclassified | Clostridia | Firmicutes |
| Clostridia sp. (HG3A.0272) | 27.9 | 0.00 | 0.00 | 0.00 | 0.02 | 1.09 | unclassified | unclassified | unclassified | Clostridia | Firmicutes |
| Clostridia sp. (HG3A.0435) | 36.5 | 0.00 | 0.00 | 0.00 | 0.00 | 0.03 | unclassified | unclassified | unclassified | Clostridia | Firmicutes |
| Clostridia sp. (HG3A.0470) | 20.7 | 0.00 | 0.00 | 0.00 | 0.00 | 1.17 | unclassified | unclassified | unclassified | Clostridia | Firmicutes |
| Clostridia sp. (HG3A.0508) | 18.8 | 0.00 | 0.00 | 0.00 | 0.00 | 0.73 | unclassified | unclassified | unclassified | Clostridia | Firmicutes |
| Clostridia sp. (HG3A.0515) | 18.9 | 0.00 | 0.00 | 0.00 | 0.00 | 0.03 | unclassified | unclassified | unclassified | Clostridia | Firmicutes |
| Clostridia sp. (HG3A.0550) | 12.4 | 0.00 | 0.00 | 0.00 | 0.00 | 0.09 | unclassified | unclassified | unclassified | Clostridia | Firmicutes |
| Clostridia sp. (HG3A.0599) | 11.6 | 0.00 | 0.00 | 0.00 | 0.00 | 0.11 | unclassified | unclassified | unclassified | Clostridia | Firmicutes |
| Clostridia sp. (HG3A.0645) | 11.1 | 0.00 | 0.00 | 0.00 | 0.00 | 0.01 | unclassified | unclassified | unclassified | Clostridia | Firmicutes |
| Clostridia sp. (HG3A.0682) | 18.4 | 0.00 | 0.00 | 0.00 | 0.00 | 0.14 | unclassified | unclassified | unclassified | Clostridia | Firmicutes |
| Clostridia sp. (HG3A.0728) | 8.7 | 0.00 | 0.00 | 0.00 | 0.00 | 0.01 | unclassified | unclassified | unclassified | Clostridia | Firmicutes |
| Clostridia sp. (HG3A.0815) | 16.4 | 0.00 | 0.00 | 0.00 | 0.00 | 0.01 | unclassified | unclassified | unclassified | Clostridia | Firmicutes |

| Clostridia sp. (HG3A.0861) | 13.9 | 0.00 | 0.00 | 0.00 | 0.00 | 0.01 | unclassified | unclassified | unclassified | Clostridia | Firmicutes |
| --- | --- | --- | --- | --- | --- | --- | --- | --- | --- | --- | --- |
| Clostridia sp. (HG3A.0879) | 8.2 | 0.00 | 0.00 | 0.00 | 0.00 | 0.00 | unclassified | unclassified | unclassified | Clostridia | Firmicutes |
| Clostridia sp. (HG3A.0931) | 6.7 | 0.00 | 0.00 | 0.00 | 0.00 | 0.00 | unclassified | unclassified | unclassified | Clostridia | Firmicutes |
| Clostridia sp. (HG3A.1008) | 3.0 | 0.00 | 0.00 | 0.00 | 0.00 | 0.00 | unclassified | unclassified | unclassified | Clostridia | Firmicutes |
| Clostridia sp. (HG3A.1020) | 6.5 | 0.00 | 0.00 | 0.00 | 0.00 | 0.00 | unclassified | unclassified | unclassified | Clostridia | Firmicutes |
| Clostridia sp. (HG3A.1057) | 6.5 | 0.00 | 0.00 | 0.00 | 0.00 | 0.00 | unclassified | unclassified | unclassified | Clostridia | Firmicutes |
| Clostridiaceae sp. (HG3A.0431) | 16.1 | 0.00 | 0.00 | 0.00 | 0.00 | 0.05 | unclassified | Clostridiaceae | Eubacteriales | Clostridia | Firmicutes |
| Clostridium sp. TF06-15AC (HG3A.0032) | 91.6 | 0.00 | 0.03 | 0.10 | 0.24 | 0.63 | Clostridium | Clostridiaceae | Eubacteriales | Clostridia | Firmicutes |
| Clostridium sp. (HG3A.0050) | 91.9 | 0.00 | 0.01 | 0.04 | 0.07 | 0.19 | Clostridium | Clostridiaceae | Eubacteriales | Clostridia | Firmicutes |
| Collinsella aerofaciens (HG3A.0019) | 91.7 | 0.00 | 0.32 | 0.66 | 1.25 | 3.10 | Collinsella | Coriobacteriaceae | Coriobacteriales | Coriobacteriia | Actinobacteria |
| Coprobacillus sp. (HG3A.0022) | 94.7 | 0.00 | 0.06 | 0.15 | 0.34 | 0.92 | Coprobacillus | Coprobacillaceae | Erysipelotrichales | Erysipelotrichia | Firmicutes |
| Coprococcus comes (HG3A.0016) | 95.3 | 0.01 | 0.13 | 0.25 | 0.44 | 0.94 | Coprococcus | Lachnospiraceae | Eubacteriales | Clostridia | Firmicutes |
| Coprococcus eutactus (HG3A.0155) | 62.3 | 0.00 | 0.00 | 0.05 | 0.75 | 4.34 | Coprococcus | Lachnospiraceae | Eubacteriales | Clostridia | Firmicutes |
| Dorea formicigenerans (HG3A.0006) | 97.9 | 0.02 | 0.06 | 0.09 | 0.16 | 0.36 | Dorea | Lachnospiraceae | Eubacteriales | Clostridia | Firmicutes |
| Dorea sp. AF36-15AT (HG3A.0052) | 96.6 | 0.00 | 0.02 | 0.03 | 0.06 | 0.16 | Dorea | Lachnospiraceae | Eubacteriales | Clostridia | Firmicutes |
| Eggerthellaceae sp. (HG3A.0171) | 87.9 | 0.00 | 0.00 | 0.01 | 0.02 | 0.06 | unclassified | Eggerthellaceae | Eggerthellales | Coriobacteriia | Actinobacteria |
| Eggerthellales sp. (HG3A.0177) | 64.4 | 0.00 | 0.00 | 0.01 | 0.11 | 0.66 | unclassified | unclassified | Eggerthellales | Coriobacteriia | Actinobacteria |
| Erysipelotrichales sp. (HG3A.1207) | 2.5 | 0.00 | 0.00 | 0.00 | 0.00 | 0.00 | unclassified | unclassified | Erysipelotrichales | Erysipelotrichia | Firmicutes |
| Eubacteriales sp. (HG3A.0069) | 89.2 | 0.00 | 0.02 | 0.07 | 0.22 | 0.76 | unclassified | unclassified | Eubacteriales | Clostridia | Firmicutes |
| Eubacteriales sp. (HG3A.0083) | 78.5 | 0.00 | 0.00 | 0.10 | 0.29 | 0.99 | unclassified | unclassified | Eubacteriales | Clostridia | Firmicutes |
| Eubacteriales sp. (HG3A.0084) | 76.8 | 0.00 | 0.00 | 0.05 | 0.11 | 0.27 | unclassified | unclassified | Eubacteriales | Clostridia | Firmicutes |
| Eubacteriales sp. (HG3A.0085) | 78.7 | 0.00 | 0.00 | 0.04 | 0.10 | 0.32 | unclassified | unclassified | Eubacteriales | Clostridia | Firmicutes |
| Eubacteriales sp. (HG3A.0100) | 70.1 | 0.00 | 0.00 | 0.27 | 1.59 | 4.79 | unclassified | unclassified | Eubacteriales | Clostridia | Firmicutes |
| Eubacteriales sp. (HG3A.0118) | 75.7 | 0.00 | 0.00 | 0.04 | 0.17 | 0.72 | unclassified | unclassified | Eubacteriales | Clostridia | Firmicutes |
| Eubacteriales sp. (HG3A.0120) | 68.4 | 0.00 | 0.00 | 0.05 | 0.17 | 0.52 | unclassified | unclassified | Eubacteriales | Clostridia | Firmicutes |
| Eubacteriales sp. (HG3A.0123) | 64.5 | 0.00 | 0.00 | 0.01 | 0.06 | 0.25 | unclassified | unclassified | Eubacteriales | Clostridia | Firmicutes |
| Eubacteriales sp. (HG3A.0149) | 59.8 | 0.00 | 0.00 | 0.02 | 0.24 | 0.94 | unclassified | unclassified | Eubacteriales | Clostridia | Firmicutes |
| Eubacteriales sp. (HG3A.0153) | 54.3 | 0.00 | 0.00 | 0.01 | 0.14 | 0.66 | unclassified | unclassified | Eubacteriales | Clostridia | Firmicutes |
| Eubacteriales sp. (HG3A.0156) | 53.6 | 0.00 | 0.00 | 0.02 | 0.17 | 0.67 | unclassified | unclassified | Eubacteriales | Clostridia | Firmicutes |
| Eubacteriales sp. (HG3A.0162) | 54.2 | 0.00 | 0.00 | 0.01 | 0.30 | 1.97 | unclassified | unclassified | Eubacteriales | Clostridia | Firmicutes |
| Eubacteriales sp. (HG3A.0193) | 62.9 | 0.00 | 0.00 | 0.00 | 0.02 | 0.08 | unclassified | unclassified | Eubacteriales | Clostridia | Firmicutes |
| Eubacteriales sp. (HG3A.0196) | 54.1 | 0.00 | 0.00 | 0.00 | 0.04 | 0.16 | unclassified | unclassified | Eubacteriales | Clostridia | Firmicutes |
| Eubacteriales sp. (HG3A.0197) | 60.4 | 0.00 | 0.00 | 0.00 | 0.07 | 0.25 | unclassified | unclassified | Eubacteriales | Clostridia | Firmicutes |
| Eubacteriales sp. (HG3A.0211) | 61.9 | 0.00 | 0.00 | 0.00 | 0.02 | 0.23 | unclassified | unclassified | Eubacteriales | Clostridia | Firmicutes |

| Eubacteriales sp. (HG3A.0215) | 42.0 | 0.00 | 0.00 | 0.00 | 0.08 | 1.14 | unclassified | unclassified | Eubacteriales | Clostridia | Firmicutes |
| --- | --- | --- | --- | --- | --- | --- | --- | --- | --- | --- | --- |
| Eubacteriales sp. (HG3A.0226) | 43.9 | 0.00 | 0.00 | 0.00 | 0.03 | 0.37 | unclassified | unclassified | Eubacteriales | Clostridia | Firmicutes |
| Eubacteriales sp. (HG3A.0229) | 38.0 | 0.00 | 0.00 | 0.00 | 0.03 | 0.30 | unclassified | unclassified | Eubacteriales | Clostridia | Firmicutes |
| Eubacteriales sp. (HG3A.0234) | 36.1 | 0.00 | 0.00 | 0.00 | 0.11 | 2.54 | unclassified | unclassified | Eubacteriales | Clostridia | Firmicutes |
| Eubacteriales sp. (HG3A.0242) | 50.3 | 0.00 | 0.00 | 0.00 | 0.01 | 0.06 | unclassified | unclassified | Eubacteriales | Clostridia | Firmicutes |
| Eubacteriales sp. (HG3A.0250) | 41.0 | 0.00 | 0.00 | 0.00 | 0.02 | 0.14 | unclassified | unclassified | Eubacteriales | Clostridia | Firmicutes |
| Eubacteriales sp. (HG3A.0269) | 42.1 | 0.00 | 0.00 | 0.00 | 0.02 | 0.09 | unclassified | unclassified | Eubacteriales | Clostridia | Firmicutes |
| Eubacteriales sp. (HG3A.0291) | 33.0 | 0.00 | 0.00 | 0.00 | 0.08 | 2.95 | unclassified | unclassified | Eubacteriales | Clostridia | Firmicutes |
| Eubacteriales sp. (HG3A.0309) | 28.8 | 0.00 | 0.00 | 0.00 | 0.01 | 0.19 | unclassified | unclassified | Eubacteriales | Clostridia | Firmicutes |
| Eubacteriales sp. (HG3A.0311) | 46.0 | 0.00 | 0.00 | 0.00 | 0.01 | 0.06 | unclassified | unclassified | Eubacteriales | Clostridia | Firmicutes |
| Eubacteriales sp. (HG3A.0321) | 32.9 | 0.00 | 0.00 | 0.00 | 0.01 | 0.26 | unclassified | unclassified | Eubacteriales | Clostridia | Firmicutes |
| Eubacteriales sp. (HG3A.0329) | 36.0 | 0.00 | 0.00 | 0.00 | 0.01 | 0.06 | unclassified | unclassified | Eubacteriales | Clostridia | Firmicutes |
| Eubacteriales sp. (HG3A.0331) | 30.4 | 0.00 | 0.00 | 0.00 | 0.00 | 0.10 | unclassified | unclassified | Eubacteriales | Clostridia | Firmicutes |
| Eubacteriales sp. (HG3A.0383) | 23.4 | 0.00 | 0.00 | 0.00 | 0.00 | 0.10 | unclassified | unclassified | Eubacteriales | Clostridia | Firmicutes |
| Eubacteriales sp. (HG3A.0419) | 37.8 | 0.00 | 0.00 | 0.00 | 0.00 | 0.02 | unclassified | unclassified | Eubacteriales | Clostridia | Firmicutes |
| Eubacteriales sp. (HG3A.0421) | 23.9 | 0.00 | 0.00 | 0.00 | 0.00 | 0.12 | unclassified | unclassified | Eubacteriales | Clostridia | Firmicutes |
| Eubacteriales sp. (HG3A.0439) | 61.3 | 0.00 | 0.00 | 0.00 | 0.01 | 0.05 | unclassified | unclassified | Eubacteriales | Clostridia | Firmicutes |
| Eubacteriales sp. (HG3A.0442) | 25.4 | 0.00 | 0.00 | 0.00 | 0.00 | 0.04 | unclassified | unclassified | Eubacteriales | Clostridia | Firmicutes |
| Eubacteriales sp. (HG3A.0468) | 19.8 | 0.00 | 0.00 | 0.00 | 0.00 | 1.65 | unclassified | unclassified | Eubacteriales | Clostridia | Firmicutes |
| Eubacteriales sp. (HG3A.0489) | 12.5 | 0.00 | 0.00 | 0.00 | 0.00 | 0.45 | unclassified | unclassified | Eubacteriales | Clostridia | Firmicutes |
| Eubacteriales sp. (HG3A.0506) | 30.2 | 0.00 | 0.00 | 0.00 | 0.00 | 0.02 | unclassified | unclassified | Eubacteriales | Clostridia | Firmicutes |
| Eubacteriales sp. (HG3A.0531) | 15.8 | 0.00 | 0.00 | 0.00 | 0.00 | 0.07 | unclassified | unclassified | Eubacteriales | Clostridia | Firmicutes |
| Eubacteriales sp. (HG3A.0548) | 22.5 | 0.00 | 0.00 | 0.00 | 0.00 | 0.02 | unclassified | unclassified | Eubacteriales | Clostridia | Firmicutes |
| Eubacteriales sp. (HG3A.0572) | 14.3 | 0.00 | 0.00 | 0.00 | 0.00 | 0.02 | unclassified | unclassified | Eubacteriales | Clostridia | Firmicutes |
| Eubacteriales sp. (HG3A.0609) | 7.8 | 0.00 | 0.00 | 0.00 | 0.00 | 0.22 | unclassified | unclassified | Eubacteriales | Clostridia | Firmicutes |
| Eubacteriales sp. (HG3A.0630) | 11.3 | 0.00 | 0.00 | 0.00 | 0.00 | 0.03 | unclassified | unclassified | Eubacteriales | Clostridia | Firmicutes |
| Eubacteriales sp. (HG3A.0635) | 46.2 | 0.00 | 0.00 | 0.00 | 0.00 | 0.02 | unclassified | unclassified | Eubacteriales | Clostridia | Firmicutes |
| Eubacteriales sp. (HG3A.0691) | 12.7 | 0.00 | 0.00 | 0.00 | 0.00 | 0.01 | unclassified | unclassified | Eubacteriales | Clostridia | Firmicutes |
| Eubacteriales sp. (HG3A.0703) | 7.8 | 0.00 | 0.00 | 0.00 | 0.00 | 0.04 | unclassified | unclassified | Eubacteriales | Clostridia | Firmicutes |
| Eubacteriales sp. (HG3A.0718) | 10.7 | 0.00 | 0.00 | 0.00 | 0.00 | 0.01 | unclassified | unclassified | Eubacteriales | Clostridia | Firmicutes |
| Eubacteriales sp. (HG3A.0786) | 5.1 | 0.00 | 0.00 | 0.00 | 0.00 | 0.00 | unclassified | unclassified | Eubacteriales | Clostridia | Firmicutes |
| Eubacteriales sp. (HG3A.0829) | 5.4 | 0.00 | 0.00 | 0.00 | 0.00 | 0.00 | unclassified | unclassified | Eubacteriales | Clostridia | Firmicutes |
| Eubacteriales sp. (HG3A.0956) | 3.2 | 0.00 | 0.00 | 0.00 | 0.00 | 0.00 | unclassified | unclassified | Eubacteriales | Clostridia | Firmicutes |
| Eubacteriales sp. (HG3A.0978) | 8.0 | 0.00 | 0.00 | 0.00 | 0.00 | 0.00 | unclassified | unclassified | Eubacteriales | Clostridia | Firmicutes |

| Eubacteriales sp. (HG3A.1019) | 7.2 | 0.00 | 0.00 | 0.00 | 0.00 | 0.01 | unclassified | unclassified | Eubacteriales | Clostridia | Firmicutes |
| --- | --- | --- | --- | --- | --- | --- | --- | --- | --- | --- | --- |
| Eubacteriales sp. (HG3A.1026) | 4.1 | 0.00 | 0.00 | 0.00 | 0.00 | 0.00 | unclassified | unclassified | Eubacteriales | Clostridia | Firmicutes |
| Eubacteriales sp. (HG3A.1294) | 1.8 | 0.00 | 0.00 | 0.00 | 0.00 | 0.00 | unclassified | unclassified | Eubacteriales | Clostridia | Firmicutes |
| Eubacteriales sp. (HG3A.1379) | 21.7 | 0.00 | 0.00 | 0.00 | 0.00 | 0.01 | unclassified | unclassified | Eubacteriales | Clostridia | Firmicutes |
| Eubacterium sp. (HG3A.0214) | 37.7 | 0.00 | 0.00 | 0.00 | 0.18 | 1.60 | Eubacterium | Eubacteriaceae | Eubacteriales | Clostridia | Firmicutes |
| Firmicutes sp. (HG3A.0301) | 31.8 | 0.00 | 0.00 | 0.00 | 0.00 | 0.20 | unclassified | unclassified | unclassified | unclassified | Firmicutes |
| Firmicutes sp. (HG3A.0341) | 29.4 | 0.00 | 0.00 | 0.00 | 0.00 | 0.22 | unclassified | unclassified | unclassified | unclassified | Firmicutes |
| Firmicutes sp. (HG3A.0397) | 21.0 | 0.00 | 0.00 | 0.00 | 0.00 | 0.19 | unclassified | unclassified | unclassified | unclassified | Firmicutes |
| Firmicutes sp. (HG3A.0398) | 27.3 | 0.00 | 0.00 | 0.00 | 0.00 | 0.07 | unclassified | unclassified | unclassified | unclassified | Firmicutes |
| Firmicutes sp. (HG3A.1085) | 3.2 | 0.00 | 0.00 | 0.00 | 0.00 | 0.00 | unclassified | unclassified | unclassified | unclassified | Firmicutes |
| Flavonifractor plautii (HG3A.0079) | 87.2 | 0.00 | 0.00 | 0.02 | 0.06 | 0.42 | Flavonifractor | Oscillospiraceae | Eubacteriales | Clostridia | Firmicutes |
| Fusicatenibacter saccharivorans  (HG3A.0004) | 97.9 | 0.06 | 0.46 | 0.98 | 1.90 | 4.35 | Fusicatenibacter | Lachnospiraceae | Eubacteriales | Clostridia | Firmicutes |
| Intestinibacillus sp. Marseille-P4005 (HG3A.0168) | 80.8 | 0.00 | 0.00 | 0.01 | 0.02 | 0.07 | Intestinibacillus | Eubacteriaceae | Eubacteriales | Clostridia | Firmicutes |
| Intestinimonas massiliensis (HG3A.0198) | 67.3 | 0.00 | 0.00 | 0.01 | 0.02 | 0.13 | Intestinimonas | unclassified | Eubacteriales | Clostridia | Firmicutes |
| Lachnospiraceae sp. (HG3A.0018) | 96.9 | 0.02 | 0.37 | 1.43 | 3.62 | 9.47 | unclassified | Lachnospiraceae | Eubacteriales | Clostridia | Firmicutes |
| Lachnospiraceae sp. (HG3A.0855) | 4.3 | 0.00 | 0.00 | 0.00 | 0.00 | 0.00 | unclassified | Lachnospiraceae | Eubacteriales | Clostridia | Firmicutes |
| Mediterraneibacter glycyrrhizinilyticus (HG3A.0314) | 27.7 | 0.00 | 0.00 | 0.00 | 0.00 | 0.07 | Mediterraneibacter | Lachnospiraceae | Eubacteriales | Clostridia | Firmicutes |
| Oscillibacter sp. (HG3A.0734) | 23.1 | 0.00 | 0.00 | 0.00 | 0.00 | 0.02 | Oscillibacter | Oscillospiraceae | Eubacteriales | Clostridia | Firmicutes |
| Oscillospiraceae sp. (HG3A.0072) | 84.1 | 0.00 | 0.03 | 0.25 | 0.68 | 1.67 | unclassified | Oscillospiraceae | Eubacteriales | Clostridia | Firmicutes |
| Oscillospiraceae sp. (HG3A.0207) | 44.0 | 0.00 | 0.00 | 0.00 | 0.05 | 0.20 | unclassified | Oscillospiraceae | Eubacteriales | Clostridia | Firmicutes |
| Oscillospiraceae sp. (HG3A.0223) | 71.2 | 0.00 | 0.00 | 0.01 | 0.02 | 0.09 | unclassified | Oscillospiraceae | Eubacteriales | Clostridia | Firmicutes |
| Oscillospiraceae sp. (HG3A.0437) | 29.7 | 0.00 | 0.00 | 0.00 | 0.00 | 0.04 | unclassified | Oscillospiraceae | Eubacteriales | Clostridia | Firmicutes |
| Oscillospiraceae sp. (HG3A.0445) | 22.5 | 0.00 | 0.00 | 0.00 | 0.00 | 0.11 | unclassified | Oscillospiraceae | Eubacteriales | Clostridia | Firmicutes |
| Oscillospiraceae sp. (HG3A.1270) | 4.8 | 0.00 | 0.00 | 0.00 | 0.00 | 0.00 | unclassified | Oscillospiraceae | Eubacteriales | Clostridia | Firmicutes |
| Pediococcus acidilactici (HG3A.1468) | 2.0 | 0.00 | 0.00 | 0.00 | 0.00 | 0.00 | Pediococcus | Lactobacillaceae | Lactobacillales | Bacilli | Firmicutes |
| Roseburia inulinivorans (HG3A.0036) | 94.4 | 0.00 | 0.03 | 0.13 | 0.46 | 1.81 | Roseburia | Lachnospiraceae | Eubacteriales | Clostridia | Firmicutes |
| Roseburia sp. AM59-24XD (HG3A.0391) | 17.6 | 0.00 | 0.00 | 0.00 | 0.00 | 0.21 | Roseburia | Lachnospiraceae | Eubacteriales | Clostridia | Firmicutes |
| [Ruminococcus] gnavus (HG3A.0239) | 38.2 | 0.00 | 0.00 | 0.00 | 0.01 | 0.32 | Mediterraneibacter | Lachnospiraceae | Eubacteriales | Clostridia | Firmicutes |
| Ruminococcus sp. AM42-11 (HG3A.0002) | 99.0 | 0.04 | 0.13 | 0.28 | 0.55 | 1.36 | Ruminococcus | Oscillospiraceae | Eubacteriales | Clostridia | Firmicutes |
| [Ruminococcus] torques (HG3A.0034) | 91.2 | 0.00 | 0.08 | 0.21 | 0.46 | 1.39 | Mediterraneibacter | Lachnospiraceae | Eubacteriales | Clostridia | Firmicutes |
| Staphylococcus aureus (HG3A.1538) | 2.9 | 0.00 | 0.00 | 0.00 | 0.00 | 0.00 | Staphylococcus | Staphylococcaceae | Bacillales | Bacilli | Firmicutes |
| Traorella massiliensis (HG3A.0669) | 14.1 | 0.00 | 0.00 | 0.00 | 0.00 | 0.01 | Traorella | Erysipelotrichaceae | Erysipelotrichales | Erysipelotrichia | Firmicutes |

Victivallis vadensis (HG3A.0689)

15.9

0.00 0.00

0.00

0.00

0.01 Victivallis

Victivallaceae

Victivallales

Lentisphaeria

Lentisphaerae
