# Supplementary material for: OSA Is Associated With the Human Gut Microbiota Composition and Functional Potential in the Population-Based Swedish CardioPulmonary bioImage Study
Source: Chest. 2023 Mar 15;164(2):503–16. doi: 10.1016/j.chest.2023.03.010 (PMC10410248; doi:10.1016/j.chest.2023.03.010)
Supplement: e-Table 9 [file mmc6.docx]

e-Table 9. Sensitivity analysis for the species associated with T90/ODI in the extended main model. Three sensitivity analyses: medication model, exclusion of antibiotic users, and exclusion of self-reported lung disease

Partial Spearman’s correlations of T90 and ODI with the specified species. Medication model: adjustment for the extended model covariates, metformin use, proton

pump inhibitor use, anti-hypertensive medication use, and use of medication for hyperlipidemia. WHR model: adjustment for the extended model covariates with additional adjustment for waist-hip ratio. Antibiotic sensitivity analysis: removal of 347 participants that used antibiotics six months before sampling. Analysis adjusted for the extended model covariates. Lung disease sensitivity analysis: removal of 29 participants that self-reported a doctor diagnosis of chronic obstructive pulmonary disease, chronic bronchitis, or pulmonary emphysema. Analysis adjusted for the extended model covariates. Adjustment for multiple testing for those species identified in the extended model using the Benjamini-Hochberg method and presented as q-values. Under the column "Metagenomics species", the information between parenthesis is the internal identifier for the respective species. AHI: apnea-hypopnea index; ODI: oxygen desaturation index; and T90: percentage of time with oxygen saturation below 90%.

**WHR Lung**

**Medication**

**Antibiotic**

| **Metagenomic species exposure** | | **correlation p-value q-value N** | | | | **correlation p-value q-value N** | | | | **correlation p-value q-value N** | | | | **correlation p-value q-value N** | | | |
| --- | --- | --- | --- | --- | --- | --- | --- | --- | --- | --- | --- | --- | --- | --- | --- | --- | --- |
| Akkermansia muciniphila  (HG3A.0110) | ODI | -0.053 | 3.39E-03 | 5.45E-03 | 3194 | -0.053 | 3.04E-03 | 4.70E-03 | 3249 | -0.059 | 1.65E-03 | 3.79E-03 | 2913 | -0.057 | 1.42E-03 | 2.64E-03 | 3224 |
| Alistipes communis (HG3A.0064) | ODI | -0.053 | 3.24E-03 | 5.27E-03 | 3194 | -0.059 | 9.15E-04 | 2.85E-03 | 3249 | -0.056 | 3.14E-03 | 5.32E-03 | 2913 | -0.057 | 1.44E-03 | 2.65E-03 | 3224 |
| Alistipes provencensis (HG3A.0877) | ODI | -0.052 | 3.83E-03 | 5.53E-03 | 3194 | -0.045 | 1.10E-02 | 1.14E-02 | 3249 | -0.043 | 2.12E-02 | 2.18E-02 | 2913 | -0.048 | 7.40E-03 | 7.45E-03 | 3224 |
| Alistipes shahii (HG3A.0054) | ODI | -0.065 | 3.00E-04 | 2.34E-03 | 3194 | -0.064 | 3.15E-04 | 2.14E-03 | 3249 | -0.065 | 5.40E-04 | 2.16E-03 | 2913 | -0.061 | 5.73E-04 | 1.65E-03 | 3224 |
| Anaerobutyricum hallii (HG3A.0012) | T90 | 0.061 | 6.90E-04 | 2.91E-03 | 3194 | 0.054 | 2.53E-03 | 4.55E-03 | 3249 | 0.055 | 3.32E-03 | 5.36E-03 | 2913 | 0.053 | 2.78E-03 | 3.51E-03 | 3224 |
| Anaerostipes sp. BG01  (HG3A.1509) | ODI | 0.048 | 8.08E-03 | 8.94E-03 | 3194 | 0.047 | 8.22E-03 | 8.88E-03 | 3249 | 0.062 | 1.04E-03 | 2.84E-03 | 2913 | 0.050 | 5.40E-03 | 5.62E-03 | 3224 |
| Bacteria sp. (HG3A.0483) | ODI | -0.058 | 1.14E-03 | 3.23E-03 | 3194 | -0.064 | 3.42E-04 | 2.14E-03 | 3249 | -0.058 | 2.15E-03 | 4.47E-03 | 2913 | -0.068 | 1.39E-04 | 9.70E-04 | 3224 |
| Bacteria sp. (HG3A.0634) | ODI | -0.063 | 4.19E-04 | 2.50E-03 | 3194 | -0.065 | 2.30E-04 | 2.14E-03 | 3249 | -0.068 | 2.67E-04 | 2.01E-03 | 2913 | -0.069 | 1.09E-04 | 9.48E-04 | 3224 |
| Bacteria sp. (HG3A.0634) | T90 | -0.057 | 1.39E-03 | 3.30E-03 | 3194 | -0.058 | 1.10E-03 | 3.02E-03 | 3249 | -0.061 | 1.27E-03 | 3.06E-03 | 2913 | -0.060 | 7.72E-04 | 1.85E-03 | 3224 |
| Bacteria sp. (HG3A.0911) | T90 | -0.054 | 2.59E-03 | 4.65E-03 | 3194 | -0.052 | 3.26E-03 | 4.95E-03 | 3249 | -0.069 | 2.46E-04 | 2.01E-03 | 2913 | -0.056 | 1.74E-03 | 2.77E-03 | 3224 |
| Blautia massiliensis (HG3A.0023) | ODI | 0.057 | 1.51E-03 | 3.31E-03 | 3194 | 0.058 | 1.13E-03 | 3.03E-03 | 3249 | 0.055 | 3.26E-03 | 5.36E-03 | 2913 | 0.062 | 4.78E-04 | 1.52E-03 | 3224 |
| Blautia obeum (HG3A.0001) | ODI | 0.057 | 1.51E-03 | 3.31E-03 | 3194 | 0.060 | 6.78E-04 | 2.63E-03 | 3249 | 0.054 | 4.07E-03 | 6.10E-03 | 2913 | 0.064 | 3.53E-04 | 1.45E-03 | 3224 |
| Blautia obeum (HG3A.0001) | T90 | 0.079 | 1.04E-05 | 8.15E-04 | 3194 | 0.075 | 2.50E-05 | 1.95E-03 | 3249 | 0.072 | 1.17E-04 | 2.01E-03 | 2913 | 0.080 | 7.65E-06 | 4.72E-04 | 3224 |
| Blautia obeum (HG3A.0009) | T90 | 0.052 | 3.53E-03 | 5.51E-03 | 3194 | 0.058 | 1.14E-03 | 3.03E-03 | 3249 | 0.061 | 1.12E-03 | 2.92E-03 | 2913 | 0.062 | 5.26E-04 | 1.55E-03 | 3224 |
| Candidatus Borkfalkiales sp. (HG3A.1329) | T90 | -0.060 | 8.76E-04 | 2.92E-03 | 3194 | -0.056 | 1.61E-03 | 3.76E-03 | 3249 | -0.066 | 4.83E-04 | 2.09E-03 | 2913 | -0.057 | 1.33E-03 | 2.59E-03 | 3224 |
| Candidatus Borkfalkiales sp.  (HG3A.1397) | ODI | -0.048 | 7.10E-03 | 7.96E-03 | 3194 | -0.053 | 2.92E-03 | 4.70E-03 | 3249 | -0.054 | 4.25E-03 | 6.12E-03 | 2913 | -0.051 | 4.08E-03 | 4.48E-03 | 3224 |
| Clostridia sp. (HG3A.0094) | ODI | -0.047 | 8.97E-03 | 9.71E-03 | 3194 | -0.054 | 2.55E-03 | 4.55E-03 | 3249 | -0.055 | 3.21E-03 | 5.35E-03 | 2913 | -0.054 | 2.42E-03 | 3.21E-03 | 3224 |

| Clostridia sp. (HG3A.0140) | ODI | -0.067 | 1.66E-04 | 2.16E-03 | 3194 | -0.065 | 2.29E-04 | 2.14E-03 | 3249 | -0.066 | 4.58E-04 | 2.09E-03 | 2913 | -0.070 | 8.17E-05 | 9.48E-04 | 3224 |
| --- | --- | --- | --- | --- | --- | --- | --- | --- | --- | --- | --- | --- | --- | --- | --- | --- | --- |
| Clostridia sp. (HG3A.0140) | T90 | -0.071 | 8.11E-05 | 2.11E-03 | 3194 | -0.065 | 2.60E-04 | 2.14E-03 | 3249 | -0.069 | 2.35E-04 | 2.01E-03 | 2913 | -0.069 | 9.95E-05 | 9.48E-04 | 3224 |
| Clostridia sp. (HG3A.0272) | ODI | -0.057 | 1.46E-03 | 3.31E-03 | 3194 | -0.059 | 8.60E-04 | 2.74E-03 | 3249 | -0.066 | 4.58E-04 | 2.09E-03 | 2913 | -0.064 | 3.65E-04 | 1.46E-03 | 3224 |
| Clostridia sp. (HG3A.0435) | ODI | -0.047 | 9.03E-03 | 9.71E-03 | 3194 | -0.054 | 2.60E-03 | 4.55E-03 | 3249 | -0.056 | 3.08E-03 | 5.32E-03 | 2913 | -0.056 | 1.66E-03 | 2.77E-03 | 3224 |
| Clostridia sp. (HG3A.0435) | T90 | -0.061 | 6.44E-04 | 2.79E-03 | 3194 | -0.061 | 5.50E-04 | 2.54E-03 | 3249 | -0.068 | 3.11E-04 | 2.01E-03 | 2913 | -0.065 | 2.85E-04 | 1.35E-03 | 3224 |
| Clostridia sp. (HG3A.0470) | ODI | -0.051 | 4.81E-03 | 6.25E-03 | 3194 | -0.053 | 2.81E-03 | 4.70E-03 | 3249 | -0.048 | 1.11E-02 | 1.22E-02 | 2913 | -0.057 | 1.47E-03 | 2.66E-03 | 3224 |
| Clostridia sp. (HG3A.0470) | T90 | -0.060 | 8.69E-04 | 2.92E-03 | 3194 | -0.063 | 4.13E-04 | 2.31E-03 | 3249 | -0.066 | 4.66E-04 | 2.09E-03 | 2913 | -0.063 | 4.33E-04 | 1.52E-03 | 3224 |
| Clostridia sp. (HG3A.0508) | ODI | -0.049 | 6.05E-03 | 6.99E-03 | 3194 | -0.053 | 2.82E-03 | 4.70E-03 | 3249 | -0.057 | 2.49E-03 | 4.68E-03 | 2913 | -0.055 | 1.96E-03 | 2.83E-03 | 3224 |
| Clostridia sp. (HG3A.0508) | T90 | -0.058 | 1.34E-03 | 3.30E-03 | 3194 | -0.059 | 8.25E-04 | 2.74E-03 | 3249 | -0.067 | 3.33E-04 | 2.01E-03 | 2913 | -0.061 | 6.63E-04 | 1.72E-03 | 3224 |
| Clostridia sp. (HG3A.0515) | ODI | -0.063 | 4.79E-04 | 2.50E-03 | 3194 | -0.067 | 1.56E-04 | 2.03E-03 | 3249 | -0.061 | 1.27E-03 | 3.06E-03 | 2913 | -0.071 | 6.73E-05 | 9.48E-04 | 3224 |
| Clostridia sp. (HG3A.0515) | T90 | -0.046 | 1.02E-02 | 1.08E-02 | 3194 | -0.050 | 4.82E-03 | 6.32E-03 | 3249 | -0.052 | 6.01E-03 | 7.62E-03 | 2913 | -0.056 | 1.68E-03 | 2.77E-03 | 3224 |
| Clostridia sp. (HG3A.0550) | T90 | -0.055 | 2.22E-03 | 4.12E-03 | 3194 | -0.053 | 3.03E-03 | 4.70E-03 | 3249 | -0.054 | 3.91E-03 | 5.97E-03 | 2913 | -0.055 | 1.89E-03 | 2.83E-03 | 3224 |
| Clostridia sp. (HG3A.0599) | ODI | -0.050 | 5.33E-03 | 6.58E-03 | 3194 | -0.054 | 2.26E-03 | 4.35E-03 | 3249 | -0.054 | 4.31E-03 | 6.12E-03 | 2913 | -0.058 | 1.16E-03 | 2.52E-03 | 3224 |
| Clostridia sp. (HG3A.0645) | T90 | -0.056 | 1.90E-03 | 3.85E-03 | 3194 | -0.056 | 1.48E-03 | 3.49E-03 | 3249 | -0.054 | 3.94E-03 | 5.97E-03 | 2913 | -0.054 | 2.49E-03 | 3.21E-03 | 3224 |
| Clostridia sp. (HG3A.0682) | ODI | -0.055 | 2.05E-03 | 4.02E-03 | 3194 | -0.057 | 1.32E-03 | 3.39E-03 | 3249 | -0.063 | 7.97E-04 | 2.54E-03 | 2913 | -0.062 | 4.61E-04 | 1.52E-03 | 3224 |
| Clostridia sp. (HG3A.0728) | ODI | -0.062 | 5.29E-04 | 2.50E-03 | 3194 | -0.061 | 5.84E-04 | 2.54E-03 | 3249 | -0.069 | 2.46E-04 | 2.01E-03 | 2913 | -0.063 | 4.16E-04 | 1.52E-03 | 3224 |
| Clostridia sp. (HG3A.0815) | ODI | -0.051 | 4.51E-03 | 5.97E-03 | 3194 | -0.049 | 5.61E-03 | 6.84E-03 | 3249 | -0.047 | 1.24E-02 | 1.32E-02 | 2913 | -0.053 | 2.94E-03 | 3.64E-03 | 3224 |
| Clostridia sp. (HG3A.0861) | T90 | -0.056 | 1.72E-03 | 3.62E-03 | 3194 | -0.059 | 9.91E-04 | 2.96E-03 | 3249 | -0.058 | 2.07E-03 | 4.43E-03 | 2913 | -0.057 | 1.28E-03 | 2.59E-03 | 3224 |
| Clostridia sp. (HG3A.0879) | ODI | -0.049 | 6.11E-03 | 7.01E-03 | 3194 | -0.047 | 8.45E-03 | 8.97E-03 | 3249 | -0.050 | 7.51E-03 | 8.87E-03 | 2913 | -0.052 | 3.87E-03 | 4.31E-03 | 3224 |
| Clostridia sp. (HG3A.0931) | T90 | -0.055 | 2.17E-03 | 4.12E-03 | 3194 | -0.053 | 2.87E-03 | 4.70E-03 | 3249 | -0.063 | 8.47E-04 | 2.56E-03 | 2913 | -0.057 | 1.27E-03 | 2.59E-03 | 3224 |
| Clostridia sp. (HG3A.1008) | T90 | -0.058 | 1.14E-03 | 3.23E-03 | 3194 | -0.055 | 1.85E-03 | 3.85E-03 | 3249 | -0.057 | 2.32E-03 | 4.53E-03 | 2913 | -0.054 | 2.47E-03 | 3.21E-03 | 3224 |
| Clostridia sp. (HG3A.1020) | ODI | -0.047 | 9.44E-03 | 1.01E-02 | 3194 | -0.046 | 9.27E-03 | 9.71E-03 | 3249 | -0.047 | 1.23E-02 | 1.32E-02 | 2913 | -0.050 | 5.26E-03 | 5.51E-03 | 3224 |
| Clostridia sp. (HG3A.1057) | T90 | -0.058 | 1.18E-03 | 3.23E-03 | 3194 | -0.049 | 5.74E-03 | 6.88E-03 | 3249 | -0.055 | 3.22E-03 | 5.35E-03 | 2913 | -0.054 | 2.48E-03 | 3.21E-03 | 3224 |
| Clostridiaceae sp. (HG3A.0431) | T90 | 0.060 | 7.45E-04 | 2.92E-03 | 3194 | 0.054 | 2.32E-03 | 4.41E-03 | 3249 | 0.053 | 4.87E-03 | 6.66E-03 | 2913 | 0.055 | 2.20E-03 | 3.01E-03 | 3224 |
| Clostridium sp. (HG3A.0050) | ODI | 0.054 | 2.80E-03 | 4.92E-03 | 3194 | 0.050 | 5.24E-03 | 6.65E-03 | 3249 | 0.050 | 8.02E-03 | 9.22E-03 | 2913 | 0.053 | 3.07E-03 | 3.74E-03 | 3224 |
| Clostridium sp. (HG3A.0050) | T90 | 0.051 | 4.22E-03 | 5.67E-03 | 3194 | 0.049 | 5.77E-03 | 6.88E-03 | 3249 | 0.054 | 4.26E-03 | 6.12E-03 | 2913 | 0.052 | 3.29E-03 | 3.80E-03 | 3224 |
| Clostridium sp. TF06-15AC  (HG3A.0032) | ODI | 0.061 | 7.26E-04 | 2.92E-03 | 3194 | 0.061 | 6.24E-04 | 2.56E-03 | 3249 | 0.070 | 1.75E-04 | 2.01E-03 | 2913 | 0.063 | 3.92E-04 | 1.52E-03 | 3224 |
| Collinsella aerofaciens (HG3A.0019) | ODI | 0.047 | 8.25E-03 | 9.06E-03 | 3194 | 0.053 | 3.04E-03 | 4.70E-03 | 3249 | 0.052 | 5.23E-03 | 7.01E-03 | 2913 | 0.053 | 3.06E-03 | 3.74E-03 | 3224 |
| Coprobacillus sp. (HG3A.0022) | ODI | 0.055 | 2.06E-03 | 4.02E-03 | 3194 | 0.047 | 7.97E-03 | 8.69E-03 | 3249 | 0.051 | 7.17E-03 | 8.70E-03 | 2913 | 0.052 | 3.52E-03 | 3.98E-03 | 3224 |
| Coprococcus comes (HG3A.0016) | ODI | 0.066 | 2.14E-04 | 2.18E-03 | 3194 | 0.064 | 3.21E-04 | 2.14E-03 | 3249 | 0.062 | 9.84E-04 | 2.74E-03 | 2913 | 0.065 | 2.66E-04 | 1.35E-03 | 3224 |
| Coprococcus comes (HG3A.0016) | T90 | 0.062 | 5.29E-04 | 2.50E-03 | 3194 | 0.058 | 1.03E-03 | 2.96E-03 | 3249 | 0.058 | 1.85E-03 | 4.12E-03 | 2913 | 0.065 | 2.93E-04 | 1.35E-03 | 3224 |

| Coprococcus eutactus (HG3A.0155) | ODI | -0.051 | 4.11E-03 | 5.67E-03 | 3194 | -0.053 | 3.01E-03 | 4.70E-03 | 3249 | -0.052 | 5.95E-03 | 7.61E-03 | 2913 | -0.054 | 2.59E-03 | 3.31E-03 | 3224 |
| --- | --- | --- | --- | --- | --- | --- | --- | --- | --- | --- | --- | --- | --- | --- | --- | --- | --- |
| Dorea formicigenerans  (HG3A.0006) | T90 | 0.085 | 1.98E-06 | 3.09E-04 | 3194 | 0.084 | 2.41E-06 | 3.77E-04 | 3249 | 0.079 | 2.30E-05 | 1.84E-03 | 2913 | 0.086 | 1.33E-06 | 2.08E-04 | 3224 |
| Dorea sp. AF36-15AT (HG3A.0052) | T90 | 0.057 | 1.56E-03 | 3.38E-03 | 3194 | 0.052 | 3.27E-03 | 4.95E-03 | 3249 | 0.056 | 3.09E-03 | 5.32E-03 | 2913 | 0.056 | 1.84E-03 | 2.83E-03 | 3224 |
| Eggerthellaceae sp. (HG3A.0171) | ODI | -0.050 | 5.15E-03 | 6.58E-03 | 3194 | -0.050 | 5.00E-03 | 6.45E-03 | 3249 | -0.069 | 2.17E-04 | 2.01E-03 | 2913 | -0.050 | 4.66E-03 | 5.02E-03 | 3224 |
| Eggerthellales sp. (HG3A.0177) | ODI | -0.058 | 1.23E-03 | 3.25E-03 | 3194 | -0.061 | 5.79E-04 | 2.54E-03 | 3249 | -0.063 | 8.20E-04 | 2.56E-03 | 2913 | -0.065 | 2.59E-04 | 1.35E-03 | 3224 |
| Erysipelotrichales sp. (HG3A.1207) | T90 | 0.057 | 1.50E-03 | 3.31E-03 | 3194 | 0.055 | 2.04E-03 | 4.10E-03 | 3249 | 0.057 | 2.44E-03 | 4.68E-03 | 2913 | 0.055 | 1.86E-03 | 2.83E-03 | 3224 |
| Eubacteriales sp. (HG3A.0069) | ODI | -0.068 | 1.54E-04 | 2.16E-03 | 3194 | -0.071 | 7.15E-05 | 2.03E-03 | 3249 | -0.079 | 2.36E-05 | 1.84E-03 | 2913 | -0.076 | 1.80E-05 | 5.41E-04 | 3224 |
| Eubacteriales sp. (HG3A.0083) | ODI | -0.045 | 1.16E-02 | 1.21E-02 | 3194 | -0.043 | 1.47E-02 | 1.47E-02 | 3249 | -0.052 | 5.30E-03 | 7.01E-03 | 2913 | -0.050 | 4.75E-03 | 5.04E-03 | 3224 |
| Eubacteriales sp. (HG3A.0084) | ODI | -0.053 | 3.15E-03 | 5.16E-03 | 3194 | -0.050 | 4.70E-03 | 6.21E-03 | 3249 | -0.052 | 5.27E-03 | 7.01E-03 | 2913 | -0.056 | 1.58E-03 | 2.77E-03 | 3224 |
| Eubacteriales sp. (HG3A.0085) | ODI | -0.050 | 5.72E-03 | 6.77E-03 | 3194 | -0.055 | 2.11E-03 | 4.17E-03 | 3249 | -0.061 | 1.24E-03 | 3.06E-03 | 2913 | -0.055 | 2.09E-03 | 2.91E-03 | 3224 |
| Eubacteriales sp. (HG3A.0100) | ODI | -0.063 | 4.12E-04 | 2.50E-03 | 3194 | -0.068 | 1.43E-04 | 2.03E-03 | 3249 | -0.072 | 1.26E-04 | 2.01E-03 | 2913 | -0.075 | 2.43E-05 | 5.41E-04 | 3224 |
| Eubacteriales sp. (HG3A.0100) | T90 | -0.060 | 8.29E-04 | 2.92E-03 | 3194 | -0.056 | 1.73E-03 | 3.79E-03 | 3249 | -0.066 | 4.50E-04 | 2.09E-03 | 2913 | -0.059 | 9.37E-04 | 2.19E-03 | 3224 |
| Eubacteriales sp. (HG3A.0118) | ODI | -0.060 | 8.62E-04 | 2.92E-03 | 3194 | -0.056 | 1.68E-03 | 3.78E-03 | 3249 | -0.070 | 2.11E-04 | 2.01E-03 | 2913 | -0.061 | 6.61E-04 | 1.72E-03 | 3224 |
| Eubacteriales sp. (HG3A.0118) | T90 | -0.061 | 6.25E-04 | 2.78E-03 | 3194 | -0.057 | 1.45E-03 | 3.49E-03 | 3249 | -0.064 | 6.67E-04 | 2.40E-03 | 2913 | -0.060 | 7.38E-04 | 1.80E-03 | 3224 |
| Eubacteriales sp. (HG3A.0120) | ODI | -0.043 | 1.55E-02 | 1.57E-02 | 3194 | -0.047 | 8.31E-03 | 8.88E-03 | 3249 | -0.050 | 8.04E-03 | 9.22E-03 | 2913 | -0.050 | 4.92E-03 | 5.18E-03 | 3224 |
| Eubacteriales sp. (HG3A.0123) | T90 | 0.060 | 8.39E-04 | 2.92E-03 | 3194 | 0.060 | 7.57E-04 | 2.74E-03 | 3249 | 0.064 | 6.12E-04 | 2.27E-03 | 2913 | 0.061 | 6.91E-04 | 1.74E-03 | 3224 |
| Eubacteriales sp. (HG3A.0149) | ODI | -0.069 | 1.22E-04 | 2.14E-03 | 3194 | -0.067 | 1.53E-04 | 2.03E-03 | 3249 | -0.075 | 6.51E-05 | 2.01E-03 | 2913 | -0.076 | 2.22E-05 | 5.41E-04 | 3224 |
| Eubacteriales sp. (HG3A.0149) | T90 | -0.067 | 1.83E-04 | 2.18E-03 | 3194 | -0.063 | 4.14E-04 | 2.31E-03 | 3249 | -0.071 | 1.43E-04 | 2.01E-03 | 2913 | -0.071 | 7.56E-05 | 9.48E-04 | 3224 |
| Eubacteriales sp. (HG3A.0153) | ODI | -0.044 | 1.50E-02 | 1.53E-02 | 3194 | -0.048 | 6.59E-03 | 7.45E-03 | 3249 | -0.050 | 7.38E-03 | 8.83E-03 | 2913 | -0.053 | 2.91E-03 | 3.63E-03 | 3224 |
| Eubacteriales sp. (HG3A.0156) | ODI | -0.064 | 3.97E-04 | 2.50E-03 | 3194 | -0.061 | 5.49E-04 | 2.54E-03 | 3249 | -0.067 | 3.35E-04 | 2.01E-03 | 2913 | -0.069 | 1.09E-04 | 9.48E-04 | 3224 |
| Eubacteriales sp. (HG3A.0162) | ODI | -0.058 | 1.25E-03 | 3.25E-03 | 3194 | -0.054 | 2.45E-03 | 4.55E-03 | 3249 | -0.066 | 4.12E-04 | 2.09E-03 | 2913 | -0.063 | 4.46E-04 | 1.52E-03 | 3224 |
| Eubacteriales sp. (HG3A.0162) | T90 | -0.063 | 4.84E-04 | 2.50E-03 | 3194 | -0.060 | 7.77E-04 | 2.74E-03 | 3249 | -0.070 | 1.80E-04 | 2.01E-03 | 2913 | -0.063 | 4.47E-04 | 1.52E-03 | 3224 |
| Eubacteriales sp. (HG3A.0193) | ODI | -0.050 | 5.12E-03 | 6.58E-03 | 3194 | -0.052 | 3.47E-03 | 5.11E-03 | 3249 | -0.066 | 4.43E-04 | 2.09E-03 | 2913 | -0.055 | 2.05E-03 | 2.88E-03 | 3224 |
| Eubacteriales sp. (HG3A.0196) | ODI | -0.052 | 3.65E-03 | 5.52E-03 | 3194 | -0.054 | 2.58E-03 | 4.55E-03 | 3249 | -0.054 | 3.93E-03 | 5.97E-03 | 2913 | -0.061 | 6.41E-04 | 1.72E-03 | 3224 |
| Eubacteriales sp. (HG3A.0196) | T90 | -0.050 | 5.35E-03 | 6.58E-03 | 3194 | -0.048 | 6.42E-03 | 7.36E-03 | 3249 | -0.057 | 2.48E-03 | 4.68E-03 | 2913 | -0.053 | 3.22E-03 | 3.75E-03 | 3224 |
| Eubacteriales sp. (HG3A.0197) | ODI | -0.064 | 3.44E-04 | 2.50E-03 | 3194 | -0.069 | 9.80E-05 | 2.03E-03 | 3249 | -0.068 | 3.01E-04 | 2.01E-03 | 2913 | -0.074 | 3.37E-05 | 5.97E-04 | 3224 |
| Eubacteriales sp. (HG3A.0211) | ODI | -0.063 | 4.78E-04 | 2.50E-03 | 3194 | -0.061 | 5.87E-04 | 2.54E-03 | 3249 | -0.068 | 2.82E-04 | 2.01E-03 | 2913 | -0.065 | 2.94E-04 | 1.35E-03 | 3224 |
| Eubacteriales sp. (HG3A.0211) | T90 | -0.057 | 1.35E-03 | 3.30E-03 | 3194 | -0.054 | 2.56E-03 | 4.55E-03 | 3249 | -0.053 | 4.61E-03 | 6.43E-03 | 2913 | -0.056 | 1.73E-03 | 2.77E-03 | 3224 |
| Eubacteriales sp. (HG3A.0215) | ODI | -0.052 | 3.80E-03 | 5.53E-03 | 3194 | -0.052 | 3.35E-03 | 4.98E-03 | 3249 | -0.056 | 2.93E-03 | 5.25E-03 | 2913 | -0.058 | 1.14E-03 | 2.50E-03 | 3224 |
| Eubacteriales sp. (HG3A.0226) | ODI | -0.057 | 1.42E-03 | 3.30E-03 | 3194 | -0.062 | 5.04E-04 | 2.54E-03 | 3249 | -0.060 | 1.38E-03 | 3.25E-03 | 2913 | -0.068 | 1.49E-04 | 9.70E-04 | 3224 |

| Eubacteriales sp. (HG3A.0229) | ODI | -0.041 | 2.07E-02 | 2.09E-02 | 3194 | -0.045 | 1.16E-02 | 1.19E-02 | 3249 | -0.041 | 2.91E-02 | 2.93E-02 | 2913 | -0.048 | 6.56E-03 | 6.64E-03 | 3224 |
| --- | --- | --- | --- | --- | --- | --- | --- | --- | --- | --- | --- | --- | --- | --- | --- | --- | --- |
| Eubacteriales sp. (HG3A.0234) | T90 | -0.059 | 9.47E-04 | 3.08E-03 | 3194 | -0.056 | 1.70E-03 | 3.78E-03 | 3249 | -0.057 | 2.26E-03 | 4.47E-03 | 2913 | -0.061 | 6.88E-04 | 1.74E-03 | 3224 |
| Eubacteriales sp. (HG3A.0242) | ODI | -0.057 | 1.36E-03 | 3.30E-03 | 3194 | -0.055 | 1.82E-03 | 3.84E-03 | 3249 | -0.053 | 5.13E-03 | 6.95E-03 | 2913 | -0.062 | 4.95E-04 | 1.54E-03 | 3224 |
| Eubacteriales sp. (HG3A.0242) | T90 | -0.064 | 3.72E-04 | 2.50E-03 | 3194 | -0.059 | 9.35E-04 | 2.86E-03 | 3249 | -0.062 | 8.80E-04 | 2.59E-03 | 2913 | -0.064 | 3.13E-04 | 1.36E-03 | 3224 |
| Eubacteriales sp. (HG3A.0250) | ODI | -0.054 | 2.39E-03 | 4.38E-03 | 3194 | -0.052 | 3.66E-03 | 5.33E-03 | 3249 | -0.056 | 2.82E-03 | 5.18E-03 | 2913 | -0.055 | 1.91E-03 | 2.83E-03 | 3224 |
| Eubacteriales sp. (HG3A.0269) | ODI | -0.062 | 5.96E-04 | 2.74E-03 | 3194 | -0.064 | 3.30E-04 | 2.14E-03 | 3249 | -0.065 | 5.28E-04 | 2.16E-03 | 2913 | -0.069 | 1.17E-04 | 9.48E-04 | 3224 |
| Eubacteriales sp. (HG3A.0291) | ODI | -0.052 | 3.69E-03 | 5.52E-03 | 3194 | -0.050 | 5.10E-03 | 6.52E-03 | 3249 | -0.053 | 4.69E-03 | 6.47E-03 | 2913 | -0.055 | 1.95E-03 | 2.83E-03 | 3224 |
| Eubacteriales sp. (HG3A.0291) | T90 | -0.058 | 1.34E-03 | 3.30E-03 | 3194 | -0.051 | 3.93E-03 | 5.47E-03 | 3249 | -0.055 | 3.34E-03 | 5.36E-03 | 2913 | -0.055 | 1.98E-03 | 2.83E-03 | 3224 |
| Eubacteriales sp. (HG3A.0309) | ODI | -0.056 | 1.93E-03 | 3.85E-03 | 3194 | -0.052 | 3.31E-03 | 4.96E-03 | 3249 | -0.052 | 5.59E-03 | 7.27E-03 | 2913 | -0.057 | 1.31E-03 | 2.59E-03 | 3224 |
| Eubacteriales sp. (HG3A.0311) | ODI | -0.060 | 8.57E-04 | 2.92E-03 | 3194 | -0.060 | 6.91E-04 | 2.63E-03 | 3249 | -0.064 | 7.12E-04 | 2.42E-03 | 2913 | -0.064 | 3.10E-04 | 1.36E-03 | 3224 |
| Eubacteriales sp. (HG3A.0311) | T90 | -0.058 | 1.18E-03 | 3.23E-03 | 3194 | -0.054 | 2.45E-03 | 4.55E-03 | 3249 | -0.056 | 3.01E-03 | 5.32E-03 | 2913 | -0.060 | 7.29E-04 | 1.80E-03 | 3224 |
| Eubacteriales sp. (HG3A.0321) | ODI | -0.052 | 3.47E-03 | 5.51E-03 | 3194 | -0.051 | 3.84E-03 | 5.40E-03 | 3249 | -0.054 | 4.10E-03 | 6.10E-03 | 2913 | -0.058 | 1.10E-03 | 2.46E-03 | 3224 |
| Eubacteriales sp. (HG3A.0321) | T90 | -0.053 | 2.91E-03 | 5.00E-03 | 3194 | -0.049 | 6.15E-03 | 7.16E-03 | 3249 | -0.058 | 2.21E-03 | 4.47E-03 | 2913 | -0.057 | 1.36E-03 | 2.59E-03 | 3224 |
| Eubacteriales sp. (HG3A.0329) | ODI | -0.044 | 1.35E-02 | 1.38E-02 | 3194 | -0.044 | 1.25E-02 | 1.27E-02 | 3249 | -0.051 | 6.48E-03 | 8.08E-03 | 2913 | -0.047 | 8.17E-03 | 8.17E-03 | 3224 |
| Eubacteriales sp. (HG3A.0331) | ODI | -0.060 | 8.79E-04 | 2.92E-03 | 3194 | -0.057 | 1.24E-03 | 3.21E-03 | 3249 | -0.057 | 2.23E-03 | 4.47E-03 | 2913 | -0.066 | 2.05E-04 | 1.18E-03 | 3224 |
| Eubacteriales sp. (HG3A.0383) | T90 | -0.051 | 4.21E-03 | 5.67E-03 | 3194 | -0.047 | 8.26E-03 | 8.88E-03 | 3249 | -0.051 | 6.76E-03 | 8.30E-03 | 2913 | -0.052 | 3.42E-03 | 3.89E-03 | 3224 |
| Eubacteriales sp. (HG3A.0419) | ODI | -0.050 | 5.38E-03 | 6.58E-03 | 3194 | -0.046 | 9.10E-03 | 9.59E-03 | 3249 | -0.044 | 1.87E-02 | 1.95E-02 | 2913 | -0.049 | 5.72E-03 | 5.83E-03 | 3224 |
| Eubacteriales sp. (HG3A.0419) | T90 | -0.065 | 2.71E-04 | 2.23E-03 | 3194 | -0.064 | 2.84E-04 | 2.14E-03 | 3249 | -0.061 | 1.10E-03 | 2.92E-03 | 2913 | -0.065 | 2.62E-04 | 1.35E-03 | 3224 |
| Eubacteriales sp. (HG3A.0421) | ODI | -0.058 | 1.17E-03 | 3.23E-03 | 3194 | -0.056 | 1.69E-03 | 3.78E-03 | 3249 | -0.053 | 4.61E-03 | 6.43E-03 | 2913 | -0.064 | 3.52E-04 | 1.45E-03 | 3224 |
| Eubacteriales sp. (HG3A.0439) | ODI | -0.051 | 4.24E-03 | 5.67E-03 | 3194 | -0.048 | 7.20E-03 | 8.03E-03 | 3249 | -0.050 | 7.72E-03 | 8.99E-03 | 2913 | -0.051 | 4.33E-03 | 4.72E-03 | 3224 |
| Eubacteriales sp. (HG3A.0442) | ODI | -0.072 | 5.40E-05 | 2.11E-03 | 3194 | -0.071 | 6.34E-05 | 2.03E-03 | 3249 | -0.072 | 1.22E-04 | 2.01E-03 | 2913 | -0.078 | 1.21E-05 | 4.72E-04 | 3224 |
| Eubacteriales sp. (HG3A.0468) | T90 | -0.066 | 2.11E-04 | 2.18E-03 | 3194 | -0.064 | 3.31E-04 | 2.14E-03 | 3249 | -0.066 | 4.81E-04 | 2.09E-03 | 2913 | -0.062 | 4.74E-04 | 1.52E-03 | 3224 |
| Eubacteriales sp. (HG3A.0489) | T90 | -0.048 | 7.15E-03 | 7.96E-03 | 3194 | -0.048 | 6.52E-03 | 7.42E-03 | 3249 | -0.047 | 1.18E-02 | 1.28E-02 | 2913 | -0.054 | 2.48E-03 | 3.21E-03 | 3224 |
| Eubacteriales sp. (HG3A.0506) | ODI | -0.059 | 1.07E-03 | 3.23E-03 | 3194 | -0.061 | 5.54E-04 | 2.54E-03 | 3249 | -0.061 | 1.23E-03 | 3.06E-03 | 2913 | -0.066 | 2.03E-04 | 1.18E-03 | 3224 |
| Eubacteriales sp. (HG3A.0506) | T90 | -0.053 | 2.92E-03 | 5.00E-03 | 3194 | -0.051 | 3.80E-03 | 5.39E-03 | 3249 | -0.056 | 3.13E-03 | 5.32E-03 | 2913 | -0.055 | 1.97E-03 | 2.83E-03 | 3224 |
| Eubacteriales sp. (HG3A.0531) | T90 | -0.058 | 1.22E-03 | 3.25E-03 | 3194 | -0.053 | 3.02E-03 | 4.70E-03 | 3249 | -0.064 | 6.76E-04 | 2.40E-03 | 2913 | -0.055 | 1.94E-03 | 2.83E-03 | 3224 |
| Eubacteriales sp. (HG3A.0548) | ODI | -0.049 | 6.66E-03 | 7.53E-03 | 3194 | -0.047 | 7.78E-03 | 8.54E-03 | 3249 | -0.057 | 2.24E-03 | 4.47E-03 | 2913 | -0.051 | 4.53E-03 | 4.90E-03 | 3224 |
| Eubacteriales sp. (HG3A.0572) | ODI | -0.065 | 2.66E-04 | 2.23E-03 | 3194 | -0.064 | 3.23E-04 | 2.14E-03 | 3249 | -0.065 | 5.33E-04 | 2.16E-03 | 2913 | -0.068 | 1.22E-04 | 9.48E-04 | 3224 |
| Eubacteriales sp. (HG3A.0609) | ODI | -0.062 | 5.19E-04 | 2.50E-03 | 3194 | -0.059 | 8.24E-04 | 2.74E-03 | 3249 | -0.064 | 6.10E-04 | 2.27E-03 | 2913 | -0.061 | 6.60E-04 | 1.72E-03 | 3224 |
| Eubacteriales sp. (HG3A.0630) | T90 | -0.052 | 3.71E-03 | 5.52E-03 | 3194 | -0.057 | 1.47E-03 | 3.49E-03 | 3249 | -0.054 | 3.77E-03 | 5.93E-03 | 2913 | -0.055 | 2.02E-03 | 2.87E-03 | 3224 |
| Eubacteriales sp. (HG3A.0635) | ODI | -0.052 | 3.62E-03 | 5.52E-03 | 3194 | -0.055 | 2.16E-03 | 4.21E-03 | 3249 | -0.054 | 4.25E-03 | 6.12E-03 | 2913 | -0.055 | 2.14E-03 | 2.95E-03 | 3224 |
| Eubacteriales sp. (HG3A.0635) | T90 | -0.054 | 2.80E-03 | 4.92E-03 | 3194 | -0.060 | 8.01E-04 | 2.74E-03 | 3249 | -0.063 | 7.74E-04 | 2.52E-03 | 2913 | -0.056 | 1.66E-03 | 2.77E-03 | 3224 |
| Eubacteriales sp. (HG3A.0691) | ODI | -0.052 | 3.68E-03 | 5.52E-03 | 3194 | -0.049 | 5.57E-03 | 6.84E-03 | 3249 | -0.037 | 4.79E-02 | 4.79E-02 | 2913 | -0.051 | 3.97E-03 | 4.40E-03 | 3224 |
| Eubacteriales sp. (HG3A.0703) | T90 | -0.072 | 6.26E-05 | 2.11E-03 | 3194 | -0.069 | 1.08E-04 | 2.03E-03 | 3249 | -0.075 | 7.00E-05 | 2.01E-03 | 2913 | -0.074 | 3.45E-05 | 5.97E-04 | 3224 |

| Eubacteriales sp. (HG3A.0718) | ODI | -0.050 | 5.40E-03 | 6.58E-03 | 3194 | -0.047 | 7.54E-03 | 8.34E-03 | 3249 | -0.049 | 9.11E-03 | 1.03E-02 | 2913 | -0.053 | 3.16E-03 | 3.75E-03 | 3224 |
| --- | --- | --- | --- | --- | --- | --- | --- | --- | --- | --- | --- | --- | --- | --- | --- | --- | --- |
| Eubacteriales sp. (HG3A.0786) | T90 | 0.050 | 5.53E-03 | 6.66E-03 | 3194 | 0.052 | 3.71E-03 | 5.33E-03 | 3249 | 0.058 | 2.11E-03 | 4.45E-03 | 2913 | 0.053 | 2.79E-03 | 3.51E-03 | 3224 |
| Eubacteriales sp. (HG3A.0829) | ODI | -0.066 | 2.37E-04 | 2.18E-03 | 3194 | -0.061 | 5.94E-04 | 2.54E-03 | 3249 | -0.067 | 3.88E-04 | 2.09E-03 | 2913 | -0.062 | 5.06E-04 | 1.55E-03 | 3224 |
| Eubacteriales sp. (HG3A.0956) | ODI | -0.055 | 2.17E-03 | 4.12E-03 | 3194 | -0.049 | 5.77E-03 | 6.88E-03 | 3249 | -0.047 | 1.32E-02 | 1.40E-02 | 2913 | -0.053 | 3.15E-03 | 3.75E-03 | 3224 |
| Eubacteriales sp. (HG3A.0978) | T90 | -0.052 | 3.83E-03 | 5.53E-03 | 3194 | -0.049 | 5.92E-03 | 6.99E-03 | 3249 | -0.058 | 2.02E-03 | 4.38E-03 | 2913 | -0.054 | 2.37E-03 | 3.18E-03 | 3224 |
| Eubacteriales sp. (HG3A.1019) | ODI | -0.046 | 1.01E-02 | 1.07E-02 | 3194 | -0.048 | 6.69E-03 | 7.51E-03 | 3249 | -0.045 | 1.58E-02 | 1.65E-02 | 2913 | -0.050 | 5.49E-03 | 5.67E-03 | 3224 |
| Eubacteriales sp. (HG3A.1026) | ODI | -0.051 | 4.67E-03 | 6.12E-03 | 3194 | -0.058 | 1.07E-03 | 2.98E-03 | 3249 | -0.050 | 7.42E-03 | 8.83E-03 | 2913 | -0.056 | 1.65E-03 | 2.77E-03 | 3224 |
| Eubacteriales sp. (HG3A.1294) | ODI | -0.050 | 5.32E-03 | 6.58E-03 | 3194 | -0.050 | 5.30E-03 | 6.67E-03 | 3249 | -0.046 | 1.39E-02 | 1.47E-02 | 2913 | -0.049 | 5.66E-03 | 5.80E-03 | 3224 |
| Eubacteriales sp. (HG3A.1379) | T90 | -0.052 | 3.87E-03 | 5.55E-03 | 3194 | -0.049 | 6.30E-03 | 7.27E-03 | 3249 | -0.056 | 2.66E-03 | 4.93E-03 | 2913 | -0.058 | 1.04E-03 | 2.38E-03 | 3224 |
| Eubacterium sp. (HG3A.0214) | ODI | 0.049 | 6.60E-03 | 7.51E-03 | 3194 | 0.053 | 2.68E-03 | 4.64E-03 | 3249 | 0.048 | 1.03E-02 | 1.14E-02 | 2913 | 0.053 | 3.20E-03 | 3.75E-03 | 3224 |
| Firmicutes sp. (HG3A.0301) | T90 | -0.050 | 5.19E-03 | 6.58E-03 | 3194 | -0.051 | 4.10E-03 | 5.57E-03 | 3249 | -0.052 | 5.48E-03 | 7.18E-03 | 2913 | -0.054 | 2.36E-03 | 3.18E-03 | 3224 |
| Firmicutes sp. (HG3A.0341) | ODI | -0.050 | 5.55E-03 | 6.66E-03 | 3194 | -0.053 | 2.84E-03 | 4.70E-03 | 3249 | -0.052 | 5.68E-03 | 7.32E-03 | 2913 | -0.058 | 1.05E-03 | 2.38E-03 | 3224 |
| Firmicutes sp. (HG3A.0397) | ODI | -0.066 | 2.25E-04 | 2.18E-03 | 3194 | -0.066 | 2.02E-04 | 2.14E-03 | 3249 | -0.063 | 7.67E-04 | 2.52E-03 | 2913 | -0.068 | 1.46E-04 | 9.70E-04 | 3224 |
| Firmicutes sp. (HG3A.0398) | ODI | -0.059 | 9.74E-04 | 3.10E-03 | 3194 | -0.060 | 6.65E-04 | 2.63E-03 | 3249 | -0.059 | 1.84E-03 | 4.12E-03 | 2913 | -0.065 | 2.68E-04 | 1.35E-03 | 3224 |
| Firmicutes sp. (HG3A.1085) | ODI | -0.052 | 3.95E-03 | 5.55E-03 | 3194 | -0.051 | 4.11E-03 | 5.57E-03 | 3249 | -0.042 | 2.45E-02 | 2.50E-02 | 2913 | -0.052 | 3.68E-03 | 4.13E-03 | 3224 |
| Flavonifractor plautii (HG3A.0079) | ODI | 0.041 | 2.09E-02 | 2.09E-02 | 3194 | 0.044 | 1.35E-02 | 1.36E-02 | 3249 | 0.051 | 7.19E-03 | 8.70E-03 | 2913 | 0.053 | 3.11E-03 | 3.75E-03 | 3224 |
| Fusicatenibacter saccharivorans (HG3A.0004) | ODI | 0.071 | 7.46E-05 | 2.11E-03 | 3194 | 0.069 | 9.87E-05 | 2.03E-03 | 3249 | 0.068 | 2.88E-04 | 2.01E-03 | 2913 | 0.068 | 1.28E-04 | 9.48E-04 | 3224 |
| Intestinibacillus sp. Marseille-P4005  (HG3A.0168) | ODI | 0.050 | 5.73E-03 | 6.77E-03 | 3194 | 0.049 | 5.57E-03 | 6.84E-03 | 3249 | 0.054 | 4.32E-03 | 6.12E-03 | 2913 | 0.057 | 1.35E-03 | 2.59E-03 | 3224 |
| Intestinimonas massiliensis (HG3A.0198) | ODI | -0.052 | 3.53E-03 | 5.51E-03 | 3194 | -0.052 | 3.72E-03 | 5.33E-03 | 3249 | -0.056 | 2.88E-03 | 5.22E-03 | 2913 | -0.055 | 1.89E-03 | 2.83E-03 | 3224 |
| Lachnospiraceae sp. (HG3A.0018) | ODI | 0.069 | 1.27E-04 | 2.14E-03 | 3194 | 0.068 | 1.43E-04 | 2.03E-03 | 3249 | 0.054 | 3.91E-03 | 5.97E-03 | 2913 | 0.067 | 1.58E-04 | 9.85E-04 | 3224 |
| Lachnospiraceae sp. (HG3A.0018) | T90 | 0.053 | 3.09E-03 | 5.13E-03 | 3194 | 0.055 | 1.96E-03 | 4.01E-03 | 3249 | 0.048 | 1.14E-02 | 1.25E-02 | 2913 | 0.056 | 1.76E-03 | 2.77E-03 | 3224 |
| Lachnospiraceae sp. (HG3A.0855) | ODI | -0.045 | 1.20E-02 | 1.24E-02 | 3194 | -0.045 | 1.22E-02 | 1.24E-02 | 3249 | -0.044 | 2.03E-02 | 2.10E-02 | 2913 | -0.050 | 4.75E-03 | 5.04E-03 | 3224 |
| Mediterraneibacter glycyrrhizinilyticus (HG3A.0314) | ODI | 0.047 | 8.48E-03 | 9.25E-03 | 3194 | 0.046 | 9.48E-03 | 9.86E-03 | 3249 | 0.048 | 1.01E-02 | 1.13E-02 | 2913 | 0.052 | 3.36E-03 | 3.86E-03 | 3224 |
| Mediterraneibacter glycyrrhizinilyticus (HG3A.0314) | T90 | 0.068 | 1.37E-04 | 2.14E-03 | 3194 | 0.065 | 2.60E-04 | 2.14E-03 | 3249 | 0.074 | 7.64E-05 | 2.01E-03 | 2913 | 0.069 | 1.10E-04 | 9.48E-04 | 3224 |
| Oscillibacter sp. (HG3A.0734) | T90 | -0.052 | 3.93E-03 | 5.55E-03 | 3194 | -0.050 | 4.87E-03 | 6.33E-03 | 3249 | -0.055 | 3.71E-03 | 5.91E-03 | 2913 | -0.057 | 1.38E-03 | 2.59E-03 | 3224 |
| Oscillospiraceae sp. (HG3A.0072) | ODI | -0.062 | 5.09E-04 | 2.50E-03 | 3194 | -0.064 | 3.48E-04 | 2.14E-03 | 3249 | -0.070 | 1.77E-04 | 2.01E-03 | 2913 | -0.070 | 7.68E-05 | 9.48E-04 | 3224 |

| Oscillospiraceae sp. (HG3A.0072) | T90 | -0.051 | 4.25E-03 | 5.67E-03 | 3194 | -0.051 | 4.05E-03 | 5.57E-03 | 3249 | -0.061 | 1.11E-03 | 2.92E-03 | 2913 | -0.057 | 1.33E-03 | 2.59E-03 | 3224 |
| --- | --- | --- | --- | --- | --- | --- | --- | --- | --- | --- | --- | --- | --- | --- | --- | --- | --- |
| Oscillospiraceae sp. (HG3A.0207) | ODI | -0.069 | 1.32E-04 | 2.14E-03 | 3194 | -0.072 | 4.46E-05 | 2.03E-03 | 3249 | -0.070 | 2.14E-04 | 2.01E-03 | 2913 | -0.079 | 1.05E-05 | 4.72E-04 | 3224 |
| Oscillospiraceae sp. (HG3A.0207) | T90 | -0.064 | 3.96E-04 | 2.50E-03 | 3194 | -0.063 | 3.57E-04 | 2.14E-03 | 3249 | -0.066 | 4.45E-04 | 2.09E-03 | 2913 | -0.068 | 1.26E-04 | 9.48E-04 | 3224 |
| Oscillospiraceae sp. (HG3A.0223) | ODI | -0.053 | 3.07E-03 | 5.13E-03 | 3194 | -0.056 | 1.78E-03 | 3.81E-03 | 3249 | -0.058 | 1.92E-03 | 4.22E-03 | 2913 | -0.057 | 1.30E-03 | 2.59E-03 | 3224 |
| Oscillospiraceae sp. (HG3A.0437) | ODI | -0.049 | 5.99E-03 | 6.97E-03 | 3194 | -0.053 | 2.93E-03 | 4.70E-03 | 3249 | -0.051 | 6.67E-03 | 8.25E-03 | 2913 | -0.058 | 1.24E-03 | 2.59E-03 | 3224 |
| Oscillospiraceae sp. (HG3A.0445) | ODI | -0.051 | 4.20E-03 | 5.67E-03 | 3194 | -0.051 | 4.19E-03 | 5.64E-03 | 3249 | -0.050 | 7.58E-03 | 8.90E-03 | 2913 | -0.056 | 1.73E-03 | 2.77E-03 | 3224 |
| Oscillospiraceae sp. (HG3A.0445) | T90 | -0.053 | 2.95E-03 | 5.01E-03 | 3194 | -0.051 | 4.33E-03 | 5.77E-03 | 3249 | -0.062 | 9.32E-04 | 2.69E-03 | 2913 | -0.056 | 1.70E-03 | 2.77E-03 | 3224 |
| Oscillospiraceae sp. (HG3A.1270) | ODI | -0.046 | 1.03E-02 | 1.08E-02 | 3194 | -0.049 | 5.97E-03 | 7.00E-03 | 3249 | -0.042 | 2.48E-02 | 2.52E-02 | 2913 | -0.053 | 3.22E-03 | 3.75E-03 | 3224 |
| Pediococcus acidilactici (HG3A.1468) | T90 | 0.057 | 1.41E-03 | 3.30E-03 | 3194 | 0.058 | 1.07E-03 | 2.98E-03 | 3249 | 0.062 | 9.58E-04 | 2.72E-03 | 2913 | 0.063 | 4.36E-04 | 1.52E-03 | 3224 |
| Roseburia inulinivorans  (HG3A.0036) | ODI | 0.056 | 1.73E-03 | 3.62E-03 | 3194 | 0.060 | 7.42E-04 | 2.74E-03 | 3249 | 0.069 | 2.36E-04 | 2.01E-03 | 2913 | 0.062 | 5.28E-04 | 1.55E-03 | 3224 |
| Roseburia sp. AM59-24XD (HG3A.0391) | ODI | -0.054 | 2.57E-03 | 4.65E-03 | 3194 | -0.055 | 2.05E-03 | 4.10E-03 | 3249 | -0.051 | 6.39E-03 | 8.04E-03 | 2913 | -0.056 | 1.59E-03 | 2.77E-03 | 3224 |
| Ruminococcus sp. AM42-11  (HG3A.0002) | T90 | 0.056 | 1.87E-03 | 3.83E-03 | 3194 | 0.049 | 5.60E-03 | 6.84E-03 | 3249 | 0.049 | 9.40E-03 | 1.05E-02 | 2913 | 0.057 | 1.28E-03 | 2.59E-03 | 3224 |
| Staphylococcus aureus (HG3A.1538) | T90 | 0.060 | 7.76E-04 | 2.92E-03 | 3194 | 0.059 | 8.47E-04 | 2.74E-03 | 3249 | 0.063 | 8.53E-04 | 2.56E-03 | 2913 | 0.061 | 6.08E-04 | 1.72E-03 | 3224 |
| Traorella massiliensis  (HG3A.0669) | T90 | -0.058 | 1.15E-03 | 3.23E-03 | 3194 | -0.057 | 1.44E-03 | 3.49E-03 | 3249 | -0.060 | 1.53E-03 | 3.56E-03 | 2913 | -0.057 | 1.49E-03 | 2.66E-03 | 3224 |
| Victivallis vadensis (HG3A.0689) | ODI | -0.056 | 1.74E-03 | 3.62E-03 | 3194 | -0.061 | 6.03E-04 | 2.54E-03 | 3249 | -0.064 | 7.07E-04 | 2.42E-03 | 2913 | -0.061 | 6.61E-04 | 1.72E-03 | 3224 |
| [Ruminococcus] gnavus  (HG3A.0239) | ODI | 0.055 | 2.19E-03 | 4.12E-03 | 3194 | 0.058 | 1.01E-03 | 2.96E-03 | 3249 | 0.065 | 5.68E-04 | 2.21E-03 | 2913 | 0.063 | 4.12E-04 | 1.52E-03 | 3224 |
| [Ruminococcus] gnavus (HG3A.0239) | T90 | 0.058 | 1.17E-03 | 3.23E-03 | 3194 | 0.057 | 1.45E-03 | 3.49E-03 | 3249 | 0.061 | 1.27E-03 | 3.06E-03 | 2913 | 0.059 | 9.42E-04 | 2.19E-03 | 3224 |
| [Ruminococcus] torques  (HG3A.0034) | ODI | 0.049 | 5.96E-03 | 6.97E-03 | 3194 | 0.056 | 1.78E-03 | 3.81E-03 | 3249 | 0.049 | 8.93E-03 | 1.02E-02 | 2913 | 0.056 | 1.70E-03 | 2.77E-03 | 3224 |
