# Supplementary material for: OSA Is Associated With the Human Gut Microbiota Composition and Functional Potential in the Population-Based Swedish CardioPulmonary bioImage Study
Source: Chest. 2023 Mar 15;164(2):503–16. doi: 10.1016/j.chest.2023.03.010 (PMC10410248; doi:10.1016/j.chest.2023.03.010)
Supplement: e-Table 10 [file mmc7.docx]

e-Table 10. Partial Spearman’s correlations of AHI with microbiota species using the extended model after imputing missing AHI values

Missing AHI values for participants with valid T90 and ODI were imputed using predicted mean matching. Only the species identified in the model not adjusted for BMI were included in this analysis. Associations adjusted for age, sex, smoking, alcohol intake, BMI, fiber intake, total energy intake, physical activity, education, birth country, season, and DNA extraction plate. Adjustment for multiple testing using the Benjamini-Hochberg method and presented as q-values. Under the column "Metagenomics species", the information between parenthesis is the internal identifier for the respective species. AHI: apnea-hypopnea index

| **Metagenomic species** | **exposure** | **Spearman's correlation** | **p-value** | **q-value** | **N** |
| --- | --- | --- | --- | --- | --- |
| Eubacteriales sp. (HG3A.0156) | AHI | -0.063 | 3.52E-04 | 0.062 | 3249 |
| Clostridia sp. (HG3A.0515) | AHI | -0.063 | 3.93E-04 | 0.062 | 3249 |
| Eubacteriales sp. (HG3A.0572) | AHI | -0.067 | 2.00E-04 | 0.062 | 3249 |
| Clostridia sp. (HG3A.0728) | AHI | -0.064 | 2.56E-04 | 0.062 | 3249 |
| Eubacteriales sp. (HG3A.0829) | AHI | -0.066 | 1.99E-04 | 0.062 | 3249 |
| Eubacteriales sp. (HG3A.0069) | AHI | -0.063 | 4.77E-04 | 0.063 | 3249 |
| Oscillospiraceae sp. (HG3A.0207) | AHI | -0.063 | 6.29E-04 | 0.071 | 3249 |
| Eubacteriales sp. (HG3A.0085) | AHI | -0.06 | 7.66E-04 | 0.075 | 3249 |
| Fusicatenibacter saccharivorans (HG3A.0004) | AHI | 0.055 | 0.002 | 0.083 | 3249 |
| Lachnospiraceae sp. (HG3A.0018) | AHI | 0.054 | 0.002 | 0.083 | 3249 |
| Coprobacillus sp. (HG3A.0022) | AHI | 0.055 | 0.002 | 0.083 | 3249 |
| Clostridium sp. TF06-15AC (HG3A.0032) | AHI | 0.053 | 0.003 | 0.083 | 3249 |
| Roseburia inulinivorans (HG3A.0036) | AHI | 0.051 | 0.004 | 0.083 | 3249 |
| Alistipes shahii (HG3A.0054) | AHI | -0.056 | 0.001 | 0.083 | 3249 |
| Alistipes communis (HG3A.0064) | AHI | -0.051 | 0.004 | 0.083 | 3249 |
| Oscillospiraceae sp. (HG3A.0072) | AHI | -0.052 | 0.003 | 0.083 | 3249 |
| Eubacteriales sp. (HG3A.0084) | AHI | -0.053 | 0.003 | 0.083 | 3249 |
| Eubacteriales sp. (HG3A.0162) | AHI | -0.053 | 0.004 | 0.083 | 3249 |
| Eggerthellales sp. (HG3A.0177) | AHI | -0.057 | 0.001 | 0.083 | 3249 |
| Eubacteriales sp. (HG3A.0197) | AHI | -0.051 | 0.004 | 0.083 | 3249 |
| Eubacterium sp. (HG3A.0214) | AHI | 0.053 | 0.003 | 0.083 | 3249 |
| Eubacteriales sp. (HG3A.0226) | AHI | -0.052 | 0.004 | 0.083 | 3249 |
| Eubacteriales sp. (HG3A.0230) | AHI | -0.053 | 0.003 | 0.083 | 3249 |
| Eubacteriales sp. (HG3A.0269) | AHI | -0.052 | 0.003 | 0.083 | 3249 |
| Eubacteriales sp. (HG3A.0284) | AHI | -0.053 | 0.003 | 0.083 | 3249 |

| Eubacteriales sp. (HG3A.0309) | AHI | -0.052 | 0.003 | 0.083 | 3249 |
| --- | --- | --- | --- | --- | --- |
| Eubacteriales sp. (HG3A.0311) | AHI | -0.051 | 0.004 | 0.083 | 3249 |
| Firmicutes sp. (HG3A.0341) | AHI | -0.051 | 0.004 | 0.083 | 3249 |
| Firmicutes sp. (HG3A.0397) | AHI | -0.058 | 0.001 | 0.083 | 3249 |
| Firmicutes sp. (HG3A.0398) | AHI | -0.055 | 0.002 | 0.083 | 3249 |
| Eubacteriales sp. (HG3A.0421) | AHI | -0.051 | 0.004 | 0.083 | 3249 |
| Eubacteriales sp. (HG3A.0442) | AHI | -0.052 | 0.004 | 0.083 | 3249 |
| Bacteria sp. (HG3A.0634) | AHI | -0.055 | 0.002 | 0.083 | 3249 |
| Eubacteriales sp. (HG3A.0635) | AHI | -0.053 | 0.003 | 0.083 | 3249 |
| Clostridia sp. (HG3A.0660) | AHI | -0.053 | 0.003 | 0.083 | 3249 |
| Clostridia sp. (HG3A.0682) | AHI | -0.053 | 0.003 | 0.083 | 3249 |
| Clostridia sp. (HG3A.1053) | AHI | -0.054 | 0.002 | 0.083 | 3249 |
| Oscillospiraceae sp. (HG3A.1270) | AHI | -0.051 | 0.004 | 0.083 | 3249 |
| Eubacteriales sp. (HG3A.0193) | AHI | -0.05 | 0.005 | 0.091 | 3249 |
| Oscillospiraceae sp. (HG3A.0437) | AHI | -0.049 | 0.005 | 0.099 | 3249 |
| Clostridia sp. (HG3A.0094) | AHI | -0.05 | 0.006 | 0.106 | 3249 |
| Mediterraneibacter glycyrrhizinilyticus (HG3A.0314) | AHI | 0.049 | 0.006 | 0.106 | 3249 |
| Holdemanella sp. (HG3A.0366) | AHI | 0.049 | 0.006 | 0.106 | 3249 |
| Eubacteriales sp. (HG3A.0718) | AHI | -0.05 | 0.006 | 0.106 | 3249 |
| Coprococcus comes (HG3A.0016) | AHI | 0.049 | 0.007 | 0.113 | 3249 |
| [Ruminococcus] torques (HG3A.0034) | AHI | 0.049 | 0.007 | 0.113 | 3249 |
| Amedibacillus dolichus (HG3A.0798) | AHI | 0.048 | 0.007 | 0.113 | 3249 |
| Eubacteriales sp. (HG3A.0506) | AHI | -0.048 | 0.007 | 0.114 | 3249 |
| Eubacteriales sp. (HG3A.0964) | AHI | -0.048 | 0.007 | 0.114 | 3249 |
| Eubacteriales sp. (HG3A.0100) | AHI | -0.047 | 0.008 | 0.121 | 3249 |
| Eubacteriales sp. (HG3A.0211) | AHI | -0.047 | 0.008 | 0.121 | 3249 |
| Eubacteriales sp. (HG3A.0235) | AHI | -0.047 | 0.009 | 0.121 | 3249 |
| Parolsenella catena (HG3A.0499) | AHI | 0.048 | 0.008 | 0.121 | 3249 |
| Sutterella seckii (HG3A.0561) | AHI | 0.047 | 0.009 | 0.121 | 3249 |
| Clostridia sp. (HG3A.0599) | AHI | -0.047 | 0.009 | 0.121 | 3249 |
| Eubacteriales sp. (HG3A.0864) | AHI | -0.047 | 0.008 | 0.121 | 3249 |
| Clostridia sp. (HG3A.0140) | AHI | -0.046 | 0.01 | 0.127 | 3249 |
| Eubacteriales sp. (HG3A.0153) | AHI | -0.045 | 0.01 | 0.127 | 3249 |
| Intestinibacillus sp. Marseille-P4005 (HG3A.0168) | AHI | 0.046 | 0.01 | 0.127 | 3249 |

| Bacteria sp. (HG3A.0483) | AHI | -0.047 | 0.01 | 0.127 | 3249 |
| --- | --- | --- | --- | --- | --- |
| Mesosutterella multiformis (HG3A.0520) | AHI | -0.046 | 0.01 | 0.127 | 3249 |
| Firmicutes sp. (HG3A.0681) | AHI | -0.046 | 0.01 | 0.127 | 3249 |
| Eubacteriales sp. (HG3A.0698) | AHI | 0.046 | 0.009 | 0.127 | 3249 |
| Clostridia sp. (HG3A.1504) | AHI | -0.046 | 0.009 | 0.127 | 3249 |
| Clostridia sp. (HG3A.0750) | AHI | -0.045 | 0.011 | 0.131 | 3249 |
| Collinsella aerofaciens (HG3A.0019) | AHI | 0.045 | 0.011 | 0.132 | 3249 |
| Eubacteriales sp. (HG3A.0081) | AHI | -0.046 | 0.011 | 0.132 | 3249 |
| Clostridium sp. SN20 (HG3A.0603) | AHI | 0.045 | 0.011 | 0.132 | 3249 |
| Oscillibacter sp. (HG3A.0245) | AHI | -0.045 | 0.012 | 0.133 | 3249 |
| Intestinimonas massiliensis (HG3A.0198) | AHI | -0.045 | 0.012 | 0.135 | 3249 |
| Blautia massiliensis (HG3A.0023) | AHI | 0.044 | 0.013 | 0.144 | 3249 |
| Eubacteriales sp. (HG3A.0118) | AHI | -0.044 | 0.014 | 0.144 | 3249 |
| Eubacteriales sp. (HG3A.0149) | AHI | -0.044 | 0.014 | 0.144 | 3249 |
| [Ruminococcus] gnavus (HG3A.0239) | AHI | 0.044 | 0.014 | 0.144 | 3249 |
| Eubacteriales sp. (HG3A.0282) | AHI | -0.045 | 0.014 | 0.144 | 3249 |
| Eubacteriales sp. (HG3A.0573) | AHI | -0.043 | 0.015 | 0.152 | 3249 |
| Eubacteriales sp. (HG3A.0691) | AHI | -0.044 | 0.015 | 0.152 | 3249 |
| Eubacteriales sp. (HG3A.1019) | AHI | -0.043 | 0.015 | 0.152 | 3249 |
| Eubacteriales sp. (HG3A.1103) | AHI | -0.043 | 0.015 | 0.152 | 3249 |
| Oscillospiraceae sp. (HG3A.0223) | AHI | -0.043 | 0.017 | 0.168 | 3249 |
| Eggerthellaceae sp. (HG3A.0171) | AHI | -0.042 | 0.018 | 0.169 | 3249 |
| Eubacteriales sp. (HG3A.0249) | AHI | -0.043 | 0.018 | 0.169 | 3249 |
| Eubacteriales sp. (HG3A.0250) | AHI | -0.042 | 0.018 | 0.169 | 3249 |
| Eubacteriales sp. (HG3A.0381) | AHI | -0.042 | 0.018 | 0.169 | 3249 |
| Firmicutes sp. (HG3A.0501) | AHI | -0.042 | 0.018 | 0.169 | 3249 |
| Oxalobacter formigenes (HG3A.0552) | AHI | -0.042 | 0.018 | 0.169 | 3249 |
| Blautia obeum (HG3A.0001) | AHI | 0.041 | 0.02 | 0.178 | 3249 |
| Blautia sp. SG-772 (HG3A.0063) | AHI | 0.041 | 0.02 | 0.178 | 3249 |
| Eubacteriales sp. (HG3A.0617) | AHI | -0.041 | 0.02 | 0.178 | 3249 |
| Eubacteriales sp. (HG3A.1377) | AHI | -0.041 | 0.021 | 0.178 | 3249 |
| Alistipes sp. An66 (HG3A.1535) | AHI | -0.041 | 0.02 | 0.178 | 3249 |
| Clostridia sp. (HG3A.1148) | AHI | -0.041 | 0.021 | 0.179 | 3249 |
| Roseburia sp. AM59-24XD (HG3A.0391) | AHI | -0.041 | 0.022 | 0.185 | 3249 |

| Eubacteriales sp. (HG3A.0730) | AHI | -0.041 | 0.022 | 0.185 | 3249 |
| --- | --- | --- | --- | --- | --- |
| Anaerostipes sp. BG01 (HG3A.1509) | AHI | 0.04 | 0.022 | 0.185 | 3249 |
| Eubacteriales sp. (HG3A.0331) | AHI | -0.04 | 0.023 | 0.189 | 3249 |
| Eubacteriales sp. (HG3A.0242) | AHI | -0.042 | 0.024 | 0.191 | 3249 |
| Lactobacillus gasseri (HG3A.0884) | AHI | 0.04 | 0.025 | 0.201 | 3249 |
| Eubacteriales sp. (HG3A.1191) | AHI | -0.04 | 0.026 | 0.203 | 3249 |
| Eubacteriales sp. (HG3A.0125) | AHI | -0.039 | 0.027 | 0.209 | 3249 |
| Oscillospiraceae sp. (HG3A.0445) | AHI | -0.04 | 0.027 | 0.209 | 3249 |
| Clostridia sp. (HG3A.0272) | AHI | -0.039 | 0.028 | 0.212 | 3249 |
| Lachnospiraceae sp. (HG3A.1190) | AHI | -0.04 | 0.028 | 0.212 | 3249 |
| Eubacteriales sp. (HG3A.0643) | AHI | -0.039 | 0.028 | 0.213 | 3249 |
| Lachnospiraceae sp. (HG3A.0855) | AHI | -0.039 | 0.028 | 0.213 | 3249 |
| Eubacteriales sp. (HG3A.0703) | AHI | -0.039 | 0.029 | 0.215 | 3249 |
| Clostridia sp. (HG3A.0756) | AHI | -0.039 | 0.029 | 0.215 | 3249 |
| Eubacteriales sp. (HG3A.1026) | AHI | -0.039 | 0.029 | 0.215 | 3249 |
| Flavonifractor plautii (HG3A.0079) | AHI | 0.037 | 0.034 | 0.218 | 3249 |
| Eubacteriales sp. (HG3A.0102) | AHI | -0.038 | 0.033 | 0.218 | 3249 |
| Lachnospiraceae sp. (HG3A.0180) | AHI | -0.038 | 0.034 | 0.218 | 3249 |
| Eubacteriales sp. (HG3A.0196) | AHI | -0.039 | 0.03 | 0.218 | 3249 |
| Eubacteriales sp. (HG3A.0329) | AHI | -0.038 | 0.034 | 0.218 | 3249 |
| Eubacteriales sp. (HG3A.0377) | AHI | -0.038 | 0.035 | 0.218 | 3249 |
| Oscillospiraceae sp. (HG3A.0380) | AHI | -0.038 | 0.032 | 0.218 | 3249 |
| Oscillospiraceae sp. (HG3A.0429) | AHI | -0.039 | 0.032 | 0.218 | 3249 |
| Clostridia sp. (HG3A.0470) | AHI | -0.038 | 0.034 | 0.218 | 3249 |
| Firmicutes sp. (HG3A.0596) | AHI | -0.038 | 0.032 | 0.218 | 3249 |
| Eubacteriales sp. (HG3A.0666) | AHI | -0.038 | 0.031 | 0.218 | 3249 |
| Desulfovibrionales sp. (HG3A.0727) | AHI | 0.038 | 0.034 | 0.218 | 3249 |
| Oscillibacter sp. (HG3A.0734) | AHI | -0.038 | 0.032 | 0.218 | 3249 |
| Clostridia sp. (HG3A.0752) | AHI | -0.037 | 0.034 | 0.218 | 3249 |
| Clostridia sp. (HG3A.1020) | AHI | -0.038 | 0.032 | 0.218 | 3249 |
| Clostridia sp. (HG3A.1403) | AHI | -0.039 | 0.033 | 0.218 | 3249 |
| Eubacteriales sp. (HG3A.1445) | AHI | -0.038 | 0.032 | 0.218 | 3249 |
| Eubacteriales sp. (HG3A.0215) | AHI | -0.038 | 0.036 | 0.219 | 3249 |
| Eubacteriales sp. (HG3A.0540) | AHI | -0.038 | 0.035 | 0.219 | 3249 |

| Clostridia sp. (HG3A.0746) | AHI | -0.038 | 0.036 | 0.219 | 3249 |
| --- | --- | --- | --- | --- | --- |
| Dorea formicigenerans (HG3A.0006) | AHI | 0.037 | 0.036 | 0.22 | 3249 |
| Eubacteriales sp. (HG3A.0179) | AHI | -0.036 | 0.038 | 0.221 | 3249 |
| Lachnospiraceae sp. (HG3A.0233) | AHI | -0.037 | 0.038 | 0.221 | 3249 |
| Eubacteriales sp. (HG3A.0321) | AHI | -0.037 | 0.038 | 0.221 | 3249 |
| Clostridia sp. (HG3A.0435) | AHI | -0.037 | 0.038 | 0.221 | 3249 |
| Clostridia sp. (HG3A.0741) | AHI | -0.037 | 0.038 | 0.221 | 3249 |
| Rikenellaceae sp. (HG3A.1022) | AHI | -0.037 | 0.038 | 0.221 | 3249 |
| Bacteria sp. (HG3A.1543) | AHI | -0.037 | 0.037 | 0.221 | 3249 |
| Eubacteriales sp. (HG3A.0854) | AHI | 0.037 | 0.039 | 0.223 | 3249 |
| Alistipes senegalensis (HG3A.0141) | AHI | -0.036 | 0.04 | 0.225 | 3249 |
| Eubacteriales sp. (HG3A.0229) | AHI | -0.036 | 0.04 | 0.225 | 3249 |
| Eubacterium sp. AF22-8LB (HG3A.0838) | AHI | 0.036 | 0.04 | 0.225 | 3249 |
| Clostridium sp. AT4 (HG3A.0347) | AHI | 0.037 | 0.041 | 0.226 | 3249 |
| Limosilactobacillus fermentum (HG3A.0990) | AHI | 0.036 | 0.041 | 0.229 | 3249 |
| Eubacteriales sp. (HG3A.0624) | AHI | -0.036 | 0.043 | 0.23 | 3249 |
| Olsenella sp. AF21-51 (HG3A.0690) | AHI | -0.036 | 0.043 | 0.23 | 3249 |
| Eubacteriales sp. (HG3A.0731) | AHI | -0.037 | 0.042 | 0.23 | 3249 |
| Clostridia sp. (HG3A.0931) | AHI | -0.036 | 0.043 | 0.23 | 3249 |
| Firmicutes sp. (HG3A.1085) | AHI | -0.036 | 0.043 | 0.23 | 3249 |
| Eubacteriales sp. (HG3A.0316) | AHI | -0.035 | 0.045 | 0.232 | 3249 |
| Eubacteriales sp. (HG3A.0419) | AHI | -0.036 | 0.045 | 0.232 | 3249 |
| Clostridiaceae sp. (HG3A.0431) | AHI | 0.036 | 0.044 | 0.232 | 3249 |
| Clostridia sp. (HG3A.0645) | AHI | -0.035 | 0.045 | 0.232 | 3249 |
| Lactobacillus acidophilus (HG3A.0672) | AHI | -0.035 | 0.046 | 0.232 | 3249 |
| Eubacteriales sp. (HG3A.0696) | AHI | -0.036 | 0.044 | 0.232 | 3249 |
| Eubacteriales sp. (HG3A.0771) | AHI | -0.036 | 0.046 | 0.232 | 3249 |
| Firmicutes sp. (HG3A.1054) | AHI | -0.035 | 0.045 | 0.232 | 3249 |
| Eubacteriales sp. (HG3A.1379) | AHI | -0.036 | 0.044 | 0.232 | 3249 |
| Eubacteriales sp. (HG3A.0280) | AHI | -0.035 | 0.047 | 0.234 | 3249 |
| Eubacteriales sp. (HG3A.0288) | AHI | -0.035 | 0.048 | 0.234 | 3249 |
| Eubacteriales sp. (HG3A.0291) | AHI | -0.035 | 0.047 | 0.234 | 3249 |
| Bacteria sp. (HG3A.0459) | AHI | -0.035 | 0.048 | 0.234 | 3249 |
| Clostridia sp. (HG3A.1141) | AHI | -0.036 | 0.048 | 0.234 | 3249 |

| Eubacteriales sp. (HG3A.0386) | AHI | -0.035 | 0.048 | 0.236 | 3249 |
| --- | --- | --- | --- | --- | --- |
| Eubacteriales sp. (HG3A.0270) | AHI | -0.034 | 0.05 | 0.24 | 3249 |
| Veillonella rogosae (HG3A.0324) | AHI | -0.035 | 0.05 | 0.24 | 3249 |
| Blautia obeum (HG3A.0009) | AHI | 0.035 | 0.054 | 0.243 | 3249 |
| Butyricicoccus sp. OM04-18BH (HG3A.0139) | AHI | -0.035 | 0.053 | 0.243 | 3249 |
| Lachnospiraceae sp. (HG3A.0236) | AHI | -0.035 | 0.053 | 0.243 | 3249 |
| Firmicutes sp. (HG3A.0587) | AHI | -0.034 | 0.052 | 0.243 | 3249 |
| Clostridia sp. (HG3A.0724) | AHI | -0.034 | 0.054 | 0.243 | 3249 |
| Eubacteriales sp. (HG3A.0757) | AHI | -0.034 | 0.053 | 0.243 | 3249 |
| Prevotella sp. (HG3A.1040) | AHI | 0.034 | 0.053 | 0.243 | 3249 |
| Oscillospiraceae sp. (HG3A.1173) | AHI | -0.035 | 0.051 | 0.243 | 3249 |
| Clostridia sp. (HG3A.1205) | AHI | -0.034 | 0.052 | 0.243 | 3249 |
| Candidatus Borkfalkiales sp. (HG3A.1284) | AHI | -0.034 | 0.054 | 0.243 | 3249 |
| Eubacteriales sp. (HG3A.0151) | AHI | -0.034 | 0.057 | 0.25 | 3249 |
| Eubacteriales sp. (HG3A.0486) | AHI | -0.034 | 0.056 | 0.25 | 3249 |
| Bacteria sp. (HG3A.0492) | AHI | -0.034 | 0.056 | 0.25 | 3249 |
| Coprococcus sp. AF21-14LB (HG3A.1047) | AHI | 0.034 | 0.056 | 0.25 | 3249 |
| Eubacteriales sp. (HG3A.0083) | AHI | -0.034 | 0.058 | 0.251 | 3249 |
| Eubacteriales sp. (HG3A.0342) | AHI | -0.034 | 0.057 | 0.251 | 3249 |
| Eubacteriales sp. (HG3A.0887) | AHI | -0.034 | 0.058 | 0.251 | 3249 |
| Parvimonas micra (HG3A.1231) | AHI | 0.033 | 0.058 | 0.251 | 3249 |
| Eubacteriales sp. (HG3A.0120) | AHI | -0.034 | 0.059 | 0.252 | 3249 |
| Eubacteriales sp. (HG3A.0128) | AHI | 0.033 | 0.06 | 0.252 | 3249 |
| Eubacterium sp. AM49-13BH (HG3A.0251) | AHI | -0.033 | 0.061 | 0.252 | 3249 |
| Eubacteriales sp. (HG3A.0371) | AHI | -0.034 | 0.059 | 0.252 | 3249 |
| Coprococcus sp. (HG3A.0404) | AHI | 0.033 | 0.061 | 0.252 | 3249 |
| Firmicutes sp. (HG3A.0436) | AHI | -0.034 | 0.06 | 0.252 | 3249 |
| Clostridiaceae sp. (HG3A.0608) | AHI | -0.034 | 0.059 | 0.252 | 3249 |
| Clostridia sp. (HG3A.0879) | AHI | -0.034 | 0.059 | 0.252 | 3249 |
| Victivallis vadensis (HG3A.0689) | AHI | -0.033 | 0.062 | 0.255 | 3249 |
| Gemmiger formicilis (HG3A.0027) | AHI | 0.034 | 0.063 | 0.257 | 3249 |
| Eubacteriales sp. (HG3A.0264) | AHI | -0.033 | 0.064 | 0.257 | 3249 |
| Longicatena caecimuris (HG3A.0571) | AHI | 0.033 | 0.064 | 0.257 | 3249 |
| Eubacteriales sp. (HG3A.0578) | AHI | -0.033 | 0.063 | 0.257 | 3249 |

| Eubacteriales sp. (HG3A.1129) | AHI | -0.033 | 0.063 | 0.257 | 3249 |
| --- | --- | --- | --- | --- | --- |
| Eubacteriales sp. (HG3A.0546) | AHI | -0.033 | 0.066 | 0.263 | 3249 |
| Clostridia sp. (HG3A.1076) | AHI | -0.032 | 0.066 | 0.263 | 3249 |
| Clostridia sp. (HG3A.0661) | AHI | -0.033 | 0.068 | 0.267 | 3249 |
| Lachnospiraceae sp. (HG3A.0899) | AHI | -0.033 | 0.067 | 0.267 | 3249 |
| Eubacteriales sp. (HG3A.1239) | AHI | -0.034 | 0.068 | 0.268 | 3249 |
| Eubacteriales sp. (HG3A.0426) | AHI | -0.032 | 0.069 | 0.271 | 3249 |
| Eubacteriales sp. (HG3A.0132) | AHI | -0.032 | 0.072 | 0.273 | 3249 |
| Eubacteriales sp. (HG3A.0154) | AHI | -0.032 | 0.074 | 0.273 | 3249 |
| Eubacteriales sp. (HG3A.0161) | AHI | -0.032 | 0.074 | 0.273 | 3249 |
| Erysipelotrichales sp. (HG3A.0303) | AHI | -0.032 | 0.071 | 0.273 | 3249 |
| Eubacteriales sp. (HG3A.0325) | AHI | -0.033 | 0.073 | 0.273 | 3249 |
| Eubacteriales sp. (HG3A.0349) | AHI | -0.032 | 0.074 | 0.273 | 3249 |
| Oscillospiraceae sp. (HG3A.0384) | AHI | -0.032 | 0.073 | 0.273 | 3249 |
| Eubacteriales sp. (HG3A.0418) | AHI | -0.032 | 0.071 | 0.273 | 3249 |
| Clostridia sp. (HG3A.0479) | AHI | -0.032 | 0.073 | 0.273 | 3249 |
| Eubacteriales sp. (HG3A.0545) | AHI | -0.033 | 0.073 | 0.273 | 3249 |
| Eubacteriales sp. (HG3A.0653) | AHI | -0.032 | 0.07 | 0.273 | 3249 |
| Bacteroidales sp. (HG3A.0894) | AHI | -0.032 | 0.074 | 0.273 | 3249 |
| Eubacteriales sp. (HG3A.0334) | AHI | -0.031 | 0.078 | 0.28 | 3249 |
| Acidaminococcus intestini (HG3A.0407) | AHI | 0.032 | 0.078 | 0.28 | 3249 |
| Eubacteriales sp. (HG3A.0626) | AHI | -0.031 | 0.077 | 0.28 | 3249 |
| Anaerostipes caccae (HG3A.0747) | AHI | 0.032 | 0.076 | 0.28 | 3249 |
| Firmicutes sp. (HG3A.1195) | AHI | -0.031 | 0.078 | 0.28 | 3249 |
| Eubacteriales sp. (HG3A.1321) | AHI | -0.031 | 0.077 | 0.28 | 3249 |
| Oscillospiraceae sp. (HG3A.0060) | AHI | -0.031 | 0.079 | 0.282 | 3249 |
| Eubacteriales sp. (HG3A.0935) | AHI | -0.031 | 0.08 | 0.282 | 3249 |
| Eubacteriales sp. (HG3A.0092) | AHI | -0.031 | 0.081 | 0.284 | 3249 |
| Eubacteriales sp. (HG3A.0604) | AHI | -0.031 | 0.081 | 0.284 | 3249 |
| Eubacteriales sp. (HG3A.0637) | AHI | -0.031 | 0.081 | 0.284 | 3249 |
| Anaerobutyricum hallii (HG3A.0112) | AHI | 0.032 | 0.084 | 0.286 | 3249 |
| Alistipes sp. AF17-16 (HG3A.0150) | AHI | -0.03 | 0.084 | 0.286 | 3249 |
| Lachnospiraceae sp. (HG3A.0257) | AHI | -0.031 | 0.084 | 0.286 | 3249 |
| Oscillospiraceae sp. (HG3A.0388) | AHI | -0.031 | 0.084 | 0.286 | 3249 |

| Eubacteriales sp. (HG3A.0870) | AHI | -0.031 | 0.083 | 0.286 | 3249 |
| --- | --- | --- | --- | --- | --- |
| Alistipes provencensis (HG3A.0877) | AHI | -0.031 | 0.084 | 0.286 | 3249 |
| Oscillospiraceae sp. (HG3A.0966) | AHI | -0.031 | 0.083 | 0.286 | 3249 |
| Candidatus Borkfalkiales sp. (HG3A.1397) | AHI | -0.031 | 0.083 | 0.286 | 3249 |
| Clostridia sp. (HG3A.0508) | AHI | -0.032 | 0.086 | 0.288 | 3249 |
| Eubacteriales sp. (HG3A.0234) | AHI | -0.031 | 0.087 | 0.291 | 3249 |
| Eubacteriales sp. (HG3A.0509) | AHI | -0.031 | 0.089 | 0.291 | 3249 |
| Eubacteriales sp. (HG3A.0531) | AHI | -0.031 | 0.088 | 0.291 | 3249 |
| Eubacteriales sp. (HG3A.0807) | AHI | -0.03 | 0.088 | 0.291 | 3249 |
| Sutterellaceae sp. (HG3A.1122) | AHI | 0.03 | 0.089 | 0.291 | 3249 |
| Clostridia sp. (HG3A.1375) | AHI | -0.031 | 0.088 | 0.291 | 3249 |
| Gemella morbillorum (HG3A.1782) | AHI | 0.031 | 0.088 | 0.291 | 3249 |
| Mogibacterium kristiansenii (HG3A.0522) | AHI | 0.03 | 0.09 | 0.293 | 3249 |
| Eubacteriales sp. (HG3A.1227) | AHI | -0.03 | 0.09 | 0.293 | 3249 |
| Coprococcus eutactus (HG3A.0155) | AHI | -0.031 | 0.093 | 0.299 | 3249 |
| Lachnoclostridium sp. (HG3A.0655) | AHI | 0.03 | 0.093 | 0.299 | 3249 |
| Oscillospiraceae sp. (HG3A.0256) | AHI | 0.03 | 0.094 | 0.3 | 3249 |
| Clostridia sp. (HG3A.0885) | AHI | -0.03 | 0.095 | 0.302 | 3249 |
| [Ruminococcus] torques (HG3A.0088) | AHI | 0.03 | 0.096 | 0.303 | 3249 |
| Alistipes ihumii (HG3A.0106) | AHI | -0.029 | 0.096 | 0.303 | 3249 |
| Clostridia sp. (HG3A.1008) | AHI | -0.03 | 0.096 | 0.303 | 3249 |
| Clostridia sp. (HG3A.1217) | AHI | -0.03 | 0.095 | 0.303 | 3249 |
| Roseburia intestinalis (HG3A.0078) | AHI | 0.03 | 0.098 | 0.305 | 3249 |
| Eggerthella lenta (HG3A.0225) | AHI | 0.029 | 0.099 | 0.305 | 3249 |
| Eubacteriales sp. (HG3A.0254) | AHI | 0.029 | 0.099 | 0.305 | 3249 |
| Clostridia sp. (HG3A.0463) | AHI | -0.029 | 0.099 | 0.305 | 3249 |
| Eubacteriales sp. (HG3A.1294) | AHI | -0.029 | 0.099 | 0.306 | 3249 |
| Eubacteriales sp. (HG3A.0390) | AHI | -0.029 | 0.101 | 0.307 | 3249 |
| Eubacteriales sp. (HG3A.0548) | AHI | -0.029 | 0.1 | 0.307 | 3249 |
| Eubacteriales sp. (HG3A.0908) | AHI | -0.029 | 0.101 | 0.308 | 3249 |
| Eubacteriales sp. (HG3A.0758) | AHI | -0.029 | 0.103 | 0.311 | 3249 |
| Eubacteriales sp. (HG3A.0794) | AHI | -0.029 | 0.103 | 0.312 | 3249 |
| Eubacteriales sp. (HG3A.0832) | AHI | -0.029 | 0.107 | 0.321 | 3249 |
| Ruminococcus champanellensis (HG3A.0716) | AHI | -0.028 | 0.108 | 0.322 | 3249 |

| Eubacteriales sp. (HG3A.0482) | AHI | -0.029 | 0.108 | 0.324 | 3249 |
| --- | --- | --- | --- | --- | --- |
| Phascolarctobacterium succinatutens (HG3A.0315) | AHI | 0.029 | 0.111 | 0.329 | 3249 |
| Eubacteriales sp. (HG3A.0759) | AHI | -0.028 | 0.112 | 0.331 | 3249 |
| Eubacteriales sp. (HG3A.0178) | AHI | -0.028 | 0.114 | 0.336 | 3249 |
| Eubacteriales sp. (HG3A.0123) | AHI | 0.028 | 0.115 | 0.338 | 3249 |
| [Clostridium] symbiosum (HG3A.0370) | AHI | 0.028 | 0.116 | 0.338 | 3249 |
| Eubacteriales sp. (HG3A.0439) | AHI | -0.029 | 0.115 | 0.338 | 3249 |
| Eubacteriales sp. (HG3A.0976) | AHI | -0.028 | 0.116 | 0.338 | 3249 |
| Eubacteriales sp. (HG3A.0450) | AHI | -0.028 | 0.117 | 0.339 | 3249 |
| Clostridium sp. TM06-18 (HG3A.0048) | AHI | 0.028 | 0.118 | 0.34 | 3249 |
| Eubacteriales sp. (HG3A.0409) | AHI | -0.028 | 0.118 | 0.34 | 3249 |
| Eubacteriales sp. (HG3A.0260) | AHI | -0.028 | 0.121 | 0.342 | 3249 |
| Eubacteriales sp. (HG3A.0312) | AHI | -0.028 | 0.12 | 0.342 | 3249 |
| Blastocystis sp. subtype 4 (HG3A.0446) | AHI | -0.028 | 0.12 | 0.342 | 3249 |
| Lachnospiraceae sp. (HG3A.0748) | AHI | -0.028 | 0.12 | 0.342 | 3249 |
| Catenibacterium mitsuokai (HG3A.0775) | AHI | 0.027 | 0.121 | 0.342 | 3249 |
| Eubacteriales sp. (HG3A.1086) | AHI | -0.027 | 0.122 | 0.342 | 3249 |
| Clostridia sp. (HG3A.1452) | AHI | -0.028 | 0.124 | 0.347 | 3249 |
| Oscillibacter sp. (HG3A.0046) | AHI | -0.027 | 0.129 | 0.35 | 3249 |
| Lachnotalea sp. AF33-28 (HG3A.0403) | AHI | -0.027 | 0.127 | 0.35 | 3249 |
| Eubacteriales sp. (HG3A.0443) | AHI | -0.027 | 0.128 | 0.35 | 3249 |
| Massilistercora timonensis (HG3A.0458) | AHI | -0.027 | 0.129 | 0.35 | 3249 |
| Firmicutes sp. (HG3A.0641) | AHI | -0.027 | 0.126 | 0.35 | 3249 |
| Clostridia sp. (HG3A.0733) | AHI | -0.027 | 0.129 | 0.35 | 3249 |
| Eubacteriales sp. (HG3A.0856) | AHI | -0.027 | 0.127 | 0.35 | 3249 |
| Clostridia sp. (HG3A.1010) | AHI | -0.027 | 0.127 | 0.35 | 3249 |
| Eubacteriales sp. (HG3A.1126) | AHI | 0.027 | 0.128 | 0.35 | 3249 |
| Dorea sp. AF36-15AT (HG3A.0052) | AHI | 0.027 | 0.13 | 0.351 | 3249 |
| Clostridia sp. (HG3A.0815) | AHI | -0.027 | 0.13 | 0.351 | 3249 |
| Eubacteriales sp. (HG3A.1243) | AHI | -0.027 | 0.131 | 0.352 | 3249 |
| Subdoligranulum sp. APC924/74 (HG3A.0015) | AHI | -0.027 | 0.132 | 0.354 | 3249 |
| Clostridium sp. (HG3A.0050) | AHI | 0.026 | 0.137 | 0.355 | 3249 |
| Eubacterium ramulus (HG3A.0068) | AHI | 0.026 | 0.137 | 0.355 | 3249 |
| Anaeromassilibacillus sp. An250 (HG3A.0169) | AHI | -0.027 | 0.135 | 0.355 | 3249 |

| Eubacteriales sp. (HG3A.0273) | AHI | -0.027 | 0.135 | 0.355 | 3249 |
| --- | --- | --- | --- | --- | --- |
| Firmicutes sp. (HG3A.0541) | AHI | -0.026 | 0.136 | 0.355 | 3249 |
| Eubacteriales sp. (HG3A.0565) | AHI | -0.027 | 0.134 | 0.355 | 3249 |
| Oscillospiraceae sp. (HG3A.0616) | AHI | -0.027 | 0.136 | 0.355 | 3249 |
| Eubacteriales sp. (HG3A.0956) | AHI | -0.026 | 0.137 | 0.355 | 3249 |
| Eubacteriales sp. (HG3A.1250) | AHI | -0.027 | 0.135 | 0.355 | 3249 |
| Blautia argi (HG3A.1450) | AHI | 0.027 | 0.136 | 0.355 | 3249 |
| Parasutterella excrementihominis (HG3A.0159) | AHI | -0.026 | 0.14 | 0.356 | 3249 |
| Eubacteriales sp. (HG3A.0654) | AHI | -0.026 | 0.14 | 0.356 | 3249 |
| Firmicutes sp. (HG3A.1075) | AHI | -0.027 | 0.138 | 0.356 | 3249 |
| Eubacteriales sp. (HG3A.1078) | AHI | -0.027 | 0.139 | 0.356 | 3249 |
| Erysipelotrichales sp. (HG3A.1207) | AHI | 0.027 | 0.139 | 0.356 | 3249 |
| Bacteria sp. (HG3A.0839) | AHI | -0.026 | 0.141 | 0.359 | 3249 |
| Odoribacter splanchnicus (HG3A.0041) | AHI | -0.026 | 0.142 | 0.36 | 3249 |
| Eubacteriales sp. (HG3A.0600) | AHI | -0.026 | 0.143 | 0.361 | 3249 |
| Eubacteriales sp. (HG3A.0609) | AHI | -0.026 | 0.143 | 0.361 | 3249 |
| Oscillospiraceae sp. (HG3A.0382) | AHI | -0.026 | 0.144 | 0.362 | 3249 |
| Barnesiellaceae sp. (HG3A.1180) | AHI | -0.025 | 0.146 | 0.366 | 3249 |
| Oscillospiraceae sp. (HG3A.1421) | AHI | 0.026 | 0.147 | 0.366 | 3249 |
| Eubacteriales sp. (HG3A.0427) | AHI | -0.026 | 0.148 | 0.367 | 3249 |
| Eubacteriales sp. (HG3A.0136) | AHI | -0.026 | 0.149 | 0.369 | 3249 |
| Eubacteriales sp. (HG3A.0158) | AHI | -0.026 | 0.15 | 0.37 | 3249 |
| Eubacteriales sp. (HG3A.0221) | AHI | -0.026 | 0.15 | 0.37 | 3249 |
| Clostridia sp. (HG3A.0011) | AHI | 0.025 | 0.153 | 0.371 | 3249 |
| Eubacteriales sp. (HG3A.0116) | AHI | -0.025 | 0.154 | 0.371 | 3249 |
| Eubacteriales sp. (HG3A.0213) | AHI | -0.025 | 0.151 | 0.371 | 3249 |
| Eubacteriales sp. (HG3A.0289) | AHI | -0.025 | 0.153 | 0.371 | 3249 |
| Anaerotruncus colihominis (HG3A.0307) | AHI | 0.025 | 0.153 | 0.371 | 3249 |
| Anaerotruncus massiliensis (HG3A.0460) | AHI | -0.025 | 0.153 | 0.371 | 3249 |
| Eubacteriales sp. (HG3A.0977) | AHI | -0.026 | 0.155 | 0.371 | 3249 |
| Eubacteriales sp. (HG3A.1167) | AHI | -0.026 | 0.153 | 0.371 | 3249 |
| Bacteria sp. (HG3A.0500) | AHI | -0.025 | 0.156 | 0.374 | 3249 |
| Collinsella sp. WCA1-178-WT-3 (M2) (HG3A.1245) | AHI | 0.025 | 0.158 | 0.377 | 3249 |
| Pediococcus acidilactici (HG3A.1468) | AHI | 0.025 | 0.158 | 0.377 | 3249 |

| Sellimonas intestinalis (HG3A.0417) | AHI | 0.025 | 0.159 | 0.378 | 3249 |
| --- | --- | --- | --- | --- | --- |
| Eubacteriales sp. (HG3A.0577) | AHI | -0.025 | 0.161 | 0.379 | 3249 |
| Clostridia sp. (HG3A.1427) | AHI | -0.025 | 0.16 | 0.379 | 3249 |
| Latilactobacillus sakei subsp. sakei (HG3A.0836) | AHI | 0.025 | 0.163 | 0.38 | 3249 |
| Eubacteriales sp. (HG3A.0985) | AHI | 0.025 | 0.162 | 0.38 | 3249 |
| Anaeroglobus geminatus (HG3A.1818) | AHI | 0.026 | 0.162 | 0.38 | 3249 |
| Clostridia sp. (HG3A.0564) | AHI | -0.025 | 0.165 | 0.384 | 3249 |
| Eubacteriales sp. (HG3A.0175) | AHI | -0.025 | 0.166 | 0.385 | 3249 |
| Firmicutes sp. (HG3A.0650) | AHI | -0.025 | 0.167 | 0.388 | 3249 |
| Lachnospiraceae sp. (HG3A.0399) | AHI | -0.025 | 0.169 | 0.391 | 3249 |
| Eubacteriales sp. (HG3A.0511) | AHI | -0.024 | 0.171 | 0.395 | 3249 |
| Faecalibacterium prausnitzii (HG3A.0010) | AHI | 0.024 | 0.178 | 0.401 | 3249 |
| Eubacteriales sp. (HG3A.0062) | AHI | -0.024 | 0.178 | 0.401 | 3249 |
| Eubacteriales sp. (HG3A.0093) | AHI | -0.024 | 0.176 | 0.401 | 3249 |
| Sutterella sp. KLE1602 (HG3A.0228) | AHI | 0.024 | 0.175 | 0.401 | 3249 |
| Eubacteriales sp. (HG3A.0328) | AHI | -0.024 | 0.176 | 0.401 | 3249 |
| Eubacteriales sp. (HG3A.0383) | AHI | -0.024 | 0.176 | 0.401 | 3249 |
| Firmicutes sp. (HG3A.0923) | AHI | -0.024 | 0.175 | 0.401 | 3249 |
| Clostridia sp. (HG3A.0996) | AHI | -0.025 | 0.177 | 0.401 | 3249 |
| Eubacteriales sp. (HG3A.0537) | AHI | -0.024 | 0.18 | 0.403 | 3249 |
| Eubacteriales sp. (HG3A.0859) | AHI | -0.024 | 0.179 | 0.403 | 3249 |
| Eubacteriales sp. (HG3A.0656) | AHI | -0.024 | 0.183 | 0.404 | 3249 |
| Eubacteriales sp. (HG3A.0668) | AHI | -0.024 | 0.181 | 0.404 | 3249 |
| Eubacteriales sp. (HG3A.0744) | AHI | -0.024 | 0.182 | 0.404 | 3249 |
| Firmicutes sp. (HG3A.0860) | AHI | -0.024 | 0.183 | 0.404 | 3249 |
| Actinomycetaceae sp. (HG3A.1068) | AHI | -0.024 | 0.182 | 0.404 | 3249 |
| Eubacteriales sp. (HG3A.0473) | AHI | -0.024 | 0.185 | 0.407 | 3249 |
| Eubacteriales sp. (HG3A.0184) | AHI | -0.023 | 0.19 | 0.409 | 3249 |
| Clostridiaceae sp. (HG3A.0238) | AHI | -0.023 | 0.187 | 0.409 | 3249 |
| Phocaeicola plebeius (HG3A.0423) | AHI | -0.023 | 0.186 | 0.409 | 3249 |
| Eubacteriales sp. (HG3A.0557) | AHI | -0.024 | 0.188 | 0.409 | 3249 |
| Eubacteriales sp. (HG3A.0630) | AHI | -0.023 | 0.189 | 0.409 | 3249 |
| Streptococcus anginosus (HG3A.0680) | AHI | 0.023 | 0.188 | 0.409 | 3249 |
| Eubacteriales sp. (HG3A.0715) | AHI | -0.023 | 0.19 | 0.409 | 3249 |

| Firmicutes sp. (HG3A.0769) | AHI | -0.023 | 0.19 | 0.409 | 3249 |
| --- | --- | --- | --- | --- | --- |
| Clostridia sp. (HG3A.1127) | AHI | -0.023 | 0.187 | 0.409 | 3249 |
| Clostridia sp. (HG3A.1039) | AHI | -0.024 | 0.191 | 0.41 | 3249 |
| Ruminococcus sp. AF46-10NS (HG3A.0271) | AHI | 0.023 | 0.193 | 0.411 | 3249 |
| Rothia mucilaginosa (HG3A.0559) | AHI | 0.023 | 0.192 | 0.411 | 3249 |
| Eubacteriales sp. (HG3A.0516) | AHI | -0.023 | 0.194 | 0.412 | 3249 |
| Oscillospiraceae sp. (HG3A.0210) | AHI | -0.023 | 0.195 | 0.413 | 3249 |
| Eubacteriales sp. (HG3A.0406) | AHI | -0.023 | 0.198 | 0.413 | 3249 |
| Blautia sp. (HG3A.0416) | AHI | 0.023 | 0.198 | 0.413 | 3249 |
| Eubacteriales sp. (HG3A.0476) | AHI | -0.024 | 0.196 | 0.413 | 3249 |
| Eubacteriales sp. (HG3A.0670) | AHI | -0.023 | 0.197 | 0.413 | 3249 |
| Dorea phocaeensis (HG3A.0865) | AHI | 0.023 | 0.196 | 0.413 | 3249 |
| Firmicutes sp. (HG3A.1124) | AHI | -0.023 | 0.197 | 0.413 | 3249 |
| Coprococcus catus (HG3A.0037) | AHI | 0.023 | 0.199 | 0.414 | 3249 |
| Butyricimonas virosa (HG3A.0199) | AHI | -0.023 | 0.2 | 0.416 | 3249 |
| Oscillospiraceae sp. (HG3A.0805) | AHI | -0.023 | 0.201 | 0.416 | 3249 |
| Eggerthellales sp. (HG3A.0848) | AHI | -0.023 | 0.202 | 0.417 | 3249 |
| Bacteroides caccae (HG3A.0066) | AHI | -0.023 | 0.206 | 0.42 | 3249 |
| Eubacteriales sp. (HG3A.0188) | AHI | -0.023 | 0.205 | 0.42 | 3249 |
| Oscillospiraceae sp. (HG3A.0693) | AHI | -0.023 | 0.205 | 0.42 | 3249 |
| Coprococcus sp. OM04-5BH (HG3A.1028) | AHI | -0.023 | 0.205 | 0.42 | 3249 |
| Eubacteriales sp. (HG3A.0851) | AHI | -0.022 | 0.207 | 0.422 | 3249 |
| Eubacteriales sp. (HG3A.0087) | AHI | -0.022 | 0.209 | 0.423 | 3249 |
| Eubacteriales sp. (HG3A.0189) | AHI | -0.022 | 0.208 | 0.423 | 3249 |
| Pseudoflavonifractor sp. An184 (HG3A.0253) | AHI | -0.023 | 0.211 | 0.424 | 3249 |
| Eubacteriales sp. (HG3A.0518) | AHI | -0.023 | 0.21 | 0.424 | 3249 |
| Clostridia sp. (HG3A.1111) | AHI | -0.022 | 0.21 | 0.424 | 3249 |
| Eubacteriales sp. (HG3A.0760) | AHI | -0.023 | 0.213 | 0.426 | 3249 |
| Parabacteroides gordonii (HG3A.0989) | AHI | 0.022 | 0.212 | 0.426 | 3249 |
| Oscillibacter sp. PEA192 (HG3A.0021) | AHI | 0.022 | 0.215 | 0.429 | 3249 |
| Eubacteriales sp. (HG3A.0363) | AHI | -0.022 | 0.216 | 0.43 | 3249 |
| Bacteroidales sp. (HG3A.1236) | AHI | -0.022 | 0.217 | 0.43 | 3249 |
| Eubacteriales sp. (HG3A.1154) | AHI | -0.022 | 0.225 | 0.445 | 3249 |
| Barnesiella intestinihominis (HG3A.0055) | AHI | -0.021 | 0.229 | 0.451 | 3249 |

| Clostridia sp. (HG3A.0783) | AHI | -0.021 | 0.23 | 0.451 | 3249 |
| --- | --- | --- | --- | --- | --- |
| Lachnospiraceae sp. (HG3A.1641) | AHI | 0.022 | 0.229 | 0.451 | 3249 |
| Clostridia sp. (HG3A.0946) | AHI | -0.021 | 0.231 | 0.452 | 3249 |
| Oxalobacter sp. (HG3A.1097) | AHI | 0.021 | 0.231 | 0.452 | 3249 |
| Clostridium sp. M62/1 (HG3A.0354) | AHI | 0.021 | 0.234 | 0.455 | 3249 |
| Clostridia sp. (HG3A.0385) | AHI | -0.021 | 0.235 | 0.455 | 3249 |
| Eubacteriales sp. (HG3A.0568) | AHI | -0.021 | 0.234 | 0.455 | 3249 |
| Clostridia sp. (HG3A.1108) | AHI | -0.021 | 0.235 | 0.455 | 3249 |
| Anaerobutyricum hallii (HG3A.0012) | AHI | 0.021 | 0.239 | 0.46 | 3249 |
| Alistipes indistinctus (HG3A.0121) | AHI | -0.021 | 0.24 | 0.46 | 3249 |
| Eubacteriales sp. (HG3A.0267) | AHI | -0.021 | 0.24 | 0.46 | 3249 |
| Eubacteriales sp. (HG3A.0791) | AHI | -0.021 | 0.239 | 0.46 | 3249 |
| Eubacteriales sp. (HG3A.0627) | AHI | -0.021 | 0.241 | 0.461 | 3249 |
| Eubacteriales sp. (HG3A.0685) | AHI | -0.021 | 0.242 | 0.461 | 3249 |
| Eubacteriales sp. (HG3A.0694) | AHI | -0.021 | 0.241 | 0.461 | 3249 |
| Eubacteriales sp. (HG3A.0232) | AHI | -0.021 | 0.245 | 0.462 | 3249 |
| Eubacteriales sp. (HG3A.0580) | AHI | -0.021 | 0.245 | 0.462 | 3249 |
| Eubacteriales sp. (HG3A.0878) | AHI | 0.021 | 0.245 | 0.462 | 3249 |
| Clostridia sp. (HG3A.1062) | AHI | -0.021 | 0.243 | 0.462 | 3249 |
| Eubacterium sp. AF16-48 (HG3A.0219) | AHI | -0.021 | 0.25 | 0.464 | 3249 |
| Alistipes dispar (HG3A.0281) | AHI | -0.02 | 0.249 | 0.464 | 3249 |
| Ruminococcus sp. (HG3A.0337) | AHI | -0.021 | 0.248 | 0.464 | 3249 |
| Eubacteriales sp. (HG3A.0950) | AHI | -0.02 | 0.247 | 0.464 | 3249 |
| Eubacteriales sp. (HG3A.1003) | AHI | -0.021 | 0.249 | 0.464 | 3249 |
| Clostridia sp. (HG3A.1410) | AHI | -0.021 | 0.248 | 0.464 | 3249 |
| Eubacteriales sp. (HG3A.0113) | AHI | -0.021 | 0.253 | 0.465 | 3249 |
| Eubacteriales sp. (HG3A.0498) | AHI | 0.02 | 0.253 | 0.465 | 3249 |
| Eubacteriales sp. (HG3A.0613) | AHI | -0.02 | 0.253 | 0.465 | 3249 |
| Firmicutes sp. (HG3A.0874) | AHI | -0.02 | 0.253 | 0.465 | 3249 |
| Oxalobacter sp. (HG3A.1218) | AHI | -0.021 | 0.252 | 0.465 | 3249 |
| Eubacteriales sp. (HG3A.0144) | AHI | -0.02 | 0.254 | 0.466 | 3249 |
| Faecalibacterium sp. (HG3A.0073) | AHI | -0.02 | 0.256 | 0.468 | 3249 |
| Bacteroides nordii (HG3A.0290) | AHI | -0.021 | 0.258 | 0.469 | 3249 |
| Eubacteriales sp. (HG3A.0474) | AHI | -0.02 | 0.258 | 0.469 | 3249 |

| Eggerthellales sp. (HG3A.0174) | AHI | -0.02 | 0.26 | 0.471 | 3249 |
| --- | --- | --- | --- | --- | --- |
| Eubacteriales sp. (HG3A.0186) | AHI | -0.02 | 0.262 | 0.471 | 3249 |
| Firmicutes sp. (HG3A.0581) | AHI | -0.02 | 0.259 | 0.471 | 3249 |
| Akkermansia sp. BIOML-A59 (HG3A.0800) | AHI | -0.02 | 0.261 | 0.471 | 3249 |
| Clostridia sp. (HG3A.1298) | AHI | -0.02 | 0.261 | 0.471 | 3249 |
| Bacteria sp. (HG3A.1349) | AHI | -0.02 | 0.264 | 0.474 | 3249 |
| Eubacteriales sp. (HG3A.0148) | AHI | -0.02 | 0.268 | 0.478 | 3249 |
| Blautia hydrogenotrophica (HG3A.0430) | AHI | 0.02 | 0.267 | 0.478 | 3249 |
| Eubacteriales sp. (HG3A.0489) | AHI | -0.019 | 0.272 | 0.485 | 3249 |
| Clostridia sp. (HG3A.0368) | AHI | -0.019 | 0.276 | 0.49 | 3249 |
| Firmicutes sp. (HG3A.0454) | AHI | -0.019 | 0.276 | 0.49 | 3249 |
| Oscillospiraceae sp. (HG3A.0475) | AHI | -0.019 | 0.278 | 0.49 | 3249 |
| Clostridia sp. (HG3A.1057) | AHI | -0.019 | 0.277 | 0.49 | 3249 |
| Methanobrevibacter smithii (HG3A.0152) | AHI | -0.019 | 0.279 | 0.491 | 3249 |
| Clostridia sp. (HG3A.0550) | AHI | -0.019 | 0.28 | 0.491 | 3249 |
| Eubacteriales sp. (HG3A.0902) | AHI | -0.019 | 0.281 | 0.491 | 3249 |
| Clostridia sp. (HG3A.1247) | AHI | -0.019 | 0.28 | 0.491 | 3249 |
| Clostridia sp. (HG3A.1038) | AHI | -0.019 | 0.283 | 0.493 | 3249 |
| Candidatus Borkfalkiales sp. (HG3A.1329) | AHI | -0.019 | 0.283 | 0.493 | 3249 |
| Pediococcus pentosaceus (HG3A.1246) | AHI | 0.019 | 0.284 | 0.494 | 3249 |
| Clostridia sp. (HG3A.1262) | AHI | -0.019 | 0.286 | 0.496 | 3249 |
| Eubacteriales sp. (HG3A.0477) | AHI | -0.019 | 0.29 | 0.502 | 3249 |
| Eubacteriales sp. (HG3A.0493) | AHI | -0.019 | 0.293 | 0.504 | 3249 |
| Clostridia sp. (HG3A.0787) | AHI | -0.019 | 0.294 | 0.504 | 3249 |
| Eubacteriales sp. (HG3A.0910) | AHI | -0.019 | 0.292 | 0.504 | 3249 |
| Eubacteriales sp. (HG3A.1305) | AHI | -0.019 | 0.293 | 0.504 | 3249 |
| Eubacteriales sp. (HG3A.1573) | AHI | -0.019 | 0.294 | 0.504 | 3249 |
| Eubacteriales sp. (HG3A.0592) | AHI | -0.019 | 0.297 | 0.507 | 3249 |
| Eubacteriales sp. (HG3A.0320) | AHI | -0.019 | 0.298 | 0.509 | 3249 |
| Flavonifractor sp. An10 (HG3A.0495) | AHI | -0.019 | 0.3 | 0.509 | 3249 |
| Eubacteriales sp. (HG3A.0711) | AHI | -0.018 | 0.301 | 0.509 | 3249 |
| Eubacteriales sp. (HG3A.0821) | AHI | -0.018 | 0.3 | 0.509 | 3249 |
| Eubacteriales sp. (HG3A.1030) | AHI | -0.019 | 0.301 | 0.509 | 3249 |
| Faecalibacterium prausnitzii (HG3A.0025) | AHI | 0.018 | 0.303 | 0.511 | 3249 |

| Turicibacter sanguinis (HG3A.0274) | AHI | -0.018 | 0.305 | 0.511 | 3249 |
| --- | --- | --- | --- | --- | --- |
| Eubacteriales sp. (HG3A.1087) | AHI | -0.019 | 0.304 | 0.511 | 3249 |
| Succinatimonas hippei (HG3A.1322) | AHI | 0.018 | 0.305 | 0.511 | 3249 |
| Allisonella histaminiformans (HG3A.0332) | AHI | 0.018 | 0.307 | 0.512 | 3249 |
| Eubacteriales sp. (HG3A.0786) | AHI | 0.018 | 0.306 | 0.512 | 3249 |
| Clostridia sp. (HG3A.0852) | AHI | -0.018 | 0.307 | 0.512 | 3249 |
| Bacteroides intestinalis (HG3A.0265) | AHI | -0.018 | 0.309 | 0.513 | 3249 |
| Clostridia sp. (HG3A.1058) | AHI | -0.018 | 0.309 | 0.513 | 3249 |
| Oscillospiraceae sp. (HG3A.0461) | AHI | -0.018 | 0.312 | 0.515 | 3249 |
| Eubacteriales sp. (HG3A.0671) | AHI | -0.018 | 0.312 | 0.515 | 3249 |
| Streptococcus gallolyticus subsp. gallolyticus (HG3A.1651) | AHI | 0.018 | 0.313 | 0.515 | 3249 |
| Eubacteriales sp. (HG3A.0192) | AHI | -0.018 | 0.314 | 0.516 | 3249 |
| Eubacteriales sp. (HG3A.0428) | AHI | -0.018 | 0.314 | 0.516 | 3249 |
| Haemophilus parainfluenzae (HG3A.0181) | AHI | -0.018 | 0.317 | 0.518 | 3249 |
| Bacteria sp. (HG3A.0218) | AHI | -0.018 | 0.318 | 0.518 | 3249 |
| Eubacteriales sp. (HG3A.0618) | AHI | -0.018 | 0.317 | 0.518 | 3249 |
| Clostridia sp. (HG3A.1128) | AHI | -0.018 | 0.317 | 0.518 | 3249 |
| Eubacteriales sp. (HG3A.0633) | AHI | -0.018 | 0.322 | 0.524 | 3249 |
| Oscillospiraceae sp. (HG3A.0412) | AHI | -0.018 | 0.324 | 0.525 | 3249 |
| Desulfovibrionales sp. (HG3A.0266) | AHI | -0.017 | 0.327 | 0.529 | 3249 |
| Eubacteriales sp. (HG3A.0350) | AHI | -0.017 | 0.33 | 0.529 | 3249 |
| Oscillospiraceae sp. (HG3A.0612) | AHI | 0.017 | 0.33 | 0.529 | 3249 |
| Streptococcus gordonii (HG3A.0713) | AHI | 0.017 | 0.329 | 0.529 | 3249 |
| Firmicutes sp. (HG3A.0915) | AHI | -0.017 | 0.328 | 0.529 | 3249 |
| Clostridia sp. (HG3A.1157) | AHI | -0.017 | 0.329 | 0.529 | 3249 |
| Parabacteroides goldsteinii (HG3A.0279) | AHI | -0.017 | 0.332 | 0.53 | 3249 |
| Senegalimassilia anaerobia (HG3A.0129) | AHI | 0.017 | 0.334 | 0.532 | 3249 |
| Eubacteriales sp. (HG3A.0345) | AHI | -0.017 | 0.335 | 0.532 | 3249 |
| Lachnospiraceae sp. (HG3A.0903) | AHI | -0.017 | 0.334 | 0.532 | 3249 |
| Akkermansia muciniphila (HG3A.0110) | AHI | -0.017 | 0.339 | 0.534 | 3249 |
| Eubacteriales sp. (HG3A.0338) | AHI | -0.017 | 0.338 | 0.534 | 3249 |
| Oscillospiraceae sp. (HG3A.0765) | AHI | -0.017 | 0.337 | 0.534 | 3249 |
| Bacteria sp. (HG3A.0911) | AHI | -0.017 | 0.339 | 0.534 | 3249 |
| Eubacteriales sp. (HG3A.1187) | AHI | 0.017 | 0.34 | 0.534 | 3249 |

| Butyricicoccus sp. (HG3A.0008) | AHI | 0.017 | 0.343 | 0.535 | 3249 |
| --- | --- | --- | --- | --- | --- |
| Eubacteriales sp. (HG3A.0468) | AHI | -0.017 | 0.344 | 0.535 | 3249 |
| Bifidobacterium animalis subsp. lactis (HG3A.0513) | AHI | -0.017 | 0.343 | 0.535 | 3249 |
| Anaerotignum lactatifermentans (HG3A.0676) | AHI | 0.017 | 0.341 | 0.535 | 3249 |
| Coprococcus sp. AM27-12LB (HG3A.0687) | AHI | 0.017 | 0.343 | 0.535 | 3249 |
| Limosilactobacillus vaginalis (HG3A.1341) | AHI | 0.017 | 0.344 | 0.535 | 3249 |
| Erysipelatoclostridium ramosum (HG3A.0538) | AHI | 0.017 | 0.35 | 0.543 | 3249 |
| Firmicutes sp. (HG3A.0526) | AHI | -0.017 | 0.353 | 0.546 | 3249 |
| Eubacteriales sp. (HG3A.1292) | AHI | -0.017 | 0.353 | 0.546 | 3249 |
| Bacteroidales sp. (HG3A.0147) | AHI | -0.017 | 0.355 | 0.547 | 3249 |
| Eubacteriales sp. (HG3A.0405) | AHI | -0.017 | 0.356 | 0.547 | 3249 |
| Eubacteriales sp. (HG3A.0441) | AHI | -0.016 | 0.356 | 0.547 | 3249 |
| Eubacteriales sp. (HG3A.0621) | AHI | -0.016 | 0.36 | 0.551 | 3249 |
| Dialister pneumosintes (HG3A.1496) | AHI | 0.017 | 0.36 | 0.551 | 3249 |
| Oscillospiraceae sp. (HG3A.0507) | AHI | -0.016 | 0.363 | 0.553 | 3249 |
| Eubacteriales sp. (HG3A.1332) | AHI | -0.017 | 0.362 | 0.553 | 3249 |
| Eubacteriales sp. (HG3A.0305) | AHI | -0.016 | 0.366 | 0.556 | 3249 |
| Eubacteriales sp. (HG3A.0857) | AHI | -0.016 | 0.366 | 0.556 | 3249 |
| Ruminococcus sp. AM42-11 (HG3A.0002) | AHI | 0.016 | 0.369 | 0.557 | 3249 |
| Eubacteriales sp. (HG3A.0323) | AHI | -0.016 | 0.369 | 0.557 | 3249 |
| Eubacteriales sp. (HG3A.0358) | AHI | -0.016 | 0.368 | 0.557 | 3249 |
| Eubacteriales sp. (HG3A.0781) | AHI | -0.016 | 0.369 | 0.557 | 3249 |
| Peptostreptococcaceae sp. (HG3A.0200) | AHI | -0.016 | 0.371 | 0.558 | 3249 |
| Proteobacteria sp. (HG3A.0327) | AHI | -0.016 | 0.373 | 0.56 | 3249 |
| Blautia sp. AF19-10LB (HG3A.0157) | AHI | -0.016 | 0.375 | 0.562 | 3249 |
| Eubacteriales sp. (HG3A.1199) | AHI | 0.016 | 0.376 | 0.562 | 3249 |
| Desulfovibrio fairfieldensis (HG3A.0529) | AHI | -0.015 | 0.383 | 0.572 | 3249 |
| Roseburia faecis (HG3A.0058) | AHI | 0.016 | 0.385 | 0.574 | 3249 |
| Eubacteriales sp. (HG3A.0939) | AHI | -0.015 | 0.388 | 0.576 | 3249 |
| Eubacteriales sp. (HG3A.0528) | AHI | -0.016 | 0.389 | 0.577 | 3249 |
| Eubacteriales sp. (HG3A.0717) | AHI | -0.015 | 0.39 | 0.577 | 3249 |
| Streptococcus parasanguinis (HG3A.0117) | AHI | 0.015 | 0.395 | 0.58 | 3249 |
| Ruminococcus sp. (HG3A.0126) | AHI | -0.015 | 0.396 | 0.58 | 3249 |
| Eubacteriales sp. (HG3A.0639) | AHI | -0.015 | 0.394 | 0.58 | 3249 |

| Eubacteriales sp. (HG3A.0773) | AHI | -0.015 | 0.394 | 0.58 | 3249 |
| --- | --- | --- | --- | --- | --- |
| Blautia producta (HG3A.0905) | AHI | 0.015 | 0.394 | 0.58 | 3249 |
| Eubacteriales sp. (HG3A.1045) | AHI | -0.015 | 0.395 | 0.58 | 3249 |
| Firmicutes sp. (HG3A.1345) | AHI | -0.015 | 0.397 | 0.58 | 3249 |
| Bacteroidales sp. (HG3A.0340) | AHI | -0.015 | 0.402 | 0.586 | 3249 |
| Eubacteriales sp. (HG3A.0376) | AHI | -0.015 | 0.407 | 0.586 | 3249 |
| Eubacteriales sp. (HG3A.0453) | AHI | -0.015 | 0.403 | 0.586 | 3249 |
| Clostridiaceae sp. (HG3A.0471) | AHI | 0.015 | 0.406 | 0.586 | 3249 |
| Eubacteriales sp. (HG3A.0628) | AHI | -0.015 | 0.405 | 0.586 | 3249 |
| Firmicutes sp. (HG3A.0817) | AHI | 0.015 | 0.407 | 0.586 | 3249 |
| Firmicutes sp. (HG3A.0948) | AHI | -0.015 | 0.406 | 0.586 | 3249 |
| Staphylococcus aureus (HG3A.1538) | AHI | 0.015 | 0.403 | 0.586 | 3249 |
| Oxalobacter formigenes (HG3A.1755) | AHI | 0.015 | 0.408 | 0.586 | 3249 |
| Eubacteriales sp. (HG3A.0308) | AHI | -0.015 | 0.409 | 0.588 | 3249 |
| Lachnospiraceae sp. (HG3A.0393) | AHI | 0.015 | 0.41 | 0.588 | 3249 |
| Eubacteriales sp. (HG3A.0163) | AHI | -0.015 | 0.416 | 0.591 | 3249 |
| Eubacteriales sp. (HG3A.0263) | AHI | -0.015 | 0.414 | 0.591 | 3249 |
| Eubacteriales sp. (HG3A.0701) | AHI | -0.014 | 0.415 | 0.591 | 3249 |
| Clostridia sp. (HG3A.0893) | AHI | -0.015 | 0.413 | 0.591 | 3249 |
| Eubacteriales sp. (HG3A.0244) | AHI | -0.015 | 0.417 | 0.592 | 3249 |
| Eubacteriales sp. (HG3A.0396) | AHI | -0.014 | 0.418 | 0.592 | 3249 |
| Oscillospiraceae sp. (HG3A.0134) | AHI | -0.014 | 0.419 | 0.593 | 3249 |
| Clostridia sp. (HG3A.1356) | AHI | -0.014 | 0.42 | 0.593 | 3249 |
| Anaerostipes hadrus (HG3A.0003) | AHI | 0.014 | 0.423 | 0.596 | 3249 |
| Eubacteriales sp. (HG3A.0589) | AHI | -0.014 | 0.427 | 0.601 | 3249 |
| Oscillibacter sp. (HG3A.0243) | AHI | -0.014 | 0.431 | 0.605 | 3249 |
| Eubacteriales sp. (HG3A.0790) | AHI | -0.014 | 0.431 | 0.605 | 3249 |
| Firmicutes sp. (HG3A.0464) | AHI | 0.014 | 0.442 | 0.619 | 3249 |
| Clostridium sp. AF37-5 (HG3A.0076) | AHI | -0.014 | 0.444 | 0.62 | 3249 |
| Clostridium sp. AF34-13 (HG3A.0173) | AHI | -0.013 | 0.446 | 0.62 | 3249 |
| Clostridiaceae sp. (HG3A.0491) | AHI | 0.013 | 0.446 | 0.62 | 3249 |
| Eubacteriales sp. (HG3A.0535) | AHI | 0.014 | 0.445 | 0.62 | 3249 |
| Streptococcus agalactiae (HG3A.1733) | AHI | 0.013 | 0.45 | 0.624 | 3249 |
| Eubacteriales sp. (HG3A.0736) | AHI | -0.013 | 0.456 | 0.63 | 3249 |

| Eubacteriales sp. (HG3A.0754) | AHI | -0.013 | 0.455 | 0.63 | 3249 |
| --- | --- | --- | --- | --- | --- |
| Enterocloster citroniae (HG3A.0285) | AHI | 0.013 | 0.459 | 0.632 | 3249 |
| Enterococcus faecium (HG3A.0886) | AHI | 0.013 | 0.459 | 0.632 | 3249 |
| Clostridia sp. (HG3A.1193) | AHI | 0.014 | 0.46 | 0.632 | 3249 |
| Pseudoruminococcus massiliensis (HG3A.0346) | AHI | -0.013 | 0.461 | 0.633 | 3249 |
| [Clostridium] spiroforme (HG3A.0259) | AHI | 0.013 | 0.463 | 0.634 | 3249 |
| Clostridia sp. (HG3A.1035) | AHI | -0.013 | 0.463 | 0.634 | 3249 |
| Intestinimonas butyriciproducens (HG3A.0187) | AHI | -0.013 | 0.468 | 0.639 | 3249 |
| Eubacteriales sp. (HG3A.0472) | AHI | -0.013 | 0.47 | 0.64 | 3249 |
| Clostridia sp. (HG3A.0512) | AHI | -0.013 | 0.47 | 0.64 | 3249 |
| Eubacteriales sp. (HG3A.0364) | AHI | -0.013 | 0.475 | 0.642 | 3249 |
| Eubacteriales sp. (HG3A.0652) | AHI | -0.013 | 0.473 | 0.642 | 3249 |
| Prevotella sp. (HG3A.1009) | AHI | 0.013 | 0.476 | 0.642 | 3249 |
| Eubacteriales sp. (HG3A.1063) | AHI | -0.013 | 0.476 | 0.642 | 3249 |
| Eubacteriales sp. (HG3A.1094) | AHI | -0.013 | 0.472 | 0.642 | 3249 |
| Streptococcus salivarius (HG3A.0071) | AHI | 0.013 | 0.48 | 0.645 | 3249 |
| Actinomyces sp. ICM58 (HG3A.0410) | AHI | -0.013 | 0.48 | 0.645 | 3249 |
| Bacteria sp. (HG3A.1545) | AHI | -0.013 | 0.479 | 0.645 | 3249 |
| Hydrogeniiclostidium mannosilyticum (HG3A.0294) | AHI | 0.012 | 0.484 | 0.648 | 3249 |
| Eubacteriales sp. (HG3A.0367) | AHI | -0.012 | 0.484 | 0.648 | 3249 |
| Alistipes timonensis (HG3A.0586) | AHI | -0.012 | 0.486 | 0.65 | 3249 |
| Levilactobacillus brevis (HG3A.1848) | AHI | 0.012 | 0.49 | 0.653 | 3249 |
| Eubacteriales sp. (HG3A.0373) | AHI | -0.012 | 0.494 | 0.657 | 3249 |
| Eubacteriales sp. (HG3A.0514) | AHI | -0.012 | 0.494 | 0.657 | 3249 |
| Alistipes sp. (HG3A.1385) | AHI | -0.012 | 0.495 | 0.657 | 3249 |
| Eubacteriales sp. (HG3A.0588) | AHI | -0.012 | 0.498 | 0.66 | 3249 |
| Oscillospiraceae sp. (HG3A.0944) | AHI | 0.012 | 0.499 | 0.66 | 3249 |
| Erysipelatoclostridium sp. (HG3A.0313) | AHI | -0.012 | 0.502 | 0.661 | 3249 |
| Clostridia sp. (HG3A.1139) | AHI | -0.012 | 0.502 | 0.661 | 3249 |
| Firmicutes sp. (HG3A.1290) | AHI | 0.012 | 0.504 | 0.661 | 3249 |
| Clostridia sp. (HG3A.1417) | AHI | -0.012 | 0.502 | 0.661 | 3249 |
| Lachnospiraceae sp. (HG3A.1525) | AHI | -0.012 | 0.504 | 0.661 | 3249 |
| Eubacteriales sp. (HG3A.1102) | AHI | -0.012 | 0.511 | 0.669 | 3249 |
| Lachnospiraceae sp. (HG3A.0831) | AHI | -0.012 | 0.512 | 0.67 | 3249 |

| Eubacteriales sp. (HG3A.1393) | AHI | -0.012 | 0.516 | 0.673 | 3249 |
| --- | --- | --- | --- | --- | --- |
| Ruthenibacterium lactatiformans (HG3A.0020) | AHI | 0.011 | 0.524 | 0.678 | 3249 |
| Eubacteriales sp. (HG3A.0457) | AHI | -0.011 | 0.522 | 0.678 | 3249 |
| Eubacteriales sp. (HG3A.0496) | AHI | -0.011 | 0.524 | 0.678 | 3249 |
| Victivallis lenta (HG3A.0525) | AHI | -0.011 | 0.524 | 0.678 | 3249 |
| Atopobiaceae sp. (HG3A.0937) | AHI | 0.011 | 0.523 | 0.678 | 3249 |
| Oscillospiraceae sp. (HG3A.1491) | AHI | 0.011 | 0.525 | 0.678 | 3249 |
| Blautia producta (HG3A.0619) | AHI | 0.011 | 0.529 | 0.679 | 3249 |
| Erysipelotrichales sp. (HG3A.0809) | AHI | 0.011 | 0.528 | 0.679 | 3249 |
| Firmicutes sp. (HG3A.1162) | AHI | -0.012 | 0.526 | 0.679 | 3249 |
| Alloscardovia omnicolens (HG3A.1279) | AHI | 0.011 | 0.529 | 0.679 | 3249 |
| Eubacteriales sp. (HG3A.0080) | AHI | 0.011 | 0.534 | 0.684 | 3249 |
| Eubacteriales sp. (HG3A.0392) | AHI | -0.011 | 0.535 | 0.684 | 3249 |
| Pseudoflavonifractor sp. (HG3A.0844) | AHI | 0.011 | 0.54 | 0.689 | 3249 |
| Intestinimonas sp. (HG3A.1018) | AHI | 0.011 | 0.54 | 0.689 | 3249 |
| Eubacteriaceae sp. (HG3A.0591) | AHI | 0.011 | 0.544 | 0.69 | 3249 |
| Eubacteriales sp. (HG3A.0881) | AHI | -0.011 | 0.543 | 0.69 | 3249 |
| Eubacteriales sp. (HG3A.0967) | AHI | -0.011 | 0.543 | 0.69 | 3249 |
| Clostridia sp. (HG3A.1493) | AHI | -0.011 | 0.545 | 0.69 | 3249 |
| [Clostridium] innocuum (HG3A.0365) | AHI | 0.011 | 0.548 | 0.692 | 3249 |
| Streptococcus mutans (HG3A.0677) | AHI | -0.011 | 0.547 | 0.692 | 3249 |
| Eubacteriales sp. (HG3A.0962) | AHI | 0.011 | 0.551 | 0.695 | 3249 |
| Clostridia sp. (HG3A.1625) | AHI | 0.01 | 0.555 | 0.699 | 3249 |
| Clostridia sp. (HG3A.1609) | AHI | -0.01 | 0.557 | 0.7 | 3249 |
| Roseburia sp. AM16-25 (HG3A.0344) | AHI | -0.011 | 0.559 | 0.702 | 3249 |
| Dorea sp. AF24-7LB (HG3A.0086) | AHI | 0.01 | 0.561 | 0.703 | 3249 |
| Lachnospiraceae sp. (HG3A.0127) | AHI | -0.01 | 0.563 | 0.703 | 3249 |
| Eubacteriales sp. (HG3A.1439) | AHI | 0.01 | 0.562 | 0.703 | 3249 |
| Eisenbergiella tayi (HG3A.0355) | AHI | -0.01 | 0.565 | 0.704 | 3249 |
| Firmicutes sp. (HG3A.1082) | AHI | -0.01 | 0.566 | 0.705 | 3249 |
| Clostridia sp. (HG3A.1220) | AHI | -0.01 | 0.567 | 0.705 | 3249 |
| Coprobacter fastidiosus (HG3A.0182) | AHI | -0.01 | 0.57 | 0.706 | 3249 |
| Clostridia sp. (HG3A.0841) | AHI | -0.01 | 0.571 | 0.706 | 3249 |
| Eubacteriales sp. (HG3A.0978) | AHI | -0.01 | 0.571 | 0.706 | 3249 |

| Dorea longicatena (HG3A.0039) | AHI | 0.01 | 0.575 | 0.71 | 3249 |
| --- | --- | --- | --- | --- | --- |
| Prevotella colorans (HG3A.1470) | AHI | -0.01 | 0.578 | 0.713 | 3249 |
| Eubacteriales sp. (HG3A.1546) | AHI | -0.01 | 0.58 | 0.714 | 3249 |
| Enterocloster clostridioformis (HG3A.0686) | AHI | 0.01 | 0.583 | 0.715 | 3249 |
| Eubacteriales sp. (HG3A.0697) | AHI | -0.01 | 0.583 | 0.715 | 3249 |
| Oscillospiraceae sp. (HG3A.0774) | AHI | -0.01 | 0.587 | 0.72 | 3249 |
| Firmicutes sp. (HG3A.1014) | AHI | 0.01 | 0.59 | 0.722 | 3249 |
| Eubacteriales sp. (HG3A.0970) | AHI | -0.009 | 0.605 | 0.739 | 3249 |
| Bacteroidales sp. (HG3A.0789) | AHI | -0.009 | 0.607 | 0.74 | 3249 |
| Bacteroidales sp. (HG3A.1002) | AHI | -0.009 | 0.608 | 0.741 | 3249 |
| Eubacteriales sp. (HG3A.0505) | AHI | -0.009 | 0.611 | 0.742 | 3249 |
| Eubacteriales sp. (HG3A.0544) | AHI | 0.009 | 0.611 | 0.742 | 3249 |
| Eubacteriales sp. (HG3A.1226) | AHI | -0.009 | 0.615 | 0.746 | 3249 |
| Eubacteriales sp. (HG3A.0610) | AHI | 0.009 | 0.626 | 0.757 | 3249 |
| Lachnospiraceae sp. (HG3A.0625) | AHI | -0.009 | 0.626 | 0.757 | 3249 |
| Clostridia sp. (HG3A.0767) | AHI | -0.009 | 0.631 | 0.761 | 3249 |
| Streptococcus sobrinus (HG3A.1366) | AHI | 0.009 | 0.632 | 0.761 | 3249 |
| Eubacteriales sp. (HG3A.0432) | AHI | 0.008 | 0.635 | 0.764 | 3249 |
| Eubacteriales sp. (HG3A.0536) | AHI | -0.009 | 0.638 | 0.766 | 3249 |
| Streptococcus oralis subsp. oralis (HG3A.0705) | AHI | 0.008 | 0.639 | 0.767 | 3249 |
| Clostridium sp. AF15-31 (HG3A.0293) | AHI | -0.008 | 0.648 | 0.771 | 3249 |
| Coprobacillus cateniformis (HG3A.0456) | AHI | -0.008 | 0.645 | 0.771 | 3249 |
| Eubacteriales sp. (HG3A.0614) | AHI | -0.008 | 0.647 | 0.771 | 3249 |
| Eubacteriales sp. (HG3A.0649) | AHI | 0.008 | 0.647 | 0.771 | 3249 |
| Eubacteriales sp. (HG3A.1257) | AHI | -0.009 | 0.644 | 0.771 | 3249 |
| Eubacteriales sp. (HG3A.0335) | AHI | 0.008 | 0.653 | 0.775 | 3249 |
| Eubacteriales sp. (HG3A.1269) | AHI | -0.008 | 0.652 | 0.775 | 3249 |
| Bacteroidales sp. (HG3A.1446) | AHI | -0.008 | 0.656 | 0.777 | 3249 |
| Eubacteriales sp. (HG3A.0868) | AHI | -0.008 | 0.663 | 0.785 | 3249 |
| Clostridia sp. (HG3A.0929) | AHI | -0.008 | 0.67 | 0.791 | 3249 |
| Bacteria sp. (HG3A.1553) | AHI | -0.007 | 0.671 | 0.791 | 3249 |
| Eubacteriales sp. (HG3A.0502) | AHI | 0.008 | 0.674 | 0.794 | 3249 |
| Eubacteriales sp. (HG3A.0551) | AHI | -0.007 | 0.677 | 0.795 | 3249 |
| Collinsella intestinalis (HG3A.0802) | AHI | -0.007 | 0.676 | 0.795 | 3249 |

| Lachnospiraceae sp. (HG3A.1155) | AHI | -0.007 | 0.678 | 0.795 | 3249 |
| --- | --- | --- | --- | --- | --- |
| Candidatus Borkfalkia ceftriaxoniphila (HG3A.0595) | AHI | -0.007 | 0.68 | 0.796 | 3249 |
| Eubacteriales sp. (HG3A.0664) | AHI | 0.007 | 0.683 | 0.799 | 3249 |
| Faecalibacterium prausnitzii (HG3A.0241) | AHI | -0.007 | 0.689 | 0.802 | 3249 |
| Enterocloster aldenensis (HG3A.0362) | AHI | 0.007 | 0.69 | 0.802 | 3249 |
| Eubacteriales sp. (HG3A.0543) | AHI | -0.007 | 0.688 | 0.802 | 3249 |
| Ligilactobacillus salivarius (HG3A.0919) | AHI | 0.007 | 0.691 | 0.802 | 3249 |
| Eubacteriales sp. (HG3A.1067) | AHI | -0.007 | 0.687 | 0.802 | 3249 |
| Clostridia sp. (HG3A.0918) | AHI | -0.007 | 0.697 | 0.808 | 3249 |
| Sutterella wadsworthensis (HG3A.0143) | AHI | 0.007 | 0.7 | 0.81 | 3249 |
| Eubacteriales sp. (HG3A.0594) | AHI | -0.007 | 0.703 | 0.812 | 3249 |
| Eubacteriales sp. (HG3A.0547) | AHI | 0.007 | 0.71 | 0.817 | 3249 |
| Eubacteriales sp. (HG3A.1051) | AHI | 0.007 | 0.709 | 0.817 | 3249 |
| Erysipelotrichales sp. (HG3A.0283) | AHI | -0.007 | 0.711 | 0.818 | 3249 |
| Eubacteriales sp. (HG3A.0792) | AHI | -0.007 | 0.713 | 0.82 | 3249 |
| Oscillospiraceae sp. (HG3A.0343) | AHI | -0.006 | 0.721 | 0.826 | 3249 |
| Eubacteriales sp. (HG3A.1177) | AHI | -0.006 | 0.721 | 0.826 | 3249 |
| Eubacteriales sp. (HG3A.0762) | AHI | -0.006 | 0.724 | 0.828 | 3249 |
| Bacteroides cellulosilyticus (HG3A.0108) | AHI | -0.006 | 0.733 | 0.833 | 3249 |
| Tyzzerella nexilis (HG3A.0574) | AHI | 0.006 | 0.733 | 0.833 | 3249 |
| Eubacteriales sp. (HG3A.0593) | AHI | -0.006 | 0.731 | 0.833 | 3249 |
| Eubacteriales sp. (HG3A.0644) | AHI | 0.006 | 0.733 | 0.833 | 3249 |
| Clostridium perfringens (HG3A.0959) | AHI | 0.006 | 0.731 | 0.833 | 3249 |
| Eubacteriales sp. (HG3A.1006) | AHI | 0.006 | 0.736 | 0.834 | 3249 |
| Clostridia sp. (HG3A.1252) | AHI | -0.006 | 0.739 | 0.836 | 3249 |
| Scardovia wiggsiae (HG3A.1737) | AHI | -0.006 | 0.739 | 0.836 | 3249 |
| Clostridium sp. OF03-18AA (HG3A.0119) | AHI | -0.006 | 0.743 | 0.839 | 3249 |
| Evtepia gabavorous (HG3A.0114) | AHI | 0.006 | 0.747 | 0.84 | 3249 |
| Eubacteriales sp. (HG3A.0138) | AHI | -0.006 | 0.745 | 0.84 | 3249 |
| Eubacteriales sp. (HG3A.0322) | AHI | 0.006 | 0.746 | 0.84 | 3249 |
| Eubacteriales sp. (HG3A.0914) | AHI | -0.006 | 0.754 | 0.847 | 3249 |
| Eubacteriales sp. (HG3A.0369) | AHI | -0.005 | 0.761 | 0.848 | 3249 |
| Oscillospiraceae sp. (HG3A.0806) | AHI | 0.005 | 0.758 | 0.848 | 3249 |
| Eubacteriales sp. (HG3A.0846) | AHI | 0.005 | 0.761 | 0.848 | 3249 |

| Clostridia sp. (HG3A.0861) | AHI | 0.005 | 0.762 | 0.848 | 3249 |
| --- | --- | --- | --- | --- | --- |
| Eubacteriales sp. (HG3A.1136) | AHI | -0.005 | 0.759 | 0.848 | 3249 |
| Eubacteriales sp. (HG3A.1256) | AHI | -0.005 | 0.76 | 0.848 | 3249 |
| Oscillospiraceae sp. (HG3A.0146) | AHI | -0.005 | 0.766 | 0.85 | 3249 |
| Butyrivibrio crossotus (HG3A.0413) | AHI | -0.005 | 0.769 | 0.85 | 3249 |
| Oscillospiraceae sp. (HG3A.0576) | AHI | -0.005 | 0.769 | 0.85 | 3249 |
| Eubacteriales sp. (HG3A.0692) | AHI | -0.005 | 0.769 | 0.85 | 3249 |
| Latilactobacillus curvatus (HG3A.1505) | AHI | 0.005 | 0.768 | 0.85 | 3249 |
| Eubacteriales sp. (HG3A.0204) | AHI | -0.005 | 0.773 | 0.852 | 3249 |
| Firmicutes sp. (HG3A.0301) | AHI | -0.005 | 0.772 | 0.852 | 3249 |
| Clostridia sp. (HG3A.0519) | AHI | 0.005 | 0.777 | 0.855 | 3249 |
| Eubacteriales sp. (HG3A.0751) | AHI | 0.005 | 0.778 | 0.855 | 3249 |
| Limosilactobacillus oris (HG3A.1462) | AHI | -0.005 | 0.784 | 0.86 | 3249 |
| Victivallales sp. (HG3A.0824) | AHI | -0.005 | 0.788 | 0.863 | 3249 |
| Clostridia sp. (HG3A.1254) | AHI | -0.005 | 0.79 | 0.863 | 3249 |
| Eubacteriales sp. (HG3A.1285) | AHI | -0.005 | 0.79 | 0.863 | 3249 |
| Ruminococcus sp. AF17-22AC (HG3A.0208) | AHI | 0.005 | 0.797 | 0.869 | 3249 |
| Bacteria sp. (HG3A.1096) | AHI | -0.005 | 0.797 | 0.869 | 3249 |
| Eubacteriales sp. (HG3A.0408) | AHI | 0.004 | 0.801 | 0.872 | 3249 |
| Clostridia sp. (HG3A.0828) | AHI | -0.004 | 0.802 | 0.872 | 3249 |
| Eubacteriales sp. (HG3A.0972) | AHI | 0.004 | 0.808 | 0.876 | 3249 |
| Clostridia sp. (HG3A.1486) | AHI | -0.004 | 0.807 | 0.876 | 3249 |
| Eubacteriales sp. (HG3A.1354) | AHI | 0.004 | 0.813 | 0.88 | 3249 |
| Eubacteriales sp. (HG3A.0352) | AHI | 0.004 | 0.817 | 0.882 | 3249 |
| Eubacteriales sp. (HG3A.0447) | AHI | -0.004 | 0.817 | 0.882 | 3249 |
| Clostridia sp. (HG3A.0845) | AHI | 0.004 | 0.818 | 0.882 | 3249 |
| Eubacteriales sp. (HG3A.0973) | AHI | -0.004 | 0.822 | 0.884 | 3249 |
| Firmicutes sp. (HG3A.1471) | AHI | -0.004 | 0.823 | 0.884 | 3249 |
| Bacteria sp. (HG3A.0361) | AHI | 0.004 | 0.826 | 0.887 | 3249 |
| Eubacteriales sp. (HG3A.0983) | AHI | -0.004 | 0.829 | 0.888 | 3249 |
| Oscillospiraceae sp. (HG3A.0665) | AHI | -0.004 | 0.83 | 0.889 | 3249 |
| Eubacteriales sp. (HG3A.0333) | AHI | 0.004 | 0.834 | 0.892 | 3249 |
| Erysipelotrichaceae sp. (HG3A.0867) | AHI | 0.004 | 0.84 | 0.897 | 3249 |
| Lacticaseibacillus paracasei subsp. paracasei (HG3A.0853) | AHI | -0.003 | 0.853 | 0.907 | 3249 |

| Eubacteriales sp. (HG3A.1109) | AHI | 0.003 | 0.852 | 0.907 | 3249 |
| --- | --- | --- | --- | --- | --- |
| Clostridia sp. (HG3A.1192) | AHI | -0.003 | 0.85 | 0.907 | 3249 |
| Eubacteriales sp. (HG3A.1219) | AHI | -0.003 | 0.859 | 0.913 | 3249 |
| Eubacteriales sp. (HG3A.0820) | AHI | 0.003 | 0.874 | 0.927 | 3249 |
| Proteobacteria sp. (HG3A.0360) | AHI | -0.003 | 0.876 | 0.928 | 3249 |
| Clostridiaceae sp. (HG3A.0330) | AHI | -0.003 | 0.88 | 0.931 | 3249 |
| Lachnospiraceae sp. (HG3A.0217) | AHI | -0.003 | 0.883 | 0.933 | 3249 |
| Eubacteriales sp. (HG3A.0647) | AHI | 0.003 | 0.886 | 0.935 | 3249 |
| Clostridia sp. (HG3A.0521) | AHI | -0.002 | 0.899 | 0.944 | 3249 |
| Oscillospiraceae sp. (HG3A.0739) | AHI | -0.002 | 0.897 | 0.944 | 3249 |
| Oscillospiraceae sp. (HG3A.0849) | AHI | 0.002 | 0.9 | 0.944 | 3249 |
| Agathobaculum desmolans (HG3A.1429) | AHI | 0.002 | 0.901 | 0.944 | 3249 |
| Solobacterium moorei (HG3A.1589) | AHI | -0.002 | 0.899 | 0.944 | 3249 |
| Eubacteriales sp. (HG3A.0916) | AHI | -0.002 | 0.904 | 0.946 | 3249 |
| Lachnospiraceae sp. (HG3A.0252) | AHI | -0.002 | 0.912 | 0.95 | 3249 |
| Eubacteriales sp. (HG3A.1131) | AHI | -0.002 | 0.913 | 0.95 | 3249 |
| Clostridia sp. (HG3A.1137) | AHI | 0.002 | 0.913 | 0.95 | 3249 |
| Oscillospiraceae sp. (HG3A.1588) | AHI | 0.002 | 0.912 | 0.95 | 3249 |
| Eubacteriales sp. (HG3A.1422) | AHI | 0.002 | 0.914 | 0.951 | 3249 |
| Eubacteriales sp. (HG3A.0302) | AHI | 0.002 | 0.926 | 0.961 | 3249 |
| Eubacterium sp. AF17-7 (HG3A.0165) | AHI | 0.002 | 0.932 | 0.964 | 3249 |
| Eubacteriales sp. (HG3A.0663) | AHI | -0.002 | 0.931 | 0.964 | 3249 |
| Clostridia sp. (HG3A.0706) | AHI | -0.002 | 0.93 | 0.964 | 3249 |
| Eubacteriales sp. (HG3A.0137) | AHI | 0.001 | 0.943 | 0.967 | 3249 |
| Eubacteriales sp. (HG3A.0490) | AHI | 0.001 | 0.939 | 0.967 | 3249 |
| Firmicutes sp. (HG3A.1091) | AHI | 0.001 | 0.941 | 0.967 | 3249 |
| Eubacteriales sp. (HG3A.1134) | AHI | -0.001 | 0.938 | 0.967 | 3249 |
| Veillonella tobetsuensis (HG3A.1344) | AHI | -0.001 | 0.941 | 0.967 | 3249 |
| Fusobacterium nucleatum subsp. animalis (HG3A.1418) | AHI | -0.001 | 0.941 | 0.967 | 3249 |
| Lachnospiraceae sp. (HG3A.0172) | AHI | -0.001 | 0.944 | 0.968 | 3249 |
| Traorella massiliensis (HG3A.0669) | AHI | -0.001 | 0.949 | 0.969 | 3249 |
| Firmicutes sp. (HG3A.1048) | AHI | -0.001 | 0.949 | 0.969 | 3249 |
| Enterocloster sp. (HG3A.1529) | AHI | 0.001 | 0.948 | 0.969 | 3249 |
| Clostridia sp. (HG3A.0401) | AHI | 0.001 | 0.959 | 0.974 | 3249 |

| Bacteria sp. (HG3A.0708) | AHI | -0.001 | 0.96 | 0.974 | 3249 |
| --- | --- | --- | --- | --- | --- |
| Eubacteriales sp. (HG3A.0873) | AHI | 0.001 | 0.959 | 0.974 | 3249 |
| Eubacteriales sp. (HG3A.0924) | AHI | 0.001 | 0.956 | 0.974 | 3249 |
| Collinsella phocaeensis (HG3A.1340) | AHI | -0.001 | 0.96 | 0.974 | 3249 |
| Clostridia sp. (HG3A.0276) | AHI | -0.001 | 0.965 | 0.977 | 3249 |
| Hungatella hathewayi (HG3A.0455) | AHI | 0.001 | 0.966 | 0.977 | 3249 |
| Firmicutes sp. (HG3A.0570) | AHI | 0.001 | 0.968 | 0.977 | 3249 |
| Eubacteriales sp. (HG3A.0827) | AHI | -0.001 | 0.969 | 0.978 | 3249 |
| Clostridium sp. OM07-9AC (HG3A.0448) | AHI | 0 | 0.981 | 0.987 | 3249 |
| Firmicutes sp. (HG3A.1050) | AHI | 0 | 0.979 | 0.987 | 3249 |
| Clostridia sp. (HG3A.0909) | AHI | 0 | 0.984 | 0.989 | 3249 |
| Clostridia sp. (HG3A.0933) | AHI | 0 | 0.986 | 0.99 | 3249 |
| Faecalibacterium sp. OF04-11AC (HG3A.0070) | AHI | 0 | 0.993 | 0.994 | 3249 |
| Eubacteriales sp. (HG3A.1473) | AHI | 0 | 0.993 | 0.994 | 3249 |
| Eubacteriales sp. (HG3A.0858) | AHI | 0 | 0.995 | 0.995 | 3249 |
