# Supplementary material for: OSA Is Associated With the Human Gut Microbiota Composition and Functional Potential in the Population-Based Swedish CardioPulmonary bioImage Study
Source: Chest. 2023 Mar 15;164(2):503–16. doi: 10.1016/j.chest.2023.03.010 (PMC10410248; doi:10.1016/j.chest.2023.03.010)
Supplement: e-Table 11 [file mmc8.docx]

e-Table 11. Stratified analysis for hemoglobin level for the species associated with T90/ODI in the extended model.

Partial Spearman’s correlations of T90 and ODI with the specified species. Participants were categorized into low or high hemoglobin groups based on the sex-specific median hemoglobin level. Associations adjusted for age, sex, smoking, alcohol intake, BMI, fiber intake, total energy intake, physical activity, education, birth country, season, and DNA extraction plate. Adjustment for multiple testing using the Benjamini-Hochberg method and presented as q-values.Under the column "Metagenomics species", the information between parenthesis is the internal identifier for the respective species. AHI: apnea-hypopnea index; ODI: oxygen desaturation index; T90: percentage of time with oxygen saturation below 90%; and se: standard error.

|  | | HB low | | | | HB high | | | | Heterogeneity test | |
| --- | --- | --- | --- | --- | --- | --- | --- | --- | --- | --- | --- |
| **Metagenomic species** | **exposure** | **correlation** | **se** | **p-value** | **N** | **correlation** | **se** | **p-value** | **N** | **p-value** | **q-value** |
| Eubacteriales sp. (HG3A.1026) | ODI | 0.008 | 0.03 | 0.754 | 1558 | -0.119 | 0.02 | 1.85E-06 | 1681 | 2.09E-04 | 0.02 |
| Clostridia sp. (HG3A.0140) | ODI | -0.120 | 0.03 | 3.96E-06 | 1558 | -0.023 | 0.03 | 0.358 | 1681 | 0.009 | 0.421 |
| Alistipes communis (HG3A.0064) | ODI | -0.013 | 0.03 | 0.608 | 1558 | -0.092 | 0.02 | 2.16E-04 | 1681 | 0.028 | 0.92 |
| Anaerobutyricum hallii (HG3A.0012) | T90 | 0.044 | 0.03 | 0.095 | 1558 | 0.071 | 0.02 | 0.004 | 1681 | 0.432 | 0.952 |
| Bacteria sp. (HG3A.0634) | T90 | -0.083 | 0.03 | 0.002 | 1558 | -0.046 | 0.03 | 0.065 | 1681 | 0.317 | 0.952 |
| Bacteria sp. (HG3A.0911) | T90 | -0.018 | 0.03 | 0.479 | 1558 | -0.088 | 0.03 | 4.40E-04 | 1681 | 0.059 | 0.952 |
| Blautia obeum (HG3A.0001) | T90 | 0.065 | 0.03 | 0.013 | 1558 | 0.099 | 0.03 | 7.12E-05 | 1681 | 0.344 | 0.952 |
| Blautia obeum (HG3A.0009) | T90 | 0.079 | 0.03 | 0.002 | 1558 | 0.039 | 0.03 | 0.118 | 1681 | 0.275 | 0.952 |
| Clostridia sp. (HG3A.0140) | T90 | -0.115 | 0.03 | 1.02E-05 | 1558 | -0.038 | 0.03 | 0.128 | 1681 | 0.039 | 0.952 |
| Clostridia sp. (HG3A.0508) | T90 | -0.091 | 0.03 | 4.89E-04 | 1558 | -0.036 | 0.02 | 0.154 | 1681 | 0.112 | 0.952 |
| Clostridia sp. (HG3A.0550) | T90 | -0.087 | 0.02 | 8.13E-04 | 1558 | -0.033 | 0.02 | 0.192 | 1681 | 0.114 | 0.952 |
| Clostridia sp. (HG3A.0861) | T90 | -0.076 | 0.03 | 0.004 | 1558 | -0.048 | 0.02 | 0.055 | 1681 | 0.431 | 0.952 |
| Clostridia sp. (HG3A.0931) | T90 | -0.077 | 0.03 | 0.003 | 1558 | -0.035 | 0.02 | 0.163 | 1681 | 0.233 | 0.952 |
| Clostridia sp. (HG3A.1008) | T90 | -0.027 | 0.03 | 0.307 | 1558 | -0.084 | 0.02 | 8.19E-04 | 1681 | 0.1 | 0.952 |
| Clostridiaceae sp. (HG3A.0431) | T90 | 0.038 | 0.03 | 0.149 | 1558 | 0.067 | 0.03 | 0.007 | 1681 | 0.424 | 0.952 |
| Eubacteriales sp. (HG3A.0100) | T90 | -0.051 | 0.03 | 0.051 | 1558 | -0.079 | 0.02 | 0.002 | 1681 | 0.439 | 0.952 |
| Eubacteriales sp. (HG3A.0118) | T90 | -0.048 | 0.03 | 0.067 | 1558 | -0.081 | 0.03 | 0.001 | 1681 | 0.375 | 0.952 |
| Eubacteriales sp. (HG3A.0196) | T90 | -0.036 | 0.03 | 0.17 | 1558 | -0.063 | 0.03 | 0.012 | 1681 | 0.452 | 0.952 |
| Eubacteriales sp. (HG3A.0234) | T90 | -0.083 | 0.03 | 0.001 | 1558 | -0.041 | 0.03 | 0.099 | 1681 | 0.244 | 0.952 |
| Eubacteriales sp. (HG3A.0291) | T90 | -0.087 | 0.03 | 7.98E-04 | 1558 | -0.032 | 0.03 | 0.198 | 1681 | 0.13 | 0.952 |
| Eubacteriales sp. (HG3A.0321) | T90 | -0.043 | 0.03 | 0.099 | 1558 | -0.073 | 0.02 | 0.004 | 1681 | 0.413 | 0.952 |
| Eubacteriales sp. (HG3A.0468) | T90 | -0.093 | 0.03 | 3.42E-04 | 1558 | -0.044 | 0.03 | 0.076 | 1681 | 0.187 | 0.952 |
| Eubacteriales sp. (HG3A.0506) | T90 | -0.04 | 0.03 | 0.129 | 1558 | -0.075 | 0.03 | 0.003 | 1681 | 0.342 | 0.952 |
| Eubacteriales sp. (HG3A.0531) | T90 | -0.037 | 0.03 | 0.161 | 1558 | -0.079 | 0.02 | 0.002 | 1681 | 0.235 | 0.952 |
| Eubacteriales sp. (HG3A.0635) | T90 | -0.038 | 0.03 | 0.146 | 1558 | -0.083 | 0.02 | 8.47E-04 | 1681 | 0.207 | 0.952 |
| Eubacteriales sp. (HG3A.0703) | T90 | -0.088 | 0.03 | 6.90E-04 | 1558 | -0.056 | 0.03 | 0.024 | 1681 | 0.369 | 0.952 |
| Eubacteriales sp. (HG3A.0978) | T90 | -0.077 | 0.03 | 0.003 | 1558 | -0.029 | 0.02 | 0.246 | 1681 | 0.177 | 0.952 |

| Mediterraneibacter glycyrrhizinilyticus (HG3A.0 | T90 | 0.044 | 0.03 | 0.091 | 1558 | 0.097 | 0.03 | 1.09E-04 | 1681 | 0.147 | 0.952 |
| --- | --- | --- | --- | --- | --- | --- | --- | --- | --- | --- | --- |
| Oscillospiraceae sp. (HG3A.0207) | T90 | -0.044 | 0.03 | 0.093 | 1558 | -0.084 | 0.03 | 8.01E-04 | 1681 | 0.275 | 0.952 |
| Oscillospiraceae sp. (HG3A.0445) | T90 | -0.085 | 0.03 | 0.001 | 1558 | -0.027 | 0.03 | 0.275 | 1681 | 0.118 | 0.952 |
| Staphylococcus aureus (HG3A.1538) | T90 | 0.024 | 0.03 | 0.354 | 1558 | 0.085 | 0.02 | 6.82E-04 | 1681 | 0.095 | 0.952 |
| Alistipes shahii (HG3A.0054) | ODI | -0.075 | 0.03 | 0.004 | 1558 | -0.056 | 0.02 | 0.026 | 1681 | 0.594 | 0.953 |
| Anaerostipes sp. BG01 (HG3A.1509) | ODI | 0.063 | 0.03 | 0.016 | 1558 | 0.041 | 0.02 | 0.102 | 1681 | 0.551 | 0.953 |
| Bacteria sp. (HG3A.0483) | ODI | -0.058 | 0.03 | 0.027 | 1558 | -0.076 | 0.02 | 0.002 | 1681 | 0.595 | 0.953 |
| Bacteria sp. (HG3A.0634) | ODI | -0.087 | 0.02 | 8.62E-04 | 1558 | -0.048 | 0.02 | 0.056 | 1681 | 0.266 | 0.953 |
| Blautia obeum (HG3A.0001) | ODI | 0.06 | 0.03 | 0.022 | 1558 | 0.079 | 0.03 | 0.002 | 1681 | 0.615 | 0.953 |
| Candidatus Borkfalkiales sp. (HG3A.1397) | ODI | -0.046 | 0.03 | 0.078 | 1558 | -0.065 | 0.02 | 0.009 | 1681 | 0.6 | 0.953 |
| Clostridia sp. (HG3A.0094) | ODI | -0.075 | 0.03 | 0.004 | 1558 | -0.029 | 0.03 | 0.239 | 1681 | 0.211 | 0.953 |
| Clostridia sp. (HG3A.0272) | ODI | -0.088 | 0.02 | 7.44E-04 | 1558 | -0.039 | 0.03 | 0.118 | 1681 | 0.173 | 0.953 |
| Clostridia sp. (HG3A.0508) | ODI | -0.089 | 0.03 | 6.10E-04 | 1558 | -0.025 | 0.02 | 0.323 | 1681 | 0.074 | 0.953 |
| Clostridia sp. (HG3A.0515) | ODI | -0.106 | 0.03 | 4.32E-05 | 1558 | -0.039 | 0.02 | 0.121 | 1681 | 0.063 | 0.953 |
| Clostridia sp. (HG3A.0599) | ODI | -0.085 | 0.03 | 0.001 | 1558 | -0.035 | 0.02 | 0.159 | 1681 | 0.159 | 0.953 |
| Clostridia sp. (HG3A.0682) | ODI | -0.093 | 0.03 | 3.35E-04 | 1558 | -0.032 | 0.03 | 0.201 | 1681 | 0.098 | 0.953 |
| Clostridia sp. (HG3A.0728) | ODI | -0.052 | 0.03 | 0.047 | 1558 | -0.081 | 0.03 | 0.001 | 1681 | 0.426 | 0.953 |
| Clostridia sp. (HG3A.0815) | ODI | -0.074 | 0.03 | 0.005 | 1558 | -0.036 | 0.02 | 0.152 | 1681 | 0.292 | 0.953 |
| Clostridia sp. (HG3A.0879) | ODI | -0.047 | 0.03 | 0.074 | 1558 | -0.071 | 0.02 | 0.005 | 1681 | 0.498 | 0.953 |
| Clostridium sp. TF06-15AC (HG3A.0032) | ODI | 0.043 | 0.03 | 0.102 | 1558 | 0.084 | 0.02 | 8.26E-04 | 1681 | 0.26 | 0.953 |
| Collinsella aerofaciens (HG3A.0019) | ODI | 0.035 | 0.02 | 0.183 | 1558 | 0.066 | 0.02 | 0.009 | 1681 | 0.374 | 0.953 |
| Coprobacillus sp. (HG3A.0022) | ODI | 0.045 | 0.03 | 0.087 | 1558 | 0.079 | 0.02 | 0.002 | 1681 | 0.341 | 0.953 |
| Coprococcus eutactus (HG3A.0155) | ODI | -0.067 | 0.03 | 0.011 | 1558 | -0.049 | 0.02 | 0.049 | 1681 | 0.625 | 0.953 |
| Eggerthellaceae sp. (HG3A.0171) | ODI | -0.07 | 0.03 | 0.007 | 1558 | -0.034 | 0.03 | 0.18 | 1681 | 0.313 | 0.953 |
| Eggerthellales sp. (HG3A.0177) | ODI | -0.04 | 0.03 | 0.12 | 1558 | -0.08 | 0.02 | 0.001 | 1681 | 0.285 | 0.953 |
| Eubacteriales sp. (HG3A.0069) | ODI | -0.053 | 0.03 | 0.04 | 1558 | -0.093 | 0.03 | 1.90E-04 | 1681 | 0.278 | 0.953 |
| Eubacteriales sp. (HG3A.0084) | ODI | -0.062 | 0.03 | 0.018 | 1558 | -0.038 | 0.03 | 0.127 | 1681 | 0.515 | 0.953 |
| Eubacteriales sp. (HG3A.0120) | ODI | -0.067 | 0.03 | 0.01 | 1558 | -0.032 | 0.02 | 0.196 | 1681 | 0.326 | 0.953 |
| Eubacteriales sp. (HG3A.0149) | ODI | -0.093 | 0.03 | 3.28E-04 | 1558 | -0.061 | 0.03 | 0.015 | 1681 | 0.376 | 0.953 |
| Eubacteriales sp. (HG3A.0156) | ODI | -0.091 | 0.03 | 4.46E-04 | 1558 | -0.048 | 0.02 | 0.056 | 1681 | 0.228 | 0.953 |
| Eubacteriales sp. (HG3A.0162) | ODI | -0.081 | 0.03 | 0.002 | 1558 | -0.048 | 0.02 | 0.054 | 1681 | 0.347 | 0.953 |
| Eubacteriales sp. (HG3A.0196) | ODI | -0.044 | 0.03 | 0.09 | 1558 | -0.061 | 0.03 | 0.014 | 1681 | 0.635 | 0.953 |
| Eubacteriales sp. (HG3A.0197) | ODI | -0.093 | 0.03 | 3.41E-04 | 1558 | -0.055 | 0.03 | 0.028 | 1681 | 0.284 | 0.953 |
| Eubacteriales sp. (HG3A.0226) | ODI | -0.083 | 0.02 | 0.001 | 1558 | -0.048 | 0.03 | 0.058 | 1681 | 0.316 | 0.953 |

| Eubacteriales sp. (HG3A.0229) | ODI | -0.079 | 0.03 | 0.002 | 1558 | -0.021 | 0.03 | 0.411 | 1681 | 0.112 | 0.953 |
| --- | --- | --- | --- | --- | --- | --- | --- | --- | --- | --- | --- |
| Eubacteriales sp. (HG3A.0250) | ODI | -0.066 | 0.03 | 0.011 | 1558 | -0.037 | 0.03 | 0.139 | 1681 | 0.417 | 0.953 |
| Eubacteriales sp. (HG3A.0291) | ODI | -0.075 | 0.03 | 0.004 | 1558 | -0.03 | 0.03 | 0.23 | 1681 | 0.217 | 0.953 |
| Eubacteriales sp. (HG3A.0309) | ODI | -0.046 | 0.03 | 0.079 | 1558 | -0.07 | 0.02 | 0.005 | 1681 | 0.488 | 0.953 |
| Eubacteriales sp. (HG3A.0321) | ODI | -0.08 | 0.03 | 0.002 | 1558 | -0.045 | 0.03 | 0.07 | 1681 | 0.339 | 0.953 |
| Eubacteriales sp. (HG3A.0331) | ODI | -0.074 | 0.03 | 0.004 | 1558 | -0.055 | 0.03 | 0.029 | 1681 | 0.588 | 0.953 |
| Eubacteriales sp. (HG3A.0421) | ODI | -0.078 | 0.03 | 0.003 | 1558 | -0.049 | 0.03 | 0.05 | 1681 | 0.413 | 0.953 |
| Eubacteriales sp. (HG3A.0439) | ODI | -0.072 | 0.03 | 0.006 | 1558 | -0.042 | 0.03 | 0.09 | 1681 | 0.438 | 0.953 |
| Eubacteriales sp. (HG3A.0442) | ODI | -0.105 | 0.03 | 5.34E-05 | 1558 | -0.057 | 0.03 | 0.023 | 1681 | 0.184 | 0.953 |
| Eubacteriales sp. (HG3A.0548) | ODI | -0.072 | 0.03 | 0.006 | 1558 | -0.042 | 0.02 | 0.095 | 1681 | 0.407 | 0.953 |
| Eubacteriales sp. (HG3A.0609) | ODI | -0.042 | 0.03 | 0.106 | 1558 | -0.082 | 0.02 | 0.001 | 1681 | 0.254 | 0.953 |
| Eubacteriales sp. (HG3A.0635) | ODI | -0.027 | 0.03 | 0.308 | 1558 | -0.082 | 0.02 | 0.001 | 1681 | 0.115 | 0.953 |
| Eubacteriales sp. (HG3A.0691) | ODI | -0.062 | 0.02 | 0.017 | 1558 | -0.04 | 0.02 | 0.107 | 1681 | 0.539 | 0.953 |
| Eubacteriales sp. (HG3A.0829) | ODI | -0.046 | 0.02 | 0.078 | 1558 | -0.065 | 0.03 | 0.009 | 1681 | 0.6 | 0.953 |
| Eubacteriales sp. (HG3A.0956) | ODI | -0.069 | 0.03 | 0.008 | 1558 | -0.024 | 0.03 | 0.335 | 1681 | 0.222 | 0.953 |
| Eubacteriales sp. (HG3A.1019) | ODI | -0.028 | 0.03 | 0.282 | 1558 | -0.08 | 0.03 | 0.001 | 1681 | 0.159 | 0.953 |
| Eubacterium sp. (HG3A.0214) | ODI | 0.035 | 0.03 | 0.174 | 1558 | 0.06 | 0.03 | 0.017 | 1681 | 0.504 | 0.953 |
| Firmicutes sp. (HG3A.0397) | ODI | -0.102 | 0.03 | 8.38E-05 | 1558 | -0.039 | 0.03 | 0.118 | 1681 | 0.081 | 0.953 |
| Firmicutes sp. (HG3A.0398) | ODI | -0.077 | 0.03 | 0.003 | 1558 | -0.052 | 0.03 | 0.037 | 1681 | 0.497 | 0.953 |
| Firmicutes sp. (HG3A.1085) | ODI | -0.063 | 0.03 | 0.015 | 1558 | -0.039 | 0.02 | 0.123 | 1681 | 0.478 | 0.953 |
| Flavonifractor plautii (HG3A.0079) | ODI | 0.03 | 0.03 | 0.245 | 1558 | 0.074 | 0.02 | 0.003 | 1681 | 0.232 | 0.953 |
| Intestinimonas massiliensis (HG3A.0198) | ODI | -0.067 | 0.03 | 0.01 | 1558 | -0.048 | 0.03 | 0.055 | 1681 | 0.608 | 0.953 |
| Lachnospiraceae sp. (HG3A.0855) | ODI | -0.066 | 0.02 | 0.011 | 1558 | -0.037 | 0.02 | 0.137 | 1681 | 0.411 | 0.953 |
| Mediterraneibacter glycyrrhizinilyticus (HG3A.0 | ODI | 0.04 | 0.03 | 0.121 | 1558 | 0.068 | 0.03 | 0.006 | 1681 | 0.447 | 0.953 |
| Oscillospiraceae sp. (HG3A.0207) | ODI | -0.086 | 0.03 | 9.47E-04 | 1558 | -0.069 | 0.02 | 0.006 | 1681 | 0.625 | 0.953 |
| Oscillospiraceae sp. (HG3A.0223) | ODI | -0.069 | 0.03 | 0.008 | 1558 | -0.052 | 0.02 | 0.037 | 1681 | 0.639 | 0.953 |
| Oscillospiraceae sp. (HG3A.0445) | ODI | -0.085 | 0.03 | 0.001 | 1558 | -0.028 | 0.03 | 0.266 | 1681 | 0.109 | 0.953 |
| Oscillospiraceae sp. (HG3A.1270) | ODI | -0.072 | 0.02 | 0.005 | 1558 | -0.048 | 0.02 | 0.055 | 1681 | 0.491 | 0.953 |
| Roseburia inulinivorans (HG3A.0036) | ODI | 0.048 | 0.03 | 0.068 | 1558 | 0.08 | 0.03 | 0.001 | 1681 | 0.372 | 0.953 |
| [Ruminococcus] gnavus (HG3A.0239) | ODI | 0.05 | 0.03 | 0.057 | 1558 | 0.073 | 0.03 | 0.003 | 1681 | 0.514 | 0.953 |
| [Ruminococcus] torques (HG3A.0034) | ODI | 0.041 | 0.03 | 0.114 | 1558 | 0.075 | 0.02 | 0.003 | 1681 | 0.341 | 0.953 |
| Victivallis vadensis (HG3A.0689) | ODI | -0.082 | 0.03 | 0.002 | 1558 | -0.039 | 0.02 | 0.116 | 1681 | 0.229 | 0.953 |
| Candidatus Borkfalkiales sp. (HG3A.1329) | T90 | -0.054 | 0.03 | 0.038 | 1558 | -0.062 | 0.02 | 0.013 | 1681 | 0.823 | 0.967 |
| Clostridia sp. (HG3A.0435) | T90 | -0.077 | 0.03 | 0.003 | 1558 | -0.063 | 0.02 | 0.012 | 1681 | 0.69 | 0.967 |

| Clostridia sp. (HG3A.0470) | T90 | -0.066 | 0.03 | 0.011 | 1558 | -0.061 | 0.02 | 0.014 | 1681 | 0.892 | 0.967 |
| --- | --- | --- | --- | --- | --- | --- | --- | --- | --- | --- | --- |
| Clostridia sp. (HG3A.0515) | T90 | -0.05 | 0.03 | 0.056 | 1558 | -0.058 | 0.03 | 0.021 | 1681 | 0.826 | 0.967 |
| Clostridia sp. (HG3A.0645) | T90 | -0.064 | 0.03 | 0.014 | 1558 | -0.052 | 0.03 | 0.037 | 1681 | 0.742 | 0.967 |
| Clostridia sp. (HG3A.1057) | T90 | -0.07 | 0.03 | 0.007 | 1558 | -0.054 | 0.02 | 0.03 | 1681 | 0.644 | 0.967 |
| Clostridium sp. (HG3A.0050) | T90 | 0.049 | 0.03 | 0.058 | 1558 | 0.062 | 0.03 | 0.014 | 1681 | 0.741 | 0.967 |
| Coprococcus comes (HG3A.0016) | T90 | 0.067 | 0.03 | 0.01 | 1558 | 0.058 | 0.03 | 0.02 | 1681 | 0.814 | 0.967 |
| Dorea formicigenerans (HG3A.0006) | T90 | 0.084 | 0.03 | 0.001 | 1558 | 0.096 | 0.02 | 1.30E-04 | 1681 | 0.742 | 0.967 |
| Dorea sp. AF36-15AT (HG3A.0052) | T90 | 0.054 | 0.03 | 0.039 | 1558 | 0.069 | 0.03 | 0.006 | 1681 | 0.691 | 0.967 |
| Erysipelotrichales sp. (HG3A.1207) | T90 | 0.058 | 0.02 | 0.025 | 1558 | 0.063 | 0.03 | 0.011 | 1681 | 0.891 | 0.967 |
| Eubacteriales sp. (HG3A.0123) | T90 | 0.066 | 0.03 | 0.012 | 1558 | 0.055 | 0.03 | 0.029 | 1681 | 0.766 | 0.967 |
| Eubacteriales sp. (HG3A.0149) | T90 | -0.07 | 0.03 | 0.007 | 1558 | -0.076 | 0.03 | 0.002 | 1681 | 0.869 | 0.967 |
| Eubacteriales sp. (HG3A.0162) | T90 | -0.07 | 0.03 | 0.007 | 1558 | -0.064 | 0.02 | 0.011 | 1681 | 0.854 | 0.967 |
| Eubacteriales sp. (HG3A.0242) | T90 | -0.071 | 0.03 | 0.006 | 1558 | -0.064 | 0.03 | 0.011 | 1681 | 0.85 | 0.967 |
| Eubacteriales sp. (HG3A.0383) | T90 | -0.043 | 0.03 | 0.102 | 1558 | -0.066 | 0.02 | 0.008 | 1681 | 0.5 | 0.967 |
| Eubacteriales sp. (HG3A.0419) | T90 | -0.079 | 0.03 | 0.002 | 1558 | -0.059 | 0.03 | 0.019 | 1681 | 0.593 | 0.967 |
| Eubacteriales sp. (HG3A.0489) | T90 | -0.042 | 0.02 | 0.105 | 1558 | -0.048 | 0.02 | 0.055 | 1681 | 0.866 | 0.967 |
| Eubacteriales sp. (HG3A.0630) | T90 | -0.059 | 0.03 | 0.023 | 1558 | -0.055 | 0.02 | 0.029 | 1681 | 0.901 | 0.967 |
| Eubacteriales sp. (HG3A.0786) | T90 | 0.052 | 0.02 | 0.048 | 1558 | 0.045 | 0.03 | 0.07 | 1681 | 0.863 | 0.967 |
| Eubacteriales sp. (HG3A.1379) | T90 | -0.05 | 0.03 | 0.055 | 1558 | -0.056 | 0.03 | 0.025 | 1681 | 0.865 | 0.967 |
| Oscillibacter sp. (HG3A.0734) | T90 | -0.041 | 0.03 | 0.119 | 1558 | -0.063 | 0.03 | 0.012 | 1681 | 0.552 | 0.967 |
| Oscillospiraceae sp. (HG3A.0072) | T90 | -0.062 | 0.03 | 0.016 | 1558 | -0.056 | 0.03 | 0.025 | 1681 | 0.867 | 0.967 |
| Pediococcus acidilactici (HG3A.1468) | T90 | 0.075 | 0.02 | 0.004 | 1558 | 0.053 | 0.03 | 0.035 | 1681 | 0.557 | 0.967 |
| [Ruminococcus] gnavus (HG3A.0239) | T90 | 0.053 | 0.03 | 0.043 | 1558 | 0.07 | 0.03 | 0.005 | 1681 | 0.645 | 0.967 |
| Ruminococcus sp. AM42-11 (HG3A.0002) | T90 | 0.063 | 0.03 | 0.015 | 1558 | 0.047 | 0.03 | 0.063 | 1681 | 0.649 | 0.967 |
| Traorella massiliensis (HG3A.0669) | T90 | -0.048 | 0.03 | 0.063 | 1558 | -0.072 | 0.02 | 0.004 | 1681 | 0.484 | 0.967 |
| Eubacteriales sp. (HG3A.0311) | T90 | -0.061 | 0.03 | 0.02 | 1558 | -0.058 | 0.02 | 0.02 | 1681 | 0.949 | 0.968 |
| Firmicutes sp. (HG3A.0301) | T90 | -0.06 | 0.03 | 0.021 | 1558 | -0.062 | 0.02 | 0.013 | 1681 | 0.949 | 0.968 |
| Lachnospiraceae sp. (HG3A.0018) | T90 | 0.062 | 0.03 | 0.018 | 1558 | 0.059 | 0.03 | 0.017 | 1681 | 0.952 | 0.968 |
| Eubacteriales sp. (HG3A.0211) | T90 | -0.06 | 0.03 | 0.02 | 1558 | -0.061 | 0.03 | 0.015 | 1681 | 0.992 | 0.992 |
| Eubacteriales sp. (HG3A.0211) | ODI | -0.06 | 0.03 | 0.021 | 1558 | -0.076 | 0.03 | 0.002 | 1681 | 0.676 | 0.993 |
| Akkermansia muciniphila (HG3A.0110) | ODI | -0.057 | 0.03 | 0.029 | 1558 | -0.056 | 0.03 | 0.025 | 1681 | 0.981 | 0.996 |
| Alistipes provencensis (HG3A.0877) | ODI | -0.042 | 0.03 | 0.105 | 1558 | -0.055 | 0.02 | 0.029 | 1681 | 0.733 | 0.996 |
| Blautia massiliensis (HG3A.0023) | ODI | 0.059 | 0.03 | 0.023 | 1558 | 0.06 | 0.03 | 0.016 | 1681 | 0.983 | 0.996 |
| Clostridia sp. (HG3A.0435) | ODI | -0.064 | 0.03 | 0.014 | 1558 | -0.053 | 0.03 | 0.033 | 1681 | 0.772 | 0.996 |

| Clostridia sp. (HG3A.0470) | ODI | -0.061 | 0.03 | 0.02 | 1558 | -0.055 | 0.02 | 0.027 | 1681 | 0.882 | 0.996 |
| --- | --- | --- | --- | --- | --- | --- | --- | --- | --- | --- | --- |
| Clostridia sp. (HG3A.1020) | ODI | -0.049 | 0.03 | 0.06 | 1558 | -0.049 | 0.02 | 0.051 | 1681 | 0.996 | 0.996 |
| Clostridium sp. (HG3A.0050) | ODI | 0.058 | 0.03 | 0.027 | 1558 | 0.06 | 0.03 | 0.016 | 1681 | 0.946 | 0.996 |
| Coprococcus comes (HG3A.0016) | ODI | 0.068 | 0.03 | 0.009 | 1558 | 0.067 | 0.03 | 0.007 | 1681 | 0.987 | 0.996 |
| Eubacteriales sp. (HG3A.0083) | ODI | -0.056 | 0.03 | 0.032 | 1558 | -0.041 | 0.03 | 0.098 | 1681 | 0.689 | 0.996 |
| Eubacteriales sp. (HG3A.0085) | ODI | -0.051 | 0.03 | 0.051 | 1558 | -0.041 | 0.03 | 0.1 | 1681 | 0.787 | 0.996 |
| Eubacteriales sp. (HG3A.0100) | ODI | -0.073 | 0.03 | 0.005 | 1558 | -0.078 | 0.03 | 0.002 | 1681 | 0.908 | 0.996 |
| Eubacteriales sp. (HG3A.0118) | ODI | -0.064 | 0.03 | 0.014 | 1558 | -0.06 | 0.03 | 0.016 | 1681 | 0.924 | 0.996 |
| Eubacteriales sp. (HG3A.0153) | ODI | -0.045 | 0.03 | 0.086 | 1558 | -0.058 | 0.03 | 0.02 | 1681 | 0.718 | 0.996 |
| Eubacteriales sp. (HG3A.0193) | ODI | -0.05 | 0.03 | 0.057 | 1558 | -0.058 | 0.03 | 0.019 | 1681 | 0.805 | 0.996 |
| Eubacteriales sp. (HG3A.0215) | ODI | -0.065 | 0.03 | 0.012 | 1558 | -0.052 | 0.03 | 0.039 | 1681 | 0.711 | 0.996 |
| Eubacteriales sp. (HG3A.0242) | ODI | -0.063 | 0.03 | 0.016 | 1558 | -0.059 | 0.03 | 0.017 | 1681 | 0.934 | 0.996 |
| Eubacteriales sp. (HG3A.0269) | ODI | -0.065 | 0.02 | 0.013 | 1558 | -0.067 | 0.03 | 0.008 | 1681 | 0.959 | 0.996 |
| Eubacteriales sp. (HG3A.0311) | ODI | -0.068 | 0.03 | 0.009 | 1558 | -0.065 | 0.03 | 0.01 | 1681 | 0.93 | 0.996 |
| Eubacteriales sp. (HG3A.0329) | ODI | -0.051 | 0.03 | 0.052 | 1558 | -0.052 | 0.03 | 0.037 | 1681 | 0.962 | 0.996 |
| Eubacteriales sp. (HG3A.0419) | ODI | -0.048 | 0.03 | 0.064 | 1558 | -0.055 | 0.02 | 0.028 | 1681 | 0.85 | 0.996 |
| Eubacteriales sp. (HG3A.0506) | ODI | -0.074 | 0.03 | 0.005 | 1558 | -0.061 | 0.03 | 0.015 | 1681 | 0.726 | 0.996 |
| Eubacteriales sp. (HG3A.0572) | ODI | -0.067 | 0.03 | 0.01 | 1558 | -0.066 | 0.03 | 0.008 | 1681 | 0.968 | 0.996 |
| Eubacteriales sp. (HG3A.0718) | ODI | -0.053 | 0.03 | 0.043 | 1558 | -0.048 | 0.02 | 0.057 | 1681 | 0.886 | 0.996 |
| Eubacteriales sp. (HG3A.1294) | ODI | -0.055 | 0.03 | 0.034 | 1558 | -0.051 | 0.03 | 0.042 | 1681 | 0.912 | 0.996 |
| Firmicutes sp. (HG3A.0341) | ODI | -0.053 | 0.03 | 0.043 | 1558 | -0.057 | 0.03 | 0.023 | 1681 | 0.908 | 0.996 |
| Fusicatenibacter saccharivorans (HG3A.0004) | ODI | 0.072 | 0.03 | 0.006 | 1558 | 0.079 | 0.03 | 0.001 | 1681 | 0.843 | 0.996 |
| Intestinibacillus sp. Marseille-P4005 (HG3A.016 | ODI | 0.054 | 0.03 | 0.037 | 1558 | 0.061 | 0.02 | 0.015 | 1681 | 0.859 | 0.996 |
| Lachnospiraceae sp. (HG3A.0018) | ODI | 0.072 | 0.03 | 0.006 | 1558 | 0.073 | 0.02 | 0.003 | 1681 | 0.974 | 0.996 |
| Oscillospiraceae sp. (HG3A.0072) | ODI | -0.071 | 0.03 | 0.006 | 1558 | -0.07 | 0.03 | 0.005 | 1681 | 0.983 | 0.996 |
| Oscillospiraceae sp. (HG3A.0437) | ODI | -0.057 | 0.03 | 0.03 | 1558 | -0.048 | 0.03 | 0.055 | 1681 | 0.81 | 0.996 |
| Roseburia sp. AM59-24XD (HG3A.0391) | ODI | -0.055 | 0.03 | 0.035 | 1558 | -0.054 | 0.02 | 0.032 | 1681 | 0.969 | 0.996 |
