# Supplementary material for: OSA Is Associated With the Human Gut Microbiota Composition and Functional Potential in the Population-Based Swedish CardioPulmonary bioImage Study
Source: Chest. 2023 Mar 15;164(2):503–16. doi: 10.1016/j.chest.2023.03.010 (PMC10410248; doi:10.1016/j.chest.2023.03.010)
Supplement: e-Table 12 [file mmc9.docx]

**e-Table 12. Enrichment for gut metabolic modules (GMM) in the associations of AHI, T90, or ODI with microbiota species.**

Enrichment analysis performed on ranked p-values of the partial Spearman’s correlations from the extended model, stratified by the direction of the Spearman's correlation coefficient. Adjustment for multiple testing using the Benjamini-Hochberg method and presented as q-values. Enrichments scores (NES) are normalized to mean enrichment score of random samples of the same size. Size is the number of species containing that module. AHI: apnea-hypopnea index; ODI: oxygen desaturation index; and T90: percentage of time with oxygen saturation below 90%.

| **exposure** | **direction** | **Gut metabolic**  **module** | **Name** | **Hierarchical Level 1** | **Hierarchical Level 2** | **NES** | **p-value** | **q-value** | **size** |
| --- | --- | --- | --- | --- | --- | --- | --- | --- | --- |
| T90 | positive | MF0022 | Galacturonate Degradation I | carbohydrate degradation | sugar acid degradation | 1.536 | 1.86E-05 | 0.001 | 174 |
| T90 | positive | MF0049 | Threonine Degradation I | amino acid degradation | polar, uncharged amino acid degradation | 1.520 | 1.33E-05 | 0.001 | 293 |
| T90 | positive | MF0058 | Lysine Degradation II | amino acid degradation | positively charged amino acid  degradation | 1.463 | 1.26E-04 | 0.004 | 173 |
| T90 | positive | MF0048 | Serine Degradation | amino acid degradation | polar, uncharged amino acid degradation | 1.472 | 3.58E-04 | 0.009 | 429 |
| T90 | positive | MF0065 | Bifidobacterium Shunt | central metabolism | energy metabolism | 1.394 | 8.20E-04 | 0.016 | 309 |
| T90 | positive | MF0050 | Threonine Degradation II | amino acid degradation | polar, uncharged amino acid degradation | 1.399 | 1.00E-03 | 0.020 | 153 |
| T90 | positive | MF0094 | Propionate Production II | organic acid metabolism | propionate metabolism | 1.470 | 1.00E-03 | 0.020 | 90 |
| T90 | positive | MF0085 | Urea Degradation | amines and polyamines degradation | urea degradation | 1.408 | 3.00E-03 | 0.032 | 92 |
| T90 | positive | MF0020 | Ribose Degradation | carbohydrate degradation | monosaccharide degradation | 1.430 | 4.00E-03 | 0.047 | 72 |
| ODI | negative | MF0016 | Fucose Degradation | organic acid metabolism | propionate metabolism | 1.385 | 8.47E-04 | 0.086 | 162 |
| T90 | positive | MF0039 | Methionine Degradation II | amino acid degradation | nonpolar, aliphatic amino acid  degradation | 1.375 | 1.00E-02 | 0.098 | 73 |
| AHI | positive | MF0049 | Threonine Degradation I | amino acid degradation | polar, uncharged amino acid degradation | 1.354 | 1.00E-03 | 0.136 | 294 |
| T90 | positive | MF0016 | Fucose Degradation | organic acid metabolism | propionate metabolism | 1.335 | 1.60E-02 | 0.146 | 84 |
| T90 | positive | MF0054 | Arginine Degradation Iv | amino acid degradation | positively charged amino acid degradation | 1.496 | 1.90E-02 | 0.159 | 20 |

|  |  |  |  |  | negatively charged amino acid |  |  |  |  |
| --- | --- | --- | --- | --- | --- | --- | --- | --- | --- |
| AHI | positive | MF0028 | Aspartate Degradation I | amino acid degradation | degradation | 1.439 | 5.00E-03 | 0.163 | 525 |
| AHI | positive | MF0044 | Cysteine Degradation I | amino acid degradation | polar, uncharged amino acid degradation | 1.476 | 5.00E-03 | 0.163 | 550 |
| T90 | positive | MF0060 | Glycerol Degradation I | lipid degradation | glycerol degradation | 1.277 | 2.60E-02 | 0.200 | 86 |
| ODI | negative | MF0006 | Lactose Degradation | carbohydrate degradation | disaccharide degradation | 1.260 | 1.10E-02 | 0.203 | 675 |
| ODI | negative | MF0025 | Tryptophan Degradation | amino acid degradation | aromatic amino acid  degradation | 1.341 | 1.10E-02 | 0.203 | 101 |
| ODI | negative | MF0026 | Tyrosine Degradation I | amino acid degradation | aromatic amino acid degradation | 1.553 | 1.20E-02 | 0.203 | 20 |
| ODI | negative | MF0044 | Cysteine Degradation I | amino acid degradation | polar, uncharged amino acid  degradation | 1.352 | 1.10E-02 | 0.203 | 930 |
| ODI | negative | MF0098 | Hydrogen Metabolism | gas metabolism | hydrogen metabolism | 1.255 | 1.10E-02 | 0.203 | 251 |
| T90 | positive | MF0015 | Fructose Degradation | carbohydrate degradation | monosaccharide degradation | 1.237 | 3.40E-02 | 0.243 | 309 |
| ODI | negative | MF0019 | Rhamnose Degradation | carbohydrate degradation | monosaccharide degradation | 1.238 | 1.90E-02 | 0.275 | 254 |
| T90 | positive | MF0011 | Sucrose Degradation II | carbohydrate degradation | disaccharide degradation | 1.255 | 4.60E-02 | 0.285 | 80 |
| T90 | positive | MF0027 | Tyrosine Degradation II | amino acid degradation | aromatic amino acid degradation | 1.547 | 0.060 | 0.285 | 5 |
| T90 | positive | MF0042 | Asparagine Degradation | amino acid degradation | polar, uncharged amino acid  degradation | 1.294 | 4.90E-02 | 0.285 | 49 |
| T90 | positive | MF0068 | Glycolysis (Pay-Off Phase) | central metabolism | energy metabolism | 1.230 | 5.50E-02 | 0.285 | 421 |
| T90 | positive | MF0074 | Pyruvate:formate Lyase | central metabolism | energy metabolism | 1.229 | 5.50E-02 | 0.285 | 407 |
| T90 | positive | MF0076 | 4-Aminobutyrate Degradation | amino acid degradation | positively charged amino acid degradation | 1.371 | 5.20E-02 | 0.285 | 26 |
| T90 | positive | MF0087 | Acetyl-Coa To Crotonyl-Coa | organic acid metabolism | butyrate metabolism | 1.319 | 0.060 | 0.285 | 31 |
| AHI | positive | MF0050 | Threonine Degradation II | amino acid degradation | polar, uncharged amino acid degradation | 1.283 | 1.90E-02 | 0.286 | 160 |
| AHI | positive | MF0054 | Arginine Degradation Iv | amino acid degradation | positively charged amino acid  degradation | 1.483 | 2.10E-02 | 0.286 | 22 |
| AHI | positive | MF0065 | Bifidobacterium Shunt | central metabolism | energy metabolism | 1.250 | 2.50E-02 | 0.286 | 327 |
| AHI | positive | MF0068 | Glycolysis (Pay-Off Phase) | central metabolism | energy metabolism | 1.305 | 1.40E-02 | 0.286 | 432 |
| AHI | positive | MF0090 | Ethanol Production I | alcohol metabolism | ethanol metabolism | 1.252 | 1.80E-02 | 0.286 | 206 |
| AHI | positive | MF0096 | Succinate Production | organic acid metabolism | succinate metabolism | 1.577 | 2.60E-02 | 0.286 | 10 |

| T90 | positive | MF0017 | Galactose Degradation | carbohydrate degradation | monosaccharide degradation | 1.201 | 0.070 | 0.318 | 347 |
| --- | --- | --- | --- | --- | --- | --- | --- | --- | --- |
| T90 | positive | MF0009 | Melibiose Degradation | carbohydrate degradation | disaccharide degradation | 1.181 | 0.077 | 0.321 | 317 |
| T90 | positive | MF0010 | Sucrose Degradation I | carbohydrate degradation | disaccharide degradation | 1.173 | 0.074 | 0.321 | 237 |
| AHI | positive | MF0098 | Hydrogen Metabolism | gas metabolism | hydrogen metabolism | 1.241 | 3.50E-02 | 0.346 | 124 |
| ODI | positive | MF0065 | Bifidobacterium Shunt | central metabolism | energy metabolism | 1.337 | 4.00E-03 | 0.353 | 314 |
| T90 | negative | MF0075 | Acetate To Acetyl-Coa | organic acid metabolism | acetate metabolism | 1.246 | 7.00E-03 | 0.358 | 311 |
| T90 | negative | MF0083 | Succinate Consumption | organic acid metabolism | butyrate metabolism | 1.609 | 1.10E-02 | 0.358 | 15 |
| T90 | negative | MF0090 | Ethanol Production I | alcohol metabolism | ethanol metabolism | 1.264 | 6.00E-03 | 0.358 | 324 |
| T90 | positive | MF0014 | Arabinose Degradation | carbohydrate degradation | monosaccharide degradation | 1.208 | 0.113 | 0.433 | 56 |
| T90 | positive | MF0028 | Aspartate Degradation I | amino acid degradation | negatively charged amino acid  degradation | 1.223 | 0.117 | 0.433 | 514 |
| T90 | positive | MF0045 | Cysteine Degradation II | amino acid degradation | polar, uncharged amino acid degradation | 1.211 | 0.114 | 0.433 | 57 |
| T90 | negative | MF0014 | Arabinose Degradation | carbohydrate degradation | monosaccharide degradation | 1.322 | 2.70E-02 | 0.439 | 70 |
| T90 | negative | MF0021 | Xylose Degradation | carbohydrate degradation | monosaccharide degradation | 1.269 | 2.20E-02 | 0.439 | 154 |
| T90 | negative | MF0098 | Hydrogen Metabolism | gas metabolism | hydrogen metabolism | 1.222 | 2.60E-02 | 0.439 | 251 |
| T90 | positive | MF0036 | Isoleucine Degradation | amino acid degradation | nonpolar, aliphatic amino acid degradation | 1.207 | 0.151 | 0.466 | 32 |
| T90 | positive | MF0044 | Cysteine Degradation I | amino acid degradation | polar, uncharged amino acid  degradation | 1.212 | 0.145 | 0.466 | 528 |
| T90 | positive | MF0051 | Arginine Degradation I | amino acid degradation | positively charged amino acid degradation | 1.136 | 0.143 | 0.466 | 294 |
| T90 | positive | MF0059 | Anaerobic Fatty Acid Beta-  Oxidation | lipid degradation | beta-oxidation | 1.289 | 0.154 | 0.466 | 12 |
| T90 | positive | MF0089 | Butyrate Production II | organic acid metabolism | butyrate metabolism | 1.195 | 0.154 | 0.466 | 41 |
| T90 | positive | MF0097 | Homoacetogenesis | gas metabolism | acetogenesis | 1.203 | 0.146 | 0.466 | 44 |
| ODI | positive | MF0034 | Alanine Degradation II | amino acid degradation | nonpolar, aliphatic amino acid degradation | 1.299 | 1.00E-02 | 0.476 | 228 |
| ODI | positive | MF0062 | Glycerol Degradation IIi | lipid degradation | glycerol degradation | 1.640 | 1.40E-02 | 0.476 | 9 |
| T90 | positive | MF0002 | Fructan Degradation | carbohydrate degradation | polysaccharide degradation | 1.126 | 0.175 | 0.500 | 341 |
| T90 | positive | MF0047 | Glutamine Degradation II | amino acid degradation | polar, uncharged amino acid  degradation | 1.123 | 0.174 | 0.500 | 332 |

| T90 | positive | MF0029 | Aspartate Degradation II | amino acid degradation | negatively charged amino acid  degradation | 1.119 | 0.186 | 0.510 | 157 |
| --- | --- | --- | --- | --- | --- | --- | --- | --- | --- |
| T90 | positive | MF0041 | Valine Degradation I | amino acid degradation | nonpolar, aliphatic amino acid  degradation | 1.254 | 0.199 | 0.510 | 9 |
| T90 | positive | MF0062 | Glycerol Degradation IIi | lipid degradation | glycerol degradation | 1.278 | 0.198 | 0.510 | 8 |
| T90 | positive | MF0079 | Lactate Consumption I | organic acid metabolism | lactate metabolism | 1.161 | 0.195 | 0.510 | 48 |
| AHI | negative | MF0039 | Methionine Degradation II | amino acid degradation | nonpolar, aliphatic amino acid degradation | 1.321 | 1.10E-02 | 0.520 | 92 |
| AHI | negative | MF0086 | Acetyl-Coa To Acetate | organic acid metabolism | acetate metabolism | 1.443 | 6.00E-03 | 0.520 | 904 |
| AHI | negative | MF0098 | Hydrogen Metabolism | gas metabolism | hydrogen metabolism | 1.249 | 1.60E-02 | 0.520 | 239 |
| T90 | positive | MF0052 | Arginine Degradation II | amino acid degradation | positively charged amino acid  degradation | 1.232 | 0.211 | 0.527 | 10 |
| T90 | positive | MF0024 | Phenylalanine Degradation | amino acid degradation | aromatic amino acid degradation | 1.217 | 0.251 | 0.570 | 7 |
| T90 | positive | MF0026 | Tyrosine Degradation I | amino acid degradation | aromatic amino acid  degradation | 1.159 | 0.246 | 0.570 | 23 |
| T90 | positive | MF0034 | Alanine Degradation II | amino acid degradation | nonpolar, aliphatic amino acid degradation | 1.087 | 0.248 | 0.570 | 234 |
| T90 | positive | MF0073 | Pyruvate:ferredoxin  Oxidoreductase | central metabolism | energy metabolism | 1.108 | 0.246 | 0.570 | 454 |
| AHI | positive | MF0015 | Fructose Degradation | carbohydrate degradation | monosaccharide degradation | 1.138 | 0.141 | 0.593 | 309 |
| AHI | positive | MF0017 | Galactose Degradation | carbohydrate degradation | monosaccharide degradation | 1.141 | 0.139 | 0.593 | 359 |
| AHI | positive | MF0022 | Galacturonate Degradation I | carbohydrate degradation | sugar acid degradation | 1.173 | 0.099 | 0.593 | 178 |
| AHI | positive | MF0030 | Glutamate Degradation I | amino acid degradation | negatively charged amino acid  degradation | 1.520 | 0.137 | 0.593 | 2 |
| AHI | positive | MF0034 | Alanine Degradation II | amino acid degradation | nonpolar, aliphatic amino acid degradation | 1.142 | 0.125 | 0.593 | 235 |
| AHI | positive | MF0045 | Cysteine Degradation II | amino acid degradation | polar, uncharged amino acid  degradation | 1.184 | 0.133 | 0.593 | 64 |
| AHI | positive | MF0048 | Serine Degradation | amino acid degradation | polar, uncharged amino acid degradation | 1.192 | 0.081 | 0.593 | 442 |
| AHI | positive | MF0051 | Arginine Degradation I | amino acid degradation | positively charged amino acid  degradation | 1.151 | 0.106 | 0.593 | 288 |

| AHI | positive | MF0062 | Glycerol Degradation IIi | lipid degradation | glycerol degradation | 1.376 | 0.103 | 0.593 | 10 |
| --- | --- | --- | --- | --- | --- | --- | --- | --- | --- |
| AHI | positive | MF0071 | Pentose Phosphate Pathway  (Non-Oxidative Branch) | central metabolism | energy metabolism | 1.281 | 0.079 | 0.593 | 548 |
| AHI | positive | MF0073 | Pyruvate:ferredoxin Oxidoreductase | central metabolism | energy metabolism | 1.181 | 0.107 | 0.593 | 461 |
| AHI | positive | MF0074 | Pyruvate:formate Lyase | central metabolism | energy metabolism | 1.139 | 0.144 | 0.593 | 423 |
| AHI | positive | MF0094 | Propionate Production II | organic acid metabolism | propionate metabolism | 1.197 | 0.080 | 0.593 | 103 |
| AHI | positive | MF0102 | Sulfate Reduction  (Dissimilatory) | gas metabolism | sulfate metabolism | 1.548 | 0.120 | 0.593 | 2 |
| T90 | negative | MF0073 | Pyruvate:ferredoxin Oxidoreductase | central metabolism | energy metabolism | 1.202 | 4.30E-02 | 0.608 | 783 |
| T90 | positive | MF0043 | Cysteine Biosynthesis/Homocysteine Degradation | amino acid degradation | polar, uncharged amino acid degradation | 1.109 | 0.275 | 0.611 | 44 |
| T90 | positive | MF0046 | Glutamine Degradation I | amino acid degradation | polar, uncharged amino acid degradation | 1.088 | 0.292 | 0.613 | 109 |
| T90 | positive | MF0053 | Arginine Degradation IIi | amino acid degradation | positively charged amino acid  degradation | 1.249 | 0.303 | 0.613 | 2 |
| T90 | positive | MF0055 | Arginine Degradation V | amino acid degradation | positively charged amino acid degradation | 1.076 | 0.313 | 0.613 | 77 |
| T90 | positive | MF0067 | Glycolysis (Preparatory  Phase) | central metabolism | energy metabolism | 1.083 | 0.305 | 0.613 | 508 |
| T90 | positive | MF0069 | Nadh:ferredoxin Oxidoreductase | gas metabolism | hydrogen metabolism | 1.142 | 0.305 | 0.613 | 10 |
| T90 | positive | MF0082 | Putrescine Degradation | amines and polyamines  degradation | biogenic amine degradation | 1.144 | 0.311 | 0.613 | 7 |
| T90 | negative | MF0001 | Arabinoxylan Degradation | carbohydrate degradation | polysaccharide degradation | 1.144 | 0.082 | 0.615 | 559 |
| T90 | negative | MF0006 | Lactose Degradation | carbohydrate degradation | disaccharide degradation | 1.145 | 0.087 | 0.615 | 658 |
| T90 | negative | MF0026 | Tyrosine Degradation I | amino acid degradation | aromatic amino acid degradation | 1.372 | 0.068 | 0.615 | 17 |
| T90 | negative | MF0038 | Methionine Degradation I | amino acid degradation | nonpolar, aliphatic amino acid  degradation | 1.191 | 0.072 | 0.615 | 137 |

T90

negative

MF0057 Lysine Degradation I

amino acid degradation

positively charged amino acid degradation

1.287

0.060

0.615

46

T90 negative MF0077 Formate Conversion organic acid metabolism formate metabolism 1.394 0.076 0.615 10

T90 negative MF0103 Mucin Degradation glycoprotein degradation mucus degradation 1.154 0.067 0.615 341

T90 positive MF0007

Lactose And Galactose

Degradation carbohydrate degradation disaccharide degradation 1.118 0.332 0.638 13

AHI

positive

MF0027 Tyrosine Degradation II

amino acid degradation

aromatic amino acid degradation

1.320

0.172

0.645

6

AHI positive MF0093 Propionate Production I organic acid metabolism propionate metabolism 1.333 0.176 0.645 4

AHI positive MF0097 Homoacetogenesis gas metabolism acetogenesis 1.188 0.163 0.645 42

T90 positive MF0070

T90 positive MF0066 Entner-Doudoroff Pathway central metabolism energy metabolism 1.051 0.367 0.679 77

Pentose Phosphate Pathway

(Oxidative Phase) central metabolism energy metabolism 1.053 0.348 0.656 87

T90 positive MF0037 Leucine Degradation amino acid degradation

ODI positive MF0002 Fructan Degradation carbohydrate degradation polysaccharide degradation 1.104 0.205 0.700 340

nonpolar, aliphatic amino acid

degradation 1.246 0.384 0.697 1

ODI positive MF0005 Starch Degradation carbohydrate degradation polysaccharide degradation 1.103 0.211 0.700 197

ODI

positive

Lactose And Galactose MF0007 Degradation

carbohydrate degradation disaccharide degradation

1.225

0.198

0.700

15

ODI positive MF0010 Sucrose Degradation I carbohydrate degradation disaccharide degradation 1.089 0.232 0.700 241

ODI positive MF0011 Sucrose Degradation II carbohydrate degradation disaccharide degradation 1.154 0.153 0.700 77

ODI positive MF0015 Fructose Degradation carbohydrate degradation monosaccharide degradation 1.192 0.067 0.700 304

ODI positive MF0017 Galactose Degradation carbohydrate degradation monosaccharide degradation 1.097 0.225 0.700 347

ODI positive MF0020 Ribose Degradation carbohydrate degradation monosaccharide degradation 1.231 0.074 0.700 74

ODI

positive

MF0028 Aspartate Degradation I

amino acid degradation

negatively charged amino acid degradation

1.247

0.083

0.700 496

ODI positive MF0029 Aspartate Degradation II amino acid degradation

ODI

positive

MF0033 Alanine Degradation I

amino acid degradation

nonpolar, aliphatic amino acid degradation 1.124

0.271

0.700

34

negatively charged amino acid

degradation 1.070 0.288 0.700 164

ODI positive MF0037 Leucine Degradation amino acid degradation

nonpolar, aliphatic amino acid

degradation 1.309 0.276 0.700 2

| ODI | positive | MF0041 | Valine Degradation I | amino acid degradation | nonpolar, aliphatic amino acid  degradation | 1.278 | 0.179 | 0.700 | 10 |
| --- | --- | --- | --- | --- | --- | --- | --- | --- | --- |
| ODI | positive | MF0046 | Glutamine Degradation I | amino acid degradation | polar, uncharged amino acid  degradation | 1.093 | 0.269 | 0.700 | 108 |
| ODI | positive | MF0048 | Serine Degradation | amino acid degradation | polar, uncharged amino acid degradation | 1.187 | 0.093 | 0.700 | 419 |
| ODI | positive | MF0049 | Threonine Degradation I | amino acid degradation | polar, uncharged amino acid  degradation | 1.159 | 0.098 | 0.700 | 294 |
| ODI | positive | MF0050 | Threonine Degradation II | amino acid degradation | polar, uncharged amino acid degradation | 1.146 | 0.128 | 0.700 | 152 |
| ODI | positive | MF0051 | Arginine Degradation I | amino acid degradation | positively charged amino acid  degradation | 1.127 | 0.145 | 0.700 | 287 |
| ODI | positive | MF0054 | Arginine Degradation Iv | amino acid degradation | positively charged amino acid degradation | 1.292 | 0.103 | 0.700 | 22 |
| ODI | positive | MF0055 | Arginine Degradation V | amino acid degradation | positively charged amino acid  degradation | 1.087 | 0.287 | 0.700 | 71 |
| ODI | positive | MF0057 | Lysine Degradation I | amino acid degradation | positively charged amino acid degradation | 1.138 | 0.290 | 0.700 | 18 |
| ODI | positive | MF0058 | Lysine Degradation II | amino acid degradation | positively charged amino acid  degradation | 1.220 | 4.90E-02 | 0.700 | 158 |
| ODI | positive | MF0067 | Glycolysis (Preparatory Phase) | central metabolism | energy metabolism | 1.111 | 0.260 | 0.700 | 487 |
| ODI | positive | MF0068 | Glycolysis (Pay-Off Phase) | central metabolism | energy metabolism | 1.261 | 2.90E-02 | 0.700 | 418 |
| ODI | positive | MF0071 | Pentose Phosphate Pathway (Non-Oxidative Branch) | central metabolism | energy metabolism | 1.126 | 0.262 | 0.700 | 512 |
| ODI | positive | MF0072 | Pyruvate Dehydrogenase  Complex | central metabolism | energy metabolism | 1.388 | 0.173 | 0.700 | 3 |
| ODI | positive | MF0074 | Pyruvate:formate Lyase | central metabolism | energy metabolism | 1.098 | 0.245 | 0.700 | 405 |
| ODI | positive | MF0075 | Acetate To Acetyl-Coa | organic acid metabolism | acetate metabolism | 1.095 | 0.219 | 0.700 | 161 |
| ODI | positive | MF0079 | Lactate Consumption I | organic acid metabolism | lactate metabolism | 1.261 | 0.072 | 0.700 | 47 |
| ODI | positive | MF0085 | Urea Degradation | amines and polyamines  degradation | urea degradation | 1.146 | 0.160 | 0.700 | 101 |
| ODI | positive | MF0086 | Acetyl-Coa To Acetate | organic acid metabolism | acetate metabolism | 1.188 | 0.158 | 0.700 | 504 |

| ODI | positive | MF0088 | Butyrate Production I | organic acid metabolism | butyrate metabolism | 1.155 | 0.148 | 0.700 | 74 |
| --- | --- | --- | --- | --- | --- | --- | --- | --- | --- |
| ODI | positive | MF0092 | Lactate Production | organic acid metabolism | lactate metabolism | 1.165 | 0.116 | 0.700 | 409 |
| ODI | positive | MF0094 | Propionate Production II | organic acid metabolism | propionate metabolism | 1.133 | 0.177 | 0.700 | 95 |
| ODI | positive | MF0096 | Succinate Production | organic acid metabolism | succinate metabolism | 1.201 | 0.260 | 0.700 | 8 |
| ODI | positive | MF0097 | Homoacetogenesis | gas metabolism | acetogenesis | 1.208 | 0.133 | 0.700 | 42 |
| ODI | positive | MF0101 | Nitrate Reduction (Dissimilatory) | inorganic nutrient metabolism | nitrogen | 1.176 | 0.194 | 0.700 | 30 |
| ODI | positive | MF0102 | Sulfate Reduction  (Dissimilatory) | gas metabolism | sulfate metabolism | 1.357 | 0.198 | 0.700 | 3 |
| AHI | positive | MF0007 | Lactose And Galactose Degradation | carbohydrate degradation | disaccharide degradation | 1.222 | 0.218 | 0.705 | 12 |
| AHI | positive | MF0020 | Ribose Degradation | carbohydrate degradation | monosaccharide degradation | 1.117 | 0.220 | 0.705 | 73 |
| AHI | positive | MF0029 | Aspartate Degradation II | amino acid degradation | negatively charged amino acid degradation | 1.104 | 0.221 | 0.705 | 163 |
| AHI | positive | MF0079 | Lactate Consumption I | organic acid metabolism | lactate metabolism | 1.134 | 0.213 | 0.705 | 50 |
| AHI | positive | MF0058 | Lysine Degradation II | amino acid degradation | positively charged amino acid degradation | 1.099 | 0.231 | 0.714 | 164 |
| AHI | positive | MF0083 | Succinate Consumption | organic acid metabolism | butyrate metabolism | 1.230 | 0.239 | 0.716 | 6 |
| AHI | negative | MF0073 | Pyruvate:ferredoxin Oxidoreductase | central metabolism | energy metabolism | 1.248 | 2.90E-02 | 0.717 | 776 |
| AHI | positive | MF0047 | Glutamine Degradation II | amino acid degradation | polar, uncharged amino acid  degradation | 1.084 | 0.248 | 0.721 | 335 |
| ODI | positive | MF0022 | Galacturonate Degradation I | carbohydrate degradation | sugar acid degradation | 1.057 | 0.322 | 0.724 | 167 |
| ODI | positive | MF0081 | Methanol Conversion | gas metabolism | methanogenesis | 1.405 | 0.316 | 0.724 | 1 |
| ODI | positive | MF0099 | Methanogenesis - Methyl- Com | gas metabolism | methanogenesis | 1.405 | 0.316 | 0.724 | 1 |
| ODI | positive | MF0047 | Glutamine Degradation II | amino acid degradation | polar, uncharged amino acid  degradation | 1.049 | 0.330 | 0.725 | 325 |
| T90 | positive | MF0019 | Rhamnose Degradation | carbohydrate degradation | monosaccharide degradation | 1.032 | 0.409 | 0.730 | 148 |
| AHI | positive | MF0067 | Glycolysis (Preparatory  Phase) | central metabolism | energy metabolism | 1.111 | 0.262 | 0.740 | 520 |
| T90 | positive | MF0001 | Arabinoxylan Degradation | carbohydrate degradation | polysaccharide degradation | 1.021 | 0.431 | 0.742 | 331 |
| T90 | positive | MF0012 | Trehalose Degradation | carbohydrate degradation | disaccharide degradation | 1.015 | 0.468 | 0.742 | 185 |

| T90 | positive | MF0013 | Allose Degradation | carbohydrate degradation | monosaccharide degradation | 1.048 | 0.432 | 0.742 | 11 |
| --- | --- | --- | --- | --- | --- | --- | --- | --- | --- |
| T90 | positive | MF0063 | Glyoxylate Bypass | lipid degradation | glyoxylate bypass | 1.031 | 0.461 | 0.742 | 6 |
| T90 | positive | MF0072 | Pyruvate Dehydrogenase Complex | central metabolism | energy metabolism | 1.051 | 0.444 | 0.742 | 4 |
| T90 | positive | MF0083 | Succinate Consumption | organic acid metabolism | butyrate metabolism | 1.030 | 0.465 | 0.742 | 5 |
| T90 | positive | MF0093 | Propionate Production I | organic acid metabolism | propionate metabolism | 1.059 | 0.459 | 0.742 | 3 |
| ODI | positive | MF0004 | Pectine Degradation II | carbohydrate degradation | polysaccharide degradation | 1.074 | 0.349 | 0.750 | 37 |
| AHI | negative | MF0026 | Tyrosine Degradation I | amino acid degradation | aromatic amino acid degradation | 1.474 | 3.80E-02 | 0.752 | 18 |
| AHI | positive | MF0039 | Methionine Degradation II | amino acid degradation | nonpolar, aliphatic amino acid  degradation | 1.080 | 0.297 | 0.753 | 76 |
| AHI | positive | MF0046 | Glutamine Degradation I | amino acid degradation | polar, uncharged amino acid degradation | 1.085 | 0.294 | 0.753 | 103 |
| AHI | positive | MF0085 | Urea Degradation | amines and polyamines  degradation | urea degradation | 1.087 | 0.289 | 0.753 | 103 |
| AHI | positive | MF0101 | Nitrate Reduction (Dissimilatory) | inorganic nutrient metabolism | nitrogen | 1.126 | 0.274 | 0.753 | 31 |
| ODI | positive | MF0030 | Glutamate Degradation I | amino acid degradation | negatively charged amino acid  degradation | 1.191 | 0.371 | 0.764 | 2 |
| ODI | positive | MF0053 | Arginine Degradation IIi | amino acid degradation | positively charged amino acid degradation | 1.291 | 0.368 | 0.764 | 1 |
| AHI | positive | MF0002 | Fructan Degradation | carbohydrate degradation | polysaccharide degradation | 1.053 | 0.321 | 0.784 | 353 |
| AHI | positive | MF0072 | Pyruvate Dehydrogenase Complex | central metabolism | energy metabolism | 1.195 | 0.325 | 0.784 | 3 |
| AHI | positive | MF0042 | Asparagine Degradation | amino acid degradation | polar, uncharged amino acid  degradation | 1.075 | 0.334 | 0.786 | 60 |
| T90 | positive | MF0003 | Pectin Degradation I | carbohydrate degradation | polysaccharide degradation | 0.988 | 0.511 | 0.788 | 21 |
| T90 | positive | MF0032 | Glutamate Degradation IIi | amino acid degradation | negatively charged amino acid  degradation | 0.973 | 0.536 | 0.788 | 17 |
| T90 | positive | MF0038 | Methionine Degradation I | amino acid degradation | nonpolar, aliphatic amino acid degradation | 0.981 | 0.551 | 0.788 | 67 |
| T90 | positive | MF0081 | Methanol Conversion | gas metabolism | methanogenesis | 1.001 | 0.530 | 0.788 | 2 |
| T90 | positive | MF0090 | Ethanol Production I | alcohol metabolism | ethanol metabolism | 0.983 | 0.551 | 0.788 | 203 |

T90 positive MF0091 Ethanol Production II alcohol metabolism ethanol metabolism 0.991 0.515 0.788 1

T90

positive

Methanogenesis - Methyl-

MF0099 Com

gas metabolism

methanogenesis

1.001

0.530

0.788

2

ODI positive MF0006 Lactose Degradation carbohydrate degradation disaccharide degradation 1.029 0.424 0.795 388

ODI positive MF0019 Rhamnose Degradation carbohydrate degradation monosaccharide degradation 1.036 0.398 0.795 138

ODI positive MF0043

ODI

positive

MF0045 Cysteine Degradation II

amino acid degradation

polar, uncharged amino acid degradation

1.025

0.426

0.795

60

Cysteine Biosynthesis/Homocysteine

Degradation amino acid degradation

polar, uncharged amino acid

degradation 1.028 0.409 0.795 53

ODI positive MF0090 Ethanol Production I alcohol metabolism ethanol metabolism 1.022 0.424 0.795 194

ODI negative MF0014 Arabinose Degradation carbohydrate degradation monosaccharide degradation 1.231 0.072 0.807 75

ODI negative MF0086 Acetyl-Coa To Acetate organic acid metabolism acetate metabolism 1.276 0.065 0.807 943

AHI positive MF0086 Acetyl-Coa To Acetate organic acid metabolism acetate metabolism 1.069 0.353 0.812 543

ODI positive MF0013 Allose Degradation carbohydrate degradation monosaccharide degradation 1.032 0.446 0.817 10

AHI positive MF0088 Butyrate Production I organic acid metabolism butyrate metabolism 1.050 0.372 0.836 80

T90 positive MF0057 Lysine Degradation I amino acid degradation

T90 positive MF0092 Lactate Production organic acid metabolism lactate metabolism 0.965 0.595 0.837 434

positively charged amino acid

degradation 0.934 0.602 0.837 21

T90 positive MF0006 Lactose Degradation carbohydrate degradation disaccharide degradation 0.952 0.625 0.857 405

T90

positive

Succinate Conversion To MF0084 Propionate

organic acid metabolism succinate metabolism

0.887

0.635

0.859

4

AHI positive MF0011 Sucrose Degradation II carbohydrate degradation disaccharide degradation 1.037 0.400 0.860 85

ODI positive MF0012 Trehalose Degradation carbohydrate degradation disaccharide degradation 1.003 0.484 0.860 184

AHI positive MF0016 Fucose Degradation organic acid metabolism propionate metabolism 1.044 0.397 0.860 77

ODI

positive

MF0042 Asparagine Degradation

amino acid degradation

polar, uncharged amino acid degradation

1.000

0.487

0.860

59

ODI positive MF0032 Glutamate Degradation IIi amino acid degradation

T90 positive MF0004 Pectine Degradation II carbohydrate degradation polysaccharide degradation 0.877 0.745 0.870 39

negatively charged amino acid

degradation 0.983 0.500 0.868 16

T90 positive MF0005 Starch Degradation carbohydrate degradation polysaccharide degradation 0.911 0.750 0.870 209

T90 positive MF0018 Mannose Degradation carbohydrate degradation monosaccharide degradation 0.912 0.756 0.870 330

| T90 | positive | MF0021 | Xylose Degradation | carbohydrate degradation | monosaccharide degradation | 0.937 | 0.670 | 0.870 | 101 |
| --- | --- | --- | --- | --- | --- | --- | --- | --- | --- |
| T90 | positive | MF0030 | Glutamate Degradation I | amino acid degradation | negatively charged amino acid degradation | 0.558 | 0.715 | 0.870 | 1 |
| T90 | positive | MF0033 | Alanine Degradation I | amino acid degradation | nonpolar, aliphatic amino acid  degradation | 0.855 | 0.765 | 0.870 | 35 |
| T90 | positive | MF0035 | Glycine Degradation | amino acid degradation | nonpolar, aliphatic amino acid degradation | 0.900 | 0.745 | 0.870 | 116 |
| T90 | positive | MF0071 | Pentose Phosphate Pathway  (Non-Oxidative Branch) | central metabolism | energy metabolism | 0.885 | 0.713 | 0.870 | 531 |
| T90 | positive | MF0077 | Formate Conversion | organic acid metabolism | formate metabolism | 0.808 | 0.736 | 0.870 | 7 |
| T90 | positive | MF0078 | Lactaldehyde Degradation | organic acid metabolism | propionate metabolism | 0.923 | 0.718 | 0.870 | 222 |
| T90 | positive | MF0080 | Lactate Consumption II | organic acid metabolism | propionate metabolism | 0.794 | 0.726 | 0.870 | 6 |
| T90 | positive | MF0086 | Acetyl-Coa To Acetate | organic acid metabolism | acetate metabolism | 0.886 | 0.727 | 0.870 | 526 |
| T90 | positive | MF0096 | Succinate Production | organic acid metabolism | succinate metabolism | 0.775 | 0.763 | 0.870 | 7 |
| T90 | positive | MF0098 | Hydrogen Metabolism | gas metabolism | hydrogen metabolism | 0.932 | 0.669 | 0.870 | 112 |
| ODI | positive | MF0091 | Ethanol Production II | alcohol metabolism | ethanol metabolism | 0.962 | 0.513 | 0.876 | 1 |
| ODI | negative | MF0050 | Threonine Degradation II | amino acid degradation | polar, uncharged amino acid  degradation | 1.154 | 0.087 | 0.878 | 216 |
| ODI | positive | MF0003 | Pectin Degradation I | carbohydrate degradation | polysaccharide degradation | 0.960 | 0.541 | 0.879 | 18 |
| ODI | positive | MF0016 | Fucose Degradation | organic acid metabolism | propionate metabolism | 0.984 | 0.538 | 0.879 | 79 |
| ODI | positive | MF0070 | Pentose Phosphate Pathway (Oxidative Phase) | central metabolism | energy metabolism | 0.983 | 0.534 | 0.879 | 94 |
| T90 | positive | MF0101 | Nitrate Reduction  (Dissimilatory) | inorganic nutrient  metabolism | nitrogen | 0.829 | 0.784 | 0.881 | 29 |
| ODI | positive | MF0014 | Arabinose Degradation | carbohydrate degradation | monosaccharide degradation | 0.959 | 0.569 | 0.884 | 51 |
| T90 | negative | MF0025 | Tryptophan Degradation | amino acid degradation | aromatic amino acid  degradation | 1.150 | 0.143 | 0.884 | 98 |
| ODI | positive | MF0036 | Isoleucine Degradation | amino acid degradation | nonpolar, aliphatic amino acid degradation | 0.960 | 0.555 | 0.884 | 32 |
| ODI | positive | MF0056 | Histidine Degradation | amino acid degradation | positively charged amino acid  degradation | 0.968 | 0.571 | 0.884 | 81 |
| T90 | negative | MF0067 | Glycolysis (Preparatory Phase) | central metabolism | energy metabolism | 1.151 | 0.135 | 0.884 | 846 |

ODI positive MF0009 Melibiose Degradation carbohydrate degradation disaccharide degradation 0.965 0.600 0.901 321

ODI positive MF0083 Succinate Consumption organic acid metabolism butyrate metabolism 0.916 0.592 0.901 5

T90 positive MF0031 Glutamate Degradation II amino acid degradation

negatively charged amino acid

degradation 0.843 0.813 0.904 52

T90 negative MF0011 Sucrose Degradation II carbohydrate degradation disaccharide degradation 1.139 0.157 0.913 107

AHI positive MF0019 Rhamnose Degradation carbohydrate degradation monosaccharide degradation 1.022 0.434 0.913 147

ODI positive MF0001 Arabinoxylan Degradation carbohydrate degradation polysaccharide degradation 0.939 0.693 0.922 319

ODI positive MF0018 Mannose Degradation carbohydrate degradation monosaccharide degradation 0.958 0.632 0.922 301

ODI

positive

MF0025 Tryptophan Degradation

amino acid degradation

aromatic amino acid degradation

0.931

0.647

0.922

54

ODI positive MF0026 Tyrosine Degradation I amino acid degradation

aromatic amino acid

degradation 0.858 0.698 0.922 20

ODI

positive

MF0031 Glutamate Degradation II

amino acid degradation

negatively charged amino acid degradation

0.922

0.677

0.922

59

ODI positive MF0039 Methionine Degradation II amino acid degradation

nonpolar, aliphatic amino acid

degradation 0.935 0.661 0.922 69

ODI

positive

MF0040 Proline Degradation

amino acid degradation

nonpolar, aliphatic amino acid degradation 0.923

0.687

0.922

68

ODI positive MF0044 Cysteine Degradation I amino acid degradation

polar, uncharged amino acid

degradation 0.895 0.694 0.922 524

ODI positive MF0087 Acetyl-Coa To Crotonyl-Coa organic acid metabolism butyrate metabolism 0.910 0.670 0.922 34

ODI negative MF0001 Arabinoxylan Degradation carbohydrate degradation polysaccharide degradation 1.110 0.128 0.933 571

ODI negative MF0009 Melibiose Degradation carbohydrate degradation disaccharide degradation 1.113 0.123 0.933 549

ODI negative MF0017 Galactose Degradation carbohydrate degradation monosaccharide degradation 1.098 0.147 0.933 523

ODI negative MF0021 Xylose Degradation carbohydrate degradation monosaccharide degradation 1.126 0.133 0.933 160

ODI negative MF0039 Methionine Degradation II amino acid degradation

nonpolar, aliphatic amino acid

degradation 1.160 0.122 0.933 99

ODI negative MF0083 Succinate Consumption organic acid metabolism butyrate metabolism 1.268 0.148 0.933 15

ODI positive MF0024 Phenylalanine Degradation amino acid degradation

ODI positive MF0060 Glycerol Degradation I lipid degradation glycerol degradation 0.909 0.733 0.934 81

aromatic amino acid

degradation 0.800 0.725 0.934 7

ODI positive MF0077 Formate Conversion organic acid metabolism formate metabolism 0.804 0.736 0.934 8

ODI positive MF0008 Maltose Degradation carbohydrate degradation disaccharide degradation 0.850 0.838 0.938 75

|  | | | | | aromatic amino acid |  | | | |
| --- | --- | --- | --- | --- | --- | --- | --- | --- | --- |
| ODI | positive | MF0027 | Tyrosine Degradation II | amino acid degradation | degradation | 0.707 | 0.811 | 0.938 | 6 |
| ODI | positive | MF0063 | Glyoxylate Bypass | lipid degradation | glyoxylate bypass | 0.723 | 0.820 | 0.938 | 8 |
| ODI | positive | MF0066 | Entner-Doudoroff Pathway | central metabolism | energy metabolism | 0.892 | 0.758 | 0.938 | 84 |
| ODI | positive | MF0069 | Nadh:ferredoxin Oxidoreductase | gas metabolism | hydrogen metabolism | 0.761 | 0.784 | 0.938 | 9 |
| ODI | positive | MF0076 | 4-Aminobutyrate  Degradation | amino acid degradation | positively charged amino acid  degradation | 0.786 | 0.843 | 0.938 | 27 |
| ODI | positive | MF0078 | Lactaldehyde Degradation | organic acid metabolism | propionate metabolism | 0.899 | 0.786 | 0.938 | 215 |
| ODI | positive | MF0080 | Lactate Consumption II | organic acid metabolism | propionate metabolism | 0.694 | 0.824 | 0.938 | 6 |
| ODI | positive | MF0082 | Putrescine Degradation | amines and polyamines degradation | biogenic amine degradation | 0.761 | 0.765 | 0.938 | 7 |
| ODI | positive | MF0089 | Butyrate Production II | organic acid metabolism | butyrate metabolism | 0.821 | 0.832 | 0.938 | 40 |
| ODI | positive | MF0098 | Hydrogen Metabolism | gas metabolism | hydrogen metabolism | 0.891 | 0.772 | 0.938 | 112 |
| ODI | negative | MF0090 | Ethanol Production I | alcohol metabolism | ethanol metabolism | 1.098 | 0.159 | 0.944 | 333 |
| ODI | positive | MF0084 | Succinate Conversion To Propionate | organic acid metabolism | succinate metabolism | 0.502 | 0.873 | 0.960 | 3 |
| ODI | positive | MF0035 | Glycine Degradation | amino acid degradation | nonpolar, aliphatic amino acid  degradation | 0.812 | 0.907 | 0.969 | 106 |
| ODI | positive | MF0073 | Pyruvate:ferredoxin Oxidoreductase | central metabolism | energy metabolism | 0.796 | 0.910 | 0.969 | 430 |
| ODI | positive | MF0093 | Propionate Production I | organic acid metabolism | propionate metabolism | 0.487 | 0.909 | 0.969 | 4 |
| ODI | positive | MF0038 | Methionine Degradation I | amino acid degradation | nonpolar, aliphatic amino acid degradation | 0.738 | 0.947 | 0.977 | 61 |
| ODI | positive | MF0059 | Anaerobic Fatty Acid Beta-  Oxidation | lipid degradation | beta-oxidation | 0.582 | 0.939 | 0.977 | 12 |
| ODI | positive | MF0103 | Mucin Degradation | glycoprotein degradation | mucus degradation | 0.810 | 0.936 | 0.977 | 167 |
| ODI | positive | MF0021 | Xylose Degradation | carbohydrate degradation | monosaccharide degradation | 0.741 | 0.967 | 0.978 | 95 |
| ODI | positive | MF0052 | Arginine Degradation II | amino acid degradation | positively charged amino acid degradation | 0.532 | 0.968 | 0.978 | 11 |
| AHI | positive | MF0001 | Arabinoxylan Degradation | carbohydrate degradation | polysaccharide degradation | 0.927 | 0.735 | 0.992 | 330 |
| AHI | positive | MF0003 | Pectin Degradation I | carbohydrate degradation | polysaccharide degradation | 0.810 | 0.769 | 0.992 | 19 |
| AHI | positive | MF0004 | Pectine Degradation II | carbohydrate degradation | polysaccharide degradation | 0.808 | 0.835 | 0.992 | 40 |

AHI positive MF0005 Starch Degradation carbohydrate degradation polysaccharide degradation 0.754 0.983 0.992 226

AHI positive MF0006 Lactose Degradation carbohydrate degradation disaccharide degradation 0.860 0.862 0.992 410

AHI positive MF0008 Maltose Degradation carbohydrate degradation disaccharide degradation 0.787 0.924 0.992 80

AHI positive MF0009 Melibiose Degradation carbohydrate degradation disaccharide degradation 0.914 0.764 0.992 338

AHI positive MF0010 Sucrose Degradation I carbohydrate degradation disaccharide degradation 0.977 0.560 0.992 245

AHI positive MF0012 Trehalose Degradation carbohydrate degradation disaccharide degradation 0.869 0.844 0.992 188

AHI positive MF0013 Allose Degradation carbohydrate degradation monosaccharide degradation 0.664 0.880 0.992 11

AHI positive MF0014 Arabinose Degradation carbohydrate degradation monosaccharide degradation 0.671 0.982 0.992 59

AHI positive MF0018 Mannose Degradation carbohydrate degradation monosaccharide degradation 0.810 0.939 0.992 327

AHI positive MF0021 Xylose Degradation carbohydrate degradation monosaccharide degradation 0.875 0.801 0.992 99

AHI

positive

MF0024 Phenylalanine Degradation amino acid degradation

aromatic amino acid degradation

0.444

0.972

0.992

9

AHI positive MF0025 Tryptophan Degradation amino acid degradation

aromatic amino acid

degradation 0.863 0.794 0.992 61

AHI

positive

MF0026 Tyrosine Degradation I

amino acid degradation

aromatic amino acid degradation

0.907

0.637

0.992

22

AHI positive MF0031 Glutamate Degradation II amino acid degradation

negatively charged amino acid

degradation 0.868 0.780 0.992 60

AHI

positive

MF0032 Glutamate Degradation IIi

amino acid degradation

negatively charged amino acid degradation

0.671

0.894

0.992

15

AHI positive MF0033 Alanine Degradation I amino acid degradation

nonpolar, aliphatic amino acid

degradation 0.631 0.967 0.992 33

AHI

positive

MF0035 Glycine Degradation

amino acid degradation

nonpolar, aliphatic amino acid degradation 0.993

0.521

0.992 111

AHI positive MF0036 Isoleucine Degradation amino acid degradation

nonpolar, aliphatic amino acid

degradation 1.004 0.500 0.992 36

AHI

positive

MF0037 Leucine Degradation

amino acid degradation

nonpolar, aliphatic amino acid degradation 0.972

0.539

0.992

2

AHI positive MF0038 Methionine Degradation I amino acid degradation

nonpolar, aliphatic amino acid

degradation 0.869 0.786 0.992 65

AHI

positive

MF0040 Proline Degradation

amino acid degradation

nonpolar, aliphatic amino acid degradation 0.844

0.840

0.992

78

| AHI | positive | MF0041 | Valine Degradation I | amino acid degradation | nonpolar, aliphatic amino acid  degradation | 0.680 | 0.853 | 0.992 | 10 |
| --- | --- | --- | --- | --- | --- | --- | --- | --- | --- |
|  |  |  | Cysteine Biosynthesis/Homocysteine |  | polar, uncharged amino acid |  |  |  |  |
| AHI | positive | MF0043 | Degradation | amino acid degradation | degradation | 0.975 | 0.566 | 0.992 | 53 |
| AHI | positive | MF0052 | Arginine Degradation II | amino acid degradation | positively charged amino acid  degradation | 0.682 | 0.880 | 0.992 | 13 |
| AHI | positive | MF0053 | Arginine Degradation IIi | amino acid degradation | positively charged amino acid degradation | 0.604 | 0.778 | 0.992 | 2 |
| AHI | positive | MF0055 | Arginine Degradation V | amino acid degradation | positively charged amino acid  degradation | 0.721 | 0.981 | 0.992 | 86 |
| AHI | positive | MF0056 | Histidine Degradation | amino acid degradation | positively charged amino acid degradation | 0.771 | 0.939 | 0.992 | 86 |
| AHI | positive | MF0057 | Lysine Degradation I | amino acid degradation | positively charged amino acid  degradation | 0.918 | 0.606 | 0.992 | 20 |
| AHI | positive | MF0059 | Anaerobic Fatty Acid Beta- Oxidation | lipid degradation | beta-oxidation | 0.719 | 0.849 | 0.992 | 15 |
| AHI | positive | MF0060 | Glycerol Degradation I | lipid degradation | glycerol degradation | 0.777 | 0.939 | 0.992 | 83 |
| AHI | positive | MF0061 | Glycerol Degradation II | lipid degradation | glycerol degradation | 0.615 | 0.958 | 0.992 | 20 |
| AHI | positive | MF0063 | Glyoxylate Bypass | lipid degradation | glyoxylate bypass | 0.679 | 0.865 | 0.992 | 9 |
| AHI | positive | MF0066 | Entner-Doudoroff Pathway | central metabolism | energy metabolism | 0.660 | 0.992 | 0.992 | 90 |
| AHI | positive | MF0069 | Nadh:ferredoxin  Oxidoreductase | gas metabolism | hydrogen metabolism | 0.490 | 0.979 | 0.992 | 13 |
| AHI | positive | MF0070 | Pentose Phosphate Pathway (Oxidative Phase) | central metabolism | energy metabolism | 0.915 | 0.711 | 0.992 | 98 |
| AHI | positive | MF0075 | Acetate To Acetyl-Coa | organic acid metabolism | acetate metabolism | 0.920 | 0.723 | 0.992 | 180 |
| AHI | positive | MF0076 | 4-Aminobutyrate Degradation | amino acid degradation | positively charged amino acid degradation | 0.951 | 0.589 | 0.992 | 30 |
| AHI | positive | MF0077 | Formate Conversion | organic acid metabolism | formate metabolism | 0.839 | 0.688 | 0.992 | 9 |
| AHI | positive | MF0078 | Lactaldehyde Degradation | organic acid metabolism | propionate metabolism | 0.999 | 0.494 | 0.992 | 233 |
| AHI | positive | MF0080 | Lactate Consumption II | organic acid metabolism | propionate metabolism | 0.881 | 0.640 | 0.992 | 6 |
| AHI | positive | MF0081 | Methanol Conversion | gas metabolism | methanogenesis | 0.731 | 0.707 | 0.992 | 2 |

AHI positive MF0082 Putrescine Degradation

AHI

positive

Succinate Conversion To MF0084 Propionate

organic acid metabolism succinate metabolism

0.904

0.621

0.992

4

amines and polyamines

degradation biogenic amine degradation 0.672 0.870 0.992 9

AHI positive MF0087 Acetyl-Coa To Crotonyl-Coa organic acid metabolism butyrate metabolism 0.921 0.637 0.992 37

T90 positive MF0088 Butyrate Production I organic acid metabolism butyrate metabolism 0.803 0.913 0.992 82

AHI positive MF0089 Butyrate Production II organic acid metabolism butyrate metabolism 0.968 0.567 0.992 42

AHI positive MF0091 Ethanol Production II alcohol metabolism ethanol metabolism 0.084 0.956 0.992 1

AHI positive MF0092 Lactate Production organic acid metabolism lactate metabolism 0.978 0.559 0.992 437

AHI

positive

Methanogenesis - Methyl-

MF0099 Com

gas metabolism

methanogenesis

0.731

0.707

0.992

2

T90 positive MF0102

AHI positive MF0103 Mucin Degradation glycoprotein degradation mucus degradation 0.775 0.969 0.992 186

Sulfate Reduction

(Dissimilatory) gas metabolism sulfate metabolism 0.435 0.910 0.992 3

ODI positive MF0061 Glycerol Degradation II lipid degradation glycerol degradation 0.423 0.995 0.995 19

AHI negative MF0001 Arabinoxylan Degradation carbohydrate degradation polysaccharide degradation 1.053 0.280 0.999 560

AHI negative MF0002 Fructan Degradation carbohydrate degradation polysaccharide degradation 1.045 0.332 0.999 505

AHI negative MF0003 Pectin Degradation I carbohydrate degradation polysaccharide degradation 0.937 0.602 0.999 32

AHI negative MF0004 Pectine Degradation II carbohydrate degradation polysaccharide degradation 0.985 0.519 0.999 61

AHI negative MF0005 Starch Degradation carbohydrate degradation polysaccharide degradation 1.112 0.136 0.999 349

AHI negative MF0006 Lactose Degradation carbohydrate degradation disaccharide degradation 1.156 0.066 0.999 653

AHI

negative

Lactose And Galactose MF0007 Degradation

carbohydrate degradation disaccharide degradation

0.275

0.990

0.999

7

T90 positive MF0008 Maltose Degradation carbohydrate degradation disaccharide degradation 0.508 0.999 0.999 80

AHI negative MF0008 Maltose Degradation carbohydrate degradation disaccharide degradation 0.853 0.865 0.999 109

AHI negative MF0009 Melibiose Degradation carbohydrate degradation disaccharide degradation 0.968 0.617 0.999 532

AHI negative MF0010 Sucrose Degradation I carbohydrate degradation disaccharide degradation 0.941 0.731 0.999 271

AHI negative MF0011 Sucrose Degradation II carbohydrate degradation disaccharide degradation 1.035 0.405 0.999 102

AHI negative MF0012 Trehalose Degradation carbohydrate degradation disaccharide degradation 0.910 0.803 0.999 226

AHI negative MF0013 Allose Degradation carbohydrate degradation monosaccharide degradation 0.747 0.779 0.999 5

AHI negative MF0014 Arabinose Degradation carbohydrate degradation monosaccharide degradation 1.049 0.375 0.999 67

AHI negative MF0015 Fructose Degradation carbohydrate degradation monosaccharide degradation 1.049 0.299 0.999 426

AHI negative MF0016 Fucose Degradation organic acid metabolism propionate metabolism 1.162 0.079 0.999 164

AHI negative MF0017 Galactose Degradation carbohydrate degradation monosaccharide degradation 1.065 0.256 0.999 511

AHI negative MF0018 Mannose Degradation carbohydrate degradation monosaccharide degradation 1.019 0.420 0.999 517

AHI negative MF0019 Rhamnose Degradation carbohydrate degradation monosaccharide degradation 1.130 0.113 0.999 245

AHI negative MF0020 Ribose Degradation carbohydrate degradation monosaccharide degradation 0.655 0.985 0.999 55

AHI negative MF0021 Xylose Degradation carbohydrate degradation monosaccharide degradation 0.893 0.830 0.999 156

AHI negative MF0022 Galacturonate Degradation I carbohydrate degradation sugar acid degradation 1.046 0.338 0.999 227

AHI negative MF0024 Phenylalanine Degradation amino acid degradation

T90

positive

MF0025 Tryptophan Degradation

amino acid degradation

aromatic amino acid degradation

0.701

0.966

0.999

57

aromatic amino acid

degradation 0.374 0.897 0.999 2

AHI negative MF0025 Tryptophan Degradation amino acid degradation

AHI

negative

MF0027 Tyrosine Degradation II

amino acid degradation

aromatic amino acid degradation

0.961

0.552

0.999

5

aromatic amino acid

degradation 1.167 0.107 0.999 94

AHI negative MF0028 Aspartate Degradation I amino acid degradation

AHI

negative

MF0029 Aspartate Degradation II

amino acid degradation

negatively charged amino acid degradation

0.990

0.550

0.999 167

negatively charged amino acid

degradation 1.103 0.191 0.999 775

AHI negative MF0030 Glutamate Degradation I amino acid degradation

AHI

negative

MF0031 Glutamate Degradation II

amino acid degradation

negatively charged amino acid degradation

0.711

0.959

0.999

57

negatively charged amino acid

degradation 0.653 0.754 0.999 2

AHI negative MF0032 Glutamate Degradation IIi amino acid degradation

AHI

negative

MF0033 Alanine Degradation I

amino acid degradation

nonpolar, aliphatic amino acid degradation 0.789

0.796

0.999

18

negatively charged amino acid

degradation 0.799 0.840 0.999 30

AHI negative MF0034 Alanine Degradation II amino acid degradation

AHI

negative

MF0035 Glycine Degradation

amino acid degradation

nonpolar, aliphatic amino acid degradation 0.990

0.524

0.999 184

nonpolar, aliphatic amino acid

degradation 0.838 0.944 0.999 283

AHI negative MF0036 Isoleucine Degradation amino acid degradation

nonpolar, aliphatic amino acid

degradation 0.651 0.934 0.999 20

| AHI | negative | MF0037 | Leucine Degradation | amino acid degradation | nonpolar, aliphatic amino acid  degradation | 1.596 | 0.226 | 0.999 | 1 |
| --- | --- | --- | --- | --- | --- | --- | --- | --- | --- |
| AHI | negative | MF0038 | Methionine Degradation I | amino acid degradation | nonpolar, aliphatic amino acid  degradation | 0.931 | 0.714 | 0.999 | 139 |
| T90 | positive | MF0040 | Proline Degradation | amino acid degradation | nonpolar, aliphatic amino acid degradation | 0.658 | 0.988 | 0.999 | 78 |
| AHI | negative | MF0040 | Proline Degradation | amino acid degradation | nonpolar, aliphatic amino acid  degradation | 0.724 | 0.974 | 0.999 | 85 |
| AHI | negative | MF0041 | Valine Degradation I | amino acid degradation | nonpolar, aliphatic amino acid degradation | 0.261 | 0.978 | 0.999 | 4 |
| AHI | negative | MF0042 | Asparagine Degradation | amino acid degradation | polar, uncharged amino acid  degradation | 0.740 | 0.913 | 0.999 | 41 |
| AHI | negative | MF0043 | Cysteine Biosynthesis/Homocysteine Degradation | amino acid degradation | polar, uncharged amino acid degradation | 0.973 | 0.561 | 0.999 | 71 |
| AHI | negative | MF0044 | Cysteine Degradation I | amino acid degradation | polar, uncharged amino acid  degradation | 1.107 | 0.252 | 0.999 | 904 |
| AHI | negative | MF0045 | Cysteine Degradation II | amino acid degradation | polar, uncharged amino acid degradation | 1.058 | 0.371 | 0.999 | 48 |
| AHI | negative | MF0046 | Glutamine Degradation I | amino acid degradation | polar, uncharged amino acid  degradation | 0.732 | 0.980 | 0.999 | 104 |
| AHI | negative | MF0047 | Glutamine Degradation II | amino acid degradation | polar, uncharged amino acid degradation | 1.012 | 0.445 | 0.999 | 485 |
| AHI | negative | MF0048 | Serine Degradation | amino acid degradation | polar, uncharged amino acid  degradation | 0.944 | 0.710 | 0.999 | 614 |
| AHI | negative | MF0049 | Threonine Degradation I | amino acid degradation | polar, uncharged amino acid degradation | 0.889 | 0.870 | 0.999 | 352 |
| AHI | negative | MF0050 | Threonine Degradation II | amino acid degradation | polar, uncharged amino acid  degradation | 0.938 | 0.727 | 0.999 | 208 |
| AHI | negative | MF0051 | Arginine Degradation I | amino acid degradation | positively charged amino acid degradation | 0.814 | 0.974 | 0.999 | 367 |
| AHI | negative | MF0054 | Arginine Degradation Iv | amino acid degradation | positively charged amino acid  degradation | 0.319 | 0.999 | 0.999 | 20 |

| AHI | negative | MF0055 | Arginine Degradation V | amino acid degradation | positively charged amino acid  degradation | 0.932 | 0.709 | 0.999 | 126 |
| --- | --- | --- | --- | --- | --- | --- | --- | --- | --- |
| T90 | positive | MF0056 | Histidine Degradation | amino acid degradation | positively charged amino acid  degradation | 0.754 | 0.945 | 0.999 | 74 |
| AHI | negative | MF0056 | Histidine Degradation | amino acid degradation | positively charged amino acid degradation | 0.982 | 0.557 | 0.999 | 89 |
| AHI | negative | MF0057 | Lysine Degradation I | amino acid degradation | positively charged amino acid  degradation | 0.711 | 0.947 | 0.999 | 47 |
| AHI | negative | MF0058 | Lysine Degradation II | amino acid degradation | positively charged amino acid degradation | 0.889 | 0.844 | 0.999 | 220 |
| AHI | negative | MF0059 | Anaerobic Fatty Acid Beta-  Oxidation | lipid degradation | beta-oxidation | 0.300 | 0.928 | 0.999 | 2 |
| AHI | negative | MF0060 | Glycerol Degradation I | lipid degradation | glycerol degradation | 0.742 | 0.949 | 0.999 | 65 |
| T90 | positive | MF0061 | Glycerol Degradation II | lipid degradation | glycerol degradation | 0.623 | 0.958 | 0.999 | 25 |
| AHI | negative | MF0061 | Glycerol Degradation II | lipid degradation | glycerol degradation | 0.428 | 0.998 | 0.999 | 27 |
| AHI | negative | MF0062 | Glycerol Degradation IIi | lipid degradation | glycerol degradation | 0.010 | 0.993 | 0.999 | 1 |
| AHI | negative | MF0063 | Glyoxylate Bypass | lipid degradation | glyoxylate bypass | 0.265 | 0.978 | 0.999 | 4 |
| AHI | negative | MF0064 | Triacylglycerol Degradation | lipid degradation | triacylglycerol degradation | 0.388 | 0.805 | 0.999 | 1 |
| AHI | negative | MF0065 | Bifidobacterium Shunt | central metabolism | energy metabolism | 0.943 | 0.726 | 0.999 | 414 |
| AHI | negative | MF0066 | Entner-Doudoroff Pathway | central metabolism | energy metabolism | 0.796 | 0.898 | 0.999 | 58 |
| AHI | negative | MF0067 | Glycolysis (Preparatory Phase) | central metabolism | energy metabolism | 1.164 | 0.112 | 0.999 | 834 |
| AHI | negative | MF0068 | Glycolysis (Pay-Off Phase) | central metabolism | energy metabolism | 0.778 | 0.983 | 0.999 | 633 |
| AHI | negative | MF0069 | Nadh:ferredoxin Oxidoreductase | gas metabolism | hydrogen metabolism | 0.582 | 0.790 | 0.999 | 2 |
| AHI | negative | MF0070 | Pentose Phosphate Pathway  (Oxidative Phase) | central metabolism | energy metabolism | 0.769 | 0.931 | 0.999 | 67 |
| AHI | negative | MF0071 | Pentose Phosphate Pathway (Non-Oxidative Branch) | central metabolism | energy metabolism | 1.150 | 0.173 | 0.999 | 881 |
| AHI | negative | MF0072 | Pyruvate Dehydrogenase  Complex | central metabolism | energy metabolism | 0.948 | 0.512 | 0.999 | 1 |
| AHI | negative | MF0074 | Pyruvate:formate Lyase | central metabolism | energy metabolism | 1.046 | 0.330 | 0.999 | 618 |
| T90 | positive | MF0075 | Acetate To Acetyl-Coa | organic acid metabolism | acetate metabolism | 0.757 | 0.970 | 0.999 | 176 |

| AHI | negative | MF0075 | Acetate To Acetyl-Coa | organic acid metabolism | acetate metabolism | 1.091 | 0.184 | 0.999 | 307 |
| --- | --- | --- | --- | --- | --- | --- | --- | --- | --- |
| AHI | negative | MF0076 | 4-Aminobutyrate  Degradation | amino acid degradation | positively charged amino acid  degradation | 0.665 | 0.918 | 0.999 | 19 |
| AHI | negative | MF0077 | Formate Conversion | organic acid metabolism | formate metabolism | 1.298 | 0.177 | 0.999 | 8 |
| AHI | negative | MF0078 | Lactaldehyde Degradation | organic acid metabolism | propionate metabolism | 0.997 | 0.504 | 0.999 | 401 |
| AHI | negative | MF0079 | Lactate Consumption I | organic acid metabolism | lactate metabolism | 0.691 | 0.941 | 0.999 | 39 |
| AHI | negative | MF0080 | Lactate Consumption II | organic acid metabolism | propionate metabolism | 0.782 | 0.604 | 0.999 | 1 |
| AHI | negative | MF0081 | Methanol Conversion | gas metabolism | methanogenesis | 1.003 | 0.494 | 0.999 | 3 |
| AHI | negative | MF0082 | Putrescine Degradation | amines and polyamines  degradation | biogenic amine degradation | 0.538 | 0.869 | 0.999 | 3 |
| AHI | negative | MF0083 | Succinate Consumption | organic acid metabolism | butyrate metabolism | 1.057 | 0.419 | 0.999 | 14 |
| AHI | negative | MF0084 | Succinate Conversion To  Propionate | organic acid metabolism | succinate metabolism | 0.300 | 0.928 | 0.999 | 2 |
| AHI | negative | MF0085 | Urea Degradation | amines and polyamines degradation | urea degradation | 0.781 | 0.956 | 0.999 | 103 |
| AHI | negative | MF0087 | Acetyl-Coa To Crotonyl-Coa | organic acid metabolism | butyrate metabolism | 0.868 | 0.696 | 0.999 | 20 |
| AHI | negative | MF0088 | Butyrate Production I | organic acid metabolism | butyrate metabolism | 0.995 | 0.505 | 0.999 | 109 |
| AHI | negative | MF0089 | Butyrate Production II | organic acid metabolism | butyrate metabolism | 0.807 | 0.886 | 0.999 | 63 |
| AHI | negative | MF0090 | Ethanol Production I | alcohol metabolism | ethanol metabolism | 1.064 | 0.268 | 0.999 | 321 |
| AHI | negative | MF0091 | Ethanol Production II | alcohol metabolism | ethanol metabolism | 0.388 | 0.805 | 0.999 | 1 |
| AHI | negative | MF0092 | Lactate Production | organic acid metabolism | lactate metabolism | 1.032 | 0.374 | 0.999 | 702 |
| AHI | negative | MF0094 | Propionate Production II | organic acid metabolism | propionate metabolism | 0.884 | 0.777 | 0.999 | 82 |
| T90 | positive | MF0095 | Propionate Production IIi | organic acid metabolism | propionate metabolism | 0.134 | 0.934 | 0.999 | 1 |
| AHI | negative | MF0095 | Propionate Production IIi | organic acid metabolism | propionate metabolism | 0.948 | 0.512 | 0.999 | 1 |
| AHI | negative | MF0096 | Succinate Production | organic acid metabolism | succinate metabolism | 0.968 | 0.535 | 0.999 | 3 |
| AHI | negative | MF0097 | Homoacetogenesis | gas metabolism | acetogenesis | 1.033 | 0.430 | 0.999 | 64 |
| AHI | negative | MF0099 | Methanogenesis - Methyl- Com | gas metabolism | methanogenesis | 1.003 | 0.494 | 0.999 | 3 |
| AHI | negative | MF0100 | Methanogenesis From  Carbon Dioxide | gas metabolism | methanogenesis | 0.817 | 0.656 | 0.999 | 2 |
| AHI | negative | MF0101 | Nitrate Reduction (Dissimilatory) | inorganic nutrient metabolism | nitrogen | 0.853 | 0.745 | 0.999 | 31 |

AHI negative MF0102

T90 positive MF0103 Mucin Degradation glycoprotein degradation mucus degradation 0.666 0.995 0.999 179

Sulfate Reduction

(Dissimilatory) gas metabolism sulfate metabolism 0.708 0.768 0.999 3

AHI negative MF0103 Mucin Degradation glycoprotein degradation mucus degradation 1.132 0.089 0.999 334

T90 negative MF0002 Fructan Degradation carbohydrate degradation polysaccharide degradation 0.900 0.846 1.000 517

ODI negative MF0002 Fructan Degradation carbohydrate degradation polysaccharide degradation 0.966 0.642 1.000 518

T90 negative MF0003 Pectin Degradation I carbohydrate degradation polysaccharide degradation 1.084 0.328 1.000 30

ODI negative MF0003 Pectin Degradation I carbohydrate degradation polysaccharide degradation 0.916 0.655 1.000 33

T90 negative MF0004 Pectine Degradation II carbohydrate degradation polysaccharide degradation 1.065 0.339 1.000 62

ODI negative MF0004 Pectine Degradation II carbohydrate degradation polysaccharide degradation 0.942 0.622 1.000 64

T90 negative MF0005 Starch Degradation carbohydrate degradation polysaccharide degradation 1.083 0.207 1.000 366

ODI negative MF0005 Starch Degradation carbohydrate degradation polysaccharide degradation 1.021 0.409 1.000 378

T90

negative

Lactose And Galactose MF0007 Degradation

carbohydrate degradation disaccharide degradation

0.419

0.970

1.000

6

ODI negative MF0007

T90 negative MF0008 Maltose Degradation carbohydrate degradation disaccharide degradation 0.967 0.597 1.000 109

Lactose And Galactose

Degradation carbohydrate degradation disaccharide degradation 0.600 0.861 1.000 4

ODI negative MF0008 Maltose Degradation carbohydrate degradation disaccharide degradation 0.898 0.792 1.000 114

T90 negative MF0009 Melibiose Degradation carbohydrate degradation disaccharide degradation 1.083 0.202 1.000 553

T90 negative MF0010 Sucrose Degradation I carbohydrate degradation disaccharide degradation 0.798 0.974 1.000 279

ODI negative MF0010 Sucrose Degradation I carbohydrate degradation disaccharide degradation 0.734 0.999 1.000 275

ODI negative MF0011 Sucrose Degradation II carbohydrate degradation disaccharide degradation 1.022 0.427 1.000 110

T90 negative MF0012 Trehalose Degradation carbohydrate degradation disaccharide degradation 0.982 0.569 1.000 229

ODI negative MF0012 Trehalose Degradation carbohydrate degradation disaccharide degradation 0.962 0.631 1.000 230

T90 negative MF0013 Allose Degradation carbohydrate degradation monosaccharide degradation 0.082 1.000 1.000 5

ODI negative MF0013 Allose Degradation carbohydrate degradation monosaccharide degradation 0.640 0.868 1.000 6

T90 negative MF0015 Fructose Degradation carbohydrate degradation monosaccharide degradation 0.880 0.893 1.000 426

ODI negative MF0015 Fructose Degradation carbohydrate degradation monosaccharide degradation 0.922 0.796 1.000 431

T90 negative MF0016 Fucose Degradation organic acid metabolism propionate metabolism 0.989 0.537 1.000 157

T90 negative MF0017 Galactose Degradation carbohydrate degradation monosaccharide degradation 0.942 0.720 1.000 523

T90 negative MF0018 Mannose Degradation carbohydrate degradation monosaccharide degradation 0.960 0.659 1.000 514

ODI negative MF0018 Mannose Degradation carbohydrate degradation monosaccharide degradation 0.928 0.773 1.000 543

T90 negative MF0019 Rhamnose Degradation carbohydrate degradation monosaccharide degradation 1.017 0.442 1.000 244

T90 negative MF0020 Ribose Degradation carbohydrate degradation monosaccharide degradation 0.669 0.982 1.000 56

ODI negative MF0020 Ribose Degradation carbohydrate degradation monosaccharide degradation 0.435 1.000 1.000 54

T90 negative MF0022 Galacturonate Degradation I carbohydrate degradation sugar acid degradation 0.928 0.734 1.000 231

ODI negative MF0022 Galacturonate Degradation I carbohydrate degradation sugar acid degradation 1.022 0.413 1.000 238

T90 negative MF0024 Phenylalanine Degradation amino acid degradation

ODI

negative

MF0024 Phenylalanine Degradation amino acid degradation

aromatic amino acid degradation

0.436

0.931

1.000

4

aromatic amino acid

degradation 0.200 0.985 1.000 4

T90 negative MF0027 Tyrosine Degradation II amino acid degradation

ODI

negative

MF0027 Tyrosine Degradation II

amino acid degradation

aromatic amino acid degradation

0.759

0.769

1.000

5

aromatic amino acid

degradation 0.725 0.803 1.000 6

T90 negative MF0028 Aspartate Degradation I amino acid degradation

ODI

negative

MF0028 Aspartate Degradation I

amino acid degradation

negatively charged amino acid degradation

0.928

0.702

1.000 804

negatively charged amino acid

degradation 0.831 0.921 1.000 786

T90 negative MF0029 Aspartate Degradation II amino acid degradation

ODI

negative

MF0029 Aspartate Degradation II

amino acid degradation

negatively charged amino acid degradation

0.950

0.663

1.000 166

negatively charged amino acid

degradation 0.793 0.970 1.000 173

T90 negative MF0030 Glutamate Degradation I amino acid degradation

ODI

negative

MF0030 Glutamate Degradation I

amino acid degradation

negatively charged amino acid degradation

1.044

0.489

1.000

2

negatively charged amino acid

degradation 0.744 0.734 1.000 3

T90 negative MF0031 Glutamate Degradation II amino acid degradation

ODI

negative

MF0031 Glutamate Degradation II

amino acid degradation

negatively charged amino acid degradation

1.025

0.420

1.000

58

negatively charged amino acid

degradation 0.935 0.638 1.000 65

T90 negative MF0032 Glutamate Degradation IIi amino acid degradation

negatively charged amino acid

degradation 1.088 0.335 1.000 28

ODI

negative

MF0032 Glutamate Degradation IIi

amino acid degradation

negatively charged amino acid degradation

0.883

0.705

1.000

29

|  | | | | | nonpolar, aliphatic amino acid |  | | | |
| --- | --- | --- | --- | --- | --- | --- | --- | --- | --- |
| T90 | negative | MF0033 | Alanine Degradation I | amino acid degradation | degradation | 1.026 | 0.468 | 1.000 | 16 |
| ODI | negative | MF0033 | Alanine Degradation I | amino acid degradation | nonpolar, aliphatic amino acid degradation | 0.446 | 0.992 | 1.000 | 17 |
| T90 | negative | MF0034 | Alanine Degradation II | amino acid degradation | nonpolar, aliphatic amino acid  degradation | 0.777 | 0.988 | 1.000 | 284 |
| ODI | negative | MF0034 | Alanine Degradation II | amino acid degradation | nonpolar, aliphatic amino acid degradation | 0.681 | 1.000 | 1.000 | 290 |
| T90 | negative | MF0035 | Glycine Degradation | amino acid degradation | nonpolar, aliphatic amino acid  degradation | 0.952 | 0.645 | 1.000 | 179 |
| ODI | negative | MF0035 | Glycine Degradation | amino acid degradation | nonpolar, aliphatic amino acid degradation | 0.954 | 0.653 | 1.000 | 189 |
| T90 | negative | MF0036 | Isoleucine Degradation | amino acid degradation | nonpolar, aliphatic amino acid  degradation | 0.870 | 0.715 | 1.000 | 24 |
| ODI | negative | MF0036 | Isoleucine Degradation | amino acid degradation | nonpolar, aliphatic amino acid degradation | 0.502 | 0.997 | 1.000 | 24 |
| T90 | negative | MF0037 | Leucine Degradation | amino acid degradation | nonpolar, aliphatic amino acid  degradation | 1.221 | 0.342 | 1.000 | 2 |
| ODI | negative | MF0037 | Leucine Degradation | amino acid degradation | nonpolar, aliphatic amino acid degradation | 1.279 | 0.359 | 1.000 | 1 |
| ODI | negative | MF0038 | Methionine Degradation I | amino acid degradation | nonpolar, aliphatic amino acid  degradation | 1.023 | 0.397 | 1.000 | 143 |
| T90 | negative | MF0039 | Methionine Degradation II | amino acid degradation | nonpolar, aliphatic amino acid degradation | 1.092 | 0.263 | 1.000 | 95 |
| T90 | negative | MF0040 | Proline Degradation | amino acid degradation | nonpolar, aliphatic amino acid  degradation | 0.668 | 0.990 | 1.000 | 85 |
| ODI | negative | MF0040 | Proline Degradation | amino acid degradation | nonpolar, aliphatic amino acid degradation | 0.600 | 1.000 | 1.000 | 95 |
| T90 | negative | MF0041 | Valine Degradation I | amino acid degradation | nonpolar, aliphatic amino acid  degradation | 0.246 | 0.989 | 1.000 | 5 |
| ODI | negative | MF0041 | Valine Degradation I | amino acid degradation | nonpolar, aliphatic amino acid degradation | 0.561 | 0.885 | 1.000 | 4 |

| T90 | negative | MF0042 | Asparagine Degradation | amino acid degradation | polar, uncharged amino acid  degradation | 0.693 | 0.968 | 1.000 | 52 |
| --- | --- | --- | --- | --- | --- | --- | --- | --- | --- |
| ODI | negative | MF0042 | Asparagine Degradation | amino acid degradation | polar, uncharged amino acid degradation | 0.613 | 0.991 | 1.000 | 42 |
| T90 | negative | MF0043 | Cysteine Biosynthesis/Homocysteine Degradation | amino acid degradation | polar, uncharged amino acid degradation | 1.009 | 0.479 | 1.000 | 80 |
| ODI | negative | MF0043 | Cysteine Biosynthesis/Homocysteine Degradation | amino acid degradation | polar, uncharged amino acid degradation | 0.860 | 0.809 | 1.000 | 71 |
| T90 | negative | MF0044 | Cysteine Degradation I | amino acid degradation | polar, uncharged amino acid  degradation | 1.155 | 0.185 | 1.000 | 926 |
| T90 | negative | MF0045 | Cysteine Degradation II | amino acid degradation | polar, uncharged amino acid degradation | 0.695 | 0.974 | 1.000 | 55 |
| ODI | negative | MF0045 | Cysteine Degradation II | amino acid degradation | polar, uncharged amino acid  degradation | 0.741 | 0.946 | 1.000 | 52 |
| T90 | negative | MF0046 | Glutamine Degradation I | amino acid degradation | polar, uncharged amino acid degradation | 0.894 | 0.770 | 1.000 | 98 |
| ODI | negative | MF0046 | Glutamine Degradation I | amino acid degradation | polar, uncharged amino acid  degradation | 0.883 | 0.797 | 1.000 | 99 |
| T90 | negative | MF0047 | Glutamine Degradation II | amino acid degradation | polar, uncharged amino acid degradation | 0.810 | 0.969 | 1.000 | 488 |
| ODI | negative | MF0047 | Glutamine Degradation II | amino acid degradation | polar, uncharged amino acid  degradation | 0.949 | 0.719 | 1.000 | 495 |
| T90 | negative | MF0048 | Serine Degradation | amino acid degradation | polar, uncharged amino acid degradation | 0.668 | 0.999 | 1.000 | 627 |
| ODI | negative | MF0048 | Serine Degradation | amino acid degradation | polar, uncharged amino acid  degradation | 0.880 | 0.878 | 1.000 | 637 |
| T90 | negative | MF0049 | Threonine Degradation I | amino acid degradation | polar, uncharged amino acid degradation | 0.600 | 1.000 | 1.000 | 353 |
| ODI | negative | MF0049 | Threonine Degradation I | amino acid degradation | polar, uncharged amino acid  degradation | 0.812 | 0.976 | 1.000 | 352 |

| T90 | negative | MF0050 | Threonine Degradation II | amino acid degradation | polar, uncharged amino acid  degradation | 1.076 | 0.259 | 1.000 | 215 |
| --- | --- | --- | --- | --- | --- | --- | --- | --- | --- |
| T90 | negative | MF0051 | Arginine Degradation I | amino acid degradation | positively charged amino acid  degradation | 0.726 | 0.998 | 1.000 | 361 |
| ODI | negative | MF0051 | Arginine Degradation I | amino acid degradation | positively charged amino acid degradation | 0.691 | 0.999 | 1.000 | 368 |
| T90 | negative | MF0052 | Arginine Degradation II | amino acid degradation | positively charged amino acid  degradation | 0.190 | 0.983 | 1.000 | 3 |
| ODI | negative | MF0052 | Arginine Degradation II | amino acid degradation | positively charged amino acid degradation | 0.439 | 0.872 | 1.000 | 2 |
| ODI | negative | MF0053 | Arginine Degradation IIi | amino acid degradation | positively charged amino acid  degradation | 0.845 | 0.590 | 1.000 | 1 |
| T90 | negative | MF0054 | Arginine Degradation Iv | amino acid degradation | positively charged amino acid degradation | 0.925 | 0.633 | 1.000 | 22 |
| ODI | negative | MF0054 | Arginine Degradation Iv | amino acid degradation | positively charged amino acid  degradation | 0.532 | 0.984 | 1.000 | 20 |
| T90 | negative | MF0055 | Arginine Degradation V | amino acid degradation | positively charged amino acid degradation | 1.016 | 0.446 | 1.000 | 135 |
| ODI | negative | MF0055 | Arginine Degradation V | amino acid degradation | positively charged amino acid  degradation | 1.019 | 0.425 | 1.000 | 141 |
| T90 | negative | MF0056 | Histidine Degradation | amino acid degradation | positively charged amino acid degradation | 0.905 | 0.761 | 1.000 | 101 |
| ODI | negative | MF0056 | Histidine Degradation | amino acid degradation | positively charged amino acid  degradation | 0.854 | 0.851 | 1.000 | 94 |
| ODI | negative | MF0057 | Lysine Degradation I | amino acid degradation | positively charged amino acid degradation | 0.914 | 0.677 | 1.000 | 49 |
| T90 | negative | MF0058 | Lysine Degradation II | amino acid degradation | positively charged amino acid  degradation | 0.576 | 1.000 | 1.000 | 211 |
| ODI | negative | MF0058 | Lysine Degradation II | amino acid degradation | positively charged amino acid degradation | 0.652 | 1.000 | 1.000 | 226 |
| T90 | negative | MF0059 | Anaerobic Fatty Acid Beta-  Oxidation | lipid degradation | beta-oxidation | 0.246 | 0.989 | 1.000 | 5 |

| ODI | negative | MF0059 | Anaerobic Fatty Acid Beta-  Oxidation | lipid degradation | beta-oxidation | 0.510 | 0.919 | 1.000 | 5 |
| --- | --- | --- | --- | --- | --- | --- | --- | --- | --- |
| T90 | negative | MF0060 | Glycerol Degradation I | lipid degradation | glycerol degradation | 0.798 | 0.891 | 1.000 | 62 |
| ODI | negative | MF0060 | Glycerol Degradation I | lipid degradation | glycerol degradation | 0.767 | 0.934 | 1.000 | 67 |
| T90 | negative | MF0061 | Glycerol Degradation II | lipid degradation | glycerol degradation | 0.846 | 0.752 | 1.000 | 22 |
| ODI | negative | MF0061 | Glycerol Degradation II | lipid degradation | glycerol degradation | 0.448 | 0.997 | 1.000 | 28 |
| T90 | negative | MF0062 | Glycerol Degradation IIi | lipid degradation | glycerol degradation | 0.370 | 0.939 | 1.000 | 3 |
| ODI | negative | MF0062 | Glycerol Degradation IIi | lipid degradation | glycerol degradation | 0.399 | 0.883 | 1.000 | 2 |
| T90 | negative | MF0063 | Glyoxylate Bypass | lipid degradation | glyoxylate bypass | 0.262 | 0.993 | 1.000 | 7 |
| ODI | negative | MF0063 | Glyoxylate Bypass | lipid degradation | glyoxylate bypass | 0.439 | 0.949 | 1.000 | 5 |
| T90 | negative | MF0064 | Triacylglycerol Degradation | lipid degradation | triacylglycerol degradation | 0.358 | 0.820 | 1.000 | 1 |
| ODI | negative | MF0064 | Triacylglycerol Degradation | lipid degradation | triacylglycerol degradation | 0.372 | 0.823 | 1.000 | 1 |
| T90 | negative | MF0065 | Bifidobacterium Shunt | central metabolism | energy metabolism | 0.719 | 0.998 | 1.000 | 432 |
| ODI | negative | MF0065 | Bifidobacterium Shunt | central metabolism | energy metabolism | 0.769 | 0.996 | 1.000 | 427 |
| T90 | negative | MF0066 | Entner-Doudoroff Pathway | central metabolism | energy metabolism | 0.975 | 0.548 | 1.000 | 71 |
| ODI | negative | MF0066 | Entner-Doudoroff Pathway | central metabolism | energy metabolism | 0.676 | 0.988 | 1.000 | 64 |
| ODI | negative | MF0067 | Glycolysis (Preparatory  Phase) | central metabolism | energy metabolism | 1.058 | 0.337 | 1.000 | 867 |
| T90 | negative | MF0068 | Glycolysis (Pay-Off Phase) | central metabolism | energy metabolism | 0.908 | 0.813 | 1.000 | 644 |
| ODI | negative | MF0068 | Glycolysis (Pay-Off Phase) | central metabolism | energy metabolism | 0.851 | 0.939 | 1.000 | 647 |
| T90 | negative | MF0069 | Nadh:ferredoxin Oxidoreductase | gas metabolism | hydrogen metabolism | 0.141 | 0.997 | 1.000 | 5 |
| ODI | negative | MF0069 | Nadh:ferredoxin  Oxidoreductase | gas metabolism | hydrogen metabolism | 0.272 | 0.992 | 1.000 | 6 |
| T90 | negative | MF0070 | Pentose Phosphate Pathway (Oxidative Phase) | central metabolism | energy metabolism | 1.047 | 0.382 | 1.000 | 78 |
| ODI | negative | MF0070 | Pentose Phosphate Pathway  (Oxidative Phase) | central metabolism | energy metabolism | 0.829 | 0.861 | 1.000 | 71 |
| T90 | negative | MF0071 | Pentose Phosphate Pathway (Non-Oxidative Branch) | central metabolism | energy metabolism | 0.928 | 0.656 | 1.000 | 898 |
| ODI | negative | MF0071 | Pentose Phosphate Pathway  (Non-Oxidative Branch) | central metabolism | energy metabolism | 0.864 | 0.810 | 1.000 | 917 |

| ODI | negative | MF0072 | Pyruvate Dehydrogenase  Complex | central metabolism | energy metabolism | 0.288 | 0.853 | 1.000 | 1 |
| --- | --- | --- | --- | --- | --- | --- | --- | --- | --- |
| ODI | negative | MF0073 | Pyruvate:ferredoxin  Oxidoreductase | central metabolism | energy metabolism | 1.098 | 0.206 | 1.000 | 807 |
| T90 | negative | MF0074 | Pyruvate:formate Lyase | central metabolism | energy metabolism | 1.028 | 0.407 | 1.000 | 634 |
| ODI | negative | MF0074 | Pyruvate:formate Lyase | central metabolism | energy metabolism | 0.993 | 0.514 | 1.000 | 636 |
| ODI | negative | MF0075 | Acetate To Acetyl-Coa | organic acid metabolism | acetate metabolism | 1.083 | 0.199 | 1.000 | 326 |
| T90 | negative | MF0076 | 4-Aminobutyrate  Degradation | amino acid degradation | positively charged amino acid  degradation | 1.106 | 0.313 | 1.000 | 23 |
| ODI | negative | MF0076 | 4-Aminobutyrate Degradation | amino acid degradation | positively charged amino acid degradation | 0.454 | 0.995 | 1.000 | 22 |
| ODI | negative | MF0077 | Formate Conversion | organic acid metabolism | formate metabolism | 1.251 | 0.192 | 1.000 | 9 |
| T90 | negative | MF0078 | Lactaldehyde Degradation | organic acid metabolism | propionate metabolism | 0.997 | 0.510 | 1.000 | 412 |
| ODI | negative | MF0078 | Lactaldehyde Degradation | organic acid metabolism | propionate metabolism | 1.033 | 0.367 | 1.000 | 419 |
| T90 | negative | MF0079 | Lactate Consumption I | organic acid metabolism | lactate metabolism | 0.780 | 0.889 | 1.000 | 41 |
| ODI | negative | MF0079 | Lactate Consumption I | organic acid metabolism | lactate metabolism | 0.481 | 0.999 | 1.000 | 42 |
| T90 | negative | MF0080 | Lactate Consumption II | organic acid metabolism | propionate metabolism | 0.468 | 0.766 | 1.000 | 1 |
| ODI | negative | MF0080 | Lactate Consumption II | organic acid metabolism | propionate metabolism | 1.170 | 0.414 | 1.000 | 1 |
| T90 | negative | MF0081 | Methanol Conversion | gas metabolism | methanogenesis | 0.820 | 0.669 | 1.000 | 3 |
| ODI | negative | MF0081 | Methanol Conversion | gas metabolism | methanogenesis | 0.811 | 0.710 | 1.000 | 4 |
| T90 | negative | MF0082 | Putrescine Degradation | amines and polyamines degradation | biogenic amine degradation | 0.246 | 0.989 | 1.000 | 5 |
| ODI | negative | MF0082 | Putrescine Degradation | amines and polyamines  degradation | biogenic amine degradation | 0.455 | 0.940 | 1.000 | 5 |
| T90 | negative | MF0084 | Succinate Conversion To Propionate | organic acid metabolism | succinate metabolism | 0.470 | 0.867 | 1.000 | 2 |
| ODI | negative | MF0084 | Succinate Conversion To  Propionate | organic acid metabolism | succinate metabolism | 0.562 | 0.856 | 1.000 | 3 |
| T90 | negative | MF0085 | Urea Degradation | amines and polyamines degradation | urea degradation | 0.658 | 0.998 | 1.000 | 114 |
| ODI | negative | MF0085 | Urea Degradation | amines and polyamines  degradation | urea degradation | 0.500 | 1.000 | 1.000 | 105 |
| T90 | negative | MF0086 | Acetyl-Coa To Acetate | organic acid metabolism | acetate metabolism | 1.097 | 0.291 | 1.000 | 921 |

| T90 | negative | MF0087 | Acetyl-Coa To Crotonyl-Coa | organic acid metabolism | butyrate metabolism | 1.063 | 0.382 | 1.000 | 26 |
| --- | --- | --- | --- | --- | --- | --- | --- | --- | --- |
| ODI | negative | MF0087 | Acetyl-Coa To Crotonyl-Coa | organic acid metabolism | butyrate metabolism | 1.014 | 0.459 | 1.000 | 23 |
| T90 | negative | MF0088 | Butyrate Production I | organic acid metabolism | butyrate metabolism | 0.642 | 0.996 | 1.000 | 107 |
| ODI | negative | MF0088 | Butyrate Production I | organic acid metabolism | butyrate metabolism | 0.910 | 0.759 | 1.000 | 115 |
| T90 | negative | MF0089 | Butyrate Production II | organic acid metabolism | butyrate metabolism | 1.024 | 0.453 | 1.000 | 64 |
| ODI | negative | MF0089 | Butyrate Production II | organic acid metabolism | butyrate metabolism | 0.850 | 0.824 | 1.000 | 65 |
| T90 | negative | MF0091 | Ethanol Production II | alcohol metabolism | ethanol metabolism | 0.358 | 0.820 | 1.000 | 1 |
| ODI | negative | MF0091 | Ethanol Production II | alcohol metabolism | ethanol metabolism | 0.372 | 0.823 | 1.000 | 1 |
| T90 | negative | MF0092 | Lactate Production | organic acid metabolism | lactate metabolism | 0.959 | 0.637 | 1.000 | 705 |
| ODI | negative | MF0092 | Lactate Production | organic acid metabolism | lactate metabolism | 0.867 | 0.895 | 1.000 | 730 |
| T90 | negative | MF0093 | Propionate Production I | organic acid metabolism | propionate metabolism | 0.211 | 0.875 | 1.000 | 1 |
| T90 | negative | MF0094 | Propionate Production II | organic acid metabolism | propionate metabolism | 0.814 | 0.911 | 1.000 | 95 |
| ODI | negative | MF0094 | Propionate Production II | organic acid metabolism | propionate metabolism | 0.948 | 0.635 | 1.000 | 90 |
| ODI | negative | MF0095 | Propionate Production IIi | organic acid metabolism | propionate metabolism | 0.288 | 0.853 | 1.000 | 1 |
| T90 | negative | MF0096 | Succinate Production | organic acid metabolism | succinate metabolism | 0.668 | 0.854 | 1.000 | 6 |
| ODI | negative | MF0096 | Succinate Production | organic acid metabolism | succinate metabolism | 0.706 | 0.805 | 1.000 | 5 |
| T90 | negative | MF0097 | Homoacetogenesis | gas metabolism | acetogenesis | 1.019 | 0.450 | 1.000 | 62 |
| ODI | negative | MF0097 | Homoacetogenesis | gas metabolism | acetogenesis | 1.082 | 0.293 | 1.000 | 64 |
| T90 | negative | MF0099 | Methanogenesis - Methyl-  Com | gas metabolism | methanogenesis | 0.820 | 0.669 | 1.000 | 3 |
| ODI | negative | MF0099 | Methanogenesis - Methyl- Com | gas metabolism | methanogenesis | 0.811 | 0.710 | 1.000 | 4 |
| T90 | negative | MF0100 | Methanogenesis From  Carbon Dioxide | gas metabolism | methanogenesis | 1.150 | 0.391 | 1.000 | 2 |
| ODI | negative | MF0100 | Methanogenesis From Carbon Dioxide | gas metabolism | methanogenesis | 1.101 | 0.432 | 1.000 | 2 |
| T90 | negative | MF0101 | Nitrate Reduction  (Dissimilatory) | inorganic nutrient  metabolism | nitrogen | 0.846 | 0.771 | 1.000 | 33 |
| ODI | negative | MF0101 | Nitrate Reduction (Dissimilatory) | inorganic nutrient metabolism | nitrogen | 0.767 | 0.878 | 1.000 | 32 |
| T90 | negative | MF0102 | Sulfate Reduction  (Dissimilatory) | gas metabolism | sulfate metabolism | 1.036 | 0.482 | 1.000 | 2 |

|  | | | Sulfate Reduction |  | | | | | |
| --- | --- | --- | --- | --- | --- | --- | --- | --- | --- |
| ODI | negative | MF0102 | (Dissimilatory) | gas metabolism | sulfate metabolism | 0.925 | 0.589 | 1.000 | 2 |
| ODI | negative | MF0103 | Mucin Degradation | glycoprotein degradation | mucus degradation | 1.055 | 0.283 | 1.000 | 353 |
