# Supplementary material for: OSA Is Associated With the Human Gut Microbiota Composition and Functional Potential in the Population-Based Swedish CardioPulmonary bioImage Study
Source: Chest. 2023 Mar 15;164(2):503–16. doi: 10.1016/j.chest.2023.03.010 (PMC10410248; doi:10.1016/j.chest.2023.03.010)
Supplement: e-Table 13 [file mmc10.docx]

e-Table 13. Partial Spearman’s correlations of microbiota features with systolic blood pressure (SBP), diastolic blood pressure (DBP), and glycated hemoglobin (Hb1Ac)

The microbiota features are the 128 species associated with T90/ODI in the extended model. Health outcomes are systolic blood pressure (SBP), diastolic blood pressure (DBP), and glycated hemoglobin (HbA1c). OSA adjusted: adjustment for age, sex, alcohol intake, smoking, fiber intake, total energy intake, leisure physical activity, country of birth, apnea-hypopnea index (AHI), oxygen desaturation index (ODI), percentage of the sleep time with oxygen saturation < 90% (T90), and DNA extraction plate. OSA and BMI adjusted: additional adjustment for body mass index. Under the column "Microbiota features", the information between parenthesis is the internal identifier for the respective species.

| **Microbiota feature** | **Outcome** | **Spearman's correlation** | **p-value** | **q-value** | **N** | **model** |
| --- | --- | --- | --- | --- | --- | --- |
| Akkermansia muciniphila (HG3A.0110) | SBP | -4.76E-02 | 0.023 | 0.104 | 2334 | OSA adjusted |
| Akkermansia muciniphila (HG3A.0110) | DBP | -3.16E-02 | 0.132 | 0.349 | 2334 | OSA adjusted |
| Akkermansia muciniphila (HG3A.0110) | HbA1c | -3.10E-02 | 0.106 | 0.73 | 2785 | OSA adjusted |
| Akkermansia muciniphila (HG3A.0110) | SBP | -4.07E-02 | 0.053 | 0.328 | 2334 | OSA and BMI adjusted |
| Akkermansia muciniphila (HG3A.0110) | DBP | -2.37E-02 | 0.259 | 0.765 | 2334 | OSA and BMI adjusted |
| Akkermansia muciniphila (HG3A.0110) | HbA1c | -2.54E-02 | 0.186 | 0.94 | 2785 | OSA and BMI adjusted |
| Alistipes communis (HG3A.0064) | SBP | -3.89E-02 | 0.064 | 0.21 | 2334 | OSA adjusted |
| Alistipes communis (HG3A.0064) | DBP | -1.92E-02 | 0.362 | 0.702 | 2334 | OSA adjusted |
| Alistipes communis (HG3A.0064) | HbA1c | -3.37E-02 | 0.079 | 0.678 | 2785 | OSA adjusted |
| Alistipes communis (HG3A.0064) | SBP | -2.16E-02 | 0.304 | 0.707 | 2334 | OSA and BMI adjusted |
| Alistipes communis (HG3A.0064) | DBP | -1.77E-06 | 1 | 1 | 2334 | OSA and BMI adjusted |
| Alistipes communis (HG3A.0064) | HbA1c | -2.35E-02 | 0.221 | 0.94 | 2785 | OSA and BMI adjusted |
| Alistipes provencensis (HG3A.0877) | SBP | 0.006 | 0.779 | 0.904 | 2334 | OSA adjusted |
| Alistipes provencensis (HG3A.0877) | DBP | 0.005 | 0.807 | 0.932 | 2334 | OSA adjusted |
| Alistipes provencensis (HG3A.0877) | HbA1c | -1.70E-02 | 0.377 | 0.875 | 2785 | OSA adjusted |
| Alistipes provencensis (HG3A.0877) | SBP | 0.005 | 0.829 | 0.922 | 2334 | OSA and BMI adjusted |
| Alistipes provencensis (HG3A.0877) | DBP | 0.004 | 0.861 | 0.989 | 2334 | OSA and BMI adjusted |
| Alistipes provencensis (HG3A.0877) | HbA1c | -1.95E-02 | 0.311 | 0.96 | 2785 | OSA and BMI adjusted |
| Alistipes shahii (HG3A.0054) | SBP | -4.83E-02 | 0.021 | 0.102 | 2334 | OSA adjusted |
| Alistipes shahii (HG3A.0054) | DBP | -4.35E-02 | 0.039 | 0.151 | 2334 | OSA adjusted |
| Alistipes shahii (HG3A.0054) | HbA1c | -3.49E-02 | 0.069 | 0.647 | 2785 | OSA adjusted |
| Alistipes shahii (HG3A.0054) | SBP | -2.84E-02 | 0.177 | 0.576 | 2334 | OSA and BMI adjusted |
| Alistipes shahii (HG3A.0054) | DBP | -2.21E-02 | 0.292 | 0.818 | 2334 | OSA and BMI adjusted |

| Alistipes shahii (HG3A.0054) | HbA1c | -2.30E-02 | 0.23 | 0.94 | 2785 | OSA and BMI adjusted |
| --- | --- | --- | --- | --- | --- | --- |
| Anaerobutyricum hallii (HG3A.0012) | SBP | 0.03 | 0.157 | 0.377 | 2334 | OSA adjusted |
| Anaerobutyricum hallii (HG3A.0012) | DBP | 0.028 | 0.182 | 0.462 | 2334 | OSA adjusted |
| Anaerobutyricum hallii (HG3A.0012) | HbA1c | 0.024 | 0.21 | 0.828 | 2785 | OSA adjusted |
| Anaerobutyricum hallii (HG3A.0012) | SBP | 0.009 | 0.679 | 0.922 | 2334 | OSA and BMI adjusted |
| Anaerobutyricum hallii (HG3A.0012) | DBP | 0.006 | 0.791 | 0.989 | 2334 | OSA and BMI adjusted |
| Anaerobutyricum hallii (HG3A.0012) | HbA1c | 0.011 | 0.571 | 0.972 | 2785 | OSA and BMI adjusted |
| Anaerostipes sp. BG01 (HG3A.1509) | SBP | 0.023 | 0.267 | 0.514 | 2334 | OSA adjusted |
| Anaerostipes sp. BG01 (HG3A.1509) | DBP | -2.07E-03 | 0.922 | 0.957 | 2334 | OSA adjusted |
| Anaerostipes sp. BG01 (HG3A.1509) | HbA1c | 0.013 | 0.495 | 0.875 | 2785 | OSA adjusted |
| Anaerostipes sp. BG01 (HG3A.1509) | SBP | 0.024 | 0.261 | 0.65 | 2334 | OSA and BMI adjusted |
| Anaerostipes sp. BG01 (HG3A.1509) | DBP | -2.67E-03 | 0.899 | 0.989 | 2334 | OSA and BMI adjusted |
| Anaerostipes sp. BG01 (HG3A.1509) | HbA1c | 0.012 | 0.54 | 0.972 | 2785 | OSA and BMI adjusted |
| Bacteria sp. (HG3A.0483) | SBP | -2.80E-02 | 0.183 | 0.425 | 2334 | OSA adjusted |
| Bacteria sp. (HG3A.0483) | DBP | -2.47E-02 | 0.239 | 0.529 | 2334 | OSA adjusted |
| Bacteria sp. (HG3A.0483) | HbA1c | 0.017 | 0.387 | 0.875 | 2785 | OSA adjusted |
| Bacteria sp. (HG3A.0483) | SBP | -1.47E-02 | 0.486 | 0.888 | 2334 | OSA and BMI adjusted |
| Bacteria sp. (HG3A.0483) | DBP | -1.04E-02 | 0.619 | 0.989 | 2334 | OSA and BMI adjusted |
| Bacteria sp. (HG3A.0483) | HbA1c | 0.026 | 0.18 | 0.94 | 2785 | OSA and BMI adjusted |
| Bacteria sp. (HG3A.0634) | SBP | 0.004 | 0.845 | 0.926 | 2334 | OSA adjusted |
| Bacteria sp. (HG3A.0634) | DBP | -1.17E-02 | 0.577 | 0.767 | 2334 | OSA adjusted |
| Bacteria sp. (HG3A.0634) | HbA1c | -1.14E-02 | 0.551 | 0.882 | 2785 | OSA adjusted |
| Bacteria sp. (HG3A.0634) | SBP | 0.011 | 0.588 | 0.922 | 2334 | OSA and BMI adjusted |
| Bacteria sp. (HG3A.0634) | DBP | -4.54E-03 | 0.829 | 0.989 | 2334 | OSA and BMI adjusted |
| Bacteria sp. (HG3A.0634) | HbA1c | -7.64E-03 | 0.691 | 0.972 | 2785 | OSA and BMI adjusted |
| Bacteria sp. (HG3A.0911) | SBP | -3.05E-02 | 0.147 | 0.359 | 2334 | OSA adjusted |
| Bacteria sp. (HG3A.0911) | DBP | -2.05E-02 | 0.331 | 0.657 | 2334 | OSA adjusted |
| Bacteria sp. (HG3A.0911) | HbA1c | 0.015 | 0.43 | 0.875 | 2785 | OSA adjusted |
| Bacteria sp. (HG3A.0911) | SBP | -3.12E-02 | 0.138 | 0.511 | 2334 | OSA and BMI adjusted |
| Bacteria sp. (HG3A.0911) | DBP | -2.08E-02 | 0.322 | 0.872 | 2334 | OSA and BMI adjusted |
| Bacteria sp. (HG3A.0911) | HbA1c | 0.017 | 0.369 | 0.972 | 2785 | OSA and BMI adjusted |
| Blautia massiliensis (HG3A.0023) | SBP | 0.031 | 0.14 | 0.359 | 2334 | OSA adjusted |

| Blautia massiliensis (HG3A.0023) | DBP | 0.019 | 0.372 | 0.702 | 2334 | OSA adjusted |
| --- | --- | --- | --- | --- | --- | --- |
| Blautia massiliensis (HG3A.0023) | HbA1c | 0.017 | 0.362 | 0.875 | 2785 | OSA adjusted |
| Blautia massiliensis (HG3A.0023) | SBP | 0.004 | 0.859 | 0.922 | 2334 | OSA and BMI adjusted |
| Blautia massiliensis (HG3A.0023) | DBP | -1.08E-02 | 0.607 | 0.989 | 2334 | OSA and BMI adjusted |
| Blautia massiliensis (HG3A.0023) | HbA1c | 7.65E-04 | 0.968 | 0.973 | 2785 | OSA and BMI adjusted |
| Blautia obeum (HG3A.0001) | SBP | 0.047 | 0.026 | 0.112 | 2334 | OSA adjusted |
| Blautia obeum (HG3A.0001) | DBP | 0.047 | 0.025 | 0.116 | 2334 | OSA adjusted |
| Blautia obeum (HG3A.0001) | HbA1c | 0.004 | 0.83 | 0.922 | 2785 | OSA adjusted |
| Blautia obeum (HG3A.0001) | SBP | 0.025 | 0.238 | 0.628 | 2334 | OSA and BMI adjusted |
| Blautia obeum (HG3A.0001) | DBP | 0.024 | 0.255 | 0.765 | 2334 | OSA and BMI adjusted |
| Blautia obeum (HG3A.0001) | HbA1c | -1.09E-02 | 0.572 | 0.972 | 2785 | OSA and BMI adjusted |
| Blautia obeum (HG3A.0009) | SBP | 0.04 | 0.056 | 0.188 | 2334 | OSA adjusted |
| Blautia obeum (HG3A.0009) | DBP | 0.027 | 0.197 | 0.463 | 2334 | OSA adjusted |
| Blautia obeum (HG3A.0009) | HbA1c | 0.029 | 0.126 | 0.82 | 2785 | OSA adjusted |
| Blautia obeum (HG3A.0009) | SBP | 0.027 | 0.194 | 0.591 | 2334 | OSA and BMI adjusted |
| Blautia obeum (HG3A.0009) | DBP | 0.013 | 0.536 | 0.989 | 2334 | OSA and BMI adjusted |
| Blautia obeum (HG3A.0009) | HbA1c | 0.018 | 0.345 | 0.972 | 2785 | OSA and BMI adjusted |
| Candidatus Borkfalkiales sp. (HG3A.1329) | SBP | -3.63E-02 | 0.084 | 0.241 | 2334 | OSA adjusted |
| Candidatus Borkfalkiales sp. (HG3A.1329) | DBP | -4.08E-02 | 0.052 | 0.188 | 2334 | OSA adjusted |
| Candidatus Borkfalkiales sp. (HG3A.1329) | HbA1c | -3.52E-02 | 0.067 | 0.647 | 2785 | OSA adjusted |
| Candidatus Borkfalkiales sp. (HG3A.1329) | SBP | -2.95E-02 | 0.16 | 0.55 | 2334 | OSA and BMI adjusted |
| Candidatus Borkfalkiales sp. (HG3A.1329) | DBP | -3.39E-02 | 0.107 | 0.462 | 2334 | OSA and BMI adjusted |
| Candidatus Borkfalkiales sp. (HG3A.1329) | HbA1c | -2.99E-02 | 0.12 | 0.94 | 2785 | OSA and BMI adjusted |
| Candidatus Borkfalkiales sp. (HG3A.1397) | SBP | 0.005 | 0.817 | 0.91 | 2334 | OSA adjusted |
| Candidatus Borkfalkiales sp. (HG3A.1397) | DBP | -1.28E-02 | 0.542 | 0.753 | 2334 | OSA adjusted |
| Candidatus Borkfalkiales sp. (HG3A.1397) | HbA1c | -1.26E-02 | 0.512 | 0.875 | 2785 | OSA adjusted |
| Candidatus Borkfalkiales sp. (HG3A.1397) | SBP | 0.016 | 0.454 | 0.851 | 2334 | OSA and BMI adjusted |
| Candidatus Borkfalkiales sp. (HG3A.1397) | DBP | -1.90E-03 | 0.928 | 0.989 | 2334 | OSA and BMI adjusted |
| Candidatus Borkfalkiales sp. (HG3A.1397) | HbA1c | -6.11E-03 | 0.75 | 0.972 | 2785 | OSA and BMI adjusted |
| Clostridia sp. (HG3A.0094) | SBP | -2.52E-02 | 0.23 | 0.467 | 2334 | OSA adjusted |
| Clostridia sp. (HG3A.0094) | DBP | -3.49E-02 | 0.096 | 0.294 | 2334 | OSA adjusted |
| Clostridia sp. (HG3A.0094) | HbA1c | -2.43E-02 | 0.205 | 0.828 | 2785 | OSA adjusted |

| Clostridia sp. (HG3A.0094) | SBP | -5.91E-03 | 0.779 | 0.922 | 2334 | OSA and BMI adjusted |
| --- | --- | --- | --- | --- | --- | --- |
| Clostridia sp. (HG3A.0094) | DBP | -1.48E-02 | 0.482 | 0.97 | 2334 | OSA and BMI adjusted |
| Clostridia sp. (HG3A.0094) | HbA1c | -1.31E-02 | 0.496 | 0.972 | 2785 | OSA and BMI adjusted |
| Clostridia sp. (HG3A.0140) | SBP | -5.30E-02 | 0.012 | 0.067 | 2334 | OSA adjusted |
| Clostridia sp. (HG3A.0140) | DBP | -4.48E-02 | 0.033 | 0.134 | 2334 | OSA adjusted |
| Clostridia sp. (HG3A.0140) | HbA1c | 0.003 | 0.883 | 0.928 | 2785 | OSA adjusted |
| Clostridia sp. (HG3A.0140) | SBP | -3.24E-02 | 0.124 | 0.497 | 2334 | OSA and BMI adjusted |
| Clostridia sp. (HG3A.0140) | DBP | -2.26E-02 | 0.283 | 0.808 | 2334 | OSA and BMI adjusted |
| Clostridia sp. (HG3A.0140) | HbA1c | 0.019 | 0.322 | 0.96 | 2785 | OSA and BMI adjusted |
| Clostridia sp. (HG3A.0272) | SBP | -5.62E-03 | 0.789 | 0.904 | 2334 | OSA adjusted |
| Clostridia sp. (HG3A.0272) | DBP | -1.86E-02 | 0.376 | 0.702 | 2334 | OSA adjusted |
| Clostridia sp. (HG3A.0272) | HbA1c | 0.015 | 0.436 | 0.875 | 2785 | OSA adjusted |
| Clostridia sp. (HG3A.0272) | SBP | 0.008 | 0.689 | 0.922 | 2334 | OSA and BMI adjusted |
| Clostridia sp. (HG3A.0272) | DBP | -4.18E-03 | 0.843 | 0.989 | 2334 | OSA and BMI adjusted |
| Clostridia sp. (HG3A.0272) | HbA1c | 0.023 | 0.236 | 0.94 | 2785 | OSA and BMI adjusted |
| Clostridia sp. (HG3A.0435) | SBP | -1.34E-02 | 0.524 | 0.741 | 2334 | OSA adjusted |
| Clostridia sp. (HG3A.0435) | DBP | -1.57E-02 | 0.455 | 0.75 | 2334 | OSA adjusted |
| Clostridia sp. (HG3A.0435) | HbA1c | -1.68E-02 | 0.382 | 0.875 | 2785 | OSA adjusted |
| Clostridia sp. (HG3A.0435) | SBP | 0.003 | 0.87 | 0.922 | 2334 | OSA and BMI adjusted |
| Clostridia sp. (HG3A.0435) | DBP | 0.002 | 0.92 | 0.989 | 2334 | OSA and BMI adjusted |
| Clostridia sp. (HG3A.0435) | HbA1c | -8.13E-03 | 0.672 | 0.972 | 2785 | OSA and BMI adjusted |
| Clostridia sp. (HG3A.0470) | SBP | -1.96E-02 | 0.352 | 0.595 | 2334 | OSA adjusted |
| Clostridia sp. (HG3A.0470) | DBP | -3.25E-02 | 0.122 | 0.327 | 2334 | OSA adjusted |
| Clostridia sp. (HG3A.0470) | HbA1c | 0.015 | 0.445 | 0.875 | 2785 | OSA adjusted |
| Clostridia sp. (HG3A.0470) | SBP | -4.43E-03 | 0.833 | 0.922 | 2334 | OSA and BMI adjusted |
| Clostridia sp. (HG3A.0470) | DBP | -1.69E-02 | 0.421 | 0.905 | 2334 | OSA and BMI adjusted |
| Clostridia sp. (HG3A.0470) | HbA1c | 0.024 | 0.206 | 0.94 | 2785 | OSA and BMI adjusted |
| Clostridia sp. (HG3A.0508) | SBP | -5.99E-03 | 0.776 | 0.904 | 2334 | OSA adjusted |
| Clostridia sp. (HG3A.0508) | DBP | -2.49E-03 | 0.906 | 0.957 | 2334 | OSA adjusted |
| Clostridia sp. (HG3A.0508) | HbA1c | 0.009 | 0.633 | 0.882 | 2785 | OSA adjusted |
| Clostridia sp. (HG3A.0508) | SBP | 0.009 | 0.662 | 0.922 | 2334 | OSA and BMI adjusted |
| Clostridia sp. (HG3A.0508) | DBP | 0.014 | 0.511 | 0.986 | 2334 | OSA and BMI adjusted |

| Clostridia sp. (HG3A.0508) | HbA1c | 0.018 | 0.35 | 0.972 | 2785 | OSA and BMI adjusted |
| --- | --- | --- | --- | --- | --- | --- |
| Clostridia sp. (HG3A.0515) | SBP | -5.60E-03 | 0.79 | 0.904 | 2334 | OSA adjusted |
| Clostridia sp. (HG3A.0515) | DBP | -2.31E-03 | 0.912 | 0.957 | 2334 | OSA adjusted |
| Clostridia sp. (HG3A.0515) | HbA1c | -5.45E-03 | 0.776 | 0.888 | 2785 | OSA adjusted |
| Clostridia sp. (HG3A.0515) | SBP | -2.10E-03 | 0.92 | 0.948 | 2334 | OSA and BMI adjusted |
| Clostridia sp. (HG3A.0515) | DBP | 0.002 | 0.942 | 0.989 | 2334 | OSA and BMI adjusted |
| Clostridia sp. (HG3A.0515) | HbA1c | -2.67E-03 | 0.889 | 0.972 | 2785 | OSA and BMI adjusted |
| Clostridia sp. (HG3A.0550) | SBP | -5.38E-02 | 0.011 | 0.063 | 2334 | OSA adjusted |
| Clostridia sp. (HG3A.0550) | DBP | -6.28E-02 | 0.003 | 0.031 | 2334 | OSA adjusted |
| Clostridia sp. (HG3A.0550) | HbA1c | -7.24E-03 | 0.706 | 0.888 | 2785 | OSA adjusted |
| Clostridia sp. (HG3A.0550) | SBP | -3.20E-02 | 0.128 | 0.497 | 2334 | OSA and BMI adjusted |
| Clostridia sp. (HG3A.0550) | DBP | -4.01E-02 | 0.056 | 0.381 | 2334 | OSA and BMI adjusted |
| Clostridia sp. (HG3A.0550) | HbA1c | 0.005 | 0.777 | 0.972 | 2785 | OSA and BMI adjusted |
| Clostridia sp. (HG3A.0599) | SBP | -2.10E-02 | 0.317 | 0.567 | 2334 | OSA adjusted |
| Clostridia sp. (HG3A.0599) | DBP | -1.85E-02 | 0.38 | 0.702 | 2334 | OSA adjusted |
| Clostridia sp. (HG3A.0599) | HbA1c | 0.015 | 0.425 | 0.875 | 2785 | OSA adjusted |
| Clostridia sp. (HG3A.0599) | SBP | -1.07E-02 | 0.61 | 0.922 | 2334 | OSA and BMI adjusted |
| Clostridia sp. (HG3A.0599) | DBP | -7.39E-03 | 0.725 | 0.989 | 2334 | OSA and BMI adjusted |
| Clostridia sp. (HG3A.0599) | HbA1c | 0.023 | 0.229 | 0.94 | 2785 | OSA and BMI adjusted |
| Clostridia sp. (HG3A.0645) | SBP | 0.027 | 0.193 | 0.442 | 2334 | OSA adjusted |
| Clostridia sp. (HG3A.0645) | DBP | 0.018 | 0.382 | 0.702 | 2334 | OSA adjusted |
| Clostridia sp. (HG3A.0645) | HbA1c | -1.60E-02 | 0.406 | 0.875 | 2785 | OSA adjusted |
| Clostridia sp. (HG3A.0645) | SBP | 0.033 | 0.118 | 0.497 | 2334 | OSA and BMI adjusted |
| Clostridia sp. (HG3A.0645) | DBP | 0.024 | 0.253 | 0.765 | 2334 | OSA and BMI adjusted |
| Clostridia sp. (HG3A.0645) | HbA1c | -1.07E-02 | 0.579 | 0.972 | 2785 | OSA and BMI adjusted |
| Clostridia sp. (HG3A.0682) | SBP | -1.84E-02 | 0.38 | 0.636 | 2334 | OSA adjusted |
| Clostridia sp. (HG3A.0682) | DBP | -1.28E-02 | 0.544 | 0.753 | 2334 | OSA adjusted |
| Clostridia sp. (HG3A.0682) | HbA1c | -2.28E-02 | 0.235 | 0.832 | 2785 | OSA adjusted |
| Clostridia sp. (HG3A.0682) | SBP | -9.99E-03 | 0.635 | 0.922 | 2334 | OSA and BMI adjusted |
| Clostridia sp. (HG3A.0682) | DBP | -3.56E-03 | 0.865 | 0.989 | 2334 | OSA and BMI adjusted |
| Clostridia sp. (HG3A.0682) | HbA1c | -1.62E-02 | 0.399 | 0.972 | 2785 | OSA and BMI adjusted |
| Clostridia sp. (HG3A.0728) | SBP | 0.01 | 0.621 | 0.811 | 2334 | OSA adjusted |

| Clostridia sp. (HG3A.0728) | DBP | -8.36E-03 | 0.691 | 0.856 | 2334 | OSA adjusted |
| --- | --- | --- | --- | --- | --- | --- |
| Clostridia sp. (HG3A.0728) | HbA1c | 0.01 | 0.59 | 0.882 | 2785 | OSA adjusted |
| Clostridia sp. (HG3A.0728) | SBP | 0.011 | 0.61 | 0.922 | 2334 | OSA and BMI adjusted |
| Clostridia sp. (HG3A.0728) | DBP | -8.71E-03 | 0.679 | 0.989 | 2334 | OSA and BMI adjusted |
| Clostridia sp. (HG3A.0728) | HbA1c | 0.012 | 0.524 | 0.972 | 2785 | OSA and BMI adjusted |
| Clostridia sp. (HG3A.0815) | SBP | -1.00E-02 | 0.633 | 0.811 | 2334 | OSA adjusted |
| Clostridia sp. (HG3A.0815) | DBP | -4.51E-03 | 0.83 | 0.932 | 2334 | OSA adjusted |
| Clostridia sp. (HG3A.0815) | HbA1c | -2.58E-02 | 0.179 | 0.822 | 2785 | OSA adjusted |
| Clostridia sp. (HG3A.0815) | SBP | 0.011 | 0.614 | 0.922 | 2334 | OSA and BMI adjusted |
| Clostridia sp. (HG3A.0815) | DBP | 0.018 | 0.399 | 0.905 | 2334 | OSA and BMI adjusted |
| Clostridia sp. (HG3A.0815) | HbA1c | -1.31E-02 | 0.496 | 0.972 | 2785 | OSA and BMI adjusted |
| Clostridia sp. (HG3A.0861) | SBP | -4.76E-02 | 0.024 | 0.104 | 2334 | OSA adjusted |
| Clostridia sp. (HG3A.0861) | DBP | -4.54E-02 | 0.031 | 0.134 | 2334 | OSA adjusted |
| Clostridia sp. (HG3A.0861) | HbA1c | -8.71E-03 | 0.65 | 0.882 | 2785 | OSA adjusted |
| Clostridia sp. (HG3A.0861) | SBP | -3.86E-02 | 0.067 | 0.37 | 2334 | OSA and BMI adjusted |
| Clostridia sp. (HG3A.0861) | DBP | -3.58E-02 | 0.089 | 0.45 | 2334 | OSA and BMI adjusted |
| Clostridia sp. (HG3A.0861) | HbA1c | -8.81E-04 | 0.963 | 0.973 | 2785 | OSA and BMI adjusted |
| Clostridia sp. (HG3A.0879) | SBP | -2.52E-02 | 0.231 | 0.467 | 2334 | OSA adjusted |
| Clostridia sp. (HG3A.0879) | DBP | -2.64E-02 | 0.21 | 0.471 | 2334 | OSA adjusted |
| Clostridia sp. (HG3A.0879) | HbA1c | 0.006 | 0.773 | 0.888 | 2785 | OSA adjusted |
| Clostridia sp. (HG3A.0879) | SBP | -1.58E-02 | 0.453 | 0.851 | 2334 | OSA and BMI adjusted |
| Clostridia sp. (HG3A.0879) | DBP | -1.64E-02 | 0.435 | 0.905 | 2334 | OSA and BMI adjusted |
| Clostridia sp. (HG3A.0879) | HbA1c | 0.012 | 0.547 | 0.972 | 2785 | OSA and BMI adjusted |
| Clostridia sp. (HG3A.0931) | SBP | -2.49E-02 | 0.235 | 0.467 | 2334 | OSA adjusted |
| Clostridia sp. (HG3A.0931) | DBP | -3.36E-02 | 0.11 | 0.313 | 2334 | OSA adjusted |
| Clostridia sp. (HG3A.0931) | HbA1c | -2.03E-02 | 0.289 | 0.86 | 2785 | OSA adjusted |
| Clostridia sp. (HG3A.0931) | SBP | -2.24E-02 | 0.286 | 0.688 | 2334 | OSA and BMI adjusted |
| Clostridia sp. (HG3A.0931) | DBP | -3.13E-02 | 0.136 | 0.51 | 2334 | OSA and BMI adjusted |
| Clostridia sp. (HG3A.0931) | HbA1c | -2.02E-02 | 0.293 | 0.96 | 2785 | OSA and BMI adjusted |
| Clostridia sp. (HG3A.1008) | SBP | 0.016 | 0.443 | 0.667 | 2334 | OSA adjusted |
| Clostridia sp. (HG3A.1008) | DBP | 0.027 | 0.193 | 0.463 | 2334 | OSA adjusted |
| Clostridia sp. (HG3A.1008) | HbA1c | 0.025 | 0.185 | 0.822 | 2785 | OSA adjusted |

| Clostridia sp. (HG3A.1008) | SBP | 0.028 | 0.186 | 0.581 | 2334 | OSA and BMI adjusted |
| --- | --- | --- | --- | --- | --- | --- |
| Clostridia sp. (HG3A.1008) | DBP | 0.04 | 0.055 | 0.381 | 2334 | OSA and BMI adjusted |
| Clostridia sp. (HG3A.1008) | HbA1c | 0.031 | 0.105 | 0.94 | 2785 | OSA and BMI adjusted |
| Clostridia sp. (HG3A.1020) | SBP | -2.62E-02 | 0.212 | 0.461 | 2334 | OSA adjusted |
| Clostridia sp. (HG3A.1020) | DBP | -2.38E-02 | 0.258 | 0.552 | 2334 | OSA adjusted |
| Clostridia sp. (HG3A.1020) | HbA1c | 0.025 | 0.186 | 0.822 | 2785 | OSA adjusted |
| Clostridia sp. (HG3A.1020) | SBP | -1.94E-02 | 0.357 | 0.776 | 2334 | OSA and BMI adjusted |
| Clostridia sp. (HG3A.1020) | DBP | -1.65E-02 | 0.434 | 0.905 | 2334 | OSA and BMI adjusted |
| Clostridia sp. (HG3A.1020) | HbA1c | 0.03 | 0.118 | 0.94 | 2785 | OSA and BMI adjusted |
| Clostridia sp. (HG3A.1057) | SBP | -3.06E-02 | 0.145 | 0.359 | 2334 | OSA adjusted |
| Clostridia sp. (HG3A.1057) | DBP | -4.78E-02 | 0.023 | 0.116 | 2334 | OSA adjusted |
| Clostridia sp. (HG3A.1057) | HbA1c | -3.76E-02 | 0.05 | 0.647 | 2785 | OSA adjusted |
| Clostridia sp. (HG3A.1057) | SBP | -1.70E-02 | 0.418 | 0.848 | 2334 | OSA and BMI adjusted |
| Clostridia sp. (HG3A.1057) | DBP | -3.41E-02 | 0.105 | 0.462 | 2334 | OSA and BMI adjusted |
| Clostridia sp. (HG3A.1057) | HbA1c | -2.85E-02 | 0.138 | 0.94 | 2785 | OSA and BMI adjusted |
| Clostridiaceae sp. (HG3A.0431) | SBP | 0.007 | 0.73 | 0.903 | 2334 | OSA adjusted |
| Clostridiaceae sp. (HG3A.0431) | DBP | 0.005 | 0.817 | 0.932 | 2334 | OSA adjusted |
| Clostridiaceae sp. (HG3A.0431) | HbA1c | 0.004 | 0.842 | 0.922 | 2785 | OSA adjusted |
| Clostridiaceae sp. (HG3A.0431) | SBP | -3.97E-03 | 0.85 | 0.922 | 2334 | OSA and BMI adjusted |
| Clostridiaceae sp. (HG3A.0431) | DBP | -7.20E-03 | 0.732 | 0.989 | 2334 | OSA and BMI adjusted |
| Clostridiaceae sp. (HG3A.0431) | HbA1c | -4.10E-03 | 0.831 | 0.972 | 2785 | OSA and BMI adjusted |
| Clostridium sp. TF06-15AC (HG3A.0032) | SBP | 0.041 | 0.049 | 0.175 | 2334 | OSA adjusted |
| Clostridium sp. TF06-15AC (HG3A.0032) | DBP | 0.065 | 0.002 | 0.029 | 2334 | OSA adjusted |
| Clostridium sp. TF06-15AC (HG3A.0032) | HbA1c | 0.006 | 0.753 | 0.888 | 2785 | OSA adjusted |
| Clostridium sp. TF06-15AC (HG3A.0032) | SBP | 0.02 | 0.335 | 0.752 | 2334 | OSA and BMI adjusted |
| Clostridium sp. TF06-15AC (HG3A.0032) | DBP | 0.043 | 0.041 | 0.328 | 2334 | OSA and BMI adjusted |
| Clostridium sp. TF06-15AC (HG3A.0032) | HbA1c | -8.12E-03 | 0.673 | 0.972 | 2785 | OSA and BMI adjusted |
| Clostridium sp. (HG3A.0050) | SBP | 0.036 | 0.083 | 0.241 | 2334 | OSA adjusted |
| Clostridium sp. (HG3A.0050) | DBP | 0.062 | 0.003 | 0.032 | 2334 | OSA adjusted |
| Clostridium sp. (HG3A.0050) | HbA1c | 0.007 | 0.718 | 0.888 | 2785 | OSA adjusted |
| Clostridium sp. (HG3A.0050) | SBP | 0.026 | 0.208 | 0.618 | 2334 | OSA and BMI adjusted |
| Clostridium sp. (HG3A.0050) | DBP | 0.052 | 0.013 | 0.254 | 2334 | OSA and BMI adjusted |

| Clostridium sp. (HG3A.0050) | HbA1c | 0.001 | 0.954 | 0.973 | 2785 | OSA and BMI adjusted |
| --- | --- | --- | --- | --- | --- | --- |
| Collinsella aerofaciens (HG3A.0019) | SBP | 0.089 | 2.10E-05 | 0.002 | 2334 | OSA adjusted |
| Collinsella aerofaciens (HG3A.0019) | DBP | 0.064 | 0.002 | 0.03 | 2334 | OSA adjusted |
| Collinsella aerofaciens (HG3A.0019) | HbA1c | 0.013 | 0.492 | 0.875 | 2785 | OSA adjusted |
| Collinsella aerofaciens (HG3A.0019) | SBP | 0.073 | 5.02E-04 | 0.034 | 2334 | OSA and BMI adjusted |
| Collinsella aerofaciens (HG3A.0019) | DBP | 0.046 | 0.03 | 0.322 | 2334 | OSA and BMI adjusted |
| Collinsella aerofaciens (HG3A.0019) | HbA1c | 0.002 | 0.919 | 0.973 | 2785 | OSA and BMI adjusted |
| Coprobacillus sp. (HG3A.0022) | SBP | 0.004 | 0.851 | 0.926 | 2334 | OSA adjusted |
| Coprobacillus sp. (HG3A.0022) | DBP | 0.005 | 0.829 | 0.932 | 2334 | OSA adjusted |
| Coprobacillus sp. (HG3A.0022) | HbA1c | 0.003 | 0.882 | 0.928 | 2785 | OSA adjusted |
| Coprobacillus sp. (HG3A.0022) | SBP | 0.002 | 0.91 | 0.948 | 2334 | OSA and BMI adjusted |
| Coprobacillus sp. (HG3A.0022) | DBP | 0.003 | 0.891 | 0.989 | 2334 | OSA and BMI adjusted |
| Coprobacillus sp. (HG3A.0022) | HbA1c | 7.52E-04 | 0.969 | 0.973 | 2785 | OSA and BMI adjusted |
| Coprococcus comes (HG3A.0016) | SBP | 0.024 | 0.251 | 0.491 | 2334 | OSA adjusted |
| Coprococcus comes (HG3A.0016) | DBP | 0.014 | 0.508 | 0.753 | 2334 | OSA adjusted |
| Coprococcus comes (HG3A.0016) | HbA1c | 0.022 | 0.247 | 0.832 | 2785 | OSA adjusted |
| Coprococcus comes (HG3A.0016) | SBP | 0.005 | 0.797 | 0.922 | 2334 | OSA and BMI adjusted |
| Coprococcus comes (HG3A.0016) | DBP | -6.45E-03 | 0.759 | 0.989 | 2334 | OSA and BMI adjusted |
| Coprococcus comes (HG3A.0016) | HbA1c | 0.008 | 0.674 | 0.972 | 2785 | OSA and BMI adjusted |
| Coprococcus eutactus (HG3A.0155) | SBP | -6.65E-03 | 0.752 | 0.904 | 2334 | OSA adjusted |
| Coprococcus eutactus (HG3A.0155) | DBP | 0.003 | 0.892 | 0.957 | 2334 | OSA adjusted |
| Coprococcus eutactus (HG3A.0155) | HbA1c | 0.017 | 0.382 | 0.875 | 2785 | OSA adjusted |
| Coprococcus eutactus (HG3A.0155) | SBP | -6.28E-03 | 0.765 | 0.922 | 2334 | OSA and BMI adjusted |
| Coprococcus eutactus (HG3A.0155) | DBP | 0.004 | 0.864 | 0.989 | 2334 | OSA and BMI adjusted |
| Coprococcus eutactus (HG3A.0155) | HbA1c | 0.018 | 0.345 | 0.972 | 2785 | OSA and BMI adjusted |
| Dorea formicigenerans (HG3A.0006) | SBP | 0.028 | 0.18 | 0.425 | 2334 | OSA adjusted |
| Dorea formicigenerans (HG3A.0006) | DBP | 0.015 | 0.472 | 0.751 | 2334 | OSA adjusted |
| Dorea formicigenerans (HG3A.0006) | HbA1c | 0.015 | 0.445 | 0.875 | 2785 | OSA adjusted |
| Dorea formicigenerans (HG3A.0006) | SBP | 0.002 | 0.915 | 0.948 | 2334 | OSA and BMI adjusted |
| Dorea formicigenerans (HG3A.0006) | DBP | -1.31E-02 | 0.534 | 0.989 | 2334 | OSA and BMI adjusted |
| Dorea formicigenerans (HG3A.0006) | HbA1c | -2.56E-03 | 0.894 | 0.972 | 2785 | OSA and BMI adjusted |
| Dorea sp. AF36-15AT (HG3A.0052) | SBP | 0.033 | 0.114 | 0.3 | 2334 | OSA adjusted |

| Dorea sp. AF36-15AT (HG3A.0052) | DBP | 0.015 | 0.468 | 0.751 | 2334 | OSA adjusted |
| --- | --- | --- | --- | --- | --- | --- |
| Dorea sp. AF36-15AT (HG3A.0052) | HbA1c | -6.32E-03 | 0.742 | 0.888 | 2785 | OSA adjusted |
| Dorea sp. AF36-15AT (HG3A.0052) | SBP | 0.022 | 0.294 | 0.695 | 2334 | OSA and BMI adjusted |
| Dorea sp. AF36-15AT (HG3A.0052) | DBP | 0.003 | 0.899 | 0.989 | 2334 | OSA and BMI adjusted |
| Dorea sp. AF36-15AT (HG3A.0052) | HbA1c | -1.52E-02 | 0.429 | 0.972 | 2785 | OSA and BMI adjusted |
| Eggerthellaceae sp. (HG3A.0171) | SBP | -2.03E-02 | 0.335 | 0.582 | 2334 | OSA adjusted |
| Eggerthellaceae sp. (HG3A.0171) | DBP | -1.30E-02 | 0.535 | 0.753 | 2334 | OSA adjusted |
| Eggerthellaceae sp. (HG3A.0171) | HbA1c | -3.16E-02 | 0.099 | 0.73 | 2785 | OSA adjusted |
| Eggerthellaceae sp. (HG3A.0171) | SBP | -1.05E-02 | 0.619 | 0.922 | 2334 | OSA and BMI adjusted |
| Eggerthellaceae sp. (HG3A.0171) | DBP | -2.35E-03 | 0.911 | 0.989 | 2334 | OSA and BMI adjusted |
| Eggerthellaceae sp. (HG3A.0171) | HbA1c | -2.72E-02 | 0.157 | 0.94 | 2785 | OSA and BMI adjusted |
| Eggerthellales sp. (HG3A.0177) | SBP | -1.70E-02 | 0.418 | 0.653 | 2334 | OSA adjusted |
| Eggerthellales sp. (HG3A.0177) | DBP | -7.73E-03 | 0.713 | 0.873 | 2334 | OSA adjusted |
| Eggerthellales sp. (HG3A.0177) | HbA1c | 0.003 | 0.883 | 0.928 | 2785 | OSA adjusted |
| Eggerthellales sp. (HG3A.0177) | SBP | -9.93E-03 | 0.637 | 0.922 | 2334 | OSA and BMI adjusted |
| Eggerthellales sp. (HG3A.0177) | DBP | 1.95E-04 | 0.993 | 1 | 2334 | OSA and BMI adjusted |
| Eggerthellales sp. (HG3A.0177) | HbA1c | 0.008 | 0.681 | 0.972 | 2785 | OSA and BMI adjusted |
| Erysipelotrichales sp. (HG3A.1207) | SBP | 0.034 | 0.11 | 0.296 | 2334 | OSA adjusted |
| Erysipelotrichales sp. (HG3A.1207) | DBP | 0.038 | 0.072 | 0.234 | 2334 | OSA adjusted |
| Erysipelotrichales sp. (HG3A.1207) | HbA1c | -7.14E-04 | 0.97 | 0.977 | 2785 | OSA adjusted |
| Erysipelotrichales sp. (HG3A.1207) | SBP | 0.032 | 0.131 | 0.497 | 2334 | OSA and BMI adjusted |
| Erysipelotrichales sp. (HG3A.1207) | DBP | 0.036 | 0.085 | 0.45 | 2334 | OSA and BMI adjusted |
| Erysipelotrichales sp. (HG3A.1207) | HbA1c | -3.26E-03 | 0.865 | 0.972 | 2785 | OSA and BMI adjusted |
| Eubacteriales sp. (HG3A.0069) | SBP | -2.10E-02 | 0.318 | 0.567 | 2334 | OSA adjusted |
| Eubacteriales sp. (HG3A.0069) | DBP | -2.39E-02 | 0.256 | 0.552 | 2334 | OSA adjusted |
| Eubacteriales sp. (HG3A.0069) | HbA1c | -1.50E-02 | 0.433 | 0.875 | 2785 | OSA adjusted |
| Eubacteriales sp. (HG3A.0069) | SBP | -8.28E-03 | 0.694 | 0.922 | 2334 | OSA and BMI adjusted |
| Eubacteriales sp. (HG3A.0069) | DBP | -1.05E-02 | 0.618 | 0.989 | 2334 | OSA and BMI adjusted |
| Eubacteriales sp. (HG3A.0069) | HbA1c | -4.66E-03 | 0.808 | 0.972 | 2785 | OSA and BMI adjusted |
| Eubacteriales sp. (HG3A.0083) | SBP | -5.07E-02 | 0.016 | 0.077 | 2334 | OSA adjusted |
| Eubacteriales sp. (HG3A.0083) | DBP | -4.74E-02 | 0.024 | 0.116 | 2334 | OSA adjusted |
| Eubacteriales sp. (HG3A.0083) | HbA1c | -7.73E-03 | 0.687 | 0.888 | 2785 | OSA adjusted |

| Eubacteriales sp. (HG3A.0083) | SBP | -2.52E-02 | 0.231 | 0.622 | 2334 | OSA and BMI adjusted |
| --- | --- | --- | --- | --- | --- | --- |
| Eubacteriales sp. (HG3A.0083) | DBP | -2.01E-02 | 0.338 | 0.872 | 2334 | OSA and BMI adjusted |
| Eubacteriales sp. (HG3A.0083) | HbA1c | 0.008 | 0.681 | 0.972 | 2785 | OSA and BMI adjusted |
| Eubacteriales sp. (HG3A.0084) | SBP | -6.63E-02 | 0.002 | 0.02 | 2334 | OSA adjusted |
| Eubacteriales sp. (HG3A.0084) | DBP | -5.82E-02 | 0.006 | 0.051 | 2334 | OSA adjusted |
| Eubacteriales sp. (HG3A.0084) | HbA1c | -2.70E-02 | 0.16 | 0.822 | 2785 | OSA adjusted |
| Eubacteriales sp. (HG3A.0084) | SBP | -5.68E-02 | 0.007 | 0.157 | 2334 | OSA and BMI adjusted |
| Eubacteriales sp. (HG3A.0084) | DBP | -4.79E-02 | 0.023 | 0.305 | 2334 | OSA and BMI adjusted |
| Eubacteriales sp. (HG3A.0084) | HbA1c | -2.06E-02 | 0.283 | 0.96 | 2785 | OSA and BMI adjusted |
| Eubacteriales sp. (HG3A.0085) | SBP | -6.39E-02 | 0.002 | 0.024 | 2334 | OSA adjusted |
| Eubacteriales sp. (HG3A.0085) | DBP | -5.29E-02 | 0.012 | 0.07 | 2334 | OSA adjusted |
| Eubacteriales sp. (HG3A.0085) | HbA1c | -2.89E-02 | 0.132 | 0.82 | 2785 | OSA adjusted |
| Eubacteriales sp. (HG3A.0085) | SBP | -5.19E-02 | 0.014 | 0.168 | 2334 | OSA and BMI adjusted |
| Eubacteriales sp. (HG3A.0085) | DBP | -3.98E-02 | 0.058 | 0.381 | 2334 | OSA and BMI adjusted |
| Eubacteriales sp. (HG3A.0085) | HbA1c | -2.26E-02 | 0.24 | 0.94 | 2785 | OSA and BMI adjusted |
| Eubacteriales sp. (HG3A.0100) | SBP | -7.89E-02 | 1.72E-04 | 0.004 | 2334 | OSA adjusted |
| Eubacteriales sp. (HG3A.0100) | DBP | -7.57E-02 | 3.14E-04 | 0.008 | 2334 | OSA adjusted |
| Eubacteriales sp. (HG3A.0100) | HbA1c | -1.05E-02 | 0.586 | 0.882 | 2785 | OSA adjusted |
| Eubacteriales sp. (HG3A.0100) | SBP | -6.30E-02 | 0.003 | 0.093 | 2334 | OSA and BMI adjusted |
| Eubacteriales sp. (HG3A.0100) | DBP | -5.88E-02 | 0.005 | 0.147 | 2334 | OSA and BMI adjusted |
| Eubacteriales sp. (HG3A.0100) | HbA1c | 8.12E-04 | 0.966 | 0.973 | 2785 | OSA and BMI adjusted |
| Eubacteriales sp. (HG3A.0118) | SBP | -4.09E-02 | 0.052 | 0.181 | 2334 | OSA adjusted |
| Eubacteriales sp. (HG3A.0118) | DBP | -3.52E-02 | 0.094 | 0.294 | 2334 | OSA adjusted |
| Eubacteriales sp. (HG3A.0118) | HbA1c | -3.77E-02 | 0.049 | 0.647 | 2785 | OSA adjusted |
| Eubacteriales sp. (HG3A.0118) | SBP | -1.82E-02 | 0.386 | 0.827 | 2334 | OSA and BMI adjusted |
| Eubacteriales sp. (HG3A.0118) | DBP | -1.08E-02 | 0.608 | 0.989 | 2334 | OSA and BMI adjusted |
| Eubacteriales sp. (HG3A.0118) | HbA1c | -2.39E-02 | 0.213 | 0.94 | 2785 | OSA and BMI adjusted |
| Eubacteriales sp. (HG3A.0120) | SBP | -6.84E-02 | 0.001 | 0.02 | 2334 | OSA adjusted |
| Eubacteriales sp. (HG3A.0120) | DBP | -6.48E-02 | 0.002 | 0.029 | 2334 | OSA adjusted |
| Eubacteriales sp. (HG3A.0120) | HbA1c | -4.15E-02 | 0.03 | 0.647 | 2785 | OSA adjusted |
| Eubacteriales sp. (HG3A.0120) | SBP | -5.35E-02 | 0.011 | 0.168 | 2334 | OSA and BMI adjusted |
| Eubacteriales sp. (HG3A.0120) | DBP | -4.90E-02 | 0.02 | 0.305 | 2334 | OSA and BMI adjusted |

| Eubacteriales sp. (HG3A.0120) | HbA1c | -3.13E-02 | 0.103 | 0.94 | 2785 | OSA and BMI adjusted |
| --- | --- | --- | --- | --- | --- | --- |
| Eubacteriales sp. (HG3A.0123) | SBP | 0.012 | 0.568 | 0.778 | 2334 | OSA adjusted |
| Eubacteriales sp. (HG3A.0123) | DBP | 0.003 | 0.878 | 0.956 | 2334 | OSA adjusted |
| Eubacteriales sp. (HG3A.0123) | HbA1c | -2.66E-02 | 0.166 | 0.822 | 2785 | OSA adjusted |
| Eubacteriales sp. (HG3A.0123) | SBP | 0.012 | 0.579 | 0.922 | 2334 | OSA and BMI adjusted |
| Eubacteriales sp. (HG3A.0123) | DBP | 0.003 | 0.903 | 0.989 | 2334 | OSA and BMI adjusted |
| Eubacteriales sp. (HG3A.0123) | HbA1c | -2.80E-02 | 0.145 | 0.94 | 2785 | OSA and BMI adjusted |
| Eubacteriales sp. (HG3A.0149) | SBP | -5.60E-02 | 0.008 | 0.049 | 2334 | OSA adjusted |
| Eubacteriales sp. (HG3A.0149) | DBP | -6.86E-02 | 0.001 | 0.019 | 2334 | OSA adjusted |
| Eubacteriales sp. (HG3A.0149) | HbA1c | -1.19E-02 | 0.535 | 0.882 | 2785 | OSA adjusted |
| Eubacteriales sp. (HG3A.0149) | SBP | -3.25E-02 | 0.122 | 0.497 | 2334 | OSA and BMI adjusted |
| Eubacteriales sp. (HG3A.0149) | DBP | -4.43E-02 | 0.035 | 0.322 | 2334 | OSA and BMI adjusted |
| Eubacteriales sp. (HG3A.0149) | HbA1c | 0.004 | 0.853 | 0.972 | 2785 | OSA and BMI adjusted |
| Eubacteriales sp. (HG3A.0153) | SBP | -1.49E-02 | 0.479 | 0.698 | 2334 | OSA adjusted |
| Eubacteriales sp. (HG3A.0153) | DBP | 0.002 | 0.925 | 0.957 | 2334 | OSA adjusted |
| Eubacteriales sp. (HG3A.0153) | HbA1c | 0.003 | 0.865 | 0.928 | 2785 | OSA adjusted |
| Eubacteriales sp. (HG3A.0153) | SBP | -1.18E-02 | 0.574 | 0.922 | 2334 | OSA and BMI adjusted |
| Eubacteriales sp. (HG3A.0153) | DBP | 0.006 | 0.779 | 0.989 | 2334 | OSA and BMI adjusted |
| Eubacteriales sp. (HG3A.0153) | HbA1c | 0.008 | 0.694 | 0.972 | 2785 | OSA and BMI adjusted |
| Eubacteriales sp. (HG3A.0156) | SBP | -4.16E-03 | 0.843 | 0.926 | 2334 | OSA adjusted |
| Eubacteriales sp. (HG3A.0156) | DBP | -1.81E-02 | 0.39 | 0.702 | 2334 | OSA adjusted |
| Eubacteriales sp. (HG3A.0156) | HbA1c | -1.24E-02 | 0.517 | 0.875 | 2785 | OSA adjusted |
| Eubacteriales sp. (HG3A.0156) | SBP | 0.012 | 0.561 | 0.922 | 2334 | OSA and BMI adjusted |
| Eubacteriales sp. (HG3A.0156) | DBP | -1.18E-03 | 0.955 | 0.989 | 2334 | OSA and BMI adjusted |
| Eubacteriales sp. (HG3A.0156) | HbA1c | -3.67E-03 | 0.849 | 0.972 | 2785 | OSA and BMI adjusted |
| Eubacteriales sp. (HG3A.0162) | SBP | -6.08E-02 | 0.004 | 0.031 | 2334 | OSA adjusted |
| Eubacteriales sp. (HG3A.0162) | DBP | -5.35E-02 | 0.011 | 0.068 | 2334 | OSA adjusted |
| Eubacteriales sp. (HG3A.0162) | HbA1c | -3.76E-02 | 0.05 | 0.647 | 2785 | OSA adjusted |
| Eubacteriales sp. (HG3A.0162) | SBP | -4.25E-02 | 0.043 | 0.327 | 2334 | OSA and BMI adjusted |
| Eubacteriales sp. (HG3A.0162) | DBP | -3.38E-02 | 0.108 | 0.462 | 2334 | OSA and BMI adjusted |
| Eubacteriales sp. (HG3A.0162) | HbA1c | -2.40E-02 | 0.212 | 0.94 | 2785 | OSA and BMI adjusted |
| Eubacteriales sp. (HG3A.0193) | SBP | -5.59E-02 | 0.008 | 0.049 | 2334 | OSA adjusted |

| Eubacteriales sp. (HG3A.0193) | DBP | -5.61E-02 | 0.008 | 0.062 | 2334 | OSA adjusted |
| --- | --- | --- | --- | --- | --- | --- |
| Eubacteriales sp. (HG3A.0193) | HbA1c | -3.10E-02 | 0.106 | 0.73 | 2785 | OSA adjusted |
| Eubacteriales sp. (HG3A.0193) | SBP | -3.59E-02 | 0.087 | 0.455 | 2334 | OSA and BMI adjusted |
| Eubacteriales sp. (HG3A.0193) | DBP | -3.50E-02 | 0.096 | 0.462 | 2334 | OSA and BMI adjusted |
| Eubacteriales sp. (HG3A.0193) | HbA1c | -1.77E-02 | 0.355 | 0.972 | 2785 | OSA and BMI adjusted |
| Eubacteriales sp. (HG3A.0196) | SBP | -8.25E-02 | 8.46E-05 | 0.002 | 2334 | OSA adjusted |
| Eubacteriales sp. (HG3A.0196) | DBP | -8.63E-02 | 3.94E-05 | 0.004 | 2334 | OSA adjusted |
| Eubacteriales sp. (HG3A.0196) | HbA1c | 0.02 | 0.308 | 0.86 | 2785 | OSA adjusted |
| Eubacteriales sp. (HG3A.0196) | SBP | -7.73E-02 | 2.32E-04 | 0.032 | 2334 | OSA and BMI adjusted |
| Eubacteriales sp. (HG3A.0196) | DBP | -8.11E-02 | 1.13E-04 | 0.015 | 2334 | OSA and BMI adjusted |
| Eubacteriales sp. (HG3A.0196) | HbA1c | 0.024 | 0.213 | 0.94 | 2785 | OSA and BMI adjusted |
| Eubacteriales sp. (HG3A.0197) | SBP | -1.61E-02 | 0.444 | 0.667 | 2334 | OSA adjusted |
| Eubacteriales sp. (HG3A.0197) | DBP | -1.23E-02 | 0.557 | 0.753 | 2334 | OSA adjusted |
| Eubacteriales sp. (HG3A.0197) | HbA1c | -5.66E-03 | 0.768 | 0.888 | 2785 | OSA adjusted |
| Eubacteriales sp. (HG3A.0197) | SBP | -5.56E-03 | 0.792 | 0.922 | 2334 | OSA and BMI adjusted |
| Eubacteriales sp. (HG3A.0197) | DBP | -9.89E-04 | 0.962 | 0.989 | 2334 | OSA and BMI adjusted |
| Eubacteriales sp. (HG3A.0197) | HbA1c | 0.003 | 0.871 | 0.972 | 2785 | OSA and BMI adjusted |
| Eubacteriales sp. (HG3A.0211) | SBP | -8.38E-04 | 0.968 | 0.973 | 2334 | OSA adjusted |
| Eubacteriales sp. (HG3A.0211) | DBP | 0.005 | 0.813 | 0.932 | 2334 | OSA adjusted |
| Eubacteriales sp. (HG3A.0211) | HbA1c | -4.55E-02 | 0.018 | 0.647 | 2785 | OSA adjusted |
| Eubacteriales sp. (HG3A.0211) | SBP | 0.025 | 0.225 | 0.618 | 2334 | OSA and BMI adjusted |
| Eubacteriales sp. (HG3A.0211) | DBP | 0.033 | 0.113 | 0.465 | 2334 | OSA and BMI adjusted |
| Eubacteriales sp. (HG3A.0211) | HbA1c | -2.99E-02 | 0.12 | 0.94 | 2785 | OSA and BMI adjusted |
| Eubacteriales sp. (HG3A.0215) | SBP | -1.80E-02 | 0.393 | 0.649 | 2334 | OSA adjusted |
| Eubacteriales sp. (HG3A.0215) | DBP | -1.08E-02 | 0.607 | 0.785 | 2334 | OSA adjusted |
| Eubacteriales sp. (HG3A.0215) | HbA1c | -1.34E-02 | 0.484 | 0.875 | 2785 | OSA adjusted |
| Eubacteriales sp. (HG3A.0215) | SBP | -6.70E-03 | 0.75 | 0.922 | 2334 | OSA and BMI adjusted |
| Eubacteriales sp. (HG3A.0215) | DBP | 0.001 | 0.945 | 0.989 | 2334 | OSA and BMI adjusted |
| Eubacteriales sp. (HG3A.0215) | HbA1c | -4.84E-03 | 0.801 | 0.972 | 2785 | OSA and BMI adjusted |
| Eubacteriales sp. (HG3A.0226) | SBP | -2.23E-02 | 0.289 | 0.536 | 2334 | OSA adjusted |
| Eubacteriales sp. (HG3A.0226) | DBP | -2.11E-02 | 0.315 | 0.644 | 2334 | OSA adjusted |
| Eubacteriales sp. (HG3A.0226) | HbA1c | 0.016 | 0.409 | 0.875 | 2785 | OSA adjusted |

| Eubacteriales sp. (HG3A.0226) | SBP | -6.41E-03 | 0.76 | 0.922 | 2334 | OSA and BMI adjusted |
| --- | --- | --- | --- | --- | --- | --- |
| Eubacteriales sp. (HG3A.0226) | DBP | -4.20E-03 | 0.842 | 0.989 | 2334 | OSA and BMI adjusted |
| Eubacteriales sp. (HG3A.0226) | HbA1c | 0.025 | 0.2 | 0.94 | 2785 | OSA and BMI adjusted |
| Eubacteriales sp. (HG3A.0229) | SBP | -2.11E-02 | 0.315 | 0.567 | 2334 | OSA adjusted |
| Eubacteriales sp. (HG3A.0229) | DBP | -8.74E-03 | 0.678 | 0.852 | 2334 | OSA adjusted |
| Eubacteriales sp. (HG3A.0229) | HbA1c | -1.19E-02 | 0.536 | 0.882 | 2785 | OSA adjusted |
| Eubacteriales sp. (HG3A.0229) | SBP | -8.01E-03 | 0.703 | 0.922 | 2334 | OSA and BMI adjusted |
| Eubacteriales sp. (HG3A.0229) | DBP | 0.006 | 0.787 | 0.989 | 2334 | OSA and BMI adjusted |
| Eubacteriales sp. (HG3A.0229) | HbA1c | -2.71E-03 | 0.888 | 0.972 | 2785 | OSA and BMI adjusted |
| Eubacteriales sp. (HG3A.0234) | SBP | -1.63E-02 | 0.437 | 0.667 | 2334 | OSA adjusted |
| Eubacteriales sp. (HG3A.0234) | DBP | -3.04E-02 | 0.149 | 0.385 | 2334 | OSA adjusted |
| Eubacteriales sp. (HG3A.0234) | HbA1c | 0.02 | 0.287 | 0.86 | 2785 | OSA adjusted |
| Eubacteriales sp. (HG3A.0234) | SBP | -5.85E-03 | 0.781 | 0.922 | 2334 | OSA and BMI adjusted |
| Eubacteriales sp. (HG3A.0234) | DBP | -1.98E-02 | 0.347 | 0.872 | 2334 | OSA and BMI adjusted |
| Eubacteriales sp. (HG3A.0234) | HbA1c | 0.026 | 0.172 | 0.94 | 2785 | OSA and BMI adjusted |
| Eubacteriales sp. (HG3A.0242) | SBP | -4.58E-02 | 0.029 | 0.122 | 2334 | OSA adjusted |
| Eubacteriales sp. (HG3A.0242) | DBP | -4.62E-02 | 0.028 | 0.127 | 2334 | OSA adjusted |
| Eubacteriales sp. (HG3A.0242) | HbA1c | -1.97E-03 | 0.918 | 0.947 | 2785 | OSA adjusted |
| Eubacteriales sp. (HG3A.0242) | SBP | -2.61E-02 | 0.214 | 0.618 | 2334 | OSA and BMI adjusted |
| Eubacteriales sp. (HG3A.0242) | DBP | -2.53E-02 | 0.228 | 0.744 | 2334 | OSA and BMI adjusted |
| Eubacteriales sp. (HG3A.0242) | HbA1c | 0.012 | 0.523 | 0.972 | 2785 | OSA and BMI adjusted |
| Eubacteriales sp. (HG3A.0250) | SBP | -4.17E-02 | 0.047 | 0.175 | 2334 | OSA adjusted |
| Eubacteriales sp. (HG3A.0250) | DBP | -3.58E-02 | 0.089 | 0.282 | 2334 | OSA adjusted |
| Eubacteriales sp. (HG3A.0250) | HbA1c | -5.25E-03 | 0.785 | 0.888 | 2785 | OSA adjusted |
| Eubacteriales sp. (HG3A.0250) | SBP | -3.45E-02 | 0.101 | 0.494 | 2334 | OSA and BMI adjusted |
| Eubacteriales sp. (HG3A.0250) | DBP | -2.80E-02 | 0.183 | 0.627 | 2334 | OSA and BMI adjusted |
| Eubacteriales sp. (HG3A.0250) | HbA1c | -1.01E-03 | 0.958 | 0.973 | 2785 | OSA and BMI adjusted |
| Eubacteriales sp. (HG3A.0269) | SBP | -3.86E-02 | 0.066 | 0.21 | 2334 | OSA adjusted |
| Eubacteriales sp. (HG3A.0269) | DBP | -4.48E-02 | 0.033 | 0.134 | 2334 | OSA adjusted |
| Eubacteriales sp. (HG3A.0269) | HbA1c | -1.75E-02 | 0.362 | 0.875 | 2785 | OSA adjusted |
| Eubacteriales sp. (HG3A.0269) | SBP | -2.56E-02 | 0.223 | 0.618 | 2334 | OSA and BMI adjusted |
| Eubacteriales sp. (HG3A.0269) | DBP | -3.12E-02 | 0.138 | 0.51 | 2334 | OSA and BMI adjusted |

| Eubacteriales sp. (HG3A.0269) | HbA1c | -8.51E-03 | 0.658 | 0.972 | 2785 | OSA and BMI adjusted |
| --- | --- | --- | --- | --- | --- | --- |
| Eubacteriales sp. (HG3A.0291) | SBP | -1.99E-02 | 0.345 | 0.59 | 2334 | OSA adjusted |
| Eubacteriales sp. (HG3A.0291) | DBP | -2.71E-02 | 0.198 | 0.463 | 2334 | OSA adjusted |
| Eubacteriales sp. (HG3A.0291) | HbA1c | 0.009 | 0.63 | 0.882 | 2785 | OSA adjusted |
| Eubacteriales sp. (HG3A.0291) | SBP | -1.63E-02 | 0.439 | 0.848 | 2334 | OSA and BMI adjusted |
| Eubacteriales sp. (HG3A.0291) | DBP | -2.36E-02 | 0.262 | 0.765 | 2334 | OSA and BMI adjusted |
| Eubacteriales sp. (HG3A.0291) | HbA1c | 0.012 | 0.529 | 0.972 | 2785 | OSA and BMI adjusted |
| Eubacteriales sp. (HG3A.0309) | SBP | -1.70E-02 | 0.419 | 0.653 | 2334 | OSA adjusted |
| Eubacteriales sp. (HG3A.0309) | DBP | -1.40E-02 | 0.506 | 0.753 | 2334 | OSA adjusted |
| Eubacteriales sp. (HG3A.0309) | HbA1c | 0.013 | 0.501 | 0.875 | 2785 | OSA adjusted |
| Eubacteriales sp. (HG3A.0309) | SBP | -1.04E-02 | 0.619 | 0.922 | 2334 | OSA and BMI adjusted |
| Eubacteriales sp. (HG3A.0309) | DBP | -6.95E-03 | 0.741 | 0.989 | 2334 | OSA and BMI adjusted |
| Eubacteriales sp. (HG3A.0309) | HbA1c | 0.017 | 0.369 | 0.972 | 2785 | OSA and BMI adjusted |
| Eubacteriales sp. (HG3A.0311) | SBP | -8.54E-02 | 4.68E-05 | 0.002 | 2334 | OSA adjusted |
| Eubacteriales sp. (HG3A.0311) | DBP | -8.45E-02 | 5.63E-05 | 0.004 | 2334 | OSA adjusted |
| Eubacteriales sp. (HG3A.0311) | HbA1c | -4.18E-02 | 0.029 | 0.647 | 2785 | OSA adjusted |
| Eubacteriales sp. (HG3A.0311) | SBP | -5.07E-02 | 0.016 | 0.168 | 2334 | OSA and BMI adjusted |
| Eubacteriales sp. (HG3A.0311) | DBP | -4.75E-02 | 0.024 | 0.305 | 2334 | OSA and BMI adjusted |
| Eubacteriales sp. (HG3A.0311) | HbA1c | -1.97E-02 | 0.304 | 0.96 | 2785 | OSA and BMI adjusted |
| Eubacteriales sp. (HG3A.0321) | SBP | -2.51E-02 | 0.233 | 0.467 | 2334 | OSA adjusted |
| Eubacteriales sp. (HG3A.0321) | DBP | -1.27E-02 | 0.545 | 0.753 | 2334 | OSA adjusted |
| Eubacteriales sp. (HG3A.0321) | HbA1c | -1.12E-02 | 0.56 | 0.882 | 2785 | OSA adjusted |
| Eubacteriales sp. (HG3A.0321) | SBP | -1.43E-02 | 0.497 | 0.893 | 2334 | OSA and BMI adjusted |
| Eubacteriales sp. (HG3A.0321) | DBP | -8.04E-04 | 0.97 | 0.989 | 2334 | OSA and BMI adjusted |
| Eubacteriales sp. (HG3A.0321) | HbA1c | -1.78E-03 | 0.926 | 0.973 | 2785 | OSA and BMI adjusted |
| Eubacteriales sp. (HG3A.0329) | SBP | -5.36E-03 | 0.799 | 0.904 | 2334 | OSA adjusted |
| Eubacteriales sp. (HG3A.0329) | DBP | -8.81E-03 | 0.675 | 0.852 | 2334 | OSA adjusted |
| Eubacteriales sp. (HG3A.0329) | HbA1c | -1.59E-02 | 0.408 | 0.875 | 2785 | OSA adjusted |
| Eubacteriales sp. (HG3A.0329) | SBP | 0.004 | 0.834 | 0.922 | 2334 | OSA and BMI adjusted |
| Eubacteriales sp. (HG3A.0329) | DBP | 0.001 | 0.945 | 0.989 | 2334 | OSA and BMI adjusted |
| Eubacteriales sp. (HG3A.0329) | HbA1c | -9.17E-03 | 0.633 | 0.972 | 2785 | OSA and BMI adjusted |
| Eubacteriales sp. (HG3A.0331) | SBP | -5.90E-02 | 0.005 | 0.038 | 2334 | OSA adjusted |

| Eubacteriales sp. (HG3A.0331) | DBP | -5.21E-02 | 0.013 | 0.076 | 2334 | OSA adjusted |
| --- | --- | --- | --- | --- | --- | --- |
| Eubacteriales sp. (HG3A.0331) | HbA1c | -2.00E-02 | 0.298 | 0.86 | 2785 | OSA adjusted |
| Eubacteriales sp. (HG3A.0331) | SBP | -3.84E-02 | 0.067 | 0.37 | 2334 | OSA and BMI adjusted |
| Eubacteriales sp. (HG3A.0331) | DBP | -3.00E-02 | 0.154 | 0.554 | 2334 | OSA and BMI adjusted |
| Eubacteriales sp. (HG3A.0331) | HbA1c | -5.75E-03 | 0.765 | 0.972 | 2785 | OSA and BMI adjusted |
| Eubacteriales sp. (HG3A.0383) | SBP | -1.70E-02 | 0.418 | 0.653 | 2334 | OSA adjusted |
| Eubacteriales sp. (HG3A.0383) | DBP | -8.28E-03 | 0.694 | 0.856 | 2334 | OSA adjusted |
| Eubacteriales sp. (HG3A.0383) | HbA1c | 0.005 | 0.78 | 0.888 | 2785 | OSA adjusted |
| Eubacteriales sp. (HG3A.0383) | SBP | 0.004 | 0.862 | 0.922 | 2334 | OSA and BMI adjusted |
| Eubacteriales sp. (HG3A.0383) | DBP | 0.014 | 0.502 | 0.986 | 2334 | OSA and BMI adjusted |
| Eubacteriales sp. (HG3A.0383) | HbA1c | 0.019 | 0.317 | 0.96 | 2785 | OSA and BMI adjusted |
| Eubacteriales sp. (HG3A.0419) | SBP | -3.83E-02 | 0.069 | 0.214 | 2334 | OSA adjusted |
| Eubacteriales sp. (HG3A.0419) | DBP | -4.00E-02 | 0.057 | 0.195 | 2334 | OSA adjusted |
| Eubacteriales sp. (HG3A.0419) | HbA1c | -1.93E-02 | 0.314 | 0.86 | 2785 | OSA adjusted |
| Eubacteriales sp. (HG3A.0419) | SBP | -1.67E-02 | 0.427 | 0.848 | 2334 | OSA and BMI adjusted |
| Eubacteriales sp. (HG3A.0419) | DBP | -1.72E-02 | 0.415 | 0.905 | 2334 | OSA and BMI adjusted |
| Eubacteriales sp. (HG3A.0419) | HbA1c | -6.07E-03 | 0.752 | 0.972 | 2785 | OSA and BMI adjusted |
| Eubacteriales sp. (HG3A.0421) | SBP | -1.77E-02 | 0.4 | 0.652 | 2334 | OSA adjusted |
| Eubacteriales sp. (HG3A.0421) | DBP | -1.25E-02 | 0.552 | 0.753 | 2334 | OSA adjusted |
| Eubacteriales sp. (HG3A.0421) | HbA1c | -3.59E-02 | 0.062 | 0.647 | 2785 | OSA adjusted |
| Eubacteriales sp. (HG3A.0421) | SBP | 0.007 | 0.742 | 0.922 | 2334 | OSA and BMI adjusted |
| Eubacteriales sp. (HG3A.0421) | DBP | 0.014 | 0.506 | 0.986 | 2334 | OSA and BMI adjusted |
| Eubacteriales sp. (HG3A.0421) | HbA1c | -2.26E-02 | 0.239 | 0.94 | 2785 | OSA and BMI adjusted |
| Eubacteriales sp. (HG3A.0439) | SBP | 0.016 | 0.448 | 0.667 | 2334 | OSA adjusted |
| Eubacteriales sp. (HG3A.0439) | DBP | 0.017 | 0.419 | 0.709 | 2334 | OSA adjusted |
| Eubacteriales sp. (HG3A.0439) | HbA1c | -1.59E-02 | 0.407 | 0.875 | 2785 | OSA adjusted |
| Eubacteriales sp. (HG3A.0439) | SBP | 0.034 | 0.105 | 0.496 | 2334 | OSA and BMI adjusted |
| Eubacteriales sp. (HG3A.0439) | DBP | 0.036 | 0.083 | 0.45 | 2334 | OSA and BMI adjusted |
| Eubacteriales sp. (HG3A.0439) | HbA1c | -4.33E-03 | 0.822 | 0.972 | 2785 | OSA and BMI adjusted |
| Eubacteriales sp. (HG3A.0442) | SBP | -1.86E-03 | 0.929 | 0.954 | 2334 | OSA adjusted |
| Eubacteriales sp. (HG3A.0442) | DBP | -1.25E-02 | 0.551 | 0.753 | 2334 | OSA adjusted |
| Eubacteriales sp. (HG3A.0442) | HbA1c | 0.019 | 0.313 | 0.86 | 2785 | OSA adjusted |

| Eubacteriales sp. (HG3A.0442) | SBP | 0.006 | 0.764 | 0.922 | 2334 | OSA and BMI adjusted |
| --- | --- | --- | --- | --- | --- | --- |
| Eubacteriales sp. (HG3A.0442) | DBP | -4.22E-03 | 0.841 | 0.989 | 2334 | OSA and BMI adjusted |
| Eubacteriales sp. (HG3A.0442) | HbA1c | 0.026 | 0.179 | 0.94 | 2785 | OSA and BMI adjusted |
| Eubacteriales sp. (HG3A.0468) | SBP | -1.58E-03 | 0.94 | 0.954 | 2334 | OSA adjusted |
| Eubacteriales sp. (HG3A.0468) | DBP | -3.38E-03 | 0.872 | 0.956 | 2334 | OSA adjusted |
| Eubacteriales sp. (HG3A.0468) | HbA1c | 0.007 | 0.713 | 0.888 | 2785 | OSA adjusted |
| Eubacteriales sp. (HG3A.0468) | SBP | 0.001 | 0.961 | 0.968 | 2334 | OSA and BMI adjusted |
| Eubacteriales sp. (HG3A.0468) | DBP | -6.83E-04 | 0.974 | 0.989 | 2334 | OSA and BMI adjusted |
| Eubacteriales sp. (HG3A.0468) | HbA1c | 0.01 | 0.602 | 0.972 | 2785 | OSA and BMI adjusted |
| Eubacteriales sp. (HG3A.0489) | SBP | -2.54E-02 | 0.226 | 0.467 | 2334 | OSA adjusted |
| Eubacteriales sp. (HG3A.0489) | DBP | -2.23E-02 | 0.288 | 0.599 | 2334 | OSA adjusted |
| Eubacteriales sp. (HG3A.0489) | HbA1c | -2.58E-02 | 0.179 | 0.822 | 2785 | OSA adjusted |
| Eubacteriales sp. (HG3A.0489) | SBP | -1.12E-02 | 0.593 | 0.922 | 2334 | OSA and BMI adjusted |
| Eubacteriales sp. (HG3A.0489) | DBP | -7.12E-03 | 0.735 | 0.989 | 2334 | OSA and BMI adjusted |
| Eubacteriales sp. (HG3A.0489) | HbA1c | -1.64E-02 | 0.394 | 0.972 | 2785 | OSA and BMI adjusted |
| Eubacteriales sp. (HG3A.0506) | SBP | -1.54E-02 | 0.465 | 0.685 | 2334 | OSA adjusted |
| Eubacteriales sp. (HG3A.0506) | DBP | 0.001 | 0.951 | 0.966 | 2334 | OSA adjusted |
| Eubacteriales sp. (HG3A.0506) | HbA1c | -1.16E-02 | 0.545 | 0.882 | 2785 | OSA adjusted |
| Eubacteriales sp. (HG3A.0506) | SBP | -1.80E-03 | 0.932 | 0.953 | 2334 | OSA and BMI adjusted |
| Eubacteriales sp. (HG3A.0506) | DBP | 0.016 | 0.436 | 0.905 | 2334 | OSA and BMI adjusted |
| Eubacteriales sp. (HG3A.0506) | HbA1c | -3.20E-03 | 0.867 | 0.972 | 2785 | OSA and BMI adjusted |
| Eubacteriales sp. (HG3A.0531) | SBP | -5.81E-02 | 0.006 | 0.041 | 2334 | OSA adjusted |
| Eubacteriales sp. (HG3A.0531) | DBP | -5.16E-02 | 0.014 | 0.077 | 2334 | OSA adjusted |
| Eubacteriales sp. (HG3A.0531) | HbA1c | 0.014 | 0.475 | 0.875 | 2785 | OSA adjusted |
| Eubacteriales sp. (HG3A.0531) | SBP | -4.09E-02 | 0.052 | 0.328 | 2334 | OSA and BMI adjusted |
| Eubacteriales sp. (HG3A.0531) | DBP | -3.31E-02 | 0.115 | 0.465 | 2334 | OSA and BMI adjusted |
| Eubacteriales sp. (HG3A.0531) | HbA1c | 0.028 | 0.149 | 0.94 | 2785 | OSA and BMI adjusted |
| Eubacteriales sp. (HG3A.0548) | SBP | 0.011 | 0.597 | 0.794 | 2334 | OSA adjusted |
| Eubacteriales sp. (HG3A.0548) | DBP | 0.033 | 0.118 | 0.324 | 2334 | OSA adjusted |
| Eubacteriales sp. (HG3A.0548) | HbA1c | -1.94E-03 | 0.92 | 0.947 | 2785 | OSA adjusted |
| Eubacteriales sp. (HG3A.0548) | SBP | 0.024 | 0.258 | 0.65 | 2334 | OSA and BMI adjusted |
| Eubacteriales sp. (HG3A.0548) | DBP | 0.047 | 0.025 | 0.305 | 2334 | OSA and BMI adjusted |

| Eubacteriales sp. (HG3A.0548) | HbA1c | 0.006 | 0.772 | 0.972 | 2785 | OSA and BMI adjusted |
| --- | --- | --- | --- | --- | --- | --- |
| Eubacteriales sp. (HG3A.0572) | SBP | 0.002 | 0.913 | 0.954 | 2334 | OSA adjusted |
| Eubacteriales sp. (HG3A.0572) | DBP | -5.85E-03 | 0.781 | 0.93 | 2334 | OSA adjusted |
| Eubacteriales sp. (HG3A.0572) | HbA1c | 0.013 | 0.507 | 0.875 | 2785 | OSA adjusted |
| Eubacteriales sp. (HG3A.0572) | SBP | 0.013 | 0.549 | 0.922 | 2334 | OSA and BMI adjusted |
| Eubacteriales sp. (HG3A.0572) | DBP | 0.005 | 0.818 | 0.989 | 2334 | OSA and BMI adjusted |
| Eubacteriales sp. (HG3A.0572) | HbA1c | 0.02 | 0.289 | 0.96 | 2785 | OSA and BMI adjusted |
| Eubacteriales sp. (HG3A.0609) | SBP | -2.18E-03 | 0.917 | 0.954 | 2334 | OSA adjusted |
| Eubacteriales sp. (HG3A.0609) | DBP | -1.33E-02 | 0.527 | 0.753 | 2334 | OSA adjusted |
| Eubacteriales sp. (HG3A.0609) | HbA1c | -1.08E-02 | 0.575 | 0.882 | 2785 | OSA adjusted |
| Eubacteriales sp. (HG3A.0609) | SBP | 0.006 | 0.79 | 0.922 | 2334 | OSA and BMI adjusted |
| Eubacteriales sp. (HG3A.0609) | DBP | -5.47E-03 | 0.795 | 0.989 | 2334 | OSA and BMI adjusted |
| Eubacteriales sp. (HG3A.0609) | HbA1c | -6.85E-03 | 0.721 | 0.972 | 2785 | OSA and BMI adjusted |
| Eubacteriales sp. (HG3A.0630) | SBP | -1.45E-02 | 0.49 | 0.706 | 2334 | OSA adjusted |
| Eubacteriales sp. (HG3A.0630) | DBP | -2.35E-02 | 0.264 | 0.556 | 2334 | OSA adjusted |
| Eubacteriales sp. (HG3A.0630) | HbA1c | -2.18E-02 | 0.255 | 0.832 | 2785 | OSA adjusted |
| Eubacteriales sp. (HG3A.0630) | SBP | -3.22E-04 | 0.988 | 0.988 | 2334 | OSA and BMI adjusted |
| Eubacteriales sp. (HG3A.0630) | DBP | -8.74E-03 | 0.678 | 0.989 | 2334 | OSA and BMI adjusted |
| Eubacteriales sp. (HG3A.0630) | HbA1c | -1.34E-02 | 0.484 | 0.972 | 2785 | OSA and BMI adjusted |
| Eubacteriales sp. (HG3A.0635) | SBP | 0.026 | 0.211 | 0.461 | 2334 | OSA adjusted |
| Eubacteriales sp. (HG3A.0635) | DBP | 0.027 | 0.192 | 0.463 | 2334 | OSA adjusted |
| Eubacteriales sp. (HG3A.0635) | HbA1c | -2.20E-02 | 0.252 | 0.832 | 2785 | OSA adjusted |
| Eubacteriales sp. (HG3A.0635) | SBP | 0.042 | 0.045 | 0.327 | 2334 | OSA and BMI adjusted |
| Eubacteriales sp. (HG3A.0635) | DBP | 0.044 | 0.035 | 0.322 | 2334 | OSA and BMI adjusted |
| Eubacteriales sp. (HG3A.0635) | HbA1c | -1.52E-02 | 0.429 | 0.972 | 2785 | OSA and BMI adjusted |
| Eubacteriales sp. (HG3A.0691) | SBP | 0.012 | 0.579 | 0.778 | 2334 | OSA adjusted |
| Eubacteriales sp. (HG3A.0691) | DBP | -3.19E-03 | 0.88 | 0.956 | 2334 | OSA adjusted |
| Eubacteriales sp. (HG3A.0691) | HbA1c | -1.14E-02 | 0.554 | 0.882 | 2785 | OSA adjusted |
| Eubacteriales sp. (HG3A.0691) | SBP | 0.02 | 0.343 | 0.758 | 2334 | OSA and BMI adjusted |
| Eubacteriales sp. (HG3A.0691) | DBP | 0.005 | 0.809 | 0.989 | 2334 | OSA and BMI adjusted |
| Eubacteriales sp. (HG3A.0691) | HbA1c | -7.20E-03 | 0.708 | 0.972 | 2785 | OSA and BMI adjusted |
| Eubacteriales sp. (HG3A.0703) | SBP | -2.13E-03 | 0.919 | 0.954 | 2334 | OSA adjusted |

| Eubacteriales sp. (HG3A.0703) | DBP | 3.66E-04 | 0.986 | 0.993 | 2334 | OSA adjusted |
| --- | --- | --- | --- | --- | --- | --- |
| Eubacteriales sp. (HG3A.0703) | HbA1c | 0.006 | 0.747 | 0.888 | 2785 | OSA adjusted |
| Eubacteriales sp. (HG3A.0703) | SBP | 0.004 | 0.862 | 0.922 | 2334 | OSA and BMI adjusted |
| Eubacteriales sp. (HG3A.0703) | DBP | 0.007 | 0.753 | 0.989 | 2334 | OSA and BMI adjusted |
| Eubacteriales sp. (HG3A.0703) | HbA1c | 0.011 | 0.583 | 0.972 | 2785 | OSA and BMI adjusted |
| Eubacteriales sp. (HG3A.0718) | SBP | -3.26E-03 | 0.877 | 0.939 | 2334 | OSA adjusted |
| Eubacteriales sp. (HG3A.0718) | DBP | -1.88E-03 | 0.929 | 0.957 | 2334 | OSA adjusted |
| Eubacteriales sp. (HG3A.0718) | HbA1c | 0.018 | 0.354 | 0.875 | 2785 | OSA adjusted |
| Eubacteriales sp. (HG3A.0718) | SBP | 0.005 | 0.81 | 0.922 | 2334 | OSA and BMI adjusted |
| Eubacteriales sp. (HG3A.0718) | DBP | 0.007 | 0.738 | 0.989 | 2334 | OSA and BMI adjusted |
| Eubacteriales sp. (HG3A.0718) | HbA1c | 0.022 | 0.255 | 0.96 | 2785 | OSA and BMI adjusted |
| Eubacteriales sp. (HG3A.0786) | SBP | -5.49E-03 | 0.794 | 0.904 | 2334 | OSA adjusted |
| Eubacteriales sp. (HG3A.0786) | DBP | -6.53E-03 | 0.756 | 0.917 | 2334 | OSA adjusted |
| Eubacteriales sp. (HG3A.0786) | HbA1c | 0.035 | 0.071 | 0.647 | 2785 | OSA adjusted |
| Eubacteriales sp. (HG3A.0786) | SBP | -1.68E-02 | 0.425 | 0.848 | 2334 | OSA and BMI adjusted |
| Eubacteriales sp. (HG3A.0786) | DBP | -1.86E-02 | 0.376 | 0.872 | 2334 | OSA and BMI adjusted |
| Eubacteriales sp. (HG3A.0786) | HbA1c | 0.029 | 0.137 | 0.94 | 2785 | OSA and BMI adjusted |
| Eubacteriales sp. (HG3A.0829) | SBP | -2.71E-02 | 0.197 | 0.442 | 2334 | OSA adjusted |
| Eubacteriales sp. (HG3A.0829) | DBP | -4.14E-02 | 0.049 | 0.182 | 2334 | OSA adjusted |
| Eubacteriales sp. (HG3A.0829) | HbA1c | -1.28E-03 | 0.947 | 0.961 | 2785 | OSA adjusted |
| Eubacteriales sp. (HG3A.0829) | SBP | -2.32E-02 | 0.269 | 0.659 | 2334 | OSA and BMI adjusted |
| Eubacteriales sp. (HG3A.0829) | DBP | -3.78E-02 | 0.072 | 0.422 | 2334 | OSA and BMI adjusted |
| Eubacteriales sp. (HG3A.0829) | HbA1c | 6.60E-04 | 0.973 | 0.973 | 2785 | OSA and BMI adjusted |
| Eubacteriales sp. (HG3A.0956) | SBP | 0.014 | 0.503 | 0.717 | 2334 | OSA adjusted |
| Eubacteriales sp. (HG3A.0956) | DBP | 0.018 | 0.395 | 0.703 | 2334 | OSA adjusted |
| Eubacteriales sp. (HG3A.0956) | HbA1c | -1.06E-02 | 0.582 | 0.882 | 2785 | OSA adjusted |
| Eubacteriales sp. (HG3A.0956) | SBP | 0.015 | 0.475 | 0.88 | 2334 | OSA and BMI adjusted |
| Eubacteriales sp. (HG3A.0956) | DBP | 0.019 | 0.365 | 0.872 | 2334 | OSA and BMI adjusted |
| Eubacteriales sp. (HG3A.0956) | HbA1c | -9.28E-03 | 0.629 | 0.972 | 2785 | OSA and BMI adjusted |
| Eubacteriales sp. (HG3A.0978) | SBP | 0.012 | 0.573 | 0.778 | 2334 | OSA adjusted |
| Eubacteriales sp. (HG3A.0978) | DBP | -1.29E-02 | 0.541 | 0.753 | 2334 | OSA adjusted |
| Eubacteriales sp. (HG3A.0978) | HbA1c | -1.56E-03 | 0.935 | 0.956 | 2785 | OSA adjusted |

| Eubacteriales sp. (HG3A.0978) | SBP | 0.024 | 0.252 | 0.65 | 2334 | OSA and BMI adjusted |
| --- | --- | --- | --- | --- | --- | --- |
| Eubacteriales sp. (HG3A.0978) | DBP | -7.95E-04 | 0.97 | 0.989 | 2334 | OSA and BMI adjusted |
| Eubacteriales sp. (HG3A.0978) | HbA1c | 0.007 | 0.712 | 0.972 | 2785 | OSA and BMI adjusted |
| Eubacteriales sp. (HG3A.1019) | SBP | -1.61E-03 | 0.939 | 0.954 | 2334 | OSA adjusted |
| Eubacteriales sp. (HG3A.1019) | DBP | -3.71E-03 | 0.86 | 0.956 | 2334 | OSA adjusted |
| Eubacteriales sp. (HG3A.1019) | HbA1c | 0.009 | 0.649 | 0.882 | 2785 | OSA adjusted |
| Eubacteriales sp. (HG3A.1019) | SBP | -9.43E-03 | 0.654 | 0.922 | 2334 | OSA and BMI adjusted |
| Eubacteriales sp. (HG3A.1019) | DBP | -1.21E-02 | 0.564 | 0.989 | 2334 | OSA and BMI adjusted |
| Eubacteriales sp. (HG3A.1019) | HbA1c | 0.007 | 0.716 | 0.972 | 2785 | OSA and BMI adjusted |
| Eubacteriales sp. (HG3A.1026) | SBP | 0.023 | 0.274 | 0.514 | 2334 | OSA adjusted |
| Eubacteriales sp. (HG3A.1026) | DBP | 0.021 | 0.322 | 0.648 | 2334 | OSA adjusted |
| Eubacteriales sp. (HG3A.1026) | HbA1c | 0.035 | 0.068 | 0.647 | 2785 | OSA adjusted |
| Eubacteriales sp. (HG3A.1026) | SBP | 0.021 | 0.314 | 0.717 | 2334 | OSA and BMI adjusted |
| Eubacteriales sp. (HG3A.1026) | DBP | 0.019 | 0.37 | 0.872 | 2334 | OSA and BMI adjusted |
| Eubacteriales sp. (HG3A.1026) | HbA1c | 0.032 | 0.095 | 0.94 | 2785 | OSA and BMI adjusted |
| Eubacteriales sp. (HG3A.1294) | SBP | -3.51E-03 | 0.868 | 0.936 | 2334 | OSA adjusted |
| Eubacteriales sp. (HG3A.1294) | DBP | 0.018 | 0.386 | 0.702 | 2334 | OSA adjusted |
| Eubacteriales sp. (HG3A.1294) | HbA1c | -9.79E-03 | 0.61 | 0.882 | 2785 | OSA adjusted |
| Eubacteriales sp. (HG3A.1294) | SBP | -3.31E-03 | 0.875 | 0.922 | 2334 | OSA and BMI adjusted |
| Eubacteriales sp. (HG3A.1294) | DBP | 0.019 | 0.36 | 0.872 | 2334 | OSA and BMI adjusted |
| Eubacteriales sp. (HG3A.1294) | HbA1c | -7.38E-03 | 0.701 | 0.972 | 2785 | OSA and BMI adjusted |
| Eubacteriales sp. (HG3A.1379) | SBP | -6.56E-02 | 0.002 | 0.02 | 2334 | OSA adjusted |
| Eubacteriales sp. (HG3A.1379) | DBP | -7.48E-02 | 3.71E-04 | 0.008 | 2334 | OSA adjusted |
| Eubacteriales sp. (HG3A.1379) | HbA1c | -2.28E-02 | 0.235 | 0.832 | 2785 | OSA adjusted |
| Eubacteriales sp. (HG3A.1379) | SBP | -5.54E-02 | 0.008 | 0.164 | 2334 | OSA and BMI adjusted |
| Eubacteriales sp. (HG3A.1379) | DBP | -6.44E-02 | 0.002 | 0.1 | 2334 | OSA and BMI adjusted |
| Eubacteriales sp. (HG3A.1379) | HbA1c | -1.46E-02 | 0.447 | 0.972 | 2785 | OSA and BMI adjusted |
| Eubacterium sp. (HG3A.0214) | SBP | -9.78E-03 | 0.642 | 0.814 | 2334 | OSA adjusted |
| Eubacterium sp. (HG3A.0214) | DBP | -1.16E-02 | 0.582 | 0.767 | 2334 | OSA adjusted |
| Eubacterium sp. (HG3A.0214) | HbA1c | -5.59E-03 | 0.771 | 0.888 | 2785 | OSA adjusted |
| Eubacterium sp. (HG3A.0214) | SBP | -9.61E-03 | 0.648 | 0.922 | 2334 | OSA and BMI adjusted |
| Eubacterium sp. (HG3A.0214) | DBP | -1.15E-02 | 0.585 | 0.989 | 2334 | OSA and BMI adjusted |

| Eubacterium sp. (HG3A.0214) | HbA1c | -5.97E-03 | 0.756 | 0.972 | 2785 | OSA and BMI adjusted |
| --- | --- | --- | --- | --- | --- | --- |
| Firmicutes sp. (HG3A.0301) | SBP | -6.66E-02 | 0.002 | 0.02 | 2334 | OSA adjusted |
| Firmicutes sp. (HG3A.0301) | DBP | -7.34E-02 | 4.70E-04 | 0.009 | 2334 | OSA adjusted |
| Firmicutes sp. (HG3A.0301) | HbA1c | -2.24E-02 | 0.244 | 0.832 | 2785 | OSA adjusted |
| Firmicutes sp. (HG3A.0301) | SBP | -5.10E-02 | 0.015 | 0.168 | 2334 | OSA and BMI adjusted |
| Firmicutes sp. (HG3A.0301) | DBP | -5.73E-02 | 0.006 | 0.147 | 2334 | OSA and BMI adjusted |
| Firmicutes sp. (HG3A.0301) | HbA1c | -1.31E-02 | 0.495 | 0.972 | 2785 | OSA and BMI adjusted |
| Firmicutes sp. (HG3A.0341) | SBP | -2.50E-03 | 0.905 | 0.954 | 2334 | OSA adjusted |
| Firmicutes sp. (HG3A.0341) | DBP | -4.64E-03 | 0.825 | 0.932 | 2334 | OSA adjusted |
| Firmicutes sp. (HG3A.0341) | HbA1c | -3.69E-03 | 0.848 | 0.922 | 2785 | OSA adjusted |
| Firmicutes sp. (HG3A.0341) | SBP | 0.012 | 0.577 | 0.922 | 2334 | OSA and BMI adjusted |
| Firmicutes sp. (HG3A.0341) | DBP | 0.01 | 0.619 | 0.989 | 2334 | OSA and BMI adjusted |
| Firmicutes sp. (HG3A.0341) | HbA1c | 0.005 | 0.779 | 0.972 | 2785 | OSA and BMI adjusted |
| Firmicutes sp. (HG3A.0397) | SBP | -1.17E-02 | 0.577 | 0.778 | 2334 | OSA adjusted |
| Firmicutes sp. (HG3A.0397) | DBP | -1.22E-02 | 0.56 | 0.753 | 2334 | OSA adjusted |
| Firmicutes sp. (HG3A.0397) | HbA1c | -6.36E-03 | 0.741 | 0.888 | 2785 | OSA adjusted |
| Firmicutes sp. (HG3A.0397) | SBP | 0.004 | 0.837 | 0.922 | 2334 | OSA and BMI adjusted |
| Firmicutes sp. (HG3A.0397) | DBP | 0.005 | 0.818 | 0.989 | 2334 | OSA and BMI adjusted |
| Firmicutes sp. (HG3A.0397) | HbA1c | 0.005 | 0.794 | 0.972 | 2785 | OSA and BMI adjusted |
| Firmicutes sp. (HG3A.0398) | SBP | -2.05E-02 | 0.329 | 0.579 | 2334 | OSA adjusted |
| Firmicutes sp. (HG3A.0398) | DBP | -1.25E-02 | 0.553 | 0.753 | 2334 | OSA adjusted |
| Firmicutes sp. (HG3A.0398) | HbA1c | -4.78E-03 | 0.803 | 0.902 | 2785 | OSA adjusted |
| Firmicutes sp. (HG3A.0398) | SBP | -4.33E-03 | 0.837 | 0.922 | 2334 | OSA and BMI adjusted |
| Firmicutes sp. (HG3A.0398) | DBP | 0.005 | 0.81 | 0.989 | 2334 | OSA and BMI adjusted |
| Firmicutes sp. (HG3A.0398) | HbA1c | 0.003 | 0.871 | 0.972 | 2785 | OSA and BMI adjusted |
| Firmicutes sp. (HG3A.1085) | SBP | 0.009 | 0.654 | 0.823 | 2334 | OSA adjusted |
| Firmicutes sp. (HG3A.1085) | DBP | 0.027 | 0.199 | 0.463 | 2334 | OSA adjusted |
| Firmicutes sp. (HG3A.1085) | HbA1c | 0.01 | 0.62 | 0.882 | 2785 | OSA adjusted |
| Firmicutes sp. (HG3A.1085) | SBP | 0.01 | 0.62 | 0.922 | 2334 | OSA and BMI adjusted |
| Firmicutes sp. (HG3A.1085) | DBP | 0.029 | 0.172 | 0.602 | 2334 | OSA and BMI adjusted |
| Firmicutes sp. (HG3A.1085) | HbA1c | 0.012 | 0.527 | 0.972 | 2785 | OSA and BMI adjusted |
| Flavonifractor plautii (HG3A.0079) | SBP | 0.023 | 0.273 | 0.514 | 2334 | OSA adjusted |

| Flavonifractor plautii (HG3A.0079) | DBP | 0.015 | 0.463 | 0.751 | 2334 | OSA adjusted |
| --- | --- | --- | --- | --- | --- | --- |
| Flavonifractor plautii (HG3A.0079) | HbA1c | -2.40E-02 | 0.212 | 0.828 | 2785 | OSA adjusted |
| Flavonifractor plautii (HG3A.0079) | SBP | 0.016 | 0.439 | 0.848 | 2334 | OSA and BMI adjusted |
| Flavonifractor plautii (HG3A.0079) | DBP | 0.008 | 0.707 | 0.989 | 2334 | OSA and BMI adjusted |
| Flavonifractor plautii (HG3A.0079) | HbA1c | -3.08E-02 | 0.109 | 0.94 | 2785 | OSA and BMI adjusted |
| Fusicatenibacter saccharivorans  (HG3A.0004) | SBP | 0.026 | 0.215 | 0.461 | 2334 | OSA adjusted |
| Fusicatenibacter saccharivorans (HG3A.0004) | DBP | 0.017 | 0.415 | 0.709 | 2334 | OSA adjusted |
| Fusicatenibacter saccharivorans  (HG3A.0004) | HbA1c | 0.013 | 0.507 | 0.875 | 2785 | OSA adjusted |
| Fusicatenibacter saccharivorans (HG3A.0004) | SBP | 0.009 | 0.654 | 0.922 | 2334 | OSA and BMI adjusted |
| Fusicatenibacter saccharivorans  (HG3A.0004) | DBP | -9.07E-04 | 0.966 | 0.989 | 2334 | OSA and BMI adjusted |
| Fusicatenibacter saccharivorans (HG3A.0004) | HbA1c | -7.74E-04 | 0.968 | 0.973 | 2785 | OSA and BMI adjusted |
| Intestinibacillus sp. Marseille-P4005  (HG3A.0168) | SBP | 0.052 | 0.013 | 0.072 | 2334 | OSA adjusted |
| Intestinibacillus sp. Marseille-P4005 (HG3A.0168) | DBP | 0.04 | 0.057 | 0.195 | 2334 | OSA adjusted |
| Intestinibacillus sp. Marseille-P4005  (HG3A.0168) | HbA1c | -8.96E-03 | 0.641 | 0.882 | 2785 | OSA adjusted |
| Intestinibacillus sp. Marseille-P4005 (HG3A.0168) | SBP | 0.047 | 0.024 | 0.221 | 2334 | OSA and BMI adjusted |
| Intestinibacillus sp. Marseille-P4005  (HG3A.0168) | DBP | 0.035 | 0.1 | 0.462 | 2334 | OSA and BMI adjusted |
| Intestinibacillus sp. Marseille-P4005 (HG3A.0168) | HbA1c | -1.39E-02 | 0.468 | 0.972 | 2785 | OSA and BMI adjusted |
| Intestinimonas massiliensis (HG3A.0198) | SBP | -6.62E-02 | 0.002 | 0.02 | 2334 | OSA adjusted |
| Intestinimonas massiliensis (HG3A.0198) | DBP | -5.60E-02 | 0.008 | 0.062 | 2334 | OSA adjusted |
| Intestinimonas massiliensis (HG3A.0198) | HbA1c | -4.26E-02 | 0.027 | 0.647 | 2785 | OSA adjusted |
| Intestinimonas massiliensis (HG3A.0198) | SBP | -3.20E-02 | 0.128 | 0.497 | 2334 | OSA and BMI adjusted |

| Intestinimonas massiliensis (HG3A.0198) | DBP | -1.92E-02 | 0.362 | 0.872 | 2334 | OSA and BMI adjusted |
| --- | --- | --- | --- | --- | --- | --- |
| Intestinimonas massiliensis (HG3A.0198) | HbA1c | -2.04E-02 | 0.288 | 0.96 | 2785 | OSA and BMI adjusted |
| Lachnospiraceae sp. (HG3A.0018) | SBP | 0.038 | 0.071 | 0.217 | 2334 | OSA adjusted |
| Lachnospiraceae sp. (HG3A.0018) | DBP | 0.055 | 0.009 | 0.062 | 2334 | OSA adjusted |
| Lachnospiraceae sp. (HG3A.0018) | HbA1c | 0.025 | 0.197 | 0.828 | 2785 | OSA adjusted |
| Lachnospiraceae sp. (HG3A.0018) | SBP | 0.028 | 0.187 | 0.581 | 2334 | OSA and BMI adjusted |
| Lachnospiraceae sp. (HG3A.0018) | DBP | 0.045 | 0.032 | 0.322 | 2334 | OSA and BMI adjusted |
| Lachnospiraceae sp. (HG3A.0018) | HbA1c | 0.017 | 0.388 | 0.972 | 2785 | OSA and BMI adjusted |
| Lachnospiraceae sp. (HG3A.0855) | SBP | 0.006 | 0.761 | 0.904 | 2334 | OSA adjusted |
| Lachnospiraceae sp. (HG3A.0855) | DBP | 0.014 | 0.492 | 0.753 | 2334 | OSA adjusted |
| Lachnospiraceae sp. (HG3A.0855) | HbA1c | 0.026 | 0.176 | 0.822 | 2785 | OSA adjusted |
| Lachnospiraceae sp. (HG3A.0855) | SBP | 0.003 | 0.872 | 0.922 | 2334 | OSA and BMI adjusted |
| Lachnospiraceae sp. (HG3A.0855) | DBP | 0.012 | 0.582 | 0.989 | 2334 | OSA and BMI adjusted |
| Lachnospiraceae sp. (HG3A.0855) | HbA1c | 0.026 | 0.184 | 0.94 | 2785 | OSA and BMI adjusted |
| Mediterraneibacter glycyrrhizinilyticus (HG3A.0314) | SBP | 0.042 | 0.046 | 0.175 | 2334 | OSA adjusted |
| Mediterraneibacter glycyrrhizinilyticus (HG3A.0314) | DBP | 0.033 | 0.117 | 0.324 | 2334 | OSA adjusted |
| Mediterraneibacter glycyrrhizinilyticus  (HG3A.0314) | HbA1c | -6.04E-03 | 0.753 | 0.888 | 2785 | OSA adjusted |
| Mediterraneibacter glycyrrhizinilyticus (HG3A.0314) | SBP | 0.03 | 0.155 | 0.55 | 2334 | OSA and BMI adjusted |
| Mediterraneibacter glycyrrhizinilyticus  (HG3A.0314) | DBP | 0.02 | 0.343 | 0.872 | 2334 | OSA and BMI adjusted |
| Mediterraneibacter glycyrrhizinilyticus (HG3A.0314) | HbA1c | -1.36E-02 | 0.479 | 0.972 | 2785 | OSA and BMI adjusted |
| Oscillibacter sp. (HG3A.0734) | SBP | -6.36E-02 | 0.002 | 0.024 | 2334 | OSA adjusted |
| Oscillibacter sp. (HG3A.0734) | DBP | -6.25E-02 | 0.003 | 0.031 | 2334 | OSA adjusted |
| Oscillibacter sp. (HG3A.0734) | HbA1c | -8.94E-03 | 0.641 | 0.882 | 2785 | OSA adjusted |
| Oscillibacter sp. (HG3A.0734) | SBP | -6.76E-02 | 0.001 | 0.059 | 2334 | OSA and BMI adjusted |
| Oscillibacter sp. (HG3A.0734) | DBP | -6.70E-02 | 0.001 | 0.099 | 2334 | OSA and BMI adjusted |
| Oscillibacter sp. (HG3A.0734) | HbA1c | -1.01E-02 | 0.599 | 0.972 | 2785 | OSA and BMI adjusted |

| Oscillospiraceae sp. (HG3A.0072) | SBP | -6.29E-02 | 0.003 | 0.025 | 2334 | OSA adjusted |
| --- | --- | --- | --- | --- | --- | --- |
| Oscillospiraceae sp. (HG3A.0072) | DBP | -5.46E-02 | 0.009 | 0.064 | 2334 | OSA adjusted |
| Oscillospiraceae sp. (HG3A.0072) | HbA1c | -2.28E-02 | 0.235 | 0.832 | 2785 | OSA adjusted |
| Oscillospiraceae sp. (HG3A.0072) | SBP | -4.13E-02 | 0.049 | 0.328 | 2334 | OSA and BMI adjusted |
| Oscillospiraceae sp. (HG3A.0072) | DBP | -3.14E-02 | 0.135 | 0.51 | 2334 | OSA and BMI adjusted |
| Oscillospiraceae sp. (HG3A.0072) | HbA1c | -7.05E-03 | 0.714 | 0.972 | 2785 | OSA and BMI adjusted |
| Oscillospiraceae sp. (HG3A.0207) | SBP | -6.25E-02 | 0.003 | 0.025 | 2334 | OSA adjusted |
| Oscillospiraceae sp. (HG3A.0207) | DBP | -4.99E-02 | 0.018 | 0.093 | 2334 | OSA adjusted |
| Oscillospiraceae sp. (HG3A.0207) | HbA1c | 3.88E-04 | 0.984 | 0.984 | 2785 | OSA adjusted |
| Oscillospiraceae sp. (HG3A.0207) | SBP | -5.20E-02 | 0.013 | 0.168 | 2334 | OSA and BMI adjusted |
| Oscillospiraceae sp. (HG3A.0207) | DBP | -3.83E-02 | 0.069 | 0.422 | 2334 | OSA and BMI adjusted |
| Oscillospiraceae sp. (HG3A.0207) | HbA1c | 0.008 | 0.666 | 0.972 | 2785 | OSA and BMI adjusted |
| Oscillospiraceae sp. (HG3A.0223) | SBP | -8.52E-02 | 4.90E-05 | 0.002 | 2334 | OSA adjusted |
| Oscillospiraceae sp. (HG3A.0223) | DBP | -7.86E-02 | 1.83E-04 | 0.006 | 2334 | OSA adjusted |
| Oscillospiraceae sp. (HG3A.0223) | HbA1c | -5.87E-02 | 0.002 | 0.303 | 2785 | OSA adjusted |
| Oscillospiraceae sp. (HG3A.0223) | SBP | -4.94E-02 | 0.019 | 0.183 | 2334 | OSA and BMI adjusted |
| Oscillospiraceae sp. (HG3A.0223) | DBP | -4.02E-02 | 0.056 | 0.381 | 2334 | OSA and BMI adjusted |
| Oscillospiraceae sp. (HG3A.0223) | HbA1c | -3.74E-02 | 0.052 | 0.94 | 2785 | OSA and BMI adjusted |
| Oscillospiraceae sp. (HG3A.0437) | SBP | -1.02E-02 | 0.627 | 0.811 | 2334 | OSA adjusted |
| Oscillospiraceae sp. (HG3A.0437) | DBP | -1.61E-02 | 0.443 | 0.74 | 2334 | OSA adjusted |
| Oscillospiraceae sp. (HG3A.0437) | HbA1c | 0.004 | 0.844 | 0.922 | 2785 | OSA adjusted |
| Oscillospiraceae sp. (HG3A.0437) | SBP | 0.009 | 0.678 | 0.922 | 2334 | OSA and BMI adjusted |
| Oscillospiraceae sp. (HG3A.0437) | DBP | 0.004 | 0.856 | 0.989 | 2334 | OSA and BMI adjusted |
| Oscillospiraceae sp. (HG3A.0437) | HbA1c | 0.016 | 0.402 | 0.972 | 2785 | OSA and BMI adjusted |
| Oscillospiraceae sp. (HG3A.0445) | SBP | -5.68E-02 | 0.007 | 0.047 | 2334 | OSA adjusted |
| Oscillospiraceae sp. (HG3A.0445) | DBP | -5.54E-02 | 0.008 | 0.062 | 2334 | OSA adjusted |
| Oscillospiraceae sp. (HG3A.0445) | HbA1c | 0.006 | 0.765 | 0.888 | 2785 | OSA adjusted |
| Oscillospiraceae sp. (HG3A.0445) | SBP | -5.87E-02 | 0.005 | 0.143 | 2334 | OSA and BMI adjusted |
| Oscillospiraceae sp. (HG3A.0445) | DBP | -5.75E-02 | 0.006 | 0.147 | 2334 | OSA and BMI adjusted |
| Oscillospiraceae sp. (HG3A.0445) | HbA1c | 0.004 | 0.816 | 0.972 | 2785 | OSA and BMI adjusted |
| Oscillospiraceae sp. (HG3A.1270) | SBP | -5.20E-03 | 0.805 | 0.904 | 2334 | OSA adjusted |
| Oscillospiraceae sp. (HG3A.1270) | DBP | 0.002 | 0.936 | 0.957 | 2334 | OSA adjusted |

| Oscillospiraceae sp. (HG3A.1270) | HbA1c | -3.21E-02 | 0.095 | 0.73 | 2785 | OSA adjusted |
| --- | --- | --- | --- | --- | --- | --- |
| Oscillospiraceae sp. (HG3A.1270) | SBP | -1.50E-03 | 0.943 | 0.957 | 2334 | OSA and BMI adjusted |
| Oscillospiraceae sp. (HG3A.1270) | DBP | 0.006 | 0.78 | 0.989 | 2334 | OSA and BMI adjusted |
| Oscillospiraceae sp. (HG3A.1270) | HbA1c | -2.75E-02 | 0.152 | 0.94 | 2785 | OSA and BMI adjusted |
| Pediococcus acidilactici (HG3A.1468) | SBP | 0.034 | 0.106 | 0.291 | 2334 | OSA adjusted |
| Pediococcus acidilactici (HG3A.1468) | DBP | 0.014 | 0.513 | 0.753 | 2334 | OSA adjusted |
| Pediococcus acidilactici (HG3A.1468) | HbA1c | 0.021 | 0.273 | 0.86 | 2785 | OSA adjusted |
| Pediococcus acidilactici (HG3A.1468) | SBP | 0.033 | 0.113 | 0.497 | 2334 | OSA and BMI adjusted |
| Pediococcus acidilactici (HG3A.1468) | DBP | 0.012 | 0.556 | 0.989 | 2334 | OSA and BMI adjusted |
| Pediococcus acidilactici (HG3A.1468) | HbA1c | 0.02 | 0.303 | 0.96 | 2785 | OSA and BMI adjusted |
| Roseburia inulinivorans (HG3A.0036) | SBP | 0.037 | 0.077 | 0.231 | 2334 | OSA adjusted |
| Roseburia inulinivorans (HG3A.0036) | DBP | 0.034 | 0.107 | 0.311 | 2334 | OSA adjusted |
| Roseburia inulinivorans (HG3A.0036) | HbA1c | 0.007 | 0.717 | 0.888 | 2785 | OSA adjusted |
| Roseburia inulinivorans (HG3A.0036) | SBP | 0.017 | 0.432 | 0.848 | 2334 | OSA and BMI adjusted |
| Roseburia inulinivorans (HG3A.0036) | DBP | 0.012 | 0.572 | 0.989 | 2334 | OSA and BMI adjusted |
| Roseburia inulinivorans (HG3A.0036) | HbA1c | -7.55E-03 | 0.694 | 0.972 | 2785 | OSA and BMI adjusted |
| Roseburia sp. AM59-24XD (HG3A.0391) | SBP | 0.01 | 0.624 | 0.811 | 2334 | OSA adjusted |
| Roseburia sp. AM59-24XD (HG3A.0391) | DBP | 0.006 | 0.765 | 0.919 | 2334 | OSA adjusted |
| Roseburia sp. AM59-24XD (HG3A.0391) | HbA1c | 0.008 | 0.671 | 0.888 | 2785 | OSA adjusted |
| Roseburia sp. AM59-24XD (HG3A.0391) | SBP | 0.01 | 0.626 | 0.922 | 2334 | OSA and BMI adjusted |
| Roseburia sp. AM59-24XD (HG3A.0391) | DBP | 0.006 | 0.772 | 0.989 | 2334 | OSA and BMI adjusted |
| Roseburia sp. AM59-24XD (HG3A.0391) | HbA1c | 0.009 | 0.651 | 0.972 | 2785 | OSA and BMI adjusted |
| [Ruminococcus] gnavus (HG3A.0239) | SBP | 0.045 | 0.031 | 0.124 | 2334 | OSA adjusted |
| [Ruminococcus] gnavus (HG3A.0239) | DBP | 0.035 | 0.1 | 0.297 | 2334 | OSA adjusted |
| [Ruminococcus] gnavus (HG3A.0239) | HbA1c | 0.003 | 0.888 | 0.928 | 2785 | OSA adjusted |
| [Ruminococcus] gnavus (HG3A.0239) | SBP | 0.036 | 0.09 | 0.455 | 2334 | OSA and BMI adjusted |
| [Ruminococcus] gnavus (HG3A.0239) | DBP | 0.024 | 0.255 | 0.765 | 2334 | OSA and BMI adjusted |
| [Ruminococcus] gnavus (HG3A.0239) | HbA1c | -4.98E-03 | 0.795 | 0.972 | 2785 | OSA and BMI adjusted |
| Ruminococcus sp. AM42-11 (HG3A.0002) | SBP | 0.031 | 0.142 | 0.359 | 2334 | OSA adjusted |
| Ruminococcus sp. AM42-11 (HG3A.0002) | DBP | 0.026 | 0.21 | 0.471 | 2334 | OSA adjusted |
| Ruminococcus sp. AM42-11 (HG3A.0002) | HbA1c | 0.01 | 0.62 | 0.882 | 2785 | OSA adjusted |
| Ruminococcus sp. AM42-11 (HG3A.0002) | SBP | 0.026 | 0.225 | 0.618 | 2334 | OSA and BMI adjusted |

| Ruminococcus sp. AM42-11 (HG3A.0002) | DBP | 0.021 | 0.328 | 0.872 | 2334 | OSA and BMI adjusted |
| --- | --- | --- | --- | --- | --- | --- |
| Ruminococcus sp. AM42-11 (HG3A.0002) | HbA1c | 0.006 | 0.76 | 0.972 | 2785 | OSA and BMI adjusted |
| [Ruminococcus] torques (HG3A.0034) | SBP | 0.017 | 0.419 | 0.653 | 2334 | OSA adjusted |
| [Ruminococcus] torques (HG3A.0034) | DBP | 0.005 | 0.8 | 0.932 | 2334 | OSA adjusted |
| [Ruminococcus] torques (HG3A.0034) | HbA1c | -1.37E-02 | 0.474 | 0.875 | 2785 | OSA adjusted |
| [Ruminococcus] torques (HG3A.0034) | SBP | 0.006 | 0.781 | 0.922 | 2334 | OSA and BMI adjusted |
| [Ruminococcus] torques (HG3A.0034) | DBP | -6.99E-03 | 0.74 | 0.989 | 2334 | OSA and BMI adjusted |
| [Ruminococcus] torques (HG3A.0034) | HbA1c | -2.32E-02 | 0.227 | 0.94 | 2785 | OSA and BMI adjusted |
| Staphylococcus aureus (HG3A.1538) | SBP | 0.035 | 0.096 | 0.268 | 2334 | OSA adjusted |
| Staphylococcus aureus (HG3A.1538) | DBP | 0.013 | 0.521 | 0.753 | 2334 | OSA adjusted |
| Staphylococcus aureus (HG3A.1538) | HbA1c | -9.83E-03 | 0.609 | 0.882 | 2785 | OSA adjusted |
| Staphylococcus aureus (HG3A.1538) | SBP | 0.039 | 0.063 | 0.37 | 2334 | OSA and BMI adjusted |
| Staphylococcus aureus (HG3A.1538) | DBP | 0.017 | 0.419 | 0.905 | 2334 | OSA and BMI adjusted |
| Staphylococcus aureus (HG3A.1538) | HbA1c | -8.75E-03 | 0.649 | 0.972 | 2785 | OSA and BMI adjusted |
| Traorella massiliensis (HG3A.0669) | SBP | -6.96E-03 | 0.741 | 0.904 | 2334 | OSA adjusted |
| Traorella massiliensis (HG3A.0669) | DBP | -1.71E-02 | 0.416 | 0.709 | 2334 | OSA adjusted |
| Traorella massiliensis (HG3A.0669) | HbA1c | -1.29E-02 | 0.503 | 0.875 | 2785 | OSA adjusted |
| Traorella massiliensis (HG3A.0669) | SBP | 0.007 | 0.744 | 0.922 | 2334 | OSA and BMI adjusted |
| Traorella massiliensis (HG3A.0669) | DBP | -2.78E-03 | 0.895 | 0.989 | 2334 | OSA and BMI adjusted |
| Traorella massiliensis (HG3A.0669) | HbA1c | -4.95E-03 | 0.796 | 0.972 | 2785 | OSA and BMI adjusted |
| Victivallis vadensis (HG3A.0689) | SBP | -7.20E-03 | 0.732 | 0.903 | 2334 | OSA adjusted |
| Victivallis vadensis (HG3A.0689) | DBP | -1.76E-02 | 0.402 | 0.707 | 2334 | OSA adjusted |
| Victivallis vadensis (HG3A.0689) | HbA1c | -1.90E-02 | 0.322 | 0.866 | 2785 | OSA adjusted |
| Victivallis vadensis (HG3A.0689) | SBP | 0.007 | 0.756 | 0.922 | 2334 | OSA and BMI adjusted |
| Victivallis vadensis (HG3A.0689) | DBP | -3.37E-03 | 0.873 | 0.989 | 2334 | OSA and BMI adjusted |
| Victivallis vadensis (HG3A.0689) | HbA1c | -1.23E-02 | 0.523 | 0.972 | 2785 | OSA and BMI adjusted |
