# Supplementary material for: OSA Is Associated With the Human Gut Microbiota Composition and Functional Potential in the Population-Based Swedish CardioPulmonary bioImage Study
Source: Chest. 2023 Mar 15;164(2):503–16. doi: 10.1016/j.chest.2023.03.010 (PMC10410248; doi:10.1016/j.chest.2023.03.010)
Supplement: e-Online Data [file mmc11.docx]

**Obstructive sleep apnea was associated with the human gut microbiota composition and functional potential in the population-based Swedish CardioPulmonary bioImage Study (SCAPIS)**

# e-Figures legends

**e-Figure 1.** Study flowchart for the 4,839 SCAPIS-Uppsala participants with fecal metagenomics data.

**e-Figure 2.** Directed acyclic graph depicting the causal assumptions on the effect of obstructive sleep apnea (OSA) on the gut microbiota composition. A directed edge (or “arrow”) from one node to another represents a direct effect between these two nodes. Green line: causal path; pink line: biasing path; pink nodes: ancestor of exposure and outcome; and blue nodes: ancestor of outcome.

**e-Figure 3.** Directed acyclic graph depicting the causal assumptions on the effect of gut microbiota on blood pressure and glycemia. A directed edge (or “arrow”) from one node to another represents a direct effect between these two nodes. Green line: causal path; pink line: biasing path; pink nodes: ancestor of exposure and outcome; and blue nodes: ancestor of outcome.

**e-Figure 4.** Co-occurrence network for the 117 species associated with T90 and/or ODI in the extended model (adjustment for age, sex, smoking, alcohol intake, BMI, fiber intake, total energy intake, physical activity, education, birth country, season, and DNA extraction plate) that had a prevalence <95%. Co-occurrence was determined using a probabilistic model described at Griffith, D. M. et al.^1^ **A.** Species are labeled by the direction of the association with T90 and/or ODI. **B.** Species are labeled by their prevalence.

**e-Figure 5.** Box plot of the combined relative abundance of the 28 species in positive association and the 100 species in negative association with T90 and/or oxygen desaturation index (ODI) in the extended model. T90: percentage of time with oxygen saturation < 90%.

# e-Tables legends

**e-Table 1.** Descriptive characteristics by groups of percentage of time with saturation below 90% (T90).

Legend: Participants with T90 data were divided into one group with T90 = 0, and the remaining divided into three groups of similar size (t1, t2, and t3) in order of ascending T90 values.

Continuous variables presented as median [interquartile range] and categorical variables presented as absolute numbers (%). AHI: apnea-hypopnea index; BMI: Body mass index; DBP: diastolic blood pressure; HbA1c: glycated hemoglobin; med.: medication; ODI: oxygen desaturation index; PPI: proton-pump inhibitors; SBP: systolic blood pressure. Percentages of highest education do not add to 100% because of participants who did not complete the compulsory education.

**e-Table 2.** Descriptive characteristics by groups of oxygen desaturation index (ODI).

Legend: Participants with ODI data were divided into quartiles (q1, q2, q3, and q4) in order of ascending ODI values. Continuous variables presented as median [interquartile range] and categorical variables presented as absolute numbers (%). AHI: apnea-hypopnea index; BMI: Body mass index; DBP: diastolic blood pressure; ESS: Epworth sleepiness scale; HbA1c: glycated hemoglobin; med.: medication; PPI: proton-pump inhibitors; SBP: systolic blood pressure; T90: percentage of time with oxygen saturation below 90%. Percentages of highest education do not add to 100% because of participants who did not complete the compulsory education.

**e-Table 3.** Partial Spearman's correlations between AHI, T90, or ODI and Shannon diversity index.

Lenged: Main model: adjustment for age, sex, smoking, alcohol intake, body mass index, and DNA extraction plate. Extended model: additional adjustment for fiber intake, total energy intake, leisure physical activity, education, birth country, and season. AHI: apnea-hypopnea index; ODI: oxygen desaturation index; T90: percentage of time with oxygen saturation below 90%.

**e-Table 4.** Pairwise comparisons of Bray-Curtis dissimilarity between OSA severity groups based on AHI, T90, or ODI using permutational analysis of variance (PERMANOVA).

Legend: Main model: adjustment for age, sex, smoking, alcohol intake, body mass index, and DNA extraction plate. Extended model: additional adjustment for fiber intake, total energy intake, leisure physical activity, education, birth country, and season. Groups based on AHI: No OSA: AHI<5; Mild: AHI 5–14.9; Moderate: AHI 15–29.9; Severe: AHI ≥30. Groups based on T90: one category including participants with T90 = 0, and the remaining participants divided into tertiles (t1: T90 = 1–3; t2: T90 = 4–14; and t3: T90 ≥15). Groups based on ODI: quartiles of ODI (q1: ODI = 0–1.8; q2: ODI = 1.9–4.3; q3: ODI = 4.4–9.4; and q4: ODI ≥ 9.5).

**e-Table 4.** Partial Spearman’s correlations of AHI, T90, and ODI with microbiota species using the main model not including BMI.

Legend: Associations adjusted for age, sex, smoking, alcohol intake, and DNA extraction plate. Adjustment for multiple testing using the Benjamini-Hochberg method and presented as q-values. Under the column "Metagenomics species", the information between parenthesis is the internal identifier for the respective species. AHI: apnea-hypopnea index; ODI: oxygen desaturation index; and T90: percentage of time with oxygen saturation below 90%.

**e-Table 6.** Partial Spearman’s correlations of AHI, T90, and ODI with microbiota species using the complete main model.

Legend: Only the species identified in the model not adjusted for body mass index (BMI) were included in this analysis. Associations adjusted for age, sex, smoking, alcohol intake, BMI, and DNA extraction plate. Adjustment for multiple testing using the Benjamini-Hochberg method and presented as q-values. Under the column "Metagenomics species", the information between pararenthesis is the internal identifier for the respective species. AHI: apnea-hypopnea index; ODI: oxygen desaturation index; and T90: percentage of time with oxygen saturation below 90%.

**e-Table 7.** Partial Spearman’s correlations of AHI, T90, and ODI with microbiota species using the extended main model.

Legend: Only the species identified in the model not adjusted for body mass index (BMI) were included in this analysis. Associations adjusted for age, sex, smoking, alcohol intake, BMI, and DNA extraction plate. Adjustment for multiple testing for those species identified in the model not adjust for BMI using the Benjamini-Hochberg method and presented as q-values. Under the column "Metagenomics species", the information between pararenthesis is the internal identifier for the respective species. AHI: apnea-hypopnea index; ODI: oxygen desaturation index; and T90: percentage of time with oxygen saturation below 90%.

**e-Table 8.** Relative abundance and taxonomy of the 128 species associated with T90/ODI.

Legend: Table showing the median and percentiles of relative abundance for the 128 species associated with T90/ODI after adjustment for the extended model covariates.

**e-Table 9.** Sensitivity analysis for the species associated with T90/ODI in the extended main model. Three sensitivity analyses: medication model, exclusion of antibiotic users, and exclusion of self-reported lung disease

Legend: Partial Spearman’s correlations of T90 and ODI with the specified species. Medication model: adjustment for the extended model covariates, metformin use, proton pump inhibitor use, anti-hypertensive medication use, and use of medication for hyperlipidemia. Whr model: adjustment for the extended model covariates with additional adjustment for waist-hip ratio. Antibiotic sensitivity analysis: removal of 347 participants that used antibiotics six months before sampling. Analysis adjusted for the extended model covariates. Lung disease sensitivity analysis: removal of 29 participants that self-reported a doctor diagnosis of chronic obstructive pulmonary disease, chronic bronchitis, or pulmonary emphysema. Analysis adjusted for the extended model covariates. Adjustment for multiple testing for those species identified in the extended model using the Benjamini-Hochberg method and presented as q-values. Under the column "Metagenomics species", the information between parenthesis is the internal identifier for the respective species. AHI: apnea-hypopnea index; ODI: oxygen desaturation index; and T90: percentage of time with oxygen saturation below 90%.

**e-Table 10.** Partial Spearman’s correlations of AHI with microbiota species using the extended model after imputing missing AHI values.

Legend: Missing AHI values for participants with valid T90 and ODI were imputed using predicted mean matching. Only the species identified in the model not adjusted for BMI were included in this analysis. Associations adjusted for age, sex, smoking, alcohol intake, BMI, and DNA extraction plate. Adjustment for multiple testing using the Benjamini-Hochberg method and presented as q-values. Under the column "Metagenomics species", the information between parenthesis is the internal identifier for the respective species. AHI: apnea-hypopnea index

**e-Table 11.** Stratified analysis for hemoglobin level for the species associated with T90/ODI in the extended model.

Legend: Partial Spearman’s correlations of T90 and ODI with the specified species.

Participants were categorized into low or high hemoglobin groups based on the sex-specific median hemoglobin level. Analysis adjusted for age, sex, smoking, alcohol intake, BMI, Shannon index, and DNA extraction plate. Under the column "Metagenomics species", the information between parenthesis is the internal identifier for the respective species. AHI: apnea-hypopnea index; ODI: oxygen desaturation index; and T90: percentage of time with oxygen saturation below 90%.

**e-Table 12.** Enrichment for gut metabolic modules (GMM) in the associations of AHI, T90, or ODI with microbiota species.

Legend: Enrichment analysis performed on ranked p-values of the partial Spearman’s correlations from the extended model, stratified by the direction of the Spearman's correlation coefficient. Adjustment for multiple testing using the Benjamini-Hochberg method and presented as q-values. Enrichments scores (NES) are normalized to mean enrichment score of random samples of the same size. Size is the number of species containing that module. AHI: apnea-hypopnea index; ODI: oxygen desaturation index; and T90: percentage of time with oxygen saturation below 90%.

**e-Table 13.** Partial Spearman’s correlations of microbiota features with systolic blood pressure (SBP), diastolic blood pressure (DBP), and glycated hemoglobin (Hb1Ac).

Legend: The microbiota features are the 128 species associated with T90/ODI in the extended model. Health outcomes are systolic blood pressure (SBP), diastolic blood pressure (DBP), and glycated hemoglobin (HbA1c). OSA adjusted: adjustment for age, sex, alcohol intake, smoking, fiber intake, total energy intake, leisure physical activity, birth country, apnea-hypopnea index (AHI), oxygen desaturation index (ODI), percentage of the sleep time with oxygen saturation < 90% (T90), and DNA extraction plate. OSA and BMI adjusted: additional adjustment for body mass index. Under the column "Microbiota features", the information between parenthesis is the internal identifier for the respective specie

**References**

1. Griffith DM, Veech JA, Marsh CJ. **cooccur** : Probabilistic Species Co-Occurrence Analysis in *R*. *J Stat Softw*. 2016;69(Code Snippet 2). doi:10.18637/jss.v069.c02
